# Supplementary material for: The overlapping burden of the three leading causes of disability and death in sub-Saharan African children
Source: Nat Commun. 2022 Dec 6;13:7457. doi: 10.1038/s41467-022-34240-6 (PMC9726883; doi:10.1038/s41467-022-34240-6)
Supplement: Supplementary file 1 — Supplementary Information [file 41467_2022_34240_MOESM1_ESM.pdf]

Supplementary Figure 1 |Fraction of total all-cause DALYs attributable to malaria, diarrhoea, or LRIs

The combined DALYs burden of malaria, diarrhoea, or LRI experienced by children under-5 is compared to the total DALYs burden experienced by children under-5 in 2000, 2005, 2010, and 2017.

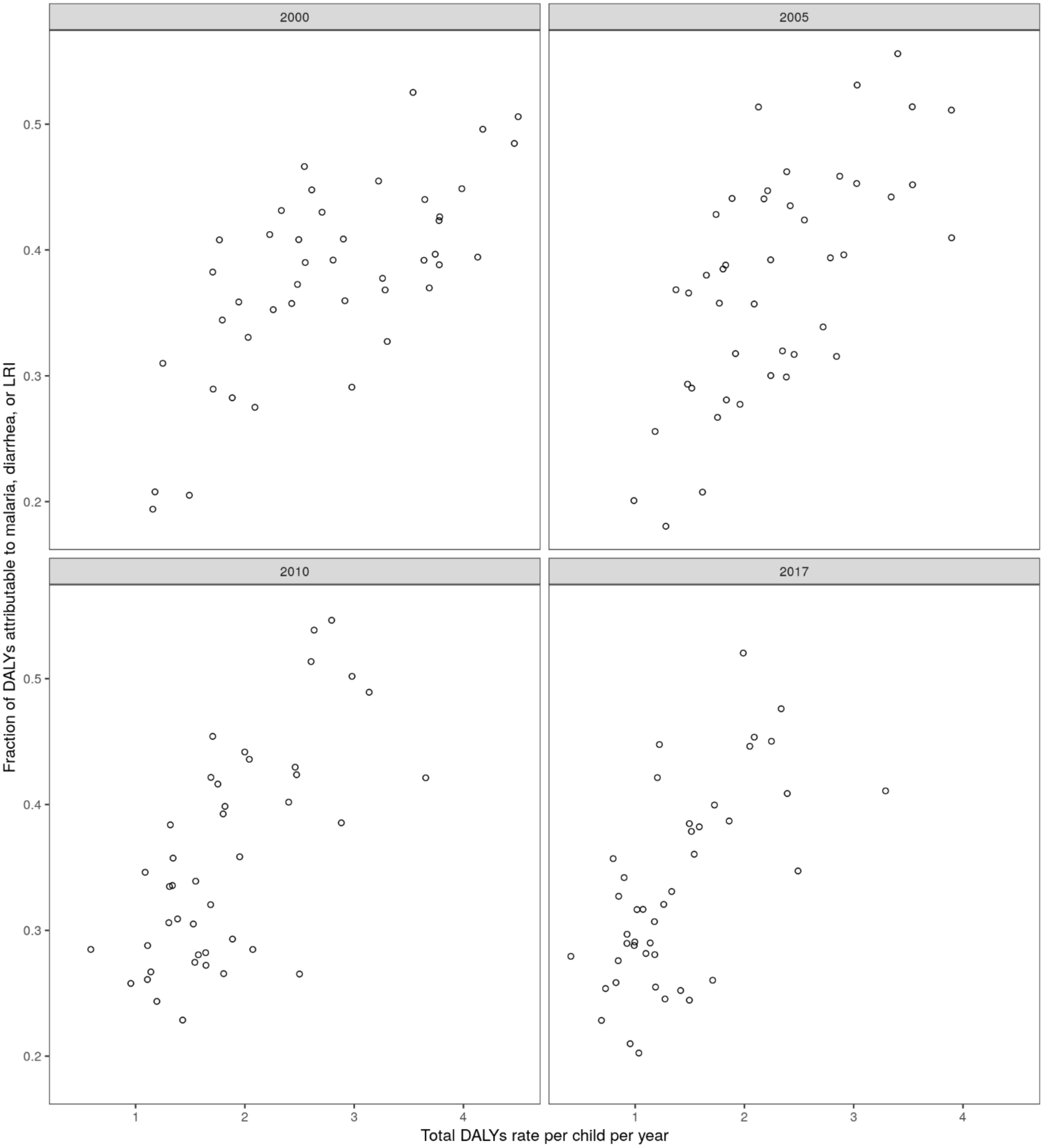

Supplementary Figure 2 | Change in DALYs by country, 2000–2017

a–b, Composition of combined diarrhoea, LRI, and malaria DALYs compared to all-cause DALYs (**a**), and the composition of diarrhoea, LRI, and malaria DALYs within this three-cause envelope (**b**). Each vertical bar represents a country (designated by **ISO 3166-1 alpha-3 codes**). **a,** The total length of the bar represents the change in all-cause DALYs between 2000 and 2017; the location of the top of the bar indicates the DALY rate in 2000, and the location of the bottom of the bar indicates the DALY rate in 2017. Each bar is divided into two sections to represent the proportion of all-cause DALYs that are due to diarrhoea, LRIs, and malaria; the top half (purple) represents the proportion of DALYs due to these three causes, the bottom half (grey) represents the proportion of DALYs that are due to every other cause. The colour gradients indicate the change in DALYs between 2000 and 2017. Darker colours represent a larger percentage relative decline than lighter colours. **b,** The total length of the bar represents the change in DALYs due to diarrhoea, LRIs, and malaria between 2000 and 2017. Each bar is divided into three segments that represent the proportion of diarrhoea, LRI, and malaria burden in 2000, respectively. The colour gradients indicate the change in DALYs between 2000 and 2017. Darker colours represent a larger percentage relative decline than lighter colours. Bars are ordered by combined DALYs in 2017 and countries with * indicate at least one of the three causes had an increase in burden from 2000 to 2017.

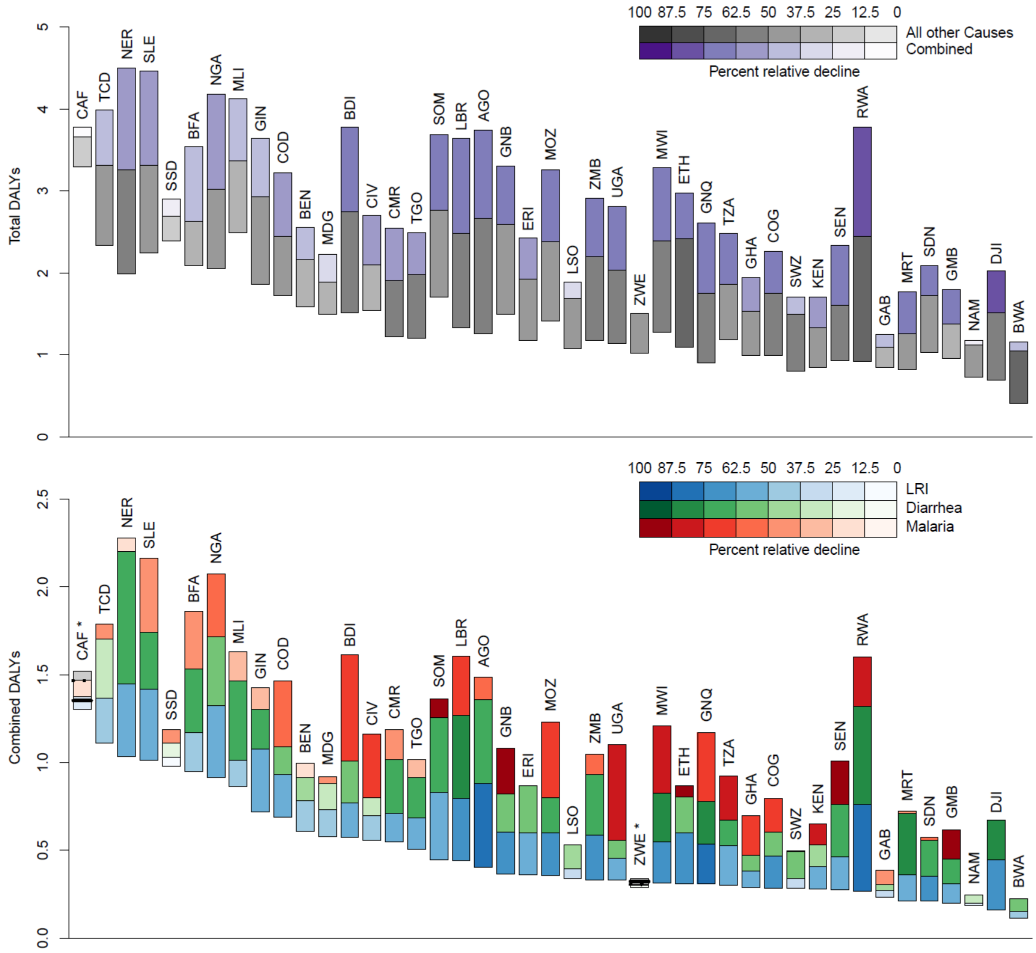

Supplementary Figure 3 |Annualised rates of change (AROC), 2000-2017

a–d, Yearly AROCs are plotted at the second administrative unit level for 2000-2017 for **a,** malaria, **b,** diarrhoea, **c,** LRIs, and **d,** the combined DALY rates. Yellow represents the small number of regions where the burden increased over this period. Maps were produced using ArcGIS Desktop 10.6.

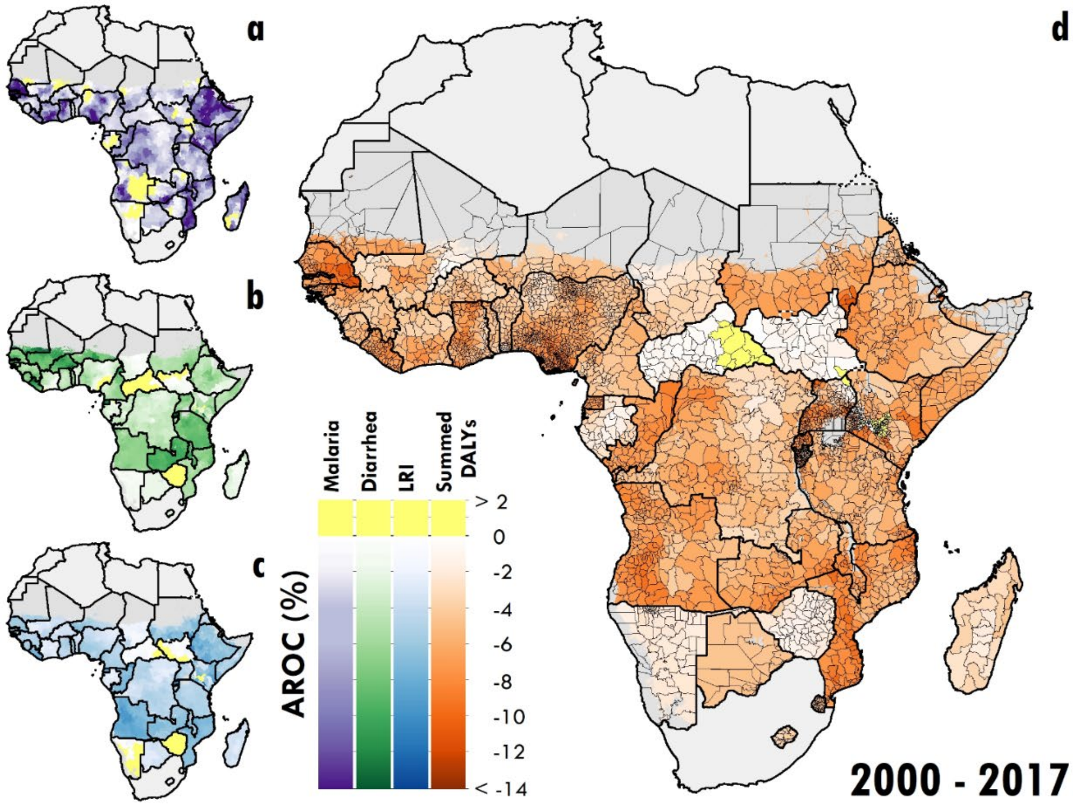

Supplementary results: DALYs rate per child per year in 2000 and 2017 by country and unit. Upper and lower values indicate bounds of 95% UI. AROC indicates annualized rate of change between 2000 and 2017.

Table 1: LRI DALYs rate by unit

Country	Unit	year	mean rate	lower rate	upper rate
**North Africa and Middle East**					
Sudan	Abu Hamad	2000	0.110	0.185	0.279
Sudan	Abu Hamad	2017	0.043	0.069	0.107
Sudan	Abu Hamad	2000-2017	-0.082	-0.055	-0.025
Sudan	Abu Jubaiyah	2000	0.158	0.268	0.421
Sudan	Abu Jubaiyah	2017	0.042	0.088	0.159
Sudan	Abu Jubaiyah	2000-2017	-0.122	-0.070	-0.023
Sudan	Abyei	2000	0.182	0.298	0.442
Sudan	Abyei	2017	0.039	0.086	0.150
Sudan	Abyei	2000-2017	-0.129	-0.081	-0.036
Sudan	Ad Damazin	2000	0.188	0.312	0.489
Sudan	Ad Damazin	2017	0.040	0.094	0.170
Sudan	Ad Damazin	2000-2017	-0.136	-0.080	-0.029
Sudan	Ad Damer	2000	0.126	0.211	0.321
Sudan	Ad Damer	2017	0.044	0.071	0.110
Sudan	Ad Damer	2000-2017	-0.087	-0.062	-0.035
Sudan	Ad Dinder	2000	0.174	0.282	0.432
Sudan	Ad Dinder	2017	0.046	0.107	0.195
Sudan	Ad Dinder	2000-2017	-0.113	-0.059	-0.012
Sudan	Ad Douiem	2000	0.149	0.239	0.357
Sudan	Ad Douiem	2017	0.044	0.087	0.155
Sudan	Ad Douiem	2000-2017	-0.093	-0.057	-0.023
Sudan	Addabah	2000	0.091	0.159	0.249
Sudan	Addabah	2017	0.040	0.065	0.103
Sudan	Addabah	2000-2017	-0.077	-0.050	-0.022
Sudan	Al Deain	2000	0.169	0.280	0.418
Sudan	Al Deain	2017	0.055	0.094	0.156
Sudan	Al Deain	2000-2017	-0.099	-0.066	-0.033
Sudan	Al Fasher	2000	0.111	0.181	0.270
Sudan	Al Fasher	2017	0.058	0.094	0.146
Sudan	Al Fasher	2000-2017	-0.067	-0.037	-0.008
Sudan	Al Faw	2000	0.155	0.256	0.387
Sudan	Al Faw	2017	0.039	0.087	0.161
Sudan	Al Faw	2000-2017	-0.125	-0.071	-0.023
Sudan	Al Fushqa	2000	0.164	0.272	0.414
Sudan	Al Fushqa	2017	0.040	0.089	0.163
Sudan	Al Fushqa	2000-2017	-0.129	-0.073	-0.025
Sudan	Al Gadaref	2000	0.155	0.252	0.390
Sudan	Al Gadaref	2017	0.039	0.082	0.154
Sudan	Al Gadaref	2000-2017	-0.125	-0.076	-0.029
Sudan	Al Galabat	2000	0.161	0.261	0.389
Sudan	Al Galabat	2017	0.040	0.089	0.158
Sudan	Al Galabat	2000-2017	-0.124	-0.070	-0.021
Sudan	Al Gash	2000	0.139	0.232	0.348
Sudan	Al Gash	2017	0.042	0.098	0.183
Sudan	Al Gash	2000-2017	-0.109	-0.055	-0.006
Sudan	Al Geneina	2000	0.114	0.198	0.319
Sudan	Al Geneina	2017	0.050	0.083	0.136
Sudan	Al Geneina	2000-2017	-0.082	-0.055	-0.028
Sudan	Al Gutaina	2000	0.138	0.227	0.339
Sudan	Al Gutaina	2017	0.046	0.084	0.146
Sudan	Al Gutaina	2000-2017	-0.092	-0.057	-0.023
Sudan	Al Jabalian	2000	0.153	0.249	0.369
Sudan	Al Jabalian	2017	0.043	0.092	0.172
Sudan	Al Jabalian	2000-2017	-0.101	-0.059	-0.021
Sudan	Al Kamlin	2000	0.131	0.217	0.333
Sudan	Al Kamlin	2017	0.043	0.070	0.112
Sudan	Al Kamlin	2000-2017	-0.095	-0.063	-0.032

Table 1: LRI DALYs rate by unit *(continued)*

Country	Unit	year	mean rate	lower rate	upper rate
Sudan	Al Kurumik	2000	0.225	0.393	0.635
Sudan	Al Kurumik	2017	0.040	0.084	0.149
Sudan	Al Kurumik	2000-2017	-0.148	-0.097	-0.048
Sudan	Al Mahagil	2000	0.137	0.224	0.343
Sudan	Al Mahagil	2017	0.043	0.079	0.137
Sudan	Al Mahagil	2000-2017	-0.095	-0.060	-0.027
Sudan	Al Matammah	2000	0.125	0.212	0.324
Sudan	Al Matammah	2017	0.042	0.068	0.104
Sudan	Al Matammah	2000-2017	-0.089	-0.064	-0.035
Sudan	Al Rahd	2000	0.159	0.260	0.382
Sudan	Al Rahd	2017	0.040	0.089	0.158
Sudan	Al Rahd	2000-2017	-0.120	-0.070	-0.023
Sudan	Al Roseires	2000	0.239	0.411	0.664
Sudan	Al Roseires	2017	0.038	0.085	0.153
Sudan	Al Roseires	2000-2017	-0.154	-0.097	-0.041
Sudan	As Salam	2000	0.178	0.293	0.432
Sudan	As Salam	2017	0.039	0.084	0.147
Sudan	As Salam	2000-2017	-0.126	-0.080	-0.037
Sudan	Atbara	2000	0.126	0.204	0.313
Sudan	Atbara	2017	0.043	0.075	0.123
Sudan	Atbara	2000-2017	-0.092	-0.058	-0.025
Sudan	Bara	2000	0.113	0.189	0.285
Sudan	Bara	2017	0.054	0.091	0.150
Sudan	Bara	2000-2017	-0.081	-0.044	-0.009
Sudan	Baw	2000	0.239	0.419	0.690
Sudan	Baw	2017	0.038	0.084	0.151
Sudan	Baw	2000-2017	-0.158	-0.102	-0.052
Sudan	Berber	2000	0.113	0.188	0.290
Sudan	Berber	2017	0.044	0.071	0.109
Sudan	Berber	2000-2017	-0.082	-0.055	-0.026
Sudan	Buram	2000	0.171	0.280	0.418
Sudan	Buram	2017	0.051	0.095	0.165
Sudan	Buram	2000-2017	-0.096	-0.057	-0.019
Sudan	Dilling	2000	0.155	0.258	0.387
Sudan	Dilling	2017	0.039	0.085	0.153
Sudan	Dilling	2000-2017	-0.119	-0.069	-0.025
Sudan	Dongola	2000	0.093	0.168	0.290
Sudan	Dongola	2017	0.038	0.065	0.111
Sudan	Dongola	2000-2017	-0.078	-0.053	-0.025
Sudan	East al Gazera	2000	0.129	0.213	0.318
Sudan	East al Gazera	2017	0.046	0.085	0.143
Sudan	East al Gazera	2000-2017	-0.093	-0.054	-0.016
Sudan	En Nuhud	2000	0.135	0.227	0.338
Sudan	En Nuhud	2017	0.054	0.092	0.152
Sudan	En Nuhud	2000-2017	-0.089	-0.057	-0.022
Sudan	Geissan	2000	0.241	0.430	0.710
Sudan	Geissan	2017	0.040	0.083	0.147
Sudan	Geissan	2000-2017	-0.157	-0.101	-0.048
Sudan	Ghebeish	2000	0.154	0.247	0.365
Sudan	Ghebeish	2017	0.053	0.092	0.153
Sudan	Ghebeish	2000-2017	-0.094	-0.063	-0.033
Sudan	Halayeb	2000	0.084	0.141	0.212
Sudan	Halayeb	2017	0.032	0.070	0.127
Sudan	Halayeb	2000-2017	-0.095	-0.042	0.003
Sudan	Hamashkorieb	2000	0.129	0.214	0.326
Sudan	Hamashkorieb	2017	0.042	0.097	0.177
Sudan	Hamashkorieb	2000-2017	-0.104	-0.051	-0.003
Sudan	Id El Ghanem	2000	0.166	0.274	0.409
Sudan	Id El Ghanem	2017	0.057	0.095	0.151
Sudan	Id El Ghanem	2000-2017	-0.091	-0.059	-0.026
Sudan	Jebrat al Sheikh	2000	0.108	0.176	0.254

Table 1: LRI DALYs rate by unit *(continued)*

Country	Unit	year	mean rate	lower rate	upper rate
Sudan	Jebrat al Sheikh	2017	0.049	0.082	0.136
Sudan	Jebrat al Sheikh	2000-2017	-0.075	-0.041	-0.005
Sudan	Kabkabiya	2000	0.111	0.191	0.292
Sudan	Kabkabiya	2017	0.057	0.093	0.148
Sudan	Kabkabiya	2000-2017	-0.074	-0.042	-0.011
Sudan	Kadugli	2000	0.154	0.258	0.402
Sudan	Kadugli	2017	0.041	0.089	0.163
Sudan	Kadugli	2000-2017	-0.123	-0.066	-0.020
Sudan	Karary	2000	0.096	0.170	0.281
Sudan	Karary	2017	0.035	0.064	0.109
Sudan	Karary	2000-2017	-0.079	-0.053	-0.027
Sudan	Kas	2000	0.126	0.218	0.355
Sudan	Kas	2017	0.058	0.097	0.152
Sudan	Kas	2000-2017	-0.086	-0.050	-0.016
Sudan	Kassala	2000	0.163	0.266	0.396
Sudan	Kassala	2017	0.041	0.093	0.170
Sudan	Kassala	2000-2017	-0.118	-0.067	-0.019
Sudan	Khartoum	2000	0.091	0.193	0.366
Sudan	Khartoum	2017	0.032	0.068	0.137
Sudan	Khartoum	2000-2017	-0.083	-0.058	-0.033
Sudan	Khartoum Bahri	2000	0.107	0.186	0.308
Sudan	Khartoum Bahri	2017	0.035	0.063	0.111
Sudan	Khartoum Bahri	2000-2017	-0.086	-0.059	-0.033
Sudan	Kosti	2000	0.160	0.257	0.390
Sudan	Kosti	2017	0.045	0.095	0.174
Sudan	Kosti	2000-2017	-0.102	-0.059	-0.019
Sudan	Kutum	2000	0.108	0.178	0.264
Sudan	Kutum	2017	0.058	0.098	0.166
Sudan	Kutum	2000-2017	-0.062	-0.033	-0.004
Sudan	Lagawa	2000	0.153	0.252	0.377
Sudan	Lagawa	2017	0.040	0.086	0.149
Sudan	Lagawa	2000-2017	-0.115	-0.068	-0.024
Sudan	Mellit	2000	0.104	0.169	0.243
Sudan	Mellit	2017	0.060	0.097	0.156
Sudan	Mellit	2000-2017	-0.062	-0.031	0.000
Sudan	Merawi	2000	0.099	0.171	0.266
Sudan	Merawi	2017	0.038	0.064	0.107
Sudan	Merawi	2000-2017	-0.081	-0.056	-0.032
Sudan	Mukjar	2000	0.152	0.257	0.386
Sudan	Mukjar	2017	0.048	0.083	0.136
Sudan	Mukjar	2000-2017	-0.103	-0.068	-0.035
Sudan	Nahr Atbara	2000	0.155	0.251	0.379
Sudan	Nahr Atbara	2017	0.042	0.091	0.161
Sudan	Nahr Atbara	2000-2017	-0.114	-0.065	-0.022
Sudan	North al Gazera	2000	0.134	0.220	0.323
Sudan	North al Gazera	2017	0.042	0.073	0.119
Sudan	North al Gazera	2000-2017	-0.096	-0.062	-0.028
Sudan	Nyala	2000	0.145	0.245	0.369
Sudan	Nyala	2000	0.132	0.227	0.338
Sudan	Nyala	2017	0.058	0.098	0.160
Sudan	Nyala	2017	0.056	0.094	0.149
Sudan	Nyala	2000-2017	-0.081	-0.054	-0.023
Sudan	Nyala	2000-2017	-0.084	-0.051	-0.017
Sudan	Omdurman	2000	0.101	0.181	0.307
Sudan	Omdurman	2017	0.035	0.066	0.117
Sudan	Omdurman	2000-2017	-0.083	-0.057	-0.031
Sudan	Port Sudan	2000	0.095	0.154	0.217
Sudan	Port Sudan	2017	0.033	0.069	0.127
Sudan	Port Sudan	2000-2017	-0.092	-0.049	-0.006
Sudan	Rashad	2000	0.161	0.263	0.405
Sudan	Rashad	2017	0.041	0.088	0.157

Table 1: LRI DALYs rate by unit *(continued)*

Country	Unit	year	mean rate	lower rate	upper rate
Sudan	Rashad	2000-2017	-0.119	-0.068	-0.021
Sudan	Sennar	2000	0.137	0.227	0.331
Sudan	Sennar	2017	0.045	0.099	0.175
Sudan	Sennar	2000-2017	-0.099	-0.052	-0.010
Sudan	Seteet	2000	0.141	0.232	0.345
Sudan	Seteet	2017	0.042	0.093	0.166
Sudan	Seteet	2000-2017	-0.109	-0.057	-0.011
Sudan	Sharg En Nile	2000	0.116	0.194	0.300
Sudan	Sharg En Nile	2017	0.039	0.063	0.099
Sudan	Sharg En Nile	2000-2017	-0.093	-0.062	-0.028
Sudan	Sharq al Gazera	2000	0.131	0.216	0.323
Sudan	Sharq al Gazera	2017	0.041	0.076	0.128
Sudan	Sharq al Gazera	2000-2017	-0.100	-0.060	-0.023
Sudan	Sheikan	2000	0.113	0.186	0.274
Sudan	Sheikan	2017	0.057	0.093	0.148
Sudan	Sheikan	2000-2017	-0.076	-0.043	-0.010
Sudan	Shendi	2000	0.115	0.195	0.289
Sudan	Shendi	2017	0.042	0.069	0.107
Sudan	Shendi	2000-2017	-0.090	-0.058	-0.028
Sudan	Singa	2000	0.170	0.276	0.423
Sudan	Singa	2017	0.047	0.107	0.194
Sudan	Singa	2000-2017	-0.112	-0.060	-0.015
Sudan	Sinkat	2000	0.081	0.137	0.205
Sudan	Sinkat	2017	0.033	0.075	0.136
Sudan	Sinkat	2000-2017	-0.090	-0.040	0.007
Sudan	South al Gazera	2000	0.138	0.223	0.326
Sudan	South al Gazera	2017	0.042	0.080	0.134
Sudan	South al Gazera	2000-2017	-0.100	-0.061	-0.026
Sudan	South Khartoum	2000	0.101	0.185	0.309
Sudan	South Khartoum	2017	0.038	0.064	0.101
Sudan	South Khartoum	2000-2017	-0.090	-0.058	-0.031
Sudan	Sowdari	2000	0.106	0.180	0.279
Sudan	Sowdari	2017	0.055	0.090	0.142
Sudan	Sowdari	2000-2017	-0.073	-0.039	-0.003
Sudan	Talodi	2000	0.154	0.263	0.418
Sudan	Talodi	2017	0.041	0.090	0.163
Sudan	Talodi	2000-2017	-0.120	-0.068	-0.019
Sudan	Tokar	2000	0.083	0.141	0.212
Sudan	Tokar	2017	0.033	0.075	0.136
Sudan	Tokar	2000-2017	-0.095	-0.043	0.003
Sudan	Tulus	2000	0.156	0.261	0.393
Sudan	Tulus	2017	0.058	0.094	0.151
Sudan	Tulus	2000-2017	-0.085	-0.057	-0.025
Sudan	Um Al Gura	2000	0.139	0.220	0.333
Sudan	Um Al Gura	2017	0.045	0.087	0.149
Sudan	Um Al Gura	2000-2017	-0.096	-0.055	-0.019
Sudan	Um Badda	2000	0.104	0.183	0.310
Sudan	Um Badda	2017	0.038	0.066	0.113
Sudan	Um Badda	2000-2017	-0.086	-0.057	-0.032
Sudan	Um Kadada	2000	0.118	0.199	0.292
Sudan	Um Kadada	2017	0.057	0.092	0.145
Sudan	Um Kadada	2000-2017	-0.075	-0.047	-0.021
Sudan	Um Rawaba	2000	0.142	0.230	0.344
Sudan	Um Rawaba	2017	0.045	0.091	0.162
Sudan	Um Rawaba	2000-2017	-0.096	-0.056	-0.016
Sudan	Wadi Halfa	2000	0.098	0.166	0.249
Sudan	Wadi Halfa	2017	0.037	0.059	0.093
Sudan	Wadi Halfa	2000-2017	-0.085	-0.057	-0.024
Sudan	Zallingi	2000	0.143	0.242	0.374
Sudan	Zallingi	2017	0.051	0.082	0.129
Sudan	Zallingi	2000-2017	-0.099	-0.070	-0.042

Table 1: LRI DALYs rate by unit *(continued)*

Country	Unit	year	mean rate	lower rate	upper rate
**Sub-Saharan Africa**					
Angola	Alto Cauale	2000	0.348	0.608	1.066
Angola	Alto Cauale	2017	0.092	0.163	0.283
Angola	Alto Cauale	2000-2017	-0.092	-0.080	-0.068
Angola	Alto Zambeze	2000	0.264	0.454	0.764
Angola	Alto Zambeze	2017	0.081	0.143	0.249
Angola	Alto Zambeze	2000-2017	-0.074	-0.068	-0.062
Angola	Ambaca	2000	0.411	0.714	1.219
Angola	Ambaca	2017	0.097	0.166	0.286
Angola	Ambaca	2000-2017	-0.100	-0.086	-0.072
Angola	Amboim	2000	0.450	0.785	1.396
Angola	Amboim	2017	0.096	0.164	0.291
Angola	Amboim	2000-2017	-0.108	-0.091	-0.075
Angola	Ambriz	2000	0.295	0.504	0.855
Angola	Ambriz	2017	0.087	0.147	0.264
Angola	Ambriz	2000-2017	-0.080	-0.071	-0.062
Angola	Ambuila	2000	0.349	0.599	1.055
Angola	Ambuila	2017	0.083	0.146	0.252
Angola	Ambuila	2000-2017	-0.098	-0.084	-0.070
Angola	Andulo	2000	0.413	0.712	1.246
Angola	Andulo	2017	0.084	0.144	0.257
Angola	Andulo	2000-2017	-0.112	-0.094	-0.077
Angola	Baía Farta	2000	0.271	0.470	0.823
Angola	Baía Farta	2017	0.078	0.133	0.236
Angola	Baía Farta	2000-2017	-0.088	-0.074	-0.062
Angola	Bailundo	2000	0.457	0.798	1.408
Angola	Bailundo	2017	0.087	0.153	0.262
Angola	Bailundo	2000-2017	-0.116	-0.097	-0.079
Angola	Balombo	2000	0.344	0.597	1.046
Angola	Balombo	2017	0.081	0.139	0.237
Angola	Balombo	2000-2017	-0.103	-0.086	-0.072
Angola	Banga	2000	0.402	0.715	1.228
Angola	Banga	2017	0.084	0.150	0.258
Angola	Banga	2000-2017	-0.107	-0.093	-0.076
Angola	Belize	2000	0.421	0.646	1.018
Angola	Belize	2017	0.108	0.176	0.288
Angola	Belize	2000-2017	-0.090	-0.077	-0.062
Angola	Bembe	2000	0.355	0.608	1.052
Angola	Bembe	2017	0.086	0.148	0.257
Angola	Bembe	2000-2017	-0.098	-0.085	-0.073
Angola	Benguela	2000	0.290	0.516	0.888
Angola	Benguela	2017	0.079	0.130	0.238
Angola	Benguela	2000-2017	-0.098	-0.082	-0.063
Angola	Bibala	2000	0.321	0.567	1.003
Angola	Bibala	2017	0.082	0.140	0.248
Angola	Bibala	2000-2017	-0.098	-0.085	-0.071
Angola	Bocoio	2000	0.293	0.513	0.866
Angola	Bocoio	2017	0.080	0.139	0.243
Angola	Bocoio	2000-2017	-0.090	-0.078	-0.064
Angola	Bolongongo	2000	0.413	0.729	1.276
Angola	Bolongongo	2017	0.092	0.158	0.273
Angola	Bolongongo	2000-2017	-0.104	-0.088	-0.074
Angola	Buco Zau	2000	0.400	0.651	1.080
Angola	Buco Zau	2017	0.096	0.159	0.264
Angola	Buco Zau	2000-2017	-0.094	-0.081	-0.068
Angola	Buengas	2000	0.356	0.624	1.065
Angola	Buengas	2017	0.095	0.167	0.294
Angola	Buengas	2000-2017	-0.093	-0.080	-0.067
Angola	Bula Atumba	2000	0.415	0.722	1.250
Angola	Bula Atumba	2017	0.091	0.154	0.274
Angola	Bula Atumba	2000-2017	-0.106	-0.089	-0.073

Table 1: LRI DALYs rate by unit *(continued)*

Country	Unit	year	mean rate	lower rate	upper rate
Angola	Bungo	2000	0.350	0.610	1.066
Angola	Bungo	2017	0.094	0.164	0.289
Angola	Bungo	2000-2017	-0.091	-0.078	-0.066
Angola	Caála	2000	0.424	0.736	1.320
Angola	Caála	2017	0.087	0.148	0.261
Angola	Caála	2000-2017	-0.112	-0.095	-0.080
Angola	Cabinda	2000	0.337	0.576	1.023
Angola	Cabinda	2017	0.088	0.144	0.268
Angola	Cabinda	2000-2017	-0.091	-0.080	-0.067
Angola	Cacolo	2000	0.271	0.470	0.792
Angola	Cacolo	2017	0.082	0.144	0.255
Angola	Cacolo	2000-2017	-0.079	-0.071	-0.063
Angola	Caconda	2000	0.314	0.550	0.966
Angola	Caconda	2017	0.083	0.142	0.247
Angola	Caconda	2000-2017	-0.095	-0.082	-0.071
Angola	Cacuaco	2000	0.252	0.432	0.788
Angola	Cacuaco	2017	0.077	0.132	0.242
Angola	Cacuaco	2000-2017	-0.085	-0.070	-0.056
Angola	Cacuzo	2000	0.369	0.633	1.090
Angola	Cacuzo	2017	0.083	0.148	0.252
Angola	Cacuzo	2000-2017	-0.101	-0.087	-0.073
Angola	Cahama	2000	0.270	0.462	0.797
Angola	Cahama	2017	0.075	0.131	0.237
Angola	Cahama	2000-2017	-0.088	-0.076	-0.066
Angola	Caiambambo	2000	0.274	0.481	0.827
Angola	Caiambambo	2017	0.079	0.138	0.242
Angola	Caiambambo	2000-2017	-0.087	-0.074	-0.062
Angola	Calai	2000	0.314	0.541	0.944
Angola	Calai	2017	0.081	0.141	0.238
Angola	Calai	2000-2017	-0.095	-0.080	-0.068
Angola	Calandula	2000	0.361	0.619	1.080
Angola	Calandula	2017	0.089	0.155	0.272
Angola	Calandula	2000-2017	-0.095	-0.082	-0.071
Angola	Caluquembe	2000	0.314	0.555	0.969
Angola	Caluquembe	2017	0.079	0.139	0.245
Angola	Caluquembe	2000-2017	-0.096	-0.084	-0.071
Angola	Camacuio	2000	0.323	0.559	0.981
Angola	Camacuio	2017	0.081	0.142	0.251
Angola	Camacuio	2000-2017	-0.094	-0.082	-0.069
Angola	Camacupa	2000	0.359	0.625	1.088
Angola	Camacupa	2017	0.080	0.141	0.250
Angola	Camacupa	2000-2017	-0.109	-0.091	-0.076
Angola	Camanongue	2000	0.269	0.469	0.810
Angola	Camanongue	2017	0.083	0.146	0.255
Angola	Camanongue	2000-2017	-0.081	-0.070	-0.061
Angola	Cambambe	2000	0.427	0.741	1.301
Angola	Cambambe	2017	0.081	0.141	0.247
Angola	Cambambe	2000-2017	-0.114	-0.098	-0.081
Angola	Cambulo	2000	0.322	0.548	0.883
Angola	Cambulo	2017	0.100	0.167	0.274
Angola	Cambulo	2000-2017	-0.085	-0.074	-0.063
Angola	Cambundi-Catembo	2000	0.290	0.512	0.862
Angola	Cambundi-Catembo	2017	0.084	0.148	0.261
Angola	Cambundi-Catembo	2000-2017	-0.084	-0.073	-0.063
Angola	Cameia	2000	0.258	0.447	0.766
Angola	Cameia	2017	0.084	0.144	0.253
Angola	Cameia	2000-2017	-0.076	-0.067	-0.059
Angola	Cangandala	2000	0.315	0.554	0.940
Angola	Cangandala	2017	0.086	0.153	0.262
Angola	Cangandala	2000-2017	-0.092	-0.078	-0.067
Angola	Caombo	2000	0.316	0.555	0.935

Table 1: LRI DALYs rate by unit *(continued)*

Country	Unit	year	mean rate	lower rate	upper rate
Angola	Caombo	2017	0.092	0.161	0.274
Angola	Caombo	2000-2017	-0.084	-0.073	-0.063
Angola	Capenda	2000	0.270	0.473	0.803
Angola	Capenda	2017	0.081	0.143	0.254
Angola	Capenda	2000-2017	-0.079	-0.070	-0.061
Angola	Cassongue	2000	0.417	0.732	1.279
Angola	Cassongue	2017	0.091	0.158	0.271
Angola	Cassongue	2000-2017	-0.108	-0.091	-0.077
Angola	Catabola	2000	0.392	0.707	1.251
Angola	Catabola	2017	0.081	0.143	0.252
Angola	Catabola	2000-2017	-0.117	-0.097	-0.078
Angola	Catchiungo	2000	0.414	0.722	1.240
Angola	Catchiungo	2017	0.087	0.152	0.265
Angola	Catchiungo	2000-2017	-0.111	-0.093	-0.076
Angola	Caungula	2000	0.296	0.511	0.858
Angola	Caungula	2017	0.090	0.156	0.271
Angola	Caungula	2000-2017	-0.082	-0.072	-0.061
Angola	Cazenga	2000	0.204	0.374	0.685
Angola	Cazenga	2017	0.071	0.132	0.248
Angola	Cazenga	2000-2017	-0.097	-0.065	-0.031
Angola	Cazengo	2000	0.414	0.731	1.253
Angola	Cazengo	2017	0.087	0.153	0.269
Angola	Cazengo	2000-2017	-0.109	-0.094	-0.079
Angola	Chibia	2000	0.295	0.515	0.907
Angola	Chibia	2017	0.081	0.139	0.242
Angola	Chibia	2000-2017	-0.095	-0.081	-0.068
Angola	Chicomba	2000	0.313	0.543	0.962
Angola	Chicomba	2017	0.078	0.135	0.237
Angola	Chicomba	2000-2017	-0.097	-0.084	-0.072
Angola	Chinguar	2000	0.398	0.682	1.194
Angola	Chinguar	2017	0.083	0.144	0.251
Angola	Chinguar	2000-2017	-0.112	-0.093	-0.077
Angola	Chipindo	2000	0.313	0.545	0.939
Angola	Chipindo	2017	0.082	0.140	0.244
Angola	Chipindo	2000-2017	-0.097	-0.083	-0.069
Angola	Chitato	2000	0.294	0.517	0.852
Angola	Chitato	2017	0.095	0.156	0.269
Angola	Chitato	2000-2017	-0.085	-0.074	-0.062
Angola	Chitembo	2000	0.313	0.554	0.965
Angola	Chitembo	2017	0.077	0.136	0.242
Angola	Chitembo	2000-2017	-0.102	-0.086	-0.072
Angola	Chongoroi	2000	0.310	0.540	0.948
Angola	Chongoroi	2017	0.076	0.133	0.232
Angola	Chongoroi	2000-2017	-0.097	-0.084	-0.071
Angola	Conda	2000	0.448	0.773	1.340
Angola	Conda	2017	0.087	0.153	0.273
Angola	Conda	2000-2017	-0.116	-0.097	-0.077
Angola	Cuaba Nzogo	2000	0.327	0.565	0.973
Angola	Cuaba Nzogo	2017	0.088	0.156	0.265
Angola	Cuaba Nzogo	2000-2017	-0.090	-0.078	-0.067
Angola	Cuangar	2000	0.326	0.559	0.968
Angola	Cuangar	2017	0.085	0.144	0.256
Angola	Cuangar	2000-2017	-0.093	-0.082	-0.071
Angola	Cuango	2000	0.280	0.489	0.812
Angola	Cuango	2017	0.090	0.156	0.274
Angola	Cuango	2000-2017	-0.080	-0.069	-0.059
Angola	Cuanhama	2000	0.264	0.460	0.772
Angola	Cuanhama	2017	0.072	0.125	0.222
Angola	Cuanhama	2000-2017	-0.087	-0.078	-0.069
Angola	Cubal	2000	0.295	0.519	0.872
Angola	Cubal	2017	0.079	0.138	0.243

Table 1: LRI DALYs rate by unit *(continued)*

Country	Unit	year	mean rate	lower rate	upper rate
Angola	Cubal	2000-2017	-0.093	-0.079	-0.066
Angola	Cuchi	2000	0.334	0.577	1.001
Angola	Cuchi	2017	0.080	0.142	0.248
Angola	Cuchi	2000-2017	-0.099	-0.084	-0.072
Angola	Cuemba	2000	0.314	0.539	0.928
Angola	Cuemba	2017	0.078	0.137	0.242
Angola	Cuemba	2000-2017	-0.097	-0.083	-0.072
Angola	Cuilo	2000	0.312	0.525	0.873
Angola	Cuilo	2017	0.095	0.161	0.276
Angola	Cuilo	2000-2017	-0.083	-0.072	-0.062
Angola	Cuimba	2000	0.375	0.637	1.096
Angola	Cuimba	2017	0.096	0.164	0.291
Angola	Cuimba	2000-2017	-0.098	-0.085	-0.072
Angola	Cuito Cuanavale	2000	0.326	0.561	0.976
Angola	Cuito Cuanavale	2017	0.080	0.141	0.250
Angola	Cuito Cuanavale	2000-2017	-0.098	-0.083	-0.071
Angola	Cunda-dia-Baza	2000	0.322	0.559	0.937
Angola	Cunda-dia-Baza	2017	0.097	0.169	0.285
Angola	Cunda-dia-Baza	2000-2017	-0.080	-0.070	-0.060
Angola	Cunhinga	2000	0.406	0.714	1.251
Angola	Cunhinga	2017	0.082	0.143	0.252
Angola	Cunhinga	2000-2017	-0.115	-0.096	-0.080
Angola	Curoca	2000	0.235	0.396	0.682
Angola	Curoca	2017	0.075	0.132	0.240
Angola	Curoca	2000-2017	-0.075	-0.067	-0.059
Angola	Cuvelai	2000	0.284	0.490	0.843
Angola	Cuvelai	2017	0.075	0.129	0.229
Angola	Cuvelai	2000-2017	-0.092	-0.080	-0.069
Angola	Dala	2000	0.265	0.457	0.778
Angola	Dala	2017	0.082	0.145	0.257
Angola	Dala	2000-2017	-0.075	-0.068	-0.060
Angola	Damba	2000	0.358	0.609	1.038
Angola	Damba	2017	0.096	0.164	0.287
Angola	Damba	2000-2017	-0.088	-0.078	-0.066
Angola	Dande	2000	0.298	0.515	0.889
Angola	Dande	2017	0.083	0.141	0.255
Angola	Dande	2000-2017	-0.086	-0.076	-0.066
Angola	Dembos	2000	0.412	0.715	1.227
Angola	Dembos	2017	0.084	0.148	0.258
Angola	Dembos	2000-2017	-0.105	-0.090	-0.075
Angola	Dirico	2000	0.302	0.533	0.943
Angola	Dirico	2017	0.085	0.150	0.268
Angola	Dirico	2000-2017	-0.083	-0.075	-0.065
Angola	Ebo	2000	0.450	0.789	1.372
Angola	Ebo	2017	0.088	0.158	0.274
Angola	Ebo	2000-2017	-0.114	-0.094	-0.077
Angola	Ekunha	2000	0.439	0.775	1.366
Angola	Ekunha	2017	0.089	0.151	0.258
Angola	Ekunha	2000-2017	-0.116	-0.097	-0.078
Angola	Gambos	2000	0.282	0.492	0.867
Angola	Gambos	2017	0.079	0.138	0.243
Angola	Gambos	2000-2017	-0.089	-0.078	-0.066
Angola	Ganda	2000	0.331	0.571	0.993
Angola	Ganda	2017	0.079	0.139	0.241
Angola	Ganda	2000-2017	-0.101	-0.086	-0.071
Angola	Golungo Alto	2000	0.434	0.745	1.273
Angola	Golungo Alto	2017	0.088	0.152	0.268
Angola	Golungo Alto	2000-2017	-0.111	-0.092	-0.076
Angola	Huambo	2000	0.438	0.773	1.373
Angola	Huambo	2017	0.083	0.139	0.250
Angola	Huambo	2000-2017	-0.118	-0.100	-0.081

Table 1: LRI DALYs rate by unit *(continued)*

Country	Unit	year	mean rate	lower rate	upper rate
Angola	Humpata	2000	0.300	0.512	0.906
Angola	Humpata	2017	0.077	0.133	0.245
Angola	Humpata	2000-2017	-0.097	-0.082	-0.068
Angola	Icolo e Bengo	2000	0.289	0.499	0.858
Angola	Icolo e Bengo	2017	0.082	0.141	0.259
Angola	Icolo e Bengo	2000-2017	-0.087	-0.076	-0.063
Angola	Ingombota	2000	0.203	0.374	0.660
Angola	Ingombota	2017	0.068	0.131	0.277
Angola	Ingombota	2000-2017	-0.109	-0.066	-0.026
Angola	Jamba	2000	0.299	0.519	0.888
Angola	Jamba	2017	0.080	0.138	0.236
Angola	Jamba	2000-2017	-0.096	-0.082	-0.070
Angola	Kilamba Kiaxi	2000	0.215	0.378	0.706
Angola	Kilamba Kiaxi	2017	0.071	0.130	0.231
Angola	Kilamba Kiaxi	2000-2017	-0.093	-0.064	-0.035
Angola	Kuito	2000	0.404	0.716	1.301
Angola	Kuito	2017	0.079	0.140	0.249
Angola	Kuito	2000-2017	-0.118	-0.098	-0.080
Angola	Kuvango	2000	0.301	0.525	0.902
Angola	Kuvango	2017	0.080	0.139	0.240
Angola	Kuvango	2000-2017	-0.094	-0.082	-0.070
Angola	Landana	2000	0.336	0.583	1.023
Angola	Landana	2017	0.089	0.149	0.262
Angola	Landana	2000-2017	-0.095	-0.080	-0.065
Angola	Léua	2000	0.268	0.468	0.798
Angola	Léua	2017	0.084	0.146	0.249
Angola	Léua	2000-2017	-0.081	-0.071	-0.062
Angola	Libolo	2000	0.453	0.792	1.360
Angola	Libolo	2017	0.086	0.151	0.258
Angola	Libolo	2000-2017	-0.117	-0.098	-0.082
Angola	Lobito	2000	0.304	0.528	0.917
Angola	Lobito	2017	0.077	0.129	0.230
Angola	Lobito	2000-2017	-0.095	-0.082	-0.068
Angola	Londuimbale	2000	0.456	0.806	1.469
Angola	Londuimbale	2017	0.087	0.151	0.266
Angola	Londuimbale	2000-2017	-0.117	-0.098	-0.078
Angola	Longonjo	2000	0.429	0.730	1.269
Angola	Longonjo	2017	0.087	0.150	0.258
Angola	Longonjo	2000-2017	-0.114	-0.095	-0.078
Angola	Luau	2000	0.302	0.522	0.905
Angola	Luau	2017	0.098	0.167	0.284
Angola	Luau	2000-2017	-0.072	-0.064	-0.056
Angola	Lubalo	2000	0.274	0.477	0.809
Angola	Lubalo	2017	0.078	0.138	0.245
Angola	Lubalo	2000-2017	-0.084	-0.074	-0.064
Angola	Lubango	2000	0.299	0.527	0.943
Angola	Lubango	2017	0.082	0.139	0.250
Angola	Lubango	2000-2017	-0.095	-0.081	-0.069
Angola	Lucala	2000	0.417	0.719	1.281
Angola	Lucala	2017	0.085	0.155	0.260
Angola	Lucala	2000-2017	-0.107	-0.092	-0.076
Angola	Lucano	2000	0.257	0.442	0.737
Angola	Lucano	2017	0.084	0.145	0.252
Angola	Lucano	2000-2017	-0.073	-0.066	-0.059
Angola	Lucapa	2000	0.272	0.484	0.815
Angola	Lucapa	2017	0.092	0.159	0.272
Angola	Lucapa	2000-2017	-0.076	-0.068	-0.060
Angola	Luchazes	2000	0.284	0.490	0.838
Angola	Luchazes	2017	0.077	0.137	0.239
Angola	Luchazes	2000-2017	-0.089	-0.078	-0.067
Angola	Lumbala-Nguimbo	2000	0.264	0.455	0.769

Table 1: LRI DALYs rate by unit *(continued)*

Country	Unit	year	mean rate	lower rate	upper rate
Angola	Lumbala-Nguimbo	2017	0.079	0.138	0.239
Angola	Lumbala-Nguimbo	2000-2017	-0.080	-0.072	-0.063
Angola	Luquembo	2000	0.314	0.545	0.934
Angola	Luquembo	2017	0.081	0.144	0.256
Angola	Luquembo	2000-2017	-0.092	-0.078	-0.067
Angola	M'Banza Congo	2000	0.340	0.584	1.018
Angola	M'Banza Congo	2017	0.091	0.155	0.276
Angola	M'Banza Congo	2000-2017	-0.091	-0.080	-0.069
Angola	Maianga	2000	0.193	0.374	0.717
Angola	Maianga	2017	0.073	0.135	0.257
Angola	Maianga	2000-2017	-0.102	-0.062	-0.026
Angola	Malanje	2000	0.313	0.547	0.954
Angola	Malanje	2017	0.089	0.155	0.274
Angola	Malanje	2000-2017	-0.091	-0.078	-0.065
Angola	Maquela do Zombo	2000	0.358	0.622	1.065
Angola	Maquela do Zombo	2017	0.095	0.161	0.280
Angola	Maquela do Zombo	2000-2017	-0.094	-0.081	-0.069
Angola	Marimba	2000	0.338	0.572	0.944
Angola	Marimba	2017	0.106	0.174	0.282
Angola	Marimba	2000-2017	-0.080	-0.070	-0.060
Angola	Massango	2000	0.351	0.606	1.033
Angola	Massango	2017	0.101	0.169	0.282
Angola	Massango	2000-2017	-0.088	-0.076	-0.066
Angola	Matala	2000	0.303	0.533	0.937
Angola	Matala	2017	0.079	0.135	0.229
Angola	Matala	2000-2017	-0.098	-0.084	-0.070
Angola	Mavinga	2000	0.318	0.552	0.949
Angola	Mavinga	2017	0.083	0.144	0.253
Angola	Mavinga	2000-2017	-0.096	-0.083	-0.072
Angola	Menongue	2000	0.342	0.597	1.031
Angola	Menongue	2017	0.080	0.134	0.241
Angola	Menongue	2000-2017	-0.102	-0.088	-0.074
Angola	Milunga	2000	0.354	0.618	1.054
Angola	Milunga	2017	0.097	0.167	0.283
Angola	Milunga	2000-2017	-0.091	-0.079	-0.068
Angola	Moxico	2000	0.285	0.496	0.852
Angola	Moxico	2017	0.081	0.142	0.247
Angola	Moxico	2000-2017	-0.085	-0.075	-0.066
Angola	Mucaba	2000	0.352	0.612	1.073
Angola	Mucaba	2017	0.095	0.164	0.288
Angola	Mucaba	2000-2017	-0.092	-0.078	-0.066
Angola	Mucari	2000	0.320	0.557	0.954
Angola	Mucari	2017	0.099	0.173	0.300
Angola	Mucari	2000-2017	-0.083	-0.072	-0.062
Angola	Muconda	2000	0.288	0.495	0.828
Angola	Muconda	2017	0.092	0.154	0.261
Angola	Muconda	2000-2017	-0.076	-0.068	-0.061
Angola	Mungo	2000	0.455	0.804	1.414
Angola	Mungo	2017	0.090	0.157	0.274
Angola	Mungo	2000-2017	-0.114	-0.095	-0.076
Angola	Mussende	2000	0.410	0.719	1.245
Angola	Mussende	2017	0.086	0.151	0.261
Angola	Mussende	2000-2017	-0.110	-0.093	-0.076
Angola	Muxima	2000	0.337	0.580	1.002
Angola	Muxima	2017	0.086	0.144	0.263
Angola	Muxima	2000-2017	-0.098	-0.083	-0.068
Angola	N'Zeto	2000	0.280	0.491	0.833
Angola	N'Zeto	2017	0.084	0.145	0.259
Angola	N'Zeto	2000-2017	-0.080	-0.071	-0.061
Angola	Namakunde	2000	0.268	0.465	0.785
Angola	Namakunde	2017	0.074	0.129	0.228

Table 1: LRI DALYs rate by unit *(continued)*

Country	Unit	year	mean rate	lower rate	upper rate
Angola	Namakunde	2000-2017	-0.088	-0.079	-0.068
Angola	Nambuangongo	2000	0.350	0.610	1.076
Angola	Nambuangongo	2017	0.083	0.144	0.253
Angola	Nambuangongo	2000-2017	-0.099	-0.086	-0.073
Angola	Namibe	2000	0.276	0.475	0.809
Angola	Namibe	2017	0.075	0.129	0.225
Angola	Namibe	2000-2017	-0.086	-0.077	-0.066
Angola	Nancova	2000	0.328	0.572	0.984
Angola	Nancova	2017	0.082	0.144	0.248
Angola	Nancova	2000-2017	-0.101	-0.086	-0.073
Angola	Negage	2000	0.353	0.610	1.078
Angola	Negage	2017	0.089	0.158	0.285
Angola	Negage	2000-2017	-0.093	-0.080	-0.067
Angola	Ngonguembo	2000	0.416	0.719	1.248
Angola	Ngonguembo	2017	0.087	0.150	0.259
Angola	Ngonguembo	2000-2017	-0.110	-0.093	-0.076
Angola	Nharea	2000	0.395	0.694	1.226
Angola	Nharea	2017	0.081	0.141	0.243
Angola	Nharea	2000-2017	-0.117	-0.096	-0.079
Angola	Noqui	2000	0.325	0.489	0.747
Angola	Noqui	2017	0.099	0.152	0.252
Angola	Noqui	2000-2017	-0.083	-0.069	-0.056
Angola	Ombadja	2000	0.273	0.456	0.789
Angola	Ombadja	2017	0.073	0.129	0.232
Angola	Ombadja	2000-2017	-0.087	-0.077	-0.067
Angola	Pango Aluquém	2000	0.422	0.733	1.275
Angola	Pango Aluquém	2017	0.081	0.144	0.249
Angola	Pango Aluquém	2000-2017	-0.112	-0.094	-0.077
Angola	Porto Amboim	2000	0.412	0.714	1.259
Angola	Porto Amboim	2017	0.082	0.142	0.261
Angola	Porto Amboim	2000-2017	-0.111	-0.094	-0.076
Angola	Puri	2000	0.348	0.612	1.062
Angola	Puri	2017	0.095	0.166	0.285
Angola	Puri	2000-2017	-0.092	-0.079	-0.066
Angola	Quela	2000	0.317	0.556	0.967
Angola	Quela	2017	0.097	0.167	0.287
Angola	Quela	2000-2017	-0.085	-0.073	-0.062
Angola	Quibala	2000	0.418	0.735	1.267
Angola	Quibala	2017	0.085	0.150	0.255
Angola	Quibala	2000-2017	-0.109	-0.093	-0.077
Angola	Quiculungo	2000	0.404	0.733	1.324
Angola	Quiculungo	2017	0.094	0.163	0.278
Angola	Quiculungo	2000-2017	-0.109	-0.089	-0.069
Angola	Quilenda	2000	0.436	0.779	1.331
Angola	Quilenda	2017	0.093	0.159	0.282
Angola	Quilenda	2000-2017	-0.113	-0.094	-0.075
Angola	Quilengues	2000	0.318	0.552	0.971
Angola	Quilengues	2017	0.079	0.139	0.247
Angola	Quilengues	2000-2017	-0.095	-0.083	-0.071
Angola	Quimbele	2000	0.367	0.626	1.062
Angola	Quimbele	2017	0.098	0.165	0.277
Angola	Quimbele	2000-2017	-0.092	-0.081	-0.069
Angola	Quirima	2000	0.266	0.466	0.794
Angola	Quirima	2017	0.080	0.142	0.247
Angola	Quirima	2000-2017	-0.080	-0.070	-0.061
Angola	Quitexe	2000	0.344	0.610	1.071
Angola	Quitexe	2017	0.084	0.144	0.249
Angola	Quitexe	2000-2017	-0.099	-0.085	-0.071
Angola	Rangel	2000	0.202	0.373	0.737
Angola	Rangel	2017	0.066	0.134	0.261
Angola	Rangel	2000-2017	-0.104	-0.065	-0.024

Table 1: LRI DALYs rate by unit *(continued)*

Country	Unit	year	mean rate	lower rate	upper rate
Angola	Rivungo	2000	0.304	0.529	0.900
Angola	Rivungo	2017	0.084	0.147	0.255
Angola	Rivungo	2000-2017	-0.087	-0.077	-0.069
Angola	Samba	2000	0.250	0.443	0.787
Angola	Samba	2017	0.077	0.132	0.233
Angola	Samba	2000-2017	-0.087	-0.071	-0.055
Angola	Samba Cajú	2000	0.405	0.707	1.224
Angola	Samba Cajú	2017	0.089	0.152	0.259
Angola	Samba Cajú	2000-2017	-0.106	-0.091	-0.076
Angola	Sambizanga	2000	0.205	0.372	0.706
Angola	Sambizanga	2017	0.075	0.136	0.263
Angola	Sambizanga	2000-2017	-0.096	-0.064	-0.030
Angola	Sanza Pombo	2000	0.351	0.618	1.058
Angola	Sanza Pombo	2017	0.094	0.163	0.278
Angola	Sanza Pombo	2000-2017	-0.093	-0.080	-0.068
Angola	Saurimo	2000	0.289	0.500	0.845
Angola	Saurimo	2017	0.092	0.159	0.275
Angola	Saurimo	2000-2017	-0.074	-0.067	-0.059
Angola	Seles	2000	0.420	0.732	1.257
Angola	Seles	2017	0.088	0.155	0.268
Angola	Seles	2000-2017	-0.109	-0.092	-0.077
Angola	Songo	2000	0.345	0.608	1.055
Angola	Songo	2017	0.089	0.154	0.267
Angola	Songo	2000-2017	-0.093	-0.081	-0.068
Angola	Soyo	2000	0.297	0.502	0.823
Angola	Soyo	2017	0.089	0.146	0.263
Angola	Soyo	2000-2017	-0.085	-0.072	-0.060
Angola	Sumbe	2000	0.410	0.719	1.281
Angola	Sumbe	2017	0.083	0.139	0.249
Angola	Sumbe	2000-2017	-0.114	-0.096	-0.078
Angola	Tchicala-Tcholoanga	2000	0.407	0.697	1.212
Angola	Tchicala-Tcholoanga	2017	0.089	0.153	0.264
Angola	Tchicala-Tcholoanga	2000-2017	-0.108	-0.091	-0.075
Angola	Tchindjenje	2000	0.388	0.703	1.201
Angola	Tchindjenje	2017	0.086	0.148	0.269
Angola	Tchindjenje	2000-2017	-0.113	-0.094	-0.075
Angola	Tchipungo	2000	0.311	0.537	0.934
Angola	Tchipungo	2017	0.081	0.139	0.244
Angola	Tchipungo	2000-2017	-0.099	-0.084	-0.072
Angola	Tomboco	2000	0.279	0.486	0.820
Angola	Tomboco	2017	0.087	0.146	0.258
Angola	Tomboco	2000-2017	-0.080	-0.070	-0.060
Angola	Tombwa	2000	0.265	0.456	0.789
Angola	Tombwa	2017	0.079	0.138	0.243
Angola	Tombwa	2000-2017	-0.078	-0.072	-0.064
Angola	Uíge	2000	0.357	0.606	1.057
Angola	Uíge	2017	0.081	0.139	0.261
Angola	Uíge	2000-2017	-0.099	-0.086	-0.071
Angola	Ukuma	2000	0.424	0.742	1.343
Angola	Ukuma	2017	0.088	0.152	0.264
Angola	Ukuma	2000-2017	-0.113	-0.095	-0.078
Angola	Viana	2000	0.268	0.458	0.810
Angola	Viana	2017	0.077	0.133	0.244
Angola	Viana	2000-2017	-0.087	-0.074	-0.062
Angola	Virei	2000	0.295	0.514	0.904
Angola	Virei	2017	0.083	0.144	0.253
Angola	Virei	2000-2017	-0.087	-0.077	-0.068
Angola	Waku Kungo	2000	0.454	0.806	1.416
Angola	Waku Kungo	2017	0.092	0.157	0.273
Angola	Waku Kungo	2000-2017	-0.114	-0.096	-0.078
Angola	Xá Muteba	2000	0.279	0.486	0.813

Table 1: LRI DALYs rate by unit *(continued)*

Country	Unit	year	mean rate	lower rate	upper rate
Angola	Xá Muteba	2017	0.085	0.151	0.261
Angola	Xá Muteba	2000-2017	-0.079	-0.069	-0.060
Benin	Abomey	2000	0.238	0.378	0.530
Benin	Abomey	2017	0.105	0.170	0.245
Benin	Abomey	2000-2017	-0.071	-0.043	-0.013
Benin	Abomey-Calavi	2000	0.194	0.317	0.465
Benin	Abomey-Calavi	2017	0.099	0.161	0.245
Benin	Abomey-Calavi	2000-2017	-0.066	-0.037	-0.006
Benin	Adja-Ouèrè	2000	0.230	0.370	0.554
Benin	Adja-Ouèrè	2017	0.104	0.168	0.260
Benin	Adja-Ouèrè	2000-2017	-0.068	-0.043	-0.017
Benin	Adjarra	2000	0.170	0.292	0.446
Benin	Adjarra	2017	0.093	0.155	0.240
Benin	Adjarra	2000-2017	-0.071	-0.035	0.005
Benin	Adjohoun	2000	0.232	0.371	0.566
Benin	Adjohoun	2017	0.103	0.167	0.257
Benin	Adjohoun	2000-2017	-0.073	-0.044	-0.013
Benin	Agbangnizoun	2000	0.247	0.387	0.567
Benin	Agbangnizoun	2017	0.112	0.182	0.278
Benin	Agbangnizoun	2000-2017	-0.066	-0.041	-0.012
Benin	Aguégués	2000	0.193	0.314	0.466
Benin	Aguégués	2017	0.103	0.162	0.247
Benin	Aguégués	2000-2017	-0.068	-0.037	-0.004
Benin	Akpro-Missérété	2000	0.183	0.305	0.461
Benin	Akpro-Missérété	2017	0.097	0.159	0.253
Benin	Akpro-Missérété	2000-2017	-0.072	-0.036	-0.003
Benin	Allada	2000	0.238	0.383	0.561
Benin	Allada	2017	0.108	0.176	0.269
Benin	Allada	2000-2017	-0.070	-0.042	-0.017
Benin	Aplahoué	2000	0.244	0.385	0.552
Benin	Aplahoué	2017	0.110	0.180	0.260
Benin	Aplahoué	2000-2017	-0.066	-0.041	-0.015
Benin	Athiémé	2000	0.234	0.376	0.553
Benin	Athiémé	2017	0.102	0.167	0.243
Benin	Athiémé	2000-2017	-0.074	-0.044	-0.017
Benin	Avrankou	2000	0.175	0.296	0.461
Benin	Avrankou	2017	0.089	0.160	0.237
Benin	Avrankou	2000-2017	-0.076	-0.035	-0.003
Benin	Banikoara	2000	0.244	0.400	0.609
Benin	Banikoara	2017	0.136	0.226	0.327
Benin	Banikoara	2000-2017	-0.055	-0.030	-0.006
Benin	Bantè	2000	0.213	0.342	0.493
Benin	Bantè	2017	0.104	0.168	0.246
Benin	Bantè	2000-2017	-0.066	-0.041	-0.014
Benin	Bassila	2000	0.230	0.368	0.542
Benin	Bassila	2017	0.117	0.186	0.269
Benin	Bassila	2000-2017	-0.063	-0.037	-0.011
Benin	Bembéréké	2000	0.233	0.388	0.572
Benin	Bembéréké	2017	0.131	0.208	0.296
Benin	Bembéréké	2000-2017	-0.057	-0.034	-0.010
Benin	Bohicon	2000	0.219	0.341	0.512
Benin	Bohicon	2017	0.103	0.167	0.258
Benin	Bohicon	2000-2017	-0.073	-0.041	-0.010
Benin	Bonou	2000	0.232	0.375	0.562
Benin	Bonou	2017	0.104	0.170	0.260
Benin	Bonou	2000-2017	-0.071	-0.043	-0.015
Benin	Bopa	2000	0.235	0.368	0.546
Benin	Bopa	2017	0.102	0.167	0.255
Benin	Bopa	2000-2017	-0.069	-0.042	-0.013
Benin	Boukoumbé	2000	0.260	0.402	0.613
Benin	Boukoumbé	2017	0.134	0.215	0.312

Table 1: LRI DALYs rate by unit *(continued)*

Country	Unit	year	mean rate	lower rate	upper rate
Benin	Boukoumbé	2000-2017	-0.060	-0.035	-0.011
Benin	Cobly	2000	0.254	0.406	0.610
Benin	Cobly	2017	0.131	0.217	0.315
Benin	Cobly	2000-2017	-0.060	-0.035	-0.010
Benin	Comè	2000	0.213	0.333	0.505
Benin	Comè	2017	0.099	0.162	0.250
Benin	Comè	2000-2017	-0.068	-0.038	-0.008
Benin	Copargo	2000	0.233	0.373	0.541
Benin	Copargo	2017	0.120	0.195	0.289
Benin	Copargo	2000-2017	-0.061	-0.036	-0.010
Benin	Cotonou	2000	0.172	0.292	0.451
Benin	Cotonou	2017	0.090	0.158	0.255
Benin	Cotonou	2000-2017	-0.078	-0.033	0.011
Benin	Covè	2000	0.229	0.366	0.546
Benin	Covè	2017	0.109	0.173	0.267
Benin	Covè	2000-2017	-0.069	-0.041	-0.015
Benin	Dangbo	2000	0.203	0.330	0.494
Benin	Dangbo	2017	0.099	0.160	0.238
Benin	Dangbo	2000-2017	-0.067	-0.039	-0.007
Benin	Dassa-Zoumè	2000	0.211	0.338	0.500
Benin	Dassa-Zoumè	2017	0.103	0.161	0.242
Benin	Dassa-Zoumè	2000-2017	-0.069	-0.041	-0.013
Benin	Djakotomey	2000	0.245	0.376	0.554
Benin	Djakotomey	2017	0.101	0.166	0.257
Benin	Djakotomey	2000-2017	-0.071	-0.043	-0.016
Benin	Djidja	2000	0.241	0.375	0.551
Benin	Djidja	2017	0.114	0.179	0.264
Benin	Djidja	2000-2017	-0.064	-0.041	-0.016
Benin	Djougou	2000	0.242	0.388	0.572
Benin	Djougou	2017	0.122	0.196	0.286
Benin	Djougou	2000-2017	-0.061	-0.036	-0.013
Benin	Dogbo	2000	0.247	0.383	0.566
Benin	Dogbo	2017	0.101	0.167	0.249
Benin	Dogbo	2000-2017	-0.072	-0.043	-0.011
Benin	Glazoué	2000	0.225	0.353	0.508
Benin	Glazoué	2017	0.104	0.162	0.240
Benin	Glazoué	2000-2017	-0.069	-0.044	-0.018
Benin	Gogounou	2000	0.248	0.400	0.593
Benin	Gogounou	2017	0.138	0.221	0.319
Benin	Gogounou	2000-2017	-0.056	-0.032	-0.008
Benin	Grand-Popo	2000	0.212	0.339	0.502
Benin	Grand-Popo	2017	0.100	0.160	0.240
Benin	Grand-Popo	2000-2017	-0.068	-0.040	-0.008
Benin	Houéyogbé	2000	0.230	0.363	0.542
Benin	Houéyogbé	2017	0.100	0.165	0.257
Benin	Houéyogbé	2000-2017	-0.071	-0.043	-0.015
Benin	Ifangni	2000	0.201	0.318	0.471
Benin	Ifangni	2017	0.098	0.157	0.237
Benin	Ifangni	2000-2017	-0.069	-0.039	-0.009
Benin	Kalalé	2000	0.233	0.384	0.586
Benin	Kalalé	2017	0.127	0.209	0.313
Benin	Kalalé	2000-2017	-0.057	-0.033	-0.010
Benin	Kandi	2000	0.243	0.395	0.610
Benin	Kandi	2017	0.137	0.222	0.326
Benin	Kandi	2000-2017	-0.054	-0.030	-0.007
Benin	Karimama	2000	0.298	0.455	0.696
Benin	Karimama	2017	0.151	0.248	0.365
Benin	Karimama	2000-2017	-0.057	-0.033	-0.010
Benin	Kérou	2000	0.263	0.415	0.617
Benin	Kérou	2017	0.143	0.225	0.330
Benin	Kérou	2000-2017	-0.057	-0.033	-0.011

Table 1: LRI DALYs rate by unit *(continued)*

Country	Unit	year	mean rate	lower rate	upper rate
Benin	Kétou	2000	0.226	0.356	0.525
Benin	Kétou	2017	0.105	0.170	0.259
Benin	Kétou	2000-2017	-0.065	-0.040	-0.014
Benin	Klouékanmè	2000	0.244	0.388	0.582
Benin	Klouékanmè	2017	0.109	0.178	0.263
Benin	Klouékanmè	2000-2017	-0.065	-0.042	-0.012
Benin	Kouandé	2000	0.249	0.406	0.606
Benin	Kouandé	2017	0.130	0.210	0.304
Benin	Kouandé	2000-2017	-0.059	-0.036	-0.014
Benin	Kpomassè	2000	0.222	0.348	0.503
Benin	Kpomassè	2017	0.101	0.162	0.246
Benin	Kpomassè	2000-2017	-0.069	-0.041	-0.009
Benin	Lalo	2000	0.244	0.383	0.567
Benin	Lalo	2017	0.111	0.176	0.264
Benin	Lalo	2000-2017	-0.069	-0.042	-0.016
Benin	Malanville	2000	0.265	0.414	0.650
Benin	Malanville	2017	0.142	0.235	0.351
Benin	Malanville	2000-2017	-0.054	-0.030	-0.004
Benin	Matéri	2000	0.261	0.414	0.614
Benin	Matéri	2017	0.136	0.221	0.322
Benin	Matéri	2000-2017	-0.058	-0.034	-0.010
Benin	N'Dali	2000	0.238	0.384	0.558
Benin	N'Dali	2017	0.131	0.204	0.287
Benin	N'Dali	2000-2017	-0.060	-0.035	-0.009
Benin	Natitingou	2000	0.244	0.395	0.591
Benin	Natitingou	2017	0.124	0.201	0.295
Benin	Natitingou	2000-2017	-0.062	-0.037	-0.015
Benin	Nikki	2000	0.227	0.377	0.560
Benin	Nikki	2017	0.122	0.196	0.287
Benin	Nikki	2000-2017	-0.061	-0.036	-0.012
Benin	Ouaké	2000	0.215	0.334	0.489
Benin	Ouaké	2017	0.114	0.178	0.257
Benin	Ouaké	2000-2017	-0.062	-0.036	-0.011
Benin	Ouèssè	2000	0.197	0.315	0.467
Benin	Ouèssè	2017	0.101	0.164	0.238
Benin	Ouèssè	2000-2017	-0.062	-0.037	-0.012
Benin	Ouidah	2000	0.204	0.338	0.506
Benin	Ouidah	2017	0.104	0.163	0.247
Benin	Ouidah	2000-2017	-0.068	-0.040	-0.010
Benin	Ouinhi	2000	0.227	0.366	0.547
Benin	Ouinhi	2017	0.105	0.163	0.250
Benin	Ouinhi	2000-2017	-0.068	-0.044	-0.013
Benin	Parakou	2000	0.226	0.364	0.536
Benin	Parakou	2017	0.111	0.178	0.257
Benin	Parakou	2000-2017	-0.067	-0.040	-0.010
Benin	Péhunco	2000	0.250	0.400	0.585
Benin	Péhunco	2017	0.130	0.209	0.304
Benin	Péhunco	2000-2017	-0.059	-0.035	-0.011
Benin	Pèrèrè	2000	0.231	0.375	0.557
Benin	Pèrèrè	2017	0.124	0.199	0.290
Benin	Pèrèrè	2000-2017	-0.060	-0.035	-0.011
Benin	Pobè	2000	0.234	0.363	0.555
Benin	Pobè	2017	0.104	0.170	0.260
Benin	Pobè	2000-2017	-0.067	-0.041	-0.013
Benin	Porto-Novo	2000	0.161	0.289	0.453
Benin	Porto-Novo	2017	0.097	0.155	0.253
Benin	Porto-Novo	2000-2017	-0.074	-0.035	0.001
Benin	Sakété	2000	0.225	0.359	0.544
Benin	Sakété	2017	0.103	0.162	0.251
Benin	Sakété	2000-2017	-0.071	-0.044	-0.018
Benin	Savalou	2000	0.219	0.342	0.501

Table 1: LRI DALYs rate by unit *(continued)*

Country	Unit	year	mean rate	lower rate	upper rate
Benin	Savalou	2017	0.102	0.163	0.237
Benin	Savalou	2000-2017	-0.064	-0.041	-0.016
Benin	Savè	2000	0.208	0.329	0.482
Benin	Savè	2017	0.105	0.166	0.249
Benin	Savè	2000-2017	-0.064	-0.040	-0.016
Benin	Segbana	2000	0.239	0.393	0.606
Benin	Segbana	2017	0.135	0.225	0.335
Benin	Segbana	2000-2017	-0.050	-0.030	-0.004
Benin	Sèmè-Kpodji	2000	0.175	0.297	0.455
Benin	Sèmè-Kpodji	2017	0.101	0.160	0.240
Benin	Sèmè-Kpodji	2000-2017	-0.074	-0.037	-0.007
Benin	Sinendé	2000	0.241	0.387	0.577
Benin	Sinendé	2017	0.131	0.208	0.294
Benin	Sinendé	2000-2017	-0.056	-0.034	-0.010
Benin	Sô-Ava	2000	0.169	0.301	0.459
Benin	Sô-Ava	2017	0.093	0.158	0.246
Benin	Sô-Ava	2000-2017	-0.070	-0.035	0.008
Benin	Tanguiéta	2000	0.264	0.418	0.630
Benin	Tanguiéta	2017	0.140	0.225	0.333
Benin	Tanguiéta	2000-2017	-0.059	-0.034	-0.010
Benin	Tchaourou	2000	0.245	0.378	0.551
Benin	Tchaourou	2017	0.123	0.196	0.280
Benin	Tchaourou	2000-2017	-0.061	-0.036	-0.011
Benin	Toffo	2000	0.234	0.376	0.555
Benin	Toffo	2017	0.107	0.171	0.261
Benin	Toffo	2000-2017	-0.069	-0.042	-0.013
Benin	Tori-Bossito	2000	0.242	0.380	0.551
Benin	Tori-Bossito	2017	0.108	0.174	0.255
Benin	Tori-Bossito	2000-2017	-0.070	-0.042	-0.013
Benin	Toucountouna	2000	0.260	0.409	0.607
Benin	Toucountouna	2017	0.130	0.212	0.305
Benin	Toucountouna	2000-2017	-0.061	-0.035	-0.010
Benin	Toviklin	2000	0.230	0.378	0.575
Benin	Toviklin	2017	0.100	0.170	0.268
Benin	Toviklin	2000-2017	-0.071	-0.042	-0.006
Benin	Za-Kpota	2000	0.225	0.369	0.559
Benin	Za-Kpota	2017	0.112	0.181	0.279
Benin	Za-Kpota	2000-2017	-0.069	-0.039	-0.012
Benin	Zagnanado	2000	0.224	0.361	0.538
Benin	Zagnanado	2017	0.103	0.166	0.255
Benin	Zagnanado	2000-2017	-0.069	-0.043	-0.016
Benin	Zè	2000	0.241	0.381	0.565
Benin	Zè	2017	0.108	0.174	0.271
Benin	Zè	2000-2017	-0.069	-0.043	-0.015
Benin	Zogbodomey	2000	0.239	0.374	0.550
Benin	Zogbodomey	2017	0.108	0.171	0.245
Benin	Zogbodomey	2000-2017	-0.067	-0.043	-0.017
Botswana	Barolong	2000	0.027	0.075	0.149
Botswana	Barolong	2017	0.014	0.039	0.090
Botswana	Barolong	2000-2017	-0.063	-0.037	-0.013
Botswana	Bobonong	2000	0.027	0.076	0.146
Botswana	Bobonong	2017	0.015	0.042	0.095
Botswana	Bobonong	2000-2017	-0.060	-0.034	-0.005
Botswana	Chobe	2000	0.034	0.083	0.159
Botswana	Chobe	2017	0.017	0.043	0.090
Botswana	Chobe	2000-2017	-0.066	-0.036	-0.007
Botswana	Francistown	2000	0.023	0.071	0.161
Botswana	Francistown	2017	0.013	0.036	0.083
Botswana	Francistown	2000-2017	-0.087	-0.038	0.004
Botswana	Gaborone	2000	0.023	0.067	0.148
Botswana	Gaborone	2017	0.012	0.034	0.066

Table 1: LRI DALYs rate by unit *(continued)*

Country	Unit	year	mean rate	lower rate	upper rate
Botswana	Gaborone	2000-2017	-0.068	-0.037	-0.006
Botswana	Gemsbok	2000	0.030	0.075	0.139
Botswana	Gemsbok	2017	0.016	0.042	0.093
Botswana	Gemsbok	2000-2017	-0.059	-0.031	-0.002
Botswana	Ghanzi	2000	0.025	0.073	0.144
Botswana	Ghanzi	2017	0.017	0.045	0.097
Botswana	Ghanzi	2000-2017	-0.061	-0.025	0.008
Botswana	Hukunsti	2000	0.026	0.073	0.143
Botswana	Hukunsti	2017	0.016	0.044	0.101
Botswana	Hukunsti	2000-2017	-0.058	-0.027	0.003
Botswana	Jwaneng	2000	0.021	0.069	0.155
Botswana	Jwaneng	2017	0.012	0.039	0.097
Botswana	Jwaneng	2000-2017	-0.095	-0.029	0.053
Botswana	Kgatleng	2000	0.028	0.074	0.147
Botswana	Kgatleng	2017	0.015	0.040	0.088
Botswana	Kgatleng	2000-2017	-0.062	-0.035	-0.012
Botswana	Kweneng North	2000	0.026	0.073	0.144
Botswana	Kweneng North	2017	0.014	0.039	0.087
Botswana	Kweneng North	2000-2017	-0.063	-0.035	-0.012
Botswana	Kweneng South	2000	0.026	0.074	0.140
Botswana	Kweneng South	2017	0.014	0.040	0.087
Botswana	Kweneng South	2000-2017	-0.064	-0.034	-0.007
Botswana	Lethlakane	2000	0.031	0.085	0.167
Botswana	Lethlakane	2017	0.016	0.046	0.106
Botswana	Lethlakane	2000-2017	-0.064	-0.034	-0.007
Botswana	Lobatse	2000	0.022	0.069	0.151
Botswana	Lobatse	2017	0.011	0.035	0.076
Botswana	Lobatse	2000-2017	-0.098	-0.037	0.021
Botswana	Machaneng	2000	0.028	0.078	0.155
Botswana	Machaneng	2017	0.015	0.042	0.102
Botswana	Machaneng	2000-2017	-0.062	-0.036	-0.012
Botswana	Mahalapye	2000	0.029	0.079	0.166
Botswana	Mahalapye	2017	0.015	0.044	0.101
Botswana	Mahalapye	2000-2017	-0.063	-0.035	-0.011
Botswana	Masungu	2000	0.025	0.071	0.141
Botswana	Masungu	2017	0.014	0.038	0.082
Botswana	Masungu	2000-2017	-0.065	-0.036	-0.011
Botswana	Ngamiland East	2000	0.034	0.093	0.177
Botswana	Ngamiland East	2017	0.017	0.048	0.108
Botswana	Ngamiland East	2000-2017	-0.065	-0.036	-0.011
Botswana	Ngamiland West	2000	0.031	0.085	0.165
Botswana	Ngamiland West	2017	0.016	0.048	0.116
Botswana	Ngamiland West	2000-2017	-0.063	-0.032	-0.002
Botswana	Ngwaketse Central	2000	0.027	0.074	0.147
Botswana	Ngwaketse Central	2017	0.014	0.039	0.087
Botswana	Ngwaketse Central	2000-2017	-0.061	-0.036	-0.016
Botswana	Ngwaketse North	2000	0.026	0.074	0.149
Botswana	Ngwaketse North	2017	0.014	0.040	0.092
Botswana	Ngwaketse North	2000-2017	-0.065	-0.035	-0.009
Botswana	Ngwaketse South	2000	0.029	0.077	0.151
Botswana	Ngwaketse South	2017	0.015	0.039	0.090
Botswana	Ngwaketse South	2000-2017	-0.061	-0.038	-0.015
Botswana	Palapye	2000	0.028	0.077	0.157
Botswana	Palapye	2017	0.015	0.041	0.095
Botswana	Palapye	2000-2017	-0.065	-0.036	-0.010
Botswana	Selibe Phikwe	2000	0.023	0.072	0.158
Botswana	Selibe Phikwe	2017	0.012	0.037	0.088
Botswana	Selibe Phikwe	2000-2017	-0.093	-0.037	0.020
Botswana	Serowe	2000	0.028	0.076	0.154
Botswana	Serowe	2017	0.015	0.042	0.103
Botswana	Serowe	2000-2017	-0.061	-0.034	-0.009

Table 1: LRI DALYs rate by unit *(continued)*

Country	Unit	year	mean rate	lower rate	upper rate
Botswana	South East	2000	0.024	0.068	0.147
Botswana	South East	2017	0.013	0.034	0.072
Botswana	South East	2000-2017	-0.063	-0.036	-0.013
Botswana	Sowa	2000	0.021	0.070	0.173
Botswana	Sowa	2017	0.012	0.042	0.104
Botswana	Sowa	2000-2017	-0.108	-0.029	0.061
Botswana	Tshabong	2000	0.029	0.076	0.147
Botswana	Tshabong	2017	0.016	0.045	0.101
Botswana	Tshabong	2000-2017	-0.058	-0.028	-0.004
Botswana	Tuli	2000	0.033	0.081	0.163
Botswana	Tuli	2017	0.016	0.043	0.099
Botswana	Tuli	2000-2017	-0.064	-0.037	-0.011
Botswana	Tutume	2000	0.029	0.079	0.160
Botswana	Tutume	2017	0.016	0.046	0.115
Botswana	Tutume	2000-2017	-0.059	-0.031	-0.004
Burkina Faso	Balé	2000	0.312	0.497	0.795
Burkina Faso	Balé	2017	0.154	0.270	0.410
Burkina Faso	Balé	2000-2017	-0.061	-0.032	-0.001
Burkina Faso	Bam	2000	0.319	0.528	0.843
Burkina Faso	Bam	2017	0.169	0.295	0.462
Burkina Faso	Bam	2000-2017	-0.064	-0.035	-0.006
Burkina Faso	Banwa	2000	0.263	0.429	0.693
Burkina Faso	Banwa	2017	0.149	0.262	0.417
Burkina Faso	Banwa	2000-2017	-0.054	-0.027	0.003
Burkina Faso	Bazèga	2000	0.319	0.529	0.816
Burkina Faso	Bazèga	2017	0.160	0.285	0.461
Burkina Faso	Bazèga	2000-2017	-0.069	-0.039	-0.010
Burkina Faso	Bougouriba	2000	0.318	0.511	0.819
Burkina Faso	Bougouriba	2017	0.159	0.272	0.442
Burkina Faso	Bougouriba	2000-2017	-0.063	-0.034	-0.006
Burkina Faso	Boulgou	2000	0.323	0.516	0.782
Burkina Faso	Boulgou	2017	0.163	0.275	0.447
Burkina Faso	Boulgou	2000-2017	-0.065	-0.036	-0.013
Burkina Faso	Boulkiemdé	2000	0.298	0.500	0.769
Burkina Faso	Boulkiemdé	2017	0.161	0.277	0.436
Burkina Faso	Boulkiemdé	2000-2017	-0.065	-0.037	-0.007
Burkina Faso	Comoé	2000	0.305	0.487	0.842
Burkina Faso	Comoé	2017	0.147	0.262	0.419
Burkina Faso	Comoé	2000-2017	-0.063	-0.037	-0.010
Burkina Faso	Ganzourgou	2000	0.315	0.513	0.801
Burkina Faso	Ganzourgou	2017	0.163	0.280	0.456
Burkina Faso	Ganzourgou	2000-2017	-0.066	-0.036	-0.011
Burkina Faso	Gnagna	2000	0.325	0.542	0.834
Burkina Faso	Gnagna	2017	0.176	0.296	0.468
Burkina Faso	Gnagna	2000-2017	-0.065	-0.035	-0.011
Burkina Faso	Gourma	2000	0.322	0.540	0.836
Burkina Faso	Gourma	2017	0.166	0.296	0.486
Burkina Faso	Gourma	2000-2017	-0.065	-0.035	-0.012
Burkina Faso	Houet	2000	0.299	0.489	0.800
Burkina Faso	Houet	2017	0.152	0.266	0.418
Burkina Faso	Houet	2000-2017	-0.065	-0.036	-0.009
Burkina Faso	Ioba	2000	0.323	0.502	0.753
Burkina Faso	Ioba	2017	0.155	0.267	0.430
Burkina Faso	Ioba	2000-2017	-0.063	-0.035	-0.006
Burkina Faso	Kadiogo	2000	0.241	0.402	0.644
Burkina Faso	Kadiogo	2017	0.135	0.242	0.389
Burkina Faso	Kadiogo	2000-2017	-0.058	-0.030	0.002
Burkina Faso	Kénédougou	2000	0.302	0.481	0.807
Burkina Faso	Kénédougou	2017	0.145	0.259	0.411
Burkina Faso	Kénédougou	2000-2017	-0.064	-0.036	-0.005
Burkina Faso	Komandjoari	2000	0.338	0.554	0.862

Table 1: LRI DALYs rate by unit *(continued)*

Country	Unit	year	mean rate	lower rate	upper rate
Burkina Faso	Komandjoari	2017	0.169	0.302	0.484
Burkina Faso	Komandjoari	2000-2017	-0.067	-0.037	-0.015
Burkina Faso	Kompienga	2000	0.319	0.527	0.835
Burkina Faso	Kompienga	2017	0.162	0.285	0.456
Burkina Faso	Kompienga	2000-2017	-0.065	-0.038	-0.012
Burkina Faso	Kossi	2000	0.263	0.433	0.689
Burkina Faso	Kossi	2017	0.157	0.274	0.441
Burkina Faso	Kossi	2000-2017	-0.056	-0.025	0.005
Burkina Faso	Koulpélogo	2000	0.320	0.525	0.786
Burkina Faso	Koulpélogo	2017	0.162	0.285	0.456
Burkina Faso	Koulpélogo	2000-2017	-0.069	-0.037	-0.014
Burkina Faso	Kouritenga	2000	0.324	0.527	0.805
Burkina Faso	Kouritenga	2017	0.159	0.281	0.450
Burkina Faso	Kouritenga	2000-2017	-0.066	-0.037	-0.013
Burkina Faso	Kourwéogo	2000	0.277	0.464	0.736
Burkina Faso	Kourwéogo	2017	0.165	0.287	0.462
Burkina Faso	Kourwéogo	2000-2017	-0.059	-0.031	-0.001
Burkina Faso	Léraba	2000	0.299	0.481	0.810
Burkina Faso	Léraba	2017	0.148	0.258	0.411
Burkina Faso	Léraba	2000-2017	-0.065	-0.037	-0.009
Burkina Faso	Loroum	2000	0.319	0.528	0.824
Burkina Faso	Loroum	2017	0.168	0.296	0.472
Burkina Faso	Loroum	2000-2017	-0.062	-0.033	-0.002
Burkina Faso	Mouhoun	2000	0.278	0.454	0.738
Burkina Faso	Mouhoun	2017	0.156	0.269	0.427
Burkina Faso	Mouhoun	2000-2017	-0.054	-0.027	0.002
Burkina Faso	Nahouri	2000	0.328	0.523	0.801
Burkina Faso	Nahouri	2017	0.169	0.293	0.467
Burkina Faso	Nahouri	2000-2017	-0.061	-0.034	-0.007
Burkina Faso	Namentenga	2000	0.325	0.533	0.853
Burkina Faso	Namentenga	2017	0.177	0.305	0.488
Burkina Faso	Namentenga	2000-2017	-0.059	-0.030	-0.005
Burkina Faso	Nayala	2000	0.264	0.443	0.715
Burkina Faso	Nayala	2017	0.159	0.283	0.456
Burkina Faso	Nayala	2000-2017	-0.054	-0.026	0.007
Burkina Faso	Noumbiel	2000	0.313	0.511	0.813
Burkina Faso	Noumbiel	2017	0.154	0.276	0.443
Burkina Faso	Noumbiel	2000-2017	-0.062	-0.034	-0.003
Burkina Faso	Oubritenga	2000	0.291	0.479	0.768
Burkina Faso	Oubritenga	2017	0.168	0.290	0.463
Burkina Faso	Oubritenga	2000-2017	-0.060	-0.033	-0.006
Burkina Faso	Oudalan	2000	0.359	0.590	0.926
Burkina Faso	Oudalan	2017	0.185	0.330	0.552
Burkina Faso	Oudalan	2000-2017	-0.062	-0.036	-0.009
Burkina Faso	Passoré	2000	0.289	0.483	0.769
Burkina Faso	Passoré	2017	0.166	0.286	0.464
Burkina Faso	Passoré	2000-2017	-0.061	-0.032	0.000
Burkina Faso	Poni	2000	0.310	0.508	0.828
Burkina Faso	Poni	2017	0.153	0.273	0.440
Burkina Faso	Poni	2000-2017	-0.064	-0.035	-0.006
Burkina Faso	Sanguié	2000	0.308	0.504	0.785
Burkina Faso	Sanguié	2017	0.159	0.276	0.437
Burkina Faso	Sanguié	2000-2017	-0.064	-0.036	-0.006
Burkina Faso	Sanmatenga	2000	0.308	0.505	0.811
Burkina Faso	Sanmatenga	2017	0.174	0.300	0.482
Burkina Faso	Sanmatenga	2000-2017	-0.061	-0.033	-0.005
Burkina Faso	Séno	2000	0.359	0.579	0.917
Burkina Faso	Séno	2017	0.181	0.319	0.535
Burkina Faso	Séno	2000-2017	-0.064	-0.036	-0.010
Burkina Faso	Sissili	2000	0.320	0.515	0.765
Burkina Faso	Sissili	2017	0.164	0.284	0.449

Table 1: LRI DALYs rate by unit *(continued)*

Country	Unit	year	mean rate	lower rate	upper rate
Burkina Faso	Sissili	2000-2017	-0.061	-0.031	-0.002
Burkina Faso	Soum	2000	0.339	0.558	0.879
Burkina Faso	Soum	2017	0.180	0.314	0.499
Burkina Faso	Soum	2000-2017	-0.061	-0.033	-0.005
Burkina Faso	Sourou	2000	0.283	0.481	0.757
Burkina Faso	Sourou	2017	0.164	0.288	0.469
Burkina Faso	Sourou	2000-2017	-0.061	-0.031	0.000
Burkina Faso	Tapoa	2000	0.328	0.541	0.868
Burkina Faso	Tapoa	2017	0.163	0.296	0.471
Burkina Faso	Tapoa	2000-2017	-0.061	-0.035	-0.011
Burkina Faso	Tuy	2000	0.321	0.499	0.807
Burkina Faso	Tuy	2017	0.151	0.265	0.409
Burkina Faso	Tuy	2000-2017	-0.066	-0.039	-0.010
Burkina Faso	Yagha	2000	0.356	0.582	0.914
Burkina Faso	Yagha	2017	0.191	0.321	0.519
Burkina Faso	Yagha	2000-2017	-0.066	-0.038	-0.011
Burkina Faso	Yatenga	2000	0.314	0.526	0.826
Burkina Faso	Yatenga	2017	0.171	0.297	0.479
Burkina Faso	Yatenga	2000-2017	-0.063	-0.034	-0.004
Burkina Faso	Ziro	2000	0.324	0.529	0.822
Burkina Faso	Ziro	2017	0.164	0.291	0.469
Burkina Faso	Ziro	2000-2017	-0.063	-0.034	-0.004
Burkina Faso	Zondoma	2000	0.307	0.515	0.807
Burkina Faso	Zondoma	2017	0.170	0.294	0.471
Burkina Faso	Zondoma	2000-2017	-0.062	-0.034	-0.004
Burkina Faso	Zoundwéogo	2000	0.319	0.524	0.791
Burkina Faso	Zoundwéogo	2017	0.157	0.278	0.463
Burkina Faso	Zoundwéogo	2000-2017	-0.066	-0.038	-0.011
Burundi	Bisoro	2000	0.292	0.406	0.566
Burundi	Bisoro	2017	0.135	0.188	0.262
Burundi	Bisoro	2000-2017	-0.062	-0.045	-0.031
Burundi	Bubanza	2000	0.265	0.370	0.513
Burundi	Bubanza	2017	0.140	0.187	0.253
Burundi	Bubanza	2000-2017	-0.056	-0.042	-0.029
Burundi	Bugabira	2000	0.304	0.418	0.571
Burundi	Bugabira	2017	0.152	0.204	0.285
Burundi	Bugabira	2000-2017	-0.058	-0.045	-0.033
Burundi	Buganda	2000	0.256	0.361	0.498
Burundi	Buganda	2017	0.143	0.192	0.264
Burundi	Buganda	2000-2017	-0.049	-0.035	-0.019
Burundi	Bugarama	2000	0.291	0.390	0.537
Burundi	Bugarama	2017	0.130	0.175	0.240
Burundi	Bugarama	2000-2017	-0.063	-0.047	-0.032
Burundi	Bugendana	2000	0.299	0.407	0.563
Burundi	Bugendana	2017	0.150	0.205	0.282
Burundi	Bugendana	2000-2017	-0.055	-0.043	-0.030
Burundi	Bugenyuzi	2000	0.294	0.405	0.564
Burundi	Bugenyuzi	2017	0.154	0.216	0.304
Burundi	Bugenyuzi	2000-2017	-0.051	-0.038	-0.027
Burundi	Buhiga	2000	0.294	0.399	0.552
Burundi	Buhiga	2017	0.166	0.224	0.307
Burundi	Buhiga	2000-2017	-0.049	-0.035	-0.022
Burundi	Buhinyuza	2000	0.300	0.404	0.570
Burundi	Buhinyuza	2017	0.165	0.227	0.317
Burundi	Buhinyuza	2000-2017	-0.046	-0.033	-0.019
Burundi	Bukemba	2000	0.272	0.369	0.519
Burundi	Bukemba	2017	0.131	0.177	0.248
Burundi	Bukemba	2000-2017	-0.056	-0.043	-0.030
Burundi	Bukeye	2000	0.304	0.422	0.591
Burundi	Bukeye	2017	0.142	0.193	0.267
Burundi	Bukeye	2000-2017	-0.064	-0.048	-0.035

Table 1: LRI DALYs rate by unit *(continued)*

Country	Unit	year	mean rate	lower rate	upper rate
Burundi	Bukinanyana	2000	0.301	0.409	0.578
Burundi	Bukinanyana	2017	0.154	0.207	0.285
Burundi	Bukinanyana	2000-2017	-0.051	-0.040	-0.028
Burundi	Bukirasazi	2000	0.267	0.382	0.539
Burundi	Bukirasazi	2017	0.131	0.197	0.275
Burundi	Bukirasazi	2000-2017	-0.064	-0.041	-0.018
Burundi	Burambi	2000	0.267	0.367	0.518
Burundi	Burambi	2017	0.125	0.167	0.229
Burundi	Burambi	2000-2017	-0.061	-0.047	-0.036
Burundi	Buraza	2000	0.286	0.386	0.537
Burundi	Buraza	2017	0.134	0.185	0.255
Burundi	Buraza	2000-2017	-0.062	-0.045	-0.029
Burundi	Bururi	2000	0.282	0.385	0.545
Burundi	Bururi	2017	0.131	0.176	0.245
Burundi	Bururi	2000-2017	-0.059	-0.049	-0.039
Burundi	Busiga	2000	0.311	0.426	0.588
Burundi	Busiga	2017	0.135	0.188	0.262
Burundi	Busiga	2000-2017	-0.068	-0.051	-0.035
Burundi	Busoni	2000	0.296	0.406	0.560
Burundi	Busoni	2017	0.149	0.200	0.275
Burundi	Busoni	2000-2017	-0.054	-0.042	-0.032
Burundi	Butaganzwa1	2000	0.285	0.402	0.539
Burundi	Butaganzwa1	2017	0.139	0.192	0.263
Burundi	Butaganzwa1	2000-2017	-0.061	-0.044	-0.028
Burundi	Butaganzwa2	2000	0.274	0.382	0.528
Burundi	Butaganzwa2	2017	0.149	0.201	0.285
Burundi	Butaganzwa2	2000-2017	-0.048	-0.038	-0.027
Burundi	Buterere	2000	0.221	0.325	0.494
Burundi	Buterere	2017	0.098	0.144	0.215
Burundi	Buterere	2000-2017	-0.083	-0.051	-0.027
Burundi	Butezi	2000	0.284	0.390	0.542
Burundi	Butezi	2017	0.156	0.216	0.309
Burundi	Butezi	2000-2017	-0.045	-0.034	-0.022
Burundi	Butihinda	2000	0.279	0.381	0.519
Burundi	Butihinda	2017	0.161	0.221	0.321
Burundi	Butihinda	2000-2017	-0.043	-0.030	-0.017
Burundi	Buyengero	2000	0.275	0.376	0.524
Burundi	Buyengero	2017	0.125	0.173	0.245
Burundi	Buyengero	2000-2017	-0.062	-0.049	-0.036
Burundi	Buyenze	2000	0.191	0.322	0.510
Burundi	Buyenze	2017	0.093	0.142	0.227
Burundi	Buyenze	2000-2017	-0.091	-0.053	-0.016
Burundi	Bwambarangwe	2000	0.283	0.401	0.567
Burundi	Bwambarangwe	2017	0.148	0.204	0.284
Burundi	Bwambarangwe	2000-2017	-0.053	-0.040	-0.028
Burundi	Bweru	2000	0.271	0.384	0.527
Burundi	Bweru	2017	0.158	0.223	0.315
Burundi	Bweru	2000-2017	-0.040	-0.027	-0.014
Burundi	Bwiza	2000	0.197	0.324	0.503
Burundi	Bwiza	2017	0.095	0.142	0.223
Burundi	Bwiza	2000-2017	-0.087	-0.053	-0.019
Burundi	Cankuzo	2000	0.272	0.379	0.543
Burundi	Cankuzo	2017	0.163	0.224	0.312
Burundi	Cankuzo	2000-2017	-0.036	-0.024	-0.011
Burundi	Cendajuru	2000	0.257	0.351	0.493
Burundi	Cendajuru	2017	0.151	0.215	0.304
Burundi	Cendajuru	2000-2017	-0.036	-0.020	-0.002
Burundi	Cibitoke	2000	0.216	0.320	0.477
Burundi	Cibitoke	2017	0.090	0.140	0.210
Burundi	Cibitoke	2000-2017	-0.087	-0.054	-0.025
Burundi	Gahombo	2000	0.288	0.404	0.568

Table 1: LRI DALYs rate by unit *(continued)*

Country	Unit	year	mean rate	lower rate	upper rate
Burundi	Gahombo	2017	0.136	0.194	0.274
Burundi	Gahombo	2000-2017	-0.066	-0.046	-0.025
Burundi	Gashikanwa	2000	0.281	0.401	0.567
Burundi	Gashikanwa	2017	0.151	0.208	0.291
Burundi	Gashikanwa	2000-2017	-0.056	-0.041	-0.024
Burundi	Gashoho	2000	0.284	0.395	0.565
Burundi	Gashoho	2017	0.162	0.232	0.314
Burundi	Gashoho	2000-2017	-0.048	-0.032	-0.015
Burundi	Gasorwe	2000	0.287	0.402	0.568
Burundi	Gasorwe	2017	0.174	0.233	0.335
Burundi	Gasorwe	2000-2017	-0.049	-0.032	-0.017
Burundi	Gatara	2000	0.285	0.409	0.568
Burundi	Gatara	2017	0.142	0.197	0.277
Burundi	Gatara	2000-2017	-0.063	-0.045	-0.027
Burundi	Gihanga	2000	0.296	0.400	0.538
Burundi	Gihanga	2017	0.126	0.172	0.231
Burundi	Gihanga	2000-2017	-0.062	-0.050	-0.037
Burundi	Giharo	2000	0.268	0.363	0.502
Burundi	Giharo	2017	0.142	0.192	0.271
Burundi	Giharo	2000-2017	-0.046	-0.035	-0.025
Burundi	Giheta	2000	0.282	0.387	0.545
Burundi	Giheta	2017	0.146	0.195	0.266
Burundi	Giheta	2000-2017	-0.056	-0.042	-0.027
Burundi	Gihogazi	2000	0.296	0.413	0.581
Burundi	Gihogazi	2017	0.155	0.211	0.295
Burundi	Gihogazi	2000-2017	-0.056	-0.042	-0.028
Burundi	Gihosha	2000	0.205	0.322	0.485
Burundi	Gihosha	2017	0.084	0.138	0.203
Burundi	Gihosha	2000-2017	-0.092	-0.054	-0.017
Burundi	Gisagara	2000	0.267	0.369	0.498
Burundi	Gisagara	2017	0.159	0.219	0.306
Burundi	Gisagara	2000-2017	-0.035	-0.022	-0.009
Burundi	Gishubi	2000	0.285	0.389	0.564
Burundi	Gishubi	2017	0.138	0.191	0.272
Burundi	Gishubi	2000-2017	-0.060	-0.042	-0.025
Burundi	Gisozi	2000	0.296	0.408	0.581
Burundi	Gisozi	2017	0.140	0.193	0.272
Burundi	Gisozi	2000-2017	-0.062	-0.045	-0.030
Burundi	Gisuru	2000	0.267	0.360	0.516
Burundi	Gisuru	2017	0.149	0.203	0.283
Burundi	Gisuru	2000-2017	-0.037	-0.026	-0.014
Burundi	Gitanga	2000	0.284	0.386	0.553
Burundi	Gitanga	2017	0.131	0.179	0.247
Burundi	Gitanga	2000-2017	-0.059	-0.047	-0.034
Burundi	Gitaramuka	2000	0.289	0.406	0.576
Burundi	Gitaramuka	2017	0.172	0.232	0.335
Burundi	Gitaramuka	2000-2017	-0.049	-0.034	-0.021
Burundi	Gitega	2000	0.260	0.367	0.517
Burundi	Gitega	2017	0.125	0.176	0.244
Burundi	Gitega	2000-2017	-0.061	-0.046	-0.032
Burundi	Giteranyi	2000	0.300	0.399	0.534
Burundi	Giteranyi	2017	0.148	0.198	0.268
Burundi	Giteranyi	2000-2017	-0.052	-0.041	-0.029
Burundi	Gitobe	2000	0.290	0.404	0.573
Burundi	Gitobe	2017	0.161	0.221	0.307
Burundi	Gitobe	2000-2017	-0.048	-0.035	-0.020
Burundi	Isale	2000	0.260	0.356	0.501
Burundi	Isale	2017	0.115	0.159	0.217
Burundi	Isale	2000-2017	-0.064	-0.049	-0.033
Burundi	Itaba	2000	0.280	0.382	0.520
Burundi	Itaba	2017	0.143	0.197	0.278

Table 1: LRI DALYs rate by unit *(continued)*

Country	Unit	year	mean rate	lower rate	upper rate
Burundi	Itaba	2000-2017	-0.053	-0.040	-0.028
Burundi	Kabarore	2000	0.319	0.434	0.602
Burundi	Kabarore	2017	0.144	0.196	0.270
Burundi	Kabarore	2000-2017	-0.062	-0.048	-0.033
Burundi	Kabezi	2000	0.265	0.378	0.513
Burundi	Kabezi	2017	0.121	0.169	0.244
Burundi	Kabezi	2000-2017	-0.068	-0.048	-0.030
Burundi	Kamenge	2000	0.200	0.321	0.493
Burundi	Kamenge	2017	0.082	0.137	0.205
Burundi	Kamenge	2000-2017	-0.094	-0.054	-0.016
Burundi	Kanyosha1	2000	0.259	0.363	0.498
Burundi	Kanyosha1	2017	0.116	0.164	0.230
Burundi	Kanyosha1	2000-2017	-0.068	-0.049	-0.030
Burundi	Kanyosha2	2000	0.241	0.366	0.552
Burundi	Kanyosha2	2017	0.107	0.166	0.258
Burundi	Kanyosha2	2000-2017	-0.083	-0.047	-0.014
Burundi	Kayanza	2000	0.302	0.424	0.562
Burundi	Kayanza	2017	0.141	0.200	0.278
Burundi	Kayanza	2000-2017	-0.063	-0.047	-0.032
Burundi	Kayogoro	2000	0.266	0.362	0.508
Burundi	Kayogoro	2017	0.131	0.176	0.243
Burundi	Kayogoro	2000-2017	-0.052	-0.042	-0.031
Burundi	Kayokwe	2000	0.279	0.399	0.561
Burundi	Kayokwe	2017	0.142	0.190	0.262
Burundi	Kayokwe	2000-2017	-0.065	-0.046	-0.028
Burundi	Kibago	2000	0.267	0.363	0.496
Burundi	Kibago	2017	0.128	0.171	0.241
Burundi	Kibago	2000-2017	-0.055	-0.042	-0.031
Burundi	Kigamba	2000	0.277	0.377	0.518
Burundi	Kigamba	2017	0.160	0.224	0.314
Burundi	Kigamba	2000-2017	-0.036	-0.024	-0.012
Burundi	Kiganda	2000	0.296	0.405	0.561
Burundi	Kiganda	2017	0.134	0.192	0.264
Burundi	Kiganda	2000-2017	-0.062	-0.046	-0.030
Burundi	Kinama	2000	0.235	0.324	0.450
Burundi	Kinama	2017	0.094	0.143	0.203
Burundi	Kinama	2000-2017	-0.077	-0.051	-0.024
Burundi	Kinindo	2000	0.228	0.341	0.526
Burundi	Kinindo	2017	0.100	0.150	0.241
Burundi	Kinindo	2000-2017	-0.082	-0.051	-0.020
Burundi	Kinyinya	2000	0.264	0.370	0.521
Burundi	Kinyinya	2017	0.160	0.218	0.302
Burundi	Kinyinya	2000-2017	-0.039	-0.026	-0.013
Burundi	Kiremba	2000	0.300	0.402	0.557
Burundi	Kiremba	2017	0.163	0.227	0.320
Burundi	Kiremba	2000-2017	-0.048	-0.034	-0.021
Burundi	Kirundo	2000	0.289	0.406	0.572
Burundi	Kirundo	2017	0.152	0.217	0.303
Burundi	Kirundo	2000-2017	-0.052	-0.039	-0.025
Burundi	Lake Tanganyika	2000	0.256	0.358	0.523
Burundi	Lake Tanganyika	2000	0.250	0.346	0.488
Burundi	Lake Tanganyika	2000	0.231	0.338	0.507
Burundi	Lake Tanganyika	2000	0.291	0.398	0.548
Burundi	Lake Tanganyika	2017	0.118	0.167	0.250
Burundi	Lake Tanganyika	2017	0.116	0.165	0.235
Burundi	Lake Tanganyika	2017	0.114	0.159	0.227
Burundi	Lake Tanganyika	2017	0.103	0.149	0.222
Burundi	Lake Tanganyika	2000-2017	-0.060	-0.042	-0.021
Burundi	Lake Tanganyika	2000-2017	-0.060	-0.045	-0.029
Burundi	Lake Tanganyika	2000-2017	-0.067	-0.050	-0.034
Burundi	Lake Tanganyika	2000-2017	-0.076	-0.051	-0.027

Table 1: LRI DALYs rate by unit *(continued)*

Country	Unit	year	mean rate	lower rate	upper rate
Burundi	Mabanda	2000	0.272	0.359	0.499
Burundi	Mabanda	2017	0.123	0.164	0.226
Burundi	Mabanda	2000-2017	-0.058	-0.046	-0.035
Burundi	Mabayi	2000	0.289	0.387	0.530
Burundi	Mabayi	2017	0.140	0.187	0.266
Burundi	Mabayi	2000-2017	-0.053	-0.041	-0.029
Burundi	Makamba	2000	0.278	0.378	0.529
Burundi	Makamba	2017	0.130	0.178	0.245
Burundi	Makamba	2000-2017	-0.061	-0.048	-0.035
Burundi	Makebuko	2000	0.272	0.381	0.519
Burundi	Makebuko	2017	0.136	0.192	0.268
Burundi	Makebuko	2000-2017	-0.056	-0.042	-0.028
Burundi	Marangara	2000	0.299	0.405	0.546
Burundi	Marangara	2017	0.159	0.216	0.307
Burundi	Marangara	2000-2017	-0.054	-0.039	-0.025
Burundi	Matana	2000	0.292	0.404	0.553
Burundi	Matana	2017	0.137	0.185	0.258
Burundi	Matana	2000-2017	-0.059	-0.045	-0.033
Burundi	Matongo	2000	0.302	0.422	0.581
Burundi	Matongo	2017	0.141	0.197	0.279
Burundi	Matongo	2000-2017	-0.064	-0.048	-0.032
Burundi	Mbuye	2000	0.296	0.406	0.556
Burundi	Mbuye	2017	0.137	0.194	0.269
Burundi	Mbuye	2000-2017	-0.061	-0.045	-0.030
Burundi	Mishiha	2000	0.250	0.333	0.472
Burundi	Mishiha	2017	0.146	0.198	0.271
Burundi	Mishiha	2000-2017	-0.034	-0.022	-0.009
Burundi	Mpanda	2000	0.275	0.375	0.520
Burundi	Mpanda	2017	0.129	0.179	0.248
Burundi	Mpanda	2000-2017	-0.062	-0.046	-0.030
Burundi	Mpinga-Kayove	2000	0.278	0.384	0.532
Burundi	Mpinga-Kayove	2017	0.143	0.193	0.275
Burundi	Mpinga-Kayove	2000-2017	-0.051	-0.038	-0.025
Burundi	Mubimbi	2000	0.265	0.378	0.545
Burundi	Mubimbi	2017	0.119	0.170	0.236
Burundi	Mubimbi	2000-2017	-0.070	-0.049	-0.030
Burundi	Mugamba	2000	0.305	0.414	0.565
Burundi	Mugamba	2017	0.141	0.197	0.267
Burundi	Mugamba	2000-2017	-0.056	-0.044	-0.032
Burundi	Mugina	2000	0.264	0.364	0.506
Burundi	Mugina	2017	0.141	0.188	0.262
Burundi	Mugina	2000-2017	-0.053	-0.039	-0.027
Burundi	Mugongomanga	2000	0.294	0.407	0.558
Burundi	Mugongomanga	2017	0.137	0.193	0.273
Burundi	Mugongomanga	2000-2017	-0.064	-0.045	-0.029
Burundi	Muhanga	2000	0.278	0.407	0.568
Burundi	Muhanga	2017	0.143	0.205	0.286
Burundi	Muhanga	2000-2017	-0.058	-0.042	-0.028
Burundi	Muhuta	2000	0.282	0.392	0.543
Burundi	Muhuta	2017	0.127	0.178	0.259
Burundi	Muhuta	2000-2017	-0.063	-0.049	-0.031
Burundi	Mukike	2000	0.302	0.410	0.552
Burundi	Mukike	2017	0.143	0.192	0.270
Burundi	Mukike	2000-2017	-0.060	-0.045	-0.029
Burundi	Muramvya	2000	0.296	0.410	0.576
Burundi	Muramvya	2017	0.139	0.191	0.265
Burundi	Muramvya	2000-2017	-0.059	-0.046	-0.031
Burundi	Muruta	2000	0.308	0.425	0.583
Burundi	Muruta	2017	0.142	0.200	0.281
Burundi	Muruta	2000-2017	-0.063	-0.047	-0.031
Burundi	Murwi	2000	0.271	0.368	0.494

Table 1: LRI DALYs rate by unit *(continued)*

Country	Unit	year	mean rate	lower rate	upper rate
Burundi	Murwi	2017	0.149	0.203	0.280
Burundi	Murwi	2000-2017	-0.050	-0.036	-0.023
Burundi	Musaga	2000	0.246	0.351	0.513
Burundi	Musaga	2017	0.108	0.156	0.226
Burundi	Musaga	2000-2017	-0.073	-0.051	-0.030
Burundi	Musigati	2000	0.286	0.402	0.549
Burundi	Musigati	2017	0.143	0.195	0.274
Burundi	Musigati	2000-2017	-0.057	-0.046	-0.036
Burundi	Musongati	2000	0.283	0.390	0.551
Burundi	Musongati	2017	0.137	0.192	0.271
Burundi	Musongati	2000-2017	-0.054	-0.041	-0.029
Burundi	Mutaho	2000	0.310	0.418	0.583
Burundi	Mutaho	2017	0.150	0.206	0.284
Burundi	Mutaho	2000-2017	-0.060	-0.044	-0.029
Burundi	Mutambu	2000	0.274	0.379	0.526
Burundi	Mutambu	2017	0.122	0.171	0.237
Burundi	Mutambu	2000-2017	-0.067	-0.049	-0.030
Burundi	Mutimbuzi	2000	0.276	0.363	0.498
Burundi	Mutimbuzi	2017	0.117	0.160	0.223
Burundi	Mutimbuzi	2000-2017	-0.061	-0.048	-0.033
Burundi	Mutumba	2000	0.289	0.402	0.569
Burundi	Mutumba	2017	0.158	0.216	0.301
Burundi	Mutumba	2000-2017	-0.052	-0.037	-0.021
Burundi	Muyinga	2000	0.286	0.389	0.531
Burundi	Muyinga	2017	0.165	0.218	0.304
Burundi	Muyinga	2000-2017	-0.044	-0.032	-0.020
Burundi	Mwakiro	2000	0.296	0.410	0.572
Burundi	Mwakiro	2017	0.155	0.218	0.305
Burundi	Mwakiro	2000-2017	-0.052	-0.036	-0.023
Burundi	Mwumba	2000	0.295	0.398	0.530
Burundi	Mwumba	2017	0.131	0.180	0.247
Burundi	Mwumba	2000-2017	-0.067	-0.052	-0.037
Burundi	Ndava	2000	0.289	0.400	0.534
Burundi	Ndava	2017	0.139	0.188	0.266
Burundi	Ndava	2000-2017	-0.061	-0.047	-0.031
Burundi	Ngagara	2000	0.194	0.320	0.500
Burundi	Ngagara	2017	0.089	0.141	0.225
Burundi	Ngagara	2000-2017	-0.094	-0.054	-0.018
Burundi	Ngozi	2000	0.280	0.399	0.546
Burundi	Ngozi	2017	0.143	0.195	0.264
Burundi	Ngozi	2000-2017	-0.059	-0.044	-0.030
Burundi	Ntega	2000	0.309	0.422	0.581
Burundi	Ntega	2017	0.154	0.215	0.307
Burundi	Ntega	2000-2017	-0.058	-0.043	-0.029
Burundi	Nyabihanga	2000	0.281	0.390	0.541
Burundi	Nyabihanga	2017	0.136	0.184	0.258
Burundi	Nyabihanga	2000-2017	-0.064	-0.045	-0.025
Burundi	Nyabikere	2000	0.297	0.407	0.561
Burundi	Nyabikere	2017	0.153	0.217	0.318
Burundi	Nyabikere	2000-2017	-0.053	-0.039	-0.023
Burundi	Nyabiraba	2000	0.279	0.394	0.565
Burundi	Nyabiraba	2017	0.129	0.175	0.240
Burundi	Nyabiraba	2000-2017	-0.069	-0.049	-0.031
Burundi	Nyabitsinda	2000	0.273	0.376	0.527
Burundi	Nyabitsinda	2017	0.150	0.212	0.304
Burundi	Nyabitsinda	2000-2017	-0.047	-0.034	-0.020
Burundi	Nyakabiga	2000	0.230	0.336	0.489
Burundi	Nyakabiga	2017	0.096	0.147	0.207
Burundi	Nyakabiga	2000-2017	-0.078	-0.053	-0.023
Burundi	Nyamurenza	2000	0.278	0.394	0.555
Burundi	Nyamurenza	2017	0.142	0.194	0.276

Table 1: LRI DALYs rate by unit *(continued)*

Country	Unit	year	mean rate	lower rate	upper rate
Burundi	Nyamurenza	2000-2017	-0.063	-0.045	-0.026
Burundi	Nyanrusange	2000	0.280	0.383	0.549
Burundi	Nyanrusange	2017	0.133	0.188	0.260
Burundi	Nyanrusange	2000-2017	-0.059	-0.042	-0.028
Burundi	Nyanza-Lac	2000	0.273	0.365	0.507
Burundi	Nyanza-Lac	2017	0.126	0.177	0.251
Burundi	Nyanza-Lac	2000-2017	-0.054	-0.043	-0.031
Burundi	Rango	2000	0.298	0.406	0.581
Burundi	Rango	2017	0.143	0.194	0.268
Burundi	Rango	2000-2017	-0.059	-0.046	-0.035
Burundi	Roherero	2000	0.229	0.343	0.516
Burundi	Roherero	2017	0.099	0.152	0.234
Burundi	Roherero	2000-2017	-0.082	-0.052	-0.023
Burundi	Rugazi	2000	0.279	0.388	0.535
Burundi	Rugazi	2017	0.126	0.175	0.237
Burundi	Rugazi	2000-2017	-0.066	-0.050	-0.033
Burundi	Rugombo	2000	0.256	0.349	0.478
Burundi	Rugombo	2017	0.141	0.191	0.258
Burundi	Rugombo	2000-2017	-0.051	-0.036	-0.022
Burundi	Ruhororo	2000	0.304	0.414	0.575
Burundi	Ruhororo	2017	0.155	0.214	0.297
Burundi	Ruhororo	2000-2017	-0.056	-0.041	-0.026
Burundi	Rumonge	2000	0.265	0.356	0.497
Burundi	Rumonge	2017	0.116	0.161	0.227
Burundi	Rumonge	2000-2017	-0.059	-0.048	-0.036
Burundi	Rusaka	2000	0.290	0.397	0.550
Burundi	Rusaka	2017	0.135	0.188	0.258
Burundi	Rusaka	2000-2017	-0.061	-0.046	-0.032
Burundi	Rutana	2000	0.280	0.385	0.522
Burundi	Rutana	2017	0.133	0.182	0.260
Burundi	Rutana	2000-2017	-0.058	-0.047	-0.033
Burundi	Rutegama	2000	0.292	0.405	0.546
Burundi	Rutegama	2017	0.133	0.190	0.270
Burundi	Rutegama	2000-2017	-0.066	-0.046	-0.027
Burundi	Rutovu	2000	0.291	0.391	0.547
Burundi	Rutovu	2017	0.132	0.181	0.249
Burundi	Rutovu	2000-2017	-0.057	-0.046	-0.033
Burundi	Ruyigi	2000	0.275	0.381	0.533
Burundi	Ruyigi	2017	0.152	0.210	0.296
Burundi	Ruyigi	2000-2017	-0.046	-0.033	-0.019
Burundi	Ryansoro	2000	0.289	0.395	0.562
Burundi	Ryansoro	2017	0.135	0.186	0.254
Burundi	Ryansoro	2000-2017	-0.058	-0.045	-0.032
Burundi	Shombo	2000	0.287	0.395	0.550
Burundi	Shombo	2017	0.156	0.214	0.307
Burundi	Shombo	2000-2017	-0.052	-0.038	-0.023
Burundi	Songa	2000	0.292	0.387	0.526
Burundi	Songa	2017	0.132	0.177	0.241
Burundi	Songa	2000-2017	-0.059	-0.046	-0.034
Burundi	Tangara	2000	0.301	0.408	0.571
Burundi	Tangara	2017	0.166	0.230	0.327
Burundi	Tangara	2000-2017	-0.049	-0.035	-0.021
Burundi	Vugizo	2000	0.270	0.370	0.511
Burundi	Vugizo	2017	0.125	0.174	0.240
Burundi	Vugizo	2000-2017	-0.063	-0.048	-0.034
Burundi	Vumbi	2000	0.300	0.411	0.590
Burundi	Vumbi	2017	0.174	0.235	0.329
Burundi	Vumbi	2000-2017	-0.047	-0.034	-0.021
Burundi	Vyanda	2000	0.278	0.376	0.529
Burundi	Vyanda	2017	0.125	0.171	0.242
Burundi	Vyanda	2000-2017	-0.061	-0.049	-0.036

Table 1: LRI DALYs rate by unit *(continued)*

Country	Unit	year	mean rate	lower rate	upper rate
Cameroon	Bamboutos	2000	0.177	0.278	0.404
Cameroon	Bamboutos	2017	0.065	0.109	0.165
Cameroon	Bamboutos	2000-2017	-0.083	-0.057	-0.028
Cameroon	Bénoué	2000	0.230	0.346	0.540
Cameroon	Bénoué	2017	0.106	0.168	0.253
Cameroon	Bénoué	2000-2017	-0.071	-0.044	-0.017
Cameroon	Boumba et Ngoko	2000	0.191	0.320	0.529
Cameroon	Boumba et Ngoko	2017	0.079	0.143	0.254
Cameroon	Boumba et Ngoko	2000-2017	-0.078	-0.049	-0.019
Cameroon	Boyo	2000	0.181	0.285	0.431
Cameroon	Boyo	2017	0.068	0.112	0.167
Cameroon	Boyo	2000-2017	-0.085	-0.058	-0.032
Cameroon	Bui	2000	0.181	0.288	0.422
Cameroon	Bui	2017	0.070	0.115	0.176
Cameroon	Bui	2000-2017	-0.084	-0.057	-0.029
Cameroon	Diamaré	2000	0.216	0.347	0.534
Cameroon	Diamaré	2017	0.106	0.172	0.261
Cameroon	Diamaré	2000-2017	-0.068	-0.041	-0.013
Cameroon	Dja et Lobo	2000	0.197	0.311	0.493
Cameroon	Dja et Lobo	2017	0.075	0.127	0.213
Cameroon	Dja et Lobo	2000-2017	-0.080	-0.054	-0.023
Cameroon	Djerem	2000	0.202	0.306	0.469
Cameroon	Djerem	2017	0.084	0.138	0.211
Cameroon	Djerem	2000-2017	-0.079	-0.047	-0.019
Cameroon	Donga Mantung	2000	0.190	0.288	0.434
Cameroon	Donga Mantung	2017	0.070	0.116	0.175
Cameroon	Donga Mantung	2000-2017	-0.086	-0.058	-0.031
Cameroon	Fako	2000	0.169	0.266	0.414
Cameroon	Fako	2017	0.063	0.105	0.165
Cameroon	Fako	2000-2017	-0.084	-0.057	-0.027
Cameroon	Faro	2000	0.225	0.337	0.531
Cameroon	Faro	2017	0.099	0.156	0.235
Cameroon	Faro	2000-2017	-0.080	-0.049	-0.021
Cameroon	Faro et Déo	2000	0.209	0.315	0.476
Cameroon	Faro et Déo	2017	0.090	0.143	0.207
Cameroon	Faro et Déo	2000-2017	-0.081	-0.049	-0.022
Cameroon	Haut Nkam	2000	0.185	0.281	0.421
Cameroon	Haut Nkam	2017	0.068	0.111	0.169
Cameroon	Haut Nkam	2000-2017	-0.086	-0.057	-0.026
Cameroon	Haut Nyong	2000	0.199	0.315	0.506
Cameroon	Haut Nyong	2017	0.078	0.133	0.220
Cameroon	Haut Nyong	2000-2017	-0.078	-0.052	-0.024
Cameroon	Haute Sanaga	2000	0.199	0.302	0.460
Cameroon	Haute Sanaga	2017	0.078	0.128	0.200
Cameroon	Haute Sanaga	2000-2017	-0.078	-0.051	-0.024
Cameroon	Hauts Plateaux	2000	0.177	0.277	0.419
Cameroon	Hauts Plateaux	2017	0.064	0.110	0.168
Cameroon	Hauts Plateaux	2000-2017	-0.085	-0.056	-0.028
Cameroon	Kadey	2000	0.191	0.312	0.497
Cameroon	Kadey	2017	0.079	0.139	0.249
Cameroon	Kadey	2000-2017	-0.078	-0.049	-0.021
Cameroon	Koung Khi	2000	0.176	0.278	0.408
Cameroon	Koung Khi	2017	0.067	0.110	0.161
Cameroon	Koung Khi	2000-2017	-0.085	-0.057	-0.024
Cameroon	Koupé Manengouba	2000	0.176	0.270	0.406
Cameroon	Koupé Manengouba	2017	0.064	0.106	0.164
Cameroon	Koupé Manengouba	2000-2017	-0.086	-0.060	-0.031
Cameroon	Lebialem	2000	0.177	0.279	0.422
Cameroon	Lebialem	2017	0.062	0.108	0.165
Cameroon	Lebialem	2000-2017	-0.087	-0.059	-0.031
Cameroon	Lekié	2000	0.186	0.292	0.434

Table 1: LRI DALYs rate by unit *(continued)*

Country	Unit	year	mean rate	lower rate	upper rate
Cameroon	Lekié	2017	0.075	0.125	0.194
Cameroon	Lekié	2000-2017	-0.077	-0.051	-0.021
Cameroon	Logone et Chari	2000	0.224	0.351	0.543
Cameroon	Logone et Chari	2017	0.116	0.181	0.276
Cameroon	Logone et Chari	2000-2017	-0.067	-0.039	-0.015
Cameroon	Lom et Djerem	2000	0.202	0.307	0.467
Cameroon	Lom et Djerem	2017	0.085	0.140	0.226
Cameroon	Lom et Djerem	2000-2017	-0.079	-0.048	-0.021
Cameroon	Manyu	2000	0.173	0.262	0.397
Cameroon	Manyu	2017	0.063	0.105	0.159
Cameroon	Manyu	2000-2017	-0.085	-0.058	-0.030
Cameroon	Mayo Banyo	2000	0.199	0.303	0.458
Cameroon	Mayo Banyo	2017	0.078	0.129	0.195
Cameroon	Mayo Banyo	2000-2017	-0.082	-0.053	-0.024
Cameroon	Mayo Danay	2000	0.228	0.357	0.543
Cameroon	Mayo Danay	2017	0.112	0.182	0.276
Cameroon	Mayo Danay	2000-2017	-0.069	-0.041	-0.015
Cameroon	Mayo Kani	2000	0.224	0.348	0.529
Cameroon	Mayo Kani	2017	0.111	0.179	0.267
Cameroon	Mayo Kani	2000-2017	-0.066	-0.038	-0.011
Cameroon	Mayo Louti	2000	0.224	0.348	0.520
Cameroon	Mayo Louti	2017	0.108	0.172	0.261
Cameroon	Mayo Louti	2000-2017	-0.069	-0.040	-0.013
Cameroon	Mayo Rey	2000	0.238	0.342	0.527
Cameroon	Mayo Rey	2017	0.102	0.166	0.253
Cameroon	Mayo Rey	2000-2017	-0.078	-0.046	-0.019
Cameroon	Mayo Sava	2000	0.219	0.354	0.559
Cameroon	Mayo Sava	2017	0.108	0.175	0.267
Cameroon	Mayo Sava	2000-2017	-0.069	-0.042	-0.015
Cameroon	Mayo Tsanaga	2000	0.226	0.343	0.516
Cameroon	Mayo Tsanaga	2017	0.108	0.173	0.255
Cameroon	Mayo Tsanaga	2000-2017	-0.066	-0.040	-0.015
Cameroon	Mbam et Inoubou	2000	0.185	0.285	0.420
Cameroon	Mbam et Inoubou	2017	0.072	0.118	0.182
Cameroon	Mbam et Inoubou	2000-2017	-0.077	-0.053	-0.024
Cameroon	Mbam et Kim	2000	0.189	0.287	0.426
Cameroon	Mbam et Kim	2017	0.076	0.126	0.195
Cameroon	Mbam et Kim	2000-2017	-0.076	-0.050	-0.022
Cameroon	Mbéré	2000	0.212	0.310	0.456
Cameroon	Mbéré	2017	0.086	0.149	0.235
Cameroon	Mbéré	2000-2017	-0.076	-0.046	-0.019
Cameroon	Mefou et Afamba	2000	0.167	0.271	0.408
Cameroon	Mefou et Afamba	2017	0.068	0.124	0.204
Cameroon	Mefou et Afamba	2000-2017	-0.082	-0.048	-0.014
Cameroon	Mefou et Akono	2000	0.174	0.275	0.421
Cameroon	Mefou et Akono	2017	0.069	0.120	0.191
Cameroon	Mefou et Akono	2000-2017	-0.080	-0.051	-0.017
Cameroon	Meme	2000	0.180	0.275	0.424
Cameroon	Meme	2017	0.063	0.105	0.162
Cameroon	Meme	2000-2017	-0.087	-0.062	-0.032
Cameroon	Menchum	2000	0.184	0.284	0.420
Cameroon	Menchum	2017	0.066	0.112	0.168
Cameroon	Menchum	2000-2017	-0.086	-0.058	-0.029
Cameroon	Menoua	2000	0.179	0.278	0.411
Cameroon	Menoua	2017	0.064	0.109	0.167
Cameroon	Menoua	2000-2017	-0.084	-0.056	-0.025
Cameroon	Mezam	2000	0.178	0.279	0.419
Cameroon	Mezam	2017	0.065	0.109	0.168
Cameroon	Mezam	2000-2017	-0.084	-0.057	-0.029
Cameroon	Mfoundi	2000	0.156	0.252	0.380
Cameroon	Mfoundi	2017	0.073	0.121	0.197

Table 1: LRI DALYs rate by unit *(continued)*

Country	Unit	year	mean rate	lower rate	upper rate
Cameroon	Mfoundi	2000-2017	-0.072	-0.044	-0.012
Cameroon	Mifi	2000	0.178	0.273	0.396
Cameroon	Mifi	2017	0.067	0.109	0.163
Cameroon	Mifi	2000-2017	-0.081	-0.055	-0.028
Cameroon	Momo	2000	0.179	0.280	0.411
Cameroon	Momo	2017	0.065	0.108	0.167
Cameroon	Momo	2000-2017	-0.086	-0.058	-0.031
Cameroon	Moungo	2000	0.184	0.279	0.426
Cameroon	Moungo	2017	0.068	0.111	0.173
Cameroon	Moungo	2000-2017	-0.085	-0.058	-0.029
Cameroon	Mvila	2000	0.189	0.309	0.483
Cameroon	Mvila	2017	0.069	0.120	0.193
Cameroon	Mvila	2000-2017	-0.083	-0.056	-0.023
Cameroon	Ndé	2000	0.183	0.277	0.404
Cameroon	Ndé	2017	0.068	0.111	0.171
Cameroon	Ndé	2000-2017	-0.084	-0.056	-0.027
Cameroon	Ndian	2000	0.175	0.268	0.417
Cameroon	Ndian	2017	0.061	0.101	0.153
Cameroon	Ndian	2000-2017	-0.090	-0.063	-0.035
Cameroon	Ngo Ketunjia	2000	0.182	0.280	0.415
Cameroon	Ngo Ketunjia	2017	0.065	0.111	0.172
Cameroon	Ngo Ketunjia	2000-2017	-0.085	-0.056	-0.025
Cameroon	Nkam	2000	0.184	0.281	0.423
Cameroon	Nkam	2017	0.067	0.111	0.170
Cameroon	Nkam	2000-2017	-0.083	-0.056	-0.028
Cameroon	Noun	2000	0.186	0.282	0.406
Cameroon	Noun	2017	0.069	0.113	0.172
Cameroon	Noun	2000-2017	-0.083	-0.055	-0.025
Cameroon	Nyong et Kéllé	2000	0.186	0.294	0.445
Cameroon	Nyong et Kéllé	2017	0.068	0.119	0.187
Cameroon	Nyong et Kéllé	2000-2017	-0.080	-0.055	-0.023
Cameroon	Nyong et Mfoumou	2000	0.199	0.307	0.487
Cameroon	Nyong et Mfoumou	2017	0.077	0.128	0.207
Cameroon	Nyong et Mfoumou	2000-2017	-0.078	-0.053	-0.023
Cameroon	Nyong et So'o	2000	0.188	0.300	0.461
Cameroon	Nyong et So'o	2017	0.070	0.122	0.192
Cameroon	Nyong et So'o	2000-2017	-0.084	-0.054	-0.019
Cameroon	Océan	2000	0.181	0.298	0.472
Cameroon	Océan	2017	0.066	0.114	0.177
Cameroon	Océan	2000-2017	-0.082	-0.056	-0.025
Cameroon	Sanaga Maritime	2000	0.168	0.271	0.418
Cameroon	Sanaga Maritime	2017	0.067	0.111	0.172
Cameroon	Sanaga Maritime	2000-2017	-0.081	-0.055	-0.024
Cameroon	Vallée du Ntem	2000	0.181	0.311	0.509
Cameroon	Vallée du Ntem	2017	0.071	0.121	0.198
Cameroon	Vallée du Ntem	2000-2017	-0.083	-0.056	-0.025
Cameroon	Vina	2000	0.207	0.307	0.450
Cameroon	Vina	2017	0.093	0.148	0.221
Cameroon	Vina	2000-2017	-0.075	-0.045	-0.017
Cameroon	Wouri	2000	0.152	0.240	0.369
Cameroon	Wouri	2017	0.064	0.108	0.170
Cameroon	Wouri	2000-2017	-0.075	-0.048	-0.015
Central African Republic	Alindao	2000	0.424	0.625	0.897
Central African Republic	Alindao	2017	0.347	0.491	0.664
Central African Republic	Alindao	2000-2017	-0.027	-0.013	0.000
Central African Republic	Baboua	2000	0.338	0.473	0.655
Central African Republic	Baboua	2017	0.325	0.454	0.625
Central African Republic	Baboua	2000-2017	-0.011	-0.004	0.004
Central African Republic	Bakala	2000	0.423	0.593	0.834
Central African Republic	Bakala	2017	0.344	0.493	0.680
Central African Republic	Bakala	2000-2017	-0.021	-0.010	0.003

Table 1: LRI DALYs rate by unit *(continued)*

Country	Unit	year	mean rate	lower rate	upper rate
Central African Republic	Bakouma	2000	0.390	0.541	0.750
Central African Republic	Bakouma	2017	0.352	0.495	0.674
Central African Republic	Bakouma	2000-2017	-0.015	-0.005	0.005
Central African Republic	Bambari	2000	0.415	0.593	0.833
Central African Republic	Bambari	2017	0.329	0.470	0.656
Central African Republic	Bambari	2000-2017	-0.026	-0.012	0.002
Central African Republic	Bambio	2000	0.331	0.461	0.638
Central African Republic	Bambio	2017	0.313	0.443	0.606
Central African Republic	Bambio	2000-2017	-0.013	-0.002	0.008
Central African Republic	Bamingui	2000	0.361	0.504	0.688
Central African Republic	Bamingui	2017	0.344	0.480	0.660
Central African Republic	Bamingui	2000-2017	-0.011	-0.004	0.002
Central African Republic	Bangassou	2000	0.375	0.529	0.731
Central African Republic	Bangassou	2017	0.346	0.491	0.691
Central African Republic	Bangassou	2000-2017	-0.019	-0.006	0.006
Central African Republic	Bangui	2000	0.328	0.501	0.769
Central African Republic	Bangui	2017	0.228	0.364	0.566
Central African Republic	Bangui	2000-2017	-0.056	-0.020	0.015
Central African Republic	Baoro	2000	0.344	0.478	0.668
Central African Republic	Baoro	2017	0.324	0.463	0.659
Central African Republic	Baoro	2000-2017	-0.015	-0.003	0.008
Central African Republic	Batangafo	2000	0.378	0.527	0.738
Central African Republic	Batangafo	2017	0.338	0.479	0.665
Central African Republic	Batangafo	2000-2017	-0.015	-0.005	0.005
Central African Republic	Berbérati	2000	0.336	0.474	0.647
Central African Republic	Berbérati	2017	0.323	0.466	0.644
Central African Republic	Berbérati	2000-2017	-0.013	-0.003	0.007
Central African Republic	Bimbo	2000	0.347	0.485	0.703
Central African Republic	Bimbo	2017	0.246	0.361	0.511
Central African Republic	Bimbo	2000-2017	-0.039	-0.018	0.003
Central African Republic	Birao	2000	0.431	0.620	0.877
Central African Republic	Birao	2017	0.407	0.566	0.782
Central African Republic	Birao	2000-2017	-0.011	-0.005	0.002
Central African Republic	Boali	2000	0.389	0.557	0.774
Central African Republic	Boali	2017	0.345	0.487	0.692
Central African Republic	Boali	2000-2017	-0.022	-0.008	0.005
Central African Republic	Bocaranga	2000	0.354	0.492	0.670
Central African Republic	Bocaranga	2017	0.303	0.429	0.599
Central African Republic	Bocaranga	2000-2017	-0.019	-0.010	-0.002
Central African Republic	Boda	2000	0.343	0.487	0.671
Central African Republic	Boda	2017	0.336	0.480	0.694
Central African Republic	Boda	2000-2017	-0.011	-0.002	0.009
Central African Republic	Bossangoa	2000	0.377	0.529	0.723
Central African Republic	Bossangoa	2017	0.317	0.447	0.618
Central African Republic	Bossangoa	2000-2017	-0.021	-0.009	0.004
Central African Republic	Bouar	2000	0.341	0.474	0.651
Central African Republic	Bouar	2017	0.300	0.428	0.590
Central African Republic	Bouar	2000-2017	-0.015	-0.006	0.005
Central African Republic	Bouca	2000	0.379	0.528	0.722
Central African Republic	Bouca	2017	0.354	0.490	0.676
Central African Republic	Bouca	2000-2017	-0.012	-0.004	0.004
Central African Republic	Bozoum	2000	0.351	0.506	0.686
Central African Republic	Bozoum	2017	0.302	0.435	0.608
Central African Republic	Bozoum	2000-2017	-0.026	-0.011	0.002
Central African Republic	Bria	2000	0.384	0.538	0.742
Central African Republic	Bria	2017	0.344	0.494	0.688
Central African Republic	Bria	2000-2017	-0.013	-0.005	0.005
Central African Republic	Carnot	2000	0.339	0.472	0.641
Central African Republic	Carnot	2017	0.326	0.463	0.639
Central African Republic	Carnot	2000-2017	-0.012	-0.002	0.007
Central African Republic	Damara	2000	0.417	0.587	0.834

Table 1: LRI DALYs rate by unit *(continued)*

Country	Unit	year	mean rate	lower rate	upper rate
Central African Republic	Damara	2017	0.355	0.492	0.671
Central African Republic	Damara	2000-2017	-0.023	-0.012	0.000
Central African Republic	Dékoa	2000	0.386	0.549	0.762
Central African Republic	Dékoa	2017	0.352	0.496	0.687
Central African Republic	Dékoa	2000-2017	-0.016	-0.005	0.006
Central African Republic	Djemah	2000	0.334	0.468	0.643
Central African Republic	Djemah	2017	0.345	0.492	0.680
Central African Republic	Djemah	2000-2017	-0.007	0.002	0.010
Central African Republic	Gambo-Ouango	2000	0.416	0.597	0.809
Central African Republic	Gambo-Ouango	2017	0.345	0.480	0.641
Central African Republic	Gambo-Ouango	2000-2017	-0.024	-0.011	0.001
Central African Republic	Gamboula	2000	0.342	0.472	0.644
Central African Republic	Gamboula	2017	0.332	0.470	0.652
Central African Republic	Gamboula	2000-2017	-0.009	-0.002	0.007
Central African Republic	Grimari	2000	0.408	0.594	0.858
Central African Republic	Grimari	2017	0.347	0.487	0.671
Central African Republic	Grimari	2000-2017	-0.027	-0.013	0.003
Central African Republic	Ippy	2000	0.426	0.598	0.836
Central African Republic	Ippy	2017	0.353	0.494	0.680
Central African Republic	Ippy	2000-2017	-0.022	-0.011	0.002
Central African Republic	Kabo	2000	0.373	0.543	0.759
Central African Republic	Kabo	2017	0.351	0.504	0.711
Central African Republic	Kabo	2000-2017	-0.018	-0.005	0.008
Central African Republic	Kaga-Bandoro	2000	0.371	0.532	0.724
Central African Republic	Kaga-Bandoro	2017	0.335	0.472	0.659
Central African Republic	Kaga-Bandoro	2000-2017	-0.018	-0.007	0.004
Central African Republic	Kembé	2000	0.431	0.627	0.886
Central African Republic	Kembé	2017	0.358	0.491	0.660
Central African Republic	Kembé	2000-2017	-0.026	-0.013	0.001
Central African Republic	Kouango	2000	0.430	0.608	0.843
Central African Republic	Kouango	2017	0.351	0.483	0.664
Central African Republic	Kouango	2000-2017	-0.022	-0.012	-0.002
Central African Republic	M'Baïki	2000	0.344	0.489	0.673
Central African Republic	M'Baïki	2017	0.327	0.459	0.633
Central African Republic	M'Baïki	2000-2017	-0.015	-0.004	0.008
Central African Republic	Markounda	2000	0.378	0.535	0.730
Central African Republic	Markounda	2017	0.316	0.447	0.621
Central African Republic	Markounda	2000-2017	-0.019	-0.010	0.000
Central African Republic	Mbrès	2000	0.374	0.538	0.744
Central African Republic	Mbrès	2017	0.352	0.495	0.693
Central African Republic	Mbrès	2000-2017	-0.016	-0.005	0.004
Central African Republic	Mingala	2000	0.437	0.624	0.892
Central African Republic	Mingala	2017	0.351	0.494	0.678
Central African Republic	Mingala	2000-2017	-0.025	-0.012	0.001
Central African Republic	Mobaye	2000	0.441	0.624	0.867
Central African Republic	Mobaye	2017	0.351	0.489	0.664
Central African Republic	Mobaye	2000-2017	-0.026	-0.013	0.000
Central African Republic	Mongoumba	2000	0.371	0.524	0.714
Central African Republic	Mongoumba	2017	0.335	0.463	0.619
Central African Republic	Mongoumba	2000-2017	-0.024	-0.008	0.008
Central African Republic	Ndélé	2000	0.372	0.516	0.707
Central African Republic	Ndélé	2017	0.345	0.487	0.664
Central African Republic	Ndélé	2000-2017	-0.011	-0.004	0.002
Central African Republic	Nola	2000	0.333	0.467	0.641
Central African Republic	Nola	2017	0.302	0.436	0.605
Central African Republic	Nola	2000-2017	-0.014	-0.005	0.004
Central African Republic	Obo	2000	0.349	0.502	0.688
Central African Republic	Obo	2017	0.325	0.464	0.638
Central African Republic	Obo	2000-2017	-0.012	-0.002	0.008
Central African Republic	Ouadda	2000	0.370	0.528	0.736
Central African Republic	Ouadda	2017	0.350	0.493	0.688

Table 1: LRI DALYs rate by unit *(continued)*

Country	Unit	year	mean rate	lower rate	upper rate
Central African Republic	Ouadda	2000-2017	-0.013	-0.004	0.005
Central African Republic	Ouanda Djallé	2000	0.380	0.535	0.747
Central African Republic	Ouanda Djallé	2017	0.366	0.514	0.706
Central African Republic	Ouanda Djallé	2000-2017	-0.010	-0.002	0.009
Central African Republic	Paoua	2000	0.361	0.508	0.689
Central African Republic	Paoua	2017	0.299	0.422	0.596
Central African Republic	Paoua	2000-2017	-0.022	-0.012	-0.002
Central African Republic	Rafaï	2000	0.341	0.475	0.640
Central African Republic	Rafaï	2017	0.349	0.491	0.681
Central African Republic	Rafaï	2000-2017	-0.006	0.000	0.007
Central African Republic	Sibut	2000	0.419	0.609	0.837
Central African Republic	Sibut	2017	0.338	0.471	0.644
Central African Republic	Sibut	2000-2017	-0.028	-0.014	-0.001
Central African Republic	Yalinga	2000	0.363	0.512	0.700
Central African Republic	Yalinga	2017	0.344	0.489	0.700
Central African Republic	Yalinga	2000-2017	-0.010	-0.003	0.005
Central African Republic	Yaloké	2000	0.383	0.537	0.741
Central African Republic	Yaloké	2017	0.336	0.477	0.663
Central African Republic	Yaloké	2000-2017	-0.017	-0.007	0.003
Central African Republic	Zémio	2000	0.345	0.493	0.673
Central African Republic	Zémio	2017	0.352	0.492	0.677
Central African Republic	Zémio	2000-2017	-0.009	0.000	0.010
Chad	Aboudeïa	2000	0.317	0.500	0.735
Chad	Aboudeïa	2017	0.236	0.380	0.597
Chad	Aboudeïa	2000-2017	-0.045	-0.014	0.022
Chad	Assoungha	2000	0.345	0.576	0.910
Chad	Assoungha	2017	0.239	0.398	0.648
Chad	Assoungha	2000-2017	-0.057	-0.020	0.023
Chad	Baguirmi	2000	0.404	0.608	0.860
Chad	Baguirmi	2017	0.273	0.428	0.630
Chad	Baguirmi	2000-2017	-0.047	-0.018	0.009
Chad	Barh Azoum	2000	0.350	0.555	0.804
Chad	Barh Azoum	2017	0.240	0.403	0.653
Chad	Barh Azoum	2000-2017	-0.047	-0.014	0.023
Chad	Barh El Gazel	2000	0.384	0.608	0.891
Chad	Barh El Gazel	2017	0.274	0.418	0.585
Chad	Barh El Gazel	2000-2017	-0.048	-0.020	0.007
Chad	Barh Köh	2000	0.336	0.545	0.775
Chad	Barh Köh	2017	0.241	0.397	0.591
Chad	Barh Köh	2000-2017	-0.049	-0.015	0.018
Chad	Barh Sara	2000	0.359	0.606	0.893
Chad	Barh Sara	2017	0.230	0.399	0.628
Chad	Barh Sara	2000-2017	-0.055	-0.022	0.010
Chad	Barh Signaka	2000	0.352	0.542	0.833
Chad	Barh Signaka	2017	0.239	0.377	0.585
Chad	Barh Signaka	2000-2017	-0.049	-0.017	0.015
Chad	Batha Est	2000	0.369	0.583	0.901
Chad	Batha Est	2017	0.250	0.391	0.604
Chad	Batha Est	2000-2017	-0.053	-0.022	0.014
Chad	Batha Oues	2000	0.359	0.582	0.874
Chad	Batha Oues	2017	0.259	0.400	0.585
Chad	Batha Oues	2000-2017	-0.051	-0.020	0.011
Chad	Béré	2000	0.535	0.900	1.363
Chad	Béré	2017	0.241	0.382	0.576
Chad	Béré	2000-2017	-0.087	-0.049	-0.017
Chad	Biltine	2000	0.353	0.572	0.908
Chad	Biltine	2017	0.237	0.391	0.638
Chad	Biltine	2000-2017	-0.055	-0.020	0.021
Chad	Bitkine	2000	0.340	0.519	0.789
Chad	Bitkine	2017	0.247	0.391	0.576
Chad	Bitkine	2000-2017	-0.045	-0.013	0.015

Table 1: LRI DALYs rate by unit *(continued)*

Country	Unit	year	mean rate	lower rate	upper rate
Chad	Borkou	2000	0.360	0.579	0.923
Chad	Borkou	2017	0.259	0.406	0.627
Chad	Borkou	2000-2017	-0.048	-0.019	0.016
Chad	Dababa	2000	0.376	0.596	0.833
Chad	Dababa	2017	0.275	0.421	0.610
Chad	Dababa	2000-2017	-0.048	-0.018	0.009
Chad	Dagana	2000	0.401	0.647	0.970
Chad	Dagana	2017	0.280	0.438	0.645
Chad	Dagana	2000-2017	-0.050	-0.021	0.006
Chad	Dar Tama	2000	0.325	0.571	0.921
Chad	Dar Tama	2017	0.225	0.393	0.640
Chad	Dar Tama	2000-2017	-0.059	-0.021	0.021
Chad	Djourf Al Ahmar	2000	0.369	0.604	0.923
Chad	Djourf Al Ahmar	2000	0.368	0.596	0.930
Chad	Djourf Al Ahmar	2017	0.238	0.383	0.628
Chad	Djourf Al Ahmar	2017	0.242	0.402	0.662
Chad	Djourf Al Ahmar	2000-2017	-0.059	-0.025	0.014
Chad	Djourf Al Ahmar	2000-2017	-0.057	-0.021	0.019
Chad	Dodjé	2000	0.499	0.857	1.291
Chad	Dodjé	2017	0.270	0.431	0.636
Chad	Dodjé	2000-2017	-0.073	-0.037	-0.008
Chad	Ennedi Est	2000	0.301	0.562	0.972
Chad	Ennedi Est	2017	0.221	0.397	0.662
Chad	Ennedi Est	2000-2017	-0.052	-0.019	0.024
Chad	Ennedi Ouest	2000	0.320	0.569	0.957
Chad	Ennedi Ouest	2017	0.231	0.401	0.660
Chad	Ennedi Ouest	2000-2017	-0.053	-0.019	0.021
Chad	Fitri	2000	0.361	0.582	0.857
Chad	Fitri	2017	0.258	0.405	0.614
Chad	Fitri	2000-2017	-0.048	-0.019	0.014
Chad	Grande Sido	2000	0.376	0.642	0.940
Chad	Grande Sido	2017	0.233	0.403	0.626
Chad	Grande Sido	2000-2017	-0.059	-0.025	0.011
Chad	Guéra	2000	0.319	0.506	0.698
Chad	Guéra	2017	0.248	0.396	0.572
Chad	Guéra	2000-2017	-0.042	-0.011	0.020
Chad	Haraze Al Biar	2000	0.389	0.622	0.925
Chad	Haraze Al Biar	2017	0.267	0.433	0.621
Chad	Haraze Al Biar	2000-2017	-0.048	-0.018	0.009
Chad	Haraze Mangueigne	2000	0.385	0.629	0.918
Chad	Haraze Mangueigne	2017	0.243	0.413	0.662
Chad	Haraze Mangueigne	2000-2017	-0.056	-0.022	0.018
Chad	Kabbia	2000	0.471	0.758	1.111
Chad	Kabbia	2017	0.293	0.456	0.671
Chad	Kabbia	2000-2017	-0.060	-0.027	0.002
Chad	Kanem	2000	0.373	0.611	0.911
Chad	Kanem	2017	0.271	0.423	0.606
Chad	Kanem	2000-2017	-0.047	-0.020	0.008
Chad	Kobé	2000	0.326	0.575	0.933
Chad	Kobé	2017	0.223	0.396	0.663
Chad	Kobé	2000-2017	-0.054	-0.020	0.022
Chad	Lac Iro	2000	0.337	0.549	0.776
Chad	Lac Iro	2017	0.232	0.392	0.594
Chad	Lac Iro	2000-2017	-0.052	-0.017	0.019
Chad	Lac Léré	2000	0.437	0.701	1.059
Chad	Lac Léré	2017	0.264	0.435	0.653
Chad	Lac Léré	2000-2017	-0.059	-0.028	0.000
Chad	Lac Wey	2000	0.525	0.892	1.346
Chad	Lac Wey	2017	0.242	0.389	0.583
Chad	Lac Wey	2000-2017	-0.084	-0.046	-0.013
Chad	Lanya	2000	0.511	0.871	1.322

Table 1: LRI DALYs rate by unit *(continued)*

Country	Unit	year	mean rate	lower rate	upper rate
Chad	Lanya	2017	0.229	0.384	0.585
Chad	Lanya	2000-2017	-0.083	-0.046	-0.015
Chad	Loug Chari	2000	0.405	0.637	0.917
Chad	Loug Chari	2017	0.249	0.393	0.576
Chad	Loug Chari	2000-2017	-0.059	-0.026	0.003
Chad	Mamdi	2000	0.371	0.622	0.930
Chad	Mamdi	2017	0.261	0.427	0.599
Chad	Mamdi	2000-2017	-0.049	-0.020	0.006
Chad	Mandoul Occidental	2000	0.361	0.605	0.892
Chad	Mandoul Occidental	2017	0.227	0.391	0.603
Chad	Mandoul Occidental	2000-2017	-0.059	-0.025	0.009
Chad	Mandoul Oriental	2000	0.362	0.597	0.890
Chad	Mandoul Oriental	2017	0.242	0.396	0.602
Chad	Mandoul Oriental	2000-2017	-0.055	-0.022	0.010
Chad	Mangalmé	2000	0.361	0.578	0.894
Chad	Mangalmé	2017	0.227	0.362	0.572
Chad	Mangalmé	2000-2017	-0.057	-0.025	0.011
Chad	Mayo-Boneye	2000	0.446	0.700	1.001
Chad	Mayo-Boneye	2017	0.267	0.426	0.630
Chad	Mayo-Boneye	2000-2017	-0.059	-0.028	0.002
Chad	Mayo-Dallah	2000	0.445	0.731	1.088
Chad	Mayo-Dallah	2017	0.276	0.451	0.663
Chad	Mayo-Dallah	2000-2017	-0.057	-0.025	0.004
Chad	Mont Illi	2000	0.470	0.752	1.090
Chad	Mont Illi	2017	0.291	0.458	0.657
Chad	Mont Illi	2000-2017	-0.058	-0.027	0.003
Chad	Monts de Lam	2000	0.473	0.800	1.202
Chad	Monts de Lam	2017	0.246	0.409	0.615
Chad	Monts de Lam	2000-2017	-0.069	-0.037	-0.008
Chad	N'Djamena	2000	0.368	0.585	0.850
Chad	N'Djamena	2000	0.310	0.506	0.751
Chad	N'Djamena	2017	0.254	0.417	0.594
Chad	N'Djamena	2017	0.234	0.381	0.575
Chad	N'Djamena	2000-2017	-0.046	-0.014	0.014
Chad	N'Djamena	2000-2017	-0.044	-0.015	0.013
Chad	Ngourkosso	2000	0.537	0.890	1.317
Chad	Ngourkosso	2017	0.236	0.374	0.568
Chad	Ngourkosso	2000-2017	-0.089	-0.050	-0.015
Chad	Nokou	2000	0.358	0.594	0.910
Chad	Nokou	2017	0.271	0.412	0.595
Chad	Nokou	2000-2017	-0.046	-0.019	0.008
Chad	Nya Pendé	2000	0.494	0.835	1.275
Chad	Nya Pendé	2017	0.231	0.388	0.578
Chad	Nya Pendé	2000-2017	-0.076	-0.041	-0.009
Chad	Ouara	2000	0.364	0.584	0.947
Chad	Ouara	2017	0.248	0.398	0.633
Chad	Ouara	2000-2017	-0.056	-0.021	0.017
Chad	Pendé	2000	0.472	0.803	1.241
Chad	Pendé	2017	0.230	0.385	0.587
Chad	Pendé	2000-2017	-0.078	-0.040	-0.008
Chad	Sila	2000	0.367	0.606	0.935
Chad	Sila	2017	0.248	0.419	0.686
Chad	Sila	2000-2017	-0.055	-0.020	0.023
Chad	Tandjilé Est	2000	0.488	0.806	1.194
Chad	Tandjilé Est	2017	0.236	0.381	0.572
Chad	Tandjilé Est	2000-2017	-0.079	-0.043	-0.011
Chad	Tandjilé Ouest	2000	0.525	0.884	1.324
Chad	Tandjilé Ouest	2017	0.267	0.415	0.605
Chad	Tandjilé Ouest	2000-2017	-0.078	-0.043	-0.011
Chad	Tibesti	2000	0.301	0.533	0.962
Chad	Tibesti	2017	0.211	0.371	0.586

Table 1: LRI DALYs rate by unit *(continued)*

Country	Unit	year	mean rate	lower rate	upper rate
Chad	Tibesti	2000-2017	-0.053	-0.019	0.012
Chad	Wayi	2000	0.382	0.632	0.948
Chad	Wayi	2017	0.273	0.435	0.642
Chad	Wayi	2000-2017	-0.049	-0.020	0.008
Côte d'Ivoire	Abidjan	2000	0.180	0.294	0.437
Côte d'Ivoire	Abidjan	2017	0.101	0.178	0.268
Côte d'Ivoire	Abidjan	2000-2017	-0.053	-0.024	0.006
Côte d'Ivoire	Agnéby-Tiassa	2000	0.209	0.328	0.499
Côte d'Ivoire	Agnéby-Tiassa	2017	0.118	0.200	0.307
Côte d'Ivoire	Agnéby-Tiassa	2000-2017	-0.051	-0.026	0.001
Côte d'Ivoire	Bafing	2000	0.191	0.293	0.430
Côte d'Ivoire	Bafing	2017	0.122	0.183	0.266
Côte d'Ivoire	Bafing	2000-2017	-0.048	-0.022	0.003
Côte d'Ivoire	Bagoué	2000	0.213	0.327	0.506
Côte d'Ivoire	Bagoué	2017	0.120	0.193	0.288
Côte d'Ivoire	Bagoué	2000-2017	-0.051	-0.025	0.001
Côte d'Ivoire	Bélier	2000	0.218	0.330	0.484
Côte d'Ivoire	Bélier	2017	0.120	0.193	0.289
Côte d'Ivoire	Bélier	2000-2017	-0.053	-0.028	-0.003
Côte d'Ivoire	Béré	2000	0.198	0.296	0.426
Côte d'Ivoire	Béré	2017	0.111	0.176	0.258
Côte d'Ivoire	Béré	2000-2017	-0.049	-0.026	-0.002
Côte d'Ivoire	Bounkani	2000	0.228	0.342	0.518
Côte d'Ivoire	Bounkani	2017	0.123	0.195	0.295
Côte d'Ivoire	Bounkani	2000-2017	-0.052	-0.029	-0.003
Côte d'Ivoire	Cavally	2000	0.246	0.386	0.605
Côte d'Ivoire	Cavally	2017	0.134	0.216	0.318
Côte d'Ivoire	Cavally	2000-2017	-0.053	-0.028	-0.002
Côte d'Ivoire	Folon	2000	0.203	0.312	0.474
Côte d'Ivoire	Folon	2017	0.112	0.181	0.269
Côte d'Ivoire	Folon	2000-2017	-0.053	-0.027	-0.002
Côte d'Ivoire	Gbeke	2000	0.207	0.309	0.464
Côte d'Ivoire	Gbeke	2017	0.120	0.199	0.302
Côte d'Ivoire	Gbeke	2000-2017	-0.049	-0.021	0.005
Côte d'Ivoire	Gbôkle	2000	0.271	0.420	0.638
Côte d'Ivoire	Gbôkle	2017	0.128	0.208	0.316
Côte d'Ivoire	Gbôkle	2000-2017	-0.062	-0.036	-0.007
Côte d'Ivoire	Gôh	2000	0.221	0.353	0.524
Côte d'Ivoire	Gôh	2017	0.123	0.201	0.299
Côte d'Ivoire	Gôh	2000-2017	-0.056	-0.029	-0.004
Côte d'Ivoire	Gontougo	2000	0.219	0.332	0.497
Côte d'Ivoire	Gontougo	2017	0.112	0.187	0.288
Côte d'Ivoire	Gontougo	2000-2017	-0.054	-0.030	-0.005
Côte d'Ivoire	Grands Ponts	2000	0.238	0.366	0.554
Côte d'Ivoire	Grands Ponts	2017	0.117	0.202	0.307
Côte d'Ivoire	Grands Ponts	2000-2017	-0.057	-0.032	-0.004
Côte d'Ivoire	Guémon	2000	0.219	0.342	0.517
Côte d'Ivoire	Guémon	2017	0.130	0.207	0.300
Côte d'Ivoire	Guémon	2000-2017	-0.051	-0.024	0.003
Côte d'Ivoire	Hambol	2000	0.220	0.321	0.480
Côte d'Ivoire	Hambol	2017	0.119	0.189	0.289
Côte d'Ivoire	Hambol	2000-2017	-0.052	-0.027	-0.004
Côte d'Ivoire	Haut-Sassandra	2000	0.213	0.336	0.495
Côte d'Ivoire	Haut-Sassandra	2017	0.120	0.194	0.284
Côte d'Ivoire	Haut-Sassandra	2000-2017	-0.051	-0.027	-0.005
Côte d'Ivoire	Iffou	2000	0.221	0.326	0.491
Côte d'Ivoire	Iffou	2017	0.118	0.193	0.288
Côte d'Ivoire	Iffou	2000-2017	-0.052	-0.027	-0.002
Côte d'Ivoire	Indénié-Djuablin	2000	0.201	0.312	0.462
Côte d'Ivoire	Indénié-Djuablin	2017	0.114	0.193	0.295
Côte d'Ivoire	Indénié-Djuablin	2000-2017	-0.049	-0.024	0.005

Table 1: LRI DALYs rate by unit *(continued)*

Country	Unit	year	mean rate	lower rate	upper rate
Côte d'Ivoire	Kabadougou	2000	0.192	0.294	0.455
Côte d'Ivoire	Kabadougou	2017	0.113	0.175	0.260
Côte d'Ivoire	Kabadougou	2000-2017	-0.050	-0.024	0.000
Côte d'Ivoire	La Mé	2000	0.200	0.313	0.457
Côte d'Ivoire	La Mé	2017	0.116	0.196	0.301
Côte d'Ivoire	La Mé	2000-2017	-0.047	-0.023	0.006
Côte d'Ivoire	Lôh-Djiboua	2000	0.236	0.375	0.573
Côte d'Ivoire	Lôh-Djiboua	2017	0.122	0.201	0.302
Côte d'Ivoire	Lôh-Djiboua	2000-2017	-0.057	-0.033	-0.007
Côte d'Ivoire	Marahoué	2000	0.217	0.331	0.481
Côte d'Ivoire	Marahoué	2017	0.121	0.192	0.282
Côte d'Ivoire	Marahoué	2000-2017	-0.052	-0.027	-0.003
Côte d'Ivoire	Moronou	2000	0.211	0.329	0.487
Côte d'Ivoire	Moronou	2017	0.121	0.196	0.295
Côte d'Ivoire	Moronou	2000-2017	-0.052	-0.027	0.001
Côte d'Ivoire	N'zi	2000	0.212	0.327	0.486
Côte d'Ivoire	N'zi	2017	0.118	0.194	0.299
Côte d'Ivoire	N'zi	2000-2017	-0.054	-0.027	-0.001
Côte d'Ivoire	Nawa	2000	0.256	0.406	0.624
Côte d'Ivoire	Nawa	2017	0.129	0.206	0.312
Côte d'Ivoire	Nawa	2000-2017	-0.060	-0.035	-0.008
Côte d'Ivoire	Poro	2000	0.213	0.319	0.489
Côte d'Ivoire	Poro	2017	0.114	0.186	0.278
Côte d'Ivoire	Poro	2000-2017	-0.051	-0.027	-0.003
Côte d'Ivoire	San-Pédro	2000	0.264	0.417	0.634
Côte d'Ivoire	San-Pédro	2017	0.129	0.215	0.332
Côte d'Ivoire	San-Pédro	2000-2017	-0.059	-0.032	-0.003
Côte d'Ivoire	Sud Comoé	2000	0.210	0.344	0.500
Côte d'Ivoire	Sud Comoé	2017	0.111	0.196	0.301
Côte d'Ivoire	Sud Comoé	2000-2017	-0.055	-0.029	0.001
Côte d'Ivoire	Tchologo	2000	0.226	0.335	0.511
Côte d'Ivoire	Tchologo	2017	0.118	0.192	0.294
Côte d'Ivoire	Tchologo	2000-2017	-0.052	-0.029	-0.003
Côte d'Ivoire	Tonkpi	2000	0.213	0.340	0.511
Côte d'Ivoire	Tonkpi	2017	0.139	0.213	0.300
Côte d'Ivoire	Tonkpi	2000-2017	-0.049	-0.022	0.004
Côte d'Ivoire	Worodougou	2000	0.186	0.283	0.416
Côte d'Ivoire	Worodougou	2017	0.108	0.176	0.251
Côte d'Ivoire	Worodougou	2000-2017	-0.046	-0.023	0.001
Côte d'Ivoire	Yamoussoukro	2000	0.215	0.326	0.475
Côte d'Ivoire	Yamoussoukro	2017	0.121	0.197	0.290
Côte d'Ivoire	Yamoussoukro	2000-2017	-0.052	-0.026	0.002
Democratic Republic of the Congo	Aba	2000	0.252	0.402	0.590
Democratic Republic of the Congo	Aba	2017	0.143	0.227	0.343
Democratic Republic of the Congo	Aba	2000-2017	-0.072	-0.038	-0.005
Democratic Republic of the Congo	Aketi	2000	0.270	0.399	0.549
Democratic Republic of the Congo	Aketi	2017	0.162	0.236	0.330
Democratic Republic of the Congo	Aketi	2000-2017	-0.042	-0.031	-0.020
Democratic Republic of the Congo	Aketi (ville)	2000	0.262	0.401	0.560
Democratic Republic of the Congo	Aketi (ville)	2017	0.155	0.231	0.340
Democratic Republic of the Congo	Aketi (ville)	2000-2017	-0.058	-0.033	-0.011
Democratic Republic of the Congo	Ango	2000	0.279	0.402	0.554
Democratic Republic of the Congo	Ango	2017	0.162	0.233	0.324
Democratic Republic of the Congo	Ango	2000-2017	-0.042	-0.031	-0.020
Democratic Republic of the Congo	Ariwara	2000	0.324	0.603	0.986
Democratic Republic of the Congo	Ariwara	2017	0.138	0.233	0.370
Democratic Republic of the Congo	Ariwara	2000-2017	-0.094	-0.054	-0.015
Democratic Republic of the Congo	Aru	2000	0.331	0.511	0.742
Democratic Republic of the Congo	Aru	2017	0.163	0.237	0.331
Democratic Republic of the Congo	Aru	2000-2017	-0.063	-0.047	-0.033
Democratic Republic of the Congo	Aru (ville)	2000	0.348	0.546	0.856

Table 1: LRI DALYs rate by unit *(continued)*

Country	Unit	year	mean rate	lower rate	upper rate
Democratic Republic of the Congo	Aru (ville)	2017	0.160	0.240	0.340
Democratic Republic of the Congo	Aru (ville)	2000-2017	-0.080	-0.051	-0.024
Democratic Republic of the Congo	Bafwasende	2000	0.248	0.357	0.489
Democratic Republic of the Congo	Bafwasende	2017	0.160	0.232	0.325
Democratic Republic of the Congo	Bafwasende	2000-2017	-0.033	-0.024	-0.016
Democratic Republic of the Congo	Bagata	2000	0.264	0.397	0.546
Democratic Republic of the Congo	Bagata	2017	0.157	0.228	0.320
Democratic Republic of the Congo	Bagata	2000-2017	-0.045	-0.031	-0.017
Democratic Republic of the Congo	Bambesa	2000	0.275	0.403	0.571
Democratic Republic of the Congo	Bambesa	2017	0.158	0.230	0.324
Democratic Republic of the Congo	Bambesa	2000-2017	-0.043	-0.031	-0.018
Democratic Republic of the Congo	Banalia	2000	0.249	0.363	0.502
Democratic Republic of the Congo	Banalia	2017	0.161	0.234	0.325
Democratic Republic of the Congo	Banalia	2000-2017	-0.035	-0.027	-0.018
Democratic Republic of the Congo	Bandundu	2000	0.243	0.377	0.554
Democratic Republic of the Congo	Bandundu	2017	0.111	0.175	0.253
Democratic Republic of the Congo	Bandundu	2000-2017	-0.071	-0.043	-0.015
Democratic Republic of the Congo	Bangu	2000	0.183	0.270	0.386
Democratic Republic of the Congo	Bangu	2017	0.119	0.194	0.283
Democratic Republic of the Congo	Bangu	2000-2017	-0.055	-0.025	0.002
Democratic Republic of the Congo	Baraka	2000	0.225	0.405	0.650
Democratic Republic of the Congo	Baraka	2017	0.117	0.217	0.374
Democratic Republic of the Congo	Baraka	2000-2017	-0.095	-0.040	0.014
Democratic Republic of the Congo	Basankusu	2000	0.369	0.606	0.907
Democratic Republic of the Congo	Basankusu	2017	0.173	0.252	0.354
Democratic Republic of the Congo	Basankusu	2000-2017	-0.068	-0.049	-0.031
Democratic Republic of the Congo	Basankusu (ville)	2000	0.326	0.561	0.879
Democratic Republic of the Congo	Basankusu (ville)	2017	0.167	0.247	0.362
Democratic Republic of the Congo	Basankusu (ville)	2000-2017	-0.075	-0.045	-0.015
Democratic Republic of the Congo	Basoko	2000	0.258	0.383	0.527
Democratic Republic of the Congo	Basoko	2017	0.164	0.238	0.341
Democratic Republic of the Congo	Basoko	2000-2017	-0.042	-0.030	-0.015
Democratic Republic of the Congo	Basoko (ville)	2000	0.232	0.400	0.588
Democratic Republic of the Congo	Basoko (ville)	2017	0.143	0.233	0.357
Democratic Republic of the Congo	Basoko (ville)	2000-2017	-0.071	-0.033	0.007
Democratic Republic of the Congo	Befale	2000	0.343	0.540	0.797
Democratic Republic of the Congo	Befale	2017	0.170	0.250	0.358
Democratic Republic of the Congo	Befale	2000-2017	-0.065	-0.046	-0.026
Democratic Republic of the Congo	Bena-Dibele	2000	NA	NA	NA
Democratic Republic of the Congo	Bena-Dibele	2017	NA	NA	NA
Democratic Republic of the Congo	Bena-Dibele	2000-2017	NA	NA	NA
Democratic Republic of the Congo	Beni	2000	0.313	0.484	0.711
Democratic Republic of the Congo	Beni	2017	0.136	0.204	0.282
Democratic Republic of the Congo	Beni	2000-2017	-0.074	-0.049	-0.025
Democratic Republic of the Congo	Bikoro	2000	0.374	0.593	0.887
Democratic Republic of the Congo	Bikoro	2017	0.175	0.248	0.349
Democratic Republic of the Congo	Bikoro	2000-2017	-0.066	-0.049	-0.033
Democratic Republic of the Congo	Boende	2000	0.359	0.566	0.837
Democratic Republic of the Congo	Boende	2017	0.174	0.248	0.349
Democratic Republic of the Congo	Boende	2000-2017	-0.068	-0.049	-0.030
Democratic Republic of the Congo	Boende (ville)	2000	0.281	0.487	0.822
Democratic Republic of the Congo	Boende (ville)	2017	0.144	0.244	0.392
Democratic Republic of the Congo	Boende (ville)	2000-2017	-0.086	-0.042	0.002
Democratic Republic of the Congo	Bokungu	2000	0.328	0.518	0.765
Democratic Republic of the Congo	Bokungu	2017	0.172	0.247	0.339
Democratic Republic of the Congo	Bokungu	2000-2017	-0.062	-0.047	-0.030
Democratic Republic of the Congo	Bolobo	2000	0.267	0.398	0.546
Democratic Republic of the Congo	Bolobo	2017	0.160	0.232	0.329
Democratic Republic of the Congo	Bolobo	2000-2017	-0.044	-0.031	-0.016
Democratic Republic of the Congo	Bolobo (ville)	2000	0.259	0.394	0.557
Democratic Republic of the Congo	Bolobo (ville)	2017	0.159	0.235	0.342

Table 1: LRI DALYs rate by unit *(continued)*

Country	Unit	year	mean rate	lower rate	upper rate
Democratic Republic of the Congo	Bolobo (ville)	2000-2017	-0.054	-0.029	0.003
Democratic Republic of the Congo	Bolomba	2000	0.368	0.592	0.885
Democratic Republic of the Congo	Bolomba	2017	0.166	0.245	0.346
Democratic Republic of the Congo	Bolomba	2000-2017	-0.065	-0.049	-0.031
Democratic Republic of the Congo	Boma	2000	0.174	0.251	0.354
Democratic Republic of the Congo	Boma	2017	0.110	0.175	0.257
Democratic Republic of the Congo	Boma	2000-2017	-0.051	-0.025	0.004
Democratic Republic of the Congo	Bomongo	2000	0.335	0.528	0.769
Democratic Republic of the Congo	Bomongo	2017	0.164	0.244	0.347
Democratic Republic of the Congo	Bomongo	2000-2017	-0.061	-0.044	-0.027
Democratic Republic of the Congo	Bondo	2000	0.294	0.427	0.592
Democratic Republic of the Congo	Bondo	2017	0.170	0.240	0.330
Democratic Republic of the Congo	Bondo	2000-2017	-0.043	-0.032	-0.021
Democratic Republic of the Congo	Bondo (ville)	2000	0.243	0.401	0.633
Democratic Republic of the Congo	Bondo (ville)	2017	0.144	0.236	0.371
Democratic Republic of the Congo	Bondo (ville)	2000-2017	-0.072	-0.032	0.008
Democratic Republic of the Congo	Bongandanga	2000	0.370	0.606	0.911
Democratic Republic of the Congo	Bongandanga	2017	0.173	0.251	0.354
Democratic Republic of the Congo	Bongandanga	2000-2017	-0.073	-0.052	-0.033
Democratic Republic of the Congo	Bosobolo	2000	0.371	0.599	0.897
Democratic Republic of the Congo	Bosobolo	2017	0.187	0.269	0.374
Democratic Republic of the Congo	Bosobolo	2000-2017	-0.062	-0.045	-0.028
Democratic Republic of the Congo	Budjala	2000	0.373	0.601	0.879
Democratic Republic of the Congo	Budjala	2017	0.175	0.255	0.352
Democratic Republic of the Congo	Budjala	2000-2017	-0.068	-0.049	-0.029
Democratic Republic of the Congo	Bukama	2000	0.370	0.599	0.908
Democratic Republic of the Congo	Bukama	2017	0.176	0.254	0.354
Democratic Republic of the Congo	Bukama	2000-2017	-0.068	-0.050	-0.033
Democratic Republic of the Congo	Bukavu	2000	0.290	0.469	0.675
Democratic Republic of the Congo	Bukavu	2017	0.140	0.244	0.390
Democratic Republic of the Congo	Bukavu	2000-2017	-0.066	-0.036	-0.005
Democratic Republic of the Congo	Bulungu	2000	0.260	0.390	0.536
Democratic Republic of the Congo	Bulungu	2017	0.145	0.216	0.308
Democratic Republic of the Congo	Bulungu	2000-2017	-0.054	-0.036	-0.020
Democratic Republic of the Congo	Bulungu (ville)	2000	0.255	0.399	0.597
Democratic Republic of the Congo	Bulungu (ville)	2017	0.124	0.206	0.318
Democratic Republic of the Congo	Bulungu (ville)	2000-2017	-0.080	-0.039	0.001
Democratic Republic of the Congo	Bumba	2000	0.365	0.591	0.876
Democratic Republic of the Congo	Bumba	2017	0.176	0.256	0.355
Democratic Republic of the Congo	Bumba	2000-2017	-0.071	-0.052	-0.033
Democratic Republic of the Congo	Bumba (ville)	2000	0.304	0.522	0.814
Democratic Republic of the Congo	Bumba (ville)	2017	0.134	0.219	0.338
Democratic Republic of the Congo	Bumba (ville)	2000-2017	-0.092	-0.052	-0.014
Democratic Republic of the Congo	Bunia	2000	0.255	0.380	0.565
Democratic Republic of the Congo	Bunia	2017	0.142	0.216	0.311
Democratic Republic of the Congo	Bunia	2000-2017	-0.059	-0.031	-0.001
Democratic Republic of the Congo	Businga	2000	0.376	0.596	0.889
Democratic Republic of the Congo	Businga	2017	0.182	0.263	0.368
Democratic Republic of the Congo	Businga	2000-2017	-0.065	-0.047	-0.030
Democratic Republic of the Congo	Buta	2000	0.268	0.399	0.563
Democratic Republic of the Congo	Buta	2017	0.156	0.230	0.320
Democratic Republic of the Congo	Buta	2000-2017	-0.044	-0.032	-0.021
Democratic Republic of the Congo	Buta (ville)	2000	0.215	0.332	0.502
Democratic Republic of the Congo	Buta (ville)	2017	0.137	0.224	0.339
Democratic Republic of the Congo	Buta (ville)	2000-2017	-0.063	-0.024	0.013
Democratic Republic of the Congo	Butembo	2000	0.315	0.516	0.792
Democratic Republic of the Congo	Butembo	2017	0.135	0.214	0.325
Democratic Republic of the Congo	Butembo	2000-2017	-0.074	-0.050	-0.029
Democratic Republic of the Congo	Dekese	2000	0.333	0.526	0.765
Democratic Republic of the Congo	Dekese	2017	0.170	0.247	0.346
Democratic Republic of the Congo	Dekese	2000-2017	-0.058	-0.042	-0.026

Table 1: LRI DALYs rate by unit *(continued)*

Country	Unit	year	mean rate	lower rate	upper rate
Democratic Republic of the Congo	Demba	2000	0.362	0.574	0.856
Democratic Republic of the Congo	Demba	2017	0.157	0.228	0.322
Democratic Republic of the Congo	Demba	2000-2017	-0.072	-0.053	-0.037
Democratic Republic of the Congo	Dibaya	2000	0.380	0.605	0.932
Democratic Republic of the Congo	Dibaya	2017	0.170	0.241	0.338
Democratic Republic of the Congo	Dibaya	2000-2017	-0.072	-0.053	-0.036
Democratic Republic of the Congo	Dibaya-Lubwe	2000	0.222	0.407	0.667
Democratic Republic of the Congo	Dibaya-Lubwe	2017	0.121	0.221	0.371
Democratic Republic of the Congo	Dibaya-Lubwe	2000-2017	-0.085	-0.037	0.016
Democratic Republic of the Congo	Dilolo	2000	0.365	0.581	0.873
Democratic Republic of the Congo	Dilolo	2017	0.190	0.277	0.377
Democratic Republic of the Congo	Dilolo	2000-2017	-0.050	-0.038	-0.025
Democratic Republic of the Congo	Dimbelenge	2000	0.383	0.609	0.923
Democratic Republic of the Congo	Dimbelenge	2017	0.168	0.242	0.344
Democratic Republic of the Congo	Dimbelenge	2000-2017	-0.073	-0.053	-0.036
Democratic Republic of the Congo	Dingila	2000	0.231	0.420	0.706
Democratic Republic of the Congo	Dingila	2017	0.134	0.231	0.382
Democratic Republic of the Congo	Dingila	2000-2017	-0.094	-0.031	0.019
Democratic Republic of the Congo	Djolu	2000	0.342	0.540	0.790
Democratic Republic of the Congo	Djolu	2017	0.172	0.250	0.347
Democratic Republic of the Congo	Djolu	2000-2017	-0.063	-0.045	-0.025
Democratic Republic of the Congo	Djugu	2000	0.290	0.426	0.596
Democratic Republic of the Congo	Djugu	2017	0.167	0.241	0.338
Democratic Republic of the Congo	Djugu	2000-2017	-0.045	-0.032	-0.020
Democratic Republic of the Congo	Dungu	2000	0.257	0.385	0.526
Democratic Republic of the Congo	Dungu	2017	0.160	0.236	0.332
Democratic Republic of the Congo	Dungu	2000-2017	-0.042	-0.030	-0.021
Democratic Republic of the Congo	Dungu (ville)	2000	0.244	0.394	0.585
Democratic Republic of the Congo	Dungu (ville)	2017	0.149	0.234	0.345
Democratic Republic of the Congo	Dungu (ville)	2000-2017	-0.062	-0.032	-0.002
Democratic Republic of the Congo	Faradje	2000	0.273	0.413	0.572
Democratic Republic of the Congo	Faradje	2017	0.157	0.229	0.319
Democratic Republic of the Congo	Faradje	2000-2017	-0.046	-0.034	-0.023
Democratic Republic of the Congo	Feshi	2000	0.296	0.454	0.641
Democratic Republic of the Congo	Feshi	2017	0.158	0.232	0.333
Democratic Republic of the Congo	Feshi	2000-2017	-0.061	-0.043	-0.024
Democratic Republic of the Congo	Fizi	2000	0.384	0.625	0.965
Democratic Republic of the Congo	Fizi	2017	0.183	0.268	0.377
Democratic Republic of the Congo	Fizi	2000-2017	-0.056	-0.041	-0.028
Democratic Republic of the Congo	Gbadolite	2000	0.344	0.530	0.782
Democratic Republic of the Congo	Gbadolite	2017	0.157	0.237	0.330
Democratic Republic of the Congo	Gbadolite	2000-2017	-0.066	-0.046	-0.026
Democratic Republic of the Congo	Gemena	2000	0.375	0.602	0.909
Democratic Republic of the Congo	Gemena	2017	0.181	0.260	0.363
Democratic Republic of the Congo	Gemena	2000-2017	-0.066	-0.047	-0.029
Democratic Republic of the Congo	Gemena (ville)	2000	0.356	0.612	0.983
Democratic Republic of the Congo	Gemena (ville)	2017	0.139	0.223	0.342
Democratic Republic of the Congo	Gemena (ville)	2000-2017	-0.096	-0.062	-0.026
Democratic Republic of the Congo	Goma	2000	0.223	0.346	0.515
Democratic Republic of the Congo	Goma	2017	0.131	0.214	0.329
Democratic Republic of the Congo	Goma	2000-2017	-0.063	-0.030	0.001
Democratic Republic of the Congo	Gungu	2000	0.272	0.406	0.567
Democratic Republic of the Congo	Gungu	2017	0.158	0.234	0.332
Democratic Republic of the Congo	Gungu	2000-2017	-0.051	-0.034	-0.016
Democratic Republic of the Congo	Gungu (ville)	2000	0.238	0.390	0.583
Democratic Republic of the Congo	Gungu (ville)	2017	0.144	0.243	0.414
Democratic Republic of the Congo	Gungu (ville)	2000-2017	-0.076	-0.033	0.010
Democratic Republic of the Congo	Idiofa	2000	0.264	0.394	0.548
Democratic Republic of the Congo	Idiofa	2017	0.148	0.218	0.311
Democratic Republic of the Congo	Idiofa	2000-2017	-0.052	-0.036	-0.019
Democratic Republic of the Congo	Idiofa (ville)	2000	0.240	0.399	0.556

Table 1: LRI DALYs rate by unit *(continued)*

Country	Unit	year	mean rate	lower rate	upper rate
Democratic Republic of the Congo	Idiofa (ville)	2017	0.133	0.224	0.353
Democratic Republic of the Congo	Idiofa (ville)	2000-2017	-0.070	-0.033	0.000
Democratic Republic of the Congo	Idjwi	2000	0.344	0.556	0.856
Democratic Republic of the Congo	Idjwi	2017	0.174	0.260	0.376
Democratic Republic of the Congo	Idjwi	2000-2017	-0.061	-0.042	-0.026
Democratic Republic of the Congo	Ikela	2000	0.339	0.526	0.772
Democratic Republic of the Congo	Ikela	2017	0.169	0.246	0.344
Democratic Republic of the Congo	Ikela	2000-2017	-0.060	-0.043	-0.026
Democratic Republic of the Congo	Ilebo	2000	0.332	0.528	0.773
Democratic Republic of the Congo	Ilebo	2017	0.162	0.239	0.334
Democratic Republic of the Congo	Ilebo	2000-2017	-0.063	-0.046	-0.029
Democratic Republic of the Congo	Ilebo (ville)	2000	0.294	0.469	0.714
Democratic Republic of the Congo	Ilebo (ville)	2017	0.136	0.215	0.315
Democratic Republic of the Congo	Ilebo (ville)	2000-2017	-0.075	-0.047	-0.016
Democratic Republic of the Congo	Ingbokolo	2000	0.239	0.416	0.717
Democratic Republic of the Congo	Ingbokolo	2017	0.134	0.236	0.414
Democratic Republic of the Congo	Ingbokolo	2000-2017	-0.085	-0.030	0.029
Democratic Republic of the Congo	Ingende	2000	0.365	0.595	0.878
Democratic Republic of the Congo	Ingende	2017	0.172	0.247	0.338
Democratic Republic of the Congo	Ingende	2000-2017	-0.068	-0.051	-0.035
Democratic Republic of the Congo	Inkisi	2000	0.174	0.279	0.409
Democratic Republic of the Congo	Inkisi	2017	0.106	0.178	0.288
Democratic Republic of the Congo	Inkisi	2000-2017	-0.062	-0.029	0.004
Democratic Republic of the Congo	Inongo	2000	0.308	0.471	0.661
Democratic Republic of the Congo	Inongo	2017	0.159	0.230	0.320
Democratic Republic of the Congo	Inongo	2000-2017	-0.053	-0.039	-0.025
Democratic Republic of the Congo	Inongo (ville)	2000	0.242	0.396	0.591
Democratic Republic of the Congo	Inongo (ville)	2017	0.145	0.226	0.352
Democratic Republic of the Congo	Inongo (ville)	2000-2017	-0.064	-0.031	0.000
Democratic Republic of the Congo	Irumu	2000	0.280	0.414	0.594
Democratic Republic of the Congo	Irumu	2017	0.163	0.238	0.332
Democratic Republic of the Congo	Irumu	2000-2017	-0.042	-0.031	-0.019
Democratic Republic of the Congo	Isangi	2000	0.284	0.418	0.586
Democratic Republic of the Congo	Isangi	2017	0.159	0.230	0.323
Democratic Republic of the Congo	Isangi	2000-2017	-0.051	-0.037	-0.022
Democratic Republic of the Congo	Isangi (ville)	2000	0.246	0.421	0.681
Democratic Republic of the Congo	Isangi (ville)	2017	0.125	0.234	0.381
Democratic Republic of the Congo	Isangi (ville)	2000-2017	-0.083	-0.035	0.011
Democratic Republic of the Congo	Isiro	2000	0.216	0.350	0.536
Democratic Republic of the Congo	Isiro	2017	0.130	0.209	0.330
Democratic Republic of the Congo	Isiro	2000-2017	-0.062	-0.029	0.000
Democratic Republic of the Congo	Kabalo	2000	0.369	0.598	0.880
Democratic Republic of the Congo	Kabalo	2017	0.174	0.253	0.354
Democratic Republic of the Congo	Kabalo	2000-2017	-0.061	-0.046	-0.031
Democratic Republic of the Congo	Kabambare	2000	0.376	0.611	0.921
Democratic Republic of the Congo	Kabambare	2017	0.176	0.252	0.345
Democratic Republic of the Congo	Kabambare	2000-2017	-0.065	-0.048	-0.034
Democratic Republic of the Congo	Kabare	2000	0.379	0.664	1.021
Democratic Republic of the Congo	Kabare	2017	0.180	0.266	0.386
Democratic Republic of the Congo	Kabare	2000-2017	-0.068	-0.050	-0.031
Democratic Republic of the Congo	Kabeya-Kamwanga	2000	0.380	0.611	0.940
Democratic Republic of the Congo	Kabeya-Kamwanga	2017	0.165	0.242	0.348
Democratic Republic of the Congo	Kabeya-Kamwanga	2000-2017	-0.072	-0.053	-0.032
Democratic Republic of the Congo	Kabinda	2000	0.355	0.558	0.833
Democratic Republic of the Congo	Kabinda	2017	0.172	0.244	0.339
Democratic Republic of the Congo	Kabinda	2000-2017	-0.065	-0.049	-0.035
Democratic Republic of the Congo	Kabinda (ville)	2000	0.302	0.501	0.762
Democratic Republic of the Congo	Kabinda (ville)	2017	0.141	0.226	0.333
Democratic Republic of the Congo	Kabinda (ville)	2000-2017	-0.079	-0.046	-0.011
Democratic Republic of the Congo	Kabongo	2000	0.358	0.577	0.864
Democratic Republic of the Congo	Kabongo	2017	0.169	0.245	0.335

Table 1: LRI DALYs rate by unit *(continued)*

Country	Unit	year	mean rate	lower rate	upper rate
Democratic Republic of the Congo	Kabongo	2000-2017	-0.067	-0.050	-0.035
Democratic Republic of the Congo	Kahemba	2000	0.324	0.499	0.712
Democratic Republic of the Congo	Kahemba	2017	0.170	0.248	0.351
Democratic Republic of the Congo	Kahemba	2000-2017	-0.058	-0.042	-0.028
Democratic Republic of the Congo	Kahemba (ville)	2000	0.308	0.497	0.738
Democratic Republic of the Congo	Kahemba (ville)	2017	0.162	0.256	0.366
Democratic Republic of the Congo	Kahemba (ville)	2000-2017	-0.066	-0.037	-0.007
Democratic Republic of the Congo	Kailo	2000	0.334	0.527	0.802
Democratic Republic of the Congo	Kailo	2017	0.168	0.243	0.341
Democratic Republic of the Congo	Kailo	2000-2017	-0.053	-0.038	-0.027
Democratic Republic of the Congo	Kalehe	2000	0.408	0.700	1.100
Democratic Republic of the Congo	Kalehe	2017	0.194	0.282	0.402
Democratic Republic of the Congo	Kalehe	2000-2017	-0.065	-0.047	-0.033
Democratic Republic of the Congo	Kalemie	2000	0.334	0.511	0.727
Democratic Republic of the Congo	Kalemie	2017	0.163	0.240	0.334
Democratic Republic of the Congo	Kalemie	2000-2017	-0.057	-0.043	-0.028
Democratic Republic of the Congo	Kalemie (ville)	2000	0.247	0.408	0.625
Democratic Republic of the Congo	Kalemie (ville)	2017	0.135	0.204	0.302
Democratic Republic of the Congo	Kalemie (ville)	2000-2017	-0.070	-0.038	-0.008
Democratic Republic of the Congo	Kalima	2000	0.355	0.582	0.885
Democratic Republic of the Congo	Kalima	2017	0.172	0.261	0.347
Democratic Republic of the Congo	Kalima	2000-2017	-0.060	-0.037	-0.015
Democratic Republic of the Congo	Kambove	2000	0.351	0.553	0.801
Democratic Republic of the Congo	Kambove	2017	0.166	0.243	0.337
Democratic Republic of the Congo	Kambove	2000-2017	-0.062	-0.047	-0.032
Democratic Republic of the Congo	Kamiji	2000	0.372	0.602	0.922
Democratic Republic of the Congo	Kamiji	2017	0.165	0.239	0.327
Democratic Republic of the Congo	Kamiji	2000-2017	-0.074	-0.055	-0.036
Democratic Republic of the Congo	Kamina	2000	0.375	0.603	0.898
Democratic Republic of the Congo	Kamina	2017	0.175	0.251	0.346
Democratic Republic of the Congo	Kamina	2000-2017	-0.066	-0.049	-0.032
Democratic Republic of the Congo	Kamina (ville)	2000	0.278	0.472	0.743
Democratic Republic of the Congo	Kamina (ville)	2017	0.136	0.208	0.308
Democratic Republic of the Congo	Kamina (ville)	2000-2017	-0.083	-0.046	-0.012
Democratic Republic of the Congo	Kamituga	2000	0.306	0.576	1.013
Democratic Republic of the Congo	Kamituga	2017	0.156	0.272	0.428
Democratic Republic of the Congo	Kamituga	2000-2017	-0.081	-0.029	0.023
Democratic Republic of the Congo	Kamonia	2000	0.370	0.599	0.894
Democratic Republic of the Congo	Kamonia	2017	0.175	0.250	0.348
Democratic Republic of the Congo	Kamonia	2000-2017	-0.068	-0.051	-0.035
Democratic Republic of the Congo	Kananga	2000	0.320	0.490	0.713
Democratic Republic of the Congo	Kananga	2017	0.134	0.196	0.279
Democratic Republic of the Congo	Kananga	2000-2017	-0.073	-0.052	-0.031
Democratic Republic of the Congo	Kaniama	2000	0.371	0.606	0.910
Democratic Republic of the Congo	Kaniama	2017	0.173	0.245	0.338
Democratic Republic of the Congo	Kaniama	2000-2017	-0.073	-0.055	-0.037
Democratic Republic of the Congo	Kaoze	2000	0.295	0.507	0.829
Democratic Republic of the Congo	Kaoze	2017	0.134	0.225	0.374
Democratic Republic of the Congo	Kaoze	2000-2017	-0.092	-0.047	0.000
Democratic Republic of the Congo	Kapanga	2000	0.374	0.604	0.914
Democratic Republic of the Congo	Kapanga	2017	0.186	0.267	0.364
Democratic Republic of the Congo	Kapanga	2000-2017	-0.062	-0.047	-0.032
Democratic Republic of the Congo	Kasaji	2000	0.358	0.602	0.960
Democratic Republic of the Congo	Kasaji	2017	0.152	0.253	0.380
Democratic Republic of the Congo	Kasaji	2000-2017	-0.081	-0.045	-0.010
Democratic Republic of the Congo	Kasangulu	2000	0.231	0.331	0.446
Democratic Republic of the Congo	Kasangulu	2017	0.138	0.206	0.290
Democratic Republic of the Congo	Kasangulu	2000-2017	-0.044	-0.032	-0.020
Democratic Republic of the Congo	Kasangulu (ville)	2000	0.179	0.282	0.456
Democratic Republic of the Congo	Kasangulu (ville)	2017	0.104	0.179	0.299
Democratic Republic of the Congo	Kasangulu (ville)	2000-2017	-0.071	-0.031	0.010

Table 1: LRI DALYs rate by unit *(continued)*

Country	Unit	year	mean rate	lower rate	upper rate
Democratic Republic of the Congo	Kasenga	2000	0.364	0.579	0.863
Democratic Republic of the Congo	Kasenga	2017	0.181	0.255	0.354
Democratic Republic of the Congo	Kasenga	2000-2017	-0.065	-0.049	-0.036
Democratic Republic of the Congo	Kasongo	2000	0.375	0.610	0.929
Democratic Republic of the Congo	Kasongo	2017	0.170	0.243	0.337
Democratic Republic of the Congo	Kasongo	2000-2017	-0.069	-0.052	-0.036
Democratic Republic of the Congo	Kasongo (ville)	2000	0.360	0.596	0.920
Democratic Republic of the Congo	Kasongo (ville)	2017	0.158	0.247	0.350
Democratic Republic of the Congo	Kasongo (ville)	2000-2017	-0.076	-0.050	-0.027
Democratic Republic of the Congo	Kasongo-Lunda	2000	0.293	0.441	0.628
Democratic Republic of the Congo	Kasongo-Lunda	2017	0.160	0.236	0.330
Democratic Republic of the Congo	Kasongo-Lunda	2000-2017	-0.055	-0.040	-0.025
Democratic Republic of the Congo	Kasongo-Lunda (ville)	2000	0.196	0.358	0.648
Democratic Republic of the Congo	Kasongo-Lunda (ville)	2017	0.128	0.227	0.339
Democratic Republic of the Congo	Kasongo-Lunda (ville)	2000-2017	-0.090	-0.034	0.019
Democratic Republic of the Congo	Katako-Kombe	2000	0.320	0.498	0.721
Democratic Republic of the Congo	Katako-Kombe	2017	0.169	0.244	0.341
Democratic Republic of the Congo	Katako-Kombe	2000-2017	-0.053	-0.037	-0.022
Democratic Republic of the Congo	Katanda	2000	0.278	0.434	0.639
Democratic Republic of the Congo	Katanda	2017	0.125	0.189	0.276
Democratic Republic of the Congo	Katanda	2000-2017	-0.078	-0.048	-0.015
Democratic Republic of the Congo	Kazumba	2000	0.366	0.597	0.903
Democratic Republic of the Congo	Kazumba	2017	0.172	0.248	0.344
Democratic Republic of the Congo	Kazumba	2000-2017	-0.070	-0.052	-0.035
Democratic Republic of the Congo	Kenge	2000	0.260	0.399	0.563
Democratic Republic of the Congo	Kenge	2017	0.160	0.231	0.326
Democratic Republic of the Congo	Kenge	2000-2017	-0.049	-0.034	-0.018
Democratic Republic of the Congo	Kenge (ville)	2000	0.243	0.395	0.587
Democratic Republic of the Congo	Kenge (ville)	2017	0.144	0.232	0.346
Democratic Republic of the Congo	Kenge (ville)	2000-2017	-0.069	-0.034	-0.005
Democratic Republic of the Congo	Kibombo	2000	0.367	0.589	0.905
Democratic Republic of the Congo	Kibombo	2017	0.172	0.243	0.339
Democratic Republic of the Congo	Kibombo	2000-2017	-0.063	-0.048	-0.034
Democratic Republic of the Congo	Kikwit	2000	0.218	0.337	0.485
Democratic Republic of the Congo	Kikwit	2017	0.110	0.177	0.292
Democratic Republic of the Congo	Kikwit	2000-2017	-0.067	-0.035	-0.002
Democratic Republic of the Congo	Kimvula	2000	0.222	0.319	0.437
Democratic Republic of the Congo	Kimvula	2017	0.151	0.220	0.308
Democratic Republic of the Congo	Kimvula	2000-2017	-0.035	-0.023	-0.012
Democratic Republic of the Congo	Kindu	2000	0.307	0.490	0.792
Democratic Republic of the Congo	Kindu	2017	0.131	0.197	0.294
Democratic Republic of the Congo	Kindu	2000-2017	-0.076	-0.046	-0.020
Democratic Republic of the Congo	Kinshasa	2000	0.163	0.228	0.311
Democratic Republic of the Congo	Kinshasa	2017	0.107	0.162	0.230
Democratic Republic of the Congo	Kinshasa	2000-2017	-0.037	-0.020	-0.004
Democratic Republic of the Congo	Kipushi	2000	0.353	0.554	0.822
Democratic Republic of the Congo	Kipushi	2017	0.160	0.235	0.331
Democratic Republic of the Congo	Kipushi	2000-2017	-0.063	-0.047	-0.032
Democratic Republic of the Congo	Kipushi (ville)	2000	0.208	0.320	0.511
Democratic Republic of the Congo	Kipushi (ville)	2017	0.110	0.189	0.294
Democratic Republic of the Congo	Kipushi (ville)	2000-2017	-0.062	-0.029	0.007
Democratic Republic of the Congo	Kiri	2000	0.308	0.479	0.677
Democratic Republic of the Congo	Kiri	2017	0.160	0.231	0.320
Democratic Republic of the Congo	Kiri	2000-2017	-0.060	-0.043	-0.028
Democratic Republic of the Congo	Kisangani	2000	0.253	0.369	0.520
Democratic Republic of the Congo	Kisangani	2017	0.125	0.188	0.268
Democratic Republic of the Congo	Kisangani	2000-2017	-0.056	-0.040	-0.023
Democratic Republic of the Congo	Kole	2000	0.329	0.511	0.758
Democratic Republic of the Congo	Kole	2017	0.169	0.247	0.347
Democratic Republic of the Congo	Kole	2000-2017	-0.059	-0.040	-0.023
Democratic Republic of the Congo	Kolwezi	2000	0.278	0.416	0.616

Table 1: LRI DALYs rate by unit *(continued)*

Country	Unit	year	mean rate	lower rate	upper rate
Democratic Republic of the Congo	Kolwezi	2017	0.139	0.205	0.294
Democratic Republic of the Congo	Kolwezi	2000-2017	-0.067	-0.042	-0.015
Democratic Republic of the Congo	Kongolo	2000	0.372	0.603	0.918
Democratic Republic of the Congo	Kongolo	2017	0.176	0.258	0.358
Democratic Republic of the Congo	Kongolo	2000-2017	-0.065	-0.048	-0.032
Democratic Republic of the Congo	Kongolo (ville)	2000	0.332	0.575	0.934
Democratic Republic of the Congo	Kongolo (ville)	2017	0.160	0.242	0.358
Democratic Republic of the Congo	Kongolo (ville)	2000-2017	-0.080	-0.047	-0.013
Democratic Republic of the Congo	Kungu	2000	0.368	0.598	0.912
Democratic Republic of the Congo	Kungu	2017	0.174	0.252	0.354
Democratic Republic of the Congo	Kungu	2000-2017	-0.067	-0.048	-0.029
Democratic Republic of the Congo	Kutu	2000	0.270	0.398	0.554
Democratic Republic of the Congo	Kutu	2017	0.157	0.227	0.319
Democratic Republic of the Congo	Kutu	2000-2017	-0.042	-0.030	-0.019
Democratic Republic of the Congo	Kwamouth	2000	0.258	0.390	0.555
Democratic Republic of the Congo	Kwamouth	2017	0.150	0.217	0.300
Democratic Republic of the Congo	Kwamouth	2000-2017	-0.050	-0.035	-0.021
Democratic Republic of the Congo	Libenge	2000	0.373	0.604	0.893
Democratic Republic of the Congo	Libenge	2017	0.179	0.255	0.352
Democratic Republic of the Congo	Libenge	2000-2017	-0.070	-0.049	-0.032
Democratic Republic of the Congo	Likasi	2000	0.290	0.455	0.676
Democratic Republic of the Congo	Likasi	2017	0.140	0.203	0.288
Democratic Republic of the Congo	Likasi	2000-2017	-0.071	-0.048	-0.028
Democratic Republic of the Congo	Lisala	2000	0.370	0.604	0.919
Democratic Republic of the Congo	Lisala	2017	0.174	0.250	0.351
Democratic Republic of the Congo	Lisala	2000-2017	-0.072	-0.051	-0.032
Democratic Republic of the Congo	Lisala (ville)	2000	0.322	0.547	0.935
Democratic Republic of the Congo	Lisala (ville)	2017	0.137	0.217	0.331
Democratic Republic of the Congo	Lisala (ville)	2000-2017	-0.099	-0.058	-0.026
Democratic Republic of the Congo	Lodja	2000	0.342	0.571	0.917
Democratic Republic of the Congo	Lodja	2017	0.168	0.255	0.378
Democratic Republic of the Congo	Lodja	2000-2017	-0.069	-0.043	-0.015
Democratic Republic of the Congo	Lodja (ville)	2000	NA	NA	NA
Democratic Republic of the Congo	Lodja (ville)	2017	NA	NA	NA
Democratic Republic of the Congo	Lodja (ville)	2000-2017	NA	NA	NA
Democratic Republic of the Congo	Lomela	2000	0.307	0.483	0.681
Democratic Republic of the Congo	Lomela	2017	0.169	0.249	0.340
Democratic Republic of the Congo	Lomela	2000-2017	-0.053	-0.036	-0.018
Democratic Republic of the Congo	Lubao	2000	0.361	0.571	0.863
Democratic Republic of the Congo	Lubao	2017	0.170	0.245	0.338
Democratic Republic of the Congo	Lubao	2000-2017	-0.064	-0.048	-0.033
Democratic Republic of the Congo	Lubao (ville)	2000	0.294	0.493	0.765
Democratic Republic of the Congo	Lubao (ville)	2017	0.156	0.249	0.379
Democratic Republic of the Congo	Lubao (ville)	2000-2017	-0.076	-0.039	-0.005
Democratic Republic of the Congo	Lubefu	2000	0.349	0.578	0.886
Democratic Republic of the Congo	Lubefu	2017	0.165	0.244	0.335
Democratic Republic of the Congo	Lubefu	2000-2017	-0.062	-0.046	-0.030
Democratic Republic of the Congo	Lubero	2000	0.387	0.619	0.950
Democratic Republic of the Congo	Lubero	2017	0.183	0.264	0.381
Democratic Republic of the Congo	Lubero	2000-2017	-0.058	-0.042	-0.027
Democratic Republic of the Congo	Lubudi	2000	0.367	0.592	0.875
Democratic Republic of the Congo	Lubudi	2017	0.166	0.243	0.342
Democratic Republic of the Congo	Lubudi	2000-2017	-0.068	-0.051	-0.035
Democratic Republic of the Congo	Lubumbashi	2000	0.275	0.428	0.599
Democratic Republic of the Congo	Lubumbashi	2017	0.138	0.208	0.301
Democratic Republic of the Congo	Lubumbashi	2000-2017	-0.059	-0.041	-0.024
Democratic Republic of the Congo	Lubutu	2000	0.338	0.533	0.788
Democratic Republic of the Congo	Lubutu	2017	0.175	0.249	0.340
Democratic Republic of the Congo	Lubutu	2000-2017	-0.056	-0.042	-0.027
Democratic Republic of the Congo	Lubutu (ville)	2000	0.318	0.522	0.860
Democratic Republic of the Congo	Lubutu (ville)	2017	0.161	0.246	0.357

Table 1: LRI DALYs rate by unit *(continued)*

Country	Unit	year	mean rate	lower rate	upper rate
Democratic Republic of the Congo	Lubutu (ville)	2000-2017	-0.073	-0.041	-0.003
Democratic Republic of the Congo	Luebo	2000	0.366	0.595	0.882
Democratic Republic of the Congo	Luebo	2017	0.161	0.238	0.331
Democratic Republic of the Congo	Luebo	2000-2017	-0.074	-0.054	-0.035
Democratic Republic of the Congo	Luebo (ville)	2000	0.292	0.494	0.752
Democratic Republic of the Congo	Luebo (ville)	2017	0.150	0.246	0.367
Democratic Republic of the Congo	Luebo (ville)	2000-2017	-0.083	-0.043	-0.006
Democratic Republic of the Congo	Luilu	2000	0.349	0.566	0.835
Democratic Republic of the Congo	Luilu	2017	0.175	0.248	0.337
Democratic Republic of the Congo	Luilu	2000-2017	-0.068	-0.051	-0.036
Democratic Republic of the Congo	Luiza	2000	0.380	0.604	0.912
Democratic Republic of the Congo	Luiza	2017	0.191	0.271	0.371
Democratic Republic of the Congo	Luiza	2000-2017	-0.064	-0.047	-0.032
Democratic Republic of the Congo	Lukalaba	2000	0.327	0.614	0.980
Democratic Republic of the Congo	Lukalaba	2017	0.148	0.237	0.352
Democratic Republic of the Congo	Lukalaba	2000-2017	-0.095	-0.056	-0.015
Democratic Republic of the Congo	Lukolela	2000	0.328	0.515	0.754
Democratic Republic of the Congo	Lukolela	2017	0.173	0.249	0.352
Democratic Republic of the Congo	Lukolela	2000-2017	-0.057	-0.041	-0.026
Democratic Republic of the Congo	Lukula	2000	0.199	0.292	0.404
Democratic Republic of the Congo	Lukula	2017	0.143	0.212	0.293
Democratic Republic of the Congo	Lukula	2000-2017	-0.032	-0.021	-0.010
Democratic Republic of the Congo	Lukula (ville)	2000	0.174	0.292	0.470
Democratic Republic of the Congo	Lukula (ville)	2017	0.097	0.177	0.299
Democratic Republic of the Congo	Lukula (ville)	2000-2017	-0.072	-0.029	0.013
Democratic Republic of the Congo	Luozi	2000	0.210	0.298	0.404
Democratic Republic of the Congo	Luozi	2017	0.150	0.219	0.307
Democratic Republic of the Congo	Luozi	2000-2017	-0.034	-0.021	-0.009
Democratic Republic of the Congo	Lupatapata	2000	0.307	0.473	0.705
Democratic Republic of the Congo	Lupatapata	2017	0.140	0.200	0.282
Democratic Republic of the Congo	Lupatapata	2000-2017	-0.069	-0.050	-0.029
Democratic Republic of the Congo	Lusambo	2000	0.373	0.589	0.906
Democratic Republic of the Congo	Lusambo	2017	0.170	0.245	0.353
Democratic Republic of the Congo	Lusambo	2000-2017	-0.067	-0.049	-0.033
Democratic Republic of the Congo	Lusambo (ville)	2000	0.322	0.533	0.813
Democratic Republic of the Congo	Lusambo (ville)	2017	0.163	0.252	0.365
Democratic Republic of the Congo	Lusambo (ville)	2000-2017	-0.073	-0.043	-0.009
Democratic Republic of the Congo	Madimba	2000	0.222	0.328	0.445
Democratic Republic of the Congo	Madimba	2017	0.147	0.215	0.304
Democratic Republic of the Congo	Madimba	2000-2017	-0.038	-0.028	-0.018
Democratic Republic of the Congo	Mahagi	2000	0.292	0.434	0.616
Democratic Republic of the Congo	Mahagi	2017	0.167	0.243	0.339
Democratic Republic of the Congo	Mahagi	2000-2017	-0.048	-0.033	-0.020
Democratic Republic of the Congo	Mahagi (ville)	2000	0.282	0.443	0.628
Democratic Republic of the Congo	Mahagi (ville)	2017	0.163	0.251	0.367
Democratic Republic of the Congo	Mahagi (ville)	2000-2017	-0.061	-0.036	-0.008
Democratic Republic of the Congo	Makanza	2000	0.365	0.598	0.922
Democratic Republic of the Congo	Makanza	2017	0.172	0.254	0.361
Democratic Republic of the Congo	Makanza	2000-2017	-0.065	-0.047	-0.026
Democratic Republic of the Congo	Malemba-Nkulu	2000	0.372	0.606	0.916
Democratic Republic of the Congo	Malemba-Nkulu	2017	0.178	0.257	0.355
Democratic Republic of the Congo	Malemba-Nkulu	2000-2017	-0.068	-0.049	-0.034
Democratic Republic of the Congo	Mambasa	2000	0.255	0.371	0.514
Democratic Republic of the Congo	Mambasa	2017	0.161	0.236	0.334
Democratic Republic of the Congo	Mambasa	2000-2017	-0.035	-0.024	-0.014
Democratic Republic of the Congo	Mangai	2000	0.244	0.400	0.595
Democratic Republic of the Congo	Mangai	2017	0.123	0.218	0.345
Democratic Republic of the Congo	Mangai	2000-2017	-0.074	-0.035	0.005
Democratic Republic of the Congo	Manono	2000	0.372	0.591	0.878
Democratic Republic of the Congo	Manono	2017	0.173	0.254	0.353
Democratic Republic of the Congo	Manono	2000-2017	-0.061	-0.045	-0.030

Table 1: LRI DALYs rate by unit *(continued)*

Country	Unit	year	mean rate	lower rate	upper rate
Democratic Republic of the Congo	Manono (ville)	2000	0.366	0.609	0.972
Democratic Republic of the Congo	Manono (ville)	2017	0.156	0.256	0.366
Democratic Republic of the Congo	Manono (ville)	2000-2017	-0.076	-0.049	-0.023
Democratic Republic of the Congo	Masi-Manimba	2000	0.271	0.408	0.572
Democratic Republic of the Congo	Masi-Manimba	2017	0.162	0.233	0.336
Democratic Republic of the Congo	Masi-Manimba	2000-2017	-0.052	-0.035	-0.018
Democratic Republic of the Congo	Masi-Manimba (ville)	2000	0.218	0.392	0.686
Democratic Republic of the Congo	Masi-Manimba (ville)	2017	0.128	0.225	0.356
Democratic Republic of the Congo	Masi-Manimba (ville)	2000-2017	-0.091	-0.032	0.020
Democratic Republic of the Congo	Masisi	2000	0.330	0.514	0.744
Democratic Republic of the Congo	Masisi	2017	0.170	0.256	0.364
Democratic Republic of the Congo	Masisi	2000-2017	-0.049	-0.036	-0.023
Democratic Republic of the Congo	Matadi	2000	0.144	0.205	0.302
Democratic Republic of the Congo	Matadi	2017	0.087	0.148	0.241
Democratic Republic of the Congo	Matadi	2000-2017	-0.059	-0.029	0.002
Democratic Republic of the Congo	Mbandaka	2000	0.319	0.494	0.728
Democratic Republic of the Congo	Mbandaka	2017	0.151	0.228	0.332
Democratic Republic of the Congo	Mbandaka	2000-2017	-0.065	-0.045	-0.025
Democratic Republic of the Congo	Mbanza-Ngungu	2000	0.211	0.309	0.429
Democratic Republic of the Congo	Mbanza-Ngungu	2017	0.145	0.209	0.297
Democratic Republic of the Congo	Mbanza-Ngungu	2000-2017	-0.038	-0.027	-0.015
Democratic Republic of the Congo	Mbanza-Ngungu (ville)	2000	0.166	0.260	0.397
Democratic Republic of the Congo	Mbanza-Ngungu (ville)	2017	0.116	0.179	0.271
Democratic Republic of the Congo	Mbanza-Ngungu (ville)	2000-2017	-0.052	-0.023	0.007
Democratic Republic of the Congo	Mbuji-Mayi	2000	0.243	0.360	0.536
Democratic Republic of the Congo	Mbuji-Mayi	2017	0.110	0.168	0.242
Democratic Republic of the Congo	Mbuji-Mayi	2000-2017	-0.075	-0.044	-0.019
Democratic Republic of the Congo	Miabi	2000	0.375	0.604	0.929
Democratic Republic of the Congo	Miabi	2017	0.167	0.245	0.343
Democratic Republic of the Congo	Miabi	2000-2017	-0.070	-0.052	-0.034
Democratic Republic of the Congo	Miabi (ville)	2000	0.358	0.606	0.949
Democratic Republic of the Congo	Miabi (ville)	2017	0.144	0.243	0.361
Democratic Republic of the Congo	Miabi (ville)	2000-2017	-0.086	-0.052	-0.020
Democratic Republic of the Congo	Mitwaba	2000	0.356	0.570	0.835
Democratic Republic of the Congo	Mitwaba	2017	0.184	0.264	0.367
Democratic Republic of the Congo	Mitwaba	2000-2017	-0.059	-0.044	-0.030
Democratic Republic of the Congo	Moanda	2000	0.200	0.286	0.393
Democratic Republic of the Congo	Moanda	2017	0.135	0.200	0.278
Democratic Republic of the Congo	Moanda	2000-2017	-0.034	-0.023	-0.012
Democratic Republic of the Congo	Moanda (ville)	2000	0.159	0.242	0.356
Democratic Republic of the Congo	Moanda (ville)	2017	0.110	0.169	0.256
Democratic Republic of the Congo	Moanda (ville)	2000-2017	-0.050	-0.022	0.005
Democratic Republic of the Congo	Moba	2000	0.330	0.502	0.714
Democratic Republic of the Congo	Moba	2017	0.166	0.247	0.345
Democratic Republic of the Congo	Moba	2000-2017	-0.053	-0.038	-0.025
Democratic Republic of the Congo	Mobayi-Mbongo	2000	0.371	0.588	0.878
Democratic Republic of the Congo	Mobayi-Mbongo	2017	0.191	0.272	0.367
Democratic Republic of the Congo	Mobayi-Mbongo	2000-2017	-0.063	-0.046	-0.030
Democratic Republic of the Congo	Mobayi-Mbongo (ville)	2000	0.323	0.513	0.821
Democratic Republic of the Congo	Mobayi-Mbongo (ville)	2017	0.175	0.267	0.396
Democratic Republic of the Congo	Mobayi-Mbongo (ville)	2000-2017	-0.078	-0.039	0.000
Democratic Republic of the Congo	Mongwalu	2000	0.225	0.414	0.712
Democratic Republic of the Congo	Mongwalu	2017	0.132	0.245	0.393
Democratic Republic of the Congo	Mongwalu	2000-2017	-0.083	-0.032	0.019
Democratic Republic of the Congo	Monkoto	2000	0.317	0.501	0.725
Democratic Republic of the Congo	Monkoto	2017	0.171	0.246	0.339
Democratic Republic of the Congo	Monkoto	2000-2017	-0.055	-0.040	-0.023
Democratic Republic of the Congo	Mushie	2000	0.283	0.424	0.600
Democratic Republic of the Congo	Mushie	2017	0.157	0.230	0.319
Democratic Republic of the Congo	Mushie	2000-2017	-0.044	-0.033	-0.021
Democratic Republic of the Congo	Mutshatsha	2000	0.335	0.515	0.741

Table 1: LRI DALYs rate by unit *(continued)*

Country	Unit	year	mean rate	lower rate	upper rate
Democratic Republic of the Congo	Mutshatsha	2017	0.160	0.231	0.323
Democratic Republic of the Congo	Mutshatsha	2000-2017	-0.060	-0.046	-0.033
Democratic Republic of the Congo	Mweka	2000	0.349	0.552	0.832
Democratic Republic of the Congo	Mweka	2017	0.162	0.236	0.334
Democratic Republic of the Congo	Mweka	2000-2017	-0.068	-0.050	-0.034
Democratic Republic of the Congo	Mwene-Ditu	2000	0.299	0.485	0.731
Democratic Republic of the Congo	Mwene-Ditu	2017	0.158	0.238	0.349
Democratic Republic of the Congo	Mwene-Ditu	2000-2017	-0.069	-0.041	-0.016
Democratic Republic of the Congo	Mwenga	2000	0.394	0.662	1.031
Democratic Republic of the Congo	Mwenga	2017	0.203	0.289	0.398
Democratic Republic of the Congo	Mwenga	2000-2017	-0.053	-0.039	-0.025
Democratic Republic of the Congo	Namoya	2000	0.345	0.606	0.972
Democratic Republic of the Congo	Namoya	2017	0.155	0.244	0.380
Democratic Republic of the Congo	Namoya	2000-2017	-0.098	-0.055	-0.008
Democratic Republic of the Congo	Ngandajika	2000	0.370	0.592	0.892
Democratic Republic of the Congo	Ngandajika	2017	0.168	0.244	0.341
Democratic Republic of the Congo	Ngandajika	2000-2017	-0.072	-0.054	-0.038
Democratic Republic of the Congo	Ngandajika (ville)	2000	0.287	0.481	0.754
Democratic Republic of the Congo	Ngandajika (ville)	2017	0.152	0.238	0.360
Democratic Republic of the Congo	Ngandajika (ville)	2000-2017	-0.075	-0.040	-0.008
Democratic Republic of the Congo	Niangara	2000	0.270	0.399	0.554
Democratic Republic of the Congo	Niangara	2017	0.155	0.231	0.328
Democratic Republic of the Congo	Niangara	2000-2017	-0.045	-0.033	-0.019
Democratic Republic of the Congo	Nioki	2000	0.235	0.385	0.571
Democratic Republic of the Congo	Nioki	2017	0.128	0.202	0.306
Democratic Republic of the Congo	Nioki	2000-2017	-0.066	-0.034	-0.005
Democratic Republic of the Congo	Nyiragongo	2000	0.318	0.486	0.723
Democratic Republic of the Congo	Nyiragongo	2017	0.149	0.224	0.326
Democratic Republic of the Congo	Nyiragongo	2000-2017	-0.068	-0.046	-0.028
Democratic Republic of the Congo	Nyunzu	2000	0.350	0.548	0.802
Democratic Republic of the Congo	Nyunzu	2017	0.173	0.254	0.348
Democratic Republic of the Congo	Nyunzu	2000-2017	-0.060	-0.044	-0.030
Democratic Republic of the Congo	Oicha	2000	0.348	0.540	0.807
Democratic Republic of the Congo	Oicha	2017	0.167	0.243	0.343
Democratic Republic of the Congo	Oicha	2000-2017	-0.056	-0.042	-0.027
Democratic Republic of the Congo	Oïcha (ville)	2000	0.273	0.466	0.743
Democratic Republic of the Congo	Oïcha (ville)	2017	0.148	0.247	0.383
Democratic Republic of the Congo	Oïcha (ville)	2000-2017	-0.069	-0.035	-0.005
Democratic Republic of the Congo	Opala	2000	0.259	0.376	0.512
Democratic Republic of the Congo	Opala	2017	0.160	0.231	0.328
Democratic Republic of the Congo	Opala	2000-2017	-0.039	-0.028	-0.016
Democratic Republic of the Congo	Oshwe	2000	0.286	0.432	0.609
Democratic Republic of the Congo	Oshwe	2017	0.160	0.231	0.327
Democratic Republic of the Congo	Oshwe	2000-2017	-0.051	-0.036	-0.022
Democratic Republic of the Congo	Pangi	2000	0.364	0.596	0.904
Democratic Republic of the Congo	Pangi	2017	0.177	0.251	0.342
Democratic Republic of the Congo	Pangi	2000-2017	-0.061	-0.046	-0.033
Democratic Republic of the Congo	Poko	2000	0.271	0.393	0.552
Democratic Republic of the Congo	Poko	2017	0.158	0.231	0.326
Democratic Republic of the Congo	Poko	2000-2017	-0.041	-0.030	-0.017
Democratic Republic of the Congo	Popokabaka	2000	0.265	0.392	0.548
Democratic Republic of the Congo	Popokabaka	2017	0.159	0.231	0.327
Democratic Republic of the Congo	Popokabaka	2000-2017	-0.046	-0.033	-0.019
Democratic Republic of the Congo	Punia	2000	0.357	0.561	0.833
Democratic Republic of the Congo	Punia	2017	0.176	0.255	0.346
Democratic Republic of the Congo	Punia	2000-2017	-0.056	-0.042	-0.028
Democratic Republic of the Congo	Punia (ville)	2000	0.249	0.419	0.654
Democratic Republic of the Congo	Punia (ville)	2017	0.146	0.235	0.382
Democratic Republic of the Congo	Punia (ville)	2000-2017	-0.068	-0.035	0.003
Democratic Republic of the Congo	Pweto	2000	0.329	0.505	0.733
Democratic Republic of the Congo	Pweto	2017	0.176	0.258	0.360

Table 1: LRI DALYs rate by unit *(continued)*

Country	Unit	year	mean rate	lower rate	upper rate
Democratic Republic of the Congo	Pweto	2000-2017	-0.056	-0.041	-0.027
Democratic Republic of the Congo	Rungu	2000	0.281	0.406	0.575
Democratic Republic of the Congo	Rungu	2017	0.160	0.230	0.322
Democratic Republic of the Congo	Rungu	2000-2017	-0.043	-0.031	-0.020
Democratic Republic of the Congo	Rutshuru	2000	0.353	0.559	0.829
Democratic Republic of the Congo	Rutshuru	2017	0.169	0.249	0.351
Democratic Republic of the Congo	Rutshuru	2000-2017	-0.059	-0.043	-0.030
Democratic Republic of the Congo	Rutshuru (ville)	2000	0.272	0.477	0.713
Democratic Republic of the Congo	Rutshuru (ville)	2017	0.143	0.236	0.372
Democratic Republic of the Congo	Rutshuru (ville)	2000-2017	-0.073	-0.037	-0.002
Democratic Republic of the Congo	Sakania	2000	0.324	0.508	0.737
Democratic Republic of the Congo	Sakania	2017	0.149	0.217	0.310
Democratic Republic of the Congo	Sakania	2000-2017	-0.062	-0.047	-0.031
Democratic Republic of the Congo	Sandoa	2000	0.375	0.604	0.894
Democratic Republic of the Congo	Sandoa	2017	0.181	0.263	0.364
Democratic Republic of the Congo	Sandoa	2000-2017	-0.061	-0.045	-0.030
Democratic Republic of the Congo	Seke-Banza	2000	0.202	0.283	0.386
Democratic Republic of the Congo	Seke-Banza	2017	0.141	0.205	0.291
Democratic Republic of the Congo	Seke-Banza	2000-2017	-0.036	-0.024	-0.012
Democratic Republic of the Congo	Shabunda	2000	0.403	0.671	1.045
Democratic Republic of the Congo	Shabunda	2017	0.204	0.292	0.399
Democratic Republic of the Congo	Shabunda	2000-2017	-0.057	-0.042	-0.030
Democratic Republic of the Congo	Shabunda (ville)	2000	0.322	0.573	0.975
Democratic Republic of the Congo	Shabunda (ville)	2017	0.170	0.285	0.462
Democratic Republic of the Congo	Shabunda (ville)	2000-2017	-0.073	-0.032	0.007
Democratic Republic of the Congo	Songololo	2000	0.203	0.291	0.398
Democratic Republic of the Congo	Songololo	2017	0.146	0.211	0.302
Democratic Republic of the Congo	Songololo	2000-2017	-0.038	-0.024	-0.012
Democratic Republic of the Congo	Tshela	2000	0.203	0.296	0.408
Democratic Republic of the Congo	Tshela	2017	0.144	0.213	0.299
Democratic Republic of the Congo	Tshela	2000-2017	-0.032	-0.022	-0.011
Democratic Republic of the Congo	Tshela (ville)	2000	0.192	0.300	0.444
Democratic Republic of the Congo	Tshela (ville)	2017	0.128	0.207	0.310
Democratic Republic of the Congo	Tshela (ville)	2000-2017	-0.050	-0.025	0.011
Democratic Republic of the Congo	Tshikapa	2000	0.311	0.496	0.765
Democratic Republic of the Congo	Tshikapa	2017	0.122	0.193	0.286
Democratic Republic of the Congo	Tshikapa	2000-2017	-0.088	-0.055	-0.022
Democratic Republic of the Congo	Tshilenge	2000	0.365	0.587	0.887
Democratic Republic of the Congo	Tshilenge	2017	0.163	0.235	0.336
Democratic Republic of the Congo	Tshilenge	2000-2017	-0.073	-0.052	-0.035
Democratic Republic of the Congo	Tshilenge (ville)	2000	0.346	0.585	0.931
Democratic Republic of the Congo	Tshilenge (ville)	2017	0.155	0.247	0.403
Democratic Republic of the Congo	Tshilenge (ville)	2000-2017	-0.088	-0.051	-0.012
Democratic Republic of the Congo	Tshimbulu	2000	0.309	0.498	0.775
Democratic Republic of the Congo	Tshimbulu	2017	0.149	0.248	0.399
Democratic Republic of the Congo	Tshimbulu	2000-2017	-0.088	-0.039	0.001
Democratic Republic of the Congo	Ubundu	2000	0.248	0.366	0.503
Democratic Republic of the Congo	Ubundu	2017	0.157	0.230	0.321
Democratic Republic of the Congo	Ubundu	2000-2017	-0.037	-0.028	-0.019
Democratic Republic of the Congo	Uvira	2000	0.374	0.609	0.945
Democratic Republic of the Congo	Uvira	2017	0.192	0.271	0.394
Democratic Republic of the Congo	Uvira	2000-2017	-0.059	-0.043	-0.028
Democratic Republic of the Congo	Uvira (ville)	2000	0.285	0.462	0.761
Democratic Republic of the Congo	Uvira (ville)	2017	0.150	0.240	0.399
Democratic Republic of the Congo	Uvira (ville)	2000-2017	-0.074	-0.038	0.002
Democratic Republic of the Congo	Walikale	2000	0.324	0.505	0.737
Democratic Republic of the Congo	Walikale	2017	0.169	0.246	0.347
Democratic Republic of the Congo	Walikale	2000-2017	-0.045	-0.033	-0.021
Democratic Republic of the Congo	Walungu	2000	0.384	0.634	0.982
Democratic Republic of the Congo	Walungu	2017	0.182	0.266	0.373
Democratic Republic of the Congo	Walungu	2000-2017	-0.062	-0.044	-0.028

Table 1: LRI DALYs rate by unit *(continued)*

Country	Unit	year	mean rate	lower rate	upper rate
Democratic Republic of the Congo	Wamba	2000	0.287	0.418	0.582
Democratic Republic of the Congo	Wamba	2017	0.158	0.230	0.327
Democratic Republic of the Congo	Wamba	2000-2017	-0.045	-0.033	-0.021
Democratic Republic of the Congo	Wamba (ville)	2000	0.272	0.419	0.633
Democratic Republic of the Congo	Wamba (ville)	2017	0.147	0.233	0.348
Democratic Republic of the Congo	Wamba (ville)	2000-2017	-0.056	-0.033	-0.007
Democratic Republic of the Congo	Watsa	2000	0.270	0.393	0.551
Democratic Republic of the Congo	Watsa	2017	0.161	0.229	0.318
Democratic Republic of the Congo	Watsa	2000-2017	-0.044	-0.033	-0.022
Democratic Republic of the Congo	Watsa (ville)	2000	0.252	0.390	0.563
Democratic Republic of the Congo	Watsa (ville)	2017	0.156	0.230	0.333
Democratic Republic of the Congo	Watsa (ville)	2000-2017	-0.061	-0.037	-0.012
Democratic Republic of the Congo	Yahuma	2000	0.263	0.395	0.553
Democratic Republic of the Congo	Yahuma	2017	0.163	0.239	0.342
Democratic Republic of the Congo	Yahuma	2000-2017	-0.046	-0.031	-0.016
Democratic Republic of the Congo	Yakoma	2000	0.374	0.608	0.929
Democratic Republic of the Congo	Yakoma	2017	0.179	0.257	0.357
Democratic Republic of the Congo	Yakoma	2000-2017	-0.072	-0.053	-0.037
Democratic Republic of the Congo	Yangambi	2000	0.245	0.418	0.614
Democratic Republic of the Congo	Yangambi	2017	0.148	0.233	0.343
Democratic Republic of the Congo	Yangambi	2000-2017	-0.072	-0.036	0.003
Democratic Republic of the Congo	Yumbi	2000	0.261	0.397	0.557
Democratic Republic of the Congo	Yumbi	2017	0.154	0.227	0.325
Democratic Republic of the Congo	Yumbi	2000-2017	-0.048	-0.032	-0.015
Democratic Republic of the Congo	Zongo	2000	0.334	0.487	0.691
Democratic Republic of the Congo	Zongo	2017	0.165	0.228	0.301
Democratic Republic of the Congo	Zongo	2000-2017	-0.068	-0.047	-0.025
Djibouti	Alaili Dadda	2000	0.279	0.566	0.972
Djibouti	Alaili Dadda	2017	0.065	0.135	0.252
Djibouti	Alaili Dadda	2000-2017	-0.123	-0.090	-0.057
Djibouti	Ali Sabieh	2000	0.268	0.539	0.947
Djibouti	Ali Sabieh	2017	0.054	0.124	0.241
Djibouti	Ali Sabieh	2000-2017	-0.130	-0.092	-0.055
Djibouti	As Eyla	2000	0.175	0.333	0.562
Djibouti	As Eyla	2017	0.051	0.118	0.222
Djibouti	As Eyla	2000-2017	-0.096	-0.064	-0.032
Djibouti	Balha	2000	0.198	0.394	0.663
Djibouti	Balha	2017	0.047	0.113	0.226
Djibouti	Balha	2000-2017	-0.107	-0.073	-0.041
Djibouti	Dikhil	2000	0.205	0.393	0.683
Djibouti	Dikhil	2017	0.050	0.118	0.240
Djibouti	Dikhil	2000-2017	-0.107	-0.072	-0.035
Djibouti	Djibouti	2000	0.161	0.354	0.640
Djibouti	Djibouti	2017	0.025	0.079	0.197
Djibouti	Djibouti	2000-2017	-0.138	-0.091	-0.041
Djibouti	Dorra	2000	0.199	0.395	0.676
Djibouti	Dorra	2017	0.046	0.106	0.211
Djibouti	Dorra	2000-2017	-0.112	-0.077	-0.043
Djibouti	Obock	2000	0.270	0.545	0.929
Djibouti	Obock	2017	0.055	0.129	0.252
Djibouti	Obock	2000-2017	-0.127	-0.090	-0.056
Djibouti	Randa	2000	0.211	0.426	0.730
Djibouti	Randa	2017	0.051	0.119	0.241
Djibouti	Randa	2000-2017	-0.109	-0.074	-0.039
Djibouti	Tadjourah	2000	0.226	0.472	0.811
Djibouti	Tadjourah	2017	0.056	0.133	0.258
Djibouti	Tadjourah	2000-2017	-0.114	-0.078	-0.044
Djibouti	Yoboki	2000	0.174	0.345	0.584
Djibouti	Yoboki	2017	0.047	0.111	0.211
Djibouti	Yoboki	2000-2017	-0.096	-0.066	-0.035
Equatorial Guinea	Aconibe	2000	0.166	0.300	0.501

Table 1: LRI DALYs rate by unit *(continued)*

Country	Unit	year	mean rate	lower rate	upper rate
Equatorial Guinea	Aconibe	2017	0.032	0.060	0.105
Equatorial Guinea	Aconibe	2000-2017	-0.116	-0.098	-0.080
Equatorial Guinea	Acurenam	2000	0.152	0.278	0.457
Equatorial Guinea	Acurenam	2017	0.032	0.060	0.106
Equatorial Guinea	Acurenam	2000-2017	-0.113	-0.092	-0.075
Equatorial Guinea	Añisok	2000	0.169	0.307	0.501
Equatorial Guinea	Añisok	2017	0.030	0.057	0.104
Equatorial Guinea	Añisok	2000-2017	-0.124	-0.104	-0.086
Equatorial Guinea	Ayene	2000	0.167	0.303	0.506
Equatorial Guinea	Ayene	2017	0.032	0.060	0.108
Equatorial Guinea	Ayene	2000-2017	-0.117	-0.100	-0.079
Equatorial Guinea	Baney	2000	0.145	0.266	0.447
Equatorial Guinea	Baney	2017	0.026	0.053	0.097
Equatorial Guinea	Baney	2000-2017	-0.118	-0.097	-0.077
Equatorial Guinea	Bata	2000	0.151	0.282	0.456
Equatorial Guinea	Bata	2017	0.028	0.056	0.098
Equatorial Guinea	Bata	2000-2017	-0.123	-0.100	-0.075
Equatorial Guinea	Bicurga	2000	0.160	0.295	0.477
Equatorial Guinea	Bicurga	2017	0.031	0.057	0.105
Equatorial Guinea	Bicurga	2000-2017	-0.119	-0.099	-0.080
Equatorial Guinea	Bidjabidjan	2000	0.172	0.313	0.523
Equatorial Guinea	Bidjabidjan	2017	0.032	0.060	0.107
Equatorial Guinea	Bidjabidjan	2000-2017	-0.122	-0.101	-0.080
Equatorial Guinea	Bitica	2000	0.153	0.281	0.462
Equatorial Guinea	Bitica	2017	0.030	0.058	0.105
Equatorial Guinea	Bitica	2000-2017	-0.117	-0.095	-0.074
Equatorial Guinea	Ciudad Nueva Oyala	2000	0.167	0.301	0.502
Equatorial Guinea	Ciudad Nueva Oyala	2017	0.033	0.059	0.107
Equatorial Guinea	Ciudad Nueva Oyala	2000-2017	-0.121	-0.100	-0.077
Equatorial Guinea	Cogo	2000	0.149	0.273	0.430
Equatorial Guinea	Cogo	2017	0.029	0.056	0.100
Equatorial Guinea	Cogo	2000-2017	-0.117	-0.097	-0.075
Equatorial Guinea	Corisco	2000	NA	NA	NA
Equatorial Guinea	Corisco	2017	NA	NA	NA
Equatorial Guinea	Corisco	2000-2017	NA	NA	NA
Equatorial Guinea	Ebebiyin	2000	0.171	0.307	0.510
Equatorial Guinea	Ebebiyin	2017	0.030	0.057	0.098
Equatorial Guinea	Ebebiyin	2000-2017	-0.122	-0.103	-0.083
Equatorial Guinea	Evinayong	2000	0.160	0.287	0.478
Equatorial Guinea	Evinayong	2017	0.030	0.059	0.103
Equatorial Guinea	Evinayong	2000-2017	-0.117	-0.097	-0.078
Equatorial Guinea	Luba	2000	0.156	0.273	0.462
Equatorial Guinea	Luba	2017	0.030	0.059	0.116
Equatorial Guinea	Luba	2000-2017	-0.111	-0.093	-0.074
Equatorial Guinea	Mabana	2000	NA	NA	NA
Equatorial Guinea	Mabana	2017	NA	NA	NA
Equatorial Guinea	Mabana	2000-2017	NA	NA	NA
Equatorial Guinea	Machinda	2000	0.154	0.285	0.459
Equatorial Guinea	Machinda	2017	0.030	0.058	0.105
Equatorial Guinea	Machinda	2000-2017	-0.120	-0.098	-0.077
Equatorial Guinea	Malabo	2000	0.146	0.259	0.437
Equatorial Guinea	Malabo	2017	0.026	0.056	0.106
Equatorial Guinea	Malabo	2000-2017	-0.119	-0.094	-0.066
Equatorial Guinea	Mbini	2000	0.151	0.279	0.449
Equatorial Guinea	Mbini	2017	0.030	0.057	0.101
Equatorial Guinea	Mbini	2000-2017	-0.116	-0.097	-0.076
Equatorial Guinea	Micomeseng	2000	0.159	0.292	0.486
Equatorial Guinea	Micomeseng	2017	0.028	0.054	0.096
Equatorial Guinea	Micomeseng	2000-2017	-0.122	-0.102	-0.084
Equatorial Guinea	Mongomeyen	2000	0.163	0.300	0.529
Equatorial Guinea	Mongomeyen	2017	0.032	0.060	0.103

Table 1: LRI DALYs rate by unit *(continued)*

Country	Unit	year	mean rate	lower rate	upper rate
Equatorial Guinea	Mongomeyen	2000-2017	-0.123	-0.098	-0.076
Equatorial Guinea	Mongomo	2000	0.167	0.304	0.503
Equatorial Guinea	Mongomo	2017	0.030	0.056	0.098
Equatorial Guinea	Mongomo	2000-2017	-0.124	-0.104	-0.083
Equatorial Guinea	Nasng	2000	0.170	0.310	0.525
Equatorial Guinea	Nasng	2017	0.031	0.059	0.104
Equatorial Guinea	Nasng	2000-2017	-0.125	-0.103	-0.082
Equatorial Guinea	Niefang	2000	0.161	0.289	0.467
Equatorial Guinea	Niefang	2017	0.029	0.057	0.105
Equatorial Guinea	Niefang	2000-2017	-0.120	-0.099	-0.081
Equatorial Guinea	Nkimi	2000	0.155	0.288	0.465
Equatorial Guinea	Nkimi	2017	0.030	0.057	0.102
Equatorial Guinea	Nkimi	2000-2017	-0.118	-0.099	-0.079
Equatorial Guinea	Nkue	2000	0.161	0.304	0.510
Equatorial Guinea	Nkue	2017	0.030	0.057	0.102
Equatorial Guinea	Nkue	2000-2017	-0.125	-0.103	-0.084
Equatorial Guinea	Nsok-Nsomo	2000	0.169	0.308	0.521
Equatorial Guinea	Nsok-Nsomo	2017	0.031	0.059	0.107
Equatorial Guinea	Nsok-Nsomo	2000-2017	-0.125	-0.103	-0.081
Equatorial Guinea	Nsork	2000	0.161	0.301	0.492
Equatorial Guinea	Nsork	2017	0.032	0.059	0.103
Equatorial Guinea	Nsork	2000-2017	-0.117	-0.098	-0.079
Equatorial Guinea	Rebola	2000	0.140	0.257	0.447
Equatorial Guinea	Rebola	2017	0.025	0.055	0.106
Equatorial Guinea	Rebola	2000-2017	-0.117	-0.094	-0.070
Equatorial Guinea	Riaba	2000	0.151	0.273	0.468
Equatorial Guinea	Riaba	2017	0.028	0.058	0.112
Equatorial Guinea	Riaba	2000-2017	-0.115	-0.094	-0.072
Equatorial Guinea	Rio Campo	2000	0.146	0.270	0.444
Equatorial Guinea	Rio Campo	2017	0.029	0.055	0.097
Equatorial Guinea	Rio Campo	2000-2017	-0.121	-0.097	-0.073
Equatorial Guinea	San Antonio de Palé	2000	NA	NA	NA
Equatorial Guinea	San Antonio de Palé	2017	NA	NA	NA
Equatorial Guinea	San Antonio de Palé	2000-2017	NA	NA	NA
Eritrea	Adi Keyih	2000	0.194	0.414	0.806
Eritrea	Adi Keyih	2017	0.082	0.186	0.354
Eritrea	Adi Keyih	2000-2017	-0.084	-0.042	-0.005
Eritrea	Adi Kwala	2000	0.208	0.435	0.840
Eritrea	Adi Kwala	2017	0.089	0.199	0.379
Eritrea	Adi Kwala	2000-2017	-0.079	-0.041	-0.008
Eritrea	Adi Teklezan	2000	0.208	0.424	0.806
Eritrea	Adi Teklezan	2017	0.089	0.192	0.351
Eritrea	Adi Teklezan	2000-2017	-0.083	-0.042	-0.003
Eritrea	Afabet	2000	0.183	0.374	0.729
Eritrea	Afabet	2017	0.083	0.187	0.355
Eritrea	Afabet	2000-2017	-0.075	-0.039	-0.003
Eritrea	Akordat	2000	0.201	0.430	0.800
Eritrea	Akordat	2017	0.095	0.205	0.382
Eritrea	Akordat	2000-2017	-0.083	-0.040	0.002
Eritrea	Areta'	2000	0.203	0.412	0.764
Eritrea	Areta'	2017	0.082	0.167	0.307
Eritrea	Areta'	2000-2017	-0.079	-0.049	-0.019
Eritrea	Areza	2000	0.212	0.432	0.830
Eritrea	Areza	2017	0.089	0.200	0.376
Eritrea	Areza	2000-2017	-0.080	-0.041	-0.004
Eritrea	Asmara City	2000	0.210	0.434	0.831
Eritrea	Asmara City	2017	0.065	0.149	0.290
Eritrea	Asmara City	2000-2017	-0.112	-0.068	-0.027
Eritrea	Asmat	2000	0.196	0.409	0.788
Eritrea	Asmat	2017	0.085	0.191	0.371
Eritrea	Asmat	2000-2017	-0.083	-0.042	-0.006

Table 1: LRI DALYs rate by unit *(continued)*

Country	Unit	year	mean rate	lower rate	upper rate
Eritrea	Barentu	2000	0.215	0.459	0.834
Eritrea	Barentu	2017	0.094	0.207	0.381
Eritrea	Barentu	2000-2017	-0.080	-0.042	-0.003
Eritrea	Berikh	2000	0.212	0.429	0.846
Eritrea	Berikh	2017	0.072	0.161	0.296
Eritrea	Berikh	2000-2017	-0.092	-0.056	-0.018
Eritrea	Central So. Red-Sea	2000	0.189	0.384	0.727
Eritrea	Central So. Red-Sea	2017	0.074	0.165	0.312
Eritrea	Central So. Red-Sea	2000-2017	-0.085	-0.046	-0.009
Eritrea	Dahlak	2000	0.304	0.623	1.139
Eritrea	Dahlak	2017	0.115	0.259	0.492
Eritrea	Dahlak	2000-2017	-0.082	-0.046	-0.014
Eritrea	Dekemehare	2000	0.204	0.428	0.828
Eritrea	Dekemehare	2017	0.083	0.192	0.360
Eritrea	Dekemehare	2000-2017	-0.080	-0.042	-0.008
Eritrea	Dghe	2000	0.202	0.414	0.767
Eritrea	Dghe	2017	0.089	0.201	0.383
Eritrea	Dghe	2000-2017	-0.078	-0.041	-0.005
Eritrea	Dibarwa	2000	0.203	0.427	0.807
Eritrea	Dibarwa	2017	0.087	0.198	0.377
Eritrea	Dibarwa	2000-2017	-0.079	-0.041	-0.002
Eritrea	Elabered	2000	0.205	0.435	0.839
Eritrea	Elabered	2017	0.091	0.199	0.384
Eritrea	Elabered	2000-2017	-0.079	-0.042	-0.008
Eritrea	Foro	2000	0.216	0.445	0.861
Eritrea	Foro	2017	0.087	0.195	0.362
Eritrea	Foro	2000-2017	-0.083	-0.044	-0.007
Eritrea	Forto	2000	0.223	0.438	0.831
Eritrea	Forto	2017	0.086	0.197	0.373
Eritrea	Forto	2000-2017	-0.090	-0.046	-0.003
Eritrea	Ghala Nefhi	2000	0.200	0.423	0.808
Eritrea	Ghala Nefhi	2017	0.085	0.188	0.355
Eritrea	Ghala Nefhi	2000-2017	-0.077	-0.043	-0.008
Eritrea	Ghelaelo’	2000	0.221	0.432	0.805
Eritrea	Ghelaelo’	2017	0.083	0.179	0.329
Eritrea	Ghelaelo’	2000-2017	-0.076	-0.045	-0.018
Eritrea	Gheleb	2000	0.213	0.433	0.814
Eritrea	Gheleb	2017	0.085	0.195	0.361
Eritrea	Gheleb	2000-2017	-0.081	-0.043	-0.006
Eritrea	Ghida‘e	2000	0.207	0.437	0.845
Eritrea	Ghida‘e	2017	0.088	0.196	0.375
Eritrea	Ghida‘e	2000-2017	-0.082	-0.043	-0.001
Eritrea	Gogne	2000	0.218	0.448	0.849
Eritrea	Gogne	2017	0.090	0.203	0.383
Eritrea	Gogne	2000-2017	-0.083	-0.044	-0.005
Eritrea	Habero	2000	0.187	0.376	0.706
Eritrea	Habero	2017	0.085	0.183	0.343
Eritrea	Habero	2000-2017	-0.078	-0.040	-0.002
Eritrea	Hagaz	2000	0.201	0.427	0.821
Eritrea	Hagaz	2017	0.092	0.202	0.371
Eritrea	Hagaz	2000-2017	-0.078	-0.041	-0.006
Eritrea	Halhal	2000	0.215	0.434	0.813
Eritrea	Halhal	2017	0.093	0.199	0.373
Eritrea	Halhal	2000-2017	-0.080	-0.043	-0.007
Eritrea	Haykota	2000	0.219	0.454	0.838
Eritrea	Haykota	2017	0.084	0.197	0.381
Eritrea	Haykota	2000-2017	-0.091	-0.048	-0.008
Eritrea	Karora	2000	0.188	0.378	0.728
Eritrea	Karora	2017	0.081	0.185	0.354
Eritrea	Karora	2000-2017	-0.078	-0.040	-0.004
Eritrea	Keren	2000	0.214	0.442	0.860

Table 1: LRI DALYs rate by unit *(continued)*

Country	Unit	year	mean rate	lower rate	upper rate
Eritrea	Keren	2017	0.094	0.203	0.368
Eritrea	Keren	2000-2017	-0.086	-0.042	0.001
Eritrea	Kerke Bet	2000	0.170	0.354	0.688
Eritrea	Kerke Bet	2017	0.080	0.182	0.340
Eritrea	Kerke Bet	2000-2017	-0.076	-0.037	0.000
Eritrea	Kudo Bu‘er	2000	0.210	0.427	0.797
Eritrea	Kudo Bu‘er	2017	0.090	0.201	0.386
Eritrea	Kudo Bu‘er	2000-2017	-0.078	-0.040	-0.004
Eritrea	La‘Elay Gash	2000	0.235	0.482	0.909
Eritrea	La‘Elay Gash	2017	0.093	0.203	0.381
Eritrea	La‘Elay Gash	2000-2017	-0.087	-0.046	-0.011
Eritrea	Logo Anseba	2000	0.201	0.431	0.830
Eritrea	Logo Anseba	2017	0.089	0.197	0.370
Eritrea	Logo Anseba	2000-2017	-0.077	-0.042	-0.007
Eritrea	Mansura	2000	0.199	0.419	0.806
Eritrea	Mansura	2017	0.094	0.201	0.376
Eritrea	Mansura	2000-2017	-0.077	-0.040	-0.005
Eritrea	May Mine	2000	0.268	0.499	0.911
Eritrea	May Mine	2017	0.093	0.195	0.368
Eritrea	May Mine	2000-2017	-0.085	-0.050	-0.019
Eritrea	Mendefera	2000	0.204	0.425	0.762
Eritrea	Mendefera	2017	0.084	0.196	0.365
Eritrea	Mendefera	2000-2017	-0.081	-0.042	-0.005
Eritrea	Mitswa‘e City	2000	0.239	0.489	0.921
Eritrea	Mitswa‘e City	2017	0.081	0.206	0.392
Eritrea	Mitswa‘e City	2000-2017	-0.090	-0.049	-0.008
Eritrea	Mogolo	2000	0.212	0.440	0.825
Eritrea	Mogolo	2017	0.094	0.204	0.385
Eritrea	Mogolo	2000-2017	-0.081	-0.042	-0.006
Eritrea	Nakfa	2000	0.196	0.402	0.775
Eritrea	Nakfa	2017	0.077	0.184	0.358
Eritrea	Nakfa	2000-2017	-0.083	-0.043	-0.006
Eritrea	Omhajer	2000	0.234	0.461	0.839
Eritrea	Omhajer	2017	0.083	0.194	0.359
Eritrea	Omhajer	2000-2017	-0.086	-0.045	-0.005
Eritrea	Segeneyiti	2000	0.201	0.421	0.799
Eritrea	Segeneyiti	2017	0.088	0.197	0.369
Eritrea	Segeneyiti	2000-2017	-0.081	-0.041	-0.004
Eritrea	Sel‘a	2000	0.171	0.348	0.670
Eritrea	Sel‘a	2017	0.079	0.180	0.346
Eritrea	Sel‘a	2000-2017	-0.078	-0.037	0.003
Eritrea	Senafe	2000	0.212	0.421	0.787
Eritrea	Senafe	2017	0.083	0.185	0.345
Eritrea	Senafe	2000-2017	-0.085	-0.043	-0.008
Eritrea	Serejeka	2000	0.203	0.424	0.816
Eritrea	Serejeka	2017	0.082	0.185	0.355
Eritrea	Serejeka	2000-2017	-0.082	-0.044	-0.003
Eritrea	Sheib	2000	0.196	0.406	0.782
Eritrea	Sheib	2017	0.089	0.199	0.375
Eritrea	Sheib	2000-2017	-0.075	-0.039	-0.004
Eritrea	Shemboko	2000	0.231	0.474	0.898
Eritrea	Shemboko	2017	0.094	0.201	0.381
Eritrea	Shemboko	2000-2017	-0.082	-0.046	-0.010
Eritrea	So. Southern Red-Sea	2000	0.221	0.447	0.821
Eritrea	So. Southern Red-Sea	2017	0.083	0.182	0.345
Eritrea	So. Southern Red-Sea	2000-2017	-0.084	-0.047	-0.004
Eritrea	Teseneye	2000	0.232	0.479	0.897
Eritrea	Teseneye	2017	0.077	0.197	0.379
Eritrea	Teseneye	2000-2017	-0.093	-0.052	-0.010
Eritrea	Tsorena	2000	0.315	0.529	0.888
Eritrea	Tsorena	2017	0.086	0.186	0.348

Table 1: LRI DALYs rate by unit *(continued)*

Country	Unit	year	mean rate	lower rate	upper rate
Eritrea	Tsorena	2000-2017	-0.086	-0.055	-0.026
Ethiopia	Addis Abeba	2000	0.136	0.218	0.310
Ethiopia	Addis Abeba	2017	0.064	0.106	0.167
Ethiopia	Addis Abeba	2000-2017	-0.068	-0.042	-0.015
Ethiopia	Afar Zone 1	2000	0.371	0.567	0.796
Ethiopia	Afar Zone 1	2017	0.128	0.193	0.275
Ethiopia	Afar Zone 1	2000-2017	-0.082	-0.062	-0.043
Ethiopia	Afar Zone 2	2000	0.411	0.628	0.887
Ethiopia	Afar Zone 2	2017	0.153	0.224	0.312
Ethiopia	Afar Zone 2	2000-2017	-0.076	-0.059	-0.043
Ethiopia	Afar Zone 3	2000	0.381	0.557	0.760
Ethiopia	Afar Zone 3	2017	0.155	0.232	0.327
Ethiopia	Afar Zone 3	2000-2017	-0.062	-0.050	-0.038
Ethiopia	Afar Zone 4	2000	0.394	0.639	0.921
Ethiopia	Afar Zone 4	2017	0.138	0.211	0.311
Ethiopia	Afar Zone 4	2000-2017	-0.085	-0.062	-0.039
Ethiopia	Afar Zone 5	2000	0.430	0.662	0.924
Ethiopia	Afar Zone 5	2017	0.141	0.210	0.292
Ethiopia	Afar Zone 5	2000-2017	-0.084	-0.065	-0.047
Ethiopia	Afder	2000	0.322	0.504	0.711
Ethiopia	Afder	2017	0.111	0.161	0.220
Ethiopia	Afder	2000-2017	-0.086	-0.068	-0.051
Ethiopia	Agew Awi	2000	0.259	0.377	0.524
Ethiopia	Agew Awi	2017	0.097	0.145	0.201
Ethiopia	Agew Awi	2000-2017	-0.071	-0.057	-0.042
Ethiopia	Agnuak	2000	0.314	0.501	0.713
Ethiopia	Agnuak	2017	0.063	0.097	0.138
Ethiopia	Agnuak	2000-2017	-0.123	-0.103	-0.082
Ethiopia	Alaba	2000	0.306	0.468	0.645
Ethiopia	Alaba	2017	0.097	0.149	0.209
Ethiopia	Alaba	2000-2017	-0.086	-0.067	-0.048
Ethiopia	Alle	2000	0.303	0.473	0.661
Ethiopia	Alle	2017	0.096	0.144	0.208
Ethiopia	Alle	2000-2017	-0.090	-0.071	-0.050
Ethiopia	Amaro	2000	0.332	0.484	0.673
Ethiopia	Amaro	2017	0.097	0.145	0.205
Ethiopia	Amaro	2000-2017	-0.093	-0.070	-0.052
Ethiopia	Argoba	2000	0.254	0.396	0.564
Ethiopia	Argoba	2017	0.080	0.127	0.192
Ethiopia	Argoba	2000-2017	-0.092	-0.064	-0.038
Ethiopia	Arsi	2000	0.273	0.398	0.536
Ethiopia	Arsi	2017	0.079	0.119	0.162
Ethiopia	Arsi	2000-2017	-0.085	-0.072	-0.060
Ethiopia	Asosa	2000	0.435	0.673	0.945
Ethiopia	Asosa	2017	0.118	0.167	0.229
Ethiopia	Asosa	2000-2017	-0.099	-0.083	-0.068
Ethiopia	Bahir Dar Special Zone	2000	0.198	0.346	0.543
Ethiopia	Bahir Dar Special Zone	2017	0.081	0.136	0.212
Ethiopia	Bahir Dar Special Zone	2000-2017	-0.090	-0.053	-0.016
Ethiopia	Bale	2000	0.249	0.359	0.482
Ethiopia	Bale	2017	0.083	0.124	0.171
Ethiopia	Bale	2000-2017	-0.076	-0.063	-0.048
Ethiopia	Basketo	2000	0.321	0.495	0.688
Ethiopia	Basketo	2017	0.098	0.143	0.198
Ethiopia	Basketo	2000-2017	-0.095	-0.075	-0.051
Ethiopia	Bench Maji	2000	0.290	0.432	0.592
Ethiopia	Bench Maji	2017	0.103	0.148	0.208
Ethiopia	Bench Maji	2000-2017	-0.080	-0.063	-0.045
Ethiopia	Borena	2000	0.330	0.512	0.709
Ethiopia	Borena	2017	0.081	0.121	0.168
Ethiopia	Borena	2000-2017	-0.103	-0.084	-0.067

Table 1: LRI DALYs rate by unit *(continued)*

Country	Unit	year	mean rate	lower rate	upper rate
Ethiopia	Burji	2000	0.329	0.495	0.707
Ethiopia	Burji	2017	0.100	0.148	0.206
Ethiopia	Burji	2000-2017	-0.090	-0.071	-0.049
Ethiopia	Dawro	2000	0.357	0.572	0.814
Ethiopia	Dawro	2017	0.101	0.150	0.208
Ethiopia	Dawro	2000-2017	-0.095	-0.076	-0.057
Ethiopia	Debub Gondar	2000	0.241	0.353	0.503
Ethiopia	Debub Gondar	2017	0.080	0.125	0.179
Ethiopia	Debub Gondar	2000-2017	-0.079	-0.063	-0.047
Ethiopia	Debub Mirab Shewa	2000	0.272	0.401	0.543
Ethiopia	Debub Mirab Shewa	2017	0.078	0.115	0.159
Ethiopia	Debub Mirab Shewa	2000-2017	-0.090	-0.075	-0.059
Ethiopia	Debub Omo	2000	0.306	0.459	0.636
Ethiopia	Debub Omo	2017	0.096	0.145	0.201
Ethiopia	Debub Omo	2000-2017	-0.086	-0.069	-0.050
Ethiopia	Debub Wollo	2000	0.236	0.344	0.490
Ethiopia	Debub Wollo	2017	0.083	0.125	0.178
Ethiopia	Debub Wollo	2000-2017	-0.076	-0.059	-0.038
Ethiopia	Debubawi	2000	0.206	0.308	0.441
Ethiopia	Debubawi	2017	0.071	0.107	0.146
Ethiopia	Debubawi	2000-2017	-0.075	-0.060	-0.044
Ethiopia	Derashe	2000	0.311	0.471	0.672
Ethiopia	Derashe	2017	0.096	0.145	0.209
Ethiopia	Derashe	2000-2017	-0.090	-0.070	-0.050
Ethiopia	Dire Dawa	2000	0.303	0.454	0.637
Ethiopia	Dire Dawa	2017	0.078	0.118	0.172
Ethiopia	Dire Dawa	2000-2017	-0.096	-0.077	-0.056
Ethiopia	Doolo	2000	0.344	0.539	0.745
Ethiopia	Doolo	2017	0.123	0.174	0.235
Ethiopia	Doolo	2000-2017	-0.078	-0.065	-0.052
Ethiopia	Fafan	2000	0.374	0.543	0.767
Ethiopia	Fafan	2017	0.133	0.192	0.261
Ethiopia	Fafan	2000-2017	-0.077	-0.062	-0.047
Ethiopia	Gamo Gofa	2000	0.319	0.467	0.648
Ethiopia	Gamo Gofa	2017	0.095	0.144	0.199
Ethiopia	Gamo Gofa	2000-2017	-0.087	-0.069	-0.051
Ethiopia	Gedeo	2000	0.310	0.459	0.629
Ethiopia	Gedeo	2017	0.098	0.147	0.208
Ethiopia	Gedeo	2000-2017	-0.088	-0.069	-0.051
Ethiopia	Guji	2000	0.310	0.460	0.637
Ethiopia	Guji	2017	0.080	0.121	0.168
Ethiopia	Guji	2000-2017	-0.100	-0.081	-0.063
Ethiopia	Gurage	2000	0.329	0.498	0.702
Ethiopia	Gurage	2017	0.098	0.145	0.202
Ethiopia	Gurage	2000-2017	-0.088	-0.072	-0.057
Ethiopia	Hadiya	2000	0.334	0.502	0.697
Ethiopia	Hadiya	2017	0.101	0.149	0.206
Ethiopia	Hadiya	2000-2017	-0.090	-0.073	-0.056
Ethiopia	Hareri	2000	0.250	0.383	0.537
Ethiopia	Hareri	2017	0.066	0.099	0.139
Ethiopia	Hareri	2000-2017	-0.099	-0.078	-0.056
Ethiopia	Horo Guduru	2000	0.267	0.396	0.535
Ethiopia	Horo Guduru	2017	0.077	0.116	0.164
Ethiopia	Horo Guduru	2000-2017	-0.089	-0.073	-0.057
Ethiopia	Ilubabor	2000	0.295	0.456	0.636
Ethiopia	Ilubabor	2017	0.083	0.121	0.167
Ethiopia	Ilubabor	2000-2017	-0.094	-0.077	-0.060
Ethiopia	Jarar	2000	0.282	0.416	0.596
Ethiopia	Jarar	2017	0.104	0.156	0.221
Ethiopia	Jarar	2000-2017	-0.070	-0.060	-0.049
Ethiopia	Jimma	2000	0.352	0.567	0.815

Table 1: LRI DALYs rate by unit *(continued)*

Country	Unit	year	mean rate	lower rate	upper rate
Ethiopia	Jimma	2017	0.084	0.124	0.171
Ethiopia	Jimma	2000-2017	-0.107	-0.089	-0.071
Ethiopia	Keffa	2000	0.327	0.508	0.711
Ethiopia	Keffa	2017	0.101	0.149	0.206
Ethiopia	Keffa	2000-2017	-0.091	-0.074	-0.057
Ethiopia	Kelem Wellega	2000	0.303	0.484	0.675
Ethiopia	Kelem Wellega	2017	0.083	0.122	0.170
Ethiopia	Kelem Wellega	2000-2017	-0.095	-0.077	-0.061
Ethiopia	Kemashi	2000	0.433	0.685	0.951
Ethiopia	Kemashi	2017	0.108	0.160	0.220
Ethiopia	Kemashi	2000-2017	-0.102	-0.085	-0.069
Ethiopia	Kembata Tembaro	2000	0.325	0.507	0.710
Ethiopia	Kembata Tembaro	2017	0.101	0.150	0.210
Ethiopia	Kembata Tembaro	2000-2017	-0.089	-0.072	-0.054
Ethiopia	Konso	2000	0.317	0.483	0.668
Ethiopia	Konso	2017	0.097	0.146	0.206
Ethiopia	Konso	2000-2017	-0.091	-0.071	-0.050
Ethiopia	Konta	2000	0.344	0.540	0.753
Ethiopia	Konta	2017	0.101	0.149	0.209
Ethiopia	Konta	2000-2017	-0.097	-0.076	-0.058
Ethiopia	Korahe	2000	0.276	0.415	0.581
Ethiopia	Korahe	2017	0.104	0.155	0.221
Ethiopia	Korahe	2000-2017	-0.071	-0.059	-0.047
Ethiopia	Liben	2000	0.395	0.625	0.875
Ethiopia	Liben	2017	0.143	0.205	0.281
Ethiopia	Liben	2000-2017	-0.080	-0.066	-0.050
Ethiopia	Majang	2000	0.308	0.471	0.655
Ethiopia	Majang	2017	0.077	0.112	0.160
Ethiopia	Majang	2000-2017	-0.112	-0.090	-0.070
Ethiopia	Mehakelegnaw	2000	0.226	0.328	0.454
Ethiopia	Mehakelegnaw	2017	0.080	0.117	0.163
Ethiopia	Mehakelegnaw	2000-2017	-0.073	-0.060	-0.045
Ethiopia	Metekel	2000	0.414	0.622	0.850
Ethiopia	Metekel	2017	0.113	0.166	0.223
Ethiopia	Metekel	2000-2017	-0.093	-0.079	-0.065
Ethiopia	Mi'irabawi	2000	0.228	0.357	0.500
Ethiopia	Mi'irabawi	2017	0.085	0.122	0.168
Ethiopia	Mi'irabawi	2000-2017	-0.078	-0.063	-0.050
Ethiopia	Mirab Arsi	2000	0.309	0.467	0.642
Ethiopia	Mirab Arsi	2017	0.083	0.125	0.171
Ethiopia	Mirab Arsi	2000-2017	-0.093	-0.077	-0.062
Ethiopia	Mirab Gojjam	2000	0.260	0.374	0.533
Ethiopia	Mirab Gojjam	2017	0.093	0.139	0.196
Ethiopia	Mirab Gojjam	2000-2017	-0.071	-0.058	-0.044
Ethiopia	Mirab Hararghe	2000	0.255	0.362	0.487
Ethiopia	Mirab Hararghe	2017	0.082	0.120	0.166
Ethiopia	Mirab Hararghe	2000-2017	-0.078	-0.066	-0.054
Ethiopia	Mirab Shewa	2000	0.285	0.420	0.578
Ethiopia	Mirab Shewa	2017	0.078	0.117	0.162
Ethiopia	Mirab Shewa	2000-2017	-0.093	-0.077	-0.062
Ethiopia	Mirab Welega	2000	0.293	0.446	0.614
Ethiopia	Mirab Welega	2017	0.082	0.121	0.164
Ethiopia	Mirab Welega	2000-2017	-0.091	-0.075	-0.058
Ethiopia	Misraq Gojjam	2000	0.243	0.354	0.493
Ethiopia	Misraq Gojjam	2017	0.091	0.136	0.193
Ethiopia	Misraq Gojjam	2000-2017	-0.068	-0.056	-0.042
Ethiopia	Misraq Harerge	2000	0.256	0.365	0.494
Ethiopia	Misraq Harerge	2017	0.084	0.124	0.170
Ethiopia	Misraq Harerge	2000-2017	-0.078	-0.064	-0.049
Ethiopia	Misraq Shewa	2000	0.273	0.410	0.561
Ethiopia	Misraq Shewa	2017	0.076	0.114	0.160

Table 1: LRI DALYs rate by unit *(continued)*

Country	Unit	year	mean rate	lower rate	upper rate
Ethiopia	Misraq Shewa	2000-2017	-0.092	-0.076	-0.059
Ethiopia	Misraq Wellega	2000	0.277	0.417	0.574
Ethiopia	Misraq Wellega	2017	0.081	0.121	0.167
Ethiopia	Misraq Wellega	2000-2017	-0.087	-0.072	-0.058
Ethiopia	Misraqawi	2000	0.211	0.303	0.418
Ethiopia	Misraqawi	2017	0.078	0.117	0.164
Ethiopia	Misraqawi	2000-2017	-0.068	-0.055	-0.042
Ethiopia	Nogob	2000	0.269	0.398	0.566
Ethiopia	Nogob	2017	0.112	0.163	0.225
Ethiopia	Nogob	2000-2017	-0.067	-0.053	-0.038
Ethiopia	North Shewa	2000	0.286	0.422	0.568
Ethiopia	North Shewa	2000	0.235	0.337	0.456
Ethiopia	North Shewa	2017	0.075	0.113	0.157
Ethiopia	North Shewa	2017	0.085	0.126	0.176
Ethiopia	North Shewa	2000-2017	-0.073	-0.058	-0.043
Ethiopia	North Shewa	2000-2017	-0.093	-0.079	-0.063
Ethiopia	Nuer	2000	0.317	0.516	0.735
Ethiopia	Nuer	2017	0.075	0.108	0.146
Ethiopia	Nuer	2000-2017	-0.110	-0.091	-0.071
Ethiopia	Oromia	2000	0.257	0.378	0.526
Ethiopia	Oromia	2017	0.083	0.127	0.188
Ethiopia	Oromia	2000-2017	-0.083	-0.062	-0.040
Ethiopia	Semen Gondar	2000	0.272	0.401	0.552
Ethiopia	Semen Gondar	2017	0.091	0.137	0.195
Ethiopia	Semen Gondar	2000-2017	-0.078	-0.063	-0.049
Ethiopia	Semen Wello	2000	0.242	0.358	0.495
Ethiopia	Semen Wello	2017	0.079	0.122	0.175
Ethiopia	Semen Wello	2000-2017	-0.083	-0.064	-0.042
Ethiopia	Semien Mi'irabaw	2000	0.236	0.370	0.519
Ethiopia	Semien Mi'irabaw	2017	0.085	0.123	0.170
Ethiopia	Semien Mi'irabaw	2000-2017	-0.081	-0.065	-0.049
Ethiopia	Shabelle	2000	0.336	0.523	0.728
Ethiopia	Shabelle	2017	0.113	0.166	0.229
Ethiopia	Shabelle	2000-2017	-0.083	-0.067	-0.051
Ethiopia	Sheka	2000	0.315	0.473	0.652
Ethiopia	Sheka	2017	0.097	0.144	0.203
Ethiopia	Sheka	2000-2017	-0.089	-0.070	-0.050
Ethiopia	Sidama	2000	0.313	0.446	0.613
Ethiopia	Sidama	2017	0.097	0.147	0.203
Ethiopia	Sidama	2000-2017	-0.080	-0.066	-0.050
Ethiopia	Silti	2000	0.307	0.467	0.657
Ethiopia	Silti	2017	0.101	0.148	0.203
Ethiopia	Silti	2000-2017	-0.082	-0.066	-0.051
Ethiopia	Siti	2000	0.342	0.517	0.708
Ethiopia	Siti	2017	0.125	0.181	0.249
Ethiopia	Siti	2000-2017	-0.074	-0.061	-0.048
Ethiopia	Wag Himra	2000	0.256	0.373	0.526
Ethiopia	Wag Himra	2017	0.079	0.124	0.178
Ethiopia	Wag Himra	2000-2017	-0.079	-0.063	-0.049
Ethiopia	Wolayita	2000	0.338	0.517	0.722
Ethiopia	Wolayita	2017	0.101	0.150	0.210
Ethiopia	Wolayita	2000-2017	-0.087	-0.071	-0.054
Ethiopia	Yem	2000	0.358	0.587	0.838
Ethiopia	Yem	2017	0.104	0.153	0.211
Ethiopia	Yem	2000-2017	-0.096	-0.075	-0.056
Gabon	Abanga-Bigné	2000	0.062	0.098	0.152
Gabon	Abanga-Bigné	2017	0.043	0.071	0.118
Gabon	Abanga-Bigné	2000-2017	-0.031	-0.022	-0.013
Gabon	Basse Banio	2000	0.060	0.094	0.145
Gabon	Basse Banio	2017	0.036	0.060	0.094
Gabon	Basse Banio	2000-2017	-0.036	-0.028	-0.020

Table 1: LRI DALYs rate by unit *(continued)*

Country	Unit	year	mean rate	lower rate	upper rate
Gabon	Bendjé	2000	0.062	0.097	0.150
Gabon	Bendjé	2017	0.040	0.064	0.105
Gabon	Bendjé	2000-2017	-0.034	-0.026	-0.018
Gabon	Boumi-lowetsi	2000	0.068	0.108	0.166
Gabon	Boumi-lowetsi	2017	0.041	0.068	0.115
Gabon	Boumi-lowetsi	2000-2017	-0.035	-0.028	-0.019
Gabon	Dola	2000	0.063	0.100	0.153
Gabon	Dola	2017	0.039	0.064	0.104
Gabon	Dola	2000-2017	-0.037	-0.028	-0.019
Gabon	Douigny	2000	0.056	0.091	0.141
Gabon	Douigny	2017	0.037	0.062	0.100
Gabon	Douigny	2000-2017	-0.033	-0.025	-0.016
Gabon	Douya Onoye	2000	0.064	0.101	0.154
Gabon	Douya Onoye	2017	0.041	0.067	0.109
Gabon	Douya Onoye	2000-2017	-0.036	-0.026	-0.017
Gabon	Étimboué	2000	0.061	0.097	0.150
Gabon	Étimboué	2017	0.039	0.063	0.100
Gabon	Étimboué	2000-2017	-0.034	-0.027	-0.020
Gabon	Haut-Como	2000	0.062	0.098	0.150
Gabon	Haut-Como	2017	0.044	0.072	0.123
Gabon	Haut-Como	2000-2017	-0.031	-0.020	-0.008
Gabon	Haut-Ntem	2000	0.067	0.108	0.170
Gabon	Haut-Ntem	2017	0.043	0.071	0.119
Gabon	Haut-Ntem	2000-2017	-0.034	-0.027	-0.019
Gabon	Haute-Banio	2000	0.060	0.093	0.145
Gabon	Haute-Banio	2017	0.037	0.062	0.100
Gabon	Haute-Banio	2000-2017	-0.038	-0.025	-0.015
Gabon	Ivindo	2000	0.063	0.099	0.155
Gabon	Ivindo	2017	0.041	0.069	0.112
Gabon	Ivindo	2000-2017	-0.031	-0.023	-0.014
Gabon	Komo	2000	0.065	0.105	0.162
Gabon	Komo	2017	0.044	0.072	0.119
Gabon	Komo	2000-2017	-0.035	-0.025	-0.017
Gabon	Komo-Mondah	2000	0.064	0.104	0.165
Gabon	Komo-Mondah	2017	0.037	0.061	0.101
Gabon	Komo-Mondah	2000-2017	-0.046	-0.033	-0.021
Gabon	Léboumbi-Leyou	2000	0.064	0.103	0.160
Gabon	Léboumbi-Leyou	2017	0.040	0.066	0.106
Gabon	Léboumbi-Leyou	2000-2017	-0.038	-0.029	-0.020
Gabon	Léconi-Djoué	2000	0.064	0.102	0.158
Gabon	Léconi-Djoué	2017	0.041	0.067	0.110
Gabon	Léconi-Djoué	2000-2017	-0.034	-0.026	-0.018
Gabon	Lékoko	2000	0.064	0.102	0.158
Gabon	Lékoko	2017	0.041	0.068	0.112
Gabon	Lékoko	2000-2017	-0.033	-0.026	-0.017
Gabon	Lolo Bouenguidi	2000	0.064	0.102	0.158
Gabon	Lolo Bouenguidi	2017	0.043	0.070	0.116
Gabon	Lolo Bouenguidi	2000-2017	-0.033	-0.025	-0.016
Gabon	Lombo-Bouenguidi	2000	0.063	0.102	0.153
Gabon	Lombo-Bouenguidi	2017	0.043	0.070	0.114
Gabon	Lombo-Bouenguidi	2000-2017	-0.034	-0.024	-0.015
Gabon	Lopé	2000	0.060	0.095	0.147
Gabon	Lopé	2017	0.042	0.069	0.114
Gabon	Lopé	2000-2017	-0.030	-0.021	-0.013
Gabon	Louetsi-Wano	2000	0.069	0.109	0.176
Gabon	Louetsi-Wano	2017	0.042	0.071	0.118
Gabon	Louetsi-Wano	2000-2017	-0.042	-0.027	-0.014
Gabon	Mougoutsi	2000	0.060	0.094	0.144
Gabon	Mougoutsi	2017	0.037	0.062	0.100
Gabon	Mougoutsi	2000-2017	-0.033	-0.026	-0.018
Gabon	Mouloudnou	2000	0.063	0.101	0.159

Table 1: LRI DALYs rate by unit *(continued)*

Country	Unit	year	mean rate	lower rate	upper rate
Gabon	Mouloudnou	2017	0.043	0.071	0.118
Gabon	Mouloudnou	2000-2017	-0.031	-0.023	-0.015
Gabon	Mpassa	2000	0.064	0.105	0.161
Gabon	Mpassa	2017	0.041	0.067	0.108
Gabon	Mpassa	2000-2017	-0.036	-0.029	-0.022
Gabon	Mvoung	2000	0.060	0.095	0.148
Gabon	Mvoung	2017	0.042	0.069	0.116
Gabon	Mvoung	2000-2017	-0.031	-0.021	-0.013
Gabon	Ndolou	2000	0.061	0.098	0.152
Gabon	Ndolou	2017	0.039	0.065	0.102
Gabon	Ndolou	2000-2017	-0.034	-0.025	-0.016
Gabon	Ndougou	2000	0.061	0.097	0.150
Gabon	Ndougou	2017	0.038	0.063	0.099
Gabon	Ndougou	2000-2017	-0.036	-0.027	-0.020
Gabon	Noya	2000	0.067	0.101	0.152
Gabon	Noya	2017	0.046	0.071	0.117
Gabon	Noya	2000-2017	-0.033	-0.023	-0.014
Gabon	Ntem	2000	0.068	0.106	0.167
Gabon	Ntem	2017	0.044	0.071	0.119
Gabon	Ntem	2000-2017	-0.034	-0.026	-0.017
Gabon	Ogooué et des Lacs	2000	0.063	0.100	0.155
Gabon	Ogooué et des Lacs	2017	0.042	0.068	0.109
Gabon	Ogooué et des Lacs	2000-2017	-0.032	-0.025	-0.017
Gabon	Ogoulou	2000	0.062	0.099	0.151
Gabon	Ogoulou	2017	0.041	0.067	0.112
Gabon	Ogoulou	2000-2017	-0.033	-0.025	-0.017
Gabon	Okano	2000	0.065	0.101	0.157
Gabon	Okano	2017	0.044	0.071	0.116
Gabon	Okano	2000-2017	-0.031	-0.023	-0.014
Gabon	Plateaux	2000	0.066	0.107	0.164
Gabon	Plateaux	2017	0.040	0.067	0.108
Gabon	Plateaux	2000-2017	-0.036	-0.029	-0.021
Gabon	Sébé-Brikolo	2000	0.061	0.098	0.153
Gabon	Sébé-Brikolo	2017	0.041	0.068	0.112
Gabon	Sébé-Brikolo	2000-2017	-0.031	-0.023	-0.015
Gabon	Tsamba Mangotsi	2000	0.062	0.097	0.150
Gabon	Tsamba Mangotsi	2017	0.040	0.066	0.106
Gabon	Tsamba Mangotsi	2000-2017	-0.032	-0.025	-0.016
Gabon	Woleu	2000	0.067	0.107	0.168
Gabon	Woleu	2017	0.045	0.072	0.119
Gabon	Woleu	2000-2017	-0.033	-0.026	-0.017
Gabon	Zadié	2000	0.065	0.100	0.153
Gabon	Zadié	2017	0.041	0.068	0.113
Gabon	Zadié	2000-2017	-0.032	-0.024	-0.017
Gambia	Banjul	2000	0.097	0.182	0.298
Gambia	Banjul	2017	0.050	0.093	0.149
Gambia	Banjul	2000-2017	-0.089	-0.038	0.008
Gambia	Central Baddibu	2000	0.151	0.223	0.314
Gambia	Central Baddibu	2017	0.076	0.115	0.174
Gambia	Central Baddibu	2000-2017	-0.066	-0.039	-0.009
Gambia	Foni Bintang Karanai	2000	0.140	0.220	0.319
Gambia	Foni Bintang Karanai	2017	0.068	0.109	0.168
Gambia	Foni Bintang Karanai	2000-2017	-0.069	-0.040	-0.012
Gambia	Foni Bondali	2000	0.130	0.215	0.328
Gambia	Foni Bondali	2017	0.065	0.108	0.168
Gambia	Foni Bondali	2000-2017	-0.069	-0.040	-0.011
Gambia	Foni Brefet	2000	0.136	0.220	0.315
Gambia	Foni Brefet	2017	0.067	0.108	0.161
Gambia	Foni Brefet	2000-2017	-0.072	-0.041	-0.012
Gambia	Foni Jarrol	2000	0.136	0.225	0.339
Gambia	Foni Jarrol	2017	0.062	0.109	0.161

Table 1: LRI DALYs rate by unit *(continued)*

Country	Unit	year	mean rate	lower rate	upper rate
Gambia	Foni Jarrol	2000-2017	-0.075	-0.042	-0.005
Gambia	Foni Kansala	2000	0.133	0.224	0.332
Gambia	Foni Kansala	2017	0.069	0.112	0.176
Gambia	Foni Kansala	2000-2017	-0.070	-0.040	-0.008
Gambia	Fulladu East	2000	0.154	0.237	0.353
Gambia	Fulladu East	2017	0.061	0.103	0.152
Gambia	Fulladu East	2000-2017	-0.072	-0.049	-0.024
Gambia	Fulladu West	2000	0.122	0.198	0.293
Gambia	Fulladu West	2017	0.056	0.098	0.150
Gambia	Fulladu West	2000-2017	-0.065	-0.041	-0.016
Gambia	Janjanbureh	2000	0.103	0.184	0.296
Gambia	Janjanbureh	2017	0.053	0.098	0.173
Gambia	Janjanbureh	2000-2017	-0.087	-0.038	0.007
Gambia	Jarra Central	2000	0.128	0.208	0.311
Gambia	Jarra Central	2017	0.061	0.102	0.156
Gambia	Jarra Central	2000-2017	-0.071	-0.041	-0.011
Gambia	Jarra East	2000	0.140	0.219	0.329
Gambia	Jarra East	2017	0.061	0.103	0.157
Gambia	Jarra East	2000-2017	-0.073	-0.043	-0.013
Gambia	Jarra West	2000	0.122	0.196	0.295
Gambia	Jarra West	2017	0.058	0.101	0.151
Gambia	Jarra West	2000-2017	-0.065	-0.039	-0.009
Gambia	Jokadu	2000	0.146	0.217	0.310
Gambia	Jokadu	2017	0.069	0.112	0.171
Gambia	Jokadu	2000-2017	-0.066	-0.038	-0.011
Gambia	Kanifing	2000	0.131	0.208	0.313
Gambia	Kanifing	2017	0.063	0.109	0.174
Gambia	Kanifing	2000-2017	-0.071	-0.038	-0.005
Gambia	Kantora	2000	0.151	0.232	0.350
Gambia	Kantora	2017	0.063	0.104	0.156
Gambia	Kantora	2000-2017	-0.071	-0.048	-0.024
Gambia	Kiang Central	2000	0.120	0.201	0.296
Gambia	Kiang Central	2017	0.062	0.100	0.157
Gambia	Kiang Central	2000-2017	-0.068	-0.040	-0.008
Gambia	Kiang East	2000	0.133	0.206	0.292
Gambia	Kiang East	2017	0.064	0.103	0.159
Gambia	Kiang East	2000-2017	-0.069	-0.041	-0.012
Gambia	Kiang West	2000	0.125	0.200	0.299
Gambia	Kiang West	2017	0.060	0.101	0.158
Gambia	Kiang West	2000-2017	-0.064	-0.040	-0.013
Gambia	Kombo Central	2000	0.127	0.212	0.321
Gambia	Kombo Central	2017	0.065	0.108	0.175
Gambia	Kombo Central	2000-2017	-0.071	-0.040	-0.005
Gambia	Kombo East	2000	0.132	0.215	0.315
Gambia	Kombo East	2017	0.065	0.108	0.171
Gambia	Kombo East	2000-2017	-0.068	-0.039	-0.010
Gambia	Kombo Saint Mary	2000	0.135	0.210	0.308
Gambia	Kombo Saint Mary	2017	0.064	0.110	0.175
Gambia	Kombo Saint Mary	2000-2017	-0.068	-0.038	-0.005
Gambia	Kombo South	2000	0.140	0.217	0.327
Gambia	Kombo South	2017	0.065	0.111	0.169
Gambia	Kombo South	2000-2017	-0.065	-0.039	-0.010
Gambia	Lower Baddibu	2000	0.144	0.217	0.314
Gambia	Lower Baddibu	2017	0.067	0.112	0.175
Gambia	Lower Baddibu	2000-2017	-0.067	-0.038	-0.004
Gambia	Lower Nuimi	2000	0.142	0.213	0.308
Gambia	Lower Nuimi	2017	0.067	0.109	0.164
Gambia	Lower Nuimi	2000-2017	-0.065	-0.038	-0.010
Gambia	Lower Saloum	2000	0.149	0.235	0.341
Gambia	Lower Saloum	2017	0.065	0.105	0.157
Gambia	Lower Saloum	2000-2017	-0.075	-0.047	-0.023

Table 1: LRI DALYs rate by unit *(continued)*

Country	Unit	year	mean rate	lower rate	upper rate
Gambia	Niamina Dankunku	2000	0.128	0.204	0.295
Gambia	Niamina Dankunku	2017	0.060	0.101	0.158
Gambia	Niamina Dankunku	2000-2017	-0.070	-0.042	-0.014
Gambia	Niamina East	2000	0.133	0.213	0.324
Gambia	Niamina East	2017	0.060	0.102	0.159
Gambia	Niamina East	2000-2017	-0.070	-0.043	-0.017
Gambia	Niamina West	2000	0.156	0.243	0.363
Gambia	Niamina West	2017	0.060	0.101	0.155
Gambia	Niamina West	2000-2017	-0.077	-0.052	-0.025
Gambia	Niani	2000	0.150	0.231	0.346
Gambia	Niani	2017	0.063	0.106	0.165
Gambia	Niani	2000-2017	-0.070	-0.046	-0.022
Gambia	Nianija	2000	0.144	0.232	0.353
Gambia	Nianija	2017	0.058	0.104	0.166
Gambia	Nianija	2000-2017	-0.076	-0.047	-0.018
Gambia	Sami	2000	0.133	0.216	0.318
Gambia	Sami	2017	0.061	0.104	0.162
Gambia	Sami	2000-2017	-0.068	-0.045	-0.019
Gambia	Sandu	2000	0.147	0.230	0.338
Gambia	Sandu	2017	0.061	0.104	0.156
Gambia	Sandu	2000-2017	-0.071	-0.047	-0.022
Gambia	Upper Baddibu	2000	0.149	0.228	0.317
Gambia	Upper Baddibu	2017	0.071	0.110	0.164
Gambia	Upper Baddibu	2000-2017	-0.067	-0.042	-0.016
Gambia	Upper Nuimi	2000	0.137	0.211	0.308
Gambia	Upper Nuimi	2017	0.064	0.108	0.171
Gambia	Upper Nuimi	2000-2017	-0.067	-0.038	-0.010
Gambia	Upper Saloum	2000	0.153	0.245	0.356
Gambia	Upper Saloum	2017	0.065	0.108	0.161
Gambia	Upper Saloum	2000-2017	-0.074	-0.048	-0.023
Gambia	Wuli	2000	0.124	0.197	0.300
Gambia	Wuli	2017	0.060	0.099	0.146
Gambia	Wuli	2000-2017	-0.063	-0.040	-0.014
Ghana	Abura-Asebu-Kwamankese	2000	0.108	0.180	0.275
Ghana	Abura-Asebu-Kwamankese	2017	0.042	0.077	0.120
Ghana	Abura-Asebu-Kwamankese	2000-2017	-0.080	-0.049	-0.017
Ghana	Accra	2000	0.086	0.149	0.230
Ghana	Accra	2017	0.042	0.070	0.106
Ghana	Accra	2000-2017	-0.077	-0.046	-0.014
Ghana	Adaklu Anyigbe	2000	0.085	0.143	0.213
Ghana	Adaklu Anyigbe	2017	0.040	0.067	0.103
Ghana	Adaklu Anyigbe	2000-2017	-0.074	-0.046	-0.015
Ghana	Adansi North	2000	0.096	0.156	0.222
Ghana	Adansi North	2017	0.044	0.077	0.123
Ghana	Adansi North	2000-2017	-0.070	-0.042	-0.013
Ghana	Adansi South	2000	0.100	0.164	0.238
Ghana	Adansi South	2017	0.047	0.080	0.117
Ghana	Adansi South	2000-2017	-0.070	-0.043	-0.013
Ghana	Afigya Sekyere	2000	0.099	0.158	0.232
Ghana	Afigya Sekyere	2017	0.046	0.077	0.118
Ghana	Afigya Sekyere	2000-2017	-0.073	-0.044	-0.015
Ghana	Afram Plains	2000	0.101	0.167	0.248
Ghana	Afram Plains	2017	0.044	0.075	0.112
Ghana	Afram Plains	2000-2017	-0.076	-0.049	-0.019
Ghana	Agona	2000	0.101	0.165	0.246
Ghana	Agona	2017	0.045	0.076	0.115
Ghana	Agona	2000-2017	-0.074	-0.046	-0.015
Ghana	Ahafo Ano North	2000	0.101	0.161	0.237
Ghana	Ahafo Ano North	2017	0.046	0.079	0.119
Ghana	Ahafo Ano North	2000-2017	-0.070	-0.044	-0.015
Ghana	Ahafo Ano South	2000	0.104	0.167	0.249

Table 1: LRI DALYs rate by unit *(continued)*

Country	Unit	year	mean rate	lower rate	upper rate
Ghana	Ahafo Ano South	2017	0.049	0.083	0.127
Ghana	Ahafo Ano South	2000-2017	-0.070	-0.044	-0.015
Ghana	Ahanta West	2000	0.097	0.169	0.260
Ghana	Ahanta West	2017	0.042	0.077	0.119
Ghana	Ahanta West	2000-2017	-0.081	-0.045	-0.011
Ghana	Ajumako-Enyan-Esiam	2000	0.101	0.167	0.255
Ghana	Ajumako-Enyan-Esiam	2017	0.045	0.075	0.116
Ghana	Ajumako-Enyan-Esiam	2000-2017	-0.078	-0.047	-0.015
Ghana	Akatsi	2000	0.092	0.153	0.232
Ghana	Akatsi	2017	0.044	0.071	0.114
Ghana	Akatsi	2000-2017	-0.073	-0.047	-0.018
Ghana	Akwapim North	2000	0.089	0.145	0.223
Ghana	Akwapim North	2017	0.040	0.068	0.104
Ghana	Akwapim North	2000-2017	-0.076	-0.045	-0.013
Ghana	Akwapim South	2000	0.092	0.154	0.238
Ghana	Akwapim South	2017	0.040	0.071	0.105
Ghana	Akwapim South	2000-2017	-0.074	-0.047	-0.012
Ghana	Amansie Central	2000	0.095	0.160	0.236
Ghana	Amansie Central	2017	0.045	0.078	0.119
Ghana	Amansie Central	2000-2017	-0.071	-0.044	-0.013
Ghana	Amansie East	2000	0.098	0.161	0.242
Ghana	Amansie East	2017	0.047	0.079	0.120
Ghana	Amansie East	2000-2017	-0.071	-0.043	-0.010
Ghana	Amansie West	2000	0.098	0.161	0.239
Ghana	Amansie West	2017	0.048	0.078	0.115
Ghana	Amansie West	2000-2017	-0.072	-0.044	-0.016
Ghana	Aowin-Suaman	2000	0.106	0.177	0.260
Ghana	Aowin-Suaman	2017	0.049	0.082	0.123
Ghana	Aowin-Suaman	2000-2017	-0.072	-0.045	-0.013
Ghana	Asante Akim North	2000	0.102	0.161	0.233
Ghana	Asante Akim North	2017	0.047	0.078	0.119
Ghana	Asante Akim North	2000-2017	-0.070	-0.045	-0.016
Ghana	Asante Akim South	2000	0.103	0.162	0.233
Ghana	Asante Akim South	2017	0.047	0.078	0.117
Ghana	Asante Akim South	2000-2017	-0.073	-0.044	-0.015
Ghana	Asikuma Odoben Brakwa	2000	0.104	0.173	0.259
Ghana	Asikuma Odoben Brakwa	2017	0.048	0.080	0.122
Ghana	Asikuma Odoben Brakwa	2000-2017	-0.075	-0.046	-0.014
Ghana	Assin North	2000	0.099	0.167	0.243
Ghana	Assin North	2017	0.047	0.080	0.124
Ghana	Assin North	2000-2017	-0.073	-0.045	-0.012
Ghana	Assin South	2000	0.100	0.169	0.248
Ghana	Assin South	2017	0.045	0.078	0.116
Ghana	Assin South	2000-2017	-0.075	-0.046	-0.013
Ghana	Asunafo North	2000	0.100	0.166	0.253
Ghana	Asunafo North	2017	0.051	0.085	0.128
Ghana	Asunafo North	2000-2017	-0.067	-0.040	-0.013
Ghana	Asunafo South	2000	0.103	0.170	0.262
Ghana	Asunafo South	2017	0.050	0.084	0.123
Ghana	Asunafo South	2000-2017	-0.070	-0.043	-0.015
Ghana	Asuogyaman	2000	0.089	0.146	0.221
Ghana	Asuogyaman	2017	0.039	0.068	0.104
Ghana	Asuogyaman	2000-2017	-0.072	-0.046	-0.017
Ghana	Asutifi	2000	0.103	0.168	0.254
Ghana	Asutifi	2017	0.049	0.083	0.124
Ghana	Asutifi	2000-2017	-0.070	-0.043	-0.012
Ghana	Atebubu-Amantin	2000	0.117	0.177	0.254
Ghana	Atebubu-Amantin	2017	0.047	0.077	0.118
Ghana	Atebubu-Amantin	2000-2017	-0.078	-0.052	-0.021
Ghana	Atiwa	2000	0.108	0.168	0.253
Ghana	Atiwa	2017	0.049	0.081	0.121

Table 1: LRI DALYs rate by unit *(continued)*

Country	Unit	year	mean rate	lower rate	upper rate
Ghana	Atiwa	2000-2017	-0.070	-0.044	-0.013
Ghana	Atwima	2000	0.094	0.154	0.229
Ghana	Atwima	2017	0.043	0.077	0.119
Ghana	Atwima	2000-2017	-0.073	-0.042	-0.007
Ghana	Atwima Mponua	2000	0.106	0.168	0.249
Ghana	Atwima Mponua	2017	0.049	0.083	0.121
Ghana	Atwima Mponua	2000-2017	-0.070	-0.043	-0.014
Ghana	Awutu Efutu Senya	2000	0.096	0.161	0.245
Ghana	Awutu Efutu Senya	2017	0.044	0.075	0.120
Ghana	Awutu Efutu Senya	2000-2017	-0.074	-0.045	-0.012
Ghana	Bawku Municipal	2000	0.121	0.186	0.280
Ghana	Bawku Municipal	2017	0.052	0.085	0.131
Ghana	Bawku Municipal	2000-2017	-0.075	-0.046	-0.020
Ghana	Bawku West	2000	0.121	0.187	0.273
Ghana	Bawku West	2017	0.055	0.088	0.130
Ghana	Bawku West	2000-2017	-0.070	-0.045	-0.019
Ghana	Berekum	2000	0.104	0.168	0.251
Ghana	Berekum	2017	0.049	0.083	0.123
Ghana	Berekum	2000-2017	-0.070	-0.040	-0.008
Ghana	Bia	2000	0.102	0.168	0.255
Ghana	Bia	2017	0.049	0.085	0.127
Ghana	Bia	2000-2017	-0.067	-0.041	-0.008
Ghana	Bibiani Anhwiaso Bekwai	2000	0.105	0.167	0.250
Ghana	Bibiani Anhwiaso Bekwai	2017	0.048	0.081	0.121
Ghana	Bibiani Anhwiaso Bekwai	2000-2017	-0.071	-0.045	-0.016
Ghana	Birim North	2000	0.101	0.166	0.245
Ghana	Birim North	2017	0.048	0.080	0.121
Ghana	Birim North	2000-2017	-0.069	-0.044	-0.014
Ghana	Birim South	2000	0.097	0.155	0.227
Ghana	Birim South	2017	0.044	0.074	0.110
Ghana	Birim South	2000-2017	-0.073	-0.045	-0.015
Ghana	Bole	2000	0.137	0.210	0.301
Ghana	Bole	2017	0.055	0.087	0.129
Ghana	Bole	2000-2017	-0.079	-0.053	-0.025
Ghana	Bolgatanga	2000	0.111	0.180	0.275
Ghana	Bolgatanga	2017	0.054	0.089	0.145
Ghana	Bolgatanga	2000-2017	-0.072	-0.042	-0.012
Ghana	Bongo	2000	0.116	0.182	0.271
Ghana	Bongo	2017	0.055	0.092	0.142
Ghana	Bongo	2000-2017	-0.071	-0.041	-0.013
Ghana	Bosomtwe-Kwanwoma	2000	0.096	0.155	0.235
Ghana	Bosomtwe-Kwanwoma	2017	0.044	0.077	0.121
Ghana	Bosomtwe-Kwanwoma	2000-2017	-0.069	-0.042	-0.010
Ghana	Builsa	2000	0.115	0.184	0.264
Ghana	Builsa	2017	0.057	0.090	0.137
Ghana	Builsa	2000-2017	-0.068	-0.042	-0.014
Ghana	Bunkpurugu Yunyoo	2000	0.132	0.208	0.325
Ghana	Bunkpurugu Yunyoo	2017	0.055	0.088	0.130
Ghana	Bunkpurugu Yunyoo	2000-2017	-0.076	-0.053	-0.028
Ghana	Cape Coast	2000	0.101	0.175	0.267
Ghana	Cape Coast	2017	0.044	0.077	0.120
Ghana	Cape Coast	2000-2017	-0.081	-0.047	-0.012
Ghana	Central Gonja	2000	0.135	0.210	0.307
Ghana	Central Gonja	2017	0.055	0.086	0.131
Ghana	Central Gonja	2000-2017	-0.080	-0.054	-0.027
Ghana	Dangbe East	2000	0.089	0.152	0.234
Ghana	Dangbe East	2017	0.041	0.070	0.102
Ghana	Dangbe East	2000-2017	-0.074	-0.046	-0.016
Ghana	Dangbe West	2000	0.083	0.141	0.218
Ghana	Dangbe West	2017	0.041	0.067	0.103
Ghana	Dangbe West	2000-2017	-0.071	-0.045	-0.013

Table 1: LRI DALYs rate by unit *(continued)*

Country	Unit	year	mean rate	lower rate	upper rate
Ghana	Dormaa	2000	0.103	0.166	0.242
Ghana	Dormaa	2017	0.050	0.084	0.126
Ghana	Dormaa	2000-2017	-0.068	-0.040	-0.010
Ghana	East Akim	2000	0.102	0.169	0.259
Ghana	East Akim	2017	0.048	0.081	0.121
Ghana	East Akim	2000-2017	-0.074	-0.045	-0.014
Ghana	East Gonja	2000	0.141	0.216	0.323
Ghana	East Gonja	2017	0.053	0.083	0.121
Ghana	East Gonja	2000-2017	-0.081	-0.057	-0.028
Ghana	East Mamprusi	2000	0.136	0.209	0.309
Ghana	East Mamprusi	2017	0.055	0.089	0.134
Ghana	East Mamprusi	2000-2017	-0.081	-0.053	-0.030
Ghana	Ejisu-Juabeng	2000	0.097	0.155	0.234
Ghana	Ejisu-Juabeng	2017	0.044	0.077	0.118
Ghana	Ejisu-Juabeng	2000-2017	-0.071	-0.044	-0.012
Ghana	Ejura Sekyedumase	2000	0.108	0.167	0.242
Ghana	Ejura Sekyedumase	2017	0.045	0.077	0.117
Ghana	Ejura Sekyedumase	2000-2017	-0.074	-0.046	-0.015
Ghana	Fanteakwa	2000	0.100	0.163	0.240
Ghana	Fanteakwa	2017	0.045	0.077	0.115
Ghana	Fanteakwa	2000-2017	-0.071	-0.044	-0.012
Ghana	Ga East	2000	0.094	0.160	0.247
Ghana	Ga East	2017	0.045	0.074	0.115
Ghana	Ga East	2000-2017	-0.078	-0.047	-0.008
Ghana	Ga West	2000	0.087	0.150	0.229
Ghana	Ga West	2017	0.042	0.071	0.109
Ghana	Ga West	2000-2017	-0.075	-0.046	-0.012
Ghana	Garu Tempane	2000	0.123	0.192	0.289
Ghana	Garu Tempane	2017	0.053	0.085	0.128
Ghana	Garu Tempane	2000-2017	-0.074	-0.048	-0.019
Ghana	Gomoa	2000	0.102	0.168	0.251
Ghana	Gomoa	2017	0.044	0.075	0.113
Ghana	Gomoa	2000-2017	-0.074	-0.047	-0.011
Ghana	Gushiegu	2000	0.138	0.211	0.325
Ghana	Gushiegu	2017	0.059	0.090	0.133
Ghana	Gushiegu	2000-2017	-0.077	-0.053	-0.028
Ghana	Ho	2000	0.086	0.145	0.218
Ghana	Ho	2017	0.039	0.067	0.102
Ghana	Ho	2000-2017	-0.074	-0.047	-0.017
Ghana	Hohoe	2000	0.092	0.149	0.223
Ghana	Hohoe	2017	0.041	0.067	0.098
Ghana	Hohoe	2000-2017	-0.076	-0.049	-0.018
Ghana	Jaman North	2000	0.106	0.168	0.243
Ghana	Jaman North	2017	0.049	0.082	0.124
Ghana	Jaman North	2000-2017	-0.070	-0.042	-0.010
Ghana	Jaman South	2000	0.106	0.168	0.254
Ghana	Jaman South	2017	0.050	0.084	0.124
Ghana	Jaman South	2000-2017	-0.070	-0.041	-0.008
Ghana	Jasikan	2000	0.102	0.168	0.241
Ghana	Jasikan	2017	0.045	0.075	0.112
Ghana	Jasikan	2000-2017	-0.074	-0.049	-0.019
Ghana	Jirapa Lambussie	2000	0.122	0.195	0.295
Ghana	Jirapa Lambussie	2017	0.055	0.090	0.145
Ghana	Jirapa Lambussie	2000-2017	-0.070	-0.044	-0.016
Ghana	Jomoro	2000	0.110	0.188	0.279
Ghana	Jomoro	2017	0.052	0.086	0.130
Ghana	Jomoro	2000-2017	-0.078	-0.046	-0.012
Ghana	Juabeso	2000	0.106	0.174	0.257
Ghana	Juabeso	2017	0.050	0.083	0.126
Ghana	Juabeso	2000-2017	-0.068	-0.045	-0.015
Ghana	Kadjebi	2000	0.107	0.174	0.255

Table 1: LRI DALYs rate by unit *(continued)*

Country	Unit	year	mean rate	lower rate	upper rate
Ghana	Kadjebi	2017	0.049	0.079	0.117
Ghana	Kadjebi	2000-2017	-0.073	-0.048	-0.018
Ghana	Karaga	2000	0.137	0.213	0.322
Ghana	Karaga	2017	0.058	0.090	0.133
Ghana	Karaga	2000-2017	-0.079	-0.054	-0.029
Ghana	Kassena Nankana	2000	0.123	0.186	0.277
Ghana	Kassena Nankana	2017	0.058	0.092	0.144
Ghana	Kassena Nankana	2000-2017	-0.069	-0.042	-0.014
Ghana	Keta	2000	0.088	0.157	0.239
Ghana	Keta	2017	0.043	0.073	0.121
Ghana	Keta	2000-2017	-0.074	-0.047	-0.017
Ghana	Ketu	2000	0.086	0.142	0.208
Ghana	Ketu	2017	0.043	0.067	0.099
Ghana	Ketu	2000-2017	-0.070	-0.046	-0.016
Ghana	Kintampo North	2000	0.117	0.180	0.260
Ghana	Kintampo North	2017	0.050	0.081	0.119
Ghana	Kintampo North	2000-2017	-0.079	-0.049	-0.020
Ghana	Kintampo South	2000	0.115	0.177	0.253
Ghana	Kintampo South	2017	0.047	0.078	0.120
Ghana	Kintampo South	2000-2017	-0.080	-0.050	-0.022
Ghana	Komenda-Edina-Eguafo-Abirem	2000	0.111	0.177	0.261
Ghana	Komenda-Edina-Eguafo-Abirem	2017	0.042	0.077	0.118
Ghana	Komenda-Edina-Eguafo-Abirem	2000-2017	-0.077	-0.048	-0.015
Ghana	Kpandu	2000	0.086	0.143	0.208
Ghana	Kpandu	2017	0.039	0.066	0.101
Ghana	Kpandu	2000-2017	-0.077	-0.048	-0.017
Ghana	Krachi	2000	0.118	0.185	0.273
Ghana	Krachi	2017	0.048	0.077	0.113
Ghana	Krachi	2000-2017	-0.078	-0.053	-0.024
Ghana	Krachi East	2000	0.108	0.174	0.257
Ghana	Krachi East	2017	0.048	0.078	0.117
Ghana	Krachi East	2000-2017	-0.072	-0.047	-0.016
Ghana	Kumasi	2000	0.099	0.158	0.235
Ghana	Kumasi	2017	0.046	0.078	0.123
Ghana	Kumasi	2000-2017	-0.073	-0.041	-0.006
Ghana	Kwabibirem	2000	0.104	0.167	0.250
Ghana	Kwabibirem	2017	0.049	0.080	0.120
Ghana	Kwabibirem	2000-2017	-0.070	-0.044	-0.014
Ghana	Kwabre	2000	0.096	0.156	0.234
Ghana	Kwabre	2017	0.045	0.077	0.119
Ghana	Kwabre	2000-2017	-0.071	-0.043	-0.007
Ghana	Kwahu South	2000	0.088	0.144	0.214
Ghana	Kwahu South	2017	0.042	0.070	0.106
Ghana	Kwahu South	2000-2017	-0.071	-0.044	-0.013
Ghana	Kwahu West	2000	0.090	0.150	0.226
Ghana	Kwahu West	2017	0.043	0.072	0.106
Ghana	Kwahu West	2000-2017	-0.073	-0.044	-0.010
Ghana	Lawra	2000	0.125	0.198	0.297
Ghana	Lawra	2017	0.055	0.089	0.140
Ghana	Lawra	2000-2017	-0.072	-0.046	-0.015
Ghana	Lower Denkyira	2000	0.103	0.170	0.248
Ghana	Lower Denkyira	2017	0.045	0.080	0.119
Ghana	Lower Denkyira	2000-2017	-0.073	-0.045	-0.012
Ghana	Manya Krobo	2000	0.098	0.161	0.248
Ghana	Manya Krobo	2017	0.043	0.076	0.114
Ghana	Manya Krobo	2000-2017	-0.075	-0.045	-0.017
Ghana	Mfantsiman	2000	0.099	0.173	0.258
Ghana	Mfantsiman	2017	0.045	0.076	0.116
Ghana	Mfantsiman	2000-2017	-0.082	-0.048	-0.016
Ghana	Mpohor Wassa East	2000	0.103	0.176	0.258
Ghana	Mpohor Wassa East	2017	0.044	0.080	0.122

Table 1: LRI DALYs rate by unit *(continued)*

Country	Unit	year	mean rate	lower rate	upper rate
Ghana	Mpohor Wassa East	2000-2017	-0.079	-0.046	-0.018
Ghana	Nadowli	2000	0.117	0.189	0.275
Ghana	Nadowli	2017	0.056	0.090	0.143
Ghana	Nadowli	2000-2017	-0.069	-0.043	-0.014
Ghana	Nanumba North	2000	0.140	0.217	0.331
Ghana	Nanumba North	2017	0.053	0.083	0.121
Ghana	Nanumba North	2000-2017	-0.081	-0.057	-0.030
Ghana	Nanumba South	2000	0.129	0.202	0.300
Ghana	Nanumba South	2017	0.053	0.082	0.124
Ghana	Nanumba South	2000-2017	-0.078	-0.053	-0.026
Ghana	New Juaben	2000	0.085	0.147	0.232
Ghana	New Juaben	2017	0.039	0.070	0.107
Ghana	New Juaben	2000-2017	-0.074	-0.045	-0.013
Ghana	Nkoranza	2000	0.107	0.169	0.240
Ghana	Nkoranza	2017	0.047	0.078	0.118
Ghana	Nkoranza	2000-2017	-0.073	-0.047	-0.018
Ghana	Nkwanta	2000	0.103	0.160	0.236
Ghana	Nkwanta	2017	0.045	0.070	0.104
Ghana	Nkwanta	2000-2017	-0.074	-0.049	-0.021
Ghana	North Tongu	2000	0.091	0.152	0.232
Ghana	North Tongu	2017	0.043	0.070	0.111
Ghana	North Tongu	2000-2017	-0.073	-0.046	-0.015
Ghana	Nzema East	2000	0.105	0.182	0.272
Ghana	Nzema East	2017	0.046	0.083	0.124
Ghana	Nzema East	2000-2017	-0.076	-0.045	-0.013
Ghana	Obuasi Municipal	2000	0.103	0.172	0.253
Ghana	Obuasi Municipal	2017	0.048	0.084	0.132
Ghana	Obuasi Municipal	2000-2017	-0.075	-0.045	-0.014
Ghana	Offinso	2000	0.099	0.160	0.236
Ghana	Offinso	2017	0.046	0.078	0.118
Ghana	Offinso	2000-2017	-0.075	-0.044	-0.012
Ghana	Pru	2000	0.123	0.185	0.275
Ghana	Pru	2017	0.049	0.079	0.119
Ghana	Pru	2000-2017	-0.080	-0.053	-0.022
Ghana	Saboba Chereponi	2000	0.135	0.206	0.308
Ghana	Saboba Chereponi	2017	0.058	0.088	0.125
Ghana	Saboba Chereponi	2000-2017	-0.076	-0.052	-0.026
Ghana	Savelugu Nanton	2000	0.135	0.213	0.322
Ghana	Savelugu Nanton	2017	0.057	0.089	0.134
Ghana	Savelugu Nanton	2000-2017	-0.079	-0.055	-0.027
Ghana	Sawa-Tuna-Kalba	2000	0.134	0.208	0.305
Ghana	Sawa-Tuna-Kalba	2017	0.055	0.089	0.134
Ghana	Sawa-Tuna-Kalba	2000-2017	-0.075	-0.050	-0.023
Ghana	Sefwi Wiawso	2000	0.104	0.172	0.256
Ghana	Sefwi Wiawso	2017	0.050	0.083	0.125
Ghana	Sefwi Wiawso	2000-2017	-0.073	-0.045	-0.016
Ghana	Sekyere East	2000	0.107	0.165	0.236
Ghana	Sekyere East	2017	0.046	0.077	0.116
Ghana	Sekyere East	2000-2017	-0.074	-0.047	-0.016
Ghana	Sekyere West	2000	0.108	0.163	0.242
Ghana	Sekyere West	2017	0.046	0.077	0.116
Ghana	Sekyere West	2000-2017	-0.072	-0.045	-0.016
Ghana	Sene	2000	0.120	0.184	0.270
Ghana	Sene	2017	0.047	0.078	0.116
Ghana	Sene	2000-2017	-0.077	-0.052	-0.023
Ghana	Shama Ahanta East	2000	0.098	0.176	0.275
Ghana	Shama Ahanta East	2017	0.045	0.079	0.119
Ghana	Shama Ahanta East	2000-2017	-0.077	-0.046	-0.006
Ghana	Sissala East	2000	0.120	0.188	0.272
Ghana	Sissala East	2017	0.057	0.092	0.145
Ghana	Sissala East	2000-2017	-0.067	-0.042	-0.016

Table 1: LRI DALYs rate by unit *(continued)*

Country	Unit	year	mean rate	lower rate	upper rate
Ghana	Sissala West	2000	0.122	0.195	0.292
Ghana	Sissala West	2017	0.057	0.091	0.146
Ghana	Sissala West	2000-2017	-0.068	-0.045	-0.019
Ghana	South Dayi	2000	0.084	0.146	0.228
Ghana	South Dayi	2017	0.039	0.067	0.097
Ghana	South Dayi	2000-2017	-0.076	-0.046	-0.011
Ghana	South Tongu	2000	0.089	0.151	0.227
Ghana	South Tongu	2017	0.043	0.070	0.110
Ghana	South Tongu	2000-2017	-0.074	-0.046	-0.014
Ghana	Suhum Kraboa Coaltar	2000	0.097	0.161	0.241
Ghana	Suhum Kraboa Coaltar	2017	0.046	0.077	0.114
Ghana	Suhum Kraboa Coaltar	2000-2017	-0.073	-0.044	-0.015
Ghana	Sunyani	2000	0.105	0.169	0.243
Ghana	Sunyani	2017	0.049	0.083	0.124
Ghana	Sunyani	2000-2017	-0.070	-0.041	-0.011
Ghana	Tain	2000	0.106	0.169	0.239
Ghana	Tain	2017	0.049	0.081	0.124
Ghana	Tain	2000-2017	-0.071	-0.043	-0.016
Ghana	Talensi Nabdam	2000	0.122	0.188	0.272
Ghana	Talensi Nabdam	2017	0.056	0.088	0.132
Ghana	Talensi Nabdam	2000-2017	-0.074	-0.046	-0.020
Ghana	Tamale	2000	0.118	0.184	0.277
Ghana	Tamale	2017	0.053	0.084	0.126
Ghana	Tamale	2000-2017	-0.076	-0.046	-0.015
Ghana	Tano North	2000	0.106	0.171	0.251
Ghana	Tano North	2017	0.050	0.083	0.123
Ghana	Tano North	2000-2017	-0.073	-0.044	-0.013
Ghana	Tano South	2000	0.105	0.167	0.250
Ghana	Tano South	2017	0.049	0.082	0.122
Ghana	Tano South	2000-2017	-0.073	-0.044	-0.012
Ghana	Techiman	2000	0.109	0.168	0.241
Ghana	Techiman	2017	0.049	0.081	0.121
Ghana	Techiman	2000-2017	-0.071	-0.043	-0.015
Ghana	Tema	2000	0.096	0.156	0.240
Ghana	Tema	2017	0.046	0.075	0.111
Ghana	Tema	2000-2017	-0.077	-0.045	-0.010
Ghana	Tolon-Kumbungu	2000	0.137	0.210	0.308
Ghana	Tolon-Kumbungu	2017	0.055	0.086	0.130
Ghana	Tolon-Kumbungu	2000-2017	-0.080	-0.054	-0.027
Ghana	Upper Denkyira	2000	0.101	0.169	0.247
Ghana	Upper Denkyira	2017	0.046	0.081	0.124
Ghana	Upper Denkyira	2000-2017	-0.075	-0.047	-0.017
Ghana	Wa	2000	0.118	0.184	0.274
Ghana	Wa	2017	0.054	0.089	0.137
Ghana	Wa	2000-2017	-0.068	-0.042	-0.016
Ghana	Wa East	2000	0.119	0.187	0.271
Ghana	Wa East	2017	0.055	0.088	0.135
Ghana	Wa East	2000-2017	-0.069	-0.044	-0.018
Ghana	Wa West	2000	0.119	0.184	0.278
Ghana	Wa West	2017	0.054	0.089	0.135
Ghana	Wa West	2000-2017	-0.069	-0.042	-0.014
Ghana	Wasa Amenfi East	2000	0.105	0.172	0.245
Ghana	Wasa Amenfi East	2017	0.048	0.081	0.123
Ghana	Wasa Amenfi East	2000-2017	-0.076	-0.047	-0.016
Ghana	Wasa Amenfi West	2000	0.107	0.176	0.255
Ghana	Wasa Amenfi West	2017	0.049	0.083	0.125
Ghana	Wasa Amenfi West	2000-2017	-0.076	-0.047	-0.016
Ghana	Wassa West	2000	0.107	0.181	0.265
Ghana	Wassa West	2017	0.048	0.083	0.125
Ghana	Wassa West	2000-2017	-0.076	-0.046	-0.015
Ghana	West Akim	2000	0.101	0.165	0.247

Table 1: LRI DALYs rate by unit *(continued)*

Country	Unit	year	mean rate	lower rate	upper rate
Ghana	West Akim	2017	0.047	0.078	0.117
Ghana	West Akim	2000-2017	-0.073	-0.045	-0.012
Ghana	West Gonja	2000	0.135	0.210	0.297
Ghana	West Gonja	2017	0.056	0.088	0.133
Ghana	West Gonja	2000-2017	-0.074	-0.050	-0.023
Ghana	West Mamprusi	2000	0.133	0.205	0.302
Ghana	West Mamprusi	2017	0.057	0.090	0.134
Ghana	West Mamprusi	2000-2017	-0.075	-0.050	-0.024
Ghana	Yendi	2000	0.143	0.217	0.332
Ghana	Yendi	2017	0.057	0.086	0.128
Ghana	Yendi	2000-2017	-0.081	-0.056	-0.031
Ghana	Yilo Krobo	2000	0.095	0.152	0.235
Ghana	Yilo Krobo	2017	0.041	0.071	0.105
Ghana	Yilo Krobo	2000-2017	-0.073	-0.045	-0.014
Ghana	Zabzugu Tatale	2000	0.133	0.201	0.299
Ghana	Zabzugu Tatale	2017	0.054	0.082	0.119
Ghana	Zabzugu Tatale	2000-2017	-0.077	-0.054	-0.029
Guinea	Beyla	2000	0.380	0.623	0.976
Guinea	Beyla	2017	0.155	0.270	0.435
Guinea	Beyla	2000-2017	-0.080	-0.052	-0.027
Guinea	Boffa	2000	0.342	0.591	0.931
Guinea	Boffa	2017	0.191	0.320	0.514
Guinea	Boffa	2000-2017	-0.063	-0.036	-0.011
Guinea	Boké	2000	0.346	0.603	0.930
Guinea	Boké	2017	0.193	0.325	0.534
Guinea	Boké	2000-2017	-0.066	-0.037	-0.011
Guinea	Conakry	2000	0.324	0.574	0.925
Guinea	Conakry	2017	0.174	0.298	0.461
Guinea	Conakry	2000-2017	-0.070	-0.037	-0.005
Guinea	Coyah	2000	0.391	0.656	1.025
Guinea	Coyah	2017	0.182	0.316	0.531
Guinea	Coyah	2000-2017	-0.074	-0.043	-0.014
Guinea	Dabola	2000	0.412	0.668	1.033
Guinea	Dabola	2017	0.190	0.313	0.507
Guinea	Dabola	2000-2017	-0.069	-0.042	-0.015
Guinea	Dalaba	2000	0.429	0.717	1.108
Guinea	Dalaba	2017	0.189	0.304	0.482
Guinea	Dalaba	2000-2017	-0.074	-0.048	-0.020
Guinea	Dinguiraye	2000	0.391	0.645	0.986
Guinea	Dinguiraye	2017	0.185	0.298	0.452
Guinea	Dinguiraye	2000-2017	-0.070	-0.044	-0.017
Guinea	Dubréka	2000	0.371	0.619	0.948
Guinea	Dubréka	2017	0.189	0.313	0.527
Guinea	Dubréka	2000-2017	-0.068	-0.042	-0.015
Guinea	Faranah	2000	0.404	0.676	1.046
Guinea	Faranah	2017	0.176	0.297	0.495
Guinea	Faranah	2000-2017	-0.070	-0.046	-0.016
Guinea	Forécariah	2000	0.386	0.641	0.965
Guinea	Forécariah	2017	0.179	0.304	0.495
Guinea	Forécariah	2000-2017	-0.073	-0.046	-0.018
Guinea	Fria	2000	0.381	0.620	0.978
Guinea	Fria	2017	0.200	0.320	0.516
Guinea	Fria	2000-2017	-0.067	-0.039	-0.013
Guinea	Gaoual	2000	0.427	0.722	1.078
Guinea	Gaoual	2017	0.194	0.318	0.529
Guinea	Gaoual	2000-2017	-0.070	-0.046	-0.022
Guinea	Guéckédou	2000	0.380	0.646	0.996
Guinea	Guéckédou	2017	0.165	0.280	0.444
Guinea	Guéckédou	2000-2017	-0.075	-0.049	-0.020
Guinea	Kankan	2000	0.370	0.614	0.968
Guinea	Kankan	2017	0.154	0.268	0.438

Table 1: LRI DALYs rate by unit *(continued)*

Country	Unit	year	mean rate	lower rate	upper rate
Guinea	Kankan	2000-2017	-0.077	-0.049	-0.018
Guinea	Kérouané	2000	0.400	0.651	1.007
Guinea	Kérouané	2017	0.155	0.271	0.437
Guinea	Kérouané	2000-2017	-0.078	-0.052	-0.025
Guinea	Kindia	2000	0.393	0.649	0.969
Guinea	Kindia	2017	0.185	0.309	0.508
Guinea	Kindia	2000-2017	-0.071	-0.045	-0.017
Guinea	Kissidougou	2000	0.399	0.666	1.039
Guinea	Kissidougou	2017	0.169	0.283	0.469
Guinea	Kissidougou	2000-2017	-0.075	-0.051	-0.021
Guinea	Koubia	2000	0.408	0.692	1.062
Guinea	Koubia	2017	0.202	0.328	0.526
Guinea	Koubia	2000-2017	-0.066	-0.042	-0.016
Guinea	Koundara	2000	0.417	0.676	1.029
Guinea	Koundara	2017	0.194	0.310	0.507
Guinea	Koundara	2000-2017	-0.069	-0.046	-0.022
Guinea	Kouroussa	2000	0.389	0.633	0.973
Guinea	Kouroussa	2017	0.174	0.286	0.457
Guinea	Kouroussa	2000-2017	-0.073	-0.047	-0.018
Guinea	Labé	2000	0.462	0.759	1.134
Guinea	Labé	2017	0.190	0.306	0.500
Guinea	Labé	2000-2017	-0.075	-0.048	-0.018
Guinea	Lélouma	2000	0.456	0.748	1.155
Guinea	Lélouma	2017	0.193	0.312	0.513
Guinea	Lélouma	2000-2017	-0.072	-0.046	-0.020
Guinea	Lola	2000	0.375	0.623	0.979
Guinea	Lola	2017	0.153	0.262	0.404
Guinea	Lola	2000-2017	-0.083	-0.053	-0.026
Guinea	Macenta	2000	0.388	0.653	1.007
Guinea	Macenta	2017	0.163	0.278	0.441
Guinea	Macenta	2000-2017	-0.079	-0.052	-0.025
Guinea	Mali	2000	0.411	0.696	1.065
Guinea	Mali	2017	0.196	0.317	0.512
Guinea	Mali	2000-2017	-0.068	-0.044	-0.020
Guinea	Mamou	2000	0.405	0.690	1.080
Guinea	Mamou	2017	0.183	0.307	0.500
Guinea	Mamou	2000-2017	-0.073	-0.046	-0.020
Guinea	Mandiana	2000	0.357	0.606	0.948
Guinea	Mandiana	2017	0.148	0.258	0.415
Guinea	Mandiana	2000-2017	-0.074	-0.051	-0.025
Guinea	Nzérékoré	2000	0.361	0.612	0.947
Guinea	Nzérékoré	2017	0.157	0.276	0.431
Guinea	Nzérékoré	2000-2017	-0.077	-0.050	-0.021
Guinea	Pita	2000	0.469	0.774	1.178
Guinea	Pita	2017	0.183	0.309	0.495
Guinea	Pita	2000-2017	-0.074	-0.050	-0.025
Guinea	Siguiri	2000	0.361	0.614	0.975
Guinea	Siguiri	2017	0.156	0.266	0.425
Guinea	Siguiri	2000-2017	-0.074	-0.049	-0.023
Guinea	Télimélé	2000	0.438	0.707	1.080
Guinea	Télimélé	2017	0.190	0.315	0.528
Guinea	Télimélé	2000-2017	-0.076	-0.048	-0.023
Guinea	Tougué	2000	0.420	0.684	1.036
Guinea	Tougué	2017	0.193	0.313	0.492
Guinea	Tougué	2000-2017	-0.067	-0.042	-0.015
Guinea	Yamou	2000	0.442	0.702	1.100
Guinea	Yamou	2017	0.171	0.283	0.432
Guinea	Yamou	2000-2017	-0.084	-0.056	-0.030
Guinea-Bissau	Bafata	2000	0.230	0.375	0.547
Guinea-Bissau	Bafata	2017	0.080	0.132	0.198
Guinea-Bissau	Bafata	2000-2017	-0.082	-0.056	-0.029

Table 1: LRI DALYs rate by unit *(continued)*

Country	Unit	year	mean rate	lower rate	upper rate
Guinea-Bissau	Bambadinca	2000	0.238	0.386	0.578
Guinea-Bissau	Bambadinca	2017	0.082	0.134	0.211
Guinea-Bissau	Bambadinca	2000-2017	-0.080	-0.055	-0.030
Guinea-Bissau	Bedanda	2000	0.215	0.341	0.541
Guinea-Bissau	Bedanda	2017	0.079	0.135	0.207
Guinea-Bissau	Bedanda	2000-2017	-0.078	-0.052	-0.026
Guinea-Bissau	Bigene	2000	0.232	0.383	0.586
Guinea-Bissau	Bigene	2017	0.083	0.136	0.202
Guinea-Bissau	Bigene	2000-2017	-0.080	-0.055	-0.028
Guinea-Bissau	Bissau	2000	0.186	0.335	0.546
Guinea-Bissau	Bissau	2017	0.070	0.118	0.192
Guinea-Bissau	Bissau	2000-2017	-0.092	-0.055	-0.018
Guinea-Bissau	Bissora	2000	0.250	0.412	0.624
Guinea-Bissau	Bissora	2017	0.082	0.134	0.206
Guinea-Bissau	Bissora	2000-2017	-0.088	-0.061	-0.032
Guinea-Bissau	Boe	2000	0.215	0.359	0.542
Guinea-Bissau	Boe	2017	0.083	0.133	0.201
Guinea-Bissau	Boe	2000-2017	-0.076	-0.054	-0.031
Guinea-Bissau	Bolama	2000	0.214	0.365	0.578
Guinea-Bissau	Bolama	2017	0.078	0.133	0.217
Guinea-Bissau	Bolama	2000-2017	-0.088	-0.057	-0.023
Guinea-Bissau	Buba	2000	0.228	0.372	0.574
Guinea-Bissau	Buba	2017	0.082	0.135	0.205
Guinea-Bissau	Buba	2000-2017	-0.082	-0.057	-0.031
Guinea-Bissau	Bubaque	2000	0.205	0.333	0.557
Guinea-Bissau	Bubaque	2017	0.079	0.132	0.206
Guinea-Bissau	Bubaque	2000-2017	-0.076	-0.048	-0.018
Guinea-Bissau	Bula	2000	0.245	0.399	0.605
Guinea-Bissau	Bula	2017	0.082	0.135	0.210
Guinea-Bissau	Bula	2000-2017	-0.086	-0.059	-0.031
Guinea-Bissau	Cacheu	2000	0.221	0.384	0.588
Guinea-Bissau	Cacheu	2017	0.080	0.135	0.205
Guinea-Bissau	Cacheu	2000-2017	-0.081	-0.054	-0.024
Guinea-Bissau	Cacine	2000	0.201	0.325	0.511
Guinea-Bissau	Cacine	2017	0.081	0.135	0.202
Guinea-Bissau	Cacine	2000-2017	-0.073	-0.045	-0.022
Guinea-Bissau	Caio	2000	0.222	0.365	0.588
Guinea-Bissau	Caio	2017	0.081	0.134	0.206
Guinea-Bissau	Caio	2000-2017	-0.080	-0.052	-0.023
Guinea-Bissau	Canghungo	2000	0.231	0.391	0.610
Guinea-Bissau	Canghungo	2017	0.082	0.135	0.204
Guinea-Bissau	Canghungo	2000-2017	-0.083	-0.056	-0.027
Guinea-Bissau	Caravela	2000	0.205	0.333	0.546
Guinea-Bissau	Caravela	2017	0.077	0.133	0.215
Guinea-Bissau	Caravela	2000-2017	-0.078	-0.047	-0.011
Guinea-Bissau	Catio	2000	0.214	0.343	0.533
Guinea-Bissau	Catio	2017	0.081	0.135	0.203
Guinea-Bissau	Catio	2000-2017	-0.080	-0.052	-0.021
Guinea-Bissau	Contuboel	2000	0.221	0.362	0.525
Guinea-Bissau	Contuboel	2017	0.083	0.132	0.195
Guinea-Bissau	Contuboel	2000-2017	-0.080	-0.055	-0.030
Guinea-Bissau	Empada	2000	0.218	0.351	0.535
Guinea-Bissau	Empada	2017	0.081	0.134	0.210
Guinea-Bissau	Empada	2000-2017	-0.082	-0.054	-0.029
Guinea-Bissau	Farim	2000	0.245	0.393	0.583
Guinea-Bissau	Farim	2017	0.086	0.135	0.197
Guinea-Bissau	Farim	2000-2017	-0.083	-0.058	-0.029
Guinea-Bissau	Fulacunda	2000	0.233	0.375	0.585
Guinea-Bissau	Fulacunda	2017	0.080	0.135	0.206
Guinea-Bissau	Fulacunda	2000-2017	-0.083	-0.057	-0.029
Guinea-Bissau	Gabu	2000	0.218	0.364	0.537

Table 1: LRI DALYs rate by unit *(continued)*

Country	Unit	year	mean rate	lower rate	upper rate
Guinea-Bissau	Gabu	2017	0.078	0.130	0.190
Guinea-Bissau	Gabu	2000-2017	-0.080	-0.055	-0.031
Guinea-Bissau	Galomaro	2000	0.222	0.380	0.575
Guinea-Bissau	Galomaro	2017	0.080	0.134	0.200
Guinea-Bissau	Galomaro	2000-2017	-0.079	-0.056	-0.028
Guinea-Bissau	Gamamundo	2000	0.227	0.384	0.566
Guinea-Bissau	Gamamundo	2017	0.082	0.132	0.200
Guinea-Bissau	Gamamundo	2000-2017	-0.081	-0.057	-0.033
Guinea-Bissau	Mansaba	2000	0.245	0.402	0.601
Guinea-Bissau	Mansaba	2017	0.081	0.134	0.208
Guinea-Bissau	Mansaba	2000-2017	-0.086	-0.059	-0.034
Guinea-Bissau	Mansoa	2000	0.242	0.402	0.612
Guinea-Bissau	Mansoa	2017	0.082	0.135	0.203
Guinea-Bissau	Mansoa	2000-2017	-0.083	-0.059	-0.031
Guinea-Bissau	Nhacra	2000	0.236	0.393	0.595
Guinea-Bissau	Nhacra	2017	0.073	0.130	0.206
Guinea-Bissau	Nhacra	2000-2017	-0.089	-0.062	-0.030
Guinea-Bissau	Piche	2000	0.215	0.349	0.515
Guinea-Bissau	Piche	2017	0.078	0.127	0.186
Guinea-Bissau	Piche	2000-2017	-0.077	-0.054	-0.031
Guinea-Bissau	Pirada	2000	0.209	0.347	0.522
Guinea-Bissau	Pirada	2017	0.079	0.128	0.191
Guinea-Bissau	Pirada	2000-2017	-0.075	-0.054	-0.030
Guinea-Bissau	Prabis	2000	0.210	0.346	0.555
Guinea-Bissau	Prabis	2017	0.077	0.131	0.206
Guinea-Bissau	Prabis	2000-2017	-0.081	-0.051	-0.017
Guinea-Bissau	Quebo	2000	0.228	0.374	0.567
Guinea-Bissau	Quebo	2017	0.081	0.134	0.204
Guinea-Bissau	Quebo	2000-2017	-0.084	-0.058	-0.031
Guinea-Bissau	Quinhamel	2000	0.224	0.357	0.552
Guinea-Bissau	Quinhamel	2017	0.081	0.134	0.211
Guinea-Bissau	Quinhamel	2000-2017	-0.081	-0.052	-0.023
Guinea-Bissau	Safim	2000	0.207	0.341	0.542
Guinea-Bissau	Safim	2017	0.074	0.127	0.201
Guinea-Bissau	Safim	2000-2017	-0.082	-0.050	-0.017
Guinea-Bissau	Sao Domingos	2000	0.212	0.346	0.513
Guinea-Bissau	Sao Domingos	2017	0.087	0.142	0.212
Guinea-Bissau	Sao Domingos	2000-2017	-0.075	-0.048	-0.019
Guinea-Bissau	Sonaco	2000	0.219	0.362	0.539
Guinea-Bissau	Sonaco	2017	0.078	0.129	0.192
Guinea-Bissau	Sonaco	2000-2017	-0.083	-0.056	-0.029
Guinea-Bissau	Tite	2000	0.224	0.364	0.552
Guinea-Bissau	Tite	2017	0.083	0.137	0.211
Guinea-Bissau	Tite	2000-2017	-0.081	-0.055	-0.026
Guinea-Bissau	Xitole	2000	0.238	0.386	0.584
Guinea-Bissau	Xitole	2017	0.080	0.134	0.202
Guinea-Bissau	Xitole	2000-2017	-0.082	-0.055	-0.028
Kenya	Ainabkoi	2000	0.095	0.127	0.163
Kenya	Ainabkoi	2017	0.062	0.085	0.117
Kenya	Ainabkoi	2000-2017	-0.032	-0.020	-0.007
Kenya	Ainamoi	2000	0.056	0.078	0.102
Kenya	Ainamoi	2017	0.072	0.097	0.128
Kenya	Ainamoi	2000-2017	-0.005	0.011	0.028
Kenya	Aldai	2000	0.079	0.105	0.137
Kenya	Aldai	2017	0.053	0.070	0.092
Kenya	Aldai	2000-2017	-0.034	-0.021	-0.009
Kenya	Alego Usonga	2000	0.317	0.413	0.535
Kenya	Alego Usonga	2017	0.144	0.186	0.239
Kenya	Alego Usonga	2000-2017	-0.062	-0.049	-0.038
Kenya	Awendo	2000	0.776	1.041	1.302
Kenya	Awendo	2017	0.237	0.309	0.401

Table 1: LRI DALYs rate by unit *(continued)*

Country	Unit	year	mean rate	lower rate	upper rate
Kenya	Awendo	2000-2017	-0.087	-0.071	-0.059
Kenya	Bahati	2000	0.094	0.123	0.159
Kenya	Bahati	2017	0.102	0.137	0.181
Kenya	Bahati	2000-2017	-0.008	0.006	0.020
Kenya	Balambala	2000	0.227	0.294	0.383
Kenya	Balambala	2017	0.056	0.072	0.091
Kenya	Balambala	2000-2017	-0.090	-0.082	-0.073
Kenya	Banissa	2000	0.225	0.302	0.397
Kenya	Banissa	2017	0.079	0.106	0.138
Kenya	Banissa	2000-2017	-0.075	-0.065	-0.055
Kenya	Baringo Central	2000	0.335	0.443	0.583
Kenya	Baringo Central	2017	0.088	0.118	0.154
Kenya	Baringo Central	2000-2017	-0.095	-0.081	-0.066
Kenya	Baringo North	2000	0.325	0.432	0.565
Kenya	Baringo North	2017	0.087	0.115	0.149
Kenya	Baringo North	2000-2017	-0.091	-0.081	-0.072
Kenya	Baringo South	2000	0.318	0.420	0.542
Kenya	Baringo South	2017	0.087	0.113	0.146
Kenya	Baringo South	2000-2017	-0.090	-0.081	-0.072
Kenya	Belgut	2000	0.059	0.078	0.101
Kenya	Belgut	2017	0.071	0.095	0.125
Kenya	Belgut	2000-2017	-0.004	0.011	0.023
Kenya	Bobasi	2000	0.229	0.305	0.399
Kenya	Bobasi	2017	0.108	0.141	0.183
Kenya	Bobasi	2000-2017	-0.059	-0.046	-0.034
Kenya	Bomachoge Borabu	2000	0.222	0.299	0.390
Kenya	Bomachoge Borabu	2017	0.102	0.141	0.186
Kenya	Bomachoge Borabu	2000-2017	-0.064	-0.045	-0.027
Kenya	Bomachoge Chache	2000	0.236	0.314	0.404
Kenya	Bomachoge Chache	2017	0.104	0.140	0.183
Kenya	Bomachoge Chache	2000-2017	-0.065	-0.047	-0.029
Kenya	Bomet Central	2000	0.042	0.057	0.076
Kenya	Bomet Central	2017	0.033	0.044	0.060
Kenya	Bomet Central	2000-2017	-0.031	-0.016	-0.002
Kenya	Bomet East	2000	0.044	0.057	0.073
Kenya	Bomet East	2017	0.031	0.041	0.054
Kenya	Bomet East	2000-2017	-0.033	-0.020	-0.007
Kenya	Bonchari	2000	0.232	0.323	0.421
Kenya	Bonchari	2017	0.102	0.139	0.189
Kenya	Bonchari	2000-2017	-0.068	-0.048	-0.026
Kenya	Bondo	2000	0.309	0.410	0.533
Kenya	Bondo	2017	0.149	0.193	0.251
Kenya	Bondo	2000-2017	-0.060	-0.047	-0.036
Kenya	Borabu	2000	0.162	0.217	0.283
Kenya	Borabu	2017	0.055	0.072	0.094
Kenya	Borabu	2000-2017	-0.079	-0.066	-0.053
Kenya	Budalangi	2000	0.252	0.353	0.474
Kenya	Budalangi	2017	0.150	0.200	0.258
Kenya	Budalangi	2000-2017	-0.052	-0.035	-0.017
Kenya	Bumula	2000	0.134	0.172	0.225
Kenya	Bumula	2017	0.095	0.126	0.164
Kenya	Bumula	2000-2017	-0.039	-0.026	-0.013
Kenya	Bura	2000	0.244	0.315	0.399
Kenya	Bura	2017	0.149	0.192	0.244
Kenya	Bura	2000-2017	-0.038	-0.027	-0.017
Kenya	Bureti	2000	0.064	0.083	0.108
Kenya	Bureti	2017	0.077	0.101	0.135
Kenya	Bureti	2000-2017	-0.002	0.011	0.025
Kenya	Butere	2000	0.374	0.499	0.647
Kenya	Butere	2017	0.141	0.181	0.244
Kenya	Butere	2000-2017	-0.079	-0.064	-0.048

Table 1: LRI DALYs rate by unit *(continued)*

Country	Unit	year	mean rate	lower rate	upper rate
Kenya	Butula	2000	0.260	0.350	0.467
Kenya	Butula	2017	0.150	0.195	0.251
Kenya	Butula	2000-2017	-0.049	-0.035	-0.022
Kenya	Buuri	2000	0.089	0.118	0.151
Kenya	Buuri	2017	0.158	0.210	0.280
Kenya	Buuri	2000-2017	0.023	0.035	0.044
Kenya	Central Imenti	2000	0.090	0.117	0.147
Kenya	Central Imenti	2017	0.152	0.204	0.267
Kenya	Central Imenti	2000-2017	0.023	0.034	0.047
Kenya	Changamwe	2000	0.129	0.187	0.260
Kenya	Changamwe	2017	0.105	0.152	0.222
Kenya	Changamwe	2000-2017	-0.042	-0.012	0.022
Kenya	Chepalungu	2000	0.045	0.058	0.075
Kenya	Chepalungu	2017	0.031	0.042	0.055
Kenya	Chepalungu	2000-2017	-0.030	-0.019	-0.008
Kenya	Cherangany	2000	0.081	0.106	0.136
Kenya	Cherangany	2017	0.066	0.087	0.114
Kenya	Cherangany	2000-2017	-0.021	-0.010	0.001
Kenya	Chesumei	2000	0.072	0.095	0.124
Kenya	Chesumei	2017	0.048	0.065	0.085
Kenya	Chesumei	2000-2017	-0.036	-0.023	-0.011
Kenya	Chuka/Igambang'Ombe	2000	0.526	0.678	0.865
Kenya	Chuka/Igambang'Ombe	2017	0.191	0.252	0.327
Kenya	Chuka/Igambang'Ombe	2000-2017	-0.071	-0.058	-0.045
Kenya	Daadab	2000	0.221	0.298	0.397
Kenya	Daadab	2017	0.053	0.070	0.091
Kenya	Daadab	2000-2017	-0.101	-0.089	-0.077
Kenya	Dagoretti North	2000	0.121	0.163	0.209
Kenya	Dagoretti North	2017	0.044	0.062	0.090
Kenya	Dagoretti North	2000-2017	-0.088	-0.058	-0.030
Kenya	Dagoretti South	2000	0.118	0.166	0.221
Kenya	Dagoretti South	2017	0.045	0.063	0.088
Kenya	Dagoretti South	2000-2017	-0.085	-0.059	-0.027
Kenya	Eldama Ravine	2000	0.332	0.441	0.574
Kenya	Eldama Ravine	2017	0.088	0.119	0.159
Kenya	Eldama Ravine	2000-2017	-0.093	-0.079	-0.067
Kenya	Eldas	2000	0.351	0.465	0.594
Kenya	Eldas	2017	0.105	0.137	0.175
Kenya	Eldas	2000-2017	-0.082	-0.073	-0.064
Kenya	Embakasi Central	2000	0.110	0.167	0.250
Kenya	Embakasi Central	2017	0.041	0.064	0.093
Kenya	Embakasi Central	2000-2017	-0.093	-0.057	-0.015
Kenya	Embakasi East	2000	0.113	0.166	0.225
Kenya	Embakasi East	2017	0.045	0.062	0.085
Kenya	Embakasi East	2000-2017	-0.083	-0.059	-0.036
Kenya	Embakasi North	2000	0.114	0.166	0.234
Kenya	Embakasi North	2017	0.041	0.063	0.087
Kenya	Embakasi North	2000-2017	-0.089	-0.057	-0.024
Kenya	Embakasi South	2000	0.118	0.169	0.235
Kenya	Embakasi South	2017	0.045	0.063	0.091
Kenya	Embakasi South	2000-2017	-0.094	-0.060	-0.028
Kenya	Embakasi West	2000	0.110	0.169	0.253
Kenya	Embakasi West	2017	0.041	0.064	0.099
Kenya	Embakasi West	2000-2017	-0.097	-0.057	-0.013
Kenya	Emgwen	2000	0.073	0.098	0.127
Kenya	Emgwen	2017	0.051	0.069	0.092
Kenya	Emgwen	2000-2017	-0.036	-0.021	-0.008
Kenya	Emuhaya	2000	0.226	0.311	0.428
Kenya	Emuhaya	2017	0.049	0.067	0.088
Kenya	Emuhaya	2000-2017	-0.112	-0.091	-0.071
Kenya	Emurua Dikirr	2000	0.126	0.165	0.219

Table 1: LRI DALYs rate by unit *(continued)*

Country	Unit	year	mean rate	lower rate	upper rate
Kenya	Emurua Dikirr	2017	0.034	0.045	0.059
Kenya	Emurua Dikirr	2000-2017	-0.094	-0.081	-0.069
Kenya	Endebess	2000	0.094	0.119	0.152
Kenya	Endebess	2017	0.071	0.092	0.120
Kenya	Endebess	2000-2017	-0.026	-0.014	-0.001
Kenya	Fafi	2000	0.226	0.301	0.397
Kenya	Fafi	2017	0.055	0.073	0.096
Kenya	Fafi	2000-2017	-0.099	-0.086	-0.071
Kenya	Funyula	2000	0.270	0.357	0.452
Kenya	Funyula	2017	0.156	0.200	0.255
Kenya	Funyula	2000-2017	-0.049	-0.036	-0.021
Kenya	Galole	2000	0.240	0.319	0.404
Kenya	Galole	2017	0.151	0.200	0.263
Kenya	Galole	2000-2017	-0.044	-0.031	-0.020
Kenya	Ganze	2000	0.282	0.367	0.469
Kenya	Ganze	2017	0.102	0.132	0.173
Kenya	Ganze	2000-2017	-0.070	-0.061	-0.052
Kenya	Garissa Township	2000	0.208	0.292	0.407
Kenya	Garissa Township	2017	0.053	0.074	0.103
Kenya	Garissa Township	2000-2017	-0.112	-0.082	-0.052
Kenya	Garsen	2000	0.264	0.342	0.436
Kenya	Garsen	2017	0.160	0.205	0.271
Kenya	Garsen	2000-2017	-0.045	-0.037	-0.028
Kenya	Gatanga	2000	0.064	0.085	0.108
Kenya	Gatanga	2017	0.112	0.146	0.190
Kenya	Gatanga	2000-2017	0.020	0.032	0.045
Kenya	Gatundu North	2000	0.072	0.095	0.122
Kenya	Gatundu North	2017	0.105	0.137	0.179
Kenya	Gatundu North	2000-2017	0.004	0.018	0.033
Kenya	Gatundu South	2000	0.072	0.098	0.127
Kenya	Gatundu South	2017	0.107	0.142	0.183
Kenya	Gatundu South	2000-2017	0.003	0.018	0.034
Kenya	Gem	2000	0.316	0.419	0.537
Kenya	Gem	2017	0.144	0.188	0.245
Kenya	Gem	2000-2017	-0.064	-0.050	-0.039
Kenya	Gichugu	2000	0.132	0.172	0.229
Kenya	Gichugu	2017	0.126	0.167	0.219
Kenya	Gichugu	2000-2017	-0.017	0.001	0.017
Kenya	Gilgil	2000	0.095	0.123	0.160
Kenya	Gilgil	2017	0.107	0.139	0.182
Kenya	Gilgil	2000-2017	-0.006	0.005	0.016
Kenya	Githunguri	2000	0.073	0.099	0.128
Kenya	Githunguri	2017	0.107	0.143	0.189
Kenya	Githunguri	2000-2017	0.003	0.018	0.034
Kenya	Hamisi	2000	0.228	0.310	0.409
Kenya	Hamisi	2017	0.052	0.068	0.089
Kenya	Hamisi	2000-2017	-0.102	-0.089	-0.073
Kenya	Homa Bay Town	2000	0.563	0.767	0.995
Kenya	Homa Bay Town	2017	0.224	0.298	0.380
Kenya	Homa Bay Town	2000-2017	-0.073	-0.057	-0.041
Kenya	Igembe Central	2000	0.087	0.115	0.149
Kenya	Igembe Central	2017	0.150	0.202	0.265
Kenya	Igembe Central	2000-2017	0.018	0.035	0.053
Kenya	Igembe North	2000	0.084	0.111	0.145
Kenya	Igembe North	2017	0.144	0.197	0.256
Kenya	Igembe North	2000-2017	0.022	0.036	0.052
Kenya	Igembe South	2000	0.531	0.693	0.876
Kenya	Igembe South	2000	0.079	0.105	0.141
Kenya	Igembe South	2017	0.195	0.260	0.343
Kenya	Igembe South	2017	0.144	0.188	0.241
Kenya	Igembe South	2000-2017	-0.075	-0.058	-0.042

Table 1: LRI DALYs rate by unit *(continued)*

Country	Unit	year	mean rate	lower rate	upper rate
Kenya	Igembe South	2000-2017	0.018	0.037	0.054
Kenya	Ijara	2000	0.248	0.320	0.409
Kenya	Ijara	2017	0.060	0.077	0.100
Kenya	Ijara	2000-2017	-0.097	-0.090	-0.081
Kenya	Ikolomani	2000	0.389	0.516	0.662
Kenya	Ikolomani	2017	0.142	0.184	0.243
Kenya	Ikolomani	2000-2017	-0.076	-0.062	-0.046
Kenya	Isiolo North	2000	0.617	0.816	1.068
Kenya	Isiolo North	2017	0.209	0.276	0.361
Kenya	Isiolo North	2000-2017	-0.077	-0.064	-0.051
Kenya	Isiolo South	2000	0.627	0.828	1.069
Kenya	Isiolo South	2017	0.207	0.272	0.343
Kenya	Isiolo South	2000-2017	-0.074	-0.066	-0.058
Kenya	Jomvu	2000	0.135	0.185	0.249
Kenya	Jomvu	2017	0.111	0.151	0.207
Kenya	Jomvu	2000-2017	-0.034	-0.012	0.012
Kenya	Juja	2000	0.071	0.092	0.120
Kenya	Juja	2017	0.099	0.131	0.175
Kenya	Juja	2000-2017	0.002	0.018	0.033
Kenya	Kabete	2000	0.074	0.100	0.132
Kenya	Kabete	2017	0.107	0.144	0.188
Kenya	Kabete	2000-2017	-0.001	0.019	0.047
Kenya	Kabondo Kasipul	2000	0.655	0.872	1.147
Kenya	Kabondo Kasipul	2017	0.241	0.316	0.401
Kenya	Kabondo Kasipul	2000-2017	-0.075	-0.061	-0.047
Kenya	Kabuchai	2000	0.140	0.187	0.239
Kenya	Kabuchai	2017	0.102	0.135	0.179
Kenya	Kabuchai	2000-2017	-0.038	-0.024	-0.009
Kenya	Kacheliba	2000	0.198	0.260	0.336
Kenya	Kacheliba	2017	0.085	0.109	0.139
Kenya	Kacheliba	2000-2017	-0.055	-0.046	-0.039
Kenya	Kaiti	2000	0.143	0.188	0.243
Kenya	Kaiti	2017	0.018	0.023	0.031
Kenya	Kaiti	2000-2017	-0.140	-0.126	-0.112
Kenya	Kajiado Central	2000	0.087	0.115	0.149
Kenya	Kajiado Central	2017	0.044	0.058	0.076
Kenya	Kajiado Central	2000-2017	-0.056	-0.044	-0.033
Kenya	Kajiado East	2000	0.095	0.126	0.166
Kenya	Kajiado East	2017	0.047	0.062	0.082
Kenya	Kajiado East	2000-2017	-0.061	-0.046	-0.034
Kenya	Kajiado North	2000	0.091	0.122	0.158
Kenya	Kajiado North	2017	0.042	0.060	0.080
Kenya	Kajiado North	2000-2017	-0.065	-0.046	-0.025
Kenya	Kajiado South	2000	0.094	0.119	0.154
Kenya	Kajiado South	2017	0.044	0.058	0.076
Kenya	Kajiado South	2000-2017	-0.056	-0.047	-0.036
Kenya	Kajiado West	2000	0.092	0.122	0.157
Kenya	Kajiado West	2017	0.045	0.059	0.077
Kenya	Kajiado West	2000-2017	-0.059	-0.047	-0.038
Kenya	Kaloleni	2000	0.273	0.361	0.471
Kenya	Kaloleni	2017	0.099	0.130	0.167
Kenya	Kaloleni	2000-2017	-0.070	-0.057	-0.045
Kenya	Kamukunji	2000	0.115	0.171	0.249
Kenya	Kamukunji	2017	0.041	0.065	0.097
Kenya	Kamukunji	2000-2017	-0.098	-0.059	-0.022
Kenya	Kandara	2000	0.065	0.085	0.109
Kenya	Kandara	2017	0.112	0.146	0.193
Kenya	Kandara	2000-2017	0.015	0.032	0.047
Kenya	Kanduyi	2000	0.134	0.174	0.226
Kenya	Kanduyi	2017	0.095	0.126	0.165
Kenya	Kanduyi	2000-2017	-0.038	-0.024	-0.011

Table 1: LRI DALYs rate by unit *(continued)*

Country	Unit	year	mean rate	lower rate	upper rate
Kenya	Kangema	2000	0.063	0.084	0.110
Kenya	Kangema	2017	0.111	0.147	0.199
Kenya	Kangema	2000-2017	0.017	0.033	0.049
Kenya	Kangundo	2000	0.091	0.115	0.148
Kenya	Kangundo	2017	0.103	0.138	0.187
Kenya	Kangundo	2000-2017	-0.007	0.011	0.030
Kenya	Kapenguria	2000	0.200	0.257	0.331
Kenya	Kapenguria	2017	0.086	0.114	0.147
Kenya	Kapenguria	2000-2017	-0.055	-0.044	-0.032
Kenya	Kapseret	2000	0.091	0.125	0.169
Kenya	Kapseret	2017	0.066	0.091	0.121
Kenya	Kapseret	2000-2017	-0.033	-0.016	0.001
Kenya	Karachuonyo	2000	0.601	0.798	1.031
Kenya	Karachuonyo	2017	0.230	0.299	0.391
Kenya	Karachuonyo	2000-2017	-0.072	-0.060	-0.046
Kenya	Kasarani	2000	0.118	0.164	0.225
Kenya	Kasarani	2017	0.045	0.062	0.083
Kenya	Kasarani	2000-2017	-0.081	-0.058	-0.036
Kenya	Kasipul	2000	0.596	0.800	1.019
Kenya	Kasipul	2017	0.241	0.311	0.396
Kenya	Kasipul	2000-2017	-0.074	-0.058	-0.041
Kenya	Kathiani	2000	0.089	0.115	0.154
Kenya	Kathiani	2017	0.102	0.137	0.184
Kenya	Kathiani	2000-2017	-0.012	0.010	0.029
Kenya	Keiyo North	2000	0.126	0.165	0.214
Kenya	Keiyo North	2017	0.079	0.104	0.138
Kenya	Keiyo North	2000-2017	-0.042	-0.030	-0.018
Kenya	Keiyo South	2000	0.131	0.172	0.225
Kenya	Keiyo South	2017	0.084	0.109	0.141
Kenya	Keiyo South	2000-2017	-0.039	-0.029	-0.018
Kenya	Kesses	2000	0.094	0.126	0.166
Kenya	Kesses	2017	0.065	0.087	0.116
Kenya	Kesses	2000-2017	-0.032	-0.019	-0.004
Kenya	Khwisero	2000	0.372	0.498	0.651
Kenya	Khwisero	2017	0.135	0.179	0.244
Kenya	Khwisero	2000-2017	-0.083	-0.065	-0.048
Kenya	Kiambaa	2000	0.075	0.099	0.133
Kenya	Kiambaa	2017	0.107	0.145	0.199
Kenya	Kiambaa	2000-2017	-0.004	0.020	0.042
Kenya	Kiambu	2000	0.067	0.091	0.120
Kenya	Kiambu	2017	0.099	0.134	0.179
Kenya	Kiambu	2000-2017	-0.002	0.021	0.043
Kenya	Kibra	2000	0.111	0.162	0.221
Kenya	Kibra	2017	0.044	0.063	0.093
Kenya	Kibra	2000-2017	-0.086	-0.057	-0.029
Kenya	Kibwezi East	2000	0.133	0.171	0.223
Kenya	Kibwezi East	2017	0.017	0.023	0.030
Kenya	Kibwezi East	2000-2017	-0.134	-0.121	-0.111
Kenya	Kibwezi West	2000	0.130	0.169	0.215
Kenya	Kibwezi West	2017	0.017	0.023	0.030
Kenya	Kibwezi West	2000-2017	-0.133	-0.121	-0.109
Kenya	Kieni	2000	0.086	0.111	0.144
Kenya	Kieni	2017	0.146	0.194	0.257
Kenya	Kieni	2000-2017	0.025	0.035	0.047
Kenya	Kigumo	2000	0.063	0.084	0.111
Kenya	Kigumo	2017	0.114	0.145	0.194
Kenya	Kigumo	2000-2017	0.018	0.032	0.046
Kenya	Kiharu	2000	0.063	0.082	0.108
Kenya	Kiharu	2017	0.108	0.143	0.186
Kenya	Kiharu	2000-2017	0.020	0.032	0.044
Kenya	Kikuyu	2000	0.075	0.100	0.131

Table 1: LRI DALYs rate by unit *(continued)*

Country	Unit	year	mean rate	lower rate	upper rate
Kenya	Kikuyu	2017	0.110	0.146	0.191
Kenya	Kikuyu	2000-2017	0.001	0.020	0.040
Kenya	Kilgoris	2000	0.125	0.164	0.214
Kenya	Kilgoris	2017	0.036	0.047	0.059
Kenya	Kilgoris	2000-2017	-0.088	-0.079	-0.071
Kenya	Kilifi North	2000	0.279	0.361	0.459
Kenya	Kilifi North	2017	0.102	0.133	0.173
Kenya	Kilifi North	2000-2017	-0.073	-0.060	-0.047
Kenya	Kilifi South	2000	0.265	0.356	0.460
Kenya	Kilifi South	2017	0.100	0.129	0.172
Kenya	Kilifi South	2000-2017	-0.073	-0.059	-0.045
Kenya	Kilome	2000	0.133	0.173	0.224
Kenya	Kilome	2017	0.018	0.023	0.031
Kenya	Kilome	2000-2017	-0.134	-0.120	-0.108
Kenya	Kimilili	2000	0.131	0.177	0.226
Kenya	Kimilili	2017	0.100	0.135	0.181
Kenya	Kimilili	2000-2017	-0.037	-0.018	0.001
Kenya	Kiminini	2000	0.079	0.106	0.135
Kenya	Kiminini	2017	0.065	0.087	0.113
Kenya	Kiminini	2000-2017	-0.023	-0.010	0.003
Kenya	Kinango	2000	0.202	0.262	0.336
Kenya	Kinango	2017	0.062	0.082	0.107
Kenya	Kinango	2000-2017	-0.078	-0.069	-0.061
Kenya	Kinangop	2000	0.069	0.091	0.117
Kenya	Kinangop	2017	0.133	0.179	0.238
Kenya	Kinangop	2000-2017	0.026	0.038	0.051
Kenya	Kipipiri	2000	0.068	0.091	0.117
Kenya	Kipipiri	2017	0.136	0.177	0.231
Kenya	Kipipiri	2000-2017	0.026	0.038	0.049
Kenya	Kipkelion East	2000	0.063	0.082	0.109
Kenya	Kipkelion East	2017	0.068	0.090	0.119
Kenya	Kipkelion East	2000-2017	-0.007	0.006	0.017
Kenya	Kipkelion West	2000	0.063	0.083	0.108
Kenya	Kipkelion West	2017	0.071	0.095	0.126
Kenya	Kipkelion West	2000-2017	-0.005	0.007	0.020
Kenya	Kirinyaga Central	2000	0.125	0.165	0.215
Kenya	Kirinyaga Central	2017	0.120	0.161	0.211
Kenya	Kirinyaga Central	2000-2017	-0.016	0.002	0.022
Kenya	Kisauni	2000	0.139	0.185	0.243
Kenya	Kisauni	2017	0.112	0.149	0.199
Kenya	Kisauni	2000-2017	-0.033	-0.012	0.009
Kenya	Kisumu Central	2000	0.275	0.400	0.569
Kenya	Kisumu Central	2017	0.119	0.178	0.262
Kenya	Kisumu Central	2000-2017	-0.088	-0.054	-0.024
Kenya	Kisumu East	2000	0.301	0.406	0.529
Kenya	Kisumu East	2017	0.133	0.182	0.250
Kenya	Kisumu East	2000-2017	-0.072	-0.052	-0.034
Kenya	Kisumu West	2000	0.307	0.415	0.546
Kenya	Kisumu West	2017	0.139	0.184	0.239
Kenya	Kisumu West	2000-2017	-0.065	-0.052	-0.039
Kenya	Kitui Central	2000	0.136	0.177	0.231
Kenya	Kitui Central	2017	0.048	0.065	0.087
Kenya	Kitui Central	2000-2017	-0.076	-0.061	-0.045
Kenya	Kitui East	2000	0.137	0.179	0.230
Kenya	Kitui East	2017	0.051	0.067	0.090
Kenya	Kitui East	2000-2017	-0.071	-0.060	-0.051
Kenya	Kitui Rural	2000	0.136	0.177	0.228
Kenya	Kitui Rural	2017	0.052	0.068	0.091
Kenya	Kitui Rural	2000-2017	-0.072	-0.060	-0.050
Kenya	Kitui South	2000	0.133	0.172	0.219
Kenya	Kitui South	2017	0.053	0.070	0.092

Table 1: LRI DALYs rate by unit *(continued)*

Country	Unit	year	mean rate	lower rate	upper rate
Kenya	Kitui South	2000-2017	-0.069	-0.058	-0.049
Kenya	Kitui West	2000	0.136	0.179	0.235
Kenya	Kitui West	2017	0.050	0.067	0.088
Kenya	Kitui West	2000-2017	-0.073	-0.060	-0.048
Kenya	Kitutu Chache North	2000	0.225	0.301	0.395
Kenya	Kitutu Chache North	2017	0.103	0.138	0.183
Kenya	Kitutu Chache North	2000-2017	-0.062	-0.044	-0.025
Kenya	Kitutu Chache South	2000	0.246	0.332	0.442
Kenya	Kitutu Chache South	2017	0.102	0.139	0.188
Kenya	Kitutu Chache South	2000-2017	-0.072	-0.049	-0.029
Kenya	Kitutu Masaba	2000	0.166	0.217	0.279
Kenya	Kitutu Masaba	2017	0.056	0.072	0.095
Kenya	Kitutu Masaba	2000-2017	-0.079	-0.064	-0.051
Kenya	Konoin	2000	0.043	0.056	0.073
Kenya	Konoin	2017	0.034	0.044	0.058
Kenya	Konoin	2000-2017	-0.027	-0.015	-0.004
Kenya	Kuresoi North	2000	0.093	0.122	0.157
Kenya	Kuresoi North	2017	0.099	0.135	0.180
Kenya	Kuresoi North	2000-2017	-0.006	0.005	0.017
Kenya	Kuresoi South	2000	0.098	0.130	0.168
Kenya	Kuresoi South	2017	0.107	0.143	0.193
Kenya	Kuresoi South	2000-2017	-0.009	0.005	0.017
Kenya	Kuria East	2000	0.804	1.064	1.385
Kenya	Kuria East	2017	0.224	0.289	0.374
Kenya	Kuria East	2000-2017	-0.092	-0.079	-0.065
Kenya	Kuria West	2000	0.810	1.069	1.399
Kenya	Kuria West	2017	0.226	0.296	0.385
Kenya	Kuria West	2000-2017	-0.092	-0.077	-0.062
Kenya	Kwanza	2000	0.080	0.105	0.135
Kenya	Kwanza	2017	0.066	0.086	0.110
Kenya	Kwanza	2000-2017	-0.023	-0.010	0.003
Kenya	Lafey	2000	0.234	0.314	0.404
Kenya	Lafey	2017	0.083	0.109	0.142
Kenya	Lafey	2000-2017	-0.075	-0.065	-0.055
Kenya	Lagdera	2000	0.221	0.292	0.379
Kenya	Lagdera	2017	0.053	0.069	0.088
Kenya	Lagdera	2000-2017	-0.091	-0.082	-0.073
Kenya	Laikipia East	2000	0.115	0.152	0.199
Kenya	Laikipia East	2017	0.035	0.047	0.062
Kenya	Laikipia East	2000-2017	-0.088	-0.073	-0.059
Kenya	Laikipia North	2000	0.117	0.152	0.196
Kenya	Laikipia North	2017	0.036	0.047	0.062
Kenya	Laikipia North	2000-2017	-0.084	-0.074	-0.065
Kenya	Laikipia West	2000	0.117	0.154	0.201
Kenya	Laikipia West	2017	0.036	0.047	0.063
Kenya	Laikipia West	2000-2017	-0.084	-0.075	-0.065
Kenya	Laisamis	2000	0.208	0.277	0.356
Kenya	Laisamis	2017	0.112	0.148	0.188
Kenya	Laisamis	2000-2017	-0.044	-0.036	-0.029
Kenya	Lamu East	2000	0.344	0.451	0.596
Kenya	Lamu East	2017	0.198	0.253	0.336
Kenya	Lamu East	2000-2017	-0.044	-0.032	-0.023
Kenya	Lamu West	2000	0.342	0.443	0.563
Kenya	Lamu West	2017	0.189	0.250	0.333
Kenya	Lamu West	2000-2017	-0.046	-0.032	-0.020
Kenya	Langata	2000	0.122	0.166	0.221
Kenya	Langata	2017	0.047	0.063	0.085
Kenya	Langata	2000-2017	-0.078	-0.058	-0.038
Kenya	Lari	2000	0.075	0.099	0.130
Kenya	Lari	2017	0.107	0.141	0.186
Kenya	Lari	2000-2017	0.005	0.018	0.030

Table 1: LRI DALYs rate by unit *(continued)*

Country	Unit	year	mean rate	lower rate	upper rate
Kenya	Likoni	2000	0.136	0.183	0.248
Kenya	Likoni	2017	0.108	0.152	0.213
Kenya	Likoni	2000-2017	-0.034	-0.011	0.013
Kenya	Likuyani	2000	0.125	0.166	0.213
Kenya	Likuyani	2017	0.102	0.136	0.179
Kenya	Likuyani	2000-2017	-0.030	-0.018	-0.003
Kenya	Limuru	2000	0.074	0.098	0.129
Kenya	Limuru	2017	0.107	0.140	0.180
Kenya	Limuru	2000-2017	0.003	0.019	0.033
Kenya	Loima	2000	0.353	0.461	0.595
Kenya	Loima	2017	0.102	0.134	0.173
Kenya	Loima	2000-2017	-0.078	-0.072	-0.066
Kenya	Luanda	2000	0.235	0.313	0.427
Kenya	Luanda	2017	0.049	0.067	0.089
Kenya	Luanda	2000-2017	-0.113	-0.093	-0.074
Kenya	Lugari	2000	0.372	0.489	0.634
Kenya	Lugari	2000	0.123	0.164	0.214
Kenya	Lugari	2017	0.107	0.138	0.182
Kenya	Lugari	2017	0.138	0.189	0.254
Kenya	Lugari	2000-2017	-0.077	-0.059	-0.041
Kenya	Lugari	2000-2017	-0.031	-0.017	-0.003
Kenya	Lungalunga	2000	0.199	0.260	0.337
Kenya	Lungalunga	2017	0.064	0.083	0.108
Kenya	Lungalunga	2000-2017	-0.078	-0.069	-0.061
Kenya	Lurambi	2000	0.395	0.516	0.661
Kenya	Lurambi	2017	0.140	0.190	0.254
Kenya	Lurambi	2000-2017	-0.078	-0.061	-0.042
Kenya	Maara	2000	0.515	0.686	0.884
Kenya	Maara	2017	0.189	0.256	0.346
Kenya	Maara	2000-2017	-0.072	-0.058	-0.045
Kenya	Machakos Town	2000	0.086	0.113	0.148
Kenya	Machakos Town	2017	0.101	0.135	0.183
Kenya	Machakos Town	2000-2017	-0.003	0.012	0.028
Kenya	Magarini	2000	0.282	0.370	0.472
Kenya	Magarini	2017	0.104	0.135	0.177
Kenya	Magarini	2000-2017	-0.071	-0.064	-0.055
Kenya	Makadara	2000	0.124	0.170	0.232
Kenya	Makadara	2017	0.045	0.064	0.088
Kenya	Makadara	2000-2017	-0.085	-0.059	-0.035
Kenya	Makueni	2000	0.132	0.171	0.219
Kenya	Makueni	2017	0.017	0.022	0.029
Kenya	Makueni	2000-2017	-0.135	-0.124	-0.112
Kenya	Malava	2000	0.392	0.508	0.666
Kenya	Malava	2017	0.150	0.192	0.253
Kenya	Malava	2000-2017	-0.072	-0.059	-0.048
Kenya	Malindi	2000	0.262	0.357	0.501
Kenya	Malindi	2017	0.093	0.130	0.174
Kenya	Malindi	2000-2017	-0.082	-0.062	-0.039
Kenya	Mandera East	2000	0.246	0.325	0.425
Kenya	Mandera East	2017	0.082	0.109	0.143
Kenya	Mandera East	2000-2017	-0.084	-0.066	-0.051
Kenya	Mandera North	2000	0.238	0.316	0.408
Kenya	Mandera North	2017	0.081	0.109	0.143
Kenya	Mandera North	2000-2017	-0.076	-0.065	-0.055
Kenya	Mandera South	2000	0.229	0.310	0.400
Kenya	Mandera South	2017	0.079	0.106	0.139
Kenya	Mandera South	2000-2017	-0.078	-0.066	-0.054
Kenya	Mandera West	2000	0.241	0.320	0.416
Kenya	Mandera West	2017	0.082	0.110	0.143
Kenya	Mandera West	2000-2017	-0.079	-0.067	-0.057
Kenya	Manyatta	2000	0.107	0.142	0.185

Table 1: LRI DALYs rate by unit *(continued)*

Country	Unit	year	mean rate	lower rate	upper rate
Kenya	Manyatta	2017	0.107	0.140	0.185
Kenya	Manyatta	2000-2017	-0.017	-0.002	0.012
Kenya	Maragwa	2000	0.062	0.081	0.104
Kenya	Maragwa	2017	0.110	0.141	0.186
Kenya	Maragwa	2000-2017	0.020	0.032	0.044
Kenya	Marakwet East	2000	0.123	0.165	0.215
Kenya	Marakwet East	2017	0.076	0.099	0.129
Kenya	Marakwet East	2000-2017	-0.041	-0.031	-0.021
Kenya	Marakwet West	2000	0.127	0.164	0.215
Kenya	Marakwet West	2017	0.076	0.103	0.135
Kenya	Marakwet West	2000-2017	-0.041	-0.029	-0.018
Kenya	Masinga	2000	0.076	0.100	0.127
Kenya	Masinga	2017	0.095	0.124	0.160
Kenya	Masinga	2000-2017	0.001	0.012	0.023
Kenya	Matayos	2000	0.349	0.453	0.583
Kenya	Matayos	2017	0.174	0.224	0.287
Kenya	Matayos	2000-2017	-0.060	-0.042	-0.023
Kenya	Mathare	2000	0.117	0.171	0.241
Kenya	Mathare	2017	0.042	0.065	0.096
Kenya	Mathare	2000-2017	-0.095	-0.059	-0.028
Kenya	Mathioya	2000	0.065	0.085	0.109
Kenya	Mathioya	2017	0.111	0.147	0.193
Kenya	Mathioya	2000-2017	0.016	0.032	0.049
Kenya	Mathira	2000	0.083	0.109	0.140
Kenya	Mathira	2017	0.144	0.191	0.254
Kenya	Mathira	2000-2017	0.021	0.037	0.051
Kenya	Matuga	2000	0.200	0.262	0.336
Kenya	Matuga	2017	0.063	0.082	0.107
Kenya	Matuga	2000-2017	-0.082	-0.071	-0.062
Kenya	Matungu	2000	0.375	0.495	0.635
Kenya	Matungu	2017	0.138	0.178	0.231
Kenya	Matungu	2000-2017	-0.079	-0.065	-0.053
Kenya	Matungulu	2000	0.084	0.107	0.139
Kenya	Matungulu	2017	0.097	0.128	0.169
Kenya	Matungulu	2000-2017	-0.004	0.010	0.023
Kenya	Mavoko	2000	0.083	0.112	0.146
Kenya	Mavoko	2017	0.100	0.135	0.182
Kenya	Mavoko	2000-2017	-0.006	0.011	0.026
Kenya	Mbeere North	2000	0.107	0.139	0.181
Kenya	Mbeere North	2017	0.106	0.139	0.184
Kenya	Mbeere North	2000-2017	-0.014	-0.002	0.010
Kenya	Mbeere South	2000	0.105	0.138	0.178
Kenya	Mbeere South	2017	0.108	0.139	0.181
Kenya	Mbeere South	2000-2017	-0.014	-0.002	0.009
Kenya	Mbita	2000	0.567	0.732	0.942
Kenya	Mbita	2017	0.221	0.286	0.361
Kenya	Mbita	2000-2017	-0.071	-0.058	-0.046
Kenya	Mbooni	2000	0.142	0.183	0.235
Kenya	Mbooni	2017	0.017	0.023	0.031
Kenya	Mbooni	2000-2017	-0.138	-0.125	-0.112
Kenya	Mogotio	2000	0.333	0.435	0.571
Kenya	Mogotio	2017	0.090	0.118	0.154
Kenya	Mogotio	2000-2017	-0.090	-0.080	-0.071
Kenya	Moiben	2000	0.095	0.125	0.165
Kenya	Moiben	2017	0.064	0.084	0.112
Kenya	Moiben	2000-2017	-0.033	-0.021	-0.011
Kenya	Molo	2000	0.092	0.125	0.160
Kenya	Molo	2017	0.102	0.137	0.183
Kenya	Molo	2000-2017	-0.012	0.004	0.020
Kenya	Mosop	2000	0.073	0.096	0.124
Kenya	Mosop	2017	0.052	0.069	0.091

Table 1: LRI DALYs rate by unit *(continued)*

Country	Unit	year	mean rate	lower rate	upper rate
Kenya	Mosop	2000-2017	-0.032	-0.020	-0.009
Kenya	Moyale	2000	0.209	0.281	0.359
Kenya	Moyale	2017	0.120	0.158	0.211
Kenya	Moyale	2000-2017	-0.051	-0.038	-0.024
Kenya	Msambweni	2000	0.199	0.263	0.349
Kenya	Msambweni	2017	0.059	0.080	0.111
Kenya	Msambweni	2000-2017	-0.085	-0.071	-0.053
Kenya	Mt. Elgon	2000	0.135	0.176	0.225
Kenya	Mt. Elgon	2017	0.104	0.138	0.178
Kenya	Mt. Elgon	2000-2017	-0.034	-0.019	-0.006
Kenya	Muhoroni	2000	0.315	0.420	0.531
Kenya	Muhoroni	2017	0.146	0.191	0.249
Kenya	Muhoroni	2000-2017	-0.059	-0.047	-0.038
Kenya	Mukurweini	2000	0.080	0.106	0.140
Kenya	Mukurweini	2017	0.143	0.186	0.244
Kenya	Mukurweini	2000-2017	0.020	0.037	0.054
Kenya	Mumias East	2000	0.375	0.498	0.653
Kenya	Mumias East	2017	0.138	0.180	0.239
Kenya	Mumias East	2000-2017	-0.076	-0.062	-0.046
Kenya	Mumias West	2000	0.374	0.503	0.663
Kenya	Mumias West	2017	0.140	0.183	0.240
Kenya	Mumias West	2000-2017	-0.083	-0.065	-0.047
Kenya	Mvita	2000	0.137	0.185	0.247
Kenya	Mvita	2017	0.111	0.152	0.215
Kenya	Mvita	2000-2017	-0.037	-0.011	0.015
Kenya	Mwala	2000	0.079	0.103	0.132
Kenya	Mwala	2017	0.097	0.126	0.167
Kenya	Mwala	2000-2017	-0.001	0.012	0.022
Kenya	Mwatate	2000	0.182	0.243	0.316
Kenya	Mwatate	2017	0.096	0.127	0.165
Kenya	Mwatate	2000-2017	-0.054	-0.041	-0.029
Kenya	Mwea	2000	0.121	0.162	0.207
Kenya	Mwea	2017	0.120	0.157	0.208
Kenya	Mwea	2000-2017	-0.013	0.001	0.015
Kenya	Mwingi Central	2000	0.121	0.159	0.205
Kenya	Mwingi Central	2017	0.051	0.067	0.088
Kenya	Mwingi Central	2000-2017	-0.066	-0.053	-0.041
Kenya	Mwingi North	2000	0.118	0.155	0.197
Kenya	Mwingi North	2017	0.051	0.067	0.087
Kenya	Mwingi North	2000-2017	-0.061	-0.050	-0.040
Kenya	Mwingi West	2000	0.130	0.171	0.223
Kenya	Mwingi West	2017	0.052	0.068	0.091
Kenya	Mwingi West	2000-2017	-0.072	-0.058	-0.046
Kenya	Naivasha	2000	0.092	0.120	0.153
Kenya	Naivasha	2017	0.102	0.136	0.182
Kenya	Naivasha	2000-2017	-0.007	0.006	0.019
Kenya	Nakuru Town East	2000	0.087	0.118	0.155
Kenya	Nakuru Town East	2017	0.097	0.131	0.180
Kenya	Nakuru Town East	2000-2017	-0.013	0.007	0.028
Kenya	Nakuru Town West	2000	0.078	0.117	0.175
Kenya	Nakuru Town West	2017	0.088	0.132	0.203
Kenya	Nakuru Town West	2000-2017	-0.025	0.008	0.045
Kenya	Nambale	2000	0.257	0.349	0.459
Kenya	Nambale	2017	0.143	0.193	0.250
Kenya	Nambale	2000-2017	-0.049	-0.036	-0.022
Kenya	Nandi Hills	2000	0.071	0.097	0.128
Kenya	Nandi Hills	2017	0.053	0.068	0.091
Kenya	Nandi Hills	2000-2017	-0.036	-0.021	-0.009
Kenya	Narok East	2000	0.124	0.160	0.214
Kenya	Narok East	2017	0.034	0.045	0.059
Kenya	Narok East	2000-2017	-0.091	-0.080	-0.070

Table 1: LRI DALYs rate by unit *(continued)*

Country	Unit	year	mean rate	lower rate	upper rate
Kenya	Narok North	2000	0.126	0.162	0.209
Kenya	Narok North	2017	0.032	0.043	0.057
Kenya	Narok North	2000-2017	-0.092	-0.083	-0.072
Kenya	Narok South	2000	0.118	0.154	0.199
Kenya	Narok South	2017	0.032	0.042	0.055
Kenya	Narok South	2000-2017	-0.091	-0.081	-0.071
Kenya	Narok West	2000	0.117	0.156	0.200
Kenya	Narok West	2017	0.032	0.043	0.056
Kenya	Narok West	2000-2017	-0.090	-0.081	-0.072
Kenya	Navakholo	2000	0.380	0.504	0.651
Kenya	Navakholo	2017	0.136	0.182	0.242
Kenya	Navakholo	2000-2017	-0.074	-0.061	-0.046
Kenya	Ndaragwa	2000	0.074	0.098	0.126
Kenya	Ndaragwa	2017	0.140	0.191	0.254
Kenya	Ndaragwa	2000-2017	0.025	0.038	0.050
Kenya	Ndhiwa	2000	0.572	0.756	0.962
Kenya	Ndhiwa	2017	0.230	0.300	0.385
Kenya	Ndhiwa	2000-2017	-0.067	-0.055	-0.045
Kenya	Ndia	2000	0.128	0.167	0.222
Kenya	Ndia	2017	0.124	0.160	0.207
Kenya	Ndia	2000-2017	-0.014	0.000	0.014
Kenya	Njoro	2000	0.093	0.123	0.159
Kenya	Njoro	2017	0.102	0.136	0.181
Kenya	Njoro	2000-2017	-0.009	0.005	0.016
Kenya	North Horr	2000	0.206	0.275	0.358
Kenya	North Horr	2017	0.114	0.149	0.192
Kenya	North Horr	2000-2017	-0.043	-0.034	-0.027
Kenya	North Imenti	2000	0.087	0.116	0.153
Kenya	North Imenti	2017	0.145	0.203	0.272
Kenya	North Imenti	2000-2017	0.019	0.035	0.051
Kenya	North Mugirango	2000	0.172	0.235	0.319
Kenya	North Mugirango	2017	0.054	0.072	0.093
Kenya	North Mugirango	2000-2017	-0.083	-0.067	-0.052
Kenya	Nyakach	2000	0.324	0.428	0.558
Kenya	Nyakach	2017	0.147	0.194	0.257
Kenya	Nyakach	2000-2017	-0.060	-0.047	-0.034
Kenya	Nyali	2000	0.133	0.185	0.252
Kenya	Nyali	2017	0.107	0.150	0.200
Kenya	Nyali	2000-2017	-0.042	-0.012	0.015
Kenya	Nyando	2000	0.311	0.409	0.529
Kenya	Nyando	2017	0.140	0.184	0.240
Kenya	Nyando	2000-2017	-0.059	-0.047	-0.034
Kenya	Nyaribari Chache	2000	0.219	0.303	0.401
Kenya	Nyaribari Chache	2017	0.099	0.134	0.181
Kenya	Nyaribari Chache	2000-2017	-0.071	-0.049	-0.029
Kenya	Nyaribari Masaba	2000	0.232	0.300	0.388
Kenya	Nyaribari Masaba	2017	0.103	0.137	0.182
Kenya	Nyaribari Masaba	2000-2017	-0.063	-0.049	-0.033
Kenya	Nyatike	2000	0.710	0.937	1.208
Kenya	Nyatike	2017	0.215	0.283	0.363
Kenya	Nyatike	2000-2017	-0.082	-0.071	-0.061
Kenya	Nyeri Town	2000	0.079	0.107	0.143
Kenya	Nyeri Town	2017	0.134	0.184	0.256
Kenya	Nyeri Town	2000-2017	0.016	0.037	0.057
Kenya	Ol Jorok	2000	0.067	0.091	0.119
Kenya	Ol Jorok	2017	0.131	0.178	0.244
Kenya	Ol Jorok	2000-2017	0.024	0.038	0.053
Kenya	Ol Kalou	2000	0.071	0.092	0.119
Kenya	Ol Kalou	2017	0.130	0.177	0.236
Kenya	Ol Kalou	2000-2017	0.028	0.038	0.049
Kenya	Othaya	2000	0.085	0.108	0.139

Table 1: LRI DALYs rate by unit *(continued)*

Country	Unit	year	mean rate	lower rate	upper rate
Kenya	Othaya	2017	0.142	0.188	0.247
Kenya	Othaya	2000-2017	0.021	0.036	0.055
Kenya	Pokot South	2000	0.199	0.260	0.334
Kenya	Pokot South	2017	0.080	0.107	0.135
Kenya	Pokot South	2000-2017	-0.058	-0.048	-0.039
Kenya	Rabai	2000	0.270	0.362	0.471
Kenya	Rabai	2017	0.101	0.131	0.176
Kenya	Rabai	2000-2017	-0.075	-0.058	-0.043
Kenya	Rangwe	2000	0.582	0.765	1.004
Kenya	Rangwe	2017	0.224	0.300	0.387
Kenya	Rangwe	2000-2017	-0.071	-0.057	-0.042
Kenya	Rarieda	2000	0.313	0.411	0.528
Kenya	Rarieda	2017	0.152	0.196	0.251
Kenya	Rarieda	2000-2017	-0.059	-0.045	-0.033
Kenya	Rongai	2000	0.094	0.124	0.160
Kenya	Rongai	2017	0.104	0.140	0.185
Kenya	Rongai	2000-2017	-0.006	0.006	0.015
Kenya	Rongo	2000	0.818	1.083	1.428
Kenya	Rongo	2017	0.238	0.310	0.413
Kenya	Rongo	2000-2017	-0.089	-0.073	-0.059
Kenya	Roysambu	2000	0.123	0.160	0.213
Kenya	Roysambu	2017	0.044	0.061	0.083
Kenya	Roysambu	2000-2017	-0.081	-0.059	-0.034
Kenya	Ruaraka	2000	0.116	0.165	0.231
Kenya	Ruaraka	2017	0.041	0.063	0.085
Kenya	Ruaraka	2000-2017	-0.091	-0.058	-0.028
Kenya	Ruiru	2000	0.066	0.090	0.121
Kenya	Ruiru	2017	0.097	0.131	0.178
Kenya	Ruiru	2000-2017	-0.003	0.020	0.040
Kenya	Runyenjes	2000	0.104	0.139	0.180
Kenya	Runyenjes	2017	0.106	0.138	0.181
Kenya	Runyenjes	2000-2017	-0.018	-0.001	0.015
Kenya	Sabatia	2000	0.221	0.312	0.415
Kenya	Sabatia	2017	0.052	0.068	0.090
Kenya	Sabatia	2000-2017	-0.109	-0.089	-0.071
Kenya	Saboti	2000	0.078	0.105	0.141
Kenya	Saboti	2017	0.063	0.088	0.119
Kenya	Saboti	2000-2017	-0.030	-0.009	0.014
Kenya	Saku	2000	0.218	0.289	0.380
Kenya	Saku	2017	0.118	0.159	0.209
Kenya	Saku	2000-2017	-0.057	-0.038	-0.023
Kenya	Samburu East	2000	0.180	0.244	0.316
Kenya	Samburu East	2017	0.068	0.089	0.115
Kenya	Samburu East	2000-2017	-0.072	-0.062	-0.053
Kenya	Samburu North	2000	0.184	0.250	0.327
Kenya	Samburu North	2017	0.067	0.090	0.115
Kenya	Samburu North	2000-2017	-0.071	-0.061	-0.052
Kenya	Samburu West	2000	0.199	0.266	0.345
Kenya	Samburu West	2017	0.074	0.099	0.133
Kenya	Samburu West	2000-2017	-0.075	-0.063	-0.051
Kenya	Seme	2000	0.305	0.409	0.530
Kenya	Seme	2017	0.141	0.185	0.233
Kenya	Seme	2000-2017	-0.063	-0.049	-0.036
Kenya	Shinyalu	2000	0.375	0.510	0.659
Kenya	Shinyalu	2017	0.143	0.191	0.253
Kenya	Shinyalu	2000-2017	-0.072	-0.059	-0.043
Kenya	Sigor	2000	0.194	0.259	0.335
Kenya	Sigor	2017	0.078	0.101	0.131
Kenya	Sigor	2000-2017	-0.060	-0.051	-0.042
Kenya	Sigowet/Soin	2000	0.065	0.084	0.108
Kenya	Sigowet/Soin	2017	0.075	0.099	0.128

Table 1: LRI DALYs rate by unit *(continued)*

Country	Unit	year	mean rate	lower rate	upper rate
Kenya	Sigowet/Soin	2000-2017	-0.003	0.010	0.023
Kenya	Sirisia	2000	0.139	0.180	0.233
Kenya	Sirisia	2017	0.102	0.134	0.174
Kenya	Sirisia	2000-2017	-0.040	-0.024	-0.009
Kenya	Sotik	2000	0.043	0.056	0.073
Kenya	Sotik	2017	0.033	0.044	0.058
Kenya	Sotik	2000-2017	-0.029	-0.015	-0.004
Kenya	South Imenti	2000	0.084	0.110	0.142
Kenya	South Imenti	2017	0.148	0.199	0.263
Kenya	South Imenti	2000-2017	0.025	0.038	0.051
Kenya	South Mugirango	2000	0.240	0.316	0.419
Kenya	South Mugirango	2017	0.104	0.140	0.185
Kenya	South Mugirango	2000-2017	-0.063	-0.047	-0.031
Kenya	Soy	2000	0.095	0.127	0.165
Kenya	Soy	2017	0.069	0.092	0.119
Kenya	Soy	2000-2017	-0.028	-0.017	-0.006
Kenya	Starehe	2000	0.121	0.171	0.233
Kenya	Starehe	2017	0.046	0.064	0.087
Kenya	Starehe	2000-2017	-0.085	-0.059	-0.035
Kenya	Suba	2000	0.582	0.776	1.000
Kenya	Suba	2017	0.225	0.298	0.382
Kenya	Suba	2000-2017	-0.069	-0.058	-0.047
Kenya	Subukia	2000	0.092	0.122	0.161
Kenya	Subukia	2017	0.103	0.139	0.189
Kenya	Subukia	2000-2017	-0.008	0.006	0.020
Kenya	Suna East	2000	0.738	1.021	1.364
Kenya	Suna East	2017	0.221	0.307	0.401
Kenya	Suna East	2000-2017	-0.092	-0.074	-0.055
Kenya	Suna West	2000	0.757	1.003	1.307
Kenya	Suna West	2017	0.219	0.296	0.386
Kenya	Suna West	2000-2017	-0.089	-0.075	-0.061
Kenya	Tarbaj	2000	0.358	0.468	0.604
Kenya	Tarbaj	2017	0.104	0.136	0.174
Kenya	Tarbaj	2000-2017	-0.081	-0.073	-0.063
Kenya	Taveta	2000	0.191	0.249	0.321
Kenya	Taveta	2017	0.094	0.124	0.166
Kenya	Taveta	2000-2017	-0.061	-0.044	-0.029
Kenya	Teso North	2000	0.333	0.427	0.544
Kenya	Teso North	2017	0.170	0.221	0.283
Kenya	Teso North	2000-2017	-0.056	-0.042	-0.026
Kenya	Teso South	2000	0.295	0.381	0.474
Kenya	Teso South	2017	0.158	0.202	0.258
Kenya	Teso South	2000-2017	-0.052	-0.038	-0.024
Kenya	Tetu	2000	0.081	0.110	0.144
Kenya	Tetu	2017	0.147	0.191	0.260
Kenya	Tetu	2000-2017	0.021	0.036	0.053
Kenya	Tharaka	2000	0.524	0.699	0.901
Kenya	Tharaka	2017	0.197	0.258	0.333
Kenya	Tharaka	2000-2017	-0.070	-0.059	-0.048
Kenya	Thika Town	2000	0.066	0.088	0.117
Kenya	Thika Town	2017	0.087	0.127	0.170
Kenya	Thika Town	2000-2017	-0.004	0.019	0.040
Kenya	Tiaty	2000	0.292	0.390	0.507
Kenya	Tiaty	2017	0.079	0.103	0.131
Kenya	Tiaty	2000-2017	-0.089	-0.079	-0.072
Kenya	Tigania East	2000	0.082	0.108	0.141
Kenya	Tigania East	2017	0.148	0.195	0.250
Kenya	Tigania East	2000-2017	0.024	0.037	0.050
Kenya	Tigania West	2000	0.087	0.114	0.145
Kenya	Tigania West	2017	0.149	0.202	0.262
Kenya	Tigania West	2000-2017	0.020	0.035	0.051

Table 1: LRI DALYs rate by unit *(continued)*

Country	Unit	year	mean rate	lower rate	upper rate
Kenya	Tinderet	2000	0.077	0.101	0.130
Kenya	Tinderet	2017	0.053	0.070	0.092
Kenya	Tinderet	2000-2017	-0.032	-0.022	-0.010
Kenya	Tongaren	2000	0.127	0.168	0.214
Kenya	Tongaren	2017	0.103	0.137	0.182
Kenya	Tongaren	2000-2017	-0.031	-0.018	-0.006
Kenya	Turbo	2000	0.095	0.127	0.165
Kenya	Turbo	2017	0.069	0.093	0.123
Kenya	Turbo	2000-2017	-0.031	-0.016	-0.002
Kenya	Turkana Central	2000	0.334	0.447	0.580
Kenya	Turkana Central	2017	0.093	0.128	0.171
Kenya	Turkana Central	2000-2017	-0.084	-0.074	-0.063
Kenya	Turkana East	2000	0.318	0.426	0.540
Kenya	Turkana East	2017	0.096	0.130	0.169
Kenya	Turkana East	2000-2017	-0.077	-0.069	-0.061
Kenya	Turkana North	2000	0.353	0.461	0.589
Kenya	Turkana North	2017	0.108	0.142	0.179
Kenya	Turkana North	2000-2017	-0.076	-0.068	-0.062
Kenya	Turkana South	2000	0.333	0.441	0.573
Kenya	Turkana South	2017	0.093	0.128	0.166
Kenya	Turkana South	2000-2017	-0.079	-0.072	-0.065
Kenya	Turkana West	2000	0.352	0.466	0.597
Kenya	Turkana West	2017	0.114	0.148	0.189
Kenya	Turkana West	2000-2017	-0.076	-0.068	-0.061
Kenya	Ugenya	2000	0.311	0.411	0.545
Kenya	Ugenya	2017	0.142	0.187	0.240
Kenya	Ugenya	2000-2017	-0.064	-0.049	-0.038
Kenya	Ugunja	2000	0.316	0.417	0.543
Kenya	Ugunja	2017	0.137	0.186	0.245
Kenya	Ugunja	2000-2017	-0.066	-0.051	-0.038
Kenya	Uriri	2000	0.809	1.049	1.360
Kenya	Uriri	2017	0.244	0.317	0.401
Kenya	Uriri	2000-2017	-0.084	-0.073	-0.060
Kenya	Vihiga	2000	0.222	0.306	0.405
Kenya	Vihiga	2017	0.049	0.067	0.088
Kenya	Vihiga	2000-2017	-0.110	-0.090	-0.071
Kenya	Voi	2000	0.183	0.243	0.319
Kenya	Voi	2017	0.098	0.128	0.170
Kenya	Voi	2000-2017	-0.054	-0.039	-0.024
Kenya	Wajir East	2000	0.348	0.465	0.615
Kenya	Wajir East	2017	0.102	0.137	0.188
Kenya	Wajir East	2000-2017	-0.093	-0.075	-0.059
Kenya	Wajir North	2000	0.351	0.475	0.614
Kenya	Wajir North	2017	0.107	0.140	0.178
Kenya	Wajir North	2000-2017	-0.085	-0.074	-0.063
Kenya	Wajir South	2000	0.355	0.469	0.612
Kenya	Wajir South	2017	0.107	0.138	0.176
Kenya	Wajir South	2000-2017	-0.083	-0.076	-0.069
Kenya	Wajir West	2000	0.340	0.452	0.592
Kenya	Wajir West	2017	0.106	0.137	0.173
Kenya	Wajir West	2000-2017	-0.080	-0.071	-0.061
Kenya	Webute West	2000	0.143	0.189	0.247
Kenya	Webute West	2017	0.101	0.136	0.178
Kenya	Webute West	2000-2017	-0.035	-0.022	-0.008
Kenya	Webuye East	2000	0.138	0.189	0.243
Kenya	Webuye East	2017	0.101	0.138	0.181
Kenya	Webuye East	2000-2017	-0.037	-0.021	-0.006
Kenya	West Mugirango	2000	0.159	0.220	0.292
Kenya	West Mugirango	2017	0.055	0.072	0.095
Kenya	West Mugirango	2000-2017	-0.080	-0.064	-0.047
Kenya	Westlands	2000	0.123	0.163	0.214

Table 1: LRI DALYs rate by unit *(continued)*

Country	Unit	year	mean rate	lower rate	upper rate
Kenya	Westlands	2017	0.046	0.062	0.083
Kenya	Westlands	2000-2017	-0.080	-0.059	-0.040
Kenya	Wundanyi	2000	0.188	0.256	0.353
Kenya	Wundanyi	2017	0.101	0.134	0.180
Kenya	Wundanyi	2000-2017	-0.060	-0.041	-0.022
Kenya	Yatta	2000	0.079	0.102	0.131
Kenya	Yatta	2017	0.095	0.124	0.165
Kenya	Yatta	2000-2017	-0.002	0.011	0.021
Lesotho	Berea	2000	0.148	0.211	0.291
Lesotho	Berea	2017	0.111	0.163	0.234
Lesotho	Berea	2000-2017	-0.029	-0.013	0.003
Lesotho	Butha-Buthe	2000	0.148	0.213	0.298
Lesotho	Butha-Buthe	2017	0.110	0.158	0.225
Lesotho	Butha-Buthe	2000-2017	-0.030	-0.016	-0.002
Lesotho	Leribe	2000	0.150	0.215	0.299
Lesotho	Leribe	2017	0.112	0.163	0.237
Lesotho	Leribe	2000-2017	-0.028	-0.015	0.000
Lesotho	Mafeteng	2000	0.139	0.199	0.274
Lesotho	Mafeteng	2017	0.109	0.153	0.221
Lesotho	Mafeteng	2000-2017	-0.027	-0.013	0.000
Lesotho	Maseru	2000	0.139	0.198	0.274
Lesotho	Maseru	2017	0.103	0.153	0.217
Lesotho	Maseru	2000-2017	-0.028	-0.013	0.002
Lesotho	Mohale's Hoek	2000	0.141	0.198	0.282
Lesotho	Mohale's Hoek	2017	0.106	0.152	0.215
Lesotho	Mohale's Hoek	2000-2017	-0.027	-0.014	0.001
Lesotho	Mokhotlong	2000	0.168	0.248	0.348
Lesotho	Mokhotlong	2017	0.099	0.142	0.205
Lesotho	Mokhotlong	2000-2017	-0.043	-0.029	-0.013
Lesotho	Qacha's Nek	2000	0.151	0.214	0.299
Lesotho	Qacha's Nek	2017	0.103	0.146	0.210
Lesotho	Qacha's Nek	2000-2017	-0.033	-0.020	-0.004
Lesotho	Quthing	2000	0.143	0.204	0.285
Lesotho	Quthing	2017	0.105	0.150	0.215
Lesotho	Quthing	2000-2017	-0.027	-0.016	-0.003
Lesotho	Thaba-Tseka	2000	0.167	0.243	0.341
Lesotho	Thaba-Tseka	2017	0.102	0.145	0.205
Lesotho	Thaba-Tseka	2000-2017	-0.042	-0.028	-0.011
Liberia	Barrobo	2000	0.391	0.633	1.007
Liberia	Barrobo	2017	0.082	0.137	0.218
Liberia	Barrobo	2000-2017	-0.116	-0.086	-0.057
Liberia	Belleh	2000	0.267	0.427	0.673
Liberia	Belleh	2017	0.078	0.134	0.204
Liberia	Belleh	2000-2017	-0.092	-0.067	-0.041
Liberia	Bokomu	2000	0.269	0.424	0.667
Liberia	Bokomu	2017	0.077	0.133	0.207
Liberia	Bokomu	2000-2017	-0.099	-0.071	-0.046
Liberia	Bopolu	2000	0.307	0.484	0.785
Liberia	Bopolu	2017	0.078	0.134	0.210
Liberia	Bopolu	2000-2017	-0.101	-0.076	-0.050
Liberia	Buah	2000	0.388	0.629	1.004
Liberia	Buah	2017	0.080	0.138	0.219
Liberia	Buah	2000-2017	-0.114	-0.085	-0.056
Liberia	Butaw	2000	0.370	0.596	0.923
Liberia	Butaw	2017	0.084	0.141	0.222
Liberia	Butaw	2000-2017	-0.109	-0.080	-0.052
Liberia	Careysburg	2000	0.326	0.545	0.838
Liberia	Careysburg	2017	0.066	0.117	0.182
Liberia	Careysburg	2000-2017	-0.116	-0.086	-0.055
Liberia	Commnwealth	2000	0.286	0.498	0.794
Liberia	Commnwealth	2017	0.079	0.136	0.219

Table 1: LRI DALYs rate by unit *(continued)*

Country	Unit	year	mean rate	lower rate	upper rate
Liberia	Commnwealth	2000-2017	-0.112	-0.076	-0.045
Liberia	District # 1	2000	0.355	0.573	0.852
Liberia	District # 1	2017	0.084	0.145	0.228
Liberia	District # 1	2000-2017	-0.107	-0.077	-0.047
Liberia	District # 2	2000	0.328	0.535	0.785
Liberia	District # 2	2017	0.086	0.148	0.236
Liberia	District # 2	2000-2017	-0.099	-0.071	-0.044
Liberia	District # 3	2000	0.337	0.561	0.853
Liberia	District # 3	2017	0.086	0.145	0.229
Liberia	District # 3	2000-2017	-0.104	-0.075	-0.044
Liberia	District # 4	2000	0.359	0.584	0.861
Liberia	District # 4	2017	0.092	0.159	0.249
Liberia	District # 4	2000-2017	-0.101	-0.073	-0.044
Liberia	Dugbe River	2000	0.366	0.579	0.936
Liberia	Dugbe River	2017	0.080	0.139	0.231
Liberia	Dugbe River	2000-2017	-0.112	-0.080	-0.052
Liberia	Firestone	2000	0.384	0.612	0.915
Liberia	Firestone	2017	0.078	0.133	0.222
Liberia	Firestone	2000-2017	-0.115	-0.083	-0.057
Liberia	Foya	2000	0.265	0.423	0.661
Liberia	Foya	2017	0.081	0.133	0.212
Liberia	Foya	2000-2017	-0.096	-0.070	-0.043
Liberia	Fuamah	2000	0.328	0.526	0.819
Liberia	Fuamah	2017	0.078	0.132	0.209
Liberia	Fuamah	2000-2017	-0.108	-0.078	-0.053
Liberia	Garwula	2000	0.348	0.580	0.896
Liberia	Garwula	2017	0.076	0.133	0.210
Liberia	Garwula	2000-2017	-0.111	-0.083	-0.056
Liberia	Gbarma	2000	0.330	0.530	0.823
Liberia	Gbarma	2017	0.081	0.134	0.211
Liberia	Gbarma	2000-2017	-0.107	-0.080	-0.052
Liberia	Gbarzon	2000	0.234	0.390	0.600
Liberia	Gbarzon	2017	0.069	0.117	0.185
Liberia	Gbarzon	2000-2017	-0.104	-0.076	-0.048
Liberia	Gbeapo	2000	0.368	0.589	0.931
Liberia	Gbeapo	2017	0.082	0.138	0.219
Liberia	Gbeapo	2000-2017	-0.109	-0.082	-0.054
Liberia	Gbehlageh	2000	0.257	0.420	0.679
Liberia	Gbehlageh	2017	0.074	0.128	0.197
Liberia	Gbehlageh	2000-2017	-0.096	-0.069	-0.043
Liberia	Gibi	2000	0.332	0.534	0.812
Liberia	Gibi	2017	0.079	0.133	0.206
Liberia	Gibi	2000-2017	-0.106	-0.078	-0.052
Liberia	Golakonneh	2000	0.337	0.542	0.835
Liberia	Golakonneh	2017	0.078	0.133	0.215
Liberia	Golakonneh	2000-2017	-0.105	-0.079	-0.052
Liberia	Greater Monrovia	2000	0.235	0.399	0.611
Liberia	Greater Monrovia	2017	0.063	0.112	0.183
Liberia	Greater Monrovia	2000-2017	-0.104	-0.070	-0.039
Liberia	Greenville	2000	0.370	0.615	0.975
Liberia	Greenville	2017	0.081	0.140	0.224
Liberia	Greenville	2000-2017	-0.117	-0.083	-0.049
Liberia	Jaedae Jaedepo	2000	0.382	0.605	0.965
Liberia	Jaedae Jaedepo	2017	0.085	0.144	0.230
Liberia	Jaedae Jaedepo	2000-2017	-0.110	-0.081	-0.053
Liberia	Jorquelleh	2000	0.265	0.423	0.654
Liberia	Jorquelleh	2017	0.078	0.132	0.200
Liberia	Jorquelleh	2000-2017	-0.098	-0.070	-0.044
Liberia	Juarzon	2000	0.379	0.611	0.965
Liberia	Juarzon	2017	0.082	0.142	0.223
Liberia	Juarzon	2000-2017	-0.112	-0.081	-0.052

Table 1: LRI DALYs rate by unit *(continued)*

Country	Unit	year	mean rate	lower rate	upper rate
Liberia	Kakata	2000	0.363	0.577	0.880
Liberia	Kakata	2017	0.074	0.132	0.208
Liberia	Kakata	2000-2017	-0.108	-0.082	-0.055
Liberia	Klay	2000	0.348	0.563	0.871
Liberia	Klay	2017	0.076	0.130	0.204
Liberia	Klay	2000-2017	-0.110	-0.083	-0.055
Liberia	Kokoyah	2000	0.284	0.460	0.709
Liberia	Kokoyah	2017	0.078	0.133	0.203
Liberia	Kokoyah	2000-2017	-0.099	-0.070	-0.044
Liberia	Kolahun	2000	0.273	0.435	0.684
Liberia	Kolahun	2017	0.083	0.136	0.216
Liberia	Kolahun	2000-2017	-0.096	-0.070	-0.044
Liberia	Kongba	2000	0.286	0.431	0.638
Liberia	Kongba	2017	0.084	0.135	0.201
Liberia	Kongba	2000-2017	-0.092	-0.067	-0.041
Liberia	Konobo	2000	0.282	0.445	0.690
Liberia	Konobo	2017	0.070	0.116	0.177
Liberia	Konobo	2000-2017	-0.107	-0.080	-0.053
Liberia	Kpayan	2000	0.374	0.608	0.939
Liberia	Kpayan	2017	0.083	0.140	0.224
Liberia	Kpayan	2000-2017	-0.113	-0.083	-0.057
Liberia	Lower Kru Coast	2000	0.271	0.431	0.656
Liberia	Lower Kru Coast	2000	0.374	0.611	1.012
Liberia	Lower Kru Coast	2017	0.078	0.137	0.213
Liberia	Lower Kru Coast	2017	0.080	0.130	0.203
Liberia	Lower Kru Coast	2000-2017	-0.113	-0.084	-0.052
Liberia	Lower Kru Coast	2000-2017	-0.098	-0.070	-0.040
Liberia	Mambah-Kaba	2000	0.336	0.555	0.868
Liberia	Mambah-Kaba	2017	0.068	0.124	0.196
Liberia	Mambah-Kaba	2000-2017	-0.113	-0.081	-0.051
Liberia	Mecca	2000	0.341	0.573	0.899
Liberia	Mecca	2017	0.076	0.131	0.208
Liberia	Mecca	2000-2017	-0.112	-0.083	-0.054
Liberia	Morweh	2000	0.345	0.554	0.832
Liberia	Morweh	2017	0.082	0.138	0.213
Liberia	Morweh	2000-2017	-0.104	-0.076	-0.048
Liberia	Owensgrove	2000	0.356	0.587	0.902
Liberia	Owensgrove	2017	0.080	0.136	0.220
Liberia	Owensgrove	2000-2017	-0.112	-0.082	-0.053
Liberia	Panta-Kpa	2000	0.262	0.423	0.683
Liberia	Panta-Kpa	2017	0.077	0.133	0.201
Liberia	Panta-Kpa	2000-2017	-0.095	-0.068	-0.040
Liberia	Pleebo/Sodeken	2000	0.346	0.565	0.945
Liberia	Pleebo/Sodeken	2017	0.077	0.135	0.213
Liberia	Pleebo/Sodeken	2000-2017	-0.112	-0.082	-0.054
Liberia	Porkpa	2000	0.296	0.472	0.719
Liberia	Porkpa	2017	0.075	0.131	0.209
Liberia	Porkpa	2000-2017	-0.099	-0.074	-0.047
Liberia	Pyneston	2000	0.363	0.584	0.906
Liberia	Pyneston	2017	0.082	0.139	0.214
Liberia	Pyneston	2000-2017	-0.109	-0.082	-0.051
Liberia	Saclepea	2000	0.263	0.426	0.687
Liberia	Saclepea	2017	0.076	0.133	0.204
Liberia	Saclepea	2000-2017	-0.097	-0.072	-0.044
Liberia	Salala	2000	0.326	0.520	0.782
Liberia	Salala	2017	0.077	0.133	0.208
Liberia	Salala	2000-2017	-0.105	-0.078	-0.051
Liberia	Salayea	2000	0.267	0.421	0.655
Liberia	Salayea	2017	0.076	0.129	0.201
Liberia	Salayea	2000-2017	-0.096	-0.070	-0.046
Liberia	Sanayea	2000	0.264	0.426	0.680

Table 1: LRI DALYs rate by unit *(continued)*

Country	Unit	year	mean rate	lower rate	upper rate
Liberia	Sanayea	2017	0.079	0.133	0.207
Liberia	Sanayea	2000-2017	-0.100	-0.072	-0.046
Liberia	Sanniquelleh-Mahn	2000	0.260	0.420	0.690
Liberia	Sanniquelleh-Mahn	2017	0.075	0.129	0.191
Liberia	Sanniquelleh-Mahn	2000-2017	-0.098	-0.069	-0.044
Liberia	Sasstown	2000	0.386	0.614	1.018
Liberia	Sasstown	2017	0.080	0.139	0.220
Liberia	Sasstown	2000-2017	-0.113	-0.083	-0.052
Liberia	St Paul River	2000	0.276	0.470	0.719
Liberia	St Paul River	2017	0.062	0.110	0.180
Liberia	St Paul River	2000-2017	-0.116	-0.081	-0.049
Liberia	Stjohnriver	2000	0.358	0.568	0.844
Liberia	Stjohnriver	2017	0.088	0.152	0.240
Liberia	Stjohnriver	2000-2017	-0.099	-0.073	-0.044
Liberia	Suakoko	2000	0.258	0.425	0.670
Liberia	Suakoko	2017	0.081	0.134	0.208
Liberia	Suakoko	2000-2017	-0.100	-0.070	-0.044
Liberia	Tappita	2000	0.256	0.425	0.663
Liberia	Tappita	2017	0.076	0.131	0.204
Liberia	Tappita	2000-2017	-0.099	-0.071	-0.043
Liberia	Tchien	2000	0.259	0.415	0.661
Liberia	Tchien	2017	0.071	0.118	0.185
Liberia	Tchien	2000-2017	-0.106	-0.077	-0.050
Liberia	Tewor	2000	0.320	0.505	0.800
Liberia	Tewor	2017	0.076	0.130	0.198
Liberia	Tewor	2000-2017	-0.106	-0.078	-0.051
Liberia	Timbo	2000	0.361	0.590	0.905
Liberia	Timbo	2017	0.087	0.146	0.230
Liberia	Timbo	2000-2017	-0.105	-0.077	-0.048
Liberia	Todee	2000	0.331	0.537	0.827
Liberia	Todee	2017	0.068	0.120	0.183
Liberia	Todee	2000-2017	-0.114	-0.087	-0.059
Liberia	Upperkrucoast	2000	0.379	0.620	1.002
Liberia	Upperkrucoast	2017	0.085	0.147	0.235
Liberia	Upperkrucoast	2000-2017	-0.111	-0.080	-0.054
Liberia	Voinjama	2000	0.272	0.432	0.677
Liberia	Voinjama	2017	0.076	0.135	0.211
Liberia	Voinjama	2000-2017	-0.097	-0.069	-0.041
Liberia	Webbo	2000	0.338	0.537	0.870
Liberia	Webbo	2017	0.079	0.131	0.209
Liberia	Webbo	2000-2017	-0.110	-0.082	-0.053
Liberia	Yarwein-Mehnsohnne	2000	0.260	0.426	0.674
Liberia	Yarwein-Mehnsohnne	2017	0.077	0.134	0.206
Liberia	Yarwein-Mehnsohnne	2000-2017	-0.100	-0.074	-0.045
Liberia	Zoegeh	2000	0.259	0.416	0.651
Liberia	Zoegeh	2017	0.075	0.128	0.196
Liberia	Zoegeh	2000-2017	-0.093	-0.068	-0.041
Liberia	Zorzor	2000	0.267	0.427	0.685
Liberia	Zorzor	2017	0.076	0.133	0.207
Liberia	Zorzor	2000-2017	-0.095	-0.069	-0.042
Liberia	Zota	2000	0.263	0.421	0.664
Liberia	Zota	2017	0.078	0.133	0.201
Liberia	Zota	2000-2017	-0.095	-0.066	-0.038
Madagascar	Alaotra-Mangoro	2000	0.279	0.395	0.559
Madagascar	Alaotra-Mangoro	2017	0.156	0.222	0.314
Madagascar	Alaotra-Mangoro	2000-2017	-0.039	-0.035	-0.030
Madagascar	Amoron'i mania	2000	0.267	0.370	0.530
Madagascar	Amoron'i mania	2017	0.175	0.246	0.355
Madagascar	Amoron'i mania	2000-2017	-0.032	-0.025	-0.020
Madagascar	Analamanga	2000	0.256	0.366	0.519
Madagascar	Analamanga	2017	0.175	0.248	0.346

Table 1: LRI DALYs rate by unit *(continued)*

Country	Unit	year	mean rate	lower rate	upper rate
Madagascar	Analamanga	2000-2017	-0.034	-0.026	-0.018
Madagascar	Analanjirofo	2000	0.277	0.392	0.548
Madagascar	Analanjirofo	2017	0.157	0.222	0.316
Madagascar	Analanjirofo	2000-2017	-0.040	-0.034	-0.029
Madagascar	Androy	2000	0.344	0.483	0.672
Madagascar	Androy	2017	0.211	0.295	0.418
Madagascar	Androy	2000-2017	-0.034	-0.030	-0.025
Madagascar	Anosy	2000	0.273	0.385	0.550
Madagascar	Anosy	2017	0.157	0.225	0.314
Madagascar	Anosy	2000-2017	-0.036	-0.032	-0.028
Madagascar	Atsimo-Andrefana	2000	0.267	0.372	0.526
Madagascar	Atsimo-Andrefana	2017	0.149	0.213	0.302
Madagascar	Atsimo-Andrefana	2000-2017	-0.040	-0.036	-0.033
Madagascar	Atsimo-Atsinana	2000	0.282	0.400	0.565
Madagascar	Atsimo-Atsinana	2017	0.165	0.232	0.324
Madagascar	Atsimo-Atsinana	2000-2017	-0.037	-0.032	-0.027
Madagascar	Atsinanana	2000	0.268	0.385	0.544
Madagascar	Atsinanana	2017	0.156	0.221	0.311
Madagascar	Atsinanana	2000-2017	-0.039	-0.034	-0.029
Madagascar	Betsiboka	2000	0.253	0.360	0.516
Madagascar	Betsiboka	2017	0.144	0.206	0.292
Madagascar	Betsiboka	2000-2017	-0.040	-0.036	-0.032
Madagascar	Boeny	2000	0.256	0.366	0.512
Madagascar	Boeny	2017	0.150	0.211	0.291
Madagascar	Boeny	2000-2017	-0.042	-0.035	-0.029
Madagascar	Bongolava	2000	0.274	0.381	0.539
Madagascar	Bongolava	2017	0.146	0.206	0.297
Madagascar	Bongolava	2000-2017	-0.042	-0.037	-0.033
Madagascar	Diana	2000	0.263	0.374	0.533
Madagascar	Diana	2017	0.154	0.221	0.312
Madagascar	Diana	2000-2017	-0.039	-0.033	-0.027
Madagascar	Haute matsiatra	2000	0.265	0.371	0.530
Madagascar	Haute matsiatra	2017	0.175	0.246	0.348
Madagascar	Haute matsiatra	2000-2017	-0.032	-0.025	-0.020
Madagascar	Ihorombe	2000	0.273	0.380	0.527
Madagascar	Ihorombe	2017	0.146	0.209	0.293
Madagascar	Ihorombe	2000-2017	-0.042	-0.036	-0.032
Madagascar	Itasy	2000	0.293	0.418	0.593
Madagascar	Itasy	2017	0.176	0.247	0.347
Madagascar	Itasy	2000-2017	-0.035	-0.030	-0.025
Madagascar	Melaky	2000	0.260	0.375	0.532
Madagascar	Melaky	2017	0.149	0.214	0.297
Madagascar	Melaky	2000-2017	-0.037	-0.033	-0.029
Madagascar	Menabe	2000	0.255	0.361	0.508
Madagascar	Menabe	2017	0.146	0.207	0.296
Madagascar	Menabe	2000-2017	-0.038	-0.034	-0.030
Madagascar	Sava	2000	0.259	0.373	0.535
Madagascar	Sava	2017	0.153	0.220	0.307
Madagascar	Sava	2000-2017	-0.038	-0.033	-0.027
Madagascar	Sofia	2000	0.291	0.413	0.572
Madagascar	Sofia	2017	0.164	0.235	0.327
Madagascar	Sofia	2000-2017	-0.038	-0.035	-0.031
Madagascar	Vakinankaratra	2000	0.281	0.400	0.563
Madagascar	Vakinankaratra	2017	0.179	0.251	0.349
Madagascar	Vakinankaratra	2000-2017	-0.031	-0.026	-0.022
Madagascar	Vatovavy Fitovinany	2000	0.288	0.402	0.567
Madagascar	Vatovavy Fitovinany	2017	0.168	0.233	0.329
Madagascar	Vatovavy Fitovinany	2000-2017	-0.038	-0.032	-0.028
Malawi	Balaka	2000	0.255	0.335	0.422
Malawi	Balaka	2017	0.091	0.122	0.158
Malawi	Balaka	2000-2017	-0.068	-0.058	-0.049

Table 1: LRI DALYs rate by unit *(continued)*

Country	Unit	year	mean rate	lower rate	upper rate
Malawi	Blantyre	2000	0.241	0.318	0.402
Malawi	Blantyre	2017	0.096	0.128	0.163
Malawi	Blantyre	2000-2017	-0.065	-0.054	-0.043
Malawi	Chikwawa	2000	0.238	0.312	0.396
Malawi	Chikwawa	2017	0.102	0.132	0.174
Malawi	Chikwawa	2000-2017	-0.059	-0.051	-0.043
Malawi	Chiradzulu	2000	0.264	0.348	0.437
Malawi	Chiradzulu	2017	0.106	0.138	0.180
Malawi	Chiradzulu	2000-2017	-0.066	-0.056	-0.046
Malawi	Chitipa	2000	0.239	0.305	0.387
Malawi	Chitipa	2017	0.098	0.129	0.166
Malawi	Chitipa	2000-2017	-0.060	-0.050	-0.040
Malawi	Dedza	2000	0.331	0.429	0.540
Malawi	Dedza	2017	0.100	0.132	0.172
Malawi	Dedza	2000-2017	-0.081	-0.070	-0.060
Malawi	Dowa	2000	0.349	0.447	0.570
Malawi	Dowa	2017	0.099	0.133	0.173
Malawi	Dowa	2000-2017	-0.085	-0.072	-0.060
Malawi	Karonga	2000	0.246	0.315	0.398
Malawi	Karonga	2017	0.099	0.131	0.170
Malawi	Karonga	2000-2017	-0.062	-0.050	-0.038
Malawi	Kasungu	2000	0.320	0.414	0.532
Malawi	Kasungu	2017	0.095	0.130	0.171
Malawi	Kasungu	2000-2017	-0.079	-0.067	-0.055
Malawi	Likoma	2000	0.223	0.310	0.435
Malawi	Likoma	2017	0.088	0.125	0.169
Malawi	Likoma	2000-2017	-0.081	-0.053	-0.024
Malawi	Lilongwe	2000	0.319	0.414	0.525
Malawi	Lilongwe	2017	0.097	0.130	0.169
Malawi	Lilongwe	2000-2017	-0.078	-0.067	-0.056
Malawi	Machinga	2000	0.258	0.329	0.422
Malawi	Machinga	2017	0.094	0.124	0.162
Malawi	Machinga	2000-2017	-0.064	-0.055	-0.047
Malawi	Mangochi	2000	0.264	0.344	0.437
Malawi	Mangochi	2017	0.091	0.119	0.155
Malawi	Mangochi	2000-2017	-0.068	-0.059	-0.050
Malawi	Mchinji	2000	0.332	0.431	0.543
Malawi	Mchinji	2017	0.092	0.126	0.164
Malawi	Mchinji	2000-2017	-0.083	-0.070	-0.056
Malawi	Mulanje	2000	0.270	0.346	0.440
Malawi	Mulanje	2017	0.106	0.137	0.181
Malawi	Mulanje	2000-2017	-0.067	-0.057	-0.048
Malawi	Mwanza	2000	0.258	0.339	0.438
Malawi	Mwanza	2017	0.105	0.138	0.184
Malawi	Mwanza	2000-2017	-0.063	-0.051	-0.038
Malawi	Mzimba	2000	0.242	0.311	0.402
Malawi	Mzimba	2017	0.094	0.124	0.162
Malawi	Mzimba	2000-2017	-0.061	-0.052	-0.043
Malawi	Neno	2000	0.274	0.355	0.456
Malawi	Neno	2017	0.099	0.132	0.173
Malawi	Neno	2000-2017	-0.067	-0.057	-0.046
Malawi	Nkhata Bay	2000	0.237	0.309	0.395
Malawi	Nkhata Bay	2017	0.098	0.129	0.165
Malawi	Nkhata Bay	2000-2017	-0.059	-0.050	-0.040
Malawi	Nkhotakota	2000	0.279	0.354	0.446
Malawi	Nkhotakota	2017	0.098	0.132	0.172
Malawi	Nkhotakota	2000-2017	-0.068	-0.056	-0.045
Malawi	Nsanje	2000	0.234	0.298	0.379
Malawi	Nsanje	2017	0.100	0.128	0.167
Malawi	Nsanje	2000-2017	-0.060	-0.050	-0.041
Malawi	Ntcheu	2000	0.308	0.403	0.502

Table 1: LRI DALYs rate by unit *(continued)*

Country	Unit	year	mean rate	lower rate	upper rate
Malawi	Ntcheu	2017	0.097	0.125	0.162
Malawi	Ntcheu	2000-2017	-0.078	-0.068	-0.058
Malawi	Ntchisi	2000	0.347	0.444	0.563
Malawi	Ntchisi	2017	0.096	0.129	0.168
Malawi	Ntchisi	2000-2017	-0.084	-0.071	-0.058
Malawi	Phalombe	2000	0.276	0.352	0.435
Malawi	Phalombe	2017	0.107	0.139	0.183
Malawi	Phalombe	2000-2017	-0.065	-0.054	-0.043
Malawi	Rumphi	2000	0.241	0.319	0.410
Malawi	Rumphi	2017	0.103	0.134	0.174
Malawi	Rumphi	2000-2017	-0.059	-0.049	-0.039
Malawi	Salima	2000	0.296	0.388	0.482
Malawi	Salima	2017	0.095	0.125	0.164
Malawi	Salima	2000-2017	-0.077	-0.065	-0.054
Malawi	Thyolo	2000	0.259	0.342	0.433
Malawi	Thyolo	2017	0.106	0.139	0.180
Malawi	Thyolo	2000-2017	-0.062	-0.053	-0.045
Malawi	Zomba	2000	0.264	0.347	0.441
Malawi	Zomba	2017	0.099	0.131	0.172
Malawi	Zomba	2000-2017	-0.066	-0.057	-0.048
Mali	Abeïbara	2000	0.213	0.364	0.575
Mali	Abeïbara	2017	0.206	0.385	0.626
Mali	Abeïbara	2000-2017	-0.032	-0.002	0.026
Mali	Ansongo	2000	0.237	0.410	0.637
Mali	Ansongo	2017	0.149	0.264	0.392
Mali	Ansongo	2000-2017	-0.056	-0.029	0.001
Mali	Bafoulabé	2000	0.204	0.338	0.499
Mali	Bafoulabé	2017	0.144	0.230	0.328
Mali	Bafoulabé	2000-2017	-0.052	-0.025	0.003
Mali	Bamako	2000	0.198	0.316	0.468
Mali	Bamako	2017	0.123	0.196	0.297
Mali	Bamako	2000-2017	-0.065	-0.030	-0.001
Mali	Banamba	2000	0.218	0.345	0.502
Mali	Banamba	2017	0.123	0.201	0.311
Mali	Banamba	2000-2017	-0.062	-0.032	-0.003
Mali	Bandiagara	2000	0.262	0.398	0.575
Mali	Bandiagara	2017	0.137	0.214	0.321
Mali	Bandiagara	2000-2017	-0.068	-0.040	-0.011
Mali	Bankass	2000	0.238	0.377	0.539
Mali	Bankass	2017	0.123	0.191	0.297
Mali	Bankass	2000-2017	-0.075	-0.046	-0.015
Mali	Barouéli	2000	0.229	0.359	0.520
Mali	Barouéli	2017	0.120	0.193	0.299
Mali	Barouéli	2000-2017	-0.069	-0.039	-0.008
Mali	Bla	2000	0.233	0.361	0.518
Mali	Bla	2017	0.123	0.193	0.302
Mali	Bla	2000-2017	-0.067	-0.040	-0.010
Mali	Bougouni	2000	0.221	0.362	0.535
Mali	Bougouni	2017	0.111	0.180	0.269
Mali	Bougouni	2000-2017	-0.078	-0.045	-0.015
Mali	Bourem	2000	0.262	0.412	0.619
Mali	Bourem	2017	0.162	0.282	0.420
Mali	Bourem	2000-2017	-0.057	-0.030	-0.001
Mali	Diéma	2000	0.239	0.383	0.582
Mali	Diéma	2017	0.144	0.224	0.318
Mali	Diéma	2000-2017	-0.058	-0.031	-0.003
Mali	Dioïla	2000	0.220	0.354	0.521
Mali	Dioïla	2017	0.112	0.179	0.271
Mali	Dioïla	2000-2017	-0.072	-0.043	-0.013
Mali	Diré	2000	0.273	0.420	0.611
Mali	Diré	2017	0.142	0.235	0.356

Table 1: LRI DALYs rate by unit *(continued)*

Country	Unit	year	mean rate	lower rate	upper rate
Mali	Diré	2000-2017	-0.070	-0.042	-0.011
Mali	Djenné	2000	0.235	0.367	0.548
Mali	Djenné	2017	0.123	0.198	0.308
Mali	Djenné	2000-2017	-0.068	-0.039	-0.010
Mali	Douentza	2000	0.269	0.413	0.585
Mali	Douentza	2017	0.149	0.240	0.356
Mali	Douentza	2000-2017	-0.060	-0.034	-0.005
Mali	Gao	2000	0.221	0.380	0.595
Mali	Gao	2017	0.148	0.269	0.413
Mali	Gao	2000-2017	-0.057	-0.024	0.010
Mali	Goundam	2000	0.266	0.414	0.600
Mali	Goundam	2017	0.148	0.241	0.360
Mali	Goundam	2000-2017	-0.064	-0.035	-0.005
Mali	Gourma-Rharous	2000	0.285	0.436	0.638
Mali	Gourma-Rharous	2017	0.145	0.247	0.366
Mali	Gourma-Rharous	2000-2017	-0.068	-0.041	-0.009
Mali	Kadiolo	2000	0.213	0.362	0.535
Mali	Kadiolo	2017	0.118	0.189	0.291
Mali	Kadiolo	2000-2017	-0.068	-0.040	-0.010
Mali	Kangaba	2000	0.212	0.333	0.496
Mali	Kangaba	2017	0.135	0.213	0.309
Mali	Kangaba	2000-2017	-0.055	-0.028	0.000
Mali	Kati	2000	0.196	0.313	0.458
Mali	Kati	2017	0.124	0.195	0.287
Mali	Kati	2000-2017	-0.057	-0.030	-0.001
Mali	Kayes	2000	0.214	0.352	0.534
Mali	Kayes	2017	0.156	0.248	0.358
Mali	Kayes	2000-2017	-0.049	-0.022	0.004
Mali	Kéniéba	2000	0.239	0.381	0.553
Mali	Kéniéba	2017	0.143	0.235	0.334
Mali	Kéniéba	2000-2017	-0.053	-0.027	-0.002
Mali	Kidal	2000	0.229	0.383	0.607
Mali	Kidal	2017	0.180	0.311	0.498
Mali	Kidal	2000-2017	-0.042	-0.016	0.011
Mali	Kita	2000	0.197	0.324	0.476
Mali	Kita	2017	0.132	0.217	0.312
Mali	Kita	2000-2017	-0.051	-0.024	0.003
Mali	Kolokani	2000	0.203	0.330	0.503
Mali	Kolokani	2017	0.128	0.205	0.303
Mali	Kolokani	2000-2017	-0.055	-0.028	-0.002
Mali	Kolondiéba	2000	0.210	0.356	0.526
Mali	Kolondiéba	2017	0.108	0.174	0.259
Mali	Kolondiéba	2000-2017	-0.082	-0.047	-0.018
Mali	Koro	2000	0.256	0.399	0.587
Mali	Koro	2017	0.126	0.202	0.309
Mali	Koro	2000-2017	-0.071	-0.044	-0.012
Mali	Koulikoro	2000	0.212	0.330	0.487
Mali	Koulikoro	2017	0.122	0.197	0.317
Mali	Koulikoro	2000-2017	-0.061	-0.031	-0.002
Mali	Koutiala	2000	0.224	0.355	0.520
Mali	Koutiala	2017	0.115	0.185	0.284
Mali	Koutiala	2000-2017	-0.071	-0.041	-0.009
Mali	Macina	2000	0.218	0.337	0.487
Mali	Macina	2017	0.125	0.199	0.315
Mali	Macina	2000-2017	-0.058	-0.030	-0.002
Mali	Ménaka	2000	0.220	0.402	0.599
Mali	Ménaka	2017	0.165	0.287	0.443
Mali	Ménaka	2000-2017	-0.053	-0.025	0.004
Mali	Mopti	2000	0.235	0.367	0.543
Mali	Mopti	2017	0.137	0.209	0.317
Mali	Mopti	2000-2017	-0.062	-0.035	-0.006

Table 1: LRI DALYs rate by unit *(continued)*

Country	Unit	year	mean rate	lower rate	upper rate
Mali	Nara	2000	0.237	0.369	0.539
Mali	Nara	2017	0.144	0.222	0.329
Mali	Nara	2000-2017	-0.058	-0.030	-0.003
Mali	Niafunké	2000	0.265	0.407	0.584
Mali	Niafunké	2017	0.145	0.227	0.339
Mali	Niafunké	2000-2017	-0.066	-0.039	-0.008
Mali	Niono	2000	0.203	0.309	0.451
Mali	Niono	2017	0.134	0.208	0.321
Mali	Niono	2000-2017	-0.053	-0.025	0.004
Mali	Nioro	2000	0.246	0.387	0.600
Mali	Nioro	2017	0.147	0.232	0.336
Mali	Nioro	2000-2017	-0.058	-0.030	0.000
Mali	San	2000	0.221	0.360	0.523
Mali	San	2017	0.119	0.195	0.303
Mali	San	2000-2017	-0.071	-0.039	-0.007
Mali	Ségou	2000	0.207	0.325	0.478
Mali	Ségou	2017	0.128	0.198	0.309
Mali	Ségou	2000-2017	-0.057	-0.030	0.000
Mali	Sikasso	2000	0.221	0.360	0.529
Mali	Sikasso	2017	0.109	0.181	0.278
Mali	Sikasso	2000-2017	-0.072	-0.043	-0.014
Mali	Ténenkou	2000	0.221	0.346	0.511
Mali	Ténenkou	2017	0.128	0.205	0.309
Mali	Ténenkou	2000-2017	-0.061	-0.034	-0.005
Mali	Tessalit	2000	0.225	0.365	0.570
Mali	Tessalit	2017	0.172	0.314	0.501
Mali	Tessalit	2000-2017	-0.039	-0.013	0.015
Mali	Tin-Essako	2000	0.223	0.383	0.597
Mali	Tin-Essako	2017	0.186	0.329	0.528
Mali	Tin-Essako	2000-2017	-0.040	-0.012	0.016
Mali	Tombouctou	2000	0.262	0.416	0.619
Mali	Tombouctou	2017	0.147	0.251	0.378
Mali	Tombouctou	2000-2017	-0.066	-0.036	0.000
Mali	Tominian	2000	0.220	0.356	0.519
Mali	Tominian	2017	0.123	0.194	0.308
Mali	Tominian	2000-2017	-0.068	-0.038	-0.008
Mali	Yanfolila	2000	0.232	0.367	0.534
Mali	Yanfolila	2017	0.124	0.199	0.289
Mali	Yanfolila	2000-2017	-0.067	-0.037	-0.011
Mali	Yélimané	2000	0.217	0.363	0.556
Mali	Yélimané	2017	0.151	0.238	0.345
Mali	Yélimané	2000-2017	-0.053	-0.027	0.002
Mali	Yorosso	2000	0.198	0.314	0.459
Mali	Yorosso	2017	0.107	0.175	0.277
Mali	Yorosso	2000-2017	-0.062	-0.034	-0.004
Mali	Youwarou	2000	0.257	0.401	0.574
Mali	Youwarou	2017	0.150	0.236	0.363
Mali	Youwarou	2000-2017	-0.061	-0.033	-0.005
Mauritania	Aïoun	2000	0.133	0.231	0.371
Mauritania	Aïoun	2017	0.057	0.093	0.140
Mauritania	Aïoun	2000-2017	-0.082	-0.053	-0.026
Mauritania	Akjoujt	2000	0.154	0.248	0.375
Mauritania	Akjoujt	2017	0.066	0.104	0.165
Mauritania	Akjoujt	2000-2017	-0.078	-0.053	-0.028
Mauritania	Aleg	2000	0.153	0.252	0.379
Mauritania	Aleg	2017	0.063	0.104	0.153
Mauritania	Aleg	2000-2017	-0.076	-0.052	-0.029
Mauritania	Amourj	2000	0.133	0.231	0.351
Mauritania	Amourj	2017	0.056	0.091	0.145
Mauritania	Amourj	2000-2017	-0.082	-0.055	-0.031
Mauritania	Aoujeft	2000	0.151	0.258	0.419

Table 1: LRI DALYs rate by unit *(continued)*

Country	Unit	year	mean rate	lower rate	upper rate
Mauritania	Aoujeft	2017	0.063	0.100	0.156
Mauritania	Aoujeft	2000-2017	-0.083	-0.059	-0.033
Mauritania	Atar	2000	0.138	0.245	0.395
Mauritania	Atar	2017	0.059	0.101	0.155
Mauritania	Atar	2000-2017	-0.084	-0.055	-0.027
Mauritania	Bababé	2000	0.156	0.252	0.371
Mauritania	Bababé	2017	0.066	0.106	0.155
Mauritania	Bababé	2000-2017	-0.079	-0.052	-0.025
Mauritania	Barkéol	2000	0.152	0.253	0.384
Mauritania	Barkéol	2017	0.062	0.101	0.145
Mauritania	Barkéol	2000-2017	-0.078	-0.054	-0.028
Mauritania	Bassikounou	2000	0.135	0.235	0.362
Mauritania	Bassikounou	2017	0.054	0.094	0.144
Mauritania	Bassikounou	2000-2017	-0.082	-0.054	-0.029
Mauritania	Bir Moghreïn	2000	0.103	0.213	0.369
Mauritania	Bir Moghreïn	2017	0.051	0.096	0.178
Mauritania	Bir Moghreïn	2000-2017	-0.079	-0.048	-0.016
Mauritania	Boghé	2000	0.157	0.255	0.383
Mauritania	Boghé	2017	0.065	0.107	0.156
Mauritania	Boghé	2000-2017	-0.075	-0.052	-0.027
Mauritania	Boumdeïd	2000	0.149	0.251	0.392
Mauritania	Boumdeïd	2017	0.061	0.100	0.144
Mauritania	Boumdeïd	2000-2017	-0.082	-0.054	-0.029
Mauritania	Boutilimit	2000	0.158	0.258	0.375
Mauritania	Boutilimit	2017	0.067	0.108	0.162
Mauritania	Boutilimit	2000-2017	-0.076	-0.052	-0.029
Mauritania	Chinguetti	2000	0.143	0.241	0.396
Mauritania	Chinguetti	2017	0.057	0.095	0.151
Mauritania	Chinguetti	2000-2017	-0.085	-0.057	-0.030
Mauritania	Djiguenni	2000	0.138	0.234	0.373
Mauritania	Djiguenni	2017	0.055	0.090	0.134
Mauritania	Djiguenni	2000-2017	-0.082	-0.055	-0.031
Mauritania	F'Dérik	2000	0.124	0.230	0.364
Mauritania	F'Dérik	2017	0.059	0.103	0.174
Mauritania	F'Dérik	2000-2017	-0.085	-0.048	-0.015
Mauritania	Guérou	2000	0.142	0.252	0.409
Mauritania	Guérou	2017	0.061	0.101	0.148
Mauritania	Guérou	2000-2017	-0.077	-0.052	-0.023
Mauritania	Kaédi	2000	0.153	0.248	0.374
Mauritania	Kaédi	2017	0.064	0.104	0.153
Mauritania	Kaédi	2000-2017	-0.077	-0.052	-0.025
Mauritania	Kankossa	2000	0.139	0.233	0.362
Mauritania	Kankossa	2017	0.058	0.096	0.147
Mauritania	Kankossa	2000-2017	-0.077	-0.052	-0.029
Mauritania	Keur-Macène	2000	0.187	0.278	0.397
Mauritania	Keur-Macène	2017	0.078	0.118	0.175
Mauritania	Keur-Macène	2000-2017	-0.078	-0.051	-0.023
Mauritania	Kiffa	2000	0.140	0.241	0.375
Mauritania	Kiffa	2017	0.059	0.099	0.148
Mauritania	Kiffa	2000-2017	-0.078	-0.053	-0.027
Mauritania	Kobenni	2000	0.139	0.234	0.372
Mauritania	Kobenni	2017	0.056	0.091	0.137
Mauritania	Kobenni	2000-2017	-0.081	-0.054	-0.029
Mauritania	M'Bagne	2000	0.154	0.252	0.377
Mauritania	M'Bagne	2017	0.065	0.105	0.157
Mauritania	M'Bagne	2000-2017	-0.077	-0.052	-0.024
Mauritania	M'Bout	2000	0.146	0.243	0.380
Mauritania	M'Bout	2017	0.061	0.100	0.148
Mauritania	M'Bout	2000-2017	-0.075	-0.052	-0.029
Mauritania	Maghama	2000	0.153	0.243	0.361
Mauritania	Maghama	2017	0.064	0.104	0.150

Table 1: LRI DALYs rate by unit *(continued)*

Country	Unit	year	mean rate	lower rate	upper rate
Mauritania	Maghama	2000-2017	-0.072	-0.050	-0.028
Mauritania	Magta-Lahjar	2000	0.158	0.262	0.409
Mauritania	Magta-Lahjar	2017	0.065	0.103	0.148
Mauritania	Magta-Lahjar	2000-2017	-0.079	-0.053	-0.029
Mauritania	Méderdra	2000	0.174	0.271	0.407
Mauritania	Méderdra	2017	0.069	0.111	0.172
Mauritania	Méderdra	2000-2017	-0.077	-0.052	-0.027
Mauritania	Monguel	2000	0.149	0.247	0.377
Mauritania	Monguel	2017	0.063	0.102	0.149
Mauritania	Monguel	2000-2017	-0.076	-0.051	-0.027
Mauritania	Moudjéria	2000	0.135	0.229	0.359
Mauritania	Moudjéria	2017	0.057	0.090	0.130
Mauritania	Moudjéria	2000-2017	-0.082	-0.056	-0.031
Mauritania	Néma	2000	0.133	0.234	0.357
Mauritania	Néma	2017	0.055	0.093	0.146
Mauritania	Néma	2000-2017	-0.084	-0.055	-0.030
Mauritania	Nouadhibou	2000	0.141	0.262	0.444
Mauritania	Nouadhibou	2017	0.065	0.113	0.199
Mauritania	Nouadhibou	2000-2017	-0.090	-0.049	-0.015
Mauritania	Nouakchott	2000	0.164	0.261	0.390
Mauritania	Nouakchott	2017	0.065	0.105	0.162
Mauritania	Nouakchott	2000-2017	-0.081	-0.054	-0.026
Mauritania	Ouad-Naga	2000	0.171	0.265	0.386
Mauritania	Ouad-Naga	2017	0.069	0.111	0.171
Mauritania	Ouad-Naga	2000-2017	-0.077	-0.052	-0.028
Mauritania	Ouadane	2000	0.139	0.241	0.358
Mauritania	Ouadane	2017	0.052	0.093	0.145
Mauritania	Ouadane	2000-2017	-0.083	-0.056	-0.033
Mauritania	Ould Yengé	2000	0.144	0.240	0.375
Mauritania	Ould Yengé	2017	0.061	0.098	0.149
Mauritania	Ould Yengé	2000-2017	-0.075	-0.052	-0.027
Mauritania	R'Kiz	2000	0.168	0.264	0.392
Mauritania	R'Kiz	2017	0.068	0.111	0.169
Mauritania	R'Kiz	2000-2017	-0.075	-0.051	-0.026
Mauritania	Rosso	2000	0.181	0.271	0.389
Mauritania	Rosso	2017	0.078	0.117	0.173
Mauritania	Rosso	2000-2017	-0.076	-0.050	-0.023
Mauritania	Sélibaby	2000	0.146	0.232	0.362
Mauritania	Sélibaby	2017	0.059	0.098	0.146
Mauritania	Sélibaby	2000-2017	-0.076	-0.051	-0.026
Mauritania	Tamchakett	2000	0.127	0.229	0.368
Mauritania	Tamchakett	2017	0.062	0.097	0.145
Mauritania	Tamchakett	2000-2017	-0.079	-0.051	-0.027
Mauritania	Tichitt	2000	0.125	0.220	0.349
Mauritania	Tichitt	2017	0.051	0.085	0.122
Mauritania	Tichitt	2000-2017	-0.083	-0.057	-0.030
Mauritania	Tidjikja	2000	0.133	0.227	0.355
Mauritania	Tidjikja	2017	0.056	0.088	0.127
Mauritania	Tidjikja	2000-2017	-0.084	-0.056	-0.031
Mauritania	Timbédra	2000	0.133	0.234	0.358
Mauritania	Timbédra	2017	0.058	0.093	0.149
Mauritania	Timbédra	2000-2017	-0.081	-0.053	-0.029
Mauritania	Tintane	2000	0.143	0.235	0.373
Mauritania	Tintane	2017	0.058	0.093	0.139
Mauritania	Tintane	2000-2017	-0.081	-0.054	-0.032
Mauritania	Zouérate	2000	0.125	0.231	0.372
Mauritania	Zouérate	2017	0.055	0.104	0.177
Mauritania	Zouérate	2000-2017	-0.086	-0.048	-0.011
Mozambique	Alto Molocue	2000	0.230	0.339	0.505
Mozambique	Alto Molocue	2017	0.073	0.108	0.161
Mozambique	Alto Molocue	2000-2017	-0.072	-0.066	-0.060

Table 1: LRI DALYs rate by unit *(continued)*

Country	Unit	year	mean rate	lower rate	upper rate
Mozambique	Ancuabe	2000	0.229	0.336	0.494
Mozambique	Ancuabe	2017	0.070	0.102	0.151
Mozambique	Ancuabe	2000-2017	-0.077	-0.069	-0.062
Mozambique	Angoche	2000	0.217	0.321	0.473
Mozambique	Angoche	2017	0.069	0.103	0.153
Mozambique	Angoche	2000-2017	-0.074	-0.067	-0.061
Mozambique	Angónia	2000	0.353	0.490	0.688
Mozambique	Angónia	2017	0.081	0.116	0.169
Mozambique	Angónia	2000-2017	-0.095	-0.085	-0.075
Mozambique	Balama	2000	0.239	0.341	0.486
Mozambique	Balama	2017	0.068	0.100	0.150
Mozambique	Balama	2000-2017	-0.079	-0.072	-0.064
Mozambique	Barue	2000	0.236	0.345	0.502
Mozambique	Barue	2017	0.067	0.100	0.147
Mozambique	Barue	2000-2017	-0.079	-0.072	-0.066
Mozambique	Bilene	2000	0.228	0.335	0.492
Mozambique	Bilene	2017	0.074	0.109	0.161
Mozambique	Bilene	2000-2017	-0.073	-0.065	-0.056
Mozambique	Boane	2000	0.228	0.342	0.501
Mozambique	Boane	2017	0.077	0.110	0.158
Mozambique	Boane	2000-2017	-0.081	-0.065	-0.049
Mozambique	Buzi	2000	0.219	0.320	0.471
Mozambique	Buzi	2017	0.070	0.104	0.152
Mozambique	Buzi	2000-2017	-0.073	-0.065	-0.059
Mozambique	Cahora Bassa	2000	0.242	0.355	0.523
Mozambique	Cahora Bassa	2017	0.072	0.105	0.152
Mozambique	Cahora Bassa	2000-2017	-0.079	-0.070	-0.062
Mozambique	Caia	2000	0.230	0.343	0.488
Mozambique	Caia	2017	0.070	0.102	0.149
Mozambique	Caia	2000-2017	-0.079	-0.071	-0.063
Mozambique	Changara	2000	0.229	0.344	0.509
Mozambique	Changara	2017	0.073	0.107	0.156
Mozambique	Changara	2000-2017	-0.075	-0.067	-0.058
Mozambique	Chemba	2000	0.232	0.343	0.503
Mozambique	Chemba	2017	0.069	0.102	0.149
Mozambique	Chemba	2000-2017	-0.077	-0.070	-0.062
Mozambique	Cheringoma	2000	0.223	0.326	0.472
Mozambique	Cheringoma	2017	0.069	0.102	0.152
Mozambique	Cheringoma	2000-2017	-0.074	-0.067	-0.060
Mozambique	Chibabava	2000	0.218	0.321	0.475
Mozambique	Chibabava	2017	0.070	0.105	0.155
Mozambique	Chibabava	2000-2017	-0.071	-0.064	-0.058
Mozambique	Chibuto	2000	0.230	0.333	0.482
Mozambique	Chibuto	2017	0.076	0.110	0.167
Mozambique	Chibuto	2000-2017	-0.077	-0.065	-0.053
Mozambique	Chicualacuala	2000	0.227	0.332	0.487
Mozambique	Chicualacuala	2017	0.073	0.109	0.160
Mozambique	Chicualacuala	2000-2017	-0.069	-0.065	-0.059
Mozambique	Chifunde	2000	0.294	0.409	0.566
Mozambique	Chifunde	2017	0.068	0.099	0.142
Mozambique	Chifunde	2000-2017	-0.091	-0.083	-0.077
Mozambique	Chigubo	2000	0.232	0.340	0.501
Mozambique	Chigubo	2017	0.074	0.111	0.163
Mozambique	Chigubo	2000-2017	-0.069	-0.065	-0.060
Mozambique	Chinde	2000	0.226	0.334	0.485
Mozambique	Chinde	2017	0.069	0.101	0.147
Mozambique	Chinde	2000-2017	-0.076	-0.069	-0.063
Mozambique	Chiúre	2000	0.228	0.330	0.484
Mozambique	Chiúre	2017	0.069	0.103	0.153
Mozambique	Chiúre	2000-2017	-0.074	-0.068	-0.061
Mozambique	Chiuta	2000	0.265	0.389	0.586

Table 1: LRI DALYs rate by unit *(continued)*

Country	Unit	year	mean rate	lower rate	upper rate
Mozambique	Chiuta	2017	0.069	0.102	0.152
Mozambique	Chiuta	2000-2017	-0.082	-0.077	-0.071
Mozambique	Chókwè	2000	0.231	0.336	0.505
Mozambique	Chókwè	2017	0.072	0.107	0.156
Mozambique	Chókwè	2000-2017	-0.075	-0.066	-0.057
Mozambique	Cidade de Matola	2000	0.247	0.368	0.550
Mozambique	Cidade de Matola	2017	0.075	0.112	0.165
Mozambique	Cidade de Matola	2000-2017	-0.083	-0.068	-0.051
Mozambique	Cuamba	2000	0.215	0.329	0.482
Mozambique	Cuamba	2017	0.070	0.100	0.153
Mozambique	Cuamba	2000-2017	-0.078	-0.069	-0.059
Mozambique	Dondo	2000	0.234	0.346	0.503
Mozambique	Dondo	2017	0.072	0.107	0.152
Mozambique	Dondo	2000-2017	-0.080	-0.068	-0.054
Mozambique	Erati	2000	0.217	0.326	0.481
Mozambique	Erati	2017	0.071	0.103	0.153
Mozambique	Erati	2000-2017	-0.072	-0.066	-0.061
Mozambique	Funhalouro	2000	0.227	0.340	0.495
Mozambique	Funhalouro	2017	0.075	0.111	0.163
Mozambique	Funhalouro	2000-2017	-0.070	-0.065	-0.060
Mozambique	Gile	2000	0.216	0.319	0.474
Mozambique	Gile	2017	0.071	0.104	0.153
Mozambique	Gile	2000-2017	-0.070	-0.065	-0.059
Mozambique	Gondola	2000	0.236	0.341	0.496
Mozambique	Gondola	2017	0.070	0.103	0.150
Mozambique	Gondola	2000-2017	-0.080	-0.070	-0.058
Mozambique	Gorongosa	2000	0.223	0.327	0.472
Mozambique	Gorongosa	2017	0.070	0.102	0.151
Mozambique	Gorongosa	2000-2017	-0.073	-0.067	-0.061
Mozambique	Govuro	2000	0.221	0.332	0.480
Mozambique	Govuro	2017	0.070	0.106	0.154
Mozambique	Govuro	2000-2017	-0.074	-0.066	-0.060
Mozambique	Guijá	2000	0.228	0.332	0.485
Mozambique	Guijá	2017	0.074	0.108	0.159
Mozambique	Guijá	2000-2017	-0.072	-0.065	-0.057
Mozambique	Guro	2000	0.223	0.326	0.478
Mozambique	Guro	2017	0.067	0.100	0.146
Mozambique	Guro	2000-2017	-0.075	-0.068	-0.060
Mozambique	Gurue	2000	0.233	0.340	0.492
Mozambique	Gurue	2017	0.069	0.102	0.153
Mozambique	Gurue	2000-2017	-0.078	-0.071	-0.064
Mozambique	Homoine	2000	0.234	0.344	0.501
Mozambique	Homoine	2017	0.073	0.107	0.162
Mozambique	Homoine	2000-2017	-0.076	-0.068	-0.058
Mozambique	Ile	2000	0.218	0.325	0.482
Mozambique	Ile	2017	0.070	0.101	0.148
Mozambique	Ile	2000-2017	-0.074	-0.068	-0.062
Mozambique	Inharrime	2000	0.230	0.341	0.488
Mozambique	Inharrime	2017	0.074	0.108	0.160
Mozambique	Inharrime	2000-2017	-0.074	-0.067	-0.058
Mozambique	Inhassoro	2000	0.230	0.342	0.490
Mozambique	Inhassoro	2017	0.074	0.108	0.158
Mozambique	Inhassoro	2000-2017	-0.074	-0.068	-0.061
Mozambique	Inhassunge	2000	0.213	0.328	0.474
Mozambique	Inhassunge	2017	0.069	0.102	0.144
Mozambique	Inhassunge	2000-2017	-0.082	-0.068	-0.056
Mozambique	Jangamo	2000	0.232	0.343	0.497
Mozambique	Jangamo	2017	0.071	0.109	0.161
Mozambique	Jangamo	2000-2017	-0.075	-0.067	-0.059
Mozambique	Lago	2000	0.284	0.420	0.613
Mozambique	Lago	2017	0.070	0.103	0.153

Table 1: LRI DALYs rate by unit *(continued)*

Country	Unit	year	mean rate	lower rate	upper rate
Mozambique	Lago	2000-2017	-0.089	-0.083	-0.076
Mozambique	Lalaua	2000	0.219	0.323	0.482
Mozambique	Lalaua	2017	0.069	0.103	0.150
Mozambique	Lalaua	2000-2017	-0.072	-0.066	-0.060
Mozambique	Lichinga	2000	0.273	0.399	0.580
Mozambique	Lichinga	2017	0.067	0.100	0.147
Mozambique	Lichinga	2000-2017	-0.092	-0.082	-0.071
Mozambique	Lugela	2000	0.238	0.350	0.518
Mozambique	Lugela	2017	0.068	0.101	0.151
Mozambique	Lugela	2000-2017	-0.080	-0.074	-0.068
Mozambique	Mabalane	2000	0.231	0.336	0.487
Mozambique	Mabalane	2017	0.075	0.110	0.161
Mozambique	Mabalane	2000-2017	-0.071	-0.065	-0.059
Mozambique	Mabote	2000	0.229	0.339	0.494
Mozambique	Mabote	2017	0.073	0.110	0.161
Mozambique	Mabote	2000-2017	-0.072	-0.066	-0.060
Mozambique	Macanga	2000	0.261	0.379	0.566
Mozambique	Macanga	2017	0.067	0.100	0.147
Mozambique	Macanga	2000-2017	-0.086	-0.078	-0.071
Mozambique	Machanga	2000	0.219	0.324	0.483
Mozambique	Machanga	2017	0.070	0.105	0.153
Mozambique	Machanga	2000-2017	-0.073	-0.066	-0.056
Mozambique	Machaze	2000	0.222	0.328	0.481
Mozambique	Machaze	2017	0.071	0.107	0.158
Mozambique	Machaze	2000-2017	-0.071	-0.065	-0.059
Mozambique	Macomia	2000	0.232	0.344	0.510
Mozambique	Macomia	2017	0.070	0.103	0.155
Mozambique	Macomia	2000-2017	-0.078	-0.071	-0.064
Mozambique	Macossa	2000	0.232	0.341	0.498
Mozambique	Macossa	2017	0.067	0.099	0.147
Mozambique	Macossa	2000-2017	-0.079	-0.072	-0.066
Mozambique	Maganja da Costa	2000	0.225	0.329	0.481
Mozambique	Maganja da Costa	2017	0.069	0.101	0.149
Mozambique	Maganja da Costa	2000-2017	-0.075	-0.069	-0.064
Mozambique	Magoe	2000	0.223	0.327	0.479
Mozambique	Magoe	2017	0.071	0.106	0.152
Mozambique	Magoe	2000-2017	-0.070	-0.065	-0.060
Mozambique	Magude	2000	0.226	0.334	0.495
Mozambique	Magude	2017	0.074	0.107	0.156
Mozambique	Magude	2000-2017	-0.072	-0.066	-0.058
Mozambique	Majune	2000	0.234	0.350	0.509
Mozambique	Majune	2017	0.067	0.100	0.151
Mozambique	Majune	2000-2017	-0.080	-0.073	-0.067
Mozambique	Malema	2000	0.217	0.317	0.468
Mozambique	Malema	2017	0.069	0.101	0.150
Mozambique	Malema	2000-2017	-0.073	-0.066	-0.060
Mozambique	Mandimba	2000	0.251	0.373	0.542
Mozambique	Mandimba	2017	0.065	0.100	0.151
Mozambique	Mandimba	2000-2017	-0.085	-0.077	-0.071
Mozambique	Mandlakazi	2000	0.232	0.337	0.489
Mozambique	Mandlakazi	2017	0.073	0.112	0.164
Mozambique	Mandlakazi	2000-2017	-0.074	-0.066	-0.058
Mozambique	Manhiça	2000	0.244	0.352	0.515
Mozambique	Manhiça	2017	0.078	0.115	0.165
Mozambique	Manhiça	2000-2017	-0.075	-0.065	-0.056
Mozambique	Manica	2000	0.237	0.342	0.496
Mozambique	Manica	2017	0.070	0.102	0.151
Mozambique	Manica	2000-2017	-0.078	-0.070	-0.064
Mozambique	Maputo	2000	0.257	0.380	0.561
Mozambique	Maputo	2017	0.077	0.113	0.162
Mozambique	Maputo	2000-2017	-0.084	-0.070	-0.058

Table 1: LRI DALYs rate by unit *(continued)*

Country	Unit	year	mean rate	lower rate	upper rate
Mozambique	Maravia	2000	0.267	0.384	0.567
Mozambique	Maravia	2017	0.067	0.101	0.147
Mozambique	Maravia	2000-2017	-0.081	-0.075	-0.070
Mozambique	Maringue	2000	0.230	0.330	0.483
Mozambique	Maringue	2017	0.066	0.099	0.147
Mozambique	Maringue	2000-2017	-0.077	-0.070	-0.063
Mozambique	Marracuene	2000	0.228	0.346	0.508
Mozambique	Marracuene	2017	0.074	0.112	0.161
Mozambique	Marracuene	2000-2017	-0.078	-0.065	-0.054
Mozambique	Marromeu	2000	0.221	0.328	0.478
Mozambique	Marromeu	2017	0.069	0.102	0.150
Mozambique	Marromeu	2000-2017	-0.076	-0.068	-0.061
Mozambique	Marrupa	2000	0.236	0.347	0.510
Mozambique	Marrupa	2017	0.067	0.101	0.151
Mozambique	Marrupa	2000-2017	-0.079	-0.072	-0.066
Mozambique	Massangena	2000	0.227	0.337	0.495
Mozambique	Massangena	2017	0.072	0.108	0.161
Mozambique	Massangena	2000-2017	-0.072	-0.066	-0.059
Mozambique	Massinga	2000	0.226	0.339	0.490
Mozambique	Massinga	2017	0.074	0.109	0.163
Mozambique	Massinga	2000-2017	-0.073	-0.066	-0.058
Mozambique	Massingir	2000	0.226	0.339	0.495
Mozambique	Massingir	2017	0.074	0.109	0.163
Mozambique	Massingir	2000-2017	-0.070	-0.066	-0.060
Mozambique	Matutuíne	2000	0.227	0.331	0.487
Mozambique	Matutuíne	2017	0.074	0.110	0.161
Mozambique	Matutuíne	2000-2017	-0.070	-0.064	-0.059
Mozambique	Maúa	2000	0.225	0.337	0.502
Mozambique	Maúa	2017	0.069	0.101	0.152
Mozambique	Maúa	2000-2017	-0.076	-0.069	-0.062
Mozambique	Mavago	2000	0.242	0.351	0.506
Mozambique	Mavago	2017	0.069	0.101	0.151
Mozambique	Mavago	2000-2017	-0.079	-0.073	-0.066
Mozambique	Mecanhelas	2000	0.304	0.443	0.637
Mozambique	Mecanhelas	2017	0.068	0.102	0.150
Mozambique	Mecanhelas	2000-2017	-0.092	-0.087	-0.080
Mozambique	Meconta	2000	0.217	0.318	0.478
Mozambique	Meconta	2017	0.069	0.102	0.150
Mozambique	Meconta	2000-2017	-0.073	-0.066	-0.058
Mozambique	Mecuburi	2000	0.213	0.315	0.473
Mozambique	Mecuburi	2017	0.068	0.102	0.152
Mozambique	Mecuburi	2000-2017	-0.071	-0.065	-0.058
Mozambique	Mecufi	2000	0.217	0.319	0.483
Mozambique	Mecufi	2017	0.068	0.103	0.155
Mozambique	Mecufi	2000-2017	-0.079	-0.065	-0.053
Mozambique	Mecula	2000	0.240	0.344	0.498
Mozambique	Mecula	2017	0.074	0.107	0.157
Mozambique	Mecula	2000-2017	-0.074	-0.069	-0.062
Mozambique	Meluco	2000	0.238	0.350	0.519
Mozambique	Meluco	2017	0.069	0.104	0.154
Mozambique	Meluco	2000-2017	-0.079	-0.071	-0.064
Mozambique	Memba	2000	0.219	0.326	0.482
Mozambique	Memba	2017	0.069	0.102	0.150
Mozambique	Memba	2000-2017	-0.072	-0.067	-0.060
Mozambique	Metarica	2000	0.219	0.329	0.498
Mozambique	Metarica	2017	0.068	0.100	0.152
Mozambique	Metarica	2000-2017	-0.075	-0.068	-0.061
Mozambique	Milange	2000	0.300	0.431	0.621
Mozambique	Milange	2017	0.073	0.104	0.150
Mozambique	Milange	2000-2017	-0.093	-0.087	-0.081
Mozambique	Moamba	2000	0.232	0.332	0.478

Table 1: LRI DALYs rate by unit *(continued)*

Country	Unit	year	mean rate	lower rate	upper rate
Mozambique	Moamba	2017	0.075	0.110	0.163
Mozambique	Moamba	2000-2017	-0.072	-0.064	-0.057
Mozambique	Moatize	2000	0.268	0.382	0.562
Mozambique	Moatize	2017	0.073	0.107	0.156
Mozambique	Moatize	2000-2017	-0.080	-0.074	-0.068
Mozambique	Mocimboa da Praia	2000	0.239	0.342	0.498
Mozambique	Mocimboa da Praia	2017	0.071	0.105	0.153
Mozambique	Mocimboa da Praia	2000-2017	-0.079	-0.070	-0.061
Mozambique	Mocuba	2000	0.225	0.328	0.486
Mozambique	Mocuba	2017	0.069	0.101	0.148
Mozambique	Mocuba	2000-2017	-0.077	-0.069	-0.060
Mozambique	Mogovolas	2000	0.214	0.318	0.470
Mozambique	Mogovolas	2017	0.069	0.101	0.148
Mozambique	Mogovolas	2000-2017	-0.072	-0.066	-0.060
Mozambique	Moma	2000	0.223	0.324	0.475
Mozambique	Moma	2017	0.070	0.103	0.153
Mozambique	Moma	2000-2017	-0.072	-0.067	-0.060
Mozambique	Monapo	2000	0.217	0.318	0.462
Mozambique	Monapo	2017	0.068	0.103	0.154
Mozambique	Monapo	2000-2017	-0.073	-0.065	-0.058
Mozambique	Mongincual	2000	0.215	0.319	0.470
Mozambique	Mongincual	2017	0.068	0.102	0.152
Mozambique	Mongincual	2000-2017	-0.073	-0.067	-0.061
Mozambique	Montepuez	2000	0.241	0.346	0.498
Mozambique	Montepuez	2017	0.068	0.100	0.154
Mozambique	Montepuez	2000-2017	-0.082	-0.073	-0.064
Mozambique	Mopeia	2000	0.218	0.328	0.481
Mozambique	Mopeia	2017	0.068	0.101	0.151
Mozambique	Mopeia	2000-2017	-0.077	-0.069	-0.062
Mozambique	Morrumbala	2000	0.256	0.372	0.534
Mozambique	Morrumbala	2017	0.072	0.106	0.155
Mozambique	Morrumbala	2000-2017	-0.081	-0.074	-0.068
Mozambique	Morrumbene	2000	0.228	0.340	0.494
Mozambique	Morrumbene	2017	0.073	0.108	0.164
Mozambique	Morrumbene	2000-2017	-0.074	-0.067	-0.059
Mozambique	Mossuril	2000	0.223	0.332	0.492
Mozambique	Mossuril	2017	0.069	0.102	0.151
Mozambique	Mossuril	2000-2017	-0.078	-0.068	-0.061
Mozambique	Mossurize	2000	0.221	0.324	0.472
Mozambique	Mossurize	2017	0.073	0.105	0.156
Mozambique	Mossurize	2000-2017	-0.071	-0.065	-0.059
Mozambique	Muanza	2000	0.224	0.326	0.486
Mozambique	Muanza	2017	0.070	0.103	0.153
Mozambique	Muanza	2000-2017	-0.073	-0.067	-0.061
Mozambique	Muecate	2000	0.217	0.315	0.466
Mozambique	Muecate	2017	0.070	0.102	0.150
Mozambique	Muecate	2000-2017	-0.072	-0.065	-0.059
Mozambique	Mueda	2000	0.246	0.352	0.513
Mozambique	Mueda	2017	0.070	0.105	0.157
Mozambique	Mueda	2000-2017	-0.080	-0.072	-0.064
Mozambique	Muembe	2000	0.251	0.369	0.546
Mozambique	Muembe	2017	0.068	0.100	0.145
Mozambique	Muembe	2000-2017	-0.086	-0.077	-0.069
Mozambique	Muidumbe	2000	0.240	0.352	0.511
Mozambique	Muidumbe	2017	0.069	0.103	0.156
Mozambique	Muidumbe	2000-2017	-0.080	-0.072	-0.063
Mozambique	Murrupula	2000	0.213	0.311	0.452
Mozambique	Murrupula	2017	0.069	0.103	0.150
Mozambique	Murrupula	2000-2017	-0.071	-0.063	-0.055
Mozambique	Mutarara	2000	0.267	0.384	0.565
Mozambique	Mutarara	2017	0.071	0.105	0.152

Table 1: LRI DALYs rate by unit *(continued)*

Country	Unit	year	mean rate	lower rate	upper rate
Mozambique	Mutarara	2000-2017	-0.083	-0.077	-0.070
Mozambique	N'gauma	2000	0.273	0.396	0.557
Mozambique	N'gauma	2017	0.069	0.099	0.144
Mozambique	N'gauma	2000-2017	-0.092	-0.084	-0.077
Mozambique	Nacala Velha	2000	0.226	0.338	0.487
Mozambique	Nacala Velha	2017	0.069	0.101	0.150
Mozambique	Nacala Velha	2000-2017	-0.082	-0.069	-0.056
Mozambique	Nacaroa	2000	0.218	0.324	0.483
Mozambique	Nacaroa	2017	0.069	0.103	0.151
Mozambique	Nacaroa	2000-2017	-0.072	-0.066	-0.059
Mozambique	Namaacha	2000	0.233	0.334	0.479
Mozambique	Namaacha	2017	0.076	0.111	0.159
Mozambique	Namaacha	2000-2017	-0.072	-0.064	-0.057
Mozambique	Namacurra	2000	0.224	0.326	0.485
Mozambique	Namacurra	2017	0.069	0.101	0.148
Mozambique	Namacurra	2000-2017	-0.075	-0.069	-0.062
Mozambique	Namarroi	2000	0.244	0.363	0.547
Mozambique	Namarroi	2017	0.071	0.107	0.157
Mozambique	Namarroi	2000-2017	-0.079	-0.073	-0.066
Mozambique	Nampula	2000	0.214	0.317	0.471
Mozambique	Nampula	2017	0.069	0.101	0.151
Mozambique	Nampula	2000-2017	-0.079	-0.066	-0.056
Mozambique	Namuno	2000	0.230	0.337	0.492
Mozambique	Namuno	2017	0.068	0.102	0.154
Mozambique	Namuno	2000-2017	-0.076	-0.070	-0.064
Mozambique	Nangade	2000	0.249	0.349	0.505
Mozambique	Nangade	2017	0.072	0.106	0.159
Mozambique	Nangade	2000-2017	-0.079	-0.071	-0.064
Mozambique	Nhamatanda	2000	0.225	0.333	0.474
Mozambique	Nhamatanda	2017	0.070	0.104	0.148
Mozambique	Nhamatanda	2000-2017	-0.075	-0.067	-0.058
Mozambique	Nicoadala	2000	0.219	0.327	0.480
Mozambique	Nicoadala	2017	0.070	0.103	0.154
Mozambique	Nicoadala	2000-2017	-0.079	-0.068	-0.060
Mozambique	Nipepe	2000	0.227	0.332	0.483
Mozambique	Nipepe	2017	0.068	0.102	0.151
Mozambique	Nipepe	2000-2017	-0.074	-0.067	-0.061
Mozambique	Palma	2000	0.233	0.334	0.484
Mozambique	Palma	2017	0.072	0.107	0.155
Mozambique	Palma	2000-2017	-0.075	-0.068	-0.060
Mozambique	Panda	2000	0.231	0.341	0.493
Mozambique	Panda	2017	0.074	0.110	0.164
Mozambique	Panda	2000-2017	-0.072	-0.066	-0.060
Mozambique	Pebane	2000	0.216	0.321	0.471
Mozambique	Pebane	2017	0.070	0.102	0.152
Mozambique	Pebane	2000-2017	-0.071	-0.066	-0.061
Mozambique	Pemba	2000	0.214	0.328	0.470
Mozambique	Pemba	2017	0.070	0.104	0.154
Mozambique	Pemba	2000-2017	-0.082	-0.066	-0.051
Mozambique	Quissanga	2000	0.226	0.339	0.504
Mozambique	Quissanga	2017	0.070	0.105	0.158
Mozambique	Quissanga	2000-2017	-0.078	-0.069	-0.061
Mozambique	Ribaue	2000	0.212	0.317	0.471
Mozambique	Ribaue	2017	0.069	0.102	0.151
Mozambique	Ribaue	2000-2017	-0.071	-0.065	-0.058
Mozambique	Sanga	2000	0.263	0.385	0.560
Mozambique	Sanga	2017	0.068	0.100	0.149
Mozambique	Sanga	2000-2017	-0.086	-0.079	-0.072
Mozambique	Sussundenga	2000	0.232	0.340	0.497
Mozambique	Sussundenga	2017	0.072	0.106	0.153
Mozambique	Sussundenga	2000-2017	-0.073	-0.067	-0.061

Table 1: LRI DALYs rate by unit *(continued)*

Country	Unit	year	mean rate	lower rate	upper rate
Mozambique	Tambara	2000	0.223	0.329	0.480
Mozambique	Tambara	2017	0.069	0.102	0.150
Mozambique	Tambara	2000-2017	-0.077	-0.068	-0.060
Mozambique	Tsangano	2000	0.296	0.425	0.612
Mozambique	Tsangano	2017	0.077	0.109	0.158
Mozambique	Tsangano	2000-2017	-0.086	-0.077	-0.069
Mozambique	Vilanculos	2000	0.233	0.344	0.494
Mozambique	Vilanculos	2017	0.073	0.109	0.160
Mozambique	Vilanculos	2000-2017	-0.074	-0.067	-0.060
Mozambique	Xai-Xai	2000	0.237	0.351	0.518
Mozambique	Xai-Xai	2017	0.078	0.117	0.171
Mozambique	Xai-Xai	2000-2017	-0.075	-0.065	-0.056
Mozambique	Zavala	2000	0.228	0.337	0.489
Mozambique	Zavala	2017	0.072	0.109	0.160
Mozambique	Zavala	2000-2017	-0.074	-0.065	-0.057
Mozambique	Zumbu	2000	0.257	0.366	0.537
Mozambique	Zumbu	2017	0.070	0.101	0.146
Mozambique	Zumbu	2000-2017	-0.080	-0.073	-0.067
Namibia	Aminius	2000	0.044	0.082	0.125
Namibia	Aminius	2017	0.045	0.087	0.146
Namibia	Aminius	2000-2017	-0.033	0.007	0.046
Namibia	Anamulenge	2000	0.051	0.097	0.168
Namibia	Anamulenge	2017	0.041	0.079	0.128
Namibia	Anamulenge	2000-2017	-0.058	-0.012	0.029
Namibia	Arandis	2000	0.042	0.086	0.147
Namibia	Arandis	2017	0.041	0.089	0.160
Namibia	Arandis	2000-2017	-0.050	0.006	0.062
Namibia	Berseba	2000	0.043	0.078	0.128
Namibia	Berseba	2017	0.042	0.078	0.129
Namibia	Berseba	2000-2017	-0.032	0.003	0.034
Namibia	Daures	2000	0.045	0.081	0.124
Namibia	Daures	2017	0.042	0.082	0.138
Namibia	Daures	2000-2017	-0.034	0.004	0.037
Namibia	Eenhana	2000	0.053	0.106	0.170
Namibia	Eenhana	2017	0.043	0.083	0.141
Namibia	Eenhana	2000-2017	-0.062	-0.018	0.024
Namibia	Elim	2000	0.047	0.096	0.161
Namibia	Elim	2017	0.042	0.080	0.129
Namibia	Elim	2000-2017	-0.060	-0.013	0.032
Namibia	Endola	2000	0.051	0.107	0.184
Namibia	Endola	2017	0.042	0.084	0.140
Namibia	Endola	2000-2017	-0.061	-0.016	0.022
Namibia	Engela	2000	0.054	0.105	0.174
Namibia	Engela	2017	0.039	0.083	0.150
Namibia	Engela	2000-2017	-0.060	-0.012	0.033
Namibia	Engodi	2000	0.064	0.125	0.210
Namibia	Engodi	2017	0.049	0.098	0.169
Namibia	Engodi	2000-2017	-0.063	-0.016	0.024
Namibia	Epembe	2000	0.050	0.098	0.158
Namibia	Epembe	2017	0.041	0.078	0.135
Namibia	Epembe	2000-2017	-0.058	-0.017	0.026
Namibia	Epukiro	2000	0.050	0.087	0.136
Namibia	Epukiro	2017	0.046	0.091	0.159
Namibia	Epukiro	2000-2017	-0.042	0.005	0.044
Namibia	Epupa	2000	0.052	0.093	0.145
Namibia	Epupa	2017	0.041	0.087	0.158
Namibia	Epupa	2000-2017	-0.039	-0.004	0.027
Namibia	Etayi	2000	0.045	0.092	0.147
Namibia	Etayi	2017	0.039	0.079	0.133
Namibia	Etayi	2000-2017	-0.049	-0.008	0.030
Namibia	Gibeon	2000	0.045	0.082	0.130

Table 1: LRI DALYs rate by unit *(continued)*

Country	Unit	year	mean rate	lower rate	upper rate
Namibia	Gibeon	2017	0.041	0.080	0.136
Namibia	Gibeon	2000-2017	-0.034	0.003	0.040
Namibia	Gobabis	2000	0.037	0.080	0.170
Namibia	Gobabis	2017	0.033	0.083	0.165
Namibia	Gobabis	2000-2017	-0.075	0.003	0.071
Namibia	Grootfontein	2000	0.046	0.087	0.149
Namibia	Grootfontein	2017	0.039	0.083	0.148
Namibia	Grootfontein	2000-2017	-0.050	0.001	0.056
Namibia	Guinas	2000	0.061	0.110	0.180
Namibia	Guinas	2017	0.043	0.087	0.152
Namibia	Guinas	2000-2017	-0.057	-0.014	0.026
Namibia	Kabe	2000	0.050	0.094	0.149
Namibia	Kabe	2017	0.045	0.090	0.152
Namibia	Kabe	2000-2017	-0.040	0.002	0.040
Namibia	Kahenge	2000	0.059	0.119	0.203
Namibia	Kahenge	2017	0.055	0.106	0.177
Namibia	Kahenge	2000-2017	-0.047	-0.002	0.046
Namibia	Kalahari	2000	0.043	0.080	0.129
Namibia	Kalahari	2017	0.044	0.085	0.148
Namibia	Kalahari	2000-2017	-0.037	0.007	0.045
Namibia	Kamanjab	2000	0.048	0.085	0.133
Namibia	Kamanjab	2017	0.038	0.080	0.152
Namibia	Kamanjab	2000-2017	-0.038	-0.004	0.030
Namibia	Kapako	2000	0.056	0.117	0.199
Namibia	Kapako	2017	0.050	0.102	0.174
Namibia	Kapako	2000-2017	-0.055	-0.004	0.047
Namibia	Karas	2000	0.045	0.077	0.125
Namibia	Karas	2017	0.043	0.080	0.141
Namibia	Karas	2000-2017	-0.040	0.007	0.043
Namibia	Karibib	2000	0.043	0.079	0.138
Namibia	Karibib	2017	0.043	0.084	0.147
Namibia	Karibib	2000-2017	-0.038	0.007	0.050
Namibia	Katima Muliro Rural	2000	0.053	0.102	0.175
Namibia	Katima Muliro Rural	2017	0.056	0.103	0.190
Namibia	Katima Muliro Rural	2000-2017	-0.038	0.005	0.045
Namibia	Katima Muliro Urban	2000	0.038	0.117	0.241
Namibia	Katima Muliro Urban	2017	0.047	0.117	0.252
Namibia	Katima Muliro Urban	2000-2017	-0.075	0.003	0.088
Namibia	Katutura Central	2000	0.039	0.102	0.227
Namibia	Katutura Central	2017	0.028	0.075	0.167
Namibia	Katutura Central	2000-2017	-0.091	-0.012	0.079
Namibia	Katutura East	2000	0.039	0.102	0.227
Namibia	Katutura East	2017	0.028	0.075	0.167
Namibia	Katutura East	2000-2017	-0.091	-0.012	0.079
Namibia	Keetmanshoop Rural	2000	0.039	0.077	0.128
Namibia	Keetmanshoop Rural	2017	0.039	0.078	0.129
Namibia	Keetmanshoop Rural	2000-2017	-0.038	0.006	0.043
Namibia	Keetmanshoop Urban	2000	0.040	0.079	0.146
Namibia	Keetmanshoop Urban	2017	0.038	0.081	0.148
Namibia	Keetmanshoop Urban	2000-2017	-0.061	0.005	0.070
Namibia	Khomasdal North	2000	0.049	0.100	0.180
Namibia	Khomasdal North	2017	0.034	0.075	0.140
Namibia	Khomasdal North	2000-2017	-0.073	-0.011	0.041
Namibia	Khorixas	2000	0.053	0.094	0.155
Namibia	Khorixas	2017	0.042	0.099	0.189
Namibia	Khorixas	2000-2017	-0.040	0.004	0.049
Namibia	Kongola	2000	0.050	0.095	0.155
Namibia	Kongola	2017	0.048	0.091	0.156
Namibia	Kongola	2000-2017	-0.039	0.003	0.042
Namibia	Linyandi	2000	0.052	0.095	0.157
Namibia	Linyandi	2017	0.050	0.098	0.165

Table 1: LRI DALYs rate by unit *(continued)*

Country	Unit	year	mean rate	lower rate	upper rate
Namibia	Linyandi	2000-2017	-0.040	0.007	0.045
Namibia	Luderitz	2000	0.041	0.085	0.155
Namibia	Luderitz	2017	0.031	0.082	0.158
Namibia	Luderitz	2000-2017	-0.071	-0.003	0.058
Namibia	Mariental Rural	2000	0.041	0.080	0.141
Namibia	Mariental Rural	2017	0.041	0.078	0.127
Namibia	Mariental Rural	2000-2017	-0.044	0.003	0.048
Namibia	Mariental Urban	2000	0.043	0.080	0.141
Namibia	Mariental Urban	2017	0.040	0.078	0.132
Namibia	Mariental Urban	2000-2017	-0.046	0.002	0.052
Namibia	Mashare	2000	0.060	0.124	0.216
Namibia	Mashare	2017	0.048	0.105	0.185
Namibia	Mashare	2000-2017	-0.052	-0.004	0.042
Namibia	Moses Garoeb	2000	0.045	0.099	0.170
Namibia	Moses Garoeb	2017	0.033	0.076	0.140
Namibia	Moses Garoeb	2000-2017	-0.073	-0.012	0.050
Namibia	Mpungu	2000	0.057	0.114	0.194
Namibia	Mpungu	2017	0.046	0.095	0.166
Namibia	Mpungu	2000-2017	-0.062	-0.013	0.036
Namibia	Mukwe	2000	0.064	0.135	0.232
Namibia	Mukwe	2017	0.057	0.113	0.199
Namibia	Mukwe	2000-2017	-0.051	-0.007	0.038
Namibia	Ndiyona	2000	0.061	0.129	0.222
Namibia	Ndiyona	2017	0.053	0.110	0.183
Namibia	Ndiyona	2000-2017	-0.061	-0.004	0.044
Namibia	Ogongo	2000	0.046	0.088	0.143
Namibia	Ogongo	2017	0.041	0.079	0.127
Namibia	Ogongo	2000-2017	-0.050	-0.007	0.031
Namibia	Ohangwena	2000	0.057	0.113	0.204
Namibia	Ohangwena	2017	0.039	0.086	0.145
Namibia	Ohangwena	2000-2017	-0.065	-0.017	0.029
Namibia	Okahandja	2000	0.039	0.082	0.158
Namibia	Okahandja	2017	0.034	0.078	0.163
Namibia	Okahandja	2000-2017	-0.067	-0.002	0.066
Namibia	Okahao	2000	0.047	0.095	0.161
Namibia	Okahao	2017	0.041	0.079	0.134
Namibia	Okahao	2000-2017	-0.053	-0.009	0.038
Namibia	Okakarara	2000	0.049	0.093	0.149
Namibia	Okakarara	2017	0.045	0.089	0.153
Namibia	Okakarara	2000-2017	-0.041	0.001	0.041
Namibia	Okaku	2000	0.039	0.088	0.164
Namibia	Okaku	2017	0.040	0.074	0.115
Namibia	Okaku	2000-2017	-0.056	-0.012	0.030
Namibia	Okalongo	2000	0.048	0.093	0.158
Namibia	Okalongo	2017	0.040	0.078	0.128
Namibia	Okalongo	2000-2017	-0.055	-0.008	0.032
Namibia	Okankolo	2000	0.061	0.122	0.207
Namibia	Okankolo	2017	0.051	0.095	0.158
Namibia	Okankolo	2000-2017	-0.062	-0.016	0.021
Namibia	Okatana	2000	0.038	0.084	0.150
Namibia	Okatana	2017	0.038	0.075	0.127
Namibia	Okatana	2000-2017	-0.055	-0.007	0.034
Namibia	Okatyali	2000	0.039	0.084	0.142
Namibia	Okatyali	2017	0.039	0.074	0.126
Namibia	Okatyali	2000-2017	-0.049	-0.004	0.045
Namibia	Okongo	2000	0.053	0.099	0.160
Namibia	Okongo	2017	0.039	0.081	0.146
Namibia	Okongo	2000-2017	-0.062	-0.014	0.027
Namibia	Olukonda	2000	0.047	0.094	0.172
Namibia	Olukonda	2017	0.041	0.080	0.143
Namibia	Olukonda	2000-2017	-0.071	-0.011	0.037

Table 1: LRI DALYs rate by unit *(continued)*

Country	Unit	year	mean rate	lower rate	upper rate
Namibia	Omaruru	2000	0.038	0.077	0.144
Namibia	Omaruru	2017	0.036	0.074	0.147
Namibia	Omaruru	2000-2017	-0.066	-0.001	0.058
Namibia	Omatako	2000	0.049	0.091	0.142
Namibia	Omatako	2017	0.044	0.085	0.153
Namibia	Omatako	2000-2017	-0.042	-0.001	0.037
Namibia	Ompundja	2000	0.037	0.083	0.151
Namibia	Ompundja	2017	0.040	0.073	0.119
Namibia	Ompundja	2000-2017	-0.044	-0.004	0.039
Namibia	Omulonga	2000	0.055	0.107	0.176
Namibia	Omulonga	2017	0.042	0.084	0.139
Namibia	Omulonga	2000-2017	-0.062	-0.016	0.024
Namibia	Omundaungilo	2000	0.052	0.096	0.147
Namibia	Omundaungilo	2017	0.040	0.079	0.143
Namibia	Omundaungilo	2000-2017	-0.058	-0.013	0.028
Namibia	Omuntele	2000	0.050	0.106	0.170
Namibia	Omuntele	2017	0.044	0.083	0.137
Namibia	Omuntele	2000-2017	-0.058	-0.016	0.024
Namibia	Omuthiyagwipundi	2000	0.052	0.105	0.171
Namibia	Omuthiyagwipundi	2017	0.045	0.085	0.143
Namibia	Omuthiyagwipundi	2000-2017	-0.057	-0.014	0.026
Namibia	Onayena	2000	0.048	0.101	0.181
Namibia	Onayena	2017	0.044	0.081	0.129
Namibia	Onayena	2000-2017	-0.063	-0.016	0.028
Namibia	Ondangwa	2000	0.038	0.082	0.153
Namibia	Ondangwa	2017	0.037	0.073	0.127
Namibia	Ondangwa	2000-2017	-0.053	-0.005	0.047
Namibia	Ondobe	2000	0.051	0.100	0.162
Namibia	Ondobe	2017	0.040	0.082	0.140
Namibia	Ondobe	2000-2017	-0.060	-0.012	0.031
Namibia	Onesi	2000	0.049	0.091	0.149
Namibia	Onesi	2017	0.042	0.082	0.134
Namibia	Onesi	2000-2017	-0.050	-0.008	0.032
Namibia	Ongenga	2000	0.052	0.102	0.171
Namibia	Ongenga	2017	0.040	0.082	0.139
Namibia	Ongenga	2000-2017	-0.051	-0.013	0.030
Namibia	Ongwediva	2000	0.037	0.082	0.152
Namibia	Ongwediva	2017	0.038	0.073	0.122
Namibia	Ongwediva	2000-2017	-0.059	-0.006	0.044
Namibia	Oniipa	2000	0.044	0.092	0.166
Namibia	Oniipa	2017	0.044	0.079	0.131
Namibia	Oniipa	2000-2017	-0.054	-0.009	0.033
Namibia	Onyaanya	2000	0.058	0.118	0.207
Namibia	Onyaanya	2017	0.049	0.092	0.150
Namibia	Onyaanya	2000-2017	-0.057	-0.016	0.024
Namibia	Opuwo	2000	0.052	0.094	0.144
Namibia	Opuwo	2017	0.041	0.087	0.159
Namibia	Opuwo	2000-2017	-0.042	-0.003	0.037
Namibia	Oranjemund	2000	0.045	0.085	0.143
Namibia	Oranjemund	2017	0.040	0.080	0.152
Namibia	Oranjemund	2000-2017	-0.050	0.001	0.046
Namibia	Oshakati East	2000	0.039	0.079	0.148
Namibia	Oshakati East	2017	0.037	0.074	0.126
Namibia	Oshakati East	2000-2017	-0.049	-0.003	0.042
Namibia	Oshakati West	2000	0.034	0.082	0.167
Namibia	Oshakati West	2017	0.036	0.077	0.155
Namibia	Oshakati West	2000-2017	-0.062	0.001	0.064
Namibia	Oshikango	2000	0.055	0.104	0.177
Namibia	Oshikango	2017	0.039	0.082	0.146
Namibia	Oshikango	2000-2017	-0.065	-0.013	0.034
Namibia	Oshikuku	2000	0.044	0.089	0.148

Table 1: LRI DALYs rate by unit *(continued)*

Country	Unit	year	mean rate	lower rate	upper rate
Namibia	Oshikuku	2017	0.039	0.080	0.132
Namibia	Oshikuku	2000-2017	-0.056	-0.010	0.036
Namibia	Otamanzi	2000	0.044	0.088	0.145
Namibia	Otamanzi	2017	0.043	0.079	0.129
Namibia	Otamanzi	2000-2017	-0.051	-0.005	0.037
Namibia	Otavi	2000	0.049	0.091	0.150
Namibia	Otavi	2017	0.036	0.082	0.152
Namibia	Otavi	2000-2017	-0.048	-0.004	0.045
Namibia	Otjinene	2000	0.047	0.090	0.141
Namibia	Otjinene	2017	0.045	0.092	0.178
Namibia	Otjinene	2000-2017	-0.044	0.003	0.045
Namibia	Otjiwarongo	2000	0.039	0.085	0.152
Namibia	Otjiwarongo	2017	0.036	0.082	0.152
Namibia	Otjiwarongo	2000-2017	-0.055	0.001	0.061
Namibia	Otjombinde	2000	0.048	0.086	0.133
Namibia	Otjombinde	2017	0.043	0.091	0.162
Namibia	Otjombinde	2000-2017	-0.036	0.006	0.045
Namibia	Outapi	2000	0.052	0.097	0.161
Namibia	Outapi	2017	0.043	0.082	0.132
Namibia	Outapi	2000-2017	-0.058	-0.010	0.028
Namibia	Outjo	2000	0.052	0.101	0.168
Namibia	Outjo	2017	0.042	0.094	0.181
Namibia	Outjo	2000-2017	-0.057	-0.004	0.048
Namibia	Rehoboth East	2000	0.036	0.082	0.168
Namibia	Rehoboth East	2017	0.038	0.087	0.176
Namibia	Rehoboth East	2000-2017	-0.071	0.006	0.077
Namibia	Rehoboth Rural	2000	0.047	0.082	0.130
Namibia	Rehoboth Rural	2017	0.044	0.083	0.140
Namibia	Rehoboth Rural	2000-2017	-0.033	0.004	0.038
Namibia	Rehoboth West	2000	0.038	0.078	0.140
Namibia	Rehoboth West	2017	0.035	0.084	0.173
Namibia	Rehoboth West	2000-2017	-0.063	0.009	0.079
Namibia	Ruacana	2000	0.053	0.096	0.156
Namibia	Ruacana	2017	0.046	0.090	0.148
Namibia	Ruacana	2000-2017	-0.040	-0.003	0.035
Namibia	Rundu Rural East	2000	0.049	0.117	0.232
Namibia	Rundu Rural East	2017	0.044	0.093	0.165
Namibia	Rundu Rural East	2000-2017	-0.067	-0.007	0.044
Namibia	Rundu Rural West	2000	0.053	0.119	0.238
Namibia	Rundu Rural West	2017	0.046	0.097	0.191
Namibia	Rundu Rural West	2000-2017	-0.076	-0.007	0.047
Namibia	Rundu Urban	2000	0.048	0.117	0.251
Namibia	Rundu Urban	2017	0.044	0.094	0.185
Namibia	Rundu Urban	2000-2017	-0.080	-0.009	0.059
Namibia	Sesfontein	2000	0.052	0.095	0.149
Namibia	Sesfontein	2017	0.044	0.093	0.168
Namibia	Sesfontein	2000-2017	-0.033	0.000	0.029
Namibia	Sibinda	2000	0.052	0.101	0.166
Namibia	Sibinda	2017	0.050	0.103	0.182
Namibia	Sibinda	2000-2017	-0.037	0.006	0.048
Namibia	Soweto	2000	0.043	0.101	0.226
Namibia	Soweto	2017	0.030	0.075	0.159
Namibia	Soweto	2000-2017	-0.087	-0.012	0.071
Namibia	Steinhausen	2000	0.046	0.087	0.140
Namibia	Steinhausen	2017	0.045	0.088	0.153
Namibia	Steinhausen	2000-2017	-0.045	0.003	0.046
Namibia	Swakopmund	2000	0.036	0.078	0.131
Namibia	Swakopmund	2017	0.034	0.074	0.145
Namibia	Swakopmund	2000-2017	-0.054	-0.001	0.057
Namibia	Tobias Hainyeko	2000	0.045	0.102	0.215
Namibia	Tobias Hainyeko	2017	0.032	0.076	0.160

Table 1: LRI DALYs rate by unit *(continued)*

Country	Unit	year	mean rate	lower rate	upper rate
Namibia	Tobias Hainyeko	2000-2017	-0.078	-0.012	0.063
Namibia	Tsandi	2000	0.047	0.089	0.139
Namibia	Tsandi	2017	0.042	0.080	0.132
Namibia	Tsandi	2000-2017	-0.044	-0.006	0.029
Namibia	Tsumeb	2000	0.052	0.107	0.203
Namibia	Tsumeb	2017	0.038	0.084	0.159
Namibia	Tsumeb	2000-2017	-0.075	-0.015	0.038
Namibia	Tsumkwe	2000	0.048	0.088	0.144
Namibia	Tsumkwe	2017	0.046	0.089	0.145
Namibia	Tsumkwe	2000-2017	-0.038	0.004	0.042
Namibia	Uukwiyu	2000	0.036	0.085	0.158
Namibia	Uukwiyu	2017	0.038	0.073	0.121
Namibia	Uukwiyu	2000-2017	-0.055	-0.006	0.045
Namibia	Uuvudhiya	2000	0.041	0.082	0.137
Namibia	Uuvudhiya	2017	0.042	0.076	0.123
Namibia	Uuvudhiya	2000-2017	-0.043	-0.003	0.032
Namibia	Walvisbay Rural	2000	0.041	0.080	0.145
Namibia	Walvisbay Rural	2017	0.038	0.074	0.126
Namibia	Walvisbay Rural	2000-2017	-0.042	-0.002	0.042
Namibia	Walvisbay Urban	2000	0.032	0.079	0.162
Namibia	Walvisbay Urban	2017	0.029	0.072	0.143
Namibia	Walvisbay Urban	2000-2017	-0.076	-0.002	0.076
Namibia	Wanaheda	2000	0.035	0.098	0.192
Namibia	Wanaheda	2017	0.028	0.075	0.152
Namibia	Wanaheda	2000-2017	-0.094	-0.012	0.074
Namibia	Windhoek East	2000	0.047	0.095	0.174
Namibia	Windhoek East	2017	0.036	0.073	0.134
Namibia	Windhoek East	2000-2017	-0.068	-0.009	0.042
Namibia	Windhoek Rural	2000	0.050	0.093	0.175
Namibia	Windhoek Rural	2017	0.037	0.075	0.137
Namibia	Windhoek Rural	2000-2017	-0.052	-0.007	0.043
Namibia	Windhoek West	2000	0.045	0.100	0.190
Namibia	Windhoek West	2017	0.038	0.075	0.141
Namibia	Windhoek West	2000-2017	-0.075	-0.011	0.039
Niger	Aguié	2000	0.451	0.678	1.029
Niger	Aguié	2017	0.168	0.290	0.468
Niger	Aguié	2000-2017	-0.082	-0.051	-0.019
Niger	Arlit	2000	0.336	0.590	0.906
Niger	Arlit	2017	0.147	0.277	0.477
Niger	Arlit	2000-2017	-0.081	-0.044	-0.012
Niger	Bilma	2000	0.303	0.550	0.913
Niger	Bilma	2017	0.151	0.291	0.491
Niger	Bilma	2000-2017	-0.071	-0.035	-0.004
Niger	Bkonni	2000	0.461	0.687	1.042
Niger	Bkonni	2017	0.164	0.276	0.431
Niger	Bkonni	2000-2017	-0.085	-0.056	-0.022
Niger	Boboye	2000	0.470	0.708	1.046
Niger	Boboye	2017	0.151	0.261	0.407
Niger	Boboye	2000-2017	-0.093	-0.061	-0.031
Niger	Bouza	2000	0.472	0.712	1.123
Niger	Bouza	2017	0.176	0.295	0.497
Niger	Bouza	2000-2017	-0.084	-0.054	-0.020
Niger	Dakoro	2000	0.455	0.698	1.086
Niger	Dakoro	2017	0.179	0.303	0.503
Niger	Dakoro	2000-2017	-0.078	-0.050	-0.017
Niger	Diffa	2000	0.446	0.707	1.046
Niger	Diffa	2017	0.207	0.373	0.613
Niger	Diffa	2000-2017	-0.068	-0.037	-0.010
Niger	Dogon-Doutchi	2000	0.462	0.705	1.071
Niger	Dogon-Doutchi	2017	0.142	0.242	0.391
Niger	Dogon-Doutchi	2000-2017	-0.095	-0.064	-0.032

Table 1: LRI DALYs rate by unit *(continued)*

Country	Unit	year	mean rate	lower rate	upper rate
Niger	Dosso	2000	0.469	0.699	1.050
Niger	Dosso	2017	0.131	0.227	0.359
Niger	Dosso	2000-2017	-0.100	-0.067	-0.038
Niger	Filingué	2000	0.501	0.745	1.101
Niger	Filingué	2017	0.153	0.260	0.411
Niger	Filingué	2000-2017	-0.093	-0.063	-0.031
Niger	Gaya	2000	0.437	0.665	0.988
Niger	Gaya	2017	0.139	0.240	0.387
Niger	Gaya	2000-2017	-0.092	-0.062	-0.033
Niger	Gouré	2000	0.467	0.738	1.074
Niger	Gouré	2017	0.207	0.359	0.587
Niger	Gouré	2000-2017	-0.072	-0.043	-0.015
Niger	Groumdji	2000	0.452	0.670	1.048
Niger	Groumdji	2017	0.160	0.281	0.447
Niger	Groumdji	2000-2017	-0.084	-0.053	-0.020
Niger	Illéla	2000	0.481	0.720	1.102
Niger	Illéla	2017	0.171	0.287	0.456
Niger	Illéla	2000-2017	-0.084	-0.056	-0.023
Niger	Keita	2000	0.491	0.734	1.105
Niger	Keita	2017	0.173	0.291	0.487
Niger	Keita	2000-2017	-0.086	-0.056	-0.023
Niger	Kollo	2000	0.462	0.702	1.047
Niger	Kollo	2017	0.131	0.226	0.346
Niger	Kollo	2000-2017	-0.102	-0.069	-0.040
Niger	Loga	2000	0.469	0.717	1.063
Niger	Loga	2017	0.136	0.238	0.369
Niger	Loga	2000-2017	-0.100	-0.066	-0.036
Niger	Madaoua	2000	0.446	0.668	1.031
Niger	Madaoua	2017	0.168	0.283	0.455
Niger	Madaoua	2000-2017	-0.083	-0.053	-0.021
Niger	Madarounfa	2000	0.440	0.655	0.981
Niger	Madarounfa	2017	0.161	0.278	0.450
Niger	Madarounfa	2000-2017	-0.085	-0.052	-0.020
Niger	Magaria	2000	0.433	0.691	0.996
Niger	Magaria	2017	0.185	0.311	0.505
Niger	Magaria	2000-2017	-0.078	-0.048	-0.020
Niger	Maïné-Soroa	2000	0.479	0.754	1.130
Niger	Maïné-Soroa	2017	0.213	0.383	0.611
Niger	Maïné-Soroa	2000-2017	-0.071	-0.040	-0.013
Niger	Matameye	2000	0.448	0.693	1.039
Niger	Matameye	2017	0.175	0.304	0.490
Niger	Matameye	2000-2017	-0.082	-0.050	-0.019
Niger	Mayahi	2000	0.456	0.698	1.065
Niger	Mayahi	2017	0.174	0.302	0.498
Niger	Mayahi	2000-2017	-0.079	-0.051	-0.018
Niger	Mirriah	2000	0.453	0.723	1.077
Niger	Mirriah	2017	0.193	0.328	0.532
Niger	Mirriah	2000-2017	-0.077	-0.047	-0.019
Niger	N'Guigmi	2000	0.454	0.747	1.135
Niger	N'Guigmi	2017	0.212	0.380	0.646
Niger	N'Guigmi	2000-2017	-0.072	-0.040	-0.011
Niger	Niamey	2000	0.337	0.511	0.776
Niger	Niamey	2017	0.118	0.212	0.332
Niger	Niamey	2000-2017	-0.086	-0.053	-0.018
Niger	Ouallam	2000	0.497	0.751	1.128
Niger	Ouallam	2017	0.152	0.263	0.405
Niger	Ouallam	2000-2017	-0.094	-0.062	-0.030
Niger	Say	2000	0.476	0.699	1.049
Niger	Say	2017	0.133	0.228	0.346
Niger	Say	2000-2017	-0.099	-0.068	-0.040
Niger	Tahoua	2000	0.475	0.735	1.103

Table 1: LRI DALYs rate by unit *(continued)*

Country	Unit	year	mean rate	lower rate	upper rate
Niger	Tahoua	2017	0.168	0.277	0.444
Niger	Tahoua	2000-2017	-0.087	-0.059	-0.025
Niger	Tanout	2000	0.454	0.719	1.065
Niger	Tanout	2017	0.192	0.334	0.555
Niger	Tanout	2000-2017	-0.076	-0.046	-0.015
Niger	Tchighozerine	2000	0.380	0.623	0.895
Niger	Tchighozerine	2017	0.161	0.275	0.455
Niger	Tchighozerine	2000-2017	-0.083	-0.048	-0.015
Niger	Tchin-Tabarade	2000	0.472	0.732	1.091
Niger	Tchin-Tabarade	2017	0.182	0.306	0.517
Niger	Tchin-Tabarade	2000-2017	-0.082	-0.053	-0.019
Niger	Téra	2000	0.479	0.755	1.156
Niger	Téra	2017	0.145	0.253	0.386
Niger	Téra	2000-2017	-0.099	-0.066	-0.038
Niger	Tessaoua	2000	0.447	0.692	1.034
Niger	Tessaoua	2017	0.174	0.302	0.495
Niger	Tessaoua	2000-2017	-0.081	-0.050	-0.017
Niger	Tillabéry	2000	0.483	0.755	1.127
Niger	Tillabéry	2017	0.149	0.253	0.396
Niger	Tillabéry	2000-2017	-0.098	-0.066	-0.038
Nigeria	Aba North	2000	0.309	0.589	0.921
Nigeria	Aba North	2017	0.157	0.267	0.437
Nigeria	Aba North	2000-2017	-0.084	-0.043	0.007
Nigeria	Aba South	2000	0.390	0.639	0.974
Nigeria	Aba South	2017	0.166	0.272	0.420
Nigeria	Aba South	2000-2017	-0.084	-0.049	-0.004
Nigeria	Abadam	2000	0.689	1.109	1.611
Nigeria	Abadam	2017	0.423	0.667	0.969
Nigeria	Abadam	2000-2017	-0.052	-0.027	-0.001
Nigeria	Abaji	2000	0.482	0.768	1.092
Nigeria	Abaji	2017	0.205	0.309	0.468
Nigeria	Abaji	2000-2017	-0.081	-0.053	-0.025
Nigeria	Abak	2000	0.429	0.757	1.193
Nigeria	Abak	2017	0.181	0.294	0.452
Nigeria	Abak	2000-2017	-0.084	-0.056	-0.017
Nigeria	Abakalik	2000	0.481	0.843	1.297
Nigeria	Abakalik	2017	0.205	0.344	0.527
Nigeria	Abakalik	2000-2017	-0.085	-0.053	-0.021
Nigeria	Abeokuta South	2000	0.343	0.577	0.903
Nigeria	Abeokuta South	2017	0.147	0.247	0.393
Nigeria	Abeokuta South	2000-2017	-0.088	-0.050	-0.012
Nigeria	AbeokutaNorth	2000	0.425	0.681	1.004
Nigeria	AbeokutaNorth	2017	0.154	0.247	0.379
Nigeria	AbeokutaNorth	2000-2017	-0.088	-0.060	-0.032
Nigeria	Abi	2000	0.494	0.777	1.143
Nigeria	Abi	2017	0.195	0.303	0.448
Nigeria	Abi	2000-2017	-0.084	-0.054	-0.024
Nigeria	Aboh-Mba	2000	0.437	0.657	0.979
Nigeria	Aboh-Mba	2017	0.177	0.266	0.376
Nigeria	Aboh-Mba	2000-2017	-0.083	-0.050	-0.019
Nigeria	Abua/Odu	2000	0.455	0.727	1.109
Nigeria	Abua/Odu	2017	0.182	0.287	0.427
Nigeria	Abua/Odu	2000-2017	-0.083	-0.055	-0.024
Nigeria	AbujaMun	2000	0.484	0.751	1.070
Nigeria	AbujaMun	2017	0.200	0.317	0.463
Nigeria	AbujaMun	2000-2017	-0.076	-0.049	-0.022
Nigeria	Adavi	2000	0.464	0.742	1.124
Nigeria	Adavi	2017	0.191	0.285	0.422
Nigeria	Adavi	2000-2017	-0.081	-0.057	-0.025
Nigeria	Ado	2000	0.523	0.818	1.178
Nigeria	Ado	2017	0.208	0.321	0.490

Table 1: LRI DALYs rate by unit *(continued)*

Country	Unit	year	mean rate	lower rate	upper rate
Nigeria	Ado	2000-2017	-0.084	-0.056	-0.029
Nigeria	Ado-Ekiti	2000	0.349	0.561	0.843
Nigeria	Ado-Ekiti	2017	0.154	0.227	0.334
Nigeria	Ado-Ekiti	2000-2017	-0.082	-0.054	-0.023
Nigeria	AdoOdo/Ota	2000	0.364	0.591	0.881
Nigeria	AdoOdo/Ota	2017	0.161	0.252	0.375
Nigeria	AdoOdo/Ota	2000-2017	-0.077	-0.050	-0.021
Nigeria	Afijio	2000	0.357	0.596	0.873
Nigeria	Afijio	2017	0.138	0.223	0.333
Nigeria	Afijio	2000-2017	-0.085	-0.059	-0.034
Nigeria	Afikpo	2000	0.512	0.837	1.243
Nigeria	Afikpo	2017	0.205	0.328	0.511
Nigeria	Afikpo	2000-2017	-0.083	-0.054	-0.022
Nigeria	AfikpoSo	2000	0.514	0.855	1.279
Nigeria	AfikpoSo	2017	0.211	0.336	0.542
Nigeria	AfikpoSo	2000-2017	-0.087	-0.054	-0.022
Nigeria	Agaie	2000	0.478	0.764	1.097
Nigeria	Agaie	2017	0.212	0.327	0.463
Nigeria	Agaie	2000-2017	-0.076	-0.049	-0.021
Nigeria	Agatu	2000	0.521	0.806	1.163
Nigeria	Agatu	2017	0.212	0.320	0.477
Nigeria	Agatu	2000-2017	-0.082	-0.054	-0.027
Nigeria	Agege	2000	0.317	0.588	0.986
Nigeria	Agege	2017	0.151	0.248	0.397
Nigeria	Agege	2000-2017	-0.094	-0.049	-0.002
Nigeria	Aguata	2000	0.361	0.595	0.912
Nigeria	Aguata	2017	0.161	0.280	0.443
Nigeria	Aguata	2000-2017	-0.072	-0.042	-0.011
Nigeria	Agwara	2000	0.490	0.778	1.132
Nigeria	Agwara	2017	0.229	0.356	0.523
Nigeria	Agwara	2000-2017	-0.070	-0.045	-0.017
Nigeria	Ahizu-Mb	2000	0.385	0.625	0.963
Nigeria	Ahizu-Mb	2017	0.168	0.264	0.416
Nigeria	Ahizu-Mb	2000-2017	-0.084	-0.047	-0.011
Nigeria	Ahoada East	2000	0.430	0.671	1.017
Nigeria	Ahoada East	2017	0.165	0.260	0.396
Nigeria	Ahoada East	2000-2017	-0.080	-0.053	-0.019
Nigeria	Ahoada West	2000	0.440	0.705	1.064
Nigeria	Ahoada West	2017	0.171	0.276	0.424
Nigeria	Ahoada West	2000-2017	-0.082	-0.054	-0.023
Nigeria	Ajaokuta	2000	0.485	0.759	1.133
Nigeria	Ajaokuta	2017	0.190	0.287	0.427
Nigeria	Ajaokuta	2000-2017	-0.082	-0.058	-0.028
Nigeria	Ajeromi/Ifelodun	2000	0.291	0.533	0.863
Nigeria	Ajeromi/Ifelodun	2017	0.141	0.239	0.376
Nigeria	Ajeromi/Ifelodun	2000-2017	-0.083	-0.045	-0.003
Nigeria	Ajingi	2000	0.573	0.906	1.375
Nigeria	Ajingi	2017	0.340	0.514	0.742
Nigeria	Ajingi	2000-2017	-0.064	-0.032	0.003
Nigeria	Akamkpa	2000	0.502	0.805	1.149
Nigeria	Akamkpa	2017	0.198	0.313	0.470
Nigeria	Akamkpa	2000-2017	-0.084	-0.058	-0.031
Nigeria	Akinyele	2000	0.346	0.553	0.817
Nigeria	Akinyele	2017	0.140	0.221	0.334
Nigeria	Akinyele	2000-2017	-0.081	-0.054	-0.028
Nigeria	Akko	2000	0.622	1.022	1.525
Nigeria	Akko	2017	0.382	0.596	0.904
Nigeria	Akko	2000-2017	-0.059	-0.030	0.000
Nigeria	Akoko North-East	2000	0.360	0.612	0.915
Nigeria	Akoko North-East	2017	0.149	0.233	0.336
Nigeria	Akoko North-East	2000-2017	-0.081	-0.057	-0.027

Table 1: LRI DALYs rate by unit *(continued)*

Country	Unit	year	mean rate	lower rate	upper rate
Nigeria	Akoko South-East	2000	0.384	0.619	0.918
Nigeria	Akoko South-East	2017	0.151	0.231	0.339
Nigeria	Akoko South-East	2000-2017	-0.084	-0.059	-0.024
Nigeria	Akoko South-West	2000	0.387	0.615	0.930
Nigeria	Akoko South-West	2017	0.150	0.231	0.330
Nigeria	Akoko South-West	2000-2017	-0.088	-0.058	-0.029
Nigeria	Akoko-Ed	2000	0.409	0.647	0.976
Nigeria	Akoko-Ed	2017	0.161	0.245	0.351
Nigeria	Akoko-Ed	2000-2017	-0.083	-0.058	-0.030
Nigeria	AkokoNorthWest	2000	0.383	0.620	0.918
Nigeria	AkokoNorthWest	2017	0.149	0.233	0.331
Nigeria	AkokoNorthWest	2000-2017	-0.082	-0.058	-0.029
Nigeria	Akpabuyo	2000	0.486	0.775	1.124
Nigeria	Akpabuyo	2017	0.195	0.300	0.449
Nigeria	Akpabuyo	2000-2017	-0.084	-0.056	-0.024
Nigeria	Akukutor	2000	0.461	0.720	1.079
Nigeria	Akukutor	2017	0.179	0.281	0.434
Nigeria	Akukutor	2000-2017	-0.088	-0.057	-0.025
Nigeria	Akure North	2000	0.352	0.584	0.897
Nigeria	Akure North	2017	0.154	0.225	0.327
Nigeria	Akure North	2000-2017	-0.081	-0.056	-0.027
Nigeria	Akure South	2000	0.334	0.554	0.844
Nigeria	Akure South	2017	0.142	0.222	0.321
Nigeria	Akure South	2000-2017	-0.081	-0.054	-0.022
Nigeria	Akwanga	2000	0.561	0.874	1.257
Nigeria	Akwanga	2017	0.251	0.381	0.545
Nigeria	Akwanga	2000-2017	-0.078	-0.047	-0.020
Nigeria	Albasu	2000	0.581	0.900	1.295
Nigeria	Albasu	2017	0.331	0.506	0.723
Nigeria	Albasu	2000-2017	-0.063	-0.033	-0.002
Nigeria	Aleiro	2000	0.486	0.814	1.225
Nigeria	Aleiro	2017	0.244	0.401	0.625
Nigeria	Aleiro	2000-2017	-0.066	-0.040	-0.009
Nigeria	Alimosho	2000	0.345	0.555	0.881
Nigeria	Alimosho	2017	0.148	0.237	0.346
Nigeria	Alimosho	2000-2017	-0.082	-0.047	-0.010
Nigeria	Alkaleri	2000	0.620	0.987	1.460
Nigeria	Alkaleri	2017	0.358	0.567	0.815
Nigeria	Alkaleri	2000-2017	-0.062	-0.032	-0.005
Nigeria	Amuwo Odofin	2000	0.331	0.545	0.815
Nigeria	Amuwo Odofin	2017	0.151	0.241	0.360
Nigeria	Amuwo Odofin	2000-2017	-0.075	-0.046	-0.012
Nigeria	Anambra East	2000	0.343	0.555	0.831
Nigeria	Anambra East	2017	0.159	0.252	0.387
Nigeria	Anambra East	2000-2017	-0.075	-0.044	-0.011
Nigeria	Anambra West	2000	0.384	0.615	0.917
Nigeria	Anambra West	2017	0.163	0.256	0.375
Nigeria	Anambra West	2000-2017	-0.074	-0.049	-0.019
Nigeria	Anaocha	2000	0.364	0.596	0.886
Nigeria	Anaocha	2017	0.164	0.279	0.436
Nigeria	Anaocha	2000-2017	-0.079	-0.043	-0.010
Nigeria	Andoni/O	2000	0.423	0.702	1.045
Nigeria	Andoni/O	2017	0.176	0.272	0.412
Nigeria	Andoni/O	2000-2017	-0.085	-0.055	-0.022
Nigeria	Aninri	2000	0.442	0.691	0.998
Nigeria	Aninri	2017	0.176	0.269	0.398
Nigeria	Aninri	2000-2017	-0.083	-0.055	-0.022
Nigeria	AniochaN	2000	0.374	0.602	0.874
Nigeria	AniochaN	2017	0.159	0.251	0.375
Nigeria	AniochaN	2000-2017	-0.075	-0.049	-0.017
Nigeria	AniochaS	2000	0.393	0.600	0.908

Table 1: LRI DALYs rate by unit *(continued)*

Country	Unit	year	mean rate	lower rate	upper rate
Nigeria	AniochaS	2017	0.161	0.251	0.377
Nigeria	AniochaS	2000-2017	-0.075	-0.049	-0.019
Nigeria	Anka	2000	0.508	0.840	1.277
Nigeria	Anka	2017	0.280	0.419	0.640
Nigeria	Anka	2000-2017	-0.066	-0.040	-0.013
Nigeria	Ankpa	2000	0.478	0.796	1.190
Nigeria	Ankpa	2017	0.203	0.310	0.443
Nigeria	Ankpa	2000-2017	-0.083	-0.057	-0.029
Nigeria	Apa	2000	0.515	0.817	1.201
Nigeria	Apa	2017	0.206	0.322	0.468
Nigeria	Apa	2000-2017	-0.081	-0.055	-0.028
Nigeria	Apapa	2000	0.335	0.561	0.872
Nigeria	Apapa	2017	0.148	0.248	0.368
Nigeria	Apapa	2000-2017	-0.083	-0.046	-0.012
Nigeria	Ardo-Kola	2000	0.617	1.002	1.449
Nigeria	Ardo-Kola	2017	0.339	0.516	0.778
Nigeria	Ardo-Kola	2000-2017	-0.067	-0.037	-0.004
Nigeria	Arewa	2000	0.603	0.934	1.335
Nigeria	Arewa	2017	0.264	0.423	0.650
Nigeria	Arewa	2000-2017	-0.071	-0.046	-0.018
Nigeria	Argungu	2000	0.502	0.828	1.253
Nigeria	Argungu	2017	0.251	0.416	0.660
Nigeria	Argungu	2000-2017	-0.065	-0.041	-0.012
Nigeria	Arochukw	2000	0.438	0.697	1.014
Nigeria	Arochukw	2017	0.172	0.272	0.397
Nigeria	Arochukw	2000-2017	-0.083	-0.054	-0.023
Nigeria	Asa	2000	0.448	0.752	1.120
Nigeria	Asa	2017	0.181	0.284	0.422
Nigeria	Asa	2000-2017	-0.084	-0.058	-0.032
Nigeria	Asari-To	2000	0.455	0.745	1.167
Nigeria	Asari-To	2017	0.183	0.297	0.440
Nigeria	Asari-To	2000-2017	-0.084	-0.052	-0.014
Nigeria	Askira/U	2000	0.700	1.081	1.577
Nigeria	Askira/U	2017	0.434	0.634	0.947
Nigeria	Askira/U	2000-2017	-0.053	-0.029	-0.003
Nigeria	Atakumosa East	2000	0.353	0.570	0.837
Nigeria	Atakumosa East	2017	0.147	0.220	0.318
Nigeria	Atakumosa East	2000-2017	-0.084	-0.055	-0.024
Nigeria	Atakumosa West	2000	0.366	0.612	0.900
Nigeria	Atakumosa West	2017	0.142	0.222	0.320
Nigeria	Atakumosa West	2000-2017	-0.087	-0.061	-0.034
Nigeria	Atiba	2000	0.367	0.615	0.918
Nigeria	Atiba	2017	0.146	0.226	0.328
Nigeria	Atiba	2000-2017	-0.084	-0.060	-0.035
Nigeria	Atisbo	2000	0.395	0.635	0.917
Nigeria	Atisbo	2017	0.143	0.230	0.346
Nigeria	Atisbo	2000-2017	-0.086	-0.060	-0.034
Nigeria	Augie	2000	0.494	0.836	1.234
Nigeria	Augie	2017	0.264	0.423	0.657
Nigeria	Augie	2000-2017	-0.063	-0.040	-0.012
Nigeria	Auyo	2000	0.579	0.945	1.476
Nigeria	Auyo	2017	0.364	0.566	0.841
Nigeria	Auyo	2000-2017	-0.058	-0.029	0.003
Nigeria	Awe	2000	0.585	0.903	1.326
Nigeria	Awe	2017	0.269	0.410	0.594
Nigeria	Awe	2000-2017	-0.072	-0.045	-0.018
Nigeria	Awgu	2000	0.394	0.664	1.005
Nigeria	Awgu	2017	0.167	0.280	0.417
Nigeria	Awgu	2000-2017	-0.078	-0.048	-0.018
Nigeria	AwkaNort	2000	0.354	0.574	0.874
Nigeria	AwkaNort	2017	0.163	0.263	0.388

Table 1: LRI DALYs rate by unit *(continued)*

Country	Unit	year	mean rate	lower rate	upper rate
Nigeria	AwkaNort	2000-2017	-0.072	-0.044	-0.012
Nigeria	AwkaSout	2000	0.354	0.581	0.885
Nigeria	AwkaSout	2017	0.167	0.274	0.431
Nigeria	AwkaSout	2000-2017	-0.076	-0.042	-0.008
Nigeria	Ayamelum	2000	0.363	0.590	0.866
Nigeria	Ayamelum	2017	0.160	0.262	0.401
Nigeria	Ayamelum	2000-2017	-0.075	-0.045	-0.016
Nigeria	Ayedaade	2000	0.401	0.602	0.871
Nigeria	Ayedaade	2017	0.142	0.222	0.320
Nigeria	Ayedaade	2000-2017	-0.087	-0.060	-0.033
Nigeria	Ayedire	2000	0.367	0.613	0.905
Nigeria	Ayedire	2017	0.138	0.223	0.331
Nigeria	Ayedire	2000-2017	-0.088	-0.061	-0.034
Nigeria	Babura	2000	0.551	0.899	1.380
Nigeria	Babura	2017	0.329	0.516	0.745
Nigeria	Babura	2000-2017	-0.061	-0.031	-0.001
Nigeria	Badagary	2000	0.328	0.538	0.798
Nigeria	Badagary	2017	0.142	0.233	0.349
Nigeria	Badagary	2000-2017	-0.079	-0.050	-0.023
Nigeria	Bade	2000	0.587	0.982	1.483
Nigeria	Bade	2017	0.399	0.613	0.879
Nigeria	Bade	2000-2017	-0.058	-0.026	0.003
Nigeria	Bagudo	2000	0.508	0.803	1.187
Nigeria	Bagudo	2017	0.236	0.376	0.569
Nigeria	Bagudo	2000-2017	-0.068	-0.044	-0.018
Nigeria	Bagwai	2000	0.533	0.851	1.276
Nigeria	Bagwai	2017	0.305	0.474	0.681
Nigeria	Bagwai	2000-2017	-0.062	-0.033	-0.001
Nigeria	Bakassi	2000	0.491	0.814	1.218
Nigeria	Bakassi	2017	0.186	0.309	0.469
Nigeria	Bakassi	2000-2017	-0.088	-0.055	-0.019
Nigeria	Bakori	2000	0.533	0.828	1.256
Nigeria	Bakori	2017	0.284	0.434	0.609
Nigeria	Bakori	2000-2017	-0.068	-0.037	-0.007
Nigeria	Bakura	2000	0.521	0.839	1.224
Nigeria	Bakura	2017	0.284	0.427	0.648
Nigeria	Bakura	2000-2017	-0.065	-0.040	-0.014
Nigeria	Balanga	2000	0.662	1.030	1.522
Nigeria	Balanga	2017	0.409	0.602	0.895
Nigeria	Balanga	2000-2017	-0.058	-0.030	-0.003
Nigeria	Bali	2000	0.652	0.992	1.428
Nigeria	Bali	2017	0.314	0.501	0.728
Nigeria	Bali	2000-2017	-0.068	-0.038	-0.012
Nigeria	Bama	2000	0.691	1.097	1.611
Nigeria	Bama	2017	0.428	0.665	0.989
Nigeria	Bama	2000-2017	-0.052	-0.027	0.001
Nigeria	Barkin Ladi	2000	0.552	0.866	1.305
Nigeria	Barkin Ladi	2017	0.248	0.390	0.588
Nigeria	Barkin Ladi	2000-2017	-0.072	-0.045	-0.016
Nigeria	Baruten	2000	0.495	0.789	1.149
Nigeria	Baruten	2017	0.184	0.290	0.428
Nigeria	Baruten	2000-2017	-0.079	-0.057	-0.032
Nigeria	Bassa	2000	0.490	0.770	1.136
Nigeria	Bassa	2000	0.527	0.853	1.233
Nigeria	Bassa	2017	0.192	0.294	0.432
Nigeria	Bassa	2017	0.283	0.434	0.617
Nigeria	Bassa	2000-2017	-0.067	-0.039	-0.007
Nigeria	Bassa	2000-2017	-0.082	-0.056	-0.027
Nigeria	Batagarawa	2000	0.523	0.854	1.273
Nigeria	Batagarawa	2017	0.296	0.475	0.701
Nigeria	Batagarawa	2000-2017	-0.068	-0.034	0.001

Table 1: LRI DALYs rate by unit *(continued)*

Country	Unit	year	mean rate	lower rate	upper rate
Nigeria	Batsari	2000	0.541	0.844	1.271
Nigeria	Batsari	2017	0.293	0.459	0.677
Nigeria	Batsari	2000-2017	-0.064	-0.035	-0.006
Nigeria	Bauchi	2000	0.610	0.951	1.439
Nigeria	Bauchi	2017	0.335	0.527	0.748
Nigeria	Bauchi	2000-2017	-0.064	-0.033	-0.003
Nigeria	Baure	2000	0.581	0.911	1.326
Nigeria	Baure	2017	0.338	0.513	0.729
Nigeria	Baure	2000-2017	-0.059	-0.032	-0.001
Nigeria	Bayo	2000	0.661	1.049	1.554
Nigeria	Bayo	2017	0.419	0.643	0.958
Nigeria	Bayo	2000-2017	-0.057	-0.027	0.001
Nigeria	Bebeji	2000	0.553	0.868	1.257
Nigeria	Bebeji	2017	0.293	0.472	0.699
Nigeria	Bebeji	2000-2017	-0.065	-0.036	-0.006
Nigeria	Bekwarra	2000	0.470	0.753	1.105
Nigeria	Bekwarra	2017	0.190	0.308	0.455
Nigeria	Bekwarra	2000-2017	-0.084	-0.052	-0.025
Nigeria	Bende	2000	0.436	0.706	1.031
Nigeria	Bende	2017	0.174	0.274	0.404
Nigeria	Bende	2000-2017	-0.083	-0.055	-0.023
Nigeria	Biase	2000	0.491	0.783	1.123
Nigeria	Biase	2017	0.195	0.304	0.468
Nigeria	Biase	2000-2017	-0.082	-0.055	-0.026
Nigeria	Bichi	2000	0.551	0.866	1.308
Nigeria	Bichi	2017	0.319	0.484	0.695
Nigeria	Bichi	2000-2017	-0.064	-0.033	-0.003
Nigeria	Bida	2000	0.429	0.765	1.160
Nigeria	Bida	2017	0.196	0.305	0.464
Nigeria	Bida	2000-2017	-0.089	-0.053	-0.012
Nigeria	Billiri	2000	0.656	1.032	1.522
Nigeria	Billiri	2017	0.379	0.585	0.827
Nigeria	Billiri	2000-2017	-0.059	-0.032	-0.003
Nigeria	Bindawa	2000	0.536	0.865	1.288
Nigeria	Bindawa	2017	0.303	0.475	0.690
Nigeria	Bindawa	2000-2017	-0.064	-0.034	-0.003
Nigeria	Binji	2000	0.501	0.845	1.264
Nigeria	Binji	2017	0.261	0.435	0.670
Nigeria	Binji	2000-2017	-0.069	-0.039	-0.007
Nigeria	Biriniwa	2000	0.578	0.958	1.472
Nigeria	Biriniwa	2017	0.377	0.580	0.872
Nigeria	Biriniwa	2000-2017	-0.059	-0.028	0.004
Nigeria	Birnin-G	2000	0.503	0.805	1.183
Nigeria	Birnin-G	2017	0.271	0.406	0.576
Nigeria	Birnin-G	2000-2017	-0.065	-0.039	-0.012
Nigeria	Birnin-Magaji/Kiyaw	2000	0.525	0.843	1.265
Nigeria	Birnin-Magaji/Kiyaw	2017	0.293	0.445	0.637
Nigeria	Birnin-Magaji/Kiyaw	2000-2017	-0.067	-0.037	-0.011
Nigeria	BirninKe	2000	0.512	0.829	1.231
Nigeria	BirninKe	2017	0.242	0.407	0.643
Nigeria	BirninKe	2000-2017	-0.068	-0.042	-0.014
Nigeria	BirninKu	2000	0.616	0.934	1.343
Nigeria	BirninKu	2017	0.332	0.524	0.757
Nigeria	BirninKu	2000-2017	-0.063	-0.033	-0.003
Nigeria	Biu	2000	0.681	1.097	1.638
Nigeria	Biu	2017	0.438	0.658	0.982
Nigeria	Biu	2000-2017	-0.055	-0.027	-0.002
Nigeria	Bodinga	2000	0.512	0.836	1.272
Nigeria	Bodinga	2017	0.283	0.430	0.666
Nigeria	Bodinga	2000-2017	-0.068	-0.039	-0.009
Nigeria	Bogoro	2000	0.591	0.917	1.351

Table 1: LRI DALYs rate by unit *(continued)*

Country	Unit	year	mean rate	lower rate	upper rate
Nigeria	Bogoro	2017	0.314	0.497	0.702
Nigeria	Bogoro	2000-2017	-0.066	-0.036	-0.006
Nigeria	Boki	2000	0.524	0.811	1.147
Nigeria	Boki	2017	0.202	0.324	0.494
Nigeria	Boki	2000-2017	-0.082	-0.055	-0.027
Nigeria	Bokkos	2000	0.594	0.883	1.271
Nigeria	Bokkos	2017	0.238	0.381	0.562
Nigeria	Bokkos	2000-2017	-0.077	-0.047	-0.019
Nigeria	Boluwaduro	2000	0.340	0.589	0.898
Nigeria	Boluwaduro	2017	0.140	0.224	0.348
Nigeria	Boluwaduro	2000-2017	-0.095	-0.058	-0.026
Nigeria	Bomadi	2000	0.452	0.718	1.092
Nigeria	Bomadi	2017	0.172	0.278	0.444
Nigeria	Bomadi	2000-2017	-0.087	-0.056	-0.020
Nigeria	Bonny	2000	0.437	0.714	1.049
Nigeria	Bonny	2017	0.177	0.276	0.413
Nigeria	Bonny	2000-2017	-0.086	-0.057	-0.022
Nigeria	Borgu	2000	0.483	0.794	1.174
Nigeria	Borgu	2017	0.234	0.346	0.503
Nigeria	Borgu	2000-2017	-0.069	-0.048	-0.022
Nigeria	Boripe	2000	0.364	0.579	0.864
Nigeria	Boripe	2017	0.137	0.223	0.337
Nigeria	Boripe	2000-2017	-0.086	-0.056	-0.028
Nigeria	Borsari	2000	0.610	1.009	1.537
Nigeria	Borsari	2017	0.416	0.636	0.908
Nigeria	Borsari	2000-2017	-0.055	-0.025	0.004
Nigeria	Bosso	2000	0.487	0.806	1.172
Nigeria	Bosso	2017	0.228	0.344	0.495
Nigeria	Bosso	2000-2017	-0.076	-0.050	-0.018
Nigeria	Brass	2000	0.452	0.707	1.065
Nigeria	Brass	2017	0.166	0.277	0.422
Nigeria	Brass	2000-2017	-0.086	-0.057	-0.027
Nigeria	Buji	2000	0.596	0.946	1.367
Nigeria	Buji	2017	0.328	0.537	0.781
Nigeria	Buji	2000-2017	-0.064	-0.032	0.001
Nigeria	Bukkuyum	2000	0.497	0.827	1.261
Nigeria	Bukkuyum	2017	0.270	0.411	0.636
Nigeria	Bukkuyum	2000-2017	-0.067	-0.041	-0.013
Nigeria	Bungudu	2000	0.484	0.821	1.240
Nigeria	Bungudu	2017	0.287	0.427	0.634
Nigeria	Bungudu	2000-2017	-0.068	-0.039	-0.009
Nigeria	Bunkure	2000	0.563	0.873	1.321
Nigeria	Bunkure	2017	0.317	0.484	0.703
Nigeria	Bunkure	2000-2017	-0.067	-0.034	-0.004
Nigeria	Bunza	2000	0.488	0.815	1.230
Nigeria	Bunza	2017	0.242	0.396	0.620
Nigeria	Bunza	2000-2017	-0.069	-0.042	-0.015
Nigeria	Buruku	2000	0.579	0.871	1.258
Nigeria	Buruku	2017	0.230	0.356	0.509
Nigeria	Buruku	2000-2017	-0.083	-0.053	-0.026
Nigeria	Burutu	2000	0.444	0.682	0.996
Nigeria	Burutu	2017	0.162	0.263	0.400
Nigeria	Burutu	2000-2017	-0.083	-0.057	-0.027
Nigeria	Bwari	2000	0.496	0.776	1.108
Nigeria	Bwari	2017	0.209	0.319	0.464
Nigeria	Bwari	2000-2017	-0.081	-0.051	-0.024
Nigeria	Calabar	2000	0.452	0.750	1.079
Nigeria	Calabar	2017	0.195	0.309	0.486
Nigeria	Calabar	2000-2017	-0.086	-0.053	-0.020
Nigeria	Calabar South	2000	0.461	0.766	1.160
Nigeria	Calabar South	2017	0.177	0.292	0.441

Table 1: LRI DALYs rate by unit *(continued)*

Country	Unit	year	mean rate	lower rate	upper rate
Nigeria	Calabar South	2000-2017	-0.094	-0.058	-0.025
Nigeria	Chanchaga	2000	0.460	0.829	1.311
Nigeria	Chanchaga	2017	0.213	0.346	0.515
Nigeria	Chanchaga	2000-2017	-0.091	-0.051	-0.011
Nigeria	Charanchi	2000	0.511	0.846	1.241
Nigeria	Charanchi	2017	0.302	0.465	0.659
Nigeria	Charanchi	2000-2017	-0.064	-0.035	-0.004
Nigeria	Chibok	2000	0.680	1.103	1.652
Nigeria	Chibok	2017	0.434	0.667	0.998
Nigeria	Chibok	2000-2017	-0.053	-0.027	-0.001
Nigeria	Chikun	2000	0.466	0.750	1.030
Nigeria	Chikun	2017	0.266	0.399	0.587
Nigeria	Chikun	2000-2017	-0.064	-0.036	-0.007
Nigeria	Dala	2000	0.539	0.891	1.376
Nigeria	Dala	2017	0.291	0.469	0.729
Nigeria	Dala	2000-2017	-0.077	-0.037	-0.001
Nigeria	Damaturu	2000	0.693	1.086	1.669
Nigeria	Damaturu	2017	0.433	0.682	0.981
Nigeria	Damaturu	2000-2017	-0.055	-0.025	0.007
Nigeria	Damban	2000	0.628	0.992	1.452
Nigeria	Damban	2017	0.387	0.601	0.835
Nigeria	Damban	2000-2017	-0.055	-0.028	0.001
Nigeria	Dambatta	2000	0.570	0.890	1.381
Nigeria	Dambatta	2017	0.309	0.506	0.733
Nigeria	Dambatta	2000-2017	-0.064	-0.032	0.000
Nigeria	Damboa	2000	0.704	1.100	1.615
Nigeria	Damboa	2017	0.456	0.679	1.020
Nigeria	Damboa	2000-2017	-0.048	-0.026	0.001
Nigeria	Dandi	2000	0.544	0.844	1.210
Nigeria	Dandi	2017	0.241	0.388	0.589
Nigeria	Dandi	2000-2017	-0.068	-0.045	-0.017
Nigeria	Dandume	2000	0.495	0.810	1.183
Nigeria	Dandume	2017	0.273	0.418	0.608
Nigeria	Dandume	2000-2017	-0.070	-0.038	-0.009
Nigeria	Dange-Shuni	2000	0.518	0.831	1.250
Nigeria	Dange-Shuni	2017	0.279	0.428	0.668
Nigeria	Dange-Shuni	2000-2017	-0.066	-0.039	-0.010
Nigeria	Danja	2000	0.529	0.818	1.188
Nigeria	Danja	2017	0.273	0.433	0.643
Nigeria	Danja	2000-2017	-0.068	-0.037	-0.007
Nigeria	Danko Wasagu	2000	0.484	0.791	1.202
Nigeria	Danko Wasagu	2017	0.257	0.390	0.589
Nigeria	Danko Wasagu	2000-2017	-0.066	-0.041	-0.013
Nigeria	Danmusa	2000	0.503	0.823	1.213
Nigeria	Danmusa	2017	0.290	0.444	0.645
Nigeria	Danmusa	2000-2017	-0.065	-0.036	-0.005
Nigeria	Darazo	2000	0.626	1.000	1.472
Nigeria	Darazo	2017	0.378	0.595	0.850
Nigeria	Darazo	2000-2017	-0.059	-0.029	0.001
Nigeria	Dass	2000	0.579	0.887	1.327
Nigeria	Dass	2017	0.316	0.496	0.725
Nigeria	Dass	2000-2017	-0.067	-0.033	-0.006
Nigeria	Daura	2000	0.534	0.886	1.360
Nigeria	Daura	2017	0.315	0.505	0.741
Nigeria	Daura	2000-2017	-0.065	-0.032	0.003
Nigeria	DawakinK	2000	0.557	0.878	1.263
Nigeria	DawakinK	2017	0.322	0.490	0.701
Nigeria	DawakinK	2000-2017	-0.064	-0.033	-0.001
Nigeria	DawakinT	2000	0.538	0.869	1.276
Nigeria	DawakinT	2017	0.304	0.488	0.704
Nigeria	DawakinT	2000-2017	-0.062	-0.033	-0.003

Table 1: LRI DALYs rate by unit *(continued)*

Country	Unit	year	mean rate	lower rate	upper rate
Nigeria	Degema	2000	0.461	0.724	1.058
Nigeria	Degema	2017	0.180	0.292	0.440
Nigeria	Degema	2000-2017	-0.082	-0.054	-0.023
Nigeria	Dekina	2000	0.486	0.784	1.192
Nigeria	Dekina	2017	0.203	0.299	0.430
Nigeria	Dekina	2000-2017	-0.080	-0.057	-0.030
Nigeria	Demsa	2000	0.669	1.066	1.527
Nigeria	Demsa	2017	0.367	0.550	0.800
Nigeria	Demsa	2000-2017	-0.065	-0.037	-0.007
Nigeria	Dikwa	2000	0.679	1.078	1.572
Nigeria	Dikwa	2017	0.424	0.650	0.950
Nigeria	Dikwa	2000-2017	-0.051	-0.027	0.000
Nigeria	Doguwa	2000	0.552	0.861	1.227
Nigeria	Doguwa	2017	0.302	0.471	0.683
Nigeria	Doguwa	2000-2017	-0.061	-0.034	-0.003
Nigeria	Doma	2000	0.557	0.835	1.185
Nigeria	Doma	2017	0.228	0.347	0.507
Nigeria	Doma	2000-2017	-0.078	-0.050	-0.024
Nigeria	Donga	2000	0.623	0.933	1.349
Nigeria	Donga	2017	0.277	0.430	0.634
Nigeria	Donga	2000-2017	-0.073	-0.043	-0.016
Nigeria	Dukku	2000	0.654	1.021	1.530
Nigeria	Dukku	2017	0.391	0.612	0.875
Nigeria	Dukku	2000-2017	-0.054	-0.029	-0.001
Nigeria	Dunukofia	2000	0.337	0.547	0.826
Nigeria	Dunukofia	2017	0.160	0.257	0.376
Nigeria	Dunukofia	2000-2017	-0.078	-0.043	-0.007
Nigeria	Dutse	2000	0.580	0.923	1.387
Nigeria	Dutse	2017	0.342	0.521	0.752
Nigeria	Dutse	2000-2017	-0.063	-0.032	0.002
Nigeria	Dutsi	2000	0.556	0.876	1.364
Nigeria	Dutsi	2017	0.311	0.492	0.715
Nigeria	Dutsi	2000-2017	-0.060	-0.032	0.000
Nigeria	Dutsin-M	2000	0.519	0.831	1.220
Nigeria	Dutsin-M	2017	0.296	0.456	0.672
Nigeria	Dutsin-M	2000-2017	-0.065	-0.035	-0.006
Nigeria	Eastern Obolo	2000	0.431	0.712	1.120
Nigeria	Eastern Obolo	2017	0.173	0.275	0.412
Nigeria	Eastern Obolo	2000-2017	-0.090	-0.054	-0.022
Nigeria	Ebonyi	2000	0.472	0.831	1.318
Nigeria	Ebonyi	2017	0.198	0.348	0.545
Nigeria	Ebonyi	2000-2017	-0.083	-0.051	-0.020
Nigeria	Edati	2000	0.466	0.762	1.095
Nigeria	Edati	2017	0.220	0.329	0.480
Nigeria	Edati	2000-2017	-0.075	-0.049	-0.021
Nigeria	Ede North	2000	0.347	0.568	0.861
Nigeria	Ede North	2017	0.140	0.220	0.320
Nigeria	Ede North	2000-2017	-0.087	-0.054	-0.020
Nigeria	Ede South	2000	0.356	0.594	0.886
Nigeria	Ede South	2017	0.138	0.223	0.327
Nigeria	Ede South	2000-2017	-0.088	-0.058	-0.031
Nigeria	Edu	2000	0.478	0.759	1.117
Nigeria	Edu	2017	0.197	0.286	0.415
Nigeria	Edu	2000-2017	-0.078	-0.056	-0.031
Nigeria	Efon	2000	0.371	0.605	0.909
Nigeria	Efon	2017	0.144	0.226	0.331
Nigeria	Efon	2000-2017	-0.088	-0.058	-0.029
Nigeria	EgbadoNorth	2000	0.460	0.740	1.050
Nigeria	EgbadoNorth	2017	0.156	0.253	0.381
Nigeria	EgbadoNorth	2000-2017	-0.088	-0.062	-0.035
Nigeria	EgbadoSouth	2000	0.404	0.669	1.019

Table 1: LRI DALYs rate by unit *(continued)*

Country	Unit	year	mean rate	lower rate	upper rate
Nigeria	EgbadoSouth	2017	0.160	0.250	0.371
Nigeria	EgbadoSouth	2000-2017	-0.084	-0.056	-0.027
Nigeria	Egbeda	2000	0.336	0.542	0.837
Nigeria	Egbeda	2017	0.139	0.221	0.316
Nigeria	Egbeda	2000-2017	-0.084	-0.053	-0.019
Nigeria	Egbedore	2000	0.342	0.554	0.818
Nigeria	Egbedore	2017	0.142	0.226	0.333
Nigeria	Egbedore	2000-2017	-0.078	-0.053	-0.022
Nigeria	Egor	2000	0.307	0.509	0.796
Nigeria	Egor	2017	0.142	0.232	0.352
Nigeria	Egor	2000-2017	-0.079	-0.043	-0.009
Nigeria	Ehime-Mb	2000	0.386	0.639	0.955
Nigeria	Ehime-Mb	2017	0.164	0.261	0.383
Nigeria	Ehime-Mb	2000-2017	-0.084	-0.050	-0.017
Nigeria	Ejigbo	2000	0.353	0.602	0.925
Nigeria	Ejigbo	2017	0.138	0.225	0.329
Nigeria	Ejigbo	2000-2017	-0.086	-0.059	-0.030
Nigeria	Ekeremor	2000	0.434	0.699	1.000
Nigeria	Ekeremor	2017	0.171	0.273	0.407
Nigeria	Ekeremor	2000-2017	-0.081	-0.056	-0.025
Nigeria	Eket	2000	0.426	0.691	1.023
Nigeria	Eket	2017	0.163	0.274	0.426
Nigeria	Eket	2000-2017	-0.086	-0.055	-0.021
Nigeria	Ekiti	2000	0.448	0.751	1.137
Nigeria	Ekiti	2017	0.168	0.273	0.396
Nigeria	Ekiti	2000-2017	-0.087	-0.061	-0.031
Nigeria	EkitiEas	2000	0.366	0.616	0.952
Nigeria	EkitiEas	2017	0.151	0.233	0.341
Nigeria	EkitiEas	2000-2017	-0.086	-0.057	-0.026
Nigeria	EkitiSouth-West	2000	0.376	0.613	0.952
Nigeria	EkitiSouth-West	2017	0.149	0.223	0.312
Nigeria	EkitiSouth-West	2000-2017	-0.086	-0.059	-0.028
Nigeria	EkitiWest	2000	0.379	0.619	0.941
Nigeria	EkitiWest	2017	0.151	0.232	0.335
Nigeria	EkitiWest	2000-2017	-0.087	-0.058	-0.030
Nigeria	Ekwusigo	2000	0.358	0.575	0.872
Nigeria	Ekwusigo	2017	0.170	0.271	0.426
Nigeria	Ekwusigo	2000-2017	-0.076	-0.043	-0.011
Nigeria	Eleme	2000	0.441	0.711	1.064
Nigeria	Eleme	2017	0.172	0.268	0.404
Nigeria	Eleme	2000-2017	-0.093	-0.059	-0.024
Nigeria	Emuoha	2000	0.437	0.695	1.021
Nigeria	Emuoha	2017	0.171	0.268	0.401
Nigeria	Emuoha	2000-2017	-0.083	-0.056	-0.026
Nigeria	Emure/Ise/Orun	2000	0.382	0.606	0.923
Nigeria	Emure/Ise/Orun	2017	0.147	0.225	0.325
Nigeria	Emure/Ise/Orun	2000-2017	-0.085	-0.059	-0.029
Nigeria	Enugu East	2000	0.370	0.597	0.913
Nigeria	Enugu East	2017	0.168	0.272	0.405
Nigeria	Enugu East	2000-2017	-0.072	-0.044	-0.014
Nigeria	Enugu North	2000	0.352	0.574	0.873
Nigeria	Enugu North	2017	0.169	0.273	0.416
Nigeria	Enugu North	2000-2017	-0.076	-0.042	-0.009
Nigeria	EnuguSou	2000	0.335	0.588	0.935
Nigeria	EnuguSou	2017	0.164	0.280	0.455
Nigeria	EnuguSou	2000-2017	-0.083	-0.042	-0.004
Nigeria	Epe	2000	0.339	0.531	0.772
Nigeria	Epe	2017	0.143	0.224	0.329
Nigeria	Epe	2000-2017	-0.077	-0.051	-0.023
Nigeria	EsanCent	2000	0.398	0.609	0.931
Nigeria	EsanCent	2017	0.156	0.241	0.355

Table 1: LRI DALYs rate by unit *(continued)*

Country	Unit	year	mean rate	lower rate	upper rate
Nigeria	EsanCent	2000-2017	-0.080	-0.052	-0.025
Nigeria	EsanNort	2000	0.394	0.605	0.931
Nigeria	EsanNort	2017	0.153	0.244	0.374
Nigeria	EsanNort	2000-2017	-0.079	-0.051	-0.019
Nigeria	EsanSout	2000	0.391	0.624	0.955
Nigeria	EsanSout	2017	0.158	0.245	0.359
Nigeria	EsanSout	2000-2017	-0.077	-0.052	-0.024
Nigeria	EsanWest	2000	0.377	0.609	0.913
Nigeria	EsanWest	2017	0.154	0.237	0.348
Nigeria	EsanWest	2000-2017	-0.079	-0.054	-0.026
Nigeria	Ese-Odo	2000	0.405	0.633	0.951
Nigeria	Ese-Odo	2017	0.151	0.227	0.335
Nigeria	Ese-Odo	2000-2017	-0.085	-0.061	-0.032
Nigeria	Esit Eket	2000	0.435	0.722	1.082
Nigeria	Esit Eket	2017	0.174	0.278	0.426
Nigeria	Esit Eket	2000-2017	-0.086	-0.057	-0.022
Nigeria	Essien-U	2000	0.448	0.740	1.120
Nigeria	Essien-U	2017	0.173	0.288	0.442
Nigeria	Essien-U	2000-2017	-0.085	-0.055	-0.025
Nigeria	Etche	2000	0.434	0.687	0.984
Nigeria	Etche	2017	0.165	0.264	0.407
Nigeria	Etche	2000-2017	-0.087	-0.058	-0.027
Nigeria	Ethiope West	2000	0.370	0.588	0.899
Nigeria	Ethiope West	2017	0.158	0.242	0.368
Nigeria	Ethiope West	2000-2017	-0.078	-0.051	-0.022
Nigeria	EthiopeE	2000	0.390	0.604	0.891
Nigeria	EthiopeE	2017	0.162	0.247	0.376
Nigeria	EthiopeE	2000-2017	-0.081	-0.051	-0.022
Nigeria	Eti-Osa	2000	0.312	0.517	0.763
Nigeria	Eti-Osa	2017	0.141	0.226	0.335
Nigeria	Eti-Osa	2000-2017	-0.079	-0.047	-0.017
Nigeria	EtimEkpo	2000	0.431	0.741	1.128
Nigeria	EtimEkpo	2017	0.177	0.285	0.440
Nigeria	EtimEkpo	2000-2017	-0.087	-0.055	-0.024
Nigeria	Etinan	2000	0.441	0.748	1.106
Nigeria	Etinan	2017	0.172	0.288	0.440
Nigeria	Etinan	2000-2017	-0.083	-0.055	-0.019
Nigeria	Etsako Central	2000	0.404	0.621	0.900
Nigeria	Etsako Central	2017	0.157	0.243	0.352
Nigeria	Etsako Central	2000-2017	-0.077	-0.053	-0.024
Nigeria	EtsakoEa	2000	0.396	0.629	0.969
Nigeria	EtsakoEa	2017	0.168	0.251	0.367
Nigeria	EtsakoEa	2000-2017	-0.079	-0.053	-0.024
Nigeria	EtsakoWe	2000	0.368	0.588	0.915
Nigeria	EtsakoWe	2017	0.159	0.239	0.344
Nigeria	EtsakoWe	2000-2017	-0.078	-0.052	-0.025
Nigeria	Etung	2000	0.534	0.830	1.192
Nigeria	Etung	2017	0.208	0.329	0.501
Nigeria	Etung	2000-2017	-0.084	-0.055	-0.027
Nigeria	Ewekoro	2000	0.405	0.685	0.989
Nigeria	Ewekoro	2017	0.157	0.251	0.383
Nigeria	Ewekoro	2000-2017	-0.087	-0.059	-0.031
Nigeria	Ezeagu	2000	0.366	0.612	0.908
Nigeria	Ezeagu	2017	0.164	0.274	0.413
Nigeria	Ezeagu	2000-2017	-0.071	-0.044	-0.014
Nigeria	Ezinihit	2000	0.401	0.664	0.982
Nigeria	Ezinihit	2017	0.167	0.269	0.402
Nigeria	Ezinihit	2000-2017	-0.082	-0.052	-0.020
Nigeria	Ezza North	2000	0.518	0.905	1.420
Nigeria	Ezza North	2017	0.202	0.347	0.544
Nigeria	Ezza North	2000-2017	-0.084	-0.055	-0.021

Table 1: LRI DALYs rate by unit *(continued)*

Country	Unit	year	mean rate	lower rate	upper rate
Nigeria	Ezza South	2000	0.507	0.883	1.355
Nigeria	Ezza South	2017	0.197	0.338	0.505
Nigeria	Ezza South	2000-2017	-0.085	-0.055	-0.027
Nigeria	Fagge	2000	0.509	0.908	1.403
Nigeria	Fagge	2017	0.244	0.422	0.631
Nigeria	Fagge	2000-2017	-0.080	-0.042	0.008
Nigeria	Fakai	2000	0.479	0.803	1.194
Nigeria	Fakai	2017	0.250	0.390	0.584
Nigeria	Fakai	2000-2017	-0.068	-0.041	-0.014
Nigeria	Faskari	2000	0.517	0.819	1.214
Nigeria	Faskari	2017	0.271	0.421	0.606
Nigeria	Faskari	2000-2017	-0.070	-0.039	-0.010
Nigeria	Fika	2000	0.647	1.048	1.533
Nigeria	Fika	2017	0.422	0.648	0.910
Nigeria	Fika	2000-2017	-0.057	-0.027	0.000
Nigeria	Fufore	2000	0.685	1.078	1.600
Nigeria	Fufore	2017	0.368	0.551	0.818
Nigeria	Fufore	2000-2017	-0.064	-0.036	-0.010
Nigeria	Funakaye	2000	0.652	1.038	1.585
Nigeria	Funakaye	2017	0.417	0.642	0.948
Nigeria	Funakaye	2000-2017	-0.055	-0.027	0.002
Nigeria	Fune	2000	0.634	1.046	1.572
Nigeria	Fune	2017	0.431	0.657	0.920
Nigeria	Fune	2000-2017	-0.051	-0.026	0.001
Nigeria	Funtua	2000	0.496	0.814	1.191
Nigeria	Funtua	2017	0.272	0.422	0.628
Nigeria	Funtua	2000-2017	-0.069	-0.037	-0.006
Nigeria	Gabasawa	2000	0.577	0.895	1.366
Nigeria	Gabasawa	2017	0.321	0.512	0.735
Nigeria	Gabasawa	2000-2017	-0.060	-0.031	-0.002
Nigeria	Gada	2000	0.580	0.941	1.415
Nigeria	Gada	2017	0.299	0.462	0.691
Nigeria	Gada	2000-2017	-0.068	-0.043	-0.013
Nigeria	Gagarawa	2000	0.584	0.938	1.413
Nigeria	Gagarawa	2017	0.354	0.546	0.823
Nigeria	Gagarawa	2000-2017	-0.061	-0.030	0.003
Nigeria	Gamawa	2000	0.609	0.975	1.501
Nigeria	Gamawa	2017	0.397	0.592	0.827
Nigeria	Gamawa	2000-2017	-0.057	-0.028	0.002
Nigeria	Gamjuwa	2000	0.613	0.960	1.419
Nigeria	Gamjuwa	2017	0.350	0.545	0.779
Nigeria	Gamjuwa	2000-2017	-0.060	-0.032	-0.002
Nigeria	Ganye	2000	0.658	1.040	1.554
Nigeria	Ganye	2017	0.339	0.513	0.751
Nigeria	Ganye	2000-2017	-0.067	-0.038	-0.011
Nigeria	Garki	2000	0.571	0.912	1.357
Nigeria	Garki	2017	0.335	0.520	0.766
Nigeria	Garki	2000-2017	-0.061	-0.032	-0.002
Nigeria	Garko	2000	0.565	0.884	1.325
Nigeria	Garko	2017	0.319	0.494	0.707
Nigeria	Garko	2000-2017	-0.065	-0.033	0.003
Nigeria	Garum Mallam	2000	0.539	0.868	1.288
Nigeria	Garum Mallam	2017	0.292	0.469	0.684
Nigeria	Garum Mallam	2000-2017	-0.063	-0.035	0.000
Nigeria	Gashaka	2000	0.629	0.985	1.397
Nigeria	Gashaka	2017	0.321	0.483	0.711
Nigeria	Gashaka	2000-2017	-0.068	-0.039	-0.014
Nigeria	Gassol	2000	0.622	0.948	1.386
Nigeria	Gassol	2017	0.312	0.495	0.722
Nigeria	Gassol	2000-2017	-0.067	-0.037	-0.008
Nigeria	Gaya	2000	0.572	0.894	1.327

Table 1: LRI DALYs rate by unit *(continued)*

Country	Unit	year	mean rate	lower rate	upper rate
Nigeria	Gaya	2017	0.335	0.506	0.726
Nigeria	Gaya	2000-2017	-0.063	-0.033	-0.004
Nigeria	Gbako	2000	0.462	0.760	1.112
Nigeria	Gbako	2017	0.224	0.333	0.462
Nigeria	Gbako	2000-2017	-0.072	-0.048	-0.018
Nigeria	Gboko	2000	0.571	0.858	1.240
Nigeria	Gboko	2017	0.223	0.354	0.531
Nigeria	Gboko	2000-2017	-0.080	-0.052	-0.022
Nigeria	Gboyin	2000	0.373	0.622	0.921
Nigeria	Gboyin	2017	0.146	0.231	0.329
Nigeria	Gboyin	2000-2017	-0.085	-0.058	-0.027
Nigeria	Geidam	2000	0.612	1.016	1.560
Nigeria	Geidam	2017	0.416	0.659	0.939
Nigeria	Geidam	2000-2017	-0.052	-0.024	0.004
Nigeria	Gezawa	2000	0.561	0.887	1.305
Nigeria	Gezawa	2017	0.319	0.487	0.717
Nigeria	Gezawa	2000-2017	-0.067	-0.034	0.001
Nigeria	Giade	2000	0.633	0.984	1.414
Nigeria	Giade	2017	0.359	0.569	0.807
Nigeria	Giade	2000-2017	-0.060	-0.031	-0.002
Nigeria	Girie	2000	0.691	1.079	1.625
Nigeria	Girie	2017	0.392	0.590	0.889
Nigeria	Girie	2000-2017	-0.058	-0.032	-0.004
Nigeria	Giwa	2000	0.524	0.818	1.173
Nigeria	Giwa	2017	0.279	0.427	0.623
Nigeria	Giwa	2000-2017	-0.065	-0.037	-0.010
Nigeria	Gokana	2000	0.441	0.722	1.100
Nigeria	Gokana	2017	0.176	0.280	0.452
Nigeria	Gokana	2000-2017	-0.094	-0.058	-0.015
Nigeria	Gombe	2000	0.569	1.007	1.580
Nigeria	Gombe	2017	0.353	0.623	1.098
Nigeria	Gombe	2000-2017	-0.077	-0.026	0.022
Nigeria	Gombi	2000	0.677	1.077	1.570
Nigeria	Gombi	2017	0.378	0.563	0.847
Nigeria	Gombi	2000-2017	-0.061	-0.034	-0.007
Nigeria	Goronyo	2000	0.505	0.846	1.291
Nigeria	Goronyo	2017	0.286	0.449	0.671
Nigeria	Goronyo	2000-2017	-0.064	-0.037	-0.006
Nigeria	Gubio	2000	0.628	1.014	1.523
Nigeria	Gubio	2017	0.421	0.668	0.974
Nigeria	Gubio	2000-2017	-0.049	-0.022	0.003
Nigeria	Gudu	2000	0.614	0.994	1.403
Nigeria	Gudu	2017	0.277	0.450	0.691
Nigeria	Gudu	2000-2017	-0.070	-0.046	-0.018
Nigeria	Gujba	2000	0.685	1.091	1.659
Nigeria	Gujba	2017	0.446	0.693	0.992
Nigeria	Gujba	2000-2017	-0.053	-0.025	0.003
Nigeria	Gulani	2000	0.650	1.058	1.598
Nigeria	Gulani	2017	0.420	0.661	0.965
Nigeria	Gulani	2000-2017	-0.052	-0.026	0.001
Nigeria	Guma	2000	0.601	0.877	1.287
Nigeria	Guma	2017	0.240	0.367	0.536
Nigeria	Guma	2000-2017	-0.080	-0.051	-0.026
Nigeria	Gumel	2000	0.548	0.940	1.439
Nigeria	Gumel	2017	0.337	0.544	0.788
Nigeria	Gumel	2000-2017	-0.063	-0.031	0.002
Nigeria	Gummi	2000	0.493	0.829	1.242
Nigeria	Gummi	2017	0.263	0.410	0.635
Nigeria	Gummi	2000-2017	-0.065	-0.041	-0.013
Nigeria	Gurara	2000	0.511	0.793	1.115
Nigeria	Gurara	2017	0.220	0.334	0.496

Table 1: LRI DALYs rate by unit *(continued)*

Country	Unit	year	mean rate	lower rate	upper rate
Nigeria	Gurara	2000-2017	-0.076	-0.049	-0.020
Nigeria	Guri	2000	0.595	0.967	1.464
Nigeria	Guri	2017	0.386	0.590	0.860
Nigeria	Guri	2000-2017	-0.056	-0.027	0.001
Nigeria	Gusau	2000	0.493	0.827	1.283
Nigeria	Gusau	2017	0.289	0.427	0.624
Nigeria	Gusau	2000-2017	-0.066	-0.039	-0.011
Nigeria	Guyuk	2000	0.657	1.051	1.547
Nigeria	Guyuk	2017	0.363	0.560	0.811
Nigeria	Guyuk	2000-2017	-0.062	-0.034	-0.005
Nigeria	Guzamala	2000	0.627	0.998	1.469
Nigeria	Guzamala	2017	0.424	0.650	0.934
Nigeria	Guzamala	2000-2017	-0.048	-0.022	0.004
Nigeria	Gwadabaw	2000	0.504	0.852	1.282
Nigeria	Gwadabaw	2017	0.274	0.445	0.697
Nigeria	Gwadabaw	2000-2017	-0.064	-0.038	-0.010
Nigeria	Gwagwala	2000	0.472	0.763	1.087
Nigeria	Gwagwala	2017	0.208	0.310	0.461
Nigeria	Gwagwala	2000-2017	-0.082	-0.053	-0.026
Nigeria	Gwale	2000	0.499	0.892	1.390
Nigeria	Gwale	2017	0.276	0.456	0.699
Nigeria	Gwale	2000-2017	-0.077	-0.039	0.006
Nigeria	Gwandu	2000	0.505	0.832	1.246
Nigeria	Gwandu	2017	0.259	0.408	0.636
Nigeria	Gwandu	2000-2017	-0.067	-0.042	-0.013
Nigeria	Gwaram	2000	0.614	0.955	1.438
Nigeria	Gwaram	2017	0.345	0.543	0.783
Nigeria	Gwaram	2000-2017	-0.062	-0.032	0.000
Nigeria	Gwarzo	2000	0.528	0.839	1.199
Nigeria	Gwarzo	2017	0.291	0.458	0.680
Nigeria	Gwarzo	2000-2017	-0.067	-0.035	-0.001
Nigeria	Gwer East	2000	0.532	0.830	1.190
Nigeria	Gwer East	2017	0.210	0.333	0.496
Nigeria	Gwer East	2000-2017	-0.083	-0.054	-0.026
Nigeria	GwerWest	2000	0.531	0.826	1.220
Nigeria	GwerWest	2017	0.207	0.330	0.486
Nigeria	GwerWest	2000-2017	-0.085	-0.055	-0.026
Nigeria	Gwiwa	2000	0.548	0.885	1.360
Nigeria	Gwiwa	2017	0.328	0.499	0.737
Nigeria	Gwiwa	2000-2017	-0.063	-0.033	-0.003
Nigeria	Gwoza	2000	0.715	1.110	1.644
Nigeria	Gwoza	2017	0.426	0.662	0.994
Nigeria	Gwoza	2000-2017	-0.053	-0.027	0.001
Nigeria	Hadejia	2000	0.544	0.972	1.587
Nigeria	Hadejia	2017	0.319	0.564	0.851
Nigeria	Hadejia	2000-2017	-0.073	-0.031	0.019
Nigeria	Hawul	2000	0.683	1.081	1.573
Nigeria	Hawul	2017	0.421	0.632	0.957
Nigeria	Hawul	2000-2017	-0.056	-0.029	-0.003
Nigeria	Hong	2000	0.691	1.072	1.630
Nigeria	Hong	2017	0.399	0.593	0.910
Nigeria	Hong	2000-2017	-0.056	-0.031	-0.002
Nigeria	IbadanNorth	2000	0.307	0.519	0.819
Nigeria	IbadanNorth	2017	0.132	0.225	0.346
Nigeria	IbadanNorth	2000-2017	-0.092	-0.053	-0.015
Nigeria	IbadanNorth-East	2000	0.291	0.506	0.786
Nigeria	IbadanNorth-East	2017	0.135	0.222	0.332
Nigeria	IbadanNorth-East	2000-2017	-0.093	-0.053	-0.013
Nigeria	IbadanNorth-West	2000	0.308	0.528	0.870
Nigeria	IbadanNorth-West	2017	0.135	0.230	0.357
Nigeria	IbadanNorth-West	2000-2017	-0.091	-0.052	-0.010

Table 1: LRI DALYs rate by unit *(continued)*

Country	Unit	year	mean rate	lower rate	upper rate
Nigeria	IbadanSouth-East	2000	0.281	0.508	0.831
Nigeria	IbadanSouth-East	2017	0.140	0.220	0.332
Nigeria	IbadanSouth-East	2000-2017	-0.091	-0.051	-0.015
Nigeria	IbadanSouth-West	2000	0.281	0.506	0.799
Nigeria	IbadanSouth-West	2017	0.135	0.220	0.330
Nigeria	IbadanSouth-West	2000-2017	-0.089	-0.051	-0.011
Nigeria	Ibaji	2000	0.477	0.772	1.156
Nigeria	Ibaji	2017	0.188	0.287	0.404
Nigeria	Ibaji	2000-2017	-0.082	-0.057	-0.029
Nigeria	Ibarapa Central	2000	0.407	0.641	0.966
Nigeria	Ibarapa Central	2017	0.144	0.227	0.335
Nigeria	Ibarapa Central	2000-2017	-0.089	-0.061	-0.034
Nigeria	Ibarapa East	2000	0.396	0.631	0.923
Nigeria	Ibarapa East	2017	0.140	0.226	0.348
Nigeria	Ibarapa East	2000-2017	-0.088	-0.061	-0.034
Nigeria	Ibarapa North	2000	0.393	0.636	0.922
Nigeria	Ibarapa North	2017	0.135	0.222	0.338
Nigeria	Ibarapa North	2000-2017	-0.090	-0.063	-0.037
Nigeria	Ibeju/Lekki	2000	0.331	0.527	0.809
Nigeria	Ibeju/Lekki	2017	0.139	0.224	0.329
Nigeria	Ibeju/Lekki	2000-2017	-0.076	-0.049	-0.022
Nigeria	Ibeno	2000	0.440	0.719	1.102
Nigeria	Ibeno	2017	0.168	0.277	0.403
Nigeria	Ibeno	2000-2017	-0.090	-0.055	-0.019
Nigeria	Ibesikpo Asutan	2000	0.438	0.759	1.139
Nigeria	Ibesikpo Asutan	2017	0.184	0.295	0.459
Nigeria	Ibesikpo Asutan	2000-2017	-0.090	-0.056	-0.022
Nigeria	Ibi	2000	0.603	0.907	1.330
Nigeria	Ibi	2017	0.288	0.445	0.646
Nigeria	Ibi	2000-2017	-0.072	-0.041	-0.012
Nigeria	Ibiono Ibom	2000	0.448	0.710	1.033
Nigeria	Ibiono Ibom	2017	0.177	0.277	0.429
Nigeria	Ibiono Ibom	2000-2017	-0.082	-0.055	-0.024
Nigeria	Idah	2000	0.440	0.722	1.124
Nigeria	Idah	2017	0.178	0.286	0.466
Nigeria	Idah	2000-2017	-0.089	-0.054	-0.020
Nigeria	Idanre	2000	0.400	0.621	0.936
Nigeria	Idanre	2017	0.153	0.229	0.340
Nigeria	Idanre	2000-2017	-0.087	-0.059	-0.032
Nigeria	Ideato South	2000	0.405	0.658	0.992
Nigeria	Ideato South	2017	0.163	0.273	0.427
Nigeria	Ideato South	2000-2017	-0.084	-0.048	-0.011
Nigeria	IdeatoNo	2000	0.387	0.641	0.943
Nigeria	IdeatoNo	2017	0.169	0.273	0.410
Nigeria	IdeatoNo	2000-2017	-0.075	-0.046	-0.014
Nigeria	Idemili North	2000	0.340	0.559	0.841
Nigeria	Idemili North	2017	0.167	0.263	0.414
Nigeria	Idemili North	2000-2017	-0.077	-0.043	-0.008
Nigeria	Idemili South	2000	0.357	0.569	0.851
Nigeria	Idemili South	2017	0.163	0.267	0.410
Nigeria	Idemili South	2000-2017	-0.076	-0.043	-0.011
Nigeria	Ido	2000	0.350	0.578	0.843
Nigeria	Ido	2017	0.148	0.222	0.331
Nigeria	Ido	2000-2017	-0.084	-0.057	-0.029
Nigeria	Ido/Osi	2000	0.369	0.611	0.920
Nigeria	Ido/Osi	2017	0.150	0.234	0.342
Nigeria	Ido/Osi	2000-2017	-0.082	-0.056	-0.028
Nigeria	Ifako/Ijaye	2000	0.355	0.589	0.939
Nigeria	Ifako/Ijaye	2017	0.151	0.251	0.407
Nigeria	Ifako/Ijaye	2000-2017	-0.082	-0.049	-0.010
Nigeria	Ife East	2000	0.373	0.600	0.897

Table 1: LRI DALYs rate by unit *(continued)*

Country	Unit	year	mean rate	lower rate	upper rate
Nigeria	Ife East	2017	0.141	0.222	0.325
Nigeria	Ife East	2000-2017	-0.089	-0.060	-0.026
Nigeria	Ife North	2000	0.342	0.580	0.838
Nigeria	Ife North	2017	0.142	0.223	0.317
Nigeria	Ife North	2000-2017	-0.082	-0.057	-0.030
Nigeria	Ife South	2000	0.397	0.612	0.897
Nigeria	Ife South	2017	0.145	0.224	0.328
Nigeria	Ife South	2000-2017	-0.082	-0.060	-0.031
Nigeria	IfeCentral	2000	0.337	0.571	0.854
Nigeria	IfeCentral	2017	0.134	0.223	0.344
Nigeria	IfeCentral	2000-2017	-0.084	-0.055	-0.019
Nigeria	Ifedayo	2000	0.381	0.632	0.932
Nigeria	Ifedayo	2017	0.158	0.245	0.359
Nigeria	Ifedayo	2000-2017	-0.084	-0.057	-0.021
Nigeria	Ifedore	2000	0.377	0.599	0.923
Nigeria	Ifedore	2017	0.143	0.224	0.334
Nigeria	Ifedore	2000-2017	-0.087	-0.059	-0.028
Nigeria	Ifelodun	2000	0.334	0.557	0.831
Nigeria	Ifelodun	2000	0.463	0.759	1.123
Nigeria	Ifelodun	2017	0.139	0.224	0.332
Nigeria	Ifelodun	2017	0.186	0.281	0.418
Nigeria	Ifelodun	2000-2017	-0.081	-0.057	-0.031
Nigeria	Ifelodun	2000-2017	-0.084	-0.054	-0.021
Nigeria	Ifo	2000	0.382	0.617	0.929
Nigeria	Ifo	2017	0.166	0.260	0.379
Nigeria	Ifo	2000-2017	-0.081	-0.050	-0.020
Nigeria	Igabi	2000	0.503	0.791	1.111
Nigeria	Igabi	2017	0.263	0.404	0.588
Nigeria	Igabi	2000-2017	-0.065	-0.037	-0.010
Nigeria	Igalamela-Odolu	2000	0.456	0.753	1.112
Nigeria	Igalamela-Odolu	2017	0.191	0.289	0.428
Nigeria	Igalamela-Odolu	2000-2017	-0.078	-0.055	-0.028
Nigeria	Igbo-Eti	2000	0.360	0.602	0.914
Nigeria	Igbo-Eti	2017	0.167	0.282	0.433
Nigeria	Igbo-Eti	2000-2017	-0.068	-0.041	-0.012
Nigeria	Igbo-eze North	2000	0.363	0.635	0.978
Nigeria	Igbo-eze North	2017	0.166	0.286	0.449
Nigeria	Igbo-eze North	2000-2017	-0.073	-0.044	-0.014
Nigeria	Igbo-eze South	2000	0.351	0.613	0.960
Nigeria	Igbo-eze South	2017	0.159	0.284	0.430
Nigeria	Igbo-eze South	2000-2017	-0.075	-0.044	-0.013
Nigeria	Igueben	2000	0.392	0.616	0.900
Nigeria	Igueben	2017	0.155	0.242	0.352
Nigeria	Igueben	2000-2017	-0.078	-0.053	-0.025
Nigeria	Ihiala	2000	0.386	0.605	0.911
Nigeria	Ihiala	2017	0.167	0.276	0.440
Nigeria	Ihiala	2000-2017	-0.072	-0.042	-0.012
Nigeria	Ihitte/U	2000	0.385	0.628	0.983
Nigeria	Ihitte/U	2017	0.171	0.259	0.406
Nigeria	Ihitte/U	2000-2017	-0.084	-0.047	-0.015
Nigeria	Ijebu North-East	2000	0.407	0.666	0.970
Nigeria	Ijebu North-East	2017	0.157	0.249	0.368
Nigeria	Ijebu North-East	2000-2017	-0.086	-0.058	-0.029
Nigeria	IjebuEast	2000	0.441	0.675	0.991
Nigeria	IjebuEast	2017	0.164	0.247	0.352
Nigeria	IjebuEast	2000-2017	-0.083	-0.059	-0.032
Nigeria	IjebuNorth	2000	0.410	0.664	0.973
Nigeria	IjebuNorth	2017	0.157	0.248	0.350
Nigeria	IjebuNorth	2000-2017	-0.081	-0.058	-0.030
Nigeria	IjebuOde	2000	0.382	0.594	0.924
Nigeria	IjebuOde	2017	0.157	0.246	0.359

Table 1: LRI DALYs rate by unit *(continued)*

Country	Unit	year	mean rate	lower rate	upper rate
Nigeria	IjebuOde	2000-2017	-0.082	-0.052	-0.023
Nigeria	Ijero	2000	0.374	0.625	0.925
Nigeria	Ijero	2017	0.162	0.242	0.365
Nigeria	Ijero	2000-2017	-0.084	-0.056	-0.028
Nigeria	Ijumu	2000	0.468	0.754	1.132
Nigeria	Ijumu	2017	0.181	0.282	0.405
Nigeria	Ijumu	2000-2017	-0.083	-0.059	-0.030
Nigeria	Ika	2000	0.420	0.730	1.123
Nigeria	Ika	2017	0.172	0.285	0.442
Nigeria	Ika	2000-2017	-0.088	-0.055	-0.022
Nigeria	IkaNorth	2000	0.383	0.594	0.904
Nigeria	IkaNorth	2017	0.164	0.249	0.365
Nigeria	IkaNorth	2000-2017	-0.075	-0.048	-0.015
Nigeria	Ikara	2000	0.543	0.845	1.185
Nigeria	Ikara	2017	0.290	0.457	0.671
Nigeria	Ikara	2000-2017	-0.065	-0.035	-0.005
Nigeria	IkaSouth	2000	0.372	0.596	0.905
Nigeria	IkaSouth	2017	0.162	0.249	0.366
Nigeria	IkaSouth	2000-2017	-0.072	-0.048	-0.019
Nigeria	Ikeduru	2000	0.402	0.617	0.903
Nigeria	Ikeduru	2017	0.171	0.262	0.400
Nigeria	Ikeduru	2000-2017	-0.078	-0.047	-0.015
Nigeria	Ikeja	2000	0.335	0.569	0.887
Nigeria	Ikeja	2017	0.145	0.239	0.374
Nigeria	Ikeja	2000-2017	-0.086	-0.050	-0.011
Nigeria	Ikenne	2000	0.401	0.629	0.968
Nigeria	Ikenne	2017	0.154	0.247	0.371
Nigeria	Ikenne	2000-2017	-0.086	-0.056	-0.025
Nigeria	Ikere	2000	0.351	0.593	0.890
Nigeria	Ikere	2017	0.144	0.225	0.342
Nigeria	Ikere	2000-2017	-0.089	-0.057	-0.024
Nigeria	Ikole	2000	0.382	0.617	0.900
Nigeria	Ikole	2017	0.151	0.232	0.337
Nigeria	Ikole	2000-2017	-0.082	-0.057	-0.031
Nigeria	Ikom	2000	0.492	0.791	1.123
Nigeria	Ikom	2017	0.189	0.311	0.479
Nigeria	Ikom	2000-2017	-0.083	-0.056	-0.026
Nigeria	Ikono	2000	0.432	0.714	1.031
Nigeria	Ikono	2017	0.174	0.279	0.420
Nigeria	Ikono	2000-2017	-0.084	-0.055	-0.024
Nigeria	Ikorodu	2000	0.309	0.511	0.805
Nigeria	Ikorodu	2017	0.138	0.223	0.320
Nigeria	Ikorodu	2000-2017	-0.076	-0.047	-0.015
Nigeria	Ikot-Aba	2000	0.441	0.730	1.059
Nigeria	Ikot-Aba	2017	0.172	0.277	0.412
Nigeria	Ikot-Aba	2000-2017	-0.088	-0.055	-0.022
Nigeria	Ikot-Ekp	2000	0.445	0.702	1.052
Nigeria	Ikot-Ekp	2017	0.174	0.279	0.435
Nigeria	Ikot-Ekp	2000-2017	-0.087	-0.054	-0.017
Nigeria	Ikpoba-Okha	2000	0.346	0.551	0.843
Nigeria	Ikpoba-Okha	2017	0.162	0.236	0.350
Nigeria	Ikpoba-Okha	2000-2017	-0.075	-0.048	-0.016
Nigeria	Ikwerre	2000	0.433	0.685	1.009
Nigeria	Ikwerre	2017	0.169	0.264	0.394
Nigeria	Ikwerre	2000-2017	-0.085	-0.057	-0.024
Nigeria	Ikwo	2000	0.504	0.874	1.330
Nigeria	Ikwo	2017	0.202	0.339	0.507
Nigeria	Ikwo	2000-2017	-0.083	-0.056	-0.027
Nigeria	Ikwuano	2000	0.441	0.696	1.034
Nigeria	Ikwuano	2017	0.169	0.269	0.396
Nigeria	Ikwuano	2000-2017	-0.082	-0.055	-0.022

Table 1: LRI DALYs rate by unit *(continued)*

Country	Unit	year	mean rate	lower rate	upper rate
Nigeria	Ila	2000	0.351	0.595	0.918
Nigeria	Ila	2017	0.150	0.232	0.346
Nigeria	Ila	2000-2017	-0.085	-0.056	-0.022
Nigeria	IlajeEseodo	2000	0.388	0.624	0.911
Nigeria	IlajeEseodo	2017	0.153	0.227	0.333
Nigeria	IlajeEseodo	2000-2017	-0.083	-0.060	-0.032
Nigeria	Ilejemeje	2000	0.374	0.619	0.910
Nigeria	Ilejemeje	2017	0.143	0.232	0.358
Nigeria	Ilejemeje	2000-2017	-0.090	-0.059	-0.027
Nigeria	IleOluji/Okeigbo	2000	0.389	0.612	0.899
Nigeria	IleOluji/Okeigbo	2017	0.149	0.224	0.323
Nigeria	IleOluji/Okeigbo	2000-2017	-0.086	-0.060	-0.031
Nigeria	Ilesha East	2000	0.324	0.558	0.867
Nigeria	Ilesha East	2017	0.139	0.229	0.355
Nigeria	Ilesha East	2000-2017	-0.087	-0.051	-0.014
Nigeria	Ilesha West	2000	0.305	0.513	0.814
Nigeria	Ilesha West	2017	0.138	0.228	0.342
Nigeria	Ilesha West	2000-2017	-0.082	-0.047	-0.010
Nigeria	Illela	2000	0.507	0.875	1.317
Nigeria	Illela	2017	0.309	0.462	0.691
Nigeria	Illela	2000-2017	-0.063	-0.038	-0.010
Nigeria	Ilorin East	2000	0.418	0.722	1.032
Nigeria	Ilorin East	2017	0.184	0.290	0.438
Nigeria	Ilorin East	2000-2017	-0.086	-0.054	-0.021
Nigeria	Ilorin South	2000	0.433	0.751	1.089
Nigeria	Ilorin South	2017	0.184	0.290	0.445
Nigeria	Ilorin South	2000-2017	-0.088	-0.057	-0.023
Nigeria	IlorinWe	2000	0.397	0.695	1.070
Nigeria	IlorinWe	2017	0.177	0.287	0.429
Nigeria	IlorinWe	2000-2017	-0.087	-0.051	-0.016
Nigeria	Imeko-Afon	2000	0.458	0.751	1.114
Nigeria	Imeko-Afon	2017	0.159	0.257	0.381
Nigeria	Imeko-Afon	2000-2017	-0.090	-0.062	-0.036
Nigeria	Ingawa	2000	0.547	0.859	1.297
Nigeria	Ingawa	2017	0.308	0.479	0.695
Nigeria	Ingawa	2000-2017	-0.064	-0.033	-0.005
Nigeria	Ini	2000	0.434	0.695	0.992
Nigeria	Ini	2017	0.172	0.273	0.417
Nigeria	Ini	2000-2017	-0.085	-0.054	-0.023
Nigeria	Ipokia	2000	0.472	0.739	1.065
Nigeria	Ipokia	2017	0.175	0.269	0.385
Nigeria	Ipokia	2000-2017	-0.083	-0.057	-0.031
Nigeria	Irele	2000	0.399	0.607	0.899
Nigeria	Irele	2017	0.144	0.219	0.323
Nigeria	Irele	2000-2017	-0.083	-0.061	-0.031
Nigeria	Irepo	2000	0.380	0.636	0.953
Nigeria	Irepo	2017	0.154	0.241	0.377
Nigeria	Irepo	2000-2017	-0.085	-0.057	-0.029
Nigeria	Irepodun	2000	0.360	0.583	0.867
Nigeria	Irepodun	2000	0.462	0.751	1.102
Nigeria	Irepodun	2017	0.138	0.228	0.370
Nigeria	Irepodun	2017	0.173	0.275	0.400
Nigeria	Irepodun	2000-2017	-0.088	-0.060	-0.035
Nigeria	Irepodun	2000-2017	-0.091	-0.056	-0.021
Nigeria	Irepodun/Ifelodun	2000	0.365	0.601	0.885
Nigeria	Irepodun/Ifelodun	2017	0.155	0.228	0.329
Nigeria	Irepodun/Ifelodun	2000-2017	-0.083	-0.057	-0.027
Nigeria	Irewole	2000	0.352	0.574	0.848
Nigeria	Irewole	2017	0.141	0.223	0.328
Nigeria	Irewole	2000-2017	-0.086	-0.055	-0.026
Nigeria	Isa	2000	0.506	0.855	1.284

Table 1: LRI DALYs rate by unit *(continued)*

Country	Unit	year	mean rate	lower rate	upper rate
Nigeria	Isa	2017	0.298	0.450	0.671
Nigeria	Isa	2000-2017	-0.066	-0.037	-0.006
Nigeria	Ise/Orun	2000	0.383	0.625	0.937
Nigeria	Ise/Orun	2017	0.158	0.231	0.330
Nigeria	Ise/Orun	2000-2017	-0.086	-0.058	-0.030
Nigeria	Iseyin	2000	0.363	0.603	0.890
Nigeria	Iseyin	2017	0.140	0.228	0.354
Nigeria	Iseyin	2000-2017	-0.082	-0.058	-0.029
Nigeria	Ishielu	2000	0.519	0.848	1.301
Nigeria	Ishielu	2017	0.201	0.327	0.478
Nigeria	Ishielu	2000-2017	-0.081	-0.054	-0.027
Nigeria	Isi-Uzo	2000	0.406	0.672	1.016
Nigeria	Isi-Uzo	2017	0.170	0.282	0.419
Nigeria	Isi-Uzo	2000-2017	-0.075	-0.050	-0.023
Nigeria	Isiala Ngwa North	2000	0.437	0.708	1.010
Nigeria	Isiala Ngwa North	2017	0.181	0.279	0.423
Nigeria	Isiala Ngwa North	2000-2017	-0.085	-0.056	-0.025
Nigeria	Isiala Ngwa South	2000	0.426	0.711	1.057
Nigeria	Isiala Ngwa South	2017	0.170	0.276	0.426
Nigeria	Isiala Ngwa South	2000-2017	-0.086	-0.055	-0.023
Nigeria	IsialaMb	2000	0.400	0.629	0.900
Nigeria	IsialaMb	2017	0.169	0.266	0.397
Nigeria	IsialaMb	2000-2017	-0.077	-0.047	-0.018
Nigeria	Isin	2000	0.458	0.744	1.116
Nigeria	Isin	2017	0.169	0.274	0.412
Nigeria	Isin	2000-2017	-0.082	-0.058	-0.031
Nigeria	Isokan	2000	0.367	0.604	0.874
Nigeria	Isokan	2017	0.137	0.218	0.317
Nigeria	Isokan	2000-2017	-0.090	-0.061	-0.029
Nigeria	IsokoNor	2000	0.390	0.627	0.911
Nigeria	IsokoNor	2017	0.160	0.250	0.378
Nigeria	IsokoNor	2000-2017	-0.081	-0.052	-0.023
Nigeria	IsokoSou	2000	0.409	0.646	0.933
Nigeria	IsokoSou	2017	0.161	0.254	0.376
Nigeria	IsokoSou	2000-2017	-0.080	-0.054	-0.025
Nigeria	Isu	2000	0.353	0.579	0.919
Nigeria	Isu	2017	0.148	0.275	0.453
Nigeria	Isu	2000-2017	-0.086	-0.040	0.003
Nigeria	Isuikwua	2000	0.431	0.697	1.029
Nigeria	Isuikwua	2017	0.175	0.276	0.413
Nigeria	Isuikwua	2000-2017	-0.082	-0.054	-0.023
Nigeria	Itas/Gad	2000	0.600	0.957	1.441
Nigeria	Itas/Gad	2017	0.358	0.555	0.781
Nigeria	Itas/Gad	2000-2017	-0.061	-0.031	0.000
Nigeria	Itesiwaju	2000	0.385	0.622	0.922
Nigeria	Itesiwaju	2017	0.142	0.228	0.354
Nigeria	Itesiwaju	2000-2017	-0.086	-0.061	-0.033
Nigeria	Itu	2000	0.431	0.718	1.052
Nigeria	Itu	2017	0.175	0.278	0.404
Nigeria	Itu	2000-2017	-0.087	-0.056	-0.021
Nigeria	Ivo	2000	0.479	0.800	1.164
Nigeria	Ivo	2017	0.193	0.317	0.476
Nigeria	Ivo	2000-2017	-0.085	-0.053	-0.020
Nigeria	Iwajowa	2000	0.394	0.639	0.936
Nigeria	Iwajowa	2017	0.144	0.227	0.352
Nigeria	Iwajowa	2000-2017	-0.087	-0.062	-0.035
Nigeria	Iwo	2000	0.360	0.571	0.812
Nigeria	Iwo	2017	0.143	0.226	0.346
Nigeria	Iwo	2000-2017	-0.089	-0.054	-0.024
Nigeria	Izzi	2000	0.490	0.895	1.387
Nigeria	Izzi	2017	0.203	0.356	0.545

Table 1: LRI DALYs rate by unit *(continued)*

Country	Unit	year	mean rate	lower rate	upper rate
Nigeria	Izzi	2000-2017	-0.081	-0.054	-0.024
Nigeria	Jaba	2000	0.452	0.742	1.044
Nigeria	Jaba	2017	0.232	0.371	0.540
Nigeria	Jaba	2000-2017	-0.067	-0.039	-0.008
Nigeria	Jada	2000	0.661	1.070	1.569
Nigeria	Jada	2017	0.376	0.547	0.834
Nigeria	Jada	2000-2017	-0.064	-0.037	-0.010
Nigeria	Jahun	2000	0.585	0.932	1.418
Nigeria	Jahun	2017	0.341	0.537	0.789
Nigeria	Jahun	2000-2017	-0.059	-0.031	0.001
Nigeria	Jakusko	2000	0.612	0.982	1.515
Nigeria	Jakusko	2017	0.388	0.609	0.861
Nigeria	Jakusko	2000-2017	-0.056	-0.026	0.002
Nigeria	Jalingo	2000	0.631	1.013	1.587
Nigeria	Jalingo	2017	0.307	0.489	0.736
Nigeria	Jalingo	2000-2017	-0.078	-0.039	0.000
Nigeria	Jama'are	2000	0.616	0.958	1.422
Nigeria	Jama'are	2017	0.347	0.550	0.787
Nigeria	Jama'are	2000-2017	-0.062	-0.031	0.002
Nigeria	Jega	2000	0.478	0.821	1.195
Nigeria	Jega	2017	0.238	0.398	0.610
Nigeria	Jega	2000-2017	-0.067	-0.042	-0.007
Nigeria	Jema'a	2000	0.509	0.812	1.185
Nigeria	Jema'a	2017	0.253	0.385	0.551
Nigeria	Jema'a	2000-2017	-0.070	-0.042	-0.013
Nigeria	Jere	2000	0.677	1.082	1.628
Nigeria	Jere	2017	0.415	0.648	0.988
Nigeria	Jere	2000-2017	-0.054	-0.027	0.001
Nigeria	Jibia	2000	0.637	0.957	1.370
Nigeria	Jibia	2017	0.313	0.473	0.680
Nigeria	Jibia	2000-2017	-0.067	-0.040	-0.010
Nigeria	Jos East	2000	0.562	0.870	1.256
Nigeria	Jos East	2017	0.278	0.436	0.639
Nigeria	Jos East	2000-2017	-0.070	-0.040	-0.009
Nigeria	Jos North	2000	0.570	0.871	1.265
Nigeria	Jos North	2017	0.249	0.392	0.566
Nigeria	Jos North	2000-2017	-0.077	-0.045	-0.013
Nigeria	Jos South	2000	0.557	0.849	1.205
Nigeria	Jos South	2017	0.244	0.384	0.550
Nigeria	Jos South	2000-2017	-0.077	-0.045	-0.014
Nigeria	Kabba/Bu	2000	0.466	0.754	1.133
Nigeria	Kabba/Bu	2017	0.188	0.285	0.418
Nigeria	Kabba/Bu	2000-2017	-0.084	-0.058	-0.030
Nigeria	Kabo	2000	0.524	0.855	1.264
Nigeria	Kabo	2017	0.317	0.468	0.683
Nigeria	Kabo	2000-2017	-0.064	-0.034	-0.002
Nigeria	Kachia	2000	0.453	0.714	1.010
Nigeria	Kachia	2017	0.253	0.376	0.558
Nigeria	Kachia	2000-2017	-0.064	-0.037	-0.009
Nigeria	Kaduna North	2000	0.525	0.846	1.217
Nigeria	Kaduna North	2017	0.236	0.373	0.554
Nigeria	Kaduna North	2000-2017	-0.078	-0.045	-0.013
Nigeria	Kaduna South	2000	0.523	0.844	1.211
Nigeria	Kaduna South	2017	0.237	0.368	0.582
Nigeria	Kaduna South	2000-2017	-0.078	-0.047	-0.011
Nigeria	KafinHau	2000	0.602	0.961	1.460
Nigeria	KafinHau	2017	0.369	0.565	0.834
Nigeria	KafinHau	2000-2017	-0.060	-0.030	0.001
Nigeria	Kafur	2000	0.533	0.832	1.203
Nigeria	Kafur	2017	0.289	0.443	0.630
Nigeria	Kafur	2000-2017	-0.068	-0.036	-0.007

Table 1: LRI DALYs rate by unit *(continued)*

Country	Unit	year	mean rate	lower rate	upper rate
Nigeria	Kaga	2000	0.687	1.087	1.686
Nigeria	Kaga	2017	0.452	0.699	1.011
Nigeria	Kaga	2000-2017	-0.050	-0.024	0.003
Nigeria	Kagarko	2000	0.465	0.729	1.030
Nigeria	Kagarko	2017	0.223	0.345	0.501
Nigeria	Kagarko	2000-2017	-0.069	-0.043	-0.014
Nigeria	Kaiama	2000	0.473	0.782	1.152
Nigeria	Kaiama	2017	0.199	0.295	0.432
Nigeria	Kaiama	2000-2017	-0.078	-0.056	-0.030
Nigeria	Kaita	2000	0.549	0.871	1.258
Nigeria	Kaita	2017	0.317	0.482	0.688
Nigeria	Kaita	2000-2017	-0.062	-0.034	-0.005
Nigeria	Kajola	2000	0.359	0.603	0.896
Nigeria	Kajola	2017	0.142	0.226	0.343
Nigeria	Kajola	2000-2017	-0.083	-0.059	-0.033
Nigeria	Kajuru	2000	0.473	0.726	1.002
Nigeria	Kajuru	2017	0.279	0.431	0.617
Nigeria	Kajuru	2000-2017	-0.059	-0.030	-0.001
Nigeria	Kala/Balge	2000	0.711	1.111	1.683
Nigeria	Kala/Balge	2017	0.440	0.662	0.981
Nigeria	Kala/Balge	2000-2017	-0.053	-0.027	-0.001
Nigeria	Kalgo	2000	0.511	0.823	1.211
Nigeria	Kalgo	2017	0.252	0.400	0.617
Nigeria	Kalgo	2000-2017	-0.067	-0.042	-0.012
Nigeria	Kaltungo	2000	0.649	1.028	1.517
Nigeria	Kaltungo	2017	0.398	0.600	0.901
Nigeria	Kaltungo	2000-2017	-0.059	-0.030	-0.004
Nigeria	Kanam	2000	0.617	0.976	1.421
Nigeria	Kanam	2017	0.318	0.502	0.720
Nigeria	Kanam	2000-2017	-0.066	-0.038	-0.010
Nigeria	Kankara	2000	0.508	0.820	1.254
Nigeria	Kankara	2017	0.277	0.435	0.641
Nigeria	Kankara	2000-2017	-0.066	-0.037	-0.007
Nigeria	Kanke	2000	0.590	0.918	1.347
Nigeria	Kanke	2017	0.280	0.439	0.636
Nigeria	Kanke	2000-2017	-0.068	-0.042	-0.013
Nigeria	Kankiya	2000	0.541	0.843	1.242
Nigeria	Kankiya	2017	0.297	0.465	0.663
Nigeria	Kankiya	2000-2017	-0.066	-0.034	-0.003
Nigeria	Kano	2000	0.487	0.881	1.467
Nigeria	Kano	2017	0.272	0.479	0.794
Nigeria	Kano	2000-2017	-0.085	-0.035	0.009
Nigeria	Karasuwa	2000	0.562	0.971	1.459
Nigeria	Karasuwa	2017	0.391	0.606	0.889
Nigeria	Karasuwa	2000-2017	-0.057	-0.026	0.003
Nigeria	Karaye	2000	0.530	0.841	1.238
Nigeria	Karaye	2017	0.291	0.462	0.686
Nigeria	Karaye	2000-2017	-0.064	-0.035	-0.005
Nigeria	Karim-La	2000	0.643	0.999	1.490
Nigeria	Karim-La	2017	0.350	0.544	0.778
Nigeria	Karim-La	2000-2017	-0.063	-0.034	-0.006
Nigeria	Karu	2000	0.523	0.819	1.182
Nigeria	Karu	2017	0.216	0.336	0.498
Nigeria	Karu	2000-2017	-0.079	-0.052	-0.024
Nigeria	Katagum	2000	0.611	0.972	1.442
Nigeria	Katagum	2017	0.371	0.570	0.800
Nigeria	Katagum	2000-2017	-0.062	-0.030	-0.001
Nigeria	Katcha	2000	0.472	0.752	1.072
Nigeria	Katcha	2017	0.214	0.333	0.460
Nigeria	Katcha	2000-2017	-0.073	-0.047	-0.019
Nigeria	Katsina (Benue)	2000	0.619	0.907	1.304

Table 1: LRI DALYs rate by unit *(continued)*

Country	Unit	year	mean rate	lower rate	upper rate
Nigeria	Katsina (Benue)	2017	0.251	0.382	0.567
Nigeria	Katsina (Benue)	2000-2017	-0.079	-0.051	-0.022
Nigeria	Katsina (K)	2000	0.507	0.857	1.304
Nigeria	Katsina (K)	2017	0.302	0.477	0.705
Nigeria	Katsina (K)	2000-2017	-0.070	-0.033	0.002
Nigeria	Kaugama	2000	0.587	0.942	1.387
Nigeria	Kaugama	2017	0.365	0.559	0.826
Nigeria	Kaugama	2000-2017	-0.058	-0.029	0.001
Nigeria	Kaura	2000	0.521	0.810	1.198
Nigeria	Kaura	2017	0.256	0.394	0.579
Nigeria	Kaura	2000-2017	-0.070	-0.041	-0.010
Nigeria	Kaura-Na	2000	0.482	0.824	1.238
Nigeria	Kaura-Na	2017	0.289	0.435	0.657
Nigeria	Kaura-Na	2000-2017	-0.063	-0.037	-0.008
Nigeria	Kauru	2000	0.486	0.747	1.057
Nigeria	Kauru	2017	0.284	0.440	0.642
Nigeria	Kauru	2000-2017	-0.059	-0.031	-0.001
Nigeria	Kazaure	2000	0.535	0.884	1.357
Nigeria	Kazaure	2017	0.318	0.505	0.746
Nigeria	Kazaure	2000-2017	-0.069	-0.032	0.003
Nigeria	Keana	2000	0.572	0.877	1.279
Nigeria	Keana	2017	0.247	0.379	0.550
Nigeria	Keana	2000-2017	-0.075	-0.047	-0.022
Nigeria	Kebbe	2000	0.494	0.815	1.226
Nigeria	Kebbe	2017	0.249	0.398	0.603
Nigeria	Kebbe	2000-2017	-0.066	-0.041	-0.015
Nigeria	Keffi	2000	0.518	0.840	1.242
Nigeria	Keffi	2017	0.219	0.347	0.502
Nigeria	Keffi	2000-2017	-0.081	-0.050	-0.015
Nigeria	Khana	2000	0.442	0.707	1.039
Nigeria	Khana	2017	0.166	0.267	0.401
Nigeria	Khana	2000-2017	-0.082	-0.056	-0.022
Nigeria	Kibiya	2000	0.560	0.879	1.263
Nigeria	Kibiya	2017	0.307	0.481	0.721
Nigeria	Kibiya	2000-2017	-0.063	-0.034	-0.003
Nigeria	Kirfi	2000	0.638	1.014	1.526
Nigeria	Kirfi	2017	0.377	0.592	0.871
Nigeria	Kirfi	2000-2017	-0.058	-0.030	-0.002
Nigeria	KiriKasa	2000	0.586	0.955	1.476
Nigeria	KiriKasa	2017	0.384	0.580	0.857
Nigeria	KiriKasa	2000-2017	-0.056	-0.028	0.004
Nigeria	Kiru	2000	0.548	0.845	1.225
Nigeria	Kiru	2017	0.281	0.460	0.690
Nigeria	Kiru	2000-2017	-0.065	-0.035	-0.006
Nigeria	Kiyawa	2000	0.620	0.937	1.359
Nigeria	Kiyawa	2017	0.342	0.534	0.777
Nigeria	Kiyawa	2000-2017	-0.060	-0.032	0.000
Nigeria	Koko/Bes	2000	0.471	0.786	1.193
Nigeria	Koko/Bes	2017	0.232	0.378	0.562
Nigeria	Koko/Bes	2000-2017	-0.068	-0.043	-0.014
Nigeria	Kokona	2000	0.569	0.854	1.223
Nigeria	Kokona	2017	0.231	0.359	0.538
Nigeria	Kokona	2000-2017	-0.080	-0.049	-0.019
Nigeria	Kolokuma/Opokuma	2000	0.442	0.715	1.071
Nigeria	Kolokuma/Opokuma	2017	0.169	0.282	0.439
Nigeria	Kolokuma/Opokuma	2000-2017	-0.080	-0.054	-0.021
Nigeria	Konduga	2000	0.692	1.100	1.630
Nigeria	Konduga	2017	0.409	0.655	0.965
Nigeria	Konduga	2000-2017	-0.057	-0.028	0.001
Nigeria	Konshish	2000	0.554	0.850	1.228
Nigeria	Konshish	2017	0.216	0.343	0.496

Table 1: LRI DALYs rate by unit *(continued)*

Country	Unit	year	mean rate	lower rate	upper rate
Nigeria	Konshish	2000-2017	-0.084	-0.054	-0.027
Nigeria	Kontogur	2000	0.483	0.797	1.191
Nigeria	Kontogur	2017	0.228	0.352	0.523
Nigeria	Kontogur	2000-2017	-0.076	-0.048	-0.020
Nigeria	Kosofe	2000	0.326	0.549	0.868
Nigeria	Kosofe	2017	0.145	0.236	0.357
Nigeria	Kosofe	2000-2017	-0.079	-0.047	-0.012
Nigeria	Kotonkar	2000	0.479	0.760	1.125
Nigeria	Kotonkar	2017	0.188	0.289	0.421
Nigeria	Kotonkar	2000-2017	-0.082	-0.056	-0.026
Nigeria	Kubau	2000	0.538	0.836	1.215
Nigeria	Kubau	2017	0.297	0.459	0.654
Nigeria	Kubau	2000-2017	-0.061	-0.033	-0.003
Nigeria	Kudan	2000	0.528	0.832	1.211
Nigeria	Kudan	2017	0.272	0.442	0.665
Nigeria	Kudan	2000-2017	-0.068	-0.036	-0.005
Nigeria	Kuje	2000	0.486	0.758	1.103
Nigeria	Kuje	2017	0.193	0.297	0.437
Nigeria	Kuje	2000-2017	-0.082	-0.056	-0.029
Nigeria	Kukawa	2000	0.636	1.020	1.519
Nigeria	Kukawa	2017	0.439	0.650	0.930
Nigeria	Kukawa	2000-2017	-0.046	-0.023	0.005
Nigeria	Kumbotso	2000	0.553	0.870	1.320
Nigeria	Kumbotso	2017	0.311	0.489	0.703
Nigeria	Kumbotso	2000-2017	-0.066	-0.033	-0.001
Nigeria	Kunchi	2000	0.534	0.865	1.275
Nigeria	Kunchi	2017	0.316	0.486	0.698
Nigeria	Kunchi	2000-2017	-0.063	-0.033	-0.001
Nigeria	Kura	2000	0.547	0.869	1.334
Nigeria	Kura	2017	0.311	0.482	0.711
Nigeria	Kura	2000-2017	-0.068	-0.035	-0.001
Nigeria	Kurfi	2000	0.514	0.844	1.246
Nigeria	Kurfi	2017	0.291	0.461	0.664
Nigeria	Kurfi	2000-2017	-0.065	-0.035	-0.007
Nigeria	Kurmi	2000	0.648	0.961	1.370
Nigeria	Kurmi	2017	0.286	0.441	0.671
Nigeria	Kurmi	2000-2017	-0.071	-0.043	-0.017
Nigeria	Kusada	2000	0.523	0.844	1.281
Nigeria	Kusada	2017	0.309	0.471	0.677
Nigeria	Kusada	2000-2017	-0.066	-0.034	-0.002
Nigeria	Kwali	2000	0.483	0.759	1.127
Nigeria	Kwali	2017	0.199	0.303	0.446
Nigeria	Kwali	2000-2017	-0.080	-0.055	-0.029
Nigeria	Kwami	2000	0.643	1.023	1.532
Nigeria	Kwami	2017	0.410	0.623	0.912
Nigeria	Kwami	2000-2017	-0.056	-0.028	0.001
Nigeria	Kwande	2000	0.569	0.884	1.259
Nigeria	Kwande	2017	0.236	0.365	0.559
Nigeria	Kwande	2000-2017	-0.082	-0.052	-0.024
Nigeria	Kware	2000	0.518	0.836	1.261
Nigeria	Kware	2017	0.281	0.436	0.673
Nigeria	Kware	2000-2017	-0.065	-0.038	-0.011
Nigeria	Kwaya Kusar	2000	0.664	1.074	1.558
Nigeria	Kwaya Kusar	2017	0.425	0.646	0.963
Nigeria	Kwaya Kusar	2000-2017	-0.057	-0.027	0.002
Nigeria	Lafia	2000	0.560	0.854	1.220
Nigeria	Lafia	2017	0.239	0.370	0.534
Nigeria	Lafia	2000-2017	-0.075	-0.048	-0.022
Nigeria	Lagelu	2000	0.327	0.542	0.821
Nigeria	Lagelu	2017	0.139	0.221	0.346
Nigeria	Lagelu	2000-2017	-0.082	-0.054	-0.024

Table 1: LRI DALYs rate by unit *(continued)*

Country	Unit	year	mean rate	lower rate	upper rate
Nigeria	LagosIsland	2000	0.309	0.527	0.823
Nigeria	LagosIsland	2017	0.147	0.228	0.332
Nigeria	LagosIsland	2000-2017	-0.078	-0.047	-0.013
Nigeria	Lake Chad	2000	0.659	1.068	1.596
Nigeria	Lake Chad	2017	0.414	0.621	0.904
Nigeria	Lake Chad	2000-2017	-0.052	-0.028	-0.002
Nigeria	Lamurde	2000	0.664	1.052	1.537
Nigeria	Lamurde	2017	0.368	0.545	0.809
Nigeria	Lamurde	2000-2017	-0.064	-0.035	-0.006
Nigeria	Langtang North	2000	0.597	0.950	1.390
Nigeria	Langtang North	2017	0.282	0.446	0.642
Nigeria	Langtang North	2000-2017	-0.074	-0.043	-0.012
Nigeria	Langtang South	2000	0.581	0.903	1.332
Nigeria	Langtang South	2017	0.285	0.443	0.647
Nigeria	Langtang South	2000-2017	-0.070	-0.041	-0.013
Nigeria	Lapai	2000	0.493	0.776	1.134
Nigeria	Lapai	2017	0.212	0.318	0.465
Nigeria	Lapai	2000-2017	-0.078	-0.051	-0.024
Nigeria	Lau	2000	0.654	1.020	1.471
Nigeria	Lau	2017	0.361	0.547	0.796
Nigeria	Lau	2000-2017	-0.063	-0.034	-0.007
Nigeria	Lavun	2000	0.471	0.765	1.161
Nigeria	Lavun	2017	0.221	0.324	0.476
Nigeria	Lavun	2000-2017	-0.079	-0.050	-0.021
Nigeria	Lere	2000	0.529	0.803	1.174
Nigeria	Lere	2017	0.289	0.459	0.648
Nigeria	Lere	2000-2017	-0.063	-0.032	-0.003
Nigeria	Logo	2000	0.596	0.887	1.273
Nigeria	Logo	2017	0.247	0.375	0.540
Nigeria	Logo	2000-2017	-0.076	-0.050	-0.022
Nigeria	Lokoja	2000	0.487	0.759	1.146
Nigeria	Lokoja	2017	0.188	0.288	0.426
Nigeria	Lokoja	2000-2017	-0.082	-0.057	-0.028
Nigeria	Machina	2000	0.613	0.999	1.494
Nigeria	Machina	2017	0.377	0.580	0.828
Nigeria	Machina	2000-2017	-0.059	-0.030	0.001
Nigeria	Madagali	2000	0.709	1.093	1.596
Nigeria	Madagali	2017	0.390	0.612	0.897
Nigeria	Madagali	2000-2017	-0.057	-0.031	-0.002
Nigeria	Madobi	2000	0.536	0.864	1.264
Nigeria	Madobi	2017	0.317	0.483	0.715
Nigeria	Madobi	2000-2017	-0.064	-0.034	-0.004
Nigeria	Mafa	2000	0.689	1.067	1.598
Nigeria	Mafa	2017	0.423	0.655	0.986
Nigeria	Mafa	2000-2017	-0.049	-0.026	0.001
Nigeria	Magama	2000	0.462	0.782	1.169
Nigeria	Magama	2017	0.241	0.354	0.495
Nigeria	Magama	2000-2017	-0.068	-0.046	-0.019
Nigeria	Magumeri	2000	0.653	1.052	1.576
Nigeria	Magumeri	2017	0.443	0.680	0.979
Nigeria	Magumeri	2000-2017	-0.050	-0.023	0.002
Nigeria	Mai'Adua	2000	0.566	0.900	1.341
Nigeria	Mai'Adua	2017	0.314	0.507	0.774
Nigeria	Mai'Adua	2000-2017	-0.062	-0.032	-0.003
Nigeria	Maidugur	2000	0.674	1.110	1.749
Nigeria	Maidugur	2017	0.398	0.632	0.987
Nigeria	Maidugur	2000-2017	-0.056	-0.030	0.001
Nigeria	Maigatari	2000	0.629	0.982	1.469
Nigeria	Maigatari	2017	0.361	0.551	0.813
Nigeria	Maigatari	2000-2017	-0.061	-0.032	-0.001
Nigeria	Maiha	2000	0.739	1.146	1.686

Table 1: LRI DALYs rate by unit *(continued)*

Country	Unit	year	mean rate	lower rate	upper rate
Nigeria	Maiha	2017	0.393	0.595	0.880
Nigeria	Maiha	2000-2017	-0.064	-0.034	-0.005
Nigeria	Mainland	2000	0.318	0.529	0.820
Nigeria	Mainland	2017	0.134	0.231	0.357
Nigeria	Mainland	2000-2017	-0.083	-0.047	-0.011
Nigeria	Maiyama	2000	0.488	0.808	1.215
Nigeria	Maiyama	2017	0.246	0.391	0.598
Nigeria	Maiyama	2000-2017	-0.066	-0.042	-0.013
Nigeria	Makarfi	2000	0.532	0.839	1.186
Nigeria	Makarfi	2017	0.285	0.448	0.644
Nigeria	Makarfi	2000-2017	-0.065	-0.036	-0.005
Nigeria	Makoda	2000	0.558	0.887	1.361
Nigeria	Makoda	2017	0.317	0.500	0.729
Nigeria	Makoda	2000-2017	-0.064	-0.032	-0.001
Nigeria	Makurdi	2000	0.524	0.820	1.209
Nigeria	Makurdi	2017	0.221	0.348	0.494
Nigeria	Makurdi	2000-2017	-0.077	-0.048	-0.020
Nigeria	MalamMad	2000	0.586	0.955	1.454
Nigeria	MalamMad	2017	0.369	0.575	0.835
Nigeria	MalamMad	2000-2017	-0.058	-0.028	0.005
Nigeria	Malumfashi	2000	0.513	0.825	1.188
Nigeria	Malumfashi	2017	0.284	0.444	0.637
Nigeria	Malumfashi	2000-2017	-0.066	-0.036	-0.006
Nigeria	Mangu	2000	0.558	0.880	1.270
Nigeria	Mangu	2017	0.247	0.396	0.577
Nigeria	Mangu	2000-2017	-0.072	-0.045	-0.018
Nigeria	Mani	2000	0.544	0.869	1.266
Nigeria	Mani	2017	0.310	0.484	0.701
Nigeria	Mani	2000-2017	-0.061	-0.034	-0.004
Nigeria	Maradun	2000	0.522	0.833	1.248
Nigeria	Maradun	2017	0.284	0.433	0.641
Nigeria	Maradun	2000-2017	-0.064	-0.038	-0.010
Nigeria	Mariga	2000	0.490	0.795	1.175
Nigeria	Mariga	2017	0.261	0.384	0.562
Nigeria	Mariga	2000-2017	-0.067	-0.042	-0.015
Nigeria	Marte	2000	0.684	1.068	1.598
Nigeria	Marte	2017	0.406	0.639	0.930
Nigeria	Marte	2000-2017	-0.054	-0.027	0.000
Nigeria	Maru	2000	0.506	0.818	1.217
Nigeria	Maru	2017	0.276	0.410	0.599
Nigeria	Maru	2000-2017	-0.067	-0.040	-0.013
Nigeria	Mashegu	2000	0.475	0.777	1.175
Nigeria	Mashegu	2017	0.240	0.343	0.482
Nigeria	Mashegu	2000-2017	-0.070	-0.047	-0.021
Nigeria	Mashi	2000	0.560	0.892	1.296
Nigeria	Mashi	2017	0.319	0.494	0.722
Nigeria	Mashi	2000-2017	-0.063	-0.035	-0.003
Nigeria	Matazu	2000	0.495	0.833	1.277
Nigeria	Matazu	2017	0.301	0.456	0.667
Nigeria	Matazu	2000-2017	-0.066	-0.035	-0.002
Nigeria	Mayo-Bel	2000	0.654	1.055	1.548
Nigeria	Mayo-Bel	2017	0.360	0.538	0.779
Nigeria	Mayo-Bel	2000-2017	-0.066	-0.036	-0.009
Nigeria	Mbaitoli	2000	0.379	0.595	0.857
Nigeria	Mbaitoli	2017	0.162	0.268	0.419
Nigeria	Mbaitoli	2000-2017	-0.076	-0.044	-0.013
Nigeria	Mbo	2000	0.441	0.724	1.026
Nigeria	Mbo	2017	0.167	0.279	0.412
Nigeria	Mbo	2000-2017	-0.087	-0.056	-0.018
Nigeria	Michika	2000	0.681	1.084	1.614
Nigeria	Michika	2017	0.394	0.587	0.884

Table 1: LRI DALYs rate by unit *(continued)*

Country	Unit	year	mean rate	lower rate	upper rate
Nigeria	Michika	2000-2017	-0.063	-0.032	-0.002
Nigeria	Miga	2000	0.575	0.933	1.406
Nigeria	Miga	2017	0.355	0.545	0.809
Nigeria	Miga	2000-2017	-0.060	-0.030	0.001
Nigeria	Mikang	2000	0.615	0.919	1.350
Nigeria	Mikang	2017	0.276	0.433	0.623
Nigeria	Mikang	2000-2017	-0.071	-0.043	-0.011
Nigeria	Minjibir	2000	0.564	0.881	1.319
Nigeria	Minjibir	2017	0.326	0.495	0.716
Nigeria	Minjibir	2000-2017	-0.062	-0.033	-0.003
Nigeria	Misau	2000	0.616	0.982	1.474
Nigeria	Misau	2017	0.383	0.583	0.831
Nigeria	Misau	2000-2017	-0.059	-0.029	0.002
Nigeria	Mkpat Enin	2000	0.444	0.746	1.101
Nigeria	Mkpat Enin	2017	0.181	0.287	0.426
Nigeria	Mkpat Enin	2000-2017	-0.088	-0.055	-0.023
Nigeria	Moba	2000	0.376	0.618	0.937
Nigeria	Moba	2017	0.142	0.231	0.333
Nigeria	Moba	2000-2017	-0.086	-0.058	-0.027
Nigeria	Mobbar	2000	0.656	1.060	1.579
Nigeria	Mobbar	2017	0.435	0.664	0.973
Nigeria	Mobbar	2000-2017	-0.051	-0.025	0.002
Nigeria	Mokwa	2000	0.473	0.761	1.134
Nigeria	Mokwa	2017	0.223	0.321	0.467
Nigeria	Mokwa	2000-2017	-0.074	-0.050	-0.022
Nigeria	Monguno	2000	0.668	1.052	1.550
Nigeria	Monguno	2017	0.419	0.669	0.982
Nigeria	Monguno	2000-2017	-0.052	-0.024	0.001
Nigeria	Mopa-Muro	2000	0.469	0.757	1.132
Nigeria	Mopa-Muro	2017	0.186	0.282	0.416
Nigeria	Mopa-Muro	2000-2017	-0.087	-0.060	-0.030
Nigeria	Moro	2000	0.458	0.776	1.113
Nigeria	Moro	2017	0.199	0.292	0.429
Nigeria	Moro	2000-2017	-0.078	-0.056	-0.032
Nigeria	Mubi North	2000	0.704	1.082	1.601
Nigeria	Mubi North	2017	0.387	0.582	0.909
Nigeria	Mubi North	2000-2017	-0.060	-0.033	-0.004
Nigeria	Mubi South	2000	0.719	1.115	1.671
Nigeria	Mubi South	2017	0.393	0.594	0.903
Nigeria	Mubi South	2000-2017	-0.063	-0.033	-0.004
Nigeria	Musawa	2000	0.528	0.831	1.175
Nigeria	Musawa	2017	0.301	0.452	0.647
Nigeria	Musawa	2000-2017	-0.064	-0.035	-0.007
Nigeria	Mushin	2000	0.298	0.526	0.837
Nigeria	Mushin	2017	0.132	0.229	0.352
Nigeria	Mushin	2000-2017	-0.087	-0.046	-0.007
Nigeria	Muya	2000	0.488	0.753	1.037
Nigeria	Muya	2017	0.244	0.357	0.511
Nigeria	Muya	2000-2017	-0.069	-0.042	-0.015
Nigeria	Nafada	2000	0.654	1.056	1.549
Nigeria	Nafada	2017	0.412	0.647	0.917
Nigeria	Nafada	2000-2017	-0.054	-0.028	0.000
Nigeria	Nangere	2000	0.590	1.010	1.481
Nigeria	Nangere	2017	0.389	0.626	0.900
Nigeria	Nangere	2000-2017	-0.059	-0.027	0.005
Nigeria	Nasarawa	2000	0.539	0.818	1.173
Nigeria	Nasarawa	2017	0.216	0.330	0.500
Nigeria	Nasarawa	2000-2017	-0.079	-0.052	-0.025
Nigeria	Nassaraw	2000	0.510	0.898	1.358
Nigeria	Nassaraw	2017	0.270	0.441	0.642
Nigeria	Nassaraw	2000-2017	-0.080	-0.040	0.004

Table 1: LRI DALYs rate by unit *(continued)*

Country	Unit	year	mean rate	lower rate	upper rate
Nigeria	Nassarawa Egon	2000	0.545	0.856	1.223
Nigeria	Nassarawa Egon	2017	0.246	0.376	0.543
Nigeria	Nassarawa Egon	2000-2017	-0.076	-0.047	-0.020
Nigeria	Ndokwa East	2000	0.410	0.645	0.944
Nigeria	Ndokwa East	2017	0.175	0.265	0.398
Nigeria	Ndokwa East	2000-2017	-0.080	-0.048	-0.019
Nigeria	Ndokwa West	2000	0.387	0.605	0.883
Nigeria	Ndokwa West	2017	0.168	0.250	0.375
Nigeria	Ndokwa West	2000-2017	-0.075	-0.048	-0.019
Nigeria	Nembe	2000	0.436	0.697	1.052
Nigeria	Nembe	2017	0.165	0.265	0.407
Nigeria	Nembe	2000-2017	-0.089	-0.059	-0.029
Nigeria	Ngala	2000	0.676	1.078	1.597
Nigeria	Ngala	2017	0.406	0.640	0.992
Nigeria	Ngala	2000-2017	-0.054	-0.028	0.002
Nigeria	Nganzai	2000	0.628	1.030	1.527
Nigeria	Nganzai	2017	0.428	0.661	0.964
Nigeria	Nganzai	2000-2017	-0.049	-0.023	0.000
Nigeria	Ngaski	2000	0.454	0.761	1.111
Nigeria	Ngaski	2017	0.228	0.349	0.512
Nigeria	Ngaski	2000-2017	-0.069	-0.045	-0.017
Nigeria	Ngor-Okp	2000	0.420	0.680	0.989
Nigeria	Ngor-Okp	2017	0.175	0.273	0.417
Nigeria	Ngor-Okp	2000-2017	-0.084	-0.053	-0.021
Nigeria	Nguru	2000	0.558	0.960	1.434
Nigeria	Nguru	2017	0.380	0.596	0.867
Nigeria	Nguru	2000-2017	-0.059	-0.027	0.006
Nigeria	Ningi	2000	0.593	0.916	1.359
Nigeria	Ningi	2017	0.320	0.508	0.734
Nigeria	Ningi	2000-2017	-0.062	-0.034	-0.004
Nigeria	Njaba	2000	0.374	0.580	0.864
Nigeria	Njaba	2017	0.164	0.273	0.412
Nigeria	Njaba	2000-2017	-0.074	-0.042	-0.009
Nigeria	Njikoka	2000	0.349	0.557	0.837
Nigeria	Njikoka	2017	0.155	0.264	0.404
Nigeria	Njikoka	2000-2017	-0.073	-0.041	-0.011
Nigeria	Nkanu East	2000	0.437	0.681	0.994
Nigeria	Nkanu East	2017	0.177	0.278	0.400
Nigeria	Nkanu East	2000-2017	-0.079	-0.052	-0.021
Nigeria	Nkanu West	2000	0.367	0.619	0.925
Nigeria	Nkanu West	2017	0.164	0.286	0.455
Nigeria	Nkanu West	2000-2017	-0.075	-0.043	-0.011
Nigeria	Nkwerre	2000	0.343	0.593	0.877
Nigeria	Nkwerre	2017	0.166	0.273	0.437
Nigeria	Nkwerre	2000-2017	-0.078	-0.042	0.000
Nigeria	NnewiNort	2000	0.377	0.577	0.851
Nigeria	NnewiNort	2017	0.170	0.274	0.450
Nigeria	NnewiNort	2000-2017	-0.077	-0.043	-0.004
Nigeria	NnewiSou	2000	0.368	0.595	0.881
Nigeria	NnewiSou	2017	0.164	0.282	0.432
Nigeria	NnewiSou	2000-2017	-0.071	-0.041	-0.010
Nigeria	Nsit Atai	2000	0.435	0.741	1.064
Nigeria	Nsit Atai	2017	0.177	0.288	0.436
Nigeria	Nsit Atai	2000-2017	-0.087	-0.057	-0.023
Nigeria	Nsit Ibom	2000	0.440	0.765	1.169
Nigeria	Nsit Ibom	2017	0.182	0.297	0.463
Nigeria	Nsit Ibom	2000-2017	-0.086	-0.056	-0.017
Nigeria	Nsit Ubium	2000	0.437	0.734	1.096
Nigeria	Nsit Ubium	2017	0.174	0.283	0.433
Nigeria	Nsit Ubium	2000-2017	-0.088	-0.055	-0.023
Nigeria	Nsukka	2000	0.367	0.613	0.944

Table 1: LRI DALYs rate by unit *(continued)*

Country	Unit	year	mean rate	lower rate	upper rate
Nigeria	Nsukka	2017	0.164	0.287	0.439
Nigeria	Nsukka	2000-2017	-0.073	-0.043	-0.015
Nigeria	Numan	2000	0.675	1.059	1.542
Nigeria	Numan	2017	0.375	0.546	0.796
Nigeria	Numan	2000-2017	-0.067	-0.036	-0.006
Nigeria	Nwangele	2000	0.385	0.622	0.902
Nigeria	Nwangele	2017	0.165	0.271	0.434
Nigeria	Nwangele	2000-2017	-0.076	-0.045	-0.005
Nigeria	Obafemi-Owode	2000	0.395	0.647	0.992
Nigeria	Obafemi-Owode	2017	0.162	0.248	0.372
Nigeria	Obafemi-Owode	2000-2017	-0.082	-0.056	-0.025
Nigeria	Obanliku	2000	0.508	0.794	1.139
Nigeria	Obanliku	2017	0.205	0.321	0.493
Nigeria	Obanliku	2000-2017	-0.083	-0.054	-0.028
Nigeria	Obi	2000	0.502	0.817	1.182
Nigeria	Obi	2000	0.576	0.874	1.272
Nigeria	Obi	2017	0.207	0.325	0.477
Nigeria	Obi	2017	0.245	0.380	0.543
Nigeria	Obi	2000-2017	-0.076	-0.048	-0.020
Nigeria	Obi	2000-2017	-0.085	-0.055	-0.026
Nigeria	Obio/Akp	2000	0.440	0.689	1.027
Nigeria	Obio/Akp	2017	0.168	0.266	0.402
Nigeria	Obio/Akp	2000-2017	-0.086	-0.055	-0.024
Nigeria	Obokun	2000	0.358	0.593	0.868
Nigeria	Obokun	2017	0.148	0.226	0.319
Nigeria	Obokun	2000-2017	-0.084	-0.057	-0.027
Nigeria	Oboma Ngwa	2000	0.406	0.678	1.008
Nigeria	Oboma Ngwa	2017	0.176	0.276	0.416
Nigeria	Oboma Ngwa	2000-2017	-0.081	-0.051	-0.019
Nigeria	Obot Akara	2000	0.441	0.713	1.068
Nigeria	Obot Akara	2017	0.177	0.279	0.421
Nigeria	Obot Akara	2000-2017	-0.085	-0.054	-0.020
Nigeria	Obowo	2000	0.390	0.621	0.944
Nigeria	Obowo	2017	0.171	0.263	0.412
Nigeria	Obowo	2000-2017	-0.078	-0.047	-0.013
Nigeria	Obubra	2000	0.478	0.794	1.164
Nigeria	Obubra	2017	0.198	0.306	0.452
Nigeria	Obubra	2000-2017	-0.083	-0.056	-0.027
Nigeria	Obudu	2000	0.492	0.777	1.146
Nigeria	Obudu	2017	0.206	0.318	0.489
Nigeria	Obudu	2000-2017	-0.082	-0.053	-0.024
Nigeria	Odeda	2000	0.434	0.697	1.006
Nigeria	Odeda	2017	0.152	0.246	0.367
Nigeria	Odeda	2000-2017	-0.087	-0.061	-0.031
Nigeria	Odigbo	2000	0.396	0.602	0.889
Nigeria	Odigbo	2017	0.146	0.221	0.325
Nigeria	Odigbo	2000-2017	-0.085	-0.060	-0.032
Nigeria	Odo0tin	2000	0.353	0.607	0.913
Nigeria	Odo0tin	2017	0.152	0.235	0.338
Nigeria	Odo0tin	2000-2017	-0.084	-0.057	-0.024
Nigeria	Odogbolu	2000	0.372	0.623	0.937
Nigeria	Odogbolu	2017	0.161	0.244	0.353
Nigeria	Odogbolu	2000-2017	-0.082	-0.055	-0.028
Nigeria	Odukpani	2000	0.481	0.759	1.110
Nigeria	Odukpani	2017	0.183	0.293	0.443
Nigeria	Odukpani	2000-2017	-0.085	-0.059	-0.028
Nigeria	Offa	2000	0.468	0.754	1.153
Nigeria	Offa	2017	0.184	0.295	0.449
Nigeria	Offa	2000-2017	-0.089	-0.055	-0.023
Nigeria	Ofu	2000	0.500	0.778	1.194
Nigeria	Ofu	2017	0.201	0.294	0.438

Table 1: LRI DALYs rate by unit *(continued)*

Country	Unit	year	mean rate	lower rate	upper rate
Nigeria	Ofu	2000-2017	-0.083	-0.058	-0.030
Nigeria	Ogba/Egbe	2000	0.400	0.649	0.959
Nigeria	Ogba/Egbe	2017	0.159	0.261	0.393
Nigeria	Ogba/Egbe	2000-2017	-0.079	-0.050	-0.019
Nigeria	Ogbadibo	2000	0.506	0.820	1.218
Nigeria	Ogbadibo	2017	0.200	0.321	0.473
Nigeria	Ogbadibo	2000-2017	-0.084	-0.056	-0.027
Nigeria	Ogbaru	2000	0.379	0.599	0.869
Nigeria	Ogbaru	2017	0.173	0.264	0.395
Nigeria	Ogbaru	2000-2017	-0.074	-0.045	-0.016
Nigeria	Ogbia	2000	0.453	0.723	1.094
Nigeria	Ogbia	2017	0.170	0.283	0.444
Nigeria	Ogbia	2000-2017	-0.085	-0.057	-0.027
Nigeria	Ogbomosho North	2000	0.328	0.549	0.847
Nigeria	Ogbomosho North	2017	0.139	0.234	0.340
Nigeria	Ogbomosho North	2000-2017	-0.081	-0.051	-0.019
Nigeria	Ogbomosho South	2000	0.306	0.546	0.850
Nigeria	Ogbomosho South	2017	0.136	0.222	0.331
Nigeria	Ogbomosho South	2000-2017	-0.093	-0.053	-0.014
Nigeria	Ogo-Oluw	2000	0.374	0.615	0.893
Nigeria	Ogo-Oluw	2017	0.145	0.225	0.338
Nigeria	Ogo-Oluw	2000-2017	-0.085	-0.061	-0.032
Nigeria	Ogoja	2000	0.482	0.754	1.100
Nigeria	Ogoja	2017	0.189	0.301	0.448
Nigeria	Ogoja	2000-2017	-0.084	-0.054	-0.026
Nigeria	Ogori/Magongo	2000	0.457	0.755	1.179
Nigeria	Ogori/Magongo	2017	0.186	0.283	0.440
Nigeria	Ogori/Magongo	2000-2017	-0.097	-0.059	-0.029
Nigeria	Ogu/Bolo	2000	0.434	0.710	1.081
Nigeria	Ogu/Bolo	2017	0.178	0.274	0.414
Nigeria	Ogu/Bolo	2000-2017	-0.087	-0.057	-0.020
Nigeria	OgunWaterside	2000	0.438	0.691	0.993
Nigeria	OgunWaterside	2017	0.156	0.249	0.361
Nigeria	OgunWaterside	2000-2017	-0.087	-0.060	-0.033
Nigeria	Oguta	2000	0.404	0.643	0.971
Nigeria	Oguta	2017	0.167	0.271	0.428
Nigeria	Oguta	2000-2017	-0.074	-0.047	-0.014
Nigeria	Ohafia Abia	2000	0.420	0.706	1.013
Nigeria	Ohafia Abia	2017	0.177	0.276	0.419
Nigeria	Ohafia Abia	2000-2017	-0.082	-0.054	-0.025
Nigeria	Ohaji/Eg	2000	0.403	0.643	0.948
Nigeria	Ohaji/Eg	2017	0.168	0.266	0.403
Nigeria	Ohaji/Eg	2000-2017	-0.075	-0.049	-0.019
Nigeria	Ohaozara	2000	0.504	0.827	1.218
Nigeria	Ohaozara	2017	0.209	0.324	0.472
Nigeria	Ohaozara	2000-2017	-0.083	-0.053	-0.023
Nigeria	Ohaukwu	2000	0.518	0.889	1.355
Nigeria	Ohaukwu	2017	0.205	0.344	0.533
Nigeria	Ohaukwu	2000-2017	-0.082	-0.055	-0.025
Nigeria	Ohimini	2000	0.511	0.811	1.200
Nigeria	Ohimini	2017	0.201	0.318	0.484
Nigeria	Ohimini	2000-2017	-0.086	-0.056	-0.029
Nigeria	Oji-River	2000	0.389	0.641	0.972
Nigeria	Oji-River	2017	0.172	0.279	0.425
Nigeria	Oji-River	2000-2017	-0.075	-0.046	-0.017
Nigeria	Ojo	2000	0.331	0.555	0.832
Nigeria	Ojo	2017	0.152	0.242	0.374
Nigeria	Ojo	2000-2017	-0.079	-0.047	-0.016
Nigeria	Oju	2000	0.516	0.831	1.183
Nigeria	Oju	2017	0.217	0.332	0.488
Nigeria	Oju	2000-2017	-0.081	-0.055	-0.027

Table 1: LRI DALYs rate by unit *(continued)*

Country	Unit	year	mean rate	lower rate	upper rate
Nigeria	Oke-Ero	2000	0.440	0.750	1.131
Nigeria	Oke-Ero	2017	0.176	0.273	0.401
Nigeria	Oke-Ero	2000-2017	-0.088	-0.061	-0.031
Nigeria	Okehi	2000	0.479	0.752	1.130
Nigeria	Okehi	2017	0.187	0.282	0.401
Nigeria	Okehi	2000-2017	-0.085	-0.059	-0.031
Nigeria	Okene	2000	0.482	0.752	1.137
Nigeria	Okene	2017	0.184	0.281	0.406
Nigeria	Okene	2000-2017	-0.089	-0.059	-0.031
Nigeria	Okigwe	2000	0.407	0.673	0.995
Nigeria	Okigwe	2017	0.168	0.267	0.391
Nigeria	Okigwe	2000-2017	-0.082	-0.053	-0.022
Nigeria	Okitipupa	2000	0.384	0.588	0.857
Nigeria	Okitipupa	2017	0.142	0.219	0.332
Nigeria	Okitipupa	2000-2017	-0.084	-0.059	-0.031
Nigeria	Okobo	2000	0.453	0.727	1.083
Nigeria	Okobo	2017	0.173	0.280	0.415
Nigeria	Okobo	2000-2017	-0.086	-0.057	-0.026
Nigeria	Okpe	2000	0.376	0.588	0.868
Nigeria	Okpe	2017	0.158	0.246	0.366
Nigeria	Okpe	2000-2017	-0.077	-0.049	-0.021
Nigeria	Okpokwu	2000	0.485	0.809	1.196
Nigeria	Okpokwu	2017	0.199	0.315	0.466
Nigeria	Okpokwu	2000-2017	-0.086	-0.057	-0.029
Nigeria	Okrika	2000	0.456	0.702	1.074
Nigeria	Okrika	2017	0.170	0.270	0.408
Nigeria	Okrika	2000-2017	-0.087	-0.058	-0.026
Nigeria	Ola-Oluwa	2000	0.365	0.617	0.912
Nigeria	Ola-Oluwa	2017	0.145	0.224	0.325
Nigeria	Ola-Oluwa	2000-2017	-0.087	-0.061	-0.034
Nigeria	Olamabor	2000	0.478	0.796	1.185
Nigeria	Olamabor	2017	0.198	0.307	0.451
Nigeria	Olamabor	2000-2017	-0.083	-0.057	-0.027
Nigeria	Olorunda	2000	0.331	0.541	0.802
Nigeria	Olorunda	2017	0.140	0.225	0.364
Nigeria	Olorunda	2000-2017	-0.089	-0.053	-0.018
Nigeria	Olorunsogo	2000	0.390	0.636	0.998
Nigeria	Olorunsogo	2017	0.158	0.240	0.361
Nigeria	Olorunsogo	2000-2017	-0.082	-0.058	-0.030
Nigeria	Oluyole	2000	0.374	0.576	0.857
Nigeria	Oluyole	2017	0.146	0.222	0.324
Nigeria	Oluyole	2000-2017	-0.086	-0.055	-0.030
Nigeria	Omala	2000	0.494	0.790	1.214
Nigeria	Omala	2017	0.203	0.309	0.455
Nigeria	Omala	2000-2017	-0.080	-0.055	-0.028
Nigeria	Omumma	2000	0.431	0.686	0.973
Nigeria	Omumma	2017	0.169	0.265	0.402
Nigeria	Omumma	2000-2017	-0.085	-0.058	-0.028
Nigeria	Ona-Ara	2000	0.357	0.558	0.849
Nigeria	Ona-Ara	2017	0.139	0.221	0.338
Nigeria	Ona-Ara	2000-2017	-0.086	-0.056	-0.025
Nigeria	Ondo East	2000	0.397	0.612	0.922
Nigeria	Ondo East	2017	0.151	0.225	0.330
Nigeria	Ondo East	2000-2017	-0.086	-0.060	-0.033
Nigeria	Ondo West	2000	0.392	0.588	0.884
Nigeria	Ondo West	2017	0.146	0.223	0.323
Nigeria	Ondo West	2000-2017	-0.087	-0.058	-0.025
Nigeria	Onicha	2000	0.502	0.834	1.221
Nigeria	Onicha	2017	0.207	0.319	0.484
Nigeria	Onicha	2000-2017	-0.084	-0.055	-0.023
Nigeria	Onitsha North	2000	0.321	0.538	0.896

Table 1: LRI DALYs rate by unit *(continued)*

Country	Unit	year	mean rate	lower rate	upper rate
Nigeria	Onitsha North	2017	0.145	0.250	0.400
Nigeria	Onitsha North	2000-2017	-0.089	-0.043	-0.001
Nigeria	Onitsha South	2000	0.331	0.550	0.847
Nigeria	Onitsha South	2017	0.153	0.250	0.385
Nigeria	Onitsha South	2000-2017	-0.087	-0.043	-0.006
Nigeria	Onna	2000	0.449	0.717	1.053
Nigeria	Onna	2017	0.174	0.275	0.413
Nigeria	Onna	2000-2017	-0.087	-0.054	-0.021
Nigeria	Opobo/Nkoro	2000	0.446	0.713	1.054
Nigeria	Opobo/Nkoro	2017	0.175	0.273	0.421
Nigeria	Opobo/Nkoro	2000-2017	-0.089	-0.056	-0.022
Nigeria	Oredo Edo	2000	0.334	0.539	0.835
Nigeria	Oredo Edo	2017	0.157	0.231	0.344
Nigeria	Oredo Edo	2000-2017	-0.079	-0.049	-0.016
Nigeria	Orelope	2000	0.375	0.606	0.904
Nigeria	Orelope	2017	0.150	0.230	0.345
Nigeria	Orelope	2000-2017	-0.083	-0.057	-0.031
Nigeria	Orhionmw	2000	0.388	0.612	0.927
Nigeria	Orhionmw	2017	0.163	0.243	0.361
Nigeria	Orhionmw	2000-2017	-0.077	-0.053	-0.025
Nigeria	Ori-Ire	2000	0.369	0.634	0.911
Nigeria	Ori-Ire	2017	0.158	0.238	0.342
Nigeria	Ori-Ire	2000-2017	-0.082	-0.059	-0.032
Nigeria	Oriade	2000	0.377	0.606	0.909
Nigeria	Oriade	2017	0.143	0.226	0.324
Nigeria	Oriade	2000-2017	-0.084	-0.058	-0.032
Nigeria	Orlu	2000	0.357	0.605	0.887
Nigeria	Orlu	2017	0.164	0.272	0.424
Nigeria	Orlu	2000-2017	-0.075	-0.045	-0.010
Nigeria	Orolu	2000	0.316	0.557	0.862
Nigeria	Orolu	2017	0.134	0.229	0.378
Nigeria	Orolu	2000-2017	-0.088	-0.052	-0.017
Nigeria	Oron	2000	0.438	0.714	1.085
Nigeria	Oron	2017	0.165	0.277	0.447
Nigeria	Oron	2000-2017	-0.099	-0.056	-0.023
Nigeria	Orsu	2000	0.376	0.595	0.884
Nigeria	Orsu	2017	0.166	0.273	0.416
Nigeria	Orsu	2000-2017	-0.074	-0.042	-0.011
Nigeria	Oru East	2000	0.371	0.595	0.871
Nigeria	Oru East	2017	0.168	0.272	0.421
Nigeria	Oru East	2000-2017	-0.073	-0.043	-0.009
Nigeria	Oru West	2000	0.378	0.614	0.916
Nigeria	Oru West	2017	0.178	0.275	0.452
Nigeria	Oru West	2000-2017	-0.077	-0.044	-0.009
Nigeria	Oruk-Ana	2000	0.458	0.740	1.096
Nigeria	Oruk-Ana	2017	0.178	0.285	0.434
Nigeria	Oruk-Ana	2000-2017	-0.085	-0.054	-0.022
Nigeria	OrumbaNo	2000	0.381	0.613	0.937
Nigeria	OrumbaNo	2017	0.167	0.281	0.438
Nigeria	OrumbaNo	2000-2017	-0.074	-0.043	-0.012
Nigeria	OrumbaSo	2000	0.419	0.652	0.965
Nigeria	OrumbaSo	2017	0.175	0.280	0.434
Nigeria	OrumbaSo	2000-2017	-0.076	-0.047	-0.015
Nigeria	Ose	2000	0.386	0.620	0.945
Nigeria	Ose	2017	0.153	0.229	0.331
Nigeria	Ose	2000-2017	-0.083	-0.059	-0.031
Nigeria	Oshimili North	2000	0.381	0.583	0.908
Nigeria	Oshimili North	2017	0.160	0.248	0.367
Nigeria	Oshimili North	2000-2017	-0.073	-0.047	-0.018
Nigeria	Oshimili South	2000	0.345	0.551	0.851
Nigeria	Oshimili South	2017	0.154	0.247	0.366

Table 1: LRI DALYs rate by unit *(continued)*

Country	Unit	year	mean rate	lower rate	upper rate
Nigeria	Oshimili South	2000-2017	-0.072	-0.044	-0.015
Nigeria	Oshodi/Isolo	2000	0.314	0.525	0.843
Nigeria	Oshodi/Isolo	2017	0.137	0.226	0.356
Nigeria	Oshodi/Isolo	2000-2017	-0.084	-0.047	-0.012
Nigeria	Osisioma Ngwa	2000	0.405	0.660	0.976
Nigeria	Osisioma Ngwa	2017	0.168	0.269	0.402
Nigeria	Osisioma Ngwa	2000-2017	-0.085	-0.051	-0.016
Nigeria	Osogbo	2000	0.336	0.555	0.834
Nigeria	Osogbo	2017	0.141	0.222	0.340
Nigeria	Osogbo	2000-2017	-0.087	-0.054	-0.017
Nigeria	Oturkpo	2000	0.508	0.806	1.178
Nigeria	Oturkpo	2017	0.213	0.326	0.486
Nigeria	Oturkpo	2000-2017	-0.081	-0.054	-0.024
Nigeria	OviaNort	2000	0.377	0.600	0.897
Nigeria	OviaNort	2017	0.155	0.231	0.339
Nigeria	OviaNort	2000-2017	-0.083	-0.057	-0.027
Nigeria	OviaSouth-West	2000	0.393	0.623	0.928
Nigeria	OviaSouth-West	2017	0.152	0.230	0.339
Nigeria	OviaSouth-West	2000-2017	-0.086	-0.060	-0.031
Nigeria	Owan East	2000	0.392	0.624	0.944
Nigeria	Owan East	2017	0.152	0.234	0.332
Nigeria	Owan East	2000-2017	-0.083	-0.058	-0.031
Nigeria	OwanWest	2000	0.401	0.617	0.944
Nigeria	OwanWest	2017	0.150	0.229	0.327
Nigeria	OwanWest	2000-2017	-0.085	-0.059	-0.030
Nigeria	Owerri Municipal	2000	0.350	0.569	0.902
Nigeria	Owerri Municipal	2017	0.163	0.268	0.432
Nigeria	Owerri Municipal	2000-2017	-0.082	-0.042	-0.002
Nigeria	Owerri North	2000	0.386	0.608	0.898
Nigeria	Owerri North	2017	0.166	0.265	0.399
Nigeria	Owerri North	2000-2017	-0.077	-0.046	-0.014
Nigeria	Owerri West	2000	0.372	0.621	0.917
Nigeria	Owerri West	2017	0.167	0.274	0.418
Nigeria	Owerri West	2000-2017	-0.074	-0.045	-0.009
Nigeria	Owo	2000	0.382	0.600	0.918
Nigeria	Owo	2017	0.150	0.227	0.328
Nigeria	Owo	2000-2017	-0.085	-0.058	-0.034
Nigeria	Oye	2000	0.369	0.619	0.921
Nigeria	Oye	2017	0.150	0.231	0.331
Nigeria	Oye	2000-2017	-0.084	-0.058	-0.031
Nigeria	Oyi	2000	0.344	0.542	0.821
Nigeria	Oyi	2017	0.157	0.257	0.391
Nigeria	Oyi	2000-2017	-0.074	-0.042	-0.009
Nigeria	Oyigbo	2000	0.452	0.695	1.008
Nigeria	Oyigbo	2017	0.173	0.267	0.396
Nigeria	Oyigbo	2000-2017	-0.089	-0.056	-0.021
Nigeria	Oyo East	2000	0.339	0.561	0.865
Nigeria	Oyo East	2017	0.138	0.223	0.338
Nigeria	Oyo East	2000-2017	-0.085	-0.055	-0.024
Nigeria	Oyo West	2000	0.335	0.557	0.835
Nigeria	Oyo West	2017	0.142	0.225	0.352
Nigeria	Oyo West	2000-2017	-0.082	-0.055	-0.027
Nigeria	Oyun	2000	0.470	0.758	1.095
Nigeria	Oyun	2017	0.183	0.283	0.407
Nigeria	Oyun	2000-2017	-0.083	-0.058	-0.032
Nigeria	Paikoro	2000	0.493	0.770	1.102
Nigeria	Paikoro	2017	0.231	0.339	0.495
Nigeria	Paikoro	2000-2017	-0.075	-0.047	-0.020
Nigeria	Pankshin	2000	0.609	0.897	1.302
Nigeria	Pankshin	2017	0.262	0.413	0.599
Nigeria	Pankshin	2000-2017	-0.072	-0.044	-0.017

Table 1: LRI DALYs rate by unit *(continued)*

Country	Unit	year	mean rate	lower rate	upper rate
Nigeria	Patani	2000	0.438	0.706	1.024
Nigeria	Patani	2017	0.169	0.278	0.423
Nigeria	Patani	2000-2017	-0.083	-0.054	-0.022
Nigeria	Pategi	2000	0.471	0.762	1.122
Nigeria	Pategi	2017	0.197	0.293	0.403
Nigeria	Pategi	2000-2017	-0.081	-0.056	-0.030
Nigeria	Port Harcourt	2000	0.448	0.683	0.978
Nigeria	Port Harcourt	2017	0.177	0.278	0.434
Nigeria	Port Harcourt	2000-2017	-0.084	-0.050	-0.018
Nigeria	Potiskum	2000	0.621	1.019	1.591
Nigeria	Potiskum	2017	0.404	0.626	0.892
Nigeria	Potiskum	2000-2017	-0.061	-0.027	0.007
Nigeria	Qua'anpa	2000	0.585	0.882	1.289
Nigeria	Qua'anpa	2017	0.262	0.404	0.574
Nigeria	Qua'anpa	2000-2017	-0.074	-0.044	-0.017
Nigeria	Rabah	2000	0.521	0.834	1.269
Nigeria	Rabah	2017	0.286	0.434	0.658
Nigeria	Rabah	2000-2017	-0.065	-0.039	-0.012
Nigeria	Rafi	2000	0.491	0.794	1.169
Nigeria	Rafi	2017	0.251	0.369	0.537
Nigeria	Rafi	2000-2017	-0.068	-0.044	-0.016
Nigeria	Rano	2000	0.558	0.874	1.291
Nigeria	Rano	2017	0.301	0.479	0.711
Nigeria	Rano	2000-2017	-0.069	-0.035	-0.007
Nigeria	Remo-North	2000	0.409	0.661	0.990
Nigeria	Remo-North	2017	0.165	0.247	0.380
Nigeria	Remo-North	2000-2017	-0.089	-0.059	-0.021
Nigeria	Rijau	2000	0.485	0.809	1.224
Nigeria	Rijau	2017	0.257	0.386	0.578
Nigeria	Rijau	2000-2017	-0.066	-0.042	-0.015
Nigeria	Rimi	2000	0.537	0.862	1.292
Nigeria	Rimi	2017	0.302	0.476	0.709
Nigeria	Rimi	2000-2017	-0.062	-0.035	-0.002
Nigeria	RiminGad	2000	0.545	0.846	1.228
Nigeria	RiminGad	2017	0.295	0.473	0.678
Nigeria	RiminGad	2000-2017	-0.064	-0.033	-0.005
Nigeria	Ringim	2000	0.567	0.910	1.366
Nigeria	Ringim	2017	0.328	0.518	0.749
Nigeria	Ringim	2000-2017	-0.062	-0.032	-0.002
Nigeria	Riyom	2000	0.558	0.849	1.246
Nigeria	Riyom	2017	0.252	0.391	0.560
Nigeria	Riyom	2000-2017	-0.075	-0.044	-0.014
Nigeria	Rogo	2000	0.530	0.828	1.206
Nigeria	Rogo	2017	0.293	0.444	0.636
Nigeria	Rogo	2000-2017	-0.065	-0.036	-0.007
Nigeria	Roni	2000	0.561	0.873	1.358
Nigeria	Roni	2017	0.319	0.491	0.697
Nigeria	Roni	2000-2017	-0.064	-0.033	-0.003
Nigeria	Sabon Birni	2000	0.502	0.861	1.263
Nigeria	Sabon Birni	2017	0.302	0.460	0.684
Nigeria	Sabon Birni	2000-2017	-0.064	-0.037	-0.006
Nigeria	Sabon-Ga	2000	0.512	0.835	1.214
Nigeria	Sabon-Ga	2017	0.282	0.435	0.662
Nigeria	Sabon-Ga	2000-2017	-0.070	-0.036	-0.002
Nigeria	Sabuwa	2000	0.520	0.825	1.223
Nigeria	Sabuwa	2017	0.282	0.424	0.619
Nigeria	Sabuwa	2000-2017	-0.066	-0.038	-0.006
Nigeria	Safana	2000	0.513	0.832	1.234
Nigeria	Safana	2017	0.294	0.451	0.651
Nigeria	Safana	2000-2017	-0.063	-0.035	-0.007
Nigeria	Sagbama	2000	0.445	0.715	1.029

Table 1: LRI DALYs rate by unit *(continued)*

Country	Unit	year	mean rate	lower rate	upper rate
Nigeria	Sagbama	2017	0.176	0.280	0.433
Nigeria	Sagbama	2000-2017	-0.082	-0.054	-0.026
Nigeria	Sakaba	2000	0.466	0.783	1.177
Nigeria	Sakaba	2017	0.251	0.385	0.580
Nigeria	Sakaba	2000-2017	-0.066	-0.041	-0.013
Nigeria	Saki East	2000	0.383	0.620	0.917
Nigeria	Saki East	2017	0.145	0.234	0.355
Nigeria	Saki East	2000-2017	-0.083	-0.057	-0.032
Nigeria	Saki West	2000	0.386	0.628	0.930
Nigeria	Saki West	2017	0.145	0.232	0.344
Nigeria	Saki West	2000-2017	-0.085	-0.059	-0.034
Nigeria	Sandamu	2000	0.524	0.888	1.331
Nigeria	Sandamu	2017	0.321	0.507	0.764
Nigeria	Sandamu	2000-2017	-0.061	-0.032	0.004
Nigeria	Sanga	2000	0.546	0.850	1.219
Nigeria	Sanga	2017	0.258	0.396	0.579
Nigeria	Sanga	2000-2017	-0.072	-0.043	-0.014
Nigeria	Sapele	2000	0.400	0.610	0.897
Nigeria	Sapele	2017	0.161	0.248	0.378
Nigeria	Sapele	2000-2017	-0.083	-0.053	-0.023
Nigeria	Sardauna	2000	0.618	0.977	1.412
Nigeria	Sardauna	2017	0.296	0.466	0.721
Nigeria	Sardauna	2000-2017	-0.068	-0.041	-0.015
Nigeria	Shagamu	2000	0.358	0.584	0.870
Nigeria	Shagamu	2017	0.158	0.245	0.354
Nigeria	Shagamu	2000-2017	-0.076	-0.051	-0.023
Nigeria	Shagari	2000	0.503	0.828	1.232
Nigeria	Shagari	2017	0.266	0.418	0.636
Nigeria	Shagari	2000-2017	-0.064	-0.040	-0.013
Nigeria	Shanga	2000	0.473	0.794	1.164
Nigeria	Shanga	2017	0.250	0.381	0.561
Nigeria	Shanga	2000-2017	-0.067	-0.043	-0.017
Nigeria	Shani	2000	0.659	1.068	1.607
Nigeria	Shani	2017	0.428	0.628	0.910
Nigeria	Shani	2000-2017	-0.057	-0.029	-0.001
Nigeria	Shanono	2000	0.535	0.841	1.221
Nigeria	Shanono	2017	0.316	0.464	0.672
Nigeria	Shanono	2000-2017	-0.063	-0.034	-0.005
Nigeria	Shelleng	2000	0.698	1.080	1.581
Nigeria	Shelleng	2017	0.387	0.579	0.833
Nigeria	Shelleng	2000-2017	-0.061	-0.033	-0.004
Nigeria	Shendam	2000	0.604	0.913	1.342
Nigeria	Shendam	2017	0.277	0.425	0.620
Nigeria	Shendam	2000-2017	-0.075	-0.044	-0.016
Nigeria	Shinkafi	2000	0.498	0.837	1.275
Nigeria	Shinkafi	2017	0.297	0.446	0.647
Nigeria	Shinkafi	2000-2017	-0.063	-0.037	-0.006
Nigeria	Shira	2000	0.587	0.957	1.397
Nigeria	Shira	2017	0.354	0.554	0.788
Nigeria	Shira	2000-2017	-0.062	-0.031	0.002
Nigeria	Shiroro	2000	0.508	0.794	1.126
Nigeria	Shiroro	2017	0.255	0.371	0.528
Nigeria	Shiroro	2000-2017	-0.070	-0.044	-0.017
Nigeria	Shomgom	2000	0.652	1.040	1.531
Nigeria	Shomgom	2017	0.370	0.575	0.831
Nigeria	Shomgom	2000-2017	-0.062	-0.033	-0.003
Nigeria	Shomolu	2000	0.302	0.521	0.798
Nigeria	Shomolu	2017	0.134	0.227	0.354
Nigeria	Shomolu	2000-2017	-0.084	-0.046	-0.008
Nigeria	Silame	2000	0.492	0.847	1.266
Nigeria	Silame	2017	0.268	0.427	0.663

Table 1: LRI DALYs rate by unit *(continued)*

Country	Unit	year	mean rate	lower rate	upper rate
Nigeria	Silame	2000-2017	-0.066	-0.040	-0.011
Nigeria	Soba	2000	0.550	0.832	1.196
Nigeria	Soba	2017	0.293	0.451	0.660
Nigeria	Soba	2000-2017	-0.063	-0.035	-0.007
Nigeria	Sokoto North	2000	0.489	0.858	1.453
Nigeria	Sokoto North	2017	0.244	0.420	0.646
Nigeria	Sokoto North	2000-2017	-0.077	-0.040	0.000
Nigeria	Sokoto South	2000	0.483	0.857	1.345
Nigeria	Sokoto South	2017	0.241	0.430	0.772
Nigeria	Sokoto South	2000-2017	-0.085	-0.040	0.007
Nigeria	Song	2000	0.692	1.098	1.641
Nigeria	Song	2017	0.400	0.586	0.863
Nigeria	Song	2000-2017	-0.060	-0.033	-0.006
Nigeria	Southern Ijaw	2000	0.436	0.715	1.052
Nigeria	Southern Ijaw	2017	0.173	0.281	0.441
Nigeria	Southern Ijaw	2000-2017	-0.082	-0.054	-0.027
Nigeria	Sule-Tan	2000	0.594	0.939	1.387
Nigeria	Sule-Tan	2017	0.337	0.531	0.781
Nigeria	Sule-Tan	2000-2017	-0.063	-0.032	-0.004
Nigeria	Suleja	2000	0.498	0.799	1.216
Nigeria	Suleja	2017	0.212	0.335	0.504
Nigeria	Suleja	2000-2017	-0.082	-0.051	-0.016
Nigeria	Sumaila	2000	0.576	0.890	1.288
Nigeria	Sumaila	2017	0.314	0.492	0.734
Nigeria	Sumaila	2000-2017	-0.065	-0.034	-0.001
Nigeria	Suru	2000	0.489	0.801	1.217
Nigeria	Suru	2017	0.241	0.384	0.591
Nigeria	Suru	2000-2017	-0.068	-0.043	-0.015
Nigeria	Surulere	2000	0.374	0.627	0.900
Nigeria	Surulere	2000	0.293	0.526	0.785
Nigeria	Surulere	2017	0.152	0.233	0.337
Nigeria	Surulere	2017	0.139	0.230	0.370
Nigeria	Surulere	2000-2017	-0.082	-0.047	-0.015
Nigeria	Surulere	2000-2017	-0.084	-0.060	-0.034
Nigeria	Tafa	2000	0.490	0.804	1.226
Nigeria	Tafa	2017	0.220	0.337	0.501
Nigeria	Tafa	2000-2017	-0.083	-0.051	-0.016
Nigeria	Tafawa-B	2000	0.588	0.905	1.357
Nigeria	Tafawa-B	2017	0.309	0.498	0.716
Nigeria	Tafawa-B	2000-2017	-0.064	-0.035	-0.006
Nigeria	Tai	2000	0.440	0.700	1.065
Nigeria	Tai	2017	0.169	0.266	0.423
Nigeria	Tai	2000-2017	-0.089	-0.057	-0.023
Nigeria	Takai	2000	0.580	0.911	1.308
Nigeria	Takai	2017	0.317	0.512	0.755
Nigeria	Takai	2000-2017	-0.062	-0.033	-0.001
Nigeria	Takum	2000	0.618	0.919	1.315
Nigeria	Takum	2017	0.251	0.396	0.590
Nigeria	Takum	2000-2017	-0.077	-0.048	-0.018
Nigeria	Talata-Mafara	2000	0.519	0.835	1.258
Nigeria	Talata-Mafara	2017	0.274	0.424	0.643
Nigeria	Talata-Mafara	2000-2017	-0.068	-0.040	-0.012
Nigeria	Tambawal	2000	0.488	0.824	1.208
Nigeria	Tambawal	2017	0.267	0.407	0.629
Nigeria	Tambawal	2000-2017	-0.067	-0.041	-0.012
Nigeria	Tangazar	2000	0.513	0.865	1.309
Nigeria	Tangazar	2017	0.270	0.447	0.678
Nigeria	Tangazar	2000-2017	-0.064	-0.039	-0.011
Nigeria	Tarauni	2000	0.519	0.903	1.427
Nigeria	Tarauni	2017	0.261	0.455	0.706
Nigeria	Tarauni	2000-2017	-0.081	-0.039	0.004

Table 1: LRI DALYs rate by unit *(continued)*

Country	Unit	year	mean rate	lower rate	upper rate
Nigeria	Tarka	2000	0.535	0.846	1.259
Nigeria	Tarka	2017	0.222	0.350	0.528
Nigeria	Tarka	2000-2017	-0.083	-0.052	-0.020
Nigeria	Tarmuwa	2000	0.667	1.057	1.640
Nigeria	Tarmuwa	2017	0.427	0.669	0.946
Nigeria	Tarmuwa	2000-2017	-0.052	-0.025	0.003
Nigeria	Taura	2000	0.583	0.919	1.369
Nigeria	Taura	2017	0.336	0.531	0.772
Nigeria	Taura	2000-2017	-0.060	-0.031	0.001
Nigeria	Teungo	2000	0.630	1.022	1.475
Nigeria	Teungo	2017	0.334	0.502	0.737
Nigeria	Teungo	2000-2017	-0.069	-0.038	-0.010
Nigeria	Tofa	2000	0.566	0.858	1.266
Nigeria	Tofa	2017	0.303	0.480	0.686
Nigeria	Tofa	2000-2017	-0.066	-0.033	-0.005
Nigeria	Toro	2000	0.579	0.894	1.313
Nigeria	Toro	2017	0.317	0.493	0.696
Nigeria	Toro	2000-2017	-0.064	-0.034	-0.005
Nigeria	Toto	2000	0.516	0.786	1.143
Nigeria	Toto	2017	0.204	0.308	0.450
Nigeria	Toto	2000-2017	-0.080	-0.054	-0.027
Nigeria	Tsafe	2000	0.513	0.817	1.241
Nigeria	Tsafe	2017	0.278	0.424	0.621
Nigeria	Tsafe	2000-2017	-0.065	-0.039	-0.010
Nigeria	Tsanyawa	2000	0.526	0.847	1.256
Nigeria	Tsanyawa	2017	0.312	0.471	0.681
Nigeria	Tsanyawa	2000-2017	-0.064	-0.034	-0.003
Nigeria	Tundun Wada	2000	0.554	0.859	1.263
Nigeria	Tundun Wada	2017	0.305	0.472	0.684
Nigeria	Tundun Wada	2000-2017	-0.063	-0.034	-0.003
Nigeria	Tureta	2000	0.499	0.826	1.222
Nigeria	Tureta	2017	0.279	0.419	0.668
Nigeria	Tureta	2000-2017	-0.067	-0.040	-0.011
Nigeria	Udenu	2000	0.392	0.635	0.965
Nigeria	Udenu	2017	0.174	0.291	0.467
Nigeria	Udenu	2000-2017	-0.072	-0.043	-0.015
Nigeria	Udi	2000	0.379	0.615	0.949
Nigeria	Udi	2017	0.164	0.288	0.437
Nigeria	Udi	2000-2017	-0.072	-0.043	-0.015
Nigeria	Udu	2000	0.372	0.575	0.831
Nigeria	Udu	2017	0.161	0.245	0.374
Nigeria	Udu	2000-2017	-0.077	-0.047	-0.012
Nigeria	Udung Uko	2000	0.423	0.724	1.092
Nigeria	Udung Uko	2017	0.169	0.279	0.414
Nigeria	Udung Uko	2000-2017	-0.094	-0.055	-0.017
Nigeria	Ughelli North	2000	0.389	0.626	0.896
Nigeria	Ughelli North	2017	0.163	0.248	0.367
Nigeria	Ughelli North	2000-2017	-0.078	-0.051	-0.018
Nigeria	Ughelli South	2000	0.419	0.660	0.951
Nigeria	Ughelli South	2017	0.164	0.257	0.390
Nigeria	Ughelli South	2000-2017	-0.080	-0.054	-0.022
Nigeria	Ugwunagbo	2000	0.458	0.713	1.109
Nigeria	Ugwunagbo	2017	0.170	0.278	0.416
Nigeria	Ugwunagbo	2000-2017	-0.092	-0.058	-0.026
Nigeria	Uhunmwonde	2000	0.377	0.591	0.894
Nigeria	Uhunmwonde	2017	0.158	0.236	0.346
Nigeria	Uhunmwonde	2000-2017	-0.079	-0.054	-0.026
Nigeria	Ukanafun	2000	0.448	0.724	1.089
Nigeria	Ukanafun	2017	0.173	0.279	0.417
Nigeria	Ukanafun	2000-2017	-0.083	-0.054	-0.023
Nigeria	Ukum	2000	0.599	0.908	1.317

Table 1: LRI DALYs rate by unit *(continued)*

Country	Unit	year	mean rate	lower rate	upper rate
Nigeria	Ukum	2017	0.249	0.386	0.584
Nigeria	Ukum	2000-2017	-0.079	-0.049	-0.022
Nigeria	Ukwa East	2000	0.443	0.709	1.060
Nigeria	Ukwa East	2017	0.163	0.272	0.406
Nigeria	Ukwa East	2000-2017	-0.084	-0.056	-0.025
Nigeria	Ukwa West	2000	0.457	0.707	1.035
Nigeria	Ukwa West	2017	0.170	0.269	0.406
Nigeria	Ukwa West	2000-2017	-0.086	-0.058	-0.026
Nigeria	Ukwuani	2000	0.384	0.594	0.897
Nigeria	Ukwuani	2017	0.159	0.250	0.382
Nigeria	Ukwuani	2000-2017	-0.074	-0.047	-0.018
Nigeria	Umu-Nneochi	2000	0.401	0.682	1.031
Nigeria	Umu-Nneochi	2017	0.174	0.274	0.413
Nigeria	Umu-Nneochi	2000-2017	-0.080	-0.052	-0.022
Nigeria	Umuahia North	2000	0.403	0.640	0.941
Nigeria	Umuahia North	2017	0.168	0.264	0.383
Nigeria	Umuahia North	2000-2017	-0.079	-0.049	-0.018
Nigeria	Umuahia South	2000	0.415	0.686	1.018
Nigeria	Umuahia South	2017	0.167	0.270	0.414
Nigeria	Umuahia South	2000-2017	-0.088	-0.054	-0.019
Nigeria	Ungogo	2000	0.541	0.880	1.315
Nigeria	Ungogo	2017	0.307	0.462	0.684
Nigeria	Ungogo	2000-2017	-0.071	-0.036	-0.001
Nigeria	Unuimo	2000	0.409	0.663	0.986
Nigeria	Unuimo	2017	0.168	0.261	0.406
Nigeria	Unuimo	2000-2017	-0.087	-0.050	-0.015
Nigeria	Uruan	2000	0.452	0.745	1.100
Nigeria	Uruan	2017	0.176	0.289	0.423
Nigeria	Uruan	2000-2017	-0.087	-0.057	-0.025
Nigeria	UrueOffo	2000	0.458	0.733	1.102
Nigeria	UrueOffo	2017	0.161	0.280	0.422
Nigeria	UrueOffo	2000-2017	-0.089	-0.057	-0.021
Nigeria	Ushongo	2000	0.568	0.874	1.257
Nigeria	Ushongo	2017	0.228	0.361	0.536
Nigeria	Ushongo	2000-2017	-0.082	-0.053	-0.027
Nigeria	Ussa	2000	0.597	0.903	1.281
Nigeria	Ussa	2017	0.252	0.384	0.583
Nigeria	Ussa	2000-2017	-0.077	-0.048	-0.020
Nigeria	Uvwie	2000	0.328	0.553	0.853
Nigeria	Uvwie	2017	0.161	0.252	0.398
Nigeria	Uvwie	2000-2017	-0.077	-0.042	-0.007
Nigeria	Uyo	2000	0.438	0.719	1.034
Nigeria	Uyo	2017	0.179	0.290	0.454
Nigeria	Uyo	2000-2017	-0.091	-0.055	-0.020
Nigeria	Uzo-Uwani	2000	0.378	0.608	0.931
Nigeria	Uzo-Uwani	2017	0.165	0.264	0.386
Nigeria	Uzo-Uwani	2000-2017	-0.071	-0.046	-0.019
Nigeria	Vandeiky	2000	0.574	0.873	1.263
Nigeria	Vandeiky	2017	0.235	0.360	0.535
Nigeria	Vandeiky	2000-2017	-0.082	-0.053	-0.025
Nigeria	Wamakko	2000	0.541	0.863	1.318
Nigeria	Wamakko	2017	0.272	0.416	0.667
Nigeria	Wamakko	2000-2017	-0.070	-0.041	-0.008
Nigeria	Wamba	2000	0.585	0.860	1.243
Nigeria	Wamba	2017	0.262	0.404	0.585
Nigeria	Wamba	2000-2017	-0.069	-0.043	-0.017
Nigeria	Warawa	2000	0.569	0.885	1.386
Nigeria	Warawa	2017	0.313	0.485	0.710
Nigeria	Warawa	2000-2017	-0.066	-0.034	-0.003
Nigeria	Warji	2000	0.611	0.938	1.395
Nigeria	Warji	2017	0.341	0.532	0.786

Table 1: LRI DALYs rate by unit *(continued)*

Country	Unit	year	mean rate	lower rate	upper rate
Nigeria	Warji	2000-2017	-0.063	-0.032	-0.001
Nigeria	Warri North	2000	0.400	0.630	0.932
Nigeria	Warri North	2017	0.164	0.248	0.371
Nigeria	Warri North	2000-2017	-0.077	-0.054	-0.025
Nigeria	Warri South	2000	0.361	0.570	0.880
Nigeria	Warri South	2017	0.161	0.251	0.376
Nigeria	Warri South	2000-2017	-0.074	-0.046	-0.012
Nigeria	Warri South-West	2000	0.403	0.615	0.893
Nigeria	Warri South-West	2017	0.157	0.248	0.377
Nigeria	Warri South-West	2000-2017	-0.079	-0.053	-0.025
Nigeria	Wase	2000	0.605	0.955	1.401
Nigeria	Wase	2017	0.304	0.489	0.701
Nigeria	Wase	2000-2017	-0.068	-0.038	-0.009
Nigeria	Wudil	2000	0.575	0.888	1.302
Nigeria	Wudil	2017	0.331	0.501	0.733
Nigeria	Wudil	2000-2017	-0.066	-0.033	-0.003
Nigeria	Wukari	2000	0.611	0.920	1.343
Nigeria	Wukari	2017	0.262	0.418	0.616
Nigeria	Wukari	2000-2017	-0.075	-0.045	-0.016
Nigeria	Wurno	2000	0.503	0.844	1.282
Nigeria	Wurno	2017	0.291	0.440	0.647
Nigeria	Wurno	2000-2017	-0.067	-0.038	-0.008
Nigeria	Wushishi	2000	0.472	0.777	1.102
Nigeria	Wushishi	2017	0.244	0.347	0.492
Nigeria	Wushishi	2000-2017	-0.070	-0.047	-0.017
Nigeria	Yabo	2000	0.501	0.836	1.252
Nigeria	Yabo	2017	0.272	0.421	0.645
Nigeria	Yabo	2000-2017	-0.065	-0.040	-0.013
Nigeria	Yagba East	2000	0.441	0.738	1.157
Nigeria	Yagba East	2017	0.174	0.276	0.401
Nigeria	Yagba East	2000-2017	-0.084	-0.059	-0.033
Nigeria	Yagba West	2000	0.462	0.740	1.102
Nigeria	Yagba West	2017	0.173	0.276	0.401
Nigeria	Yagba West	2000-2017	-0.085	-0.059	-0.031
Nigeria	Yakurr	2000	0.477	0.782	1.116
Nigeria	Yakurr	2017	0.190	0.303	0.448
Nigeria	Yakurr	2000-2017	-0.082	-0.055	-0.025
Nigeria	Yala Cross	2000	0.477	0.768	1.110
Nigeria	Yala Cross	2017	0.193	0.308	0.457
Nigeria	Yala Cross	2000-2017	-0.082	-0.053	-0.026
Nigeria	Yamaltu	2000	0.628	1.019	1.472
Nigeria	Yamaltu	2017	0.404	0.620	0.922
Nigeria	Yamaltu	2000-2017	-0.055	-0.028	-0.001
Nigeria	Yankwashi	2000	0.539	0.883	1.327
Nigeria	Yankwashi	2017	0.320	0.503	0.740
Nigeria	Yankwashi	2000-2017	-0.062	-0.032	0.002
Nigeria	Yauri	2000	0.460	0.764	1.169
Nigeria	Yauri	2017	0.236	0.362	0.548
Nigeria	Yauri	2000-2017	-0.068	-0.043	-0.014
Nigeria	Yenegoa	2000	0.439	0.713	1.082
Nigeria	Yenegoa	2017	0.170	0.281	0.451
Nigeria	Yenegoa	2000-2017	-0.083	-0.055	-0.025
Nigeria	Yola North	2000	0.631	1.070	1.541
Nigeria	Yola North	2017	0.335	0.535	0.772
Nigeria	Yola North	2000-2017	-0.075	-0.038	-0.005
Nigeria	Yola South	2000	0.662	1.079	1.646
Nigeria	Yola South	2017	0.349	0.534	0.827
Nigeria	Yola South	2000-2017	-0.075	-0.038	-0.010
Nigeria	Yorro	2000	0.678	1.042	1.517
Nigeria	Yorro	2017	0.354	0.540	0.779
Nigeria	Yorro	2000-2017	-0.063	-0.036	-0.008

Table 1: LRI DALYs rate by unit *(continued)*

Country	Unit	year	mean rate	lower rate	upper rate
Nigeria	Yunusari	2000	0.653	1.052	1.552
Nigeria	Yunusari	2017	0.424	0.648	0.940
Nigeria	Yunusari	2000-2017	-0.054	-0.027	-0.001
Nigeria	Yusufari	2000	0.595	0.977	1.456
Nigeria	Yusufari	2017	0.408	0.610	0.896
Nigeria	Yusufari	2000-2017	-0.056	-0.026	0.003
Nigeria	Zaki	2000	0.584	0.963	1.464
Nigeria	Zaki	2017	0.375	0.578	0.836
Nigeria	Zaki	2000-2017	-0.060	-0.028	0.000
Nigeria	Zango	2000	0.664	0.998	1.456
Nigeria	Zango	2017	0.341	0.515	0.735
Nigeria	Zango	2000-2017	-0.065	-0.037	-0.009
Nigeria	ZangonKa	2000	0.480	0.734	1.064
Nigeria	ZangonKa	2017	0.253	0.398	0.567
Nigeria	ZangonKa	2000-2017	-0.064	-0.036	-0.006
Nigeria	Zaria	2000	0.541	0.844	1.219
Nigeria	Zaria	2017	0.273	0.430	0.627
Nigeria	Zaria	2000-2017	-0.075	-0.038	-0.005
Nigeria	Zing	2000	0.654	1.028	1.494
Nigeria	Zing	2017	0.357	0.538	0.805
Nigeria	Zing	2000-2017	-0.066	-0.036	-0.007
Nigeria	Zurmi	2000	0.533	0.837	1.237
Nigeria	Zurmi	2017	0.300	0.446	0.670
Nigeria	Zurmi	2000-2017	-0.063	-0.036	-0.008
Nigeria	Zuru	2000	0.464	0.793	1.233
Nigeria	Zuru	2017	0.245	0.388	0.608
Nigeria	Zuru	2000-2017	-0.072	-0.041	-0.009
Republic of Congo	Abala	2000	0.179	0.287	0.430
Republic of Congo	Abala	2017	0.060	0.090	0.130
Republic of Congo	Abala	2000-2017	-0.086	-0.071	-0.056
Republic of Congo	Bambama	2000	0.194	0.307	0.444
Republic of Congo	Bambama	2017	0.061	0.091	0.133
Republic of Congo	Bambama	2000-2017	-0.085	-0.071	-0.058
Republic of Congo	Boko	2000	0.186	0.301	0.449
Republic of Congo	Boko	2017	0.061	0.081	0.114
Republic of Congo	Boko	2000-2017	-0.099	-0.079	-0.057
Republic of Congo	Boko-Songho	2000	0.187	0.291	0.423
Republic of Congo	Boko-Songho	2017	0.060	0.088	0.129
Republic of Congo	Boko-Songho	2000-2017	-0.088	-0.073	-0.058
Republic of Congo	Boundji	2000	0.183	0.291	0.437
Republic of Congo	Boundji	2017	0.061	0.090	0.131
Republic of Congo	Boundji	2000-2017	-0.087	-0.072	-0.057
Republic of Congo	Brazzaville	2000	0.138	0.221	0.323
Republic of Congo	Brazzaville	2017	0.041	0.065	0.102
Republic of Congo	Brazzaville	2000-2017	-0.109	-0.074	-0.043
Republic of Congo	Divénié	2000	0.192	0.303	0.450
Republic of Congo	Divénié	2017	0.060	0.088	0.128
Republic of Congo	Divénié	2000-2017	-0.090	-0.078	-0.063
Republic of Congo	Djambala	2000	0.179	0.287	0.425
Republic of Congo	Djambala	2017	0.060	0.089	0.128
Republic of Congo	Djambala	2000-2017	-0.086	-0.072	-0.058
Republic of Congo	Dongou	2000	0.254	0.368	0.518
Republic of Congo	Dongou	2017	0.062	0.090	0.131
Republic of Congo	Dongou	2000-2017	-0.101	-0.086	-0.070
Republic of Congo	Epéna	2000	0.167	0.274	0.411
Republic of Congo	Epéna	2017	0.061	0.090	0.132
Republic of Congo	Epéna	2000-2017	-0.087	-0.067	-0.048
Republic of Congo	Ewo	2000	0.184	0.288	0.421
Republic of Congo	Ewo	2017	0.060	0.089	0.132
Republic of Congo	Ewo	2000-2017	-0.085	-0.070	-0.054
Republic of Congo	Gamboma	2000	0.180	0.291	0.438

Table 1: LRI DALYs rate by unit *(continued)*

Country	Unit	year	mean rate	lower rate	upper rate
Republic of Congo	Gamboma	2017	0.061	0.089	0.130
Republic of Congo	Gamboma	2000-2017	-0.087	-0.072	-0.056
Republic of Congo	Impfondo	2000	0.206	0.301	0.430
Republic of Congo	Impfondo	2017	0.061	0.090	0.131
Republic of Congo	Impfondo	2000-2017	-0.092	-0.073	-0.054
Republic of Congo	Kakamoeka	2000	0.184	0.287	0.418
Republic of Congo	Kakamoeka	2017	0.055	0.084	0.122
Republic of Congo	Kakamoeka	2000-2017	-0.090	-0.076	-0.064
Republic of Congo	Kéllé	2000	0.190	0.294	0.435
Republic of Congo	Kéllé	2017	0.061	0.090	0.133
Republic of Congo	Kéllé	2000-2017	-0.085	-0.071	-0.057
Republic of Congo	Kibangou	2000	0.185	0.287	0.427
Republic of Congo	Kibangou	2017	0.058	0.087	0.127
Republic of Congo	Kibangou	2000-2017	-0.092	-0.077	-0.064
Republic of Congo	Kimongo	2000	0.192	0.291	0.424
Republic of Congo	Kimongo	2017	0.061	0.087	0.122
Republic of Congo	Kimongo	2000-2017	-0.087	-0.074	-0.059
Republic of Congo	Kindamba	2000	0.176	0.284	0.419
Republic of Congo	Kindamba	2017	0.060	0.089	0.130
Republic of Congo	Kindamba	2000-2017	-0.084	-0.070	-0.055
Republic of Congo	Kinkala	2000	0.177	0.291	0.446
Republic of Congo	Kinkala	2017	0.058	0.086	0.128
Republic of Congo	Kinkala	2000-2017	-0.093	-0.073	-0.056
Republic of Congo	Komono	2000	0.188	0.299	0.451
Republic of Congo	Komono	2017	0.060	0.090	0.132
Republic of Congo	Komono	2000-2017	-0.086	-0.071	-0.056
Republic of Congo	Lékana	2000	0.179	0.286	0.421
Republic of Congo	Lékana	2017	0.061	0.089	0.131
Republic of Congo	Lékana	2000-2017	-0.082	-0.070	-0.056
Republic of Congo	Loandjili	2000	0.160	0.243	0.361
Republic of Congo	Loandjili	2017	0.049	0.074	0.106
Republic of Congo	Loandjili	2000-2017	-0.087	-0.072	-0.055
Republic of Congo	Loudima	2000	0.180	0.288	0.424
Republic of Congo	Loudima	2017	0.059	0.088	0.128
Republic of Congo	Loudima	2000-2017	-0.084	-0.070	-0.056
Republic of Congo	Loukoléla	2000	0.223	0.326	0.477
Republic of Congo	Loukoléla	2017	0.063	0.088	0.127
Republic of Congo	Loukoléla	2000-2017	-0.094	-0.077	-0.062
Republic of Congo	Louvakou (Loubomo)	2000	0.183	0.287	0.424
Republic of Congo	Louvakou (Loubomo)	2017	0.059	0.088	0.128
Republic of Congo	Louvakou (Loubomo)	2000-2017	-0.087	-0.072	-0.060
Republic of Congo	Madingo-Kayes	2000	0.172	0.270	0.400
Republic of Congo	Madingo-Kayes	2017	0.054	0.082	0.119
Republic of Congo	Madingo-Kayes	2000-2017	-0.087	-0.073	-0.060
Republic of Congo	Madingou	2000	0.182	0.286	0.428
Republic of Congo	Madingou	2017	0.058	0.087	0.129
Republic of Congo	Madingou	2000-2017	-0.089	-0.072	-0.056
Republic of Congo	Makoua	2000	0.178	0.282	0.417
Republic of Congo	Makoua	2017	0.062	0.091	0.135
Republic of Congo	Makoua	2000-2017	-0.084	-0.069	-0.053
Republic of Congo	Mayama	2000	0.172	0.283	0.422
Republic of Congo	Mayama	2017	0.060	0.088	0.128
Republic of Congo	Mayama	2000-2017	-0.088	-0.071	-0.055
Republic of Congo	Mayoko	2000	0.193	0.310	0.470
Republic of Congo	Mayoko	2017	0.061	0.091	0.133
Republic of Congo	Mayoko	2000-2017	-0.089	-0.073	-0.057
Republic of Congo	Mbomo	2000	0.185	0.288	0.428
Republic of Congo	Mbomo	2017	0.061	0.091	0.132
Republic of Congo	Mbomo	2000-2017	-0.082	-0.068	-0.054
Republic of Congo	Mfouati	2000	0.188	0.290	0.413
Republic of Congo	Mfouati	2017	0.061	0.089	0.132

Table 1: LRI DALYs rate by unit *(continued)*

Country	Unit	year	mean rate	lower rate	upper rate
Republic of Congo	Mfouati	2000-2017	-0.089	-0.072	-0.052
Republic of Congo	Mindouli	2000	0.185	0.287	0.426
Republic of Congo	Mindouli	2017	0.063	0.090	0.135
Republic of Congo	Mindouli	2000-2017	-0.087	-0.070	-0.052
Republic of Congo	Mossaka	2000	0.189	0.304	0.452
Republic of Congo	Mossaka	2017	0.063	0.091	0.133
Republic of Congo	Mossaka	2000-2017	-0.093	-0.074	-0.055
Republic of Congo	Mossendjo	2000	0.185	0.294	0.432
Republic of Congo	Mossendjo	2017	0.060	0.090	0.131
Republic of Congo	Mossendjo	2000-2017	-0.087	-0.073	-0.059
Republic of Congo	Mouyondzi	2000	0.178	0.286	0.421
Republic of Congo	Mouyondzi	2017	0.059	0.089	0.131
Republic of Congo	Mouyondzi	2000-2017	-0.087	-0.071	-0.057
Republic of Congo	Mvouti	2000	0.184	0.289	0.424
Republic of Congo	Mvouti	2017	0.057	0.085	0.121
Republic of Congo	Mvouti	2000-2017	-0.086	-0.075	-0.064
Republic of Congo	Ngabé	2000	0.182	0.297	0.443
Republic of Congo	Ngabé	2017	0.062	0.084	0.122
Republic of Congo	Ngabé	2000-2017	-0.092	-0.076	-0.060
Republic of Congo	Ngamaba	2000	0.144	0.235	0.355
Republic of Congo	Ngamaba	2017	0.039	0.063	0.095
Republic of Congo	Ngamaba	2000-2017	-0.104	-0.080	-0.056
Republic of Congo	Nkayi District	2000	0.179	0.282	0.409
Republic of Congo	Nkayi District	2017	0.059	0.088	0.127
Republic of Congo	Nkayi District	2000-2017	-0.088	-0.071	-0.054
Republic of Congo	Okoyo	2000	0.179	0.286	0.427
Republic of Congo	Okoyo	2017	0.061	0.090	0.132
Republic of Congo	Okoyo	2000-2017	-0.083	-0.069	-0.054
Republic of Congo	Ouesso	2000	0.166	0.270	0.401
Republic of Congo	Ouesso	2017	0.061	0.090	0.131
Republic of Congo	Ouesso	2000-2017	-0.083	-0.066	-0.050
Republic of Congo	Owando	2000	0.183	0.291	0.432
Republic of Congo	Owando	2017	0.061	0.090	0.132
Republic of Congo	Owando	2000-2017	-0.088	-0.072	-0.056
Republic of Congo	Pointe Noire	2000	0.147	0.246	0.385
Republic of Congo	Pointe Noire	2017	0.042	0.067	0.106
Republic of Congo	Pointe Noire	2000-2017	-0.107	-0.079	-0.052
Republic of Congo	Sembé	2000	0.192	0.311	0.452
Republic of Congo	Sembé	2017	0.059	0.087	0.126
Republic of Congo	Sembé	2000-2017	-0.090	-0.076	-0.062
Republic of Congo	Sibiti	2000	0.178	0.287	0.428
Republic of Congo	Sibiti	2017	0.060	0.090	0.131
Republic of Congo	Sibiti	2000-2017	-0.083	-0.070	-0.056
Republic of Congo	Souanké	2000	0.205	0.322	0.485
Republic of Congo	Souanké	2017	0.061	0.089	0.128
Republic of Congo	Souanké	2000-2017	-0.088	-0.077	-0.065
Republic of Congo	Zanaga	2000	0.179	0.293	0.429
Republic of Congo	Zanaga	2017	0.060	0.090	0.132
Republic of Congo	Zanaga	2000-2017	-0.085	-0.070	-0.056
Rwanda	Bugesera	2000	0.526	0.678	0.866
Rwanda	Bugesera	2017	0.106	0.140	0.181
Rwanda	Bugesera	2000-2017	-0.104	-0.093	-0.081
Rwanda	Burera	2000	0.499	0.669	0.869
Rwanda	Burera	2017	0.093	0.128	0.167
Rwanda	Burera	2000-2017	-0.113	-0.099	-0.086
Rwanda	Gakenke	2000	0.462	0.626	0.813
Rwanda	Gakenke	2017	0.093	0.127	0.168
Rwanda	Gakenke	2000-2017	-0.107	-0.094	-0.082
Rwanda	Gasabo	2000	0.409	0.551	0.729
Rwanda	Gasabo	2017	0.086	0.118	0.159
Rwanda	Gasabo	2000-2017	-0.109	-0.091	-0.074

Table 1: LRI DALYs rate by unit *(continued)*

Country	Unit	year	mean rate	lower rate	upper rate
Rwanda	Gatsibo	2000	0.465	0.629	0.804
Rwanda	Gatsibo	2017	0.098	0.134	0.176
Rwanda	Gatsibo	2000-2017	-0.104	-0.093	-0.080
Rwanda	Gicumbi	2000	0.481	0.637	0.828
Rwanda	Gicumbi	2017	0.096	0.130	0.175
Rwanda	Gicumbi	2000-2017	-0.108	-0.096	-0.083
Rwanda	Gisagara	2000	0.533	0.689	0.897
Rwanda	Gisagara	2017	0.108	0.140	0.182
Rwanda	Gisagara	2000-2017	-0.106	-0.095	-0.083
Rwanda	Huye	2000	0.474	0.628	0.838
Rwanda	Huye	2017	0.093	0.129	0.168
Rwanda	Huye	2000-2017	-0.107	-0.093	-0.079
Rwanda	Kamonyi	2000	0.472	0.633	0.825
Rwanda	Kamonyi	2017	0.095	0.128	0.167
Rwanda	Kamonyi	2000-2017	-0.107	-0.093	-0.079
Rwanda	Karongi	2000	0.467	0.618	0.804
Rwanda	Karongi	2017	0.096	0.131	0.177
Rwanda	Karongi	2000-2017	-0.105	-0.092	-0.079
Rwanda	Kayonza	2000	0.474	0.641	0.832
Rwanda	Kayonza	2017	0.102	0.137	0.178
Rwanda	Kayonza	2000-2017	-0.105	-0.092	-0.080
Rwanda	Kicukiro	2000	0.385	0.549	0.754
Rwanda	Kicukiro	2017	0.082	0.118	0.160
Rwanda	Kicukiro	2000-2017	-0.112	-0.092	-0.070
Rwanda	Kirehe	2000	0.503	0.657	0.856
Rwanda	Kirehe	2017	0.103	0.137	0.180
Rwanda	Kirehe	2000-2017	-0.105	-0.093	-0.080
Rwanda	Muhanga	2000	0.466	0.616	0.815
Rwanda	Muhanga	2017	0.093	0.126	0.169
Rwanda	Muhanga	2000-2017	-0.106	-0.093	-0.079
Rwanda	Musanze	2000	0.452	0.615	0.804
Rwanda	Musanze	2017	0.086	0.120	0.160
Rwanda	Musanze	2000-2017	-0.111	-0.098	-0.082
Rwanda	Ngoma	2000	0.507	0.671	0.860
Rwanda	Ngoma	2017	0.102	0.138	0.182
Rwanda	Ngoma	2000-2017	-0.106	-0.094	-0.082
Rwanda	Ngororero	2000	0.458	0.630	0.823
Rwanda	Ngororero	2017	0.092	0.126	0.171
Rwanda	Ngororero	2000-2017	-0.110	-0.096	-0.084
Rwanda	Nyabihu	2000	0.455	0.620	0.805
Rwanda	Nyabihu	2017	0.087	0.122	0.162
Rwanda	Nyabihu	2000-2017	-0.112	-0.097	-0.083
Rwanda	Nyagatare	2000	0.470	0.618	0.807
Rwanda	Nyagatare	2017	0.100	0.133	0.175
Rwanda	Nyagatare	2000-2017	-0.105	-0.093	-0.082
Rwanda	Nyamagabe	2000	0.478	0.635	0.851
Rwanda	Nyamagabe	2017	0.095	0.130	0.175
Rwanda	Nyamagabe	2000-2017	-0.106	-0.094	-0.081
Rwanda	Nyamasheke	2000	0.433	0.588	0.787
Rwanda	Nyamasheke	2017	0.095	0.132	0.176
Rwanda	Nyamasheke	2000-2017	-0.102	-0.089	-0.077
Rwanda	Nyanza	2000	0.494	0.642	0.838
Rwanda	Nyanza	2017	0.096	0.129	0.172
Rwanda	Nyanza	2000-2017	-0.107	-0.094	-0.083
Rwanda	Nyarugenge	2000	0.384	0.545	0.741
Rwanda	Nyarugenge	2017	0.080	0.114	0.155
Rwanda	Nyarugenge	2000-2017	-0.114	-0.094	-0.072
Rwanda	Nyaruguru	2000	0.518	0.672	0.883
Rwanda	Nyaruguru	2017	0.099	0.134	0.173
Rwanda	Nyaruguru	2000-2017	-0.106	-0.095	-0.085
Rwanda	Rubavu	2000	0.425	0.594	0.796

Table 1: LRI DALYs rate by unit *(continued)*

Country	Unit	year	mean rate	lower rate	upper rate
Rwanda	Rubavu	2017	0.087	0.125	0.166
Rwanda	Rubavu	2000-2017	-0.111	-0.094	-0.077
Rwanda	Ruhango	2000	0.434	0.598	0.807
Rwanda	Ruhango	2017	0.092	0.125	0.166
Rwanda	Ruhango	2000-2017	-0.106	-0.092	-0.080
Rwanda	Rulindo	2000	0.459	0.627	0.823
Rwanda	Rulindo	2017	0.093	0.127	0.167
Rwanda	Rulindo	2000-2017	-0.109	-0.095	-0.081
Rwanda	Rusizi	2000	0.425	0.566	0.749
Rwanda	Rusizi	2017	0.098	0.136	0.187
Rwanda	Rusizi	2000-2017	-0.102	-0.086	-0.071
Rwanda	Rutsiro	2000	0.480	0.636	0.844
Rwanda	Rutsiro	2017	0.095	0.128	0.172
Rwanda	Rutsiro	2000-2017	-0.108	-0.095	-0.081
Rwanda	Rwamagana	2000	0.483	0.644	0.840
Rwanda	Rwamagana	2017	0.100	0.134	0.179
Rwanda	Rwamagana	2000-2017	-0.107	-0.093	-0.081
Senegal	Bakel	2000	0.203	0.286	0.398
Senegal	Bakel	2017	0.077	0.110	0.156
Senegal	Bakel	2000-2017	-0.083	-0.061	-0.036
Senegal	Bambey	2000	0.223	0.314	0.403
Senegal	Bambey	2017	0.083	0.123	0.164
Senegal	Bambey	2000-2017	-0.082	-0.058	-0.031
Senegal	Bignona	2000	0.226	0.324	0.452
Senegal	Bignona	2017	0.090	0.128	0.184
Senegal	Bignona	2000-2017	-0.082	-0.057	-0.031
Senegal	Birkilane	2000	0.225	0.315	0.421
Senegal	Birkilane	2017	0.089	0.126	0.171
Senegal	Birkilane	2000-2017	-0.079	-0.056	-0.030
Senegal	Bounkiling	2000	0.240	0.332	0.451
Senegal	Bounkiling	2017	0.086	0.123	0.171
Senegal	Bounkiling	2000-2017	-0.083	-0.059	-0.036
Senegal	Dagana	2000	0.224	0.316	0.432
Senegal	Dagana	2017	0.086	0.124	0.176
Senegal	Dagana	2000-2017	-0.079	-0.057	-0.033
Senegal	Dakar	2000	0.191	0.295	0.423
Senegal	Dakar	2017	0.071	0.110	0.164
Senegal	Dakar	2000-2017	-0.094	-0.060	-0.025
Senegal	Diourbel	2000	0.217	0.314	0.424
Senegal	Diourbel	2017	0.084	0.123	0.164
Senegal	Diourbel	2000-2017	-0.083	-0.058	-0.032
Senegal	Fatick	2000	0.219	0.316	0.419
Senegal	Fatick	2017	0.089	0.127	0.172
Senegal	Fatick	2000-2017	-0.081	-0.056	-0.028
Senegal	Foundiougne	2000	0.221	0.310	0.415
Senegal	Foundiougne	2017	0.091	0.128	0.172
Senegal	Foundiougne	2000-2017	-0.080	-0.054	-0.030
Senegal	Gossas	2000	0.224	0.309	0.410
Senegal	Gossas	2017	0.084	0.121	0.162
Senegal	Gossas	2000-2017	-0.085	-0.058	-0.032
Senegal	Goudiry	2000	0.215	0.290	0.395
Senegal	Goudiry	2017	0.082	0.115	0.161
Senegal	Goudiry	2000-2017	-0.081	-0.060	-0.038
Senegal	Goudomp	2000	0.238	0.336	0.473
Senegal	Goudomp	2017	0.087	0.123	0.177
Senegal	Goudomp	2000-2017	-0.085	-0.060	-0.036
Senegal	Guédiawaye	2000	0.180	0.293	0.442
Senegal	Guédiawaye	2017	0.067	0.109	0.169
Senegal	Guédiawaye	2000-2017	-0.096	-0.060	-0.019
Senegal	Guinguinéo	2000	0.225	0.312	0.416
Senegal	Guinguinéo	2017	0.089	0.128	0.171

Table 1: LRI DALYs rate by unit *(continued)*

Country	Unit	year	mean rate	lower rate	upper rate
Senegal	Guinguinéo	2000-2017	-0.079	-0.055	-0.028
Senegal	Kaffrine	2000	0.226	0.310	0.409
Senegal	Kaffrine	2017	0.087	0.122	0.163
Senegal	Kaffrine	2000-2017	-0.083	-0.058	-0.032
Senegal	Kanel	2000	0.192	0.280	0.384
Senegal	Kanel	2017	0.076	0.112	0.157
Senegal	Kanel	2000-2017	-0.078	-0.057	-0.034
Senegal	Kaolack	2000	0.221	0.310	0.414
Senegal	Kaolack	2017	0.088	0.128	0.174
Senegal	Kaolack	2000-2017	-0.076	-0.054	-0.026
Senegal	Kébémer	2000	0.220	0.314	0.409
Senegal	Kébémer	2017	0.083	0.124	0.169
Senegal	Kébémer	2000-2017	-0.079	-0.057	-0.032
Senegal	Kédougou	2000	0.221	0.312	0.422
Senegal	Kédougou	2017	0.077	0.111	0.156
Senegal	Kédougou	2000-2017	-0.091	-0.067	-0.044
Senegal	Kolda	2000	0.243	0.330	0.458
Senegal	Kolda	2017	0.084	0.119	0.166
Senegal	Kolda	2000-2017	-0.086	-0.061	-0.038
Senegal	Koungheul	2000	0.227	0.309	0.422
Senegal	Koungheul	2017	0.088	0.121	0.164
Senegal	Koungheul	2000-2017	-0.078	-0.057	-0.033
Senegal	Koupentoum	2000	0.220	0.295	0.396
Senegal	Koupentoum	2017	0.084	0.119	0.164
Senegal	Koupentoum	2000-2017	-0.078	-0.056	-0.034
Senegal	Linguère	2000	0.221	0.303	0.408
Senegal	Linguère	2017	0.087	0.120	0.162
Senegal	Linguère	2000-2017	-0.078	-0.056	-0.032
Senegal	Louga	2000	0.226	0.317	0.423
Senegal	Louga	2017	0.085	0.124	0.174
Senegal	Louga	2000-2017	-0.079	-0.057	-0.031
Senegal	Malème Hodar	2000	0.221	0.305	0.415
Senegal	Malème Hodar	2017	0.087	0.121	0.159
Senegal	Malème Hodar	2000-2017	-0.078	-0.057	-0.033
Senegal	Matam	2000	0.197	0.282	0.386
Senegal	Matam	2017	0.079	0.114	0.157
Senegal	Matam	2000-2017	-0.079	-0.056	-0.033
Senegal	Mbacké	2000	0.214	0.306	0.408
Senegal	Mbacké	2017	0.086	0.122	0.161
Senegal	Mbacké	2000-2017	-0.080	-0.056	-0.031
Senegal	Mbour	2000	0.223	0.317	0.422
Senegal	Mbour	2017	0.084	0.125	0.174
Senegal	Mbour	2000-2017	-0.084	-0.057	-0.027
Senegal	Médina Yoro Foula	2000	0.227	0.307	0.420
Senegal	Médina Yoro Foula	2017	0.082	0.114	0.158
Senegal	Médina Yoro Foula	2000-2017	-0.084	-0.060	-0.037
Senegal	Nioro du Rip	2000	0.221	0.307	0.411
Senegal	Nioro du Rip	2017	0.084	0.120	0.162
Senegal	Nioro du Rip	2000-2017	-0.079	-0.057	-0.034
Senegal	Oussouye	2000	0.206	0.308	0.433
Senegal	Oussouye	2017	0.080	0.121	0.175
Senegal	Oussouye	2000-2017	-0.079	-0.056	-0.025
Senegal	Pikine	2000	0.190	0.295	0.405
Senegal	Pikine	2017	0.070	0.110	0.161
Senegal	Pikine	2000-2017	-0.095	-0.061	-0.029
Senegal	Podor	2000	0.210	0.292	0.405
Senegal	Podor	2017	0.085	0.118	0.169
Senegal	Podor	2000-2017	-0.078	-0.056	-0.030
Senegal	Ranérou Ferlo	2000	0.211	0.285	0.393
Senegal	Ranérou Ferlo	2017	0.081	0.115	0.159
Senegal	Ranérou Ferlo	2000-2017	-0.077	-0.056	-0.035

Table 1: LRI DALYs rate by unit *(continued)*

Country	Unit	year	mean rate	lower rate	upper rate
Senegal	Rufisque	2000	0.190	0.283	0.379
Senegal	Rufisque	2017	0.072	0.107	0.152
Senegal	Rufisque	2000-2017	-0.085	-0.060	-0.031
Senegal	Saint-Louis	2000	0.219	0.322	0.444
Senegal	Saint-Louis	2017	0.083	0.126	0.181
Senegal	Saint-Louis	2000-2017	-0.084	-0.057	-0.030
Senegal	Salémata	2000	0.226	0.310	0.414
Senegal	Salémata	2017	0.079	0.113	0.160
Senegal	Salémata	2000-2017	-0.087	-0.065	-0.041
Senegal	Saraya	2000	0.224	0.312	0.430
Senegal	Saraya	2017	0.073	0.103	0.146
Senegal	Saraya	2000-2017	-0.095	-0.071	-0.048
Senegal	Sédhiou	2000	0.239	0.335	0.462
Senegal	Sédhiou	2017	0.088	0.125	0.180
Senegal	Sédhiou	2000-2017	-0.084	-0.060	-0.034
Senegal	Tambacounda	2000	0.220	0.295	0.405
Senegal	Tambacounda	2017	0.081	0.116	0.160
Senegal	Tambacounda	2000-2017	-0.079	-0.059	-0.036
Senegal	Thiès	2000	0.201	0.294	0.392
Senegal	Thiès	2017	0.078	0.117	0.158
Senegal	Thiès	2000-2017	-0.081	-0.056	-0.032
Senegal	Tivaouane	2000	0.222	0.320	0.420
Senegal	Tivaouane	2017	0.084	0.125	0.171
Senegal	Tivaouane	2000-2017	-0.080	-0.057	-0.031
Senegal	Vélingara	2000	0.237	0.314	0.425
Senegal	Vélingara	2017	0.079	0.113	0.160
Senegal	Vélingara	2000-2017	-0.083	-0.061	-0.036
Senegal	Ziguinchor	2000	0.203	0.292	0.403
Senegal	Ziguinchor	2017	0.072	0.109	0.156
Senegal	Ziguinchor	2000-2017	-0.087	-0.060	-0.032
Sierra Leone	Bo	2000	0.491	0.737	1.056
Sierra Leone	Bo	2017	0.184	0.278	0.391
Sierra Leone	Bo	2000-2017	-0.088	-0.057	-0.028
Sierra Leone	Bombali	2000	0.508	0.751	1.031
Sierra Leone	Bombali	2017	0.196	0.296	0.416
Sierra Leone	Bombali	2000-2017	-0.080	-0.055	-0.028
Sierra Leone	Bonthe	2000	0.481	0.735	1.062
Sierra Leone	Bonthe	2017	0.207	0.310	0.447
Sierra Leone	Bonthe	2000-2017	-0.076	-0.050	-0.021
Sierra Leone	Kailahun	2000	0.430	0.646	0.939
Sierra Leone	Kailahun	2017	0.187	0.279	0.400
Sierra Leone	Kailahun	2000-2017	-0.073	-0.048	-0.022
Sierra Leone	Kambia	2000	0.518	0.765	1.077
Sierra Leone	Kambia	2017	0.209	0.310	0.444
Sierra Leone	Kambia	2000-2017	-0.078	-0.052	-0.024
Sierra Leone	Kenema	2000	0.489	0.735	1.074
Sierra Leone	Kenema	2017	0.184	0.277	0.400
Sierra Leone	Kenema	2000-2017	-0.087	-0.056	-0.028
Sierra Leone	Koinadugu	2000	0.456	0.699	0.972
Sierra Leone	Koinadugu	2017	0.216	0.334	0.467
Sierra Leone	Koinadugu	2000-2017	-0.066	-0.042	-0.018
Sierra Leone	Kono	2000	0.490	0.734	1.026
Sierra Leone	Kono	2017	0.182	0.285	0.401
Sierra Leone	Kono	2000-2017	-0.075	-0.052	-0.023
Sierra Leone	Moyamba	2000	0.516	0.750	1.051
Sierra Leone	Moyamba	2017	0.199	0.301	0.435
Sierra Leone	Moyamba	2000-2017	-0.078	-0.052	-0.024
Sierra Leone	Port Loko	2000	0.499	0.730	1.024
Sierra Leone	Port Loko	2017	0.201	0.299	0.420
Sierra Leone	Port Loko	2000-2017	-0.078	-0.052	-0.024
Sierra Leone	Pujehun	2000	0.470	0.728	1.036

Table 1: LRI DALYs rate by unit *(continued)*

Country	Unit	year	mean rate	lower rate	upper rate
Sierra Leone	Pujehun	2017	0.208	0.312	0.449
Sierra Leone	Pujehun	2000-2017	-0.074	-0.049	-0.021
Sierra Leone	Tonkolili	2000	0.519	0.762	1.051
Sierra Leone	Tonkolili	2017	0.203	0.308	0.439
Sierra Leone	Tonkolili	2000-2017	-0.079	-0.053	-0.026
Sierra Leone	Western Rural	2000	0.326	0.508	0.751
Sierra Leone	Western Rural	2017	0.170	0.270	0.389
Sierra Leone	Western Rural	2000-2017	-0.071	-0.037	-0.004
Sierra Leone	Western Urban	2000	0.277	0.465	0.691
Sierra Leone	Western Urban	2017	0.160	0.256	0.394
Sierra Leone	Western Urban	2000-2017	-0.074	-0.035	0.001
Somalia	Aadan	2000	0.487	0.787	1.291
Somalia	Aadan	2017	0.123	0.211	0.327
Somalia	Aadan	2000-2017	-0.092	-0.075	-0.060
Somalia	Afgooye	2000	0.457	0.762	1.225
Somalia	Afgooye	2017	0.169	0.285	0.454
Somalia	Afgooye	2000-2017	-0.072	-0.055	-0.038
Somalia	Afmadow	2000	0.510	0.841	1.379
Somalia	Afmadow	2017	0.161	0.265	0.406
Somalia	Afmadow	2000-2017	-0.081	-0.067	-0.053
Somalia	Baar-Dheere	2000	0.386	0.619	1.010
Somalia	Baar-Dheere	2017	0.147	0.239	0.360
Somalia	Baar-Dheere	2000-2017	-0.069	-0.054	-0.040
Somalia	Badhaadhe	2000	0.652	1.148	1.966
Somalia	Badhaadhe	2017	0.207	0.336	0.518
Somalia	Badhaadhe	2000-2017	-0.087	-0.071	-0.053
Somalia	Badhan	2000	0.290	0.467	0.726
Somalia	Badhan	2017	0.116	0.193	0.298
Somalia	Badhan	2000-2017	-0.059	-0.047	-0.037
Somalia	Baki	2000	0.285	0.436	0.684
Somalia	Baki	2017	0.075	0.127	0.203
Somalia	Baki	2000-2017	-0.089	-0.073	-0.057
Somalia	Balcad	2000	0.514	0.841	1.374
Somalia	Balcad	2017	0.188	0.310	0.480
Somalia	Balcad	2000-2017	-0.072	-0.056	-0.041
Somalia	Bander-Beyla	2000	0.212	0.329	0.513
Somalia	Bander-Beyla	2017	0.088	0.146	0.225
Somalia	Bander-Beyla	2000-2017	-0.054	-0.042	-0.030
Somalia	Baraawe	2000	0.521	0.826	1.319
Somalia	Baraawe	2017	0.199	0.324	0.503
Somalia	Baraawe	2000-2017	-0.071	-0.055	-0.040
Somalia	Baydhabo	2000	0.447	0.708	1.142
Somalia	Baydhabo	2017	0.180	0.290	0.443
Somalia	Baydhabo	2000-2017	-0.057	-0.046	-0.034
Somalia	Beled Weyn	2000	0.270	0.416	0.665
Somalia	Beled Weyn	2017	0.110	0.181	0.280
Somalia	Beled Weyn	2000-2017	-0.057	-0.047	-0.037
Somalia	Beled Xaawo	2000	0.365	0.578	0.916
Somalia	Beled Xaawo	2017	0.125	0.206	0.314
Somalia	Beled Xaawo	2000-2017	-0.078	-0.063	-0.047
Somalia	Berbera	2000	0.224	0.354	0.554
Somalia	Berbera	2017	0.068	0.119	0.183
Somalia	Berbera	2000-2017	-0.070	-0.056	-0.043
Somalia	Boorama	2000	0.272	0.426	0.676
Somalia	Boorama	2017	0.080	0.131	0.211
Somalia	Boorama	2000-2017	-0.087	-0.072	-0.056
Somalia	Bosaaso	2000	0.259	0.407	0.659
Somalia	Bosaaso	2017	0.107	0.175	0.274
Somalia	Bosaaso	2000-2017	-0.062	-0.048	-0.036
Somalia	Bu'aale	2000	0.476	0.755	1.173
Somalia	Bu'aale	2017	0.192	0.314	0.487

Table 1: LRI DALYs rate by unit *(continued)*

Country	Unit	year	mean rate	lower rate	upper rate
Somalia	Bu'aale	2000-2017	-0.060	-0.047	-0.034
Somalia	Burao	2000	0.324	0.506	0.767
Somalia	Burao	2017	0.090	0.158	0.245
Somalia	Burao	2000-2017	-0.076	-0.064	-0.052
Somalia	Burtinle	2000	0.285	0.450	0.684
Somalia	Burtinle	2017	0.099	0.174	0.272
Somalia	Burtinle	2000-2017	-0.067	-0.055	-0.043
Somalia	Buuhoodle	2000	0.223	0.360	0.569
Somalia	Buuhoodle	2017	0.082	0.139	0.221
Somalia	Buuhoodle	2000-2017	-0.066	-0.056	-0.045
Somalia	Buulo Burdo	2000	0.302	0.464	0.719
Somalia	Buulo Burdo	2017	0.115	0.192	0.296
Somalia	Buulo Burdo	2000-2017	-0.062	-0.049	-0.039
Somalia	Buur Xakaba	2000	0.457	0.729	1.180
Somalia	Buur Xakaba	2017	0.185	0.303	0.462
Somalia	Buur Xakaba	2000-2017	-0.064	-0.051	-0.038
Somalia	Caabudwaaq	2000	0.273	0.431	0.655
Somalia	Caabudwaaq	2017	0.123	0.212	0.325
Somalia	Caabudwaaq	2000-2017	-0.045	-0.036	-0.027
Somalia	Cadaado	2000	0.296	0.465	0.702
Somalia	Cadaado	2017	0.127	0.222	0.346
Somalia	Cadaado	2000-2017	-0.047	-0.037	-0.029
Somalia	Cadale	2000	0.505	0.819	1.311
Somalia	Cadale	2017	0.138	0.232	0.360
Somalia	Cadale	2000-2017	-0.081	-0.067	-0.050
Somalia	Calawla	2000	0.242	0.378	0.601
Somalia	Calawla	2017	0.116	0.184	0.282
Somalia	Calawla	2000-2017	-0.046	-0.038	-0.029
Somalia	Caynabo	2000	0.383	0.613	0.953
Somalia	Caynabo	2017	0.097	0.174	0.273
Somalia	Caynabo	2000-2017	-0.086	-0.072	-0.058
Somalia	Ceel Barde	2000	0.358	0.548	0.872
Somalia	Ceel Barde	2017	0.147	0.233	0.355
Somalia	Ceel Barde	2000-2017	-0.059	-0.049	-0.038
Somalia	Ceel Buur	2000	0.282	0.444	0.684
Somalia	Ceel Buur	2017	0.103	0.185	0.284
Somalia	Ceel Buur	2000-2017	-0.057	-0.045	-0.033
Somalia	Ceel Dheer	2000	0.441	0.714	1.094
Somalia	Ceel Dheer	2017	0.125	0.217	0.339
Somalia	Ceel Dheer	2000-2017	-0.085	-0.069	-0.056
Somalia	Ceel Waaq	2000	0.388	0.605	0.976
Somalia	Ceel Waaq	2017	0.142	0.233	0.358
Somalia	Ceel Waaq	2000-2017	-0.068	-0.054	-0.039
Somalia	Ceel-Afwein	2000	0.265	0.422	0.640
Somalia	Ceel-Afwein	2017	0.085	0.147	0.231
Somalia	Ceel-Afwein	2000-2017	-0.069	-0.057	-0.044
Somalia	Ceerigaabo	2000	0.253	0.410	0.626
Somalia	Ceerigaabo	2017	0.101	0.169	0.264
Somalia	Ceerigaabo	2000-2017	-0.053	-0.044	-0.035
Somalia	Dhuusamareeb	2000	0.235	0.376	0.582
Somalia	Dhuusamareeb	2017	0.111	0.193	0.299
Somalia	Dhuusamareeb	2000-2017	-0.041	-0.032	-0.021
Somalia	Diinsoor	2000	0.472	0.763	1.224
Somalia	Diinsoor	2017	0.208	0.328	0.491
Somalia	Diinsoor	2000-2017	-0.058	-0.047	-0.034
Somalia	Dolow	2000	0.307	0.476	0.750
Somalia	Dolow	2017	0.087	0.150	0.253
Somalia	Dolow	2000-2017	-0.094	-0.072	-0.047
Somalia	Eyl	2000	0.286	0.451	0.687
Somalia	Eyl	2017	0.103	0.182	0.285
Somalia	Eyl	2000-2017	-0.064	-0.051	-0.038

Table 1: LRI DALYs rate by unit *(continued)*

Country	Unit	year	mean rate	lower rate	upper rate
Somalia	Gaalkacayo	2000	0.208	0.329	0.506
Somalia	Gaalkacayo	2017	0.078	0.133	0.207
Somalia	Gaalkacayo	2000-2017	-0.060	-0.049	-0.040
Somalia	Gabiley	2000	0.345	0.531	0.826
Somalia	Gabiley	2017	0.112	0.185	0.286
Somalia	Gabiley	2000-2017	-0.076	-0.062	-0.045
Somalia	Garbahaaray	2000	0.330	0.508	0.811
Somalia	Garbahaaray	2017	0.117	0.191	0.293
Somalia	Garbahaaray	2000-2017	-0.072	-0.058	-0.045
Somalia	Garoowe	2000	0.301	0.492	0.748
Somalia	Garoowe	2017	0.110	0.197	0.307
Somalia	Garoowe	2000-2017	-0.063	-0.051	-0.039
Somalia	Goldogob	2000	0.255	0.410	0.644
Somalia	Goldogob	2017	0.099	0.170	0.264
Somalia	Goldogob	2000-2017	-0.060	-0.048	-0.036
Somalia	Hargeysa	2000	0.288	0.442	0.704
Somalia	Hargeysa	2017	0.089	0.149	0.234
Somalia	Hargeysa	2000-2017	-0.074	-0.060	-0.047
Somalia	Hobyo	2000	0.274	0.434	0.676
Somalia	Hobyo	2017	0.095	0.161	0.247
Somalia	Hobyo	2000-2017	-0.069	-0.056	-0.045
Somalia	Iskushuban	2000	0.233	0.359	0.568
Somalia	Iskushuban	2017	0.100	0.162	0.250
Somalia	Iskushuban	2000-2017	-0.052	-0.040	-0.029
Somalia	Jalalaqsi	2000	0.387	0.610	0.961
Somalia	Jalalaqsi	2017	0.141	0.236	0.363
Somalia	Jalalaqsi	2000-2017	-0.067	-0.054	-0.039
Somalia	Jamaame	2000	0.668	1.134	1.918
Somalia	Jamaame	2017	0.259	0.423	0.666
Somalia	Jamaame	2000-2017	-0.068	-0.056	-0.043
Somalia	Jariiban	2000	0.286	0.451	0.671
Somalia	Jariiban	2017	0.094	0.169	0.261
Somalia	Jariiban	2000-2017	-0.069	-0.057	-0.043
Somalia	Jawhar	2000	0.504	0.831	1.350
Somalia	Jawhar	2017	0.198	0.320	0.499
Somalia	Jawhar	2000-2017	-0.069	-0.054	-0.039
Somalia	Jilib	2000	0.477	0.743	1.182
Somalia	Jilib	2017	0.189	0.311	0.486
Somalia	Jilib	2000-2017	-0.061	-0.049	-0.037
Somalia	Kismaayo	2000	0.584	0.991	1.653
Somalia	Kismaayo	2017	0.210	0.340	0.514
Somalia	Kismaayo	2000-2017	-0.075	-0.062	-0.047
Somalia	Kuntuwaaray	2000	0.489	0.805	1.314
Somalia	Kuntuwaaray	2017	0.195	0.323	0.498
Somalia	Kuntuwaaray	2000-2017	-0.073	-0.058	-0.043
Somalia	Lascaanod	2000	0.335	0.532	0.816
Somalia	Lascaanod	2017	0.101	0.179	0.278
Somalia	Lascaanod	2000-2017	-0.075	-0.062	-0.050
Somalia	Lughaya	2000	0.301	0.473	0.749
Somalia	Lughaya	2017	0.082	0.140	0.216
Somalia	Lughaya	2000-2017	-0.086	-0.071	-0.054
Somalia	Luuk	2000	0.288	0.450	0.714
Somalia	Luuk	2017	0.097	0.162	0.256
Somalia	Luuk	2000-2017	-0.074	-0.060	-0.045
Somalia	Marka	2000	0.509	0.814	1.321
Somalia	Marka	2017	0.201	0.323	0.510
Somalia	Marka	2000-2017	-0.071	-0.054	-0.037
Somalia	Mogadisho	2000	0.362	0.597	0.910
Somalia	Mogadisho	2017	0.110	0.194	0.310
Somalia	Mogadisho	2000-2017	-0.088	-0.065	-0.037
Somalia	Oodweyne	2000	0.396	0.628	0.959

Table 1: LRI DALYs rate by unit *(continued)*

Country	Unit	year	mean rate	lower rate	upper rate
Somalia	Oodweyne	2017	0.102	0.170	0.257
Somalia	Oodweyne	2000-2017	-0.085	-0.073	-0.058
Somalia	Qandala	2000	0.265	0.423	0.692
Somalia	Qandala	2017	0.110	0.179	0.282
Somalia	Qandala	2000-2017	-0.060	-0.048	-0.036
Somalia	Qansax Dheere	2000	0.481	0.765	1.233
Somalia	Qansax Dheere	2017	0.194	0.315	0.482
Somalia	Qansax Dheere	2000-2017	-0.063	-0.051	-0.038
Somalia	Qardho	2000	0.237	0.366	0.582
Somalia	Qardho	2017	0.094	0.155	0.244
Somalia	Qardho	2000-2017	-0.057	-0.046	-0.035
Somalia	Qoryooley	2000	0.488	0.794	1.310
Somalia	Qoryooley	2017	0.207	0.328	0.510
Somalia	Qoryooley	2000-2017	-0.069	-0.053	-0.039
Somalia	Rab Dhuure	2000	0.388	0.604	0.961
Somalia	Rab Dhuure	2017	0.148	0.249	0.383
Somalia	Rab Dhuure	2000-2017	-0.065	-0.051	-0.033
Somalia	Saakow	2000	0.463	0.727	1.161
Somalia	Saakow	2017	0.189	0.315	0.484
Somalia	Saakow	2000-2017	-0.055	-0.043	-0.029
Somalia	Sablale	2000	0.510	0.832	1.346
Somalia	Sablale	2017	0.211	0.341	0.517
Somalia	Sablale	2000-2017	-0.065	-0.051	-0.037
Somalia	Sheekh	2000	0.353	0.576	0.879
Somalia	Sheekh	2017	0.090	0.156	0.243
Somalia	Sheekh	2000-2017	-0.084	-0.070	-0.054
Somalia	Taleex	2000	0.423	0.691	1.089
Somalia	Taleex	2017	0.156	0.267	0.420
Somalia	Taleex	2000-2017	-0.059	-0.049	-0.038
Somalia	Tiyeeglow	2000	0.406	0.622	0.984
Somalia	Tiyeeglow	2017	0.163	0.264	0.393
Somalia	Tiyeeglow	2000-2017	-0.060	-0.048	-0.035
Somalia	Wajid	2000	0.350	0.541	0.870
Somalia	Wajid	2017	0.137	0.228	0.352
Somalia	Wajid	2000-2017	-0.063	-0.049	-0.034
Somalia	Wanla Weyn	2000	0.488	0.790	1.290
Somalia	Wanla Weyn	2017	0.199	0.323	0.496
Somalia	Wanla Weyn	2000-2017	-0.065	-0.051	-0.037
Somalia	Xarardheere	2000	0.378	0.610	0.956
Somalia	Xarardheere	2017	0.121	0.207	0.330
Somalia	Xarardheere	2000-2017	-0.076	-0.062	-0.047
Somalia	Xudun	2000	0.302	0.482	0.729
Somalia	Xudun	2017	0.096	0.173	0.265
Somalia	Xudun	2000-2017	-0.071	-0.059	-0.047
Somalia	Xudur	2000	0.341	0.533	0.856
Somalia	Xudur	2017	0.143	0.234	0.363
Somalia	Xudur	2000-2017	-0.059	-0.048	-0.035
Somalia	Zeylac	2000	0.309	0.479	0.769
Somalia	Zeylac	2017	0.098	0.156	0.239
Somalia	Zeylac	2000-2017	-0.082	-0.067	-0.054
South Sudan	Akobo	2000	0.268	0.493	0.822
South Sudan	Akobo	2017	0.235	0.433	0.675
South Sudan	Akobo	2000-2017	-0.017	-0.008	0.001
South Sudan	Al Leiri	2000	0.309	0.566	0.934
South Sudan	Al Leiri	2017	0.272	0.507	0.811
South Sudan	Al Leiri	2000-2017	-0.012	-0.005	0.002
South Sudan	Al Mabien	2000	0.274	0.496	0.822
South Sudan	Al Mabien	2017	0.244	0.441	0.687
South Sudan	Al Mabien	2000-2017	-0.014	-0.007	0.001
South Sudan	Al Mayom	2000	0.302	0.556	0.930
South Sudan	Al Mayom	2017	0.269	0.496	0.804

Table 1: LRI DALYs rate by unit *(continued)*

Country	Unit	year	mean rate	lower rate	upper rate
South Sudan	Al Mayom	2000-2017	-0.013	-0.004	0.005
South Sudan	Al Renk	2000	0.265	0.490	0.807
South Sudan	Al Renk	2017	0.255	0.460	0.739
South Sudan	Al Renk	2000-2017	-0.007	-0.002	0.004
South Sudan	Aliab	2000	0.278	0.511	0.825
South Sudan	Aliab	2017	0.276	0.495	0.787
South Sudan	Aliab	2000-2017	-0.012	-0.003	0.006
South Sudan	Amatonge	2000	0.267	0.488	0.790
South Sudan	Amatonge	2017	0.283	0.524	0.809
South Sudan	Amatonge	2000-2017	-0.007	0.006	0.018
South Sudan	Aryat	2000	0.301	0.561	0.941
South Sudan	Aryat	2017	0.234	0.425	0.650
South Sudan	Aryat	2000-2017	-0.027	-0.011	0.005
South Sudan	Aweil	2000	0.300	0.554	0.932
South Sudan	Aweil	2017	0.237	0.440	0.673
South Sudan	Aweil	2000-2017	-0.022	-0.009	0.005
South Sudan	Ayod	2000	0.249	0.458	0.762
South Sudan	Ayod	2017	0.217	0.399	0.651
South Sudan	Ayod	2000-2017	-0.014	-0.006	0.002
South Sudan	Bahr al Jabal	2000	0.229	0.422	0.671
South Sudan	Bahr al Jabal	2017	0.246	0.453	0.694
South Sudan	Bahr al Jabal	2000-2017	-0.008	0.004	0.015
South Sudan	Baleit	2000	0.273	0.503	0.842
South Sudan	Baleit	2017	0.247	0.462	0.737
South Sudan	Baleit	2000-2017	-0.011	-0.003	0.007
South Sudan	Bor	2000	0.280	0.515	0.836
South Sudan	Bor	2017	0.266	0.481	0.772
South Sudan	Bor	2000-2017	-0.015	-0.005	0.005
South Sudan	Fam al Zaraf	2000	0.258	0.485	0.794
South Sudan	Fam al Zaraf	2017	0.240	0.446	0.723
South Sudan	Fam al Zaraf	2000-2017	-0.012	-0.003	0.006
South Sudan	Faring	2000	0.298	0.537	0.856
South Sudan	Faring	2017	0.277	0.495	0.778
South Sudan	Faring	2000-2017	-0.009	-0.003	0.004
South Sudan	Fashooda	2000	0.271	0.490	0.804
South Sudan	Fashooda	2017	0.247	0.443	0.712
South Sudan	Fashooda	2000-2017	-0.009	-0.003	0.002
South Sudan	Gogrial	2000	0.300	0.558	0.925
South Sudan	Gogrial	2017	0.213	0.399	0.640
South Sudan	Gogrial	2000-2017	-0.026	-0.016	-0.006
South Sudan	Kajo Kaii	2000	0.236	0.424	0.678
South Sudan	Kajo Kaii	2017	0.230	0.433	0.660
South Sudan	Kajo Kaii	2000-2017	-0.010	0.003	0.017
South Sudan	Kapoeta	2000	0.274	0.506	0.822
South Sudan	Kapoeta	2017	0.252	0.462	0.705
South Sudan	Kapoeta	2000-2017	-0.015	-0.004	0.007
South Sudan	Magwi	2000	0.258	0.484	0.773
South Sudan	Magwi	2017	0.265	0.498	0.769
South Sudan	Magwi	2000-2017	-0.010	0.002	0.014
South Sudan	Malek	2000	0.322	0.581	0.957
South Sudan	Malek	2017	0.220	0.426	0.656
South Sudan	Malek	2000-2017	-0.028	-0.015	-0.001
South Sudan	Malut	2000	0.275	0.497	0.825
South Sudan	Malut	2017	0.239	0.441	0.711
South Sudan	Malut	2000-2017	-0.011	-0.005	0.001
South Sudan	Mayot	2000	0.296	0.526	0.876
South Sudan	Mayot	2017	0.208	0.374	0.585
South Sudan	Mayot	2000-2017	-0.029	-0.018	-0.006
South Sudan	Meridi	2000	0.242	0.448	0.716
South Sudan	Meridi	2017	0.250	0.464	0.708
South Sudan	Meridi	2000-2017	-0.005	0.005	0.014

Table 1: LRI DALYs rate by unit *(continued)*

Country	Unit	year	mean rate	lower rate	upper rate
South Sudan	Mundri	2000	0.238	0.442	0.713
South Sudan	Mundri	2017	0.239	0.429	0.665
South Sudan	Mundri	2000-2017	-0.009	-0.001	0.007
South Sudan	Nahr Atiem	2000	0.290	0.529	0.876
South Sudan	Nahr Atiem	2017	0.228	0.419	0.649
South Sudan	Nahr Atiem	2000-2017	-0.023	-0.013	-0.001
South Sudan	Nahr Lol	2000	0.286	0.527	0.898
South Sudan	Nahr Lol	2017	0.211	0.395	0.625
South Sudan	Nahr Lol	2000-2017	-0.025	-0.013	-0.002
South Sudan	Nahr Yei	2000	0.233	0.426	0.709
South Sudan	Nahr Yei	2017	0.258	0.477	0.740
South Sudan	Nahr Yei	2000-2017	-0.004	0.008	0.019
South Sudan	Pibor	2000	0.278	0.513	0.842
South Sudan	Pibor	2017	0.210	0.392	0.605
South Sudan	Pibor	2000-2017	-0.028	-0.017	-0.005
South Sudan	Rabkona	2000	0.291	0.542	0.903
South Sudan	Rabkona	2017	0.286	0.516	0.834
South Sudan	Rabkona	2000-2017	-0.007	-0.001	0.006
South Sudan	Raja	2000	0.239	0.419	0.676
South Sudan	Raja	2017	0.268	0.449	0.688
South Sudan	Raja	2000-2017	0.000	0.009	0.018
South Sudan	Rumbek	2000	0.296	0.539	0.873
South Sudan	Rumbek	2017	0.258	0.480	0.756
South Sudan	Rumbek	2000-2017	-0.012	-0.006	0.000
South Sudan	Shobet	2000	0.295	0.550	0.893
South Sudan	Shobet	2017	0.247	0.461	0.747
South Sudan	Shobet	2000-2017	-0.018	-0.010	-0.003
South Sudan	Shokodom	2000	0.274	0.499	0.804
South Sudan	Shokodom	2017	0.279	0.512	0.793
South Sudan	Shokodom	2000-2017	-0.008	0.003	0.015
South Sudan	Sobat	2000	0.277	0.511	0.857
South Sudan	Sobat	2017	0.224	0.413	0.653
South Sudan	Sobat	2000-2017	-0.019	-0.009	0.003
South Sudan	Terkaka	2000	0.245	0.442	0.698
South Sudan	Terkaka	2017	0.238	0.430	0.675
South Sudan	Terkaka	2000-2017	-0.010	-0.003	0.005
South Sudan	Tombura	2000	0.246	0.453	0.715
South Sudan	Tombura	2017	0.248	0.458	0.721
South Sudan	Tombura	2000-2017	-0.003	0.003	0.011
South Sudan	Tonga	2000	0.254	0.488	0.826
South Sudan	Tonga	2017	0.243	0.450	0.716
South Sudan	Tonga	2000-2017	-0.015	-0.002	0.010
South Sudan	Tonj	2000	0.286	0.527	0.862
South Sudan	Tonj	2017	0.239	0.443	0.701
South Sudan	Tonj	2000-2017	-0.016	-0.009	-0.002
South Sudan	Wanjuk	2000	0.308	0.564	0.956
South Sudan	Wanjuk	2017	0.226	0.423	0.646
South Sudan	Wanjuk	2000-2017	-0.028	-0.013	0.002
South Sudan	Warab	2000	0.278	0.518	0.857
South Sudan	Warab	2017	0.223	0.408	0.662
South Sudan	Warab	2000-2017	-0.020	-0.011	-0.004
South Sudan	Wat	2000	0.276	0.513	0.857
South Sudan	Wat	2017	0.236	0.443	0.692
South Sudan	Wat	2000-2017	-0.018	-0.007	0.003
South Sudan	Wau	2000	0.258	0.474	0.790
South Sudan	Wau	2017	0.246	0.457	0.720
South Sudan	Wau	2000-2017	-0.006	0.000	0.006
South Sudan	Yambio	2000	0.246	0.451	0.730
South Sudan	Yambio	2017	0.242	0.460	0.715
South Sudan	Yambio	2000-2017	-0.003	0.004	0.012
South Sudan	Yerol	2000	0.286	0.519	0.829

Table 1: LRI DALYs rate by unit *(continued)*

Country	Unit	year	mean rate	lower rate	upper rate
South Sudan	Yerol	2017	0.251	0.471	0.743
South Sudan	Yerol	2000-2017	-0.012	-0.005	0.001
Swaziland	Dvokodvweni	2000	0.122	0.193	0.295
Swaziland	Dvokodvweni	2017	0.083	0.133	0.207
Swaziland	Dvokodvweni	2000-2017	-0.034	-0.022	-0.010
Swaziland	Ekukhanyeni	2000	0.120	0.190	0.293
Swaziland	Ekukhanyeni	2017	0.083	0.137	0.215
Swaziland	Ekukhanyeni	2000-2017	-0.036	-0.019	-0.002
Swaziland	Gege	2000	0.120	0.187	0.281
Swaziland	Gege	2017	0.085	0.135	0.202
Swaziland	Gege	2000-2017	-0.029	-0.019	-0.007
Swaziland	Hhukwini	2000	0.125	0.192	0.290
Swaziland	Hhukwini	2017	0.085	0.138	0.212
Swaziland	Hhukwini	2000-2017	-0.036	-0.020	-0.004
Swaziland	Hlane	2000	0.119	0.185	0.278
Swaziland	Hlane	2017	0.080	0.131	0.203
Swaziland	Hlane	2000-2017	-0.033	-0.020	-0.006
Swaziland	Hosea	2000	0.130	0.199	0.302
Swaziland	Hosea	2017	0.087	0.140	0.217
Swaziland	Hosea	2000-2017	-0.035	-0.022	-0.006
Swaziland	Kubuta	2000	0.120	0.195	0.292
Swaziland	Kubuta	2017	0.090	0.141	0.218
Swaziland	Kubuta	2000-2017	-0.032	-0.020	-0.006
Swaziland	Kwaluseni	2000	0.101	0.163	0.263
Swaziland	Kwaluseni	2017	0.073	0.122	0.184
Swaziland	Kwaluseni	2000-2017	-0.047	-0.018	0.010
Swaziland	Lamgabhi	2000	0.127	0.193	0.295
Swaziland	Lamgabhi	2017	0.084	0.136	0.208
Swaziland	Lamgabhi	2000-2017	-0.043	-0.021	-0.001
Swaziland	Lobamba	2000	0.123	0.193	0.294
Swaziland	Lobamba	2017	0.090	0.142	0.228
Swaziland	Lobamba	2000-2017	-0.037	-0.018	0.005
Swaziland	Lobamba Lomdzala	2000	0.116	0.184	0.278
Swaziland	Lobamba Lomdzala	2017	0.085	0.133	0.208
Swaziland	Lobamba Lomdzala	2000-2017	-0.035	-0.020	-0.004
Swaziland	Lomahasha	2000	0.114	0.174	0.254
Swaziland	Lomahasha	2017	0.077	0.122	0.185
Swaziland	Lomahasha	2000-2017	-0.033	-0.021	-0.008
Swaziland	Lubuli	2000	0.126	0.187	0.273
Swaziland	Lubuli	2017	0.082	0.132	0.207
Swaziland	Lubuli	2000-2017	-0.034	-0.020	-0.007
Swaziland	Ludzeludze	2000	0.115	0.178	0.258
Swaziland	Ludzeludze	2017	0.081	0.132	0.209
Swaziland	Ludzeludze	2000-2017	-0.037	-0.018	0.002
Swaziland	Lugongolweni	2000	0.113	0.170	0.256
Swaziland	Lugongolweni	2017	0.077	0.123	0.184
Swaziland	Lugongolweni	2000-2017	-0.031	-0.019	-0.008
Swaziland	Madlangempisi	2000	0.118	0.179	0.277
Swaziland	Madlangempisi	2017	0.078	0.124	0.188
Swaziland	Madlangempisi	2000-2017	-0.035	-0.021	-0.006
Swaziland	Mafutseni	2000	0.122	0.190	0.294
Swaziland	Mafutseni	2017	0.083	0.134	0.204
Swaziland	Mafutseni	2000-2017	-0.039	-0.021	-0.005
Swaziland	Mahlangatja	2000	0.120	0.189	0.288
Swaziland	Mahlangatja	2017	0.088	0.135	0.206
Swaziland	Mahlangatja	2000-2017	-0.032	-0.020	-0.009
Swaziland	Mangcongco	2000	0.111	0.174	0.264
Swaziland	Mangcongco	2017	0.081	0.128	0.196
Swaziland	Mangcongco	2000-2017	-0.029	-0.017	-0.003
Swaziland	Manzini North	2000	0.094	0.166	0.276
Swaziland	Manzini North	2017	0.074	0.122	0.201

Table 1: LRI DALYs rate by unit *(continued)*

Country	Unit	year	mean rate	lower rate	upper rate
Swaziland	Manzini North	2000-2017	-0.044	-0.017	0.010
Swaziland	Manzini South	2000	0.089	0.163	0.269
Swaziland	Manzini South	2017	0.066	0.117	0.176
Swaziland	Manzini South	2000-2017	-0.056	-0.019	0.015
Swaziland	Maseyisini	2000	0.125	0.195	0.304
Swaziland	Maseyisini	2017	0.086	0.136	0.204
Swaziland	Maseyisini	2000-2017	-0.035	-0.021	-0.005
Swaziland	Matsanjeni North	2000	0.109	0.169	0.252
Swaziland	Matsanjeni North	2017	0.076	0.120	0.189
Swaziland	Matsanjeni North	2000-2017	-0.032	-0.019	-0.007
Swaziland	Matsanjeni South	2000	0.131	0.204	0.300
Swaziland	Matsanjeni South	2017	0.085	0.140	0.210
Swaziland	Matsanjeni South	2000-2017	-0.033	-0.022	-0.011
Swaziland	Mayiwane	2000	0.112	0.177	0.276
Swaziland	Mayiwane	2017	0.076	0.124	0.196
Swaziland	Mayiwane	2000-2017	-0.038	-0.021	-0.006
Swaziland	Mbabane East	2000	0.103	0.167	0.254
Swaziland	Mbabane East	2017	0.073	0.123	0.192
Swaziland	Mbabane East	2000-2017	-0.051	-0.018	0.019
Swaziland	Mbabane West	2000	0.109	0.174	0.277
Swaziland	Mbabane West	2017	0.080	0.127	0.199
Swaziland	Mbabane West	2000-2017	-0.044	-0.018	0.005
Swaziland	Mbangweni	2000	0.125	0.194	0.299
Swaziland	Mbangweni	2017	0.078	0.132	0.203
Swaziland	Mbangweni	2000-2017	-0.038	-0.024	-0.009
Swaziland	Mhlambanyatsi	2000	0.122	0.188	0.285
Swaziland	Mhlambanyatsi	2017	0.085	0.133	0.201
Swaziland	Mhlambanyatsi	2000-2017	-0.035	-0.020	-0.009
Swaziland	Mhlangatane	2000	0.109	0.176	0.265
Swaziland	Mhlangatane	2017	0.080	0.125	0.195
Swaziland	Mhlangatane	2000-2017	-0.034	-0.021	-0.008
Swaziland	Mhlume	2000	0.114	0.176	0.266
Swaziland	Mhlume	2017	0.078	0.126	0.194
Swaziland	Mhlume	2000-2017	-0.032	-0.020	-0.008
Swaziland	Mkhiweni	2000	0.117	0.188	0.282
Swaziland	Mkhiweni	2017	0.082	0.130	0.200
Swaziland	Mkhiweni	2000-2017	-0.034	-0.022	-0.008
Swaziland	Motjane	2000	0.126	0.198	0.293
Swaziland	Motjane	2017	0.089	0.141	0.217
Swaziland	Motjane	2000-2017	-0.036	-0.020	-0.004
Swaziland	Mphalaleni	2000	0.119	0.189	0.290
Swaziland	Mphalaleni	2017	0.087	0.134	0.205
Swaziland	Mphalaleni	2000-2017	-0.034	-0.020	-0.008
Swaziland	Mpholonjeni	2000	0.116	0.183	0.274
Swaziland	Mpholonjeni	2017	0.079	0.128	0.202
Swaziland	Mpholonjeni	2000-2017	-0.032	-0.021	-0.009
Swaziland	Mthongwaneni	2000	0.121	0.188	0.287
Swaziland	Mthongwaneni	2017	0.085	0.131	0.203
Swaziland	Mthongwaneni	2000-2017	-0.037	-0.021	-0.006
Swaziland	Mtsambama	2000	0.123	0.194	0.298
Swaziland	Mtsambama	2017	0.086	0.137	0.209
Swaziland	Mtsambama	2000-2017	-0.035	-0.021	-0.005
Swaziland	Ndzingeni	2000	0.116	0.180	0.265
Swaziland	Ndzingeni	2017	0.080	0.125	0.198
Swaziland	Ndzingeni	2000-2017	-0.038	-0.022	-0.008
Swaziland	Ngudzeni	2000	0.120	0.193	0.293
Swaziland	Ngudzeni	2017	0.085	0.136	0.212
Swaziland	Ngudzeni	2000-2017	-0.036	-0.021	-0.004
Swaziland	Ngwenpisi	2000	0.123	0.193	0.291
Swaziland	Ngwenpisi	2017	0.088	0.138	0.209
Swaziland	Ngwenpisi	2000-2017	-0.031	-0.019	-0.007

Table 1: LRI DALYs rate by unit *(continued)*

Country	Unit	year	mean rate	lower rate	upper rate
Swaziland	Nhlambeni	2000	0.119	0.181	0.273
Swaziland	Nhlambeni	2017	0.079	0.130	0.190
Swaziland	Nhlambeni	2000-2017	-0.037	-0.020	-0.003
Swaziland	Nkhaba	2000	0.120	0.193	0.295
Swaziland	Nkhaba	2017	0.085	0.139	0.208
Swaziland	Nkhaba	2000-2017	-0.034	-0.019	-0.005
Swaziland	Nkilongo	2000	0.116	0.182	0.268
Swaziland	Nkilongo	2017	0.079	0.127	0.195
Swaziland	Nkilongo	2000-2017	-0.035	-0.021	-0.010
Swaziland	Nkwene	2000	0.117	0.194	0.293
Swaziland	Nkwene	2017	0.086	0.136	0.206
Swaziland	Nkwene	2000-2017	-0.035	-0.022	-0.009
Swaziland	Ntfonjeni	2000	0.107	0.173	0.265
Swaziland	Ntfonjeni	2017	0.074	0.123	0.185
Swaziland	Ntfonjeni	2000-2017	-0.033	-0.020	-0.005
Swaziland	Ntondozi	2000	0.128	0.195	0.296
Swaziland	Ntondozi	2017	0.089	0.139	0.213
Swaziland	Ntondozi	2000-2017	-0.036	-0.020	-0.008
Swaziland	Pigg's Peak	2000	0.113	0.176	0.271
Swaziland	Pigg's Peak	2017	0.078	0.127	0.188
Swaziland	Pigg's Peak	2000-2017	-0.035	-0.021	-0.009
Swaziland	Sandleni	2000	0.126	0.196	0.291
Swaziland	Sandleni	2017	0.090	0.141	0.218
Swaziland	Sandleni	2000-2017	-0.035	-0.020	-0.006
Swaziland	Shiselweni	2000	0.125	0.200	0.307
Swaziland	Shiselweni	2017	0.087	0.140	0.220
Swaziland	Shiselweni	2000-2017	-0.035	-0.021	-0.007
Swaziland	Sigwe	2000	0.122	0.196	0.301
Swaziland	Sigwe	2017	0.081	0.138	0.210
Swaziland	Sigwe	2000-2017	-0.035	-0.021	-0.004
Swaziland	Siphofaneni	2000	0.126	0.198	0.302
Swaziland	Siphofaneni	2017	0.087	0.136	0.208
Swaziland	Siphofaneni	2000-2017	-0.035	-0.022	-0.009
Swaziland	Sithobela	2000	0.131	0.199	0.308
Swaziland	Sithobela	2017	0.086	0.140	0.217
Swaziland	Sithobela	2000-2017	-0.033	-0.021	-0.007
Swaziland	Somntongo	2000	0.121	0.189	0.282
Swaziland	Somntongo	2017	0.082	0.128	0.202
Swaziland	Somntongo	2000-2017	-0.037	-0.023	-0.008
Swaziland	Timpisini	2000	0.108	0.177	0.288
Swaziland	Timpisini	2017	0.076	0.128	0.198
Swaziland	Timpisini	2000-2017	-0.041	-0.019	0.005
Swaziland	Zombodze	2000	0.124	0.200	0.310
Swaziland	Zombodze	2017	0.085	0.138	0.214
Swaziland	Zombodze	2000-2017	-0.038	-0.022	-0.006
Tanzania	Arusha	2000	0.284	0.412	0.594
Tanzania	Arusha	2017	0.115	0.180	0.259
Tanzania	Arusha	2000-2017	-0.060	-0.048	-0.036
Tanzania	Arusha Urban	2000	0.251	0.366	0.525
Tanzania	Arusha Urban	2017	0.110	0.164	0.241
Tanzania	Arusha Urban	2000-2017	-0.065	-0.047	-0.031
Tanzania	Babati	2000	0.263	0.387	0.546
Tanzania	Babati	2017	0.113	0.166	0.240
Tanzania	Babati	2000-2017	-0.056	-0.050	-0.044
Tanzania	Babati Urban	2000	0.244	0.367	0.534
Tanzania	Babati Urban	2017	0.109	0.161	0.237
Tanzania	Babati Urban	2000-2017	-0.063	-0.048	-0.032
Tanzania	Bagamoyo	2000	0.255	0.369	0.520
Tanzania	Bagamoyo	2017	0.115	0.170	0.243
Tanzania	Bagamoyo	2000-2017	-0.052	-0.046	-0.040
Tanzania	Bahi	2000	0.249	0.367	0.516

Table 1: LRI DALYs rate by unit *(continued)*

Country	Unit	year	mean rate	lower rate	upper rate
Tanzania	Bahi	2017	0.110	0.159	0.226
Tanzania	Bahi	2000-2017	-0.054	-0.049	-0.044
Tanzania	Bariadi	2000	0.251	0.361	0.505
Tanzania	Bariadi	2017	0.114	0.167	0.237
Tanzania	Bariadi	2000-2017	-0.052	-0.046	-0.040
Tanzania	Biharamulo	2000	0.302	0.433	0.607
Tanzania	Biharamulo	2017	0.119	0.173	0.244
Tanzania	Biharamulo	2000-2017	-0.056	-0.050	-0.045
Tanzania	Buhigwe	2000	0.300	0.429	0.614
Tanzania	Buhigwe	2017	0.120	0.172	0.238
Tanzania	Buhigwe	2000-2017	-0.057	-0.050	-0.042
Tanzania	Bukoba Rural	2000	0.280	0.411	0.575
Tanzania	Bukoba Rural	2017	0.131	0.189	0.267
Tanzania	Bukoba Rural	2000-2017	-0.050	-0.043	-0.035
Tanzania	Bukoba Urban	2000	0.268	0.400	0.588
Tanzania	Bukoba Urban	2017	0.121	0.197	0.301
Tanzania	Bukoba Urban	2000-2017	-0.066	-0.039	-0.013
Tanzania	Bukombe	2000	0.269	0.383	0.551
Tanzania	Bukombe	2017	0.111	0.162	0.232
Tanzania	Bukombe	2000-2017	-0.059	-0.049	-0.041
Tanzania	Bunda	2000	0.239	0.346	0.487
Tanzania	Bunda	2017	0.114	0.162	0.230
Tanzania	Bunda	2000-2017	-0.052	-0.045	-0.037
Tanzania	Busega	2000	0.244	0.348	0.494
Tanzania	Busega	2017	0.110	0.162	0.230
Tanzania	Busega	2000-2017	-0.052	-0.045	-0.037
Tanzania	Butiama	2000	0.256	0.364	0.517
Tanzania	Butiama	2017	0.120	0.172	0.242
Tanzania	Butiama	2000-2017	-0.050	-0.044	-0.037
Tanzania	Chake	2000	0.247	0.375	0.547
Tanzania	Chake	2017	0.112	0.165	0.238
Tanzania	Chake	2000-2017	-0.063	-0.047	-0.031
Tanzania	Chamwino	2000	0.258	0.377	0.536
Tanzania	Chamwino	2017	0.107	0.161	0.230
Tanzania	Chamwino	2000-2017	-0.056	-0.050	-0.046
Tanzania	Chato	2000	0.259	0.377	0.534
Tanzania	Chato	2017	0.112	0.167	0.238
Tanzania	Chato	2000-2017	-0.053	-0.046	-0.039
Tanzania	Chemba	2000	0.252	0.377	0.535
Tanzania	Chemba	2017	0.110	0.163	0.235
Tanzania	Chemba	2000-2017	-0.054	-0.050	-0.045
Tanzania	Chunya	2000	0.266	0.381	0.535
Tanzania	Chunya	2017	0.108	0.159	0.225
Tanzania	Chunya	2000-2017	-0.056	-0.051	-0.046
Tanzania	Dodoma Urban	2000	0.254	0.360	0.494
Tanzania	Dodoma Urban	2017	0.106	0.155	0.226
Tanzania	Dodoma Urban	2000-2017	-0.061	-0.049	-0.038
Tanzania	Gairo	2000	0.266	0.390	0.549
Tanzania	Gairo	2017	0.113	0.165	0.237
Tanzania	Gairo	2000-2017	-0.059	-0.051	-0.044
Tanzania	Geita	2000	0.257	0.373	0.534
Tanzania	Geita	2017	0.115	0.168	0.238
Tanzania	Geita	2000-2017	-0.053	-0.046	-0.039
Tanzania	Hai	2000	0.268	0.402	0.568
Tanzania	Hai	2017	0.120	0.184	0.265
Tanzania	Hai	2000-2017	-0.056	-0.045	-0.034
Tanzania	Hanang	2000	0.263	0.388	0.550
Tanzania	Hanang	2017	0.115	0.170	0.247
Tanzania	Hanang	2000-2017	-0.055	-0.048	-0.042
Tanzania	Handeni	2000	0.284	0.405	0.570
Tanzania	Handeni	2017	0.122	0.181	0.263

Table 1: LRI DALYs rate by unit *(continued)*

Country	Unit	year	mean rate	lower rate	upper rate
Tanzania	Handeni	2000-2017	-0.053	-0.048	-0.042
Tanzania	Handeni Township Authority	2000	0.258	0.376	0.544
Tanzania	Handeni Township Authority	2017	0.111	0.167	0.257
Tanzania	Handeni Township Authority	2000-2017	-0.063	-0.047	-0.033
Tanzania	Igunga	2000	0.250	0.359	0.504
Tanzania	Igunga	2017	0.116	0.168	0.239
Tanzania	Igunga	2000-2017	-0.050	-0.045	-0.039
Tanzania	Ikungi	2000	0.249	0.360	0.513
Tanzania	Ikungi	2017	0.110	0.165	0.239
Tanzania	Ikungi	2000-2017	-0.050	-0.046	-0.042
Tanzania	Ilala	2000	0.257	0.383	0.551
Tanzania	Ilala	2017	0.119	0.178	0.253
Tanzania	Ilala	2000-2017	-0.059	-0.044	-0.032
Tanzania	Ileje	2000	0.347	0.491	0.680
Tanzania	Ileje	2017	0.120	0.171	0.238
Tanzania	Ileje	2000-2017	-0.068	-0.061	-0.054
Tanzania	Ilemela	2000	0.248	0.370	0.548
Tanzania	Ilemela	2017	0.114	0.169	0.248
Tanzania	Ilemela	2000-2017	-0.062	-0.046	-0.027
Tanzania	Iramba	2000	0.244	0.355	0.500
Tanzania	Iramba	2017	0.113	0.165	0.237
Tanzania	Iramba	2000-2017	-0.050	-0.045	-0.039
Tanzania	Iringa Rural	2000	0.285	0.409	0.581
Tanzania	Iringa Rural	2017	0.116	0.170	0.244
Tanzania	Iringa Rural	2000-2017	-0.056	-0.051	-0.045
Tanzania	Iringa Urban	2000	0.300	0.432	0.629
Tanzania	Iringa Urban	2017	0.107	0.167	0.250
Tanzania	Iringa Urban	2000-2017	-0.078	-0.056	-0.035
Tanzania	Itilima	2000	0.252	0.367	0.511
Tanzania	Itilima	2017	0.116	0.169	0.236
Tanzania	Itilima	2000-2017	-0.052	-0.046	-0.040
Tanzania	Kahama	2000	0.245	0.351	0.499
Tanzania	Kahama	2017	0.110	0.157	0.224
Tanzania	Kahama	2000-2017	-0.051	-0.047	-0.041
Tanzania	Kahama Township Authority	2000	0.242	0.354	0.516
Tanzania	Kahama Township Authority	2017	0.110	0.164	0.235
Tanzania	Kahama Township Authority	2000-2017	-0.057	-0.044	-0.029
Tanzania	Kakonko	2000	0.331	0.468	0.657
Tanzania	Kakonko	2017	0.125	0.175	0.245
Tanzania	Kakonko	2000-2017	-0.062	-0.055	-0.047
Tanzania	Kalambo	2000	0.308	0.443	0.623
Tanzania	Kalambo	2017	0.124	0.181	0.259
Tanzania	Kalambo	2000-2017	-0.054	-0.049	-0.043
Tanzania	Kaliua	2000	0.257	0.362	0.506
Tanzania	Kaliua	2017	0.109	0.158	0.222
Tanzania	Kaliua	2000-2017	-0.053	-0.048	-0.044
Tanzania	Karagwe	2000	0.339	0.492	0.684
Tanzania	Karagwe	2017	0.136	0.194	0.275
Tanzania	Karagwe	2000-2017	-0.056	-0.049	-0.041
Tanzania	Karatu	2000	0.251	0.380	0.536
Tanzania	Karatu	2017	0.111	0.166	0.239
Tanzania	Karatu	2000-2017	-0.058	-0.049	-0.043
Tanzania	Kaskazini 'A'	2000	0.243	0.363	0.517
Tanzania	Kaskazini 'A'	2017	0.114	0.172	0.243
Tanzania	Kaskazini 'A'	2000-2017	-0.057	-0.044	-0.031
Tanzania	Kaskazini 'B'	2000	0.256	0.386	0.556
Tanzania	Kaskazini 'B'	2017	0.122	0.178	0.247
Tanzania	Kaskazini 'B'	2000-2017	-0.061	-0.045	-0.029
Tanzania	Kasulu	2000	0.304	0.432	0.613
Tanzania	Kasulu	2017	0.115	0.165	0.228
Tanzania	Kasulu	2000-2017	-0.059	-0.053	-0.047

Table 1: LRI DALYs rate by unit *(continued)*

Country	Unit	year	mean rate	lower rate	upper rate
Tanzania	Kasulu Township Authority	2000	0.273	0.404	0.591
Tanzania	Kasulu Township Authority	2017	0.112	0.165	0.232
Tanzania	Kasulu Township Authority	2000-2017	-0.062	-0.050	-0.037
Tanzania	Kati	2000	0.249	0.359	0.500
Tanzania	Kati	2017	0.111	0.165	0.235
Tanzania	Kati	2000-2017	-0.058	-0.045	-0.033
Tanzania	Kibaha	2000	0.258	0.376	0.547
Tanzania	Kibaha	2017	0.119	0.172	0.249
Tanzania	Kibaha	2000-2017	-0.055	-0.045	-0.035
Tanzania	Kibaha Urban	2000	0.257	0.385	0.540
Tanzania	Kibaha Urban	2017	0.124	0.178	0.254
Tanzania	Kibaha Urban	2000-2017	-0.058	-0.045	-0.030
Tanzania	Kibondo	2000	0.310	0.445	0.629
Tanzania	Kibondo	2017	0.115	0.164	0.230
Tanzania	Kibondo	2000-2017	-0.062	-0.055	-0.049
Tanzania	Kigoma Rural	2000	0.283	0.413	0.596
Tanzania	Kigoma Rural	2017	0.122	0.179	0.252
Tanzania	Kigoma Rural	2000-2017	-0.055	-0.047	-0.038
Tanzania	Kigoma Urban	2000	0.272	0.427	0.632
Tanzania	Kigoma Urban	2017	0.118	0.186	0.277
Tanzania	Kigoma Urban	2000-2017	-0.068	-0.044	-0.016
Tanzania	Kilindi	2000	0.267	0.392	0.552
Tanzania	Kilindi	2017	0.112	0.164	0.237
Tanzania	Kilindi	2000-2017	-0.056	-0.051	-0.046
Tanzania	Kilolo	2000	0.287	0.418	0.592
Tanzania	Kilolo	2017	0.116	0.170	0.243
Tanzania	Kilolo	2000-2017	-0.059	-0.052	-0.046
Tanzania	Kilombero	2000	0.265	0.383	0.539
Tanzania	Kilombero	2017	0.110	0.162	0.229
Tanzania	Kilombero	2000-2017	-0.057	-0.051	-0.045
Tanzania	Kilosa	2000	0.258	0.375	0.532
Tanzania	Kilosa	2017	0.110	0.161	0.232
Tanzania	Kilosa	2000-2017	-0.055	-0.050	-0.045
Tanzania	Kilwa	2000	0.260	0.378	0.534
Tanzania	Kilwa	2017	0.111	0.165	0.239
Tanzania	Kilwa	2000-2017	-0.054	-0.049	-0.044
Tanzania	Kinondoni	2000	0.264	0.386	0.546
Tanzania	Kinondoni	2017	0.118	0.177	0.260
Tanzania	Kinondoni	2000-2017	-0.059	-0.045	-0.030
Tanzania	Kisarawe	2000	0.257	0.367	0.517
Tanzania	Kisarawe	2017	0.118	0.170	0.241
Tanzania	Kisarawe	2000-2017	-0.052	-0.045	-0.037
Tanzania	Kishapu	2000	0.263	0.380	0.540
Tanzania	Kishapu	2017	0.120	0.177	0.250
Tanzania	Kishapu	2000-2017	-0.051	-0.045	-0.039
Tanzania	Kiteto	2000	0.267	0.397	0.566
Tanzania	Kiteto	2017	0.110	0.164	0.237
Tanzania	Kiteto	2000-2017	-0.058	-0.053	-0.046
Tanzania	Kondoa	2000	0.266	0.385	0.544
Tanzania	Kondoa	2017	0.114	0.167	0.240
Tanzania	Kondoa	2000-2017	-0.055	-0.049	-0.043
Tanzania	Kongwa	2000	0.263	0.385	0.544
Tanzania	Kongwa	2017	0.112	0.164	0.235
Tanzania	Kongwa	2000-2017	-0.056	-0.050	-0.044
Tanzania	Korogwe	2000	0.268	0.391	0.553
Tanzania	Korogwe	2017	0.117	0.172	0.246
Tanzania	Korogwe	2000-2017	-0.055	-0.048	-0.041
Tanzania	Korogwe Township Authority	2000	0.278	0.423	0.591
Tanzania	Korogwe Township Authority	2017	0.121	0.189	0.284
Tanzania	Korogwe Township Authority	2000-2017	-0.068	-0.047	-0.027
Tanzania	Kusini	2000	0.246	0.368	0.531

Table 1: LRI DALYs rate by unit *(continued)*

Country	Unit	year	mean rate	lower rate	upper rate
Tanzania	Kusini	2017	0.119	0.174	0.249
Tanzania	Kusini	2000-2017	-0.058	-0.044	-0.030
Tanzania	Kwimba	2000	0.262	0.384	0.545
Tanzania	Kwimba	2017	0.121	0.173	0.244
Tanzania	Kwimba	2000-2017	-0.052	-0.046	-0.040
Tanzania	Kyela	2000	0.333	0.483	0.678
Tanzania	Kyela	2017	0.115	0.166	0.235
Tanzania	Kyela	2000-2017	-0.078	-0.065	-0.053
Tanzania	Kyerwa	2000	0.365	0.519	0.722
Tanzania	Kyerwa	2017	0.139	0.193	0.272
Tanzania	Kyerwa	2000-2017	-0.058	-0.051	-0.044
Tanzania	Lake Eyasi	2000	0.261	0.393	0.562
Tanzania	Lake Eyasi	2000	0.247	0.361	0.517
Tanzania	Lake Eyasi	2017	0.116	0.176	0.264
Tanzania	Lake Eyasi	2017	0.110	0.163	0.230
Tanzania	Lake Eyasi	2000-2017	-0.060	-0.048	-0.032
Tanzania	Lake Eyasi	2000-2017	-0.063	-0.047	-0.029
Tanzania	Lake Manyara	2000	0.242	0.369	0.535
Tanzania	Lake Manyara	2000	0.247	0.367	0.528
Tanzania	Lake Manyara	2017	0.105	0.159	0.241
Tanzania	Lake Manyara	2017	0.107	0.158	0.233
Tanzania	Lake Manyara	2000-2017	-0.070	-0.049	-0.028
Tanzania	Lake Manyara	2000-2017	-0.068	-0.049	-0.032
Tanzania	Lake Rukwa	2000	0.292	0.431	0.619
Tanzania	Lake Rukwa	2000	0.260	0.383	0.551
Tanzania	Lake Rukwa	2000	0.266	0.380	0.541
Tanzania	Lake Rukwa	2017	0.106	0.157	0.223
Tanzania	Lake Rukwa	2017	0.109	0.161	0.232
Tanzania	Lake Rukwa	2017	0.112	0.160	0.230
Tanzania	Lake Rukwa	2000-2017	-0.066	-0.052	-0.037
Tanzania	Lake Rukwa	2000-2017	-0.082	-0.061	-0.041
Tanzania	Lake Rukwa	2000-2017	-0.062	-0.052	-0.042
Tanzania	Lake Tanganyika	2000	0.242	0.357	0.518
Tanzania	Lake Tanganyika	2000	0.272	0.392	0.558
Tanzania	Lake Tanganyika	2000	0.269	0.388	0.555
Tanzania	Lake Tanganyika	2017	0.110	0.158	0.226
Tanzania	Lake Tanganyika	2017	0.112	0.160	0.223
Tanzania	Lake Tanganyika	2017	0.117	0.171	0.243
Tanzania	Lake Tanganyika	2000-2017	-0.064	-0.049	-0.033
Tanzania	Lake Tanganyika	2000-2017	-0.061	-0.052	-0.043
Tanzania	Lake Tanganyika	2000-2017	-0.056	-0.046	-0.036
Tanzania	Lake Victoria	2000	0.233	0.341	0.492
Tanzania	Lake Victoria	2000	0.274	0.390	0.554
Tanzania	Lake Victoria	2000	0.252	0.364	0.517
Tanzania	Lake Victoria	2000	0.251	0.369	0.523
Tanzania	Lake Victoria	2017	0.104	0.158	0.227
Tanzania	Lake Victoria	2017	0.121	0.178	0.256
Tanzania	Lake Victoria	2017	0.117	0.167	0.233
Tanzania	Lake Victoria	2017	0.119	0.169	0.243
Tanzania	Lake Victoria	2000-2017	-0.052	-0.045	-0.037
Tanzania	Lake Victoria	2000-2017	-0.052	-0.045	-0.037
Tanzania	Lake Victoria	2000-2017	-0.060	-0.045	-0.031
Tanzania	Lake Victoria	2000-2017	-0.053	-0.044	-0.033
Tanzania	Lindi Rural	2000	0.284	0.412	0.584
Tanzania	Lindi Rural	2017	0.115	0.170	0.241
Tanzania	Lindi Rural	2000-2017	-0.057	-0.050	-0.042
Tanzania	Lindi Urban	2000	0.288	0.411	0.600
Tanzania	Lindi Urban	2017	0.118	0.173	0.253
Tanzania	Lindi Urban	2000-2017	-0.066	-0.050	-0.034
Tanzania	Liwale	2000	0.275	0.398	0.561
Tanzania	Liwale	2017	0.111	0.163	0.238

Table 1: LRI DALYs rate by unit *(continued)*

Country	Unit	year	mean rate	lower rate	upper rate
Tanzania	Liwale	2000-2017	-0.060	-0.052	-0.043
Tanzania	Longido	2000	0.259	0.370	0.520
Tanzania	Longido	2017	0.112	0.168	0.241
Tanzania	Longido	2000-2017	-0.051	-0.045	-0.040
Tanzania	Ludewa	2000	0.304	0.437	0.609
Tanzania	Ludewa	2017	0.119	0.174	0.246
Tanzania	Ludewa	2000-2017	-0.059	-0.052	-0.044
Tanzania	Lushoto	2000	0.276	0.395	0.563
Tanzania	Lushoto	2017	0.114	0.169	0.243
Tanzania	Lushoto	2000-2017	-0.055	-0.049	-0.043
Tanzania	Mafia	2000	0.270	0.399	0.585
Tanzania	Mafia	2017	0.121	0.178	0.248
Tanzania	Mafia	2000-2017	-0.060	-0.048	-0.036
Tanzania	Mafinga Township Authority	2000	0.274	0.401	0.577
Tanzania	Mafinga Township Authority	2017	0.113	0.168	0.238
Tanzania	Mafinga Township Authority	2000-2017	-0.067	-0.049	-0.030
Tanzania	Magharibi	2000	0.248	0.379	0.567
Tanzania	Magharibi	2017	0.117	0.174	0.257
Tanzania	Magharibi	2000-2017	-0.069	-0.047	-0.023
Tanzania	Magu	2000	0.247	0.363	0.512
Tanzania	Magu	2017	0.115	0.170	0.240
Tanzania	Magu	2000-2017	-0.052	-0.044	-0.037
Tanzania	Makambako Township Authority	2000	0.299	0.453	0.670
Tanzania	Makambako Township Authority	2017	0.112	0.166	0.247
Tanzania	Makambako Township Authority	2000-2017	-0.075	-0.057	-0.039
Tanzania	Makete	2000	0.313	0.458	0.651
Tanzania	Makete	2017	0.124	0.179	0.257
Tanzania	Makete	2000-2017	-0.061	-0.053	-0.045
Tanzania	Manyoni	2000	0.252	0.362	0.507
Tanzania	Manyoni	2017	0.109	0.160	0.230
Tanzania	Manyoni	2000-2017	-0.053	-0.048	-0.043
Tanzania	Masasi	2000	0.284	0.404	0.569
Tanzania	Masasi	2017	0.114	0.166	0.238
Tanzania	Masasi	2000-2017	-0.058	-0.050	-0.041
Tanzania	Masasi Township Authority	2000	0.271	0.398	0.567
Tanzania	Masasi Township Authority	2017	0.113	0.167	0.245
Tanzania	Masasi Township Authority	2000-2017	-0.063	-0.049	-0.035
Tanzania	Maswa	2000	0.260	0.378	0.537
Tanzania	Maswa	2017	0.118	0.175	0.247
Tanzania	Maswa	2000-2017	-0.052	-0.046	-0.041
Tanzania	Mbarali	2000	0.293	0.428	0.607
Tanzania	Mbarali	2017	0.110	0.160	0.222
Tanzania	Mbarali	2000-2017	-0.065	-0.058	-0.051
Tanzania	Mbeya Rural	2000	0.338	0.479	0.679
Tanzania	Mbeya Rural	2017	0.122	0.176	0.250
Tanzania	Mbeya Rural	2000-2017	-0.068	-0.060	-0.051
Tanzania	Mbeya Urban	2000	0.341	0.502	0.748
Tanzania	Mbeya Urban	2017	0.121	0.182	0.272
Tanzania	Mbeya Urban	2000-2017	-0.078	-0.063	-0.046
Tanzania	Mbinga	2000	0.309	0.444	0.634
Tanzania	Mbinga	2017	0.110	0.165	0.231
Tanzania	Mbinga	2000-2017	-0.062	-0.055	-0.046
Tanzania	Mbogwe	2000	0.241	0.351	0.499
Tanzania	Mbogwe	2017	0.108	0.155	0.218
Tanzania	Mbogwe	2000-2017	-0.054	-0.047	-0.040
Tanzania	Mbozi	2000	0.312	0.460	0.646
Tanzania	Mbozi	2017	0.117	0.165	0.232
Tanzania	Mbozi	2000-2017	-0.067	-0.060	-0.053
Tanzania	Mbulu	2000	0.284	0.413	0.585
Tanzania	Mbulu	2017	0.119	0.179	0.259
Tanzania	Mbulu	2000-2017	-0.055	-0.049	-0.044

Table 1: LRI DALYs rate by unit *(continued)*

Country	Unit	year	mean rate	lower rate	upper rate
Tanzania	Meatu	2000	0.251	0.366	0.513
Tanzania	Meatu	2017	0.117	0.171	0.244
Tanzania	Meatu	2000-2017	-0.051	-0.045	-0.040
Tanzania	Meru	2000	0.267	0.394	0.556
Tanzania	Meru	2017	0.117	0.176	0.250
Tanzania	Meru	2000-2017	-0.057	-0.047	-0.037
Tanzania	Micheweni	2000	0.259	0.379	0.547
Tanzania	Micheweni	2017	0.119	0.174	0.252
Tanzania	Micheweni	2000-2017	-0.060	-0.046	-0.031
Tanzania	Missenyi	2000	0.316	0.458	0.648
Tanzania	Missenyi	2017	0.133	0.193	0.276
Tanzania	Missenyi	2000-2017	-0.054	-0.047	-0.040
Tanzania	Misungwi	2000	0.265	0.387	0.547
Tanzania	Misungwi	2017	0.119	0.176	0.247
Tanzania	Misungwi	2000-2017	-0.052	-0.046	-0.040
Tanzania	Mjini	2000	0.250	0.391	0.579
Tanzania	Mjini	2017	0.114	0.176	0.262
Tanzania	Mjini	2000-2017	-0.073	-0.048	-0.019
Tanzania	Mkalama	2000	0.252	0.366	0.519
Tanzania	Mkalama	2017	0.115	0.168	0.241
Tanzania	Mkalama	2000-2017	-0.051	-0.045	-0.039
Tanzania	Mkinga	2000	0.265	0.375	0.533
Tanzania	Mkinga	2017	0.117	0.172	0.242
Tanzania	Mkinga	2000-2017	-0.052	-0.045	-0.036
Tanzania	Mkoani	2000	0.262	0.400	0.555
Tanzania	Mkoani	2017	0.122	0.181	0.254
Tanzania	Mkoani	2000-2017	-0.059	-0.046	-0.032
Tanzania	Mkuranga	2000	0.273	0.396	0.559
Tanzania	Mkuranga	2017	0.124	0.182	0.260
Tanzania	Mkuranga	2000-2017	-0.054	-0.045	-0.036
Tanzania	Mlele	2000	0.278	0.399	0.555
Tanzania	Mlele	2017	0.112	0.161	0.226
Tanzania	Mlele	2000-2017	-0.058	-0.054	-0.049
Tanzania	Momba	2000	0.295	0.421	0.593
Tanzania	Momba	2017	0.110	0.158	0.224
Tanzania	Momba	2000-2017	-0.062	-0.057	-0.051
Tanzania	Monduli	2000	0.255	0.369	0.521
Tanzania	Monduli	2017	0.114	0.165	0.236
Tanzania	Monduli	2000-2017	-0.054	-0.047	-0.040
Tanzania	Morogoro Rural	2000	0.263	0.377	0.522
Tanzania	Morogoro Rural	2017	0.113	0.163	0.229
Tanzania	Morogoro Rural	2000-2017	-0.055	-0.049	-0.043
Tanzania	Morogoro Urban	2000	0.255	0.382	0.552
Tanzania	Morogoro Urban	2017	0.115	0.174	0.256
Tanzania	Morogoro Urban	2000-2017	-0.067	-0.045	-0.025
Tanzania	Moshi Rural	2000	0.262	0.386	0.561
Tanzania	Moshi Rural	2017	0.118	0.176	0.253
Tanzania	Moshi Rural	2000-2017	-0.053	-0.045	-0.037
Tanzania	Moshi Urban	2000	0.241	0.386	0.591
Tanzania	Moshi Urban	2017	0.112	0.180	0.266
Tanzania	Moshi Urban	2000-2017	-0.072	-0.045	-0.019
Tanzania	Mpanda	2000	0.294	0.426	0.596
Tanzania	Mpanda	2017	0.112	0.164	0.229
Tanzania	Mpanda	2000-2017	-0.060	-0.056	-0.052
Tanzania	Mpanda Urban	2000	0.234	0.376	0.554
Tanzania	Mpanda Urban	2017	0.102	0.165	0.251
Tanzania	Mpanda Urban	2000-2017	-0.079	-0.050	-0.022
Tanzania	Mpwapwa	2000	0.266	0.391	0.553
Tanzania	Mpwapwa	2017	0.111	0.165	0.233
Tanzania	Mpwapwa	2000-2017	-0.056	-0.051	-0.046
Tanzania	Mtwara Rural	2000	0.284	0.413	0.578

Table 1: LRI DALYs rate by unit *(continued)*

Country	Unit	year	mean rate	lower rate	upper rate
Tanzania	Mtwara Rural	2017	0.115	0.170	0.244
Tanzania	Mtwara Rural	2000-2017	-0.058	-0.051	-0.044
Tanzania	Mtwara Urban	2000	0.271	0.434	0.635
Tanzania	Mtwara Urban	2017	0.118	0.192	0.277
Tanzania	Mtwara Urban	2000-2017	-0.071	-0.047	-0.023
Tanzania	Mufindi	2000	0.281	0.406	0.569
Tanzania	Mufindi	2017	0.118	0.172	0.244
Tanzania	Mufindi	2000-2017	-0.056	-0.049	-0.043
Tanzania	Muheza	2000	0.250	0.366	0.518
Tanzania	Muheza	2017	0.114	0.165	0.237
Tanzania	Muheza	2000-2017	-0.057	-0.047	-0.038
Tanzania	Muleba	2000	0.273	0.400	0.568
Tanzania	Muleba	2017	0.123	0.179	0.255
Tanzania	Muleba	2000-2017	-0.052	-0.045	-0.038
Tanzania	Musoma Rural	2000	0.250	0.361	0.516
Tanzania	Musoma Rural	2017	0.117	0.169	0.238
Tanzania	Musoma Rural	2000-2017	-0.053	-0.044	-0.035
Tanzania	Musoma Urban	2000	0.221	0.350	0.531
Tanzania	Musoma Urban	2017	0.103	0.163	0.245
Tanzania	Musoma Urban	2000-2017	-0.076	-0.044	-0.008
Tanzania	Mvomero	2000	0.255	0.374	0.527
Tanzania	Mvomero	2017	0.111	0.162	0.231
Tanzania	Mvomero	2000-2017	-0.055	-0.049	-0.043
Tanzania	Mwanga	2000	0.254	0.382	0.548
Tanzania	Mwanga	2017	0.113	0.172	0.251
Tanzania	Mwanga	2000-2017	-0.054	-0.046	-0.037
Tanzania	Nachingwea	2000	0.276	0.403	0.574
Tanzania	Nachingwea	2017	0.113	0.166	0.239
Tanzania	Nachingwea	2000-2017	-0.059	-0.050	-0.042
Tanzania	Namtumbo	2000	0.279	0.400	0.573
Tanzania	Namtumbo	2017	0.110	0.163	0.232
Tanzania	Namtumbo	2000-2017	-0.058	-0.051	-0.045
Tanzania	Nanyumbu	2000	0.284	0.405	0.586
Tanzania	Nanyumbu	2017	0.112	0.167	0.239
Tanzania	Nanyumbu	2000-2017	-0.058	-0.050	-0.043
Tanzania	Newala	2000	0.273	0.395	0.561
Tanzania	Newala	2017	0.112	0.164	0.237
Tanzania	Newala	2000-2017	-0.059	-0.050	-0.040
Tanzania	Ngara	2000	0.400	0.565	0.789
Tanzania	Ngara	2017	0.153	0.214	0.299
Tanzania	Ngara	2000-2017	-0.060	-0.053	-0.047
Tanzania	Ngorongoro	2000	0.266	0.379	0.537
Tanzania	Ngorongoro	2017	0.113	0.166	0.236
Tanzania	Ngorongoro	2000-2017	-0.053	-0.049	-0.045
Tanzania	Njombe	2000	0.281	0.407	0.579
Tanzania	Njombe	2017	0.118	0.171	0.245
Tanzania	Njombe	2000-2017	-0.058	-0.050	-0.041
Tanzania	Njombe Urban	2000	0.315	0.458	0.655
Tanzania	Njombe Urban	2017	0.124	0.181	0.262
Tanzania	Njombe Urban	2000-2017	-0.067	-0.053	-0.040
Tanzania	Nkasi	2000	0.298	0.428	0.594
Tanzania	Nkasi	2017	0.115	0.167	0.236
Tanzania	Nkasi	2000-2017	-0.059	-0.054	-0.049
Tanzania	Nyamagana	2000	0.249	0.375	0.537
Tanzania	Nyamagana	2017	0.115	0.174	0.250
Tanzania	Nyamagana	2000-2017	-0.063	-0.044	-0.026
Tanzania	Nyang'wale	2000	0.243	0.351	0.505
Tanzania	Nyang'wale	2017	0.109	0.156	0.218
Tanzania	Nyang'wale	2000-2017	-0.054	-0.047	-0.040
Tanzania	Nyasa	2000	0.315	0.444	0.624
Tanzania	Nyasa	2017	0.112	0.163	0.228

Table 1: LRI DALYs rate by unit *(continued)*

Country	Unit	year	mean rate	lower rate	upper rate
Tanzania	Nyasa	2000-2017	-0.065	-0.057	-0.049
Tanzania	Nzega	2000	0.240	0.348	0.492
Tanzania	Nzega	2017	0.110	0.161	0.229
Tanzania	Nzega	2000-2017	-0.052	-0.045	-0.040
Tanzania	Pangani	2000	0.250	0.372	0.516
Tanzania	Pangani	2017	0.121	0.171	0.242
Tanzania	Pangani	2000-2017	-0.055	-0.045	-0.036
Tanzania	Rombo	2000	0.276	0.403	0.577
Tanzania	Rombo	2017	0.125	0.182	0.265
Tanzania	Rombo	2000-2017	-0.053	-0.045	-0.036
Tanzania	Rorya	2000	0.259	0.370	0.514
Tanzania	Rorya	2017	0.120	0.171	0.242
Tanzania	Rorya	2000-2017	-0.051	-0.044	-0.038
Tanzania	Ruangwa	2000	0.287	0.416	0.591
Tanzania	Ruangwa	2017	0.117	0.172	0.242
Tanzania	Ruangwa	2000-2017	-0.058	-0.050	-0.041
Tanzania	Rufiji	2000	0.252	0.366	0.517
Tanzania	Rufiji	2017	0.112	0.162	0.232
Tanzania	Rufiji	2000-2017	-0.053	-0.047	-0.042
Tanzania	Rungwe	2000	0.324	0.479	0.672
Tanzania	Rungwe	2017	0.119	0.171	0.240
Tanzania	Rungwe	2000-2017	-0.071	-0.063	-0.056
Tanzania	Same	2000	0.279	0.405	0.570
Tanzania	Same	2017	0.117	0.177	0.258
Tanzania	Same	2000-2017	-0.055	-0.048	-0.042
Tanzania	Sengerema	2000	0.259	0.373	0.532
Tanzania	Sengerema	2017	0.116	0.170	0.241
Tanzania	Sengerema	2000-2017	-0.052	-0.045	-0.039
Tanzania	Serengeti	2000	0.252	0.363	0.505
Tanzania	Serengeti	2017	0.116	0.168	0.238
Tanzania	Serengeti	2000-2017	-0.052	-0.045	-0.040
Tanzania	Shinyanga Rural	2000	0.260	0.379	0.538
Tanzania	Shinyanga Rural	2017	0.117	0.172	0.243
Tanzania	Shinyanga Rural	2000-2017	-0.051	-0.046	-0.041
Tanzania	Shinyanga Urban	2000	0.248	0.369	0.530
Tanzania	Shinyanga Urban	2017	0.113	0.170	0.246
Tanzania	Shinyanga Urban	2000-2017	-0.059	-0.046	-0.031
Tanzania	Siha	2000	0.276	0.404	0.575
Tanzania	Siha	2017	0.121	0.184	0.267
Tanzania	Siha	2000-2017	-0.057	-0.046	-0.037
Tanzania	Sikonge	2000	0.251	0.355	0.497
Tanzania	Sikonge	2017	0.107	0.160	0.228
Tanzania	Sikonge	2000-2017	-0.051	-0.046	-0.042
Tanzania	Simanjiro	2000	0.265	0.382	0.541
Tanzania	Simanjiro	2017	0.116	0.170	0.244
Tanzania	Simanjiro	2000-2017	-0.054	-0.047	-0.041
Tanzania	Singida Rural	2000	0.250	0.369	0.523
Tanzania	Singida Rural	2017	0.114	0.169	0.242
Tanzania	Singida Rural	2000-2017	-0.052	-0.046	-0.040
Tanzania	Singida Urban	2000	0.264	0.389	0.546
Tanzania	Singida Urban	2017	0.114	0.176	0.252
Tanzania	Singida Urban	2000-2017	-0.060	-0.046	-0.033
Tanzania	Songea Rural	2000	0.283	0.407	0.575
Tanzania	Songea Rural	2017	0.114	0.165	0.232
Tanzania	Songea Rural	2000-2017	-0.059	-0.052	-0.044
Tanzania	Songea Urban	2000	0.330	0.500	0.751
Tanzania	Songea Urban	2017	0.114	0.174	0.261
Tanzania	Songea Urban	2000-2017	-0.086	-0.061	-0.033
Tanzania	Sumbawanga Rural	2000	0.303	0.437	0.609
Tanzania	Sumbawanga Rural	2017	0.114	0.165	0.236
Tanzania	Sumbawanga Rural	2000-2017	-0.061	-0.056	-0.051

Table 1: LRI DALYs rate by unit *(continued)*

Country	Unit	year	mean rate	lower rate	upper rate
Tanzania	Sumbawanga Urban	2000	0.329	0.481	0.682
Tanzania	Sumbawanga Urban	2017	0.123	0.178	0.258
Tanzania	Sumbawanga Urban	2000-2017	-0.073	-0.057	-0.043
Tanzania	Tabora Urban	2000	0.226	0.345	0.497
Tanzania	Tabora Urban	2017	0.105	0.159	0.232
Tanzania	Tabora Urban	2000-2017	-0.063	-0.045	-0.025
Tanzania	Tandahimba	2000	0.282	0.406	0.579
Tanzania	Tandahimba	2017	0.114	0.167	0.242
Tanzania	Tandahimba	2000-2017	-0.059	-0.050	-0.042
Tanzania	Tanga	2000	0.239	0.366	0.577
Tanzania	Tanga	2017	0.110	0.174	0.258
Tanzania	Tanga	2000-2017	-0.069	-0.043	-0.017
Tanzania	Tarime	2000	0.304	0.420	0.588
Tanzania	Tarime	2017	0.130	0.190	0.264
Tanzania	Tarime	2000-2017	-0.054	-0.045	-0.037
Tanzania	Temeke	2000	0.246	0.380	0.565
Tanzania	Temeke	2017	0.119	0.176	0.256
Tanzania	Temeke	2000-2017	-0.061	-0.045	-0.028
Tanzania	Tunduma	2000	0.290	0.404	0.534
Tanzania	Tunduma	2017	0.106	0.148	0.205
Tanzania	Tunduma	2000-2017	-0.088	-0.059	-0.032
Tanzania	Tunduru	2000	0.284	0.399	0.567
Tanzania	Tunduru	2017	0.107	0.162	0.230
Tanzania	Tunduru	2000-2017	-0.058	-0.051	-0.045
Tanzania	Ukerewe	2000	0.256	0.377	0.547
Tanzania	Ukerewe	2017	0.117	0.174	0.248
Tanzania	Ukerewe	2000-2017	-0.055	-0.044	-0.035
Tanzania	Ulanga	2000	0.265	0.384	0.539
Tanzania	Ulanga	2017	0.110	0.160	0.227
Tanzania	Ulanga	2000-2017	-0.056	-0.051	-0.046
Tanzania	Urambo	2000	0.242	0.349	0.490
Tanzania	Urambo	2017	0.107	0.157	0.222
Tanzania	Urambo	2000-2017	-0.053	-0.046	-0.038
Tanzania	Uvinza	2000	0.278	0.394	0.566
Tanzania	Uvinza	2017	0.111	0.164	0.228
Tanzania	Uvinza	2000-2017	-0.055	-0.051	-0.046
Tanzania	Uyui	2000	0.245	0.349	0.485
Tanzania	Uyui	2017	0.109	0.160	0.225
Tanzania	Uyui	2000-2017	-0.050	-0.045	-0.040
Tanzania	Wanging'ombe	2000	0.297	0.436	0.615
Tanzania	Wanging'ombe	2017	0.120	0.174	0.249
Tanzania	Wanging'ombe	2000-2017	-0.059	-0.053	-0.047
Tanzania	Wete	2000	0.250	0.374	0.534
Tanzania	Wete	2017	0.111	0.167	0.244
Tanzania	Wete	2000-2017	-0.065	-0.047	-0.033
Togo	Amou	2000	0.158	0.284	0.448
Togo	Amou	2017	0.064	0.106	0.171
Togo	Amou	2000-2017	-0.085	-0.058	-0.029
Togo	Assoli	2000	0.161	0.284	0.452
Togo	Assoli	2017	0.068	0.111	0.172
Togo	Assoli	2000-2017	-0.079	-0.055	-0.028
Togo	Bassar	2000	0.165	0.298	0.484
Togo	Bassar	2017	0.072	0.118	0.184
Togo	Bassar	2000-2017	-0.077	-0.054	-0.029
Togo	Bimah	2000	0.162	0.289	0.472
Togo	Bimah	2017	0.068	0.113	0.181
Togo	Bimah	2000-2017	-0.082	-0.055	-0.030
Togo	Doufelgou	2000	0.161	0.288	0.475
Togo	Doufelgou	2017	0.067	0.114	0.180
Togo	Doufelgou	2000-2017	-0.079	-0.054	-0.030
Togo	Golfe (incl Lomé)	2000	0.145	0.267	0.454

Table 1: LRI DALYs rate by unit *(continued)*

Country	Unit	year	mean rate	lower rate	upper rate
Togo	Golfe (incl Lomé)	2017	0.061	0.101	0.168
Togo	Golfe (incl Lomé)	2000-2017	-0.083	-0.056	-0.024
Togo	Haho	2000	0.159	0.286	0.442
Togo	Haho	2017	0.067	0.107	0.172
Togo	Haho	2000-2017	-0.083	-0.058	-0.032
Togo	Kéran	2000	0.169	0.300	0.502
Togo	Kéran	2017	0.072	0.120	0.187
Togo	Kéran	2000-2017	-0.079	-0.054	-0.029
Togo	Kloto	2000	0.162	0.286	0.438
Togo	Kloto	2017	0.065	0.108	0.171
Togo	Kloto	2000-2017	-0.083	-0.058	-0.031
Togo	Kozah	2000	0.158	0.286	0.459
Togo	Kozah	2017	0.065	0.113	0.177
Togo	Kozah	2000-2017	-0.078	-0.054	-0.029
Togo	Lacs	2000	0.154	0.283	0.458
Togo	Lacs	2017	0.063	0.106	0.178
Togo	Lacs	2000-2017	-0.082	-0.058	-0.030
Togo	Ogou	2000	0.154	0.280	0.437
Togo	Ogou	2017	0.064	0.107	0.166
Togo	Ogou	2000-2017	-0.080	-0.057	-0.031
Togo	Oti	2000	0.187	0.329	0.540
Togo	Oti	2017	0.074	0.122	0.189
Togo	Oti	2000-2017	-0.086	-0.060	-0.037
Togo	Sotouboua	2000	0.156	0.280	0.440
Togo	Sotouboua	2017	0.064	0.109	0.175
Togo	Sotouboua	2000-2017	-0.081	-0.056	-0.029
Togo	Tchamba (Nyala)	2000	0.148	0.278	0.436
Togo	Tchamba (Nyala)	2017	0.064	0.108	0.170
Togo	Tchamba (Nyala)	2000-2017	-0.079	-0.055	-0.029
Togo	Tchaudjo	2000	0.151	0.277	0.425
Togo	Tchaudjo	2017	0.067	0.109	0.172
Togo	Tchaudjo	2000-2017	-0.081	-0.055	-0.030
Togo	Tône	2000	0.169	0.304	0.500
Togo	Tône	2017	0.076	0.124	0.194
Togo	Tône	2000-2017	-0.077	-0.052	-0.029
Togo	Vo	2000	0.158	0.283	0.475
Togo	Vo	2017	0.062	0.106	0.174
Togo	Vo	2000-2017	-0.082	-0.057	-0.030
Togo	Wawa	2000	0.163	0.291	0.455
Togo	Wawa	2017	0.068	0.110	0.174
Togo	Wawa	2000-2017	-0.081	-0.057	-0.029
Togo	Yoto	2000	0.166	0.283	0.445
Togo	Yoto	2017	0.063	0.105	0.166
Togo	Yoto	2000-2017	-0.083	-0.059	-0.032
Togo	Zio	2000	0.157	0.274	0.443
Togo	Zio	2017	0.060	0.103	0.166
Togo	Zio	2000-2017	-0.080	-0.058	-0.027
Uganda	Agago	2000	0.201	0.274	0.370
Uganda	Agago	2017	0.086	0.116	0.152
Uganda	Agago	2000-2017	-0.055	-0.046	-0.037
Uganda	Agule	2000	0.191	0.255	0.331
Uganda	Agule	2017	0.084	0.117	0.160
Uganda	Agule	2000-2017	-0.052	-0.041	-0.029
Uganda	Amuria	2000	0.202	0.276	0.372
Uganda	Amuria	2017	0.088	0.120	0.165
Uganda	Amuria	2000-2017	-0.052	-0.045	-0.038
Uganda	Apac Municipality	2000	0.189	0.260	0.337
Uganda	Apac Municipality	2017	0.083	0.113	0.151
Uganda	Apac Municipality	2000-2017	-0.057	-0.044	-0.033
Uganda	Aringa	2000	0.145	0.196	0.262
Uganda	Aringa	2017	0.043	0.061	0.083

Table 1: LRI DALYs rate by unit *(continued)*

Country	Unit	year	mean rate	lower rate	upper rate
Uganda	Aringa	2000-2017	-0.079	-0.067	-0.054
Uganda	Arua Municipality	2000	0.112	0.164	0.241
Uganda	Arua Municipality	2017	0.033	0.055	0.078
Uganda	Arua Municipality	2000-2017	-0.093	-0.060	-0.020
Uganda	Aruu	2000	0.193	0.263	0.353
Uganda	Aruu	2017	0.082	0.112	0.150
Uganda	Aruu	2000-2017	-0.055	-0.045	-0.036
Uganda	Aswa	2000	0.186	0.253	0.338
Uganda	Aswa	2017	0.075	0.104	0.139
Uganda	Aswa	2000-2017	-0.058	-0.047	-0.035
Uganda	Ayivu	2000	0.131	0.182	0.239
Uganda	Ayivu	2017	0.040	0.059	0.080
Uganda	Ayivu	2000-2017	-0.078	-0.061	-0.046
Uganda	Bamunanika	2000	0.128	0.173	0.233
Uganda	Bamunanika	2017	0.051	0.070	0.090
Uganda	Bamunanika	2000-2017	-0.057	-0.048	-0.039
Uganda	Bbaale	2000	0.131	0.176	0.231
Uganda	Bbaale	2017	0.055	0.074	0.095
Uganda	Bbaale	2000-2017	-0.054	-0.046	-0.037
Uganda	Bokora	2000	0.209	0.292	0.404
Uganda	Bokora	2017	0.129	0.176	0.243
Uganda	Bokora	2000-2017	-0.033	-0.025	-0.019
Uganda	Bubulo East	2000	0.175	0.243	0.325
Uganda	Bubulo East	2017	0.085	0.116	0.156
Uganda	Bubulo East	2000-2017	-0.052	-0.039	-0.024
Uganda	Bubulo West	2000	0.180	0.244	0.325
Uganda	Bubulo West	2017	0.082	0.111	0.146
Uganda	Bubulo West	2000-2017	-0.055	-0.042	-0.028
Uganda	Budadiri	2000	0.189	0.262	0.354
Uganda	Budadiri	2017	0.082	0.116	0.153
Uganda	Budadiri	2000-2017	-0.055	-0.044	-0.032
Uganda	Budaka	2000	0.185	0.249	0.347
Uganda	Budaka	2017	0.081	0.113	0.147
Uganda	Budaka	2000-2017	-0.054	-0.042	-0.028
Uganda	Budiope	2000	0.189	0.259	0.348
Uganda	Budiope	2017	0.086	0.118	0.157
Uganda	Budiope	2000-2017	-0.049	-0.041	-0.033
Uganda	Bufumbira	2000	0.143	0.195	0.251
Uganda	Bufumbira	2017	0.040	0.056	0.075
Uganda	Bufumbira	2000-2017	-0.083	-0.071	-0.058
Uganda	Bugabula	2000	0.187	0.259	0.348
Uganda	Bugabula	2017	0.083	0.116	0.154
Uganda	Bugabula	2000-2017	-0.051	-0.042	-0.033
Uganda	Bugahya	2000	0.125	0.170	0.222
Uganda	Bugahya	2017	0.043	0.062	0.082
Uganda	Bugahya	2000-2017	-0.067	-0.055	-0.044
Uganda	Bugangaizi	2000	0.117	0.160	0.207
Uganda	Bugangaizi	2017	0.044	0.061	0.079
Uganda	Bugangaizi	2000-2017	-0.059	-0.051	-0.042
Uganda	Bughendera	2000	0.124	0.171	0.225
Uganda	Bughendera	2017	0.036	0.053	0.073
Uganda	Bughendera	2000-2017	-0.080	-0.066	-0.052
Uganda	Bugiri Municipality	2000	0.161	0.242	0.350
Uganda	Bugiri Municipality	2017	0.071	0.111	0.163
Uganda	Bugiri Municipality	2000-2017	-0.077	-0.040	-0.006
Uganda	Bugweri	2000	0.178	0.242	0.326
Uganda	Bugweri	2017	0.082	0.109	0.144
Uganda	Bugweri	2000-2017	-0.054	-0.042	-0.031
Uganda	Buhaguzi	2000	0.124	0.168	0.217
Uganda	Buhaguzi	2017	0.044	0.061	0.080
Uganda	Buhaguzi	2000-2017	-0.064	-0.055	-0.045

Table 1: LRI DALYs rate by unit *(continued)*

Country	Unit	year	mean rate	lower rate	upper rate
Uganda	Buhweju	2000	0.141	0.190	0.245
Uganda	Buhweju	2017	0.043	0.061	0.080
Uganda	Buhweju	2000-2017	-0.075	-0.064	-0.053
Uganda	Buikwe	2000	0.124	0.169	0.224
Uganda	Buikwe	2017	0.047	0.063	0.082
Uganda	Buikwe	2000-2017	-0.065	-0.053	-0.044
Uganda	Bujenje	2000	0.125	0.172	0.223
Uganda	Bujenje	2017	0.046	0.065	0.085
Uganda	Bujenje	2000-2017	-0.063	-0.052	-0.040
Uganda	Bujumba	2000	0.123	0.167	0.224
Uganda	Bujumba	2017	0.047	0.063	0.083
Uganda	Bujumba	2000-2017	-0.064	-0.052	-0.041
Uganda	Bukanga	2000	0.137	0.181	0.235
Uganda	Bukanga	2017	0.047	0.063	0.084
Uganda	Bukanga	2000-2017	-0.070	-0.062	-0.053
Uganda	Bukedea	2000	0.198	0.263	0.352
Uganda	Bukedea	2017	0.090	0.122	0.165
Uganda	Bukedea	2000-2017	-0.050	-0.041	-0.032
Uganda	Bukomansimbi	2000	0.121	0.168	0.221
Uganda	Bukomansimbi	2017	0.052	0.072	0.097
Uganda	Bukomansimbi	2000-2017	-0.058	-0.048	-0.039
Uganda	Bukonzo	2000	0.134	0.181	0.236
Uganda	Bukonzo	2017	0.037	0.053	0.072
Uganda	Bukonzo	2000-2017	-0.086	-0.072	-0.056
Uganda	Bukooli	2000	0.177	0.239	0.321
Uganda	Bukooli	2017	0.082	0.111	0.144
Uganda	Bukooli	2000-2017	-0.050	-0.040	-0.031
Uganda	Bukooli North	2000	0.183	0.248	0.326
Uganda	Bukooli North	2017	0.082	0.114	0.149
Uganda	Bukooli North	2000-2017	-0.050	-0.041	-0.032
Uganda	Bukoto	2000	0.121	0.165	0.223
Uganda	Bukoto	2000	0.122	0.165	0.215
Uganda	Bukoto	2017	0.050	0.069	0.091
Uganda	Bukoto	2017	0.050	0.068	0.091
Uganda	Bukoto	2000-2017	-0.059	-0.051	-0.043
Uganda	Bukoto	2000-2017	-0.059	-0.050	-0.040
Uganda	Bulambuli	2000	0.199	0.267	0.356
Uganda	Bulambuli	2017	0.086	0.120	0.163
Uganda	Bulambuli	2000-2017	-0.055	-0.043	-0.030
Uganda	Bulamogi	2000	0.186	0.257	0.338
Uganda	Bulamogi	2017	0.084	0.116	0.154
Uganda	Bulamogi	2000-2017	-0.050	-0.042	-0.033
Uganda	Buliisa	2000	0.121	0.164	0.212
Uganda	Buliisa	2017	0.047	0.065	0.086
Uganda	Buliisa	2000-2017	-0.059	-0.049	-0.039
Uganda	Bungokho	2000	0.177	0.248	0.329
Uganda	Bungokho	2017	0.081	0.110	0.145
Uganda	Bungokho	2000-2017	-0.054	-0.044	-0.034
Uganda	Bunya	2000	0.188	0.253	0.340
Uganda	Bunya	2017	0.082	0.113	0.149
Uganda	Bunya	2000-2017	-0.051	-0.043	-0.035
Uganda	Bunyangabu	2000	0.129	0.173	0.227
Uganda	Bunyangabu	2017	0.039	0.057	0.077
Uganda	Bunyangabu	2000-2017	-0.077	-0.062	-0.049
Uganda	Bunyaruguru	2000	0.132	0.177	0.231
Uganda	Bunyaruguru	2017	0.038	0.054	0.073
Uganda	Bunyaruguru	2000-2017	-0.081	-0.067	-0.052
Uganda	Bunyole	2000	0.176	0.241	0.319
Uganda	Bunyole	2017	0.082	0.111	0.147
Uganda	Bunyole	2000-2017	-0.052	-0.041	-0.031
Uganda	Burahya	2000	0.127	0.174	0.225

Table 1: LRI DALYs rate by unit *(continued)*

Country	Unit	year	mean rate	lower rate	upper rate
Uganda	Burahya	2017	0.041	0.059	0.079
Uganda	Burahya	2000-2017	-0.073	-0.061	-0.050
Uganda	Buruli	2000	0.124	0.171	0.226
Uganda	Buruli	2000	0.125	0.172	0.225
Uganda	Buruli	2017	0.050	0.069	0.088
Uganda	Buruli	2017	0.054	0.073	0.094
Uganda	Buruli	2000-2017	-0.052	-0.046	-0.039
Uganda	Buruli	2000-2017	-0.057	-0.048	-0.040
Uganda	Bushenyi-Ishaka Municipality	2000	0.124	0.174	0.242
Uganda	Bushenyi-Ishaka Municipality	2017	0.038	0.056	0.079
Uganda	Bushenyi-Ishaka Municipality	2000-2017	-0.090	-0.066	-0.044
Uganda	Busia Municipality	2000	0.147	0.232	0.345
Uganda	Busia Municipality	2017	0.075	0.116	0.184
Uganda	Busia Municipality	2000-2017	-0.066	-0.030	0.010
Uganda	Busiki	2000	0.183	0.249	0.331
Uganda	Busiki	2017	0.084	0.116	0.153
Uganda	Busiki	2000-2017	-0.050	-0.040	-0.031
Uganda	Busiro	2000	0.109	0.152	0.202
Uganda	Busiro	2017	0.039	0.057	0.077
Uganda	Busiro	2000-2017	-0.067	-0.057	-0.046
Uganda	Busongora	2000	0.135	0.183	0.242
Uganda	Busongora	2017	0.039	0.056	0.076
Uganda	Busongora	2000-2017	-0.081	-0.068	-0.055
Uganda	Busujju	2000	0.123	0.170	0.222
Uganda	Busujju	2017	0.051	0.069	0.090
Uganda	Busujju	2000-2017	-0.060	-0.049	-0.038
Uganda	Butambala	2000	0.128	0.177	0.233
Uganda	Butambala	2017	0.054	0.074	0.098
Uganda	Butambala	2000-2017	-0.059	-0.048	-0.036
Uganda	Butebo	2000	0.188	0.255	0.335
Uganda	Butebo	2017	0.085	0.116	0.155
Uganda	Butebo	2000-2017	-0.052	-0.042	-0.031
Uganda	Butembe	2000	0.178	0.242	0.328
Uganda	Butembe	2017	0.070	0.096	0.131
Uganda	Butembe	2000-2017	-0.069	-0.052	-0.035
Uganda	Buvuma Island	2000	0.135	0.183	0.241
Uganda	Buvuma Island	2017	0.055	0.076	0.102
Uganda	Buvuma Island	2000-2017	-0.057	-0.048	-0.039
Uganda	Buwekula	2000	0.118	0.161	0.209
Uganda	Buwekula	2017	0.048	0.065	0.083
Uganda	Buwekula	2000-2017	-0.057	-0.049	-0.039
Uganda	Buyaga	2000	0.122	0.162	0.207
Uganda	Buyaga	2017	0.039	0.057	0.075
Uganda	Buyaga	2000-2017	-0.069	-0.057	-0.046
Uganda	Buyanja	2000	0.116	0.157	0.201
Uganda	Buyanja	2017	0.042	0.058	0.075
Uganda	Buyanja	2000-2017	-0.064	-0.053	-0.043
Uganda	Buzaaya	2000	0.189	0.258	0.350
Uganda	Buzaaya	2017	0.082	0.112	0.149
Uganda	Buzaaya	2000-2017	-0.056	-0.044	-0.031
Uganda	Bwamba	2000	0.106	0.150	0.195
Uganda	Bwamba	2017	0.035	0.052	0.071
Uganda	Bwamba	2000-2017	-0.075	-0.059	-0.040
Uganda	Chekwii	2000	0.190	0.266	0.372
Uganda	Chekwii	2017	0.124	0.176	0.246
Uganda	Chekwii	2000-2017	-0.027	-0.019	-0.010
Uganda	Chua	2000	0.197	0.267	0.351
Uganda	Chua	2017	0.084	0.113	0.150
Uganda	Chua	2000-2017	-0.054	-0.045	-0.035
Uganda	Dodoth	2000	0.217	0.295	0.402
Uganda	Dodoth	2017	0.110	0.150	0.204

Table 1: LRI DALYs rate by unit *(continued)*

Country	Unit	year	mean rate	lower rate	upper rate
Uganda	Dodoth	2000-2017	-0.046	-0.038	-0.030
Uganda	Dokolo	2000	0.195	0.266	0.357
Uganda	Dokolo	2017	0.086	0.116	0.156
Uganda	Dokolo	2000-2017	-0.053	-0.044	-0.036
Uganda	East Moyo	2000	0.122	0.168	0.220
Uganda	East Moyo	2017	0.045	0.062	0.083
Uganda	East Moyo	2000-2017	-0.064	-0.053	-0.041
Uganda	Entebbe Municipality	2000	0.106	0.152	0.205
Uganda	Entebbe Municipality	2017	0.039	0.056	0.079
Uganda	Entebbe Municipality	2000-2017	-0.084	-0.056	-0.031
Uganda	Erute	2000	0.187	0.257	0.342
Uganda	Erute	2017	0.081	0.108	0.142
Uganda	Erute	2000-2017	-0.056	-0.046	-0.037
Uganda	Fort Portal Municipality	2000	0.101	0.149	0.213
Uganda	Fort Portal Municipality	2017	0.035	0.055	0.082
Uganda	Fort Portal Municipality	2000-2017	-0.090	-0.060	-0.027
Uganda	Gomba	2000	0.124	0.173	0.227
Uganda	Gomba	2017	0.053	0.072	0.094
Uganda	Gomba	2000-2017	-0.056	-0.047	-0.039
Uganda	Gulu Municipality	2000	0.162	0.238	0.342
Uganda	Gulu Municipality	2017	0.060	0.093	0.136
Uganda	Gulu Municipality	2000-2017	-0.087	-0.051	-0.017
Uganda	Hoima Municipality	2000	0.106	0.155	0.207
Uganda	Hoima Municipality	2017	0.039	0.056	0.079
Uganda	Hoima Municipality	2000-2017	-0.079	-0.056	-0.035
Uganda	Ibanda	2000	0.142	0.193	0.255
Uganda	Ibanda	2017	0.043	0.061	0.081
Uganda	Ibanda	2000-2017	-0.075	-0.064	-0.052
Uganda	Ibanda Municipality	2000	0.143	0.191	0.253
Uganda	Ibanda Municipality	2017	0.041	0.060	0.079
Uganda	Ibanda Municipality	2000-2017	-0.079	-0.065	-0.051
Uganda	Iganga Municipality	2000	0.139	0.234	0.355
Uganda	Iganga Municipality	2017	0.063	0.099	0.148
Uganda	Iganga Municipality	2000-2017	-0.089	-0.046	0.001
Uganda	Igara	2000	0.132	0.182	0.241
Uganda	Igara	2017	0.043	0.059	0.079
Uganda	Igara	2000-2017	-0.078	-0.065	-0.054
Uganda	Iki-Iki	2000	0.188	0.253	0.348
Uganda	Iki-Iki	2017	0.082	0.114	0.155
Uganda	Iki-Iki	2000-2017	-0.057	-0.042	-0.028
Uganda	Isingiro	2000	0.138	0.188	0.244
Uganda	Isingiro	2017	0.045	0.062	0.082
Uganda	Isingiro	2000-2017	-0.072	-0.063	-0.054
Uganda	Jie	2000	0.222	0.309	0.427
Uganda	Jie	2017	0.126	0.173	0.236
Uganda	Jie	2000-2017	-0.038	-0.030	-0.022
Uganda	Jinja Municipality	2000	0.143	0.211	0.298
Uganda	Jinja Municipality	2017	0.051	0.079	0.115
Uganda	Jinja Municipality	2000-2017	-0.091	-0.059	-0.031
Uganda	Jonam	2000	0.124	0.173	0.230
Uganda	Jonam	2017	0.050	0.070	0.092
Uganda	Jonam	2000-2017	-0.060	-0.048	-0.036
Uganda	Kabale Municipality	2000	0.109	0.154	0.219
Uganda	Kabale Municipality	2017	0.035	0.054	0.075
Uganda	Kabale Municipality	2000-2017	-0.096	-0.064	-0.034
Uganda	Kaberamaido	2000	0.192	0.263	0.358
Uganda	Kaberamaido	2017	0.085	0.116	0.157
Uganda	Kaberamaido	2000-2017	-0.053	-0.044	-0.034
Uganda	Kabula	2000	0.137	0.183	0.242
Uganda	Kabula	2017	0.051	0.069	0.088
Uganda	Kabula	2000-2017	-0.066	-0.056	-0.046

Table 1: LRI DALYs rate by unit *(continued)*

Country	Unit	year	mean rate	lower rate	upper rate
Uganda	Kagoma	2000	0.177	0.248	0.329
Uganda	Kagoma	2017	0.076	0.106	0.142
Uganda	Kagoma	2000-2017	-0.060	-0.047	-0.035
Uganda	Kajara	2000	0.132	0.179	0.238
Uganda	Kajara	2017	0.044	0.061	0.082
Uganda	Kajara	2000-2017	-0.082	-0.067	-0.052
Uganda	Kakuuto North	2000	0.125	0.173	0.231
Uganda	Kakuuto North	2017	0.048	0.067	0.091
Uganda	Kakuuto North	2000-2017	-0.070	-0.054	-0.039
Uganda	Kalaki	2000	0.194	0.269	0.362
Uganda	Kalaki	2017	0.086	0.117	0.157
Uganda	Kalaki	2000-2017	-0.053	-0.044	-0.034
Uganda	Kalungu	2000	0.119	0.168	0.220
Uganda	Kalungu	2017	0.053	0.073	0.096
Uganda	Kalungu	2000-2017	-0.055	-0.047	-0.038
Uganda	Kamuli Municipality	2000	0.180	0.260	0.360
Uganda	Kamuli Municipality	2017	0.081	0.115	0.163
Uganda	Kamuli Municipality	2000-2017	-0.066	-0.044	-0.025
Uganda	Kapchorwa Municipality	2000	0.199	0.279	0.382
Uganda	Kapchorwa Municipality	2017	0.082	0.116	0.157
Uganda	Kapchorwa Municipality	2000-2017	-0.066	-0.049	-0.032
Uganda	Kapelebyong	2000	0.204	0.281	0.379
Uganda	Kapelebyong	2017	0.089	0.123	0.169
Uganda	Kapelebyong	2000-2017	-0.052	-0.044	-0.036
Uganda	Kasambya	2000	0.124	0.168	0.221
Uganda	Kasambya	2017	0.050	0.068	0.090
Uganda	Kasambya	2000-2017	-0.060	-0.050	-0.040
Uganda	Kasese Municipality	2000	0.131	0.177	0.231
Uganda	Kasese Municipality	2017	0.038	0.055	0.078
Uganda	Kasese Municipality	2000-2017	-0.086	-0.066	-0.046
Uganda	Kashari	2000	0.137	0.187	0.239
Uganda	Kashari	2017	0.045	0.062	0.081
Uganda	Kashari	2000-2017	-0.075	-0.064	-0.055
Uganda	Kasilo	2000	0.193	0.261	0.362
Uganda	Kasilo	2017	0.088	0.120	0.163
Uganda	Kasilo	2000-2017	-0.050	-0.041	-0.031
Uganda	Kassanda	2000	0.122	0.164	0.216
Uganda	Kassanda	2017	0.049	0.067	0.087
Uganda	Kassanda	2000-2017	-0.057	-0.049	-0.040
Uganda	Katerera	2000	0.129	0.177	0.234
Uganda	Katerera	2017	0.037	0.054	0.073
Uganda	Katerera	2000-2017	-0.085	-0.068	-0.050
Uganda	Katikamu	2000	0.118	0.161	0.215
Uganda	Katikamu	2017	0.048	0.064	0.084
Uganda	Katikamu	2000-2017	-0.061	-0.050	-0.039
Uganda	Katuuto East	2000	0.126	0.172	0.225
Uganda	Katuuto East	2017	0.050	0.067	0.088
Uganda	Katuuto East	2000-2017	-0.063	-0.053	-0.041
Uganda	Katuuto West	2000	0.131	0.174	0.229
Uganda	Katuuto West	2017	0.051	0.069	0.093
Uganda	Katuuto West	2000-2017	-0.063	-0.052	-0.042
Uganda	Kazo	2000	0.132	0.175	0.225
Uganda	Kazo	2017	0.048	0.066	0.089
Uganda	Kazo	2000-2017	-0.064	-0.055	-0.044
Uganda	Kcca	2000	0.094	0.137	0.192
Uganda	Kcca	2017	0.030	0.048	0.071
Uganda	Kcca	2000-2017	-0.078	-0.062	-0.044
Uganda	Kibale	2000	0.122	0.168	0.222
Uganda	Kibale	2017	0.042	0.058	0.077
Uganda	Kibale	2000-2017	-0.069	-0.059	-0.048
Uganda	Kibanda	2000	0.128	0.175	0.230

Table 1: LRI DALYs rate by unit *(continued)*

Country	Unit	year	mean rate	lower rate	upper rate
Uganda	Kibanda	2017	0.053	0.070	0.092
Uganda	Kibanda	2000-2017	-0.058	-0.048	-0.039
Uganda	Kiboga	2000	0.119	0.163	0.217
Uganda	Kiboga	2000	0.121	0.161	0.209
Uganda	Kiboga	2017	0.047	0.065	0.085
Uganda	Kiboga	2017	0.046	0.063	0.082
Uganda	Kiboga	2000-2017	-0.059	-0.049	-0.039
Uganda	Kiboga	2000-2017	-0.059	-0.050	-0.040
Uganda	Kibuku	2000	0.185	0.254	0.346
Uganda	Kibuku	2017	0.085	0.116	0.153
Uganda	Kibuku	2000-2017	-0.050	-0.041	-0.030
Uganda	Kigulu	2000	0.184	0.250	0.334
Uganda	Kigulu	2017	0.080	0.111	0.149
Uganda	Kigulu	2000-2017	-0.054	-0.043	-0.031
Uganda	Kilak	2000	0.183	0.249	0.323
Uganda	Kilak	2017	0.079	0.106	0.141
Uganda	Kilak	2000-2017	-0.055	-0.045	-0.035
Uganda	Kinkiizi	2000	0.135	0.181	0.236
Uganda	Kinkiizi	2017	0.042	0.058	0.076
Uganda	Kinkiizi	2000-2017	-0.074	-0.066	-0.055
Uganda	Kioga	2000	0.194	0.261	0.350
Uganda	Kioga	2017	0.087	0.117	0.158
Uganda	Kioga	2000-2017	-0.051	-0.043	-0.035
Uganda	Kira Municipality	2000	0.094	0.135	0.186
Uganda	Kira Municipality	2017	0.032	0.049	0.072
Uganda	Kira Municipality	2000-2017	-0.078	-0.061	-0.039
Uganda	Kisoro Municipality	2000	0.119	0.176	0.251
Uganda	Kisoro Municipality	2017	0.037	0.054	0.078
Uganda	Kisoro Municipality	2000-2017	-0.103	-0.071	-0.039
Uganda	Kitagwenda	2000	0.140	0.190	0.248
Uganda	Kitagwenda	2017	0.041	0.059	0.078
Uganda	Kitagwenda	2000-2017	-0.077	-0.065	-0.053
Uganda	Kitgum Municipality	2000	0.168	0.247	0.346
Uganda	Kitgum Municipality	2017	0.066	0.097	0.142
Uganda	Kitgum Municipality	2000-2017	-0.079	-0.048	-0.021
Uganda	Koboko	2000	0.139	0.193	0.254
Uganda	Koboko	2017	0.042	0.060	0.080
Uganda	Koboko	2000-2017	-0.080	-0.067	-0.052
Uganda	Koboko Municipality	2000	0.125	0.182	0.262
Uganda	Koboko Municipality	2017	0.036	0.056	0.077
Uganda	Koboko Municipality	2000-2017	-0.093	-0.070	-0.045
Uganda	Kole	2000	0.194	0.260	0.342
Uganda	Kole	2017	0.081	0.112	0.148
Uganda	Kole	2000-2017	-0.053	-0.045	-0.034
Uganda	Kongasis	2000	0.202	0.287	0.400
Uganda	Kongasis	2017	0.078	0.111	0.149
Uganda	Kongasis	2000-2017	-0.066	-0.053	-0.039
Uganda	Kooki	2000	0.129	0.172	0.228
Uganda	Kooki	2017	0.048	0.067	0.087
Uganda	Kooki	2000-2017	-0.064	-0.055	-0.048
Uganda	Kotido Municipality	2000	0.237	0.337	0.472
Uganda	Kotido Municipality	2017	0.124	0.171	0.232
Uganda	Kotido Municipality	2000-2017	-0.052	-0.037	-0.022
Uganda	Kumi	2000	0.186	0.255	0.337
Uganda	Kumi	2017	0.083	0.113	0.153
Uganda	Kumi	2000-2017	-0.052	-0.043	-0.034
Uganda	Kumi Municipality	2000	0.173	0.249	0.339
Uganda	Kumi Municipality	2017	0.074	0.107	0.146
Uganda	Kumi Municipality	2000-2017	-0.067	-0.046	-0.025
Uganda	Kwania	2000	0.192	0.255	0.339
Uganda	Kwania	2017	0.081	0.112	0.151

Table 1: LRI DALYs rate by unit *(continued)*

Country	Unit	year	mean rate	lower rate	upper rate
Uganda	Kwania	2000-2017	-0.052	-0.044	-0.035
Uganda	Kween	2000	0.213	0.291	0.390
Uganda	Kween	2017	0.087	0.116	0.156
Uganda	Kween	2000-2017	-0.062	-0.051	-0.039
Uganda	Kyadondo	2000	0.098	0.143	0.196
Uganda	Kyadondo	2017	0.034	0.052	0.071
Uganda	Kyadondo	2000-2017	-0.078	-0.058	-0.038
Uganda	Kyaka	2000	0.117	0.158	0.202
Uganda	Kyaka	2017	0.046	0.063	0.082
Uganda	Kyaka	2000-2017	-0.057	-0.050	-0.042
Uganda	Kyamuswa	2000	0.120	0.164	0.221
Uganda	Kyamuswa	2017	0.048	0.066	0.085
Uganda	Kyamuswa	2000-2017	-0.058	-0.049	-0.040
Uganda	Kyotera	2000	0.124	0.168	0.223
Uganda	Kyotera	2017	0.051	0.069	0.093
Uganda	Kyotera	2000-2017	-0.061	-0.051	-0.041
Uganda	Labwor	2000	0.236	0.333	0.459
Uganda	Labwor	2017	0.138	0.197	0.272
Uganda	Labwor	2000-2017	-0.035	-0.027	-0.018
Uganda	Lamwo	2000	0.192	0.261	0.351
Uganda	Lamwo	2017	0.084	0.111	0.148
Uganda	Lamwo	2000-2017	-0.053	-0.045	-0.036
Uganda	Lira Municipality	2000	0.154	0.231	0.336
Uganda	Lira Municipality	2017	0.061	0.091	0.133
Uganda	Lira Municipality	2000-2017	-0.079	-0.049	-0.014
Uganda	Lugazi Municipality	2000	0.120	0.167	0.228
Uganda	Lugazi Municipality	2017	0.045	0.063	0.086
Uganda	Lugazi Municipality	2000-2017	-0.072	-0.055	-0.038
Uganda	Luuka	2000	0.188	0.254	0.340
Uganda	Luuka	2017	0.082	0.112	0.146
Uganda	Luuka	2000-2017	-0.054	-0.043	-0.033
Uganda	Lwemiyaga	2000	0.123	0.167	0.215
Uganda	Lwemiyaga	2017	0.050	0.069	0.093
Uganda	Lwemiyaga	2000-2017	-0.058	-0.048	-0.038
Uganda	Madi Okollo	2000	0.128	0.176	0.231
Uganda	Madi Okollo	2017	0.048	0.067	0.088
Uganda	Madi Okollo	2000-2017	-0.061	-0.052	-0.042
Uganda	Makindye Ssabagabo Municipality	2000	0.097	0.141	0.201
Uganda	Makindye Ssabagabo Municipality	2017	0.033	0.050	0.074
Uganda	Makindye Ssabagabo Municipality	2000-2017	-0.085	-0.062	-0.042
Uganda	Manjiya	2000	0.191	0.261	0.357
Uganda	Manjiya	2017	0.086	0.118	0.166
Uganda	Manjiya	2000-2017	-0.055	-0.043	-0.030
Uganda	Maracha	2000	0.142	0.196	0.258
Uganda	Maracha	2017	0.043	0.061	0.084
Uganda	Maracha	2000-2017	-0.078	-0.066	-0.053
Uganda	Maruzi	2000	0.188	0.258	0.344
Uganda	Maruzi	2017	0.085	0.113	0.151
Uganda	Maruzi	2000-2017	-0.052	-0.044	-0.035
Uganda	Masaka Municipality	2000	0.105	0.149	0.213
Uganda	Masaka Municipality	2017	0.038	0.055	0.080
Uganda	Masaka Municipality	2000-2017	-0.085	-0.057	-0.032
Uganda	Masindi Municipality	2000	0.123	0.172	0.228
Uganda	Masindi Municipality	2017	0.045	0.064	0.084
Uganda	Masindi Municipality	2000-2017	-0.075	-0.054	-0.034
Uganda	Matheniko	2000	0.185	0.262	0.356
Uganda	Matheniko	2017	0.116	0.161	0.219
Uganda	Matheniko	2000-2017	-0.035	-0.024	-0.013
Uganda	Mawogola	2000	0.125	0.171	0.225
Uganda	Mawogola	2017	0.053	0.073	0.095
Uganda	Mawogola	2000-2017	-0.057	-0.048	-0.038

Table 1: LRI DALYs rate by unit *(continued)*

Country	Unit	year	mean rate	lower rate	upper rate
Uganda	Mawokota	2000	0.127	0.176	0.232
Uganda	Mawokota	2017	0.053	0.071	0.093
Uganda	Mawokota	2000-2017	-0.057	-0.050	-0.043
Uganda	Mbale Municipality	2000	0.163	0.241	0.341
Uganda	Mbale Municipality	2017	0.067	0.103	0.146
Uganda	Mbale Municipality	2000-2017	-0.076	-0.045	-0.017
Uganda	Mbarara Municipality	2000	0.119	0.167	0.227
Uganda	Mbarara Municipality	2017	0.040	0.058	0.078
Uganda	Mbarara Municipality	2000-2017	-0.079	-0.062	-0.048
Uganda	Mityana	2000	0.119	0.165	0.213
Uganda	Mityana	2017	0.049	0.065	0.084
Uganda	Mityana	2000-2017	-0.060	-0.050	-0.041
Uganda	Mityana Municipality	2000	0.117	0.163	0.222
Uganda	Mityana Municipality	2017	0.045	0.067	0.096
Uganda	Mityana Municipality	2000-2017	-0.069	-0.049	-0.029
Uganda	Moroto	2000	0.189	0.262	0.350
Uganda	Moroto	2017	0.083	0.112	0.147
Uganda	Moroto	2000-2017	-0.053	-0.045	-0.036
Uganda	Moroto Municipality	2000	0.181	0.328	0.505
Uganda	Moroto Municipality	2017	0.091	0.162	0.257
Uganda	Moroto Municipality	2000-2017	-0.088	-0.043	-0.003
Uganda	Mubende Municipality	2000	0.115	0.163	0.221
Uganda	Mubende Municipality	2017	0.048	0.066	0.089
Uganda	Mubende Municipality	2000-2017	-0.074	-0.050	-0.030
Uganda	Mukono	2000	0.124	0.169	0.223
Uganda	Mukono	2017	0.046	0.063	0.081
Uganda	Mukono	2000-2017	-0.064	-0.055	-0.045
Uganda	Mukono Municipality	2000	0.109	0.155	0.214
Uganda	Mukono Municipality	2017	0.037	0.055	0.077
Uganda	Mukono Municipality	2000-2017	-0.078	-0.060	-0.039
Uganda	Mwenge	2000	0.125	0.171	0.219
Uganda	Mwenge	2017	0.043	0.060	0.079
Uganda	Mwenge	2000-2017	-0.067	-0.059	-0.048
Uganda	Nakaseke	2000	0.120	0.165	0.213
Uganda	Nakaseke	2017	0.050	0.067	0.088
Uganda	Nakaseke	2000-2017	-0.056	-0.048	-0.040
Uganda	Nakifuma	2000	0.127	0.176	0.233
Uganda	Nakifuma	2017	0.050	0.067	0.087
Uganda	Nakifuma	2000-2017	-0.065	-0.054	-0.044
Uganda	Nansana Municipality	2000	0.105	0.151	0.205
Uganda	Nansana Municipality	2017	0.037	0.055	0.074
Uganda	Nansana Municipality	2000-2017	-0.073	-0.058	-0.042
Uganda	Ndorwa	2000	0.125	0.171	0.226
Uganda	Ndorwa	2017	0.043	0.059	0.077
Uganda	Ndorwa	2000-2017	-0.071	-0.061	-0.050
Uganda	Nebbi Municipality	2000	0.117	0.172	0.248
Uganda	Nebbi Municipality	2017	0.040	0.062	0.093
Uganda	Nebbi Municipality	2000-2017	-0.083	-0.056	-0.028
Uganda	Ngora	2000	0.190	0.256	0.351
Uganda	Ngora	2017	0.084	0.113	0.152
Uganda	Ngora	2000-2017	-0.053	-0.044	-0.034
Uganda	Njeru Municipality	2000	0.124	0.169	0.222
Uganda	Njeru Municipality	2017	0.046	0.065	0.087
Uganda	Njeru Municipality	2000-2017	-0.067	-0.053	-0.038
Uganda	Ntenjeru	2000	0.133	0.178	0.238
Uganda	Ntenjeru	2017	0.051	0.070	0.092
Uganda	Ntenjeru	2000-2017	-0.061	-0.051	-0.038
Uganda	Ntoroko	2000	0.135	0.181	0.235
Uganda	Ntoroko	2017	0.045	0.063	0.084
Uganda	Ntoroko	2000-2017	-0.072	-0.058	-0.045
Uganda	Ntungamo Municipality	2000	0.120	0.179	0.258

Table 1: LRI DALYs rate by unit *(continued)*

Country	Unit	year	mean rate	lower rate	upper rate
Uganda	Ntungamo Municipality	2017	0.038	0.057	0.085
Uganda	Ntungamo Municipality	2000-2017	-0.094	-0.067	-0.038
Uganda	Nwoya	2000	0.175	0.239	0.324
Uganda	Nwoya	2017	0.078	0.106	0.139
Uganda	Nwoya	2000-2017	-0.050	-0.043	-0.036
Uganda	Nyabushozi	2000	0.137	0.184	0.238
Uganda	Nyabushozi	2017	0.051	0.068	0.088
Uganda	Nyabushozi	2000-2017	-0.065	-0.057	-0.049
Uganda	Obongi	2000	0.122	0.171	0.223
Uganda	Obongi	2017	0.043	0.060	0.081
Uganda	Obongi	2000-2017	-0.071	-0.057	-0.044
Uganda	Okoro	2000	0.148	0.203	0.268
Uganda	Okoro	2017	0.047	0.065	0.084
Uganda	Okoro	2000-2017	-0.072	-0.062	-0.051
Uganda	Omoro	2000	0.181	0.245	0.326
Uganda	Omoro	2017	0.073	0.099	0.132
Uganda	Omoro	2000-2017	-0.058	-0.047	-0.036
Uganda	Otuke	2000	0.196	0.270	0.364
Uganda	Otuke	2017	0.089	0.120	0.162
Uganda	Otuke	2000-2017	-0.052	-0.043	-0.034
Uganda	Oyam	2000	0.191	0.259	0.344
Uganda	Oyam	2017	0.082	0.110	0.147
Uganda	Oyam	2000-2017	-0.053	-0.045	-0.036
Uganda	Padyere	2000	0.120	0.166	0.219
Uganda	Padyere	2017	0.046	0.063	0.084
Uganda	Padyere	2000-2017	-0.065	-0.052	-0.041
Uganda	Pallisa	2000	0.184	0.256	0.346
Uganda	Pallisa	2017	0.083	0.115	0.158
Uganda	Pallisa	2000-2017	-0.056	-0.042	-0.028
Uganda	Pian	2000	0.197	0.281	0.388
Uganda	Pian	2017	0.130	0.184	0.258
Uganda	Pian	2000-2017	-0.029	-0.021	-0.012
Uganda	Pokot	2000	0.204	0.282	0.381
Uganda	Pokot	2017	0.124	0.173	0.239
Uganda	Pokot	2000-2017	-0.032	-0.023	-0.013
Uganda	Rubabo	2000	0.130	0.179	0.234
Uganda	Rubabo	2017	0.043	0.060	0.078
Uganda	Rubabo	2000-2017	-0.079	-0.067	-0.056
Uganda	Rubanda	2000	0.126	0.171	0.225
Uganda	Rubanda	2017	0.041	0.057	0.076
Uganda	Rubanda	2000-2017	-0.077	-0.066	-0.056
Uganda	Ruhaama	2000	0.139	0.193	0.252
Uganda	Ruhaama	2017	0.044	0.061	0.081
Uganda	Ruhaama	2000-2017	-0.075	-0.065	-0.057
Uganda	Ruhinda	2000	0.132	0.180	0.232
Uganda	Ruhinda	2017	0.042	0.058	0.077
Uganda	Ruhinda	2000-2017	-0.081	-0.070	-0.060
Uganda	Rujumbura	2000	0.132	0.180	0.237
Uganda	Rujumbura	2017	0.043	0.058	0.078
Uganda	Rujumbura	2000-2017	-0.080	-0.070	-0.057
Uganda	Rukiga	2000	0.134	0.182	0.242
Uganda	Rukiga	2017	0.043	0.059	0.077
Uganda	Rukiga	2000-2017	-0.077	-0.066	-0.055
Uganda	Rukungiri Municipality	2000	0.111	0.164	0.220
Uganda	Rukungiri Municipality	2017	0.040	0.057	0.077
Uganda	Rukungiri Municipality	2000-2017	-0.089	-0.067	-0.042
Uganda	Rushenyi	2000	0.142	0.192	0.249
Uganda	Rushenyi	2017	0.045	0.061	0.081
Uganda	Rushenyi	2000-2017	-0.079	-0.067	-0.058
Uganda	Rwampara	2000	0.145	0.195	0.256
Uganda	Rwampara	2017	0.045	0.062	0.082

Table 1: LRI DALYs rate by unit *(continued)*

Country	Unit	year	mean rate	lower rate	upper rate
Uganda	Rwampara	2000-2017	-0.073	-0.064	-0.054
Uganda	Samia-Bugwe	2000	0.174	0.234	0.309
Uganda	Samia-Bugwe	2017	0.084	0.112	0.151
Uganda	Samia-Bugwe	2000-2017	-0.046	-0.037	-0.029
Uganda	Serere	2000	0.201	0.270	0.358
Uganda	Serere	2017	0.089	0.121	0.162
Uganda	Serere	2000-2017	-0.051	-0.042	-0.034
Uganda	Sheema	2000	0.134	0.188	0.250
Uganda	Sheema	2017	0.042	0.059	0.080
Uganda	Sheema	2000-2017	-0.080	-0.068	-0.056
Uganda	Sheema Municipality	2000	0.138	0.190	0.254
Uganda	Sheema Municipality	2017	0.045	0.062	0.085
Uganda	Sheema Municipality	2000-2017	-0.080	-0.066	-0.051
Uganda	Soroti	2000	0.196	0.268	0.363
Uganda	Soroti	2017	0.088	0.119	0.158
Uganda	Soroti	2000-2017	-0.051	-0.044	-0.036
Uganda	Soroti Municipality	2000	0.168	0.247	0.345
Uganda	Soroti Municipality	2017	0.073	0.107	0.152
Uganda	Soroti Municipality	2000-2017	-0.073	-0.045	-0.019
Uganda	Terego	2000	0.133	0.182	0.236
Uganda	Terego	2017	0.043	0.061	0.083
Uganda	Terego	2000-2017	-0.073	-0.061	-0.049
Uganda	Tingey	2000	0.185	0.270	0.361
Uganda	Tingey	2017	0.085	0.119	0.168
Uganda	Tingey	2000-2017	-0.060	-0.045	-0.027
Uganda	Toroma	2000	0.206	0.285	0.395
Uganda	Toroma	2017	0.096	0.131	0.179
Uganda	Toroma	2000-2017	-0.052	-0.042	-0.032
Uganda	Tororo	2000	0.184	0.246	0.331
Uganda	Tororo	2017	0.084	0.113	0.148
Uganda	Tororo	2000-2017	-0.052	-0.040	-0.028
Uganda	Tororo Municipality	2000	0.174	0.255	0.375
Uganda	Tororo Municipality	2017	0.075	0.113	0.167
Uganda	Tororo Municipality	2000-2017	-0.081	-0.043	-0.008
Uganda	Usuk	2000	0.208	0.284	0.389
Uganda	Usuk	2017	0.094	0.129	0.177
Uganda	Usuk	2000-2017	-0.050	-0.042	-0.032
Uganda	Vurra	2000	0.146	0.198	0.259
Uganda	Vurra	2017	0.049	0.069	0.091
Uganda	Vurra	2000-2017	-0.069	-0.057	-0.046
Uganda	West Budama	2000	0.176	0.238	0.309
Uganda	West Budama	2017	0.081	0.109	0.140
Uganda	West Budama	2000-2017	-0.049	-0.041	-0.032
Uganda	West Moyo	2000	0.121	0.170	0.225
Uganda	West Moyo	2017	0.044	0.061	0.082
Uganda	West Moyo	2000-2017	-0.071	-0.055	-0.042
Zambia	Chadiza	2000	0.286	0.415	0.572
Zambia	Chadiza	2017	0.087	0.127	0.181
Zambia	Chadiza	2000-2017	-0.078	-0.068	-0.057
Zambia	Chama	2000	0.255	0.382	0.541
Zambia	Chama	2017	0.085	0.130	0.185
Zambia	Chama	2000-2017	-0.076	-0.064	-0.052
Zambia	Chavuma	2000	0.265	0.403	0.587
Zambia	Chavuma	2017	0.084	0.127	0.186
Zambia	Chavuma	2000-2017	-0.080	-0.068	-0.054
Zambia	Chibombo	2000	0.252	0.405	0.584
Zambia	Chibombo	2017	0.091	0.142	0.214
Zambia	Chibombo	2000-2017	-0.078	-0.061	-0.047
Zambia	Chiengi	2000	0.311	0.473	0.707
Zambia	Chiengi	2017	0.108	0.165	0.244
Zambia	Chiengi	2000-2017	-0.069	-0.059	-0.049

Table 1: LRI DALYs rate by unit *(continued)*

Country	Unit	year	mean rate	lower rate	upper rate
Zambia	Chililabombwe	2000	0.254	0.406	0.594
Zambia	Chililabombwe	2017	0.108	0.161	0.237
Zambia	Chililabombwe	2000-2017	-0.075	-0.058	-0.038
Zambia	Chilubi	2000	0.241	0.376	0.555
Zambia	Chilubi	2017	0.092	0.139	0.201
Zambia	Chilubi	2000-2017	-0.067	-0.057	-0.044
Zambia	Chingola	2000	0.257	0.411	0.612
Zambia	Chingola	2017	0.106	0.165	0.255
Zambia	Chingola	2000-2017	-0.071	-0.054	-0.035
Zambia	Chinsali	2000	0.247	0.378	0.546
Zambia	Chinsali	2017	0.085	0.128	0.188
Zambia	Chinsali	2000-2017	-0.076	-0.063	-0.049
Zambia	Chipata	2000	0.270	0.410	0.589
Zambia	Chipata	2017	0.083	0.129	0.192
Zambia	Chipata	2000-2017	-0.081	-0.070	-0.056
Zambia	Choma	2000	0.242	0.372	0.537
Zambia	Choma	2017	0.084	0.128	0.185
Zambia	Choma	2000-2017	-0.073	-0.063	-0.051
Zambia	Chongwe	2000	0.259	0.395	0.568
Zambia	Chongwe	2017	0.082	0.125	0.183
Zambia	Chongwe	2000-2017	-0.080	-0.067	-0.054
Zambia	Gwembe	2000	0.242	0.371	0.534
Zambia	Gwembe	2017	0.084	0.127	0.188
Zambia	Gwembe	2000-2017	-0.072	-0.061	-0.049
Zambia	Isoka	2000	0.267	0.396	0.568
Zambia	Isoka	2017	0.088	0.133	0.192
Zambia	Isoka	2000-2017	-0.073	-0.064	-0.052
Zambia	Itezhi-Tezhi	2000	0.226	0.346	0.487
Zambia	Itezhi-Tezhi	2017	0.082	0.128	0.186
Zambia	Itezhi-Tezhi	2000-2017	-0.070	-0.059	-0.047
Zambia	Kabompo	2000	0.270	0.410	0.582
Zambia	Kabompo	2017	0.080	0.121	0.179
Zambia	Kabompo	2000-2017	-0.085	-0.072	-0.058
Zambia	Kabwe	2000	0.251	0.401	0.641
Zambia	Kabwe	2017	0.082	0.129	0.189
Zambia	Kabwe	2000-2017	-0.086	-0.065	-0.049
Zambia	Kafue	2000	0.263	0.407	0.603
Zambia	Kafue	2017	0.089	0.135	0.203
Zambia	Kafue	2000-2017	-0.079	-0.064	-0.048
Zambia	Kalabo	2000	0.265	0.411	0.598
Zambia	Kalabo	2017	0.082	0.126	0.187
Zambia	Kalabo	2000-2017	-0.084	-0.071	-0.056
Zambia	Kalomo	2000	0.227	0.347	0.494
Zambia	Kalomo	2017	0.086	0.129	0.192
Zambia	Kalomo	2000-2017	-0.068	-0.059	-0.048
Zambia	Kalulushi	2000	0.269	0.424	0.619
Zambia	Kalulushi	2017	0.111	0.165	0.238
Zambia	Kalulushi	2000-2017	-0.076	-0.059	-0.042
Zambia	Kaoma	2000	0.268	0.409	0.600
Zambia	Kaoma	2017	0.079	0.122	0.178
Zambia	Kaoma	2000-2017	-0.083	-0.071	-0.057
Zambia	Kapiri Mposhi	2000	0.258	0.395	0.574
Zambia	Kapiri Mposhi	2017	0.086	0.129	0.190
Zambia	Kapiri Mposhi	2000-2017	-0.076	-0.066	-0.055
Zambia	Kaputa	2000	0.251	0.384	0.550
Zambia	Kaputa	2017	0.100	0.148	0.218
Zambia	Kaputa	2000-2017	-0.066	-0.056	-0.046
Zambia	Kasama	2000	0.256	0.393	0.571
Zambia	Kasama	2017	0.095	0.145	0.219
Zambia	Kasama	2000-2017	-0.069	-0.058	-0.047
Zambia	Kasempa	2000	0.237	0.365	0.527

Table 1: LRI DALYs rate by unit *(continued)*

Country	Unit	year	mean rate	lower rate	upper rate
Zambia	Kasempa	2017	0.080	0.120	0.174
Zambia	Kasempa	2000-2017	-0.082	-0.070	-0.058
Zambia	Katete	2000	0.259	0.387	0.569
Zambia	Katete	2017	0.083	0.127	0.186
Zambia	Katete	2000-2017	-0.077	-0.068	-0.056
Zambia	Kawambwa	2000	0.266	0.410	0.588
Zambia	Kawambwa	2017	0.103	0.158	0.240
Zambia	Kawambwa	2000-2017	-0.068	-0.058	-0.048
Zambia	Kazungula	2000	0.213	0.330	0.476
Zambia	Kazungula	2017	0.087	0.130	0.191
Zambia	Kazungula	2000-2017	-0.066	-0.055	-0.044
Zambia	Kitwe	2000	0.266	0.424	0.631
Zambia	Kitwe	2017	0.108	0.168	0.251
Zambia	Kitwe	2000-2017	-0.067	-0.052	-0.036
Zambia	Livingstone	2000	0.217	0.340	0.505
Zambia	Livingstone	2017	0.087	0.133	0.191
Zambia	Livingstone	2000-2017	-0.077	-0.056	-0.037
Zambia	Luangwa	2000	0.260	0.382	0.563
Zambia	Luangwa	2017	0.084	0.123	0.176
Zambia	Luangwa	2000-2017	-0.077	-0.064	-0.052
Zambia	Luanshya	2000	0.273	0.432	0.666
Zambia	Luanshya	2017	0.107	0.165	0.239
Zambia	Luanshya	2000-2017	-0.073	-0.058	-0.039
Zambia	Lufwanyama	2000	0.245	0.382	0.557
Zambia	Lufwanyama	2017	0.085	0.130	0.184
Zambia	Lufwanyama	2000-2017	-0.080	-0.067	-0.052
Zambia	Lukulu	2000	0.269	0.413	0.594
Zambia	Lukulu	2017	0.082	0.124	0.180
Zambia	Lukulu	2000-2017	-0.086	-0.073	-0.057
Zambia	Lundazi	2000	0.269	0.405	0.578
Zambia	Lundazi	2017	0.087	0.133	0.189
Zambia	Lundazi	2000-2017	-0.078	-0.066	-0.054
Zambia	Lusaka	2000	0.258	0.425	0.654
Zambia	Lusaka	2017	0.102	0.156	0.229
Zambia	Lusaka	2000-2017	-0.074	-0.058	-0.041
Zambia	Luwingu	2000	0.241	0.373	0.546
Zambia	Luwingu	2017	0.092	0.139	0.200
Zambia	Luwingu	2000-2017	-0.067	-0.059	-0.047
Zambia	Mambwe	2000	0.254	0.393	0.572
Zambia	Mambwe	2017	0.083	0.127	0.190
Zambia	Mambwe	2000-2017	-0.081	-0.069	-0.056
Zambia	Mansa	2000	0.256	0.387	0.567
Zambia	Mansa	2017	0.106	0.160	0.241
Zambia	Mansa	2000-2017	-0.063	-0.055	-0.046
Zambia	Masaiti	2000	0.279	0.429	0.610
Zambia	Masaiti	2017	0.094	0.143	0.207
Zambia	Masaiti	2000-2017	-0.078	-0.066	-0.053
Zambia	Mazabuka	2000	0.234	0.365	0.523
Zambia	Mazabuka	2017	0.084	0.129	0.187
Zambia	Mazabuka	2000-2017	-0.072	-0.061	-0.050
Zambia	Mbala	2000	0.246	0.378	0.537
Zambia	Mbala	2017	0.106	0.157	0.232
Zambia	Mbala	2000-2017	-0.059	-0.050	-0.040
Zambia	Milenge	2000	0.250	0.378	0.554
Zambia	Milenge	2017	0.089	0.136	0.204
Zambia	Milenge	2000-2017	-0.073	-0.062	-0.050
Zambia	Mkushi	2000	0.254	0.390	0.560
Zambia	Mkushi	2017	0.084	0.126	0.182
Zambia	Mkushi	2000-2017	-0.076	-0.066	-0.055
Zambia	Mongu	2000	0.263	0.399	0.586
Zambia	Mongu	2017	0.082	0.124	0.182

Table 1: LRI DALYs rate by unit *(continued)*

Country	Unit	year	mean rate	lower rate	upper rate
Zambia	Mongu	2000-2017	-0.086	-0.071	-0.055
Zambia	Monze	2000	0.232	0.352	0.502
Zambia	Monze	2017	0.083	0.128	0.188
Zambia	Monze	2000-2017	-0.070	-0.059	-0.046
Zambia	Mpika	2000	0.253	0.384	0.558
Zambia	Mpika	2017	0.087	0.129	0.190
Zambia	Mpika	2000-2017	-0.077	-0.065	-0.051
Zambia	MPongwe	2000	0.248	0.384	0.548
Zambia	MPongwe	2017	0.086	0.129	0.189
Zambia	MPongwe	2000-2017	-0.076	-0.065	-0.052
Zambia	Mporokoso	2000	0.249	0.379	0.551
Zambia	Mporokoso	2017	0.094	0.141	0.208
Zambia	Mporokoso	2000-2017	-0.066	-0.058	-0.048
Zambia	Mpulungu	2000	0.242	0.375	0.546
Zambia	Mpulungu	2017	0.096	0.142	0.212
Zambia	Mpulungu	2000-2017	-0.067	-0.055	-0.045
Zambia	Mufulira	2000	0.272	0.434	0.621
Zambia	Mufulira	2017	0.108	0.166	0.243
Zambia	Mufulira	2000-2017	-0.074	-0.058	-0.043
Zambia	Mufumbwe	2000	0.261	0.399	0.570
Zambia	Mufumbwe	2017	0.079	0.121	0.174
Zambia	Mufumbwe	2000-2017	-0.083	-0.072	-0.059
Zambia	Mumbwa	2000	0.237	0.361	0.522
Zambia	Mumbwa	2017	0.083	0.126	0.186
Zambia	Mumbwa	2000-2017	-0.074	-0.064	-0.053
Zambia	Mungwi	2000	0.245	0.377	0.544
Zambia	Mungwi	2017	0.090	0.135	0.198
Zambia	Mungwi	2000-2017	-0.069	-0.059	-0.047
Zambia	Mwense	2000	0.278	0.424	0.613
Zambia	Mwense	2017	0.104	0.157	0.233
Zambia	Mwense	2000-2017	-0.069	-0.058	-0.048
Zambia	Mwinilunga	2000	0.259	0.395	0.572
Zambia	Mwinilunga	2017	0.082	0.125	0.184
Zambia	Mwinilunga	2000-2017	-0.082	-0.070	-0.056
Zambia	Nakonde	2000	0.235	0.349	0.495
Zambia	Nakonde	2017	0.088	0.129	0.190
Zambia	Nakonde	2000-2017	-0.069	-0.056	-0.040
Zambia	Namwala	2000	0.226	0.345	0.502
Zambia	Namwala	2017	0.082	0.127	0.188
Zambia	Namwala	2000-2017	-0.071	-0.060	-0.049
Zambia	Nchelenge	2000	0.290	0.447	0.637
Zambia	Nchelenge	2017	0.112	0.167	0.248
Zambia	Nchelenge	2000-2017	-0.070	-0.059	-0.046
Zambia	Ndola	2000	0.267	0.430	0.644
Zambia	Ndola	2017	0.112	0.168	0.242
Zambia	Ndola	2000-2017	-0.069	-0.054	-0.036
Zambia	Nyimba	2000	0.262	0.392	0.582
Zambia	Nyimba	2017	0.085	0.127	0.184
Zambia	Nyimba	2000-2017	-0.077	-0.067	-0.055
Zambia	Petauke	2000	0.263	0.396	0.568
Zambia	Petauke	2017	0.085	0.126	0.181
Zambia	Petauke	2000-2017	-0.079	-0.068	-0.055
Zambia	Samfya	2000	0.251	0.388	0.561
Zambia	Samfya	2017	0.094	0.142	0.206
Zambia	Samfya	2000-2017	-0.071	-0.061	-0.050
Zambia	Senanga	2000	0.262	0.403	0.596
Zambia	Senanga	2017	0.084	0.126	0.183
Zambia	Senanga	2000-2017	-0.081	-0.069	-0.054
Zambia	Serenje	2000	0.250	0.378	0.550
Zambia	Serenje	2017	0.083	0.125	0.181
Zambia	Serenje	2000-2017	-0.076	-0.066	-0.053

Table 1: LRI DALYs rate by unit *(continued)*

Country	Unit	year	mean rate	lower rate	upper rate
Zambia	Sesheke	2000	0.218	0.338	0.494
Zambia	Sesheke	2017	0.086	0.130	0.191
Zambia	Sesheke	2000-2017	-0.068	-0.057	-0.045
Zambia	Shangombo	2000	0.257	0.395	0.582
Zambia	Shangombo	2017	0.084	0.126	0.184
Zambia	Shangombo	2000-2017	-0.078	-0.067	-0.054
Zambia	Siavonga	2000	0.245	0.365	0.519
Zambia	Siavonga	2017	0.086	0.128	0.187
Zambia	Siavonga	2000-2017	-0.073	-0.061	-0.047
Zambia	Sinazongwe	2000	0.261	0.408	0.592
Zambia	Sinazongwe	2017	0.091	0.138	0.200
Zambia	Sinazongwe	2000-2017	-0.074	-0.062	-0.050
Zambia	Solwezi	2000	0.235	0.362	0.521
Zambia	Solwezi	2017	0.093	0.143	0.211
Zambia	Solwezi	2000-2017	-0.067	-0.057	-0.044
Zambia	Zambezi	2000	0.267	0.404	0.577
Zambia	Zambezi	2017	0.081	0.123	0.180
Zambia	Zambezi	2000-2017	-0.083	-0.070	-0.056
Zimbabwe	Beitbridge	2000	0.140	0.215	0.309
Zimbabwe	Beitbridge	2017	0.173	0.257	0.359
Zimbabwe	Beitbridge	2000-2017	-0.001	0.011	0.025
Zimbabwe	Bikita	2000	0.113	0.179	0.255
Zimbabwe	Bikita	2017	0.143	0.213	0.302
Zimbabwe	Bikita	2000-2017	-0.004	0.011	0.022
Zimbabwe	Bindura	2000	0.121	0.192	0.272
Zimbabwe	Bindura	2017	0.151	0.228	0.333
Zimbabwe	Bindura	2000-2017	-0.003	0.011	0.023
Zimbabwe	Binga	2000	0.111	0.170	0.244
Zimbabwe	Binga	2017	0.136	0.200	0.274
Zimbabwe	Binga	2000-2017	0.000	0.011	0.021
Zimbabwe	Bubi	2000	0.109	0.173	0.239
Zimbabwe	Bubi	2017	0.140	0.206	0.292
Zimbabwe	Bubi	2000-2017	-0.002	0.011	0.023
Zimbabwe	Buhera	2000	0.111	0.178	0.255
Zimbabwe	Buhera	2017	0.145	0.212	0.296
Zimbabwe	Buhera	2000-2017	-0.002	0.012	0.025
Zimbabwe	Bulawayo	2000	0.105	0.168	0.243
Zimbabwe	Bulawayo	2017	0.135	0.200	0.285
Zimbabwe	Bulawayo	2000-2017	-0.006	0.012	0.030
Zimbabwe	Bulilima (North)	2000	0.126	0.199	0.281
Zimbabwe	Bulilima (North)	2017	0.160	0.237	0.330
Zimbabwe	Bulilima (North)	2000-2017	0.000	0.011	0.024
Zimbabwe	Centenary	2000	0.119	0.189	0.270
Zimbabwe	Centenary	2017	0.153	0.224	0.319
Zimbabwe	Centenary	2000-2017	0.000	0.011	0.023
Zimbabwe	Chegutu	2000	0.115	0.184	0.275
Zimbabwe	Chegutu	2017	0.145	0.221	0.314
Zimbabwe	Chegutu	2000-2017	-0.003	0.012	0.026
Zimbabwe	Chikomba	2000	0.111	0.178	0.260
Zimbabwe	Chikomba	2017	0.144	0.213	0.301
Zimbabwe	Chikomba	2000-2017	-0.001	0.012	0.025
Zimbabwe	Chimanimani	2000	0.119	0.188	0.264
Zimbabwe	Chimanimani	2017	0.148	0.218	0.301
Zimbabwe	Chimanimani	2000-2017	-0.005	0.010	0.020
Zimbabwe	Chipinge	2000	0.116	0.180	0.251
Zimbabwe	Chipinge	2017	0.144	0.213	0.299
Zimbabwe	Chipinge	2000-2017	-0.001	0.011	0.022
Zimbabwe	Chiredzi	2000	0.115	0.179	0.252
Zimbabwe	Chiredzi	2017	0.147	0.217	0.303
Zimbabwe	Chiredzi	2000-2017	-0.001	0.012	0.022
Zimbabwe	Chirumhanzu	2000	0.106	0.174	0.254

Table 1: LRI DALYs rate by unit *(continued)*

Country	Unit	year	mean rate	lower rate	upper rate
Zimbabwe	Chirumhanzu	2017	0.142	0.207	0.292
Zimbabwe	Chirumhanzu	2000-2017	-0.002	0.012	0.024
Zimbabwe	Chivi	2000	0.111	0.175	0.253
Zimbabwe	Chivi	2017	0.141	0.210	0.295
Zimbabwe	Chivi	2000-2017	-0.001	0.012	0.023
Zimbabwe	Gokwe North	2000	0.114	0.179	0.256
Zimbabwe	Gokwe North	2017	0.140	0.207	0.287
Zimbabwe	Gokwe North	2000-2017	-0.002	0.010	0.021
Zimbabwe	Gokwe South	2000	0.111	0.173	0.251
Zimbabwe	Gokwe South	2017	0.139	0.204	0.282
Zimbabwe	Gokwe South	2000-2017	-0.001	0.011	0.024
Zimbabwe	Goromonzi	2000	0.118	0.191	0.274
Zimbabwe	Goromonzi	2017	0.154	0.227	0.320
Zimbabwe	Goromonzi	2000-2017	-0.001	0.011	0.025
Zimbabwe	Guruve	2000	0.116	0.183	0.263
Zimbabwe	Guruve	2017	0.149	0.217	0.304
Zimbabwe	Guruve	2000-2017	0.000	0.011	0.022
Zimbabwe	Gutu	2000	0.110	0.176	0.256
Zimbabwe	Gutu	2017	0.143	0.210	0.296
Zimbabwe	Gutu	2000-2017	-0.001	0.012	0.023
Zimbabwe	Gwanda	2000	0.133	0.204	0.286
Zimbabwe	Gwanda	2017	0.164	0.243	0.339
Zimbabwe	Gwanda	2000-2017	-0.001	0.011	0.023
Zimbabwe	Gweru	2000	0.101	0.164	0.236
Zimbabwe	Gweru	2017	0.128	0.197	0.288
Zimbabwe	Gweru	2000-2017	-0.004	0.012	0.027
Zimbabwe	Harare	2000	0.108	0.175	0.252
Zimbabwe	Harare	2017	0.139	0.206	0.293
Zimbabwe	Harare	2000-2017	-0.003	0.011	0.025
Zimbabwe	Hurungwe	2000	0.113	0.177	0.252
Zimbabwe	Hurungwe	2017	0.141	0.209	0.291
Zimbabwe	Hurungwe	2000-2017	-0.001	0.011	0.022
Zimbabwe	Hwange	2000	0.122	0.182	0.260
Zimbabwe	Hwange	2017	0.145	0.207	0.279
Zimbabwe	Hwange	2000-2017	-0.007	0.007	0.020
Zimbabwe	Insiza	2000	0.127	0.200	0.283
Zimbabwe	Insiza	2017	0.158	0.238	0.338
Zimbabwe	Insiza	2000-2017	-0.001	0.011	0.023
Zimbabwe	Kadoma	2000	0.118	0.187	0.276
Zimbabwe	Kadoma	2017	0.152	0.225	0.319
Zimbabwe	Kadoma	2000-2017	-0.001	0.012	0.024
Zimbabwe	Kariba	2000	0.130	0.192	0.265
Zimbabwe	Kariba	2017	0.149	0.214	0.297
Zimbabwe	Kariba	2000-2017	-0.010	0.005	0.020
Zimbabwe	Kwekwe	2000	0.106	0.171	0.249
Zimbabwe	Kwekwe	2017	0.139	0.206	0.291
Zimbabwe	Kwekwe	2000-2017	-0.002	0.012	0.026
Zimbabwe	Lupane	2000	0.111	0.171	0.243
Zimbabwe	Lupane	2017	0.138	0.205	0.284
Zimbabwe	Lupane	2000-2017	0.000	0.011	0.022
Zimbabwe	Makonde	2000	0.116	0.188	0.272
Zimbabwe	Makonde	2017	0.148	0.227	0.325
Zimbabwe	Makonde	2000-2017	0.000	0.012	0.025
Zimbabwe	Makoni	2000	0.114	0.181	0.261
Zimbabwe	Makoni	2017	0.147	0.216	0.299
Zimbabwe	Makoni	2000-2017	-0.002	0.012	0.023
Zimbabwe	Mangwe (South)	2000	0.127	0.200	0.282
Zimbabwe	Mangwe (South)	2017	0.158	0.237	0.333
Zimbabwe	Mangwe (South)	2000-2017	-0.002	0.011	0.023
Zimbabwe	Marondera	2000	0.112	0.183	0.276
Zimbabwe	Marondera	2017	0.151	0.220	0.303

Table 1: LRI DALYs rate by unit *(continued)*

Country	Unit	year	mean rate	lower rate	upper rate
Zimbabwe	Marondera	2000-2017	-0.002	0.012	0.026
Zimbabwe	Masvingo	2000	0.108	0.171	0.242
Zimbabwe	Masvingo	2017	0.138	0.204	0.277
Zimbabwe	Masvingo	2000-2017	-0.004	0.012	0.026
Zimbabwe	Matobo	2000	0.127	0.203	0.285
Zimbabwe	Matobo	2017	0.160	0.241	0.345
Zimbabwe	Matobo	2000-2017	-0.003	0.011	0.026
Zimbabwe	Mazowe	2000	0.120	0.195	0.284
Zimbabwe	Mazowe	2017	0.157	0.230	0.319
Zimbabwe	Mazowe	2000-2017	-0.001	0.011	0.022
Zimbabwe	Mberengwa	2000	0.109	0.175	0.255
Zimbabwe	Mberengwa	2017	0.138	0.208	0.297
Zimbabwe	Mberengwa	2000-2017	-0.001	0.011	0.024
Zimbabwe	Mount Darwin	2000	0.117	0.187	0.261
Zimbabwe	Mount Darwin	2017	0.148	0.219	0.311
Zimbabwe	Mount Darwin	2000-2017	-0.001	0.011	0.021
Zimbabwe	Mudzi	2000	0.115	0.181	0.257
Zimbabwe	Mudzi	2017	0.144	0.214	0.299
Zimbabwe	Mudzi	2000-2017	0.000	0.011	0.022
Zimbabwe	Murehwa	2000	0.115	0.183	0.268
Zimbabwe	Murehwa	2017	0.148	0.218	0.309
Zimbabwe	Murehwa	2000-2017	-0.001	0.011	0.022
Zimbabwe	Mutare	2000	0.115	0.178	0.252
Zimbabwe	Mutare	2017	0.146	0.212	0.293
Zimbabwe	Mutare	2000-2017	-0.002	0.011	0.026
Zimbabwe	Mutasa	2000	0.120	0.190	0.267
Zimbabwe	Mutasa	2017	0.152	0.218	0.305
Zimbabwe	Mutasa	2000-2017	-0.005	0.009	0.022
Zimbabwe	Mutoko	2000	0.111	0.178	0.255
Zimbabwe	Mutoko	2017	0.142	0.212	0.297
Zimbabwe	Mutoko	2000-2017	-0.001	0.011	0.024
Zimbabwe	Mwenezi	2000	0.116	0.182	0.258
Zimbabwe	Mwenezi	2017	0.149	0.219	0.309
Zimbabwe	Mwenezi	2000-2017	-0.001	0.012	0.023
Zimbabwe	Nkayi	2000	0.110	0.171	0.245
Zimbabwe	Nkayi	2017	0.141	0.205	0.286
Zimbabwe	Nkayi	2000-2017	0.000	0.012	0.022
Zimbabwe	Nyanga	2000	0.117	0.186	0.265
Zimbabwe	Nyanga	2017	0.148	0.217	0.306
Zimbabwe	Nyanga	2000-2017	-0.001	0.010	0.022
Zimbabwe	Rushinga	2000	0.118	0.191	0.277
Zimbabwe	Rushinga	2017	0.150	0.221	0.310
Zimbabwe	Rushinga	2000-2017	-0.002	0.010	0.022
Zimbabwe	Seke	2000	0.107	0.175	0.261
Zimbabwe	Seke	2017	0.143	0.209	0.293
Zimbabwe	Seke	2000-2017	-0.004	0.011	0.025
Zimbabwe	Shamva	2000	0.117	0.189	0.271
Zimbabwe	Shamva	2017	0.153	0.224	0.313
Zimbabwe	Shamva	2000-2017	-0.002	0.011	0.023
Zimbabwe	Shurugwi	2000	0.110	0.175	0.252
Zimbabwe	Shurugwi	2017	0.137	0.208	0.294
Zimbabwe	Shurugwi	2000-2017	-0.002	0.011	0.024
Zimbabwe	Tsholotsho	2000	0.107	0.172	0.244
Zimbabwe	Tsholotsho	2017	0.138	0.206	0.287
Zimbabwe	Tsholotsho	2000-2017	-0.001	0.011	0.023
Zimbabwe	Umguza	2000	0.108	0.172	0.241
Zimbabwe	Umguza	2017	0.139	0.207	0.292
Zimbabwe	Umguza	2000-2017	-0.003	0.012	0.026
Zimbabwe	UMP	2000	0.113	0.180	0.258
Zimbabwe	UMP	2017	0.145	0.214	0.295
Zimbabwe	UMP	2000-2017	-0.001	0.011	0.022

Table 1: LRI DALYs rate by unit *(continued)*

Country	Unit	year	mean rate	lower rate	upper rate
Zimbabwe	Umzingwane	2000	0.126	0.199	0.290
Zimbabwe	Umzingwane	2017	0.159	0.238	0.342
Zimbabwe	Umzingwane	2000-2017	-0.003	0.011	0.024
Zimbabwe	Wedza	2000	0.113	0.178	0.256
Zimbabwe	Wedza	2017	0.143	0.213	0.299
Zimbabwe	Wedza	2000-2017	0.000	0.012	0.023
Zimbabwe	Zaka	2000	0.113	0.175	0.247
Zimbabwe	Zaka	2017	0.141	0.209	0.291
Zimbabwe	Zaka	2000-2017	-0.002	0.012	0.025
Zimbabwe	Zvimba	2000	0.118	0.189	0.268
Zimbabwe	Zvimba	2017	0.154	0.225	0.318
Zimbabwe	Zvimba	2000-2017	0.001	0.012	0.024
Zimbabwe	Zvishavane	2000	0.106	0.169	0.253
Zimbabwe	Zvishavane	2017	0.135	0.201	0.276
Zimbabwe	Zvishavane	2000-2017	-0.005	0.012	0.027

Table 2: Diarrhea DALYs rate by unit

Country	Unit	year	mean rate	lower rate	upper rate
**North Africa and Middle East**					
Sudan	Abu Hamad	2000	0.214	0.292	0.382
Sudan	Abu Hamad	2017	0.059	0.091	0.134
Sudan	Abu Hamad	2000-2017	-0.115	-0.073	-0.026
Sudan	Abu Jubaiyah	2000	0.185	0.247	0.316
Sudan	Abu Jubaiyah	2017	0.062	0.094	0.137
Sudan	Abu Jubaiyah	2000-2017	-0.105	-0.059	-0.011
Sudan	Abyei	2000	0.211	0.280	0.376
Sudan	Abyei	2017	0.069	0.099	0.135
Sudan	Abyei	2000-2017	-0.098	-0.052	-0.004
Sudan	Ad Damazin	2000	0.327	0.414	0.508
Sudan	Ad Damazin	2017	0.070	0.100	0.143
Sudan	Ad Damazin	2000-2017	-0.128	-0.084	-0.036
Sudan	Ad Damer	2000	0.233	0.318	0.414
Sudan	Ad Damer	2017	0.064	0.094	0.143
Sudan	Ad Damer	2000-2017	-0.125	-0.078	-0.029
Sudan	Ad Dinder	2000	0.272	0.360	0.454
Sudan	Ad Dinder	2017	0.072	0.103	0.147
Sudan	Ad Dinder	2000-2017	-0.114	-0.073	-0.030
Sudan	Ad Douiem	2000	0.236	0.310	0.390
Sudan	Ad Douiem	2017	0.082	0.121	0.172
Sudan	Ad Douiem	2000-2017	-0.110	-0.055	-0.010
Sudan	Addabah	2000	0.210	0.283	0.383
Sudan	Addabah	2017	0.069	0.102	0.149
Sudan	Addabah	2000-2017	-0.123	-0.075	-0.032
Sudan	Al Deain	2000	0.245	0.321	0.416
Sudan	Al Deain	2017	0.078	0.113	0.153
Sudan	Al Deain	2000-2017	-0.093	-0.047	-0.004
Sudan	Al Fasher	2000	0.215	0.280	0.363
Sudan	Al Fasher	2017	0.072	0.109	0.160
Sudan	Al Fasher	2000-2017	-0.091	-0.050	-0.005
Sudan	Al Faw	2000	0.290	0.375	0.462
Sudan	Al Faw	2017	0.084	0.122	0.170
Sudan	Al Faw	2000-2017	-0.101	-0.060	-0.013
Sudan	Al Fushqa	2000	0.285	0.375	0.478
Sudan	Al Fushqa	2017	0.081	0.120	0.170
Sudan	Al Fushqa	2000-2017	-0.103	-0.060	-0.017
Sudan	Al Gadaref	2000	0.266	0.367	0.454
Sudan	Al Gadaref	2017	0.075	0.113	0.166
Sudan	Al Gadaref	2000-2017	-0.105	-0.061	-0.011
Sudan	Al Galabat	2000	0.291	0.387	0.485
Sudan	Al Galabat	2017	0.075	0.110	0.161
Sudan	Al Galabat	2000-2017	-0.115	-0.067	-0.018
Sudan	Al Gash	2000	0.227	0.295	0.379
Sudan	Al Gash	2017	0.071	0.108	0.151
Sudan	Al Gash	2000-2017	-0.106	-0.064	-0.023
Sudan	Al Geneina	2000	0.249	0.329	0.415
Sudan	Al Geneina	2017	0.071	0.103	0.146
Sudan	Al Geneina	2000-2017	-0.102	-0.056	-0.014
Sudan	Al Gutaina	2000	0.245	0.315	0.407
Sudan	Al Gutaina	2017	0.089	0.126	0.180
Sudan	Al Gutaina	2000-2017	-0.111	-0.054	-0.010
Sudan	Al Jabalian	2000	0.253	0.325	0.420
Sudan	Al Jabalian	2017	0.075	0.113	0.162
Sudan	Al Jabalian	2000-2017	-0.123	-0.066	-0.016
Sudan	Al Kamlin	2000	0.239	0.311	0.402
Sudan	Al Kamlin	2017	0.086	0.122	0.177
Sudan	Al Kamlin	2000-2017	-0.104	-0.058	-0.015
Sudan	Al Kurumik	2000	0.334	0.423	0.538
Sudan	Al Kurumik	2017	0.064	0.096	0.135
Sudan	Al Kurumik	2000-2017	-0.118	-0.071	-0.030

Table 2: Diarrhea DALYs rate by unit *(continued)*

Country	Unit	year	mean rate	lower rate	upper rate
Sudan	Al Mahagil	2000	0.218	0.288	0.373
Sudan	Al Mahagil	2017	0.075	0.115	0.164
Sudan	Al Mahagil	2000-2017	-0.113	-0.056	-0.011
Sudan	Al Matammah	2000	0.239	0.315	0.400
Sudan	Al Matammah	2017	0.071	0.106	0.152
Sudan	Al Matammah	2000-2017	-0.117	-0.068	-0.022
Sudan	Al Rahd	2000	0.278	0.369	0.461
Sudan	Al Rahd	2017	0.077	0.113	0.164
Sudan	Al Rahd	2000-2017	-0.108	-0.062	-0.019
Sudan	Al Roseires	2000	0.355	0.461	0.571
Sudan	Al Roseires	2017	0.067	0.099	0.140
Sudan	Al Roseires	2000-2017	-0.122	-0.078	-0.032
Sudan	As Salam	2000	0.196	0.263	0.349
Sudan	As Salam	2017	0.059	0.095	0.136
Sudan	As Salam	2000-2017	-0.093	-0.052	-0.006
Sudan	Atbara	2000	0.232	0.311	0.406
Sudan	Atbara	2017	0.062	0.094	0.145
Sudan	Atbara	2000-2017	-0.120	-0.076	-0.030
Sudan	Bara	2000	0.214	0.283	0.372
Sudan	Bara	2017	0.066	0.100	0.145
Sudan	Bara	2000-2017	-0.104	-0.052	0.000
Sudan	Baw	2000	0.355	0.442	0.561
Sudan	Baw	2017	0.068	0.099	0.142
Sudan	Baw	2000-2017	-0.114	-0.070	-0.025
Sudan	Berber	2000	0.222	0.312	0.414
Sudan	Berber	2017	0.058	0.091	0.140
Sudan	Berber	2000-2017	-0.127	-0.079	-0.032
Sudan	Buram	2000	0.266	0.342	0.429
Sudan	Buram	2017	0.081	0.118	0.163
Sudan	Buram	2000-2017	-0.093	-0.048	-0.005
Sudan	Dilling	2000	0.173	0.226	0.292
Sudan	Dilling	2017	0.060	0.089	0.125
Sudan	Dilling	2000-2017	-0.096	-0.050	-0.002
Sudan	Dongola	2000	0.193	0.264	0.352
Sudan	Dongola	2017	0.063	0.098	0.141
Sudan	Dongola	2000-2017	-0.116	-0.069	-0.018
Sudan	East al Gazera	2000	0.227	0.301	0.381
Sudan	East al Gazera	2017	0.073	0.109	0.153
Sudan	East al Gazera	2000-2017	-0.114	-0.064	-0.018
Sudan	En Nuhud	2000	0.199	0.267	0.347
Sudan	En Nuhud	2017	0.062	0.096	0.140
Sudan	En Nuhud	2000-2017	-0.102	-0.051	-0.003
Sudan	Geissan	2000	0.384	0.479	0.590
Sudan	Geissan	2017	0.065	0.097	0.142
Sudan	Geissan	2000-2017	-0.119	-0.074	-0.026
Sudan	Ghebeish	2000	0.202	0.274	0.356
Sudan	Ghebeish	2017	0.067	0.101	0.143
Sudan	Ghebeish	2000-2017	-0.094	-0.046	0.004
Sudan	Halayeb	2000	0.177	0.261	0.353
Sudan	Halayeb	2017	0.058	0.088	0.131
Sudan	Halayeb	2000-2017	-0.119	-0.070	-0.030
Sudan	Hamashkorieb	2000	0.208	0.274	0.364
Sudan	Hamashkorieb	2017	0.066	0.104	0.145
Sudan	Hamashkorieb	2000-2017	-0.105	-0.060	-0.012
Sudan	Id El Ghanem	2000	0.257	0.343	0.432
Sudan	Id El Ghanem	2017	0.083	0.119	0.174
Sudan	Id El Ghanem	2000-2017	-0.093	-0.046	-0.003
Sudan	Jebrat al Sheikh	2000	0.257	0.331	0.427
Sudan	Jebrat al Sheikh	2017	0.087	0.124	0.176
Sudan	Jebrat al Sheikh	2000-2017	-0.105	-0.051	-0.003
Sudan	Kabkabiya	2000	0.223	0.298	0.384

Table 2: Diarrhea DALYs rate by unit *(continued)*

Country	Unit	year	mean rate	lower rate	upper rate
Sudan	Kabkabiya	2017	0.073	0.107	0.153
Sudan	Kabkabiya	2000-2017	-0.096	-0.052	-0.006
Sudan	Kadugli	2000	0.169	0.224	0.288
Sudan	Kadugli	2017	0.058	0.091	0.129
Sudan	Kadugli	2000-2017	-0.106	-0.054	-0.011
Sudan	Karary	2000	0.250	0.321	0.409
Sudan	Karary	2017	0.082	0.120	0.175
Sudan	Karary	2000-2017	-0.114	-0.063	-0.017
Sudan	Kas	2000	0.235	0.329	0.418
Sudan	Kas	2017	0.077	0.116	0.167
Sudan	Kas	2000-2017	-0.098	-0.051	-0.005
Sudan	Kassala	2000	0.231	0.300	0.391
Sudan	Kassala	2017	0.074	0.110	0.161
Sudan	Kassala	2000-2017	-0.108	-0.062	-0.010
Sudan	Khartoum	2000	0.252	0.326	0.421
Sudan	Khartoum	2017	0.089	0.131	0.189
Sudan	Khartoum	2000-2017	-0.113	-0.062	-0.013
Sudan	Khartoum Bahri	2000	0.253	0.325	0.419
Sudan	Khartoum Bahri	2017	0.084	0.126	0.183
Sudan	Khartoum Bahri	2000-2017	-0.110	-0.063	-0.017
Sudan	Kosti	2000	0.238	0.309	0.400
Sudan	Kosti	2017	0.076	0.112	0.156
Sudan	Kosti	2000-2017	-0.120	-0.063	-0.014
Sudan	Kutum	2000	0.212	0.285	0.374
Sudan	Kutum	2017	0.066	0.100	0.146
Sudan	Kutum	2000-2017	-0.098	-0.059	-0.015
Sudan	Lagawa	2000	0.184	0.247	0.320
Sudan	Lagawa	2017	0.061	0.093	0.127
Sudan	Lagawa	2000-2017	-0.091	-0.050	-0.003
Sudan	Mellit	2000	0.204	0.277	0.363
Sudan	Mellit	2017	0.074	0.107	0.157
Sudan	Mellit	2000-2017	-0.094	-0.051	-0.008
Sudan	Merawi	2000	0.220	0.295	0.386
Sudan	Merawi	2017	0.068	0.101	0.152
Sudan	Merawi	2000-2017	-0.120	-0.075	-0.024
Sudan	Mukjar	2000	0.254	0.335	0.428
Sudan	Mukjar	2017	0.081	0.116	0.167
Sudan	Mukjar	2000-2017	-0.096	-0.049	-0.012
Sudan	Nahr Atbara	2000	0.233	0.303	0.380
Sudan	Nahr Atbara	2017	0.073	0.110	0.159
Sudan	Nahr Atbara	2000-2017	-0.102	-0.058	-0.008
Sudan	North al Gazera	2000	0.223	0.293	0.378
Sudan	North al Gazera	2017	0.080	0.114	0.166
Sudan	North al Gazera	2000-2017	-0.111	-0.058	-0.016
Sudan	Nyala	2000	0.250	0.328	0.417
Sudan	Nyala	2000	0.254	0.339	0.434
Sudan	Nyala	2017	0.079	0.118	0.173
Sudan	Nyala	2017	0.081	0.119	0.169
Sudan	Nyala	2000-2017	-0.098	-0.054	-0.010
Sudan	Nyala	2000-2017	-0.104	-0.055	-0.012
Sudan	Omdurman	2000	0.254	0.328	0.419
Sudan	Omdurman	2017	0.086	0.127	0.184
Sudan	Omdurman	2000-2017	-0.111	-0.061	-0.010
Sudan	Port Sudan	2000	0.171	0.231	0.327
Sudan	Port Sudan	2017	0.051	0.080	0.120
Sudan	Port Sudan	2000-2017	-0.120	-0.072	-0.023
Sudan	Rashad	2000	0.192	0.252	0.333
Sudan	Rashad	2017	0.067	0.099	0.142
Sudan	Rashad	2000-2017	-0.105	-0.053	-0.001
Sudan	Sennar	2000	0.265	0.344	0.429
Sudan	Sennar	2017	0.078	0.115	0.163

Table 2: Diarrhea DALYs rate by unit *(continued)*

Country	Unit	year	mean rate	lower rate	upper rate
Sudan	Sennar	2000-2017	-0.115	-0.065	-0.021
Sudan	Seteet	2000	0.234	0.305	0.382
Sudan	Seteet	2017	0.073	0.109	0.155
Sudan	Seteet	2000-2017	-0.098	-0.058	-0.015
Sudan	Sharg En Nile	2000	0.255	0.328	0.424
Sudan	Sharg En Nile	2017	0.086	0.127	0.184
Sudan	Sharg En Nile	2000-2017	-0.104	-0.060	-0.015
Sudan	Sharq al Gazera	2000	0.227	0.302	0.386
Sudan	Sharq al Gazera	2017	0.078	0.115	0.170
Sudan	Sharq al Gazera	2000-2017	-0.105	-0.059	-0.014
Sudan	Sheikan	2000	0.220	0.297	0.381
Sudan	Sheikan	2017	0.070	0.105	0.152
Sudan	Sheikan	2000-2017	-0.099	-0.054	-0.005
Sudan	Shendi	2000	0.239	0.313	0.391
Sudan	Shendi	2017	0.069	0.106	0.155
Sudan	Shendi	2000-2017	-0.113	-0.068	-0.024
Sudan	Singa	2000	0.268	0.355	0.450
Sudan	Singa	2017	0.072	0.106	0.151
Sudan	Singa	2000-2017	-0.118	-0.072	-0.024
Sudan	Sinkat	2000	0.188	0.250	0.338
Sudan	Sinkat	2017	0.059	0.088	0.122
Sudan	Sinkat	2000-2017	-0.108	-0.068	-0.025
Sudan	South al Gazera	2000	0.221	0.291	0.380
Sudan	South al Gazera	2017	0.073	0.114	0.167
Sudan	South al Gazera	2000-2017	-0.116	-0.058	-0.016
Sudan	South Khartoum	2000	0.256	0.326	0.427
Sudan	South Khartoum	2017	0.090	0.131	0.192
Sudan	South Khartoum	2000-2017	-0.105	-0.057	-0.013
Sudan	Sowdari	2000	0.214	0.291	0.376
Sudan	Sowdari	2017	0.071	0.104	0.148
Sudan	Sowdari	2000-2017	-0.097	-0.051	-0.006
Sudan	Talodi	2000	0.171	0.230	0.307
Sudan	Talodi	2017	0.060	0.091	0.134
Sudan	Talodi	2000-2017	-0.099	-0.053	-0.007
Sudan	Tokar	2000	0.177	0.238	0.318
Sudan	Tokar	2017	0.056	0.087	0.121
Sudan	Tokar	2000-2017	-0.117	-0.070	-0.024
Sudan	Tulus	2000	0.248	0.338	0.429
Sudan	Tulus	2017	0.080	0.119	0.176
Sudan	Tulus	2000-2017	-0.094	-0.046	0.002
Sudan	Um Al Gura	2000	0.231	0.302	0.386
Sudan	Um Al Gura	2017	0.075	0.110	0.155
Sudan	Um Al Gura	2000-2017	-0.119	-0.066	-0.026
Sudan	Um Badda	2000	0.258	0.328	0.427
Sudan	Um Badda	2017	0.089	0.132	0.191
Sudan	Um Badda	2000-2017	-0.111	-0.058	-0.007
Sudan	Um Kadada	2000	0.219	0.283	0.373
Sudan	Um Kadada	2017	0.072	0.108	0.153
Sudan	Um Kadada	2000-2017	-0.094	-0.044	0.001
Sudan	Um Rawaba	2000	0.229	0.301	0.399
Sudan	Um Rawaba	2017	0.071	0.108	0.145
Sudan	Um Rawaba	2000-2017	-0.105	-0.053	-0.004
Sudan	Wadi Halfa	2000	0.192	0.265	0.354
Sudan	Wadi Halfa	2017	0.065	0.095	0.135
Sudan	Wadi Halfa	2000-2017	-0.117	-0.074	-0.024
Sudan	Zallingi	2000	0.245	0.320	0.411
Sudan	Zallingi	2017	0.075	0.111	0.157
Sudan	Zallingi	2000-2017	-0.092	-0.050	-0.009
**Sub-Saharan Africa**					
Angola	Alto Cauale	2000	0.430	0.573	0.761
Angola	Alto Cauale	2017	0.130	0.168	0.213

Table 2: Diarrhea DALYs rate by unit *(continued)*

Country	Unit	year	mean rate	lower rate	upper rate
Angola	Alto Cauale	2000-2017	-0.090	-0.058	-0.023
Angola	Alto Zambeze	2000	0.478	0.612	0.769
Angola	Alto Zambeze	2017	0.151	0.181	0.221
Angola	Alto Zambeze	2000-2017	-0.095	-0.069	-0.043
Angola	Ambaca	2000	0.409	0.573	0.741
Angola	Ambaca	2017	0.140	0.177	0.222
Angola	Ambaca	2000-2017	-0.097	-0.060	-0.025
Angola	Amboim	2000	0.601	0.781	0.992
Angola	Amboim	2017	0.174	0.224	0.281
Angola	Amboim	2000-2017	-0.100	-0.066	-0.033
Angola	Ambriz	2000	0.499	0.659	0.865
Angola	Ambriz	2017	0.167	0.206	0.264
Angola	Ambriz	2000-2017	-0.100	-0.064	-0.029
Angola	Ambuila	2000	0.431	0.582	0.760
Angola	Ambuila	2017	0.135	0.172	0.220
Angola	Ambuila	2000-2017	-0.092	-0.058	-0.026
Angola	Andulo	2000	0.498	0.658	0.856
Angola	Andulo	2017	0.140	0.176	0.220
Angola	Andulo	2000-2017	-0.103	-0.069	-0.034
Angola	Baía Farta	2000	0.582	0.828	1.065
Angola	Baía Farta	2017	0.186	0.240	0.305
Angola	Baía Farta	2000-2017	-0.086	-0.057	-0.025
Angola	Bailundo	2000	0.610	0.809	1.048
Angola	Bailundo	2017	0.167	0.211	0.258
Angola	Bailundo	2000-2017	-0.101	-0.068	-0.036
Angola	Balombo	2000	0.599	0.809	1.050
Angola	Balombo	2017	0.163	0.215	0.272
Angola	Balombo	2000-2017	-0.098	-0.063	-0.031
Angola	Banga	2000	0.448	0.626	0.824
Angola	Banga	2017	0.144	0.188	0.229
Angola	Banga	2000-2017	-0.110	-0.073	-0.039
Angola	Belize	2000	0.509	0.651	0.821
Angola	Belize	2017	0.136	0.175	0.217
Angola	Belize	2000-2017	-0.105	-0.076	-0.042
Angola	Bembe	2000	0.424	0.590	0.781
Angola	Bembe	2017	0.141	0.180	0.221
Angola	Bembe	2000-2017	-0.084	-0.056	-0.028
Angola	Benguela	2000	0.560	0.815	1.078
Angola	Benguela	2017	0.172	0.222	0.285
Angola	Benguela	2000-2017	-0.088	-0.054	-0.022
Angola	Bibala	2000	0.487	0.636	0.838
Angola	Bibala	2017	0.163	0.210	0.263
Angola	Bibala	2000-2017	-0.100	-0.068	-0.036
Angola	Bocoio	2000	0.571	0.781	1.010
Angola	Bocoio	2017	0.164	0.211	0.265
Angola	Bocoio	2000-2017	-0.096	-0.068	-0.039
Angola	Bolongongo	2000	0.413	0.602	0.778
Angola	Bolongongo	2017	0.139	0.179	0.220
Angola	Bolongongo	2000-2017	-0.094	-0.057	-0.023
Angola	Buco Zau	2000	0.485	0.626	0.787
Angola	Buco Zau	2017	0.136	0.175	0.215
Angola	Buco Zau	2000-2017	-0.096	-0.067	-0.037
Angola	Buengas	2000	0.431	0.552	0.711
Angola	Buengas	2017	0.130	0.170	0.222
Angola	Buengas	2000-2017	-0.090	-0.056	-0.021
Angola	Bula Atumba	2000	0.443	0.620	0.801
Angola	Bula Atumba	2017	0.138	0.177	0.218
Angola	Bula Atumba	2000-2017	-0.086	-0.051	-0.017
Angola	Bungo	2000	0.404	0.537	0.717
Angola	Bungo	2017	0.138	0.178	0.227
Angola	Bungo	2000-2017	-0.076	-0.045	-0.012

Table 2: Diarrhea DALYs rate by unit *(continued)*

Country	Unit	year	mean rate	lower rate	upper rate
Angola	Caála	2000	0.500	0.664	0.851
Angola	Caála	2017	0.153	0.194	0.248
Angola	Caála	2000-2017	-0.099	-0.067	-0.035
Angola	Cabinda	2000	0.459	0.606	0.778
Angola	Cabinda	2017	0.125	0.161	0.206
Angola	Cabinda	2000-2017	-0.104	-0.069	-0.037
Angola	Cacolo	2000	0.453	0.609	0.751
Angola	Cacolo	2017	0.138	0.178	0.221
Angola	Cacolo	2000-2017	-0.099	-0.066	-0.033
Angola	Caconda	2000	0.460	0.647	0.837
Angola	Caconda	2017	0.146	0.195	0.245
Angola	Caconda	2000-2017	-0.097	-0.062	-0.029
Angola	Cacuaco	2000	0.435	0.601	0.809
Angola	Cacuaco	2017	0.125	0.163	0.210
Angola	Cacuaco	2000-2017	-0.109	-0.070	-0.032
Angola	Cacuzo	2000	0.590	0.781	1.019
Angola	Cacuzo	2017	0.180	0.223	0.275
Angola	Cacuzo	2000-2017	-0.102	-0.065	-0.032
Angola	Cahama	2000	0.443	0.624	0.836
Angola	Cahama	2017	0.143	0.181	0.239
Angola	Cahama	2000-2017	-0.100	-0.067	-0.030
Angola	Caiambambo	2000	0.533	0.769	0.971
Angola	Caiambambo	2017	0.153	0.200	0.255
Angola	Caiambambo	2000-2017	-0.099	-0.069	-0.038
Angola	Calai	2000	0.528	0.697	0.866
Angola	Calai	2017	0.167	0.210	0.273
Angola	Calai	2000-2017	-0.088	-0.057	-0.026
Angola	Calandula	2000	0.534	0.710	0.917
Angola	Calandula	2017	0.168	0.210	0.262
Angola	Calandula	2000-2017	-0.104	-0.068	-0.037
Angola	Caluquembe	2000	0.492	0.663	0.868
Angola	Caluquembe	2017	0.139	0.184	0.232
Angola	Caluquembe	2000-2017	-0.103	-0.066	-0.032
Angola	Camacuio	2000	0.543	0.727	0.927
Angola	Camacuio	2017	0.170	0.220	0.269
Angola	Camacuio	2000-2017	-0.102	-0.071	-0.037
Angola	Camacupa	2000	0.421	0.577	0.738
Angola	Camacupa	2017	0.132	0.171	0.216
Angola	Camacupa	2000-2017	-0.096	-0.065	-0.031
Angola	Camanongue	2000	0.480	0.637	0.821
Angola	Camanongue	2017	0.137	0.181	0.230
Angola	Camanongue	2000-2017	-0.100	-0.069	-0.034
Angola	Cambambe	2000	0.486	0.646	0.831
Angola	Cambambe	2017	0.147	0.191	0.241
Angola	Cambambe	2000-2017	-0.099	-0.065	-0.032
Angola	Cambulo	2000	0.489	0.674	0.869
Angola	Cambulo	2017	0.156	0.197	0.244
Angola	Cambulo	2000-2017	-0.098	-0.067	-0.034
Angola	Cambundi-Catembo	2000	0.575	0.764	0.990
Angola	Cambundi-Catembo	2017	0.158	0.208	0.263
Angola	Cambundi-Catembo	2000-2017	-0.103	-0.070	-0.039
Angola	Cameia	2000	0.496	0.639	0.842
Angola	Cameia	2017	0.138	0.177	0.226
Angola	Cameia	2000-2017	-0.102	-0.069	-0.035
Angola	Cangandala	2000	0.617	0.800	1.061
Angola	Cangandala	2017	0.173	0.218	0.268
Angola	Cangandala	2000-2017	-0.099	-0.067	-0.037
Angola	Caombo	2000	0.573	0.772	1.001
Angola	Caombo	2017	0.157	0.200	0.252
Angola	Caombo	2000-2017	-0.101	-0.071	-0.037
Angola	Capenda	2000	0.432	0.580	0.753

Table 2: Diarrhea DALYs rate by unit *(continued)*

Country	Unit	year	mean rate	lower rate	upper rate
Angola	Capenda	2017	0.129	0.166	0.208
Angola	Capenda	2000-2017	-0.102	-0.065	-0.035
Angola	Cassongue	2000	0.625	0.822	1.053
Angola	Cassongue	2017	0.163	0.203	0.251
Angola	Cassongue	2000-2017	-0.107	-0.074	-0.042
Angola	Catabola	2000	0.431	0.583	0.757
Angola	Catabola	2017	0.129	0.170	0.220
Angola	Catabola	2000-2017	-0.103	-0.066	-0.034
Angola	Catchiungo	2000	0.488	0.647	0.811
Angola	Catchiungo	2017	0.157	0.200	0.249
Angola	Catchiungo	2000-2017	-0.100	-0.062	-0.025
Angola	Caungula	2000	0.462	0.629	0.820
Angola	Caungula	2017	0.144	0.186	0.226
Angola	Caungula	2000-2017	-0.097	-0.063	-0.029
Angola	Cazenga	2000	0.407	0.579	0.785
Angola	Cazenga	2017	0.126	0.168	0.217
Angola	Cazenga	2000-2017	-0.102	-0.063	-0.022
Angola	Cazengo	2000	0.491	0.655	0.855
Angola	Cazengo	2017	0.144	0.183	0.225
Angola	Cazengo	2000-2017	-0.107	-0.074	-0.039
Angola	Chibia	2000	0.480	0.647	0.840
Angola	Chibia	2017	0.169	0.218	0.278
Angola	Chibia	2000-2017	-0.090	-0.060	-0.029
Angola	Chicomba	2000	0.477	0.658	0.892
Angola	Chicomba	2017	0.152	0.195	0.246
Angola	Chicomba	2000-2017	-0.099	-0.063	-0.031
Angola	Chinguar	2000	0.448	0.594	0.750
Angola	Chinguar	2017	0.140	0.180	0.231
Angola	Chinguar	2000-2017	-0.101	-0.065	-0.034
Angola	Chipindo	2000	0.493	0.672	0.883
Angola	Chipindo	2017	0.153	0.196	0.246
Angola	Chipindo	2000-2017	-0.099	-0.064	-0.034
Angola	Chitato	2000	0.485	0.656	0.864
Angola	Chitato	2017	0.155	0.204	0.248
Angola	Chitato	2000-2017	-0.090	-0.062	-0.031
Angola	Chitembo	2000	0.447	0.579	0.719
Angola	Chitembo	2017	0.132	0.165	0.208
Angola	Chitembo	2000-2017	-0.096	-0.066	-0.037
Angola	Chongoroi	2000	0.564	0.771	0.980
Angola	Chongoroi	2017	0.164	0.214	0.273
Angola	Chongoroi	2000-2017	-0.095	-0.066	-0.035
Angola	Conda	2000	0.627	0.812	1.040
Angola	Conda	2017	0.176	0.228	0.281
Angola	Conda	2000-2017	-0.101	-0.068	-0.036
Angola	Cuaba Nzogo	2000	0.571	0.776	1.013
Angola	Cuaba Nzogo	2017	0.172	0.221	0.273
Angola	Cuaba Nzogo	2000-2017	-0.100	-0.069	-0.036
Angola	Cuangar	2000	0.555	0.701	0.886
Angola	Cuangar	2017	0.172	0.211	0.259
Angola	Cuangar	2000-2017	-0.096	-0.062	-0.030
Angola	Cuango	2000	0.477	0.638	0.814
Angola	Cuango	2017	0.138	0.173	0.214
Angola	Cuango	2000-2017	-0.100	-0.068	-0.037
Angola	Cuanhama	2000	0.446	0.597	0.772
Angola	Cuanhama	2017	0.117	0.151	0.191
Angola	Cuanhama	2000-2017	-0.108	-0.075	-0.048
Angola	Cubal	2000	0.540	0.752	0.963
Angola	Cubal	2017	0.150	0.195	0.254
Angola	Cubal	2000-2017	-0.095	-0.067	-0.037
Angola	Cuchi	2000	0.513	0.641	0.785
Angola	Cuchi	2017	0.142	0.178	0.225

Table 2: Diarrhea DALYs rate by unit *(continued)*

Country	Unit	year	mean rate	lower rate	upper rate
Angola	Cuchi	2000-2017	-0.101	-0.068	-0.038
Angola	Cuemba	2000	0.425	0.569	0.709
Angola	Cuemba	2017	0.133	0.170	0.217
Angola	Cuemba	2000-2017	-0.098	-0.065	-0.034
Angola	Cuilo	2000	0.469	0.620	0.808
Angola	Cuilo	2017	0.145	0.186	0.228
Angola	Cuilo	2000-2017	-0.097	-0.065	-0.036
Angola	Cuimba	2000	0.490	0.645	0.830
Angola	Cuimba	2017	0.151	0.189	0.235
Angola	Cuimba	2000-2017	-0.098	-0.067	-0.034
Angola	Cuito Cuanavale	2000	0.513	0.648	0.801
Angola	Cuito Cuanavale	2017	0.149	0.183	0.218
Angola	Cuito Cuanavale	2000-2017	-0.096	-0.067	-0.036
Angola	Cunda-dia-Baza	2000	0.538	0.736	0.945
Angola	Cunda-dia-Baza	2017	0.150	0.193	0.246
Angola	Cunda-dia-Baza	2000-2017	-0.102	-0.069	-0.031
Angola	Cunhinga	2000	0.415	0.572	0.737
Angola	Cunhinga	2017	0.128	0.169	0.213
Angola	Cunhinga	2000-2017	-0.102	-0.066	-0.033
Angola	Curoca	2000	0.413	0.559	0.713
Angola	Curoca	2017	0.137	0.173	0.218
Angola	Curoca	2000-2017	-0.092	-0.061	-0.030
Angola	Cuvelai	2000	0.467	0.617	0.788
Angola	Cuvelai	2017	0.135	0.168	0.209
Angola	Cuvelai	2000-2017	-0.103	-0.068	-0.035
Angola	Dala	2000	0.484	0.627	0.811
Angola	Dala	2017	0.134	0.177	0.221
Angola	Dala	2000-2017	-0.105	-0.070	-0.035
Angola	Damba	2000	0.441	0.552	0.710
Angola	Damba	2017	0.142	0.178	0.227
Angola	Damba	2000-2017	-0.083	-0.053	-0.022
Angola	Dande	2000	0.475	0.643	0.831
Angola	Dande	2017	0.153	0.193	0.244
Angola	Dande	2000-2017	-0.106	-0.066	-0.035
Angola	Dembos	2000	0.444	0.611	0.812
Angola	Dembos	2017	0.145	0.184	0.233
Angola	Dembos	2000-2017	-0.095	-0.061	-0.025
Angola	Dirico	2000	0.535	0.671	0.816
Angola	Dirico	2017	0.174	0.215	0.265
Angola	Dirico	2000-2017	-0.087	-0.055	-0.028
Angola	Ebo	2000	0.587	0.783	0.997
Angola	Ebo	2017	0.164	0.214	0.264
Angola	Ebo	2000-2017	-0.109	-0.073	-0.040
Angola	Ekunha	2000	0.535	0.697	0.941
Angola	Ekunha	2017	0.162	0.204	0.258
Angola	Ekunha	2000-2017	-0.094	-0.058	-0.025
Angola	Gambos	2000	0.462	0.642	0.825
Angola	Gambos	2017	0.166	0.212	0.275
Angola	Gambos	2000-2017	-0.088	-0.058	-0.025
Angola	Ganda	2000	0.523	0.705	0.898
Angola	Ganda	2017	0.155	0.199	0.251
Angola	Ganda	2000-2017	-0.086	-0.060	-0.029
Angola	Golungo Alto	2000	0.478	0.637	0.827
Angola	Golungo Alto	2017	0.146	0.186	0.231
Angola	Golungo Alto	2000-2017	-0.110	-0.074	-0.041
Angola	Huambo	2000	0.518	0.676	0.881
Angola	Huambo	2017	0.151	0.191	0.241
Angola	Huambo	2000-2017	-0.099	-0.064	-0.030
Angola	Humpata	2000	0.526	0.700	0.917
Angola	Humpata	2017	0.155	0.203	0.258
Angola	Humpata	2000-2017	-0.107	-0.073	-0.041

Table 2: Diarrhea DALYs rate by unit *(continued)*

Country	Unit	year	mean rate	lower rate	upper rate
Angola	Icolo e Bengo	2000	0.469	0.636	0.849
Angola	Icolo e Bengo	2017	0.148	0.192	0.243
Angola	Icolo e Bengo	2000-2017	-0.104	-0.063	-0.025
Angola	Ingombota	2000	0.379	0.545	0.746
Angola	Ingombota	2017	0.121	0.161	0.208
Angola	Ingombota	2000-2017	-0.097	-0.059	-0.018
Angola	Jamba	2000	0.516	0.665	0.866
Angola	Jamba	2017	0.151	0.194	0.242
Angola	Jamba	2000-2017	-0.101	-0.064	-0.032
Angola	Kilamba Kiaxi	2000	0.407	0.583	0.795
Angola	Kilamba Kiaxi	2017	0.123	0.164	0.213
Angola	Kilamba Kiaxi	2000-2017	-0.106	-0.066	-0.024
Angola	Kuito	2000	0.436	0.592	0.748
Angola	Kuito	2017	0.134	0.177	0.226
Angola	Kuito	2000-2017	-0.100	-0.066	-0.036
Angola	Kuvango	2000	0.474	0.623	0.796
Angola	Kuvango	2017	0.142	0.180	0.223
Angola	Kuvango	2000-2017	-0.101	-0.065	-0.037
Angola	Landana	2000	0.473	0.616	0.766
Angola	Landana	2017	0.146	0.185	0.230
Angola	Landana	2000-2017	-0.085	-0.053	-0.024
Angola	Léua	2000	0.491	0.649	0.837
Angola	Léua	2017	0.137	0.182	0.228
Angola	Léua	2000-2017	-0.106	-0.069	-0.034
Angola	Libolo	2000	0.557	0.707	0.896
Angola	Libolo	2017	0.161	0.201	0.248
Angola	Libolo	2000-2017	-0.099	-0.066	-0.033
Angola	Lobito	2000	0.589	0.814	1.089
Angola	Lobito	2017	0.186	0.232	0.294
Angola	Lobito	2000-2017	-0.087	-0.056	-0.025
Angola	Londuimbale	2000	0.585	0.778	1.028
Angola	Londuimbale	2017	0.175	0.224	0.280
Angola	Londuimbale	2000-2017	-0.100	-0.063	-0.030
Angola	Longonjo	2000	0.525	0.702	0.939
Angola	Longonjo	2017	0.156	0.198	0.253
Angola	Longonjo	2000-2017	-0.102	-0.069	-0.038
Angola	Luau	2000	0.472	0.622	0.816
Angola	Luau	2017	0.137	0.175	0.224
Angola	Luau	2000-2017	-0.099	-0.068	-0.033
Angola	Lubalo	2000	0.433	0.579	0.719
Angola	Lubalo	2017	0.132	0.167	0.207
Angola	Lubalo	2000-2017	-0.100	-0.066	-0.031
Angola	Lubango	2000	0.502	0.673	0.869
Angola	Lubango	2017	0.155	0.204	0.258
Angola	Lubango	2000-2017	-0.098	-0.069	-0.035
Angola	Lucala	2000	0.504	0.687	0.898
Angola	Lucala	2017	0.159	0.201	0.246
Angola	Lucala	2000-2017	-0.098	-0.063	-0.025
Angola	Lucano	2000	0.476	0.616	0.806
Angola	Lucano	2017	0.138	0.175	0.218
Angola	Lucano	2000-2017	-0.097	-0.067	-0.036
Angola	Lucapa	2000	0.486	0.625	0.812
Angola	Lucapa	2017	0.150	0.189	0.234
Angola	Lucapa	2000-2017	-0.092	-0.063	-0.033
Angola	Luchazes	2000	0.467	0.622	0.780
Angola	Luchazes	2017	0.144	0.182	0.223
Angola	Luchazes	2000-2017	-0.092	-0.065	-0.040
Angola	Lumbala-Nguimbo	2000	0.477	0.616	0.806
Angola	Lumbala-Nguimbo	2017	0.143	0.181	0.225
Angola	Lumbala-Nguimbo	2000-2017	-0.092	-0.064	-0.034
Angola	Luquembo	2000	0.533	0.680	0.860

Table 2: Diarrhea DALYs rate by unit *(continued)*

Country	Unit	year	mean rate	lower rate	upper rate
Angola	Luquembo	2017	0.146	0.189	0.237
Angola	Luquembo	2000-2017	-0.097	-0.067	-0.032
Angola	M'Banza Congo	2000	0.508	0.686	0.908
Angola	M'Banza Congo	2017	0.144	0.182	0.229
Angola	M'Banza Congo	2000-2017	-0.106	-0.074	-0.040
Angola	Maianga	2000	0.395	0.566	0.771
Angola	Maianga	2017	0.123	0.165	0.215
Angola	Maianga	2000-2017	-0.104	-0.063	-0.023
Angola	Malanje	2000	0.606	0.799	1.073
Angola	Malanje	2017	0.166	0.210	0.262
Angola	Malanje	2000-2017	-0.099	-0.071	-0.041
Angola	Maquela do Zombo	2000	0.447	0.571	0.734
Angola	Maquela do Zombo	2017	0.144	0.179	0.216
Angola	Maquela do Zombo	2000-2017	-0.093	-0.062	-0.033
Angola	Marimba	2000	0.570	0.766	0.986
Angola	Marimba	2017	0.152	0.191	0.246
Angola	Marimba	2000-2017	-0.099	-0.067	-0.034
Angola	Massango	2000	0.545	0.694	0.890
Angola	Massango	2017	0.150	0.188	0.236
Angola	Massango	2000-2017	-0.095	-0.069	-0.036
Angola	Matala	2000	0.488	0.674	0.895
Angola	Matala	2017	0.147	0.186	0.231
Angola	Matala	2000-2017	-0.100	-0.068	-0.031
Angola	Mavinga	2000	0.540	0.661	0.817
Angola	Mavinga	2017	0.154	0.190	0.230
Angola	Mavinga	2000-2017	-0.094	-0.064	-0.040
Angola	Menongue	2000	0.516	0.646	0.821
Angola	Menongue	2017	0.147	0.184	0.226
Angola	Menongue	2000-2017	-0.097	-0.068	-0.035
Angola	Milunga	2000	0.446	0.583	0.742
Angola	Milunga	2017	0.136	0.174	0.219
Angola	Milunga	2000-2017	-0.095	-0.063	-0.033
Angola	Moxico	2000	0.465	0.621	0.766
Angola	Moxico	2017	0.141	0.179	0.216
Angola	Moxico	2000-2017	-0.097	-0.068	-0.041
Angola	Mucaba	2000	0.397	0.530	0.708
Angola	Mucaba	2017	0.147	0.188	0.240
Angola	Mucaba	2000-2017	-0.071	-0.037	-0.003
Angola	Mucari	2000	0.591	0.793	1.039
Angola	Mucari	2017	0.169	0.213	0.266
Angola	Mucari	2000-2017	-0.102	-0.072	-0.038
Angola	Muconda	2000	0.511	0.637	0.778
Angola	Muconda	2017	0.155	0.190	0.237
Angola	Muconda	2000-2017	-0.096	-0.069	-0.039
Angola	Mungo	2000	0.586	0.773	1.023
Angola	Mungo	2017	0.158	0.207	0.259
Angola	Mungo	2000-2017	-0.103	-0.070	-0.037
Angola	Mussende	2000	0.561	0.723	0.933
Angola	Mussende	2017	0.145	0.184	0.222
Angola	Mussende	2000-2017	-0.103	-0.070	-0.041
Angola	Muxima	2000	0.519	0.666	0.834
Angola	Muxima	2017	0.157	0.199	0.245
Angola	Muxima	2000-2017	-0.093	-0.063	-0.033
Angola	N'Zeto	2000	0.529	0.699	0.932
Angola	N'Zeto	2017	0.157	0.194	0.241
Angola	N'Zeto	2000-2017	-0.099	-0.069	-0.035
Angola	Namakunde	2000	0.452	0.603	0.799
Angola	Namakunde	2017	0.125	0.159	0.203
Angola	Namakunde	2000-2017	-0.101	-0.071	-0.042
Angola	Nambuangongo	2000	0.512	0.656	0.860
Angola	Nambuangongo	2017	0.142	0.183	0.234

Table 2: Diarrhea DALYs rate by unit *(continued)*

Country	Unit	year	mean rate	lower rate	upper rate
Angola	Nambuangongo	2000-2017	-0.095	-0.063	-0.030
Angola	Namibe	2000	0.548	0.719	0.924
Angola	Namibe	2017	0.175	0.227	0.282
Angola	Namibe	2000-2017	-0.098	-0.068	-0.037
Angola	Nancova	2000	0.512	0.659	0.852
Angola	Nancova	2017	0.146	0.184	0.241
Angola	Nancova	2000-2017	-0.095	-0.065	-0.031
Angola	Negage	2000	0.405	0.549	0.732
Angola	Negage	2017	0.139	0.178	0.219
Angola	Negage	2000-2017	-0.087	-0.055	-0.016
Angola	Ngonguembo	2000	0.473	0.641	0.826
Angola	Ngonguembo	2017	0.156	0.194	0.239
Angola	Ngonguembo	2000-2017	-0.108	-0.071	-0.036
Angola	Nharea	2000	0.439	0.578	0.748
Angola	Nharea	2017	0.135	0.172	0.221
Angola	Nharea	2000-2017	-0.100	-0.064	-0.030
Angola	Noqui	2000	0.451	0.620	0.780
Angola	Noqui	2017	0.134	0.170	0.217
Angola	Noqui	2000-2017	-0.101	-0.071	-0.039
Angola	Ombadja	2000	0.430	0.572	0.744
Angola	Ombadja	2017	0.128	0.164	0.208
Angola	Ombadja	2000-2017	-0.099	-0.064	-0.034
Angola	Pango Aluquém	2000	0.433	0.566	0.746
Angola	Pango Aluquém	2017	0.137	0.174	0.221
Angola	Pango Aluquém	2000-2017	-0.097	-0.061	-0.028
Angola	Porto Amboim	2000	0.606	0.784	1.044
Angola	Porto Amboim	2017	0.148	0.200	0.251
Angola	Porto Amboim	2000-2017	-0.105	-0.073	-0.041
Angola	Puri	2000	0.404	0.538	0.727
Angola	Puri	2017	0.132	0.174	0.221
Angola	Puri	2000-2017	-0.077	-0.044	-0.005
Angola	Quela	2000	0.568	0.766	0.977
Angola	Quela	2017	0.166	0.219	0.276
Angola	Quela	2000-2017	-0.099	-0.069	-0.035
Angola	Quibala	2000	0.573	0.740	0.961
Angola	Quibala	2017	0.147	0.192	0.230
Angola	Quibala	2000-2017	-0.110	-0.074	-0.048
Angola	Quiculungo	2000	0.407	0.595	0.774
Angola	Quiculungo	2017	0.139	0.178	0.222
Angola	Quiculungo	2000-2017	-0.107	-0.071	-0.036
Angola	Quilenda	2000	0.588	0.773	0.972
Angola	Quilenda	2017	0.166	0.219	0.274
Angola	Quilenda	2000-2017	-0.108	-0.071	-0.038
Angola	Quilengues	2000	0.506	0.675	0.859
Angola	Quilengues	2017	0.150	0.198	0.251
Angola	Quilengues	2000-2017	-0.099	-0.066	-0.033
Angola	Quimbele	2000	0.439	0.563	0.738
Angola	Quimbele	2017	0.131	0.172	0.223
Angola	Quimbele	2000-2017	-0.099	-0.063	-0.031
Angola	Quirima	2000	0.475	0.628	0.784
Angola	Quirima	2017	0.143	0.186	0.232
Angola	Quirima	2000-2017	-0.094	-0.065	-0.036
Angola	Quitexe	2000	0.430	0.581	0.749
Angola	Quitexe	2017	0.139	0.181	0.229
Angola	Quitexe	2000-2017	-0.090	-0.057	-0.021
Angola	Rangel	2000	0.395	0.563	0.763
Angola	Rangel	2017	0.125	0.166	0.215
Angola	Rangel	2000-2017	-0.099	-0.059	-0.018
Angola	Rivungo	2000	0.521	0.642	0.760
Angola	Rivungo	2017	0.157	0.190	0.232
Angola	Rivungo	2000-2017	-0.089	-0.061	-0.036

Table 2: Diarrhea DALYs rate by unit *(continued)*

Country	Unit	year	mean rate	lower rate	upper rate
Angola	Samba	2000	0.422	0.592	0.820
Angola	Samba	2017	0.116	0.158	0.204
Angola	Samba	2000-2017	-0.109	-0.071	-0.030
Angola	Samba Cajú	2000	0.437	0.622	0.805
Angola	Samba Cajú	2017	0.147	0.186	0.230
Angola	Samba Cajú	2000-2017	-0.097	-0.060	-0.024
Angola	Sambizanga	2000	0.403	0.567	0.771
Angola	Sambizanga	2017	0.125	0.166	0.215
Angola	Sambizanga	2000-2017	-0.099	-0.060	-0.019
Angola	Sanza Pombo	2000	0.427	0.553	0.719
Angola	Sanza Pombo	2017	0.127	0.168	0.211
Angola	Sanza Pombo	2000-2017	-0.083	-0.052	-0.020
Angola	Saurimo	2000	0.474	0.633	0.787
Angola	Saurimo	2017	0.153	0.191	0.236
Angola	Saurimo	2000-2017	-0.099	-0.069	-0.035
Angola	Seles	2000	0.704	0.892	1.141
Angola	Seles	2017	0.194	0.241	0.297
Angola	Seles	2000-2017	-0.105	-0.070	-0.037
Angola	Songo	2000	0.419	0.547	0.724
Angola	Songo	2017	0.142	0.186	0.233
Angola	Songo	2000-2017	-0.074	-0.043	-0.010
Angola	Soyo	2000	0.486	0.642	0.809
Angola	Soyo	2017	0.122	0.155	0.200
Angola	Soyo	2000-2017	-0.113	-0.079	-0.048
Angola	Sumbe	2000	0.723	0.925	1.235
Angola	Sumbe	2017	0.185	0.237	0.287
Angola	Sumbe	2000-2017	-0.101	-0.069	-0.033
Angola	Tchicala-Tcholoanga	2000	0.497	0.645	0.816
Angola	Tchicala-Tcholoanga	2017	0.164	0.207	0.262
Angola	Tchicala-Tcholoanga	2000-2017	-0.093	-0.056	-0.024
Angola	Tchindjenje	2000	0.559	0.745	0.959
Angola	Tchindjenje	2017	0.173	0.221	0.276
Angola	Tchindjenje	2000-2017	-0.081	-0.050	-0.019
Angola	Tchipungo	2000	0.475	0.637	0.838
Angola	Tchipungo	2017	0.152	0.195	0.244
Angola	Tchipungo	2000-2017	-0.096	-0.062	-0.029
Angola	Tomboco	2000	0.517	0.709	0.910
Angola	Tomboco	2017	0.147	0.181	0.222
Angola	Tomboco	2000-2017	-0.106	-0.078	-0.050
Angola	Tombwa	2000	0.454	0.595	0.759
Angola	Tombwa	2017	0.146	0.183	0.227
Angola	Tombwa	2000-2017	-0.096	-0.069	-0.041
Angola	Uíge	2000	0.401	0.544	0.718
Angola	Uíge	2017	0.128	0.168	0.213
Angola	Uíge	2000-2017	-0.092	-0.059	-0.025
Angola	Ukuma	2000	0.523	0.722	0.943
Angola	Ukuma	2017	0.164	0.208	0.258
Angola	Ukuma	2000-2017	-0.094	-0.062	-0.031
Angola	Viana	2000	0.438	0.606	0.838
Angola	Viana	2017	0.118	0.159	0.207
Angola	Viana	2000-2017	-0.112	-0.073	-0.033
Angola	Virei	2000	0.473	0.613	0.779
Angola	Virei	2017	0.159	0.201	0.246
Angola	Virei	2000-2017	-0.095	-0.066	-0.038
Angola	Waku Kungo	2000	0.595	0.775	1.013
Angola	Waku Kungo	2017	0.157	0.205	0.252
Angola	Waku Kungo	2000-2017	-0.108	-0.072	-0.042
Angola	Xá Muteba	2000	0.471	0.638	0.819
Angola	Xá Muteba	2017	0.142	0.176	0.221
Angola	Xá Muteba	2000-2017	-0.096	-0.068	-0.035
Benin	Abomey	2000	0.141	0.205	0.276

Table 2: Diarrhea DALYs rate by unit *(continued)*

Country	Unit	year	mean rate	lower rate	upper rate
Benin	Abomey	2017	0.087	0.121	0.160
Benin	Abomey	2000-2017	-0.097	-0.011	0.086
Benin	Abomey-Calavi	2000	0.162	0.245	0.339
Benin	Abomey-Calavi	2017	0.104	0.140	0.187
Benin	Abomey-Calavi	2000-2017	-0.106	-0.022	0.070
Benin	Adja-Ouèrè	2000	0.159	0.237	0.328
Benin	Adja-Ouèrè	2017	0.105	0.144	0.189
Benin	Adja-Ouèrè	2000-2017	-0.107	-0.020	0.080
Benin	Adjarra	2000	0.158	0.239	0.341
Benin	Adjarra	2017	0.107	0.147	0.192
Benin	Adjarra	2000-2017	-0.102	-0.013	0.083
Benin	Adjohoun	2000	0.142	0.214	0.301
Benin	Adjohoun	2017	0.101	0.134	0.174
Benin	Adjohoun	2000-2017	-0.103	-0.017	0.077
Benin	Agbangnizoun	2000	0.153	0.222	0.297
Benin	Agbangnizoun	2017	0.096	0.132	0.171
Benin	Agbangnizoun	2000-2017	-0.099	-0.014	0.079
Benin	Aguégués	2000	0.163	0.245	0.343
Benin	Aguégués	2017	0.113	0.152	0.199
Benin	Aguégués	2000-2017	-0.101	-0.016	0.079
Benin	Akpro-Missérété	2000	0.150	0.229	0.322
Benin	Akpro-Missérété	2017	0.106	0.145	0.188
Benin	Akpro-Missérété	2000-2017	-0.102	-0.012	0.086
Benin	Allada	2000	0.158	0.238	0.323
Benin	Allada	2017	0.103	0.138	0.179
Benin	Allada	2000-2017	-0.105	-0.018	0.073
Benin	Aplahoué	2000	0.189	0.280	0.366
Benin	Aplahoué	2017	0.117	0.162	0.211
Benin	Aplahoué	2000-2017	-0.099	-0.013	0.078
Benin	Athiémé	2000	0.152	0.220	0.299
Benin	Athiémé	2017	0.098	0.132	0.171
Benin	Athiémé	2000-2017	-0.096	-0.004	0.088
Benin	Avrankou	2000	0.143	0.217	0.310
Benin	Avrankou	2017	0.102	0.142	0.184
Benin	Avrankou	2000-2017	-0.103	-0.012	0.085
Benin	Banikoara	2000	0.281	0.398	0.556
Benin	Banikoara	2017	0.160	0.221	0.292
Benin	Banikoara	2000-2017	-0.118	-0.026	0.068
Benin	Bantè	2000	0.194	0.304	0.421
Benin	Bantè	2017	0.105	0.152	0.211
Benin	Bantè	2000-2017	-0.087	-0.004	0.092
Benin	Bassila	2000	0.241	0.346	0.467
Benin	Bassila	2017	0.124	0.181	0.251
Benin	Bassila	2000-2017	-0.098	-0.006	0.091
Benin	Bembéréké	2000	0.272	0.393	0.568
Benin	Bembéréké	2017	0.132	0.185	0.243
Benin	Bembéréké	2000-2017	-0.129	-0.030	0.070
Benin	Bohicon	2000	0.149	0.220	0.302
Benin	Bohicon	2017	0.093	0.128	0.168
Benin	Bohicon	2000-2017	-0.095	-0.010	0.086
Benin	Bonou	2000	0.146	0.219	0.304
Benin	Bonou	2017	0.098	0.135	0.171
Benin	Bonou	2000-2017	-0.101	-0.017	0.078
Benin	Bopa	2000	0.173	0.256	0.352
Benin	Bopa	2017	0.114	0.151	0.195
Benin	Bopa	2000-2017	-0.097	-0.007	0.083
Benin	Boukoumbé	2000	0.295	0.404	0.521
Benin	Boukoumbé	2017	0.186	0.263	0.349
Benin	Boukoumbé	2000-2017	-0.097	-0.011	0.086
Benin	Cobly	2000	0.325	0.451	0.589
Benin	Cobly	2017	0.209	0.293	0.394

Table 2: Diarrhea DALYs rate by unit *(continued)*

Country	Unit	year	mean rate	lower rate	upper rate
Benin	Cobly	2000-2017	-0.104	-0.016	0.074
Benin	Comè	2000	0.180	0.257	0.355
Benin	Comè	2017	0.116	0.156	0.204
Benin	Comè	2000-2017	-0.098	-0.006	0.084
Benin	Copargo	2000	0.240	0.339	0.449
Benin	Copargo	2017	0.154	0.216	0.290
Benin	Copargo	2000-2017	-0.088	-0.004	0.091
Benin	Cotonou	2000	0.144	0.219	0.305
Benin	Cotonou	2017	0.098	0.131	0.173
Benin	Cotonou	2000-2017	-0.097	-0.012	0.081
Benin	Covè	2000	0.163	0.243	0.344
Benin	Covè	2017	0.105	0.140	0.184
Benin	Covè	2000-2017	-0.096	-0.010	0.093
Benin	Dangbo	2000	0.145	0.221	0.309
Benin	Dangbo	2017	0.101	0.137	0.180
Benin	Dangbo	2000-2017	-0.102	-0.016	0.079
Benin	Dassa-Zoumè	2000	0.176	0.272	0.381
Benin	Dassa-Zoumè	2017	0.100	0.139	0.188
Benin	Dassa-Zoumè	2000-2017	-0.102	-0.011	0.086
Benin	Djakotomey	2000	0.184	0.272	0.362
Benin	Djakotomey	2017	0.119	0.164	0.212
Benin	Djakotomey	2000-2017	-0.105	-0.014	0.076
Benin	Djidja	2000	0.151	0.225	0.305
Benin	Djidja	2017	0.089	0.124	0.166
Benin	Djidja	2000-2017	-0.105	-0.014	0.078
Benin	Djougou	2000	0.245	0.350	0.471
Benin	Djougou	2017	0.142	0.200	0.269
Benin	Djougou	2000-2017	-0.096	-0.007	0.097
Benin	Dogbo	2000	0.174	0.258	0.350
Benin	Dogbo	2017	0.114	0.155	0.201
Benin	Dogbo	2000-2017	-0.106	-0.014	0.076
Benin	Glazoué	2000	0.191	0.299	0.422
Benin	Glazoué	2017	0.102	0.147	0.203
Benin	Glazoué	2000-2017	-0.100	-0.011	0.086
Benin	Gogounou	2000	0.274	0.398	0.566
Benin	Gogounou	2017	0.145	0.194	0.254
Benin	Gogounou	2000-2017	-0.124	-0.030	0.062
Benin	Grand-Popo	2000	0.176	0.249	0.341
Benin	Grand-Popo	2017	0.114	0.153	0.199
Benin	Grand-Popo	2000-2017	-0.100	-0.006	0.084
Benin	Houéyogbé	2000	0.174	0.256	0.354
Benin	Houéyogbé	2017	0.118	0.157	0.203
Benin	Houéyogbé	2000-2017	-0.095	-0.003	0.087
Benin	Ifangni	2000	0.137	0.210	0.298
Benin	Ifangni	2017	0.100	0.138	0.180
Benin	Ifangni	2000-2017	-0.107	-0.019	0.074
Benin	Kalalé	2000	0.250	0.366	0.531
Benin	Kalalé	2017	0.121	0.167	0.227
Benin	Kalalé	2000-2017	-0.118	-0.034	0.058
Benin	Kandi	2000	0.268	0.392	0.535
Benin	Kandi	2017	0.142	0.197	0.253
Benin	Kandi	2000-2017	-0.119	-0.029	0.075
Benin	Karimama	2000	0.294	0.401	0.554
Benin	Karimama	2017	0.167	0.229	0.316
Benin	Karimama	2000-2017	-0.108	-0.018	0.078
Benin	Kérou	2000	0.327	0.465	0.635
Benin	Kérou	2017	0.189	0.258	0.339
Benin	Kérou	2000-2017	-0.130	-0.030	0.064
Benin	Kétou	2000	0.177	0.265	0.369
Benin	Kétou	2017	0.110	0.153	0.205
Benin	Kétou	2000-2017	-0.106	-0.018	0.084

Table 2: Diarrhea DALYs rate by unit *(continued)*

Country	Unit	year	mean rate	lower rate	upper rate
Benin	Klouékanmè	2000	0.168	0.248	0.329
Benin	Klouékanmè	2017	0.104	0.144	0.185
Benin	Klouékanmè	2000-2017	-0.103	-0.015	0.079
Benin	Kouandé	2000	0.291	0.403	0.555
Benin	Kouandé	2017	0.170	0.238	0.319
Benin	Kouandé	2000-2017	-0.117	-0.021	0.071
Benin	Kpomassè	2000	0.168	0.246	0.341
Benin	Kpomassè	2017	0.109	0.146	0.192
Benin	Kpomassè	2000-2017	-0.105	-0.016	0.078
Benin	Lalo	2000	0.184	0.271	0.371
Benin	Lalo	2017	0.116	0.158	0.203
Benin	Lalo	2000-2017	-0.109	-0.019	0.074
Benin	Malanville	2000	0.283	0.392	0.542
Benin	Malanville	2017	0.150	0.208	0.284
Benin	Malanville	2000-2017	-0.105	-0.023	0.077
Benin	Matéri	2000	0.341	0.463	0.604
Benin	Matéri	2017	0.217	0.295	0.392
Benin	Matéri	2000-2017	-0.111	-0.024	0.066
Benin	N'Dali	2000	0.265	0.393	0.546
Benin	N'Dali	2017	0.131	0.182	0.245
Benin	N'Dali	2000-2017	-0.123	-0.028	0.067
Benin	Natitingou	2000	0.270	0.365	0.485
Benin	Natitingou	2017	0.171	0.240	0.322
Benin	Natitingou	2000-2017	-0.101	-0.012	0.081
Benin	Nikki	2000	0.242	0.354	0.499
Benin	Nikki	2017	0.109	0.157	0.213
Benin	Nikki	2000-2017	-0.123	-0.038	0.053
Benin	Ouaké	2000	0.236	0.334	0.447
Benin	Ouaké	2017	0.136	0.193	0.263
Benin	Ouaké	2000-2017	-0.096	-0.007	0.087
Benin	Ouèssè	2000	0.205	0.319	0.431
Benin	Ouèssè	2017	0.105	0.148	0.206
Benin	Ouèssè	2000-2017	-0.106	-0.021	0.078
Benin	Ouidah	2000	0.154	0.228	0.312
Benin	Ouidah	2017	0.103	0.137	0.180
Benin	Ouidah	2000-2017	-0.107	-0.017	0.078
Benin	Ouinhi	2000	0.163	0.241	0.339
Benin	Ouinhi	2017	0.106	0.147	0.194
Benin	Ouinhi	2000-2017	-0.099	-0.016	0.083
Benin	Parakou	2000	0.230	0.339	0.484
Benin	Parakou	2017	0.106	0.154	0.208
Benin	Parakou	2000-2017	-0.124	-0.028	0.073
Benin	Péhunco	2000	0.283	0.404	0.583
Benin	Péhunco	2017	0.156	0.219	0.300
Benin	Péhunco	2000-2017	-0.120	-0.023	0.073
Benin	Pèrèrè	2000	0.234	0.356	0.501
Benin	Pèrèrè	2017	0.111	0.159	0.220
Benin	Pèrèrè	2000-2017	-0.122	-0.037	0.050
Benin	Pobè	2000	0.159	0.235	0.326
Benin	Pobè	2017	0.100	0.140	0.185
Benin	Pobè	2000-2017	-0.102	-0.017	0.086
Benin	Porto-Novo	2000	0.164	0.247	0.351
Benin	Porto-Novo	2017	0.110	0.150	0.195
Benin	Porto-Novo	2000-2017	-0.101	-0.013	0.083
Benin	Sakété	2000	0.154	0.230	0.326
Benin	Sakété	2017	0.109	0.147	0.192
Benin	Sakété	2000-2017	-0.108	-0.021	0.074
Benin	Savalou	2000	0.186	0.283	0.384
Benin	Savalou	2017	0.103	0.147	0.207
Benin	Savalou	2000-2017	-0.092	-0.004	0.087
Benin	Savè	2000	0.188	0.291	0.414

Table 2: Diarrhea DALYs rate by unit *(continued)*

Country	Unit	year	mean rate	lower rate	upper rate
Benin	Savè	2017	0.103	0.145	0.201
Benin	Savè	2000-2017	-0.115	-0.021	0.077
Benin	Segbana	2000	0.253	0.364	0.506
Benin	Segbana	2017	0.130	0.175	0.230
Benin	Segbana	2000-2017	-0.118	-0.032	0.062
Benin	Sèmè-Kpodji	2000	0.163	0.245	0.347
Benin	Sèmè-Kpodji	2017	0.111	0.149	0.194
Benin	Sèmè-Kpodji	2000-2017	-0.099	-0.013	0.079
Benin	Sinendé	2000	0.274	0.403	0.581
Benin	Sinendé	2017	0.149	0.204	0.270
Benin	Sinendé	2000-2017	-0.121	-0.026	0.071
Benin	Sô-Ava	2000	0.149	0.224	0.313
Benin	Sô-Ava	2017	0.099	0.134	0.178
Benin	Sô-Ava	2000-2017	-0.099	-0.017	0.076
Benin	Tanguiéta	2000	0.343	0.460	0.606
Benin	Tanguiéta	2017	0.210	0.286	0.375
Benin	Tanguiéta	2000-2017	-0.117	-0.024	0.069
Benin	Tchaourou	2000	0.231	0.345	0.478
Benin	Tchaourou	2017	0.113	0.162	0.218
Benin	Tchaourou	2000-2017	-0.118	-0.024	0.075
Benin	Toffo	2000	0.149	0.221	0.305
Benin	Toffo	2017	0.097	0.131	0.172
Benin	Toffo	2000-2017	-0.097	-0.013	0.078
Benin	Tori-Bossito	2000	0.149	0.223	0.308
Benin	Tori-Bossito	2017	0.096	0.128	0.167
Benin	Tori-Bossito	2000-2017	-0.107	-0.020	0.072
Benin	Toucountouna	2000	0.283	0.393	0.528
Benin	Toucountouna	2017	0.180	0.249	0.331
Benin	Toucountouna	2000-2017	-0.117	-0.019	0.075
Benin	Toviklin	2000	0.180	0.269	0.363
Benin	Toviklin	2017	0.118	0.158	0.206
Benin	Toviklin	2000-2017	-0.107	-0.018	0.073
Benin	Za-Kpota	2000	0.153	0.225	0.312
Benin	Za-Kpota	2017	0.096	0.130	0.173
Benin	Za-Kpota	2000-2017	-0.092	-0.007	0.092
Benin	Zagnanado	2000	0.168	0.253	0.358
Benin	Zagnanado	2017	0.108	0.147	0.196
Benin	Zagnanado	2000-2017	-0.098	-0.012	0.093
Benin	Zè	2000	0.141	0.213	0.297
Benin	Zè	2017	0.098	0.130	0.173
Benin	Zè	2000-2017	-0.100	-0.015	0.077
Benin	Zogbodomey	2000	0.162	0.237	0.325
Benin	Zogbodomey	2017	0.104	0.144	0.187
Benin	Zogbodomey	2000-2017	-0.094	-0.006	0.088
Botswana	Barolong	2000	0.133	0.145	0.157
Botswana	Barolong	2017	0.063	0.072	0.082
Botswana	Barolong	2000-2017	-0.027	-0.018	-0.009
Botswana	Bobonong	2000	0.142	0.155	0.171
Botswana	Bobonong	2017	0.070	0.078	0.087
Botswana	Bobonong	2000-2017	-0.026	-0.018	-0.011
Botswana	Chobe	2000	0.145	0.159	0.175
Botswana	Chobe	2017	0.074	0.082	0.091
Botswana	Chobe	2000-2017	-0.024	-0.017	-0.011
Botswana	Francistown	2000	0.119	0.130	0.143
Botswana	Francistown	2017	0.056	0.065	0.074
Botswana	Francistown	2000-2017	-0.033	-0.022	-0.012
Botswana	Gaborone	2000	0.102	0.114	0.129
Botswana	Gaborone	2017	0.048	0.057	0.066
Botswana	Gaborone	2000-2017	-0.029	-0.019	-0.010
Botswana	Gemsbok	2000	0.178	0.196	0.219
Botswana	Gemsbok	2017	0.089	0.096	0.104

Table 2: Diarrhea DALYs rate by unit *(continued)*

Country	Unit	year	mean rate	lower rate	upper rate
Botswana	Gemsbok	2000-2017	-0.026	-0.020	-0.013
Botswana	Ghanzi	2000	0.178	0.192	0.207
Botswana	Ghanzi	2017	0.084	0.095	0.106
Botswana	Ghanzi	2000-2017	-0.025	-0.019	-0.014
Botswana	Hukunsti	2000	0.172	0.187	0.207
Botswana	Hukunsti	2017	0.084	0.093	0.102
Botswana	Hukunsti	2000-2017	-0.026	-0.019	-0.013
Botswana	Jwaneng	2000	0.118	0.131	0.147
Botswana	Jwaneng	2017	0.059	0.066	0.074
Botswana	Jwaneng	2000-2017	-0.029	-0.019	-0.008
Botswana	Kgatleng	2000	0.126	0.138	0.151
Botswana	Kgatleng	2017	0.060	0.068	0.076
Botswana	Kgatleng	2000-2017	-0.028	-0.019	-0.011
Botswana	Kweneng North	2000	0.125	0.137	0.151
Botswana	Kweneng North	2017	0.061	0.068	0.076
Botswana	Kweneng North	2000-2017	-0.026	-0.019	-0.010
Botswana	Kweneng South	2000	0.132	0.144	0.157
Botswana	Kweneng South	2017	0.063	0.071	0.080
Botswana	Kweneng South	2000-2017	-0.027	-0.020	-0.014
Botswana	Lethlakane	2000	0.144	0.159	0.176
Botswana	Lethlakane	2017	0.071	0.080	0.089
Botswana	Lethlakane	2000-2017	-0.029	-0.021	-0.013
Botswana	Lobatse	2000	0.117	0.129	0.141
Botswana	Lobatse	2017	0.055	0.063	0.072
Botswana	Lobatse	2000-2017	-0.032	-0.019	-0.009
Botswana	Machaneng	2000	0.137	0.150	0.165
Botswana	Machaneng	2017	0.066	0.075	0.084
Botswana	Machaneng	2000-2017	-0.031	-0.021	-0.013
Botswana	Mahalapye	2000	0.139	0.152	0.165
Botswana	Mahalapye	2017	0.067	0.075	0.084
Botswana	Mahalapye	2000-2017	-0.027	-0.020	-0.013
Botswana	Masungu	2000	0.121	0.133	0.146
Botswana	Masungu	2017	0.060	0.067	0.074
Botswana	Masungu	2000-2017	-0.030	-0.021	-0.011
Botswana	Ngamiland East	2000	0.159	0.172	0.189
Botswana	Ngamiland East	2017	0.077	0.086	0.095
Botswana	Ngamiland East	2000-2017	-0.025	-0.019	-0.013
Botswana	Ngamiland West	2000	0.191	0.206	0.222
Botswana	Ngamiland West	2017	0.090	0.102	0.115
Botswana	Ngamiland West	2000-2017	-0.025	-0.019	-0.013
Botswana	Ngwaketse Central	2000	0.129	0.142	0.156
Botswana	Ngwaketse Central	2017	0.061	0.070	0.079
Botswana	Ngwaketse Central	2000-2017	-0.028	-0.019	-0.012
Botswana	Ngwaketse North	2000	0.130	0.141	0.155
Botswana	Ngwaketse North	2017	0.061	0.070	0.080
Botswana	Ngwaketse North	2000-2017	-0.027	-0.019	-0.012
Botswana	Ngwaketse South	2000	0.133	0.147	0.160
Botswana	Ngwaketse South	2017	0.064	0.073	0.082
Botswana	Ngwaketse South	2000-2017	-0.027	-0.018	-0.011
Botswana	Palapye	2000	0.135	0.146	0.160
Botswana	Palapye	2017	0.064	0.073	0.082
Botswana	Palapye	2000-2017	-0.030	-0.021	-0.013
Botswana	Selibe Phikwe	2000	0.126	0.140	0.157
Botswana	Selibe Phikwe	2017	0.063	0.069	0.077
Botswana	Selibe Phikwe	2000-2017	-0.029	-0.019	-0.009
Botswana	Serowe	2000	0.139	0.149	0.162
Botswana	Serowe	2017	0.065	0.074	0.084
Botswana	Serowe	2000-2017	-0.027	-0.020	-0.014
Botswana	South East	2000	0.111	0.122	0.135
Botswana	South East	2017	0.054	0.061	0.068
Botswana	South East	2000-2017	-0.030	-0.019	-0.010

Table 2: Diarrhea DALYs rate by unit *(continued)*

Country	Unit	year	mean rate	lower rate	upper rate
Botswana	Sowa	2000	0.138	0.152	0.167
Botswana	Sowa	2017	0.066	0.076	0.085
Botswana	Sowa	2000-2017	-0.031	-0.020	-0.008
Botswana	Tshabong	2000	0.174	0.189	0.206
Botswana	Tshabong	2017	0.084	0.094	0.103
Botswana	Tshabong	2000-2017	-0.025	-0.019	-0.012
Botswana	Tuli	2000	0.143	0.155	0.170
Botswana	Tuli	2017	0.069	0.077	0.085
Botswana	Tuli	2000-2017	-0.028	-0.020	-0.012
Botswana	Tutume	2000	0.143	0.155	0.170
Botswana	Tutume	2017	0.068	0.078	0.087
Botswana	Tutume	2000-2017	-0.027	-0.021	-0.013
Burkina Faso	Balé	2000	0.340	0.550	0.782
Burkina Faso	Balé	2017	0.117	0.182	0.285
Burkina Faso	Balé	2000-2017	-0.173	-0.094	-0.005
Burkina Faso	Bam	2000	0.358	0.559	0.805
Burkina Faso	Bam	2017	0.124	0.187	0.275
Burkina Faso	Bam	2000-2017	-0.223	-0.122	-0.031
Burkina Faso	Banwa	2000	0.357	0.531	0.755
Burkina Faso	Banwa	2017	0.115	0.179	0.277
Burkina Faso	Banwa	2000-2017	-0.182	-0.092	-0.002
Burkina Faso	Bazèga	2000	0.349	0.525	0.738
Burkina Faso	Bazèga	2017	0.124	0.184	0.272
Burkina Faso	Bazèga	2000-2017	-0.193	-0.098	-0.004
Burkina Faso	Bougouriba	2000	0.359	0.544	0.797
Burkina Faso	Bougouriba	2017	0.116	0.179	0.261
Burkina Faso	Bougouriba	2000-2017	-0.164	-0.084	-0.007
Burkina Faso	Boulgou	2000	0.399	0.571	0.793
Burkina Faso	Boulgou	2017	0.125	0.189	0.260
Burkina Faso	Boulgou	2000-2017	-0.196	-0.115	-0.035
Burkina Faso	Boulkiemdé	2000	0.336	0.544	0.770
Burkina Faso	Boulkiemdé	2017	0.120	0.182	0.280
Burkina Faso	Boulkiemdé	2000-2017	-0.188	-0.103	-0.009
Burkina Faso	Comoé	2000	0.336	0.485	0.678
Burkina Faso	Comoé	2017	0.096	0.142	0.211
Burkina Faso	Comoé	2000-2017	-0.163	-0.076	0.008
Burkina Faso	Ganzourgou	2000	0.392	0.558	0.781
Burkina Faso	Ganzourgou	2017	0.124	0.188	0.267
Burkina Faso	Ganzourgou	2000-2017	-0.197	-0.105	-0.009
Burkina Faso	Gnagna	2000	0.394	0.553	0.783
Burkina Faso	Gnagna	2017	0.122	0.189	0.281
Burkina Faso	Gnagna	2000-2017	-0.190	-0.097	-0.002
Burkina Faso	Gourma	2000	0.416	0.573	0.796
Burkina Faso	Gourma	2017	0.124	0.182	0.258
Burkina Faso	Gourma	2000-2017	-0.202	-0.113	-0.022
Burkina Faso	Houet	2000	0.369	0.536	0.776
Burkina Faso	Houet	2017	0.108	0.166	0.245
Burkina Faso	Houet	2000-2017	-0.173	-0.088	-0.002
Burkina Faso	Ioba	2000	0.355	0.552	0.779
Burkina Faso	Ioba	2017	0.114	0.181	0.282
Burkina Faso	Ioba	2000-2017	-0.170	-0.084	-0.002
Burkina Faso	Kadiogo	2000	0.280	0.440	0.629
Burkina Faso	Kadiogo	2017	0.115	0.170	0.253
Burkina Faso	Kadiogo	2000-2017	-0.194	-0.096	0.007
Burkina Faso	Kénédougou	2000	0.368	0.526	0.743
Burkina Faso	Kénédougou	2017	0.100	0.154	0.229
Burkina Faso	Kénédougou	2000-2017	-0.164	-0.087	0.006
Burkina Faso	Komandjoari	2000	0.380	0.531	0.731
Burkina Faso	Komandjoari	2017	0.117	0.186	0.267
Burkina Faso	Komandjoari	2000-2017	-0.192	-0.098	-0.009
Burkina Faso	Kompienga	2000	0.485	0.646	0.855

Table 2: Diarrhea DALYs rate by unit *(continued)*

Country	Unit	year	mean rate	lower rate	upper rate
Burkina Faso	Kompienga	2017	0.143	0.191	0.261
Burkina Faso	Kompienga	2000-2017	-0.218	-0.134	-0.056
Burkina Faso	Kossi	2000	0.337	0.526	0.744
Burkina Faso	Kossi	2017	0.117	0.177	0.282
Burkina Faso	Kossi	2000-2017	-0.183	-0.093	0.007
Burkina Faso	Koulpélogo	2000	0.431	0.591	0.813
Burkina Faso	Koulpélogo	2017	0.132	0.192	0.271
Burkina Faso	Koulpélogo	2000-2017	-0.212	-0.128	-0.057
Burkina Faso	Kouritenga	2000	0.377	0.549	0.780
Burkina Faso	Kouritenga	2017	0.118	0.183	0.260
Burkina Faso	Kouritenga	2000-2017	-0.194	-0.105	-0.010
Burkina Faso	Kourwéogo	2000	0.328	0.539	0.748
Burkina Faso	Kourwéogo	2017	0.125	0.185	0.279
Burkina Faso	Kourwéogo	2000-2017	-0.204	-0.105	-0.014
Burkina Faso	Léraba	2000	0.341	0.481	0.661
Burkina Faso	Léraba	2017	0.085	0.130	0.187
Burkina Faso	Léraba	2000-2017	-0.163	-0.081	0.004
Burkina Faso	Loroum	2000	0.391	0.585	0.834
Burkina Faso	Loroum	2017	0.116	0.185	0.288
Burkina Faso	Loroum	2000-2017	-0.213	-0.109	-0.019
Burkina Faso	Mouhoun	2000	0.336	0.538	0.763
Burkina Faso	Mouhoun	2017	0.118	0.182	0.283
Burkina Faso	Mouhoun	2000-2017	-0.179	-0.098	-0.002
Burkina Faso	Nahouri	2000	0.366	0.532	0.734
Burkina Faso	Nahouri	2017	0.115	0.175	0.252
Burkina Faso	Nahouri	2000-2017	-0.184	-0.094	-0.012
Burkina Faso	Namentenga	2000	0.368	0.531	0.767
Burkina Faso	Namentenga	2017	0.130	0.193	0.285
Burkina Faso	Namentenga	2000-2017	-0.197	-0.095	0.006
Burkina Faso	Nayala	2000	0.332	0.536	0.759
Burkina Faso	Nayala	2017	0.116	0.181	0.285
Burkina Faso	Nayala	2000-2017	-0.190	-0.102	0.005
Burkina Faso	Noumbiel	2000	0.366	0.522	0.748
Burkina Faso	Noumbiel	2017	0.104	0.163	0.243
Burkina Faso	Noumbiel	2000-2017	-0.153	-0.069	0.013
Burkina Faso	Oubritenga	2000	0.349	0.550	0.782
Burkina Faso	Oubritenga	2017	0.128	0.188	0.270
Burkina Faso	Oubritenga	2000-2017	-0.212	-0.106	-0.012
Burkina Faso	Oudalan	2000	0.390	0.572	0.787
Burkina Faso	Oudalan	2017	0.136	0.204	0.300
Burkina Faso	Oudalan	2000-2017	-0.179	-0.087	0.008
Burkina Faso	Passoré	2000	0.352	0.552	0.766
Burkina Faso	Passoré	2017	0.117	0.180	0.281
Burkina Faso	Passoré	2000-2017	-0.209	-0.112	-0.023
Burkina Faso	Poni	2000	0.372	0.546	0.785
Burkina Faso	Poni	2017	0.111	0.168	0.251
Burkina Faso	Poni	2000-2017	-0.160	-0.077	0.000
Burkina Faso	Sanguié	2000	0.341	0.561	0.803
Burkina Faso	Sanguié	2017	0.118	0.181	0.277
Burkina Faso	Sanguié	2000-2017	-0.181	-0.101	-0.011
Burkina Faso	Sanmatenga	2000	0.348	0.530	0.774
Burkina Faso	Sanmatenga	2017	0.127	0.191	0.281
Burkina Faso	Sanmatenga	2000-2017	-0.215	-0.102	-0.010
Burkina Faso	Séno	2000	0.403	0.564	0.791
Burkina Faso	Séno	2017	0.132	0.195	0.285
Burkina Faso	Séno	2000-2017	-0.181	-0.092	-0.003
Burkina Faso	Sissili	2000	0.381	0.579	0.847
Burkina Faso	Sissili	2017	0.118	0.182	0.284
Burkina Faso	Sissili	2000-2017	-0.172	-0.090	-0.016
Burkina Faso	Soum	2000	0.409	0.583	0.825
Burkina Faso	Soum	2017	0.132	0.194	0.295

Table 2: Diarrhea DALYs rate by unit *(continued)*

Country	Unit	year	mean rate	lower rate	upper rate
Burkina Faso	Soum	2000-2017	-0.206	-0.103	-0.019
Burkina Faso	Sourou	2000	0.325	0.518	0.721
Burkina Faso	Sourou	2017	0.111	0.178	0.287
Burkina Faso	Sourou	2000-2017	-0.190	-0.101	-0.007
Burkina Faso	Tapoa	2000	0.435	0.582	0.798
Burkina Faso	Tapoa	2017	0.127	0.177	0.243
Burkina Faso	Tapoa	2000-2017	-0.203	-0.103	-0.018
Burkina Faso	Tuy	2000	0.348	0.535	0.781
Burkina Faso	Tuy	2017	0.117	0.180	0.267
Burkina Faso	Tuy	2000-2017	-0.159	-0.086	-0.006
Burkina Faso	Yagha	2000	0.361	0.522	0.721
Burkina Faso	Yagha	2017	0.125	0.192	0.277
Burkina Faso	Yagha	2000-2017	-0.184	-0.089	0.001
Burkina Faso	Yatenga	2000	0.385	0.571	0.794
Burkina Faso	Yatenga	2017	0.112	0.178	0.280
Burkina Faso	Yatenga	2000-2017	-0.218	-0.115	-0.028
Burkina Faso	Ziro	2000	0.378	0.585	0.813
Burkina Faso	Ziro	2017	0.123	0.188	0.289
Burkina Faso	Ziro	2000-2017	-0.185	-0.099	-0.016
Burkina Faso	Zondoma	2000	0.366	0.565	0.793
Burkina Faso	Zondoma	2017	0.110	0.177	0.281
Burkina Faso	Zondoma	2000-2017	-0.208	-0.116	-0.030
Burkina Faso	Zoundwéogo	2000	0.372	0.543	0.780
Burkina Faso	Zoundwéogo	2017	0.120	0.185	0.264
Burkina Faso	Zoundwéogo	2000-2017	-0.196	-0.105	-0.022
Burundi	Bisoro	2000	0.226	0.302	0.393
Burundi	Bisoro	2017	0.090	0.123	0.162
Burundi	Bisoro	2000-2017	-0.086	-0.055	-0.028
Burundi	Bubanza	2000	0.319	0.409	0.518
Burundi	Bubanza	2017	0.126	0.166	0.214
Burundi	Bubanza	2000-2017	-0.098	-0.063	-0.032
Burundi	Bugabira	2000	0.342	0.442	0.555
Burundi	Bugabira	2017	0.127	0.166	0.217
Burundi	Bugabira	2000-2017	-0.107	-0.076	-0.047
Burundi	Buganda	2000	0.391	0.500	0.630
Burundi	Buganda	2017	0.132	0.176	0.229
Burundi	Buganda	2000-2017	-0.110	-0.077	-0.048
Burundi	Bugarama	2000	0.263	0.345	0.449
Burundi	Bugarama	2017	0.097	0.130	0.175
Burundi	Bugarama	2000-2017	-0.094	-0.065	-0.039
Burundi	Bugendana	2000	0.268	0.352	0.450
Burundi	Bugendana	2017	0.111	0.148	0.191
Burundi	Bugendana	2000-2017	-0.084	-0.054	-0.027
Burundi	Bugenyuzi	2000	0.311	0.410	0.523
Burundi	Bugenyuzi	2017	0.122	0.163	0.209
Burundi	Bugenyuzi	2000-2017	-0.091	-0.060	-0.031
Burundi	Buhiga	2000	0.301	0.396	0.508
Burundi	Buhiga	2017	0.116	0.158	0.202
Burundi	Buhiga	2000-2017	-0.088	-0.058	-0.030
Burundi	Buhinyuza	2000	0.332	0.431	0.548
Burundi	Buhinyuza	2017	0.117	0.157	0.202
Burundi	Buhinyuza	2000-2017	-0.095	-0.068	-0.040
Burundi	Bukemba	2000	0.282	0.376	0.504
Burundi	Bukemba	2017	0.109	0.144	0.184
Burundi	Bukemba	2000-2017	-0.078	-0.053	-0.025
Burundi	Bukeye	2000	0.270	0.355	0.450
Burundi	Bukeye	2017	0.114	0.149	0.195
Burundi	Bukeye	2000-2017	-0.088	-0.057	-0.028
Burundi	Bukinanyana	2000	0.315	0.402	0.502
Burundi	Bukinanyana	2017	0.119	0.156	0.200
Burundi	Bukinanyana	2000-2017	-0.105	-0.070	-0.040

Table 2: Diarrhea DALYs rate by unit *(continued)*

Country	Unit	year	mean rate	lower rate	upper rate
Burundi	Bukirasazi	2000	0.244	0.327	0.440
Burundi	Bukirasazi	2017	0.095	0.130	0.172
Burundi	Bukirasazi	2000-2017	-0.083	-0.053	-0.026
Burundi	Burambi	2000	0.245	0.323	0.414
Burundi	Burambi	2017	0.094	0.127	0.168
Burundi	Burambi	2000-2017	-0.088	-0.059	-0.033
Burundi	Buraza	2000	0.241	0.322	0.439
Burundi	Buraza	2017	0.092	0.126	0.166
Burundi	Buraza	2000-2017	-0.085	-0.055	-0.027
Burundi	Bururi	2000	0.238	0.315	0.416
Burundi	Bururi	2017	0.096	0.126	0.166
Burundi	Bururi	2000-2017	-0.086	-0.055	-0.030
Burundi	Busiga	2000	0.311	0.397	0.500
Burundi	Busiga	2017	0.122	0.159	0.205
Burundi	Busiga	2000-2017	-0.093	-0.059	-0.029
Burundi	Busoni	2000	0.364	0.470	0.586
Burundi	Busoni	2017	0.121	0.159	0.203
Burundi	Busoni	2000-2017	-0.116	-0.086	-0.058
Burundi	Butaganzwa1	2000	0.301	0.388	0.487
Burundi	Butaganzwa1	2017	0.126	0.165	0.216
Burundi	Butaganzwa1	2000-2017	-0.086	-0.055	-0.027
Burundi	Butaganzwa2	2000	0.276	0.364	0.471
Burundi	Butaganzwa2	2017	0.102	0.141	0.189
Burundi	Butaganzwa2	2000-2017	-0.085	-0.057	-0.029
Burundi	Buterere	2000	0.270	0.356	0.461
Burundi	Buterere	2017	0.092	0.124	0.161
Burundi	Buterere	2000-2017	-0.099	-0.069	-0.040
Burundi	Butezi	2000	0.277	0.371	0.473
Burundi	Butezi	2017	0.104	0.144	0.193
Burundi	Butezi	2000-2017	-0.086	-0.057	-0.029
Burundi	Butihinda	2000	0.348	0.452	0.565
Burundi	Butihinda	2017	0.120	0.159	0.205
Burundi	Butihinda	2000-2017	-0.105	-0.077	-0.048
Burundi	Buyengero	2000	0.248	0.326	0.422
Burundi	Buyengero	2017	0.099	0.132	0.176
Burundi	Buyengero	2000-2017	-0.085	-0.056	-0.028
Burundi	Buyenze	2000	0.265	0.348	0.450
Burundi	Buyenze	2017	0.090	0.121	0.158
Burundi	Buyenze	2000-2017	-0.099	-0.069	-0.040
Burundi	Bwambarangwe	2000	0.362	0.466	0.586
Burundi	Bwambarangwe	2017	0.119	0.156	0.200
Burundi	Bwambarangwe	2000-2017	-0.114	-0.085	-0.057
Burundi	Bweru	2000	0.257	0.344	0.451
Burundi	Bweru	2017	0.100	0.138	0.186
Burundi	Bweru	2000-2017	-0.083	-0.053	-0.023
Burundi	Bwiza	2000	0.264	0.346	0.447
Burundi	Bwiza	2017	0.090	0.121	0.157
Burundi	Bwiza	2000-2017	-0.097	-0.068	-0.040
Burundi	Cankuzo	2000	0.258	0.342	0.455
Burundi	Cankuzo	2017	0.098	0.134	0.176
Burundi	Cankuzo	2000-2017	-0.084	-0.055	-0.024
Burundi	Cendajuru	2000	0.246	0.321	0.427
Burundi	Cendajuru	2017	0.096	0.130	0.173
Burundi	Cendajuru	2000-2017	-0.079	-0.050	-0.020
Burundi	Cibitoke	2000	0.270	0.356	0.459
Burundi	Cibitoke	2017	0.092	0.124	0.161
Burundi	Cibitoke	2000-2017	-0.098	-0.069	-0.041
Burundi	Gahombo	2000	0.308	0.398	0.500
Burundi	Gahombo	2017	0.129	0.169	0.221
Burundi	Gahombo	2000-2017	-0.092	-0.059	-0.029
Burundi	Gashikanwa	2000	0.344	0.443	0.559

Table 2: Diarrhea DALYs rate by unit *(continued)*

Country	Unit	year	mean rate	lower rate	upper rate
Burundi	Gashikanwa	2017	0.124	0.163	0.208
Burundi	Gashikanwa	2000-2017	-0.101	-0.067	-0.037
Burundi	Gashoho	2000	0.337	0.436	0.543
Burundi	Gashoho	2017	0.125	0.162	0.210
Burundi	Gashoho	2000-2017	-0.100	-0.069	-0.041
Burundi	Gasorwe	2000	0.328	0.428	0.535
Burundi	Gasorwe	2017	0.122	0.161	0.208
Burundi	Gasorwe	2000-2017	-0.100	-0.069	-0.041
Burundi	Gatara	2000	0.302	0.389	0.492
Burundi	Gatara	2017	0.129	0.168	0.220
Burundi	Gatara	2000-2017	-0.088	-0.055	-0.024
Burundi	Gihanga	2000	0.336	0.437	0.557
Burundi	Gihanga	2017	0.124	0.163	0.209
Burundi	Gihanga	2000-2017	-0.099	-0.069	-0.040
Burundi	Giharo	2000	0.285	0.385	0.515
Burundi	Giharo	2017	0.110	0.146	0.191
Burundi	Giharo	2000-2017	-0.080	-0.055	-0.027
Burundi	Giheta	2000	0.263	0.352	0.448
Burundi	Giheta	2017	0.108	0.146	0.191
Burundi	Giheta	2000-2017	-0.083	-0.053	-0.025
Burundi	Gihogazi	2000	0.298	0.395	0.505
Burundi	Gihogazi	2017	0.123	0.162	0.208
Burundi	Gihogazi	2000-2017	-0.089	-0.056	-0.028
Burundi	Gihosha	2000	0.276	0.363	0.466
Burundi	Gihosha	2017	0.094	0.127	0.167
Burundi	Gihosha	2000-2017	-0.095	-0.066	-0.038
Burundi	Gisagara	2000	0.233	0.305	0.400
Burundi	Gisagara	2017	0.094	0.126	0.165
Burundi	Gisagara	2000-2017	-0.075	-0.049	-0.019
Burundi	Gishubi	2000	0.247	0.327	0.433
Burundi	Gishubi	2017	0.097	0.135	0.176
Burundi	Gishubi	2000-2017	-0.081	-0.052	-0.025
Burundi	Gisozi	2000	0.230	0.305	0.399
Burundi	Gisozi	2017	0.092	0.124	0.167
Burundi	Gisozi	2000-2017	-0.089	-0.058	-0.031
Burundi	Gisuru	2000	0.263	0.338	0.439
Burundi	Gisuru	2017	0.099	0.136	0.181
Burundi	Gisuru	2000-2017	-0.079	-0.049	-0.020
Burundi	Gitanga	2000	0.266	0.355	0.479
Burundi	Gitanga	2017	0.099	0.134	0.172
Burundi	Gitanga	2000-2017	-0.085	-0.057	-0.031
Burundi	Gitaramuka	2000	0.310	0.406	0.513
Burundi	Gitaramuka	2017	0.121	0.161	0.205
Burundi	Gitaramuka	2000-2017	-0.093	-0.062	-0.035
Burundi	Gitega	2000	0.254	0.338	0.438
Burundi	Gitega	2017	0.101	0.137	0.179
Burundi	Gitega	2000-2017	-0.082	-0.053	-0.026
Burundi	Giteranyi	2000	0.340	0.434	0.542
Burundi	Giteranyi	2017	0.114	0.151	0.190
Burundi	Giteranyi	2000-2017	-0.114	-0.084	-0.055
Burundi	Gitobe	2000	0.348	0.453	0.571
Burundi	Gitobe	2017	0.124	0.161	0.210
Burundi	Gitobe	2000-2017	-0.106	-0.076	-0.047
Burundi	Isale	2000	0.286	0.374	0.480
Burundi	Isale	2017	0.104	0.140	0.184
Burundi	Isale	2000-2017	-0.094	-0.065	-0.038
Burundi	Itaba	2000	0.259	0.344	0.448
Burundi	Itaba	2017	0.096	0.133	0.177
Burundi	Itaba	2000-2017	-0.085	-0.056	-0.028
Burundi	Kabarore	2000	0.292	0.370	0.465
Burundi	Kabarore	2017	0.121	0.156	0.202

Table 2: Diarrhea DALYs rate by unit *(continued)*

Country	Unit	year	mean rate	lower rate	upper rate
Burundi	Kabarore	2000-2017	-0.093	-0.059	-0.026
Burundi	Kabezi	2000	0.293	0.383	0.494
Burundi	Kabezi	2017	0.104	0.141	0.186
Burundi	Kabezi	2000-2017	-0.095	-0.067	-0.042
Burundi	Kamenge	2000	0.276	0.363	0.467
Burundi	Kamenge	2017	0.094	0.126	0.166
Burundi	Kamenge	2000-2017	-0.095	-0.066	-0.038
Burundi	Kanyosha1	2000	0.284	0.373	0.480
Burundi	Kanyosha1	2017	0.104	0.141	0.186
Burundi	Kanyosha1	2000-2017	-0.093	-0.066	-0.038
Burundi	Kanyosha2	2000	0.258	0.342	0.442
Burundi	Kanyosha2	2017	0.091	0.122	0.160
Burundi	Kanyosha2	2000-2017	-0.095	-0.066	-0.039
Burundi	Kayanza	2000	0.302	0.389	0.495
Burundi	Kayanza	2017	0.126	0.163	0.214
Burundi	Kayanza	2000-2017	-0.090	-0.056	-0.026
Burundi	Kayogoro	2000	0.278	0.364	0.478
Burundi	Kayogoro	2017	0.102	0.137	0.175
Burundi	Kayogoro	2000-2017	-0.084	-0.054	-0.027
Burundi	Kayokwe	2000	0.239	0.318	0.415
Burundi	Kayokwe	2017	0.093	0.129	0.174
Burundi	Kayokwe	2000-2017	-0.086	-0.055	-0.026
Burundi	Kibago	2000	0.245	0.323	0.423
Burundi	Kibago	2017	0.098	0.129	0.166
Burundi	Kibago	2000-2017	-0.085	-0.053	-0.026
Burundi	Kigamba	2000	0.283	0.365	0.467
Burundi	Kigamba	2017	0.102	0.138	0.182
Burundi	Kigamba	2000-2017	-0.087	-0.061	-0.030
Burundi	Kiganda	2000	0.263	0.348	0.439
Burundi	Kiganda	2017	0.109	0.144	0.189
Burundi	Kiganda	2000-2017	-0.085	-0.055	-0.028
Burundi	Kinama	2000	0.270	0.355	0.458
Burundi	Kinama	2017	0.093	0.125	0.164
Burundi	Kinama	2000-2017	-0.093	-0.065	-0.037
Burundi	Kinindo	2000	0.252	0.332	0.428
Burundi	Kinindo	2017	0.088	0.119	0.155
Burundi	Kinindo	2000-2017	-0.091	-0.061	-0.034
Burundi	Kinyinya	2000	0.277	0.368	0.479
Burundi	Kinyinya	2017	0.106	0.145	0.190
Burundi	Kinyinya	2000-2017	-0.076	-0.050	-0.022
Burundi	Kiremba	2000	0.336	0.433	0.542
Burundi	Kiremba	2017	0.128	0.167	0.213
Burundi	Kiremba	2000-2017	-0.098	-0.064	-0.035
Burundi	Kirundo	2000	0.362	0.466	0.591
Burundi	Kirundo	2017	0.132	0.174	0.229
Burundi	Kirundo	2000-2017	-0.107	-0.074	-0.045
Burundi	Lake Tanganyika	2000	0.287	0.387	0.519
Burundi	Lake Tanganyika	2000	0.298	0.390	0.512
Burundi	Lake Tanganyika	2000	0.259	0.341	0.439
Burundi	Lake Tanganyika	2000	0.315	0.412	0.531
Burundi	Lake Tanganyika	2017	0.105	0.143	0.185
Burundi	Lake Tanganyika	2017	0.103	0.138	0.181
Burundi	Lake Tanganyika	2017	0.105	0.137	0.178
Burundi	Lake Tanganyika	2017	0.089	0.120	0.156
Burundi	Lake Tanganyika	2000-2017	-0.093	-0.064	-0.036
Burundi	Lake Tanganyika	2000-2017	-0.096	-0.067	-0.039
Burundi	Lake Tanganyika	2000-2017	-0.103	-0.074	-0.049
Burundi	Lake Tanganyika	2000-2017	-0.096	-0.066	-0.038
Burundi	Mabanda	2000	0.233	0.315	0.421
Burundi	Mabanda	2017	0.095	0.126	0.160
Burundi	Mabanda	2000-2017	-0.087	-0.055	-0.029

Table 2: Diarrhea DALYs rate by unit *(continued)*

Country	Unit	year	mean rate	lower rate	upper rate
Burundi	Mabayi	2000	0.307	0.401	0.509
Burundi	Mabayi	2017	0.111	0.147	0.192
Burundi	Mabayi	2000-2017	-0.110	-0.073	-0.045
Burundi	Makamba	2000	0.248	0.334	0.439
Burundi	Makamba	2017	0.100	0.132	0.171
Burundi	Makamba	2000-2017	-0.081	-0.052	-0.027
Burundi	Makebuko	2000	0.257	0.338	0.442
Burundi	Makebuko	2017	0.099	0.137	0.180
Burundi	Makebuko	2000-2017	-0.081	-0.052	-0.024
Burundi	Marangara	2000	0.363	0.465	0.579
Burundi	Marangara	2017	0.123	0.162	0.208
Burundi	Marangara	2000-2017	-0.107	-0.074	-0.045
Burundi	Matana	2000	0.229	0.304	0.400
Burundi	Matana	2017	0.093	0.125	0.165
Burundi	Matana	2000-2017	-0.084	-0.054	-0.026
Burundi	Matongo	2000	0.297	0.382	0.484
Burundi	Matongo	2017	0.122	0.158	0.204
Burundi	Matongo	2000-2017	-0.089	-0.057	-0.027
Burundi	Mbuye	2000	0.283	0.372	0.468
Burundi	Mbuye	2017	0.117	0.153	0.199
Burundi	Mbuye	2000-2017	-0.084	-0.055	-0.028
Burundi	Mishiha	2000	0.250	0.328	0.427
Burundi	Mishiha	2017	0.096	0.128	0.166
Burundi	Mishiha	2000-2017	-0.083	-0.053	-0.023
Burundi	Mpanda	2000	0.328	0.419	0.533
Burundi	Mpanda	2017	0.127	0.167	0.215
Burundi	Mpanda	2000-2017	-0.091	-0.061	-0.032
Burundi	Mpinga-Kayove	2000	0.268	0.365	0.492
Burundi	Mpinga-Kayove	2017	0.103	0.138	0.178
Burundi	Mpinga-Kayove	2000-2017	-0.084	-0.057	-0.029
Burundi	Mubimbi	2000	0.297	0.387	0.494
Burundi	Mubimbi	2017	0.113	0.150	0.197
Burundi	Mubimbi	2000-2017	-0.093	-0.062	-0.035
Burundi	Mugamba	2000	0.231	0.303	0.399
Burundi	Mugamba	2017	0.093	0.127	0.172
Burundi	Mugamba	2000-2017	-0.085	-0.056	-0.030
Burundi	Mugina	2000	0.324	0.421	0.546
Burundi	Mugina	2017	0.125	0.164	0.213
Burundi	Mugina	2000-2017	-0.102	-0.066	-0.036
Burundi	Mugongomanga	2000	0.260	0.344	0.438
Burundi	Mugongomanga	2017	0.100	0.136	0.182
Burundi	Mugongomanga	2000-2017	-0.089	-0.061	-0.033
Burundi	Muhanga	2000	0.313	0.407	0.519
Burundi	Muhanga	2017	0.130	0.170	0.220
Burundi	Muhanga	2000-2017	-0.091	-0.059	-0.030
Burundi	Muhuta	2000	0.266	0.350	0.457
Burundi	Muhuta	2017	0.098	0.132	0.178
Burundi	Muhuta	2000-2017	-0.095	-0.066	-0.039
Burundi	Mukike	2000	0.250	0.329	0.426
Burundi	Mukike	2017	0.097	0.132	0.179
Burundi	Mukike	2000-2017	-0.089	-0.060	-0.034
Burundi	Muramvya	2000	0.262	0.349	0.439
Burundi	Muramvya	2017	0.109	0.143	0.188
Burundi	Muramvya	2000-2017	-0.089	-0.059	-0.032
Burundi	Muruta	2000	0.301	0.382	0.484
Burundi	Muruta	2017	0.121	0.157	0.202
Burundi	Muruta	2000-2017	-0.090	-0.056	-0.025
Burundi	Murwi	2000	0.367	0.466	0.586
Burundi	Murwi	2017	0.129	0.169	0.224
Burundi	Murwi	2000-2017	-0.109	-0.075	-0.046
Burundi	Musaga	2000	0.264	0.348	0.449

Table 2: Diarrhea DALYs rate by unit *(continued)*

Country	Unit	year	mean rate	lower rate	upper rate
Burundi	Musaga	2017	0.094	0.126	0.165
Burundi	Musaga	2000-2017	-0.093	-0.064	-0.037
Burundi	Musigati	2000	0.295	0.375	0.475
Burundi	Musigati	2017	0.117	0.154	0.199
Burundi	Musigati	2000-2017	-0.093	-0.060	-0.029
Burundi	Musongati	2000	0.272	0.369	0.497
Burundi	Musongati	2017	0.099	0.136	0.177
Burundi	Musongati	2000-2017	-0.089	-0.061	-0.033
Burundi	Mutaho	2000	0.282	0.370	0.470
Burundi	Mutaho	2017	0.118	0.154	0.198
Burundi	Mutaho	2000-2017	-0.087	-0.056	-0.028
Burundi	Mutambu	2000	0.272	0.354	0.460
Burundi	Mutambu	2017	0.101	0.137	0.184
Burundi	Mutambu	2000-2017	-0.094	-0.065	-0.039
Burundi	Mutimbuzi	2000	0.316	0.411	0.528
Burundi	Mutimbuzi	2017	0.106	0.143	0.186
Burundi	Mutimbuzi	2000-2017	-0.100	-0.070	-0.042
Burundi	Mutumba	2000	0.293	0.386	0.498
Burundi	Mutumba	2017	0.109	0.150	0.196
Burundi	Mutumba	2000-2017	-0.088	-0.059	-0.031
Burundi	Muyinga	2000	0.339	0.436	0.541
Burundi	Muyinga	2017	0.116	0.155	0.200
Burundi	Muyinga	2000-2017	-0.103	-0.075	-0.046
Burundi	Mwakiro	2000	0.294	0.384	0.492
Burundi	Mwakiro	2017	0.109	0.151	0.198
Burundi	Mwakiro	2000-2017	-0.087	-0.058	-0.029
Burundi	Mwumba	2000	0.332	0.427	0.535
Burundi	Mwumba	2017	0.120	0.158	0.201
Burundi	Mwumba	2000-2017	-0.103	-0.069	-0.037
Burundi	Ndava	2000	0.254	0.336	0.423
Burundi	Ndava	2017	0.102	0.135	0.177
Burundi	Ndava	2000-2017	-0.087	-0.057	-0.030
Burundi	Ngagara	2000	0.268	0.353	0.457
Burundi	Ngagara	2017	0.091	0.122	0.159
Burundi	Ngagara	2000-2017	-0.100	-0.070	-0.041
Burundi	Ngozi	2000	0.313	0.406	0.515
Burundi	Ngozi	2017	0.124	0.163	0.211
Burundi	Ngozi	2000-2017	-0.095	-0.063	-0.033
Burundi	Ntega	2000	0.358	0.465	0.589
Burundi	Ntega	2017	0.130	0.170	0.222
Burundi	Ntega	2000-2017	-0.105	-0.072	-0.042
Burundi	Nyabihanga	2000	0.255	0.335	0.426
Burundi	Nyabihanga	2017	0.098	0.135	0.177
Burundi	Nyabihanga	2000-2017	-0.087	-0.056	-0.030
Burundi	Nyabikere	2000	0.293	0.389	0.501
Burundi	Nyabikere	2017	0.110	0.153	0.202
Burundi	Nyabikere	2000-2017	-0.088	-0.059	-0.030
Burundi	Nyabiraba	2000	0.274	0.357	0.461
Burundi	Nyabiraba	2017	0.102	0.138	0.185
Burundi	Nyabiraba	2000-2017	-0.091	-0.063	-0.036
Burundi	Nyabitsinda	2000	0.283	0.373	0.494
Burundi	Nyabitsinda	2017	0.105	0.146	0.195
Burundi	Nyabitsinda	2000-2017	-0.079	-0.052	-0.024
Burundi	Nyakabiga	2000	0.278	0.366	0.470
Burundi	Nyakabiga	2017	0.097	0.131	0.172
Burundi	Nyakabiga	2000-2017	-0.094	-0.066	-0.037
Burundi	Nyamurenza	2000	0.362	0.467	0.587
Burundi	Nyamurenza	2017	0.123	0.163	0.210
Burundi	Nyamurenza	2000-2017	-0.108	-0.074	-0.044
Burundi	Nyanrusange	2000	0.239	0.320	0.419
Burundi	Nyanrusange	2017	0.096	0.133	0.173

Table 2: Diarrhea DALYs rate by unit *(continued)*

Country	Unit	year	mean rate	lower rate	upper rate
Burundi	Nyanrusange	2000-2017	-0.082	-0.052	-0.025
Burundi	Nyanza-Lac	2000	0.271	0.368	0.491
Burundi	Nyanza-Lac	2017	0.107	0.143	0.184
Burundi	Nyanza-Lac	2000-2017	-0.089	-0.059	-0.033
Burundi	Rango	2000	0.293	0.383	0.484
Burundi	Rango	2017	0.122	0.159	0.207
Burundi	Rango	2000-2017	-0.087	-0.056	-0.029
Burundi	Roherero	2000	0.264	0.346	0.445
Burundi	Roherero	2017	0.093	0.126	0.164
Burundi	Roherero	2000-2017	-0.092	-0.062	-0.035
Burundi	Rugazi	2000	0.313	0.404	0.513
Burundi	Rugazi	2017	0.117	0.154	0.200
Burundi	Rugazi	2000-2017	-0.091	-0.060	-0.031
Burundi	Rugombo	2000	0.382	0.494	0.644
Burundi	Rugombo	2017	0.134	0.176	0.232
Burundi	Rugombo	2000-2017	-0.113	-0.079	-0.048
Burundi	Ruhororo	2000	0.323	0.421	0.534
Burundi	Ruhororo	2017	0.126	0.167	0.215
Burundi	Ruhororo	2000-2017	-0.095	-0.062	-0.033
Burundi	Rumonge	2000	0.280	0.367	0.483
Burundi	Rumonge	2017	0.104	0.136	0.178
Burundi	Rumonge	2000-2017	-0.091	-0.062	-0.035
Burundi	Rusaka	2000	0.242	0.320	0.419
Burundi	Rusaka	2017	0.093	0.128	0.170
Burundi	Rusaka	2000-2017	-0.090	-0.059	-0.031
Burundi	Rutana	2000	0.268	0.359	0.488
Burundi	Rutana	2017	0.101	0.136	0.174
Burundi	Rutana	2000-2017	-0.085	-0.056	-0.029
Burundi	Rutegama	2000	0.275	0.361	0.455
Burundi	Rutegama	2017	0.112	0.147	0.192
Burundi	Rutegama	2000-2017	-0.086	-0.056	-0.030
Burundi	Rutovu	2000	0.244	0.322	0.436
Burundi	Rutovu	2017	0.091	0.125	0.165
Burundi	Rutovu	2000-2017	-0.087	-0.055	-0.028
Burundi	Ruyigi	2000	0.261	0.347	0.451
Burundi	Ruyigi	2017	0.100	0.140	0.191
Burundi	Ruyigi	2000-2017	-0.079	-0.051	-0.023
Burundi	Ryansoro	2000	0.233	0.306	0.407
Burundi	Ryansoro	2017	0.091	0.125	0.162
Burundi	Ryansoro	2000-2017	-0.083	-0.053	-0.026
Burundi	Shombo	2000	0.299	0.395	0.511
Burundi	Shombo	2017	0.116	0.157	0.207
Burundi	Shombo	2000-2017	-0.086	-0.056	-0.029
Burundi	Songa	2000	0.233	0.306	0.403
Burundi	Songa	2017	0.094	0.126	0.165
Burundi	Songa	2000-2017	-0.084	-0.054	-0.026
Burundi	Tangara	2000	0.313	0.409	0.520
Burundi	Tangara	2017	0.125	0.164	0.210
Burundi	Tangara	2000-2017	-0.092	-0.060	-0.030
Burundi	Vugizo	2000	0.242	0.323	0.435
Burundi	Vugizo	2017	0.096	0.126	0.165
Burundi	Vugizo	2000-2017	-0.085	-0.057	-0.032
Burundi	Vumbi	2000	0.347	0.445	0.556
Burundi	Vumbi	2017	0.129	0.166	0.216
Burundi	Vumbi	2000-2017	-0.102	-0.070	-0.041
Burundi	Vyanda	2000	0.238	0.316	0.416
Burundi	Vyanda	2017	0.098	0.127	0.168
Burundi	Vyanda	2000-2017	-0.087	-0.056	-0.030
Cameroon	Bamboutos	2000	0.202	0.280	0.378
Cameroon	Bamboutos	2017	0.073	0.115	0.180
Cameroon	Bamboutos	2000-2017	-0.120	-0.028	0.069

Table 2: Diarrhea DALYs rate by unit *(continued)*

Country	Unit	year	mean rate	lower rate	upper rate
Cameroon	Bénoué	2000	0.560	0.739	0.972
Cameroon	Bénoué	2017	0.131	0.232	0.385
Cameroon	Bénoué	2000-2017	-0.194	-0.095	0.011
Cameroon	Boumba et Ngoko	2000	0.332	0.448	0.587
Cameroon	Boumba et Ngoko	2017	0.123	0.202	0.326
Cameroon	Boumba et Ngoko	2000-2017	-0.160	-0.059	0.041
Cameroon	Boyo	2000	0.200	0.283	0.396
Cameroon	Boyo	2017	0.075	0.120	0.192
Cameroon	Boyo	2000-2017	-0.116	-0.024	0.068
Cameroon	Bui	2000	0.204	0.290	0.419
Cameroon	Bui	2017	0.075	0.121	0.203
Cameroon	Bui	2000-2017	-0.131	-0.033	0.063
Cameroon	Diamaré	2000	0.584	0.756	0.970
Cameroon	Diamaré	2017	0.144	0.251	0.410
Cameroon	Diamaré	2000-2017	-0.201	-0.107	-0.014
Cameroon	Dja et Lobo	2000	0.350	0.459	0.580
Cameroon	Dja et Lobo	2017	0.117	0.185	0.285
Cameroon	Dja et Lobo	2000-2017	-0.154	-0.056	0.046
Cameroon	Djerem	2000	0.284	0.406	0.532
Cameroon	Djerem	2017	0.101	0.168	0.270
Cameroon	Djerem	2000-2017	-0.142	-0.050	0.046
Cameroon	Donga Mantung	2000	0.214	0.301	0.431
Cameroon	Donga Mantung	2017	0.078	0.123	0.207
Cameroon	Donga Mantung	2000-2017	-0.128	-0.032	0.066
Cameroon	Fako	2000	0.232	0.302	0.384
Cameroon	Fako	2017	0.072	0.122	0.190
Cameroon	Fako	2000-2017	-0.134	-0.038	0.060
Cameroon	Faro	2000	0.414	0.571	0.783
Cameroon	Faro	2017	0.120	0.200	0.305
Cameroon	Faro	2000-2017	-0.168	-0.080	0.014
Cameroon	Faro et Déo	2000	0.295	0.437	0.616
Cameroon	Faro et Déo	2017	0.103	0.175	0.273
Cameroon	Faro et Déo	2000-2017	-0.150	-0.053	0.036
Cameroon	Haut Nkam	2000	0.217	0.306	0.402
Cameroon	Haut Nkam	2017	0.079	0.128	0.202
Cameroon	Haut Nkam	2000-2017	-0.116	-0.027	0.074
Cameroon	Haut Nyong	2000	0.361	0.470	0.599
Cameroon	Haut Nyong	2017	0.117	0.183	0.297
Cameroon	Haut Nyong	2000-2017	-0.165	-0.067	0.030
Cameroon	Haute Sanaga	2000	0.289	0.420	0.561
Cameroon	Haute Sanaga	2017	0.103	0.158	0.238
Cameroon	Haute Sanaga	2000-2017	-0.153	-0.058	0.036
Cameroon	Hauts Plateaux	2000	0.201	0.286	0.375
Cameroon	Hauts Plateaux	2017	0.072	0.119	0.192
Cameroon	Hauts Plateaux	2000-2017	-0.121	-0.027	0.073
Cameroon	Kadey	2000	0.338	0.472	0.628
Cameroon	Kadey	2017	0.119	0.191	0.318
Cameroon	Kadey	2000-2017	-0.185	-0.069	0.035
Cameroon	Koung Khi	2000	0.206	0.295	0.389
Cameroon	Koung Khi	2017	0.075	0.121	0.195
Cameroon	Koung Khi	2000-2017	-0.123	-0.027	0.075
Cameroon	Koupé Manengouba	2000	0.226	0.309	0.407
Cameroon	Koupé Manengouba	2017	0.076	0.128	0.202
Cameroon	Koupé Manengouba	2000-2017	-0.115	-0.028	0.058
Cameroon	Lebialem	2000	0.208	0.286	0.379
Cameroon	Lebialem	2017	0.080	0.127	0.199
Cameroon	Lebialem	2000-2017	-0.117	-0.024	0.076
Cameroon	Lekié	2000	0.280	0.394	0.513
Cameroon	Lekié	2017	0.092	0.148	0.224
Cameroon	Lekié	2000-2017	-0.154	-0.047	0.051
Cameroon	Logone et Chari	2000	0.558	0.692	0.851

Table 2: Diarrhea DALYs rate by unit *(continued)*

Country	Unit	year	mean rate	lower rate	upper rate
Cameroon	Logone et Chari	2017	0.169	0.263	0.407
Cameroon	Logone et Chari	2000-2017	-0.185	-0.098	-0.007
Cameroon	Lom et Djerem	2000	0.318	0.440	0.577
Cameroon	Lom et Djerem	2017	0.116	0.183	0.283
Cameroon	Lom et Djerem	2000-2017	-0.181	-0.066	0.034
Cameroon	Manyu	2000	0.231	0.314	0.426
Cameroon	Manyu	2017	0.078	0.127	0.204
Cameroon	Manyu	2000-2017	-0.106	-0.022	0.060
Cameroon	Mayo Banyo	2000	0.269	0.390	0.551
Cameroon	Mayo Banyo	2017	0.090	0.152	0.254
Cameroon	Mayo Banyo	2000-2017	-0.131	-0.045	0.049
Cameroon	Mayo Danay	2000	0.589	0.726	0.900
Cameroon	Mayo Danay	2017	0.161	0.268	0.413
Cameroon	Mayo Danay	2000-2017	-0.191	-0.095	-0.002
Cameroon	Mayo Kani	2000	0.614	0.775	0.986
Cameroon	Mayo Kani	2017	0.156	0.261	0.419
Cameroon	Mayo Kani	2000-2017	-0.203	-0.101	0.000
Cameroon	Mayo Louti	2000	0.632	0.805	1.051
Cameroon	Mayo Louti	2017	0.144	0.241	0.413
Cameroon	Mayo Louti	2000-2017	-0.207	-0.102	0.004
Cameroon	Mayo Rey	2000	0.491	0.616	0.768
Cameroon	Mayo Rey	2017	0.126	0.217	0.347
Cameroon	Mayo Rey	2000-2017	-0.177	-0.082	0.013
Cameroon	Mayo Sava	2000	0.590	0.790	1.032
Cameroon	Mayo Sava	2017	0.149	0.253	0.397
Cameroon	Mayo Sava	2000-2017	-0.187	-0.108	-0.006
Cameroon	Mayo Tsanaga	2000	0.583	0.764	1.015
Cameroon	Mayo Tsanaga	2017	0.142	0.244	0.405
Cameroon	Mayo Tsanaga	2000-2017	-0.203	-0.110	-0.013
Cameroon	Mbam et Inoubou	2000	0.232	0.332	0.427
Cameroon	Mbam et Inoubou	2017	0.078	0.127	0.199
Cameroon	Mbam et Inoubou	2000-2017	-0.141	-0.041	0.052
Cameroon	Mbam et Kim	2000	0.278	0.394	0.504
Cameroon	Mbam et Kim	2017	0.094	0.150	0.232
Cameroon	Mbam et Kim	2000-2017	-0.129	-0.047	0.039
Cameroon	Mbéré	2000	0.305	0.426	0.571
Cameroon	Mbéré	2017	0.107	0.183	0.286
Cameroon	Mbéré	2000-2017	-0.178	-0.066	0.035
Cameroon	Mefou et Afamba	2000	0.293	0.407	0.546
Cameroon	Mefou et Afamba	2017	0.086	0.140	0.222
Cameroon	Mefou et Afamba	2000-2017	-0.154	-0.048	0.052
Cameroon	Mefou et Akono	2000	0.289	0.410	0.538
Cameroon	Mefou et Akono	2017	0.087	0.142	0.228
Cameroon	Mefou et Akono	2000-2017	-0.162	-0.051	0.045
Cameroon	Meme	2000	0.239	0.317	0.417
Cameroon	Meme	2017	0.075	0.125	0.192
Cameroon	Meme	2000-2017	-0.128	-0.035	0.054
Cameroon	Menchum	2000	0.216	0.319	0.447
Cameroon	Menchum	2017	0.076	0.125	0.195
Cameroon	Menchum	2000-2017	-0.113	-0.024	0.058
Cameroon	Menoua	2000	0.201	0.279	0.368
Cameroon	Menoua	2017	0.075	0.119	0.188
Cameroon	Menoua	2000-2017	-0.112	-0.025	0.073
Cameroon	Mezam	2000	0.192	0.266	0.368
Cameroon	Mezam	2017	0.073	0.115	0.182
Cameroon	Mezam	2000-2017	-0.116	-0.027	0.061
Cameroon	Mfoundi	2000	0.278	0.393	0.519
Cameroon	Mfoundi	2017	0.082	0.133	0.214
Cameroon	Mfoundi	2000-2017	-0.161	-0.050	0.051
Cameroon	Mifi	2000	0.220	0.306	0.409
Cameroon	Mifi	2017	0.072	0.115	0.183

Table 2: Diarrhea DALYs rate by unit *(continued)*

Country	Unit	year	mean rate	lower rate	upper rate
Cameroon	Mifi	2000-2017	-0.126	-0.029	0.073
Cameroon	Momo	2000	0.213	0.301	0.418
Cameroon	Momo	2017	0.076	0.120	0.193
Cameroon	Momo	2000-2017	-0.121	-0.029	0.061
Cameroon	Moungo	2000	0.235	0.307	0.389
Cameroon	Moungo	2017	0.071	0.117	0.181
Cameroon	Moungo	2000-2017	-0.131	-0.036	0.057
Cameroon	Mvila	2000	0.347	0.459	0.594
Cameroon	Mvila	2017	0.107	0.175	0.271
Cameroon	Mvila	2000-2017	-0.153	-0.050	0.055
Cameroon	Ndé	2000	0.212	0.295	0.378
Cameroon	Ndé	2017	0.079	0.127	0.207
Cameroon	Ndé	2000-2017	-0.124	-0.027	0.073
Cameroon	Ndian	2000	0.231	0.314	0.422
Cameroon	Ndian	2017	0.080	0.129	0.200
Cameroon	Ndian	2000-2017	-0.128	-0.035	0.052
Cameroon	Ngo Ketunjia	2000	0.213	0.293	0.411
Cameroon	Ngo Ketunjia	2017	0.074	0.119	0.191
Cameroon	Ngo Ketunjia	2000-2017	-0.129	-0.033	0.053
Cameroon	Nkam	2000	0.249	0.339	0.425
Cameroon	Nkam	2017	0.078	0.127	0.205
Cameroon	Nkam	2000-2017	-0.125	-0.038	0.064
Cameroon	Noun	2000	0.247	0.344	0.464
Cameroon	Noun	2017	0.080	0.131	0.209
Cameroon	Noun	2000-2017	-0.126	-0.034	0.060
Cameroon	Nyong et Kéllé	2000	0.291	0.401	0.532
Cameroon	Nyong et Kéllé	2017	0.095	0.155	0.249
Cameroon	Nyong et Kéllé	2000-2017	-0.139	-0.038	0.065
Cameroon	Nyong et Mfoumou	2000	0.321	0.451	0.597
Cameroon	Nyong et Mfoumou	2017	0.100	0.163	0.248
Cameroon	Nyong et Mfoumou	2000-2017	-0.173	-0.063	0.039
Cameroon	Nyong et So'o	2000	0.336	0.456	0.596
Cameroon	Nyong et So'o	2017	0.101	0.166	0.264
Cameroon	Nyong et So'o	2000-2017	-0.164	-0.052	0.040
Cameroon	Océan	2000	0.319	0.422	0.564
Cameroon	Océan	2017	0.095	0.157	0.248
Cameroon	Océan	2000-2017	-0.141	-0.049	0.056
Cameroon	Sanaga Maritime	2000	0.256	0.348	0.448
Cameroon	Sanaga Maritime	2017	0.078	0.129	0.208
Cameroon	Sanaga Maritime	2000-2017	-0.130	-0.040	0.053
Cameroon	Vallée du Ntem	2000	0.339	0.440	0.576
Cameroon	Vallée du Ntem	2017	0.101	0.165	0.264
Cameroon	Vallée du Ntem	2000-2017	-0.153	-0.056	0.046
Cameroon	Vina	2000	0.318	0.437	0.580
Cameroon	Vina	2017	0.096	0.171	0.262
Cameroon	Vina	2000-2017	-0.180	-0.077	0.021
Cameroon	Wouri	2000	0.227	0.303	0.393
Cameroon	Wouri	2017	0.067	0.113	0.184
Cameroon	Wouri	2000-2017	-0.135	-0.040	0.056
Central African Republic	Alindao	2000	0.558	0.667	0.795
Central African Republic	Alindao	2017	0.519	0.661	0.799
Central African Republic	Alindao	2000-2017	-0.013	0.020	0.053
Central African Republic	Baboua	2000	0.418	0.505	0.603
Central African Republic	Baboua	2017	0.464	0.570	0.691
Central African Republic	Baboua	2000-2017	-0.010	0.017	0.049
Central African Republic	Bakala	2000	0.503	0.604	0.712
Central African Republic	Bakala	2017	0.497	0.635	0.787
Central African Republic	Bakala	2000-2017	-0.015	0.015	0.041
Central African Republic	Bakouma	2000	0.493	0.605	0.718
Central African Republic	Bakouma	2017	0.483	0.603	0.734
Central African Republic	Bakouma	2000-2017	-0.016	0.011	0.038

Table 2: Diarrhea DALYs rate by unit *(continued)*

Country	Unit	year	mean rate	lower rate	upper rate
Central African Republic	Bambari	2000	0.480	0.583	0.692
Central African Republic	Bambari	2017	0.503	0.623	0.790
Central African Republic	Bambari	2000-2017	-0.013	0.019	0.050
Central African Republic	Bambio	2000	0.420	0.501	0.610
Central African Republic	Bambio	2017	0.433	0.544	0.674
Central African Republic	Bambio	2000-2017	-0.023	0.012	0.043
Central African Republic	Bamingui	2000	0.483	0.575	0.688
Central African Republic	Bamingui	2017	0.496	0.609	0.751
Central African Republic	Bamingui	2000-2017	-0.027	0.004	0.030
Central African Republic	Bangassou	2000	0.472	0.575	0.693
Central African Republic	Bangassou	2017	0.447	0.576	0.739
Central African Republic	Bangassou	2000-2017	-0.032	0.000	0.032
Central African Republic	Bangui	2000	0.384	0.445	0.540
Central African Republic	Bangui	2017	0.423	0.547	0.689
Central African Republic	Bangui	2000-2017	-0.028	0.004	0.037
Central African Republic	Baoro	2000	0.397	0.491	0.577
Central African Republic	Baoro	2017	0.440	0.566	0.696
Central African Republic	Baoro	2000-2017	-0.013	0.019	0.049
Central African Republic	Batangafo	2000	0.452	0.547	0.652
Central African Republic	Batangafo	2017	0.498	0.629	0.779
Central African Republic	Batangafo	2000-2017	-0.009	0.020	0.052
Central African Republic	Berbérati	2000	0.419	0.513	0.619
Central African Republic	Berbérati	2017	0.443	0.556	0.689
Central African Republic	Berbérati	2000-2017	-0.029	0.001	0.030
Central African Republic	Bimbo	2000	0.434	0.505	0.606
Central African Republic	Bimbo	2017	0.468	0.603	0.756
Central African Republic	Bimbo	2000-2017	-0.025	0.007	0.041
Central African Republic	Birao	2000	0.594	0.704	0.813
Central African Republic	Birao	2017	0.597	0.719	0.854
Central African Republic	Birao	2000-2017	-0.024	0.003	0.031
Central African Republic	Boali	2000	0.463	0.562	0.695
Central African Republic	Boali	2017	0.481	0.628	0.770
Central African Republic	Boali	2000-2017	-0.011	0.019	0.049
Central African Republic	Bocaranga	2000	0.473	0.566	0.685
Central African Republic	Bocaranga	2017	0.483	0.609	0.753
Central African Republic	Bocaranga	2000-2017	-0.018	0.013	0.041
Central African Republic	Boda	2000	0.420	0.495	0.589
Central African Republic	Boda	2017	0.455	0.575	0.727
Central African Republic	Boda	2000-2017	-0.009	0.023	0.053
Central African Republic	Bossangoa	2000	0.442	0.547	0.676
Central African Republic	Bossangoa	2017	0.480	0.616	0.772
Central African Republic	Bossangoa	2000-2017	-0.011	0.022	0.050
Central African Republic	Bouar	2000	0.411	0.491	0.588
Central African Republic	Bouar	2017	0.462	0.576	0.744
Central African Republic	Bouar	2000-2017	-0.007	0.024	0.055
Central African Republic	Bouca	2000	0.457	0.560	0.660
Central African Republic	Bouca	2017	0.504	0.632	0.803
Central African Republic	Bouca	2000-2017	0.000	0.027	0.058
Central African Republic	Bozoum	2000	0.452	0.556	0.687
Central African Republic	Bozoum	2017	0.481	0.608	0.752
Central African Republic	Bozoum	2000-2017	-0.021	0.011	0.039
Central African Republic	Bria	2000	0.562	0.661	0.786
Central African Republic	Bria	2017	0.484	0.609	0.733
Central African Republic	Bria	2000-2017	-0.031	0.000	0.029
Central African Republic	Carnot	2000	0.418	0.494	0.588
Central African Republic	Carnot	2017	0.441	0.553	0.665
Central African Republic	Carnot	2000-2017	-0.020	0.008	0.037
Central African Republic	Damara	2000	0.490	0.574	0.684
Central African Republic	Damara	2017	0.513	0.650	0.786
Central African Republic	Damara	2000-2017	-0.016	0.012	0.038
Central African Republic	Dékoa	2000	0.544	0.644	0.758

Table 2: Diarrhea DALYs rate by unit *(continued)*

Country	Unit	year	mean rate	lower rate	upper rate
Central African Republic	Dékoa	2017	0.550	0.698	0.885
Central African Republic	Dékoa	2000-2017	-0.020	0.010	0.041
Central African Republic	Djemah	2000	0.537	0.649	0.779
Central African Republic	Djemah	2017	0.543	0.652	0.793
Central African Republic	Djemah	2000-2017	-0.029	-0.001	0.026
Central African Republic	Gambo-Ouango	2000	0.486	0.584	0.693
Central African Republic	Gambo-Ouango	2017	0.425	0.541	0.669
Central African Republic	Gambo-Ouango	2000-2017	-0.030	0.000	0.037
Central African Republic	Gamboula	2000	0.426	0.522	0.646
Central African Republic	Gamboula	2017	0.442	0.556	0.694
Central African Republic	Gamboula	2000-2017	-0.028	0.002	0.031
Central African Republic	Grimari	2000	0.468	0.580	0.689
Central African Republic	Grimari	2017	0.484	0.623	0.795
Central African Republic	Grimari	2000-2017	-0.019	0.013	0.039
Central African Republic	Ippy	2000	0.508	0.604	0.705
Central African Republic	Ippy	2017	0.491	0.607	0.761
Central African Republic	Ippy	2000-2017	-0.018	0.014	0.044
Central African Republic	Kabo	2000	0.457	0.559	0.670
Central African Republic	Kabo	2017	0.473	0.604	0.736
Central African Republic	Kabo	2000-2017	-0.037	-0.002	0.031
Central African Republic	Kaga-Bandoro	2000	0.486	0.581	0.690
Central African Republic	Kaga-Bandoro	2017	0.507	0.641	0.824
Central African Republic	Kaga-Bandoro	2000-2017	-0.021	0.013	0.042
Central African Republic	Kembé	2000	0.535	0.658	0.769
Central African Republic	Kembé	2017	0.491	0.623	0.768
Central African Republic	Kembé	2000-2017	-0.020	0.008	0.040
Central African Republic	Kouango	2000	0.463	0.564	0.670
Central African Republic	Kouango	2017	0.462	0.595	0.726
Central African Republic	Kouango	2000-2017	-0.012	0.017	0.046
Central African Republic	M'Baïki	2000	0.408	0.476	0.557
Central African Republic	M'Baïki	2017	0.458	0.583	0.707
Central African Republic	M'Baïki	2000-2017	-0.001	0.028	0.061
Central African Republic	Markounda	2000	0.461	0.567	0.698
Central African Republic	Markounda	2017	0.487	0.608	0.745
Central African Republic	Markounda	2000-2017	-0.014	0.014	0.048
Central African Republic	Mbrès	2000	0.477	0.586	0.702
Central African Republic	Mbrès	2017	0.492	0.643	0.829
Central African Republic	Mbrès	2000-2017	-0.023	0.011	0.041
Central African Republic	Mingala	2000	0.535	0.653	0.785
Central African Republic	Mingala	2017	0.525	0.651	0.803
Central African Republic	Mingala	2000-2017	-0.017	0.017	0.045
Central African Republic	Mobaye	2000	0.549	0.660	0.776
Central African Republic	Mobaye	2017	0.481	0.616	0.759
Central African Republic	Mobaye	2000-2017	-0.028	0.008	0.043
Central African Republic	Mongoumba	2000	0.410	0.497	0.593
Central African Republic	Mongoumba	2017	0.426	0.538	0.665
Central African Republic	Mongoumba	2000-2017	-0.017	0.016	0.048
Central African Republic	Ndélé	2000	0.494	0.590	0.692
Central African Republic	Ndélé	2017	0.516	0.631	0.749
Central African Republic	Ndélé	2000-2017	-0.022	0.006	0.031
Central African Republic	Nola	2000	0.417	0.502	0.594
Central African Republic	Nola	2017	0.436	0.545	0.684
Central African Republic	Nola	2000-2017	-0.014	0.016	0.046
Central African Republic	Obo	2000	0.485	0.595	0.739
Central African Republic	Obo	2017	0.488	0.619	0.769
Central African Republic	Obo	2000-2017	-0.030	0.000	0.033
Central African Republic	Ouadda	2000	0.526	0.622	0.730
Central African Republic	Ouadda	2017	0.484	0.600	0.731
Central African Republic	Ouadda	2000-2017	-0.032	0.000	0.031
Central African Republic	Ouanda Djallé	2000	0.520	0.645	0.778
Central African Republic	Ouanda Djallé	2017	0.513	0.654	0.808

Table 2: Diarrhea DALYs rate by unit *(continued)*

Country	Unit	year	mean rate	lower rate	upper rate
Central African Republic	Ouanda Djallé	2000-2017	-0.029	0.004	0.037
Central African Republic	Paoua	2000	0.495	0.597	0.720
Central African Republic	Paoua	2017	0.513	0.632	0.785
Central African Republic	Paoua	2000-2017	-0.018	0.009	0.037
Central African Republic	Rafaï	2000	0.479	0.580	0.698
Central African Republic	Rafaï	2017	0.482	0.580	0.720
Central African Republic	Rafaï	2000-2017	-0.018	0.011	0.037
Central African Republic	Sibut	2000	0.539	0.632	0.755
Central African Republic	Sibut	2017	0.546	0.694	0.862
Central African Republic	Sibut	2000-2017	-0.021	0.006	0.035
Central African Republic	Yalinga	2000	0.542	0.623	0.722
Central African Republic	Yalinga	2017	0.500	0.600	0.699
Central African Republic	Yalinga	2000-2017	-0.030	-0.003	0.021
Central African Republic	Yaloké	2000	0.449	0.546	0.650
Central African Republic	Yaloké	2017	0.480	0.597	0.740
Central African Republic	Yaloké	2000-2017	-0.024	0.009	0.039
Central African Republic	Zémio	2000	0.465	0.600	0.758
Central African Republic	Zémio	2017	0.478	0.602	0.739
Central African Republic	Zémio	2000-2017	-0.025	0.006	0.037
Chad	Aboudeïa	2000	0.521	0.734	0.980
Chad	Aboudeïa	2017	0.346	0.557	0.861
Chad	Aboudeïa	2000-2017	-0.102	-0.002	0.099
Chad	Assoungha	2000	0.591	0.795	1.081
Chad	Assoungha	2017	0.311	0.542	0.893
Chad	Assoungha	2000-2017	-0.115	-0.005	0.112
Chad	Baguirmi	2000	0.664	0.881	1.139
Chad	Baguirmi	2017	0.407	0.630	0.940
Chad	Baguirmi	2000-2017	-0.111	-0.023	0.064
Chad	Barh Azoum	2000	0.591	0.801	1.061
Chad	Barh Azoum	2017	0.365	0.584	0.909
Chad	Barh Azoum	2000-2017	-0.112	-0.003	0.090
Chad	Barh El Gazel	2000	0.528	0.714	0.997
Chad	Barh El Gazel	2017	0.388	0.573	0.897
Chad	Barh El Gazel	2000-2017	-0.108	-0.010	0.084
Chad	Barh Köh	2000	0.707	0.933	1.175
Chad	Barh Köh	2017	0.364	0.586	0.917
Chad	Barh Köh	2000-2017	-0.116	-0.020	0.072
Chad	Barh Sara	2000	0.739	0.965	1.218
Chad	Barh Sara	2017	0.338	0.590	0.918
Chad	Barh Sara	2000-2017	-0.124	-0.020	0.084
Chad	Barh Signaka	2000	0.490	0.693	0.927
Chad	Barh Signaka	2017	0.311	0.538	0.821
Chad	Barh Signaka	2000-2017	-0.108	-0.009	0.093
Chad	Batha Est	2000	0.501	0.723	0.962
Chad	Batha Est	2017	0.297	0.548	0.812
Chad	Batha Est	2000-2017	-0.107	-0.004	0.093
Chad	Batha Oues	2000	0.490	0.702	0.977
Chad	Batha Oues	2017	0.332	0.557	0.842
Chad	Batha Oues	2000-2017	-0.112	-0.007	0.087
Chad	Béré	2000	0.887	1.146	1.454
Chad	Béré	2017	0.335	0.602	0.967
Chad	Béré	2000-2017	-0.131	-0.024	0.081
Chad	Biltine	2000	0.539	0.738	1.009
Chad	Biltine	2017	0.288	0.495	0.797
Chad	Biltine	2000-2017	-0.117	-0.006	0.101
Chad	Bitkine	2000	0.487	0.694	0.933
Chad	Bitkine	2017	0.309	0.546	0.876
Chad	Bitkine	2000-2017	-0.108	-0.009	0.084
Chad	Borkou	2000	0.474	0.697	1.017
Chad	Borkou	2017	0.317	0.478	0.700
Chad	Borkou	2000-2017	-0.093	-0.006	0.077

Table 2: Diarrhea DALYs rate by unit *(continued)*

Country	Unit	year	mean rate	lower rate	upper rate
Chad	Dababa	2000	0.543	0.759	0.980
Chad	Dababa	2017	0.370	0.585	0.905
Chad	Dababa	2000-2017	-0.114	-0.013	0.071
Chad	Dagana	2000	0.645	0.828	1.116
Chad	Dagana	2017	0.407	0.623	0.983
Chad	Dagana	2000-2017	-0.118	-0.020	0.075
Chad	Dar Tama	2000	0.553	0.770	1.028
Chad	Dar Tama	2017	0.280	0.482	0.774
Chad	Dar Tama	2000-2017	-0.122	-0.009	0.102
Chad	Djourf Al Ahmar	2000	0.552	0.775	1.068
Chad	Djourf Al Ahmar	2000	0.576	0.788	1.101
Chad	Djourf Al Ahmar	2017	0.334	0.561	0.864
Chad	Djourf Al Ahmar	2017	0.329	0.565	0.904
Chad	Djourf Al Ahmar	2000-2017	-0.109	-0.007	0.095
Chad	Djourf Al Ahmar	2000-2017	-0.116	-0.005	0.103
Chad	Dodjé	2000	0.790	1.027	1.328
Chad	Dodjé	2017	0.310	0.551	0.918
Chad	Dodjé	2000-2017	-0.136	-0.028	0.068
Chad	Ennedi Est	2000	0.455	0.702	0.995
Chad	Ennedi Est	2017	0.291	0.442	0.679
Chad	Ennedi Est	2000-2017	-0.113	-0.005	0.112
Chad	Ennedi Ouest	2000	0.496	0.720	1.069
Chad	Ennedi Ouest	2017	0.305	0.469	0.716
Chad	Ennedi Ouest	2000-2017	-0.106	-0.007	0.087
Chad	Fitri	2000	0.506	0.698	0.953
Chad	Fitri	2017	0.334	0.546	0.846
Chad	Fitri	2000-2017	-0.096	-0.001	0.095
Chad	Grande Sido	2000	0.702	0.913	1.145
Chad	Grande Sido	2017	0.345	0.584	0.931
Chad	Grande Sido	2000-2017	-0.111	-0.016	0.078
Chad	Guéra	2000	0.551	0.772	0.992
Chad	Guéra	2017	0.362	0.577	0.895
Chad	Guéra	2000-2017	-0.093	-0.006	0.087
Chad	Haraze Al Biar	2000	0.730	0.911	1.146
Chad	Haraze Al Biar	2017	0.393	0.616	0.956
Chad	Haraze Al Biar	2000-2017	-0.122	-0.029	0.057
Chad	Haraze Mangueigne	2000	0.659	0.864	1.090
Chad	Haraze Mangueigne	2017	0.377	0.596	0.894
Chad	Haraze Mangueigne	2000-2017	-0.099	-0.002	0.086
Chad	Kabbia	2000	0.801	1.016	1.265
Chad	Kabbia	2017	0.336	0.578	0.909
Chad	Kabbia	2000-2017	-0.135	-0.024	0.063
Chad	Kanem	2000	0.545	0.741	1.005
Chad	Kanem	2017	0.405	0.590	0.921
Chad	Kanem	2000-2017	-0.121	-0.014	0.090
Chad	Kobé	2000	0.489	0.695	0.917
Chad	Kobé	2017	0.261	0.435	0.694
Chad	Kobé	2000-2017	-0.127	-0.010	0.092
Chad	Lac Iro	2000	0.663	0.891	1.137
Chad	Lac Iro	2017	0.346	0.584	0.900
Chad	Lac Iro	2000-2017	-0.111	-0.011	0.081
Chad	Lac Léré	2000	0.825	1.052	1.340
Chad	Lac Léré	2017	0.340	0.586	1.000
Chad	Lac Léré	2000-2017	-0.137	-0.035	0.074
Chad	Lac Wey	2000	0.865	1.117	1.414
Chad	Lac Wey	2017	0.335	0.592	0.987
Chad	Lac Wey	2000-2017	-0.136	-0.027	0.074
Chad	Lanya	2000	0.855	1.102	1.381
Chad	Lanya	2017	0.343	0.617	0.995
Chad	Lanya	2000-2017	-0.129	-0.026	0.078
Chad	Loug Chari	2000	0.661	0.921	1.226

Table 2: Diarrhea DALYs rate by unit *(continued)*

Country	Unit	year	mean rate	lower rate	upper rate
Chad	Loug Chari	2017	0.366	0.626	1.008
Chad	Loug Chari	2000-2017	-0.121	-0.019	0.075
Chad	Mamdi	2000	0.578	0.792	1.107
Chad	Mamdi	2017	0.371	0.594	0.928
Chad	Mamdi	2000-2017	-0.113	-0.020	0.074
Chad	Mandoul Occidental	2000	0.738	1.007	1.273
Chad	Mandoul Occidental	2017	0.330	0.595	0.957
Chad	Mandoul Occidental	2000-2017	-0.122	-0.024	0.089
Chad	Mandoul Oriental	2000	0.746	0.979	1.254
Chad	Mandoul Oriental	2017	0.339	0.592	0.926
Chad	Mandoul Oriental	2000-2017	-0.124	-0.025	0.073
Chad	Mangalmé	2000	0.510	0.715	0.941
Chad	Mangalmé	2017	0.307	0.546	0.832
Chad	Mangalmé	2000-2017	-0.111	-0.008	0.086
Chad	Mayo-Boneye	2000	0.768	0.972	1.228
Chad	Mayo-Boneye	2017	0.376	0.621	0.937
Chad	Mayo-Boneye	2000-2017	-0.121	-0.026	0.064
Chad	Mayo-Dallah	2000	0.806	1.003	1.250
Chad	Mayo-Dallah	2017	0.327	0.561	0.919
Chad	Mayo-Dallah	2000-2017	-0.130	-0.024	0.067
Chad	Mont Illi	2000	0.829	1.048	1.309
Chad	Mont Illi	2017	0.361	0.595	0.941
Chad	Mont Illi	2000-2017	-0.132	-0.027	0.056
Chad	Monts de Lam	2000	0.743	0.954	1.221
Chad	Monts de Lam	2017	0.303	0.539	0.893
Chad	Monts de Lam	2000-2017	-0.137	-0.023	0.072
Chad	N'Djamena	2000	0.734	0.935	1.182
Chad	N'Djamena	2000	0.731	0.911	1.146
Chad	N'Djamena	2017	0.401	0.626	0.958
Chad	N'Djamena	2017	0.375	0.591	0.893
Chad	N'Djamena	2000-2017	-0.125	-0.035	0.057
Chad	N'Djamena	2000-2017	-0.120	-0.032	0.063
Chad	Ngourkosso	2000	0.872	1.144	1.463
Chad	Ngourkosso	2017	0.339	0.625	1.033
Chad	Ngourkosso	2000-2017	-0.133	-0.026	0.082
Chad	Nokou	2000	0.504	0.733	1.045
Chad	Nokou	2017	0.378	0.588	0.885
Chad	Nokou	2000-2017	-0.113	-0.014	0.090
Chad	Nya Pendé	2000	0.798	1.007	1.263
Chad	Nya Pendé	2017	0.328	0.570	0.923
Chad	Nya Pendé	2000-2017	-0.127	-0.027	0.075
Chad	Ouara	2000	0.554	0.770	1.051
Chad	Ouara	2017	0.314	0.544	0.854
Chad	Ouara	2000-2017	-0.119	-0.004	0.097
Chad	Pendé	2000	0.793	1.054	1.313
Chad	Pendé	2017	0.327	0.601	0.977
Chad	Pendé	2000-2017	-0.122	-0.024	0.079
Chad	Sila	2000	0.636	0.841	1.101
Chad	Sila	2017	0.367	0.582	0.890
Chad	Sila	2000-2017	-0.117	-0.008	0.087
Chad	Tandjilé Est	2000	0.799	1.062	1.342
Chad	Tandjilé Est	2017	0.340	0.613	0.964
Chad	Tandjilé Est	2000-2017	-0.125	-0.023	0.078
Chad	Tandjilé Ouest	2000	0.880	1.134	1.420
Chad	Tandjilé Ouest	2017	0.343	0.593	0.975
Chad	Tandjilé Ouest	2000-2017	-0.133	-0.025	0.071
Chad	Tibesti	2000	0.431	0.626	0.889
Chad	Tibesti	2017	0.292	0.456	0.709
Chad	Tibesti	2000-2017	-0.094	-0.013	0.066
Chad	Wayi	2000	0.596	0.794	1.069
Chad	Wayi	2017	0.378	0.600	0.946

Table 2: Diarrhea DALYs rate by unit *(continued)*

Country	Unit	year	mean rate	lower rate	upper rate
Chad	Wayi	2000-2017	-0.125	-0.017	0.095
Côte d'Ivoire	Abidjan	2000	0.154	0.206	0.277
Côte d'Ivoire	Abidjan	2017	0.095	0.154	0.225
Côte d'Ivoire	Abidjan	2000-2017	-0.110	-0.008	0.093
Côte d'Ivoire	Agnéby-Tiassa	2000	0.182	0.243	0.341
Côte d'Ivoire	Agnéby-Tiassa	2017	0.106	0.170	0.254
Côte d'Ivoire	Agnéby-Tiassa	2000-2017	-0.100	-0.009	0.091
Côte d'Ivoire	Bafing	2000	0.211	0.287	0.381
Côte d'Ivoire	Bafing	2017	0.105	0.168	0.247
Côte d'Ivoire	Bafing	2000-2017	-0.152	-0.054	0.028
Côte d'Ivoire	Bagoué	2000	0.220	0.306	0.396
Côte d'Ivoire	Bagoué	2017	0.105	0.167	0.248
Côte d'Ivoire	Bagoué	2000-2017	-0.135	-0.041	0.044
Côte d'Ivoire	Bélier	2000	0.205	0.277	0.376
Côte d'Ivoire	Bélier	2017	0.117	0.190	0.289
Côte d'Ivoire	Bélier	2000-2017	-0.102	-0.015	0.084
Côte d'Ivoire	Béré	2000	0.219	0.286	0.381
Côte d'Ivoire	Béré	2017	0.106	0.178	0.264
Côte d'Ivoire	Béré	2000-2017	-0.128	-0.033	0.068
Côte d'Ivoire	Bounkani	2000	0.251	0.352	0.489
Côte d'Ivoire	Bounkani	2017	0.139	0.211	0.317
Côte d'Ivoire	Bounkani	2000-2017	-0.109	-0.019	0.066
Côte d'Ivoire	Cavally	2000	0.243	0.318	0.422
Côte d'Ivoire	Cavally	2017	0.114	0.182	0.263
Côte d'Ivoire	Cavally	2000-2017	-0.118	-0.042	0.032
Côte d'Ivoire	Folon	2000	0.189	0.273	0.368
Côte d'Ivoire	Folon	2017	0.105	0.163	0.252
Côte d'Ivoire	Folon	2000-2017	-0.137	-0.038	0.047
Côte d'Ivoire	Gbeke	2000	0.214	0.286	0.374
Côte d'Ivoire	Gbeke	2017	0.104	0.179	0.272
Côte d'Ivoire	Gbeke	2000-2017	-0.122	-0.029	0.072
Côte d'Ivoire	Gbôkle	2000	0.219	0.300	0.401
Côte d'Ivoire	Gbôkle	2017	0.122	0.190	0.287
Côte d'Ivoire	Gbôkle	2000-2017	-0.102	-0.007	0.081
Côte d'Ivoire	Gôh	2000	0.232	0.321	0.428
Côte d'Ivoire	Gôh	2017	0.111	0.181	0.259
Côte d'Ivoire	Gôh	2000-2017	-0.119	-0.028	0.068
Côte d'Ivoire	Gontougo	2000	0.209	0.294	0.393
Côte d'Ivoire	Gontougo	2017	0.131	0.202	0.301
Côte d'Ivoire	Gontougo	2000-2017	-0.097	-0.007	0.093
Côte d'Ivoire	Grands Ponts	2000	0.176	0.241	0.328
Côte d'Ivoire	Grands Ponts	2017	0.106	0.171	0.261
Côte d'Ivoire	Grands Ponts	2000-2017	-0.097	-0.006	0.096
Côte d'Ivoire	Guémon	2000	0.239	0.324	0.425
Côte d'Ivoire	Guémon	2017	0.114	0.181	0.265
Côte d'Ivoire	Guémon	2000-2017	-0.136	-0.051	0.035
Côte d'Ivoire	Hambol	2000	0.220	0.304	0.394
Côte d'Ivoire	Hambol	2017	0.106	0.183	0.264
Côte d'Ivoire	Hambol	2000-2017	-0.120	-0.027	0.068
Côte d'Ivoire	Haut-Sassandra	2000	0.253	0.342	0.451
Côte d'Ivoire	Haut-Sassandra	2017	0.108	0.176	0.257
Côte d'Ivoire	Haut-Sassandra	2000-2017	-0.144	-0.046	0.043
Côte d'Ivoire	Iffou	2000	0.197	0.267	0.365
Côte d'Ivoire	Iffou	2017	0.126	0.195	0.287
Côte d'Ivoire	Iffou	2000-2017	-0.105	-0.006	0.103
Côte d'Ivoire	Indénié-Djuablin	2000	0.161	0.223	0.297
Côte d'Ivoire	Indénié-Djuablin	2017	0.106	0.165	0.243
Côte d'Ivoire	Indénié-Djuablin	2000-2017	-0.107	-0.002	0.097
Côte d'Ivoire	Kabadougou	2000	0.195	0.273	0.357
Côte d'Ivoire	Kabadougou	2017	0.099	0.161	0.248
Côte d'Ivoire	Kabadougou	2000-2017	-0.147	-0.047	0.046

Table 2: Diarrhea DALYs rate by unit *(continued)*

Country	Unit	year	mean rate	lower rate	upper rate
Côte d'Ivoire	La Mé	2000	0.178	0.238	0.327
Côte d'Ivoire	La Mé	2017	0.114	0.181	0.259
Côte d'Ivoire	La Mé	2000-2017	-0.111	-0.008	0.086
Côte d'Ivoire	Lôh-Djiboua	2000	0.194	0.279	0.386
Côte d'Ivoire	Lôh-Djiboua	2017	0.111	0.181	0.272
Côte d'Ivoire	Lôh-Djiboua	2000-2017	-0.103	-0.012	0.083
Côte d'Ivoire	Marahoué	2000	0.239	0.319	0.429
Côte d'Ivoire	Marahoué	2017	0.101	0.171	0.253
Côte d'Ivoire	Marahoué	2000-2017	-0.132	-0.038	0.063
Côte d'Ivoire	Moronou	2000	0.197	0.261	0.362
Côte d'Ivoire	Moronou	2017	0.123	0.189	0.286
Côte d'Ivoire	Moronou	2000-2017	-0.105	-0.007	0.087
Côte d'Ivoire	N'zi	2000	0.201	0.270	0.364
Côte d'Ivoire	N'zi	2017	0.122	0.191	0.293
Côte d'Ivoire	N'zi	2000-2017	-0.107	-0.013	0.084
Côte d'Ivoire	Nawa	2000	0.231	0.312	0.406
Côte d'Ivoire	Nawa	2017	0.114	0.184	0.273
Côte d'Ivoire	Nawa	2000-2017	-0.108	-0.020	0.063
Côte d'Ivoire	Poro	2000	0.226	0.303	0.402
Côte d'Ivoire	Poro	2017	0.102	0.165	0.244
Côte d'Ivoire	Poro	2000-2017	-0.126	-0.035	0.065
Côte d'Ivoire	San-Pédro	2000	0.202	0.285	0.380
Côte d'Ivoire	San-Pédro	2017	0.115	0.182	0.271
Côte d'Ivoire	San-Pédro	2000-2017	-0.089	0.001	0.080
Côte d'Ivoire	Sud Comoé	2000	0.184	0.251	0.335
Côte d'Ivoire	Sud Comoé	2017	0.113	0.177	0.264
Côte d'Ivoire	Sud Comoé	2000-2017	-0.110	-0.016	0.082
Côte d'Ivoire	Tchologo	2000	0.230	0.317	0.422
Côte d'Ivoire	Tchologo	2017	0.117	0.176	0.257
Côte d'Ivoire	Tchologo	2000-2017	-0.115	-0.026	0.069
Côte d'Ivoire	Tonkpi	2000	0.232	0.316	0.427
Côte d'Ivoire	Tonkpi	2017	0.112	0.179	0.263
Côte d'Ivoire	Tonkpi	2000-2017	-0.137	-0.059	0.029
Côte d'Ivoire	Worodougou	2000	0.211	0.279	0.366
Côte d'Ivoire	Worodougou	2017	0.102	0.175	0.259
Côte d'Ivoire	Worodougou	2000-2017	-0.137	-0.038	0.061
Côte d'Ivoire	Yamoussoukro	2000	0.200	0.279	0.380
Côte d'Ivoire	Yamoussoukro	2017	0.113	0.185	0.285
Côte d'Ivoire	Yamoussoukro	2000-2017	-0.106	-0.016	0.094
Democratic Republic of the Congo	Aba	2000	0.220	0.282	0.359
Democratic Republic of the Congo	Aba	2017	0.098	0.124	0.158
Democratic Republic of the Congo	Aba	2000-2017	-0.079	-0.043	-0.009
Democratic Republic of the Congo	Aketi	2000	0.207	0.265	0.326
Democratic Republic of the Congo	Aketi	2017	0.113	0.142	0.174
Democratic Republic of the Congo	Aketi	2000-2017	-0.049	-0.023	0.007
Democratic Republic of the Congo	Aketi (ville)	2000	0.205	0.276	0.352
Democratic Republic of the Congo	Aketi (ville)	2017	0.113	0.145	0.183
Democratic Republic of the Congo	Aketi (ville)	2000-2017	-0.063	-0.030	0.006
Democratic Republic of the Congo	Ango	2000	0.221	0.277	0.335
Democratic Republic of the Congo	Ango	2017	0.113	0.137	0.171
Democratic Republic of the Congo	Ango	2000-2017	-0.045	-0.022	0.005
Democratic Republic of the Congo	Ariwara	2000	0.237	0.296	0.364
Democratic Republic of the Congo	Ariwara	2017	0.086	0.110	0.130
Democratic Republic of the Congo	Ariwara	2000-2017	-0.103	-0.060	-0.029
Democratic Republic of the Congo	Aru	2000	0.234	0.296	0.354
Democratic Republic of the Congo	Aru	2017	0.090	0.113	0.134
Democratic Republic of the Congo	Aru	2000-2017	-0.089	-0.052	-0.023
Democratic Republic of the Congo	Aru (ville)	2000	0.232	0.302	0.372
Democratic Republic of the Congo	Aru (ville)	2017	0.085	0.110	0.136
Democratic Republic of the Congo	Aru (ville)	2000-2017	-0.106	-0.068	-0.037
Democratic Republic of the Congo	Bafwasende	2000	0.214	0.262	0.323

Table 2: Diarrhea DALYs rate by unit *(continued)*

Country	Unit	year	mean rate	lower rate	upper rate
Democratic Republic of the Congo	Bafwasende	2017	0.098	0.124	0.151
Democratic Republic of the Congo	Bafwasende	2000-2017	-0.058	-0.029	-0.001
Democratic Republic of the Congo	Bagata	2000	0.227	0.276	0.343
Democratic Republic of the Congo	Bagata	2017	0.094	0.115	0.141
Democratic Republic of the Congo	Bagata	2000-2017	-0.072	-0.042	-0.014
Democratic Republic of the Congo	Bambesa	2000	0.203	0.265	0.323
Democratic Republic of the Congo	Bambesa	2017	0.115	0.141	0.177
Democratic Republic of the Congo	Bambesa	2000-2017	-0.046	-0.019	0.011
Democratic Republic of the Congo	Banalia	2000	0.203	0.256	0.320
Democratic Republic of the Congo	Banalia	2017	0.105	0.134	0.162
Democratic Republic of the Congo	Banalia	2000-2017	-0.057	-0.026	0.004
Democratic Republic of the Congo	Bandundu	2000	0.190	0.245	0.318
Democratic Republic of the Congo	Bandundu	2017	0.086	0.110	0.138
Democratic Republic of the Congo	Bandundu	2000-2017	-0.073	-0.034	0.001
Democratic Republic of the Congo	Bangu	2000	0.184	0.236	0.291
Democratic Republic of the Congo	Bangu	2017	0.080	0.105	0.131
Democratic Republic of the Congo	Bangu	2000-2017	-0.069	-0.040	-0.005
Democratic Republic of the Congo	Baraka	2000	0.238	0.300	0.381
Democratic Republic of the Congo	Baraka	2017	0.102	0.129	0.168
Democratic Republic of the Congo	Baraka	2000-2017	-0.077	-0.048	-0.018
Democratic Republic of the Congo	Basankusu	2000	0.232	0.295	0.376
Democratic Republic of the Congo	Basankusu	2017	0.102	0.129	0.162
Democratic Republic of the Congo	Basankusu	2000-2017	-0.059	-0.029	0.000
Democratic Republic of the Congo	Basankusu (ville)	2000	0.229	0.296	0.390
Democratic Republic of the Congo	Basankusu (ville)	2017	0.100	0.130	0.169
Democratic Republic of the Congo	Basankusu (ville)	2000-2017	-0.061	-0.029	0.003
Democratic Republic of the Congo	Basoko	2000	0.200	0.256	0.316
Democratic Republic of the Congo	Basoko	2017	0.104	0.133	0.165
Democratic Republic of the Congo	Basoko	2000-2017	-0.062	-0.031	-0.002
Democratic Republic of the Congo	Basoko (ville)	2000	0.179	0.246	0.318
Democratic Republic of the Congo	Basoko (ville)	2017	0.087	0.117	0.156
Democratic Republic of the Congo	Basoko (ville)	2000-2017	-0.066	-0.032	0.002
Democratic Republic of the Congo	Befale	2000	0.227	0.285	0.354
Democratic Republic of the Congo	Befale	2017	0.097	0.125	0.151
Democratic Republic of the Congo	Befale	2000-2017	-0.063	-0.033	-0.006
Democratic Republic of the Congo	Bena-Dibele	2000	NA	NA	NA
Democratic Republic of the Congo	Bena-Dibele	2017	NA	NA	NA
Democratic Republic of the Congo	Bena-Dibele	2000-2017	NA	NA	NA
Democratic Republic of the Congo	Beni	2000	0.199	0.250	0.317
Democratic Republic of the Congo	Beni	2017	0.092	0.116	0.148
Democratic Republic of the Congo	Beni	2000-2017	-0.068	-0.036	-0.002
Democratic Republic of the Congo	Bikoro	2000	0.204	0.264	0.337
Democratic Republic of the Congo	Bikoro	2017	0.092	0.118	0.148
Democratic Republic of the Congo	Bikoro	2000-2017	-0.066	-0.033	-0.004
Democratic Republic of the Congo	Boende	2000	0.224	0.280	0.352
Democratic Republic of the Congo	Boende	2017	0.103	0.131	0.165
Democratic Republic of the Congo	Boende	2000-2017	-0.057	-0.027	0.002
Democratic Republic of the Congo	Boende (ville)	2000	0.223	0.290	0.382
Democratic Republic of the Congo	Boende (ville)	2017	0.097	0.130	0.171
Democratic Republic of the Congo	Boende (ville)	2000-2017	-0.068	-0.029	0.007
Democratic Republic of the Congo	Bokungu	2000	0.219	0.282	0.351
Democratic Republic of the Congo	Bokungu	2017	0.107	0.132	0.165
Democratic Republic of the Congo	Bokungu	2000-2017	-0.054	-0.024	0.007
Democratic Republic of the Congo	Bolobo	2000	0.205	0.263	0.339
Democratic Republic of the Congo	Bolobo	2017	0.084	0.106	0.126
Democratic Republic of the Congo	Bolobo	2000-2017	-0.068	-0.036	-0.003
Democratic Republic of the Congo	Bolobo (ville)	2000	0.190	0.251	0.328
Democratic Republic of the Congo	Bolobo (ville)	2017	0.081	0.103	0.128
Democratic Republic of the Congo	Bolobo (ville)	2000-2017	-0.074	-0.032	0.002
Democratic Republic of the Congo	Bolomba	2000	0.233	0.282	0.341
Democratic Republic of the Congo	Bolomba	2017	0.095	0.116	0.141

Table 2: Diarrhea DALYs rate by unit *(continued)*

Country	Unit	year	mean rate	lower rate	upper rate
Democratic Republic of the Congo	Bolomba	2000-2017	-0.067	-0.038	-0.012
Democratic Republic of the Congo	Boma	2000	0.192	0.251	0.322
Democratic Republic of the Congo	Boma	2017	0.078	0.103	0.137
Democratic Republic of the Congo	Boma	2000-2017	-0.067	-0.036	0.002
Democratic Republic of the Congo	Bomongo	2000	0.226	0.277	0.332
Democratic Republic of the Congo	Bomongo	2017	0.098	0.118	0.143
Democratic Republic of the Congo	Bomongo	2000-2017	-0.063	-0.039	-0.013
Democratic Republic of the Congo	Bondo	2000	0.232	0.277	0.335
Democratic Republic of the Congo	Bondo	2017	0.110	0.138	0.166
Democratic Republic of the Congo	Bondo	2000-2017	-0.054	-0.031	-0.004
Democratic Republic of the Congo	Bondo (ville)	2000	0.199	0.261	0.333
Democratic Republic of the Congo	Bondo (ville)	2017	0.094	0.124	0.157
Democratic Republic of the Congo	Bondo (ville)	2000-2017	-0.066	-0.032	0.004
Democratic Republic of the Congo	Bongandanga	2000	0.237	0.288	0.343
Democratic Republic of the Congo	Bongandanga	2017	0.104	0.126	0.156
Democratic Republic of the Congo	Bongandanga	2000-2017	-0.060	-0.034	-0.005
Democratic Republic of the Congo	Bosobolo	2000	0.246	0.297	0.348
Democratic Republic of the Congo	Bosobolo	2017	0.121	0.152	0.181
Democratic Republic of the Congo	Bosobolo	2000-2017	-0.069	-0.039	-0.009
Democratic Republic of the Congo	Budjala	2000	0.245	0.302	0.379
Democratic Republic of the Congo	Budjala	2017	0.112	0.141	0.173
Democratic Republic of the Congo	Budjala	2000-2017	-0.057	-0.029	0.003
Democratic Republic of the Congo	Bukama	2000	0.247	0.294	0.357
Democratic Republic of the Congo	Bukama	2017	0.100	0.125	0.153
Democratic Republic of the Congo	Bukama	2000-2017	-0.076	-0.049	-0.021
Democratic Republic of the Congo	Bukavu	2000	0.275	0.330	0.387
Democratic Republic of the Congo	Bukavu	2017	0.108	0.134	0.163
Democratic Republic of the Congo	Bukavu	2000-2017	-0.087	-0.051	-0.021
Democratic Republic of the Congo	Bulungu	2000	0.217	0.269	0.325
Democratic Republic of the Congo	Bulungu	2017	0.092	0.112	0.136
Democratic Republic of the Congo	Bulungu	2000-2017	-0.071	-0.045	-0.012
Democratic Republic of the Congo	Bulungu (ville)	2000	0.215	0.274	0.339
Democratic Republic of the Congo	Bulungu (ville)	2017	0.090	0.113	0.142
Democratic Republic of the Congo	Bulungu (ville)	2000-2017	-0.080	-0.048	-0.016
Democratic Republic of the Congo	Bumba	2000	0.233	0.290	0.357
Democratic Republic of the Congo	Bumba	2017	0.107	0.136	0.171
Democratic Republic of the Congo	Bumba	2000-2017	-0.067	-0.037	-0.011
Democratic Republic of the Congo	Bumba (ville)	2000	0.221	0.294	0.384
Democratic Republic of the Congo	Bumba (ville)	2017	0.095	0.127	0.165
Democratic Republic of the Congo	Bumba (ville)	2000-2017	-0.064	-0.024	0.010
Democratic Republic of the Congo	Bunia	2000	0.228	0.291	0.359
Democratic Republic of the Congo	Bunia	2017	0.089	0.111	0.141
Democratic Republic of the Congo	Bunia	2000-2017	-0.092	-0.059	-0.023
Democratic Republic of the Congo	Businga	2000	0.241	0.298	0.375
Democratic Republic of the Congo	Businga	2017	0.100	0.126	0.155
Democratic Republic of the Congo	Businga	2000-2017	-0.080	-0.048	-0.017
Democratic Republic of the Congo	Buta	2000	0.202	0.263	0.322
Democratic Republic of the Congo	Buta	2017	0.107	0.136	0.169
Democratic Republic of the Congo	Buta	2000-2017	-0.049	-0.019	0.009
Democratic Republic of the Congo	Buta (ville)	2000	0.221	0.291	0.370
Democratic Republic of the Congo	Buta (ville)	2017	0.100	0.133	0.174
Democratic Republic of the Congo	Buta (ville)	2000-2017	-0.071	-0.035	-0.001
Democratic Republic of the Congo	Butembo	2000	0.178	0.235	0.297
Democratic Republic of the Congo	Butembo	2017	0.088	0.113	0.146
Democratic Republic of the Congo	Butembo	2000-2017	-0.066	-0.030	0.004
Democratic Republic of the Congo	Dekese	2000	0.263	0.340	0.423
Democratic Republic of the Congo	Dekese	2017	0.137	0.169	0.207
Democratic Republic of the Congo	Dekese	2000-2017	-0.058	-0.029	-0.001
Democratic Republic of the Congo	Demba	2000	0.310	0.383	0.478
Democratic Republic of the Congo	Demba	2017	0.122	0.153	0.186
Democratic Republic of the Congo	Demba	2000-2017	-0.079	-0.050	-0.019

Table 2: Diarrhea DALYs rate by unit *(continued)*

Country	Unit	year	mean rate	lower rate	upper rate
Democratic Republic of the Congo	Dibaya	2000	0.313	0.386	0.465
Democratic Republic of the Congo	Dibaya	2017	0.130	0.160	0.195
Democratic Republic of the Congo	Dibaya	2000-2017	-0.075	-0.045	-0.015
Democratic Republic of the Congo	Dibaya-Lubwe	2000	0.177	0.232	0.295
Democratic Republic of the Congo	Dibaya-Lubwe	2017	0.087	0.111	0.145
Democratic Republic of the Congo	Dibaya-Lubwe	2000-2017	-0.067	-0.031	0.007
Democratic Republic of the Congo	Dilolo	2000	0.249	0.308	0.371
Democratic Republic of the Congo	Dilolo	2017	0.124	0.153	0.185
Democratic Republic of the Congo	Dilolo	2000-2017	-0.072	-0.045	-0.015
Democratic Republic of the Congo	Dimbelenge	2000	0.302	0.371	0.443
Democratic Republic of the Congo	Dimbelenge	2017	0.127	0.156	0.194
Democratic Republic of the Congo	Dimbelenge	2000-2017	-0.073	-0.044	-0.015
Democratic Republic of the Congo	Dingila	2000	0.200	0.268	0.338
Democratic Republic of the Congo	Dingila	2017	0.107	0.136	0.178
Democratic Republic of the Congo	Dingila	2000-2017	-0.035	-0.005	0.029
Democratic Republic of the Congo	Djolu	2000	0.220	0.285	0.351
Democratic Republic of the Congo	Djolu	2017	0.107	0.131	0.159
Democratic Republic of the Congo	Djolu	2000-2017	-0.060	-0.029	0.000
Democratic Republic of the Congo	Djugu	2000	0.226	0.279	0.340
Democratic Republic of the Congo	Djugu	2017	0.091	0.111	0.135
Democratic Republic of the Congo	Djugu	2000-2017	-0.080	-0.047	-0.016
Democratic Republic of the Congo	Dungu	2000	0.242	0.291	0.344
Democratic Republic of the Congo	Dungu	2017	0.106	0.126	0.151
Democratic Republic of the Congo	Dungu	2000-2017	-0.054	-0.030	-0.003
Democratic Republic of the Congo	Dungu (ville)	2000	0.238	0.301	0.390
Democratic Republic of the Congo	Dungu (ville)	2017	0.083	0.110	0.142
Democratic Republic of the Congo	Dungu (ville)	2000-2017	-0.076	-0.041	-0.006
Democratic Republic of the Congo	Faradje	2000	0.227	0.277	0.340
Democratic Republic of the Congo	Faradje	2017	0.102	0.126	0.153
Democratic Republic of the Congo	Faradje	2000-2017	-0.059	-0.031	0.003
Democratic Republic of the Congo	Feshi	2000	0.209	0.261	0.315
Democratic Republic of the Congo	Feshi	2017	0.088	0.109	0.133
Democratic Republic of the Congo	Feshi	2000-2017	-0.072	-0.044	-0.009
Democratic Republic of the Congo	Fizi	2000	0.233	0.283	0.341
Democratic Republic of the Congo	Fizi	2017	0.109	0.132	0.165
Democratic Republic of the Congo	Fizi	2000-2017	-0.068	-0.041	-0.013
Democratic Republic of the Congo	Gbadolite	2000	0.243	0.297	0.362
Democratic Republic of the Congo	Gbadolite	2017	0.103	0.135	0.170
Democratic Republic of the Congo	Gbadolite	2000-2017	-0.080	-0.047	-0.010
Democratic Republic of the Congo	Gemena	2000	0.242	0.297	0.362
Democratic Republic of the Congo	Gemena	2017	0.111	0.136	0.167
Democratic Republic of the Congo	Gemena	2000-2017	-0.074	-0.047	-0.016
Democratic Republic of the Congo	Gemena (ville)	2000	0.242	0.313	0.400
Democratic Republic of the Congo	Gemena (ville)	2017	0.104	0.135	0.175
Democratic Republic of the Congo	Gemena (ville)	2000-2017	-0.093	-0.060	-0.022
Democratic Republic of the Congo	Goma	2000	0.255	0.302	0.358
Democratic Republic of the Congo	Goma	2017	0.104	0.130	0.157
Democratic Republic of the Congo	Goma	2000-2017	-0.074	-0.042	-0.012
Democratic Republic of the Congo	Gungu	2000	0.219	0.262	0.323
Democratic Republic of the Congo	Gungu	2017	0.090	0.111	0.137
Democratic Republic of the Congo	Gungu	2000-2017	-0.061	-0.035	-0.006
Democratic Republic of the Congo	Gungu (ville)	2000	0.208	0.254	0.322
Democratic Republic of the Congo	Gungu (ville)	2017	0.086	0.110	0.140
Democratic Republic of the Congo	Gungu (ville)	2000-2017	-0.061	-0.030	0.003
Democratic Republic of the Congo	Idiofa	2000	0.206	0.248	0.299
Democratic Republic of the Congo	Idiofa	2017	0.096	0.116	0.144
Democratic Republic of the Congo	Idiofa	2000-2017	-0.060	-0.034	-0.004
Democratic Republic of the Congo	Idiofa (ville)	2000	0.192	0.236	0.293
Democratic Republic of the Congo	Idiofa (ville)	2017	0.086	0.109	0.138
Democratic Republic of the Congo	Idiofa (ville)	2000-2017	-0.065	-0.031	0.001
Democratic Republic of the Congo	Idjwi	2000	0.270	0.317	0.364

Table 2: Diarrhea DALYs rate by unit *(continued)*

Country	Unit	year	mean rate	lower rate	upper rate
Democratic Republic of the Congo	Idjwi	2017	0.107	0.132	0.156
Democratic Republic of the Congo	Idjwi	2000-2017	-0.061	-0.034	-0.005
Democratic Republic of the Congo	Ikela	2000	0.226	0.278	0.337
Democratic Republic of the Congo	Ikela	2017	0.111	0.139	0.178
Democratic Republic of the Congo	Ikela	2000-2017	-0.060	-0.032	0.001
Democratic Republic of the Congo	Ilebo	2000	0.283	0.341	0.415
Democratic Republic of the Congo	Ilebo	2017	0.131	0.158	0.194
Democratic Republic of the Congo	Ilebo	2000-2017	-0.061	-0.038	-0.012
Democratic Republic of the Congo	Ilebo (ville)	2000	0.267	0.333	0.426
Democratic Republic of the Congo	Ilebo (ville)	2017	0.127	0.167	0.222
Democratic Republic of the Congo	Ilebo (ville)	2000-2017	-0.066	-0.032	0.001
Democratic Republic of the Congo	Ingbokolo	2000	0.234	0.290	0.358
Democratic Republic of the Congo	Ingbokolo	2017	0.084	0.107	0.128
Democratic Republic of the Congo	Ingbokolo	2000-2017	-0.090	-0.053	-0.024
Democratic Republic of the Congo	Ingende	2000	0.216	0.268	0.328
Democratic Republic of the Congo	Ingende	2017	0.095	0.117	0.144
Democratic Republic of the Congo	Ingende	2000-2017	-0.063	-0.034	-0.009
Democratic Republic of the Congo	Inkisi	2000	0.202	0.251	0.316
Democratic Republic of the Congo	Inkisi	2017	0.087	0.113	0.141
Democratic Republic of the Congo	Inkisi	2000-2017	-0.080	-0.045	-0.012
Democratic Republic of the Congo	Inongo	2000	0.216	0.268	0.333
Democratic Republic of the Congo	Inongo	2017	0.097	0.120	0.149
Democratic Republic of the Congo	Inongo	2000-2017	-0.063	-0.033	-0.002
Democratic Republic of the Congo	Inongo (ville)	2000	0.195	0.250	0.326
Democratic Republic of the Congo	Inongo (ville)	2017	0.093	0.120	0.153
Democratic Republic of the Congo	Inongo (ville)	2000-2017	-0.063	-0.030	0.008
Democratic Republic of the Congo	Irumu	2000	0.217	0.270	0.331
Democratic Republic of the Congo	Irumu	2017	0.092	0.112	0.143
Democratic Republic of the Congo	Irumu	2000-2017	-0.075	-0.046	-0.019
Democratic Republic of the Congo	Isangi	2000	0.191	0.249	0.307
Democratic Republic of the Congo	Isangi	2017	0.098	0.127	0.159
Democratic Republic of the Congo	Isangi	2000-2017	-0.058	-0.024	0.009
Democratic Republic of the Congo	Isangi (ville)	2000	0.175	0.243	0.308
Democratic Republic of the Congo	Isangi (ville)	2017	0.088	0.119	0.155
Democratic Republic of the Congo	Isangi (ville)	2000-2017	-0.043	-0.005	0.032
Democratic Republic of the Congo	Isiro	2000	0.214	0.280	0.357
Democratic Republic of the Congo	Isiro	2017	0.093	0.122	0.153
Democratic Republic of the Congo	Isiro	2000-2017	-0.072	-0.038	-0.007
Democratic Republic of the Congo	Kabalo	2000	0.254	0.319	0.383
Democratic Republic of the Congo	Kabalo	2017	0.125	0.155	0.188
Democratic Republic of the Congo	Kabalo	2000-2017	-0.063	-0.033	0.000
Democratic Republic of the Congo	Kabambare	2000	0.250	0.308	0.376
Democratic Republic of the Congo	Kabambare	2017	0.111	0.134	0.165
Democratic Republic of the Congo	Kabambare	2000-2017	-0.066	-0.040	-0.011
Democratic Republic of the Congo	Kabare	2000	0.246	0.294	0.341
Democratic Republic of the Congo	Kabare	2017	0.105	0.129	0.156
Democratic Republic of the Congo	Kabare	2000-2017	-0.086	-0.051	-0.023
Democratic Republic of the Congo	Kabeya-Kamwanga	2000	0.275	0.349	0.423
Democratic Republic of the Congo	Kabeya-Kamwanga	2017	0.116	0.143	0.174
Democratic Republic of the Congo	Kabeya-Kamwanga	2000-2017	-0.068	-0.036	-0.003
Democratic Republic of the Congo	Kabinda	2000	0.278	0.339	0.416
Democratic Republic of the Congo	Kabinda	2017	0.124	0.151	0.183
Democratic Republic of the Congo	Kabinda	2000-2017	-0.070	-0.043	-0.014
Democratic Republic of the Congo	Kabinda (ville)	2000	0.293	0.376	0.474
Democratic Republic of the Congo	Kabinda (ville)	2017	0.113	0.144	0.182
Democratic Republic of the Congo	Kabinda (ville)	2000-2017	-0.103	-0.070	-0.029
Democratic Republic of the Congo	Kabongo	2000	0.234	0.299	0.367
Democratic Republic of the Congo	Kabongo	2017	0.118	0.147	0.178
Democratic Republic of the Congo	Kabongo	2000-2017	-0.066	-0.036	-0.007
Democratic Republic of the Congo	Kahemba	2000	0.201	0.250	0.309
Democratic Republic of the Congo	Kahemba	2017	0.089	0.111	0.139

Table 2: Diarrhea DALYs rate by unit *(continued)*

Country	Unit	year	mean rate	lower rate	upper rate
Democratic Republic of the Congo	Kahemba	2000-2017	-0.073	-0.041	-0.013
Democratic Republic of the Congo	Kahemba (ville)	2000	0.185	0.241	0.320
Democratic Republic of the Congo	Kahemba (ville)	2017	0.083	0.107	0.141
Democratic Republic of the Congo	Kahemba (ville)	2000-2017	-0.075	-0.040	-0.004
Democratic Republic of the Congo	Kailo	2000	0.219	0.274	0.341
Democratic Republic of the Congo	Kailo	2017	0.107	0.133	0.163
Democratic Republic of the Congo	Kailo	2000-2017	-0.069	-0.037	-0.006
Democratic Republic of the Congo	Kalehe	2000	0.235	0.282	0.334
Democratic Republic of the Congo	Kalehe	2017	0.103	0.128	0.150
Democratic Republic of the Congo	Kalehe	2000-2017	-0.065	-0.039	-0.011
Democratic Republic of the Congo	Kalemie	2000	0.252	0.311	0.384
Democratic Republic of the Congo	Kalemie	2017	0.117	0.151	0.183
Democratic Republic of the Congo	Kalemie	2000-2017	-0.063	-0.036	-0.003
Democratic Republic of the Congo	Kalemie (ville)	2000	0.253	0.325	0.416
Democratic Republic of the Congo	Kalemie (ville)	2017	0.107	0.142	0.179
Democratic Republic of the Congo	Kalemie (ville)	2000-2017	-0.072	-0.038	-0.002
Democratic Republic of the Congo	Kalima	2000	0.198	0.254	0.321
Democratic Republic of the Congo	Kalima	2017	0.091	0.120	0.154
Democratic Republic of the Congo	Kalima	2000-2017	-0.071	-0.038	-0.001
Democratic Republic of the Congo	Kambove	2000	0.254	0.319	0.396
Democratic Republic of the Congo	Kambove	2017	0.106	0.132	0.158
Democratic Republic of the Congo	Kambove	2000-2017	-0.079	-0.051	-0.021
Democratic Republic of the Congo	Kamiji	2000	0.280	0.347	0.432
Democratic Republic of the Congo	Kamiji	2017	0.125	0.157	0.192
Democratic Republic of the Congo	Kamiji	2000-2017	-0.066	-0.037	-0.006
Democratic Republic of the Congo	Kamina	2000	0.251	0.302	0.360
Democratic Republic of the Congo	Kamina	2017	0.104	0.129	0.152
Democratic Republic of the Congo	Kamina	2000-2017	-0.071	-0.047	-0.020
Democratic Republic of the Congo	Kamina (ville)	2000	0.206	0.277	0.350
Democratic Republic of the Congo	Kamina (ville)	2017	0.091	0.118	0.148
Democratic Republic of the Congo	Kamina (ville)	2000-2017	-0.080	-0.047	-0.011
Democratic Republic of the Congo	Kamituga	2000	0.257	0.326	0.399
Democratic Republic of the Congo	Kamituga	2017	0.092	0.121	0.152
Democratic Republic of the Congo	Kamituga	2000-2017	-0.099	-0.064	-0.025
Democratic Republic of the Congo	Kamonia	2000	0.306	0.366	0.439
Democratic Republic of the Congo	Kamonia	2017	0.142	0.173	0.205
Democratic Republic of the Congo	Kamonia	2000-2017	-0.062	-0.035	-0.011
Democratic Republic of the Congo	Kananga	2000	0.333	0.423	0.529
Democratic Republic of the Congo	Kananga	2017	0.129	0.164	0.204
Democratic Republic of the Congo	Kananga	2000-2017	-0.087	-0.056	-0.023
Democratic Republic of the Congo	Kaniama	2000	0.256	0.314	0.389
Democratic Republic of the Congo	Kaniama	2017	0.119	0.144	0.180
Democratic Republic of the Congo	Kaniama	2000-2017	-0.068	-0.040	-0.012
Democratic Republic of the Congo	Kaoze	2000	0.257	0.330	0.421
Democratic Republic of the Congo	Kaoze	2017	0.125	0.160	0.203
Democratic Republic of the Congo	Kaoze	2000-2017	-0.064	-0.033	-0.001
Democratic Republic of the Congo	Kapanga	2000	0.252	0.308	0.374
Democratic Republic of the Congo	Kapanga	2017	0.110	0.137	0.167
Democratic Republic of the Congo	Kapanga	2000-2017	-0.076	-0.046	-0.014
Democratic Republic of the Congo	Kasaji	2000	0.256	0.324	0.396
Democratic Republic of the Congo	Kasaji	2017	0.126	0.159	0.204
Democratic Republic of the Congo	Kasaji	2000-2017	-0.074	-0.044	-0.010
Democratic Republic of the Congo	Kasangulu	2000	0.223	0.272	0.328
Democratic Republic of the Congo	Kasangulu	2017	0.095	0.118	0.143
Democratic Republic of the Congo	Kasangulu	2000-2017	-0.074	-0.044	-0.014
Democratic Republic of the Congo	Kasangulu (ville)	2000	0.213	0.264	0.323
Democratic Republic of the Congo	Kasangulu (ville)	2017	0.094	0.118	0.144
Democratic Republic of the Congo	Kasangulu (ville)	2000-2017	-0.069	-0.041	-0.010
Democratic Republic of the Congo	Kasenga	2000	0.261	0.316	0.375
Democratic Republic of the Congo	Kasenga	2017	0.103	0.129	0.156
Democratic Republic of the Congo	Kasenga	2000-2017	-0.077	-0.050	-0.022

Table 2: Diarrhea DALYs rate by unit *(continued)*

Country	Unit	year	mean rate	lower rate	upper rate
Democratic Republic of the Congo	Kasongo	2000	0.246	0.297	0.356
Democratic Republic of the Congo	Kasongo	2017	0.103	0.127	0.157
Democratic Republic of the Congo	Kasongo	2000-2017	-0.063	-0.036	-0.010
Democratic Republic of the Congo	Kasongo (ville)	2000	0.241	0.297	0.364
Democratic Republic of the Congo	Kasongo (ville)	2017	0.098	0.127	0.165
Democratic Republic of the Congo	Kasongo (ville)	2000-2017	-0.064	-0.031	0.003
Democratic Republic of the Congo	Kasongo-Lunda	2000	0.203	0.251	0.304
Democratic Republic of the Congo	Kasongo-Lunda	2017	0.097	0.118	0.143
Democratic Republic of the Congo	Kasongo-Lunda	2000-2017	-0.067	-0.041	-0.010
Democratic Republic of the Congo	Kasongo-Lunda (ville)	2000	0.171	0.220	0.288
Democratic Republic of the Congo	Kasongo-Lunda (ville)	2017	0.100	0.128	0.159
Democratic Republic of the Congo	Kasongo-Lunda (ville)	2000-2017	-0.066	-0.031	0.007
Democratic Republic of the Congo	Katako-Kombe	2000	0.239	0.298	0.365
Democratic Republic of the Congo	Katako-Kombe	2017	0.119	0.150	0.189
Democratic Republic of the Congo	Katako-Kombe	2000-2017	-0.063	-0.035	-0.003
Democratic Republic of the Congo	Katanda	2000	0.280	0.346	0.423
Democratic Republic of the Congo	Katanda	2017	0.116	0.145	0.180
Democratic Republic of the Congo	Katanda	2000-2017	-0.075	-0.042	-0.011
Democratic Republic of the Congo	Kazumba	2000	0.311	0.385	0.467
Democratic Republic of the Congo	Kazumba	2017	0.146	0.179	0.215
Democratic Republic of the Congo	Kazumba	2000-2017	-0.066	-0.038	-0.010
Democratic Republic of the Congo	Kenge	2000	0.207	0.261	0.320
Democratic Republic of the Congo	Kenge	2017	0.092	0.112	0.137
Democratic Republic of the Congo	Kenge	2000-2017	-0.068	-0.042	-0.011
Democratic Republic of the Congo	Kenge (ville)	2000	0.171	0.227	0.292
Democratic Republic of the Congo	Kenge (ville)	2017	0.079	0.102	0.129
Democratic Republic of the Congo	Kenge (ville)	2000-2017	-0.071	-0.037	-0.001
Democratic Republic of the Congo	Kibombo	2000	0.248	0.298	0.351
Democratic Republic of the Congo	Kibombo	2017	0.110	0.138	0.171
Democratic Republic of the Congo	Kibombo	2000-2017	-0.069	-0.042	-0.017
Democratic Republic of the Congo	Kikwit	2000	0.211	0.258	0.315
Democratic Republic of the Congo	Kikwit	2017	0.088	0.112	0.141
Democratic Republic of the Congo	Kikwit	2000-2017	-0.069	-0.040	-0.005
Democratic Republic of the Congo	Kimvula	2000	0.182	0.225	0.286
Democratic Republic of the Congo	Kimvula	2017	0.101	0.128	0.162
Democratic Republic of the Congo	Kimvula	2000-2017	-0.055	-0.022	0.008
Democratic Republic of the Congo	Kindu	2000	0.213	0.271	0.352
Democratic Republic of the Congo	Kindu	2017	0.098	0.126	0.160
Democratic Republic of the Congo	Kindu	2000-2017	-0.082	-0.041	-0.005
Democratic Republic of the Congo	Kinshasa	2000	0.244	0.297	0.352
Democratic Republic of the Congo	Kinshasa	2017	0.114	0.139	0.168
Democratic Republic of the Congo	Kinshasa	2000-2017	-0.067	-0.038	-0.008
Democratic Republic of the Congo	Kipushi	2000	0.255	0.316	0.397
Democratic Republic of the Congo	Kipushi	2017	0.100	0.126	0.162
Democratic Republic of the Congo	Kipushi	2000-2017	-0.082	-0.049	-0.012
Democratic Republic of the Congo	Kipushi (ville)	2000	0.212	0.272	0.355
Democratic Republic of the Congo	Kipushi (ville)	2017	0.092	0.120	0.157
Democratic Republic of the Congo	Kipushi (ville)	2000-2017	-0.079	-0.042	0.000
Democratic Republic of the Congo	Kiri	2000	0.206	0.257	0.314
Democratic Republic of the Congo	Kiri	2017	0.097	0.123	0.154
Democratic Republic of the Congo	Kiri	2000-2017	-0.055	-0.026	0.006
Democratic Republic of the Congo	Kisangani	2000	0.197	0.260	0.339
Democratic Republic of the Congo	Kisangani	2017	0.078	0.104	0.138
Democratic Republic of the Congo	Kisangani	2000-2017	-0.080	-0.040	0.000
Democratic Republic of the Congo	Kole	2000	0.277	0.337	0.424
Democratic Republic of the Congo	Kole	2017	0.130	0.166	0.208
Democratic Republic of the Congo	Kole	2000-2017	-0.052	-0.023	0.008
Democratic Republic of the Congo	Kolwezi	2000	0.228	0.297	0.368
Democratic Republic of the Congo	Kolwezi	2017	0.096	0.123	0.155
Democratic Republic of the Congo	Kolwezi	2000-2017	-0.097	-0.058	-0.017
Democratic Republic of the Congo	Kongolo	2000	0.253	0.318	0.393

Table 2: Diarrhea DALYs rate by unit *(continued)*

Country	Unit	year	mean rate	lower rate	upper rate
Democratic Republic of the Congo	Kongolo	2017	0.115	0.143	0.176
Democratic Republic of the Congo	Kongolo	2000-2017	-0.074	-0.044	-0.015
Democratic Republic of the Congo	Kongolo (ville)	2000	0.257	0.330	0.416
Democratic Republic of the Congo	Kongolo (ville)	2017	0.109	0.139	0.174
Democratic Republic of the Congo	Kongolo (ville)	2000-2017	-0.086	-0.049	-0.016
Democratic Republic of the Congo	Kungu	2000	0.236	0.293	0.354
Democratic Republic of the Congo	Kungu	2017	0.105	0.129	0.160
Democratic Republic of the Congo	Kungu	2000-2017	-0.066	-0.039	-0.007
Democratic Republic of the Congo	Kutu	2000	0.202	0.250	0.317
Democratic Republic of the Congo	Kutu	2017	0.091	0.111	0.138
Democratic Republic of the Congo	Kutu	2000-2017	-0.066	-0.038	-0.007
Democratic Republic of the Congo	Kwamouth	2000	0.206	0.254	0.324
Democratic Republic of the Congo	Kwamouth	2017	0.092	0.113	0.136
Democratic Republic of the Congo	Kwamouth	2000-2017	-0.065	-0.035	-0.005
Democratic Republic of the Congo	Libenge	2000	0.241	0.286	0.335
Democratic Republic of the Congo	Libenge	2017	0.114	0.138	0.167
Democratic Republic of the Congo	Libenge	2000-2017	-0.069	-0.042	-0.013
Democratic Republic of the Congo	Likasi	2000	0.221	0.284	0.358
Democratic Republic of the Congo	Likasi	2017	0.094	0.121	0.147
Democratic Republic of the Congo	Likasi	2000-2017	-0.082	-0.049	-0.019
Democratic Republic of the Congo	Lisala	2000	0.240	0.290	0.351
Democratic Republic of the Congo	Lisala	2017	0.100	0.125	0.154
Democratic Republic of the Congo	Lisala	2000-2017	-0.060	-0.032	-0.003
Democratic Republic of the Congo	Lisala (ville)	2000	0.200	0.266	0.333
Democratic Republic of the Congo	Lisala (ville)	2017	0.088	0.115	0.152
Democratic Republic of the Congo	Lisala (ville)	2000-2017	-0.067	-0.034	0.001
Democratic Republic of the Congo	Lodja	2000	0.251	0.320	0.425
Democratic Republic of the Congo	Lodja	2017	0.126	0.164	0.208
Democratic Republic of the Congo	Lodja	2000-2017	-0.040	-0.010	0.022
Democratic Republic of the Congo	Lodja (ville)	2000	NA	NA	NA
Democratic Republic of the Congo	Lodja (ville)	2017	NA	NA	NA
Democratic Republic of the Congo	Lodja (ville)	2000-2017	NA	NA	NA
Democratic Republic of the Congo	Lomela	2000	0.241	0.293	0.353
Democratic Republic of the Congo	Lomela	2017	0.110	0.136	0.163
Democratic Republic of the Congo	Lomela	2000-2017	-0.064	-0.040	-0.014
Democratic Republic of the Congo	Lubao	2000	0.262	0.326	0.405
Democratic Republic of the Congo	Lubao	2017	0.118	0.143	0.175
Democratic Republic of the Congo	Lubao	2000-2017	-0.070	-0.042	-0.012
Democratic Republic of the Congo	Lubao (ville)	2000	0.257	0.337	0.453
Democratic Republic of the Congo	Lubao (ville)	2017	0.106	0.134	0.177
Democratic Republic of the Congo	Lubao (ville)	2000-2017	-0.085	-0.052	-0.015
Democratic Republic of the Congo	Lubefu	2000	0.256	0.318	0.394
Democratic Republic of the Congo	Lubefu	2017	0.119	0.146	0.181
Democratic Republic of the Congo	Lubefu	2000-2017	-0.070	-0.043	-0.012
Democratic Republic of the Congo	Lubero	2000	0.196	0.243	0.296
Democratic Republic of the Congo	Lubero	2017	0.096	0.117	0.145
Democratic Republic of the Congo	Lubero	2000-2017	-0.064	-0.033	-0.003
Democratic Republic of the Congo	Lubudi	2000	0.263	0.323	0.397
Democratic Republic of the Congo	Lubudi	2017	0.105	0.129	0.156
Democratic Republic of the Congo	Lubudi	2000-2017	-0.089	-0.055	-0.027
Democratic Republic of the Congo	Lubumbashi	2000	0.232	0.290	0.374
Democratic Republic of the Congo	Lubumbashi	2017	0.090	0.116	0.153
Democratic Republic of the Congo	Lubumbashi	2000-2017	-0.085	-0.050	-0.008
Democratic Republic of the Congo	Lubutu	2000	0.204	0.261	0.330
Democratic Republic of the Congo	Lubutu	2017	0.092	0.118	0.146
Democratic Republic of the Congo	Lubutu	2000-2017	-0.062	-0.031	0.000
Democratic Republic of the Congo	Lubutu (ville)	2000	0.194	0.261	0.336
Democratic Republic of the Congo	Lubutu (ville)	2017	0.083	0.110	0.138
Democratic Republic of the Congo	Lubutu (ville)	2000-2017	-0.081	-0.045	-0.004
Democratic Republic of the Congo	Luebo	2000	0.298	0.363	0.457
Democratic Republic of the Congo	Luebo	2017	0.128	0.160	0.196

Table 2: Diarrhea DALYs rate by unit *(continued)*

Country	Unit	year	mean rate	lower rate	upper rate
Democratic Republic of the Congo	Luebo	2000-2017	-0.067	-0.038	-0.006
Democratic Republic of the Congo	Luebo (ville)	2000	0.280	0.363	0.455
Democratic Republic of the Congo	Luebo (ville)	2017	0.118	0.153	0.194
Democratic Republic of the Congo	Luebo (ville)	2000-2017	-0.082	-0.051	-0.012
Democratic Republic of the Congo	Luilu	2000	0.265	0.323	0.382
Democratic Republic of the Congo	Luilu	2017	0.118	0.143	0.181
Democratic Republic of the Congo	Luilu	2000-2017	-0.071	-0.041	-0.012
Democratic Republic of the Congo	Luiza	2000	0.284	0.360	0.455
Democratic Republic of the Congo	Luiza	2017	0.136	0.169	0.210
Democratic Republic of the Congo	Luiza	2000-2017	-0.069	-0.040	-0.010
Democratic Republic of the Congo	Lukalaba	2000	0.279	0.350	0.439
Democratic Republic of the Congo	Lukalaba	2017	0.127	0.159	0.198
Democratic Republic of the Congo	Lukalaba	2000-2017	-0.060	-0.031	0.001
Democratic Republic of the Congo	Lukolela	2000	0.214	0.275	0.355
Democratic Republic of the Congo	Lukolela	2017	0.095	0.116	0.141
Democratic Republic of the Congo	Lukolela	2000-2017	-0.074	-0.040	-0.004
Democratic Republic of the Congo	Lukula	2000	0.191	0.239	0.296
Democratic Republic of the Congo	Lukula	2017	0.082	0.103	0.129
Democratic Republic of the Congo	Lukula	2000-2017	-0.066	-0.034	-0.005
Democratic Republic of the Congo	Lukula (ville)	2000	0.179	0.230	0.287
Democratic Republic of the Congo	Lukula (ville)	2017	0.077	0.100	0.127
Democratic Republic of the Congo	Lukula (ville)	2000-2017	-0.064	-0.034	-0.002
Democratic Republic of the Congo	Luozi	2000	0.204	0.253	0.314
Democratic Republic of the Congo	Luozi	2017	0.087	0.110	0.135
Democratic Republic of the Congo	Luozi	2000-2017	-0.065	-0.036	-0.007
Democratic Republic of the Congo	Lupatapata	2000	0.278	0.348	0.424
Democratic Republic of the Congo	Lupatapata	2017	0.118	0.147	0.182
Democratic Republic of the Congo	Lupatapata	2000-2017	-0.069	-0.039	-0.006
Democratic Republic of the Congo	Lusambo	2000	0.259	0.327	0.411
Democratic Republic of the Congo	Lusambo	2017	0.130	0.155	0.195
Democratic Republic of the Congo	Lusambo	2000-2017	-0.069	-0.041	-0.012
Democratic Republic of the Congo	Lusambo (ville)	2000	0.252	0.323	0.416
Democratic Republic of the Congo	Lusambo (ville)	2017	0.125	0.153	0.193
Democratic Republic of the Congo	Lusambo (ville)	2000-2017	-0.080	-0.048	-0.017
Democratic Republic of the Congo	Madimba	2000	0.200	0.244	0.299
Democratic Republic of the Congo	Madimba	2017	0.090	0.114	0.137
Democratic Republic of the Congo	Madimba	2000-2017	-0.068	-0.034	-0.006
Democratic Republic of the Congo	Mahagi	2000	0.237	0.293	0.366
Democratic Republic of the Congo	Mahagi	2017	0.089	0.113	0.136
Democratic Republic of the Congo	Mahagi	2000-2017	-0.090	-0.055	-0.023
Democratic Republic of the Congo	Mahagi (ville)	2000	0.222	0.278	0.345
Democratic Republic of the Congo	Mahagi (ville)	2017	0.086	0.110	0.134
Democratic Republic of the Congo	Mahagi (ville)	2000-2017	-0.089	-0.054	-0.020
Democratic Republic of the Congo	Makanza	2000	0.229	0.294	0.361
Democratic Republic of the Congo	Makanza	2017	0.099	0.124	0.156
Democratic Republic of the Congo	Makanza	2000-2017	-0.063	-0.033	-0.005
Democratic Republic of the Congo	Malemba-Nkulu	2000	0.235	0.295	0.358
Democratic Republic of the Congo	Malemba-Nkulu	2017	0.112	0.137	0.172
Democratic Republic of the Congo	Malemba-Nkulu	2000-2017	-0.063	-0.035	-0.008
Democratic Republic of the Congo	Mambasa	2000	0.214	0.260	0.309
Democratic Republic of the Congo	Mambasa	2017	0.093	0.114	0.142
Democratic Republic of the Congo	Mambasa	2000-2017	-0.069	-0.040	-0.015
Democratic Republic of the Congo	Mangai	2000	0.171	0.222	0.286
Democratic Republic of the Congo	Mangai	2017	0.082	0.107	0.144
Democratic Republic of the Congo	Mangai	2000-2017	-0.070	-0.032	0.003
Democratic Republic of the Congo	Manono	2000	0.270	0.317	0.377
Democratic Republic of the Congo	Manono	2017	0.126	0.155	0.188
Democratic Republic of the Congo	Manono	2000-2017	-0.060	-0.033	-0.011
Democratic Republic of the Congo	Manono (ville)	2000	0.264	0.331	0.408
Democratic Republic of the Congo	Manono (ville)	2017	0.121	0.160	0.209
Democratic Republic of the Congo	Manono (ville)	2000-2017	-0.081	-0.045	-0.012

Table 2: Diarrhea DALYs rate by unit *(continued)*

Country	Unit	year	mean rate	lower rate	upper rate
Democratic Republic of the Congo	Masi-Manimba	2000	0.217	0.271	0.338
Democratic Republic of the Congo	Masi-Manimba	2017	0.087	0.106	0.130
Democratic Republic of the Congo	Masi-Manimba	2000-2017	-0.081	-0.054	-0.021
Democratic Republic of the Congo	Masi-Manimba (ville)	2000	0.191	0.250	0.317
Democratic Republic of the Congo	Masi-Manimba (ville)	2017	0.081	0.103	0.131
Democratic Republic of the Congo	Masi-Manimba (ville)	2000-2017	-0.088	-0.055	-0.022
Democratic Republic of the Congo	Masisi	2000	0.240	0.281	0.333
Democratic Republic of the Congo	Masisi	2017	0.098	0.121	0.151
Democratic Republic of the Congo	Masisi	2000-2017	-0.080	-0.051	-0.019
Democratic Republic of the Congo	Matadi	2000	0.141	0.193	0.248
Democratic Republic of the Congo	Matadi	2017	0.073	0.096	0.123
Democratic Republic of the Congo	Matadi	2000-2017	-0.054	-0.018	0.018
Democratic Republic of the Congo	Mbandaka	2000	0.205	0.275	0.367
Democratic Republic of the Congo	Mbandaka	2017	0.079	0.103	0.132
Democratic Republic of the Congo	Mbandaka	2000-2017	-0.092	-0.054	-0.021
Democratic Republic of the Congo	Mbanza-Ngungu	2000	0.206	0.254	0.308
Democratic Republic of the Congo	Mbanza-Ngungu	2017	0.087	0.111	0.141
Democratic Republic of the Congo	Mbanza-Ngungu	2000-2017	-0.068	-0.041	-0.013
Democratic Republic of the Congo	Mbanza-Ngungu (ville)	2000	0.198	0.248	0.309
Democratic Republic of the Congo	Mbanza-Ngungu (ville)	2017	0.084	0.109	0.140
Democratic Republic of the Congo	Mbanza-Ngungu (ville)	2000-2017	-0.082	-0.050	-0.020
Democratic Republic of the Congo	Mbuji-Mayi	2000	0.278	0.348	0.426
Democratic Republic of the Congo	Mbuji-Mayi	2017	0.117	0.146	0.182
Democratic Republic of the Congo	Mbuji-Mayi	2000-2017	-0.071	-0.040	-0.006
Democratic Republic of the Congo	Miabi	2000	0.278	0.349	0.422
Democratic Republic of the Congo	Miabi	2017	0.124	0.153	0.191
Democratic Republic of the Congo	Miabi	2000-2017	-0.063	-0.033	-0.001
Democratic Republic of the Congo	Miabi (ville)	2000	0.279	0.351	0.437
Democratic Republic of the Congo	Miabi (ville)	2017	0.113	0.142	0.184
Democratic Republic of the Congo	Miabi (ville)	2000-2017	-0.073	-0.039	-0.003
Democratic Republic of the Congo	Mitwaba	2000	0.267	0.318	0.382
Democratic Republic of the Congo	Mitwaba	2017	0.115	0.141	0.174
Democratic Republic of the Congo	Mitwaba	2000-2017	-0.065	-0.036	-0.007
Democratic Republic of the Congo	Moanda	2000	0.199	0.254	0.316
Democratic Republic of the Congo	Moanda	2017	0.084	0.105	0.134
Democratic Republic of the Congo	Moanda	2000-2017	-0.064	-0.035	-0.006
Democratic Republic of the Congo	Moanda (ville)	2000	0.168	0.224	0.284
Democratic Republic of the Congo	Moanda (ville)	2017	0.075	0.098	0.126
Democratic Republic of the Congo	Moanda (ville)	2000-2017	-0.066	-0.033	0.000
Democratic Republic of the Congo	Moba	2000	0.264	0.322	0.406
Democratic Republic of the Congo	Moba	2017	0.128	0.160	0.200
Democratic Republic of the Congo	Moba	2000-2017	-0.059	-0.031	-0.003
Democratic Republic of the Congo	Mobayi-Mbongo	2000	0.255	0.306	0.357
Democratic Republic of the Congo	Mobayi-Mbongo	2017	0.107	0.138	0.168
Democratic Republic of the Congo	Mobayi-Mbongo	2000-2017	-0.075	-0.047	-0.017
Democratic Republic of the Congo	Mobayi-Mbongo (ville)	2000	0.274	0.336	0.398
Democratic Republic of the Congo	Mobayi-Mbongo (ville)	2017	0.117	0.154	0.194
Democratic Republic of the Congo	Mobayi-Mbongo (ville)	2000-2017	-0.079	-0.042	-0.004
Democratic Republic of the Congo	Mongwalu	2000	0.215	0.271	0.328
Democratic Republic of the Congo	Mongwalu	2017	0.090	0.111	0.138
Democratic Republic of the Congo	Mongwalu	2000-2017	-0.072	-0.038	-0.008
Democratic Republic of the Congo	Monkoto	2000	0.244	0.297	0.354
Democratic Republic of the Congo	Monkoto	2017	0.112	0.138	0.172
Democratic Republic of the Congo	Monkoto	2000-2017	-0.058	-0.029	0.003
Democratic Republic of the Congo	Mushie	2000	0.215	0.263	0.331
Democratic Republic of the Congo	Mushie	2017	0.094	0.116	0.142
Democratic Republic of the Congo	Mushie	2000-2017	-0.065	-0.033	-0.004
Democratic Republic of the Congo	Mutshatsha	2000	0.246	0.311	0.378
Democratic Republic of the Congo	Mutshatsha	2017	0.103	0.129	0.158
Democratic Republic of the Congo	Mutshatsha	2000-2017	-0.089	-0.056	-0.020
Democratic Republic of the Congo	Mweka	2000	0.287	0.351	0.427

Table 2: Diarrhea DALYs rate by unit *(continued)*

Country	Unit	year	mean rate	lower rate	upper rate
Democratic Republic of the Congo	Mweka	2017	0.127	0.160	0.196
Democratic Republic of the Congo	Mweka	2000-2017	-0.063	-0.039	-0.011
Democratic Republic of the Congo	Mwene-Ditu	2000	0.244	0.311	0.379
Democratic Republic of the Congo	Mwene-Ditu	2017	0.107	0.137	0.170
Democratic Republic of the Congo	Mwene-Ditu	2000-2017	-0.077	-0.048	-0.017
Democratic Republic of the Congo	Mwenga	2000	0.240	0.298	0.358
Democratic Republic of the Congo	Mwenga	2017	0.099	0.127	0.154
Democratic Republic of the Congo	Mwenga	2000-2017	-0.074	-0.043	-0.011
Democratic Republic of the Congo	Namoya	2000	0.245	0.318	0.394
Democratic Republic of the Congo	Namoya	2017	0.110	0.143	0.182
Democratic Republic of the Congo	Namoya	2000-2017	-0.078	-0.046	-0.011
Democratic Republic of the Congo	Ngandajika	2000	0.284	0.355	0.442
Democratic Republic of the Congo	Ngandajika	2017	0.132	0.162	0.201
Democratic Republic of the Congo	Ngandajika	2000-2017	-0.068	-0.040	-0.008
Democratic Republic of the Congo	Ngandajika (ville)	2000	0.285	0.372	0.466
Democratic Republic of the Congo	Ngandajika (ville)	2017	0.126	0.160	0.203
Democratic Republic of the Congo	Ngandajika (ville)	2000-2017	-0.075	-0.044	-0.013
Democratic Republic of the Congo	Niangara	2000	0.227	0.285	0.348
Democratic Republic of the Congo	Niangara	2017	0.094	0.120	0.146
Democratic Republic of the Congo	Niangara	2000-2017	-0.060	-0.033	-0.002
Democratic Republic of the Congo	Nioki	2000	0.177	0.230	0.305
Democratic Republic of the Congo	Nioki	2017	0.080	0.105	0.141
Democratic Republic of the Congo	Nioki	2000-2017	-0.075	-0.033	0.004
Democratic Republic of the Congo	Nyiragongo	2000	0.259	0.302	0.358
Democratic Republic of the Congo	Nyiragongo	2017	0.099	0.121	0.149
Democratic Republic of the Congo	Nyiragongo	2000-2017	-0.083	-0.051	-0.021
Democratic Republic of the Congo	Nyunzu	2000	0.260	0.326	0.395
Democratic Republic of the Congo	Nyunzu	2017	0.126	0.155	0.193
Democratic Republic of the Congo	Nyunzu	2000-2017	-0.070	-0.039	-0.007
Democratic Republic of the Congo	Oicha	2000	0.199	0.249	0.310
Democratic Republic of the Congo	Oicha	2017	0.096	0.118	0.146
Democratic Republic of the Congo	Oicha	2000-2017	-0.064	-0.036	-0.004
Democratic Republic of the Congo	Oïcha (ville)	2000	0.201	0.253	0.323
Democratic Republic of the Congo	Oïcha (ville)	2017	0.091	0.117	0.151
Democratic Republic of the Congo	Oïcha (ville)	2000-2017	-0.066	-0.034	0.002
Democratic Republic of the Congo	Opala	2000	0.209	0.265	0.318
Democratic Republic of the Congo	Opala	2017	0.106	0.136	0.168
Democratic Republic of the Congo	Opala	2000-2017	-0.059	-0.030	-0.001
Democratic Republic of the Congo	Oshwe	2000	0.227	0.270	0.322
Democratic Republic of the Congo	Oshwe	2017	0.106	0.127	0.156
Democratic Republic of the Congo	Oshwe	2000-2017	-0.052	-0.029	0.001
Democratic Republic of the Congo	Pangi	2000	0.219	0.274	0.333
Democratic Republic of the Congo	Pangi	2017	0.102	0.123	0.150
Democratic Republic of the Congo	Pangi	2000-2017	-0.066	-0.038	-0.013
Democratic Republic of the Congo	Poko	2000	0.211	0.269	0.339
Democratic Republic of the Congo	Poko	2017	0.111	0.137	0.173
Democratic Republic of the Congo	Poko	2000-2017	-0.051	-0.022	0.005
Democratic Republic of the Congo	Popokabaka	2000	0.191	0.236	0.291
Democratic Republic of the Congo	Popokabaka	2017	0.109	0.134	0.164
Democratic Republic of the Congo	Popokabaka	2000-2017	-0.055	-0.022	0.010
Democratic Republic of the Congo	Punia	2000	0.206	0.261	0.322
Democratic Republic of the Congo	Punia	2017	0.090	0.114	0.146
Democratic Republic of the Congo	Punia	2000-2017	-0.071	-0.044	-0.014
Democratic Republic of the Congo	Punia (ville)	2000	0.183	0.247	0.328
Democratic Republic of the Congo	Punia (ville)	2017	0.080	0.105	0.138
Democratic Republic of the Congo	Punia (ville)	2000-2017	-0.078	-0.045	-0.009
Democratic Republic of the Congo	Pweto	2000	0.256	0.310	0.368
Democratic Republic of the Congo	Pweto	2017	0.113	0.138	0.170
Democratic Republic of the Congo	Pweto	2000-2017	-0.066	-0.040	-0.011
Democratic Republic of the Congo	Rungu	2000	0.214	0.275	0.345
Democratic Republic of the Congo	Rungu	2017	0.103	0.131	0.162

Table 2: Diarrhea DALYs rate by unit *(continued)*

Country	Unit	year	mean rate	lower rate	upper rate
Democratic Republic of the Congo	Rungu	2000-2017	-0.059	-0.027	-0.001
Democratic Republic of the Congo	Rutshuru	2000	0.234	0.278	0.322
Democratic Republic of the Congo	Rutshuru	2017	0.100	0.121	0.149
Democratic Republic of the Congo	Rutshuru	2000-2017	-0.075	-0.042	-0.013
Democratic Republic of the Congo	Rutshuru (ville)	2000	0.236	0.281	0.334
Democratic Republic of the Congo	Rutshuru (ville)	2017	0.100	0.122	0.151
Democratic Republic of the Congo	Rutshuru (ville)	2000-2017	-0.081	-0.049	-0.019
Democratic Republic of the Congo	Sakania	2000	0.253	0.305	0.369
Democratic Republic of the Congo	Sakania	2017	0.112	0.137	0.168
Democratic Republic of the Congo	Sakania	2000-2017	-0.072	-0.040	-0.014
Democratic Republic of the Congo	Sandoa	2000	0.272	0.327	0.397
Democratic Republic of the Congo	Sandoa	2017	0.118	0.147	0.177
Democratic Republic of the Congo	Sandoa	2000-2017	-0.064	-0.039	-0.010
Democratic Republic of the Congo	Seke-Banza	2000	0.192	0.248	0.307
Democratic Republic of the Congo	Seke-Banza	2017	0.084	0.108	0.135
Democratic Republic of the Congo	Seke-Banza	2000-2017	-0.063	-0.030	0.004
Democratic Republic of the Congo	Shabunda	2000	0.245	0.294	0.346
Democratic Republic of the Congo	Shabunda	2017	0.103	0.125	0.150
Democratic Republic of the Congo	Shabunda	2000-2017	-0.067	-0.042	-0.015
Democratic Republic of the Congo	Shabunda (ville)	2000	0.235	0.293	0.366
Democratic Republic of the Congo	Shabunda (ville)	2017	0.085	0.113	0.142
Democratic Republic of the Congo	Shabunda (ville)	2000-2017	-0.070	-0.036	-0.003
Democratic Republic of the Congo	Songololo	2000	0.201	0.250	0.304
Democratic Republic of the Congo	Songololo	2017	0.084	0.108	0.132
Democratic Republic of the Congo	Songololo	2000-2017	-0.060	-0.035	-0.002
Democratic Republic of the Congo	Tshela	2000	0.183	0.232	0.288
Democratic Republic of the Congo	Tshela	2017	0.081	0.104	0.129
Democratic Republic of the Congo	Tshela	2000-2017	-0.065	-0.036	-0.006
Democratic Republic of the Congo	Tshela (ville)	2000	0.179	0.224	0.281
Democratic Republic of the Congo	Tshela (ville)	2017	0.078	0.102	0.128
Democratic Republic of the Congo	Tshela (ville)	2000-2017	-0.066	-0.035	-0.004
Democratic Republic of the Congo	Tshikapa	2000	0.314	0.394	0.493
Democratic Republic of the Congo	Tshikapa	2017	0.136	0.173	0.216
Democratic Republic of the Congo	Tshikapa	2000-2017	-0.075	-0.043	-0.014
Democratic Republic of the Congo	Tshilenge	2000	0.282	0.347	0.434
Democratic Republic of the Congo	Tshilenge	2017	0.126	0.154	0.192
Democratic Republic of the Congo	Tshilenge	2000-2017	-0.066	-0.035	-0.002
Democratic Republic of the Congo	Tshilenge (ville)	2000	0.286	0.356	0.441
Democratic Republic of the Congo	Tshilenge (ville)	2017	0.115	0.143	0.176
Democratic Republic of the Congo	Tshilenge (ville)	2000-2017	-0.076	-0.045	-0.013
Democratic Republic of the Congo	Tshimbulu	2000	0.312	0.392	0.493
Democratic Republic of the Congo	Tshimbulu	2017	0.121	0.153	0.189
Democratic Republic of the Congo	Tshimbulu	2000-2017	-0.084	-0.050	-0.014
Democratic Republic of the Congo	Ubundu	2000	0.195	0.249	0.305
Democratic Republic of the Congo	Ubundu	2017	0.089	0.114	0.143
Democratic Republic of the Congo	Ubundu	2000-2017	-0.063	-0.033	-0.004
Democratic Republic of the Congo	Uvira	2000	0.258	0.305	0.366
Democratic Republic of the Congo	Uvira	2017	0.122	0.149	0.182
Democratic Republic of the Congo	Uvira	2000-2017	-0.071	-0.040	-0.011
Democratic Republic of the Congo	Uvira (ville)	2000	0.255	0.310	0.379
Democratic Republic of the Congo	Uvira (ville)	2017	0.114	0.142	0.178
Democratic Republic of the Congo	Uvira (ville)	2000-2017	-0.078	-0.049	-0.021
Democratic Republic of the Congo	Walikale	2000	0.234	0.280	0.343
Democratic Republic of the Congo	Walikale	2017	0.100	0.123	0.150
Democratic Republic of the Congo	Walikale	2000-2017	-0.069	-0.043	-0.015
Democratic Republic of the Congo	Walungu	2000	0.256	0.302	0.356
Democratic Republic of the Congo	Walungu	2017	0.110	0.135	0.162
Democratic Republic of the Congo	Walungu	2000-2017	-0.078	-0.046	-0.015
Democratic Republic of the Congo	Wamba	2000	0.210	0.269	0.333
Democratic Republic of the Congo	Wamba	2017	0.100	0.131	0.167
Democratic Republic of the Congo	Wamba	2000-2017	-0.058	-0.028	0.002

Table 2: Diarrhea DALYs rate by unit *(continued)*

Country	Unit	year	mean rate	lower rate	upper rate
Democratic Republic of the Congo	Wamba (ville)	2000	0.229	0.287	0.363
Democratic Republic of the Congo	Wamba (ville)	2017	0.100	0.133	0.175
Democratic Republic of the Congo	Wamba (ville)	2000-2017	-0.076	-0.040	-0.006
Democratic Republic of the Congo	Watsa	2000	0.229	0.275	0.327
Democratic Republic of the Congo	Watsa	2017	0.090	0.114	0.139
Democratic Republic of the Congo	Watsa	2000-2017	-0.069	-0.041	-0.012
Democratic Republic of the Congo	Watsa (ville)	2000	0.210	0.262	0.323
Democratic Republic of the Congo	Watsa (ville)	2017	0.085	0.107	0.132
Democratic Republic of the Congo	Watsa (ville)	2000-2017	-0.074	-0.041	-0.007
Democratic Republic of the Congo	Yahuma	2000	0.210	0.263	0.323
Democratic Republic of the Congo	Yahuma	2017	0.107	0.131	0.159
Democratic Republic of the Congo	Yahuma	2000-2017	-0.060	-0.034	-0.008
Democratic Republic of the Congo	Yakoma	2000	0.233	0.292	0.354
Democratic Republic of the Congo	Yakoma	2017	0.096	0.122	0.150
Democratic Republic of the Congo	Yakoma	2000-2017	-0.080	-0.053	-0.024
Democratic Republic of the Congo	Yangambi	2000	0.176	0.248	0.324
Democratic Republic of the Congo	Yangambi	2017	0.091	0.126	0.165
Democratic Republic of the Congo	Yangambi	2000-2017	-0.060	-0.025	0.012
Democratic Republic of the Congo	Yumbi	2000	0.189	0.252	0.324
Democratic Republic of the Congo	Yumbi	2017	0.076	0.099	0.126
Democratic Republic of the Congo	Yumbi	2000-2017	-0.077	-0.038	-0.002
Democratic Republic of the Congo	Zongo	2000	0.228	0.264	0.319
Democratic Republic of the Congo	Zongo	2017	0.121	0.156	0.194
Democratic Republic of the Congo	Zongo	2000-2017	-0.068	-0.036	-0.005
Djibouti	Alaili Dadda	2000	0.192	0.307	0.462
Djibouti	Alaili Dadda	2017	0.044	0.071	0.119
Djibouti	Alaili Dadda	2000-2017	-0.112	-0.056	-0.005
Djibouti	Ali Sabieh	2000	0.188	0.294	0.447
Djibouti	Ali Sabieh	2017	0.034	0.056	0.091
Djibouti	Ali Sabieh	2000-2017	-0.126	-0.075	-0.021
Djibouti	As Eyla	2000	0.192	0.294	0.437
Djibouti	As Eyla	2017	0.033	0.057	0.091
Djibouti	As Eyla	2000-2017	-0.124	-0.073	-0.023
Djibouti	Balha	2000	0.174	0.291	0.432
Djibouti	Balha	2017	0.039	0.064	0.107
Djibouti	Balha	2000-2017	-0.117	-0.063	-0.013
Djibouti	Dikhil	2000	0.191	0.292	0.437
Djibouti	Dikhil	2017	0.032	0.056	0.091
Djibouti	Dikhil	2000-2017	-0.126	-0.077	-0.023
Djibouti	Djibouti	2000	0.183	0.285	0.423
Djibouti	Djibouti	2017	0.037	0.061	0.100
Djibouti	Djibouti	2000-2017	-0.119	-0.064	-0.009
Djibouti	Dorra	2000	0.175	0.293	0.433
Djibouti	Dorra	2017	0.041	0.068	0.114
Djibouti	Dorra	2000-2017	-0.111	-0.057	-0.006
Djibouti	Obock	2000	0.194	0.305	0.459
Djibouti	Obock	2017	0.041	0.067	0.116
Djibouti	Obock	2000-2017	-0.116	-0.061	-0.010
Djibouti	Randa	2000	0.176	0.292	0.429
Djibouti	Randa	2017	0.040	0.066	0.109
Djibouti	Randa	2000-2017	-0.114	-0.061	-0.012
Djibouti	Tadjourah	2000	0.180	0.293	0.433
Djibouti	Tadjourah	2017	0.039	0.064	0.107
Djibouti	Tadjourah	2000-2017	-0.116	-0.063	-0.015
Djibouti	Yoboki	2000	0.173	0.282	0.411
Djibouti	Yoboki	2017	0.035	0.060	0.095
Djibouti	Yoboki	2000-2017	-0.120	-0.067	-0.020
Equatorial Guinea	Aconibe	2000	0.266	0.332	0.403
Equatorial Guinea	Aconibe	2017	0.041	0.055	0.069
Equatorial Guinea	Aconibe	2000-2017	-0.181	-0.144	-0.109
Equatorial Guinea	Acurenam	2000	0.243	0.297	0.364

Table 2: Diarrhea DALYs rate by unit *(continued)*

Country	Unit	year	mean rate	lower rate	upper rate
Equatorial Guinea	Acurenam	2017	0.040	0.050	0.061
Equatorial Guinea	Acurenam	2000-2017	-0.159	-0.127	-0.095
Equatorial Guinea	Añisok	2000	0.253	0.318	0.378
Equatorial Guinea	Añisok	2017	0.040	0.051	0.064
Equatorial Guinea	Añisok	2000-2017	-0.166	-0.131	-0.102
Equatorial Guinea	Ayene	2000	0.266	0.328	0.394
Equatorial Guinea	Ayene	2017	0.042	0.054	0.067
Equatorial Guinea	Ayene	2000-2017	-0.175	-0.141	-0.110
Equatorial Guinea	Baney	2000	0.169	0.217	0.273
Equatorial Guinea	Baney	2017	0.027	0.036	0.046
Equatorial Guinea	Baney	2000-2017	-0.151	-0.121	-0.083
Equatorial Guinea	Bata	2000	0.248	0.299	0.360
Equatorial Guinea	Bata	2017	0.032	0.041	0.051
Equatorial Guinea	Bata	2000-2017	-0.187	-0.152	-0.116
Equatorial Guinea	Bicurga	2000	0.239	0.295	0.364
Equatorial Guinea	Bicurga	2017	0.038	0.049	0.061
Equatorial Guinea	Bicurga	2000-2017	-0.168	-0.133	-0.099
Equatorial Guinea	Bidjabidjan	2000	0.288	0.349	0.422
Equatorial Guinea	Bidjabidjan	2017	0.042	0.054	0.068
Equatorial Guinea	Bidjabidjan	2000-2017	-0.143	-0.113	-0.080
Equatorial Guinea	Bitica	2000	0.239	0.292	0.352
Equatorial Guinea	Bitica	2017	0.036	0.046	0.059
Equatorial Guinea	Bitica	2000-2017	-0.186	-0.150	-0.113
Equatorial Guinea	Ciudad Nueva Oyala	2000	0.244	0.304	0.364
Equatorial Guinea	Ciudad Nueva Oyala	2017	0.039	0.050	0.063
Equatorial Guinea	Ciudad Nueva Oyala	2000-2017	-0.162	-0.127	-0.094
Equatorial Guinea	Cogo	2000	0.236	0.283	0.345
Equatorial Guinea	Cogo	2017	0.038	0.047	0.058
Equatorial Guinea	Cogo	2000-2017	-0.173	-0.141	-0.107
Equatorial Guinea	Corisco	2000	NA	NA	NA
Equatorial Guinea	Corisco	2017	NA	NA	NA
Equatorial Guinea	Corisco	2000-2017	NA	NA	NA
Equatorial Guinea	Ebebiyin	2000	0.281	0.359	0.434
Equatorial Guinea	Ebebiyin	2017	0.042	0.055	0.070
Equatorial Guinea	Ebebiyin	2000-2017	-0.146	-0.114	-0.085
Equatorial Guinea	Evinayong	2000	0.238	0.296	0.362
Equatorial Guinea	Evinayong	2017	0.040	0.050	0.062
Equatorial Guinea	Evinayong	2000-2017	-0.158	-0.126	-0.091
Equatorial Guinea	Luba	2000	0.216	0.278	0.346
Equatorial Guinea	Luba	2017	0.035	0.045	0.058
Equatorial Guinea	Luba	2000-2017	-0.163	-0.131	-0.091
Equatorial Guinea	Mabana	2000	NA	NA	NA
Equatorial Guinea	Mabana	2017	NA	NA	NA
Equatorial Guinea	Mabana	2000-2017	NA	NA	NA
Equatorial Guinea	Machinda	2000	0.250	0.303	0.374
Equatorial Guinea	Machinda	2017	0.032	0.041	0.051
Equatorial Guinea	Machinda	2000-2017	-0.194	-0.160	-0.125
Equatorial Guinea	Malabo	2000	0.168	0.214	0.268
Equatorial Guinea	Malabo	2017	0.025	0.032	0.042
Equatorial Guinea	Malabo	2000-2017	-0.159	-0.124	-0.087
Equatorial Guinea	Mbini	2000	0.241	0.287	0.348
Equatorial Guinea	Mbini	2017	0.035	0.044	0.057
Equatorial Guinea	Mbini	2000-2017	-0.189	-0.155	-0.118
Equatorial Guinea	Micomeseng	2000	0.252	0.316	0.374
Equatorial Guinea	Micomeseng	2017	0.039	0.050	0.065
Equatorial Guinea	Micomeseng	2000-2017	-0.169	-0.135	-0.104
Equatorial Guinea	Mongomeyen	2000	0.255	0.311	0.375
Equatorial Guinea	Mongomeyen	2017	0.039	0.051	0.063
Equatorial Guinea	Mongomeyen	2000-2017	-0.168	-0.134	-0.103
Equatorial Guinea	Mongomo	2000	0.272	0.334	0.401
Equatorial Guinea	Mongomo	2017	0.041	0.053	0.067

Table 2: Diarrhea DALYs rate by unit *(continued)*

Country	Unit	year	mean rate	lower rate	upper rate
Equatorial Guinea	Mongomo	2000-2017	-0.180	-0.147	-0.117
Equatorial Guinea	Nasng	2000	0.265	0.338	0.406
Equatorial Guinea	Nasng	2017	0.040	0.053	0.067
Equatorial Guinea	Nasng	2000-2017	-0.140	-0.109	-0.079
Equatorial Guinea	Niefang	2000	0.243	0.302	0.363
Equatorial Guinea	Niefang	2017	0.037	0.047	0.059
Equatorial Guinea	Niefang	2000-2017	-0.178	-0.143	-0.109
Equatorial Guinea	Nkimi	2000	0.251	0.307	0.371
Equatorial Guinea	Nkimi	2017	0.037	0.048	0.060
Equatorial Guinea	Nkimi	2000-2017	-0.177	-0.141	-0.110
Equatorial Guinea	Nkue	2000	0.264	0.330	0.400
Equatorial Guinea	Nkue	2017	0.040	0.051	0.066
Equatorial Guinea	Nkue	2000-2017	-0.147	-0.114	-0.083
Equatorial Guinea	Nsok-Nsomo	2000	0.271	0.332	0.398
Equatorial Guinea	Nsok-Nsomo	2017	0.041	0.053	0.068
Equatorial Guinea	Nsok-Nsomo	2000-2017	-0.148	-0.116	-0.085
Equatorial Guinea	Nsork	2000	0.280	0.347	0.428
Equatorial Guinea	Nsork	2017	0.043	0.057	0.073
Equatorial Guinea	Nsork	2000-2017	-0.183	-0.148	-0.115
Equatorial Guinea	Rebola	2000	0.171	0.219	0.275
Equatorial Guinea	Rebola	2017	0.027	0.035	0.045
Equatorial Guinea	Rebola	2000-2017	-0.154	-0.122	-0.085
Equatorial Guinea	Riaba	2000	0.214	0.278	0.347
Equatorial Guinea	Riaba	2017	0.034	0.045	0.057
Equatorial Guinea	Riaba	2000-2017	-0.164	-0.131	-0.091
Equatorial Guinea	Rio Campo	2000	0.239	0.287	0.356
Equatorial Guinea	Rio Campo	2017	0.040	0.051	0.063
Equatorial Guinea	Rio Campo	2000-2017	-0.154	-0.122	-0.088
Equatorial Guinea	San Antonio de Palé	2000	NA	NA	NA
Equatorial Guinea	San Antonio de Palé	2017	NA	NA	NA
Equatorial Guinea	San Antonio de Palé	2000-2017	NA	NA	NA
Eritrea	Adi Keyih	2000	0.306	0.467	0.663
Eritrea	Adi Keyih	2017	0.106	0.169	0.260
Eritrea	Adi Keyih	2000-2017	-0.112	-0.063	-0.009
Eritrea	Adi Kwala	2000	0.316	0.466	0.652
Eritrea	Adi Kwala	2017	0.107	0.173	0.267
Eritrea	Adi Kwala	2000-2017	-0.108	-0.057	-0.002
Eritrea	Adi Teklezan	2000	0.277	0.424	0.636
Eritrea	Adi Teklezan	2017	0.105	0.170	0.262
Eritrea	Adi Teklezan	2000-2017	-0.105	-0.055	0.002
Eritrea	Afabet	2000	0.279	0.427	0.612
Eritrea	Afabet	2017	0.108	0.167	0.259
Eritrea	Afabet	2000-2017	-0.102	-0.052	0.003
Eritrea	Akordat	2000	0.282	0.427	0.610
Eritrea	Akordat	2017	0.116	0.177	0.257
Eritrea	Akordat	2000-2017	-0.091	-0.045	0.010
Eritrea	Areta’	2000	0.245	0.395	0.562
Eritrea	Areta’	2017	0.102	0.175	0.265
Eritrea	Areta’	2000-2017	-0.087	-0.039	0.012
Eritrea	Areza	2000	0.316	0.471	0.690
Eritrea	Areza	2017	0.108	0.175	0.270
Eritrea	Areza	2000-2017	-0.103	-0.056	-0.003
Eritrea	Asmara City	2000	0.276	0.429	0.621
Eritrea	Asmara City	2017	0.106	0.170	0.256
Eritrea	Asmara City	2000-2017	-0.106	-0.056	0.002
Eritrea	Asmat	2000	0.278	0.400	0.562
Eritrea	Asmat	2017	0.106	0.163	0.237
Eritrea	Asmat	2000-2017	-0.092	-0.047	0.006
Eritrea	Barentu	2000	0.295	0.444	0.650
Eritrea	Barentu	2017	0.113	0.180	0.271
Eritrea	Barentu	2000-2017	-0.097	-0.045	0.010

Table 2: Diarrhea DALYs rate by unit *(continued)*

Country	Unit	year	mean rate	lower rate	upper rate
Eritrea	Berikh	2000	0.279	0.431	0.637
Eritrea	Berikh	2017	0.107	0.171	0.256
Eritrea	Berikh	2000-2017	-0.106	-0.056	0.002
Eritrea	Central So. Red-Sea	2000	0.230	0.375	0.557
Eritrea	Central So. Red-Sea	2017	0.102	0.165	0.258
Eritrea	Central So. Red-Sea	2000-2017	-0.092	-0.041	0.003
Eritrea	Dahlak	2000	0.279	0.443	0.665
Eritrea	Dahlak	2017	0.110	0.180	0.277
Eritrea	Dahlak	2000-2017	-0.104	-0.048	0.002
Eritrea	Dekemehare	2000	0.301	0.471	0.676
Eritrea	Dekemehare	2017	0.106	0.171	0.261
Eritrea	Dekemehare	2000-2017	-0.112	-0.061	-0.005
Eritrea	Dghe	2000	0.295	0.426	0.608
Eritrea	Dghe	2017	0.116	0.175	0.256
Eritrea	Dghe	2000-2017	-0.088	-0.040	0.009
Eritrea	Dibarwa	2000	0.307	0.469	0.678
Eritrea	Dibarwa	2017	0.109	0.172	0.265
Eritrea	Dibarwa	2000-2017	-0.109	-0.058	-0.004
Eritrea	Elabered	2000	0.285	0.417	0.626
Eritrea	Elabered	2017	0.107	0.170	0.257
Eritrea	Elabered	2000-2017	-0.100	-0.053	0.005
Eritrea	Foro	2000	0.295	0.460	0.661
Eritrea	Foro	2017	0.106	0.169	0.249
Eritrea	Foro	2000-2017	-0.112	-0.060	-0.012
Eritrea	Forto	2000	0.320	0.447	0.618
Eritrea	Forto	2017	0.120	0.181	0.276
Eritrea	Forto	2000-2017	-0.091	-0.043	0.008
Eritrea	Ghala Nefhi	2000	0.286	0.447	0.649
Eritrea	Ghala Nefhi	2017	0.108	0.171	0.261
Eritrea	Ghala Nefhi	2000-2017	-0.110	-0.058	-0.001
Eritrea	Ghelaelo’	2000	0.276	0.441	0.621
Eritrea	Ghelaelo’	2017	0.108	0.172	0.255
Eritrea	Ghelaelo’	2000-2017	-0.102	-0.049	0.003
Eritrea	Gheleb	2000	0.281	0.420	0.618
Eritrea	Gheleb	2017	0.104	0.168	0.254
Eritrea	Gheleb	2000-2017	-0.104	-0.055	0.006
Eritrea	Ghida‘e	2000	0.292	0.449	0.635
Eritrea	Ghida‘e	2017	0.108	0.171	0.257
Eritrea	Ghida‘e	2000-2017	-0.110	-0.060	-0.003
Eritrea	Gogne	2000	0.306	0.454	0.655
Eritrea	Gogne	2017	0.114	0.180	0.268
Eritrea	Gogne	2000-2017	-0.092	-0.042	0.011
Eritrea	Habero	2000	0.287	0.411	0.598
Eritrea	Habero	2017	0.103	0.163	0.248
Eritrea	Habero	2000-2017	-0.097	-0.050	0.001
Eritrea	Hagaz	2000	0.281	0.414	0.618
Eritrea	Hagaz	2017	0.109	0.171	0.250
Eritrea	Hagaz	2000-2017	-0.095	-0.049	0.007
Eritrea	Halhal	2000	0.280	0.408	0.608
Eritrea	Halhal	2017	0.106	0.167	0.252
Eritrea	Halhal	2000-2017	-0.096	-0.051	0.003
Eritrea	Haykota	2000	0.308	0.450	0.648
Eritrea	Haykota	2017	0.114	0.180	0.266
Eritrea	Haykota	2000-2017	-0.089	-0.041	0.011
Eritrea	Karora	2000	0.281	0.417	0.615
Eritrea	Karora	2017	0.099	0.155	0.236
Eritrea	Karora	2000-2017	-0.101	-0.050	0.001
Eritrea	Keren	2000	0.272	0.410	0.618
Eritrea	Keren	2017	0.108	0.171	0.256
Eritrea	Keren	2000-2017	-0.097	-0.050	0.010
Eritrea	Kerke Bet	2000	0.282	0.399	0.570

Table 2: Diarrhea DALYs rate by unit *(continued)*

Country	Unit	year	mean rate	lower rate	upper rate
Eritrea	Kerke Bet	2017	0.110	0.167	0.241
Eritrea	Kerke Bet	2000-2017	-0.088	-0.040	0.010
Eritrea	Kudo Bu'er	2000	0.321	0.480	0.692
Eritrea	Kudo Bu'er	2017	0.109	0.174	0.271
Eritrea	Kudo Bu'er	2000-2017	-0.110	-0.058	-0.002
Eritrea	La'Elay Gash	2000	0.302	0.448	0.657
Eritrea	La'Elay Gash	2017	0.111	0.182	0.287
Eritrea	La'Elay Gash	2000-2017	-0.090	-0.042	0.013
Eritrea	Logo Anseba	2000	0.304	0.447	0.667
Eritrea	Logo Anseba	2017	0.108	0.172	0.265
Eritrea	Logo Anseba	2000-2017	-0.105	-0.056	0.004
Eritrea	Mansura	2000	0.309	0.451	0.659
Eritrea	Mansura	2017	0.113	0.175	0.257
Eritrea	Mansura	2000-2017	-0.093	-0.048	0.004
Eritrea	May Mine	2000	0.329	0.487	0.703
Eritrea	May Mine	2017	0.108	0.176	0.278
Eritrea	May Mine	2000-2017	-0.106	-0.059	-0.008
Eritrea	Mendefera	2000	0.321	0.473	0.690
Eritrea	Mendefera	2017	0.108	0.172	0.266
Eritrea	Mendefera	2000-2017	-0.111	-0.059	-0.003
Eritrea	Mitswa'e City	2000	0.282	0.435	0.624
Eritrea	Mitswa'e City	2017	0.112	0.173	0.268
Eritrea	Mitswa'e City	2000-2017	-0.109	-0.057	-0.004
Eritrea	Mogolo	2000	0.300	0.448	0.644
Eritrea	Mogolo	2017	0.114	0.178	0.269
Eritrea	Mogolo	2000-2017	-0.089	-0.042	0.011
Eritrea	Nakfa	2000	0.280	0.408	0.580
Eritrea	Nakfa	2017	0.103	0.161	0.244
Eritrea	Nakfa	2000-2017	-0.102	-0.050	0.003
Eritrea	Omhajer	2000	0.341	0.483	0.672
Eritrea	Omhajer	2017	0.113	0.189	0.287
Eritrea	Omhajer	2000-2017	-0.089	-0.042	0.012
Eritrea	Segeneyiti	2000	0.309	0.473	0.676
Eritrea	Segeneyiti	2017	0.106	0.170	0.256
Eritrea	Segeneyiti	2000-2017	-0.115	-0.062	-0.007
Eritrea	Sel'a	2000	0.273	0.401	0.569
Eritrea	Sel'a	2017	0.107	0.162	0.235
Eritrea	Sel'a	2000-2017	-0.090	-0.045	0.003
Eritrea	Senafe	2000	0.307	0.458	0.661
Eritrea	Senafe	2017	0.103	0.167	0.255
Eritrea	Senafe	2000-2017	-0.111	-0.062	-0.009
Eritrea	Serejeka	2000	0.277	0.428	0.623
Eritrea	Serejeka	2017	0.107	0.170	0.258
Eritrea	Serejeka	2000-2017	-0.105	-0.056	0.003
Eritrea	Sheib	2000	0.284	0.437	0.619
Eritrea	Sheib	2017	0.107	0.168	0.253
Eritrea	Sheib	2000-2017	-0.108	-0.055	-0.002
Eritrea	Shemboko	2000	0.322	0.472	0.683
Eritrea	Shemboko	2017	0.115	0.179	0.277
Eritrea	Shemboko	2000-2017	-0.093	-0.051	0.002
Eritrea	So. Southern Red-Sea	2000	0.250	0.387	0.577
Eritrea	So. Southern Red-Sea	2017	0.107	0.170	0.268
Eritrea	So. Southern Red-Sea	2000-2017	-0.100	-0.049	-0.003
Eritrea	Teseneye	2000	0.313	0.459	0.648
Eritrea	Teseneye	2017	0.112	0.186	0.283
Eritrea	Teseneye	2000-2017	-0.088	-0.040	0.012
Eritrea	Tsorena	2000	0.344	0.490	0.683
Eritrea	Tsorena	2017	0.104	0.168	0.253
Eritrea	Tsorena	2000-2017	-0.117	-0.067	-0.014
Ethiopia	Addis Abeba	2000	0.114	0.155	0.203
Ethiopia	Addis Abeba	2017	0.068	0.101	0.142

Table 2: Diarrhea DALYs rate by unit *(continued)*

Country	Unit	year	mean rate	lower rate	upper rate
Ethiopia	Addis Abeba	2000-2017	-0.082	-0.033	0.021
Ethiopia	Afar Zone 1	2000	0.242	0.332	0.436
Ethiopia	Afar Zone 1	2017	0.148	0.224	0.318
Ethiopia	Afar Zone 1	2000-2017	-0.075	-0.026	0.018
Ethiopia	Afar Zone 2	2000	0.284	0.383	0.501
Ethiopia	Afar Zone 2	2017	0.181	0.260	0.351
Ethiopia	Afar Zone 2	2000-2017	-0.066	-0.017	0.022
Ethiopia	Afar Zone 3	2000	0.301	0.395	0.507
Ethiopia	Afar Zone 3	2017	0.138	0.199	0.281
Ethiopia	Afar Zone 3	2000-2017	-0.092	-0.043	0.003
Ethiopia	Afar Zone 4	2000	0.263	0.353	0.466
Ethiopia	Afar Zone 4	2017	0.161	0.241	0.338
Ethiopia	Afar Zone 4	2000-2017	-0.068	-0.021	0.022
Ethiopia	Afar Zone 5	2000	0.272	0.357	0.466
Ethiopia	Afar Zone 5	2017	0.148	0.210	0.314
Ethiopia	Afar Zone 5	2000-2017	-0.087	-0.036	0.010
Ethiopia	Afder	2000	0.231	0.333	0.447
Ethiopia	Afder	2017	0.165	0.237	0.335
Ethiopia	Afder	2000-2017	-0.064	-0.017	0.029
Ethiopia	Agew Awi	2000	0.320	0.419	0.554
Ethiopia	Agew Awi	2017	0.145	0.207	0.293
Ethiopia	Agew Awi	2000-2017	-0.087	-0.037	0.014
Ethiopia	Agnuak	2000	0.275	0.356	0.457
Ethiopia	Agnuak	2017	0.092	0.130	0.183
Ethiopia	Agnuak	2000-2017	-0.114	-0.072	-0.024
Ethiopia	Alaba	2000	0.311	0.409	0.529
Ethiopia	Alaba	2017	0.108	0.162	0.232
Ethiopia	Alaba	2000-2017	-0.102	-0.059	-0.013
Ethiopia	Alle	2000	0.267	0.371	0.511
Ethiopia	Alle	2017	0.108	0.166	0.242
Ethiopia	Alle	2000-2017	-0.096	-0.050	-0.001
Ethiopia	Amaro	2000	0.315	0.420	0.547
Ethiopia	Amaro	2017	0.109	0.163	0.234
Ethiopia	Amaro	2000-2017	-0.110	-0.063	-0.016
Ethiopia	Argoba	2000	0.269	0.358	0.472
Ethiopia	Argoba	2017	0.114	0.169	0.250
Ethiopia	Argoba	2000-2017	-0.121	-0.071	-0.022
Ethiopia	Arsi	2000	0.281	0.369	0.483
Ethiopia	Arsi	2017	0.091	0.132	0.187
Ethiopia	Arsi	2000-2017	-0.134	-0.079	-0.027
Ethiopia	Asosa	2000	0.359	0.448	0.548
Ethiopia	Asosa	2017	0.130	0.184	0.263
Ethiopia	Asosa	2000-2017	-0.108	-0.062	-0.013
Ethiopia	Bahir Dar Special Zone	2000	0.247	0.339	0.440
Ethiopia	Bahir Dar Special Zone	2017	0.125	0.182	0.263
Ethiopia	Bahir Dar Special Zone	2000-2017	-0.095	-0.038	0.017
Ethiopia	Bale	2000	0.249	0.341	0.448
Ethiopia	Bale	2017	0.098	0.143	0.205
Ethiopia	Bale	2000-2017	-0.110	-0.055	-0.002
Ethiopia	Basketo	2000	0.305	0.417	0.566
Ethiopia	Basketo	2017	0.117	0.176	0.258
Ethiopia	Basketo	2000-2017	-0.108	-0.062	-0.020
Ethiopia	Bench Maji	2000	0.289	0.371	0.494
Ethiopia	Bench Maji	2017	0.120	0.177	0.246
Ethiopia	Bench Maji	2000-2017	-0.088	-0.043	-0.003
Ethiopia	Borena	2000	0.329	0.428	0.552
Ethiopia	Borena	2017	0.129	0.185	0.271
Ethiopia	Borena	2000-2017	-0.093	-0.047	-0.003
Ethiopia	Burji	2000	0.299	0.406	0.535
Ethiopia	Burji	2017	0.102	0.159	0.227
Ethiopia	Burji	2000-2017	-0.107	-0.061	-0.009

Table 2: Diarrhea DALYs rate by unit *(continued)*

Country	Unit	year	mean rate	lower rate	upper rate
Ethiopia	Dawro	2000	0.313	0.419	0.573
Ethiopia	Dawro	2017	0.127	0.185	0.267
Ethiopia	Dawro	2000-2017	-0.099	-0.050	-0.006
Ethiopia	Debub Gondar	2000	0.316	0.417	0.544
Ethiopia	Debub Gondar	2017	0.142	0.195	0.273
Ethiopia	Debub Gondar	2000-2017	-0.097	-0.046	0.002
Ethiopia	Debub Mirab Shewa	2000	0.276	0.367	0.492
Ethiopia	Debub Mirab Shewa	2017	0.101	0.148	0.208
Ethiopia	Debub Mirab Shewa	2000-2017	-0.113	-0.063	-0.017
Ethiopia	Debub Omo	2000	0.300	0.416	0.560
Ethiopia	Debub Omo	2017	0.123	0.184	0.262
Ethiopia	Debub Omo	2000-2017	-0.100	-0.054	-0.008
Ethiopia	Debub Wollo	2000	0.258	0.344	0.479
Ethiopia	Debub Wollo	2017	0.115	0.166	0.237
Ethiopia	Debub Wollo	2000-2017	-0.104	-0.055	-0.005
Ethiopia	Debubawi	2000	0.194	0.253	0.337
Ethiopia	Debubawi	2017	0.096	0.136	0.192
Ethiopia	Debubawi	2000-2017	-0.088	-0.042	0.002
Ethiopia	Derashe	2000	0.257	0.354	0.473
Ethiopia	Derashe	2017	0.104	0.158	0.227
Ethiopia	Derashe	2000-2017	-0.094	-0.051	0.001
Ethiopia	Dire Dawa	2000	0.253	0.337	0.422
Ethiopia	Dire Dawa	2017	0.108	0.164	0.227
Ethiopia	Dire Dawa	2000-2017	-0.095	-0.046	0.004
Ethiopia	Doolo	2000	0.194	0.293	0.429
Ethiopia	Doolo	2017	0.155	0.238	0.332
Ethiopia	Doolo	2000-2017	-0.065	-0.018	0.033
Ethiopia	Fafan	2000	0.277	0.362	0.462
Ethiopia	Fafan	2017	0.160	0.225	0.312
Ethiopia	Fafan	2000-2017	-0.093	-0.045	-0.002
Ethiopia	Gamo Gofa	2000	0.293	0.392	0.507
Ethiopia	Gamo Gofa	2017	0.114	0.167	0.239
Ethiopia	Gamo Gofa	2000-2017	-0.100	-0.057	-0.012
Ethiopia	Gedeo	2000	0.314	0.418	0.529
Ethiopia	Gedeo	2017	0.108	0.163	0.247
Ethiopia	Gedeo	2000-2017	-0.109	-0.061	-0.014
Ethiopia	Guji	2000	0.314	0.415	0.515
Ethiopia	Guji	2017	0.109	0.163	0.250
Ethiopia	Guji	2000-2017	-0.104	-0.054	-0.010
Ethiopia	Gurage	2000	0.266	0.356	0.475
Ethiopia	Gurage	2017	0.113	0.159	0.216
Ethiopia	Gurage	2000-2017	-0.108	-0.057	-0.011
Ethiopia	Hadiya	2000	0.296	0.392	0.509
Ethiopia	Hadiya	2017	0.116	0.168	0.231
Ethiopia	Hadiya	2000-2017	-0.097	-0.049	-0.005
Ethiopia	Hareri	2000	0.229	0.310	0.392
Ethiopia	Hareri	2017	0.103	0.152	0.207
Ethiopia	Hareri	2000-2017	-0.091	-0.044	0.005
Ethiopia	Horo Guduru	2000	0.251	0.354	0.485
Ethiopia	Horo Guduru	2017	0.117	0.173	0.242
Ethiopia	Horo Guduru	2000-2017	-0.100	-0.050	-0.005
Ethiopia	Ilubabor	2000	0.309	0.415	0.528
Ethiopia	Ilubabor	2017	0.116	0.170	0.231
Ethiopia	Ilubabor	2000-2017	-0.096	-0.053	-0.009
Ethiopia	Jarar	2000	0.218	0.316	0.442
Ethiopia	Jarar	2017	0.129	0.196	0.266
Ethiopia	Jarar	2000-2017	-0.087	-0.036	0.013
Ethiopia	Jimma	2000	0.320	0.425	0.559
Ethiopia	Jimma	2017	0.150	0.207	0.280
Ethiopia	Jimma	2000-2017	-0.088	-0.042	0.007
Ethiopia	Keffa	2000	0.305	0.393	0.511

Table 2: Diarrhea DALYs rate by unit *(continued)*

Country	Unit	year	mean rate	lower rate	upper rate
Ethiopia	Keffa	2017	0.121	0.176	0.242
Ethiopia	Keffa	2000-2017	-0.096	-0.049	-0.006
Ethiopia	Kelem Wellega	2000	0.302	0.403	0.527
Ethiopia	Kelem Wellega	2017	0.116	0.167	0.236
Ethiopia	Kelem Wellega	2000-2017	-0.085	-0.042	0.005
Ethiopia	Kemashi	2000	0.337	0.439	0.576
Ethiopia	Kemashi	2017	0.133	0.199	0.288
Ethiopia	Kemashi	2000-2017	-0.097	-0.052	-0.008
Ethiopia	Kembata Tembaro	2000	0.298	0.388	0.510
Ethiopia	Kembata Tembaro	2017	0.115	0.168	0.232
Ethiopia	Kembata Tembaro	2000-2017	-0.099	-0.053	-0.008
Ethiopia	Konso	2000	0.252	0.352	0.481
Ethiopia	Konso	2017	0.107	0.162	0.232
Ethiopia	Konso	2000-2017	-0.093	-0.048	0.003
Ethiopia	Konta	2000	0.302	0.406	0.553
Ethiopia	Konta	2017	0.131	0.188	0.269
Ethiopia	Konta	2000-2017	-0.098	-0.045	-0.001
Ethiopia	Korahe	2000	0.210	0.307	0.429
Ethiopia	Korahe	2017	0.140	0.211	0.290
Ethiopia	Korahe	2000-2017	-0.072	-0.024	0.034
Ethiopia	Liben	2000	0.249	0.341	0.453
Ethiopia	Liben	2017	0.150	0.227	0.312
Ethiopia	Liben	2000-2017	-0.071	-0.021	0.025
Ethiopia	Majang	2000	0.266	0.344	0.461
Ethiopia	Majang	2017	0.090	0.133	0.185
Ethiopia	Majang	2000-2017	-0.098	-0.054	-0.011
Ethiopia	Mehakelegnaw	2000	0.207	0.267	0.344
Ethiopia	Mehakelegnaw	2017	0.105	0.150	0.205
Ethiopia	Mehakelegnaw	2000-2017	-0.082	-0.038	0.009
Ethiopia	Metekel	2000	0.304	0.394	0.504
Ethiopia	Metekel	2017	0.141	0.199	0.279
Ethiopia	Metekel	2000-2017	-0.090	-0.043	0.004
Ethiopia	Mi'irabawi	2000	0.263	0.341	0.444
Ethiopia	Mi'irabawi	2017	0.113	0.163	0.234
Ethiopia	Mi'irabawi	2000-2017	-0.090	-0.041	0.011
Ethiopia	Mirab Arsi	2000	0.312	0.408	0.528
Ethiopia	Mirab Arsi	2017	0.107	0.156	0.218
Ethiopia	Mirab Arsi	2000-2017	-0.098	-0.056	-0.010
Ethiopia	Mirab Gojjam	2000	0.292	0.395	0.511
Ethiopia	Mirab Gojjam	2017	0.141	0.200	0.273
Ethiopia	Mirab Gojjam	2000-2017	-0.089	-0.037	0.013
Ethiopia	Mirab Hararghe	2000	0.264	0.364	0.481
Ethiopia	Mirab Hararghe	2017	0.094	0.145	0.211
Ethiopia	Mirab Hararghe	2000-2017	-0.110	-0.065	-0.013
Ethiopia	Mirab Shewa	2000	0.279	0.372	0.484
Ethiopia	Mirab Shewa	2017	0.105	0.157	0.218
Ethiopia	Mirab Shewa	2000-2017	-0.118	-0.070	-0.026
Ethiopia	Mirab Welega	2000	0.318	0.419	0.537
Ethiopia	Mirab Welega	2017	0.109	0.158	0.230
Ethiopia	Mirab Welega	2000-2017	-0.092	-0.049	-0.002
Ethiopia	Misraq Gojjam	2000	0.304	0.423	0.564
Ethiopia	Misraq Gojjam	2017	0.134	0.196	0.277
Ethiopia	Misraq Gojjam	2000-2017	-0.102	-0.051	-0.004
Ethiopia	Misraq Harerge	2000	0.266	0.355	0.439
Ethiopia	Misraq Harerge	2017	0.099	0.143	0.195
Ethiopia	Misraq Harerge	2000-2017	-0.108	-0.061	-0.008
Ethiopia	Misraq Shewa	2000	0.258	0.338	0.441
Ethiopia	Misraq Shewa	2017	0.090	0.131	0.188
Ethiopia	Misraq Shewa	2000-2017	-0.125	-0.073	-0.023
Ethiopia	Misraq Wellega	2000	0.289	0.386	0.518
Ethiopia	Misraq Wellega	2017	0.116	0.174	0.242

Table 2: Diarrhea DALYs rate by unit *(continued)*

Country	Unit	year	mean rate	lower rate	upper rate
Ethiopia	Misraq Wellega	2000-2017	-0.096	-0.048	-0.003
Ethiopia	Misraqawi	2000	0.179	0.241	0.310
Ethiopia	Misraqawi	2017	0.097	0.142	0.192
Ethiopia	Misraqawi	2000-2017	-0.083	-0.035	0.008
Ethiopia	Nogob	2000	0.253	0.352	0.467
Ethiopia	Nogob	2017	0.118	0.189	0.271
Ethiopia	Nogob	2000-2017	-0.098	-0.048	0.003
Ethiopia	North Shewa	2000	0.246	0.326	0.427
Ethiopia	North Shewa	2000	0.321	0.411	0.526
Ethiopia	North Shewa	2017	0.087	0.132	0.184
Ethiopia	North Shewa	2017	0.110	0.160	0.222
Ethiopia	North Shewa	2000-2017	-0.126	-0.078	-0.026
Ethiopia	North Shewa	2000-2017	-0.129	-0.081	-0.032
Ethiopia	Nuer	2000	0.244	0.341	0.453
Ethiopia	Nuer	2017	0.102	0.143	0.200
Ethiopia	Nuer	2000-2017	-0.115	-0.066	-0.020
Ethiopia	Oromia	2000	0.284	0.376	0.482
Ethiopia	Oromia	2017	0.124	0.175	0.260
Ethiopia	Oromia	2000-2017	-0.123	-0.071	-0.024
Ethiopia	Semen Gondar	2000	0.343	0.453	0.572
Ethiopia	Semen Gondar	2017	0.162	0.224	0.317
Ethiopia	Semen Gondar	2000-2017	-0.090	-0.041	0.012
Ethiopia	Semen Wello	2000	0.269	0.357	0.472
Ethiopia	Semen Wello	2017	0.128	0.185	0.265
Ethiopia	Semen Wello	2000-2017	-0.100	-0.048	0.006
Ethiopia	Semien Mi'irabaw	2000	0.243	0.318	0.412
Ethiopia	Semien Mi'irabaw	2017	0.111	0.159	0.227
Ethiopia	Semien Mi'irabaw	2000-2017	-0.086	-0.041	0.009
Ethiopia	Shabelle	2000	0.207	0.315	0.430
Ethiopia	Shabelle	2017	0.138	0.213	0.306
Ethiopia	Shabelle	2000-2017	-0.074	-0.023	0.031
Ethiopia	Sheka	2000	0.275	0.351	0.457
Ethiopia	Sheka	2017	0.110	0.165	0.225
Ethiopia	Sheka	2000-2017	-0.085	-0.040	0.004
Ethiopia	Sidama	2000	0.306	0.399	0.508
Ethiopia	Sidama	2017	0.099	0.147	0.209
Ethiopia	Sidama	2000-2017	-0.114	-0.067	-0.020
Ethiopia	Silti	2000	0.284	0.379	0.499
Ethiopia	Silti	2017	0.113	0.160	0.229
Ethiopia	Silti	2000-2017	-0.103	-0.056	-0.012
Ethiopia	Siti	2000	0.256	0.336	0.419
Ethiopia	Siti	2017	0.142	0.203	0.283
Ethiopia	Siti	2000-2017	-0.098	-0.053	-0.005
Ethiopia	Wag Himra	2000	0.297	0.381	0.498
Ethiopia	Wag Himra	2017	0.149	0.214	0.300
Ethiopia	Wag Himra	2000-2017	-0.094	-0.046	0.008
Ethiopia	Wolayita	2000	0.325	0.415	0.556
Ethiopia	Wolayita	2017	0.115	0.170	0.240
Ethiopia	Wolayita	2000-2017	-0.106	-0.062	-0.017
Ethiopia	Yem	2000	0.289	0.396	0.532
Ethiopia	Yem	2017	0.119	0.170	0.240
Ethiopia	Yem	2000-2017	-0.104	-0.051	-0.006
Gabon	Abanga-Bigné	2000	0.063	0.079	0.098
Gabon	Abanga-Bigné	2017	0.037	0.048	0.059
Gabon	Abanga-Bigné	2000-2017	-0.053	-0.026	0.005
Gabon	Basse Banio	2000	0.060	0.077	0.091
Gabon	Basse Banio	2017	0.036	0.046	0.058
Gabon	Basse Banio	2000-2017	-0.053	-0.024	0.003
Gabon	Bendjé	2000	0.061	0.076	0.093
Gabon	Bendjé	2017	0.037	0.046	0.058
Gabon	Bendjé	2000-2017	-0.068	-0.035	-0.004

Table 2: Diarrhea DALYs rate by unit *(continued)*

Country	Unit	year	mean rate	lower rate	upper rate
Gabon	Boumi-lowetsi	2000	0.058	0.075	0.093
Gabon	Boumi-lowetsi	2017	0.035	0.044	0.056
Gabon	Boumi-lowetsi	2000-2017	-0.056	-0.024	0.004
Gabon	Dola	2000	0.063	0.081	0.099
Gabon	Dola	2017	0.035	0.045	0.057
Gabon	Dola	2000-2017	-0.062	-0.030	-0.001
Gabon	Douigny	2000	0.062	0.078	0.099
Gabon	Douigny	2017	0.034	0.042	0.055
Gabon	Douigny	2000-2017	-0.062	-0.031	0.002
Gabon	Douya Onoye	2000	0.061	0.079	0.099
Gabon	Douya Onoye	2017	0.033	0.043	0.054
Gabon	Douya Onoye	2000-2017	-0.057	-0.027	0.004
Gabon	Étimboué	2000	0.060	0.076	0.094
Gabon	Étimboué	2017	0.034	0.043	0.054
Gabon	Étimboué	2000-2017	-0.065	-0.037	-0.007
Gabon	Haut-Como	2000	0.056	0.071	0.087
Gabon	Haut-Como	2017	0.040	0.053	0.066
Gabon	Haut-Como	2000-2017	-0.045	-0.010	0.021
Gabon	Haut-Ntem	2000	0.072	0.088	0.108
Gabon	Haut-Ntem	2017	0.047	0.059	0.072
Gabon	Haut-Ntem	2000-2017	-0.020	0.005	0.033
Gabon	Haute-Banio	2000	0.063	0.081	0.100
Gabon	Haute-Banio	2017	0.037	0.049	0.061
Gabon	Haute-Banio	2000-2017	-0.044	-0.014	0.019
Gabon	Ivindo	2000	0.064	0.079	0.095
Gabon	Ivindo	2017	0.041	0.051	0.062
Gabon	Ivindo	2000-2017	-0.039	-0.014	0.012
Gabon	Komo	2000	0.077	0.094	0.112
Gabon	Komo	2017	0.037	0.047	0.060
Gabon	Komo	2000-2017	-0.072	-0.044	-0.011
Gabon	Komo-Mondah	2000	0.068	0.084	0.103
Gabon	Komo-Mondah	2017	0.035	0.044	0.058
Gabon	Komo-Mondah	2000-2017	-0.067	-0.025	0.006
Gabon	Léboumbi-Leyou	2000	0.062	0.077	0.094
Gabon	Léboumbi-Leyou	2017	0.035	0.045	0.055
Gabon	Léboumbi-Leyou	2000-2017	-0.057	-0.026	0.007
Gabon	Léconi-Djoué	2000	0.061	0.077	0.095
Gabon	Léconi-Djoué	2017	0.035	0.044	0.054
Gabon	Léconi-Djoué	2000-2017	-0.056	-0.025	0.010
Gabon	Lékoko	2000	0.059	0.076	0.094
Gabon	Lékoko	2017	0.037	0.048	0.058
Gabon	Lékoko	2000-2017	-0.047	-0.019	0.013
Gabon	Lolo Bouenguidi	2000	0.066	0.080	0.100
Gabon	Lolo Bouenguidi	2017	0.042	0.052	0.066
Gabon	Lolo Bouenguidi	2000-2017	-0.050	-0.019	0.012
Gabon	Lombo-Bouenguidi	2000	0.067	0.082	0.103
Gabon	Lombo-Bouenguidi	2017	0.039	0.050	0.064
Gabon	Lombo-Bouenguidi	2000-2017	-0.054	-0.024	0.009
Gabon	Lopé	2000	0.065	0.080	0.098
Gabon	Lopé	2017	0.040	0.050	0.063
Gabon	Lopé	2000-2017	-0.052	-0.021	0.006
Gabon	Louetsi-Wano	2000	0.061	0.078	0.096
Gabon	Louetsi-Wano	2017	0.034	0.043	0.056
Gabon	Louetsi-Wano	2000-2017	-0.063	-0.028	0.004
Gabon	Mougoutsi	2000	0.065	0.080	0.098
Gabon	Mougoutsi	2017	0.038	0.047	0.059
Gabon	Mougoutsi	2000-2017	-0.055	-0.025	0.003
Gabon	Mouloudnou	2000	0.067	0.081	0.098
Gabon	Mouloudnou	2017	0.039	0.048	0.059
Gabon	Mouloudnou	2000-2017	-0.053	-0.026	0.006
Gabon	Mpassa	2000	0.063	0.077	0.094

Table 2: Diarrhea DALYs rate by unit *(continued)*

Country	Unit	year	mean rate	lower rate	upper rate
Gabon	Mpassa	2017	0.036	0.045	0.055
Gabon	Mpassa	2000-2017	-0.052	-0.024	0.011
Gabon	Mvoung	2000	0.063	0.081	0.102
Gabon	Mvoung	2017	0.040	0.050	0.062
Gabon	Mvoung	2000-2017	-0.056	-0.022	0.010
Gabon	Ndolou	2000	0.060	0.076	0.095
Gabon	Ndolou	2017	0.032	0.042	0.054
Gabon	Ndolou	2000-2017	-0.063	-0.033	-0.003
Gabon	Ndougou	2000	0.060	0.077	0.096
Gabon	Ndougou	2017	0.033	0.042	0.054
Gabon	Ndougou	2000-2017	-0.065	-0.034	-0.001
Gabon	Noya	2000	0.076	0.091	0.112
Gabon	Noya	2017	0.043	0.053	0.069
Gabon	Noya	2000-2017	-0.081	-0.045	-0.014
Gabon	Ntem	2000	0.068	0.084	0.102
Gabon	Ntem	2017	0.044	0.056	0.070
Gabon	Ntem	2000-2017	-0.020	0.011	0.040
Gabon	Ogooué et des Lacs	2000	0.062	0.078	0.097
Gabon	Ogooué et des Lacs	2017	0.038	0.048	0.060
Gabon	Ogooué et des Lacs	2000-2017	-0.059	-0.028	0.004
Gabon	Ogoulou	2000	0.062	0.076	0.095
Gabon	Ogoulou	2017	0.036	0.046	0.056
Gabon	Ogoulou	2000-2017	-0.047	-0.017	0.011
Gabon	Okano	2000	0.069	0.086	0.108
Gabon	Okano	2017	0.041	0.054	0.067
Gabon	Okano	2000-2017	-0.057	-0.023	0.007
Gabon	Plateaux	2000	0.060	0.075	0.093
Gabon	Plateaux	2017	0.035	0.045	0.055
Gabon	Plateaux	2000-2017	-0.053	-0.025	0.009
Gabon	Sébé-Brikolo	2000	0.061	0.077	0.095
Gabon	Sébé-Brikolo	2017	0.038	0.048	0.059
Gabon	Sébé-Brikolo	2000-2017	-0.042	-0.012	0.017
Gabon	Tsamba Mangotsi	2000	0.062	0.076	0.094
Gabon	Tsamba Mangotsi	2017	0.036	0.045	0.057
Gabon	Tsamba Mangotsi	2000-2017	-0.052	-0.022	0.009
Gabon	Woleu	2000	0.068	0.083	0.104
Gabon	Woleu	2017	0.042	0.053	0.068
Gabon	Woleu	2000-2017	-0.050	-0.020	0.014
Gabon	Zadié	2000	0.064	0.080	0.100
Gabon	Zadié	2017	0.043	0.054	0.064
Gabon	Zadié	2000-2017	-0.029	-0.001	0.030
Gambia	Banjul	2000	NA	NA	NA
Gambia	Banjul	2017	NA	NA	NA
Gambia	Banjul	2000-2017	NA	NA	NA
Gambia	Central Baddibu	2000	0.189	0.245	0.312
Gambia	Central Baddibu	2017	0.061	0.086	0.118
Gambia	Central Baddibu	2000-2017	-0.177	-0.088	-0.006
Gambia	Foni Bintang Karanai	2000	0.174	0.228	0.285
Gambia	Foni Bintang Karanai	2017	0.068	0.097	0.132
Gambia	Foni Bintang Karanai	2000-2017	-0.172	-0.078	0.006
Gambia	Foni Bondali	2000	0.180	0.233	0.293
Gambia	Foni Bondali	2017	0.066	0.095	0.129
Gambia	Foni Bondali	2000-2017	-0.166	-0.076	0.012
Gambia	Foni Brefet	2000	0.169	0.222	0.278
Gambia	Foni Brefet	2017	0.066	0.096	0.131
Gambia	Foni Brefet	2000-2017	-0.172	-0.075	0.016
Gambia	Foni Jarrol	2000	0.188	0.243	0.304
Gambia	Foni Jarrol	2017	0.068	0.096	0.133
Gambia	Foni Jarrol	2000-2017	-0.163	-0.073	0.013
Gambia	Foni Kansala	2000	0.176	0.232	0.292
Gambia	Foni Kansala	2017	0.067	0.096	0.130

Table 2: Diarrhea DALYs rate by unit *(continued)*

Country	Unit	year	mean rate	lower rate	upper rate
Gambia	Foni Kansala	2000-2017	-0.166	-0.081	0.007
Gambia	Fulladu East	2000	0.187	0.235	0.304
Gambia	Fulladu East	2017	0.047	0.066	0.092
Gambia	Fulladu East	2000-2017	-0.159	-0.053	0.033
Gambia	Fulladu West	2000	0.188	0.243	0.311
Gambia	Fulladu West	2017	0.055	0.076	0.107
Gambia	Fulladu West	2000-2017	-0.161	-0.064	0.025
Gambia	Janjanbureh	2000	0.186	0.244	0.311
Gambia	Janjanbureh	2017	0.051	0.074	0.106
Gambia	Janjanbureh	2000-2017	-0.165	-0.066	0.025
Gambia	Jarra Central	2000	0.193	0.252	0.321
Gambia	Jarra Central	2017	0.060	0.082	0.114
Gambia	Jarra Central	2000-2017	-0.164	-0.079	0.007
Gambia	Jarra East	2000	0.195	0.254	0.324
Gambia	Jarra East	2017	0.059	0.081	0.112
Gambia	Jarra East	2000-2017	-0.160	-0.075	0.012
Gambia	Jarra West	2000	0.192	0.251	0.318
Gambia	Jarra West	2017	0.059	0.082	0.113
Gambia	Jarra West	2000-2017	-0.176	-0.087	-0.001
Gambia	Jokadu	2000	0.188	0.238	0.299
Gambia	Jokadu	2017	0.060	0.088	0.119
Gambia	Jokadu	2000-2017	-0.167	-0.082	-0.003
Gambia	Kanifing	2000	0.159	0.203	0.256
Gambia	Kanifing	2017	0.057	0.088	0.120
Gambia	Kanifing	2000-2017	-0.165	-0.074	0.018
Gambia	Kantora	2000	0.185	0.233	0.297
Gambia	Kantora	2017	0.044	0.062	0.086
Gambia	Kantora	2000-2017	-0.142	-0.050	0.038
Gambia	Kiang Central	2000	0.187	0.242	0.306
Gambia	Kiang Central	2017	0.061	0.086	0.119
Gambia	Kiang Central	2000-2017	-0.176	-0.086	-0.001
Gambia	Kiang East	2000	0.194	0.252	0.317
Gambia	Kiang East	2017	0.061	0.086	0.119
Gambia	Kiang East	2000-2017	-0.179	-0.087	-0.001
Gambia	Kiang West	2000	0.182	0.233	0.296
Gambia	Kiang West	2017	0.065	0.092	0.125
Gambia	Kiang West	2000-2017	-0.167	-0.080	0.005
Gambia	Kombo Central	2000	0.148	0.192	0.243
Gambia	Kombo Central	2017	0.056	0.086	0.119
Gambia	Kombo Central	2000-2017	-0.165	-0.073	0.023
Gambia	Kombo East	2000	0.163	0.213	0.267
Gambia	Kombo East	2017	0.061	0.092	0.126
Gambia	Kombo East	2000-2017	-0.164	-0.073	0.017
Gambia	Kombo Saint Mary	2000	0.156	0.201	0.255
Gambia	Kombo Saint Mary	2017	0.056	0.087	0.119
Gambia	Kombo Saint Mary	2000-2017	-0.164	-0.072	0.019
Gambia	Kombo South	2000	0.142	0.186	0.234
Gambia	Kombo South	2017	0.054	0.084	0.115
Gambia	Kombo South	2000-2017	-0.168	-0.073	0.019
Gambia	Lower Baddibu	2000	0.186	0.236	0.303
Gambia	Lower Baddibu	2017	0.061	0.087	0.118
Gambia	Lower Baddibu	2000-2017	-0.170	-0.084	-0.002
Gambia	Lower Nuimi	2000	0.181	0.226	0.285
Gambia	Lower Nuimi	2017	0.057	0.088	0.124
Gambia	Lower Nuimi	2000-2017	-0.165	-0.080	0.005
Gambia	Lower Saloum	2000	0.193	0.252	0.330
Gambia	Lower Saloum	2017	0.057	0.080	0.110
Gambia	Lower Saloum	2000-2017	-0.172	-0.079	0.007
Gambia	Niamina Dankunku	2000	0.197	0.254	0.329
Gambia	Niamina Dankunku	2017	0.059	0.082	0.112
Gambia	Niamina Dankunku	2000-2017	-0.169	-0.079	0.007

Table 2: Diarrhea DALYs rate by unit *(continued)*

Country	Unit	year	mean rate	lower rate	upper rate
Gambia	Niamina East	2000	0.194	0.253	0.321
Gambia	Niamina East	2017	0.057	0.080	0.109
Gambia	Niamina East	2000-2017	-0.166	-0.074	0.012
Gambia	Niamina West	2000	0.196	0.255	0.328
Gambia	Niamina West	2017	0.060	0.083	0.113
Gambia	Niamina West	2000-2017	-0.163	-0.075	0.012
Gambia	Niani	2000	0.195	0.253	0.321
Gambia	Niani	2017	0.056	0.078	0.110
Gambia	Niani	2000-2017	-0.164	-0.070	0.014
Gambia	Nianija	2000	0.195	0.253	0.323
Gambia	Nianija	2017	0.058	0.079	0.109
Gambia	Nianija	2000-2017	-0.166	-0.075	0.009
Gambia	Sami	2000	0.188	0.246	0.316
Gambia	Sami	2017	0.053	0.074	0.106
Gambia	Sami	2000-2017	-0.158	-0.063	0.025
Gambia	Sandu	2000	0.189	0.241	0.312
Gambia	Sandu	2017	0.050	0.071	0.100
Gambia	Sandu	2000-2017	-0.158	-0.055	0.032
Gambia	Upper Baddibu	2000	0.194	0.253	0.318
Gambia	Upper Baddibu	2017	0.059	0.083	0.110
Gambia	Upper Baddibu	2000-2017	-0.173	-0.088	-0.006
Gambia	Upper Nuimi	2000	0.186	0.234	0.290
Gambia	Upper Nuimi	2017	0.060	0.091	0.127
Gambia	Upper Nuimi	2000-2017	-0.166	-0.079	0.004
Gambia	Upper Saloum	2000	0.197	0.257	0.331
Gambia	Upper Saloum	2017	0.058	0.080	0.110
Gambia	Upper Saloum	2000-2017	-0.172	-0.079	0.005
Gambia	Wuli	2000	0.184	0.236	0.306
Gambia	Wuli	2017	0.047	0.066	0.092
Gambia	Wuli	2000-2017	-0.151	-0.051	0.037
Ghana	Abura-Asebu-Kwamankese	2000	0.084	0.134	0.198
Ghana	Abura-Asebu-Kwamankese	2017	0.041	0.065	0.100
Ghana	Abura-Asebu-Kwamankese	2000-2017	-0.154	-0.050	0.039
Ghana	Accra	2000	0.095	0.153	0.227
Ghana	Accra	2017	0.056	0.086	0.130
Ghana	Accra	2000-2017	-0.147	-0.050	0.047
Ghana	Adaklu Anyigbe	2000	0.097	0.145	0.203
Ghana	Adaklu Anyigbe	2017	0.043	0.065	0.092
Ghana	Adaklu Anyigbe	2000-2017	-0.156	-0.061	0.041
Ghana	Adansi North	2000	0.088	0.137	0.201
Ghana	Adansi North	2017	0.045	0.069	0.101
Ghana	Adansi North	2000-2017	-0.136	-0.039	0.057
Ghana	Adansi South	2000	0.100	0.157	0.235
Ghana	Adansi South	2017	0.050	0.077	0.113
Ghana	Adansi South	2000-2017	-0.140	-0.043	0.053
Ghana	Afigya Sekyere	2000	0.094	0.149	0.217
Ghana	Afigya Sekyere	2017	0.047	0.074	0.112
Ghana	Afigya Sekyere	2000-2017	-0.144	-0.035	0.078
Ghana	Afram Plains	2000	0.116	0.171	0.245
Ghana	Afram Plains	2017	0.048	0.076	0.106
Ghana	Afram Plains	2000-2017	-0.147	-0.049	0.040
Ghana	Agona	2000	0.095	0.152	0.222
Ghana	Agona	2017	0.043	0.067	0.102
Ghana	Agona	2000-2017	-0.150	-0.051	0.042
Ghana	Ahafo Ano North	2000	0.103	0.154	0.222
Ghana	Ahafo Ano North	2017	0.051	0.078	0.115
Ghana	Ahafo Ano North	2000-2017	-0.129	-0.035	0.080
Ghana	Ahafo Ano South	2000	0.105	0.161	0.230
Ghana	Ahafo Ano South	2017	0.053	0.082	0.122
Ghana	Ahafo Ano South	2000-2017	-0.135	-0.037	0.080
Ghana	Ahanta West	2000	0.086	0.132	0.200

Table 2: Diarrhea DALYs rate by unit *(continued)*

Country	Unit	year	mean rate	lower rate	upper rate
Ghana	Ahanta West	2017	0.041	0.066	0.099
Ghana	Ahanta West	2000-2017	-0.153	-0.051	0.042
Ghana	Ajumako-Enyan-Esiam	2000	0.090	0.145	0.205
Ghana	Ajumako-Enyan-Esiam	2017	0.043	0.067	0.102
Ghana	Ajumako-Enyan-Esiam	2000-2017	-0.152	-0.051	0.042
Ghana	Akatsi	2000	0.101	0.150	0.210
Ghana	Akatsi	2017	0.047	0.069	0.096
Ghana	Akatsi	2000-2017	-0.171	-0.067	0.025
Ghana	Akwapim North	2000	0.089	0.138	0.200
Ghana	Akwapim North	2017	0.039	0.061	0.088
Ghana	Akwapim North	2000-2017	-0.153	-0.060	0.046
Ghana	Akwapim South	2000	0.090	0.143	0.213
Ghana	Akwapim South	2017	0.040	0.062	0.092
Ghana	Akwapim South	2000-2017	-0.152	-0.056	0.038
Ghana	Amansie Central	2000	0.093	0.140	0.208
Ghana	Amansie Central	2017	0.046	0.071	0.105
Ghana	Amansie Central	2000-2017	-0.137	-0.038	0.068
Ghana	Amansie East	2000	0.101	0.152	0.223
Ghana	Amansie East	2017	0.048	0.075	0.110
Ghana	Amansie East	2000-2017	-0.139	-0.040	0.070
Ghana	Amansie West	2000	0.098	0.147	0.221
Ghana	Amansie West	2017	0.050	0.076	0.115
Ghana	Amansie West	2000-2017	-0.135	-0.038	0.070
Ghana	Aowin-Suaman	2000	0.111	0.164	0.229
Ghana	Aowin-Suaman	2017	0.049	0.079	0.118
Ghana	Aowin-Suaman	2000-2017	-0.151	-0.054	0.043
Ghana	Asante Akim North	2000	0.092	0.143	0.204
Ghana	Asante Akim North	2017	0.044	0.069	0.105
Ghana	Asante Akim North	2000-2017	-0.135	-0.037	0.068
Ghana	Asante Akim South	2000	0.103	0.156	0.228
Ghana	Asante Akim South	2017	0.049	0.076	0.116
Ghana	Asante Akim South	2000-2017	-0.136	-0.039	0.070
Ghana	Asikuma Odoben Brakwa	2000	0.101	0.165	0.238
Ghana	Asikuma Odoben Brakwa	2017	0.050	0.076	0.116
Ghana	Asikuma Odoben Brakwa	2000-2017	-0.148	-0.049	0.042
Ghana	Assin North	2000	0.102	0.159	0.231
Ghana	Assin North	2017	0.052	0.078	0.115
Ghana	Assin North	2000-2017	-0.149	-0.045	0.047
Ghana	Assin South	2000	0.094	0.152	0.217
Ghana	Assin South	2017	0.049	0.074	0.110
Ghana	Assin South	2000-2017	-0.153	-0.047	0.044
Ghana	Asunafo North	2000	0.114	0.166	0.227
Ghana	Asunafo North	2017	0.059	0.088	0.128
Ghana	Asunafo North	2000-2017	-0.138	-0.036	0.066
Ghana	Asunafo South	2000	0.112	0.164	0.229
Ghana	Asunafo South	2017	0.058	0.087	0.128
Ghana	Asunafo South	2000-2017	-0.132	-0.035	0.063
Ghana	Asuogyaman	2000	0.089	0.140	0.206
Ghana	Asuogyaman	2017	0.042	0.066	0.095
Ghana	Asuogyaman	2000-2017	-0.149	-0.053	0.046
Ghana	Asutifi	2000	0.114	0.166	0.239
Ghana	Asutifi	2017	0.057	0.086	0.126
Ghana	Asutifi	2000-2017	-0.128	-0.035	0.075
Ghana	Atebubu-Amantin	2000	0.129	0.194	0.278
Ghana	Atebubu-Amantin	2017	0.050	0.081	0.123
Ghana	Atebubu-Amantin	2000-2017	-0.141	-0.043	0.057
Ghana	Atiwa	2000	0.100	0.153	0.219
Ghana	Atiwa	2017	0.047	0.074	0.111
Ghana	Atiwa	2000-2017	-0.138	-0.044	0.046
Ghana	Atwima	2000	0.092	0.141	0.213
Ghana	Atwima	2017	0.046	0.070	0.108

Table 2: Diarrhea DALYs rate by unit *(continued)*

Country	Unit	year	mean rate	lower rate	upper rate
Ghana	Atwima	2000-2017	-0.136	-0.038	0.077
Ghana	Atwima Mponua	2000	0.108	0.162	0.236
Ghana	Atwima Mponua	2017	0.056	0.084	0.130
Ghana	Atwima Mponua	2000-2017	-0.130	-0.036	0.069
Ghana	Awutu Efutu Senya	2000	0.091	0.144	0.207
Ghana	Awutu Efutu Senya	2017	0.041	0.064	0.100
Ghana	Awutu Efutu Senya	2000-2017	-0.152	-0.054	0.037
Ghana	Bawku Municipal	2000	0.179	0.255	0.355
Ghana	Bawku Municipal	2017	0.074	0.115	0.165
Ghana	Bawku Municipal	2000-2017	-0.169	-0.087	-0.010
Ghana	Bawku West	2000	0.178	0.254	0.355
Ghana	Bawku West	2017	0.074	0.118	0.171
Ghana	Bawku West	2000-2017	-0.166	-0.080	-0.001
Ghana	Berekum	2000	0.114	0.163	0.231
Ghana	Berekum	2017	0.053	0.082	0.120
Ghana	Berekum	2000-2017	-0.126	-0.035	0.062
Ghana	Bia	2000	0.109	0.157	0.207
Ghana	Bia	2017	0.055	0.083	0.122
Ghana	Bia	2000-2017	-0.146	-0.039	0.062
Ghana	Bibiani Anhwiaso Bekwai	2000	0.096	0.145	0.211
Ghana	Bibiani Anhwiaso Bekwai	2017	0.050	0.076	0.115
Ghana	Bibiani Anhwiaso Bekwai	2000-2017	-0.135	-0.037	0.063
Ghana	Birim North	2000	0.105	0.161	0.233
Ghana	Birim North	2017	0.050	0.077	0.113
Ghana	Birim North	2000-2017	-0.130	-0.044	0.056
Ghana	Birim South	2000	0.100	0.159	0.236
Ghana	Birim South	2017	0.048	0.073	0.108
Ghana	Birim South	2000-2017	-0.146	-0.048	0.048
Ghana	Bole	2000	0.160	0.222	0.308
Ghana	Bole	2017	0.061	0.098	0.151
Ghana	Bole	2000-2017	-0.130	-0.039	0.052
Ghana	Bolgatanga	2000	0.153	0.230	0.316
Ghana	Bolgatanga	2017	0.070	0.113	0.168
Ghana	Bolgatanga	2000-2017	-0.161	-0.069	0.013
Ghana	Bongo	2000	0.157	0.236	0.324
Ghana	Bongo	2017	0.072	0.114	0.169
Ghana	Bongo	2000-2017	-0.167	-0.074	0.007
Ghana	Bosomtwe-Kwanwoma	2000	0.091	0.139	0.209
Ghana	Bosomtwe-Kwanwoma	2017	0.044	0.069	0.105
Ghana	Bosomtwe-Kwanwoma	2000-2017	-0.133	-0.039	0.073
Ghana	Builsa	2000	0.165	0.239	0.328
Ghana	Builsa	2017	0.074	0.119	0.176
Ghana	Builsa	2000-2017	-0.147	-0.060	0.013
Ghana	Bunkpurugu Yunyoo	2000	0.194	0.271	0.365
Ghana	Bunkpurugu Yunyoo	2017	0.078	0.121	0.172
Ghana	Bunkpurugu Yunyoo	2000-2017	-0.157	-0.073	0.008
Ghana	Cape Coast	2000	0.087	0.137	0.204
Ghana	Cape Coast	2017	0.042	0.067	0.103
Ghana	Cape Coast	2000-2017	-0.156	-0.050	0.040
Ghana	Central Gonja	2000	0.162	0.236	0.330
Ghana	Central Gonja	2017	0.065	0.103	0.152
Ghana	Central Gonja	2000-2017	-0.143	-0.034	0.048
Ghana	Dangbe East	2000	0.094	0.147	0.214
Ghana	Dangbe East	2017	0.042	0.066	0.095
Ghana	Dangbe East	2000-2017	-0.154	-0.059	0.048
Ghana	Dangbe West	2000	0.089	0.135	0.188
Ghana	Dangbe West	2017	0.040	0.061	0.089
Ghana	Dangbe West	2000-2017	-0.159	-0.063	0.035
Ghana	Dormaa	2000	0.112	0.163	0.225
Ghana	Dormaa	2017	0.055	0.084	0.122
Ghana	Dormaa	2000-2017	-0.139	-0.039	0.065

Table 2: Diarrhea DALYs rate by unit *(continued)*

Country	Unit	year	mean rate	lower rate	upper rate
Ghana	East Akim	2000	0.095	0.150	0.219
Ghana	East Akim	2017	0.047	0.072	0.106
Ghana	East Akim	2000-2017	-0.140	-0.046	0.041
Ghana	East Gonja	2000	0.168	0.238	0.334
Ghana	East Gonja	2017	0.062	0.096	0.137
Ghana	East Gonja	2000-2017	-0.134	-0.042	0.031
Ghana	East Mamprusi	2000	0.182	0.259	0.354
Ghana	East Mamprusi	2017	0.073	0.121	0.176
Ghana	East Mamprusi	2000-2017	-0.151	-0.063	0.018
Ghana	Ejisu-Juabeng	2000	0.092	0.142	0.212
Ghana	Ejisu-Juabeng	2017	0.044	0.070	0.107
Ghana	Ejisu-Juabeng	2000-2017	-0.137	-0.037	0.078
Ghana	Ejura Sekyedumase	2000	0.097	0.153	0.226
Ghana	Ejura Sekyedumase	2017	0.046	0.072	0.110
Ghana	Ejura Sekyedumase	2000-2017	-0.138	-0.034	0.068
Ghana	Fanteakwa	2000	0.093	0.144	0.212
Ghana	Fanteakwa	2017	0.044	0.069	0.100
Ghana	Fanteakwa	2000-2017	-0.142	-0.047	0.055
Ghana	Ga East	2000	0.094	0.150	0.223
Ghana	Ga East	2017	0.049	0.075	0.113
Ghana	Ga East	2000-2017	-0.148	-0.051	0.044
Ghana	Ga West	2000	0.091	0.148	0.219
Ghana	Ga West	2017	0.043	0.066	0.101
Ghana	Ga West	2000-2017	-0.151	-0.054	0.041
Ghana	Garu Tempane	2000	0.182	0.256	0.344
Ghana	Garu Tempane	2017	0.073	0.116	0.166
Ghana	Garu Tempane	2000-2017	-0.162	-0.082	-0.003
Ghana	Gomoa	2000	0.092	0.146	0.208
Ghana	Gomoa	2017	0.043	0.065	0.102
Ghana	Gomoa	2000-2017	-0.153	-0.052	0.040
Ghana	Gushiegu	2000	0.183	0.261	0.362
Ghana	Gushiegu	2017	0.072	0.117	0.169
Ghana	Gushiegu	2000-2017	-0.153	-0.057	0.021
Ghana	Ho	2000	0.094	0.136	0.197
Ghana	Ho	2017	0.041	0.062	0.086
Ghana	Ho	2000-2017	-0.162	-0.057	0.045
Ghana	Hohoe	2000	0.099	0.146	0.211
Ghana	Hohoe	2017	0.041	0.064	0.090
Ghana	Hohoe	2000-2017	-0.153	-0.056	0.038
Ghana	Jaman North	2000	0.120	0.177	0.250
Ghana	Jaman North	2017	0.054	0.086	0.131
Ghana	Jaman North	2000-2017	-0.132	-0.039	0.060
Ghana	Jaman South	2000	0.121	0.175	0.241
Ghana	Jaman South	2017	0.055	0.086	0.130
Ghana	Jaman South	2000-2017	-0.136	-0.043	0.064
Ghana	Jasikan	2000	0.111	0.166	0.243
Ghana	Jasikan	2017	0.044	0.071	0.100
Ghana	Jasikan	2000-2017	-0.148	-0.055	0.038
Ghana	Jirapa Lambussie	2000	0.156	0.236	0.350
Ghana	Jirapa Lambussie	2017	0.071	0.112	0.177
Ghana	Jirapa Lambussie	2000-2017	-0.148	-0.064	0.016
Ghana	Jomoro	2000	0.116	0.165	0.232
Ghana	Jomoro	2017	0.049	0.079	0.118
Ghana	Jomoro	2000-2017	-0.157	-0.053	0.056
Ghana	Juabeso	2000	0.106	0.152	0.208
Ghana	Juabeso	2017	0.052	0.080	0.117
Ghana	Juabeso	2000-2017	-0.144	-0.042	0.056
Ghana	Kadjebi	2000	0.130	0.185	0.268
Ghana	Kadjebi	2017	0.049	0.076	0.110
Ghana	Kadjebi	2000-2017	-0.151	-0.055	0.043
Ghana	Karaga	2000	0.175	0.248	0.350

Table 2: Diarrhea DALYs rate by unit *(continued)*

Country	Unit	year	mean rate	lower rate	upper rate
Ghana	Karaga	2017	0.067	0.113	0.170
Ghana	Karaga	2000-2017	-0.141	-0.047	0.033
Ghana	Kassena Nankana	2000	0.159	0.233	0.324
Ghana	Kassena Nankana	2017	0.074	0.115	0.168
Ghana	Kassena Nankana	2000-2017	-0.158	-0.069	0.011
Ghana	Keta	2000	0.104	0.155	0.215
Ghana	Keta	2017	0.050	0.073	0.102
Ghana	Keta	2000-2017	-0.174	-0.068	0.031
Ghana	Ketu	2000	0.104	0.146	0.204
Ghana	Ketu	2017	0.046	0.065	0.091
Ghana	Ketu	2000-2017	-0.179	-0.071	0.016
Ghana	Kintampo North	2000	0.134	0.196	0.269
Ghana	Kintampo North	2017	0.058	0.090	0.139
Ghana	Kintampo North	2000-2017	-0.130	-0.033	0.052
Ghana	Kintampo South	2000	0.115	0.175	0.258
Ghana	Kintampo South	2017	0.054	0.083	0.130
Ghana	Kintampo South	2000-2017	-0.126	-0.032	0.058
Ghana	Komenda-Edina-Eguafo-Abirem	2000	0.085	0.134	0.200
Ghana	Komenda-Edina-Eguafo-Abirem	2017	0.040	0.065	0.101
Ghana	Komenda-Edina-Eguafo-Abirem	2000-2017	-0.157	-0.052	0.038
Ghana	Kpandu	2000	0.099	0.149	0.223
Ghana	Kpandu	2017	0.042	0.066	0.093
Ghana	Kpandu	2000-2017	-0.150	-0.053	0.045
Ghana	Krachi	2000	0.141	0.206	0.303
Ghana	Krachi	2017	0.050	0.082	0.120
Ghana	Krachi	2000-2017	-0.133	-0.045	0.042
Ghana	Krachi East	2000	0.136	0.194	0.285
Ghana	Krachi East	2017	0.050	0.081	0.114
Ghana	Krachi East	2000-2017	-0.148	-0.052	0.045
Ghana	Kumasi	2000	0.092	0.141	0.215
Ghana	Kumasi	2017	0.048	0.074	0.113
Ghana	Kumasi	2000-2017	-0.131	-0.036	0.077
Ghana	Kwabibirem	2000	0.101	0.157	0.228
Ghana	Kwabibirem	2017	0.049	0.075	0.112
Ghana	Kwabibirem	2000-2017	-0.134	-0.045	0.053
Ghana	Kwabre	2000	0.089	0.137	0.208
Ghana	Kwabre	2017	0.044	0.069	0.104
Ghana	Kwabre	2000-2017	-0.138	-0.037	0.076
Ghana	Kwahu South	2000	0.094	0.143	0.203
Ghana	Kwahu South	2017	0.045	0.069	0.106
Ghana	Kwahu South	2000-2017	-0.134	-0.041	0.052
Ghana	Kwahu West	2000	0.097	0.147	0.209
Ghana	Kwahu West	2017	0.047	0.072	0.112
Ghana	Kwahu West	2000-2017	-0.130	-0.040	0.055
Ghana	Lawra	2000	0.160	0.240	0.347
Ghana	Lawra	2017	0.072	0.113	0.175
Ghana	Lawra	2000-2017	-0.150	-0.066	0.016
Ghana	Lower Denkyira	2000	0.101	0.155	0.223
Ghana	Lower Denkyira	2017	0.050	0.077	0.114
Ghana	Lower Denkyira	2000-2017	-0.156	-0.045	0.046
Ghana	Manya Krobo	2000	0.086	0.135	0.201
Ghana	Manya Krobo	2017	0.041	0.064	0.092
Ghana	Manya Krobo	2000-2017	-0.151	-0.051	0.056
Ghana	Mfantsiman	2000	0.089	0.143	0.207
Ghana	Mfantsiman	2017	0.043	0.066	0.102
Ghana	Mfantsiman	2000-2017	-0.153	-0.053	0.036
Ghana	Mpohor Wassa East	2000	0.100	0.148	0.223
Ghana	Mpohor Wassa East	2017	0.045	0.071	0.104
Ghana	Mpohor Wassa East	2000-2017	-0.160	-0.050	0.045
Ghana	Nadowli	2000	0.165	0.244	0.349
Ghana	Nadowli	2017	0.068	0.113	0.174

Table 2: Diarrhea DALYs rate by unit *(continued)*

Country	Unit	year	mean rate	lower rate	upper rate
Ghana	Nadowli	2000-2017	-0.148	-0.059	0.015
Ghana	Nanumba North	2000	0.172	0.244	0.345
Ghana	Nanumba North	2017	0.062	0.097	0.141
Ghana	Nanumba North	2000-2017	-0.145	-0.045	0.031
Ghana	Nanumba South	2000	0.168	0.241	0.349
Ghana	Nanumba South	2017	0.058	0.092	0.137
Ghana	Nanumba South	2000-2017	-0.139	-0.047	0.032
Ghana	New Juaben	2000	0.089	0.141	0.207
Ghana	New Juaben	2017	0.043	0.065	0.096
Ghana	New Juaben	2000-2017	-0.146	-0.053	0.046
Ghana	Nkoranza	2000	0.110	0.168	0.252
Ghana	Nkoranza	2017	0.052	0.080	0.123
Ghana	Nkoranza	2000-2017	-0.133	-0.034	0.062
Ghana	Nkwanta	2000	0.144	0.206	0.308
Ghana	Nkwanta	2017	0.051	0.079	0.114
Ghana	Nkwanta	2000-2017	-0.149	-0.053	0.037
Ghana	North Tongu	2000	0.096	0.147	0.209
Ghana	North Tongu	2017	0.044	0.068	0.096
Ghana	North Tongu	2000-2017	-0.148	-0.060	0.042
Ghana	Nzema East	2000	0.104	0.154	0.222
Ghana	Nzema East	2017	0.046	0.074	0.110
Ghana	Nzema East	2000-2017	-0.162	-0.052	0.048
Ghana	Obuasi Municipal	2000	0.105	0.159	0.234
Ghana	Obuasi Municipal	2017	0.053	0.080	0.119
Ghana	Obuasi Municipal	2000-2017	-0.143	-0.041	0.056
Ghana	Offinso	2000	0.098	0.150	0.221
Ghana	Offinso	2017	0.049	0.075	0.112
Ghana	Offinso	2000-2017	-0.136	-0.035	0.078
Ghana	Pru	2000	0.144	0.213	0.300
Ghana	Pru	2017	0.057	0.088	0.130
Ghana	Pru	2000-2017	-0.135	-0.041	0.047
Ghana	Saboba Chereponi	2000	0.194	0.266	0.358
Ghana	Saboba Chereponi	2017	0.073	0.113	0.167
Ghana	Saboba Chereponi	2000-2017	-0.159	-0.065	0.015
Ghana	Savelugu Nanton	2000	0.162	0.233	0.332
Ghana	Savelugu Nanton	2017	0.068	0.111	0.168
Ghana	Savelugu Nanton	2000-2017	-0.132	-0.039	0.042
Ghana	Sawa-Tuna-Kalba	2000	0.170	0.240	0.339
Ghana	Sawa-Tuna-Kalba	2017	0.063	0.101	0.157
Ghana	Sawa-Tuna-Kalba	2000-2017	-0.143	-0.050	0.036
Ghana	Sefwi Wiawso	2000	0.103	0.151	0.210
Ghana	Sefwi Wiawso	2017	0.049	0.079	0.118
Ghana	Sefwi Wiawso	2000-2017	-0.141	-0.040	0.062
Ghana	Sekyere East	2000	0.099	0.153	0.216
Ghana	Sekyere East	2017	0.046	0.073	0.111
Ghana	Sekyere East	2000-2017	-0.137	-0.037	0.060
Ghana	Sekyere West	2000	0.092	0.146	0.211
Ghana	Sekyere West	2017	0.044	0.069	0.106
Ghana	Sekyere West	2000-2017	-0.140	-0.036	0.067
Ghana	Sene	2000	0.140	0.204	0.292
Ghana	Sene	2017	0.055	0.084	0.122
Ghana	Sene	2000-2017	-0.138	-0.045	0.037
Ghana	Shama Ahanta East	2000	0.089	0.134	0.205
Ghana	Shama Ahanta East	2017	0.042	0.067	0.100
Ghana	Shama Ahanta East	2000-2017	-0.153	-0.050	0.048
Ghana	Sissala East	2000	0.162	0.241	0.335
Ghana	Sissala East	2017	0.073	0.116	0.175
Ghana	Sissala East	2000-2017	-0.149	-0.060	0.015
Ghana	Sissala West	2000	0.161	0.242	0.352
Ghana	Sissala West	2017	0.072	0.115	0.179
Ghana	Sissala West	2000-2017	-0.150	-0.063	0.007

Table 2: Diarrhea DALYs rate by unit *(continued)*

Country	Unit	year	mean rate	lower rate	upper rate
Ghana	South Dayi	2000	0.092	0.137	0.198
Ghana	South Dayi	2017	0.041	0.063	0.088
Ghana	South Dayi	2000-2017	-0.152	-0.053	0.049
Ghana	South Tongu	2000	0.095	0.144	0.199
Ghana	South Tongu	2017	0.045	0.067	0.095
Ghana	South Tongu	2000-2017	-0.164	-0.065	0.038
Ghana	Suhum Kraboa Coaltar	2000	0.094	0.151	0.221
Ghana	Suhum Kraboa Coaltar	2017	0.044	0.067	0.101
Ghana	Suhum Kraboa Coaltar	2000-2017	-0.144	-0.053	0.034
Ghana	Sunyani	2000	0.111	0.163	0.244
Ghana	Sunyani	2017	0.054	0.083	0.120
Ghana	Sunyani	2000-2017	-0.125	-0.034	0.080
Ghana	Tain	2000	0.120	0.175	0.253
Ghana	Tain	2017	0.056	0.086	0.127
Ghana	Tain	2000-2017	-0.124	-0.033	0.069
Ghana	Talensi Nabdam	2000	0.164	0.241	0.331
Ghana	Talensi Nabdam	2017	0.071	0.117	0.172
Ghana	Talensi Nabdam	2000-2017	-0.158	-0.069	0.011
Ghana	Tamale	2000	0.145	0.209	0.301
Ghana	Tamale	2017	0.060	0.101	0.155
Ghana	Tamale	2000-2017	-0.128	-0.031	0.055
Ghana	Tano North	2000	0.107	0.164	0.244
Ghana	Tano North	2017	0.054	0.083	0.119
Ghana	Tano North	2000-2017	-0.127	-0.035	0.080
Ghana	Tano South	2000	0.107	0.163	0.240
Ghana	Tano South	2017	0.053	0.082	0.120
Ghana	Tano South	2000-2017	-0.132	-0.035	0.084
Ghana	Techiman	2000	0.108	0.164	0.236
Ghana	Techiman	2017	0.054	0.081	0.124
Ghana	Techiman	2000-2017	-0.126	-0.032	0.071
Ghana	Tema	2000	0.107	0.168	0.246
Ghana	Tema	2017	0.059	0.091	0.132
Ghana	Tema	2000-2017	-0.150	-0.051	0.050
Ghana	Tolon-Kumbungu	2000	0.165	0.235	0.341
Ghana	Tolon-Kumbungu	2017	0.065	0.107	0.164
Ghana	Tolon-Kumbungu	2000-2017	-0.134	-0.038	0.042
Ghana	Upper Denkyira	2000	0.103	0.159	0.233
Ghana	Upper Denkyira	2017	0.053	0.080	0.120
Ghana	Upper Denkyira	2000-2017	-0.146	-0.041	0.064
Ghana	Wa	2000	0.161	0.238	0.331
Ghana	Wa	2017	0.064	0.106	0.166
Ghana	Wa	2000-2017	-0.144	-0.053	0.026
Ghana	Wa East	2000	0.174	0.255	0.355
Ghana	Wa East	2017	0.074	0.117	0.180
Ghana	Wa East	2000-2017	-0.136	-0.049	0.029
Ghana	Wa West	2000	0.165	0.241	0.342
Ghana	Wa West	2017	0.065	0.107	0.158
Ghana	Wa West	2000-2017	-0.141	-0.055	0.023
Ghana	Wasa Amenfi East	2000	0.103	0.156	0.228
Ghana	Wasa Amenfi East	2017	0.050	0.077	0.116
Ghana	Wasa Amenfi East	2000-2017	-0.146	-0.043	0.067
Ghana	Wasa Amenfi West	2000	0.107	0.161	0.231
Ghana	Wasa Amenfi West	2017	0.050	0.078	0.115
Ghana	Wasa Amenfi West	2000-2017	-0.143	-0.046	0.065
Ghana	Wassa West	2000	0.103	0.157	0.230
Ghana	Wassa West	2017	0.048	0.075	0.108
Ghana	Wassa West	2000-2017	-0.157	-0.049	0.051
Ghana	West Akim	2000	0.094	0.152	0.218
Ghana	West Akim	2017	0.045	0.068	0.102
Ghana	West Akim	2000-2017	-0.143	-0.050	0.041
Ghana	West Gonja	2000	0.176	0.250	0.343

Table 2: Diarrhea DALYs rate by unit *(continued)*

Country	Unit	year	mean rate	lower rate	upper rate
Ghana	West Gonja	2017	0.069	0.110	0.167
Ghana	West Gonja	2000-2017	-0.135	-0.042	0.038
Ghana	West Mamprusi	2000	0.177	0.257	0.358
Ghana	West Mamprusi	2017	0.075	0.123	0.178
Ghana	West Mamprusi	2000-2017	-0.143	-0.056	0.017
Ghana	Yendi	2000	0.176	0.242	0.335
Ghana	Yendi	2017	0.066	0.103	0.152
Ghana	Yendi	2000-2017	-0.154	-0.047	0.038
Ghana	Yilo Krobo	2000	0.083	0.131	0.193
Ghana	Yilo Krobo	2017	0.040	0.062	0.090
Ghana	Yilo Krobo	2000-2017	-0.148	-0.052	0.052
Ghana	Zabzugu Tatale	2000	0.178	0.250	0.360
Ghana	Zabzugu Tatale	2017	0.064	0.096	0.141
Ghana	Zabzugu Tatale	2000-2017	-0.153	-0.056	0.031
Guinea	Beyla	2000	0.242	0.335	0.453
Guinea	Beyla	2017	0.077	0.129	0.191
Guinea	Beyla	2000-2017	-0.177	-0.077	0.011
Guinea	Boffa	2000	0.253	0.346	0.465
Guinea	Boffa	2017	0.104	0.146	0.215
Guinea	Boffa	2000-2017	-0.161	-0.063	0.027
Guinea	Boké	2000	0.258	0.355	0.466
Guinea	Boké	2017	0.108	0.153	0.222
Guinea	Boké	2000-2017	-0.163	-0.068	0.017
Guinea	Conakry	2000	0.204	0.287	0.394
Guinea	Conakry	2017	0.085	0.115	0.162
Guinea	Conakry	2000-2017	-0.161	-0.061	0.036
Guinea	Coyah	2000	0.238	0.331	0.452
Guinea	Coyah	2017	0.090	0.122	0.167
Guinea	Coyah	2000-2017	-0.158	-0.062	0.037
Guinea	Dabola	2000	0.262	0.382	0.539
Guinea	Dabola	2017	0.079	0.116	0.160
Guinea	Dabola	2000-2017	-0.146	-0.063	0.035
Guinea	Dalaba	2000	0.253	0.359	0.486
Guinea	Dalaba	2017	0.090	0.127	0.181
Guinea	Dalaba	2000-2017	-0.127	-0.042	0.061
Guinea	Dinguiraye	2000	0.264	0.390	0.554
Guinea	Dinguiraye	2017	0.087	0.131	0.191
Guinea	Dinguiraye	2000-2017	-0.127	-0.048	0.040
Guinea	Dubréka	2000	0.261	0.355	0.487
Guinea	Dubréka	2017	0.098	0.132	0.187
Guinea	Dubréka	2000-2017	-0.153	-0.061	0.034
Guinea	Faranah	2000	0.251	0.356	0.480
Guinea	Faranah	2017	0.072	0.106	0.135
Guinea	Faranah	2000-2017	-0.152	-0.071	0.017
Guinea	Forécariah	2000	0.257	0.350	0.470
Guinea	Forécariah	2017	0.089	0.118	0.154
Guinea	Forécariah	2000-2017	-0.146	-0.063	0.028
Guinea	Fria	2000	0.261	0.351	0.479
Guinea	Fria	2017	0.098	0.134	0.193
Guinea	Fria	2000-2017	-0.165	-0.059	0.046
Guinea	Gaoual	2000	0.279	0.367	0.475
Guinea	Gaoual	2017	0.103	0.146	0.212
Guinea	Gaoual	2000-2017	-0.145	-0.047	0.052
Guinea	Guéckédou	2000	0.249	0.345	0.486
Guinea	Guéckédou	2017	0.069	0.095	0.127
Guinea	Guéckédou	2000-2017	-0.176	-0.100	-0.025
Guinea	Kankan	2000	0.248	0.366	0.518
Guinea	Kankan	2017	0.080	0.122	0.171
Guinea	Kankan	2000-2017	-0.149	-0.060	0.030
Guinea	Kérouané	2000	0.265	0.390	0.537
Guinea	Kérouané	2017	0.076	0.120	0.167

Table 2: Diarrhea DALYs rate by unit *(continued)*

Country	Unit	year	mean rate	lower rate	upper rate
Guinea	Kérouané	2000-2017	-0.175	-0.082	0.006
Guinea	Kindia	2000	0.258	0.352	0.466
Guinea	Kindia	2017	0.091	0.119	0.167
Guinea	Kindia	2000-2017	-0.150	-0.056	0.036
Guinea	Kissidougou	2000	0.260	0.367	0.522
Guinea	Kissidougou	2017	0.073	0.106	0.138
Guinea	Kissidougou	2000-2017	-0.168	-0.086	0.007
Guinea	Koubia	2000	0.271	0.384	0.542
Guinea	Koubia	2017	0.089	0.130	0.185
Guinea	Koubia	2000-2017	-0.132	-0.042	0.059
Guinea	Koundara	2000	0.262	0.351	0.463
Guinea	Koundara	2017	0.107	0.149	0.206
Guinea	Koundara	2000-2017	-0.146	-0.040	0.058
Guinea	Kouroussa	2000	0.268	0.386	0.543
Guinea	Kouroussa	2017	0.084	0.122	0.165
Guinea	Kouroussa	2000-2017	-0.151	-0.060	0.027
Guinea	Labé	2000	0.248	0.353	0.479
Guinea	Labé	2017	0.086	0.123	0.177
Guinea	Labé	2000-2017	-0.135	-0.043	0.061
Guinea	Lélouma	2000	0.269	0.373	0.498
Guinea	Lélouma	2017	0.093	0.130	0.185
Guinea	Lélouma	2000-2017	-0.139	-0.046	0.060
Guinea	Lola	2000	0.243	0.344	0.466
Guinea	Lola	2017	0.090	0.137	0.205
Guinea	Lola	2000-2017	-0.167	-0.079	0.001
Guinea	Macenta	2000	0.235	0.327	0.455
Guinea	Macenta	2017	0.081	0.118	0.165
Guinea	Macenta	2000-2017	-0.171	-0.084	-0.008
Guinea	Mali	2000	0.279	0.387	0.522
Guinea	Mali	2017	0.096	0.133	0.183
Guinea	Mali	2000-2017	-0.139	-0.045	0.059
Guinea	Mamou	2000	0.262	0.364	0.502
Guinea	Mamou	2017	0.082	0.117	0.163
Guinea	Mamou	2000-2017	-0.136	-0.055	0.032
Guinea	Mandiana	2000	0.239	0.367	0.526
Guinea	Mandiana	2017	0.089	0.137	0.205
Guinea	Mandiana	2000-2017	-0.129	-0.042	0.050
Guinea	Nzérékoré	2000	0.237	0.336	0.446
Guinea	Nzérékoré	2017	0.089	0.135	0.196
Guinea	Nzérékoré	2000-2017	-0.154	-0.073	0.013
Guinea	Pita	2000	0.259	0.357	0.481
Guinea	Pita	2017	0.090	0.127	0.183
Guinea	Pita	2000-2017	-0.136	-0.044	0.056
Guinea	Siguiri	2000	0.276	0.407	0.585
Guinea	Siguiri	2017	0.087	0.141	0.207
Guinea	Siguiri	2000-2017	-0.132	-0.044	0.046
Guinea	Télimélé	2000	0.277	0.372	0.490
Guinea	Télimélé	2017	0.101	0.141	0.207
Guinea	Télimélé	2000-2017	-0.157	-0.050	0.044
Guinea	Tougué	2000	0.263	0.369	0.513
Guinea	Tougué	2017	0.090	0.128	0.186
Guinea	Tougué	2000-2017	-0.126	-0.041	0.060
Guinea	Yamou	2000	0.235	0.333	0.459
Guinea	Yamou	2017	0.085	0.126	0.176
Guinea	Yamou	2000-2017	-0.152	-0.073	0.012
Guinea-Bissau	Bafata	2000	0.292	0.383	0.482
Guinea-Bissau	Bafata	2017	0.131	0.189	0.259
Guinea-Bissau	Bafata	2000-2017	-0.178	-0.068	0.021
Guinea-Bissau	Bambadinca	2000	0.294	0.394	0.496
Guinea-Bissau	Bambadinca	2017	0.138	0.197	0.280
Guinea-Bissau	Bambadinca	2000-2017	-0.183	-0.078	0.008

Table 2: Diarrhea DALYs rate by unit *(continued)*

Country	Unit	year	mean rate	lower rate	upper rate
Guinea-Bissau	Bedanda	2000	0.283	0.378	0.490
Guinea-Bissau	Bedanda	2017	0.146	0.215	0.304
Guinea-Bissau	Bedanda	2000-2017	-0.160	-0.062	0.029
Guinea-Bissau	Bigene	2000	0.376	0.477	0.612
Guinea-Bissau	Bigene	2017	0.170	0.236	0.326
Guinea-Bissau	Bigene	2000-2017	-0.196	-0.107	-0.019
Guinea-Bissau	Bissau	2000	0.331	0.424	0.544
Guinea-Bissau	Bissau	2017	0.153	0.213	0.296
Guinea-Bissau	Bissau	2000-2017	-0.198	-0.102	-0.014
Guinea-Bissau	Bissora	2000	0.413	0.520	0.672
Guinea-Bissau	Bissora	2017	0.172	0.236	0.326
Guinea-Bissau	Bissora	2000-2017	-0.181	-0.090	-0.002
Guinea-Bissau	Boe	2000	0.258	0.342	0.440
Guinea-Bissau	Boe	2017	0.112	0.166	0.240
Guinea-Bissau	Boe	2000-2017	-0.150	-0.048	0.048
Guinea-Bissau	Bolama	2000	0.339	0.447	0.582
Guinea-Bissau	Bolama	2017	0.161	0.238	0.333
Guinea-Bissau	Bolama	2000-2017	-0.160	-0.065	0.024
Guinea-Bissau	Buba	2000	0.275	0.363	0.464
Guinea-Bissau	Buba	2017	0.149	0.214	0.306
Guinea-Bissau	Buba	2000-2017	-0.161	-0.064	0.031
Guinea-Bissau	Bubaque	2000	0.355	0.475	0.625
Guinea-Bissau	Bubaque	2017	0.150	0.235	0.336
Guinea-Bissau	Bubaque	2000-2017	-0.178	-0.080	0.010
Guinea-Bissau	Bula	2000	0.379	0.478	0.601
Guinea-Bissau	Bula	2017	0.173	0.239	0.335
Guinea-Bissau	Bula	2000-2017	-0.195	-0.100	-0.020
Guinea-Bissau	Cacheu	2000	0.353	0.453	0.581
Guinea-Bissau	Cacheu	2017	0.186	0.260	0.360
Guinea-Bissau	Cacheu	2000-2017	-0.177	-0.094	-0.012
Guinea-Bissau	Cacine	2000	0.291	0.399	0.513
Guinea-Bissau	Cacine	2017	0.144	0.210	0.305
Guinea-Bissau	Cacine	2000-2017	-0.169	-0.075	0.010
Guinea-Bissau	Caio	2000	0.344	0.445	0.582
Guinea-Bissau	Caio	2017	0.167	0.244	0.340
Guinea-Bissau	Caio	2000-2017	-0.171	-0.082	0.000
Guinea-Bissau	Canghungo	2000	0.368	0.471	0.606
Guinea-Bissau	Canghungo	2017	0.174	0.243	0.337
Guinea-Bissau	Canghungo	2000-2017	-0.185	-0.098	-0.017
Guinea-Bissau	Caravela	2000	0.357	0.469	0.618
Guinea-Bissau	Caravela	2017	0.157	0.246	0.348
Guinea-Bissau	Caravela	2000-2017	-0.179	-0.085	0.001
Guinea-Bissau	Catio	2000	0.302	0.403	0.520
Guinea-Bissau	Catio	2017	0.155	0.227	0.324
Guinea-Bissau	Catio	2000-2017	-0.158	-0.067	0.026
Guinea-Bissau	Contuboel	2000	0.344	0.447	0.566
Guinea-Bissau	Contuboel	2017	0.140	0.201	0.278
Guinea-Bissau	Contuboel	2000-2017	-0.186	-0.077	0.017
Guinea-Bissau	Empada	2000	0.283	0.382	0.490
Guinea-Bissau	Empada	2017	0.154	0.228	0.318
Guinea-Bissau	Empada	2000-2017	-0.152	-0.059	0.034
Guinea-Bissau	Farim	2000	0.404	0.512	0.662
Guinea-Bissau	Farim	2017	0.159	0.224	0.315
Guinea-Bissau	Farim	2000-2017	-0.176	-0.087	0.005
Guinea-Bissau	Fulacunda	2000	0.287	0.379	0.484
Guinea-Bissau	Fulacunda	2017	0.159	0.223	0.318
Guinea-Bissau	Fulacunda	2000-2017	-0.152	-0.057	0.036
Guinea-Bissau	Gabu	2000	0.273	0.360	0.460
Guinea-Bissau	Gabu	2017	0.119	0.174	0.249
Guinea-Bissau	Gabu	2000-2017	-0.162	-0.060	0.030
Guinea-Bissau	Galomaro	2000	0.282	0.374	0.465

Table 2: Diarrhea DALYs rate by unit *(continued)*

Country	Unit	year	mean rate	lower rate	upper rate
Guinea-Bissau	Galomaro	2017	0.130	0.187	0.261
Guinea-Bissau	Galomaro	2000-2017	-0.175	-0.068	0.021
Guinea-Bissau	Gamamundo	2000	0.326	0.432	0.546
Guinea-Bissau	Gamamundo	2017	0.139	0.198	0.276
Guinea-Bissau	Gamamundo	2000-2017	-0.186	-0.079	0.007
Guinea-Bissau	Mansaba	2000	0.404	0.522	0.662
Guinea-Bissau	Mansaba	2017	0.159	0.219	0.297
Guinea-Bissau	Mansaba	2000-2017	-0.180	-0.087	0.005
Guinea-Bissau	Mansoa	2000	0.374	0.478	0.599
Guinea-Bissau	Mansoa	2017	0.161	0.220	0.310
Guinea-Bissau	Mansoa	2000-2017	-0.173	-0.082	0.010
Guinea-Bissau	Nhacra	2000	0.360	0.458	0.585
Guinea-Bissau	Nhacra	2017	0.168	0.235	0.326
Guinea-Bissau	Nhacra	2000-2017	-0.185	-0.088	0.002
Guinea-Bissau	Piche	2000	0.259	0.347	0.453
Guinea-Bissau	Piche	2017	0.115	0.165	0.233
Guinea-Bissau	Piche	2000-2017	-0.137	-0.044	0.048
Guinea-Bissau	Pirada	2000	0.302	0.388	0.497
Guinea-Bissau	Pirada	2017	0.121	0.178	0.249
Guinea-Bissau	Pirada	2000-2017	-0.156	-0.061	0.031
Guinea-Bissau	Prabis	2000	0.349	0.446	0.570
Guinea-Bissau	Prabis	2017	0.173	0.243	0.342
Guinea-Bissau	Prabis	2000-2017	-0.183	-0.086	-0.002
Guinea-Bissau	Quebo	2000	0.263	0.348	0.448
Guinea-Bissau	Quebo	2017	0.133	0.193	0.275
Guinea-Bissau	Quebo	2000-2017	-0.156	-0.063	0.026
Guinea-Bissau	Quinhamel	2000	0.365	0.463	0.591
Guinea-Bissau	Quinhamel	2017	0.176	0.246	0.350
Guinea-Bissau	Quinhamel	2000-2017	-0.181	-0.085	-0.002
Guinea-Bissau	Safim	2000	0.330	0.423	0.543
Guinea-Bissau	Safim	2017	0.163	0.227	0.317
Guinea-Bissau	Safim	2000-2017	-0.193	-0.096	-0.010
Guinea-Bissau	Sao Domingos	2000	0.320	0.410	0.524
Guinea-Bissau	Sao Domingos	2017	0.178	0.257	0.364
Guinea-Bissau	Sao Domingos	2000-2017	-0.174	-0.092	-0.002
Guinea-Bissau	Sonaco	2000	0.309	0.399	0.509
Guinea-Bissau	Sonaco	2017	0.125	0.182	0.253
Guinea-Bissau	Sonaco	2000-2017	-0.179	-0.071	0.023
Guinea-Bissau	Tite	2000	0.310	0.402	0.515
Guinea-Bissau	Tite	2017	0.166	0.236	0.332
Guinea-Bissau	Tite	2000-2017	-0.154	-0.058	0.037
Guinea-Bissau	Xitole	2000	0.276	0.367	0.464
Guinea-Bissau	Xitole	2017	0.134	0.192	0.274
Guinea-Bissau	Xitole	2000-2017	-0.168	-0.073	0.018
Kenya	Ainabkoi	2000	0.138	0.172	0.219
Kenya	Ainabkoi	2017	0.096	0.125	0.173
Kenya	Ainabkoi	2000-2017	-0.041	-0.014	0.021
Kenya	Ainamoi	2000	0.075	0.096	0.125
Kenya	Ainamoi	2017	0.089	0.115	0.155
Kenya	Ainamoi	2000-2017	-0.012	0.020	0.055
Kenya	Aldai	2000	0.114	0.142	0.182
Kenya	Aldai	2017	0.078	0.099	0.130
Kenya	Aldai	2000-2017	-0.046	-0.015	0.015
Kenya	Alego Usonga	2000	0.273	0.340	0.410
Kenya	Alego Usonga	2017	0.123	0.155	0.195
Kenya	Alego Usonga	2000-2017	-0.066	-0.039	-0.011
Kenya	Awendo	2000	0.629	0.817	1.026
Kenya	Awendo	2017	0.200	0.253	0.331
Kenya	Awendo	2000-2017	-0.099	-0.072	-0.043
Kenya	Bahati	2000	0.103	0.133	0.169
Kenya	Bahati	2017	0.079	0.103	0.135

Table 2: Diarrhea DALYs rate by unit *(continued)*

Country	Unit	year	mean rate	lower rate	upper rate
Kenya	Bahati	2000-2017	-0.063	-0.031	-0.001
Kenya	Balambala	2000	0.275	0.384	0.524
Kenya	Balambala	2017	0.079	0.105	0.136
Kenya	Balambala	2000-2017	-0.101	-0.071	-0.043
Kenya	Banissa	2000	0.167	0.232	0.318
Kenya	Banissa	2017	0.092	0.128	0.173
Kenya	Banissa	2000-2017	-0.044	-0.013	0.019
Kenya	Baringo Central	2000	0.291	0.366	0.466
Kenya	Baringo Central	2017	0.103	0.132	0.182
Kenya	Baringo Central	2000-2017	-0.101	-0.073	-0.039
Kenya	Baringo North	2000	0.294	0.374	0.468
Kenya	Baringo North	2017	0.101	0.130	0.175
Kenya	Baringo North	2000-2017	-0.104	-0.076	-0.045
Kenya	Baringo South	2000	0.280	0.363	0.464
Kenya	Baringo South	2017	0.100	0.131	0.175
Kenya	Baringo South	2000-2017	-0.102	-0.072	-0.038
Kenya	Belgut	2000	0.076	0.096	0.123
Kenya	Belgut	2017	0.091	0.118	0.154
Kenya	Belgut	2000-2017	-0.009	0.022	0.057
Kenya	Bobasi	2000	0.245	0.309	0.380
Kenya	Bobasi	2017	0.132	0.175	0.231
Kenya	Bobasi	2000-2017	-0.059	-0.030	0.000
Kenya	Bomachoge Borabu	2000	0.242	0.306	0.383
Kenya	Bomachoge Borabu	2017	0.136	0.178	0.237
Kenya	Bomachoge Borabu	2000-2017	-0.058	-0.030	0.000
Kenya	Bomachoge Chache	2000	0.254	0.320	0.400
Kenya	Bomachoge Chache	2017	0.138	0.181	0.238
Kenya	Bomachoge Chache	2000-2017	-0.060	-0.033	-0.002
Kenya	Bomet Central	2000	0.052	0.067	0.084
Kenya	Bomet Central	2017	0.044	0.058	0.075
Kenya	Bomet Central	2000-2017	-0.007	0.024	0.058
Kenya	Bomet East	2000	0.053	0.068	0.087
Kenya	Bomet East	2017	0.045	0.060	0.077
Kenya	Bomet East	2000-2017	-0.002	0.027	0.064
Kenya	Bonchari	2000	0.269	0.340	0.427
Kenya	Bonchari	2017	0.139	0.180	0.236
Kenya	Bonchari	2000-2017	-0.062	-0.035	-0.006
Kenya	Bondo	2000	0.281	0.353	0.430
Kenya	Bondo	2017	0.126	0.159	0.202
Kenya	Bondo	2000-2017	-0.069	-0.044	-0.014
Kenya	Borabu	2000	0.189	0.238	0.294
Kenya	Borabu	2017	0.053	0.069	0.093
Kenya	Borabu	2000-2017	-0.090	-0.062	-0.029
Kenya	Budalangi	2000	0.239	0.298	0.368
Kenya	Budalangi	2017	0.142	0.178	0.225
Kenya	Budalangi	2000-2017	-0.041	-0.015	0.013
Kenya	Bumula	2000	0.137	0.174	0.214
Kenya	Bumula	2017	0.159	0.197	0.251
Kenya	Bumula	2000-2017	-0.014	0.015	0.046
Kenya	Bura	2000	0.208	0.284	0.378
Kenya	Bura	2017	0.102	0.134	0.172
Kenya	Bura	2000-2017	-0.067	-0.039	-0.009
Kenya	Bureti	2000	0.076	0.096	0.120
Kenya	Bureti	2017	0.093	0.120	0.156
Kenya	Bureti	2000-2017	-0.008	0.023	0.056
Kenya	Butere	2000	0.329	0.411	0.501
Kenya	Butere	2017	0.150	0.191	0.244
Kenya	Butere	2000-2017	-0.060	-0.034	-0.006
Kenya	Butula	2000	0.220	0.278	0.345
Kenya	Butula	2017	0.132	0.166	0.207
Kenya	Butula	2000-2017	-0.042	-0.016	0.015

Table 2: Diarrhea DALYs rate by unit *(continued)*

Country	Unit	year	mean rate	lower rate	upper rate
Kenya	Buuri	2000	0.127	0.169	0.216
Kenya	Buuri	2017	0.152	0.202	0.259
Kenya	Buuri	2000-2017	-0.013	0.017	0.048
Kenya	Central Imenti	2000	0.132	0.182	0.234
Kenya	Central Imenti	2017	0.156	0.213	0.274
Kenya	Central Imenti	2000-2017	-0.012	0.017	0.051
Kenya	Changamwe	2000	0.131	0.174	0.227
Kenya	Changamwe	2017	0.099	0.131	0.168
Kenya	Changamwe	2000-2017	-0.037	-0.004	0.030
Kenya	Chepalungu	2000	0.053	0.068	0.087
Kenya	Chepalungu	2017	0.046	0.061	0.078
Kenya	Chepalungu	2000-2017	-0.001	0.028	0.063
Kenya	Cherangany	2000	0.124	0.159	0.206
Kenya	Cherangany	2017	0.097	0.124	0.161
Kenya	Cherangany	2000-2017	-0.031	0.000	0.031
Kenya	Chesumei	2000	0.107	0.131	0.169
Kenya	Chesumei	2017	0.066	0.085	0.115
Kenya	Chesumei	2000-2017	-0.053	-0.025	0.007
Kenya	Chuka/Igambang'Ombe	2000	0.635	0.868	1.103
Kenya	Chuka/Igambang'Ombe	2017	0.280	0.374	0.482
Kenya	Chuka/Igambang'Ombe	2000-2017	-0.076	-0.046	-0.015
Kenya	Daadab	2000	0.265	0.388	0.551
Kenya	Daadab	2017	0.071	0.096	0.130
Kenya	Daadab	2000-2017	-0.114	-0.080	-0.049
Kenya	Dagoretti North	2000	0.093	0.121	0.153
Kenya	Dagoretti North	2017	0.023	0.031	0.040
Kenya	Dagoretti North	2000-2017	-0.113	-0.084	-0.053
Kenya	Dagoretti South	2000	0.095	0.124	0.157
Kenya	Dagoretti South	2017	0.023	0.031	0.039
Kenya	Dagoretti South	2000-2017	-0.115	-0.084	-0.052
Kenya	Eldama Ravine	2000	0.285	0.361	0.460
Kenya	Eldama Ravine	2017	0.105	0.139	0.189
Kenya	Eldama Ravine	2000-2017	-0.102	-0.072	-0.034
Kenya	Eldas	2000	0.417	0.573	0.769
Kenya	Eldas	2017	0.163	0.220	0.289
Kenya	Eldas	2000-2017	-0.088	-0.053	-0.022
Kenya	Embakasi Central	2000	0.087	0.115	0.148
Kenya	Embakasi Central	2017	0.027	0.037	0.047
Kenya	Embakasi Central	2000-2017	-0.099	-0.070	-0.037
Kenya	Embakasi East	2000	0.082	0.108	0.140
Kenya	Embakasi East	2017	0.022	0.029	0.038
Kenya	Embakasi East	2000-2017	-0.109	-0.081	-0.047
Kenya	Embakasi North	2000	0.089	0.117	0.150
Kenya	Embakasi North	2017	0.027	0.035	0.045
Kenya	Embakasi North	2000-2017	-0.101	-0.072	-0.038
Kenya	Embakasi South	2000	0.083	0.110	0.140
Kenya	Embakasi South	2017	0.021	0.029	0.038
Kenya	Embakasi South	2000-2017	-0.109	-0.081	-0.047
Kenya	Embakasi West	2000	0.089	0.117	0.151
Kenya	Embakasi West	2017	0.029	0.039	0.050
Kenya	Embakasi West	2000-2017	-0.097	-0.068	-0.034
Kenya	Emgwen	2000	0.109	0.134	0.173
Kenya	Emgwen	2017	0.072	0.094	0.126
Kenya	Emgwen	2000-2017	-0.051	-0.022	0.011
Kenya	Emuhaya	2000	0.234	0.290	0.353
Kenya	Emuhaya	2017	0.053	0.068	0.087
Kenya	Emuhaya	2000-2017	-0.118	-0.090	-0.062
Kenya	Emurua Dikirr	2000	0.152	0.196	0.244
Kenya	Emurua Dikirr	2017	0.038	0.049	0.065
Kenya	Emurua Dikirr	2000-2017	-0.113	-0.084	-0.049
Kenya	Endebess	2000	0.127	0.162	0.203

Table 2: Diarrhea DALYs rate by unit *(continued)*

Country	Unit	year	mean rate	lower rate	upper rate
Kenya	Endebess	2017	0.104	0.132	0.166
Kenya	Endebess	2000-2017	-0.033	-0.001	0.030
Kenya	Fafi	2000	0.292	0.411	0.559
Kenya	Fafi	2017	0.077	0.102	0.134
Kenya	Fafi	2000-2017	-0.101	-0.071	-0.041
Kenya	Funyula	2000	0.218	0.280	0.348
Kenya	Funyula	2017	0.134	0.172	0.215
Kenya	Funyula	2000-2017	-0.039	-0.014	0.015
Kenya	Galole	2000	0.221	0.294	0.396
Kenya	Galole	2017	0.103	0.137	0.179
Kenya	Galole	2000-2017	-0.068	-0.040	-0.010
Kenya	Ganze	2000	0.351	0.468	0.599
Kenya	Ganze	2017	0.164	0.213	0.272
Kenya	Ganze	2000-2017	-0.073	-0.044	-0.010
Kenya	Garissa Township	2000	0.268	0.376	0.516
Kenya	Garissa Township	2017	0.073	0.097	0.128
Kenya	Garissa Township	2000-2017	-0.102	-0.070	-0.038
Kenya	Garsen	2000	0.204	0.278	0.361
Kenya	Garsen	2017	0.129	0.170	0.222
Kenya	Garsen	2000-2017	-0.052	-0.024	0.005
Kenya	Gatanga	2000	0.027	0.036	0.046
Kenya	Gatanga	2017	0.023	0.030	0.039
Kenya	Gatanga	2000-2017	-0.020	0.007	0.040
Kenya	Gatundu North	2000	0.103	0.132	0.166
Kenya	Gatundu North	2017	0.077	0.101	0.130
Kenya	Gatundu North	2000-2017	-0.041	-0.014	0.017
Kenya	Gatundu South	2000	0.101	0.129	0.164
Kenya	Gatundu South	2017	0.077	0.100	0.130
Kenya	Gatundu South	2000-2017	-0.043	-0.014	0.018
Kenya	Gem	2000	0.264	0.329	0.391
Kenya	Gem	2017	0.121	0.154	0.198
Kenya	Gem	2000-2017	-0.065	-0.037	-0.008
Kenya	Gichugu	2000	0.163	0.224	0.285
Kenya	Gichugu	2017	0.131	0.173	0.223
Kenya	Gichugu	2000-2017	-0.057	-0.025	0.010
Kenya	Gilgil	2000	0.095	0.123	0.158
Kenya	Gilgil	2017	0.076	0.099	0.127
Kenya	Gilgil	2000-2017	-0.056	-0.026	0.004
Kenya	Githunguri	2000	0.097	0.126	0.161
Kenya	Githunguri	2017	0.076	0.100	0.130
Kenya	Githunguri	2000-2017	-0.043	-0.014	0.019
Kenya	Hamisi	2000	0.217	0.269	0.339
Kenya	Hamisi	2017	0.050	0.064	0.082
Kenya	Hamisi	2000-2017	-0.119	-0.089	-0.061
Kenya	Homa Bay Town	2000	0.452	0.562	0.706
Kenya	Homa Bay Town	2017	0.165	0.209	0.265
Kenya	Homa Bay Town	2000-2017	-0.087	-0.061	-0.034
Kenya	Igembe Central	2000	0.151	0.203	0.259
Kenya	Igembe Central	2017	0.153	0.210	0.268
Kenya	Igembe Central	2000-2017	-0.023	0.006	0.040
Kenya	Igembe North	2000	0.153	0.203	0.263
Kenya	Igembe North	2017	0.153	0.208	0.266
Kenya	Igembe North	2000-2017	-0.022	0.008	0.042
Kenya	Igembe South	2000	0.740	1.020	1.317
Kenya	Igembe South	2000	0.154	0.209	0.268
Kenya	Igembe South	2017	0.275	0.377	0.480
Kenya	Igembe South	2017	0.153	0.211	0.267
Kenya	Igembe South	2000-2017	-0.095	-0.065	-0.033
Kenya	Igembe South	2000-2017	-0.024	0.004	0.038
Kenya	Ijara	2000	0.323	0.428	0.565
Kenya	Ijara	2017	0.089	0.117	0.155

Table 2: Diarrhea DALYs rate by unit *(continued)*

Country	Unit	year	mean rate	lower rate	upper rate
Kenya	Ijara	2000-2017	-0.097	-0.070	-0.039
Kenya	Ikolomani	2000	0.317	0.395	0.483
Kenya	Ikolomani	2017	0.140	0.179	0.233
Kenya	Ikolomani	2000-2017	-0.063	-0.034	-0.005
Kenya	Isiolo North	2000	0.246	0.314	0.390
Kenya	Isiolo North	2017	0.080	0.104	0.128
Kenya	Isiolo North	2000-2017	-0.079	-0.053	-0.021
Kenya	Isiolo South	2000	0.247	0.331	0.430
Kenya	Isiolo South	2017	0.080	0.105	0.136
Kenya	Isiolo South	2000-2017	-0.082	-0.056	-0.025
Kenya	Jomvu	2000	0.132	0.175	0.230
Kenya	Jomvu	2017	0.099	0.131	0.169
Kenya	Jomvu	2000-2017	-0.037	-0.004	0.030
Kenya	Juja	2000	0.105	0.135	0.175
Kenya	Juja	2017	0.078	0.102	0.135
Kenya	Juja	2000-2017	-0.043	-0.016	0.020
Kenya	Kabete	2000	0.097	0.126	0.159
Kenya	Kabete	2017	0.080	0.105	0.136
Kenya	Kabete	2000-2017	-0.042	-0.011	0.021
Kenya	Kabondo Kasipul	2000	0.415	0.520	0.636
Kenya	Kabondo Kasipul	2017	0.145	0.184	0.235
Kenya	Kabondo Kasipul	2000-2017	-0.081	-0.054	-0.026
Kenya	Kabuchai	2000	0.131	0.164	0.205
Kenya	Kabuchai	2017	0.146	0.183	0.233
Kenya	Kabuchai	2000-2017	-0.015	0.012	0.044
Kenya	Kacheliba	2000	0.328	0.413	0.543
Kenya	Kacheliba	2017	0.160	0.210	0.264
Kenya	Kacheliba	2000-2017	-0.051	-0.022	0.007
Kenya	Kaiti	2000	0.221	0.289	0.377
Kenya	Kaiti	2017	0.032	0.042	0.053
Kenya	Kaiti	2000-2017	-0.161	-0.133	-0.102
Kenya	Kajiado Central	2000	0.086	0.116	0.153
Kenya	Kajiado Central	2017	0.042	0.057	0.075
Kenya	Kajiado Central	2000-2017	-0.080	-0.047	-0.013
Kenya	Kajiado East	2000	0.086	0.111	0.141
Kenya	Kajiado East	2017	0.040	0.055	0.072
Kenya	Kajiado East	2000-2017	-0.075	-0.047	-0.014
Kenya	Kajiado North	2000	0.079	0.104	0.133
Kenya	Kajiado North	2017	0.041	0.056	0.073
Kenya	Kajiado North	2000-2017	-0.070	-0.039	-0.006
Kenya	Kajiado South	2000	0.082	0.113	0.159
Kenya	Kajiado South	2017	0.040	0.054	0.071
Kenya	Kajiado South	2000-2017	-0.086	-0.052	-0.021
Kenya	Kajiado West	2000	0.091	0.119	0.154
Kenya	Kajiado West	2017	0.045	0.062	0.081
Kenya	Kajiado West	2000-2017	-0.070	-0.039	-0.007
Kenya	Kaloleni	2000	0.343	0.455	0.594
Kenya	Kaloleni	2017	0.151	0.192	0.251
Kenya	Kaloleni	2000-2017	-0.079	-0.047	-0.015
Kenya	Kamukunji	2000	0.101	0.131	0.169
Kenya	Kamukunji	2017	0.033	0.044	0.057
Kenya	Kamukunji	2000-2017	-0.104	-0.075	-0.042
Kenya	Kandara	2000	0.028	0.037	0.047
Kenya	Kandara	2017	0.024	0.032	0.041
Kenya	Kandara	2000-2017	-0.020	0.007	0.041
Kenya	Kanduyi	2000	0.134	0.169	0.211
Kenya	Kanduyi	2017	0.152	0.190	0.246
Kenya	Kanduyi	2000-2017	-0.013	0.015	0.047
Kenya	Kangema	2000	0.027	0.037	0.047
Kenya	Kangema	2017	0.025	0.032	0.041
Kenya	Kangema	2000-2017	-0.024	0.007	0.038

Table 2: Diarrhea DALYs rate by unit *(continued)*

Country	Unit	year	mean rate	lower rate	upper rate
Kenya	Kangundo	2000	0.156	0.207	0.267
Kenya	Kangundo	2017	0.150	0.199	0.259
Kenya	Kangundo	2000-2017	-0.034	-0.006	0.029
Kenya	Kapenguria	2000	0.313	0.396	0.508
Kenya	Kapenguria	2017	0.162	0.204	0.259
Kenya	Kapenguria	2000-2017	-0.059	-0.028	0.001
Kenya	Kapseret	2000	0.141	0.175	0.227
Kenya	Kapseret	2017	0.100	0.129	0.176
Kenya	Kapseret	2000-2017	-0.043	-0.014	0.018
Kenya	Karachuonyo	2000	0.477	0.592	0.717
Kenya	Karachuonyo	2017	0.165	0.208	0.262
Kenya	Karachuonyo	2000-2017	-0.084	-0.057	-0.030
Kenya	Kasarani	2000	0.087	0.114	0.147
Kenya	Kasarani	2017	0.022	0.030	0.039
Kenya	Kasarani	2000-2017	-0.109	-0.080	-0.046
Kenya	Kasipul	2000	0.460	0.569	0.691
Kenya	Kasipul	2017	0.157	0.200	0.255
Kenya	Kasipul	2000-2017	-0.080	-0.053	-0.026
Kenya	Kathiani	2000	0.156	0.205	0.263
Kenya	Kathiani	2017	0.144	0.198	0.251
Kenya	Kathiani	2000-2017	-0.032	-0.005	0.031
Kenya	Keiyo North	2000	0.109	0.135	0.173
Kenya	Keiyo North	2017	0.108	0.140	0.187
Kenya	Keiyo North	2000-2017	-0.024	0.003	0.037
Kenya	Keiyo South	2000	0.111	0.138	0.176
Kenya	Keiyo South	2017	0.114	0.149	0.204
Kenya	Keiyo South	2000-2017	-0.024	0.005	0.042
Kenya	Kesses	2000	0.145	0.177	0.228
Kenya	Kesses	2017	0.100	0.130	0.179
Kenya	Kesses	2000-2017	-0.041	-0.014	0.020
Kenya	Khwisero	2000	0.332	0.411	0.503
Kenya	Khwisero	2017	0.145	0.187	0.238
Kenya	Khwisero	2000-2017	-0.062	-0.034	-0.004
Kenya	Kiambaa	2000	0.097	0.127	0.162
Kenya	Kiambaa	2017	0.079	0.103	0.133
Kenya	Kiambaa	2000-2017	-0.042	-0.013	0.022
Kenya	Kiambu	2000	0.102	0.135	0.173
Kenya	Kiambu	2017	0.081	0.106	0.137
Kenya	Kiambu	2000-2017	-0.043	-0.014	0.019
Kenya	Kibra	2000	0.098	0.129	0.163
Kenya	Kibra	2017	0.023	0.031	0.040
Kenya	Kibra	2000-2017	-0.114	-0.086	-0.054
Kenya	Kibwezi East	2000	0.214	0.294	0.397
Kenya	Kibwezi East	2017	0.032	0.043	0.057
Kenya	Kibwezi East	2000-2017	-0.166	-0.133	-0.104
Kenya	Kibwezi West	2000	0.218	0.292	0.389
Kenya	Kibwezi West	2017	0.033	0.042	0.055
Kenya	Kibwezi West	2000-2017	-0.162	-0.134	-0.106
Kenya	Kieni	2000	0.074	0.099	0.126
Kenya	Kieni	2017	0.072	0.097	0.126
Kenya	Kieni	2000-2017	-0.039	-0.009	0.022
Kenya	Kigumo	2000	0.027	0.037	0.046
Kenya	Kigumo	2017	0.024	0.032	0.041
Kenya	Kigumo	2000-2017	-0.022	0.007	0.039
Kenya	Kiharu	2000	0.028	0.038	0.048
Kenya	Kiharu	2017	0.025	0.032	0.042
Kenya	Kiharu	2000-2017	-0.023	0.005	0.036
Kenya	Kikuyu	2000	0.099	0.130	0.163
Kenya	Kikuyu	2017	0.079	0.105	0.135
Kenya	Kikuyu	2000-2017	-0.045	-0.012	0.021
Kenya	Kilgoris	2000	0.155	0.199	0.251

Table 2: Diarrhea DALYs rate by unit *(continued)*

Country	Unit	year	mean rate	lower rate	upper rate
Kenya	Kilgoris	2017	0.038	0.050	0.066
Kenya	Kilgoris	2000-2017	-0.112	-0.084	-0.051
Kenya	Kilifi North	2000	0.346	0.462	0.589
Kenya	Kilifi North	2017	0.165	0.212	0.269
Kenya	Kilifi North	2000-2017	-0.072	-0.041	-0.009
Kenya	Kilifi South	2000	0.333	0.439	0.567
Kenya	Kilifi South	2017	0.150	0.191	0.248
Kenya	Kilifi South	2000-2017	-0.075	-0.042	-0.008
Kenya	Kilome	2000	0.216	0.286	0.370
Kenya	Kilome	2017	0.031	0.041	0.053
Kenya	Kilome	2000-2017	-0.162	-0.132	-0.100
Kenya	Kimilili	2000	0.123	0.159	0.204
Kenya	Kimilili	2017	0.134	0.171	0.219
Kenya	Kimilili	2000-2017	-0.017	0.012	0.042
Kenya	Kiminini	2000	0.126	0.161	0.205
Kenya	Kiminini	2017	0.102	0.129	0.168
Kenya	Kiminini	2000-2017	-0.031	0.002	0.032
Kenya	Kinango	2000	0.234	0.317	0.416
Kenya	Kinango	2017	0.067	0.086	0.110
Kenya	Kinango	2000-2017	-0.096	-0.065	-0.035
Kenya	Kinangop	2000	0.081	0.106	0.138
Kenya	Kinangop	2017	0.107	0.138	0.174
Kenya	Kinangop	2000-2017	-0.011	0.018	0.052
Kenya	Kipipiri	2000	0.082	0.108	0.139
Kenya	Kipipiri	2017	0.103	0.136	0.174
Kenya	Kipipiri	2000-2017	-0.014	0.016	0.047
Kenya	Kipkelion East	2000	0.080	0.102	0.131
Kenya	Kipkelion East	2017	0.089	0.114	0.156
Kenya	Kipkelion East	2000-2017	-0.015	0.016	0.051
Kenya	Kipkelion West	2000	0.080	0.102	0.130
Kenya	Kipkelion West	2017	0.091	0.117	0.157
Kenya	Kipkelion West	2000-2017	-0.013	0.018	0.054
Kenya	Kirinyaga Central	2000	0.158	0.216	0.273
Kenya	Kirinyaga Central	2017	0.128	0.168	0.217
Kenya	Kirinyaga Central	2000-2017	-0.049	-0.018	0.017
Kenya	Kisauni	2000	0.128	0.174	0.227
Kenya	Kisauni	2017	0.100	0.130	0.169
Kenya	Kisauni	2000-2017	-0.039	-0.007	0.027
Kenya	Kisumu Central	2000	0.164	0.201	0.247
Kenya	Kisumu Central	2017	0.086	0.109	0.137
Kenya	Kisumu Central	2000-2017	-0.063	-0.034	-0.006
Kenya	Kisumu East	2000	0.156	0.193	0.237
Kenya	Kisumu East	2017	0.083	0.105	0.133
Kenya	Kisumu East	2000-2017	-0.063	-0.034	-0.006
Kenya	Kisumu West	2000	0.152	0.188	0.227
Kenya	Kisumu West	2017	0.078	0.098	0.127
Kenya	Kisumu West	2000-2017	-0.064	-0.036	-0.008
Kenya	Kitui Central	2000	0.205	0.277	0.367
Kenya	Kitui Central	2017	0.089	0.118	0.155
Kenya	Kitui Central	2000-2017	-0.080	-0.049	-0.017
Kenya	Kitui East	2000	0.204	0.278	0.368
Kenya	Kitui East	2017	0.093	0.123	0.162
Kenya	Kitui East	2000-2017	-0.076	-0.048	-0.016
Kenya	Kitui Rural	2000	0.197	0.262	0.336
Kenya	Kitui Rural	2017	0.090	0.118	0.153
Kenya	Kitui Rural	2000-2017	-0.070	-0.042	-0.011
Kenya	Kitui South	2000	0.192	0.266	0.355
Kenya	Kitui South	2017	0.100	0.131	0.171
Kenya	Kitui South	2000-2017	-0.065	-0.038	-0.007
Kenya	Kitui West	2000	0.205	0.278	0.365
Kenya	Kitui West	2017	0.087	0.116	0.151

Table 2: Diarrhea DALYs rate by unit *(continued)*

Country	Unit	year	mean rate	lower rate	upper rate
Kenya	Kitui West	2000-2017	-0.080	-0.049	-0.016
Kenya	Kitutu Chache North	2000	0.268	0.336	0.414
Kenya	Kitutu Chache North	2017	0.135	0.176	0.229
Kenya	Kitutu Chache North	2000-2017	-0.061	-0.032	-0.003
Kenya	Kitutu Chache South	2000	0.267	0.335	0.413
Kenya	Kitutu Chache South	2017	0.138	0.180	0.234
Kenya	Kitutu Chache South	2000-2017	-0.061	-0.033	-0.004
Kenya	Kitutu Masaba	2000	0.202	0.254	0.312
Kenya	Kitutu Masaba	2017	0.054	0.071	0.094
Kenya	Kitutu Masaba	2000-2017	-0.092	-0.064	-0.033
Kenya	Konoin	2000	0.051	0.065	0.083
Kenya	Konoin	2017	0.044	0.058	0.076
Kenya	Konoin	2000-2017	-0.007	0.024	0.059
Kenya	Kuresoi North	2000	0.104	0.132	0.170
Kenya	Kuresoi North	2017	0.085	0.108	0.145
Kenya	Kuresoi North	2000-2017	-0.060	-0.027	0.007
Kenya	Kuresoi South	2000	0.098	0.127	0.167
Kenya	Kuresoi South	2017	0.081	0.106	0.141
Kenya	Kuresoi South	2000-2017	-0.059	-0.025	0.009
Kenya	Kuria East	2000	0.601	0.770	0.976
Kenya	Kuria East	2017	0.172	0.227	0.295
Kenya	Kuria East	2000-2017	-0.098	-0.072	-0.042
Kenya	Kuria West	2000	0.617	0.798	1.010
Kenya	Kuria West	2017	0.182	0.237	0.312
Kenya	Kuria West	2000-2017	-0.102	-0.075	-0.046
Kenya	Kwanza	2000	0.125	0.159	0.202
Kenya	Kwanza	2017	0.103	0.130	0.164
Kenya	Kwanza	2000-2017	-0.031	0.002	0.031
Kenya	Lafey	2000	0.175	0.236	0.315
Kenya	Lafey	2017	0.102	0.135	0.181
Kenya	Lafey	2000-2017	-0.039	-0.005	0.025
Kenya	Lagdera	2000	0.286	0.395	0.552
Kenya	Lagdera	2017	0.070	0.094	0.121
Kenya	Lagdera	2000-2017	-0.103	-0.076	-0.047
Kenya	Laikipia East	2000	0.103	0.134	0.171
Kenya	Laikipia East	2017	0.046	0.062	0.082
Kenya	Laikipia East	2000-2017	-0.074	-0.047	-0.014
Kenya	Laikipia North	2000	0.117	0.151	0.189
Kenya	Laikipia North	2017	0.047	0.062	0.080
Kenya	Laikipia North	2000-2017	-0.079	-0.053	-0.021
Kenya	Laikipia West	2000	0.109	0.145	0.183
Kenya	Laikipia West	2017	0.049	0.064	0.083
Kenya	Laikipia West	2000-2017	-0.076	-0.048	-0.014
Kenya	Laisamis	2000	0.224	0.300	0.402
Kenya	Laisamis	2017	0.152	0.199	0.260
Kenya	Laisamis	2000-2017	-0.045	-0.015	0.019
Kenya	Lamu East	2000	0.215	0.300	0.402
Kenya	Lamu East	2017	0.113	0.160	0.221
Kenya	Lamu East	2000-2017	-0.053	-0.026	0.004
Kenya	Lamu West	2000	0.188	0.265	0.346
Kenya	Lamu West	2017	0.123	0.163	0.218
Kenya	Lamu West	2000-2017	-0.048	-0.019	0.012
Kenya	Langata	2000	0.089	0.117	0.148
Kenya	Langata	2017	0.022	0.029	0.038
Kenya	Langata	2000-2017	-0.110	-0.081	-0.048
Kenya	Lari	2000	0.100	0.129	0.168
Kenya	Lari	2017	0.077	0.101	0.129
Kenya	Lari	2000-2017	-0.044	-0.014	0.019
Kenya	Likoni	2000	0.127	0.171	0.223
Kenya	Likoni	2017	0.102	0.134	0.172
Kenya	Likoni	2000-2017	-0.039	-0.006	0.027

Table 2: Diarrhea DALYs rate by unit *(continued)*

Country	Unit	year	mean rate	lower rate	upper rate
Kenya	Likuyani	2000	0.115	0.146	0.190
Kenya	Likuyani	2017	0.125	0.160	0.213
Kenya	Likuyani	2000-2017	-0.016	0.015	0.048
Kenya	Limuru	2000	0.096	0.124	0.158
Kenya	Limuru	2017	0.075	0.100	0.129
Kenya	Limuru	2000-2017	-0.044	-0.012	0.021
Kenya	Loima	2000	0.282	0.379	0.509
Kenya	Loima	2017	0.127	0.174	0.226
Kenya	Loima	2000-2017	-0.067	-0.037	-0.004
Kenya	Luanda	2000	0.226	0.280	0.338
Kenya	Luanda	2017	0.051	0.065	0.084
Kenya	Luanda	2000-2017	-0.119	-0.090	-0.060
Kenya	Lugari	2000	0.305	0.386	0.485
Kenya	Lugari	2000	0.119	0.150	0.190
Kenya	Lugari	2017	0.129	0.166	0.219
Kenya	Lugari	2017	0.138	0.178	0.231
Kenya	Lugari	2000-2017	-0.064	-0.035	-0.007
Kenya	Lugari	2000-2017	-0.015	0.015	0.047
Kenya	Lungalunga	2000	0.209	0.290	0.388
Kenya	Lungalunga	2017	0.058	0.079	0.100
Kenya	Lungalunga	2000-2017	-0.097	-0.066	-0.028
Kenya	Lurambi	2000	0.316	0.395	0.488
Kenya	Lurambi	2017	0.142	0.182	0.237
Kenya	Lurambi	2000-2017	-0.061	-0.034	-0.004
Kenya	Maara	2000	0.671	0.913	1.164
Kenya	Maara	2017	0.289	0.388	0.507
Kenya	Maara	2000-2017	-0.079	-0.048	-0.016
Kenya	Machakos Town	2000	0.154	0.203	0.260
Kenya	Machakos Town	2017	0.145	0.200	0.256
Kenya	Machakos Town	2000-2017	-0.031	-0.004	0.031
Kenya	Magarini	2000	0.309	0.435	0.569
Kenya	Magarini	2017	0.162	0.217	0.283
Kenya	Magarini	2000-2017	-0.064	-0.034	-0.003
Kenya	Makadara	2000	0.090	0.117	0.151
Kenya	Makadara	2017	0.027	0.037	0.046
Kenya	Makadara	2000-2017	-0.104	-0.075	-0.041
Kenya	Makueni	2000	0.227	0.298	0.385
Kenya	Makueni	2017	0.033	0.042	0.054
Kenya	Makueni	2000-2017	-0.162	-0.134	-0.105
Kenya	Malava	2000	0.314	0.397	0.496
Kenya	Malava	2017	0.136	0.177	0.232
Kenya	Malava	2000-2017	-0.066	-0.037	-0.010
Kenya	Malindi	2000	0.309	0.430	0.562
Kenya	Malindi	2017	0.164	0.218	0.282
Kenya	Malindi	2000-2017	-0.063	-0.034	-0.001
Kenya	Mandera East	2000	0.155	0.216	0.305
Kenya	Mandera East	2017	0.101	0.138	0.187
Kenya	Mandera East	2000-2017	-0.041	-0.006	0.028
Kenya	Mandera North	2000	0.163	0.220	0.297
Kenya	Mandera North	2017	0.099	0.131	0.182
Kenya	Mandera North	2000-2017	-0.043	-0.009	0.024
Kenya	Mandera South	2000	0.167	0.227	0.300
Kenya	Mandera South	2017	0.092	0.128	0.170
Kenya	Mandera South	2000-2017	-0.037	-0.008	0.022
Kenya	Mandera West	2000	0.171	0.233	0.311
Kenya	Mandera West	2017	0.093	0.131	0.175
Kenya	Mandera West	2000-2017	-0.041	-0.007	0.023
Kenya	Manyatta	2000	0.125	0.169	0.216
Kenya	Manyatta	2017	0.115	0.153	0.196
Kenya	Manyatta	2000-2017	-0.036	-0.005	0.030
Kenya	Maragwa	2000	0.028	0.038	0.050

Table 2: Diarrhea DALYs rate by unit *(continued)*

Country	Unit	year	mean rate	lower rate	upper rate
Kenya	Maragwa	2017	0.025	0.032	0.043
Kenya	Maragwa	2000-2017	-0.022	0.006	0.040
Kenya	Marakwet East	2000	0.128	0.165	0.213
Kenya	Marakwet East	2017	0.119	0.156	0.202
Kenya	Marakwet East	2000-2017	-0.030	-0.001	0.032
Kenya	Marakwet West	2000	0.116	0.146	0.191
Kenya	Marakwet West	2017	0.115	0.149	0.196
Kenya	Marakwet West	2000-2017	-0.030	-0.001	0.031
Kenya	Masinga	2000	0.155	0.209	0.268
Kenya	Masinga	2017	0.144	0.191	0.243
Kenya	Masinga	2000-2017	-0.036	-0.010	0.020
Kenya	Matayos	2000	0.212	0.268	0.329
Kenya	Matayos	2017	0.139	0.173	0.212
Kenya	Matayos	2000-2017	-0.040	-0.014	0.016
Kenya	Mathare	2000	0.099	0.129	0.166
Kenya	Mathare	2017	0.032	0.043	0.056
Kenya	Mathare	2000-2017	-0.104	-0.074	-0.041
Kenya	Mathioya	2000	0.027	0.037	0.046
Kenya	Mathioya	2017	0.024	0.031	0.041
Kenya	Mathioya	2000-2017	-0.025	0.005	0.036
Kenya	Mathira	2000	0.078	0.104	0.131
Kenya	Mathira	2017	0.075	0.099	0.129
Kenya	Mathira	2000-2017	-0.039	-0.009	0.026
Kenya	Matuga	2000	0.212	0.287	0.376
Kenya	Matuga	2017	0.061	0.081	0.105
Kenya	Matuga	2000-2017	-0.091	-0.060	-0.030
Kenya	Matungu	2000	0.319	0.402	0.492
Kenya	Matungu	2017	0.151	0.190	0.240
Kenya	Matungu	2000-2017	-0.061	-0.033	-0.003
Kenya	Matungulu	2000	0.151	0.202	0.259
Kenya	Matungulu	2017	0.142	0.193	0.250
Kenya	Matungulu	2000-2017	-0.032	-0.006	0.030
Kenya	Mavoko	2000	0.143	0.189	0.246
Kenya	Mavoko	2017	0.134	0.186	0.247
Kenya	Mavoko	2000-2017	-0.033	-0.004	0.032
Kenya	Mbeere North	2000	0.133	0.183	0.233
Kenya	Mbeere North	2017	0.124	0.164	0.214
Kenya	Mbeere North	2000-2017	-0.034	-0.006	0.027
Kenya	Mbeere South	2000	0.132	0.177	0.229
Kenya	Mbeere South	2017	0.122	0.161	0.204
Kenya	Mbeere South	2000-2017	-0.032	-0.005	0.027
Kenya	Mbita	2000	0.471	0.595	0.724
Kenya	Mbita	2017	0.170	0.215	0.270
Kenya	Mbita	2000-2017	-0.098	-0.072	-0.045
Kenya	Mbooni	2000	0.219	0.289	0.375
Kenya	Mbooni	2017	0.032	0.042	0.052
Kenya	Mbooni	2000-2017	-0.162	-0.135	-0.101
Kenya	Mogotio	2000	0.279	0.358	0.452
Kenya	Mogotio	2017	0.104	0.135	0.179
Kenya	Mogotio	2000-2017	-0.101	-0.072	-0.037
Kenya	Moiben	2000	0.132	0.166	0.219
Kenya	Moiben	2017	0.091	0.119	0.160
Kenya	Moiben	2000-2017	-0.043	-0.015	0.016
Kenya	Molo	2000	0.104	0.133	0.172
Kenya	Molo	2017	0.083	0.107	0.143
Kenya	Molo	2000-2017	-0.062	-0.028	0.005
Kenya	Mosop	2000	0.108	0.135	0.174
Kenya	Mosop	2017	0.068	0.087	0.116
Kenya	Mosop	2000-2017	-0.051	-0.023	0.008
Kenya	Moyale	2000	0.168	0.234	0.322
Kenya	Moyale	2017	0.105	0.144	0.191

Table 2: Diarrhea DALYs rate by unit *(continued)*

Country	Unit	year	mean rate	lower rate	upper rate
Kenya	Moyale	2000-2017	-0.061	-0.028	0.004
Kenya	Msambweni	2000	0.199	0.276	0.362
Kenya	Msambweni	2017	0.058	0.079	0.104
Kenya	Msambweni	2000-2017	-0.090	-0.059	-0.026
Kenya	Mt. Elgon	2000	0.122	0.157	0.196
Kenya	Mt. Elgon	2017	0.135	0.171	0.217
Kenya	Mt. Elgon	2000-2017	-0.018	0.010	0.041
Kenya	Muhoroni	2000	0.138	0.174	0.225
Kenya	Muhoroni	2017	0.076	0.095	0.127
Kenya	Muhoroni	2000-2017	-0.061	-0.030	0.003
Kenya	Mukurweini	2000	0.081	0.109	0.138
Kenya	Mukurweini	2017	0.080	0.106	0.139
Kenya	Mukurweini	2000-2017	-0.038	-0.008	0.027
Kenya	Mumias East	2000	0.323	0.406	0.498
Kenya	Mumias East	2017	0.151	0.190	0.245
Kenya	Mumias East	2000-2017	-0.059	-0.033	-0.003
Kenya	Mumias West	2000	0.325	0.410	0.507
Kenya	Mumias West	2017	0.152	0.190	0.242
Kenya	Mumias West	2000-2017	-0.062	-0.035	-0.006
Kenya	Mvita	2000	0.127	0.173	0.225
Kenya	Mvita	2017	0.101	0.133	0.173
Kenya	Mvita	2000-2017	-0.037	-0.005	0.028
Kenya	Mwala	2000	0.155	0.207	0.269
Kenya	Mwala	2017	0.148	0.195	0.251
Kenya	Mwala	2000-2017	-0.036	-0.009	0.025
Kenya	Mwatate	2000	0.187	0.261	0.351
Kenya	Mwatate	2017	0.087	0.117	0.159
Kenya	Mwatate	2000-2017	-0.086	-0.050	-0.013
Kenya	Mwea	2000	0.170	0.229	0.292
Kenya	Mwea	2017	0.133	0.180	0.230
Kenya	Mwea	2000-2017	-0.055	-0.025	0.007
Kenya	Mwingi Central	2000	0.222	0.294	0.378
Kenya	Mwingi Central	2017	0.086	0.115	0.146
Kenya	Mwingi Central	2000-2017	-0.079	-0.053	-0.021
Kenya	Mwingi North	2000	0.222	0.300	0.387
Kenya	Mwingi North	2017	0.090	0.118	0.148
Kenya	Mwingi North	2000-2017	-0.075	-0.047	-0.017
Kenya	Mwingi West	2000	0.206	0.279	0.361
Kenya	Mwingi West	2017	0.084	0.112	0.145
Kenya	Mwingi West	2000-2017	-0.081	-0.051	-0.019
Kenya	Naivasha	2000	0.090	0.119	0.153
Kenya	Naivasha	2017	0.078	0.100	0.126
Kenya	Naivasha	2000-2017	-0.053	-0.022	0.011
Kenya	Nakuru Town East	2000	0.109	0.139	0.178
Kenya	Nakuru Town East	2017	0.083	0.110	0.146
Kenya	Nakuru Town East	2000-2017	-0.060	-0.028	0.002
Kenya	Nakuru Town West	2000	0.118	0.152	0.194
Kenya	Nakuru Town West	2017	0.089	0.119	0.158
Kenya	Nakuru Town West	2000-2017	-0.061	-0.028	0.003
Kenya	Nambale	2000	0.214	0.272	0.334
Kenya	Nambale	2017	0.133	0.166	0.207
Kenya	Nambale	2000-2017	-0.044	-0.016	0.017
Kenya	Nandi Hills	2000	0.107	0.133	0.171
Kenya	Nandi Hills	2017	0.071	0.092	0.124
Kenya	Nandi Hills	2000-2017	-0.051	-0.022	0.014
Kenya	Narok East	2000	0.151	0.198	0.261
Kenya	Narok East	2017	0.035	0.047	0.060
Kenya	Narok East	2000-2017	-0.115	-0.085	-0.050
Kenya	Narok North	2000	0.153	0.199	0.253
Kenya	Narok North	2017	0.035	0.046	0.059
Kenya	Narok North	2000-2017	-0.117	-0.086	-0.055

Table 2: Diarrhea DALYs rate by unit *(continued)*

Country	Unit	year	mean rate	lower rate	upper rate
Kenya	Narok South	2000	0.152	0.194	0.252
Kenya	Narok South	2017	0.036	0.047	0.060
Kenya	Narok South	2000-2017	-0.111	-0.083	-0.050
Kenya	Narok West	2000	0.156	0.202	0.263
Kenya	Narok West	2017	0.036	0.047	0.061
Kenya	Narok West	2000-2017	-0.111	-0.084	-0.048
Kenya	Navakholo	2000	0.320	0.402	0.490
Kenya	Navakholo	2017	0.149	0.189	0.243
Kenya	Navakholo	2000-2017	-0.062	-0.034	-0.004
Kenya	Ndaragwa	2000	0.080	0.107	0.135
Kenya	Ndaragwa	2017	0.101	0.134	0.178
Kenya	Ndaragwa	2000-2017	-0.013	0.016	0.050
Kenya	Ndhiwa	2000	0.451	0.572	0.720
Kenya	Ndhiwa	2017	0.178	0.224	0.286
Kenya	Ndhiwa	2000-2017	-0.083	-0.056	-0.027
Kenya	Ndia	2000	0.167	0.225	0.284
Kenya	Ndia	2017	0.131	0.174	0.227
Kenya	Ndia	2000-2017	-0.047	-0.018	0.017
Kenya	Njoro	2000	0.107	0.135	0.173
Kenya	Njoro	2017	0.081	0.106	0.139
Kenya	Njoro	2000-2017	-0.062	-0.029	0.002
Kenya	North Horr	2000	0.248	0.326	0.432
Kenya	North Horr	2017	0.160	0.208	0.277
Kenya	North Horr	2000-2017	-0.038	-0.007	0.024
Kenya	North Imenti	2000	0.131	0.179	0.229
Kenya	North Imenti	2017	0.155	0.209	0.269
Kenya	North Imenti	2000-2017	-0.012	0.017	0.051
Kenya	North Mugirango	2000	0.204	0.259	0.320
Kenya	North Mugirango	2017	0.058	0.074	0.097
Kenya	North Mugirango	2000-2017	-0.094	-0.065	-0.034
Kenya	Nyakach	2000	0.146	0.183	0.224
Kenya	Nyakach	2017	0.085	0.107	0.137
Kenya	Nyakach	2000-2017	-0.052	-0.023	0.007
Kenya	Nyali	2000	0.128	0.175	0.228
Kenya	Nyali	2017	0.102	0.133	0.174
Kenya	Nyali	2000-2017	-0.038	-0.005	0.030
Kenya	Nyando	2000	0.152	0.189	0.238
Kenya	Nyando	2017	0.084	0.105	0.135
Kenya	Nyando	2000-2017	-0.061	-0.030	0.000
Kenya	Nyaribari Chache	2000	0.238	0.300	0.368
Kenya	Nyaribari Chache	2017	0.125	0.164	0.217
Kenya	Nyaribari Chache	2000-2017	-0.058	-0.030	0.000
Kenya	Nyaribari Masaba	2000	0.230	0.290	0.357
Kenya	Nyaribari Masaba	2017	0.131	0.171	0.229
Kenya	Nyaribari Masaba	2000-2017	-0.056	-0.027	0.005
Kenya	Nyatike	2000	0.668	0.853	1.115
Kenya	Nyatike	2017	0.226	0.278	0.356
Kenya	Nyatike	2000-2017	-0.098	-0.072	-0.044
Kenya	Nyeri Town	2000	0.075	0.103	0.128
Kenya	Nyeri Town	2017	0.075	0.100	0.130
Kenya	Nyeri Town	2000-2017	-0.038	-0.007	0.026
Kenya	Ol Jorok	2000	0.083	0.112	0.140
Kenya	Ol Jorok	2017	0.106	0.137	0.180
Kenya	Ol Jorok	2000-2017	-0.018	0.013	0.047
Kenya	Ol Kalou	2000	0.086	0.114	0.145
Kenya	Ol Kalou	2017	0.106	0.138	0.179
Kenya	Ol Kalou	2000-2017	-0.019	0.012	0.042
Kenya	Othaya	2000	0.076	0.104	0.131
Kenya	Othaya	2017	0.079	0.104	0.137
Kenya	Othaya	2000-2017	-0.035	-0.005	0.026
Kenya	Pokot South	2000	0.326	0.407	0.511

Table 2: Diarrhea DALYs rate by unit *(continued)*

Country	Unit	year	mean rate	lower rate	upper rate
Kenya	Pokot South	2017	0.149	0.194	0.250
Kenya	Pokot South	2000-2017	-0.060	-0.030	-0.001
Kenya	Rabai	2000	0.325	0.422	0.550
Kenya	Rabai	2017	0.139	0.179	0.232
Kenya	Rabai	2000-2017	-0.078	-0.045	-0.014
Kenya	Rangwe	2000	0.458	0.564	0.706
Kenya	Rangwe	2017	0.160	0.205	0.263
Kenya	Rangwe	2000-2017	-0.084	-0.058	-0.032
Kenya	Rarieda	2000	0.282	0.353	0.427
Kenya	Rarieda	2017	0.130	0.162	0.205
Kenya	Rarieda	2000-2017	-0.067	-0.041	-0.013
Kenya	Rongai	2000	0.105	0.133	0.167
Kenya	Rongai	2017	0.080	0.103	0.136
Kenya	Rongai	2000-2017	-0.064	-0.031	0.001
Kenya	Rongo	2000	0.634	0.801	1.011
Kenya	Rongo	2017	0.178	0.230	0.302
Kenya	Rongo	2000-2017	-0.102	-0.076	-0.048
Kenya	Roysambu	2000	0.097	0.128	0.165
Kenya	Roysambu	2017	0.024	0.031	0.040
Kenya	Roysambu	2000-2017	-0.111	-0.082	-0.049
Kenya	Ruaraka	2000	0.095	0.124	0.159
Kenya	Ruaraka	2017	0.028	0.037	0.046
Kenya	Ruaraka	2000-2017	-0.105	-0.076	-0.042
Kenya	Ruiru	2000	0.101	0.132	0.169
Kenya	Ruiru	2017	0.078	0.105	0.136
Kenya	Ruiru	2000-2017	-0.045	-0.017	0.018
Kenya	Runyenjes	2000	0.127	0.172	0.219
Kenya	Runyenjes	2017	0.121	0.159	0.204
Kenya	Runyenjes	2000-2017	-0.035	-0.004	0.030
Kenya	Sabatia	2000	0.222	0.276	0.342
Kenya	Sabatia	2017	0.051	0.066	0.086
Kenya	Sabatia	2000-2017	-0.119	-0.089	-0.060
Kenya	Saboti	2000	0.126	0.160	0.201
Kenya	Saboti	2017	0.104	0.132	0.171
Kenya	Saboti	2000-2017	-0.029	0.003	0.034
Kenya	Saku	2000	0.168	0.232	0.326
Kenya	Saku	2017	0.119	0.162	0.208
Kenya	Saku	2000-2017	-0.060	-0.025	0.012
Kenya	Samburu East	2000	0.209	0.280	0.360
Kenya	Samburu East	2017	0.095	0.125	0.158
Kenya	Samburu East	2000-2017	-0.082	-0.052	-0.025
Kenya	Samburu North	2000	0.209	0.278	0.367
Kenya	Samburu North	2017	0.094	0.121	0.156
Kenya	Samburu North	2000-2017	-0.080	-0.048	-0.017
Kenya	Samburu West	2000	0.200	0.265	0.340
Kenya	Samburu West	2017	0.090	0.115	0.149
Kenya	Samburu West	2000-2017	-0.080	-0.051	-0.020
Kenya	Seme	2000	0.154	0.192	0.230
Kenya	Seme	2017	0.082	0.103	0.132
Kenya	Seme	2000-2017	-0.065	-0.037	-0.010
Kenya	Shinyalu	2000	0.306	0.387	0.480
Kenya	Shinyalu	2017	0.134	0.174	0.231
Kenya	Shinyalu	2000-2017	-0.064	-0.036	-0.007
Kenya	Sigor	2000	0.361	0.453	0.570
Kenya	Sigor	2017	0.164	0.216	0.272
Kenya	Sigor	2000-2017	-0.057	-0.026	0.004
Kenya	Sigowet/Soin	2000	0.085	0.108	0.137
Kenya	Sigowet/Soin	2017	0.097	0.125	0.163
Kenya	Sigowet/Soin	2000-2017	-0.012	0.019	0.052
Kenya	Sirisia	2000	0.132	0.168	0.209
Kenya	Sirisia	2017	0.150	0.187	0.237

Table 2: Diarrhea DALYs rate by unit *(continued)*

Country	Unit	year	mean rate	lower rate	upper rate
Kenya	Sirisia	2000-2017	-0.017	0.010	0.042
Kenya	Sotik	2000	0.051	0.065	0.081
Kenya	Sotik	2017	0.045	0.058	0.076
Kenya	Sotik	2000-2017	0.000	0.029	0.063
Kenya	South Imenti	2000	0.131	0.180	0.232
Kenya	South Imenti	2017	0.155	0.215	0.282
Kenya	South Imenti	2000-2017	-0.012	0.017	0.051
Kenya	South Mugirango	2000	0.262	0.331	0.419
Kenya	South Mugirango	2017	0.146	0.190	0.250
Kenya	South Mugirango	2000-2017	-0.060	-0.032	-0.004
Kenya	Soy	2000	0.139	0.175	0.231
Kenya	Soy	2017	0.099	0.126	0.169
Kenya	Soy	2000-2017	-0.043	-0.014	0.016
Kenya	Starehe	2000	0.092	0.121	0.154
Kenya	Starehe	2017	0.027	0.036	0.045
Kenya	Starehe	2000-2017	-0.106	-0.077	-0.044
Kenya	Suba	2000	0.455	0.583	0.727
Kenya	Suba	2017	0.182	0.226	0.288
Kenya	Suba	2000-2017	-0.091	-0.064	-0.037
Kenya	Subukia	2000	0.097	0.128	0.163
Kenya	Subukia	2017	0.078	0.101	0.132
Kenya	Subukia	2000-2017	-0.059	-0.029	0.005
Kenya	Suna East	2000	0.633	0.820	1.037
Kenya	Suna East	2017	0.201	0.258	0.337
Kenya	Suna East	2000-2017	-0.098	-0.071	-0.044
Kenya	Suna West	2000	0.645	0.821	1.049
Kenya	Suna West	2017	0.205	0.263	0.344
Kenya	Suna West	2000-2017	-0.098	-0.071	-0.044
Kenya	Tarbaj	2000	0.382	0.518	0.686
Kenya	Tarbaj	2017	0.148	0.198	0.264
Kenya	Tarbaj	2000-2017	-0.092	-0.060	-0.026
Kenya	Taveta	2000	0.180	0.249	0.342
Kenya	Taveta	2017	0.079	0.107	0.142
Kenya	Taveta	2000-2017	-0.087	-0.051	-0.016
Kenya	Teso North	2000	0.224	0.287	0.353
Kenya	Teso North	2017	0.134	0.166	0.208
Kenya	Teso North	2000-2017	-0.051	-0.023	0.010
Kenya	Teso South	2000	0.212	0.269	0.326
Kenya	Teso South	2017	0.136	0.168	0.208
Kenya	Teso South	2000-2017	-0.045	-0.018	0.015
Kenya	Tetu	2000	0.077	0.105	0.132
Kenya	Tetu	2017	0.078	0.103	0.134
Kenya	Tetu	2000-2017	-0.036	-0.006	0.029
Kenya	Tharaka	2000	0.751	1.032	1.328
Kenya	Tharaka	2017	0.281	0.383	0.488
Kenya	Tharaka	2000-2017	-0.097	-0.066	-0.035
Kenya	Thika Town	2000	0.105	0.139	0.181
Kenya	Thika Town	2017	0.079	0.105	0.139
Kenya	Thika Town	2000-2017	-0.044	-0.016	0.020
Kenya	Tiaty	2000	0.355	0.451	0.561
Kenya	Tiaty	2017	0.110	0.140	0.173
Kenya	Tiaty	2000-2017	-0.096	-0.067	-0.037
Kenya	Tigania East	2000	0.151	0.201	0.256
Kenya	Tigania East	2017	0.158	0.216	0.270
Kenya	Tigania East	2000-2017	-0.019	0.008	0.042
Kenya	Tigania West	2000	0.145	0.195	0.251
Kenya	Tigania West	2017	0.163	0.222	0.279
Kenya	Tigania West	2000-2017	-0.018	0.012	0.046
Kenya	Tinderet	2000	0.106	0.134	0.173
Kenya	Tinderet	2017	0.072	0.092	0.125
Kenya	Tinderet	2000-2017	-0.050	-0.020	0.015

Table 2: Diarrhea DALYs rate by unit *(continued)*

Country	Unit	year	mean rate	lower rate	upper rate
Kenya	Tongaren	2000	0.124	0.156	0.196
Kenya	Tongaren	2017	0.134	0.171	0.225
Kenya	Tongaren	2000-2017	-0.017	0.015	0.046
Kenya	Turbo	2000	0.145	0.181	0.236
Kenya	Turbo	2017	0.103	0.133	0.179
Kenya	Turbo	2000-2017	-0.043	-0.014	0.018
Kenya	Turkana Central	2000	0.312	0.427	0.577
Kenya	Turkana Central	2017	0.139	0.184	0.246
Kenya	Turkana Central	2000-2017	-0.078	-0.048	-0.012
Kenya	Turkana East	2000	0.348	0.452	0.592
Kenya	Turkana East	2017	0.135	0.176	0.223
Kenya	Turkana East	2000-2017	-0.086	-0.055	-0.024
Kenya	Turkana North	2000	0.358	0.470	0.629
Kenya	Turkana North	2017	0.137	0.184	0.253
Kenya	Turkana North	2000-2017	-0.080	-0.052	-0.022
Kenya	Turkana South	2000	0.323	0.429	0.585
Kenya	Turkana South	2017	0.134	0.178	0.230
Kenya	Turkana South	2000-2017	-0.073	-0.041	-0.010
Kenya	Turkana West	2000	0.314	0.408	0.520
Kenya	Turkana West	2017	0.126	0.168	0.232
Kenya	Turkana West	2000-2017	-0.086	-0.055	-0.029
Kenya	Ugenya	2000	0.263	0.328	0.405
Kenya	Ugenya	2017	0.125	0.158	0.197
Kenya	Ugenya	2000-2017	-0.060	-0.035	-0.007
Kenya	Ugunja	2000	0.268	0.339	0.413
Kenya	Ugunja	2017	0.123	0.155	0.197
Kenya	Ugunja	2000-2017	-0.066	-0.039	-0.010
Kenya	Uriri	2000	0.617	0.803	1.013
Kenya	Uriri	2017	0.213	0.272	0.350
Kenya	Uriri	2000-2017	-0.096	-0.069	-0.041
Kenya	Vihiga	2000	0.218	0.273	0.337
Kenya	Vihiga	2017	0.050	0.064	0.083
Kenya	Vihiga	2000-2017	-0.117	-0.089	-0.060
Kenya	Voi	2000	0.195	0.272	0.361
Kenya	Voi	2017	0.089	0.120	0.160
Kenya	Voi	2000-2017	-0.086	-0.051	-0.017
Kenya	Wajir East	2000	0.411	0.555	0.741
Kenya	Wajir East	2017	0.152	0.204	0.270
Kenya	Wajir East	2000-2017	-0.098	-0.069	-0.038
Kenya	Wajir North	2000	0.362	0.487	0.647
Kenya	Wajir North	2017	0.144	0.203	0.265
Kenya	Wajir North	2000-2017	-0.083	-0.051	-0.023
Kenya	Wajir South	2000	0.428	0.569	0.763
Kenya	Wajir South	2017	0.173	0.227	0.302
Kenya	Wajir South	2000-2017	-0.082	-0.052	-0.024
Kenya	Wajir West	2000	0.453	0.609	0.822
Kenya	Wajir West	2017	0.166	0.222	0.293
Kenya	Wajir West	2000-2017	-0.095	-0.062	-0.033
Kenya	Webute West	2000	0.129	0.163	0.203
Kenya	Webute West	2017	0.143	0.180	0.231
Kenya	Webute West	2000-2017	-0.014	0.015	0.043
Kenya	Webuye East	2000	0.124	0.159	0.202
Kenya	Webuye East	2017	0.137	0.177	0.229
Kenya	Webuye East	2000-2017	-0.016	0.014	0.042
Kenya	West Mugirango	2000	0.198	0.249	0.305
Kenya	West Mugirango	2017	0.055	0.071	0.094
Kenya	West Mugirango	2000-2017	-0.092	-0.064	-0.034
Kenya	Westlands	2000	0.091	0.119	0.150
Kenya	Westlands	2017	0.024	0.032	0.041
Kenya	Westlands	2000-2017	-0.110	-0.081	-0.048
Kenya	Wundanyi	2000	0.184	0.254	0.340

Table 2: Diarrhea DALYs rate by unit *(continued)*

Country	Unit	year	mean rate	lower rate	upper rate
Kenya	Wundanyi	2017	0.083	0.113	0.154
Kenya	Wundanyi	2000-2017	-0.085	-0.050	-0.014
Kenya	Yatta	2000	0.157	0.211	0.270
Kenya	Yatta	2017	0.142	0.194	0.249
Kenya	Yatta	2000-2017	-0.038	-0.011	0.021
Lesotho	Berea	2000	0.240	0.328	0.455
Lesotho	Berea	2017	0.139	0.188	0.247
Lesotho	Berea	2000-2017	-0.067	-0.035	-0.004
Lesotho	Butha-Buthe	2000	0.218	0.306	0.406
Lesotho	Butha-Buthe	2017	0.131	0.183	0.248
Lesotho	Butha-Buthe	2000-2017	-0.066	-0.031	0.002
Lesotho	Leribe	2000	0.232	0.320	0.432
Lesotho	Leribe	2017	0.133	0.184	0.245
Lesotho	Leribe	2000-2017	-0.066	-0.034	-0.003
Lesotho	Mafeteng	2000	0.225	0.312	0.437
Lesotho	Mafeteng	2017	0.135	0.184	0.257
Lesotho	Mafeteng	2000-2017	-0.059	-0.030	0.000
Lesotho	Maseru	2000	0.236	0.324	0.443
Lesotho	Maseru	2017	0.142	0.187	0.249
Lesotho	Maseru	2000-2017	-0.063	-0.033	-0.001
Lesotho	Mohale's Hoek	2000	0.234	0.315	0.435
Lesotho	Mohale's Hoek	2017	0.129	0.174	0.240
Lesotho	Mohale's Hoek	2000-2017	-0.062	-0.033	-0.001
Lesotho	Mokhotlong	2000	0.239	0.329	0.445
Lesotho	Mokhotlong	2017	0.145	0.199	0.271
Lesotho	Mokhotlong	2000-2017	-0.064	-0.029	0.009
Lesotho	Qacha's Nek	2000	0.224	0.308	0.415
Lesotho	Qacha's Nek	2017	0.128	0.174	0.237
Lesotho	Qacha's Nek	2000-2017	-0.060	-0.029	0.005
Lesotho	Quthing	2000	0.234	0.328	0.451
Lesotho	Quthing	2017	0.123	0.171	0.242
Lesotho	Quthing	2000-2017	-0.067	-0.036	0.001
Lesotho	Thaba-Tseka	2000	0.244	0.333	0.459
Lesotho	Thaba-Tseka	2017	0.143	0.196	0.269
Lesotho	Thaba-Tseka	2000-2017	-0.062	-0.029	0.005
Liberia	Barrobo	2000	0.456	0.669	0.953
Liberia	Barrobo	2017	0.118	0.193	0.287
Liberia	Barrobo	2000-2017	-0.155	-0.068	0.025
Liberia	Belleh	2000	0.491	0.720	0.970
Liberia	Belleh	2017	0.110	0.160	0.218
Liberia	Belleh	2000-2017	-0.215	-0.139	-0.061
Liberia	Bokomu	2000	0.491	0.721	0.985
Liberia	Bokomu	2017	0.112	0.158	0.221
Liberia	Bokomu	2000-2017	-0.208	-0.133	-0.047
Liberia	Bopolu	2000	0.472	0.670	0.901
Liberia	Bopolu	2017	0.107	0.148	0.204
Liberia	Bopolu	2000-2017	-0.202	-0.122	-0.049
Liberia	Buah	2000	0.465	0.691	0.982
Liberia	Buah	2017	0.127	0.202	0.296
Liberia	Buah	2000-2017	-0.160	-0.068	0.028
Liberia	Butaw	2000	0.460	0.682	0.975
Liberia	Butaw	2017	0.122	0.192	0.284
Liberia	Butaw	2000-2017	-0.176	-0.088	0.006
Liberia	Careysburg	2000	0.356	0.530	0.727
Liberia	Careysburg	2017	0.093	0.128	0.194
Liberia	Careysburg	2000-2017	-0.191	-0.109	-0.033
Liberia	Commnwealth	2000	0.398	0.581	0.808
Liberia	Commnwealth	2017	0.087	0.127	0.187
Liberia	Commnwealth	2000-2017	-0.194	-0.111	-0.033
Liberia	District # 1	2000	0.436	0.650	0.935
Liberia	District # 1	2017	0.110	0.162	0.240

Table 2: Diarrhea DALYs rate by unit *(continued)*

Country	Unit	year	mean rate	lower rate	upper rate
Liberia	District # 1	2000-2017	-0.177	-0.098	-0.022
Liberia	District # 2	2000	0.462	0.684	0.985
Liberia	District # 2	2017	0.117	0.176	0.263
Liberia	District # 2	2000-2017	-0.174	-0.101	-0.021
Liberia	District # 3	2000	0.428	0.634	0.911
Liberia	District # 3	2017	0.112	0.169	0.254
Liberia	District # 3	2000-2017	-0.172	-0.096	-0.020
Liberia	District # 4	2000	0.425	0.623	0.921
Liberia	District # 4	2017	0.111	0.168	0.253
Liberia	District # 4	2000-2017	-0.180	-0.092	-0.016
Liberia	Dugbe River	2000	0.447	0.663	0.975
Liberia	Dugbe River	2017	0.113	0.190	0.287
Liberia	Dugbe River	2000-2017	-0.161	-0.076	0.024
Liberia	Firestone	2000	0.407	0.602	0.849
Liberia	Firestone	2017	0.101	0.145	0.213
Liberia	Firestone	2000-2017	-0.183	-0.102	-0.028
Liberia	Foya	2000	0.539	0.731	1.010
Liberia	Foya	2017	0.105	0.145	0.190
Liberia	Foya	2000-2017	-0.241	-0.159	-0.089
Liberia	Fuamah	2000	0.466	0.666	0.930
Liberia	Fuamah	2017	0.107	0.150	0.213
Liberia	Fuamah	2000-2017	-0.195	-0.115	-0.025
Liberia	Garwula	2000	0.410	0.595	0.820
Liberia	Garwula	2017	0.090	0.128	0.185
Liberia	Garwula	2000-2017	-0.197	-0.117	-0.037
Liberia	Gbarma	2000	0.438	0.640	0.859
Liberia	Gbarma	2017	0.102	0.139	0.192
Liberia	Gbarma	2000-2017	-0.197	-0.121	-0.044
Liberia	Gbarzon	2000	0.468	0.662	0.897
Liberia	Gbarzon	2017	0.122	0.183	0.274
Liberia	Gbarzon	2000-2017	-0.184	-0.104	-0.024
Liberia	Gbeapo	2000	0.494	0.698	0.975
Liberia	Gbeapo	2017	0.123	0.195	0.287
Liberia	Gbeapo	2000-2017	-0.165	-0.079	0.009
Liberia	Gbehlageh	2000	0.510	0.706	0.961
Liberia	Gbehlageh	2017	0.123	0.189	0.275
Liberia	Gbehlageh	2000-2017	-0.207	-0.124	-0.044
Liberia	Gibi	2000	0.445	0.683	0.960
Liberia	Gibi	2017	0.109	0.165	0.240
Liberia	Gibi	2000-2017	-0.182	-0.109	-0.025
Liberia	Golakonneh	2000	0.436	0.632	0.859
Liberia	Golakonneh	2017	0.098	0.134	0.183
Liberia	Golakonneh	2000-2017	-0.197	-0.122	-0.038
Liberia	Greater Monrovia	2000	0.317	0.474	0.658
Liberia	Greater Monrovia	2017	0.081	0.118	0.181
Liberia	Greater Monrovia	2000-2017	-0.181	-0.096	-0.017
Liberia	Greenville	2000	0.423	0.645	0.934
Liberia	Greenville	2017	0.106	0.182	0.277
Liberia	Greenville	2000-2017	-0.173	-0.081	0.022
Liberia	Jaedae Jaedepo	2000	0.490	0.711	1.035
Liberia	Jaedae Jaedepo	2017	0.121	0.198	0.293
Liberia	Jaedae Jaedepo	2000-2017	-0.167	-0.082	0.019
Liberia	Jorquelleh	2000	0.468	0.669	0.944
Liberia	Jorquelleh	2017	0.115	0.168	0.244
Liberia	Jorquelleh	2000-2017	-0.192	-0.114	-0.032
Liberia	Juarzon	2000	0.473	0.682	0.981
Liberia	Juarzon	2017	0.119	0.191	0.286
Liberia	Juarzon	2000-2017	-0.169	-0.087	0.011
Liberia	Kakata	2000	0.422	0.629	0.891
Liberia	Kakata	2017	0.103	0.147	0.218
Liberia	Kakata	2000-2017	-0.182	-0.108	-0.021

Table 2: Diarrhea DALYs rate by unit *(continued)*

Country	Unit	year	mean rate	lower rate	upper rate
Liberia	Klay	2000	0.424	0.607	0.820
Liberia	Klay	2017	0.095	0.134	0.198
Liberia	Klay	2000-2017	-0.191	-0.116	-0.041
Liberia	Kokoyah	2000	0.476	0.702	1.003
Liberia	Kokoyah	2017	0.121	0.178	0.260
Liberia	Kokoyah	2000-2017	-0.185	-0.112	-0.029
Liberia	Kolahun	2000	0.519	0.714	0.967
Liberia	Kolahun	2017	0.106	0.146	0.190
Liberia	Kolahun	2000-2017	-0.231	-0.155	-0.080
Liberia	Kongba	2000	0.507	0.700	0.935
Liberia	Kongba	2017	0.103	0.144	0.184
Liberia	Kongba	2000-2017	-0.222	-0.145	-0.062
Liberia	Konobo	2000	0.487	0.650	0.872
Liberia	Konobo	2017	0.117	0.189	0.276
Liberia	Konobo	2000-2017	-0.169	-0.089	-0.020
Liberia	Kpayan	2000	0.456	0.672	0.958
Liberia	Kpayan	2017	0.112	0.190	0.288
Liberia	Kpayan	2000-2017	-0.168	-0.081	0.022
Liberia	Lower Kru Coast	2000	0.524	0.707	0.951
Liberia	Lower Kru Coast	2000	0.397	0.605	0.876
Liberia	Lower Kru Coast	2017	0.104	0.177	0.257
Liberia	Lower Kru Coast	2017	0.104	0.141	0.180
Liberia	Lower Kru Coast	2000-2017	-0.150	-0.060	0.028
Liberia	Lower Kru Coast	2000-2017	-0.235	-0.158	-0.076
Liberia	Mambah-Kaba	2000	0.357	0.527	0.743
Liberia	Mambah-Kaba	2017	0.092	0.130	0.196
Liberia	Mambah-Kaba	2000-2017	-0.186	-0.103	-0.028
Liberia	Mecca	2000	0.454	0.657	0.876
Liberia	Mecca	2017	0.103	0.142	0.210
Liberia	Mecca	2000-2017	-0.183	-0.111	-0.030
Liberia	Morweh	2000	0.476	0.715	1.006
Liberia	Morweh	2017	0.125	0.186	0.276
Liberia	Morweh	2000-2017	-0.186	-0.103	-0.027
Liberia	Owensgrove	2000	0.407	0.628	0.902
Liberia	Owensgrove	2017	0.107	0.158	0.236
Liberia	Owensgrove	2000-2017	-0.179	-0.096	-0.018
Liberia	Panta-Kpa	2000	0.488	0.685	0.963
Liberia	Panta-Kpa	2017	0.119	0.172	0.247
Liberia	Panta-Kpa	2000-2017	-0.194	-0.120	-0.038
Liberia	Pleebo/Sodeken	2000	0.407	0.613	0.871
Liberia	Pleebo/Sodeken	2017	0.105	0.179	0.262
Liberia	Pleebo/Sodeken	2000-2017	-0.149	-0.062	0.031
Liberia	Porkpa	2000	0.459	0.642	0.890
Liberia	Porkpa	2017	0.097	0.132	0.179
Liberia	Porkpa	2000-2017	-0.207	-0.131	-0.042
Liberia	Pyneston	2000	0.480	0.700	0.983
Liberia	Pyneston	2017	0.121	0.197	0.292
Liberia	Pyneston	2000-2017	-0.169	-0.090	0.004
Liberia	Saclepea	2000	0.514	0.700	0.978
Liberia	Saclepea	2017	0.121	0.175	0.246
Liberia	Saclepea	2000-2017	-0.200	-0.121	-0.046
Liberia	Salala	2000	0.455	0.681	0.952
Liberia	Salala	2017	0.107	0.158	0.229
Liberia	Salala	2000-2017	-0.186	-0.113	-0.028
Liberia	Salayea	2000	0.466	0.688	0.986
Liberia	Salayea	2017	0.109	0.163	0.231
Liberia	Salayea	2000-2017	-0.220	-0.135	-0.052
Liberia	Sanayea	2000	0.468	0.684	0.948
Liberia	Sanayea	2017	0.110	0.159	0.224
Liberia	Sanayea	2000-2017	-0.193	-0.120	-0.036
Liberia	Sanniquelleh-Mahn	2000	0.492	0.684	0.941

Table 2: Diarrhea DALYs rate by unit *(continued)*

Country	Unit	year	mean rate	lower rate	upper rate
Liberia	Sanniquelleh-Mahn	2017	0.124	0.180	0.255
Liberia	Sanniquelleh-Mahn	2000-2017	-0.204	-0.125	-0.047
Liberia	Sasstown	2000	0.455	0.675	0.983
Liberia	Sasstown	2017	0.113	0.192	0.286
Liberia	Sasstown	2000-2017	-0.160	-0.070	0.030
Liberia	St Paul River	2000	0.318	0.472	0.654
Liberia	St Paul River	2017	0.079	0.115	0.174
Liberia	St Paul River	2000-2017	-0.187	-0.103	-0.025
Liberia	Stjohnriver	2000	0.420	0.633	0.934
Liberia	Stjohnriver	2017	0.110	0.167	0.256
Liberia	Stjohnriver	2000-2017	-0.177	-0.093	-0.016
Liberia	Suakoko	2000	0.461	0.672	0.926
Liberia	Suakoko	2017	0.111	0.165	0.231
Liberia	Suakoko	2000-2017	-0.192	-0.112	-0.029
Liberia	Tappita	2000	0.538	0.746	1.014
Liberia	Tappita	2017	0.129	0.191	0.283
Liberia	Tappita	2000-2017	-0.188	-0.112	-0.036
Liberia	Tchien	2000	0.476	0.645	0.861
Liberia	Tchien	2017	0.116	0.182	0.264
Liberia	Tchien	2000-2017	-0.171	-0.098	-0.020
Liberia	Tewor	2000	0.421	0.603	0.861
Liberia	Tewor	2017	0.088	0.124	0.177
Liberia	Tewor	2000-2017	-0.192	-0.116	-0.025
Liberia	Timbo	2000	0.440	0.658	0.931
Liberia	Timbo	2017	0.117	0.181	0.271
Liberia	Timbo	2000-2017	-0.177	-0.092	-0.011
Liberia	Todee	2000	0.379	0.566	0.790
Liberia	Todee	2017	0.095	0.132	0.196
Liberia	Todee	2000-2017	-0.184	-0.109	-0.023
Liberia	Upperkrucoast	2000	0.386	0.582	0.835
Liberia	Upperkrucoast	2017	0.098	0.170	0.254
Liberia	Upperkrucoast	2000-2017	-0.157	-0.063	0.035
Liberia	Voinjama	2000	0.505	0.699	0.991
Liberia	Voinjama	2017	0.106	0.154	0.211
Liberia	Voinjama	2000-2017	-0.237	-0.151	-0.082
Liberia	Webbo	2000	0.495	0.700	0.961
Liberia	Webbo	2017	0.124	0.200	0.291
Liberia	Webbo	2000-2017	-0.160	-0.073	0.012
Liberia	Yarwein-Mehnsohnne	2000	0.512	0.733	1.013
Liberia	Yarwein-Mehnsohnne	2017	0.126	0.184	0.274
Liberia	Yarwein-Mehnsohnne	2000-2017	-0.194	-0.116	-0.039
Liberia	Zoegeh	2000	0.540	0.734	1.019
Liberia	Zoegeh	2017	0.125	0.189	0.270
Liberia	Zoegeh	2000-2017	-0.199	-0.119	-0.037
Liberia	Zorzor	2000	0.473	0.678	0.954
Liberia	Zorzor	2017	0.107	0.160	0.224
Liberia	Zorzor	2000-2017	-0.227	-0.141	-0.068
Liberia	Zota	2000	0.481	0.683	0.968
Liberia	Zota	2017	0.117	0.169	0.244
Liberia	Zota	2000-2017	-0.201	-0.121	-0.039
Madagascar	Alaotra-Mangoro	2000	0.357	0.475	0.606
Madagascar	Alaotra-Mangoro	2017	0.214	0.302	0.420
Madagascar	Alaotra-Mangoro	2000-2017	-0.046	-0.017	0.012
Madagascar	Amoron'i mania	2000	0.269	0.372	0.493
Madagascar	Amoron'i mania	2017	0.185	0.264	0.352
Madagascar	Amoron'i mania	2000-2017	-0.044	-0.015	0.015
Madagascar	Analamanga	2000	0.242	0.332	0.440
Madagascar	Analamanga	2017	0.169	0.253	0.354
Madagascar	Analamanga	2000-2017	-0.039	-0.006	0.025
Madagascar	Analanjirofo	2000	0.351	0.474	0.647
Madagascar	Analanjirofo	2017	0.204	0.301	0.425

Table 2: Diarrhea DALYs rate by unit *(continued)*

Country	Unit	year	mean rate	lower rate	upper rate
Madagascar	Analanjirofo	2000-2017	-0.052	-0.020	0.010
Madagascar	Androy	2000	0.514	0.706	0.929
Madagascar	Androy	2017	0.320	0.450	0.644
Madagascar	Androy	2000-2017	-0.052	-0.022	0.010
Madagascar	Anosy	2000	0.415	0.571	0.756
Madagascar	Anosy	2017	0.262	0.360	0.486
Madagascar	Anosy	2000-2017	-0.052	-0.024	0.003
Madagascar	Atsimo-Andrefana	2000	0.429	0.573	0.737
Madagascar	Atsimo-Andrefana	2017	0.237	0.332	0.458
Madagascar	Atsimo-Andrefana	2000-2017	-0.053	-0.025	0.001
Madagascar	Atsimo-Atsinana	2000	0.302	0.410	0.560
Madagascar	Atsimo-Atsinana	2017	0.199	0.282	0.392
Madagascar	Atsimo-Atsinana	2000-2017	-0.056	-0.024	0.005
Madagascar	Atsinanana	2000	0.335	0.441	0.576
Madagascar	Atsinanana	2017	0.199	0.276	0.368
Madagascar	Atsinanana	2000-2017	-0.047	-0.018	0.007
Madagascar	Betsiboka	2000	0.399	0.539	0.705
Madagascar	Betsiboka	2017	0.241	0.342	0.496
Madagascar	Betsiboka	2000-2017	-0.037	-0.010	0.022
Madagascar	Boeny	2000	0.400	0.560	0.741
Madagascar	Boeny	2017	0.230	0.345	0.499
Madagascar	Boeny	2000-2017	-0.042	-0.011	0.018
Madagascar	Bongolava	2000	0.276	0.383	0.517
Madagascar	Bongolava	2017	0.190	0.289	0.405
Madagascar	Bongolava	2000-2017	-0.043	-0.010	0.019
Madagascar	Diana	2000	0.341	0.479	0.661
Madagascar	Diana	2017	0.166	0.250	0.343
Madagascar	Diana	2000-2017	-0.068	-0.039	-0.009
Madagascar	Haute matsiatra	2000	0.300	0.420	0.591
Madagascar	Haute matsiatra	2017	0.194	0.281	0.382
Madagascar	Haute matsiatra	2000-2017	-0.052	-0.020	0.010
Madagascar	Ihorombe	2000	0.315	0.431	0.576
Madagascar	Ihorombe	2017	0.211	0.299	0.429
Madagascar	Ihorombe	2000-2017	-0.047	-0.014	0.016
Madagascar	Itasy	2000	0.258	0.360	0.475
Madagascar	Itasy	2017	0.178	0.273	0.385
Madagascar	Itasy	2000-2017	-0.043	-0.010	0.019
Madagascar	Melaky	2000	0.388	0.546	0.724
Madagascar	Melaky	2017	0.210	0.318	0.440
Madagascar	Melaky	2000-2017	-0.063	-0.035	-0.008
Madagascar	Menabe	2000	0.408	0.553	0.749
Madagascar	Menabe	2017	0.226	0.333	0.477
Madagascar	Menabe	2000-2017	-0.056	-0.027	0.003
Madagascar	Sava	2000	0.372	0.501	0.681
Madagascar	Sava	2017	0.189	0.279	0.402
Madagascar	Sava	2000-2017	-0.057	-0.032	-0.005
Madagascar	Sofia	2000	0.392	0.530	0.695
Madagascar	Sofia	2017	0.214	0.316	0.445
Madagascar	Sofia	2000-2017	-0.054	-0.022	0.007
Madagascar	Vakinankaratra	2000	0.258	0.344	0.453
Madagascar	Vakinankaratra	2017	0.167	0.254	0.345
Madagascar	Vakinankaratra	2000-2017	-0.043	-0.013	0.015
Madagascar	Vatovavy Fitovinany	2000	0.298	0.416	0.543
Madagascar	Vatovavy Fitovinany	2017	0.200	0.281	0.371
Madagascar	Vatovavy Fitovinany	2000-2017	-0.035	-0.004	0.025
Malawi	Balaka	2000	0.259	0.342	0.440
Malawi	Balaka	2017	0.064	0.083	0.107
Malawi	Balaka	2000-2017	-0.127	-0.095	-0.063
Malawi	Blantyre	2000	0.233	0.305	0.398
Malawi	Blantyre	2017	0.061	0.080	0.103
Malawi	Blantyre	2000-2017	-0.120	-0.092	-0.064

Table 2: Diarrhea DALYs rate by unit *(continued)*

Country	Unit	year	mean rate	lower rate	upper rate
Malawi	Chikwawa	2000	0.265	0.338	0.443
Malawi	Chikwawa	2017	0.061	0.080	0.106
Malawi	Chikwawa	2000-2017	-0.128	-0.098	-0.073
Malawi	Chiradzulu	2000	0.259	0.341	0.444
Malawi	Chiradzulu	2017	0.067	0.089	0.115
Malawi	Chiradzulu	2000-2017	-0.121	-0.094	-0.066
Malawi	Chitipa	2000	0.211	0.284	0.359
Malawi	Chitipa	2017	0.048	0.067	0.086
Malawi	Chitipa	2000-2017	-0.129	-0.099	-0.069
Malawi	Dedza	2000	0.299	0.379	0.472
Malawi	Dedza	2017	0.066	0.087	0.112
Malawi	Dedza	2000-2017	-0.127	-0.095	-0.067
Malawi	Dowa	2000	0.349	0.451	0.566
Malawi	Dowa	2017	0.072	0.095	0.123
Malawi	Dowa	2000-2017	-0.143	-0.110	-0.079
Malawi	Karonga	2000	0.190	0.256	0.331
Malawi	Karonga	2017	0.048	0.064	0.083
Malawi	Karonga	2000-2017	-0.124	-0.095	-0.066
Malawi	Kasungu	2000	0.336	0.433	0.566
Malawi	Kasungu	2017	0.073	0.095	0.124
Malawi	Kasungu	2000-2017	-0.136	-0.104	-0.076
Malawi	Likoma	2000	0.234	0.323	0.421
Malawi	Likoma	2017	0.044	0.060	0.080
Malawi	Likoma	2000-2017	-0.149	-0.114	-0.080
Malawi	Lilongwe	2000	0.317	0.408	0.510
Malawi	Lilongwe	2017	0.071	0.093	0.119
Malawi	Lilongwe	2000-2017	-0.130	-0.097	-0.067
Malawi	Machinga	2000	0.259	0.345	0.444
Malawi	Machinga	2017	0.070	0.089	0.111
Malawi	Machinga	2000-2017	-0.128	-0.097	-0.066
Malawi	Mangochi	2000	0.277	0.366	0.472
Malawi	Mangochi	2017	0.064	0.085	0.104
Malawi	Mangochi	2000-2017	-0.129	-0.097	-0.065
Malawi	Mchinji	2000	0.311	0.395	0.495
Malawi	Mchinji	2017	0.069	0.090	0.118
Malawi	Mchinji	2000-2017	-0.131	-0.101	-0.071
Malawi	Mulanje	2000	0.262	0.341	0.437
Malawi	Mulanje	2017	0.069	0.093	0.121
Malawi	Mulanje	2000-2017	-0.120	-0.092	-0.065
Malawi	Mwanza	2000	0.241	0.312	0.403
Malawi	Mwanza	2017	0.057	0.076	0.098
Malawi	Mwanza	2000-2017	-0.126	-0.091	-0.062
Malawi	Mzimba	2000	0.238	0.306	0.381
Malawi	Mzimba	2017	0.058	0.077	0.099
Malawi	Mzimba	2000-2017	-0.119	-0.090	-0.063
Malawi	Neno	2000	0.245	0.324	0.427
Malawi	Neno	2017	0.062	0.082	0.105
Malawi	Neno	2000-2017	-0.124	-0.089	-0.060
Malawi	Nkhata Bay	2000	0.228	0.300	0.381
Malawi	Nkhata Bay	2017	0.055	0.071	0.094
Malawi	Nkhata Bay	2000-2017	-0.126	-0.101	-0.069
Malawi	Nkhotakota	2000	0.309	0.405	0.514
Malawi	Nkhotakota	2017	0.068	0.090	0.115
Malawi	Nkhotakota	2000-2017	-0.136	-0.101	-0.072
Malawi	Nsanje	2000	0.263	0.341	0.439
Malawi	Nsanje	2017	0.062	0.084	0.111
Malawi	Nsanje	2000-2017	-0.126	-0.097	-0.074
Malawi	Ntcheu	2000	0.258	0.334	0.429
Malawi	Ntcheu	2017	0.060	0.080	0.103
Malawi	Ntcheu	2000-2017	-0.123	-0.090	-0.059
Malawi	Ntchisi	2000	0.353	0.447	0.569

Table 2: Diarrhea DALYs rate by unit *(continued)*

Country	Unit	year	mean rate	lower rate	upper rate
Malawi	Ntchisi	2017	0.069	0.093	0.121
Malawi	Ntchisi	2000-2017	-0.149	-0.113	-0.081
Malawi	Phalombe	2000	0.292	0.382	0.500
Malawi	Phalombe	2017	0.072	0.097	0.124
Malawi	Phalombe	2000-2017	-0.126	-0.098	-0.072
Malawi	Rumphi	2000	0.188	0.248	0.314
Malawi	Rumphi	2017	0.053	0.070	0.090
Malawi	Rumphi	2000-2017	-0.114	-0.085	-0.056
Malawi	Salima	2000	0.313	0.419	0.546
Malawi	Salima	2017	0.063	0.086	0.112
Malawi	Salima	2000-2017	-0.143	-0.108	-0.076
Malawi	Thyolo	2000	0.268	0.343	0.454
Malawi	Thyolo	2017	0.071	0.093	0.120
Malawi	Thyolo	2000-2017	-0.120	-0.092	-0.066
Malawi	Zomba	2000	0.262	0.344	0.454
Malawi	Zomba	2017	0.065	0.087	0.111
Malawi	Zomba	2000-2017	-0.126	-0.097	-0.070
Mali	Abeïbara	2000	0.419	0.675	1.004
Mali	Abeïbara	2017	0.183	0.293	0.445
Mali	Abeïbara	2000-2017	-0.186	-0.081	0.025
Mali	Ansongo	2000	0.516	0.804	1.100
Mali	Ansongo	2017	0.179	0.275	0.408
Mali	Ansongo	2000-2017	-0.184	-0.088	0.000
Mali	Bafoulabé	2000	0.470	0.681	0.924
Mali	Bafoulabé	2017	0.141	0.212	0.314
Mali	Bafoulabé	2000-2017	-0.172	-0.088	0.005
Mali	Bamako	2000	0.392	0.610	0.908
Mali	Bamako	2017	0.096	0.169	0.255
Mali	Bamako	2000-2017	-0.183	-0.083	0.004
Mali	Banamba	2000	0.438	0.641	0.921
Mali	Banamba	2017	0.104	0.175	0.266
Mali	Banamba	2000-2017	-0.188	-0.095	-0.009
Mali	Bandiagara	2000	0.452	0.715	1.047
Mali	Bandiagara	2017	0.129	0.205	0.315
Mali	Bandiagara	2000-2017	-0.212	-0.107	-0.001
Mali	Bankass	2000	0.448	0.691	0.994
Mali	Bankass	2017	0.131	0.207	0.349
Mali	Bankass	2000-2017	-0.204	-0.107	-0.005
Mali	Barouéli	2000	0.404	0.616	0.864
Mali	Barouéli	2017	0.130	0.221	0.333
Mali	Barouéli	2000-2017	-0.182	-0.078	0.010
Mali	Bla	2000	0.414	0.618	0.863
Mali	Bla	2017	0.137	0.236	0.353
Mali	Bla	2000-2017	-0.167	-0.077	0.006
Mali	Bougouni	2000	0.403	0.582	0.867
Mali	Bougouni	2017	0.120	0.188	0.284
Mali	Bougouni	2000-2017	-0.172	-0.076	-0.001
Mali	Bourem	2000	0.522	0.787	1.147
Mali	Bourem	2017	0.169	0.269	0.426
Mali	Bourem	2000-2017	-0.196	-0.097	-0.006
Mali	Diéma	2000	0.490	0.712	1.037
Mali	Diéma	2017	0.140	0.222	0.339
Mali	Diéma	2000-2017	-0.179	-0.095	-0.008
Mali	Dioïla	2000	0.404	0.588	0.834
Mali	Dioïla	2017	0.101	0.173	0.257
Mali	Dioïla	2000-2017	-0.190	-0.084	-0.002
Mali	Diré	2000	0.534	0.829	1.211
Mali	Diré	2017	0.166	0.272	0.425
Mali	Diré	2000-2017	-0.212	-0.098	0.007
Mali	Djenné	2000	0.438	0.671	0.953
Mali	Djenné	2017	0.129	0.204	0.333

Table 2: Diarrhea DALYs rate by unit *(continued)*

Country	Unit	year	mean rate	lower rate	upper rate
Mali	Djenné	2000-2017	-0.199	-0.105	-0.015
Mali	Douentza	2000	0.514	0.771	1.064
Mali	Douentza	2017	0.134	0.218	0.330
Mali	Douentza	2000-2017	-0.216	-0.112	-0.017
Mali	Gao	2000	0.505	0.761	1.067
Mali	Gao	2017	0.158	0.259	0.401
Mali	Gao	2000-2017	-0.199	-0.094	0.002
Mali	Goundam	2000	0.558	0.854	1.279
Mali	Goundam	2017	0.174	0.271	0.411
Mali	Goundam	2000-2017	-0.203	-0.100	-0.005
Mali	Gourma-Rharous	2000	0.509	0.798	1.093
Mali	Gourma-Rharous	2017	0.174	0.273	0.419
Mali	Gourma-Rharous	2000-2017	-0.194	-0.098	-0.009
Mali	Kadiolo	2000	0.401	0.563	0.774
Mali	Kadiolo	2017	0.111	0.177	0.255
Mali	Kadiolo	2000-2017	-0.169	-0.088	0.003
Mali	Kangaba	2000	0.356	0.562	0.865
Mali	Kangaba	2017	0.098	0.161	0.238
Mali	Kangaba	2000-2017	-0.182	-0.080	0.016
Mali	Kati	2000	0.391	0.601	0.903
Mali	Kati	2017	0.096	0.168	0.253
Mali	Kati	2000-2017	-0.182	-0.081	0.006
Mali	Kayes	2000	0.453	0.637	0.879
Mali	Kayes	2017	0.149	0.213	0.310
Mali	Kayes	2000-2017	-0.172	-0.096	-0.026
Mali	Kéniéba	2000	0.415	0.613	0.863
Mali	Kéniéba	2017	0.127	0.183	0.258
Mali	Kéniéba	2000-2017	-0.168	-0.079	0.023
Mali	Kidal	2000	0.449	0.759	1.133
Mali	Kidal	2017	0.166	0.262	0.401
Mali	Kidal	2000-2017	-0.189	-0.093	-0.005
Mali	Kita	2000	0.449	0.679	0.965
Mali	Kita	2017	0.129	0.204	0.306
Mali	Kita	2000-2017	-0.179	-0.091	0.006
Mali	Kolokani	2000	0.426	0.640	0.905
Mali	Kolokani	2017	0.102	0.170	0.268
Mali	Kolokani	2000-2017	-0.183	-0.090	-0.005
Mali	Kolondiéba	2000	0.417	0.595	0.866
Mali	Kolondiéba	2017	0.123	0.196	0.298
Mali	Kolondiéba	2000-2017	-0.175	-0.080	0.001
Mali	Koro	2000	0.486	0.746	1.098
Mali	Koro	2017	0.129	0.211	0.336
Mali	Koro	2000-2017	-0.205	-0.111	-0.020
Mali	Koulikoro	2000	0.412	0.606	0.866
Mali	Koulikoro	2017	0.100	0.171	0.254
Mali	Koulikoro	2000-2017	-0.196	-0.089	-0.001
Mali	Koutiala	2000	0.427	0.661	0.939
Mali	Koutiala	2017	0.135	0.224	0.340
Mali	Koutiala	2000-2017	-0.158	-0.081	0.004
Mali	Macina	2000	0.414	0.642	0.871
Mali	Macina	2017	0.145	0.243	0.410
Mali	Macina	2000-2017	-0.171	-0.082	0.010
Mali	Ménaka	2000	0.515	0.753	1.077
Mali	Ménaka	2017	0.167	0.261	0.386
Mali	Ménaka	2000-2017	-0.169	-0.085	0.002
Mali	Mopti	2000	0.471	0.723	1.019
Mali	Mopti	2017	0.125	0.202	0.306
Mali	Mopti	2000-2017	-0.215	-0.112	-0.016
Mali	Nara	2000	0.531	0.717	0.970
Mali	Nara	2017	0.111	0.181	0.274
Mali	Nara	2000-2017	-0.193	-0.105	-0.014

Table 2: Diarrhea DALYs rate by unit *(continued)*

Country	Unit	year	mean rate	lower rate	upper rate
Mali	Niafunké	2000	0.564	0.875	1.262
Mali	Niafunké	2017	0.169	0.272	0.417
Mali	Niafunké	2000-2017	-0.206	-0.102	0.002
Mali	Niono	2000	0.455	0.692	0.964
Mali	Niono	2017	0.141	0.234	0.374
Mali	Niono	2000-2017	-0.183	-0.093	0.011
Mali	Nioro	2000	0.470	0.670	0.915
Mali	Nioro	2017	0.135	0.226	0.344
Mali	Nioro	2000-2017	-0.173	-0.088	-0.002
Mali	San	2000	0.411	0.623	0.843
Mali	San	2017	0.158	0.243	0.384
Mali	San	2000-2017	-0.169	-0.079	0.013
Mali	Ségou	2000	0.414	0.632	0.893
Mali	Ségou	2017	0.137	0.234	0.358
Mali	Ségou	2000-2017	-0.174	-0.083	0.004
Mali	Sikasso	2000	0.426	0.631	0.869
Mali	Sikasso	2017	0.122	0.196	0.308
Mali	Sikasso	2000-2017	-0.170	-0.087	-0.001
Mali	Ténenkou	2000	0.457	0.681	0.952
Mali	Ténenkou	2017	0.127	0.205	0.339
Mali	Ténenkou	2000-2017	-0.203	-0.103	0.001
Mali	Tessalit	2000	0.416	0.689	0.994
Mali	Tessalit	2017	0.182	0.279	0.419
Mali	Tessalit	2000-2017	-0.177	-0.086	0.014
Mali	Tin-Essako	2000	0.472	0.749	1.155
Mali	Tin-Essako	2017	0.167	0.271	0.429
Mali	Tin-Essako	2000-2017	-0.185	-0.089	0.009
Mali	Tombouctou	2000	0.501	0.774	1.130
Mali	Tombouctou	2017	0.184	0.266	0.392
Mali	Tombouctou	2000-2017	-0.197	-0.096	-0.002
Mali	Tominian	2000	0.429	0.662	0.957
Mali	Tominian	2017	0.163	0.250	0.404
Mali	Tominian	2000-2017	-0.180	-0.088	0.007
Mali	Yanfolila	2000	0.355	0.526	0.781
Mali	Yanfolila	2017	0.114	0.176	0.267
Mali	Yanfolila	2000-2017	-0.181	-0.080	0.008
Mali	Yélimané	2000	0.436	0.602	0.861
Mali	Yélimané	2017	0.142	0.226	0.351
Mali	Yélimané	2000-2017	-0.167	-0.080	-0.004
Mali	Yorosso	2000	0.475	0.712	0.999
Mali	Yorosso	2017	0.149	0.233	0.353
Mali	Yorosso	2000-2017	-0.171	-0.089	0.000
Mali	Youwarou	2000	0.509	0.789	1.129
Mali	Youwarou	2017	0.132	0.214	0.323
Mali	Youwarou	2000-2017	-0.212	-0.109	0.003
Mauritania	Aïoun	2000	0.271	0.392	0.531
Mauritania	Aïoun	2017	0.053	0.084	0.134
Mauritania	Aïoun	2000-2017	-0.207	-0.126	-0.037
Mauritania	Akjoujt	2000	0.309	0.457	0.630
Mauritania	Akjoujt	2017	0.062	0.097	0.145
Mauritania	Akjoujt	2000-2017	-0.197	-0.111	-0.010
Mauritania	Aleg	2000	0.290	0.399	0.576
Mauritania	Aleg	2017	0.074	0.111	0.156
Mauritania	Aleg	2000-2017	-0.186	-0.100	-0.025
Mauritania	Amourj	2000	0.389	0.533	0.758
Mauritania	Amourj	2017	0.060	0.099	0.158
Mauritania	Amourj	2000-2017	-0.234	-0.136	-0.041
Mauritania	Aoujeft	2000	0.345	0.499	0.694
Mauritania	Aoujeft	2017	0.076	0.118	0.179
Mauritania	Aoujeft	2000-2017	-0.208	-0.112	-0.010
Mauritania	Atar	2000	0.349	0.505	0.731

Table 2: Diarrhea DALYs rate by unit *(continued)*

Country	Unit	year	mean rate	lower rate	upper rate
Mauritania	Atar	2017	0.072	0.113	0.177
Mauritania	Atar	2000-2017	-0.219	-0.122	-0.020
Mauritania	Bababé	2000	0.298	0.397	0.569
Mauritania	Bababé	2017	0.072	0.106	0.147
Mauritania	Bababé	2000-2017	-0.187	-0.105	-0.029
Mauritania	Barkéol	2000	0.330	0.452	0.620
Mauritania	Barkéol	2017	0.077	0.111	0.159
Mauritania	Barkéol	2000-2017	-0.173	-0.091	-0.010
Mauritania	Bassikounou	2000	0.383	0.564	0.785
Mauritania	Bassikounou	2017	0.064	0.111	0.176
Mauritania	Bassikounou	2000-2017	-0.242	-0.139	-0.038
Mauritania	Bir Moghreïn	2000	0.255	0.408	0.571
Mauritania	Bir Moghreïn	2017	0.063	0.098	0.152
Mauritania	Bir Moghreïn	2000-2017	-0.223	-0.126	-0.031
Mauritania	Boghé	2000	0.292	0.395	0.549
Mauritania	Boghé	2017	0.066	0.097	0.134
Mauritania	Boghé	2000-2017	-0.199	-0.113	-0.031
Mauritania	Boumdeïd	2000	0.253	0.388	0.556
Mauritania	Boumdeïd	2017	0.067	0.106	0.155
Mauritania	Boumdeïd	2000-2017	-0.149	-0.062	0.043
Mauritania	Boutilimit	2000	0.285	0.406	0.551
Mauritania	Boutilimit	2017	0.063	0.095	0.133
Mauritania	Boutilimit	2000-2017	-0.184	-0.108	-0.022
Mauritania	Chinguetti	2000	0.363	0.502	0.705
Mauritania	Chinguetti	2017	0.074	0.116	0.170
Mauritania	Chinguetti	2000-2017	-0.201	-0.114	-0.014
Mauritania	Djiguenni	2000	0.348	0.477	0.642
Mauritania	Djiguenni	2017	0.055	0.089	0.141
Mauritania	Djiguenni	2000-2017	-0.218	-0.127	-0.032
Mauritania	F'Dérik	2000	0.278	0.431	0.640
Mauritania	F'Dérik	2017	0.051	0.085	0.141
Mauritania	F'Dérik	2000-2017	-0.262	-0.161	-0.048
Mauritania	Guérou	2000	0.298	0.428	0.577
Mauritania	Guérou	2017	0.070	0.106	0.152
Mauritania	Guérou	2000-2017	-0.174	-0.087	0.004
Mauritania	Kaédi	2000	0.353	0.453	0.598
Mauritania	Kaédi	2017	0.085	0.119	0.164
Mauritania	Kaédi	2000-2017	-0.184	-0.097	-0.027
Mauritania	Kankossa	2000	0.297	0.409	0.562
Mauritania	Kankossa	2017	0.067	0.101	0.149
Mauritania	Kankossa	2000-2017	-0.186	-0.108	-0.030
Mauritania	Keur-Macène	2000	0.296	0.401	0.526
Mauritania	Keur-Macène	2017	0.062	0.089	0.122
Mauritania	Keur-Macène	2000-2017	-0.187	-0.116	-0.036
Mauritania	Kiffa	2000	0.286	0.397	0.538
Mauritania	Kiffa	2017	0.065	0.103	0.152
Mauritania	Kiffa	2000-2017	-0.167	-0.085	0.001
Mauritania	Kobenni	2000	0.325	0.443	0.596
Mauritania	Kobenni	2017	0.052	0.085	0.133
Mauritania	Kobenni	2000-2017	-0.209	-0.124	-0.039
Mauritania	M'Bagne	2000	0.320	0.420	0.582
Mauritania	M'Bagne	2017	0.074	0.108	0.150
Mauritania	M'Bagne	2000-2017	-0.191	-0.104	-0.031
Mauritania	M'Bout	2000	0.370	0.478	0.621
Mauritania	M'Bout	2017	0.085	0.120	0.173
Mauritania	M'Bout	2000-2017	-0.174	-0.093	-0.019
Mauritania	Maghama	2000	0.383	0.492	0.627
Mauritania	Maghama	2017	0.078	0.114	0.158
Mauritania	Maghama	2000-2017	-0.177	-0.105	-0.038
Mauritania	Magta-Lahjar	2000	0.296	0.417	0.590
Mauritania	Magta-Lahjar	2017	0.074	0.112	0.159

Table 2: Diarrhea DALYs rate by unit *(continued)*

Country	Unit	year	mean rate	lower rate	upper rate
Mauritania	Magta-Lahjar	2000-2017	-0.176	-0.088	0.000
Mauritania	Méderdra	2000	0.297	0.398	0.515
Mauritania	Méderdra	2017	0.059	0.089	0.124
Mauritania	Méderdra	2000-2017	-0.181	-0.114	-0.035
Mauritania	Monguel	2000	0.332	0.450	0.614
Mauritania	Monguel	2017	0.083	0.119	0.169
Mauritania	Monguel	2000-2017	-0.170	-0.086	-0.012
Mauritania	Moudjéria	2000	0.284	0.404	0.549
Mauritania	Moudjéria	2017	0.074	0.114	0.161
Mauritania	Moudjéria	2000-2017	-0.159	-0.068	0.024
Mauritania	Néma	2000	0.371	0.515	0.744
Mauritania	Néma	2017	0.057	0.092	0.144
Mauritania	Néma	2000-2017	-0.225	-0.128	-0.031
Mauritania	Nouadhibou	2000	0.295	0.442	0.618
Mauritania	Nouadhibou	2017	0.038	0.064	0.099
Mauritania	Nouadhibou	2000-2017	-0.233	-0.137	-0.044
Mauritania	Nouakchott	2000	0.337	0.458	0.603
Mauritania	Nouakchott	2017	0.058	0.088	0.130
Mauritania	Nouakchott	2000-2017	-0.188	-0.104	-0.019
Mauritania	Ouad-Naga	2000	0.332	0.445	0.594
Mauritania	Ouad-Naga	2017	0.059	0.089	0.129
Mauritania	Ouad-Naga	2000-2017	-0.186	-0.105	-0.027
Mauritania	Ouadane	2000	0.355	0.517	0.725
Mauritania	Ouadane	2017	0.066	0.097	0.148
Mauritania	Ouadane	2000-2017	-0.220	-0.126	-0.027
Mauritania	Ould Yengé	2000	0.363	0.486	0.632
Mauritania	Ould Yengé	2017	0.080	0.115	0.165
Mauritania	Ould Yengé	2000-2017	-0.192	-0.117	-0.041
Mauritania	R'Kiz	2000	0.304	0.401	0.543
Mauritania	R'Kiz	2017	0.059	0.090	0.127
Mauritania	R'Kiz	2000-2017	-0.195	-0.118	-0.034
Mauritania	Rosso	2000	0.288	0.384	0.520
Mauritania	Rosso	2017	0.059	0.088	0.127
Mauritania	Rosso	2000-2017	-0.186	-0.118	-0.034
Mauritania	Sélibaby	2000	0.396	0.522	0.684
Mauritania	Sélibaby	2017	0.079	0.117	0.165
Mauritania	Sélibaby	2000-2017	-0.192	-0.122	-0.055
Mauritania	Tamchakett	2000	0.253	0.375	0.512
Mauritania	Tamchakett	2017	0.060	0.090	0.137
Mauritania	Tamchakett	2000-2017	-0.195	-0.109	-0.011
Mauritania	Tichitt	2000	0.262	0.384	0.534
Mauritania	Tichitt	2017	0.067	0.104	0.153
Mauritania	Tichitt	2000-2017	-0.179	-0.082	0.019
Mauritania	Tidjikja	2000	0.276	0.402	0.553
Mauritania	Tidjikja	2017	0.072	0.114	0.165
Mauritania	Tidjikja	2000-2017	-0.155	-0.063	0.036
Mauritania	Timbédra	2000	0.366	0.492	0.678
Mauritania	Timbédra	2017	0.055	0.090	0.140
Mauritania	Timbédra	2000-2017	-0.224	-0.130	-0.035
Mauritania	Tintane	2000	0.275	0.404	0.559
Mauritania	Tintane	2017	0.054	0.086	0.137
Mauritania	Tintane	2000-2017	-0.204	-0.121	-0.040
Mauritania	Zouérate	2000	0.277	0.439	0.651
Mauritania	Zouérate	2017	0.054	0.092	0.151
Mauritania	Zouérate	2000-2017	-0.259	-0.156	-0.040
Mozambique	Alto Molocue	2000	0.231	0.312	0.410
Mozambique	Alto Molocue	2017	0.073	0.101	0.142
Mozambique	Alto Molocue	2000-2017	-0.077	-0.046	-0.019
Mozambique	Ancuabe	2000	0.217	0.316	0.426
Mozambique	Ancuabe	2017	0.055	0.077	0.106
Mozambique	Ancuabe	2000-2017	-0.107	-0.078	-0.048

Table 2: Diarrhea DALYs rate by unit *(continued)*

Country	Unit	year	mean rate	lower rate	upper rate
Mozambique	Angoche	2000	0.239	0.345	0.469
Mozambique	Angoche	2017	0.062	0.091	0.128
Mozambique	Angoche	2000-2017	-0.111	-0.080	-0.050
Mozambique	Angónia	2000	0.173	0.224	0.295
Mozambique	Angónia	2017	0.067	0.087	0.117
Mozambique	Angónia	2000-2017	-0.074	-0.039	-0.009
Mozambique	Balama	2000	0.198	0.287	0.367
Mozambique	Balama	2017	0.057	0.080	0.107
Mozambique	Balama	2000-2017	-0.103	-0.068	-0.039
Mozambique	Barue	2000	0.173	0.247	0.331
Mozambique	Barue	2017	0.057	0.082	0.110
Mozambique	Barue	2000-2017	-0.090	-0.063	-0.035
Mozambique	Bilene	2000	0.169	0.230	0.305
Mozambique	Bilene	2017	0.054	0.074	0.105
Mozambique	Bilene	2000-2017	-0.086	-0.052	-0.019
Mozambique	Boane	2000	0.178	0.235	0.310
Mozambique	Boane	2017	0.047	0.069	0.091
Mozambique	Boane	2000-2017	-0.097	-0.065	-0.032
Mozambique	Buzi	2000	0.196	0.265	0.352
Mozambique	Buzi	2017	0.057	0.078	0.105
Mozambique	Buzi	2000-2017	-0.090	-0.059	-0.029
Mozambique	Cahora Bassa	2000	0.153	0.213	0.289
Mozambique	Cahora Bassa	2017	0.054	0.076	0.103
Mozambique	Cahora Bassa	2000-2017	-0.086	-0.056	-0.027
Mozambique	Caia	2000	0.163	0.236	0.308
Mozambique	Caia	2017	0.056	0.079	0.106
Mozambique	Caia	2000-2017	-0.082	-0.053	-0.024
Mozambique	Changara	2000	0.152	0.215	0.291
Mozambique	Changara	2017	0.055	0.078	0.100
Mozambique	Changara	2000-2017	-0.078	-0.049	-0.022
Mozambique	Chemba	2000	0.174	0.239	0.322
Mozambique	Chemba	2017	0.060	0.079	0.107
Mozambique	Chemba	2000-2017	-0.076	-0.046	-0.018
Mozambique	Cheringoma	2000	0.176	0.244	0.324
Mozambique	Cheringoma	2017	0.053	0.075	0.101
Mozambique	Cheringoma	2000-2017	-0.088	-0.058	-0.029
Mozambique	Chibabava	2000	0.183	0.247	0.326
Mozambique	Chibabava	2017	0.055	0.077	0.107
Mozambique	Chibabava	2000-2017	-0.085	-0.057	-0.026
Mozambique	Chibuto	2000	0.163	0.218	0.291
Mozambique	Chibuto	2017	0.053	0.075	0.106
Mozambique	Chibuto	2000-2017	-0.078	-0.047	-0.014
Mozambique	Chicualacuala	2000	0.135	0.199	0.293
Mozambique	Chicualacuala	2017	0.048	0.067	0.094
Mozambique	Chicualacuala	2000-2017	-0.084	-0.053	-0.022
Mozambique	Chifunde	2000	0.193	0.257	0.347
Mozambique	Chifunde	2017	0.068	0.092	0.121
Mozambique	Chifunde	2000-2017	-0.084	-0.054	-0.025
Mozambique	Chigubo	2000	0.141	0.206	0.280
Mozambique	Chigubo	2017	0.048	0.067	0.096
Mozambique	Chigubo	2000-2017	-0.083	-0.052	-0.026
Mozambique	Chinde	2000	0.192	0.272	0.374
Mozambique	Chinde	2017	0.057	0.081	0.107
Mozambique	Chinde	2000-2017	-0.093	-0.060	-0.030
Mozambique	Chiúre	2000	0.225	0.324	0.434
Mozambique	Chiúre	2017	0.056	0.078	0.107
Mozambique	Chiúre	2000-2017	-0.107	-0.078	-0.047
Mozambique	Chiuta	2000	0.169	0.234	0.313
Mozambique	Chiuta	2017	0.058	0.081	0.106
Mozambique	Chiuta	2000-2017	-0.084	-0.051	-0.023
Mozambique	Chókwè	2000	0.162	0.224	0.299

Table 2: Diarrhea DALYs rate by unit *(continued)*

Country	Unit	year	mean rate	lower rate	upper rate
Mozambique	Chókwè	2017	0.055	0.078	0.110
Mozambique	Chókwè	2000-2017	-0.082	-0.048	-0.013
Mozambique	Cidade de Matola	2000	0.179	0.239	0.311
Mozambique	Cidade de Matola	2017	0.049	0.071	0.094
Mozambique	Cidade de Matola	2000-2017	-0.094	-0.062	-0.029
Mozambique	Cuamba	2000	0.209	0.282	0.369
Mozambique	Cuamba	2017	0.063	0.085	0.112
Mozambique	Cuamba	2000-2017	-0.104	-0.075	-0.045
Mozambique	Dondo	2000	0.171	0.230	0.322
Mozambique	Dondo	2017	0.053	0.076	0.103
Mozambique	Dondo	2000-2017	-0.084	-0.051	-0.018
Mozambique	Erati	2000	0.240	0.341	0.456
Mozambique	Erati	2017	0.057	0.082	0.111
Mozambique	Erati	2000-2017	-0.108	-0.080	-0.050
Mozambique	Funhalouro	2000	0.148	0.212	0.294
Mozambique	Funhalouro	2017	0.047	0.067	0.094
Mozambique	Funhalouro	2000-2017	-0.087	-0.055	-0.024
Mozambique	Gile	2000	0.229	0.321	0.426
Mozambique	Gile	2017	0.071	0.098	0.132
Mozambique	Gile	2000-2017	-0.088	-0.059	-0.030
Mozambique	Gondola	2000	0.185	0.249	0.336
Mozambique	Gondola	2017	0.059	0.081	0.112
Mozambique	Gondola	2000-2017	-0.087	-0.059	-0.032
Mozambique	Gorongosa	2000	0.187	0.251	0.347
Mozambique	Gorongosa	2017	0.058	0.081	0.115
Mozambique	Gorongosa	2000-2017	-0.082	-0.054	-0.028
Mozambique	Govuro	2000	0.153	0.222	0.315
Mozambique	Govuro	2017	0.051	0.073	0.100
Mozambique	Govuro	2000-2017	-0.083	-0.054	-0.024
Mozambique	Guijá	2000	0.155	0.214	0.288
Mozambique	Guijá	2017	0.052	0.072	0.105
Mozambique	Guijá	2000-2017	-0.080	-0.049	-0.014
Mozambique	Guro	2000	0.155	0.226	0.309
Mozambique	Guro	2017	0.055	0.078	0.101
Mozambique	Guro	2000-2017	-0.079	-0.050	-0.021
Mozambique	Gurue	2000	0.225	0.304	0.398
Mozambique	Gurue	2017	0.072	0.099	0.136
Mozambique	Gurue	2000-2017	-0.074	-0.047	-0.017
Mozambique	Homoine	2000	0.155	0.223	0.297
Mozambique	Homoine	2017	0.049	0.070	0.099
Mozambique	Homoine	2000-2017	-0.092	-0.059	-0.030
Mozambique	Ile	2000	0.210	0.288	0.383
Mozambique	Ile	2017	0.071	0.096	0.130
Mozambique	Ile	2000-2017	-0.084	-0.055	-0.026
Mozambique	Inharrime	2000	0.159	0.221	0.305
Mozambique	Inharrime	2017	0.050	0.072	0.102
Mozambique	Inharrime	2000-2017	-0.087	-0.056	-0.030
Mozambique	Inhassoro	2000	0.150	0.223	0.303
Mozambique	Inhassoro	2017	0.051	0.072	0.101
Mozambique	Inhassoro	2000-2017	-0.085	-0.053	-0.024
Mozambique	Inhassunge	2000	0.222	0.302	0.409
Mozambique	Inhassunge	2017	0.057	0.082	0.109
Mozambique	Inhassunge	2000-2017	-0.101	-0.067	-0.033
Mozambique	Jangamo	2000	0.153	0.221	0.300
Mozambique	Jangamo	2017	0.050	0.071	0.101
Mozambique	Jangamo	2000-2017	-0.091	-0.059	-0.031
Mozambique	Lago	2000	0.176	0.243	0.323
Mozambique	Lago	2017	0.055	0.076	0.101
Mozambique	Lago	2000-2017	-0.103	-0.067	-0.035
Mozambique	Lalaua	2000	0.234	0.323	0.422
Mozambique	Lalaua	2017	0.062	0.088	0.118

Table 2: Diarrhea DALYs rate by unit *(continued)*

Country	Unit	year	mean rate	lower rate	upper rate
Mozambique	Lalaua	2000-2017	-0.096	-0.064	-0.034
Mozambique	Lichinga	2000	0.197	0.267	0.361
Mozambique	Lichinga	2017	0.062	0.085	0.113
Mozambique	Lichinga	2000-2017	-0.101	-0.065	-0.031
Mozambique	Lugela	2000	0.222	0.305	0.405
Mozambique	Lugela	2017	0.070	0.097	0.128
Mozambique	Lugela	2000-2017	-0.069	-0.038	-0.009
Mozambique	Mabalane	2000	0.136	0.198	0.282
Mozambique	Mabalane	2017	0.048	0.068	0.102
Mozambique	Mabalane	2000-2017	-0.080	-0.048	-0.016
Mozambique	Mabote	2000	0.150	0.211	0.299
Mozambique	Mabote	2017	0.050	0.070	0.099
Mozambique	Mabote	2000-2017	-0.082	-0.049	-0.016
Mozambique	Macanga	2000	0.173	0.239	0.321
Mozambique	Macanga	2017	0.066	0.088	0.116
Mozambique	Macanga	2000-2017	-0.075	-0.041	-0.012
Mozambique	Machanga	2000	0.170	0.242	0.339
Mozambique	Machanga	2017	0.055	0.078	0.106
Mozambique	Machanga	2000-2017	-0.084	-0.056	-0.024
Mozambique	Machaze	2000	0.172	0.241	0.332
Mozambique	Machaze	2017	0.057	0.076	0.104
Mozambique	Machaze	2000-2017	-0.081	-0.054	-0.023
Mozambique	Macomia	2000	0.197	0.280	0.398
Mozambique	Macomia	2017	0.049	0.072	0.101
Mozambique	Macomia	2000-2017	-0.111	-0.078	-0.045
Mozambique	Macossa	2000	0.180	0.244	0.329
Mozambique	Macossa	2017	0.058	0.083	0.110
Mozambique	Macossa	2000-2017	-0.081	-0.053	-0.026
Mozambique	Maganja da Costa	2000	0.214	0.313	0.421
Mozambique	Maganja da Costa	2017	0.064	0.090	0.117
Mozambique	Maganja da Costa	2000-2017	-0.081	-0.050	-0.021
Mozambique	Magoe	2000	0.176	0.239	0.335
Mozambique	Magoe	2017	0.060	0.084	0.112
Mozambique	Magoe	2000-2017	-0.081	-0.053	-0.024
Mozambique	Magude	2000	0.154	0.214	0.282
Mozambique	Magude	2017	0.050	0.070	0.097
Mozambique	Magude	2000-2017	-0.090	-0.056	-0.024
Mozambique	Majune	2000	0.186	0.254	0.344
Mozambique	Majune	2017	0.060	0.082	0.110
Mozambique	Majune	2000-2017	-0.099	-0.066	-0.033
Mozambique	Malema	2000	0.222	0.296	0.394
Mozambique	Malema	2017	0.067	0.090	0.120
Mozambique	Malema	2000-2017	-0.089	-0.060	-0.033
Mozambique	Mandimba	2000	0.208	0.279	0.383
Mozambique	Mandimba	2017	0.060	0.082	0.111
Mozambique	Mandimba	2000-2017	-0.103	-0.068	-0.035
Mozambique	Mandlakazi	2000	0.160	0.219	0.301
Mozambique	Mandlakazi	2017	0.050	0.074	0.104
Mozambique	Mandlakazi	2000-2017	-0.082	-0.052	-0.023
Mozambique	Manhiça	2000	0.167	0.227	0.301
Mozambique	Manhiça	2017	0.051	0.073	0.097
Mozambique	Manhiça	2000-2017	-0.093	-0.059	-0.027
Mozambique	Manica	2000	0.176	0.243	0.324
Mozambique	Manica	2017	0.057	0.078	0.106
Mozambique	Manica	2000-2017	-0.096	-0.066	-0.038
Mozambique	Maputo	2000	0.185	0.247	0.326
Mozambique	Maputo	2017	0.051	0.073	0.097
Mozambique	Maputo	2000-2017	-0.093	-0.062	-0.030
Mozambique	Maravia	2000	0.174	0.240	0.338
Mozambique	Maravia	2017	0.065	0.087	0.116
Mozambique	Maravia	2000-2017	-0.082	-0.053	-0.022

Table 2: Diarrhea DALYs rate by unit *(continued)*

Country	Unit	year	mean rate	lower rate	upper rate
Mozambique	Maringue	2000	0.178	0.242	0.322
Mozambique	Maringue	2017	0.058	0.082	0.109
Mozambique	Maringue	2000-2017	-0.074	-0.046	-0.019
Mozambique	Marracuene	2000	0.189	0.253	0.337
Mozambique	Marracuene	2017	0.053	0.073	0.098
Mozambique	Marracuene	2000-2017	-0.096	-0.065	-0.033
Mozambique	Marromeu	2000	0.181	0.253	0.356
Mozambique	Marromeu	2017	0.056	0.082	0.109
Mozambique	Marromeu	2000-2017	-0.089	-0.056	-0.028
Mozambique	Marrupa	2000	0.208	0.289	0.395
Mozambique	Marrupa	2017	0.061	0.089	0.123
Mozambique	Marrupa	2000-2017	-0.104	-0.070	-0.043
Mozambique	Massangena	2000	0.137	0.202	0.292
Mozambique	Massangena	2017	0.048	0.067	0.094
Mozambique	Massangena	2000-2017	-0.081	-0.050	-0.019
Mozambique	Massinga	2000	0.164	0.230	0.319
Mozambique	Massinga	2017	0.050	0.071	0.104
Mozambique	Massinga	2000-2017	-0.093	-0.060	-0.031
Mozambique	Massingir	2000	0.141	0.208	0.297
Mozambique	Massingir	2017	0.049	0.070	0.100
Mozambique	Massingir	2000-2017	-0.081	-0.051	-0.020
Mozambique	Matutuíne	2000	0.186	0.247	0.328
Mozambique	Matutuíne	2017	0.047	0.068	0.091
Mozambique	Matutuíne	2000-2017	-0.098	-0.069	-0.040
Mozambique	Maúa	2000	0.200	0.278	0.378
Mozambique	Maúa	2017	0.057	0.083	0.112
Mozambique	Maúa	2000-2017	-0.107	-0.073	-0.039
Mozambique	Mavago	2000	0.174	0.240	0.336
Mozambique	Mavago	2017	0.058	0.083	0.113
Mozambique	Mavago	2000-2017	-0.089	-0.059	-0.029
Mozambique	Mecanhelas	2000	0.238	0.308	0.415
Mozambique	Mecanhelas	2017	0.075	0.098	0.127
Mozambique	Mecanhelas	2000-2017	-0.098	-0.066	-0.038
Mozambique	Meconta	2000	0.264	0.368	0.499
Mozambique	Meconta	2017	0.060	0.087	0.117
Mozambique	Meconta	2000-2017	-0.117	-0.089	-0.062
Mozambique	Mecuburi	2000	0.244	0.350	0.470
Mozambique	Mecuburi	2017	0.064	0.091	0.123
Mozambique	Mecuburi	2000-2017	-0.098	-0.068	-0.039
Mozambique	Mecufi	2000	0.216	0.309	0.418
Mozambique	Mecufi	2017	0.052	0.077	0.108
Mozambique	Mecufi	2000-2017	-0.110	-0.078	-0.048
Mozambique	Mecula	2000	0.179	0.255	0.342
Mozambique	Mecula	2017	0.060	0.085	0.113
Mozambique	Mecula	2000-2017	-0.088	-0.057	-0.028
Mozambique	Meluco	2000	0.217	0.302	0.416
Mozambique	Meluco	2017	0.056	0.078	0.104
Mozambique	Meluco	2000-2017	-0.106	-0.075	-0.044
Mozambique	Memba	2000	0.271	0.371	0.501
Mozambique	Memba	2017	0.057	0.084	0.113
Mozambique	Memba	2000-2017	-0.113	-0.086	-0.057
Mozambique	Metarica	2000	0.199	0.274	0.374
Mozambique	Metarica	2017	0.058	0.081	0.107
Mozambique	Metarica	2000-2017	-0.105	-0.073	-0.043
Mozambique	Milange	2000	0.224	0.296	0.382
Mozambique	Milange	2017	0.074	0.099	0.131
Mozambique	Milange	2000-2017	-0.074	-0.046	-0.019
Mozambique	Moamba	2000	0.160	0.213	0.293
Mozambique	Moamba	2017	0.045	0.064	0.087
Mozambique	Moamba	2000-2017	-0.093	-0.065	-0.033
Mozambique	Moatize	2000	0.158	0.218	0.278

Table 2: Diarrhea DALYs rate by unit *(continued)*

Country	Unit	year	mean rate	lower rate	upper rate
Mozambique	Moatize	2017	0.057	0.077	0.102
Mozambique	Moatize	2000-2017	-0.081	-0.049	-0.023
Mozambique	Mocimboa da Praia	2000	0.193	0.285	0.405
Mozambique	Mocimboa da Praia	2017	0.049	0.071	0.102
Mozambique	Mocimboa da Praia	2000-2017	-0.114	-0.078	-0.043
Mozambique	Mocuba	2000	0.205	0.285	0.382
Mozambique	Mocuba	2017	0.070	0.095	0.130
Mozambique	Mocuba	2000-2017	-0.083	-0.051	-0.018
Mozambique	Mogovolas	2000	0.239	0.341	0.458
Mozambique	Mogovolas	2017	0.060	0.086	0.117
Mozambique	Mogovolas	2000-2017	-0.110	-0.081	-0.052
Mozambique	Moma	2000	0.240	0.338	0.451
Mozambique	Moma	2017	0.067	0.097	0.134
Mozambique	Moma	2000-2017	-0.100	-0.069	-0.041
Mozambique	Monapo	2000	0.278	0.385	0.512
Mozambique	Monapo	2017	0.065	0.094	0.127
Mozambique	Monapo	2000-2017	-0.115	-0.087	-0.060
Mozambique	Mongincual	2000	0.245	0.346	0.477
Mozambique	Mongincual	2017	0.055	0.081	0.115
Mozambique	Mongincual	2000-2017	-0.119	-0.087	-0.057
Mozambique	Montepuez	2000	0.197	0.289	0.384
Mozambique	Montepuez	2017	0.053	0.074	0.097
Mozambique	Montepuez	2000-2017	-0.106	-0.073	-0.043
Mozambique	Mopeia	2000	0.209	0.291	0.389
Mozambique	Mopeia	2017	0.058	0.082	0.112
Mozambique	Mopeia	2000-2017	-0.091	-0.059	-0.030
Mozambique	Morrumbala	2000	0.222	0.294	0.381
Mozambique	Morrumbala	2017	0.065	0.088	0.115
Mozambique	Morrumbala	2000-2017	-0.082	-0.052	-0.026
Mozambique	Morrumbene	2000	0.163	0.227	0.309
Mozambique	Morrumbene	2017	0.048	0.069	0.101
Mozambique	Morrumbene	2000-2017	-0.097	-0.063	-0.034
Mozambique	Mossuril	2000	0.281	0.375	0.522
Mozambique	Mossuril	2017	0.058	0.086	0.118
Mozambique	Mossuril	2000-2017	-0.122	-0.093	-0.064
Mozambique	Mossurize	2000	0.174	0.249	0.343
Mozambique	Mossurize	2017	0.056	0.076	0.106
Mozambique	Mossurize	2000-2017	-0.088	-0.060	-0.030
Mozambique	Muanza	2000	0.178	0.242	0.323
Mozambique	Muanza	2017	0.050	0.073	0.099
Mozambique	Muanza	2000-2017	-0.094	-0.059	-0.028
Mozambique	Muecate	2000	0.274	0.375	0.510
Mozambique	Muecate	2017	0.063	0.090	0.117
Mozambique	Muecate	2000-2017	-0.111	-0.083	-0.055
Mozambique	Mueda	2000	0.191	0.268	0.368
Mozambique	Mueda	2017	0.054	0.078	0.104
Mozambique	Mueda	2000-2017	-0.097	-0.066	-0.031
Mozambique	Muembe	2000	0.171	0.246	0.344
Mozambique	Muembe	2017	0.060	0.082	0.113
Mozambique	Muembe	2000-2017	-0.101	-0.067	-0.035
Mozambique	Muidumbe	2000	0.190	0.270	0.376
Mozambique	Muidumbe	2017	0.051	0.073	0.101
Mozambique	Muidumbe	2000-2017	-0.107	-0.074	-0.038
Mozambique	Murrupula	2000	0.238	0.344	0.457
Mozambique	Murrupula	2017	0.066	0.093	0.125
Mozambique	Murrupula	2000-2017	-0.090	-0.060	-0.030
Mozambique	Mutarara	2000	0.188	0.252	0.330
Mozambique	Mutarara	2017	0.060	0.078	0.105
Mozambique	Mutarara	2000-2017	-0.084	-0.056	-0.032
Mozambique	N'gauma	2000	0.206	0.277	0.361
Mozambique	N'gauma	2017	0.062	0.085	0.115

Table 2: Diarrhea DALYs rate by unit *(continued)*

Country	Unit	year	mean rate	lower rate	upper rate
Mozambique	N'gauma	2000-2017	-0.097	-0.062	-0.024
Mozambique	Nacala Velha	2000	0.278	0.384	0.532
Mozambique	Nacala Velha	2017	0.061	0.088	0.125
Mozambique	Nacala Velha	2000-2017	-0.123	-0.094	-0.063
Mozambique	Nacaroa	2000	0.247	0.359	0.489
Mozambique	Nacaroa	2017	0.060	0.086	0.114
Mozambique	Nacaroa	2000-2017	-0.108	-0.082	-0.051
Mozambique	Namaacha	2000	0.180	0.236	0.318
Mozambique	Namaacha	2017	0.046	0.066	0.088
Mozambique	Namaacha	2000-2017	-0.100	-0.072	-0.040
Mozambique	Namacurra	2000	0.225	0.316	0.434
Mozambique	Namacurra	2017	0.062	0.087	0.113
Mozambique	Namacurra	2000-2017	-0.088	-0.057	-0.027
Mozambique	Namarroi	2000	0.222	0.308	0.404
Mozambique	Namarroi	2017	0.074	0.101	0.137
Mozambique	Namarroi	2000-2017	-0.073	-0.043	-0.012
Mozambique	Nampula	2000	0.276	0.383	0.520
Mozambique	Nampula	2017	0.065	0.091	0.120
Mozambique	Nampula	2000-2017	-0.113	-0.084	-0.052
Mozambique	Namuno	2000	0.216	0.315	0.412
Mozambique	Namuno	2017	0.059	0.082	0.108
Mozambique	Namuno	2000-2017	-0.100	-0.067	-0.036
Mozambique	Nangade	2000	0.187	0.267	0.360
Mozambique	Nangade	2017	0.055	0.076	0.105
Mozambique	Nangade	2000-2017	-0.101	-0.067	-0.031
Mozambique	Nhamatanda	2000	0.180	0.246	0.334
Mozambique	Nhamatanda	2017	0.054	0.076	0.105
Mozambique	Nhamatanda	2000-2017	-0.088	-0.054	-0.025
Mozambique	Nicoadala	2000	0.205	0.287	0.384
Mozambique	Nicoadala	2017	0.055	0.081	0.106
Mozambique	Nicoadala	2000-2017	-0.100	-0.066	-0.032
Mozambique	Nipepe	2000	0.222	0.305	0.427
Mozambique	Nipepe	2017	0.059	0.084	0.114
Mozambique	Nipepe	2000-2017	-0.100	-0.068	-0.037
Mozambique	Palma	2000	0.180	0.270	0.388
Mozambique	Palma	2017	0.048	0.072	0.107
Mozambique	Palma	2000-2017	-0.103	-0.068	-0.035
Mozambique	Panda	2000	0.152	0.211	0.289
Mozambique	Panda	2017	0.048	0.068	0.094
Mozambique	Panda	2000-2017	-0.085	-0.055	-0.025
Mozambique	Pebane	2000	0.211	0.296	0.391
Mozambique	Pebane	2017	0.061	0.088	0.120
Mozambique	Pebane	2000-2017	-0.083	-0.053	-0.026
Mozambique	Pemba	2000	0.215	0.301	0.405
Mozambique	Pemba	2017	0.052	0.076	0.110
Mozambique	Pemba	2000-2017	-0.112	-0.079	-0.047
Mozambique	Quissanga	2000	0.219	0.307	0.418
Mozambique	Quissanga	2017	0.053	0.075	0.105
Mozambique	Quissanga	2000-2017	-0.113	-0.080	-0.051
Mozambique	Ribaue	2000	0.244	0.325	0.426
Mozambique	Ribaue	2017	0.069	0.095	0.127
Mozambique	Ribaue	2000-2017	-0.091	-0.063	-0.035
Mozambique	Sanga	2000	0.171	0.241	0.331
Mozambique	Sanga	2017	0.058	0.080	0.109
Mozambique	Sanga	2000-2017	-0.100	-0.066	-0.034
Mozambique	Sussundenga	2000	0.187	0.254	0.328
Mozambique	Sussundenga	2017	0.061	0.080	0.106
Mozambique	Sussundenga	2000-2017	-0.088	-0.060	-0.034
Mozambique	Tambara	2000	0.169	0.236	0.322
Mozambique	Tambara	2017	0.057	0.078	0.103
Mozambique	Tambara	2000-2017	-0.076	-0.045	-0.013

Table 2: Diarrhea DALYs rate by unit *(continued)*

Country	Unit	year	mean rate	lower rate	upper rate
Mozambique	Tsangano	2000	0.155	0.203	0.271
Mozambique	Tsangano	2017	0.060	0.081	0.109
Mozambique	Tsangano	2000-2017	-0.073	-0.039	-0.009
Mozambique	Vilanculos	2000	0.157	0.226	0.319
Mozambique	Vilanculos	2017	0.050	0.072	0.102
Mozambique	Vilanculos	2000-2017	-0.088	-0.055	-0.025
Mozambique	Xai-Xai	2000	0.167	0.231	0.302
Mozambique	Xai-Xai	2017	0.055	0.077	0.106
Mozambique	Xai-Xai	2000-2017	-0.082	-0.051	-0.018
Mozambique	Zavala	2000	0.162	0.226	0.309
Mozambique	Zavala	2017	0.051	0.074	0.105
Mozambique	Zavala	2000-2017	-0.088	-0.056	-0.029
Mozambique	Zumbu	2000	0.177	0.251	0.356
Mozambique	Zumbu	2017	0.065	0.091	0.120
Mozambique	Zumbu	2000-2017	-0.073	-0.047	-0.014
Namibia	Aminius	2000	0.138	0.152	0.167
Namibia	Aminius	2017	0.095	0.107	0.120
Namibia	Aminius	2000-2017	-0.017	-0.010	-0.003
Namibia	Anamulenge	2000	0.127	0.138	0.151
Namibia	Anamulenge	2017	0.086	0.096	0.108
Namibia	Anamulenge	2000-2017	-0.019	-0.011	-0.003
Namibia	Arandis	2000	0.110	0.119	0.129
Namibia	Arandis	2017	0.068	0.079	0.091
Namibia	Arandis	2000-2017	-0.020	-0.012	-0.004
Namibia	Berseba	2000	0.139	0.151	0.165
Namibia	Berseba	2017	0.086	0.098	0.111
Namibia	Berseba	2000-2017	-0.018	-0.011	-0.006
Namibia	Daures	2000	0.118	0.127	0.138
Namibia	Daures	2017	0.074	0.083	0.093
Namibia	Daures	2000-2017	-0.020	-0.012	-0.004
Namibia	Eenhana	2000	0.131	0.144	0.160
Namibia	Eenhana	2017	0.088	0.101	0.116
Namibia	Eenhana	2000-2017	-0.019	-0.011	-0.002
Namibia	Elim	2000	0.125	0.136	0.149
Namibia	Elim	2017	0.082	0.092	0.103
Namibia	Elim	2000-2017	-0.019	-0.011	-0.004
Namibia	Endola	2000	0.128	0.140	0.154
Namibia	Endola	2017	0.083	0.096	0.109
Namibia	Endola	2000-2017	-0.021	-0.013	-0.005
Namibia	Engela	2000	0.128	0.142	0.156
Namibia	Engela	2017	0.084	0.096	0.111
Namibia	Engela	2000-2017	-0.019	-0.011	-0.003
Namibia	Engodi	2000	0.144	0.161	0.180
Namibia	Engodi	2017	0.100	0.116	0.131
Namibia	Engodi	2000-2017	-0.016	-0.009	-0.002
Namibia	Epembe	2000	0.136	0.150	0.167
Namibia	Epembe	2017	0.095	0.109	0.124
Namibia	Epembe	2000-2017	-0.017	-0.009	-0.001
Namibia	Epukiro	2000	0.142	0.158	0.175
Namibia	Epukiro	2017	0.096	0.109	0.124
Namibia	Epukiro	2000-2017	-0.017	-0.010	-0.004
Namibia	Epupa	2000	0.151	0.164	0.179
Namibia	Epupa	2017	0.109	0.127	0.146
Namibia	Epupa	2000-2017	-0.011	-0.003	0.004
Namibia	Etayi	2000	0.126	0.137	0.149
Namibia	Etayi	2017	0.082	0.093	0.104
Namibia	Etayi	2000-2017	-0.019	-0.011	-0.003
Namibia	Gibeon	2000	0.133	0.145	0.157
Namibia	Gibeon	2017	0.084	0.094	0.105
Namibia	Gibeon	2000-2017	-0.018	-0.012	-0.006
Namibia	Gobabis	2000	0.130	0.145	0.162

Table 2: Diarrhea DALYs rate by unit *(continued)*

Country	Unit	year	mean rate	lower rate	upper rate
Namibia	Gobabis	2017	0.084	0.097	0.111
Namibia	Gobabis	2000-2017	-0.019	-0.010	-0.001
Namibia	Grootfontein	2000	0.146	0.162	0.181
Namibia	Grootfontein	2017	0.095	0.109	0.124
Namibia	Grootfontein	2000-2017	-0.017	-0.009	-0.002
Namibia	Guinas	2000	0.145	0.161	0.179
Namibia	Guinas	2017	0.099	0.112	0.127
Namibia	Guinas	2000-2017	-0.016	-0.010	-0.003
Namibia	Kabe	2000	0.131	0.145	0.159
Namibia	Kabe	2017	0.093	0.106	0.118
Namibia	Kabe	2000-2017	-0.017	-0.009	-0.003
Namibia	Kahenge	2000	0.158	0.178	0.199
Namibia	Kahenge	2017	0.109	0.131	0.154
Namibia	Kahenge	2000-2017	-0.015	-0.008	-0.002
Namibia	Kalahari	2000	0.135	0.149	0.163
Namibia	Kalahari	2017	0.088	0.099	0.111
Namibia	Kalahari	2000-2017	-0.017	-0.010	-0.004
Namibia	Kamanjab	2000	0.141	0.157	0.175
Namibia	Kamanjab	2017	0.090	0.106	0.122
Namibia	Kamanjab	2000-2017	-0.017	-0.009	-0.003
Namibia	Kapako	2000	0.167	0.189	0.211
Namibia	Kapako	2017	0.115	0.140	0.165
Namibia	Kapako	2000-2017	-0.014	-0.007	-0.001
Namibia	Karas	2000	0.130	0.141	0.154
Namibia	Karas	2017	0.084	0.092	0.100
Namibia	Karas	2000-2017	-0.016	-0.011	-0.006
Namibia	Karibib	2000	0.116	0.125	0.135
Namibia	Karibib	2017	0.072	0.081	0.091
Namibia	Karibib	2000-2017	-0.021	-0.012	-0.004
Namibia	Katima Muliro Rural	2000	0.130	0.143	0.157
Namibia	Katima Muliro Rural	2017	0.091	0.101	0.113
Namibia	Katima Muliro Rural	2000-2017	-0.018	-0.010	-0.003
Namibia	Katima Muliro Urban	2000	0.129	0.145	0.161
Namibia	Katima Muliro Urban	2017	0.090	0.101	0.114
Namibia	Katima Muliro Urban	2000-2017	-0.020	-0.010	-0.001
Namibia	Katutura Central	2000	0.112	0.127	0.144
Namibia	Katutura Central	2017	0.074	0.088	0.102
Namibia	Katutura Central	2000-2017	-0.024	-0.014	-0.005
Namibia	Katutura East	2000	0.112	0.127	0.144
Namibia	Katutura East	2017	0.074	0.088	0.102
Namibia	Katutura East	2000-2017	-0.024	-0.014	-0.005
Namibia	Keetmanshoop Rural	2000	0.138	0.149	0.162
Namibia	Keetmanshoop Rural	2017	0.087	0.097	0.109
Namibia	Keetmanshoop Rural	2000-2017	-0.017	-0.012	-0.006
Namibia	Keetmanshoop Urban	2000	0.137	0.152	0.170
Namibia	Keetmanshoop Urban	2017	0.084	0.099	0.113
Namibia	Keetmanshoop Urban	2000-2017	-0.022	-0.012	-0.003
Namibia	Khomasdal North	2000	0.110	0.124	0.139
Namibia	Khomasdal North	2017	0.073	0.086	0.100
Namibia	Khomasdal North	2000-2017	-0.024	-0.014	-0.005
Namibia	Khorixas	2000	0.128	0.144	0.162
Namibia	Khorixas	2017	0.079	0.095	0.111
Namibia	Khorixas	2000-2017	-0.017	-0.009	0.000
Namibia	Kongola	2000	0.139	0.153	0.168
Namibia	Kongola	2017	0.096	0.108	0.123
Namibia	Kongola	2000-2017	-0.017	-0.009	-0.003
Namibia	Linyandi	2000	0.138	0.152	0.165
Namibia	Linyandi	2017	0.094	0.105	0.119
Namibia	Linyandi	2000-2017	-0.016	-0.010	-0.003
Namibia	Luderitz	2000	0.148	0.166	0.188
Namibia	Luderitz	2017	0.097	0.113	0.132

Table 2: Diarrhea DALYs rate by unit *(continued)*

Country	Unit	year	mean rate	lower rate	upper rate
Namibia	Luderitz	2000-2017	-0.020	-0.011	-0.003
Namibia	Mariental Rural	2000	0.130	0.141	0.153
Namibia	Mariental Rural	2017	0.082	0.092	0.103
Namibia	Mariental Rural	2000-2017	-0.018	-0.012	-0.005
Namibia	Mariental Urban	2000	0.126	0.139	0.151
Namibia	Mariental Urban	2017	0.080	0.090	0.101
Namibia	Mariental Urban	2000-2017	-0.021	-0.013	-0.004
Namibia	Mashare	2000	0.172	0.194	0.219
Namibia	Mashare	2017	0.120	0.143	0.166
Namibia	Mashare	2000-2017	-0.016	-0.009	-0.003
Namibia	Moses Garoeb	2000	0.111	0.123	0.137
Namibia	Moses Garoeb	2017	0.074	0.086	0.099
Namibia	Moses Garoeb	2000-2017	-0.024	-0.014	-0.005
Namibia	Mpungu	2000	0.149	0.166	0.184
Namibia	Mpungu	2017	0.106	0.123	0.143
Namibia	Mpungu	2000-2017	-0.015	-0.008	-0.002
Namibia	Mukwe	2000	0.167	0.190	0.217
Namibia	Mukwe	2017	0.115	0.138	0.164
Namibia	Mukwe	2000-2017	-0.019	-0.012	-0.005
Namibia	Ndiyona	2000	0.173	0.194	0.221
Namibia	Ndiyona	2017	0.117	0.141	0.165
Namibia	Ndiyona	2000-2017	-0.017	-0.010	-0.004
Namibia	Ogongo	2000	0.129	0.140	0.153
Namibia	Ogongo	2017	0.085	0.095	0.107
Namibia	Ogongo	2000-2017	-0.019	-0.011	-0.003
Namibia	Ohangwena	2000	0.127	0.141	0.154
Namibia	Ohangwena	2017	0.083	0.095	0.109
Namibia	Ohangwena	2000-2017	-0.021	-0.012	-0.004
Namibia	Okahandja	2000	0.117	0.132	0.149
Namibia	Okahandja	2017	0.079	0.092	0.106
Namibia	Okahandja	2000-2017	-0.017	-0.009	0.001
Namibia	Okahao	2000	0.133	0.145	0.159
Namibia	Okahao	2017	0.087	0.098	0.110
Namibia	Okahao	2000-2017	-0.019	-0.011	-0.002
Namibia	Okakarara	2000	0.134	0.146	0.160
Namibia	Okakarara	2017	0.087	0.097	0.109
Namibia	Okakarara	2000-2017	-0.016	-0.010	-0.003
Namibia	Okaku	2000	0.116	0.126	0.137
Namibia	Okaku	2017	0.075	0.085	0.095
Namibia	Okaku	2000-2017	-0.021	-0.013	-0.005
Namibia	Okalongo	2000	0.126	0.137	0.150
Namibia	Okalongo	2017	0.084	0.094	0.106
Namibia	Okalongo	2000-2017	-0.020	-0.012	-0.003
Namibia	Okankolo	2000	0.142	0.157	0.174
Namibia	Okankolo	2017	0.098	0.113	0.128
Namibia	Okankolo	2000-2017	-0.015	-0.009	-0.002
Namibia	Okatana	2000	0.120	0.130	0.141
Namibia	Okatana	2017	0.078	0.088	0.099
Namibia	Okatana	2000-2017	-0.021	-0.012	-0.004
Namibia	Okatyali	2000	0.124	0.135	0.147
Namibia	Okatyali	2017	0.084	0.094	0.105
Namibia	Okatyali	2000-2017	-0.019	-0.010	-0.002
Namibia	Okongo	2000	0.144	0.160	0.180
Namibia	Okongo	2017	0.102	0.119	0.135
Namibia	Okongo	2000-2017	-0.014	-0.007	0.001
Namibia	Olukonda	2000	0.124	0.136	0.149
Namibia	Olukonda	2017	0.082	0.094	0.107
Namibia	Olukonda	2000-2017	-0.019	-0.010	0.000
Namibia	Omaruru	2000	0.117	0.127	0.140
Namibia	Omaruru	2017	0.074	0.083	0.092
Namibia	Omaruru	2000-2017	-0.020	-0.011	-0.002

Table 2: Diarrhea DALYs rate by unit *(continued)*

Country	Unit	year	mean rate	lower rate	upper rate
Namibia	Omatako	2000	0.126	0.137	0.150
Namibia	Omatako	2017	0.082	0.092	0.105
Namibia	Omatako	2000-2017	-0.015	-0.009	-0.002
Namibia	Ompundja	2000	0.122	0.133	0.146
Namibia	Ompundja	2017	0.080	0.091	0.102
Namibia	Ompundja	2000-2017	-0.019	-0.011	-0.002
Namibia	Omulonga	2000	0.130	0.143	0.157
Namibia	Omulonga	2017	0.085	0.098	0.112
Namibia	Omulonga	2000-2017	-0.022	-0.012	-0.004
Namibia	Omundaungilo	2000	0.137	0.152	0.169
Namibia	Omundaungilo	2017	0.093	0.107	0.123
Namibia	Omundaungilo	2000-2017	-0.017	-0.009	-0.001
Namibia	Omuntele	2000	0.134	0.145	0.158
Namibia	Omuntele	2017	0.090	0.101	0.113
Namibia	Omuntele	2000-2017	-0.019	-0.011	-0.002
Namibia	Omuthiyagwipundi	2000	0.133	0.145	0.159
Namibia	Omuthiyagwipundi	2017	0.090	0.102	0.114
Namibia	Omuthiyagwipundi	2000-2017	-0.019	-0.011	-0.003
Namibia	Onayena	2000	0.126	0.138	0.150
Namibia	Onayena	2017	0.085	0.096	0.107
Namibia	Onayena	2000-2017	-0.019	-0.011	-0.002
Namibia	Ondangwa	2000	0.113	0.125	0.136
Namibia	Ondangwa	2017	0.074	0.084	0.096
Namibia	Ondangwa	2000-2017	-0.019	-0.011	-0.002
Namibia	Ondobe	2000	0.130	0.144	0.158
Namibia	Ondobe	2017	0.086	0.099	0.113
Namibia	Ondobe	2000-2017	-0.019	-0.011	-0.001
Namibia	Onesi	2000	0.129	0.141	0.154
Namibia	Onesi	2017	0.092	0.102	0.115
Namibia	Onesi	2000-2017	-0.016	-0.009	0.000
Namibia	Ongenga	2000	0.129	0.143	0.156
Namibia	Ongenga	2017	0.085	0.098	0.112
Namibia	Ongenga	2000-2017	-0.020	-0.012	-0.003
Namibia	Ongwediva	2000	0.115	0.126	0.137
Namibia	Ongwediva	2017	0.075	0.085	0.095
Namibia	Ongwediva	2000-2017	-0.022	-0.012	-0.004
Namibia	Oniipa	2000	0.124	0.136	0.147
Namibia	Oniipa	2017	0.080	0.092	0.104
Namibia	Oniipa	2000-2017	-0.020	-0.012	-0.004
Namibia	Onyaanya	2000	0.127	0.138	0.151
Namibia	Onyaanya	2017	0.087	0.098	0.109
Namibia	Onyaanya	2000-2017	-0.019	-0.011	-0.002
Namibia	Opuwo	2000	0.143	0.154	0.168
Namibia	Opuwo	2017	0.102	0.117	0.133
Namibia	Opuwo	2000-2017	-0.012	-0.004	0.004
Namibia	Oranjemund	2000	0.145	0.165	0.186
Namibia	Oranjemund	2017	0.093	0.111	0.127
Namibia	Oranjemund	2000-2017	-0.018	-0.012	-0.005
Namibia	Oshakati East	2000	0.117	0.127	0.140
Namibia	Oshakati East	2017	0.076	0.087	0.098
Namibia	Oshakati East	2000-2017	-0.021	-0.012	-0.004
Namibia	Oshakati West	2000	0.115	0.125	0.137
Namibia	Oshakati West	2017	0.075	0.085	0.096
Namibia	Oshakati West	2000-2017	-0.022	-0.012	-0.004
Namibia	Oshikango	2000	0.130	0.143	0.157
Namibia	Oshikango	2017	0.083	0.096	0.111
Namibia	Oshikango	2000-2017	-0.020	-0.011	-0.004
Namibia	Oshikuku	2000	0.125	0.136	0.149
Namibia	Oshikuku	2017	0.081	0.092	0.103
Namibia	Oshikuku	2000-2017	-0.019	-0.012	-0.003
Namibia	Otamanzi	2000	0.141	0.154	0.170

Table 2: Diarrhea DALYs rate by unit *(continued)*

Country	Unit	year	mean rate	lower rate	upper rate
Namibia	Otamanzi	2017	0.091	0.104	0.118
Namibia	Otamanzi	2000-2017	-0.019	-0.011	-0.002
Namibia	Otavi	2000	0.134	0.146	0.160
Namibia	Otavi	2017	0.084	0.097	0.110
Namibia	Otavi	2000-2017	-0.019	-0.011	-0.004
Namibia	Otjinene	2000	0.134	0.147	0.162
Namibia	Otjinene	2017	0.087	0.100	0.114
Namibia	Otjinene	2000-2017	-0.019	-0.011	-0.003
Namibia	Otjiwarongo	2000	0.125	0.138	0.152
Namibia	Otjiwarongo	2017	0.085	0.096	0.109
Namibia	Otjiwarongo	2000-2017	-0.016	-0.006	0.004
Namibia	Otjombinde	2000	0.152	0.169	0.188
Namibia	Otjombinde	2017	0.103	0.119	0.139
Namibia	Otjombinde	2000-2017	-0.014	-0.008	-0.002
Namibia	Outapi	2000	0.129	0.140	0.152
Namibia	Outapi	2017	0.090	0.100	0.112
Namibia	Outapi	2000-2017	-0.018	-0.011	-0.002
Namibia	Outjo	2000	0.132	0.147	0.165
Namibia	Outjo	2017	0.085	0.100	0.114
Namibia	Outjo	2000-2017	-0.016	-0.007	0.001
Namibia	Rehoboth East	2000	0.115	0.126	0.137
Namibia	Rehoboth East	2017	0.073	0.083	0.094
Namibia	Rehoboth East	2000-2017	-0.022	-0.012	-0.003
Namibia	Rehoboth Rural	2000	0.124	0.135	0.145
Namibia	Rehoboth Rural	2017	0.079	0.088	0.099
Namibia	Rehoboth Rural	2000-2017	-0.022	-0.013	-0.005
Namibia	Rehoboth West	2000	0.114	0.126	0.136
Namibia	Rehoboth West	2017	0.072	0.083	0.093
Namibia	Rehoboth West	2000-2017	-0.021	-0.011	-0.002
Namibia	Ruacana	2000	0.136	0.147	0.160
Namibia	Ruacana	2017	0.096	0.108	0.123
Namibia	Ruacana	2000-2017	-0.014	-0.007	0.000
Namibia	Rundu Rural East	2000	0.166	0.187	0.210
Namibia	Rundu Rural East	2017	0.117	0.140	0.165
Namibia	Rundu Rural East	2000-2017	-0.014	-0.007	0.000
Namibia	Rundu Rural West	2000	0.166	0.190	0.218
Namibia	Rundu Rural West	2017	0.115	0.141	0.169
Namibia	Rundu Rural West	2000-2017	-0.014	-0.007	0.001
Namibia	Rundu Urban	2000	0.165	0.190	0.217
Namibia	Rundu Urban	2017	0.115	0.141	0.169
Namibia	Rundu Urban	2000-2017	-0.014	-0.007	0.001
Namibia	Sesfontein	2000	0.147	0.167	0.186
Namibia	Sesfontein	2017	0.102	0.120	0.138
Namibia	Sesfontein	2000-2017	-0.016	-0.009	-0.003
Namibia	Sibinda	2000	0.133	0.145	0.159
Namibia	Sibinda	2017	0.091	0.101	0.113
Namibia	Sibinda	2000-2017	-0.020	-0.010	-0.003
Namibia	Soweto	2000	0.112	0.127	0.144
Namibia	Soweto	2017	0.074	0.088	0.102
Namibia	Soweto	2000-2017	-0.024	-0.014	-0.005
Namibia	Steinhausen	2000	0.131	0.143	0.156
Namibia	Steinhausen	2017	0.084	0.095	0.107
Namibia	Steinhausen	2000-2017	-0.018	-0.011	-0.006
Namibia	Swakopmund	2000	0.108	0.119	0.130
Namibia	Swakopmund	2017	0.066	0.077	0.088
Namibia	Swakopmund	2000-2017	-0.022	-0.012	-0.002
Namibia	Tobias Hainyeko	2000	0.111	0.125	0.141
Namibia	Tobias Hainyeko	2017	0.074	0.086	0.100
Namibia	Tobias Hainyeko	2000-2017	-0.024	-0.014	-0.005
Namibia	Tsandi	2000	0.130	0.142	0.156
Namibia	Tsandi	2017	0.088	0.098	0.109

Table 2: Diarrhea DALYs rate by unit *(continued)*

Country	Unit	year	mean rate	lower rate	upper rate
Namibia	Tsandi	2000-2017	-0.020	-0.012	-0.003
Namibia	Tsumeb	2000	0.147	0.165	0.181
Namibia	Tsumeb	2017	0.096	0.109	0.123
Namibia	Tsumeb	2000-2017	-0.022	-0.013	-0.003
Namibia	Tsumkwe	2000	0.159	0.176	0.194
Namibia	Tsumkwe	2017	0.110	0.126	0.142
Namibia	Tsumkwe	2000-2017	-0.015	-0.009	-0.003
Namibia	Uukwiyu	2000	0.117	0.129	0.140
Namibia	Uukwiyu	2017	0.077	0.088	0.099
Namibia	Uukwiyu	2000-2017	-0.019	-0.011	-0.002
Namibia	Uuvudhiya	2000	0.140	0.154	0.170
Namibia	Uuvudhiya	2017	0.089	0.103	0.119
Namibia	Uuvudhiya	2000-2017	-0.020	-0.012	-0.004
Namibia	Walvisbay Rural	2000	0.105	0.116	0.129
Namibia	Walvisbay Rural	2017	0.066	0.078	0.089
Namibia	Walvisbay Rural	2000-2017	-0.022	-0.012	-0.003
Namibia	Walvisbay Urban	2000	0.103	0.115	0.127
Namibia	Walvisbay Urban	2017	0.068	0.079	0.090
Namibia	Walvisbay Urban	2000-2017	-0.022	-0.011	-0.001
Namibia	Wanaheda	2000	0.110	0.123	0.138
Namibia	Wanaheda	2017	0.074	0.087	0.101
Namibia	Wanaheda	2000-2017	-0.025	-0.014	-0.005
Namibia	Windhoek East	2000	0.107	0.117	0.128
Namibia	Windhoek East	2017	0.072	0.081	0.091
Namibia	Windhoek East	2000-2017	-0.021	-0.012	-0.002
Namibia	Windhoek Rural	2000	0.112	0.123	0.135
Namibia	Windhoek Rural	2017	0.074	0.083	0.093
Namibia	Windhoek Rural	2000-2017	-0.022	-0.013	-0.006
Namibia	Windhoek West	2000	0.108	0.120	0.133
Namibia	Windhoek West	2017	0.072	0.083	0.096
Namibia	Windhoek West	2000-2017	-0.023	-0.013	-0.003
Niger	Aguié	2000	0.966	1.295	1.656
Niger	Aguié	2017	0.210	0.329	0.512
Niger	Aguié	2000-2017	-0.195	-0.116	-0.036
Niger	Arlit	2000	0.490	0.701	1.000
Niger	Arlit	2017	0.321	0.493	0.703
Niger	Arlit	2000-2017	-0.165	-0.067	0.027
Niger	Bilma	2000	0.457	0.650	0.893
Niger	Bilma	2017	0.305	0.430	0.657
Niger	Bilma	2000-2017	-0.155	-0.077	0.015
Niger	Bkonni	2000	0.777	1.080	1.473
Niger	Bkonni	2017	0.207	0.312	0.469
Niger	Bkonni	2000-2017	-0.171	-0.089	0.013
Niger	Boboye	2000	0.829	1.117	1.484
Niger	Boboye	2017	0.205	0.300	0.436
Niger	Boboye	2000-2017	-0.157	-0.073	0.018
Niger	Bouza	2000	0.789	1.082	1.505
Niger	Bouza	2017	0.222	0.335	0.501
Niger	Bouza	2000-2017	-0.177	-0.088	0.002
Niger	Dakoro	2000	0.998	1.303	1.708
Niger	Dakoro	2017	0.231	0.349	0.503
Niger	Dakoro	2000-2017	-0.208	-0.114	-0.025
Niger	Diffa	2000	0.640	0.904	1.269
Niger	Diffa	2017	0.207	0.331	0.501
Niger	Diffa	2000-2017	-0.198	-0.104	-0.018
Niger	Dogon-Doutchi	2000	0.865	1.191	1.603
Niger	Dogon-Doutchi	2017	0.213	0.301	0.420
Niger	Dogon-Doutchi	2000-2017	-0.159	-0.080	-0.001
Niger	Dosso	2000	0.872	1.161	1.529
Niger	Dosso	2017	0.202	0.294	0.414
Niger	Dosso	2000-2017	-0.152	-0.076	0.013

Table 2: Diarrhea DALYs rate by unit *(continued)*

Country	Unit	year	mean rate	lower rate	upper rate
Niger	Filingué	2000	0.754	1.033	1.390
Niger	Filingué	2017	0.213	0.324	0.464
Niger	Filingué	2000-2017	-0.153	-0.075	0.008
Niger	Gaya	2000	0.863	1.124	1.481
Niger	Gaya	2017	0.203	0.279	0.386
Niger	Gaya	2000-2017	-0.162	-0.085	0.008
Niger	Gouré	2000	0.765	1.041	1.391
Niger	Gouré	2017	0.217	0.349	0.539
Niger	Gouré	2000-2017	-0.161	-0.074	0.008
Niger	Groumdji	2000	1.000	1.326	1.689
Niger	Groumdji	2017	0.209	0.322	0.498
Niger	Groumdji	2000-2017	-0.204	-0.123	-0.022
Niger	Illéla	2000	0.769	1.059	1.446
Niger	Illéla	2017	0.216	0.330	0.497
Niger	Illéla	2000-2017	-0.169	-0.086	0.014
Niger	Keita	2000	0.774	1.074	1.442
Niger	Keita	2017	0.221	0.336	0.494
Niger	Keita	2000-2017	-0.181	-0.089	0.000
Niger	Kollo	2000	0.715	0.944	1.207
Niger	Kollo	2017	0.215	0.320	0.465
Niger	Kollo	2000-2017	-0.154	-0.073	0.010
Niger	Loga	2000	0.850	1.161	1.580
Niger	Loga	2017	0.201	0.301	0.435
Niger	Loga	2000-2017	-0.147	-0.074	0.001
Niger	Madaoua	2000	0.802	1.082	1.465
Niger	Madaoua	2017	0.202	0.313	0.482
Niger	Madaoua	2000-2017	-0.181	-0.094	0.000
Niger	Madarounfa	2000	1.002	1.320	1.692
Niger	Madarounfa	2017	0.204	0.325	0.490
Niger	Madarounfa	2000-2017	-0.203	-0.119	-0.029
Niger	Magaria	2000	0.795	1.079	1.413
Niger	Magaria	2017	0.204	0.326	0.512
Niger	Magaria	2000-2017	-0.163	-0.076	0.006
Niger	Maïné-Soroa	2000	0.674	0.978	1.357
Niger	Maïné-Soroa	2017	0.206	0.330	0.503
Niger	Maïné-Soroa	2000-2017	-0.179	-0.090	-0.003
Niger	Matameye	2000	0.836	1.153	1.513
Niger	Matameye	2017	0.217	0.347	0.547
Niger	Matameye	2000-2017	-0.166	-0.079	0.005
Niger	Mayahi	2000	0.969	1.309	1.673
Niger	Mayahi	2017	0.218	0.343	0.533
Niger	Mayahi	2000-2017	-0.206	-0.113	-0.031
Niger	Mirriah	2000	0.818	1.097	1.454
Niger	Mirriah	2017	0.229	0.354	0.561
Niger	Mirriah	2000-2017	-0.162	-0.073	-0.002
Niger	N'Guigmi	2000	0.651	0.941	1.326
Niger	N'Guigmi	2017	0.220	0.348	0.509
Niger	N'Guigmi	2000-2017	-0.172	-0.089	0.000
Niger	Niamey	2000	0.532	0.721	0.953
Niger	Niamey	2017	0.254	0.377	0.539
Niger	Niamey	2000-2017	-0.156	-0.071	0.015
Niger	Ouallam	2000	0.701	0.984	1.359
Niger	Ouallam	2017	0.227	0.337	0.493
Niger	Ouallam	2000-2017	-0.153	-0.068	0.001
Niger	Say	2000	0.675	0.926	1.226
Niger	Say	2017	0.221	0.324	0.457
Niger	Say	2000-2017	-0.161	-0.078	0.005
Niger	Tahoua	2000	0.768	1.064	1.461
Niger	Tahoua	2017	0.219	0.339	0.481
Niger	Tahoua	2000-2017	-0.181	-0.088	0.008
Niger	Tanout	2000	0.797	1.085	1.461

Table 2: Diarrhea DALYs rate by unit *(continued)*

Country	Unit	year	mean rate	lower rate	upper rate
Niger	Tanout	2017	0.246	0.384	0.580
Niger	Tanout	2000-2017	-0.178	-0.079	0.004
Niger	Tchighozerine	2000	0.513	0.714	1.015
Niger	Tchighozerine	2017	0.328	0.496	0.743
Niger	Tchighozerine	2000-2017	-0.179	-0.069	0.028
Niger	Tchin-Tabarade	2000	0.747	1.033	1.399
Niger	Tchin-Tabarade	2017	0.241	0.372	0.535
Niger	Tchin-Tabarade	2000-2017	-0.176	-0.085	-0.005
Niger	Téra	2000	0.696	0.986	1.318
Niger	Téra	2017	0.236	0.353	0.502
Niger	Téra	2000-2017	-0.149	-0.064	0.022
Niger	Tessaoua	2000	0.942	1.264	1.590
Niger	Tessaoua	2017	0.214	0.342	0.512
Niger	Tessaoua	2000-2017	-0.195	-0.109	-0.034
Niger	Tillabéry	2000	0.667	0.962	1.291
Niger	Tillabéry	2017	0.220	0.340	0.493
Niger	Tillabéry	2000-2017	-0.152	-0.064	0.024
Nigeria	Aba North	2000	0.280	0.445	0.645
Nigeria	Aba North	2017	0.096	0.160	0.244
Nigeria	Aba North	2000-2017	-0.110	-0.016	0.075
Nigeria	Aba South	2000	0.274	0.433	0.630
Nigeria	Aba South	2017	0.096	0.159	0.242
Nigeria	Aba South	2000-2017	-0.110	-0.016	0.074
Nigeria	Abadam	2000	0.576	0.821	1.160
Nigeria	Abadam	2017	0.237	0.389	0.602
Nigeria	Abadam	2000-2017	-0.124	-0.028	0.063
Nigeria	Abaji	2000	0.422	0.663	1.013
Nigeria	Abaji	2017	0.144	0.234	0.372
Nigeria	Abaji	2000-2017	-0.139	-0.048	0.030
Nigeria	Abak	2000	0.264	0.399	0.566
Nigeria	Abak	2017	0.092	0.144	0.213
Nigeria	Abak	2000-2017	-0.100	0.001	0.089
Nigeria	Abakalik	2000	0.304	0.469	0.728
Nigeria	Abakalik	2017	0.098	0.170	0.277
Nigeria	Abakalik	2000-2017	-0.087	0.004	0.090
Nigeria	Abeokuta South	2000	0.303	0.456	0.675
Nigeria	Abeokuta South	2017	0.105	0.167	0.233
Nigeria	Abeokuta South	2000-2017	-0.134	-0.043	0.044
Nigeria	AbeokutaNorth	2000	0.311	0.471	0.696
Nigeria	AbeokutaNorth	2017	0.113	0.172	0.236
Nigeria	AbeokutaNorth	2000-2017	-0.129	-0.043	0.048
Nigeria	Abi	2000	0.278	0.431	0.649
Nigeria	Abi	2017	0.091	0.156	0.250
Nigeria	Abi	2000-2017	-0.092	0.006	0.094
Nigeria	Aboh-Mba	2000	0.286	0.436	0.643
Nigeria	Aboh-Mba	2017	0.096	0.162	0.244
Nigeria	Aboh-Mba	2000-2017	-0.108	-0.014	0.075
Nigeria	Abua/Odu	2000	0.319	0.504	0.771
Nigeria	Abua/Odu	2017	0.122	0.195	0.296
Nigeria	Abua/Odu	2000-2017	-0.112	-0.018	0.077
Nigeria	AbujaMun	2000	0.406	0.621	0.946
Nigeria	AbujaMun	2017	0.130	0.213	0.324
Nigeria	AbujaMun	2000-2017	-0.146	-0.056	0.034
Nigeria	Adavi	2000	0.332	0.527	0.805
Nigeria	Adavi	2017	0.116	0.193	0.302
Nigeria	Adavi	2000-2017	-0.127	-0.037	0.053
Nigeria	Ado	2000	0.334	0.537	0.839
Nigeria	Ado	2017	0.121	0.197	0.305
Nigeria	Ado	2000-2017	-0.080	0.005	0.088
Nigeria	Ado-Ekiti	2000	0.255	0.428	0.668
Nigeria	Ado-Ekiti	2017	0.094	0.151	0.223

Table 2: Diarrhea DALYs rate by unit *(continued)*

Country	Unit	year	mean rate	lower rate	upper rate
Nigeria	Ado-Ekiti	2000-2017	-0.141	-0.052	0.040
Nigeria	AdoOdo/Ota	2000	0.289	0.439	0.635
Nigeria	AdoOdo/Ota	2017	0.116	0.173	0.234
Nigeria	AdoOdo/Ota	2000-2017	-0.132	-0.046	0.046
Nigeria	Afijio	2000	0.272	0.427	0.642
Nigeria	Afijio	2017	0.096	0.149	0.214
Nigeria	Afijio	2000-2017	-0.140	-0.053	0.036
Nigeria	Afikpo	2000	0.328	0.503	0.759
Nigeria	Afikpo	2017	0.107	0.185	0.291
Nigeria	Afikpo	2000-2017	-0.093	0.005	0.089
Nigeria	AfikpoSo	2000	0.322	0.488	0.728
Nigeria	AfikpoSo	2017	0.103	0.175	0.280
Nigeria	AfikpoSo	2000-2017	-0.100	-0.004	0.084
Nigeria	Agaie	2000	0.411	0.629	0.979
Nigeria	Agaie	2017	0.146	0.242	0.365
Nigeria	Agaie	2000-2017	-0.131	-0.038	0.052
Nigeria	Agatu	2000	0.352	0.554	0.857
Nigeria	Agatu	2017	0.131	0.212	0.333
Nigeria	Agatu	2000-2017	-0.096	-0.013	0.074
Nigeria	Agege	2000	0.282	0.431	0.629
Nigeria	Agege	2017	0.118	0.184	0.251
Nigeria	Agege	2000-2017	-0.128	-0.037	0.056
Nigeria	Aguata	2000	0.243	0.381	0.578
Nigeria	Aguata	2017	0.086	0.142	0.227
Nigeria	Aguata	2000-2017	-0.109	-0.010	0.087
Nigeria	Agwara	2000	0.550	0.852	1.284
Nigeria	Agwara	2017	0.206	0.301	0.428
Nigeria	Agwara	2000-2017	-0.130	-0.041	0.050
Nigeria	Ahizu-Mb	2000	0.297	0.458	0.682
Nigeria	Ahizu-Mb	2017	0.100	0.169	0.255
Nigeria	Ahizu-Mb	2000-2017	-0.103	-0.009	0.088
Nigeria	Ahoada East	2000	0.280	0.434	0.671
Nigeria	Ahoada East	2017	0.101	0.162	0.254
Nigeria	Ahoada East	2000-2017	-0.112	-0.019	0.072
Nigeria	Ahoada West	2000	0.312	0.478	0.746
Nigeria	Ahoada West	2017	0.114	0.181	0.284
Nigeria	Ahoada West	2000-2017	-0.114	-0.021	0.074
Nigeria	Ajaokuta	2000	0.336	0.525	0.839
Nigeria	Ajaokuta	2017	0.118	0.198	0.306
Nigeria	Ajaokuta	2000-2017	-0.124	-0.029	0.061
Nigeria	Ajeromi/Ifelodun	2000	0.270	0.411	0.596
Nigeria	Ajeromi/Ifelodun	2017	0.116	0.182	0.250
Nigeria	Ajeromi/Ifelodun	2000-2017	-0.129	-0.038	0.058
Nigeria	Ajingi	2000	0.532	0.803	1.154
Nigeria	Ajingi	2017	0.241	0.374	0.585
Nigeria	Ajingi	2000-2017	-0.091	-0.011	0.083
Nigeria	Akamkpa	2000	0.353	0.518	0.724
Nigeria	Akamkpa	2017	0.117	0.195	0.301
Nigeria	Akamkpa	2000-2017	-0.075	0.014	0.089
Nigeria	Akinyele	2000	0.277	0.440	0.677
Nigeria	Akinyele	2017	0.096	0.154	0.218
Nigeria	Akinyele	2000-2017	-0.147	-0.060	0.023
Nigeria	Akko	2000	0.520	0.833	1.221
Nigeria	Akko	2017	0.219	0.352	0.561
Nigeria	Akko	2000-2017	-0.117	-0.017	0.070
Nigeria	Akoko North-East	2000	0.267	0.432	0.673
Nigeria	Akoko North-East	2017	0.092	0.153	0.226
Nigeria	Akoko North-East	2000-2017	-0.150	-0.057	0.032
Nigeria	Akoko South-East	2000	0.278	0.436	0.675
Nigeria	Akoko South-East	2017	0.093	0.153	0.231
Nigeria	Akoko South-East	2000-2017	-0.153	-0.059	0.030

Table 2: Diarrhea DALYs rate by unit *(continued)*

Country	Unit	year	mean rate	lower rate	upper rate
Nigeria	Akoko South-West	2000	0.265	0.431	0.675
Nigeria	Akoko South-West	2017	0.092	0.150	0.222
Nigeria	Akoko South-West	2000-2017	-0.153	-0.061	0.025
Nigeria	Akoko-Ed	2000	0.267	0.430	0.659
Nigeria	Akoko-Ed	2017	0.095	0.158	0.240
Nigeria	Akoko-Ed	2000-2017	-0.138	-0.045	0.045
Nigeria	AkokoNorthWest	2000	0.265	0.432	0.676
Nigeria	AkokoNorthWest	2017	0.094	0.154	0.231
Nigeria	AkokoNorthWest	2000-2017	-0.142	-0.050	0.036
Nigeria	Akpabuyo	2000	0.316	0.469	0.630
Nigeria	Akpabuyo	2017	0.104	0.173	0.262
Nigeria	Akpabuyo	2000-2017	-0.081	0.012	0.095
Nigeria	Akukutor	2000	0.306	0.459	0.688
Nigeria	Akukutor	2017	0.113	0.183	0.275
Nigeria	Akukutor	2000-2017	-0.106	-0.013	0.081
Nigeria	Akure North	2000	0.270	0.439	0.687
Nigeria	Akure North	2017	0.097	0.152	0.231
Nigeria	Akure North	2000-2017	-0.142	-0.054	0.041
Nigeria	Akure South	2000	0.275	0.443	0.683
Nigeria	Akure South	2017	0.094	0.151	0.226
Nigeria	Akure South	2000-2017	-0.151	-0.062	0.032
Nigeria	Akwanga	2000	0.413	0.691	1.048
Nigeria	Akwanga	2017	0.156	0.251	0.393
Nigeria	Akwanga	2000-2017	-0.117	-0.028	0.057
Nigeria	Albasu	2000	0.524	0.808	1.180
Nigeria	Albasu	2017	0.243	0.389	0.591
Nigeria	Albasu	2000-2017	-0.093	-0.006	0.085
Nigeria	Aleiro	2000	0.659	0.924	1.319
Nigeria	Aleiro	2017	0.258	0.368	0.510
Nigeria	Aleiro	2000-2017	-0.106	-0.026	0.063
Nigeria	Alimosho	2000	0.277	0.420	0.603
Nigeria	Alimosho	2017	0.109	0.169	0.231
Nigeria	Alimosho	2000-2017	-0.131	-0.042	0.053
Nigeria	Alkaleri	2000	0.564	0.906	1.359
Nigeria	Alkaleri	2017	0.215	0.365	0.564
Nigeria	Alkaleri	2000-2017	-0.103	-0.013	0.072
Nigeria	Amuwo Odofin	2000	0.264	0.403	0.586
Nigeria	Amuwo Odofin	2017	0.112	0.175	0.241
Nigeria	Amuwo Odofin	2000-2017	-0.128	-0.038	0.058
Nigeria	Anambra East	2000	0.259	0.405	0.627
Nigeria	Anambra East	2017	0.095	0.156	0.246
Nigeria	Anambra East	2000-2017	-0.116	-0.016	0.077
Nigeria	Anambra West	2000	0.256	0.399	0.625
Nigeria	Anambra West	2017	0.095	0.155	0.240
Nigeria	Anambra West	2000-2017	-0.122	-0.022	0.074
Nigeria	Anaocha	2000	0.243	0.382	0.581
Nigeria	Anaocha	2017	0.086	0.142	0.223
Nigeria	Anaocha	2000-2017	-0.111	-0.011	0.083
Nigeria	Andoni/O	2000	0.293	0.435	0.632
Nigeria	Andoni/O	2017	0.101	0.158	0.238
Nigeria	Andoni/O	2000-2017	-0.102	-0.005	0.098
Nigeria	Aninri	2000	0.318	0.484	0.758
Nigeria	Aninri	2017	0.104	0.179	0.288
Nigeria	Aninri	2000-2017	-0.106	-0.010	0.081
Nigeria	AniochaN	2000	0.250	0.384	0.615
Nigeria	AniochaN	2017	0.090	0.146	0.225
Nigeria	AniochaN	2000-2017	-0.135	-0.029	0.060
Nigeria	AniochaS	2000	0.239	0.373	0.580
Nigeria	AniochaS	2017	0.095	0.149	0.235
Nigeria	AniochaS	2000-2017	-0.133	-0.029	0.054
Nigeria	Anka	2000	0.689	1.010	1.454

Table 2: Diarrhea DALYs rate by unit *(continued)*

Country	Unit	year	mean rate	lower rate	upper rate
Nigeria	Anka	2017	0.259	0.405	0.606
Nigeria	Anka	2000-2017	-0.121	-0.036	0.076
Nigeria	Ankpa	2000	0.338	0.540	0.852
Nigeria	Ankpa	2017	0.128	0.200	0.317
Nigeria	Ankpa	2000-2017	-0.103	-0.016	0.068
Nigeria	Apa	2000	0.362	0.568	0.882
Nigeria	Apa	2017	0.135	0.212	0.329
Nigeria	Apa	2000-2017	-0.091	-0.009	0.075
Nigeria	Apapa	2000	0.269	0.412	0.598
Nigeria	Apapa	2017	0.118	0.185	0.256
Nigeria	Apapa	2000-2017	-0.125	-0.035	0.061
Nigeria	Ardo-Kola	2000	0.496	0.788	1.166
Nigeria	Ardo-Kola	2017	0.189	0.312	0.488
Nigeria	Ardo-Kola	2000-2017	-0.106	0.000	0.084
Nigeria	Arewa	2000	0.715	0.967	1.321
Nigeria	Arewa	2017	0.272	0.379	0.512
Nigeria	Arewa	2000-2017	-0.096	-0.021	0.067
Nigeria	Argungu	2000	0.610	0.872	1.197
Nigeria	Argungu	2017	0.258	0.362	0.498
Nigeria	Argungu	2000-2017	-0.110	-0.026	0.063
Nigeria	Arochukw	2000	0.306	0.453	0.667
Nigeria	Arochukw	2017	0.100	0.168	0.254
Nigeria	Arochukw	2000-2017	-0.090	0.002	0.091
Nigeria	Asa	2000	0.403	0.626	0.973
Nigeria	Asa	2017	0.115	0.191	0.279
Nigeria	Asa	2000-2017	-0.163	-0.068	0.023
Nigeria	Asari-To	2000	0.332	0.504	0.766
Nigeria	Asari-To	2017	0.118	0.190	0.284
Nigeria	Asari-To	2000-2017	-0.114	-0.022	0.071
Nigeria	Askira/U	2000	0.687	0.949	1.290
Nigeria	Askira/U	2017	0.234	0.390	0.634
Nigeria	Askira/U	2000-2017	-0.132	-0.047	0.049
Nigeria	Atakumosa East	2000	0.272	0.455	0.708
Nigeria	Atakumosa East	2017	0.093	0.156	0.233
Nigeria	Atakumosa East	2000-2017	-0.159	-0.069	0.020
Nigeria	Atakumosa West	2000	0.268	0.454	0.696
Nigeria	Atakumosa West	2017	0.095	0.159	0.228
Nigeria	Atakumosa West	2000-2017	-0.153	-0.064	0.025
Nigeria	Atiba	2000	0.310	0.477	0.717
Nigeria	Atiba	2017	0.102	0.162	0.225
Nigeria	Atiba	2000-2017	-0.141	-0.053	0.033
Nigeria	Atisbo	2000	0.344	0.524	0.754
Nigeria	Atisbo	2017	0.109	0.163	0.229
Nigeria	Atisbo	2000-2017	-0.144	-0.051	0.031
Nigeria	Augie	2000	0.615	0.908	1.241
Nigeria	Augie	2017	0.263	0.383	0.535
Nigeria	Augie	2000-2017	-0.111	-0.027	0.067
Nigeria	Auyo	2000	0.595	0.828	1.136
Nigeria	Auyo	2017	0.246	0.399	0.629
Nigeria	Auyo	2000-2017	-0.094	-0.005	0.096
Nigeria	Awe	2000	0.402	0.663	1.000
Nigeria	Awe	2017	0.151	0.246	0.371
Nigeria	Awe	2000-2017	-0.090	-0.004	0.085
Nigeria	Awgu	2000	0.272	0.406	0.626
Nigeria	Awgu	2017	0.086	0.150	0.244
Nigeria	Awgu	2000-2017	-0.102	-0.011	0.078
Nigeria	AwkaNort	2000	0.264	0.400	0.631
Nigeria	AwkaNort	2017	0.094	0.154	0.243
Nigeria	AwkaNort	2000-2017	-0.108	-0.013	0.080
Nigeria	AwkaSout	2000	0.251	0.383	0.599
Nigeria	AwkaSout	2017	0.088	0.144	0.226

Table 2: Diarrhea DALYs rate by unit *(continued)*

Country	Unit	year	mean rate	lower rate	upper rate
Nigeria	AwkaSout	2000-2017	-0.110	-0.010	0.084
Nigeria	Ayamelum	2000	0.259	0.404	0.653
Nigeria	Ayamelum	2017	0.094	0.154	0.241
Nigeria	Ayamelum	2000-2017	-0.112	-0.016	0.075
Nigeria	Ayedaade	2000	0.265	0.434	0.671
Nigeria	Ayedaade	2017	0.099	0.160	0.233
Nigeria	Ayedaade	2000-2017	-0.151	-0.060	0.028
Nigeria	Ayedire	2000	0.252	0.406	0.643
Nigeria	Ayedire	2017	0.089	0.146	0.211
Nigeria	Ayedire	2000-2017	-0.144	-0.057	0.032
Nigeria	Babura	2000	0.626	0.895	1.223
Nigeria	Babura	2017	0.245	0.377	0.586
Nigeria	Babura	2000-2017	-0.102	-0.020	0.068
Nigeria	Badagary	2000	0.252	0.389	0.555
Nigeria	Badagary	2017	0.111	0.160	0.214
Nigeria	Badagary	2000-2017	-0.122	-0.038	0.054
Nigeria	Bade	2000	0.589	0.835	1.172
Nigeria	Bade	2017	0.242	0.388	0.635
Nigeria	Bade	2000-2017	-0.101	-0.009	0.086
Nigeria	Bagudo	2000	0.685	0.963	1.389
Nigeria	Bagudo	2017	0.246	0.341	0.452
Nigeria	Bagudo	2000-2017	-0.113	-0.031	0.064
Nigeria	Bagwai	2000	0.567	0.830	1.200
Nigeria	Bagwai	2017	0.229	0.357	0.535
Nigeria	Bagwai	2000-2017	-0.114	-0.023	0.063
Nigeria	Bakassi	2000	0.380	0.546	0.715
Nigeria	Bakassi	2017	0.115	0.191	0.296
Nigeria	Bakassi	2000-2017	-0.075	0.016	0.099
Nigeria	Bakori	2000	0.560	0.846	1.248
Nigeria	Bakori	2017	0.219	0.353	0.533
Nigeria	Bakori	2000-2017	-0.114	-0.028	0.058
Nigeria	Bakura	2000	0.679	0.950	1.362
Nigeria	Bakura	2017	0.269	0.413	0.607
Nigeria	Bakura	2000-2017	-0.110	-0.028	0.086
Nigeria	Balanga	2000	0.540	0.839	1.195
Nigeria	Balanga	2017	0.204	0.345	0.528
Nigeria	Balanga	2000-2017	-0.107	-0.021	0.069
Nigeria	Bali	2000	0.457	0.707	0.988
Nigeria	Bali	2017	0.168	0.280	0.465
Nigeria	Bali	2000-2017	-0.092	0.010	0.095
Nigeria	Bama	2000	0.772	1.022	1.315
Nigeria	Bama	2017	0.266	0.438	0.667
Nigeria	Bama	2000-2017	-0.133	-0.046	0.050
Nigeria	Barkin Ladi	2000	0.362	0.599	0.881
Nigeria	Barkin Ladi	2017	0.134	0.225	0.348
Nigeria	Barkin Ladi	2000-2017	-0.118	-0.036	0.052
Nigeria	Baruten	2000	0.473	0.726	1.057
Nigeria	Baruten	2017	0.137	0.201	0.281
Nigeria	Baruten	2000-2017	-0.138	-0.059	0.029
Nigeria	Bassa	2000	0.345	0.540	0.854
Nigeria	Bassa	2000	0.374	0.605	0.898
Nigeria	Bassa	2017	0.131	0.210	0.327
Nigeria	Bassa	2017	0.151	0.241	0.366
Nigeria	Bassa	2000-2017	-0.116	-0.032	0.064
Nigeria	Bassa	2000-2017	-0.126	-0.029	0.052
Nigeria	Batagarawa	2000	0.594	0.840	1.140
Nigeria	Batagarawa	2017	0.215	0.338	0.504
Nigeria	Batagarawa	2000-2017	-0.117	-0.028	0.060
Nigeria	Batsari	2000	0.601	0.849	1.190
Nigeria	Batsari	2017	0.212	0.336	0.505
Nigeria	Batsari	2000-2017	-0.125	-0.034	0.053

Table 2: Diarrhea DALYs rate by unit *(continued)*

Country	Unit	year	mean rate	lower rate	upper rate
Nigeria	Bauchi	2000	0.518	0.843	1.294
Nigeria	Bauchi	2017	0.204	0.343	0.524
Nigeria	Bauchi	2000-2017	-0.108	-0.018	0.074
Nigeria	Baure	2000	0.578	0.836	1.125
Nigeria	Baure	2017	0.222	0.347	0.554
Nigeria	Baure	2000-2017	-0.105	-0.021	0.066
Nigeria	Bayo	2000	0.561	0.886	1.227
Nigeria	Bayo	2017	0.225	0.383	0.599
Nigeria	Bayo	2000-2017	-0.117	-0.023	0.074
Nigeria	Bebeji	2000	0.485	0.741	1.089
Nigeria	Bebeji	2017	0.222	0.352	0.521
Nigeria	Bebeji	2000-2017	-0.107	-0.016	0.069
Nigeria	Bekwarra	2000	0.316	0.481	0.702
Nigeria	Bekwarra	2017	0.103	0.167	0.275
Nigeria	Bekwarra	2000-2017	-0.083	0.010	0.091
Nigeria	Bende	2000	0.285	0.424	0.628
Nigeria	Bende	2017	0.096	0.161	0.252
Nigeria	Bende	2000-2017	-0.088	0.003	0.095
Nigeria	Biase	2000	0.334	0.496	0.731
Nigeria	Biase	2017	0.108	0.183	0.281
Nigeria	Biase	2000-2017	-0.092	0.003	0.086
Nigeria	Bichi	2000	0.566	0.830	1.180
Nigeria	Bichi	2017	0.227	0.349	0.533
Nigeria	Bichi	2000-2017	-0.112	-0.020	0.069
Nigeria	Bida	2000	0.399	0.613	0.967
Nigeria	Bida	2017	0.134	0.225	0.336
Nigeria	Bida	2000-2017	-0.137	-0.047	0.047
Nigeria	Billiri	2000	0.549	0.883	1.279
Nigeria	Billiri	2017	0.212	0.348	0.564
Nigeria	Billiri	2000-2017	-0.120	-0.020	0.069
Nigeria	Bindawa	2000	0.581	0.827	1.130
Nigeria	Bindawa	2017	0.214	0.340	0.517
Nigeria	Bindawa	2000-2017	-0.118	-0.025	0.062
Nigeria	Binji	2000	0.568	0.845	1.215
Nigeria	Binji	2017	0.261	0.377	0.532
Nigeria	Binji	2000-2017	-0.112	-0.021	0.080
Nigeria	Biriniwa	2000	0.575	0.819	1.082
Nigeria	Biriniwa	2017	0.245	0.384	0.638
Nigeria	Biriniwa	2000-2017	-0.097	-0.007	0.093
Nigeria	Birnin-G	2000	0.481	0.720	1.055
Nigeria	Birnin-G	2017	0.187	0.290	0.441
Nigeria	Birnin-G	2000-2017	-0.118	-0.030	0.065
Nigeria	Birnin-Magaji/Kiyaw	2000	0.682	0.944	1.353
Nigeria	Birnin-Magaji/Kiyaw	2017	0.257	0.395	0.610
Nigeria	Birnin-Magaji/Kiyaw	2000-2017	-0.136	-0.034	0.050
Nigeria	BirninKe	2000	0.659	0.909	1.295
Nigeria	BirninKe	2017	0.261	0.364	0.502
Nigeria	BirninKe	2000-2017	-0.102	-0.024	0.064
Nigeria	BirninKu	2000	0.528	0.819	1.226
Nigeria	BirninKu	2017	0.242	0.399	0.614
Nigeria	BirninKu	2000-2017	-0.092	-0.004	0.088
Nigeria	Biu	2000	0.651	0.967	1.347
Nigeria	Biu	2017	0.251	0.411	0.666
Nigeria	Biu	2000-2017	-0.113	-0.031	0.067
Nigeria	Bodinga	2000	0.590	0.855	1.227
Nigeria	Bodinga	2017	0.250	0.375	0.530
Nigeria	Bodinga	2000-2017	-0.112	-0.025	0.075
Nigeria	Bogoro	2000	0.459	0.731	1.061
Nigeria	Bogoro	2017	0.167	0.279	0.431
Nigeria	Bogoro	2000-2017	-0.115	-0.021	0.064
Nigeria	Boki	2000	0.353	0.542	0.781

Table 2: Diarrhea DALYs rate by unit *(continued)*

Country	Unit	year	mean rate	lower rate	upper rate
Nigeria	Boki	2017	0.121	0.195	0.324
Nigeria	Boki	2000-2017	-0.080	0.012	0.092
Nigeria	Bokkos	2000	0.375	0.635	0.923
Nigeria	Bokkos	2017	0.133	0.221	0.340
Nigeria	Bokkos	2000-2017	-0.123	-0.038	0.045
Nigeria	Boluwaduro	2000	0.286	0.480	0.737
Nigeria	Boluwaduro	2017	0.101	0.163	0.236
Nigeria	Boluwaduro	2000-2017	-0.149	-0.058	0.023
Nigeria	Bomadi	2000	0.323	0.510	0.803
Nigeria	Bomadi	2017	0.131	0.205	0.308
Nigeria	Bomadi	2000-2017	-0.131	-0.032	0.057
Nigeria	Bonny	2000	0.288	0.429	0.626
Nigeria	Bonny	2017	0.103	0.164	0.249
Nigeria	Bonny	2000-2017	-0.100	-0.005	0.094
Nigeria	Borgu	2000	0.520	0.820	1.213
Nigeria	Borgu	2017	0.189	0.270	0.389
Nigeria	Borgu	2000-2017	-0.127	-0.045	0.045
Nigeria	Boripe	2000	0.284	0.475	0.737
Nigeria	Boripe	2017	0.101	0.163	0.235
Nigeria	Boripe	2000-2017	-0.148	-0.058	0.024
Nigeria	Borsari	2000	0.593	0.865	1.236
Nigeria	Borsari	2017	0.240	0.403	0.659
Nigeria	Borsari	2000-2017	-0.101	-0.015	0.078
Nigeria	Bosso	2000	0.452	0.673	1.028
Nigeria	Bosso	2017	0.155	0.248	0.377
Nigeria	Bosso	2000-2017	-0.126	-0.037	0.052
Nigeria	Brass	2000	0.285	0.440	0.662
Nigeria	Brass	2017	0.116	0.183	0.270
Nigeria	Brass	2000-2017	-0.113	-0.016	0.081
Nigeria	Buji	2000	0.523	0.804	1.188
Nigeria	Buji	2017	0.238	0.400	0.616
Nigeria	Buji	2000-2017	-0.095	-0.005	0.087
Nigeria	Bukkuyum	2000	0.696	1.017	1.452
Nigeria	Bukkuyum	2017	0.259	0.390	0.589
Nigeria	Bukkuyum	2000-2017	-0.117	-0.034	0.069
Nigeria	Bungudu	2000	0.624	0.901	1.282
Nigeria	Bungudu	2017	0.245	0.384	0.598
Nigeria	Bungudu	2000-2017	-0.134	-0.036	0.060
Nigeria	Bunkure	2000	0.516	0.782	1.131
Nigeria	Bunkure	2017	0.248	0.388	0.585
Nigeria	Bunkure	2000-2017	-0.104	-0.013	0.082
Nigeria	Bunza	2000	0.660	0.901	1.281
Nigeria	Bunza	2017	0.261	0.355	0.489
Nigeria	Bunza	2000-2017	-0.113	-0.031	0.059
Nigeria	Buruku	2000	0.334	0.561	0.837
Nigeria	Buruku	2017	0.125	0.198	0.312
Nigeria	Buruku	2000-2017	-0.098	0.007	0.098
Nigeria	Burutu	2000	0.292	0.459	0.707
Nigeria	Burutu	2017	0.125	0.195	0.288
Nigeria	Burutu	2000-2017	-0.131	-0.032	0.060
Nigeria	Bwari	2000	0.396	0.615	0.936
Nigeria	Bwari	2017	0.131	0.212	0.316
Nigeria	Bwari	2000-2017	-0.146	-0.054	0.043
Nigeria	Calabar	2000	0.332	0.489	0.670
Nigeria	Calabar	2017	0.111	0.183	0.281
Nigeria	Calabar	2000-2017	-0.082	0.013	0.097
Nigeria	Calabar South	2000	0.339	0.508	0.703
Nigeria	Calabar South	2017	0.120	0.195	0.300
Nigeria	Calabar South	2000-2017	-0.083	0.013	0.100
Nigeria	Chanchaga	2000	0.438	0.653	0.997
Nigeria	Chanchaga	2017	0.145	0.237	0.361

Table 2: Diarrhea DALYs rate by unit *(continued)*

Country	Unit	year	mean rate	lower rate	upper rate
Nigeria	Chanchaga	2000-2017	-0.125	-0.037	0.054
Nigeria	Charanchi	2000	0.582	0.825	1.123
Nigeria	Charanchi	2017	0.214	0.334	0.508
Nigeria	Charanchi	2000-2017	-0.120	-0.029	0.056
Nigeria	Chibok	2000	0.704	1.002	1.367
Nigeria	Chibok	2017	0.250	0.421	0.692
Nigeria	Chibok	2000-2017	-0.133	-0.045	0.049
Nigeria	Chikun	2000	0.425	0.665	0.984
Nigeria	Chikun	2017	0.159	0.252	0.382
Nigeria	Chikun	2000-2017	-0.125	-0.032	0.062
Nigeria	Dala	2000	0.527	0.767	1.106
Nigeria	Dala	2017	0.217	0.331	0.504
Nigeria	Dala	2000-2017	-0.100	-0.014	0.077
Nigeria	Damaturu	2000	0.666	0.992	1.384
Nigeria	Damaturu	2017	0.254	0.448	0.735
Nigeria	Damaturu	2000-2017	-0.127	-0.027	0.073
Nigeria	Damban	2000	0.541	0.867	1.287
Nigeria	Damban	2017	0.228	0.393	0.623
Nigeria	Damban	2000-2017	-0.125	-0.019	0.079
Nigeria	Dambatta	2000	0.573	0.813	1.144
Nigeria	Dambatta	2017	0.225	0.352	0.555
Nigeria	Dambatta	2000-2017	-0.103	-0.017	0.066
Nigeria	Damboa	2000	0.717	1.009	1.386
Nigeria	Damboa	2017	0.255	0.428	0.692
Nigeria	Damboa	2000-2017	-0.125	-0.042	0.051
Nigeria	Dandi	2000	0.701	0.968	1.326
Nigeria	Dandi	2017	0.262	0.360	0.490
Nigeria	Dandi	2000-2017	-0.101	-0.026	0.065
Nigeria	Dandume	2000	0.480	0.721	1.097
Nigeria	Dandume	2017	0.190	0.305	0.461
Nigeria	Dandume	2000-2017	-0.117	-0.029	0.064
Nigeria	Dange-Shuni	2000	0.604	0.868	1.246
Nigeria	Dange-Shuni	2017	0.257	0.382	0.540
Nigeria	Dange-Shuni	2000-2017	-0.107	-0.025	0.076
Nigeria	Danja	2000	0.488	0.734	1.079
Nigeria	Danja	2017	0.205	0.323	0.484
Nigeria	Danja	2000-2017	-0.118	-0.026	0.057
Nigeria	Danko Wasagu	2000	0.611	0.940	1.369
Nigeria	Danko Wasagu	2017	0.236	0.343	0.515
Nigeria	Danko Wasagu	2000-2017	-0.125	-0.038	0.057
Nigeria	Danmusa	2000	0.567	0.823	1.167
Nigeria	Danmusa	2017	0.215	0.339	0.522
Nigeria	Danmusa	2000-2017	-0.130	-0.033	0.055
Nigeria	Darazo	2000	0.586	0.891	1.315
Nigeria	Darazo	2017	0.238	0.408	0.635
Nigeria	Darazo	2000-2017	-0.115	-0.017	0.083
Nigeria	Dass	2000	0.445	0.717	1.092
Nigeria	Dass	2017	0.169	0.284	0.452
Nigeria	Dass	2000-2017	-0.112	-0.022	0.073
Nigeria	Daura	2000	0.545	0.802	1.079
Nigeria	Daura	2017	0.208	0.327	0.501
Nigeria	Daura	2000-2017	-0.112	-0.023	0.061
Nigeria	DawakinK	2000	0.537	0.793	1.160
Nigeria	DawakinK	2017	0.246	0.377	0.564
Nigeria	DawakinK	2000-2017	-0.100	-0.014	0.083
Nigeria	DawakinT	2000	0.543	0.801	1.162
Nigeria	DawakinT	2017	0.229	0.351	0.532
Nigeria	DawakinT	2000-2017	-0.109	-0.019	0.069
Nigeria	Degema	2000	0.320	0.477	0.721
Nigeria	Degema	2017	0.115	0.186	0.283
Nigeria	Degema	2000-2017	-0.104	-0.015	0.079

Table 2: Diarrhea DALYs rate by unit *(continued)*

Country	Unit	year	mean rate	lower rate	upper rate
Nigeria	Dekina	2000	0.320	0.510	0.805
Nigeria	Dekina	2017	0.123	0.199	0.303
Nigeria	Dekina	2000-2017	-0.120	-0.022	0.060
Nigeria	Demsa	2000	0.620	0.896	1.253
Nigeria	Demsa	2017	0.203	0.344	0.540
Nigeria	Demsa	2000-2017	-0.125	-0.034	0.055
Nigeria	Dikwa	2000	0.712	0.946	1.230
Nigeria	Dikwa	2017	0.250	0.425	0.654
Nigeria	Dikwa	2000-2017	-0.134	-0.042	0.053
Nigeria	Doguwa	2000	0.495	0.768	1.120
Nigeria	Doguwa	2017	0.217	0.338	0.503
Nigeria	Doguwa	2000-2017	-0.100	-0.015	0.070
Nigeria	Doma	2000	0.362	0.579	0.902
Nigeria	Doma	2017	0.135	0.215	0.328
Nigeria	Doma	2000-2017	-0.108	-0.016	0.073
Nigeria	Donga	2000	0.408	0.653	0.947
Nigeria	Donga	2017	0.148	0.249	0.383
Nigeria	Donga	2000-2017	-0.075	0.018	0.099
Nigeria	Dukku	2000	0.583	0.910	1.330
Nigeria	Dukku	2017	0.248	0.411	0.633
Nigeria	Dukku	2000-2017	-0.108	-0.016	0.079
Nigeria	Dunukofia	2000	0.274	0.427	0.674
Nigeria	Dunukofia	2017	0.097	0.160	0.253
Nigeria	Dunukofia	2000-2017	-0.110	-0.012	0.081
Nigeria	Dutse	2000	0.557	0.857	1.269
Nigeria	Dutse	2017	0.253	0.404	0.626
Nigeria	Dutse	2000-2017	-0.092	-0.007	0.087
Nigeria	Dutsi	2000	0.552	0.813	1.094
Nigeria	Dutsi	2017	0.212	0.338	0.508
Nigeria	Dutsi	2000-2017	-0.104	-0.023	0.056
Nigeria	Dutsin-M	2000	0.580	0.831	1.143
Nigeria	Dutsin-M	2017	0.213	0.335	0.508
Nigeria	Dutsin-M	2000-2017	-0.125	-0.030	0.059
Nigeria	Eastern Obolo	2000	0.313	0.464	0.641
Nigeria	Eastern Obolo	2017	0.111	0.173	0.256
Nigeria	Eastern Obolo	2000-2017	-0.099	0.002	0.101
Nigeria	Ebonyi	2000	0.294	0.460	0.717
Nigeria	Ebonyi	2017	0.099	0.166	0.271
Nigeria	Ebonyi	2000-2017	-0.089	0.001	0.088
Nigeria	Edati	2000	0.432	0.668	1.055
Nigeria	Edati	2017	0.145	0.240	0.356
Nigeria	Edati	2000-2017	-0.139	-0.046	0.049
Nigeria	Ede North	2000	0.252	0.422	0.661
Nigeria	Ede North	2017	0.094	0.149	0.217
Nigeria	Ede North	2000-2017	-0.144	-0.053	0.036
Nigeria	Ede South	2000	0.253	0.418	0.655
Nigeria	Ede South	2017	0.092	0.149	0.216
Nigeria	Ede South	2000-2017	-0.142	-0.054	0.038
Nigeria	Edu	2000	0.430	0.647	0.978
Nigeria	Edu	2017	0.130	0.217	0.329
Nigeria	Edu	2000-2017	-0.143	-0.053	0.030
Nigeria	Efon	2000	0.262	0.450	0.699
Nigeria	Efon	2017	0.094	0.155	0.223
Nigeria	Efon	2000-2017	-0.150	-0.057	0.039
Nigeria	EgbadoNorth	2000	0.333	0.497	0.710
Nigeria	EgbadoNorth	2017	0.126	0.183	0.253
Nigeria	EgbadoNorth	2000-2017	-0.131	-0.042	0.058
Nigeria	EgbadoSouth	2000	0.295	0.453	0.659
Nigeria	EgbadoSouth	2017	0.125	0.180	0.241
Nigeria	EgbadoSouth	2000-2017	-0.130	-0.040	0.053
Nigeria	Egbeda	2000	0.282	0.450	0.703

Table 2: Diarrhea DALYs rate by unit *(continued)*

Country	Unit	year	mean rate	lower rate	upper rate
Nigeria	Egbeda	2017	0.099	0.157	0.227
Nigeria	Egbeda	2000-2017	-0.155	-0.065	0.022
Nigeria	Egbedore	2000	0.257	0.428	0.678
Nigeria	Egbedore	2017	0.093	0.149	0.217
Nigeria	Egbedore	2000-2017	-0.147	-0.055	0.033
Nigeria	Egor	2000	0.272	0.425	0.657
Nigeria	Egor	2017	0.100	0.155	0.228
Nigeria	Egor	2000-2017	-0.153	-0.056	0.033
Nigeria	Ehime-Mb	2000	0.308	0.472	0.715
Nigeria	Ehime-Mb	2017	0.104	0.175	0.271
Nigeria	Ehime-Mb	2000-2017	-0.097	-0.006	0.091
Nigeria	Ejigbo	2000	0.262	0.417	0.651
Nigeria	Ejigbo	2017	0.094	0.150	0.214
Nigeria	Ejigbo	2000-2017	-0.139	-0.051	0.043
Nigeria	Ekeremor	2000	0.292	0.472	0.731
Nigeria	Ekeremor	2017	0.126	0.195	0.286
Nigeria	Ekeremor	2000-2017	-0.131	-0.032	0.058
Nigeria	Eket	2000	0.312	0.470	0.656
Nigeria	Eket	2017	0.114	0.177	0.258
Nigeria	Eket	2000-2017	-0.099	0.002	0.099
Nigeria	Ekiti	2000	0.327	0.522	0.806
Nigeria	Ekiti	2017	0.105	0.172	0.246
Nigeria	Ekiti	2000-2017	-0.144	-0.058	0.035
Nigeria	EkitiEas	2000	0.271	0.437	0.676
Nigeria	EkitiEas	2017	0.100	0.159	0.236
Nigeria	EkitiEas	2000-2017	-0.138	-0.046	0.043
Nigeria	EkitiSouth-West	2000	0.257	0.442	0.698
Nigeria	EkitiSouth-West	2017	0.096	0.155	0.230
Nigeria	EkitiSouth-West	2000-2017	-0.150	-0.057	0.041
Nigeria	EkitiWest	2000	0.266	0.461	0.717
Nigeria	EkitiWest	2017	0.100	0.162	0.233
Nigeria	EkitiWest	2000-2017	-0.148	-0.055	0.044
Nigeria	Ekwusigo	2000	0.237	0.380	0.575
Nigeria	Ekwusigo	2017	0.088	0.142	0.228
Nigeria	Ekwusigo	2000-2017	-0.112	-0.014	0.075
Nigeria	Eleme	2000	0.277	0.417	0.623
Nigeria	Eleme	2017	0.100	0.159	0.244
Nigeria	Eleme	2000-2017	-0.108	-0.018	0.078
Nigeria	Emuoha	2000	0.280	0.438	0.668
Nigeria	Emuoha	2017	0.105	0.166	0.256
Nigeria	Emuoha	2000-2017	-0.112	-0.019	0.069
Nigeria	Emure/Ise/Orun	2000	0.261	0.436	0.696
Nigeria	Emure/Ise/Orun	2017	0.099	0.158	0.232
Nigeria	Emure/Ise/Orun	2000-2017	-0.142	-0.049	0.041
Nigeria	Enugu East	2000	0.272	0.423	0.674
Nigeria	Enugu East	2017	0.091	0.154	0.246
Nigeria	Enugu East	2000-2017	-0.102	-0.014	0.075
Nigeria	Enugu North	2000	0.282	0.438	0.704
Nigeria	Enugu North	2017	0.091	0.157	0.250
Nigeria	Enugu North	2000-2017	-0.100	-0.014	0.075
Nigeria	EnuguSou	2000	0.264	0.407	0.646
Nigeria	EnuguSou	2017	0.086	0.149	0.237
Nigeria	EnuguSou	2000-2017	-0.101	-0.015	0.078
Nigeria	Epe	2000	0.270	0.425	0.627
Nigeria	Epe	2017	0.098	0.164	0.235
Nigeria	Epe	2000-2017	-0.132	-0.049	0.042
Nigeria	EsanCent	2000	0.256	0.386	0.634
Nigeria	EsanCent	2017	0.089	0.142	0.214
Nigeria	EsanCent	2000-2017	-0.147	-0.044	0.055
Nigeria	EsanNort	2000	0.254	0.388	0.631
Nigeria	EsanNort	2017	0.087	0.141	0.213

Table 2: Diarrhea DALYs rate by unit *(continued)*

Country	Unit	year	mean rate	lower rate	upper rate
Nigeria	EsanNort	2000-2017	-0.142	-0.042	0.059
Nigeria	EsanSout	2000	0.262	0.398	0.640
Nigeria	EsanSout	2017	0.092	0.151	0.229
Nigeria	EsanSout	2000-2017	-0.134	-0.034	0.063
Nigeria	EsanWest	2000	0.261	0.399	0.631
Nigeria	EsanWest	2017	0.094	0.148	0.224
Nigeria	EsanWest	2000-2017	-0.149	-0.046	0.050
Nigeria	Ese-Odo	2000	0.278	0.444	0.666
Nigeria	Ese-Odo	2017	0.108	0.167	0.247
Nigeria	Ese-Odo	2000-2017	-0.131	-0.048	0.049
Nigeria	Esit Eket	2000	0.314	0.474	0.655
Nigeria	Esit Eket	2017	0.112	0.174	0.262
Nigeria	Esit Eket	2000-2017	-0.093	0.005	0.100
Nigeria	Essien-U	2000	0.268	0.408	0.575
Nigeria	Essien-U	2017	0.091	0.147	0.217
Nigeria	Essien-U	2000-2017	-0.091	-0.003	0.083
Nigeria	Etche	2000	0.263	0.411	0.609
Nigeria	Etche	2017	0.098	0.155	0.241
Nigeria	Etche	2000-2017	-0.119	-0.023	0.060
Nigeria	Ethiope West	2000	0.267	0.414	0.631
Nigeria	Ethiope West	2017	0.105	0.160	0.233
Nigeria	Ethiope West	2000-2017	-0.128	-0.040	0.054
Nigeria	EthiopeE	2000	0.249	0.388	0.587
Nigeria	EthiopeE	2017	0.095	0.152	0.228
Nigeria	EthiopeE	2000-2017	-0.123	-0.033	0.057
Nigeria	Eti-Osa	2000	0.253	0.387	0.558
Nigeria	Eti-Osa	2017	0.100	0.160	0.225
Nigeria	Eti-Osa	2000-2017	-0.136	-0.043	0.055
Nigeria	EtimEkpo	2000	0.279	0.423	0.590
Nigeria	EtimEkpo	2017	0.094	0.151	0.221
Nigeria	EtimEkpo	2000-2017	-0.108	-0.005	0.080
Nigeria	Etinan	2000	0.278	0.418	0.589
Nigeria	Etinan	2017	0.097	0.150	0.219
Nigeria	Etinan	2000-2017	-0.106	-0.001	0.090
Nigeria	Etsako Central	2000	0.266	0.412	0.657
Nigeria	Etsako Central	2017	0.092	0.153	0.230
Nigeria	Etsako Central	2000-2017	-0.127	-0.031	0.063
Nigeria	EtsakoEa	2000	0.270	0.419	0.658
Nigeria	EtsakoEa	2017	0.093	0.157	0.239
Nigeria	EtsakoEa	2000-2017	-0.129	-0.031	0.061
Nigeria	EtsakoWe	2000	0.266	0.404	0.647
Nigeria	EtsakoWe	2017	0.086	0.145	0.219
Nigeria	EtsakoWe	2000-2017	-0.143	-0.046	0.042
Nigeria	Etung	2000	0.362	0.546	0.811
Nigeria	Etung	2017	0.121	0.200	0.318
Nigeria	Etung	2000-2017	-0.074	0.010	0.090
Nigeria	Ewekoro	2000	0.285	0.436	0.643
Nigeria	Ewekoro	2017	0.111	0.169	0.236
Nigeria	Ewekoro	2000-2017	-0.132	-0.040	0.049
Nigeria	Ezeagu	2000	0.258	0.384	0.602
Nigeria	Ezeagu	2017	0.090	0.148	0.231
Nigeria	Ezeagu	2000-2017	-0.111	-0.017	0.075
Nigeria	Ezinihit	2000	0.292	0.441	0.644
Nigeria	Ezinihit	2017	0.095	0.162	0.242
Nigeria	Ezinihit	2000-2017	-0.104	-0.010	0.085
Nigeria	Ezza North	2000	0.285	0.450	0.700
Nigeria	Ezza North	2017	0.096	0.163	0.263
Nigeria	Ezza North	2000-2017	-0.103	-0.003	0.082
Nigeria	Ezza South	2000	0.302	0.474	0.732
Nigeria	Ezza South	2017	0.100	0.172	0.282
Nigeria	Ezza South	2000-2017	-0.095	0.003	0.089

Table 2: Diarrhea DALYs rate by unit *(continued)*

Country	Unit	year	mean rate	lower rate	upper rate
Nigeria	Fagge	2000	0.588	0.840	1.202
Nigeria	Fagge	2017	0.248	0.381	0.577
Nigeria	Fagge	2000-2017	-0.101	-0.013	0.080
Nigeria	Fakai	2000	0.636	0.978	1.438
Nigeria	Fakai	2017	0.241	0.359	0.537
Nigeria	Fakai	2000-2017	-0.123	-0.034	0.058
Nigeria	Faskari	2000	0.550	0.814	1.225
Nigeria	Faskari	2017	0.216	0.347	0.525
Nigeria	Faskari	2000-2017	-0.125	-0.031	0.055
Nigeria	Fika	2000	0.626	0.950	1.325
Nigeria	Fika	2017	0.262	0.449	0.710
Nigeria	Fika	2000-2017	-0.124	-0.017	0.084
Nigeria	Fufore	2000	0.629	0.891	1.220
Nigeria	Fufore	2017	0.205	0.348	0.543
Nigeria	Fufore	2000-2017	-0.120	-0.032	0.066
Nigeria	Funakaye	2000	0.551	0.889	1.205
Nigeria	Funakaye	2017	0.247	0.414	0.670
Nigeria	Funakaye	2000-2017	-0.114	-0.020	0.083
Nigeria	Fune	2000	0.646	0.967	1.334
Nigeria	Fune	2017	0.270	0.446	0.737
Nigeria	Fune	2000-2017	-0.126	-0.023	0.073
Nigeria	Funtua	2000	0.514	0.786	1.199
Nigeria	Funtua	2017	0.204	0.327	0.500
Nigeria	Funtua	2000-2017	-0.118	-0.028	0.059
Nigeria	Gabasawa	2000	0.552	0.815	1.158
Nigeria	Gabasawa	2017	0.240	0.368	0.571
Nigeria	Gabasawa	2000-2017	-0.096	-0.014	0.078
Nigeria	Gada	2000	0.622	0.920	1.273
Nigeria	Gada	2017	0.259	0.388	0.574
Nigeria	Gada	2000-2017	-0.108	-0.022	0.080
Nigeria	Gagarawa	2000	0.586	0.834	1.144
Nigeria	Gagarawa	2017	0.251	0.390	0.611
Nigeria	Gagarawa	2000-2017	-0.097	-0.010	0.086
Nigeria	Gamawa	2000	0.527	0.793	1.094
Nigeria	Gamawa	2017	0.235	0.390	0.615
Nigeria	Gamawa	2000-2017	-0.101	-0.009	0.089
Nigeria	Gamjuwa	2000	0.517	0.839	1.262
Nigeria	Gamjuwa	2017	0.218	0.371	0.567
Nigeria	Gamjuwa	2000-2017	-0.106	-0.018	0.076
Nigeria	Ganye	2000	0.483	0.735	1.037
Nigeria	Ganye	2017	0.157	0.274	0.424
Nigeria	Ganye	2000-2017	-0.117	-0.015	0.078
Nigeria	Garki	2000	0.590	0.853	1.187
Nigeria	Garki	2017	0.251	0.393	0.597
Nigeria	Garki	2000-2017	-0.101	-0.013	0.079
Nigeria	Garko	2000	0.512	0.779	1.151
Nigeria	Garko	2017	0.239	0.378	0.568
Nigeria	Garko	2000-2017	-0.099	-0.013	0.081
Nigeria	Garum Mallam	2000	0.502	0.762	1.102
Nigeria	Garum Mallam	2017	0.234	0.373	0.559
Nigeria	Garum Mallam	2000-2017	-0.106	-0.014	0.075
Nigeria	Gashaka	2000	0.410	0.639	0.893
Nigeria	Gashaka	2017	0.163	0.265	0.428
Nigeria	Gashaka	2000-2017	-0.077	0.012	0.105
Nigeria	Gassol	2000	0.432	0.694	1.001
Nigeria	Gassol	2017	0.169	0.276	0.444
Nigeria	Gassol	2000-2017	-0.087	0.008	0.096
Nigeria	Gaya	2000	0.531	0.806	1.169
Nigeria	Gaya	2017	0.247	0.386	0.589
Nigeria	Gaya	2000-2017	-0.094	-0.013	0.079
Nigeria	Gbako	2000	0.411	0.636	0.982

Table 2: Diarrhea DALYs rate by unit *(continued)*

Country	Unit	year	mean rate	lower rate	upper rate
Nigeria	Gbako	2017	0.142	0.235	0.346
Nigeria	Gbako	2000-2017	-0.131	-0.043	0.046
Nigeria	Gboko	2000	0.327	0.543	0.815
Nigeria	Gboko	2017	0.121	0.189	0.289
Nigeria	Gboko	2000-2017	-0.098	0.005	0.094
Nigeria	Gboyin	2000	0.269	0.446	0.695
Nigeria	Gboyin	2017	0.101	0.162	0.239
Nigeria	Gboyin	2000-2017	-0.143	-0.050	0.039
Nigeria	Geidam	2000	0.601	0.858	1.199
Nigeria	Geidam	2017	0.243	0.406	0.664
Nigeria	Geidam	2000-2017	-0.112	-0.022	0.068
Nigeria	Gezawa	2000	0.591	0.845	1.195
Nigeria	Gezawa	2017	0.246	0.377	0.572
Nigeria	Gezawa	2000-2017	-0.096	-0.009	0.084
Nigeria	Giade	2000	0.513	0.808	1.218
Nigeria	Giade	2017	0.231	0.401	0.620
Nigeria	Giade	2000-2017	-0.094	-0.006	0.092
Nigeria	Girie	2000	0.677	0.935	1.250
Nigeria	Girie	2017	0.219	0.368	0.599
Nigeria	Girie	2000-2017	-0.138	-0.043	0.051
Nigeria	Giwa	2000	0.438	0.665	0.971
Nigeria	Giwa	2017	0.181	0.282	0.431
Nigeria	Giwa	2000-2017	-0.115	-0.025	0.063
Nigeria	Gokana	2000	0.296	0.433	0.634
Nigeria	Gokana	2017	0.104	0.164	0.250
Nigeria	Gokana	2000-2017	-0.103	-0.006	0.097
Nigeria	Gombe	2000	0.512	0.824	1.189
Nigeria	Gombe	2017	0.218	0.353	0.569
Nigeria	Gombe	2000-2017	-0.120	-0.018	0.078
Nigeria	Gombi	2000	0.636	0.900	1.237
Nigeria	Gombi	2017	0.213	0.355	0.566
Nigeria	Gombi	2000-2017	-0.133	-0.037	0.060
Nigeria	Goronyo	2000	0.637	0.901	1.264
Nigeria	Goronyo	2017	0.266	0.403	0.600
Nigeria	Goronyo	2000-2017	-0.109	-0.021	0.078
Nigeria	Gubio	2000	0.610	0.891	1.280
Nigeria	Gubio	2017	0.238	0.396	0.633
Nigeria	Gubio	2000-2017	-0.133	-0.030	0.058
Nigeria	Gudu	2000	0.631	0.925	1.265
Nigeria	Gudu	2017	0.270	0.393	0.550
Nigeria	Gudu	2000-2017	-0.098	-0.018	0.083
Nigeria	Gujba	2000	0.658	0.994	1.368
Nigeria	Gujba	2017	0.265	0.453	0.748
Nigeria	Gujba	2000-2017	-0.119	-0.027	0.076
Nigeria	Gulani	2000	0.590	0.916	1.241
Nigeria	Gulani	2017	0.249	0.424	0.684
Nigeria	Gulani	2000-2017	-0.118	-0.022	0.078
Nigeria	Guma	2000	0.373	0.613	0.943
Nigeria	Guma	2017	0.143	0.228	0.341
Nigeria	Guma	2000-2017	-0.099	-0.005	0.083
Nigeria	Gumel	2000	0.588	0.845	1.145
Nigeria	Gumel	2017	0.234	0.376	0.604
Nigeria	Gumel	2000-2017	-0.108	-0.012	0.090
Nigeria	Gummi	2000	0.687	1.015	1.444
Nigeria	Gummi	2017	0.261	0.385	0.559
Nigeria	Gummi	2000-2017	-0.115	-0.033	0.067
Nigeria	Gurara	2000	0.422	0.655	0.967
Nigeria	Gurara	2017	0.145	0.234	0.361
Nigeria	Gurara	2000-2017	-0.126	-0.038	0.046
Nigeria	Guri	2000	0.595	0.836	1.125
Nigeria	Guri	2017	0.255	0.403	0.644

Table 2: Diarrhea DALYs rate by unit *(continued)*

Country	Unit	year	mean rate	lower rate	upper rate
Nigeria	Guri	2000-2017	-0.098	-0.007	0.090
Nigeria	Gusau	2000	0.630	0.918	1.311
Nigeria	Gusau	2017	0.237	0.380	0.583
Nigeria	Gusau	2000-2017	-0.124	-0.034	0.056
Nigeria	Guyuk	2000	0.548	0.854	1.188
Nigeria	Guyuk	2017	0.202	0.335	0.520
Nigeria	Guyuk	2000-2017	-0.118	-0.032	0.058
Nigeria	Guzamala	2000	0.573	0.843	1.215
Nigeria	Guzamala	2017	0.241	0.396	0.629
Nigeria	Guzamala	2000-2017	-0.126	-0.027	0.064
Nigeria	Gwadabaw	2000	0.591	0.861	1.204
Nigeria	Gwadabaw	2017	0.261	0.391	0.575
Nigeria	Gwadabaw	2000-2017	-0.110	-0.022	0.076
Nigeria	Gwagwala	2000	0.412	0.649	0.991
Nigeria	Gwagwala	2017	0.135	0.223	0.347
Nigeria	Gwagwala	2000-2017	-0.148	-0.057	0.026
Nigeria	Gwale	2000	0.525	0.765	1.103
Nigeria	Gwale	2017	0.215	0.330	0.503
Nigeria	Gwale	2000-2017	-0.099	-0.013	0.079
Nigeria	Gwandu	2000	0.640	0.908	1.288
Nigeria	Gwandu	2017	0.256	0.373	0.529
Nigeria	Gwandu	2000-2017	-0.115	-0.029	0.072
Nigeria	Gwaram	2000	0.515	0.814	1.240
Nigeria	Gwaram	2017	0.230	0.396	0.611
Nigeria	Gwaram	2000-2017	-0.097	-0.010	0.086
Nigeria	Gwarzo	2000	0.557	0.799	1.167
Nigeria	Gwarzo	2017	0.220	0.345	0.516
Nigeria	Gwarzo	2000-2017	-0.116	-0.023	0.063
Nigeria	Gwer East	2000	0.327	0.547	0.880
Nigeria	Gwer East	2017	0.123	0.195	0.307
Nigeria	Gwer East	2000-2017	-0.087	0.001	0.084
Nigeria	GwerWest	2000	0.331	0.524	0.824
Nigeria	GwerWest	2017	0.123	0.194	0.308
Nigeria	GwerWest	2000-2017	-0.096	-0.007	0.079
Nigeria	Gwiwa	2000	0.597	0.876	1.190
Nigeria	Gwiwa	2017	0.230	0.359	0.548
Nigeria	Gwiwa	2000-2017	-0.108	-0.023	0.063
Nigeria	Gwoza	2000	0.742	1.034	1.377
Nigeria	Gwoza	2017	0.241	0.426	0.674
Nigeria	Gwoza	2000-2017	-0.127	-0.050	0.043
Nigeria	Hadejia	2000	0.583	0.819	1.108
Nigeria	Hadejia	2017	0.246	0.383	0.609
Nigeria	Hadejia	2000-2017	-0.098	-0.007	0.092
Nigeria	Hawul	2000	0.631	0.929	1.287
Nigeria	Hawul	2017	0.230	0.379	0.602
Nigeria	Hawul	2000-2017	-0.123	-0.032	0.064
Nigeria	Hong	2000	0.686	0.943	1.257
Nigeria	Hong	2017	0.216	0.372	0.614
Nigeria	Hong	2000-2017	-0.144	-0.050	0.052
Nigeria	IbadanNorth	2000	0.307	0.485	0.750
Nigeria	IbadanNorth	2017	0.104	0.166	0.236
Nigeria	IbadanNorth	2000-2017	-0.152	-0.062	0.021
Nigeria	IbadanNorth-East	2000	0.293	0.462	0.717
Nigeria	IbadanNorth-East	2017	0.101	0.161	0.230
Nigeria	IbadanNorth-East	2000-2017	-0.156	-0.064	0.021
Nigeria	IbadanNorth-West	2000	0.312	0.493	0.758
Nigeria	IbadanNorth-West	2017	0.107	0.169	0.239
Nigeria	IbadanNorth-West	2000-2017	-0.152	-0.060	0.023
Nigeria	IbadanSouth-East	2000	0.287	0.453	0.697
Nigeria	IbadanSouth-East	2017	0.099	0.158	0.225
Nigeria	IbadanSouth-East	2000-2017	-0.153	-0.061	0.023

Table 2: Diarrhea DALYs rate by unit *(continued)*

Country	Unit	year	mean rate	lower rate	upper rate
Nigeria	IbadanSouth-West	2000	0.290	0.458	0.702
Nigeria	IbadanSouth-West	2017	0.100	0.160	0.226
Nigeria	IbadanSouth-West	2000-2017	-0.152	-0.060	0.023
Nigeria	Ibaji	2000	0.307	0.479	0.785
Nigeria	Ibaji	2017	0.111	0.183	0.277
Nigeria	Ibaji	2000-2017	-0.117	-0.021	0.081
Nigeria	Ibarapa Central	2000	0.297	0.446	0.645
Nigeria	Ibarapa Central	2017	0.104	0.158	0.219
Nigeria	Ibarapa Central	2000-2017	-0.140	-0.045	0.042
Nigeria	Ibarapa East	2000	0.329	0.499	0.703
Nigeria	Ibarapa East	2017	0.113	0.173	0.242
Nigeria	Ibarapa East	2000-2017	-0.143	-0.050	0.040
Nigeria	Ibarapa North	2000	0.338	0.506	0.715
Nigeria	Ibarapa North	2017	0.111	0.166	0.231
Nigeria	Ibarapa North	2000-2017	-0.143	-0.047	0.043
Nigeria	Ibeju/Lekki	2000	0.275	0.427	0.631
Nigeria	Ibeju/Lekki	2017	0.098	0.165	0.236
Nigeria	Ibeju/Lekki	2000-2017	-0.133	-0.047	0.048
Nigeria	Ibeno	2000	0.319	0.486	0.659
Nigeria	Ibeno	2017	0.115	0.178	0.265
Nigeria	Ibeno	2000-2017	-0.093	0.004	0.098
Nigeria	Ibesikpo Asutan	2000	0.277	0.411	0.576
Nigeria	Ibesikpo Asutan	2017	0.095	0.148	0.220
Nigeria	Ibesikpo Asutan	2000-2017	-0.096	0.004	0.091
Nigeria	Ibi	2000	0.406	0.672	0.987
Nigeria	Ibi	2017	0.155	0.255	0.400
Nigeria	Ibi	2000-2017	-0.091	0.005	0.094
Nigeria	Ibiono Ibom	2000	0.310	0.463	0.675
Nigeria	Ibiono Ibom	2017	0.102	0.166	0.249
Nigeria	Ibiono Ibom	2000-2017	-0.089	0.004	0.091
Nigeria	Idah	2000	0.331	0.512	0.832
Nigeria	Idah	2017	0.114	0.189	0.285
Nigeria	Idah	2000-2017	-0.127	-0.027	0.072
Nigeria	Idanre	2000	0.292	0.470	0.714
Nigeria	Idanre	2017	0.103	0.168	0.250
Nigeria	Idanre	2000-2017	-0.152	-0.063	0.029
Nigeria	Ideato South	2000	0.271	0.407	0.627
Nigeria	Ideato South	2017	0.091	0.153	0.239
Nigeria	Ideato South	2000-2017	-0.100	-0.009	0.084
Nigeria	IdeatoNo	2000	0.265	0.402	0.621
Nigeria	IdeatoNo	2017	0.090	0.153	0.242
Nigeria	IdeatoNo	2000-2017	-0.100	-0.009	0.084
Nigeria	Idemili North	2000	0.255	0.401	0.618
Nigeria	Idemili North	2017	0.092	0.150	0.235
Nigeria	Idemili North	2000-2017	-0.114	-0.014	0.078
Nigeria	Idemili South	2000	0.249	0.395	0.599
Nigeria	Idemili South	2017	0.090	0.148	0.234
Nigeria	Idemili South	2000-2017	-0.113	-0.014	0.078
Nigeria	Ido	2000	0.273	0.428	0.655
Nigeria	Ido	2017	0.097	0.153	0.214
Nigeria	Ido	2000-2017	-0.149	-0.058	0.026
Nigeria	Ido/Osi	2000	0.279	0.466	0.718
Nigeria	Ido/Osi	2017	0.101	0.162	0.237
Nigeria	Ido/Osi	2000-2017	-0.143	-0.054	0.037
Nigeria	Ifako/Ijaye	2000	0.301	0.456	0.661
Nigeria	Ifako/Ijaye	2017	0.121	0.187	0.254
Nigeria	Ifako/Ijaye	2000-2017	-0.135	-0.043	0.051
Nigeria	Ife East	2000	0.284	0.486	0.751
Nigeria	Ife East	2017	0.098	0.167	0.244
Nigeria	Ife East	2000-2017	-0.156	-0.067	0.021
Nigeria	Ife North	2000	0.259	0.441	0.680

Table 2: Diarrhea DALYs rate by unit *(continued)*

Country	Unit	year	mean rate	lower rate	upper rate
Nigeria	Ife North	2017	0.095	0.158	0.229
Nigeria	Ife North	2000-2017	-0.153	-0.062	0.025
Nigeria	Ife South	2000	0.290	0.491	0.757
Nigeria	Ife South	2017	0.100	0.171	0.249
Nigeria	Ife South	2000-2017	-0.157	-0.068	0.019
Nigeria	IfeCentral	2000	0.265	0.454	0.694
Nigeria	IfeCentral	2017	0.092	0.157	0.227
Nigeria	IfeCentral	2000-2017	-0.161	-0.066	0.022
Nigeria	Ifedayo	2000	0.305	0.508	0.770
Nigeria	Ifedayo	2017	0.110	0.176	0.252
Nigeria	Ifedayo	2000-2017	-0.143	-0.055	0.034
Nigeria	Ifedore	2000	0.275	0.460	0.721
Nigeria	Ifedore	2017	0.096	0.158	0.237
Nigeria	Ifedore	2000-2017	-0.155	-0.064	0.032
Nigeria	Ifelodun	2000	0.288	0.480	0.749
Nigeria	Ifelodun	2000	0.403	0.622	0.928
Nigeria	Ifelodun	2017	0.100	0.162	0.233
Nigeria	Ifelodun	2017	0.114	0.192	0.279
Nigeria	Ifelodun	2000-2017	-0.151	-0.063	0.025
Nigeria	Ifelodun	2000-2017	-0.148	-0.056	0.028
Nigeria	Ifo	2000	0.313	0.472	0.692
Nigeria	Ifo	2017	0.121	0.183	0.253
Nigeria	Ifo	2000-2017	-0.143	-0.048	0.044
Nigeria	Igabi	2000	0.461	0.705	1.021
Nigeria	Igabi	2017	0.176	0.276	0.417
Nigeria	Igabi	2000-2017	-0.121	-0.028	0.059
Nigeria	Igalamela-Odolu	2000	0.327	0.508	0.833
Nigeria	Igalamela-Odolu	2017	0.116	0.194	0.294
Nigeria	Igalamela-Odolu	2000-2017	-0.115	-0.022	0.079
Nigeria	Igbo-Eti	2000	0.260	0.406	0.635
Nigeria	Igbo-Eti	2017	0.089	0.149	0.236
Nigeria	Igbo-Eti	2000-2017	-0.100	-0.010	0.083
Nigeria	Igbo-eze North	2000	0.253	0.401	0.646
Nigeria	Igbo-eze North	2017	0.086	0.143	0.222
Nigeria	Igbo-eze North	2000-2017	-0.105	-0.013	0.081
Nigeria	Igbo-eze South	2000	0.252	0.396	0.630
Nigeria	Igbo-eze South	2017	0.086	0.143	0.219
Nigeria	Igbo-eze South	2000-2017	-0.103	-0.013	0.081
Nigeria	Igueben	2000	0.257	0.392	0.630
Nigeria	Igueben	2017	0.090	0.147	0.222
Nigeria	Igueben	2000-2017	-0.150	-0.044	0.053
Nigeria	Ihiala	2000	0.236	0.376	0.571
Nigeria	Ihiala	2017	0.089	0.142	0.227
Nigeria	Ihiala	2000-2017	-0.109	-0.013	0.080
Nigeria	Ihitte/U	2000	0.311	0.478	0.712
Nigeria	Ihitte/U	2017	0.104	0.176	0.269
Nigeria	Ihitte/U	2000-2017	-0.096	-0.003	0.096
Nigeria	Ijebu North-East	2000	0.283	0.435	0.640
Nigeria	Ijebu North-East	2017	0.098	0.159	0.230
Nigeria	Ijebu North-East	2000-2017	-0.150	-0.059	0.024
Nigeria	IjebuEast	2000	0.289	0.448	0.661
Nigeria	IjebuEast	2017	0.104	0.171	0.252
Nigeria	IjebuEast	2000-2017	-0.140	-0.052	0.033
Nigeria	IjebuNorth	2000	0.286	0.444	0.656
Nigeria	IjebuNorth	2017	0.099	0.161	0.232
Nigeria	IjebuNorth	2000-2017	-0.153	-0.059	0.022
Nigeria	IjebuOde	2000	0.280	0.431	0.637
Nigeria	IjebuOde	2017	0.097	0.160	0.231
Nigeria	IjebuOde	2000-2017	-0.142	-0.052	0.033
Nigeria	Ijero	2000	0.292	0.486	0.760
Nigeria	Ijero	2017	0.106	0.170	0.246

Table 2: Diarrhea DALYs rate by unit *(continued)*

Country	Unit	year	mean rate	lower rate	upper rate
Nigeria	Ijero	2000-2017	-0.144	-0.054	0.040
Nigeria	Ijumu	2000	0.332	0.517	0.818
Nigeria	Ijumu	2017	0.110	0.178	0.279
Nigeria	Ijumu	2000-2017	-0.140	-0.048	0.040
Nigeria	Ika	2000	0.282	0.428	0.592
Nigeria	Ika	2017	0.094	0.153	0.226
Nigeria	Ika	2000-2017	-0.108	-0.009	0.077
Nigeria	IkaNorth	2000	0.241	0.373	0.587
Nigeria	IkaNorth	2017	0.090	0.142	0.218
Nigeria	IkaNorth	2000-2017	-0.145	-0.039	0.046
Nigeria	Ikara	2000	0.485	0.753	1.085
Nigeria	Ikara	2017	0.230	0.353	0.526
Nigeria	Ikara	2000-2017	-0.106	-0.014	0.072
Nigeria	IkaSouth	2000	0.247	0.374	0.589
Nigeria	IkaSouth	2017	0.090	0.142	0.216
Nigeria	IkaSouth	2000-2017	-0.148	-0.043	0.045
Nigeria	Ikeduru	2000	0.282	0.437	0.655
Nigeria	Ikeduru	2017	0.097	0.164	0.249
Nigeria	Ikeduru	2000-2017	-0.103	-0.009	0.082
Nigeria	Ikeja	2000	0.279	0.425	0.614
Nigeria	Ikeja	2017	0.113	0.176	0.241
Nigeria	Ikeja	2000-2017	-0.132	-0.040	0.055
Nigeria	Ikenne	2000	0.293	0.454	0.674
Nigeria	Ikenne	2017	0.101	0.167	0.234
Nigeria	Ikenne	2000-2017	-0.142	-0.050	0.036
Nigeria	Ikere	2000	0.241	0.413	0.651
Nigeria	Ikere	2017	0.090	0.145	0.212
Nigeria	Ikere	2000-2017	-0.141	-0.052	0.039
Nigeria	Ikole	2000	0.271	0.436	0.671
Nigeria	Ikole	2017	0.097	0.157	0.230
Nigeria	Ikole	2000-2017	-0.142	-0.049	0.040
Nigeria	Ikom	2000	0.319	0.496	0.740
Nigeria	Ikom	2017	0.109	0.181	0.294
Nigeria	Ikom	2000-2017	-0.077	0.013	0.092
Nigeria	Ikono	2000	0.299	0.444	0.646
Nigeria	Ikono	2017	0.099	0.159	0.237
Nigeria	Ikono	2000-2017	-0.091	-0.001	0.087
Nigeria	Ikorodu	2000	0.278	0.423	0.618
Nigeria	Ikorodu	2017	0.102	0.163	0.228
Nigeria	Ikorodu	2000-2017	-0.146	-0.053	0.039
Nigeria	Ikot-Aba	2000	0.290	0.429	0.607
Nigeria	Ikot-Aba	2017	0.103	0.158	0.231
Nigeria	Ikot-Aba	2000-2017	-0.104	-0.002	0.097
Nigeria	Ikot-Ekp	2000	0.284	0.426	0.614
Nigeria	Ikot-Ekp	2017	0.098	0.155	0.231
Nigeria	Ikot-Ekp	2000-2017	-0.089	-0.001	0.086
Nigeria	Ikpoba-Okha	2000	0.267	0.416	0.640
Nigeria	Ikpoba-Okha	2017	0.101	0.154	0.227
Nigeria	Ikpoba-Okha	2000-2017	-0.147	-0.053	0.034
Nigeria	Ikwerre	2000	0.268	0.419	0.631
Nigeria	Ikwerre	2017	0.100	0.158	0.242
Nigeria	Ikwerre	2000-2017	-0.118	-0.023	0.060
Nigeria	Ikwo	2000	0.308	0.471	0.718
Nigeria	Ikwo	2017	0.096	0.168	0.279
Nigeria	Ikwo	2000-2017	-0.085	0.008	0.096
Nigeria	Ikwuano	2000	0.297	0.440	0.643
Nigeria	Ikwuano	2017	0.100	0.165	0.248
Nigeria	Ikwuano	2000-2017	-0.095	-0.003	0.091
Nigeria	Ila	2000	0.295	0.497	0.758
Nigeria	Ila	2017	0.106	0.169	0.243
Nigeria	Ila	2000-2017	-0.147	-0.057	0.027

Table 2: Diarrhea DALYs rate by unit *(continued)*

Country	Unit	year	mean rate	lower rate	upper rate
Nigeria	IlajeEseodo	2000	0.268	0.432	0.639
Nigeria	IlajeEseodo	2017	0.106	0.168	0.246
Nigeria	IlajeEseodo	2000-2017	-0.133	-0.048	0.051
Nigeria	Ilejemeje	2000	0.260	0.430	0.663
Nigeria	Ilejemeje	2017	0.091	0.147	0.216
Nigeria	Ilejemeje	2000-2017	-0.144	-0.055	0.036
Nigeria	IleOluji/Okeigbo	2000	0.286	0.481	0.756
Nigeria	IleOluji/Okeigbo	2017	0.098	0.164	0.245
Nigeria	IleOluji/Okeigbo	2000-2017	-0.157	-0.069	0.024
Nigeria	Ilesha East	2000	0.257	0.439	0.667
Nigeria	Ilesha East	2017	0.091	0.151	0.216
Nigeria	Ilesha East	2000-2017	-0.156	-0.064	0.026
Nigeria	Ilesha West	2000	0.253	0.433	0.657
Nigeria	Ilesha West	2017	0.091	0.150	0.216
Nigeria	Ilesha West	2000-2017	-0.157	-0.064	0.026
Nigeria	Illela	2000	0.638	0.897	1.251
Nigeria	Illela	2017	0.261	0.395	0.591
Nigeria	Illela	2000-2017	-0.110	-0.020	0.078
Nigeria	Ilorin East	2000	0.398	0.618	0.950
Nigeria	Ilorin East	2017	0.113	0.189	0.278
Nigeria	Ilorin East	2000-2017	-0.164	-0.070	0.024
Nigeria	Ilorin South	2000	0.404	0.628	0.961
Nigeria	Ilorin South	2017	0.115	0.190	0.280
Nigeria	Ilorin South	2000-2017	-0.164	-0.071	0.021
Nigeria	IlorinWe	2000	0.381	0.599	0.924
Nigeria	IlorinWe	2017	0.111	0.185	0.271
Nigeria	IlorinWe	2000-2017	-0.166	-0.070	0.022
Nigeria	Imeko-Afon	2000	0.368	0.554	0.792
Nigeria	Imeko-Afon	2017	0.129	0.191	0.267
Nigeria	Imeko-Afon	2000-2017	-0.137	-0.045	0.054
Nigeria	Ingawa	2000	0.594	0.843	1.144
Nigeria	Ingawa	2017	0.224	0.352	0.536
Nigeria	Ingawa	2000-2017	-0.113	-0.025	0.061
Nigeria	Ini	2000	0.304	0.451	0.650
Nigeria	Ini	2017	0.103	0.168	0.253
Nigeria	Ini	2000-2017	-0.087	0.004	0.091
Nigeria	Ipokia	2000	0.292	0.451	0.639
Nigeria	Ipokia	2017	0.132	0.185	0.243
Nigeria	Ipokia	2000-2017	-0.119	-0.033	0.058
Nigeria	Irele	2000	0.270	0.440	0.661
Nigeria	Irele	2017	0.098	0.158	0.229
Nigeria	Irele	2000-2017	-0.137	-0.051	0.045
Nigeria	Irepo	2000	0.409	0.634	0.983
Nigeria	Irepo	2017	0.113	0.179	0.254
Nigeria	Irepo	2000-2017	-0.161	-0.072	0.018
Nigeria	Irepodun	2000	0.257	0.424	0.667
Nigeria	Irepodun	2000	0.362	0.586	0.884
Nigeria	Irepodun	2017	0.092	0.147	0.212
Nigeria	Irepodun	2017	0.114	0.184	0.263
Nigeria	Irepodun	2000-2017	-0.152	-0.066	0.024
Nigeria	Irepodun	2000-2017	-0.147	-0.057	0.033
Nigeria	Irepodun/Ifelodun	2000	0.270	0.448	0.690
Nigeria	Irepodun/Ifelodun	2017	0.098	0.158	0.231
Nigeria	Irepodun/Ifelodun	2000-2017	-0.143	-0.053	0.040
Nigeria	Irewole	2000	0.272	0.437	0.689
Nigeria	Irewole	2017	0.095	0.157	0.234
Nigeria	Irewole	2000-2017	-0.150	-0.063	0.027
Nigeria	Isa	2000	0.673	0.921	1.256
Nigeria	Isa	2017	0.249	0.390	0.598
Nigeria	Isa	2000-2017	-0.109	-0.027	0.071
Nigeria	Ise/Orun	2000	0.267	0.453	0.720

Table 2: Diarrhea DALYs rate by unit *(continued)*

Country	Unit	year	mean rate	lower rate	upper rate
Nigeria	Ise/Orun	2017	0.102	0.165	0.243
Nigeria	Ise/Orun	2000-2017	-0.143	-0.051	0.040
Nigeria	Iseyin	2000	0.294	0.456	0.654
Nigeria	Iseyin	2017	0.100	0.153	0.216
Nigeria	Iseyin	2000-2017	-0.140	-0.049	0.038
Nigeria	Ishielu	2000	0.311	0.480	0.752
Nigeria	Ishielu	2017	0.102	0.176	0.287
Nigeria	Ishielu	2000-2017	-0.095	-0.003	0.084
Nigeria	Isi-Uzo	2000	0.265	0.416	0.658
Nigeria	Isi-Uzo	2017	0.092	0.151	0.239
Nigeria	Isi-Uzo	2000-2017	-0.087	0.001	0.088
Nigeria	Isiala Ngwa North	2000	0.282	0.420	0.607
Nigeria	Isiala Ngwa North	2017	0.093	0.156	0.235
Nigeria	Isiala Ngwa North	2000-2017	-0.101	-0.012	0.082
Nigeria	Isiala Ngwa South	2000	0.277	0.425	0.616
Nigeria	Isiala Ngwa South	2017	0.095	0.158	0.239
Nigeria	Isiala Ngwa South	2000-2017	-0.100	-0.013	0.075
Nigeria	IsialaMb	2000	0.282	0.428	0.652
Nigeria	IsialaMb	2017	0.095	0.160	0.249
Nigeria	IsialaMb	2000-2017	-0.102	-0.009	0.085
Nigeria	Isin	2000	0.339	0.545	0.829
Nigeria	Isin	2017	0.105	0.171	0.245
Nigeria	Isin	2000-2017	-0.155	-0.069	0.023
Nigeria	Isokan	2000	0.277	0.442	0.692
Nigeria	Isokan	2017	0.102	0.166	0.246
Nigeria	Isokan	2000-2017	-0.147	-0.062	0.024
Nigeria	IsokoNor	2000	0.254	0.398	0.603
Nigeria	IsokoNor	2017	0.097	0.155	0.245
Nigeria	IsokoNor	2000-2017	-0.124	-0.026	0.058
Nigeria	IsokoSou	2000	0.265	0.412	0.628
Nigeria	IsokoSou	2017	0.101	0.160	0.253
Nigeria	IsokoSou	2000-2017	-0.124	-0.026	0.061
Nigeria	Isu	2000	0.257	0.395	0.594
Nigeria	Isu	2017	0.087	0.146	0.230
Nigeria	Isu	2000-2017	-0.103	-0.009	0.081
Nigeria	Isuikwua	2000	0.284	0.423	0.642
Nigeria	Isuikwua	2017	0.095	0.160	0.252
Nigeria	Isuikwua	2000-2017	-0.091	0.000	0.094
Nigeria	Itas/Gad	2000	0.557	0.817	1.182
Nigeria	Itas/Gad	2017	0.242	0.410	0.625
Nigeria	Itas/Gad	2000-2017	-0.103	-0.008	0.089
Nigeria	Itesiwaju	2000	0.337	0.512	0.733
Nigeria	Itesiwaju	2017	0.106	0.163	0.229
Nigeria	Itesiwaju	2000-2017	-0.143	-0.051	0.034
Nigeria	Itu	2000	0.308	0.455	0.658
Nigeria	Itu	2017	0.099	0.164	0.245
Nigeria	Itu	2000-2017	-0.091	0.005	0.090
Nigeria	Ivo	2000	0.340	0.512	0.798
Nigeria	Ivo	2017	0.111	0.190	0.307
Nigeria	Ivo	2000-2017	-0.106	-0.009	0.082
Nigeria	Iwajowa	2000	0.338	0.515	0.733
Nigeria	Iwajowa	2017	0.110	0.161	0.227
Nigeria	Iwajowa	2000-2017	-0.138	-0.048	0.045
Nigeria	Iwo	2000	0.251	0.405	0.633
Nigeria	Iwo	2017	0.088	0.143	0.205
Nigeria	Iwo	2000-2017	-0.142	-0.055	0.034
Nigeria	Izzi	2000	0.289	0.454	0.709
Nigeria	Izzi	2017	0.096	0.160	0.262
Nigeria	Izzi	2000-2017	-0.080	0.008	0.090
Nigeria	Jaba	2000	0.372	0.610	0.967
Nigeria	Jaba	2017	0.135	0.215	0.327

Table 2: Diarrhea DALYs rate by unit *(continued)*

Country	Unit	year	mean rate	lower rate	upper rate
Nigeria	Jaba	2000-2017	-0.128	-0.035	0.058
Nigeria	Jada	2000	0.551	0.794	1.122
Nigeria	Jada	2017	0.172	0.295	0.456
Nigeria	Jada	2000-2017	-0.109	-0.020	0.072
Nigeria	Jahun	2000	0.573	0.845	1.199
Nigeria	Jahun	2017	0.237	0.385	0.591
Nigeria	Jahun	2000-2017	-0.096	-0.013	0.086
Nigeria	Jakusko	2000	0.553	0.816	1.139
Nigeria	Jakusko	2017	0.244	0.391	0.630
Nigeria	Jakusko	2000-2017	-0.101	-0.010	0.084
Nigeria	Jalingo	2000	0.507	0.804	1.177
Nigeria	Jalingo	2017	0.188	0.313	0.499
Nigeria	Jalingo	2000-2017	-0.109	-0.001	0.085
Nigeria	Jama'are	2000	0.520	0.781	1.144
Nigeria	Jama'are	2017	0.224	0.386	0.603
Nigeria	Jama'are	2000-2017	-0.099	-0.007	0.089
Nigeria	Jega	2000	0.651	0.915	1.326
Nigeria	Jega	2017	0.261	0.365	0.513
Nigeria	Jega	2000-2017	-0.109	-0.030	0.055
Nigeria	Jema'a	2000	0.405	0.652	0.996
Nigeria	Jema'a	2017	0.145	0.233	0.362
Nigeria	Jema'a	2000-2017	-0.117	-0.032	0.062
Nigeria	Jere	2000	0.653	0.953	1.308
Nigeria	Jere	2017	0.229	0.405	0.664
Nigeria	Jere	2000-2017	-0.136	-0.043	0.040
Nigeria	Jibia	2000	0.636	0.902	1.210
Nigeria	Jibia	2017	0.225	0.355	0.520
Nigeria	Jibia	2000-2017	-0.119	-0.029	0.056
Nigeria	Jos East	2000	0.373	0.613	0.908
Nigeria	Jos East	2017	0.141	0.235	0.376
Nigeria	Jos East	2000-2017	-0.121	-0.033	0.057
Nigeria	Jos North	2000	0.360	0.588	0.901
Nigeria	Jos North	2017	0.142	0.233	0.364
Nigeria	Jos North	2000-2017	-0.123	-0.038	0.053
Nigeria	Jos South	2000	0.336	0.553	0.839
Nigeria	Jos South	2017	0.133	0.218	0.345
Nigeria	Jos South	2000-2017	-0.115	-0.032	0.061
Nigeria	Kabba/Bu	2000	0.337	0.523	0.827
Nigeria	Kabba/Bu	2017	0.111	0.188	0.287
Nigeria	Kabba/Bu	2000-2017	-0.137	-0.046	0.039
Nigeria	Kabo	2000	0.567	0.830	1.210
Nigeria	Kabo	2017	0.240	0.382	0.587
Nigeria	Kabo	2000-2017	-0.107	-0.016	0.069
Nigeria	Kachia	2000	0.418	0.663	1.017
Nigeria	Kachia	2017	0.153	0.246	0.378
Nigeria	Kachia	2000-2017	-0.120	-0.033	0.051
Nigeria	Kaduna North	2000	0.484	0.744	1.086
Nigeria	Kaduna North	2017	0.175	0.278	0.426
Nigeria	Kaduna North	2000-2017	-0.126	-0.033	0.052
Nigeria	Kaduna South	2000	0.462	0.713	1.040
Nigeria	Kaduna South	2017	0.167	0.265	0.404
Nigeria	Kaduna South	2000-2017	-0.128	-0.034	0.054
Nigeria	KafinHau	2000	0.573	0.816	1.130
Nigeria	KafinHau	2017	0.239	0.399	0.629
Nigeria	KafinHau	2000-2017	-0.099	-0.006	0.096
Nigeria	Kafur	2000	0.566	0.839	1.215
Nigeria	Kafur	2017	0.224	0.357	0.541
Nigeria	Kafur	2000-2017	-0.117	-0.026	0.057
Nigeria	Kaga	2000	0.677	0.995	1.375
Nigeria	Kaga	2017	0.250	0.449	0.711
Nigeria	Kaga	2000-2017	-0.122	-0.027	0.065

Table 2: Diarrhea DALYs rate by unit *(continued)*

Country	Unit	year	mean rate	lower rate	upper rate
Nigeria	Kagarko	2000	0.400	0.628	0.968
Nigeria	Kagarko	2017	0.142	0.223	0.333
Nigeria	Kagarko	2000-2017	-0.135	-0.038	0.056
Nigeria	Kaiama	2000	0.468	0.727	1.091
Nigeria	Kaiama	2017	0.137	0.216	0.310
Nigeria	Kaiama	2000-2017	-0.146	-0.060	0.031
Nigeria	Kaita	2000	0.628	0.870	1.167
Nigeria	Kaita	2017	0.220	0.348	0.529
Nigeria	Kaita	2000-2017	-0.113	-0.028	0.065
Nigeria	Kajola	2000	0.327	0.497	0.716
Nigeria	Kajola	2017	0.104	0.157	0.226
Nigeria	Kajola	2000-2017	-0.146	-0.050	0.036
Nigeria	Kajuru	2000	0.398	0.635	0.954
Nigeria	Kajuru	2017	0.156	0.250	0.373
Nigeria	Kajuru	2000-2017	-0.124	-0.033	0.065
Nigeria	Kala/Balge	2000	0.798	1.015	1.288
Nigeria	Kala/Balge	2017	0.272	0.451	0.725
Nigeria	Kala/Balge	2000-2017	-0.130	-0.038	0.059
Nigeria	Kalgo	2000	0.654	0.906	1.293
Nigeria	Kalgo	2017	0.264	0.364	0.503
Nigeria	Kalgo	2000-2017	-0.105	-0.027	0.061
Nigeria	Kaltungo	2000	0.519	0.829	1.156
Nigeria	Kaltungo	2017	0.206	0.343	0.543
Nigeria	Kaltungo	2000-2017	-0.112	-0.020	0.070
Nigeria	Kanam	2000	0.499	0.781	1.156
Nigeria	Kanam	2017	0.171	0.290	0.443
Nigeria	Kanam	2000-2017	-0.119	-0.022	0.060
Nigeria	Kankara	2000	0.533	0.791	1.142
Nigeria	Kankara	2017	0.209	0.330	0.503
Nigeria	Kankara	2000-2017	-0.135	-0.033	0.053
Nigeria	Kanke	2000	0.407	0.668	0.981
Nigeria	Kanke	2017	0.142	0.236	0.355
Nigeria	Kanke	2000-2017	-0.119	-0.033	0.051
Nigeria	Kankiya	2000	0.599	0.848	1.162
Nigeria	Kankiya	2017	0.216	0.342	0.521
Nigeria	Kankiya	2000-2017	-0.124	-0.028	0.057
Nigeria	Kano	2000	0.555	0.805	1.147
Nigeria	Kano	2017	0.222	0.340	0.510
Nigeria	Kano	2000-2017	-0.099	-0.014	0.079
Nigeria	Karasuwa	2000	0.571	0.804	1.144
Nigeria	Karasuwa	2017	0.240	0.378	0.626
Nigeria	Karasuwa	2000-2017	-0.102	-0.009	0.088
Nigeria	Karaye	2000	0.535	0.792	1.137
Nigeria	Karaye	2017	0.225	0.356	0.547
Nigeria	Karaye	2000-2017	-0.113	-0.022	0.065
Nigeria	Karim-La	2000	0.522	0.833	1.219
Nigeria	Karim-La	2017	0.205	0.338	0.521
Nigeria	Karim-La	2000-2017	-0.093	-0.002	0.076
Nigeria	Karu	2000	0.389	0.615	0.962
Nigeria	Karu	2017	0.133	0.220	0.337
Nigeria	Karu	2000-2017	-0.125	-0.043	0.052
Nigeria	Katagum	2000	0.508	0.779	1.137
Nigeria	Katagum	2017	0.221	0.383	0.579
Nigeria	Katagum	2000-2017	-0.098	-0.007	0.091
Nigeria	Katcha	2000	0.399	0.613	0.956
Nigeria	Katcha	2017	0.143	0.234	0.351
Nigeria	Katcha	2000-2017	-0.133	-0.041	0.047
Nigeria	Katsina (Benue)	2000	0.375	0.620	0.942
Nigeria	Katsina (Benue)	2017	0.140	0.223	0.353
Nigeria	Katsina (Benue)	2000-2017	-0.090	0.014	0.102
Nigeria	Katsina (K)	2000	0.609	0.856	1.157

Table 2: Diarrhea DALYs rate by unit *(continued)*

Country	Unit	year	mean rate	lower rate	upper rate
Nigeria	Katsina (K)	2017	0.215	0.338	0.510
Nigeria	Katsina (K)	2000-2017	-0.112	-0.025	0.065
Nigeria	Kaugama	2000	0.584	0.830	1.143
Nigeria	Kaugama	2017	0.241	0.392	0.608
Nigeria	Kaugama	2000-2017	-0.097	-0.009	0.090
Nigeria	Kaura	2000	0.420	0.668	1.021
Nigeria	Kaura	2017	0.152	0.243	0.379
Nigeria	Kaura	2000-2017	-0.111	-0.032	0.061
Nigeria	Kaura-Na	2000	0.631	0.887	1.258
Nigeria	Kaura-Na	2017	0.237	0.373	0.575
Nigeria	Kaura-Na	2000-2017	-0.135	-0.038	0.059
Nigeria	Kauru	2000	0.421	0.657	1.006
Nigeria	Kauru	2017	0.180	0.276	0.415
Nigeria	Kauru	2000-2017	-0.101	-0.024	0.065
Nigeria	Kazaure	2000	0.618	0.904	1.255
Nigeria	Kazaure	2017	0.233	0.366	0.579
Nigeria	Kazaure	2000-2017	-0.110	-0.022	0.061
Nigeria	Keana	2000	0.385	0.629	0.962
Nigeria	Keana	2017	0.141	0.226	0.343
Nigeria	Keana	2000-2017	-0.105	-0.014	0.077
Nigeria	Kebbe	2000	0.655	0.960	1.340
Nigeria	Kebbe	2017	0.261	0.376	0.547
Nigeria	Kebbe	2000-2017	-0.118	-0.032	0.063
Nigeria	Keffi	2000	0.407	0.653	1.007
Nigeria	Keffi	2017	0.141	0.233	0.363
Nigeria	Keffi	2000-2017	-0.118	-0.037	0.061
Nigeria	Khana	2000	0.293	0.432	0.627
Nigeria	Khana	2017	0.104	0.160	0.239
Nigeria	Khana	2000-2017	-0.107	-0.005	0.097
Nigeria	Kibiya	2000	0.486	0.735	1.075
Nigeria	Kibiya	2017	0.237	0.370	0.561
Nigeria	Kibiya	2000-2017	-0.099	-0.010	0.084
Nigeria	Kirfi	2000	0.593	0.948	1.391
Nigeria	Kirfi	2017	0.238	0.398	0.607
Nigeria	Kirfi	2000-2017	-0.109	-0.016	0.078
Nigeria	KiriKasa	2000	0.607	0.844	1.139
Nigeria	KiriKasa	2017	0.252	0.395	0.633
Nigeria	KiriKasa	2000-2017	-0.097	-0.007	0.093
Nigeria	Kiru	2000	0.503	0.771	1.115
Nigeria	Kiru	2017	0.223	0.356	0.538
Nigeria	Kiru	2000-2017	-0.114	-0.018	0.066
Nigeria	Kiyawa	2000	0.546	0.825	1.203
Nigeria	Kiyawa	2017	0.239	0.394	0.593
Nigeria	Kiyawa	2000-2017	-0.095	-0.009	0.083
Nigeria	Koko/Bes	2000	0.636	0.922	1.305
Nigeria	Koko/Bes	2017	0.235	0.339	0.466
Nigeria	Koko/Bes	2000-2017	-0.117	-0.034	0.057
Nigeria	Kokona	2000	0.429	0.682	1.053
Nigeria	Kokona	2017	0.154	0.251	0.389
Nigeria	Kokona	2000-2017	-0.118	-0.030	0.065
Nigeria	Kolokuma/Opokuma	2000	0.319	0.495	0.754
Nigeria	Kolokuma/Opokuma	2017	0.124	0.193	0.298
Nigeria	Kolokuma/Opokuma	2000-2017	-0.117	-0.024	0.067
Nigeria	Konduga	2000	0.676	0.972	1.346
Nigeria	Konduga	2017	0.237	0.412	0.680
Nigeria	Konduga	2000-2017	-0.132	-0.043	0.042
Nigeria	Konshish	2000	0.341	0.557	0.839
Nigeria	Konshish	2017	0.122	0.196	0.316
Nigeria	Konshish	2000-2017	-0.088	0.008	0.092
Nigeria	Kontogur	2000	0.448	0.708	1.092
Nigeria	Kontogur	2017	0.166	0.252	0.396

Table 2: Diarrhea DALYs rate by unit *(continued)*

Country	Unit	year	mean rate	lower rate	upper rate
Nigeria	Kontogur	2000-2017	-0.140	-0.046	0.056
Nigeria	Kosofe	2000	0.276	0.422	0.599
Nigeria	Kosofe	2017	0.108	0.169	0.233
Nigeria	Kosofe	2000-2017	-0.139	-0.045	0.049
Nigeria	Kotonkar	2000	0.359	0.557	0.888
Nigeria	Kotonkar	2017	0.124	0.211	0.348
Nigeria	Kotonkar	2000-2017	-0.132	-0.035	0.042
Nigeria	Kubau	2000	0.472	0.725	1.066
Nigeria	Kubau	2017	0.213	0.323	0.477
Nigeria	Kubau	2000-2017	-0.098	-0.015	0.076
Nigeria	Kudan	2000	0.540	0.812	1.179
Nigeria	Kudan	2017	0.223	0.345	0.518
Nigeria	Kudan	2000-2017	-0.110	-0.018	0.071
Nigeria	Kuje	2000	0.375	0.576	0.882
Nigeria	Kuje	2017	0.127	0.205	0.328
Nigeria	Kuje	2000-2017	-0.147	-0.051	0.029
Nigeria	Kukawa	2000	0.635	0.874	1.201
Nigeria	Kukawa	2017	0.255	0.415	0.654
Nigeria	Kukawa	2000-2017	-0.131	-0.029	0.067
Nigeria	Kumbotso	2000	0.562	0.812	1.163
Nigeria	Kumbotso	2017	0.228	0.351	0.526
Nigeria	Kumbotso	2000-2017	-0.096	-0.012	0.080
Nigeria	Kunchi	2000	0.589	0.856	1.196
Nigeria	Kunchi	2017	0.231	0.356	0.555
Nigeria	Kunchi	2000-2017	-0.112	-0.023	0.067
Nigeria	Kura	2000	0.560	0.829	1.191
Nigeria	Kura	2017	0.239	0.377	0.569
Nigeria	Kura	2000-2017	-0.097	-0.011	0.081
Nigeria	Kurfi	2000	0.576	0.826	1.136
Nigeria	Kurfi	2017	0.213	0.332	0.503
Nigeria	Kurfi	2000-2017	-0.121	-0.032	0.056
Nigeria	Kurmi	2000	0.412	0.631	0.893
Nigeria	Kurmi	2017	0.152	0.253	0.413
Nigeria	Kurmi	2000-2017	-0.071	0.025	0.111
Nigeria	Kusada	2000	0.583	0.838	1.170
Nigeria	Kusada	2017	0.219	0.341	0.513
Nigeria	Kusada	2000-2017	-0.115	-0.026	0.060
Nigeria	Kwali	2000	0.404	0.627	0.979
Nigeria	Kwali	2017	0.135	0.223	0.361
Nigeria	Kwali	2000-2017	-0.148	-0.053	0.029
Nigeria	Kwami	2000	0.527	0.861	1.212
Nigeria	Kwami	2017	0.238	0.384	0.583
Nigeria	Kwami	2000-2017	-0.118	-0.017	0.084
Nigeria	Kwande	2000	0.346	0.536	0.786
Nigeria	Kwande	2017	0.115	0.190	0.307
Nigeria	Kwande	2000-2017	-0.080	0.017	0.099
Nigeria	Kware	2000	0.586	0.857	1.230
Nigeria	Kware	2017	0.262	0.388	0.547
Nigeria	Kware	2000-2017	-0.107	-0.022	0.070
Nigeria	Kwaya Kusar	2000	0.605	0.933	1.293
Nigeria	Kwaya Kusar	2017	0.235	0.395	0.619
Nigeria	Kwaya Kusar	2000-2017	-0.116	-0.024	0.074
Nigeria	Lafia	2000	0.365	0.601	0.909
Nigeria	Lafia	2017	0.129	0.210	0.324
Nigeria	Lafia	2000-2017	-0.115	-0.023	0.065
Nigeria	Lagelu	2000	0.280	0.441	0.684
Nigeria	Lagelu	2017	0.096	0.154	0.220
Nigeria	Lagelu	2000-2017	-0.152	-0.062	0.023
Nigeria	LagosIsland	2000	0.262	0.401	0.567
Nigeria	LagosIsland	2017	0.102	0.163	0.226
Nigeria	LagosIsland	2000-2017	-0.137	-0.045	0.051

Table 2: Diarrhea DALYs rate by unit *(continued)*

Country	Unit	year	mean rate	lower rate	upper rate
Nigeria	Lake Chad	2000	0.663	0.894	1.231
Nigeria	Lake Chad	2017	0.270	0.440	0.696
Nigeria	Lake Chad	2000-2017	-0.121	-0.027	0.066
Nigeria	Lamurde	2000	0.579	0.875	1.226
Nigeria	Lamurde	2017	0.202	0.337	0.527
Nigeria	Lamurde	2000-2017	-0.118	-0.026	0.063
Nigeria	Langtang North	2000	0.437	0.700	1.007
Nigeria	Langtang North	2017	0.149	0.245	0.367
Nigeria	Langtang North	2000-2017	-0.113	-0.024	0.058
Nigeria	Langtang South	2000	0.361	0.610	0.906
Nigeria	Langtang South	2017	0.135	0.218	0.341
Nigeria	Langtang South	2000-2017	-0.102	-0.011	0.073
Nigeria	Lapai	2000	0.434	0.659	1.019
Nigeria	Lapai	2017	0.148	0.245	0.379
Nigeria	Lapai	2000-2017	-0.126	-0.037	0.049
Nigeria	Lau	2000	0.559	0.846	1.230
Nigeria	Lau	2017	0.201	0.336	0.513
Nigeria	Lau	2000-2017	-0.106	-0.006	0.080
Nigeria	Lavun	2000	0.414	0.644	1.000
Nigeria	Lavun	2017	0.140	0.234	0.348
Nigeria	Lavun	2000-2017	-0.132	-0.047	0.046
Nigeria	Lere	2000	0.436	0.682	1.018
Nigeria	Lere	2017	0.180	0.283	0.433
Nigeria	Lere	2000-2017	-0.107	-0.026	0.074
Nigeria	Logo	2000	0.353	0.598	0.902
Nigeria	Logo	2017	0.139	0.223	0.345
Nigeria	Logo	2000-2017	-0.090	0.009	0.091
Nigeria	Lokoja	2000	0.365	0.553	0.876
Nigeria	Lokoja	2017	0.125	0.208	0.325
Nigeria	Lokoja	2000-2017	-0.128	-0.037	0.045
Nigeria	Machina	2000	0.567	0.798	1.064
Nigeria	Machina	2017	0.223	0.359	0.596
Nigeria	Machina	2000-2017	-0.103	-0.009	0.089
Nigeria	Madagali	2000	0.734	1.005	1.349
Nigeria	Madagali	2017	0.231	0.402	0.650
Nigeria	Madagali	2000-2017	-0.135	-0.051	0.044
Nigeria	Madobi	2000	0.550	0.806	1.156
Nigeria	Madobi	2017	0.231	0.368	0.558
Nigeria	Madobi	2000-2017	-0.097	-0.012	0.075
Nigeria	Mafa	2000	0.694	0.953	1.238
Nigeria	Mafa	2017	0.238	0.417	0.667
Nigeria	Mafa	2000-2017	-0.130	-0.042	0.051
Nigeria	Magama	2000	0.479	0.783	1.178
Nigeria	Magama	2017	0.181	0.271	0.405
Nigeria	Magama	2000-2017	-0.134	-0.045	0.052
Nigeria	Magumeri	2000	0.640	0.931	1.292
Nigeria	Magumeri	2017	0.227	0.412	0.672
Nigeria	Magumeri	2000-2017	-0.130	-0.032	0.056
Nigeria	Mai'Adua	2000	0.571	0.829	1.110
Nigeria	Mai'Adua	2017	0.210	0.338	0.525
Nigeria	Mai'Adua	2000-2017	-0.108	-0.021	0.062
Nigeria	Maidugur	2000	0.657	0.957	1.327
Nigeria	Maidugur	2017	0.227	0.402	0.663
Nigeria	Maidugur	2000-2017	-0.138	-0.045	0.039
Nigeria	Maigatari	2000	0.607	0.856	1.141
Nigeria	Maigatari	2017	0.240	0.380	0.612
Nigeria	Maigatari	2000-2017	-0.107	-0.012	0.088
Nigeria	Maiha	2000	0.834	1.115	1.451
Nigeria	Maiha	2017	0.226	0.389	0.650
Nigeria	Maiha	2000-2017	-0.154	-0.049	0.050
Nigeria	Mainland	2000	0.252	0.383	0.551

Table 2: Diarrhea DALYs rate by unit *(continued)*

Country	Unit	year	mean rate	lower rate	upper rate
Nigeria	Mainland	2017	0.109	0.172	0.240
Nigeria	Mainland	2000-2017	-0.126	-0.035	0.060
Nigeria	Maiyama	2000	0.647	0.905	1.294
Nigeria	Maiyama	2017	0.253	0.355	0.499
Nigeria	Maiyama	2000-2017	-0.115	-0.034	0.057
Nigeria	Makarfi	2000	0.515	0.800	1.152
Nigeria	Makarfi	2017	0.231	0.354	0.538
Nigeria	Makarfi	2000-2017	-0.111	-0.017	0.067
Nigeria	Makoda	2000	0.573	0.830	1.180
Nigeria	Makoda	2017	0.224	0.348	0.547
Nigeria	Makoda	2000-2017	-0.106	-0.024	0.060
Nigeria	Makurdi	2000	0.334	0.563	0.873
Nigeria	Makurdi	2017	0.127	0.203	0.304
Nigeria	Makurdi	2000-2017	-0.104	-0.007	0.081
Nigeria	MalamMad	2000	0.578	0.811	1.089
Nigeria	MalamMad	2017	0.249	0.389	0.610
Nigeria	MalamMad	2000-2017	-0.102	-0.006	0.095
Nigeria	Malumfashi	2000	0.564	0.841	1.224
Nigeria	Malumfashi	2017	0.218	0.351	0.533
Nigeria	Malumfashi	2000-2017	-0.119	-0.026	0.059
Nigeria	Mangu	2000	0.361	0.614	0.893
Nigeria	Mangu	2017	0.131	0.219	0.323
Nigeria	Mangu	2000-2017	-0.115	-0.036	0.051
Nigeria	Mani	2000	0.566	0.820	1.127
Nigeria	Mani	2017	0.212	0.341	0.514
Nigeria	Mani	2000-2017	-0.108	-0.025	0.060
Nigeria	Maradun	2000	0.659	0.920	1.282
Nigeria	Maradun	2017	0.264	0.407	0.611
Nigeria	Maradun	2000-2017	-0.123	-0.034	0.068
Nigeria	Mariga	2000	0.494	0.756	1.138
Nigeria	Mariga	2017	0.192	0.291	0.447
Nigeria	Mariga	2000-2017	-0.129	-0.035	0.057
Nigeria	Marte	2000	0.684	0.936	1.241
Nigeria	Marte	2017	0.254	0.436	0.696
Nigeria	Marte	2000-2017	-0.136	-0.036	0.057
Nigeria	Maru	2000	0.623	0.919	1.292
Nigeria	Maru	2017	0.256	0.385	0.581
Nigeria	Maru	2000-2017	-0.113	-0.032	0.067
Nigeria	Mashegu	2000	0.464	0.719	1.048
Nigeria	Mashegu	2017	0.165	0.253	0.381
Nigeria	Mashegu	2000-2017	-0.135	-0.045	0.040
Nigeria	Mashi	2000	0.572	0.824	1.111
Nigeria	Mashi	2017	0.211	0.340	0.524
Nigeria	Mashi	2000-2017	-0.111	-0.026	0.063
Nigeria	Matazu	2000	0.565	0.820	1.144
Nigeria	Matazu	2017	0.207	0.329	0.504
Nigeria	Matazu	2000-2017	-0.125	-0.031	0.058
Nigeria	Mayo-Bel	2000	0.552	0.819	1.148
Nigeria	Mayo-Bel	2017	0.173	0.308	0.466
Nigeria	Mayo-Bel	2000-2017	-0.120	-0.019	0.069
Nigeria	Mbaitoli	2000	0.260	0.406	0.611
Nigeria	Mbaitoli	2017	0.091	0.151	0.233
Nigeria	Mbaitoli	2000-2017	-0.102	-0.010	0.080
Nigeria	Mbo	2000	0.321	0.486	0.667
Nigeria	Mbo	2017	0.113	0.178	0.270
Nigeria	Mbo	2000-2017	-0.088	0.008	0.097
Nigeria	Michika	2000	0.711	0.967	1.345
Nigeria	Michika	2017	0.223	0.378	0.623
Nigeria	Michika	2000-2017	-0.140	-0.054	0.045
Nigeria	Miga	2000	0.588	0.852	1.204
Nigeria	Miga	2017	0.242	0.393	0.612

Table 2: Diarrhea DALYs rate by unit *(continued)*

Country	Unit	year	mean rate	lower rate	upper rate
Nigeria	Miga	2000-2017	-0.095	-0.010	0.088
Nigeria	Mikang	2000	0.403	0.668	0.978
Nigeria	Mikang	2017	0.141	0.226	0.335
Nigeria	Mikang	2000-2017	-0.116	-0.026	0.056
Nigeria	Minjibir	2000	0.563	0.812	1.155
Nigeria	Minjibir	2017	0.234	0.364	0.561
Nigeria	Minjibir	2000-2017	-0.100	-0.014	0.073
Nigeria	Misau	2000	0.508	0.805	1.177
Nigeria	Misau	2017	0.217	0.380	0.581
Nigeria	Misau	2000-2017	-0.110	-0.012	0.088
Nigeria	Mkpat Enin	2000	0.273	0.412	0.568
Nigeria	Mkpat Enin	2017	0.097	0.150	0.218
Nigeria	Mkpat Enin	2000-2017	-0.106	-0.001	0.092
Nigeria	Moba	2000	0.272	0.450	0.685
Nigeria	Moba	2017	0.095	0.152	0.224
Nigeria	Moba	2000-2017	-0.142	-0.054	0.037
Nigeria	Mobbar	2000	0.570	0.821	1.185
Nigeria	Mobbar	2017	0.236	0.385	0.599
Nigeria	Mobbar	2000-2017	-0.119	-0.029	0.059
Nigeria	Mokwa	2000	0.445	0.691	1.038
Nigeria	Mokwa	2017	0.147	0.236	0.349
Nigeria	Mokwa	2000-2017	-0.139	-0.051	0.030
Nigeria	Monguno	2000	0.638	0.922	1.268
Nigeria	Monguno	2017	0.255	0.430	0.705
Nigeria	Monguno	2000-2017	-0.129	-0.031	0.057
Nigeria	Mopa-Muro	2000	0.332	0.520	0.828
Nigeria	Mopa-Muro	2017	0.110	0.178	0.273
Nigeria	Mopa-Muro	2000-2017	-0.138	-0.048	0.039
Nigeria	Moro	2000	0.449	0.690	1.068
Nigeria	Moro	2017	0.125	0.208	0.307
Nigeria	Moro	2000-2017	-0.162	-0.066	0.021
Nigeria	Mubi North	2000	0.709	0.960	1.298
Nigeria	Mubi North	2017	0.224	0.374	0.617
Nigeria	Mubi North	2000-2017	-0.150	-0.055	0.047
Nigeria	Mubi South	2000	0.749	1.004	1.339
Nigeria	Mubi South	2017	0.224	0.380	0.630
Nigeria	Mubi South	2000-2017	-0.155	-0.055	0.046
Nigeria	Musawa	2000	0.563	0.819	1.183
Nigeria	Musawa	2017	0.207	0.331	0.502
Nigeria	Musawa	2000-2017	-0.126	-0.031	0.056
Nigeria	Mushin	2000	0.235	0.360	0.519
Nigeria	Mushin	2017	0.098	0.153	0.212
Nigeria	Mushin	2000-2017	-0.131	-0.038	0.057
Nigeria	Muya	2000	0.419	0.636	0.956
Nigeria	Muya	2017	0.145	0.240	0.373
Nigeria	Muya	2000-2017	-0.124	-0.032	0.056
Nigeria	Nafada	2000	0.627	0.955	1.325
Nigeria	Nafada	2017	0.263	0.449	0.719
Nigeria	Nafada	2000-2017	-0.118	-0.018	0.085
Nigeria	Nangere	2000	0.581	0.883	1.274
Nigeria	Nangere	2017	0.230	0.395	0.632
Nigeria	Nangere	2000-2017	-0.124	-0.020	0.072
Nigeria	Nasarawa	2000	0.375	0.590	0.923
Nigeria	Nasarawa	2017	0.143	0.228	0.344
Nigeria	Nasarawa	2000-2017	-0.111	-0.021	0.063
Nigeria	Nassaraw	2000	0.564	0.815	1.162
Nigeria	Nassaraw	2017	0.226	0.347	0.528
Nigeria	Nassaraw	2000-2017	-0.103	-0.016	0.077
Nigeria	Nassarawa Egon	2000	0.381	0.630	0.951
Nigeria	Nassarawa Egon	2017	0.137	0.218	0.339
Nigeria	Nassarawa Egon	2000-2017	-0.119	-0.030	0.055

Table 2: Diarrhea DALYs rate by unit *(continued)*

Country	Unit	year	mean rate	lower rate	upper rate
Nigeria	Ndokwa East	2000	0.296	0.458	0.723
Nigeria	Ndokwa East	2017	0.115	0.182	0.287
Nigeria	Ndokwa East	2000-2017	-0.114	-0.021	0.071
Nigeria	Ndokwa West	2000	0.236	0.369	0.568
Nigeria	Ndokwa West	2017	0.093	0.149	0.231
Nigeria	Ndokwa West	2000-2017	-0.122	-0.025	0.071
Nigeria	Nembe	2000	0.254	0.403	0.610
Nigeria	Nembe	2017	0.106	0.165	0.250
Nigeria	Nembe	2000-2017	-0.115	-0.020	0.078
Nigeria	Ngala	2000	0.714	0.939	1.230
Nigeria	Ngala	2017	0.260	0.438	0.717
Nigeria	Ngala	2000-2017	-0.137	-0.037	0.062
Nigeria	Nganzai	2000	0.607	0.888	1.250
Nigeria	Nganzai	2017	0.236	0.406	0.639
Nigeria	Nganzai	2000-2017	-0.129	-0.033	0.051
Nigeria	Ngaski	2000	0.558	0.892	1.336
Nigeria	Ngaski	2017	0.214	0.314	0.455
Nigeria	Ngaski	2000-2017	-0.130	-0.043	0.049
Nigeria	Ngor-Okp	2000	0.265	0.413	0.609
Nigeria	Ngor-Okp	2017	0.096	0.157	0.237
Nigeria	Ngor-Okp	2000-2017	-0.103	-0.017	0.069
Nigeria	Nguru	2000	0.544	0.775	1.054
Nigeria	Nguru	2017	0.230	0.360	0.590
Nigeria	Nguru	2000-2017	-0.096	-0.006	0.090
Nigeria	Ningi	2000	0.526	0.828	1.228
Nigeria	Ningi	2017	0.236	0.380	0.572
Nigeria	Ningi	2000-2017	-0.094	-0.011	0.074
Nigeria	Njaba	2000	0.256	0.396	0.597
Nigeria	Njaba	2017	0.088	0.146	0.233
Nigeria	Njaba	2000-2017	-0.103	-0.010	0.082
Nigeria	Njikoka	2000	0.262	0.407	0.640
Nigeria	Njikoka	2017	0.092	0.151	0.237
Nigeria	Njikoka	2000-2017	-0.110	-0.012	0.081
Nigeria	Nkanu East	2000	0.282	0.429	0.673
Nigeria	Nkanu East	2017	0.091	0.158	0.257
Nigeria	Nkanu East	2000-2017	-0.094	-0.011	0.079
Nigeria	Nkanu West	2000	0.257	0.388	0.609
Nigeria	Nkanu West	2017	0.083	0.143	0.230
Nigeria	Nkanu West	2000-2017	-0.101	-0.011	0.081
Nigeria	Nkwerre	2000	0.268	0.408	0.625
Nigeria	Nkwerre	2017	0.091	0.152	0.240
Nigeria	Nkwerre	2000-2017	-0.099	-0.008	0.085
Nigeria	NnewiNort	2000	0.243	0.386	0.578
Nigeria	NnewiNort	2017	0.088	0.144	0.230
Nigeria	NnewiNort	2000-2017	-0.112	-0.014	0.075
Nigeria	NnewiSou	2000	0.231	0.368	0.548
Nigeria	NnewiSou	2017	0.086	0.138	0.223
Nigeria	NnewiSou	2000-2017	-0.108	-0.012	0.080
Nigeria	Nsit Atai	2000	0.295	0.441	0.606
Nigeria	Nsit Atai	2017	0.100	0.157	0.236
Nigeria	Nsit Atai	2000-2017	-0.095	0.002	0.091
Nigeria	Nsit Ibom	2000	0.270	0.404	0.561
Nigeria	Nsit Ibom	2017	0.093	0.144	0.213
Nigeria	Nsit Ibom	2000-2017	-0.103	0.001	0.088
Nigeria	Nsit Ubium	2000	0.289	0.435	0.606
Nigeria	Nsit Ubium	2017	0.102	0.158	0.234
Nigeria	Nsit Ubium	2000-2017	-0.097	0.002	0.094
Nigeria	Nsukka	2000	0.253	0.398	0.639
Nigeria	Nsukka	2017	0.088	0.145	0.223
Nigeria	Nsukka	2000-2017	-0.101	-0.012	0.082
Nigeria	Numan	2000	0.601	0.914	1.280

Table 2: Diarrhea DALYs rate by unit *(continued)*

Country	Unit	year	mean rate	lower rate	upper rate
Nigeria	Numan	2017	0.210	0.352	0.550
Nigeria	Numan	2000-2017	-0.123	-0.028	0.058
Nigeria	Nwangele	2000	0.274	0.416	0.634
Nigeria	Nwangele	2017	0.092	0.154	0.242
Nigeria	Nwangele	2000-2017	-0.101	-0.008	0.085
Nigeria	Obafemi-Owode	2000	0.293	0.447	0.655
Nigeria	Obafemi-Owode	2017	0.106	0.168	0.235
Nigeria	Obafemi-Owode	2000-2017	-0.140	-0.047	0.042
Nigeria	Obanliku	2000	0.292	0.456	0.669
Nigeria	Obanliku	2017	0.097	0.158	0.255
Nigeria	Obanliku	2000-2017	-0.087	0.009	0.091
Nigeria	Obi	2000	0.329	0.535	0.831
Nigeria	Obi	2000	0.393	0.637	0.969
Nigeria	Obi	2017	0.119	0.187	0.296
Nigeria	Obi	2017	0.136	0.221	0.334
Nigeria	Obi	2000-2017	-0.110	-0.019	0.069
Nigeria	Obi	2000-2017	-0.081	0.006	0.088
Nigeria	Obio/Akp	2000	0.282	0.432	0.655
Nigeria	Obio/Akp	2017	0.104	0.167	0.257
Nigeria	Obio/Akp	2000-2017	-0.121	-0.028	0.065
Nigeria	Obokun	2000	0.266	0.460	0.701
Nigeria	Obokun	2017	0.098	0.160	0.230
Nigeria	Obokun	2000-2017	-0.150	-0.063	0.020
Nigeria	Oboma Ngwa	2000	0.272	0.422	0.599
Nigeria	Oboma Ngwa	2017	0.094	0.156	0.237
Nigeria	Oboma Ngwa	2000-2017	-0.104	-0.013	0.077
Nigeria	Obot Akara	2000	0.289	0.432	0.619
Nigeria	Obot Akara	2017	0.096	0.157	0.236
Nigeria	Obot Akara	2000-2017	-0.092	-0.004	0.082
Nigeria	Obowo	2000	0.306	0.468	0.683
Nigeria	Obowo	2017	0.101	0.173	0.260
Nigeria	Obowo	2000-2017	-0.099	-0.003	0.096
Nigeria	Obubra	2000	0.274	0.422	0.627
Nigeria	Obubra	2017	0.090	0.152	0.247
Nigeria	Obubra	2000-2017	-0.082	0.010	0.093
Nigeria	Obudu	2000	0.304	0.467	0.679
Nigeria	Obudu	2017	0.099	0.161	0.266
Nigeria	Obudu	2000-2017	-0.090	0.004	0.087
Nigeria	Odeda	2000	0.297	0.449	0.678
Nigeria	Odeda	2017	0.104	0.163	0.224
Nigeria	Odeda	2000-2017	-0.139	-0.046	0.043
Nigeria	Odigbo	2000	0.267	0.439	0.687
Nigeria	Odigbo	2017	0.097	0.159	0.233
Nigeria	Odigbo	2000-2017	-0.148	-0.064	0.022
Nigeria	Odo0tin	2000	0.292	0.483	0.756
Nigeria	Odo0tin	2017	0.099	0.162	0.231
Nigeria	Odo0tin	2000-2017	-0.148	-0.058	0.028
Nigeria	Odogbolu	2000	0.286	0.447	0.661
Nigeria	Odogbolu	2017	0.103	0.168	0.234
Nigeria	Odogbolu	2000-2017	-0.143	-0.049	0.036
Nigeria	Odukpani	2000	0.332	0.492	0.697
Nigeria	Odukpani	2017	0.113	0.183	0.281
Nigeria	Odukpani	2000-2017	-0.085	0.010	0.094
Nigeria	Offa	2000	0.395	0.655	1.023
Nigeria	Offa	2017	0.127	0.208	0.301
Nigeria	Offa	2000-2017	-0.162	-0.067	0.024
Nigeria	Ofu	2000	0.320	0.507	0.809
Nigeria	Ofu	2017	0.118	0.194	0.298
Nigeria	Ofu	2000-2017	-0.116	-0.020	0.069
Nigeria	Ogba/Egbe	2000	0.281	0.431	0.650
Nigeria	Ogba/Egbe	2017	0.104	0.163	0.259

Table 2: Diarrhea DALYs rate by unit *(continued)*

Country	Unit	year	mean rate	lower rate	upper rate
Nigeria	Ogba/Egbe	2000-2017	-0.103	-0.020	0.061
Nigeria	Ogbadibo	2000	0.300	0.484	0.768
Nigeria	Ogbadibo	2017	0.105	0.174	0.262
Nigeria	Ogbadibo	2000-2017	-0.099	-0.008	0.084
Nigeria	Ogbaru	2000	0.260	0.409	0.627
Nigeria	Ogbaru	2017	0.101	0.161	0.252
Nigeria	Ogbaru	2000-2017	-0.115	-0.019	0.069
Nigeria	Ogbia	2000	0.319	0.495	0.752
Nigeria	Ogbia	2017	0.124	0.196	0.300
Nigeria	Ogbia	2000-2017	-0.112	-0.022	0.061
Nigeria	Ogbomosho North	2000	0.330	0.515	0.831
Nigeria	Ogbomosho North	2017	0.106	0.172	0.246
Nigeria	Ogbomosho North	2000-2017	-0.149	-0.058	0.036
Nigeria	Ogbomosho South	2000	0.317	0.492	0.793
Nigeria	Ogbomosho South	2017	0.102	0.163	0.235
Nigeria	Ogbomosho South	2000-2017	-0.152	-0.061	0.031
Nigeria	Ogo-Oluw	2000	0.292	0.462	0.727
Nigeria	Ogo-Oluw	2017	0.103	0.163	0.236
Nigeria	Ogo-Oluw	2000-2017	-0.144	-0.055	0.035
Nigeria	Ogoja	2000	0.301	0.460	0.692
Nigeria	Ogoja	2017	0.100	0.162	0.263
Nigeria	Ogoja	2000-2017	-0.079	0.014	0.100
Nigeria	Ogori/Magongo	2000	0.299	0.489	0.752
Nigeria	Ogori/Magongo	2017	0.107	0.176	0.269
Nigeria	Ogori/Magongo	2000-2017	-0.136	-0.041	0.054
Nigeria	Ogu/Bolo	2000	0.287	0.423	0.624
Nigeria	Ogu/Bolo	2017	0.100	0.160	0.245
Nigeria	Ogu/Bolo	2000-2017	-0.100	-0.007	0.093
Nigeria	OgunWaterside	2000	0.297	0.460	0.702
Nigeria	OgunWaterside	2017	0.114	0.182	0.267
Nigeria	OgunWaterside	2000-2017	-0.134	-0.048	0.039
Nigeria	Oguta	2000	0.249	0.388	0.592
Nigeria	Oguta	2017	0.092	0.148	0.232
Nigeria	Oguta	2000-2017	-0.112	-0.020	0.071
Nigeria	Ohafia Abia	2000	0.288	0.430	0.639
Nigeria	Ohafia Abia	2017	0.094	0.158	0.245
Nigeria	Ohafia Abia	2000-2017	-0.089	0.005	0.094
Nigeria	Ohaji/Eg	2000	0.268	0.413	0.620
Nigeria	Ohaji/Eg	2017	0.099	0.158	0.244
Nigeria	Ohaji/Eg	2000-2017	-0.104	-0.019	0.063
Nigeria	Ohaozara	2000	0.338	0.509	0.782
Nigeria	Ohaozara	2017	0.108	0.187	0.304
Nigeria	Ohaozara	2000-2017	-0.094	0.000	0.087
Nigeria	Ohaukwu	2000	0.295	0.461	0.724
Nigeria	Ohaukwu	2017	0.100	0.166	0.267
Nigeria	Ohaukwu	2000-2017	-0.088	0.001	0.086
Nigeria	Ohimini	2000	0.322	0.521	0.832
Nigeria	Ohimini	2017	0.117	0.184	0.289
Nigeria	Ohimini	2000-2017	-0.093	-0.003	0.076
Nigeria	Oji-River	2000	0.268	0.394	0.604
Nigeria	Oji-River	2017	0.089	0.150	0.237
Nigeria	Oji-River	2000-2017	-0.111	-0.016	0.074
Nigeria	Ojo	2000	0.269	0.411	0.596
Nigeria	Ojo	2017	0.117	0.179	0.242
Nigeria	Ojo	2000-2017	-0.123	-0.037	0.058
Nigeria	Oju	2000	0.333	0.543	0.847
Nigeria	Oju	2017	0.120	0.197	0.323
Nigeria	Oju	2000-2017	-0.078	0.012	0.091
Nigeria	Oke-Ero	2000	0.335	0.540	0.833
Nigeria	Oke-Ero	2017	0.107	0.173	0.248
Nigeria	Oke-Ero	2000-2017	-0.147	-0.061	0.031

Table 2: Diarrhea DALYs rate by unit *(continued)*

Country	Unit	year	mean rate	lower rate	upper rate
Nigeria	Okehi	2000	0.333	0.534	0.819
Nigeria	Okehi	2017	0.117	0.193	0.301
Nigeria	Okehi	2000-2017	-0.133	-0.041	0.048
Nigeria	Okene	2000	0.320	0.510	0.775
Nigeria	Okene	2017	0.111	0.185	0.278
Nigeria	Okene	2000-2017	-0.132	-0.040	0.054
Nigeria	Okigwe	2000	0.284	0.428	0.670
Nigeria	Okigwe	2017	0.096	0.164	0.261
Nigeria	Okigwe	2000-2017	-0.100	-0.006	0.089
Nigeria	Okitipupa	2000	0.253	0.420	0.643
Nigeria	Okitipupa	2017	0.099	0.158	0.235
Nigeria	Okitipupa	2000-2017	-0.142	-0.058	0.033
Nigeria	Okobo	2000	0.301	0.458	0.633
Nigeria	Okobo	2017	0.104	0.166	0.251
Nigeria	Okobo	2000-2017	-0.090	0.006	0.095
Nigeria	Okpe	2000	0.287	0.447	0.680
Nigeria	Okpe	2017	0.111	0.175	0.260
Nigeria	Okpe	2000-2017	-0.124	-0.036	0.057
Nigeria	Okpokwu	2000	0.306	0.498	0.798
Nigeria	Okpokwu	2017	0.111	0.179	0.275
Nigeria	Okpokwu	2000-2017	-0.094	-0.004	0.084
Nigeria	Okrika	2000	0.283	0.420	0.627
Nigeria	Okrika	2017	0.100	0.160	0.248
Nigeria	Okrika	2000-2017	-0.101	-0.011	0.088
Nigeria	Ola-Oluwa	2000	0.253	0.406	0.633
Nigeria	Ola-Oluwa	2017	0.091	0.146	0.208
Nigeria	Ola-Oluwa	2000-2017	-0.138	-0.051	0.040
Nigeria	Olamabor	2000	0.322	0.522	0.825
Nigeria	Olamabor	2017	0.118	0.190	0.296
Nigeria	Olamabor	2000-2017	-0.105	-0.011	0.078
Nigeria	Olorunda	2000	0.262	0.441	0.685
Nigeria	Olorunda	2017	0.095	0.150	0.216
Nigeria	Olorunda	2000-2017	-0.147	-0.055	0.034
Nigeria	Olorunsogo	2000	0.374	0.590	0.923
Nigeria	Olorunsogo	2017	0.107	0.177	0.260
Nigeria	Olorunsogo	2000-2017	-0.149	-0.064	0.034
Nigeria	Oluyole	2000	0.272	0.422	0.639
Nigeria	Oluyole	2017	0.095	0.152	0.215
Nigeria	Oluyole	2000-2017	-0.155	-0.060	0.026
Nigeria	Omala	2000	0.332	0.508	0.801
Nigeria	Omala	2017	0.127	0.199	0.321
Nigeria	Omala	2000-2017	-0.103	-0.018	0.066
Nigeria	Omumma	2000	0.279	0.427	0.620
Nigeria	Omumma	2017	0.098	0.158	0.246
Nigeria	Omumma	2000-2017	-0.115	-0.022	0.064
Nigeria	Ona-Ara	2000	0.288	0.452	0.699
Nigeria	Ona-Ara	2017	0.101	0.159	0.230
Nigeria	Ona-Ara	2000-2017	-0.155	-0.065	0.020
Nigeria	Ondo East	2000	0.289	0.477	0.748
Nigeria	Ondo East	2017	0.099	0.165	0.247
Nigeria	Ondo East	2000-2017	-0.153	-0.068	0.021
Nigeria	Ondo West	2000	0.275	0.456	0.701
Nigeria	Ondo West	2017	0.096	0.158	0.237
Nigeria	Ondo West	2000-2017	-0.154	-0.068	0.020
Nigeria	Onicha	2000	0.350	0.527	0.823
Nigeria	Onicha	2017	0.112	0.191	0.306
Nigeria	Onicha	2000-2017	-0.102	-0.005	0.082
Nigeria	Onitsha North	2000	0.253	0.406	0.622
Nigeria	Onitsha North	2017	0.096	0.155	0.245
Nigeria	Onitsha North	2000-2017	-0.116	-0.014	0.077
Nigeria	Onitsha South	2000	0.247	0.397	0.602

Table 2: Diarrhea DALYs rate by unit *(continued)*

Country	Unit	year	mean rate	lower rate	upper rate
Nigeria	Onitsha South	2017	0.097	0.156	0.248
Nigeria	Onitsha South	2000-2017	-0.117	-0.016	0.073
Nigeria	Onna	2000	0.304	0.457	0.634
Nigeria	Onna	2017	0.108	0.169	0.247
Nigeria	Onna	2000-2017	-0.099	0.001	0.096
Nigeria	Opobo/Nkoro	2000	0.297	0.439	0.625
Nigeria	Opobo/Nkoro	2017	0.103	0.160	0.239
Nigeria	Opobo/Nkoro	2000-2017	-0.103	-0.003	0.097
Nigeria	Oredo Edo	2000	0.263	0.413	0.639
Nigeria	Oredo Edo	2017	0.099	0.152	0.224
Nigeria	Oredo Edo	2000-2017	-0.153	-0.057	0.030
Nigeria	Orelope	2000	0.385	0.598	0.902
Nigeria	Orelope	2017	0.113	0.178	0.252
Nigeria	Orelope	2000-2017	-0.148	-0.061	0.026
Nigeria	Orhionmw	2000	0.256	0.386	0.589
Nigeria	Orhionmw	2017	0.098	0.151	0.227
Nigeria	Orhionmw	2000-2017	-0.133	-0.037	0.057
Nigeria	Ori-Ire	2000	0.346	0.527	0.837
Nigeria	Ori-Ire	2017	0.110	0.178	0.250
Nigeria	Ori-Ire	2000-2017	-0.143	-0.056	0.034
Nigeria	Oriade	2000	0.255	0.436	0.674
Nigeria	Oriade	2017	0.090	0.150	0.216
Nigeria	Oriade	2000-2017	-0.158	-0.066	0.030
Nigeria	Orlu	2000	0.257	0.398	0.606
Nigeria	Orlu	2017	0.089	0.148	0.230
Nigeria	Orlu	2000-2017	-0.102	-0.010	0.084
Nigeria	Orolu	2000	0.263	0.434	0.682
Nigeria	Orolu	2017	0.091	0.147	0.213
Nigeria	Orolu	2000-2017	-0.146	-0.057	0.034
Nigeria	Oron	2000	0.314	0.473	0.650
Nigeria	Oron	2017	0.107	0.175	0.269
Nigeria	Oron	2000-2017	-0.086	0.011	0.099
Nigeria	Orsu	2000	0.245	0.385	0.587
Nigeria	Orsu	2017	0.089	0.145	0.228
Nigeria	Orsu	2000-2017	-0.103	-0.009	0.087
Nigeria	Oru East	2000	0.252	0.396	0.597
Nigeria	Oru East	2017	0.092	0.150	0.236
Nigeria	Oru East	2000-2017	-0.104	-0.010	0.083
Nigeria	Oru West	2000	0.246	0.389	0.591
Nigeria	Oru West	2017	0.091	0.149	0.237
Nigeria	Oru West	2000-2017	-0.105	-0.011	0.082
Nigeria	Oruk-Ana	2000	0.277	0.419	0.592
Nigeria	Oruk-Ana	2017	0.098	0.152	0.223
Nigeria	Oruk-Ana	2000-2017	-0.110	-0.002	0.088
Nigeria	OrumbaNo	2000	0.245	0.375	0.569
Nigeria	OrumbaNo	2017	0.085	0.142	0.229
Nigeria	OrumbaNo	2000-2017	-0.112	-0.014	0.080
Nigeria	OrumbaSo	2000	0.262	0.394	0.613
Nigeria	OrumbaSo	2017	0.087	0.148	0.238
Nigeria	OrumbaSo	2000-2017	-0.108	-0.014	0.080
Nigeria	Ose	2000	0.289	0.447	0.700
Nigeria	Ose	2017	0.099	0.158	0.227
Nigeria	Ose	2000-2017	-0.150	-0.059	0.028
Nigeria	Oshimili North	2000	0.251	0.396	0.625
Nigeria	Oshimili North	2017	0.098	0.157	0.246
Nigeria	Oshimili North	2000-2017	-0.126	-0.022	0.070
Nigeria	Oshimili South	2000	0.251	0.401	0.618
Nigeria	Oshimili South	2017	0.100	0.161	0.256
Nigeria	Oshimili South	2000-2017	-0.122	-0.018	0.070
Nigeria	Oshodi/Isolo	2000	0.243	0.373	0.536
Nigeria	Oshodi/Isolo	2017	0.100	0.156	0.215

Table 2: Diarrhea DALYs rate by unit *(continued)*

Country	Unit	year	mean rate	lower rate	upper rate
Nigeria	Oshodi/Isolo	2000-2017	-0.131	-0.040	0.055
Nigeria	Osisioma Ngwa	2000	0.273	0.430	0.618
Nigeria	Osisioma Ngwa	2017	0.096	0.160	0.246
Nigeria	Osisioma Ngwa	2000-2017	-0.107	-0.015	0.072
Nigeria	Osogbo	2000	0.256	0.433	0.671
Nigeria	Osogbo	2017	0.093	0.148	0.213
Nigeria	Osogbo	2000-2017	-0.146	-0.055	0.033
Nigeria	Oturkpo	2000	0.333	0.544	0.865
Nigeria	Oturkpo	2017	0.125	0.195	0.304
Nigeria	Oturkpo	2000-2017	-0.089	-0.002	0.076
Nigeria	OviaNort	2000	0.280	0.434	0.666
Nigeria	OviaNort	2017	0.106	0.162	0.238
Nigeria	OviaNort	2000-2017	-0.144	-0.053	0.038
Nigeria	OviaSouth-West	2000	0.280	0.443	0.687
Nigeria	OviaSouth-West	2017	0.106	0.166	0.243
Nigeria	OviaSouth-West	2000-2017	-0.133	-0.049	0.042
Nigeria	Owan East	2000	0.263	0.416	0.653
Nigeria	Owan East	2017	0.093	0.150	0.227
Nigeria	Owan East	2000-2017	-0.143	-0.050	0.036
Nigeria	OwanWest	2000	0.284	0.438	0.703
Nigeria	OwanWest	2017	0.099	0.157	0.230
Nigeria	OwanWest	2000-2017	-0.146	-0.055	0.036
Nigeria	Owerri Municipal	2000	0.262	0.414	0.620
Nigeria	Owerri Municipal	2017	0.094	0.155	0.232
Nigeria	Owerri Municipal	2000-2017	-0.102	-0.011	0.080
Nigeria	Owerri North	2000	0.274	0.426	0.640
Nigeria	Owerri North	2017	0.096	0.160	0.240
Nigeria	Owerri North	2000-2017	-0.102	-0.009	0.082
Nigeria	Owerri West	2000	0.252	0.396	0.587
Nigeria	Owerri West	2017	0.091	0.149	0.225
Nigeria	Owerri West	2000-2017	-0.100	-0.011	0.078
Nigeria	Owo	2000	0.277	0.441	0.689
Nigeria	Owo	2017	0.098	0.156	0.234
Nigeria	Owo	2000-2017	-0.149	-0.061	0.027
Nigeria	Oye	2000	0.269	0.441	0.675
Nigeria	Oye	2017	0.096	0.156	0.227
Nigeria	Oye	2000-2017	-0.141	-0.051	0.041
Nigeria	Oyi	2000	0.263	0.413	0.646
Nigeria	Oyi	2017	0.095	0.155	0.244
Nigeria	Oyi	2000-2017	-0.113	-0.013	0.081
Nigeria	Oyigbo	2000	0.271	0.412	0.599
Nigeria	Oyigbo	2017	0.098	0.155	0.235
Nigeria	Oyigbo	2000-2017	-0.110	-0.019	0.072
Nigeria	Oyo East	2000	0.291	0.454	0.699
Nigeria	Oyo East	2017	0.100	0.157	0.226
Nigeria	Oyo East	2000-2017	-0.140	-0.050	0.040
Nigeria	Oyo West	2000	0.293	0.460	0.687
Nigeria	Oyo West	2017	0.101	0.158	0.224
Nigeria	Oyo West	2000-2017	-0.139	-0.052	0.038
Nigeria	Oyun	2000	0.384	0.628	0.992
Nigeria	Oyun	2017	0.123	0.200	0.290
Nigeria	Oyun	2000-2017	-0.158	-0.066	0.025
Nigeria	Paikoro	2000	0.433	0.655	0.975
Nigeria	Paikoro	2017	0.146	0.241	0.363
Nigeria	Paikoro	2000-2017	-0.123	-0.036	0.052
Nigeria	Pankshin	2000	0.383	0.643	0.950
Nigeria	Pankshin	2017	0.135	0.222	0.330
Nigeria	Pankshin	2000-2017	-0.121	-0.035	0.054
Nigeria	Patani	2000	0.309	0.495	0.757
Nigeria	Patani	2017	0.124	0.196	0.305
Nigeria	Patani	2000-2017	-0.121	-0.027	0.069

Table 2: Diarrhea DALYs rate by unit *(continued)*

Country	Unit	year	mean rate	lower rate	upper rate
Nigeria	Pategi	2000	0.402	0.628	0.985
Nigeria	Pategi	2017	0.136	0.222	0.329
Nigeria	Pategi	2000-2017	-0.147	-0.050	0.039
Nigeria	Port Harcourt	2000	0.294	0.442	0.673
Nigeria	Port Harcourt	2017	0.104	0.169	0.257
Nigeria	Port Harcourt	2000-2017	-0.113	-0.022	0.076
Nigeria	Potiskum	2000	0.580	0.893	1.270
Nigeria	Potiskum	2017	0.232	0.397	0.639
Nigeria	Potiskum	2000-2017	-0.123	-0.020	0.069
Nigeria	Qua'anpa	2000	0.367	0.621	0.906
Nigeria	Qua'anpa	2017	0.130	0.212	0.322
Nigeria	Qua'anpa	2000-2017	-0.109	-0.026	0.060
Nigeria	Rabah	2000	0.639	0.889	1.278
Nigeria	Rabah	2017	0.264	0.396	0.581
Nigeria	Rabah	2000-2017	-0.109	-0.028	0.086
Nigeria	Rafi	2000	0.487	0.718	1.133
Nigeria	Rafi	2017	0.178	0.277	0.436
Nigeria	Rafi	2000-2017	-0.116	-0.032	0.061
Nigeria	Rano	2000	0.500	0.773	1.121
Nigeria	Rano	2017	0.250	0.388	0.585
Nigeria	Rano	2000-2017	-0.095	-0.005	0.087
Nigeria	Remo-North	2000	0.289	0.451	0.657
Nigeria	Remo-North	2017	0.103	0.167	0.234
Nigeria	Remo-North	2000-2017	-0.147	-0.051	0.033
Nigeria	Rijau	2000	0.601	0.939	1.442
Nigeria	Rijau	2017	0.231	0.341	0.509
Nigeria	Rijau	2000-2017	-0.127	-0.038	0.054
Nigeria	Rimi	2000	0.583	0.830	1.140
Nigeria	Rimi	2017	0.212	0.336	0.511
Nigeria	Rimi	2000-2017	-0.116	-0.029	0.060
Nigeria	RiminGad	2000	0.564	0.840	1.217
Nigeria	RiminGad	2017	0.241	0.374	0.570
Nigeria	RiminGad	2000-2017	-0.111	-0.020	0.063
Nigeria	Ringim	2000	0.571	0.856	1.222
Nigeria	Ringim	2017	0.253	0.390	0.605
Nigeria	Ringim	2000-2017	-0.094	-0.013	0.076
Nigeria	Riyom	2000	0.362	0.600	0.898
Nigeria	Riyom	2017	0.137	0.225	0.353
Nigeria	Riyom	2000-2017	-0.119	-0.035	0.055
Nigeria	Rogo	2000	0.532	0.803	1.160
Nigeria	Rogo	2017	0.226	0.349	0.530
Nigeria	Rogo	2000-2017	-0.114	-0.022	0.058
Nigeria	Roni	2000	0.624	0.897	1.249
Nigeria	Roni	2017	0.231	0.366	0.563
Nigeria	Roni	2000-2017	-0.110	-0.023	0.061
Nigeria	Sabon Birni	2000	0.648	0.886	1.183
Nigeria	Sabon Birni	2017	0.249	0.378	0.582
Nigeria	Sabon Birni	2000-2017	-0.113	-0.027	0.074
Nigeria	Sabon-Ga	2000	0.461	0.704	1.053
Nigeria	Sabon-Ga	2017	0.184	0.284	0.435
Nigeria	Sabon-Ga	2000-2017	-0.114	-0.024	0.059
Nigeria	Sabuwa	2000	0.516	0.773	1.155
Nigeria	Sabuwa	2017	0.202	0.318	0.480
Nigeria	Sabuwa	2000-2017	-0.117	-0.029	0.064
Nigeria	Safana	2000	0.619	0.886	1.242
Nigeria	Safana	2017	0.227	0.356	0.547
Nigeria	Safana	2000-2017	-0.128	-0.032	0.057
Nigeria	Sagbama	2000	0.316	0.499	0.766
Nigeria	Sagbama	2017	0.127	0.198	0.308
Nigeria	Sagbama	2000-2017	-0.121	-0.027	0.062
Nigeria	Sakaba	2000	0.613	0.941	1.373

Table 2: Diarrhea DALYs rate by unit *(continued)*

Country	Unit	year	mean rate	lower rate	upper rate
Nigeria	Sakaba	2017	0.228	0.335	0.506
Nigeria	Sakaba	2000-2017	-0.134	-0.040	0.060
Nigeria	Saki East	2000	0.347	0.523	0.768
Nigeria	Saki East	2017	0.102	0.157	0.218
Nigeria	Saki East	2000-2017	-0.147	-0.057	0.034
Nigeria	Saki West	2000	0.362	0.562	0.804
Nigeria	Saki West	2017	0.113	0.166	0.229
Nigeria	Saki West	2000-2017	-0.148	-0.055	0.028
Nigeria	Sandamu	2000	0.562	0.812	1.086
Nigeria	Sandamu	2017	0.212	0.330	0.514
Nigeria	Sandamu	2000-2017	-0.109	-0.024	0.062
Nigeria	Sanga	2000	0.400	0.677	1.036
Nigeria	Sanga	2017	0.149	0.245	0.379
Nigeria	Sanga	2000-2017	-0.123	-0.030	0.062
Nigeria	Sapele	2000	0.299	0.467	0.721
Nigeria	Sapele	2017	0.120	0.185	0.270
Nigeria	Sapele	2000-2017	-0.123	-0.037	0.055
Nigeria	Sardauna	2000	0.397	0.617	0.893
Nigeria	Sardauna	2017	0.160	0.269	0.451
Nigeria	Sardauna	2000-2017	-0.065	0.023	0.117
Nigeria	Shagamu	2000	0.291	0.455	0.676
Nigeria	Shagamu	2017	0.106	0.172	0.239
Nigeria	Shagamu	2000-2017	-0.143	-0.049	0.037
Nigeria	Shagari	2000	0.596	0.892	1.288
Nigeria	Shagari	2017	0.258	0.378	0.544
Nigeria	Shagari	2000-2017	-0.124	-0.035	0.069
Nigeria	Shanga	2000	0.653	0.968	1.358
Nigeria	Shanga	2017	0.240	0.351	0.506
Nigeria	Shanga	2000-2017	-0.128	-0.037	0.051
Nigeria	Shani	2000	0.581	0.898	1.228
Nigeria	Shani	2017	0.222	0.370	0.572
Nigeria	Shani	2000-2017	-0.117	-0.027	0.069
Nigeria	Shanono	2000	0.582	0.833	1.217
Nigeria	Shanono	2017	0.226	0.352	0.524
Nigeria	Shanono	2000-2017	-0.114	-0.024	0.062
Nigeria	Shelleng	2000	0.608	0.908	1.238
Nigeria	Shelleng	2017	0.218	0.365	0.570
Nigeria	Shelleng	2000-2017	-0.122	-0.036	0.053
Nigeria	Shendam	2000	0.382	0.652	0.958
Nigeria	Shendam	2017	0.139	0.221	0.329
Nigeria	Shendam	2000-2017	-0.104	-0.023	0.064
Nigeria	Shinkafi	2000	0.686	0.944	1.285
Nigeria	Shinkafi	2017	0.242	0.382	0.587
Nigeria	Shinkafi	2000-2017	-0.122	-0.036	0.063
Nigeria	Shira	2000	0.491	0.777	1.190
Nigeria	Shira	2017	0.221	0.388	0.598
Nigeria	Shira	2000-2017	-0.089	-0.004	0.095
Nigeria	Shiroro	2000	0.454	0.675	1.009
Nigeria	Shiroro	2017	0.165	0.262	0.415
Nigeria	Shiroro	2000-2017	-0.123	-0.032	0.057
Nigeria	Shomgom	2000	0.550	0.893	1.266
Nigeria	Shomgom	2017	0.214	0.356	0.576
Nigeria	Shomgom	2000-2017	-0.115	-0.017	0.074
Nigeria	Shomolu	2000	0.241	0.371	0.529
Nigeria	Shomolu	2017	0.098	0.154	0.213
Nigeria	Shomolu	2000-2017	-0.133	-0.040	0.055
Nigeria	Silame	2000	0.590	0.888	1.266
Nigeria	Silame	2017	0.266	0.389	0.554
Nigeria	Silame	2000-2017	-0.112	-0.025	0.074
Nigeria	Soba	2000	0.494	0.755	1.114
Nigeria	Soba	2017	0.218	0.330	0.500

Table 2: Diarrhea DALYs rate by unit *(continued)*

Country	Unit	year	mean rate	lower rate	upper rate
Nigeria	Soba	2000-2017	-0.104	-0.015	0.077
Nigeria	Sokoto North	2000	0.641	0.949	1.364
Nigeria	Sokoto North	2017	0.266	0.396	0.557
Nigeria	Sokoto North	2000-2017	-0.107	-0.019	0.072
Nigeria	Sokoto South	2000	0.632	0.942	1.364
Nigeria	Sokoto South	2017	0.270	0.398	0.561
Nigeria	Sokoto South	2000-2017	-0.105	-0.016	0.075
Nigeria	Song	2000	0.655	0.915	1.227
Nigeria	Song	2017	0.216	0.366	0.578
Nigeria	Song	2000-2017	-0.137	-0.042	0.053
Nigeria	Southern Ijaw	2000	0.305	0.470	0.720
Nigeria	Southern Ijaw	2017	0.133	0.208	0.309
Nigeria	Southern Ijaw	2000-2017	-0.106	-0.021	0.068
Nigeria	Sule-Tan	2000	0.610	0.878	1.180
Nigeria	Sule-Tan	2017	0.245	0.382	0.600
Nigeria	Sule-Tan	2000-2017	-0.105	-0.016	0.077
Nigeria	Suleja	2000	0.412	0.634	0.953
Nigeria	Suleja	2017	0.138	0.223	0.344
Nigeria	Suleja	2000-2017	-0.131	-0.044	0.041
Nigeria	Sumaila	2000	0.502	0.768	1.127
Nigeria	Sumaila	2017	0.234	0.370	0.565
Nigeria	Sumaila	2000-2017	-0.100	-0.013	0.079
Nigeria	Suru	2000	0.641	0.895	1.281
Nigeria	Suru	2017	0.253	0.346	0.488
Nigeria	Suru	2000-2017	-0.108	-0.031	0.061
Nigeria	Surulere	2000	0.299	0.479	0.767
Nigeria	Surulere	2000	0.246	0.376	0.545
Nigeria	Surulere	2017	0.103	0.166	0.240
Nigeria	Surulere	2017	0.106	0.168	0.232
Nigeria	Surulere	2000-2017	-0.128	-0.036	0.058
Nigeria	Surulere	2000-2017	-0.152	-0.062	0.025
Nigeria	Tafa	2000	0.405	0.621	0.933
Nigeria	Tafa	2017	0.135	0.217	0.329
Nigeria	Tafa	2000-2017	-0.133	-0.045	0.043
Nigeria	Tafawa-B	2000	0.468	0.742	1.119
Nigeria	Tafawa-B	2017	0.172	0.286	0.448
Nigeria	Tafawa-B	2000-2017	-0.115	-0.023	0.068
Nigeria	Tai	2000	0.277	0.412	0.602
Nigeria	Tai	2017	0.097	0.155	0.238
Nigeria	Tai	2000-2017	-0.106	-0.011	0.088
Nigeria	Takai	2000	0.509	0.784	1.133
Nigeria	Takai	2017	0.231	0.375	0.576
Nigeria	Takai	2000-2017	-0.096	-0.011	0.081
Nigeria	Takum	2000	0.379	0.615	0.912
Nigeria	Takum	2017	0.135	0.228	0.363
Nigeria	Takum	2000-2017	-0.078	0.015	0.099
Nigeria	Talata-Mafara	2000	0.654	0.937	1.310
Nigeria	Talata-Mafara	2017	0.263	0.402	0.598
Nigeria	Talata-Mafara	2000-2017	-0.123	-0.033	0.079
Nigeria	Tambawal	2000	0.621	0.919	1.322
Nigeria	Tambawal	2017	0.262	0.377	0.529
Nigeria	Tambawal	2000-2017	-0.118	-0.033	0.066
Nigeria	Tangazar	2000	0.596	0.850	1.208
Nigeria	Tangazar	2017	0.250	0.379	0.555
Nigeria	Tangazar	2000-2017	-0.105	-0.019	0.087
Nigeria	Tarauni	2000	0.577	0.834	1.197
Nigeria	Tarauni	2017	0.232	0.356	0.538
Nigeria	Tarauni	2000-2017	-0.104	-0.016	0.078
Nigeria	Tarka	2000	0.313	0.536	0.842
Nigeria	Tarka	2017	0.119	0.191	0.290
Nigeria	Tarka	2000-2017	-0.104	-0.002	0.085

Table 2: Diarrhea DALYs rate by unit *(continued)*

Country	Unit	year	mean rate	lower rate	upper rate
Nigeria	Tarmuwa	2000	0.642	0.961	1.394
Nigeria	Tarmuwa	2017	0.253	0.439	0.729
Nigeria	Tarmuwa	2000-2017	-0.119	-0.024	0.069
Nigeria	Taura	2000	0.588	0.872	1.241
Nigeria	Taura	2017	0.248	0.394	0.610
Nigeria	Taura	2000-2017	-0.091	-0.011	0.084
Nigeria	Teungo	2000	0.460	0.696	0.969
Nigeria	Teungo	2017	0.158	0.275	0.420
Nigeria	Teungo	2000-2017	-0.110	-0.005	0.088
Nigeria	Tofa	2000	0.564	0.831	1.204
Nigeria	Tofa	2017	0.236	0.363	0.549
Nigeria	Tofa	2000-2017	-0.108	-0.019	0.067
Nigeria	Toro	2000	0.479	0.755	1.101
Nigeria	Toro	2017	0.188	0.319	0.484
Nigeria	Toro	2000-2017	-0.099	-0.020	0.070
Nigeria	Toto	2000	0.370	0.572	0.894
Nigeria	Toto	2017	0.132	0.221	0.349
Nigeria	Toto	2000-2017	-0.128	-0.029	0.051
Nigeria	Tsafe	2000	0.575	0.844	1.257
Nigeria	Tsafe	2017	0.232	0.370	0.565
Nigeria	Tsafe	2000-2017	-0.130	-0.033	0.054
Nigeria	Tsanyawa	2000	0.589	0.851	1.219
Nigeria	Tsanyawa	2017	0.222	0.346	0.524
Nigeria	Tsanyawa	2000-2017	-0.112	-0.025	0.061
Nigeria	Tundun Wada	2000	0.510	0.793	1.163
Nigeria	Tundun Wada	2017	0.246	0.380	0.567
Nigeria	Tundun Wada	2000-2017	-0.104	-0.013	0.075
Nigeria	Tureta	2000	0.596	0.884	1.274
Nigeria	Tureta	2017	0.255	0.386	0.555
Nigeria	Tureta	2000-2017	-0.118	-0.031	0.080
Nigeria	Udenu	2000	0.253	0.403	0.641
Nigeria	Udenu	2017	0.087	0.144	0.224
Nigeria	Udenu	2000-2017	-0.101	-0.012	0.082
Nigeria	Udi	2000	0.256	0.387	0.599
Nigeria	Udi	2017	0.086	0.144	0.227
Nigeria	Udi	2000-2017	-0.105	-0.014	0.076
Nigeria	Udu	2000	0.278	0.433	0.658
Nigeria	Udu	2017	0.104	0.169	0.253
Nigeria	Udu	2000-2017	-0.131	-0.034	0.055
Nigeria	Udung Uko	2000	0.311	0.468	0.643
Nigeria	Udung Uko	2017	0.105	0.170	0.259
Nigeria	Udung Uko	2000-2017	-0.086	0.011	0.099
Nigeria	Ughelli North	2000	0.257	0.402	0.611
Nigeria	Ughelli North	2017	0.099	0.160	0.243
Nigeria	Ughelli North	2000-2017	-0.122	-0.029	0.058
Nigeria	Ughelli South	2000	0.278	0.444	0.694
Nigeria	Ughelli South	2017	0.111	0.179	0.267
Nigeria	Ughelli South	2000-2017	-0.130	-0.032	0.054
Nigeria	Ugwunagbo	2000	0.262	0.413	0.592
Nigeria	Ugwunagbo	2017	0.095	0.155	0.234
Nigeria	Ugwunagbo	2000-2017	-0.109	-0.015	0.073
Nigeria	Uhunmwonde	2000	0.276	0.425	0.657
Nigeria	Uhunmwonde	2017	0.104	0.160	0.241
Nigeria	Uhunmwonde	2000-2017	-0.149	-0.052	0.039
Nigeria	Ukanafun	2000	0.287	0.439	0.619
Nigeria	Ukanafun	2017	0.099	0.157	0.233
Nigeria	Ukanafun	2000-2017	-0.112	-0.005	0.084
Nigeria	Ukum	2000	0.377	0.623	0.966
Nigeria	Ukum	2017	0.136	0.226	0.366
Nigeria	Ukum	2000-2017	-0.087	0.013	0.103
Nigeria	Ukwa East	2000	0.289	0.441	0.626

Table 2: Diarrhea DALYs rate by unit *(continued)*

Country	Unit	year	mean rate	lower rate	upper rate
Nigeria	Ukwa East	2017	0.101	0.162	0.245
Nigeria	Ukwa East	2000-2017	-0.111	-0.014	0.075
Nigeria	Ukwa West	2000	0.269	0.420	0.607
Nigeria	Ukwa West	2017	0.098	0.158	0.241
Nigeria	Ukwa West	2000-2017	-0.115	-0.022	0.064
Nigeria	Ukwuani	2000	0.233	0.363	0.555
Nigeria	Ukwuani	2017	0.090	0.143	0.220
Nigeria	Ukwuani	2000-2017	-0.134	-0.035	0.061
Nigeria	Umu-Nneochi	2000	0.273	0.416	0.646
Nigeria	Umu-Nneochi	2017	0.091	0.156	0.252
Nigeria	Umu-Nneochi	2000-2017	-0.107	-0.010	0.080
Nigeria	Umuahia North	2000	0.297	0.449	0.659
Nigeria	Umuahia North	2017	0.099	0.168	0.254
Nigeria	Umuahia North	2000-2017	-0.095	0.000	0.099
Nigeria	Umuahia South	2000	0.290	0.436	0.633
Nigeria	Umuahia South	2017	0.095	0.161	0.243
Nigeria	Umuahia South	2000-2017	-0.102	-0.008	0.091
Nigeria	Ungogo	2000	0.571	0.821	1.169
Nigeria	Ungogo	2017	0.231	0.355	0.543
Nigeria	Ungogo	2000-2017	-0.101	-0.013	0.077
Nigeria	Unuimo	2000	0.293	0.444	0.691
Nigeria	Unuimo	2017	0.098	0.167	0.265
Nigeria	Unuimo	2000-2017	-0.100	-0.007	0.087
Nigeria	Uruan	2000	0.292	0.439	0.617
Nigeria	Uruan	2017	0.099	0.157	0.233
Nigeria	Uruan	2000-2017	-0.089	0.007	0.092
Nigeria	UrueOffo	2000	0.297	0.450	0.622
Nigeria	UrueOffo	2017	0.103	0.163	0.250
Nigeria	UrueOffo	2000-2017	-0.091	0.005	0.097
Nigeria	Ushongo	2000	0.351	0.566	0.838
Nigeria	Ushongo	2017	0.122	0.196	0.315
Nigeria	Ushongo	2000-2017	-0.092	0.012	0.097
Nigeria	Ussa	2000	0.350	0.554	0.829
Nigeria	Ussa	2017	0.124	0.205	0.324
Nigeria	Ussa	2000-2017	-0.080	0.018	0.101
Nigeria	Uvwie	2000	0.300	0.461	0.708
Nigeria	Uvwie	2017	0.109	0.175	0.262
Nigeria	Uvwie	2000-2017	-0.128	-0.037	0.055
Nigeria	Uyo	2000	0.278	0.419	0.595
Nigeria	Uyo	2017	0.096	0.154	0.229
Nigeria	Uyo	2000-2017	-0.093	0.005	0.091
Nigeria	Uzo-Uwani	2000	0.258	0.408	0.658
Nigeria	Uzo-Uwani	2017	0.096	0.157	0.246
Nigeria	Uzo-Uwani	2000-2017	-0.104	-0.011	0.086
Nigeria	Vandeiky	2000	0.368	0.573	0.850
Nigeria	Vandeiky	2017	0.121	0.198	0.318
Nigeria	Vandeiky	2000-2017	-0.081	0.016	0.101
Nigeria	Wamakko	2000	0.623	0.925	1.333
Nigeria	Wamakko	2017	0.262	0.387	0.546
Nigeria	Wamakko	2000-2017	-0.112	-0.021	0.071
Nigeria	Wamba	2000	0.371	0.644	0.976
Nigeria	Wamba	2017	0.148	0.239	0.364
Nigeria	Wamba	2000-2017	-0.117	-0.025	0.058
Nigeria	Warawa	2000	0.558	0.805	1.157
Nigeria	Warawa	2017	0.238	0.364	0.545
Nigeria	Warawa	2000-2017	-0.099	-0.013	0.081
Nigeria	Warji	2000	0.495	0.798	1.218
Nigeria	Warji	2017	0.219	0.376	0.585
Nigeria	Warji	2000-2017	-0.100	-0.012	0.082
Nigeria	Warri North	2000	0.315	0.486	0.741
Nigeria	Warri North	2017	0.126	0.196	0.283

Table 2: Diarrhea DALYs rate by unit *(continued)*

Country	Unit	year	mean rate	lower rate	upper rate
Nigeria	Warri North	2000-2017	-0.127	-0.041	0.054
Nigeria	Warri South	2000	0.292	0.443	0.684
Nigeria	Warri South	2017	0.112	0.175	0.264
Nigeria	Warri South	2000-2017	-0.124	-0.037	0.057
Nigeria	Warri South-West	2000	0.283	0.443	0.683
Nigeria	Warri South-West	2017	0.122	0.185	0.271
Nigeria	Warri South-West	2000-2017	-0.123	-0.035	0.059
Nigeria	Wase	2000	0.467	0.738	1.052
Nigeria	Wase	2017	0.170	0.276	0.419
Nigeria	Wase	2000-2017	-0.106	-0.015	0.071
Nigeria	Wudil	2000	0.533	0.805	1.168
Nigeria	Wudil	2017	0.242	0.381	0.571
Nigeria	Wudil	2000-2017	-0.094	-0.009	0.085
Nigeria	Wukari	2000	0.399	0.658	0.949
Nigeria	Wukari	2017	0.152	0.249	0.394
Nigeria	Wukari	2000-2017	-0.087	0.011	0.096
Nigeria	Wurno	2000	0.607	0.857	1.221
Nigeria	Wurno	2017	0.263	0.391	0.568
Nigeria	Wurno	2000-2017	-0.107	-0.025	0.070
Nigeria	Wushishi	2000	0.476	0.705	1.070
Nigeria	Wushishi	2017	0.172	0.266	0.400
Nigeria	Wushishi	2000-2017	-0.124	-0.039	0.053
Nigeria	Yabo	2000	0.575	0.849	1.219
Nigeria	Yabo	2017	0.250	0.373	0.533
Nigeria	Yabo	2000-2017	-0.118	-0.028	0.070
Nigeria	Yagba East	2000	0.337	0.524	0.823
Nigeria	Yagba East	2017	0.112	0.178	0.264
Nigeria	Yagba East	2000-2017	-0.139	-0.052	0.037
Nigeria	Yagba West	2000	0.357	0.564	0.866
Nigeria	Yagba West	2017	0.113	0.187	0.273
Nigeria	Yagba West	2000-2017	-0.148	-0.060	0.038
Nigeria	Yakurr	2000	0.261	0.407	0.612
Nigeria	Yakurr	2017	0.085	0.148	0.236
Nigeria	Yakurr	2000-2017	-0.092	0.003	0.091
Nigeria	Yala Cross	2000	0.297	0.467	0.703
Nigeria	Yala Cross	2017	0.101	0.166	0.268
Nigeria	Yala Cross	2000-2017	-0.080	0.015	0.096
Nigeria	Yamaltu	2000	0.511	0.827	1.159
Nigeria	Yamaltu	2017	0.223	0.362	0.561
Nigeria	Yamaltu	2000-2017	-0.117	-0.019	0.075
Nigeria	Yankwashi	2000	0.600	0.885	1.216
Nigeria	Yankwashi	2017	0.233	0.365	0.571
Nigeria	Yankwashi	2000-2017	-0.110	-0.023	0.060
Nigeria	Yauri	2000	0.596	0.898	1.319
Nigeria	Yauri	2017	0.222	0.326	0.478
Nigeria	Yauri	2000-2017	-0.127	-0.039	0.053
Nigeria	Yenegoa	2000	0.324	0.498	0.759
Nigeria	Yenegoa	2017	0.122	0.192	0.296
Nigeria	Yenegoa	2000-2017	-0.117	-0.024	0.066
Nigeria	Yola North	2000	0.612	0.891	1.258
Nigeria	Yola North	2017	0.195	0.338	0.534
Nigeria	Yola North	2000-2017	-0.128	-0.036	0.059
Nigeria	Yola South	2000	0.613	0.891	1.254
Nigeria	Yola South	2017	0.191	0.335	0.527
Nigeria	Yola South	2000-2017	-0.128	-0.036	0.060
Nigeria	Yorro	2000	0.532	0.800	1.147
Nigeria	Yorro	2017	0.184	0.314	0.489
Nigeria	Yorro	2000-2017	-0.113	-0.006	0.081
Nigeria	Yunusari	2000	0.579	0.836	1.200
Nigeria	Yunusari	2017	0.239	0.395	0.639
Nigeria	Yunusari	2000-2017	-0.106	-0.022	0.073

Table 2: Diarrhea DALYs rate by unit *(continued)*

Country	Unit	year	mean rate	lower rate	upper rate
Nigeria	Yusufari	2000	0.569	0.800	1.125
Nigeria	Yusufari	2017	0.240	0.385	0.632
Nigeria	Yusufari	2000-2017	-0.100	-0.011	0.084
Nigeria	Zaki	2000	0.547	0.798	1.093
Nigeria	Zaki	2017	0.242	0.396	0.608
Nigeria	Zaki	2000-2017	-0.103	-0.008	0.086
Nigeria	Zango	2000	0.584	0.838	1.130
Nigeria	Zango	2017	0.216	0.343	0.546
Nigeria	Zango	2000-2017	-0.110	-0.022	0.065
Nigeria	ZangonKa	2000	0.419	0.658	1.042
Nigeria	ZangonKa	2017	0.155	0.244	0.381
Nigeria	ZangonKa	2000-2017	-0.113	-0.032	0.059
Nigeria	Zaria	2000	0.469	0.725	1.099
Nigeria	Zaria	2017	0.190	0.294	0.447
Nigeria	Zaria	2000-2017	-0.113	-0.023	0.062
Nigeria	Zing	2000	0.524	0.783	1.088
Nigeria	Zing	2017	0.165	0.297	0.458
Nigeria	Zing	2000-2017	-0.121	-0.012	0.075
Nigeria	Zurmi	2000	0.684	0.934	1.303
Nigeria	Zurmi	2017	0.252	0.387	0.579
Nigeria	Zurmi	2000-2017	-0.127	-0.034	0.057
Nigeria	Zuru	2000	0.599	0.911	1.381
Nigeria	Zuru	2017	0.217	0.313	0.465
Nigeria	Zuru	2000-2017	-0.128	-0.041	0.052
Republic of Congo	Abala	2000	0.191	0.244	0.312
Republic of Congo	Abala	2017	0.081	0.100	0.128
Republic of Congo	Abala	2000-2017	-0.054	-0.021	0.011
Republic of Congo	Bambama	2000	0.163	0.211	0.261
Republic of Congo	Bambama	2017	0.079	0.103	0.130
Republic of Congo	Bambama	2000-2017	-0.058	-0.027	0.006
Republic of Congo	Boko	2000	0.212	0.262	0.318
Republic of Congo	Boko	2017	0.069	0.088	0.108
Republic of Congo	Boko	2000-2017	-0.088	-0.061	-0.032
Republic of Congo	Boko-Songho	2000	0.180	0.227	0.285
Republic of Congo	Boko-Songho	2017	0.076	0.098	0.122
Republic of Congo	Boko-Songho	2000-2017	-0.073	-0.039	-0.007
Republic of Congo	Boundji	2000	0.172	0.230	0.289
Republic of Congo	Boundji	2017	0.076	0.098	0.123
Republic of Congo	Boundji	2000-2017	-0.070	-0.033	0.004
Republic of Congo	Brazzaville	2000	0.210	0.260	0.308
Republic of Congo	Brazzaville	2017	0.086	0.106	0.130
Republic of Congo	Brazzaville	2000-2017	-0.059	-0.029	0.001
Republic of Congo	Divénié	2000	0.164	0.205	0.257
Republic of Congo	Divénié	2017	0.074	0.098	0.125
Republic of Congo	Divénié	2000-2017	-0.073	-0.041	-0.006
Republic of Congo	Djambala	2000	0.208	0.259	0.326
Republic of Congo	Djambala	2017	0.080	0.100	0.121
Republic of Congo	Djambala	2000-2017	-0.047	-0.022	0.008
Republic of Congo	Dongou	2000	0.206	0.246	0.281
Republic of Congo	Dongou	2017	0.086	0.105	0.126
Republic of Congo	Dongou	2000-2017	-0.058	-0.030	-0.006
Republic of Congo	Epéna	2000	0.210	0.251	0.298
Republic of Congo	Epéna	2017	0.086	0.104	0.126
Republic of Congo	Epéna	2000-2017	-0.064	-0.038	-0.013
Republic of Congo	Ewo	2000	0.185	0.234	0.291
Republic of Congo	Ewo	2017	0.078	0.099	0.128
Republic of Congo	Ewo	2000-2017	-0.069	-0.035	-0.002
Republic of Congo	Gamboma	2000	0.201	0.257	0.327
Republic of Congo	Gamboma	2017	0.078	0.096	0.116
Republic of Congo	Gamboma	2000-2017	-0.054	-0.022	0.012
Republic of Congo	Impfondo	2000	0.198	0.246	0.297

Table 2: Diarrhea DALYs rate by unit *(continued)*

Country	Unit	year	mean rate	lower rate	upper rate
Republic of Congo	Impfondo	2017	0.081	0.099	0.121
Republic of Congo	Impfondo	2000-2017	-0.068	-0.041	-0.014
Republic of Congo	Kakamoeka	2000	0.169	0.217	0.281
Republic of Congo	Kakamoeka	2017	0.078	0.099	0.119
Republic of Congo	Kakamoeka	2000-2017	-0.055	-0.023	0.008
Republic of Congo	Kéllé	2000	0.197	0.244	0.301
Republic of Congo	Kéllé	2017	0.081	0.108	0.134
Republic of Congo	Kéllé	2000-2017	-0.063	-0.033	-0.001
Republic of Congo	Kibangou	2000	0.170	0.211	0.269
Republic of Congo	Kibangou	2017	0.081	0.103	0.128
Republic of Congo	Kibangou	2000-2017	-0.073	-0.040	-0.009
Republic of Congo	Kimongo	2000	0.176	0.224	0.282
Republic of Congo	Kimongo	2017	0.074	0.093	0.117
Republic of Congo	Kimongo	2000-2017	-0.069	-0.038	-0.005
Republic of Congo	Kindamba	2000	0.188	0.240	0.297
Republic of Congo	Kindamba	2017	0.077	0.098	0.123
Republic of Congo	Kindamba	2000-2017	-0.075	-0.045	-0.018
Republic of Congo	Kinkala	2000	0.235	0.287	0.350
Republic of Congo	Kinkala	2017	0.081	0.102	0.127
Republic of Congo	Kinkala	2000-2017	-0.078	-0.053	-0.024
Republic of Congo	Komono	2000	0.166	0.212	0.262
Republic of Congo	Komono	2017	0.082	0.105	0.131
Republic of Congo	Komono	2000-2017	-0.065	-0.032	0.002
Republic of Congo	Lékana	2000	0.181	0.233	0.295
Republic of Congo	Lékana	2017	0.074	0.098	0.125
Republic of Congo	Lékana	2000-2017	-0.050	-0.021	0.013
Republic of Congo	Loandjili	2000	0.163	0.208	0.264
Republic of Congo	Loandjili	2017	0.076	0.095	0.116
Republic of Congo	Loandjili	2000-2017	-0.046	-0.015	0.021
Republic of Congo	Loudima	2000	0.176	0.223	0.285
Republic of Congo	Loudima	2017	0.082	0.103	0.126
Republic of Congo	Loudima	2000-2017	-0.068	-0.037	-0.001
Republic of Congo	Loukoléla	2000	0.198	0.246	0.312
Republic of Congo	Loukoléla	2017	0.080	0.100	0.125
Republic of Congo	Loukoléla	2000-2017	-0.072	-0.046	-0.020
Republic of Congo	Louvakou (Loubomo)	2000	0.170	0.214	0.267
Republic of Congo	Louvakou (Loubomo)	2017	0.081	0.100	0.120
Republic of Congo	Louvakou (Loubomo)	2000-2017	-0.067	-0.036	-0.004
Republic of Congo	Madingo-Kayes	2000	0.170	0.215	0.266
Republic of Congo	Madingo-Kayes	2017	0.078	0.100	0.121
Republic of Congo	Madingo-Kayes	2000-2017	-0.058	-0.022	0.011
Republic of Congo	Madingou	2000	0.177	0.225	0.282
Republic of Congo	Madingou	2017	0.079	0.100	0.123
Republic of Congo	Madingou	2000-2017	-0.076	-0.040	-0.007
Republic of Congo	Makoua	2000	0.192	0.245	0.305
Republic of Congo	Makoua	2017	0.083	0.107	0.132
Republic of Congo	Makoua	2000-2017	-0.073	-0.040	-0.010
Republic of Congo	Mayama	2000	0.203	0.259	0.321
Republic of Congo	Mayama	2017	0.084	0.103	0.125
Republic of Congo	Mayama	2000-2017	-0.072	-0.041	-0.012
Republic of Congo	Mayoko	2000	0.148	0.197	0.247
Republic of Congo	Mayoko	2017	0.075	0.094	0.115
Republic of Congo	Mayoko	2000-2017	-0.067	-0.035	-0.003
Republic of Congo	Mbomo	2000	0.199	0.252	0.312
Republic of Congo	Mbomo	2017	0.087	0.112	0.136
Republic of Congo	Mbomo	2000-2017	-0.064	-0.032	0.001
Republic of Congo	Mfouati	2000	0.186	0.234	0.291
Republic of Congo	Mfouati	2017	0.074	0.097	0.121
Republic of Congo	Mfouati	2000-2017	-0.078	-0.045	-0.010
Republic of Congo	Mindouli	2000	0.204	0.254	0.311
Republic of Congo	Mindouli	2017	0.076	0.099	0.120

Table 2: Diarrhea DALYs rate by unit *(continued)*

Country	Unit	year	mean rate	lower rate	upper rate
Republic of Congo	Mindouli	2000-2017	-0.081	-0.053	-0.023
Republic of Congo	Mossaka	2000	0.197	0.246	0.306
Republic of Congo	Mossaka	2017	0.082	0.103	0.128
Republic of Congo	Mossaka	2000-2017	-0.068	-0.042	-0.013
Republic of Congo	Mossendjo	2000	0.162	0.208	0.266
Republic of Congo	Mossendjo	2017	0.078	0.101	0.127
Republic of Congo	Mossendjo	2000-2017	-0.072	-0.036	-0.003
Republic of Congo	Mouyondzi	2000	0.182	0.228	0.283
Republic of Congo	Mouyondzi	2017	0.078	0.099	0.119
Republic of Congo	Mouyondzi	2000-2017	-0.076	-0.044	-0.015
Republic of Congo	Mvouti	2000	0.179	0.225	0.282
Republic of Congo	Mvouti	2017	0.079	0.097	0.119
Republic of Congo	Mvouti	2000-2017	-0.056	-0.026	0.004
Republic of Congo	Ngabé	2000	0.198	0.249	0.311
Republic of Congo	Ngabé	2017	0.077	0.093	0.113
Republic of Congo	Ngabé	2000-2017	-0.075	-0.043	-0.006
Republic of Congo	Ngamaba	2000	0.215	0.264	0.314
Republic of Congo	Ngamaba	2017	0.090	0.109	0.133
Republic of Congo	Ngamaba	2000-2017	-0.062	-0.033	-0.002
Republic of Congo	Nkayi District	2000	0.174	0.224	0.283
Republic of Congo	Nkayi District	2017	0.082	0.102	0.127
Republic of Congo	Nkayi District	2000-2017	-0.073	-0.036	-0.005
Republic of Congo	Okoyo	2000	0.178	0.223	0.278
Republic of Congo	Okoyo	2017	0.073	0.093	0.123
Republic of Congo	Okoyo	2000-2017	-0.063	-0.030	0.004
Republic of Congo	Ouesso	2000	0.196	0.236	0.276
Republic of Congo	Ouesso	2017	0.090	0.107	0.128
Republic of Congo	Ouesso	2000-2017	-0.053	-0.029	-0.005
Republic of Congo	Owando	2000	0.195	0.241	0.304
Republic of Congo	Owando	2017	0.081	0.104	0.131
Republic of Congo	Owando	2000-2017	-0.073	-0.038	-0.005
Republic of Congo	Pointe Noire	2000	0.147	0.188	0.237
Republic of Congo	Pointe Noire	2017	0.071	0.089	0.109
Republic of Congo	Pointe Noire	2000-2017	-0.046	-0.015	0.019
Republic of Congo	Sembé	2000	0.186	0.227	0.280
Republic of Congo	Sembé	2017	0.089	0.107	0.129
Republic of Congo	Sembé	2000-2017	-0.055	-0.024	0.003
Republic of Congo	Sibiti	2000	0.176	0.220	0.279
Republic of Congo	Sibiti	2017	0.083	0.104	0.129
Republic of Congo	Sibiti	2000-2017	-0.070	-0.037	-0.009
Republic of Congo	Souanké	2000	0.185	0.231	0.282
Republic of Congo	Souanké	2017	0.089	0.110	0.135
Republic of Congo	Souanké	2000-2017	-0.053	-0.026	0.001
Republic of Congo	Zanaga	2000	0.169	0.216	0.270
Republic of Congo	Zanaga	2017	0.081	0.102	0.125
Republic of Congo	Zanaga	2000-2017	-0.064	-0.031	0.001
Rwanda	Bugesera	2000	0.593	0.764	0.934
Rwanda	Bugesera	2017	0.070	0.094	0.122
Rwanda	Bugesera	2000-2017	-0.186	-0.156	-0.124
Rwanda	Burera	2000	0.502	0.625	0.771
Rwanda	Burera	2017	0.072	0.092	0.119
Rwanda	Burera	2000-2017	-0.160	-0.133	-0.102
Rwanda	Gakenke	2000	0.463	0.581	0.701
Rwanda	Gakenke	2017	0.062	0.083	0.111
Rwanda	Gakenke	2000-2017	-0.160	-0.130	-0.097
Rwanda	Gasabo	2000	0.422	0.536	0.659
Rwanda	Gasabo	2017	0.051	0.071	0.091
Rwanda	Gasabo	2000-2017	-0.171	-0.141	-0.108
Rwanda	Gatsibo	2000	0.482	0.616	0.751
Rwanda	Gatsibo	2017	0.062	0.084	0.110
Rwanda	Gatsibo	2000-2017	-0.166	-0.136	-0.104

Table 2: Diarrhea DALYs rate by unit *(continued)*

Country	Unit	year	mean rate	lower rate	upper rate
Rwanda	Gicumbi	2000	0.499	0.637	0.782
Rwanda	Gicumbi	2017	0.063	0.082	0.105
Rwanda	Gicumbi	2000-2017	-0.170	-0.141	-0.108
Rwanda	Gisagara	2000	0.658	0.822	1.009
Rwanda	Gisagara	2017	0.083	0.110	0.149
Rwanda	Gisagara	2000-2017	-0.181	-0.147	-0.115
Rwanda	Huye	2000	0.560	0.712	0.884
Rwanda	Huye	2017	0.070	0.095	0.130
Rwanda	Huye	2000-2017	-0.175	-0.140	-0.108
Rwanda	Kamonyi	2000	0.478	0.605	0.742
Rwanda	Kamonyi	2017	0.057	0.080	0.103
Rwanda	Kamonyi	2000-2017	-0.171	-0.139	-0.106
Rwanda	Karongi	2000	0.500	0.634	0.791
Rwanda	Karongi	2017	0.067	0.094	0.123
Rwanda	Karongi	2000-2017	-0.160	-0.125	-0.094
Rwanda	Kayonza	2000	0.493	0.620	0.761
Rwanda	Kayonza	2017	0.062	0.084	0.108
Rwanda	Kayonza	2000-2017	-0.164	-0.135	-0.105
Rwanda	Kicukiro	2000	0.455	0.581	0.711
Rwanda	Kicukiro	2017	0.052	0.072	0.092
Rwanda	Kicukiro	2000-2017	-0.176	-0.145	-0.111
Rwanda	Kirehe	2000	0.522	0.660	0.833
Rwanda	Kirehe	2017	0.066	0.089	0.117
Rwanda	Kirehe	2000-2017	-0.165	-0.137	-0.108
Rwanda	Muhanga	2000	0.443	0.567	0.699
Rwanda	Muhanga	2017	0.057	0.079	0.102
Rwanda	Muhanga	2000-2017	-0.163	-0.132	-0.100
Rwanda	Musanze	2000	0.458	0.577	0.696
Rwanda	Musanze	2017	0.068	0.091	0.117
Rwanda	Musanze	2000-2017	-0.153	-0.124	-0.093
Rwanda	Ngoma	2000	0.532	0.677	0.843
Rwanda	Ngoma	2017	0.062	0.084	0.108
Rwanda	Ngoma	2000-2017	-0.178	-0.149	-0.120
Rwanda	Ngororero	2000	0.465	0.598	0.752
Rwanda	Ngororero	2017	0.070	0.097	0.124
Rwanda	Ngororero	2000-2017	-0.154	-0.121	-0.090
Rwanda	Nyabihu	2000	0.472	0.606	0.732
Rwanda	Nyabihu	2017	0.074	0.101	0.131
Rwanda	Nyabihu	2000-2017	-0.151	-0.119	-0.084
Rwanda	Nyagatare	2000	0.513	0.642	0.798
Rwanda	Nyagatare	2017	0.064	0.087	0.114
Rwanda	Nyagatare	2000-2017	-0.167	-0.137	-0.106
Rwanda	Nyamagabe	2000	0.562	0.710	0.898
Rwanda	Nyamagabe	2017	0.070	0.096	0.130
Rwanda	Nyamagabe	2000-2017	-0.172	-0.136	-0.106
Rwanda	Nyamasheke	2000	0.551	0.714	0.890
Rwanda	Nyamasheke	2017	0.070	0.097	0.130
Rwanda	Nyamasheke	2000-2017	-0.169	-0.133	-0.101
Rwanda	Nyanza	2000	0.549	0.701	0.887
Rwanda	Nyanza	2017	0.070	0.093	0.125
Rwanda	Nyanza	2000-2017	-0.177	-0.143	-0.107
Rwanda	Nyarugenge	2000	0.444	0.567	0.701
Rwanda	Nyarugenge	2017	0.052	0.072	0.092
Rwanda	Nyarugenge	2000-2017	-0.177	-0.145	-0.111
Rwanda	Nyaruguru	2000	0.596	0.762	0.936
Rwanda	Nyaruguru	2017	0.082	0.109	0.147
Rwanda	Nyaruguru	2000-2017	-0.174	-0.137	-0.104
Rwanda	Rubavu	2000	0.510	0.653	0.795
Rwanda	Rubavu	2017	0.072	0.097	0.126
Rwanda	Rubavu	2000-2017	-0.158	-0.125	-0.091
Rwanda	Ruhango	2000	0.509	0.645	0.804

Table 2: Diarrhea DALYs rate by unit *(continued)*

Country	Unit	year	mean rate	lower rate	upper rate
Rwanda	Ruhango	2017	0.065	0.088	0.118
Rwanda	Ruhango	2000-2017	-0.169	-0.135	-0.100
Rwanda	Rulindo	2000	0.474	0.604	0.736
Rwanda	Rulindo	2017	0.062	0.082	0.107
Rwanda	Rulindo	2000-2017	-0.165	-0.138	-0.105
Rwanda	Rusizi	2000	0.581	0.734	0.915
Rwanda	Rusizi	2017	0.077	0.102	0.135
Rwanda	Rusizi	2000-2017	-0.167	-0.133	-0.102
Rwanda	Rutsiro	2000	0.499	0.639	0.786
Rwanda	Rutsiro	2017	0.073	0.099	0.128
Rwanda	Rutsiro	2000-2017	-0.158	-0.123	-0.092
Rwanda	Rwamagana	2000	0.465	0.586	0.723
Rwanda	Rwamagana	2017	0.054	0.075	0.097
Rwanda	Rwamagana	2000-2017	-0.174	-0.142	-0.110
Senegal	Bakel	2000	0.346	0.454	0.585
Senegal	Bakel	2017	0.070	0.102	0.138
Senegal	Bakel	2000-2017	-0.144	-0.072	-0.004
Senegal	Bambey	2000	0.348	0.446	0.554
Senegal	Bambey	2017	0.094	0.131	0.174
Senegal	Bambey	2000-2017	-0.162	-0.080	0.000
Senegal	Bignona	2000	0.312	0.401	0.495
Senegal	Bignona	2017	0.109	0.159	0.217
Senegal	Bignona	2000-2017	-0.173	-0.091	0.005
Senegal	Birkilane	2000	0.397	0.515	0.658
Senegal	Birkilane	2017	0.108	0.150	0.202
Senegal	Birkilane	2000-2017	-0.198	-0.105	-0.023
Senegal	Bounkiling	2000	0.409	0.525	0.652
Senegal	Bounkiling	2017	0.119	0.163	0.229
Senegal	Bounkiling	2000-2017	-0.205	-0.108	-0.017
Senegal	Dagana	2000	0.258	0.344	0.456
Senegal	Dagana	2017	0.085	0.121	0.166
Senegal	Dagana	2000-2017	-0.120	-0.052	0.027
Senegal	Dakar	2000	0.294	0.400	0.524
Senegal	Dakar	2017	0.098	0.142	0.190
Senegal	Dakar	2000-2017	-0.161	-0.079	0.009
Senegal	Diourbel	2000	0.357	0.462	0.586
Senegal	Diourbel	2017	0.097	0.133	0.177
Senegal	Diourbel	2000-2017	-0.164	-0.081	0.000
Senegal	Fatick	2000	0.358	0.453	0.566
Senegal	Fatick	2017	0.104	0.144	0.191
Senegal	Fatick	2000-2017	-0.169	-0.088	-0.012
Senegal	Foundiougne	2000	0.369	0.470	0.593
Senegal	Foundiougne	2017	0.110	0.156	0.211
Senegal	Foundiougne	2000-2017	-0.188	-0.098	-0.022
Senegal	Gossas	2000	0.373	0.481	0.625
Senegal	Gossas	2017	0.100	0.136	0.180
Senegal	Gossas	2000-2017	-0.177	-0.090	-0.010
Senegal	Goudiry	2000	0.381	0.480	0.611
Senegal	Goudiry	2017	0.071	0.102	0.142
Senegal	Goudiry	2000-2017	-0.149	-0.069	0.012
Senegal	Goudomp	2000	0.341	0.420	0.532
Senegal	Goudomp	2017	0.103	0.140	0.194
Senegal	Goudomp	2000-2017	-0.186	-0.096	-0.002
Senegal	Guédiawaye	2000	0.309	0.408	0.534
Senegal	Guédiawaye	2017	0.108	0.155	0.208
Senegal	Guédiawaye	2000-2017	-0.160	-0.079	0.008
Senegal	Guinguinéo	2000	0.385	0.500	0.636
Senegal	Guinguinéo	2017	0.104	0.145	0.190
Senegal	Guinguinéo	2000-2017	-0.187	-0.096	-0.018
Senegal	Kaffrine	2000	0.389	0.512	0.654
Senegal	Kaffrine	2017	0.104	0.144	0.187

Table 2: Diarrhea DALYs rate by unit *(continued)*

Country	Unit	year	mean rate	lower rate	upper rate
Senegal	Kaffrine	2000-2017	-0.203	-0.104	-0.024
Senegal	Kanel	2000	0.332	0.420	0.533
Senegal	Kanel	2017	0.074	0.107	0.146
Senegal	Kanel	2000-2017	-0.146	-0.070	-0.001
Senegal	Kaolack	2000	0.370	0.473	0.594
Senegal	Kaolack	2017	0.104	0.144	0.192
Senegal	Kaolack	2000-2017	-0.186	-0.097	-0.017
Senegal	Kébémer	2000	0.300	0.389	0.496
Senegal	Kébémer	2017	0.087	0.121	0.160
Senegal	Kébémer	2000-2017	-0.143	-0.067	0.013
Senegal	Kédougou	2000	0.285	0.401	0.537
Senegal	Kédougou	2017	0.062	0.086	0.113
Senegal	Kédougou	2000-2017	-0.151	-0.056	0.043
Senegal	Kolda	2000	0.375	0.466	0.588
Senegal	Kolda	2017	0.096	0.129	0.175
Senegal	Kolda	2000-2017	-0.182	-0.087	0.001
Senegal	Koungheul	2000	0.385	0.506	0.657
Senegal	Koungheul	2017	0.095	0.133	0.179
Senegal	Koungheul	2000-2017	-0.189	-0.100	-0.020
Senegal	Koupentoum	2000	0.388	0.503	0.647
Senegal	Koupentoum	2017	0.087	0.124	0.173
Senegal	Koupentoum	2000-2017	-0.177	-0.089	-0.008
Senegal	Linguère	2000	0.312	0.412	0.547
Senegal	Linguère	2017	0.088	0.121	0.163
Senegal	Linguère	2000-2017	-0.146	-0.069	0.016
Senegal	Louga	2000	0.276	0.364	0.469
Senegal	Louga	2017	0.087	0.120	0.163
Senegal	Louga	2000-2017	-0.131	-0.058	0.026
Senegal	Malème Hodar	2000	0.383	0.511	0.649
Senegal	Malème Hodar	2017	0.101	0.139	0.185
Senegal	Malème Hodar	2000-2017	-0.193	-0.100	-0.022
Senegal	Matam	2000	0.295	0.376	0.475
Senegal	Matam	2017	0.077	0.111	0.152
Senegal	Matam	2000-2017	-0.144	-0.060	0.011
Senegal	Mbacké	2000	0.348	0.454	0.580
Senegal	Mbacké	2017	0.093	0.127	0.169
Senegal	Mbacké	2000-2017	-0.168	-0.083	0.000
Senegal	Mbour	2000	0.322	0.415	0.520
Senegal	Mbour	2017	0.097	0.138	0.186
Senegal	Mbour	2000-2017	-0.163	-0.082	0.004
Senegal	Médina Yoro Foula	2000	0.405	0.505	0.638
Senegal	Médina Yoro Foula	2017	0.107	0.141	0.193
Senegal	Médina Yoro Foula	2000-2017	-0.187	-0.090	-0.003
Senegal	Nioro du Rip	2000	0.386	0.499	0.627
Senegal	Nioro du Rip	2017	0.110	0.153	0.204
Senegal	Nioro du Rip	2000-2017	-0.196	-0.103	-0.022
Senegal	Oussouye	2000	0.267	0.345	0.438
Senegal	Oussouye	2017	0.097	0.143	0.204
Senegal	Oussouye	2000-2017	-0.179	-0.088	0.003
Senegal	Pikine	2000	0.307	0.411	0.535
Senegal	Pikine	2017	0.092	0.134	0.182
Senegal	Pikine	2000-2017	-0.169	-0.088	-0.002
Senegal	Podor	2000	0.271	0.348	0.465
Senegal	Podor	2017	0.082	0.118	0.155
Senegal	Podor	2000-2017	-0.137	-0.060	0.016
Senegal	Ranérou Ferlo	2000	0.337	0.436	0.560
Senegal	Ranérou Ferlo	2017	0.082	0.115	0.159
Senegal	Ranérou Ferlo	2000-2017	-0.149	-0.073	0.009
Senegal	Rufisque	2000	0.287	0.382	0.490
Senegal	Rufisque	2017	0.085	0.125	0.170
Senegal	Rufisque	2000-2017	-0.166	-0.087	-0.001

Table 2: Diarrhea DALYs rate by unit *(continued)*

Country	Unit	year	mean rate	lower rate	upper rate
Senegal	Saint-Louis	2000	0.256	0.349	0.453
Senegal	Saint-Louis	2017	0.085	0.121	0.165
Senegal	Saint-Louis	2000-2017	-0.130	-0.053	0.026
Senegal	Salémata	2000	0.257	0.363	0.490
Senegal	Salémata	2017	0.062	0.085	0.115
Senegal	Salémata	2000-2017	-0.149	-0.050	0.051
Senegal	Saraya	2000	0.322	0.447	0.607
Senegal	Saraya	2017	0.063	0.092	0.124
Senegal	Saraya	2000-2017	-0.155	-0.056	0.047
Senegal	Sédhiou	2000	0.377	0.467	0.588
Senegal	Sédhiou	2017	0.112	0.152	0.215
Senegal	Sédhiou	2000-2017	-0.191	-0.099	-0.007
Senegal	Tambacounda	2000	0.387	0.479	0.614
Senegal	Tambacounda	2017	0.080	0.109	0.150
Senegal	Tambacounda	2000-2017	-0.163	-0.074	0.007
Senegal	Thiès	2000	0.310	0.393	0.496
Senegal	Thiès	2017	0.089	0.127	0.170
Senegal	Thiès	2000-2017	-0.158	-0.077	0.007
Senegal	Tivaouane	2000	0.305	0.398	0.508
Senegal	Tivaouane	2017	0.089	0.126	0.167
Senegal	Tivaouane	2000-2017	-0.154	-0.071	0.009
Senegal	Vélingara	2000	0.351	0.444	0.562
Senegal	Vélingara	2017	0.079	0.111	0.154
Senegal	Vélingara	2000-2017	-0.178	-0.072	0.010
Senegal	Ziguinchor	2000	0.277	0.348	0.441
Senegal	Ziguinchor	2017	0.095	0.138	0.192
Senegal	Ziguinchor	2000-2017	-0.167	-0.085	0.012
Sierra Leone	Bo	2000	0.335	0.475	0.631
Sierra Leone	Bo	2017	0.121	0.161	0.212
Sierra Leone	Bo	2000-2017	-0.147	-0.076	0.017
Sierra Leone	Bombali	2000	0.376	0.508	0.649
Sierra Leone	Bombali	2017	0.124	0.166	0.221
Sierra Leone	Bombali	2000-2017	-0.152	-0.075	0.013
Sierra Leone	Bonthe	2000	0.345	0.491	0.657
Sierra Leone	Bonthe	2017	0.114	0.159	0.210
Sierra Leone	Bonthe	2000-2017	-0.152	-0.071	0.016
Sierra Leone	Kailahun	2000	0.395	0.529	0.710
Sierra Leone	Kailahun	2017	0.137	0.185	0.232
Sierra Leone	Kailahun	2000-2017	-0.170	-0.092	-0.005
Sierra Leone	Kambia	2000	0.428	0.583	0.759
Sierra Leone	Kambia	2017	0.180	0.232	0.301
Sierra Leone	Kambia	2000-2017	-0.148	-0.069	0.031
Sierra Leone	Kenema	2000	0.367	0.501	0.665
Sierra Leone	Kenema	2017	0.128	0.171	0.219
Sierra Leone	Kenema	2000-2017	-0.152	-0.078	0.013
Sierra Leone	Koinadugu	2000	0.406	0.556	0.747
Sierra Leone	Koinadugu	2017	0.144	0.190	0.248
Sierra Leone	Koinadugu	2000-2017	-0.149	-0.078	0.010
Sierra Leone	Kono	2000	0.363	0.505	0.689
Sierra Leone	Kono	2017	0.129	0.172	0.219
Sierra Leone	Kono	2000-2017	-0.158	-0.076	0.014
Sierra Leone	Moyamba	2000	0.357	0.500	0.667
Sierra Leone	Moyamba	2017	0.131	0.175	0.234
Sierra Leone	Moyamba	2000-2017	-0.135	-0.061	0.040
Sierra Leone	Port Loko	2000	0.414	0.557	0.734
Sierra Leone	Port Loko	2017	0.154	0.206	0.270
Sierra Leone	Port Loko	2000-2017	-0.139	-0.061	0.038
Sierra Leone	Pujehun	2000	0.322	0.440	0.596
Sierra Leone	Pujehun	2017	0.124	0.165	0.222
Sierra Leone	Pujehun	2000-2017	-0.139	-0.070	0.013
Sierra Leone	Tonkolili	2000	0.367	0.523	0.686

Table 2: Diarrhea DALYs rate by unit *(continued)*

Country	Unit	year	mean rate	lower rate	upper rate
Sierra Leone	Tonkolili	2017	0.130	0.171	0.220
Sierra Leone	Tonkolili	2000-2017	-0.140	-0.071	0.017
Sierra Leone	Western Rural	2000	0.316	0.444	0.605
Sierra Leone	Western Rural	2017	0.132	0.182	0.242
Sierra Leone	Western Rural	2000-2017	-0.145	-0.058	0.051
Sierra Leone	Western Urban	2000	0.313	0.437	0.601
Sierra Leone	Western Urban	2017	0.129	0.176	0.229
Sierra Leone	Western Urban	2000-2017	-0.144	-0.059	0.040
Somalia	Aadan	2000	0.416	0.651	0.990
Somalia	Aadan	2017	0.110	0.190	0.294
Somalia	Aadan	2000-2017	-0.111	-0.058	-0.003
Somalia	Afgooye	2000	0.393	0.626	0.961
Somalia	Afgooye	2017	0.117	0.190	0.301
Somalia	Afgooye	2000-2017	-0.104	-0.050	0.013
Somalia	Afmadow	2000	0.449	0.747	1.143
Somalia	Afmadow	2017	0.153	0.241	0.375
Somalia	Afmadow	2000-2017	-0.090	-0.044	0.011
Somalia	Baar-Dheere	2000	0.377	0.618	0.968
Somalia	Baar-Dheere	2017	0.126	0.201	0.316
Somalia	Baar-Dheere	2000-2017	-0.092	-0.043	0.012
Somalia	Badhaadhe	2000	0.414	0.721	1.148
Somalia	Badhaadhe	2017	0.144	0.238	0.380
Somalia	Badhaadhe	2000-2017	-0.086	-0.040	0.009
Somalia	Badhan	2000	0.332	0.562	0.836
Somalia	Badhan	2017	0.147	0.221	0.340
Somalia	Badhan	2000-2017	-0.107	-0.051	0.003
Somalia	Baki	2000	0.399	0.604	0.889
Somalia	Baki	2017	0.122	0.192	0.284
Somalia	Baki	2000-2017	-0.127	-0.076	-0.028
Somalia	Balcad	2000	0.411	0.632	0.983
Somalia	Balcad	2017	0.114	0.193	0.310
Somalia	Balcad	2000-2017	-0.105	-0.051	0.011
Somalia	Bander-Beyla	2000	0.348	0.592	0.955
Somalia	Bander-Beyla	2017	0.142	0.224	0.343
Somalia	Bander-Beyla	2000-2017	-0.106	-0.051	-0.004
Somalia	Baraawe	2000	0.466	0.757	1.214
Somalia	Baraawe	2017	0.139	0.221	0.354
Somalia	Baraawe	2000-2017	-0.111	-0.052	0.006
Somalia	Baydhabo	2000	0.366	0.591	0.927
Somalia	Baydhabo	2017	0.107	0.187	0.294
Somalia	Baydhabo	2000-2017	-0.101	-0.045	0.020
Somalia	Beled Weyn	2000	0.418	0.674	1.060
Somalia	Beled Weyn	2017	0.114	0.194	0.299
Somalia	Beled Weyn	2000-2017	-0.115	-0.064	-0.014
Somalia	Beled Xaawo	2000	0.407	0.645	0.960
Somalia	Beled Xaawo	2017	0.126	0.200	0.315
Somalia	Beled Xaawo	2000-2017	-0.101	-0.052	0.000
Somalia	Berbera	2000	0.344	0.555	0.853
Somalia	Berbera	2017	0.139	0.208	0.296
Somalia	Berbera	2000-2017	-0.108	-0.060	-0.007
Somalia	Boorama	2000	0.446	0.671	0.949
Somalia	Boorama	2017	0.112	0.175	0.247
Somalia	Boorama	2000-2017	-0.133	-0.090	-0.045
Somalia	Bosaaso	2000	0.316	0.541	0.810
Somalia	Bosaaso	2017	0.137	0.221	0.328
Somalia	Bosaaso	2000-2017	-0.101	-0.047	0.008
Somalia	Bu'aale	2000	0.521	0.861	1.379
Somalia	Bu'aale	2017	0.154	0.266	0.415
Somalia	Bu'aale	2000-2017	-0.105	-0.051	0.002
Somalia	Burao	2000	0.351	0.561	0.889
Somalia	Burao	2017	0.125	0.194	0.305

Table 2: Diarrhea DALYs rate by unit *(continued)*

Country	Unit	year	mean rate	lower rate	upper rate
Somalia	Burao	2000-2017	-0.125	-0.071	-0.018
Somalia	Burtinle	2000	0.347	0.594	0.958
Somalia	Burtinle	2017	0.136	0.213	0.315
Somalia	Burtinle	2000-2017	-0.119	-0.056	0.002
Somalia	Buuhoodle	2000	0.335	0.577	0.888
Somalia	Buuhoodle	2017	0.121	0.194	0.287
Somalia	Buuhoodle	2000-2017	-0.117	-0.066	-0.013
Somalia	Buulo Burdo	2000	0.453	0.714	1.127
Somalia	Buulo Burdo	2017	0.121	0.203	0.304
Somalia	Buulo Burdo	2000-2017	-0.117	-0.062	-0.010
Somalia	Buur Xakaba	2000	0.432	0.679	1.055
Somalia	Buur Xakaba	2017	0.116	0.199	0.320
Somalia	Buur Xakaba	2000-2017	-0.105	-0.050	0.006
Somalia	Caabudwaaq	2000	0.417	0.658	1.053
Somalia	Caabudwaaq	2017	0.116	0.190	0.275
Somalia	Caabudwaaq	2000-2017	-0.125	-0.068	-0.015
Somalia	Cadaado	2000	0.365	0.613	0.956
Somalia	Cadaado	2017	0.121	0.191	0.271
Somalia	Cadaado	2000-2017	-0.122	-0.066	-0.005
Somalia	Cadale	2000	0.406	0.644	0.999
Somalia	Cadale	2017	0.114	0.194	0.304
Somalia	Cadale	2000-2017	-0.105	-0.049	0.008
Somalia	Calawla	2000	0.318	0.565	0.912
Somalia	Calawla	2017	0.145	0.233	0.330
Somalia	Calawla	2000-2017	-0.105	-0.046	0.011
Somalia	Caynabo	2000	0.358	0.607	0.926
Somalia	Caynabo	2017	0.132	0.210	0.317
Somalia	Caynabo	2000-2017	-0.116	-0.064	-0.007
Somalia	Ceel Barde	2000	0.314	0.526	0.834
Somalia	Ceel Barde	2017	0.097	0.166	0.259
Somalia	Ceel Barde	2000-2017	-0.098	-0.049	0.008
Somalia	Ceel Buur	2000	0.430	0.681	1.045
Somalia	Ceel Buur	2017	0.125	0.206	0.303
Somalia	Ceel Buur	2000-2017	-0.117	-0.063	-0.009
Somalia	Ceel Dheer	2000	0.438	0.700	1.092
Somalia	Ceel Dheer	2017	0.128	0.211	0.324
Somalia	Ceel Dheer	2000-2017	-0.114	-0.061	-0.007
Somalia	Ceel Waaq	2000	0.403	0.650	1.014
Somalia	Ceel Waaq	2017	0.123	0.196	0.315
Somalia	Ceel Waaq	2000-2017	-0.096	-0.048	0.007
Somalia	Ceel-Afwein	2000	0.361	0.593	0.897
Somalia	Ceel-Afwein	2017	0.139	0.214	0.307
Somalia	Ceel-Afwein	2000-2017	-0.113	-0.061	0.002
Somalia	Ceerigaabo	2000	0.338	0.567	0.883
Somalia	Ceerigaabo	2017	0.138	0.214	0.309
Somalia	Ceerigaabo	2000-2017	-0.106	-0.057	0.001
Somalia	Dhuusamareeb	2000	0.416	0.660	1.021
Somalia	Dhuusamareeb	2017	0.122	0.197	0.284
Somalia	Dhuusamareeb	2000-2017	-0.119	-0.065	-0.012
Somalia	Diinsoor	2000	0.434	0.702	1.060
Somalia	Diinsoor	2017	0.127	0.213	0.339
Somalia	Diinsoor	2000-2017	-0.104	-0.049	0.008
Somalia	Dolow	2000	0.355	0.590	0.861
Somalia	Dolow	2017	0.105	0.171	0.273
Somalia	Dolow	2000-2017	-0.105	-0.055	-0.001
Somalia	Eyl	2000	0.347	0.602	0.957
Somalia	Eyl	2017	0.143	0.215	0.319
Somalia	Eyl	2000-2017	-0.121	-0.061	-0.009
Somalia	Gaalkacayo	2000	0.332	0.571	0.882
Somalia	Gaalkacayo	2017	0.124	0.188	0.280
Somalia	Gaalkacayo	2000-2017	-0.123	-0.065	-0.004

Table 2: Diarrhea DALYs rate by unit *(continued)*

Country	Unit	year	mean rate	lower rate	upper rate
Somalia	Gabiley	2000	0.360	0.549	0.789
Somalia	Gabiley	2017	0.107	0.170	0.249
Somalia	Gabiley	2000-2017	-0.125	-0.077	-0.028
Somalia	Garbahaaray	2000	0.342	0.559	0.858
Somalia	Garbahaaray	2017	0.110	0.179	0.284
Somalia	Garbahaaray	2000-2017	-0.089	-0.043	0.011
Somalia	Garoowe	2000	0.358	0.601	0.952
Somalia	Garoowe	2017	0.136	0.221	0.326
Somalia	Garoowe	2000-2017	-0.114	-0.056	0.002
Somalia	Goldogob	2000	0.335	0.562	0.872
Somalia	Goldogob	2017	0.115	0.184	0.260
Somalia	Goldogob	2000-2017	-0.120	-0.067	-0.007
Somalia	Hargeysa	2000	0.339	0.511	0.726
Somalia	Hargeysa	2017	0.113	0.183	0.266
Somalia	Hargeysa	2000-2017	-0.118	-0.066	-0.016
Somalia	Hobyo	2000	0.360	0.587	0.893
Somalia	Hobyo	2017	0.128	0.197	0.300
Somalia	Hobyo	2000-2017	-0.117	-0.063	-0.008
Somalia	Iskushuban	2000	0.333	0.576	0.894
Somalia	Iskushuban	2017	0.147	0.232	0.347
Somalia	Iskushuban	2000-2017	-0.098	-0.048	0.003
Somalia	Jalalaqsi	2000	0.487	0.761	1.187
Somalia	Jalalaqsi	2017	0.121	0.205	0.326
Somalia	Jalalaqsi	2000-2017	-0.122	-0.062	-0.003
Somalia	Jamaame	2000	0.425	0.723	1.195
Somalia	Jamaame	2017	0.140	0.228	0.365
Somalia	Jamaame	2000-2017	-0.093	-0.046	0.011
Somalia	Jariiban	2000	0.363	0.594	0.917
Somalia	Jariiban	2017	0.130	0.214	0.315
Somalia	Jariiban	2000-2017	-0.123	-0.059	-0.003
Somalia	Jawhar	2000	0.438	0.677	1.052
Somalia	Jawhar	2017	0.113	0.191	0.310
Somalia	Jawhar	2000-2017	-0.112	-0.054	0.008
Somalia	Jilib	2000	0.508	0.861	1.370
Somalia	Jilib	2017	0.155	0.261	0.436
Somalia	Jilib	2000-2017	-0.099	-0.050	0.011
Somalia	Kismaayo	2000	0.394	0.673	1.066
Somalia	Kismaayo	2017	0.141	0.228	0.360
Somalia	Kismaayo	2000-2017	-0.086	-0.042	0.010
Somalia	Kuntuwaaray	2000	0.427	0.712	1.093
Somalia	Kuntuwaaray	2017	0.120	0.204	0.322
Somalia	Kuntuwaaray	2000-2017	-0.114	-0.052	0.009
Somalia	Lascaanod	2000	0.339	0.591	0.951
Somalia	Lascaanod	2017	0.132	0.203	0.289
Somalia	Lascaanod	2000-2017	-0.113	-0.063	-0.005
Somalia	Lughaya	2000	0.412	0.637	0.954
Somalia	Lughaya	2017	0.133	0.207	0.299
Somalia	Lughaya	2000-2017	-0.130	-0.076	-0.029
Somalia	Luuk	2000	0.321	0.532	0.788
Somalia	Luuk	2017	0.099	0.167	0.265
Somalia	Luuk	2000-2017	-0.090	-0.044	0.015
Somalia	Marka	2000	0.423	0.722	1.168
Somalia	Marka	2017	0.123	0.207	0.321
Somalia	Marka	2000-2017	-0.116	-0.054	0.010
Somalia	Mogadisho	2000	0.378	0.598	0.928
Somalia	Mogadisho	2017	0.116	0.191	0.303
Somalia	Mogadisho	2000-2017	-0.102	-0.049	0.015
Somalia	Oodweyne	2000	0.334	0.518	0.793
Somalia	Oodweyne	2017	0.119	0.186	0.281
Somalia	Oodweyne	2000-2017	-0.125	-0.070	-0.013
Somalia	Qandala	2000	0.317	0.550	0.833

Table 2: Diarrhea DALYs rate by unit *(continued)*

Country	Unit	year	mean rate	lower rate	upper rate
Somalia	Qandala	2017	0.144	0.230	0.329
Somalia	Qandala	2000-2017	-0.098	-0.044	0.010
Somalia	Qansax Dheere	2000	0.378	0.610	0.941
Somalia	Qansax Dheere	2017	0.111	0.190	0.315
Somalia	Qansax Dheere	2000-2017	-0.096	-0.044	0.015
Somalia	Qardho	2000	0.340	0.581	0.879
Somalia	Qardho	2017	0.137	0.221	0.336
Somalia	Qardho	2000-2017	-0.107	-0.051	0.007
Somalia	Qoryooley	2000	0.415	0.703	1.085
Somalia	Qoryooley	2017	0.118	0.201	0.316
Somalia	Qoryooley	2000-2017	-0.116	-0.054	0.009
Somalia	Rab Dhuure	2000	0.292	0.491	0.734
Somalia	Rab Dhuure	2017	0.089	0.158	0.246
Somalia	Rab Dhuure	2000-2017	-0.089	-0.041	0.021
Somalia	Saakow	2000	0.479	0.823	1.270
Somalia	Saakow	2017	0.154	0.254	0.396
Somalia	Saakow	2000-2017	-0.103	-0.050	0.002
Somalia	Sablale	2000	0.459	0.744	1.196
Somalia	Sablale	2017	0.132	0.215	0.352
Somalia	Sablale	2000-2017	-0.106	-0.051	0.010
Somalia	Sheekh	2000	0.319	0.531	0.857
Somalia	Sheekh	2017	0.128	0.192	0.283
Somalia	Sheekh	2000-2017	-0.119	-0.065	-0.005
Somalia	Taleex	2000	0.341	0.597	0.972
Somalia	Taleex	2017	0.142	0.224	0.336
Somalia	Taleex	2000-2017	-0.104	-0.054	0.002
Somalia	Tiyeeglow	2000	0.339	0.547	0.871
Somalia	Tiyeeglow	2017	0.099	0.169	0.274
Somalia	Tiyeeglow	2000-2017	-0.105	-0.047	0.009
Somalia	Wajid	2000	0.304	0.493	0.766
Somalia	Wajid	2017	0.091	0.163	0.253
Somalia	Wajid	2000-2017	-0.096	-0.043	0.026
Somalia	Wanla Weyn	2000	0.423	0.654	1.001
Somalia	Wanla Weyn	2017	0.119	0.187	0.295
Somalia	Wanla Weyn	2000-2017	-0.107	-0.053	0.010
Somalia	Xarardheere	2000	0.391	0.646	0.995
Somalia	Xarardheere	2017	0.127	0.218	0.341
Somalia	Xarardheere	2000-2017	-0.115	-0.059	0.001
Somalia	Xudun	2000	0.343	0.601	0.994
Somalia	Xudun	2017	0.131	0.213	0.296
Somalia	Xudun	2000-2017	-0.111	-0.061	-0.006
Somalia	Xudur	2000	0.299	0.493	0.783
Somalia	Xudur	2017	0.094	0.161	0.250
Somalia	Xudur	2000-2017	-0.098	-0.042	0.016
Somalia	Zeylac	2000	0.380	0.592	0.845
Somalia	Zeylac	2017	0.121	0.180	0.270
Somalia	Zeylac	2000-2017	-0.134	-0.082	-0.033
South Sudan	Akobo	2000	0.340	0.482	0.654
South Sudan	Akobo	2017	0.228	0.362	0.552
South Sudan	Akobo	2000-2017	-0.053	-0.007	0.041
South Sudan	Al Leiri	2000	0.336	0.497	0.702
South Sudan	Al Leiri	2017	0.241	0.396	0.611
South Sudan	Al Leiri	2000-2017	-0.045	-0.002	0.050
South Sudan	Al Mabien	2000	0.367	0.497	0.663
South Sudan	Al Mabien	2017	0.221	0.353	0.529
South Sudan	Al Mabien	2000-2017	-0.054	-0.010	0.032
South Sudan	Al Mayom	2000	0.317	0.494	0.713
South Sudan	Al Mayom	2017	0.267	0.441	0.642
South Sudan	Al Mayom	2000-2017	-0.040	0.006	0.058
South Sudan	Al Renk	2000	0.339	0.463	0.634
South Sudan	Al Renk	2017	0.262	0.393	0.567

Table 2: Diarrhea DALYs rate by unit *(continued)*

Country	Unit	year	mean rate	lower rate	upper rate
South Sudan	Al Renk	2000-2017	-0.043	0.004	0.049
South Sudan	Aliab	2000	0.333	0.483	0.657
South Sudan	Aliab	2017	0.228	0.361	0.562
South Sudan	Aliab	2000-2017	-0.058	-0.006	0.042
South Sudan	Amatonge	2000	0.373	0.544	0.749
South Sudan	Amatonge	2017	0.255	0.410	0.576
South Sudan	Amatonge	2000-2017	-0.048	-0.006	0.042
South Sudan	Aryat	2000	0.307	0.468	0.674
South Sudan	Aryat	2017	0.323	0.504	0.725
South Sudan	Aryat	2000-2017	-0.049	0.006	0.057
South Sudan	Aweil	2000	0.323	0.478	0.683
South Sudan	Aweil	2017	0.326	0.499	0.715
South Sudan	Aweil	2000-2017	-0.049	0.006	0.055
South Sudan	Ayod	2000	0.309	0.458	0.630
South Sudan	Ayod	2017	0.236	0.369	0.570
South Sudan	Ayod	2000-2017	-0.051	0.000	0.046
South Sudan	Bahr al Jabal	2000	0.311	0.451	0.609
South Sudan	Bahr al Jabal	2017	0.221	0.349	0.514
South Sudan	Bahr al Jabal	2000-2017	-0.062	-0.012	0.034
South Sudan	Baleit	2000	0.310	0.442	0.598
South Sudan	Baleit	2017	0.232	0.355	0.529
South Sudan	Baleit	2000-2017	-0.047	0.001	0.047
South Sudan	Bor	2000	0.349	0.496	0.697
South Sudan	Bor	2017	0.234	0.360	0.562
South Sudan	Bor	2000-2017	-0.059	-0.007	0.046
South Sudan	Fam al Zaraf	2000	0.301	0.437	0.600
South Sudan	Fam al Zaraf	2017	0.233	0.375	0.578
South Sudan	Fam al Zaraf	2000-2017	-0.043	0.006	0.058
South Sudan	Faring	2000	0.287	0.446	0.642
South Sudan	Faring	2017	0.244	0.415	0.650
South Sudan	Faring	2000-2017	-0.039	0.010	0.059
South Sudan	Fashooda	2000	0.301	0.414	0.558
South Sudan	Fashooda	2017	0.234	0.374	0.550
South Sudan	Fashooda	2000-2017	-0.038	0.006	0.050
South Sudan	Gogrial	2000	0.311	0.467	0.644
South Sudan	Gogrial	2017	0.267	0.435	0.656
South Sudan	Gogrial	2000-2017	-0.052	0.001	0.053
South Sudan	Kajo Kaii	2000	0.293	0.441	0.628
South Sudan	Kajo Kaii	2017	0.218	0.350	0.521
South Sudan	Kajo Kaii	2000-2017	-0.065	-0.016	0.032
South Sudan	Kapoeta	2000	0.364	0.548	0.769
South Sudan	Kapoeta	2017	0.256	0.408	0.605
South Sudan	Kapoeta	2000-2017	-0.048	-0.004	0.042
South Sudan	Magwi	2000	0.376	0.548	0.769
South Sudan	Magwi	2017	0.267	0.423	0.601
South Sudan	Magwi	2000-2017	-0.054	-0.006	0.039
South Sudan	Malek	2000	0.316	0.470	0.664
South Sudan	Malek	2017	0.293	0.475	0.730
South Sudan	Malek	2000-2017	-0.052	0.006	0.056
South Sudan	Malut	2000	0.306	0.433	0.588
South Sudan	Malut	2017	0.229	0.366	0.550
South Sudan	Malut	2000-2017	-0.042	0.003	0.051
South Sudan	Mayot	2000	0.345	0.485	0.657
South Sudan	Mayot	2017	0.244	0.380	0.544
South Sudan	Mayot	2000-2017	-0.051	-0.003	0.045
South Sudan	Meridi	2000	0.385	0.578	0.804
South Sudan	Meridi	2017	0.244	0.393	0.594
South Sudan	Meridi	2000-2017	-0.062	-0.020	0.030
South Sudan	Mundri	2000	0.383	0.564	0.766
South Sudan	Mundri	2017	0.249	0.379	0.574
South Sudan	Mundri	2000-2017	-0.063	-0.017	0.030

Table 2: Diarrhea DALYs rate by unit *(continued)*

Country	Unit	year	mean rate	lower rate	upper rate
South Sudan	Nahr Atiem	2000	0.349	0.496	0.679
South Sudan	Nahr Atiem	2017	0.240	0.375	0.560
South Sudan	Nahr Atiem	2000-2017	-0.049	-0.006	0.042
South Sudan	Nahr Lol	2000	0.293	0.459	0.662
South Sudan	Nahr Lol	2017	0.261	0.432	0.632
South Sudan	Nahr Lol	2000-2017	-0.051	0.005	0.054
South Sudan	Nahr Yei	2000	0.301	0.445	0.638
South Sudan	Nahr Yei	2017	0.208	0.323	0.477
South Sudan	Nahr Yei	2000-2017	-0.077	-0.025	0.022
South Sudan	Pibor	2000	0.376	0.524	0.711
South Sudan	Pibor	2017	0.239	0.381	0.556
South Sudan	Pibor	2000-2017	-0.049	-0.010	0.034
South Sudan	Rabkona	2000	0.329	0.501	0.709
South Sudan	Rabkona	2017	0.269	0.433	0.647
South Sudan	Rabkona	2000-2017	-0.045	0.005	0.054
South Sudan	Raja	2000	0.370	0.534	0.749
South Sudan	Raja	2017	0.330	0.486	0.737
South Sudan	Raja	2000-2017	-0.043	0.002	0.045
South Sudan	Rumbek	2000	0.324	0.481	0.651
South Sudan	Rumbek	2017	0.236	0.392	0.598
South Sudan	Rumbek	2000-2017	-0.043	-0.002	0.049
South Sudan	Shobet	2000	0.314	0.473	0.657
South Sudan	Shobet	2017	0.231	0.397	0.610
South Sudan	Shobet	2000-2017	-0.048	-0.003	0.044
South Sudan	Shokodom	2000	0.359	0.546	0.779
South Sudan	Shokodom	2017	0.252	0.405	0.591
South Sudan	Shokodom	2000-2017	-0.053	-0.007	0.037
South Sudan	Sobat	2000	0.331	0.467	0.641
South Sudan	Sobat	2017	0.225	0.356	0.511
South Sudan	Sobat	2000-2017	-0.052	-0.004	0.045
South Sudan	Terkaka	2000	0.304	0.434	0.594
South Sudan	Terkaka	2017	0.220	0.334	0.517
South Sudan	Terkaka	2000-2017	-0.061	-0.010	0.035
South Sudan	Tombura	2000	0.387	0.569	0.810
South Sudan	Tombura	2017	0.260	0.425	0.622
South Sudan	Tombura	2000-2017	-0.045	-0.002	0.040
South Sudan	Tonga	2000	0.282	0.411	0.568
South Sudan	Tonga	2017	0.224	0.360	0.550
South Sudan	Tonga	2000-2017	-0.037	0.009	0.060
South Sudan	Tonj	2000	0.326	0.473	0.643
South Sudan	Tonj	2017	0.244	0.405	0.628
South Sudan	Tonj	2000-2017	-0.049	-0.001	0.050
South Sudan	Wanjuk	2000	0.310	0.466	0.654
South Sudan	Wanjuk	2017	0.311	0.498	0.747
South Sudan	Wanjuk	2000-2017	-0.049	0.007	0.057
South Sudan	Warab	2000	0.323	0.471	0.651
South Sudan	Warab	2017	0.252	0.417	0.642
South Sudan	Warab	2000-2017	-0.052	-0.001	0.050
South Sudan	Wat	2000	0.330	0.473	0.648
South Sudan	Wat	2017	0.233	0.365	0.551
South Sudan	Wat	2000-2017	-0.052	-0.003	0.043
South Sudan	Wau	2000	0.366	0.537	0.729
South Sudan	Wau	2017	0.274	0.439	0.700
South Sudan	Wau	2000-2017	-0.055	-0.007	0.037
South Sudan	Yambio	2000	0.396	0.588	0.874
South Sudan	Yambio	2017	0.237	0.407	0.594
South Sudan	Yambio	2000-2017	-0.058	-0.012	0.030
South Sudan	Yerol	2000	0.328	0.484	0.636
South Sudan	Yerol	2017	0.245	0.381	0.578
South Sudan	Yerol	2000-2017	-0.054	-0.004	0.046
Swaziland	Dvokodvweni	2000	0.257	0.340	0.448

Table 2: Diarrhea DALYs rate by unit *(continued)*

Country	Unit	year	mean rate	lower rate	upper rate
Swaziland	Dvokodvweni	2017	0.116	0.158	0.207
Swaziland	Dvokodvweni	2000-2017	-0.059	-0.029	0.003
Swaziland	Ekukhanyeni	2000	0.231	0.306	0.403
Swaziland	Ekukhanyeni	2017	0.107	0.149	0.198
Swaziland	Ekukhanyeni	2000-2017	-0.055	-0.023	0.009
Swaziland	Gege	2000	0.211	0.295	0.395
Swaziland	Gege	2017	0.116	0.156	0.209
Swaziland	Gege	2000-2017	-0.051	-0.018	0.016
Swaziland	Hhukwini	2000	0.219	0.292	0.375
Swaziland	Hhukwini	2017	0.107	0.146	0.188
Swaziland	Hhukwini	2000-2017	-0.050	-0.020	0.010
Swaziland	Hlane	2000	0.263	0.343	0.451
Swaziland	Hlane	2017	0.116	0.157	0.205
Swaziland	Hlane	2000-2017	-0.062	-0.031	0.000
Swaziland	Hosea	2000	0.239	0.318	0.422
Swaziland	Hosea	2017	0.119	0.161	0.212
Swaziland	Hosea	2000-2017	-0.050	-0.019	0.014
Swaziland	Kubuta	2000	0.245	0.329	0.436
Swaziland	Kubuta	2017	0.119	0.167	0.219
Swaziland	Kubuta	2000-2017	-0.051	-0.020	0.014
Swaziland	Kwaluseni	2000	0.224	0.305	0.400
Swaziland	Kwaluseni	2017	0.111	0.155	0.202
Swaziland	Kwaluseni	2000-2017	-0.053	-0.019	0.014
Swaziland	Lamgabhi	2000	0.208	0.286	0.375
Swaziland	Lamgabhi	2017	0.108	0.151	0.195
Swaziland	Lamgabhi	2000-2017	-0.053	-0.020	0.014
Swaziland	Lobamba	2000	0.222	0.299	0.389
Swaziland	Lobamba	2017	0.109	0.152	0.196
Swaziland	Lobamba	2000-2017	-0.052	-0.020	0.011
Swaziland	Lobamba Lomdzala	2000	0.228	0.303	0.397
Swaziland	Lobamba Lomdzala	2017	0.110	0.155	0.200
Swaziland	Lobamba Lomdzala	2000-2017	-0.055	-0.022	0.010
Swaziland	Lomahasha	2000	0.262	0.339	0.438
Swaziland	Lomahasha	2017	0.112	0.155	0.203
Swaziland	Lomahasha	2000-2017	-0.057	-0.030	0.002
Swaziland	Lubuli	2000	0.251	0.335	0.436
Swaziland	Lubuli	2017	0.117	0.162	0.213
Swaziland	Lubuli	2000-2017	-0.053	-0.022	0.011
Swaziland	Ludzeludze	2000	0.226	0.299	0.390
Swaziland	Ludzeludze	2017	0.109	0.151	0.197
Swaziland	Ludzeludze	2000-2017	-0.054	-0.021	0.013
Swaziland	Lugongolweni	2000	0.265	0.354	0.453
Swaziland	Lugongolweni	2017	0.118	0.162	0.209
Swaziland	Lugongolweni	2000-2017	-0.060	-0.032	0.000
Swaziland	Madlangempisi	2000	0.236	0.312	0.407
Swaziland	Madlangempisi	2017	0.108	0.145	0.190
Swaziland	Madlangempisi	2000-2017	-0.054	-0.026	0.007
Swaziland	Mafutseni	2000	0.231	0.311	0.413
Swaziland	Mafutseni	2017	0.106	0.148	0.198
Swaziland	Mafutseni	2000-2017	-0.058	-0.025	0.009
Swaziland	Mahlangatja	2000	0.220	0.299	0.396
Swaziland	Mahlangatja	2017	0.105	0.151	0.200
Swaziland	Mahlangatja	2000-2017	-0.054	-0.020	0.013
Swaziland	Mangcongco	2000	0.192	0.272	0.364
Swaziland	Mangcongco	2017	0.100	0.137	0.180
Swaziland	Mangcongco	2000-2017	-0.051	-0.021	0.011
Swaziland	Manzini North	2000	0.225	0.306	0.402
Swaziland	Manzini North	2017	0.109	0.152	0.202
Swaziland	Manzini North	2000-2017	-0.054	-0.021	0.013
Swaziland	Manzini South	2000	0.226	0.308	0.407
Swaziland	Manzini South	2017	0.110	0.155	0.203

Table 2: Diarrhea DALYs rate by unit *(continued)*

Country	Unit	year	mean rate	lower rate	upper rate
Swaziland	Manzini South	2000-2017	-0.055	-0.021	0.013
Swaziland	Maseyisini	2000	0.211	0.296	0.399
Swaziland	Maseyisini	2017	0.115	0.157	0.212
Swaziland	Maseyisini	2000-2017	-0.051	-0.019	0.016
Swaziland	Matsanjeni North	2000	0.271	0.356	0.452
Swaziland	Matsanjeni North	2017	0.117	0.160	0.212
Swaziland	Matsanjeni North	2000-2017	-0.062	-0.032	0.000
Swaziland	Matsanjeni South	2000	0.235	0.312	0.409
Swaziland	Matsanjeni South	2017	0.118	0.159	0.210
Swaziland	Matsanjeni South	2000-2017	-0.050	-0.019	0.012
Swaziland	Mayiwane	2000	0.219	0.293	0.387
Swaziland	Mayiwane	2017	0.102	0.138	0.181
Swaziland	Mayiwane	2000-2017	-0.053	-0.023	0.011
Swaziland	Mbabane East	2000	0.206	0.279	0.363
Swaziland	Mbabane East	2017	0.107	0.145	0.184
Swaziland	Mbabane East	2000-2017	-0.048	-0.018	0.014
Swaziland	Mbabane West	2000	0.203	0.278	0.361
Swaziland	Mbabane West	2017	0.106	0.145	0.185
Swaziland	Mbabane West	2000-2017	-0.050	-0.018	0.013
Swaziland	Mbangweni	2000	0.218	0.300	0.401
Swaziland	Mbangweni	2017	0.117	0.161	0.214
Swaziland	Mbangweni	2000-2017	-0.050	-0.018	0.018
Swaziland	Mhlambanyatsi	2000	0.197	0.275	0.368
Swaziland	Mhlambanyatsi	2017	0.105	0.144	0.187
Swaziland	Mhlambanyatsi	2000-2017	-0.049	-0.019	0.015
Swaziland	Mhlangatane	2000	0.231	0.303	0.399
Swaziland	Mhlangatane	2017	0.107	0.143	0.188
Swaziland	Mhlangatane	2000-2017	-0.053	-0.024	0.009
Swaziland	Mhlume	2000	0.255	0.327	0.423
Swaziland	Mhlume	2017	0.117	0.157	0.202
Swaziland	Mhlume	2000-2017	-0.056	-0.029	0.002
Swaziland	Mkhiweni	2000	0.236	0.311	0.407
Swaziland	Mkhiweni	2017	0.105	0.146	0.189
Swaziland	Mkhiweni	2000-2017	-0.056	-0.026	0.005
Swaziland	Motjane	2000	0.197	0.273	0.363
Swaziland	Motjane	2017	0.102	0.138	0.179
Swaziland	Motjane	2000-2017	-0.051	-0.020	0.012
Swaziland	Mphalaleni	2000	0.229	0.305	0.390
Swaziland	Mphalaleni	2017	0.105	0.146	0.192
Swaziland	Mphalaleni	2000-2017	-0.052	-0.024	0.008
Swaziland	Mpholonjeni	2000	0.267	0.352	0.455
Swaziland	Mpholonjeni	2017	0.117	0.162	0.210
Swaziland	Mpholonjeni	2000-2017	-0.060	-0.030	0.002
Swaziland	Mthongwaneni	2000	0.232	0.311	0.412
Swaziland	Mthongwaneni	2017	0.106	0.150	0.199
Swaziland	Mthongwaneni	2000-2017	-0.058	-0.023	0.011
Swaziland	Mtsambama	2000	0.232	0.313	0.415
Swaziland	Mtsambama	2017	0.116	0.164	0.216
Swaziland	Mtsambama	2000-2017	-0.048	-0.017	0.017
Swaziland	Ndzingeni	2000	0.222	0.300	0.388
Swaziland	Ndzingeni	2017	0.105	0.142	0.186
Swaziland	Ndzingeni	2000-2017	-0.053	-0.024	0.009
Swaziland	Ngudzeni	2000	0.242	0.325	0.431
Swaziland	Ngudzeni	2017	0.122	0.166	0.216
Swaziland	Ngudzeni	2000-2017	-0.048	-0.018	0.016
Swaziland	Ngwenpisi	2000	0.203	0.283	0.375
Swaziland	Ngwenpisi	2017	0.106	0.148	0.197
Swaziland	Ngwenpisi	2000-2017	-0.052	-0.019	0.016
Swaziland	Nhlambeni	2000	0.230	0.307	0.405
Swaziland	Nhlambeni	2017	0.109	0.152	0.201
Swaziland	Nhlambeni	2000-2017	-0.057	-0.022	0.011

Table 2: Diarrhea DALYs rate by unit *(continued)*

Country	Unit	year	mean rate	lower rate	upper rate
Swaziland	Nkhaba	2000	0.208	0.278	0.366
Swaziland	Nkhaba	2017	0.102	0.140	0.179
Swaziland	Nkhaba	2000-2017	-0.049	-0.020	0.011
Swaziland	Nkilongo	2000	0.257	0.340	0.431
Swaziland	Nkilongo	2017	0.116	0.162	0.213
Swaziland	Nkilongo	2000-2017	-0.054	-0.025	0.007
Swaziland	Nkwene	2000	0.225	0.309	0.411
Swaziland	Nkwene	2017	0.112	0.160	0.208
Swaziland	Nkwene	2000-2017	-0.052	-0.020	0.015
Swaziland	Ntfonjeni	2000	0.216	0.285	0.371
Swaziland	Ntfonjeni	2017	0.102	0.139	0.181
Swaziland	Ntfonjeni	2000-2017	-0.050	-0.022	0.011
Swaziland	Ntondozi	2000	0.226	0.301	0.397
Swaziland	Ntondozi	2017	0.110	0.151	0.199
Swaziland	Ntondozi	2000-2017	-0.056	-0.021	0.012
Swaziland	Pigg's Peak	2000	0.215	0.282	0.364
Swaziland	Pigg's Peak	2017	0.100	0.138	0.179
Swaziland	Pigg's Peak	2000-2017	-0.052	-0.024	0.010
Swaziland	Sandleni	2000	0.237	0.316	0.421
Swaziland	Sandleni	2017	0.117	0.164	0.217
Swaziland	Sandleni	2000-2017	-0.048	-0.018	0.016
Swaziland	Shiselweni	2000	0.230	0.309	0.411
Swaziland	Shiselweni	2017	0.119	0.161	0.211
Swaziland	Shiselweni	2000-2017	-0.048	-0.017	0.015
Swaziland	Sigwe	2000	0.241	0.319	0.415
Swaziland	Sigwe	2017	0.118	0.160	0.210
Swaziland	Sigwe	2000-2017	-0.052	-0.021	0.012
Swaziland	Siphofaneni	2000	0.254	0.339	0.446
Swaziland	Siphofaneni	2017	0.116	0.162	0.216
Swaziland	Siphofaneni	2000-2017	-0.056	-0.024	0.007
Swaziland	Sithobela	2000	0.251	0.331	0.432
Swaziland	Sithobela	2017	0.115	0.162	0.212
Swaziland	Sithobela	2000-2017	-0.053	-0.022	0.011
Swaziland	Somntongo	2000	0.224	0.310	0.406
Swaziland	Somntongo	2017	0.113	0.154	0.205
Swaziland	Somntongo	2000-2017	-0.052	-0.021	0.009
Swaziland	Timpisini	2000	0.222	0.290	0.385
Swaziland	Timpisini	2017	0.102	0.139	0.181
Swaziland	Timpisini	2000-2017	-0.052	-0.023	0.011
Swaziland	Zombodze	2000	0.219	0.302	0.408
Swaziland	Zombodze	2017	0.117	0.160	0.211
Swaziland	Zombodze	2000-2017	-0.049	-0.018	0.016
Tanzania	Arusha	2000	0.130	0.184	0.259
Tanzania	Arusha	2017	0.032	0.045	0.061
Tanzania	Arusha	2000-2017	-0.125	-0.087	-0.050
Tanzania	Arusha Urban	2000	0.132	0.187	0.262
Tanzania	Arusha Urban	2017	0.033	0.047	0.062
Tanzania	Arusha Urban	2000-2017	-0.125	-0.088	-0.051
Tanzania	Babati	2000	0.126	0.180	0.240
Tanzania	Babati	2017	0.031	0.044	0.057
Tanzania	Babati	2000-2017	-0.119	-0.084	-0.048
Tanzania	Babati Urban	2000	0.126	0.181	0.245
Tanzania	Babati Urban	2017	0.031	0.045	0.060
Tanzania	Babati Urban	2000-2017	-0.118	-0.082	-0.044
Tanzania	Bagamoyo	2000	0.123	0.165	0.222
Tanzania	Bagamoyo	2017	0.033	0.044	0.058
Tanzania	Bagamoyo	2000-2017	-0.116	-0.084	-0.053
Tanzania	Bahi	2000	0.131	0.185	0.248
Tanzania	Bahi	2017	0.034	0.047	0.065
Tanzania	Bahi	2000-2017	-0.120	-0.084	-0.047
Tanzania	Bariadi	2000	0.162	0.220	0.283

Table 2: Diarrhea DALYs rate by unit *(continued)*

Country	Unit	year	mean rate	lower rate	upper rate
Tanzania	Bariadi	2017	0.042	0.055	0.073
Tanzania	Bariadi	2000-2017	-0.117	-0.084	-0.053
Tanzania	Biharamulo	2000	0.159	0.210	0.278
Tanzania	Biharamulo	2017	0.041	0.055	0.072
Tanzania	Biharamulo	2000-2017	-0.105	-0.079	-0.051
Tanzania	Buhigwe	2000	0.169	0.234	0.309
Tanzania	Buhigwe	2017	0.049	0.066	0.086
Tanzania	Buhigwe	2000-2017	-0.103	-0.073	-0.045
Tanzania	Bukoba Rural	2000	0.136	0.182	0.243
Tanzania	Bukoba Rural	2017	0.039	0.053	0.070
Tanzania	Bukoba Rural	2000-2017	-0.106	-0.075	-0.044
Tanzania	Bukoba Urban	2000	0.131	0.180	0.242
Tanzania	Bukoba Urban	2017	0.040	0.055	0.074
Tanzania	Bukoba Urban	2000-2017	-0.104	-0.070	-0.040
Tanzania	Bukombe	2000	0.165	0.224	0.293
Tanzania	Bukombe	2017	0.040	0.054	0.070
Tanzania	Bukombe	2000-2017	-0.117	-0.089	-0.060
Tanzania	Bunda	2000	0.160	0.209	0.272
Tanzania	Bunda	2017	0.045	0.059	0.078
Tanzania	Bunda	2000-2017	-0.115	-0.081	-0.050
Tanzania	Busega	2000	0.145	0.196	0.255
Tanzania	Busega	2017	0.041	0.055	0.074
Tanzania	Busega	2000-2017	-0.115	-0.081	-0.051
Tanzania	Butiama	2000	0.164	0.219	0.282
Tanzania	Butiama	2017	0.047	0.061	0.081
Tanzania	Butiama	2000-2017	-0.111	-0.082	-0.050
Tanzania	Chake	2000	0.160	0.235	0.307
Tanzania	Chake	2017	0.042	0.056	0.074
Tanzania	Chake	2000-2017	-0.123	-0.089	-0.055
Tanzania	Chamwino	2000	0.127	0.183	0.241
Tanzania	Chamwino	2017	0.034	0.046	0.064
Tanzania	Chamwino	2000-2017	-0.119	-0.083	-0.049
Tanzania	Chato	2000	0.147	0.198	0.270
Tanzania	Chato	2017	0.041	0.054	0.071
Tanzania	Chato	2000-2017	-0.106	-0.078	-0.051
Tanzania	Chemba	2000	0.127	0.184	0.250
Tanzania	Chemba	2017	0.034	0.046	0.061
Tanzania	Chemba	2000-2017	-0.117	-0.083	-0.048
Tanzania	Chunya	2000	0.132	0.189	0.264
Tanzania	Chunya	2017	0.035	0.049	0.067
Tanzania	Chunya	2000-2017	-0.117	-0.084	-0.054
Tanzania	Dodoma Urban	2000	0.122	0.176	0.239
Tanzania	Dodoma Urban	2017	0.032	0.044	0.060
Tanzania	Dodoma Urban	2000-2017	-0.121	-0.085	-0.048
Tanzania	Gairo	2000	0.129	0.183	0.245
Tanzania	Gairo	2017	0.034	0.048	0.064
Tanzania	Gairo	2000-2017	-0.105	-0.075	-0.044
Tanzania	Geita	2000	0.133	0.179	0.243
Tanzania	Geita	2017	0.038	0.051	0.066
Tanzania	Geita	2000-2017	-0.108	-0.076	-0.046
Tanzania	Hai	2000	0.130	0.181	0.251
Tanzania	Hai	2017	0.035	0.049	0.066
Tanzania	Hai	2000-2017	-0.120	-0.083	-0.048
Tanzania	Hanang	2000	0.124	0.181	0.235
Tanzania	Hanang	2017	0.031	0.043	0.058
Tanzania	Hanang	2000-2017	-0.121	-0.086	-0.050
Tanzania	Handeni	2000	0.141	0.207	0.273
Tanzania	Handeni	2017	0.037	0.051	0.068
Tanzania	Handeni	2000-2017	-0.117	-0.085	-0.053
Tanzania	Handeni Township Authority	2000	0.133	0.205	0.284
Tanzania	Handeni Township Authority	2017	0.037	0.052	0.070

Table 2: Diarrhea DALYs rate by unit *(continued)*

Country	Unit	year	mean rate	lower rate	upper rate
Tanzania	Handeni Township Authority	2000-2017	-0.110	-0.080	-0.051
Tanzania	Igunga	2000	0.157	0.219	0.291
Tanzania	Igunga	2017	0.033	0.044	0.063
Tanzania	Igunga	2000-2017	-0.120	-0.090	-0.059
Tanzania	Ikungi	2000	0.135	0.188	0.248
Tanzania	Ikungi	2017	0.031	0.043	0.060
Tanzania	Ikungi	2000-2017	-0.121	-0.086	-0.052
Tanzania	Ilala	2000	0.120	0.161	0.217
Tanzania	Ilala	2017	0.032	0.043	0.057
Tanzania	Ilala	2000-2017	-0.119	-0.087	-0.052
Tanzania	Ileje	2000	0.134	0.178	0.241
Tanzania	Ileje	2017	0.033	0.045	0.058
Tanzania	Ileje	2000-2017	-0.126	-0.092	-0.062
Tanzania	Ilemela	2000	0.116	0.158	0.213
Tanzania	Ilemela	2017	0.036	0.049	0.064
Tanzania	Ilemela	2000-2017	-0.107	-0.074	-0.041
Tanzania	Iramba	2000	0.132	0.189	0.248
Tanzania	Iramba	2017	0.031	0.043	0.060
Tanzania	Iramba	2000-2017	-0.113	-0.082	-0.048
Tanzania	Iringa Rural	2000	0.132	0.185	0.253
Tanzania	Iringa Rural	2017	0.032	0.045	0.061
Tanzania	Iringa Rural	2000-2017	-0.119	-0.086	-0.051
Tanzania	Iringa Urban	2000	0.135	0.192	0.262
Tanzania	Iringa Urban	2017	0.032	0.046	0.063
Tanzania	Iringa Urban	2000-2017	-0.121	-0.085	-0.050
Tanzania	Itilima	2000	0.163	0.219	0.279
Tanzania	Itilima	2017	0.039	0.052	0.070
Tanzania	Itilima	2000-2017	-0.118	-0.085	-0.053
Tanzania	Kahama	2000	0.150	0.200	0.258
Tanzania	Kahama	2017	0.035	0.047	0.061
Tanzania	Kahama	2000-2017	-0.122	-0.092	-0.063
Tanzania	Kahama Township Authority	2000	0.155	0.210	0.276
Tanzania	Kahama Township Authority	2017	0.033	0.045	0.059
Tanzania	Kahama Township Authority	2000-2017	-0.129	-0.098	-0.068
Tanzania	Kakonko	2000	0.170	0.222	0.291
Tanzania	Kakonko	2017	0.043	0.059	0.077
Tanzania	Kakonko	2000-2017	-0.106	-0.077	-0.048
Tanzania	Kalambo	2000	0.157	0.222	0.298
Tanzania	Kalambo	2017	0.045	0.062	0.083
Tanzania	Kalambo	2000-2017	-0.104	-0.073	-0.045
Tanzania	Kaliua	2000	0.161	0.223	0.290
Tanzania	Kaliua	2017	0.039	0.054	0.073
Tanzania	Kaliua	2000-2017	-0.117	-0.087	-0.058
Tanzania	Karagwe	2000	0.152	0.198	0.255
Tanzania	Karagwe	2017	0.042	0.055	0.072
Tanzania	Karagwe	2000-2017	-0.110	-0.078	-0.049
Tanzania	Karatu	2000	0.131	0.182	0.250
Tanzania	Karatu	2017	0.030	0.044	0.060
Tanzania	Karatu	2000-2017	-0.120	-0.084	-0.050
Tanzania	Kaskazini 'A'	2000	0.136	0.186	0.249
Tanzania	Kaskazini 'A'	2017	0.037	0.050	0.064
Tanzania	Kaskazini 'A'	2000-2017	-0.108	-0.077	-0.046
Tanzania	Kaskazini 'B'	2000	0.124	0.172	0.231
Tanzania	Kaskazini 'B'	2017	0.034	0.046	0.060
Tanzania	Kaskazini 'B'	2000-2017	-0.112	-0.081	-0.046
Tanzania	Kasulu	2000	0.169	0.236	0.311
Tanzania	Kasulu	2017	0.049	0.066	0.088
Tanzania	Kasulu	2000-2017	-0.098	-0.070	-0.040
Tanzania	Kasulu Township Authority	2000	0.163	0.235	0.315
Tanzania	Kasulu Township Authority	2017	0.049	0.066	0.088
Tanzania	Kasulu Township Authority	2000-2017	-0.102	-0.071	-0.040

Table 2: Diarrhea DALYs rate by unit *(continued)*

Country	Unit	year	mean rate	lower rate	upper rate
Tanzania	Kati	2000	0.124	0.170	0.231
Tanzania	Kati	2017	0.034	0.046	0.058
Tanzania	Kati	2000-2017	-0.115	-0.084	-0.050
Tanzania	Kibaha	2000	0.118	0.159	0.216
Tanzania	Kibaha	2017	0.030	0.042	0.054
Tanzania	Kibaha	2000-2017	-0.118	-0.086	-0.052
Tanzania	Kibaha Urban	2000	0.119	0.161	0.219
Tanzania	Kibaha Urban	2017	0.031	0.042	0.055
Tanzania	Kibaha Urban	2000-2017	-0.119	-0.087	-0.053
Tanzania	Kibondo	2000	0.170	0.228	0.301
Tanzania	Kibondo	2017	0.045	0.062	0.083
Tanzania	Kibondo	2000-2017	-0.100	-0.072	-0.045
Tanzania	Kigoma Rural	2000	0.169	0.233	0.322
Tanzania	Kigoma Rural	2017	0.049	0.065	0.086
Tanzania	Kigoma Rural	2000-2017	-0.109	-0.078	-0.049
Tanzania	Kigoma Urban	2000	0.180	0.247	0.341
Tanzania	Kigoma Urban	2017	0.055	0.073	0.096
Tanzania	Kigoma Urban	2000-2017	-0.106	-0.076	-0.046
Tanzania	Kilindi	2000	0.142	0.205	0.268
Tanzania	Kilindi	2017	0.037	0.052	0.068
Tanzania	Kilindi	2000-2017	-0.109	-0.077	-0.047
Tanzania	Kilolo	2000	0.140	0.192	0.256
Tanzania	Kilolo	2017	0.034	0.048	0.066
Tanzania	Kilolo	2000-2017	-0.116	-0.085	-0.053
Tanzania	Kilombero	2000	0.127	0.174	0.227
Tanzania	Kilombero	2017	0.032	0.044	0.060
Tanzania	Kilombero	2000-2017	-0.118	-0.089	-0.061
Tanzania	Kilosa	2000	0.134	0.186	0.236
Tanzania	Kilosa	2017	0.034	0.049	0.066
Tanzania	Kilosa	2000-2017	-0.113	-0.081	-0.051
Tanzania	Kilwa	2000	0.147	0.215	0.293
Tanzania	Kilwa	2017	0.040	0.054	0.072
Tanzania	Kilwa	2000-2017	-0.118	-0.087	-0.058
Tanzania	Kinondoni	2000	0.117	0.158	0.214
Tanzania	Kinondoni	2017	0.032	0.042	0.054
Tanzania	Kinondoni	2000-2017	-0.118	-0.087	-0.053
Tanzania	Kisarawe	2000	0.120	0.166	0.222
Tanzania	Kisarawe	2017	0.032	0.044	0.057
Tanzania	Kisarawe	2000-2017	-0.118	-0.087	-0.053
Tanzania	Kishapu	2000	0.142	0.195	0.255
Tanzania	Kishapu	2017	0.032	0.043	0.057
Tanzania	Kishapu	2000-2017	-0.126	-0.094	-0.061
Tanzania	Kiteto	2000	0.128	0.180	0.253
Tanzania	Kiteto	2017	0.033	0.045	0.059
Tanzania	Kiteto	2000-2017	-0.106	-0.076	-0.045
Tanzania	Kondoa	2000	0.125	0.181	0.243
Tanzania	Kondoa	2017	0.032	0.045	0.061
Tanzania	Kondoa	2000-2017	-0.119	-0.085	-0.049
Tanzania	Kongwa	2000	0.129	0.187	0.255
Tanzania	Kongwa	2017	0.033	0.049	0.068
Tanzania	Kongwa	2000-2017	-0.113	-0.078	-0.048
Tanzania	Korogwe	2000	0.124	0.181	0.241
Tanzania	Korogwe	2017	0.033	0.046	0.060
Tanzania	Korogwe	2000-2017	-0.117	-0.087	-0.054
Tanzania	Korogwe Township Authority	2000	0.128	0.186	0.248
Tanzania	Korogwe Township Authority	2017	0.034	0.048	0.064
Tanzania	Korogwe Township Authority	2000-2017	-0.119	-0.086	-0.051
Tanzania	Kusini	2000	0.111	0.159	0.216
Tanzania	Kusini	2017	0.030	0.042	0.055
Tanzania	Kusini	2000-2017	-0.114	-0.086	-0.053
Tanzania	Kwimba	2000	0.129	0.174	0.228

Table 2: Diarrhea DALYs rate by unit *(continued)*

Country	Unit	year	mean rate	lower rate	upper rate
Tanzania	Kwimba	2017	0.036	0.048	0.063
Tanzania	Kwimba	2000-2017	-0.113	-0.081	-0.050
Tanzania	Kyela	2000	0.124	0.164	0.225
Tanzania	Kyela	2017	0.029	0.040	0.052
Tanzania	Kyela	2000-2017	-0.129	-0.097	-0.068
Tanzania	Kyerwa	2000	0.161	0.203	0.254
Tanzania	Kyerwa	2017	0.042	0.057	0.073
Tanzania	Kyerwa	2000-2017	-0.116	-0.082	-0.048
Tanzania	Lake Eyasi	2000	0.134	0.187	0.246
Tanzania	Lake Eyasi	2000	0.130	0.183	0.247
Tanzania	Lake Eyasi	2017	0.031	0.044	0.062
Tanzania	Lake Eyasi	2017	0.030	0.044	0.060
Tanzania	Lake Eyasi	2000-2017	-0.119	-0.085	-0.051
Tanzania	Lake Eyasi	2000-2017	-0.119	-0.087	-0.050
Tanzania	Lake Manyara	2000	0.131	0.185	0.253
Tanzania	Lake Manyara	2000	0.133	0.186	0.260
Tanzania	Lake Manyara	2017	0.030	0.044	0.059
Tanzania	Lake Manyara	2017	0.030	0.044	0.060
Tanzania	Lake Manyara	2000-2017	-0.123	-0.087	-0.053
Tanzania	Lake Manyara	2000-2017	-0.123	-0.085	-0.051
Tanzania	Lake Rukwa	2000	0.155	0.224	0.316
Tanzania	Lake Rukwa	2000	0.151	0.219	0.309
Tanzania	Lake Rukwa	2000	0.139	0.201	0.287
Tanzania	Lake Rukwa	2017	0.043	0.062	0.085
Tanzania	Lake Rukwa	2017	0.043	0.061	0.085
Tanzania	Lake Rukwa	2017	0.038	0.054	0.077
Tanzania	Lake Rukwa	2000-2017	-0.101	-0.068	-0.035
Tanzania	Lake Rukwa	2000-2017	-0.109	-0.075	-0.043
Tanzania	Lake Rukwa	2000-2017	-0.113	-0.078	-0.046
Tanzania	Lake Tanganyika	2000	0.145	0.213	0.292
Tanzania	Lake Tanganyika	2000	0.170	0.233	0.309
Tanzania	Lake Tanganyika	2000	0.176	0.234	0.307
Tanzania	Lake Tanganyika	2017	0.038	0.053	0.073
Tanzania	Lake Tanganyika	2017	0.043	0.060	0.079
Tanzania	Lake Tanganyika	2017	0.048	0.064	0.083
Tanzania	Lake Tanganyika	2000-2017	-0.112	-0.081	-0.046
Tanzania	Lake Tanganyika	2000-2017	-0.107	-0.077	-0.048
Tanzania	Lake Tanganyika	2000-2017	-0.105	-0.078	-0.048
Tanzania	Lake Victoria	2000	0.141	0.189	0.247
Tanzania	Lake Victoria	2000	0.135	0.177	0.241
Tanzania	Lake Victoria	2000	0.166	0.217	0.281
Tanzania	Lake Victoria	2000	0.128	0.172	0.231
Tanzania	Lake Victoria	2017	0.041	0.055	0.074
Tanzania	Lake Victoria	2017	0.039	0.053	0.069
Tanzania	Lake Victoria	2017	0.048	0.063	0.083
Tanzania	Lake Victoria	2017	0.039	0.052	0.066
Tanzania	Lake Victoria	2000-2017	-0.108	-0.078	-0.047
Tanzania	Lake Victoria	2000-2017	-0.100	-0.072	-0.042
Tanzania	Lake Victoria	2000-2017	-0.112	-0.079	-0.050
Tanzania	Lake Victoria	2000-2017	-0.104	-0.071	-0.041
Tanzania	Lindi Rural	2000	0.152	0.213	0.295
Tanzania	Lindi Rural	2017	0.035	0.049	0.065
Tanzania	Lindi Rural	2000-2017	-0.130	-0.096	-0.058
Tanzania	Lindi Urban	2000	0.143	0.206	0.297
Tanzania	Lindi Urban	2017	0.033	0.047	0.063
Tanzania	Lindi Urban	2000-2017	-0.135	-0.100	-0.062
Tanzania	Liwale	2000	0.149	0.211	0.282
Tanzania	Liwale	2017	0.038	0.052	0.072
Tanzania	Liwale	2000-2017	-0.123	-0.091	-0.062
Tanzania	Longido	2000	0.159	0.221	0.302
Tanzania	Longido	2017	0.039	0.053	0.069

Table 2: Diarrhea DALYs rate by unit *(continued)*

Country	Unit	year	mean rate	lower rate	upper rate
Tanzania	Longido	2000-2017	-0.117	-0.084	-0.048
Tanzania	Ludewa	2000	0.125	0.164	0.215
Tanzania	Ludewa	2017	0.032	0.044	0.058
Tanzania	Ludewa	2000-2017	-0.114	-0.086	-0.056
Tanzania	Lushoto	2000	0.140	0.196	0.264
Tanzania	Lushoto	2017	0.036	0.050	0.066
Tanzania	Lushoto	2000-2017	-0.116	-0.084	-0.049
Tanzania	Mafia	2000	0.106	0.153	0.203
Tanzania	Mafia	2017	0.029	0.041	0.057
Tanzania	Mafia	2000-2017	-0.121	-0.089	-0.055
Tanzania	Mafinga Township Authority	2000	0.131	0.198	0.270
Tanzania	Mafinga Township Authority	2017	0.034	0.048	0.067
Tanzania	Mafinga Township Authority	2000-2017	-0.117	-0.084	-0.053
Tanzania	Magharibi	2000	0.131	0.178	0.242
Tanzania	Magharibi	2017	0.035	0.048	0.061
Tanzania	Magharibi	2000-2017	-0.113	-0.082	-0.048
Tanzania	Magu	2000	0.135	0.177	0.237
Tanzania	Magu	2017	0.040	0.052	0.068
Tanzania	Magu	2000-2017	-0.110	-0.077	-0.044
Tanzania	Makambako Township Authority	2000	0.128	0.180	0.255
Tanzania	Makambako Township Authority	2017	0.032	0.046	0.064
Tanzania	Makambako Township Authority	2000-2017	-0.119	-0.086	-0.057
Tanzania	Makete	2000	0.124	0.165	0.233
Tanzania	Makete	2017	0.029	0.041	0.054
Tanzania	Makete	2000-2017	-0.125	-0.096	-0.063
Tanzania	Manyoni	2000	0.133	0.187	0.252
Tanzania	Manyoni	2017	0.033	0.047	0.064
Tanzania	Manyoni	2000-2017	-0.121	-0.082	-0.048
Tanzania	Masasi	2000	0.154	0.220	0.295
Tanzania	Masasi	2017	0.037	0.051	0.070
Tanzania	Masasi	2000-2017	-0.125	-0.092	-0.056
Tanzania	Masasi Township Authority	2000	0.148	0.215	0.293
Tanzania	Masasi Township Authority	2017	0.036	0.051	0.070
Tanzania	Masasi Township Authority	2000-2017	-0.130	-0.092	-0.056
Tanzania	Maswa	2000	0.149	0.199	0.257
Tanzania	Maswa	2017	0.035	0.046	0.060
Tanzania	Maswa	2000-2017	-0.124	-0.091	-0.059
Tanzania	Mbarali	2000	0.130	0.180	0.247
Tanzania	Mbarali	2017	0.032	0.045	0.059
Tanzania	Mbarali	2000-2017	-0.121	-0.089	-0.061
Tanzania	Mbeya Rural	2000	0.127	0.172	0.237
Tanzania	Mbeya Rural	2017	0.031	0.043	0.057
Tanzania	Mbeya Rural	2000-2017	-0.125	-0.091	-0.061
Tanzania	Mbeya Urban	2000	0.125	0.173	0.243
Tanzania	Mbeya Urban	2017	0.031	0.044	0.058
Tanzania	Mbeya Urban	2000-2017	-0.123	-0.089	-0.058
Tanzania	Mbinga	2000	0.110	0.156	0.207
Tanzania	Mbinga	2017	0.029	0.043	0.059
Tanzania	Mbinga	2000-2017	-0.114	-0.086	-0.055
Tanzania	Mbogwe	2000	0.153	0.208	0.272
Tanzania	Mbogwe	2017	0.037	0.050	0.066
Tanzania	Mbogwe	2000-2017	-0.121	-0.089	-0.061
Tanzania	Mbozi	2000	0.149	0.203	0.276
Tanzania	Mbozi	2017	0.038	0.053	0.071
Tanzania	Mbozi	2000-2017	-0.120	-0.084	-0.054
Tanzania	Mbulu	2000	0.121	0.179	0.237
Tanzania	Mbulu	2017	0.031	0.044	0.058
Tanzania	Mbulu	2000-2017	-0.120	-0.084	-0.046
Tanzania	Meatu	2000	0.152	0.207	0.265
Tanzania	Meatu	2017	0.034	0.047	0.066
Tanzania	Meatu	2000-2017	-0.116	-0.085	-0.050

Table 2: Diarrhea DALYs rate by unit *(continued)*

Country	Unit	year	mean rate	lower rate	upper rate
Tanzania	Meru	2000	0.136	0.190	0.268
Tanzania	Meru	2017	0.034	0.048	0.065
Tanzania	Meru	2000-2017	-0.124	-0.085	-0.049
Tanzania	Micheweni	2000	0.155	0.224	0.298
Tanzania	Micheweni	2017	0.041	0.055	0.074
Tanzania	Micheweni	2000-2017	-0.118	-0.084	-0.046
Tanzania	Missenyi	2000	0.145	0.189	0.253
Tanzania	Missenyi	2017	0.041	0.054	0.072
Tanzania	Missenyi	2000-2017	-0.105	-0.075	-0.044
Tanzania	Misungwi	2000	0.119	0.160	0.211
Tanzania	Misungwi	2017	0.035	0.047	0.060
Tanzania	Misungwi	2000-2017	-0.108	-0.076	-0.044
Tanzania	Mjini	2000	0.133	0.182	0.247
Tanzania	Mjini	2017	0.035	0.049	0.063
Tanzania	Mjini	2000-2017	-0.113	-0.081	-0.047
Tanzania	Mkalama	2000	0.128	0.177	0.233
Tanzania	Mkalama	2017	0.030	0.042	0.057
Tanzania	Mkalama	2000-2017	-0.118	-0.086	-0.048
Tanzania	Mkinga	2000	0.135	0.187	0.248
Tanzania	Mkinga	2017	0.034	0.046	0.059
Tanzania	Mkinga	2000-2017	-0.124	-0.091	-0.056
Tanzania	Mkoani	2000	0.165	0.233	0.305
Tanzania	Mkoani	2017	0.039	0.052	0.070
Tanzania	Mkoani	2000-2017	-0.125	-0.091	-0.059
Tanzania	Mkuranga	2000	0.129	0.175	0.235
Tanzania	Mkuranga	2017	0.033	0.046	0.061
Tanzania	Mkuranga	2000-2017	-0.120	-0.088	-0.053
Tanzania	Mlele	2000	0.148	0.208	0.280
Tanzania	Mlele	2017	0.039	0.057	0.077
Tanzania	Mlele	2000-2017	-0.107	-0.078	-0.048
Tanzania	Momba	2000	0.156	0.215	0.289
Tanzania	Momba	2017	0.043	0.060	0.080
Tanzania	Momba	2000-2017	-0.107	-0.074	-0.046
Tanzania	Monduli	2000	0.154	0.209	0.286
Tanzania	Monduli	2017	0.036	0.049	0.064
Tanzania	Monduli	2000-2017	-0.123	-0.087	-0.053
Tanzania	Morogoro Rural	2000	0.116	0.164	0.218
Tanzania	Morogoro Rural	2017	0.031	0.044	0.058
Tanzania	Morogoro Rural	2000-2017	-0.114	-0.083	-0.053
Tanzania	Morogoro Urban	2000	0.124	0.179	0.236
Tanzania	Morogoro Urban	2017	0.034	0.049	0.064
Tanzania	Morogoro Urban	2000-2017	-0.116	-0.083	-0.051
Tanzania	Moshi Rural	2000	0.137	0.190	0.261
Tanzania	Moshi Rural	2017	0.037	0.052	0.068
Tanzania	Moshi Rural	2000-2017	-0.117	-0.081	-0.048
Tanzania	Moshi Urban	2000	0.125	0.177	0.248
Tanzania	Moshi Urban	2017	0.034	0.048	0.064
Tanzania	Moshi Urban	2000-2017	-0.116	-0.082	-0.048
Tanzania	Mpanda	2000	0.139	0.205	0.277
Tanzania	Mpanda	2017	0.041	0.056	0.075
Tanzania	Mpanda	2000-2017	-0.106	-0.078	-0.047
Tanzania	Mpanda Urban	2000	0.141	0.209	0.287
Tanzania	Mpanda Urban	2017	0.037	0.057	0.080
Tanzania	Mpanda Urban	2000-2017	-0.116	-0.085	-0.052
Tanzania	Mpwapwa	2000	0.127	0.183	0.241
Tanzania	Mpwapwa	2017	0.034	0.047	0.067
Tanzania	Mpwapwa	2000-2017	-0.116	-0.083	-0.053
Tanzania	Mtwara Rural	2000	0.155	0.219	0.294
Tanzania	Mtwara Rural	2017	0.034	0.049	0.068
Tanzania	Mtwara Rural	2000-2017	-0.126	-0.094	-0.059
Tanzania	Mtwara Urban	2000	0.154	0.222	0.311

Table 2: Diarrhea DALYs rate by unit *(continued)*

Country	Unit	year	mean rate	lower rate	upper rate
Tanzania	Mtwara Urban	2017	0.035	0.051	0.072
Tanzania	Mtwara Urban	2000-2017	-0.130	-0.095	-0.061
Tanzania	Mufindi	2000	0.126	0.185	0.256
Tanzania	Mufindi	2017	0.033	0.047	0.064
Tanzania	Mufindi	2000-2017	-0.118	-0.087	-0.058
Tanzania	Muheza	2000	0.125	0.181	0.237
Tanzania	Muheza	2017	0.032	0.045	0.059
Tanzania	Muheza	2000-2017	-0.123	-0.090	-0.057
Tanzania	Muleba	2000	0.139	0.183	0.243
Tanzania	Muleba	2017	0.039	0.053	0.068
Tanzania	Muleba	2000-2017	-0.106	-0.074	-0.047
Tanzania	Musoma Rural	2000	0.156	0.205	0.269
Tanzania	Musoma Rural	2017	0.044	0.059	0.080
Tanzania	Musoma Rural	2000-2017	-0.116	-0.082	-0.049
Tanzania	Musoma Urban	2000	0.169	0.232	0.300
Tanzania	Musoma Urban	2017	0.052	0.069	0.093
Tanzania	Musoma Urban	2000-2017	-0.108	-0.079	-0.047
Tanzania	Mvomero	2000	0.129	0.177	0.234
Tanzania	Mvomero	2017	0.035	0.047	0.061
Tanzania	Mvomero	2000-2017	-0.111	-0.081	-0.052
Tanzania	Mwanga	2000	0.129	0.183	0.257
Tanzania	Mwanga	2017	0.035	0.050	0.067
Tanzania	Mwanga	2000-2017	-0.118	-0.082	-0.049
Tanzania	Nachingwea	2000	0.151	0.214	0.286
Tanzania	Nachingwea	2017	0.037	0.051	0.067
Tanzania	Nachingwea	2000-2017	-0.128	-0.094	-0.056
Tanzania	Namtumbo	2000	0.130	0.183	0.243
Tanzania	Namtumbo	2017	0.033	0.047	0.064
Tanzania	Namtumbo	2000-2017	-0.116	-0.090	-0.059
Tanzania	Nanyumbu	2000	0.148	0.212	0.282
Tanzania	Nanyumbu	2017	0.037	0.051	0.070
Tanzania	Nanyumbu	2000-2017	-0.120	-0.086	-0.051
Tanzania	Newala	2000	0.155	0.217	0.294
Tanzania	Newala	2017	0.036	0.051	0.068
Tanzania	Newala	2000-2017	-0.133	-0.098	-0.058
Tanzania	Ngara	2000	0.191	0.240	0.325
Tanzania	Ngara	2017	0.049	0.065	0.085
Tanzania	Ngara	2000-2017	-0.111	-0.082	-0.053
Tanzania	Ngorongoro	2000	0.162	0.222	0.297
Tanzania	Ngorongoro	2017	0.039	0.052	0.069
Tanzania	Ngorongoro	2000-2017	-0.109	-0.079	-0.046
Tanzania	Njombe	2000	0.130	0.174	0.242
Tanzania	Njombe	2017	0.031	0.044	0.060
Tanzania	Njombe	2000-2017	-0.121	-0.089	-0.058
Tanzania	Njombe Urban	2000	0.130	0.169	0.230
Tanzania	Njombe Urban	2017	0.029	0.042	0.057
Tanzania	Njombe Urban	2000-2017	-0.119	-0.091	-0.057
Tanzania	Nkasi	2000	0.149	0.208	0.282
Tanzania	Nkasi	2017	0.040	0.056	0.077
Tanzania	Nkasi	2000-2017	-0.104	-0.074	-0.042
Tanzania	Nyamagana	2000	0.115	0.156	0.208
Tanzania	Nyamagana	2017	0.034	0.047	0.061
Tanzania	Nyamagana	2000-2017	-0.106	-0.074	-0.041
Tanzania	Nyang'wale	2000	0.125	0.170	0.229
Tanzania	Nyang'wale	2017	0.036	0.049	0.064
Tanzania	Nyang'wale	2000-2017	-0.107	-0.074	-0.044
Tanzania	Nyasa	2000	0.114	0.158	0.210
Tanzania	Nyasa	2017	0.031	0.045	0.062
Tanzania	Nyasa	2000-2017	-0.109	-0.082	-0.051
Tanzania	Nzega	2000	0.151	0.206	0.266
Tanzania	Nzega	2017	0.032	0.042	0.057

Table 2: Diarrhea DALYs rate by unit *(continued)*

Country	Unit	year	mean rate	lower rate	upper rate
Tanzania	Nzega	2000-2017	-0.132	-0.101	-0.069
Tanzania	Pangani	2000	0.116	0.165	0.217
Tanzania	Pangani	2017	0.031	0.042	0.055
Tanzania	Pangani	2000-2017	-0.118	-0.086	-0.053
Tanzania	Rombo	2000	0.146	0.205	0.275
Tanzania	Rombo	2017	0.041	0.056	0.076
Tanzania	Rombo	2000-2017	-0.116	-0.080	-0.046
Tanzania	Rorya	2000	0.190	0.256	0.329
Tanzania	Rorya	2017	0.055	0.071	0.093
Tanzania	Rorya	2000-2017	-0.104	-0.077	-0.048
Tanzania	Ruangwa	2000	0.146	0.212	0.290
Tanzania	Ruangwa	2017	0.035	0.049	0.065
Tanzania	Ruangwa	2000-2017	-0.130	-0.099	-0.061
Tanzania	Rufiji	2000	0.123	0.178	0.241
Tanzania	Rufiji	2017	0.034	0.047	0.063
Tanzania	Rufiji	2000-2017	-0.117	-0.086	-0.052
Tanzania	Rungwe	2000	0.120	0.160	0.220
Tanzania	Rungwe	2017	0.028	0.039	0.050
Tanzania	Rungwe	2000-2017	-0.128	-0.097	-0.065
Tanzania	Same	2000	0.136	0.189	0.258
Tanzania	Same	2017	0.036	0.051	0.067
Tanzania	Same	2000-2017	-0.117	-0.082	-0.048
Tanzania	Sengerema	2000	0.124	0.169	0.225
Tanzania	Sengerema	2017	0.038	0.052	0.066
Tanzania	Sengerema	2000-2017	-0.099	-0.070	-0.037
Tanzania	Serengeti	2000	0.166	0.222	0.284
Tanzania	Serengeti	2017	0.047	0.062	0.080
Tanzania	Serengeti	2000-2017	-0.108	-0.076	-0.048
Tanzania	Shinyanga Rural	2000	0.135	0.188	0.247
Tanzania	Shinyanga Rural	2017	0.031	0.043	0.055
Tanzania	Shinyanga Rural	2000-2017	-0.125	-0.093	-0.066
Tanzania	Shinyanga Urban	2000	0.142	0.197	0.259
Tanzania	Shinyanga Urban	2017	0.031	0.044	0.058
Tanzania	Shinyanga Urban	2000-2017	-0.128	-0.097	-0.065
Tanzania	Siha	2000	0.128	0.181	0.260
Tanzania	Siha	2017	0.034	0.047	0.064
Tanzania	Siha	2000-2017	-0.119	-0.083	-0.047
Tanzania	Sikonge	2000	0.156	0.217	0.279
Tanzania	Sikonge	2017	0.033	0.047	0.063
Tanzania	Sikonge	2000-2017	-0.117	-0.084	-0.054
Tanzania	Simanjiro	2000	0.122	0.168	0.228
Tanzania	Simanjiro	2017	0.030	0.042	0.053
Tanzania	Simanjiro	2000-2017	-0.119	-0.085	-0.052
Tanzania	Singida Rural	2000	0.124	0.181	0.240
Tanzania	Singida Rural	2017	0.030	0.042	0.057
Tanzania	Singida Rural	2000-2017	-0.121	-0.087	-0.051
Tanzania	Singida Urban	2000	0.127	0.190	0.253
Tanzania	Singida Urban	2017	0.030	0.043	0.060
Tanzania	Singida Urban	2000-2017	-0.125	-0.089	-0.053
Tanzania	Songea Rural	2000	0.121	0.172	0.228
Tanzania	Songea Rural	2017	0.030	0.044	0.060
Tanzania	Songea Rural	2000-2017	-0.118	-0.092	-0.063
Tanzania	Songea Urban	2000	0.125	0.180	0.245
Tanzania	Songea Urban	2017	0.031	0.047	0.066
Tanzania	Songea Urban	2000-2017	-0.116	-0.088	-0.058
Tanzania	Sumbawanga Rural	2000	0.155	0.219	0.290
Tanzania	Sumbawanga Rural	2017	0.045	0.062	0.083
Tanzania	Sumbawanga Rural	2000-2017	-0.098	-0.068	-0.039
Tanzania	Sumbawanga Urban	2000	0.155	0.216	0.290
Tanzania	Sumbawanga Urban	2017	0.043	0.060	0.085
Tanzania	Sumbawanga Urban	2000-2017	-0.104	-0.072	-0.037

Table 2: Diarrhea DALYs rate by unit *(continued)*

Country	Unit	year	mean rate	lower rate	upper rate
Tanzania	Tabora Urban	2000	0.131	0.188	0.249
Tanzania	Tabora Urban	2017	0.028	0.039	0.051
Tanzania	Tabora Urban	2000-2017	-0.131	-0.095	-0.062
Tanzania	Tandahimba	2000	0.151	0.214	0.286
Tanzania	Tandahimba	2017	0.035	0.049	0.065
Tanzania	Tandahimba	2000-2017	-0.130	-0.094	-0.055
Tanzania	Tanga	2000	0.121	0.170	0.225
Tanzania	Tanga	2017	0.031	0.043	0.056
Tanzania	Tanga	2000-2017	-0.122	-0.089	-0.056
Tanzania	Tarime	2000	0.187	0.244	0.315
Tanzania	Tarime	2017	0.054	0.069	0.090
Tanzania	Tarime	2000-2017	-0.102	-0.075	-0.047
Tanzania	Temeke	2000	0.119	0.163	0.218
Tanzania	Temeke	2017	0.032	0.043	0.057
Tanzania	Temeke	2000-2017	-0.119	-0.088	-0.051
Tanzania	Tunduma	2000	0.179	0.246	0.320
Tanzania	Tunduma	2017	0.045	0.064	0.084
Tanzania	Tunduma	2000-2017	-0.117	-0.083	-0.051
Tanzania	Tunduru	2000	0.142	0.202	0.264
Tanzania	Tunduru	2017	0.034	0.052	0.071
Tanzania	Tunduru	2000-2017	-0.115	-0.084	-0.049
Tanzania	Ukerewe	2000	0.131	0.176	0.246
Tanzania	Ukerewe	2017	0.039	0.054	0.074
Tanzania	Ukerewe	2000-2017	-0.106	-0.073	-0.042
Tanzania	Ulanga	2000	0.131	0.184	0.245
Tanzania	Ulanga	2017	0.033	0.047	0.065
Tanzania	Ulanga	2000-2017	-0.119	-0.089	-0.061
Tanzania	Urambo	2000	0.151	0.216	0.296
Tanzania	Urambo	2017	0.032	0.046	0.063
Tanzania	Urambo	2000-2017	-0.128	-0.097	-0.067
Tanzania	Uvinza	2000	0.153	0.214	0.279
Tanzania	Uvinza	2017	0.046	0.061	0.080
Tanzania	Uvinza	2000-2017	-0.104	-0.076	-0.048
Tanzania	Uyui	2000	0.169	0.227	0.302
Tanzania	Uyui	2017	0.034	0.047	0.061
Tanzania	Uyui	2000-2017	-0.126	-0.092	-0.063
Tanzania	Wanging'ombe	2000	0.129	0.172	0.235
Tanzania	Wanging'ombe	2017	0.031	0.044	0.061
Tanzania	Wanging'ombe	2000-2017	-0.116	-0.086	-0.056
Tanzania	Wete	2000	0.153	0.221	0.296
Tanzania	Wete	2017	0.039	0.053	0.071
Tanzania	Wete	2000-2017	-0.122	-0.086	-0.050
Togo	Amou	2000	0.224	0.334	0.465
Togo	Amou	2017	0.077	0.116	0.163
Togo	Amou	2000-2017	-0.143	-0.057	0.030
Togo	Assoli	2000	0.284	0.393	0.526
Togo	Assoli	2017	0.096	0.135	0.187
Togo	Assoli	2000-2017	-0.157	-0.066	0.026
Togo	Bassar	2000	0.310	0.423	0.583
Togo	Bassar	2017	0.108	0.162	0.235
Togo	Bassar	2000-2017	-0.148	-0.056	0.025
Togo	Bimah	2000	0.288	0.404	0.543
Togo	Bimah	2017	0.102	0.145	0.198
Togo	Bimah	2000-2017	-0.159	-0.071	0.022
Togo	Doufelgou	2000	0.301	0.415	0.558
Togo	Doufelgou	2017	0.107	0.156	0.216
Togo	Doufelgou	2000-2017	-0.156	-0.066	0.024
Togo	Golfe (incl Lomé)	2000	0.197	0.273	0.376
Togo	Golfe (incl Lomé)	2017	0.080	0.113	0.156
Togo	Golfe (incl Lomé)	2000-2017	-0.168	-0.062	0.028
Togo	Haho	2000	0.225	0.326	0.433

Table 2: Diarrhea DALYs rate by unit *(continued)*

Country	Unit	year	mean rate	lower rate	upper rate
Togo	Haho	2017	0.082	0.117	0.155
Togo	Haho	2000-2017	-0.161	-0.064	0.025
Togo	Kéran	2000	0.328	0.450	0.602
Togo	Kéran	2017	0.121	0.176	0.243
Togo	Kéran	2000-2017	-0.151	-0.069	0.024
Togo	Kloto	2000	0.212	0.303	0.440
Togo	Kloto	2017	0.077	0.115	0.160
Togo	Kloto	2000-2017	-0.160	-0.061	0.035
Togo	Kozah	2000	0.291	0.398	0.538
Togo	Kozah	2017	0.099	0.143	0.199
Togo	Kozah	2000-2017	-0.158	-0.070	0.022
Togo	Lacs	2000	0.199	0.284	0.392
Togo	Lacs	2017	0.079	0.108	0.139
Togo	Lacs	2000-2017	-0.169	-0.068	0.027
Togo	Ogou	2000	0.245	0.355	0.494
Togo	Ogou	2017	0.083	0.117	0.165
Togo	Ogou	2000-2017	-0.149	-0.061	0.031
Togo	Oti	2000	0.399	0.539	0.709
Togo	Oti	2017	0.145	0.209	0.298
Togo	Oti	2000-2017	-0.163	-0.079	0.003
Togo	Sotouboua	2000	0.258	0.380	0.547
Togo	Sotouboua	2017	0.081	0.124	0.182
Togo	Sotouboua	2000-2017	-0.141	-0.054	0.026
Togo	Tchamba (Nyala)	2000	0.270	0.392	0.545
Togo	Tchamba (Nyala)	2017	0.081	0.122	0.171
Togo	Tchamba (Nyala)	2000-2017	-0.154	-0.066	0.035
Togo	Tchaudjo	2000	0.272	0.387	0.545
Togo	Tchaudjo	2017	0.085	0.128	0.181
Togo	Tchaudjo	2000-2017	-0.153	-0.065	0.030
Togo	Tône	2000	0.393	0.537	0.696
Togo	Tône	2017	0.139	0.205	0.289
Togo	Tône	2000-2017	-0.183	-0.098	-0.018
Togo	Vo	2000	0.210	0.295	0.407
Togo	Vo	2017	0.082	0.111	0.145
Togo	Vo	2000-2017	-0.170	-0.067	0.029
Togo	Wawa	2000	0.237	0.344	0.488
Togo	Wawa	2017	0.079	0.122	0.173
Togo	Wawa	2000-2017	-0.144	-0.054	0.033
Togo	Yoto	2000	0.212	0.306	0.414
Togo	Yoto	2017	0.082	0.114	0.150
Togo	Yoto	2000-2017	-0.162	-0.063	0.028
Togo	Zio	2000	0.223	0.309	0.424
Togo	Zio	2017	0.084	0.117	0.158
Togo	Zio	2000-2017	-0.174	-0.068	0.019
Uganda	Agago	2000	0.154	0.205	0.260
Uganda	Agago	2017	0.060	0.076	0.096
Uganda	Agago	2000-2017	-0.110	-0.075	-0.047
Uganda	Agule	2000	0.151	0.190	0.242
Uganda	Agule	2017	0.063	0.081	0.101
Uganda	Agule	2000-2017	-0.099	-0.068	-0.038
Uganda	Amuria	2000	0.157	0.213	0.268
Uganda	Amuria	2017	0.064	0.083	0.103
Uganda	Amuria	2000-2017	-0.107	-0.074	-0.045
Uganda	Apac Municipality	2000	0.161	0.215	0.277
Uganda	Apac Municipality	2017	0.056	0.073	0.090
Uganda	Apac Municipality	2000-2017	-0.110	-0.082	-0.050
Uganda	Aringa	2000	0.138	0.184	0.239
Uganda	Aringa	2017	0.044	0.059	0.080
Uganda	Aringa	2000-2017	-0.122	-0.086	-0.052
Uganda	Arua Municipality	2000	0.135	0.180	0.238
Uganda	Arua Municipality	2017	0.043	0.060	0.082

Table 2: Diarrhea DALYs rate by unit *(continued)*

Country	Unit	year	mean rate	lower rate	upper rate
Uganda	Arua Municipality	2000-2017	-0.120	-0.091	-0.060
Uganda	Aruu	2000	0.159	0.210	0.277
Uganda	Aruu	2017	0.061	0.079	0.101
Uganda	Aruu	2000-2017	-0.109	-0.075	-0.042
Uganda	Aswa	2000	0.155	0.209	0.274
Uganda	Aswa	2017	0.061	0.080	0.104
Uganda	Aswa	2000-2017	-0.098	-0.067	-0.036
Uganda	Ayivu	2000	0.141	0.188	0.247
Uganda	Ayivu	2017	0.043	0.060	0.082
Uganda	Ayivu	2000-2017	-0.121	-0.092	-0.062
Uganda	Bamunanika	2000	0.108	0.138	0.178
Uganda	Bamunanika	2017	0.049	0.064	0.083
Uganda	Bamunanika	2000-2017	-0.084	-0.056	-0.025
Uganda	Bbaale	2000	0.120	0.153	0.195
Uganda	Bbaale	2017	0.052	0.069	0.090
Uganda	Bbaale	2000-2017	-0.090	-0.060	-0.030
Uganda	Bokora	2000	0.133	0.182	0.234
Uganda	Bokora	2017	0.054	0.071	0.090
Uganda	Bokora	2000-2017	-0.085	-0.056	-0.024
Uganda	Bubulo East	2000	0.164	0.207	0.262
Uganda	Bubulo East	2017	0.056	0.071	0.091
Uganda	Bubulo East	2000-2017	-0.112	-0.085	-0.053
Uganda	Bubulo West	2000	0.168	0.216	0.272
Uganda	Bubulo West	2017	0.059	0.075	0.095
Uganda	Bubulo West	2000-2017	-0.114	-0.086	-0.055
Uganda	Budadiri	2000	0.160	0.207	0.261
Uganda	Budadiri	2017	0.057	0.073	0.091
Uganda	Budadiri	2000-2017	-0.113	-0.083	-0.054
Uganda	Budaka	2000	0.158	0.201	0.252
Uganda	Budaka	2017	0.064	0.082	0.104
Uganda	Budaka	2000-2017	-0.098	-0.071	-0.040
Uganda	Budiope	2000	0.149	0.193	0.243
Uganda	Budiope	2017	0.066	0.084	0.106
Uganda	Budiope	2000-2017	-0.094	-0.064	-0.035
Uganda	Bufumbira	2000	0.113	0.149	0.190
Uganda	Bufumbira	2017	0.048	0.062	0.079
Uganda	Bufumbira	2000-2017	-0.096	-0.067	-0.041
Uganda	Bugabula	2000	0.140	0.177	0.224
Uganda	Bugabula	2017	0.064	0.083	0.104
Uganda	Bugabula	2000-2017	-0.089	-0.060	-0.031
Uganda	Bugahya	2000	0.128	0.177	0.242
Uganda	Bugahya	2017	0.044	0.057	0.076
Uganda	Bugahya	2000-2017	-0.104	-0.072	-0.041
Uganda	Bugangaizi	2000	0.100	0.135	0.179
Uganda	Bugangaizi	2017	0.039	0.052	0.066
Uganda	Bugangaizi	2000-2017	-0.088	-0.059	-0.029
Uganda	Bughendera	2000	0.107	0.147	0.191
Uganda	Bughendera	2017	0.049	0.063	0.083
Uganda	Bughendera	2000-2017	-0.099	-0.068	-0.035
Uganda	Bugiri Municipality	2000	0.141	0.177	0.223
Uganda	Bugiri Municipality	2017	0.061	0.079	0.100
Uganda	Bugiri Municipality	2000-2017	-0.083	-0.056	-0.024
Uganda	Bugweri	2000	0.149	0.190	0.240
Uganda	Bugweri	2017	0.065	0.084	0.105
Uganda	Bugweri	2000-2017	-0.088	-0.060	-0.028
Uganda	Buhaguzi	2000	0.121	0.164	0.217
Uganda	Buhaguzi	2017	0.042	0.055	0.071
Uganda	Buhaguzi	2000-2017	-0.099	-0.069	-0.038
Uganda	Buhweju	2000	0.104	0.137	0.173
Uganda	Buhweju	2017	0.044	0.058	0.075
Uganda	Buhweju	2000-2017	-0.097	-0.065	-0.035

Table 2: Diarrhea DALYs rate by unit *(continued)*

Country	Unit	year	mean rate	lower rate	upper rate
Uganda	Buikwe	2000	0.121	0.157	0.200
Uganda	Buikwe	2017	0.058	0.075	0.094
Uganda	Buikwe	2000-2017	-0.096	-0.066	-0.034
Uganda	Bujenje	2000	0.123	0.171	0.225
Uganda	Bujenje	2017	0.041	0.054	0.071
Uganda	Bujenje	2000-2017	-0.107	-0.075	-0.044
Uganda	Bujumba	2000	0.113	0.147	0.202
Uganda	Bujumba	2017	0.045	0.061	0.079
Uganda	Bujumba	2000-2017	-0.093	-0.062	-0.031
Uganda	Bukanga	2000	0.127	0.169	0.222
Uganda	Bukanga	2017	0.053	0.071	0.092
Uganda	Bukanga	2000-2017	-0.105	-0.071	-0.037
Uganda	Bukedea	2000	0.155	0.198	0.254
Uganda	Bukedea	2017	0.062	0.078	0.098
Uganda	Bukedea	2000-2017	-0.102	-0.072	-0.042
Uganda	Bukomansimbi	2000	0.125	0.170	0.227
Uganda	Bukomansimbi	2017	0.050	0.066	0.084
Uganda	Bukomansimbi	2000-2017	-0.096	-0.064	-0.029
Uganda	Bukonzo	2000	0.116	0.155	0.195
Uganda	Bukonzo	2017	0.053	0.068	0.089
Uganda	Bukonzo	2000-2017	-0.092	-0.066	-0.037
Uganda	Bukooli	2000	0.165	0.210	0.259
Uganda	Bukooli	2017	0.065	0.087	0.110
Uganda	Bukooli	2000-2017	-0.085	-0.060	-0.031
Uganda	Bukooli North	2000	0.146	0.185	0.232
Uganda	Bukooli North	2017	0.063	0.082	0.104
Uganda	Bukooli North	2000-2017	-0.085	-0.058	-0.027
Uganda	Bukoto	2000	0.130	0.171	0.223
Uganda	Bukoto	2000	0.136	0.178	0.232
Uganda	Bukoto	2017	0.052	0.067	0.087
Uganda	Bukoto	2017	0.052	0.069	0.088
Uganda	Bukoto	2000-2017	-0.095	-0.065	-0.034
Uganda	Bukoto	2000-2017	-0.092	-0.063	-0.031
Uganda	Bulambuli	2000	0.155	0.203	0.260
Uganda	Bulambuli	2017	0.056	0.072	0.090
Uganda	Bulambuli	2000-2017	-0.114	-0.083	-0.054
Uganda	Bulamogi	2000	0.144	0.188	0.240
Uganda	Bulamogi	2017	0.069	0.089	0.114
Uganda	Bulamogi	2000-2017	-0.087	-0.058	-0.026
Uganda	Buliisa	2000	0.159	0.214	0.281
Uganda	Buliisa	2017	0.049	0.066	0.087
Uganda	Buliisa	2000-2017	-0.115	-0.081	-0.053
Uganda	Bungokho	2000	0.158	0.202	0.253
Uganda	Bungokho	2017	0.058	0.075	0.093
Uganda	Bungokho	2000-2017	-0.108	-0.080	-0.049
Uganda	Bunya	2000	0.149	0.190	0.240
Uganda	Bunya	2017	0.067	0.087	0.109
Uganda	Bunya	2000-2017	-0.088	-0.060	-0.029
Uganda	Bunyangabu	2000	0.102	0.138	0.180
Uganda	Bunyangabu	2017	0.048	0.062	0.081
Uganda	Bunyangabu	2000-2017	-0.091	-0.061	-0.029
Uganda	Bunyaruguru	2000	0.115	0.155	0.194
Uganda	Bunyaruguru	2017	0.049	0.065	0.085
Uganda	Bunyaruguru	2000-2017	-0.093	-0.064	-0.033
Uganda	Bunyole	2000	0.157	0.197	0.247
Uganda	Bunyole	2017	0.065	0.082	0.103
Uganda	Bunyole	2000-2017	-0.095	-0.067	-0.033
Uganda	Burahya	2000	0.104	0.145	0.190
Uganda	Burahya	2017	0.047	0.060	0.078
Uganda	Burahya	2000-2017	-0.093	-0.065	-0.033
Uganda	Buruli	2000	0.134	0.185	0.243

Table 2: Diarrhea DALYs rate by unit *(continued)*

Country	Unit	year	mean rate	lower rate	upper rate
Uganda	Buruli	2000	0.120	0.158	0.205
Uganda	Buruli	2017	0.045	0.059	0.078
Uganda	Buruli	2017	0.048	0.062	0.077
Uganda	Buruli	2000-2017	-0.096	-0.066	-0.037
Uganda	Buruli	2000-2017	-0.108	-0.075	-0.046
Uganda	Bushenyi-Ishaka Municipality	2000	0.108	0.140	0.180
Uganda	Bushenyi-Ishaka Municipality	2017	0.046	0.060	0.078
Uganda	Bushenyi-Ishaka Municipality	2000-2017	-0.094	-0.064	-0.034
Uganda	Busia Municipality	2000	0.138	0.177	0.221
Uganda	Busia Municipality	2017	0.056	0.073	0.094
Uganda	Busia Municipality	2000-2017	-0.097	-0.070	-0.040
Uganda	Busiki	2000	0.149	0.188	0.239
Uganda	Busiki	2017	0.067	0.086	0.108
Uganda	Busiki	2000-2017	-0.085	-0.058	-0.025
Uganda	Busiro	2000	0.113	0.144	0.187
Uganda	Busiro	2017	0.051	0.064	0.080
Uganda	Busiro	2000-2017	-0.091	-0.060	-0.030
Uganda	Busongora	2000	0.107	0.143	0.182
Uganda	Busongora	2017	0.050	0.065	0.084
Uganda	Busongora	2000-2017	-0.088	-0.061	-0.031
Uganda	Busujju	2000	0.111	0.146	0.196
Uganda	Busujju	2017	0.046	0.061	0.076
Uganda	Busujju	2000-2017	-0.090	-0.059	-0.027
Uganda	Butambala	2000	0.110	0.145	0.191
Uganda	Butambala	2017	0.046	0.061	0.078
Uganda	Butambala	2000-2017	-0.090	-0.061	-0.029
Uganda	Butebo	2000	0.157	0.198	0.250
Uganda	Butebo	2017	0.061	0.079	0.100
Uganda	Butebo	2000-2017	-0.102	-0.073	-0.045
Uganda	Butembe	2000	0.137	0.173	0.216
Uganda	Butembe	2017	0.065	0.084	0.105
Uganda	Butembe	2000-2017	-0.096	-0.063	-0.029
Uganda	Buvuma Island	2000	0.135	0.176	0.225
Uganda	Buvuma Island	2017	0.066	0.086	0.110
Uganda	Buvuma Island	2000-2017	-0.092	-0.063	-0.034
Uganda	Buwekula	2000	0.106	0.141	0.188
Uganda	Buwekula	2017	0.043	0.059	0.076
Uganda	Buwekula	2000-2017	-0.079	-0.048	-0.015
Uganda	Buyaga	2000	0.137	0.179	0.235
Uganda	Buyaga	2017	0.044	0.056	0.072
Uganda	Buyaga	2000-2017	-0.106	-0.074	-0.043
Uganda	Buyanja	2000	0.107	0.143	0.186
Uganda	Buyanja	2017	0.041	0.054	0.070
Uganda	Buyanja	2000-2017	-0.090	-0.058	-0.029
Uganda	Buzaaya	2000	0.131	0.165	0.210
Uganda	Buzaaya	2017	0.061	0.082	0.103
Uganda	Buzaaya	2000-2017	-0.086	-0.057	-0.026
Uganda	Bwamba	2000	0.115	0.155	0.200
Uganda	Bwamba	2017	0.053	0.068	0.091
Uganda	Bwamba	2000-2017	-0.097	-0.066	-0.033
Uganda	Chekwii	2000	0.142	0.190	0.245
Uganda	Chekwii	2017	0.052	0.067	0.085
Uganda	Chekwii	2000-2017	-0.099	-0.070	-0.041
Uganda	Chua	2000	0.167	0.225	0.294
Uganda	Chua	2017	0.059	0.075	0.097
Uganda	Chua	2000-2017	-0.114	-0.083	-0.051
Uganda	Dodoth	2000	0.144	0.199	0.261
Uganda	Dodoth	2017	0.046	0.062	0.082
Uganda	Dodoth	2000-2017	-0.119	-0.083	-0.054
Uganda	Dokolo	2000	0.172	0.221	0.283
Uganda	Dokolo	2017	0.060	0.077	0.095

Table 2: Diarrhea DALYs rate by unit *(continued)*

Country	Unit	year	mean rate	lower rate	upper rate
Uganda	Dokolo	2000-2017	-0.117	-0.085	-0.054
Uganda	East Moyo	2000	0.147	0.200	0.256
Uganda	East Moyo	2017	0.053	0.070	0.095
Uganda	East Moyo	2000-2017	-0.113	-0.077	-0.048
Uganda	Entebbe Municipality	2000	0.113	0.148	0.194
Uganda	Entebbe Municipality	2017	0.051	0.065	0.081
Uganda	Entebbe Municipality	2000-2017	-0.092	-0.061	-0.029
Uganda	Erute	2000	0.176	0.227	0.291
Uganda	Erute	2017	0.063	0.081	0.100
Uganda	Erute	2000-2017	-0.115	-0.083	-0.053
Uganda	Fort Portal Municipality	2000	0.100	0.138	0.184
Uganda	Fort Portal Municipality	2017	0.045	0.058	0.076
Uganda	Fort Portal Municipality	2000-2017	-0.092	-0.064	-0.032
Uganda	Gomba	2000	0.114	0.150	0.196
Uganda	Gomba	2017	0.045	0.061	0.078
Uganda	Gomba	2000-2017	-0.086	-0.058	-0.029
Uganda	Gulu Municipality	2000	0.155	0.214	0.282
Uganda	Gulu Municipality	2017	0.063	0.084	0.110
Uganda	Gulu Municipality	2000-2017	-0.098	-0.067	-0.034
Uganda	Hoima Municipality	2000	0.118	0.166	0.227
Uganda	Hoima Municipality	2017	0.039	0.052	0.068
Uganda	Hoima Municipality	2000-2017	-0.105	-0.073	-0.043
Uganda	Ibanda	2000	0.103	0.141	0.177
Uganda	Ibanda	2017	0.044	0.060	0.077
Uganda	Ibanda	2000-2017	-0.093	-0.061	-0.026
Uganda	Ibanda Municipality	2000	0.103	0.143	0.179
Uganda	Ibanda Municipality	2017	0.044	0.059	0.076
Uganda	Ibanda Municipality	2000-2017	-0.097	-0.064	-0.029
Uganda	Iganga Municipality	2000	0.136	0.174	0.224
Uganda	Iganga Municipality	2017	0.066	0.086	0.108
Uganda	Iganga Municipality	2000-2017	-0.085	-0.055	-0.022
Uganda	Igara	2000	0.111	0.145	0.184
Uganda	Igara	2017	0.046	0.060	0.078
Uganda	Igara	2000-2017	-0.093	-0.064	-0.035
Uganda	Iki-Iki	2000	0.165	0.207	0.262
Uganda	Iki-Iki	2017	0.063	0.081	0.102
Uganda	Iki-Iki	2000-2017	-0.104	-0.075	-0.046
Uganda	Isingiro	2000	0.123	0.160	0.208
Uganda	Isingiro	2017	0.051	0.069	0.088
Uganda	Isingiro	2000-2017	-0.103	-0.070	-0.040
Uganda	Jie	2000	0.146	0.201	0.261
Uganda	Jie	2017	0.051	0.069	0.090
Uganda	Jie	2000-2017	-0.107	-0.075	-0.048
Uganda	Jinja Municipality	2000	0.132	0.169	0.212
Uganda	Jinja Municipality	2017	0.065	0.084	0.106
Uganda	Jinja Municipality	2000-2017	-0.098	-0.065	-0.031
Uganda	Jonam	2000	0.155	0.205	0.268
Uganda	Jonam	2017	0.049	0.065	0.088
Uganda	Jonam	2000-2017	-0.120	-0.087	-0.051
Uganda	Kabale Municipality	2000	0.120	0.156	0.199
Uganda	Kabale Municipality	2017	0.052	0.067	0.086
Uganda	Kabale Municipality	2000-2017	-0.097	-0.069	-0.041
Uganda	Kaberamaido	2000	0.166	0.218	0.283
Uganda	Kaberamaido	2017	0.060	0.078	0.097
Uganda	Kaberamaido	2000-2017	-0.115	-0.082	-0.053
Uganda	Kabula	2000	0.137	0.179	0.233
Uganda	Kabula	2017	0.050	0.067	0.087
Uganda	Kabula	2000-2017	-0.099	-0.068	-0.033
Uganda	Kagoma	2000	0.136	0.172	0.216
Uganda	Kagoma	2017	0.064	0.084	0.105
Uganda	Kagoma	2000-2017	-0.093	-0.061	-0.029

Table 2: Diarrhea DALYs rate by unit *(continued)*

Country	Unit	year	mean rate	lower rate	upper rate
Uganda	Kajara	2000	0.109	0.144	0.184
Uganda	Kajara	2017	0.049	0.065	0.086
Uganda	Kajara	2000-2017	-0.087	-0.058	-0.029
Uganda	Kakuuto North	2000	0.133	0.175	0.232
Uganda	Kakuuto North	2017	0.054	0.070	0.091
Uganda	Kakuuto North	2000-2017	-0.090	-0.059	-0.028
Uganda	Kalaki	2000	0.165	0.220	0.281
Uganda	Kalaki	2017	0.061	0.079	0.098
Uganda	Kalaki	2000-2017	-0.116	-0.081	-0.052
Uganda	Kalungu	2000	0.125	0.165	0.220
Uganda	Kalungu	2017	0.049	0.065	0.082
Uganda	Kalungu	2000-2017	-0.093	-0.063	-0.032
Uganda	Kamuli Municipality	2000	0.135	0.171	0.218
Uganda	Kamuli Municipality	2017	0.064	0.084	0.107
Uganda	Kamuli Municipality	2000-2017	-0.088	-0.058	-0.028
Uganda	Kapchorwa Municipality	2000	0.146	0.193	0.249
Uganda	Kapchorwa Municipality	2017	0.052	0.069	0.088
Uganda	Kapchorwa Municipality	2000-2017	-0.115	-0.085	-0.054
Uganda	Kapelebyong	2000	0.157	0.211	0.272
Uganda	Kapelebyong	2017	0.063	0.082	0.102
Uganda	Kapelebyong	2000-2017	-0.106	-0.075	-0.047
Uganda	Kasambya	2000	0.112	0.150	0.194
Uganda	Kasambya	2017	0.046	0.062	0.082
Uganda	Kasambya	2000-2017	-0.078	-0.046	-0.014
Uganda	Kasese Municipality	2000	0.107	0.143	0.182
Uganda	Kasese Municipality	2017	0.050	0.065	0.084
Uganda	Kasese Municipality	2000-2017	-0.089	-0.060	-0.030
Uganda	Kashari	2000	0.113	0.152	0.195
Uganda	Kashari	2017	0.047	0.062	0.080
Uganda	Kashari	2000-2017	-0.100	-0.069	-0.039
Uganda	Kasilo	2000	0.151	0.201	0.260
Uganda	Kasilo	2017	0.064	0.082	0.105
Uganda	Kasilo	2000-2017	-0.105	-0.071	-0.042
Uganda	Kassanda	2000	0.110	0.147	0.193
Uganda	Kassanda	2017	0.045	0.060	0.078
Uganda	Kassanda	2000-2017	-0.078	-0.052	-0.016
Uganda	Katerera	2000	0.115	0.157	0.197
Uganda	Katerera	2017	0.048	0.065	0.085
Uganda	Katerera	2000-2017	-0.097	-0.066	-0.034
Uganda	Katikamu	2000	0.116	0.148	0.192
Uganda	Katikamu	2017	0.048	0.063	0.082
Uganda	Katikamu	2000-2017	-0.087	-0.059	-0.029
Uganda	Katuuto East	2000	0.113	0.152	0.204
Uganda	Katuuto East	2017	0.049	0.066	0.086
Uganda	Katuuto East	2000-2017	-0.088	-0.057	-0.027
Uganda	Katuuto West	2000	0.123	0.165	0.225
Uganda	Katuuto West	2017	0.052	0.069	0.089
Uganda	Katuuto West	2000-2017	-0.089	-0.059	-0.027
Uganda	Kazo	2000	0.099	0.137	0.173
Uganda	Kazo	2017	0.044	0.060	0.078
Uganda	Kazo	2000-2017	-0.086	-0.056	-0.020
Uganda	Kcca	2000	0.093	0.121	0.158
Uganda	Kcca	2017	0.044	0.057	0.071
Uganda	Kcca	2000-2017	-0.089	-0.057	-0.026
Uganda	Kibale	2000	0.101	0.136	0.174
Uganda	Kibale	2017	0.046	0.062	0.080
Uganda	Kibale	2000-2017	-0.086	-0.053	-0.019
Uganda	Kibanda	2000	0.134	0.187	0.244
Uganda	Kibanda	2017	0.048	0.063	0.082
Uganda	Kibanda	2000-2017	-0.107	-0.074	-0.045
Uganda	Kiboga	2000	0.102	0.137	0.180

Table 2: Diarrhea DALYs rate by unit *(continued)*

Country	Unit	year	mean rate	lower rate	upper rate
Uganda	Kiboga	2000	0.099	0.136	0.178
Uganda	Kiboga	2017	0.043	0.057	0.073
Uganda	Kiboga	2017	0.040	0.053	0.069
Uganda	Kiboga	2000-2017	-0.086	-0.057	-0.024
Uganda	Kiboga	2000-2017	-0.088	-0.060	-0.027
Uganda	Kibuku	2000	0.153	0.192	0.241
Uganda	Kibuku	2017	0.064	0.082	0.103
Uganda	Kibuku	2000-2017	-0.094	-0.068	-0.036
Uganda	Kigulu	2000	0.143	0.183	0.234
Uganda	Kigulu	2017	0.066	0.084	0.106
Uganda	Kigulu	2000-2017	-0.090	-0.060	-0.026
Uganda	Kilak	2000	0.155	0.211	0.271
Uganda	Kilak	2017	0.059	0.078	0.099
Uganda	Kilak	2000-2017	-0.106	-0.071	-0.039
Uganda	Kinkiizi	2000	0.119	0.154	0.193
Uganda	Kinkiizi	2017	0.050	0.066	0.088
Uganda	Kinkiizi	2000-2017	-0.093	-0.064	-0.037
Uganda	Kioga	2000	0.150	0.193	0.245
Uganda	Kioga	2017	0.056	0.073	0.090
Uganda	Kioga	2000-2017	-0.104	-0.078	-0.050
Uganda	Kira Municipality	2000	0.108	0.139	0.182
Uganda	Kira Municipality	2017	0.052	0.067	0.083
Uganda	Kira Municipality	2000-2017	-0.089	-0.058	-0.027
Uganda	Kisoro Municipality	2000	0.105	0.140	0.180
Uganda	Kisoro Municipality	2017	0.049	0.062	0.080
Uganda	Kisoro Municipality	2000-2017	-0.093	-0.064	-0.038
Uganda	Kitagwenda	2000	0.111	0.148	0.184
Uganda	Kitagwenda	2017	0.046	0.062	0.080
Uganda	Kitagwenda	2000-2017	-0.094	-0.063	-0.029
Uganda	Kitgum Municipality	2000	0.162	0.222	0.295
Uganda	Kitgum Municipality	2017	0.061	0.079	0.102
Uganda	Kitgum Municipality	2000-2017	-0.106	-0.072	-0.038
Uganda	Koboko	2000	0.141	0.188	0.249
Uganda	Koboko	2017	0.041	0.056	0.073
Uganda	Koboko	2000-2017	-0.128	-0.096	-0.063
Uganda	Koboko Municipality	2000	0.137	0.186	0.247
Uganda	Koboko Municipality	2017	0.041	0.056	0.074
Uganda	Koboko Municipality	2000-2017	-0.126	-0.094	-0.061
Uganda	Kole	2000	0.172	0.228	0.289
Uganda	Kole	2017	0.061	0.079	0.098
Uganda	Kole	2000-2017	-0.112	-0.081	-0.051
Uganda	Kongasis	2000	0.151	0.194	0.250
Uganda	Kongasis	2017	0.051	0.067	0.087
Uganda	Kongasis	2000-2017	-0.114	-0.084	-0.055
Uganda	Kooki	2000	0.131	0.172	0.230
Uganda	Kooki	2017	0.054	0.070	0.090
Uganda	Kooki	2000-2017	-0.092	-0.061	-0.029
Uganda	Kotido Municipality	2000	0.144	0.203	0.267
Uganda	Kotido Municipality	2017	0.051	0.070	0.093
Uganda	Kotido Municipality	2000-2017	-0.107	-0.074	-0.046
Uganda	Kumi	2000	0.155	0.197	0.253
Uganda	Kumi	2017	0.064	0.081	0.103
Uganda	Kumi	2000-2017	-0.100	-0.068	-0.038
Uganda	Kumi Municipality	2000	0.157	0.197	0.254
Uganda	Kumi Municipality	2017	0.063	0.081	0.103
Uganda	Kumi Municipality	2000-2017	-0.096	-0.062	-0.029
Uganda	Kwania	2000	0.161	0.212	0.268
Uganda	Kwania	2017	0.058	0.075	0.094
Uganda	Kwania	2000-2017	-0.110	-0.081	-0.050
Uganda	Kween	2000	0.150	0.196	0.253
Uganda	Kween	2017	0.051	0.067	0.086

Table 2: Diarrhea DALYs rate by unit *(continued)*

Country	Unit	year	mean rate	lower rate	upper rate
Uganda	Kween	2000-2017	-0.116	-0.086	-0.056
Uganda	Kyadondo	2000	0.112	0.141	0.184
Uganda	Kyadondo	2017	0.051	0.066	0.082
Uganda	Kyadondo	2000-2017	-0.089	-0.059	-0.028
Uganda	Kyaka	2000	0.106	0.143	0.185
Uganda	Kyaka	2017	0.044	0.058	0.077
Uganda	Kyaka	2000-2017	-0.082	-0.049	-0.017
Uganda	Kyamuswa	2000	0.110	0.143	0.192
Uganda	Kyamuswa	2017	0.048	0.063	0.082
Uganda	Kyamuswa	2000-2017	-0.089	-0.059	-0.028
Uganda	Kyotera	2000	0.135	0.178	0.237
Uganda	Kyotera	2017	0.053	0.069	0.090
Uganda	Kyotera	2000-2017	-0.091	-0.062	-0.031
Uganda	Labwor	2000	0.139	0.189	0.242
Uganda	Labwor	2017	0.051	0.068	0.087
Uganda	Labwor	2000-2017	-0.114	-0.082	-0.053
Uganda	Lamwo	2000	0.170	0.225	0.295
Uganda	Lamwo	2017	0.056	0.073	0.094
Uganda	Lamwo	2000-2017	-0.116	-0.083	-0.053
Uganda	Lira Municipality	2000	0.163	0.213	0.271
Uganda	Lira Municipality	2017	0.060	0.077	0.097
Uganda	Lira Municipality	2000-2017	-0.107	-0.078	-0.045
Uganda	Lugazi Municipality	2000	0.116	0.149	0.192
Uganda	Lugazi Municipality	2017	0.058	0.073	0.091
Uganda	Lugazi Municipality	2000-2017	-0.090	-0.060	-0.028
Uganda	Luuka	2000	0.138	0.175	0.224
Uganda	Luuka	2017	0.065	0.084	0.105
Uganda	Luuka	2000-2017	-0.089	-0.061	-0.028
Uganda	Lwemiyaga	2000	0.112	0.151	0.195
Uganda	Lwemiyaga	2017	0.047	0.062	0.081
Uganda	Lwemiyaga	2000-2017	-0.087	-0.054	-0.021
Uganda	Madi Okollo	2000	0.148	0.197	0.260
Uganda	Madi Okollo	2017	0.047	0.063	0.084
Uganda	Madi Okollo	2000-2017	-0.119	-0.088	-0.056
Uganda	Makindye Ssabagabo Municipality	2000	0.111	0.143	0.185
Uganda	Makindye Ssabagabo Municipality	2017	0.052	0.067	0.083
Uganda	Makindye Ssabagabo Municipality	2000-2017	-0.093	-0.060	-0.029
Uganda	Manjiya	2000	0.167	0.213	0.270
Uganda	Manjiya	2017	0.055	0.070	0.088
Uganda	Manjiya	2000-2017	-0.122	-0.094	-0.064
Uganda	Maracha	2000	0.133	0.177	0.237
Uganda	Maracha	2017	0.043	0.058	0.076
Uganda	Maracha	2000-2017	-0.116	-0.088	-0.056
Uganda	Maruzi	2000	0.157	0.207	0.268
Uganda	Maruzi	2017	0.055	0.070	0.087
Uganda	Maruzi	2000-2017	-0.108	-0.079	-0.048
Uganda	Masaka Municipality	2000	0.125	0.167	0.222
Uganda	Masaka Municipality	2017	0.051	0.067	0.086
Uganda	Masaka Municipality	2000-2017	-0.094	-0.063	-0.030
Uganda	Masindi Municipality	2000	0.129	0.180	0.236
Uganda	Masindi Municipality	2017	0.041	0.055	0.072
Uganda	Masindi Municipality	2000-2017	-0.115	-0.080	-0.051
Uganda	Matheniko	2000	0.137	0.185	0.246
Uganda	Matheniko	2017	0.053	0.071	0.093
Uganda	Matheniko	2000-2017	-0.079	-0.049	-0.018
Uganda	Mawogola	2000	0.127	0.172	0.222
Uganda	Mawogola	2017	0.050	0.067	0.086
Uganda	Mawogola	2000-2017	-0.094	-0.064	-0.030
Uganda	Mawokota	2000	0.113	0.147	0.190
Uganda	Mawokota	2017	0.048	0.062	0.078
Uganda	Mawokota	2000-2017	-0.090	-0.061	-0.029

Table 2: Diarrhea DALYs rate by unit *(continued)*

Country	Unit	year	mean rate	lower rate	upper rate
Uganda	Mbale Municipality	2000	0.153	0.194	0.242
Uganda	Mbale Municipality	2017	0.061	0.078	0.098
Uganda	Mbale Municipality	2000-2017	-0.102	-0.073	-0.041
Uganda	Mbarara Municipality	2000	0.114	0.154	0.203
Uganda	Mbarara Municipality	2017	0.049	0.065	0.084
Uganda	Mbarara Municipality	2000-2017	-0.101	-0.069	-0.035
Uganda	Mityana	2000	0.110	0.147	0.193
Uganda	Mityana	2017	0.045	0.060	0.076
Uganda	Mityana	2000-2017	-0.087	-0.057	-0.025
Uganda	Mityana Municipality	2000	0.107	0.143	0.191
Uganda	Mityana Municipality	2017	0.044	0.059	0.075
Uganda	Mityana Municipality	2000-2017	-0.092	-0.060	-0.028
Uganda	Moroto	2000	0.176	0.235	0.301
Uganda	Moroto	2017	0.064	0.082	0.101
Uganda	Moroto	2000-2017	-0.123	-0.089	-0.060
Uganda	Moroto Municipality	2000	0.125	0.172	0.234
Uganda	Moroto Municipality	2017	0.053	0.071	0.093
Uganda	Moroto Municipality	2000-2017	-0.082	-0.050	-0.017
Uganda	Mubende Municipality	2000	0.107	0.144	0.188
Uganda	Mubende Municipality	2017	0.044	0.060	0.078
Uganda	Mubende Municipality	2000-2017	-0.076	-0.045	-0.012
Uganda	Mukono	2000	0.112	0.145	0.191
Uganda	Mukono	2017	0.055	0.070	0.086
Uganda	Mukono	2000-2017	-0.092	-0.060	-0.028
Uganda	Mukono Municipality	2000	0.110	0.143	0.189
Uganda	Mukono Municipality	2017	0.053	0.068	0.084
Uganda	Mukono Municipality	2000-2017	-0.091	-0.059	-0.028
Uganda	Mwenge	2000	0.119	0.159	0.206
Uganda	Mwenge	2017	0.046	0.059	0.076
Uganda	Mwenge	2000-2017	-0.098	-0.065	-0.035
Uganda	Nakaseke	2000	0.116	0.151	0.198
Uganda	Nakaseke	2017	0.045	0.060	0.078
Uganda	Nakaseke	2000-2017	-0.090	-0.061	-0.032
Uganda	Nakifuma	2000	0.108	0.137	0.176
Uganda	Nakifuma	2017	0.052	0.068	0.087
Uganda	Nakifuma	2000-2017	-0.082	-0.054	-0.021
Uganda	Nansana Municipality	2000	0.110	0.138	0.182
Uganda	Nansana Municipality	2017	0.050	0.065	0.081
Uganda	Nansana Municipality	2000-2017	-0.087	-0.057	-0.026
Uganda	Ndorwa	2000	0.125	0.159	0.200
Uganda	Ndorwa	2017	0.053	0.067	0.086
Uganda	Ndorwa	2000-2017	-0.092	-0.067	-0.038
Uganda	Nebbi Municipality	2000	0.141	0.185	0.246
Uganda	Nebbi Municipality	2017	0.045	0.060	0.079
Uganda	Nebbi Municipality	2000-2017	-0.120	-0.090	-0.055
Uganda	Ngora	2000	0.158	0.201	0.257
Uganda	Ngora	2017	0.064	0.083	0.104
Uganda	Ngora	2000-2017	-0.103	-0.068	-0.037
Uganda	Njeru Municipality	2000	0.126	0.160	0.203
Uganda	Njeru Municipality	2017	0.059	0.076	0.097
Uganda	Njeru Municipality	2000-2017	-0.098	-0.066	-0.033
Uganda	Ntenjeru	2000	0.113	0.144	0.181
Uganda	Ntenjeru	2017	0.056	0.072	0.091
Uganda	Ntenjeru	2000-2017	-0.083	-0.054	-0.021
Uganda	Ntoroko	2000	0.130	0.180	0.238
Uganda	Ntoroko	2017	0.048	0.063	0.082
Uganda	Ntoroko	2000-2017	-0.114	-0.082	-0.052
Uganda	Ntungamo Municipality	2000	0.106	0.140	0.181
Uganda	Ntungamo Municipality	2017	0.049	0.066	0.088
Uganda	Ntungamo Municipality	2000-2017	-0.087	-0.058	-0.026
Uganda	Nwoya	2000	0.144	0.194	0.248

Table 2: Diarrhea DALYs rate by unit *(continued)*

Country	Unit	year	mean rate	lower rate	upper rate
Uganda	Nwoya	2017	0.054	0.072	0.095
Uganda	Nwoya	2000-2017	-0.103	-0.071	-0.041
Uganda	Nyabushozi	2000	0.119	0.159	0.200
Uganda	Nyabushozi	2017	0.048	0.064	0.082
Uganda	Nyabushozi	2000-2017	-0.100	-0.069	-0.035
Uganda	Obongi	2000	0.147	0.195	0.255
Uganda	Obongi	2017	0.050	0.067	0.090
Uganda	Obongi	2000-2017	-0.117	-0.081	-0.045
Uganda	Okoro	2000	0.134	0.179	0.241
Uganda	Okoro	2017	0.041	0.055	0.073
Uganda	Okoro	2000-2017	-0.125	-0.094	-0.061
Uganda	Omoro	2000	0.148	0.202	0.264
Uganda	Omoro	2017	0.060	0.077	0.099
Uganda	Omoro	2000-2017	-0.102	-0.069	-0.038
Uganda	Otuke	2000	0.161	0.219	0.285
Uganda	Otuke	2017	0.062	0.080	0.101
Uganda	Otuke	2000-2017	-0.114	-0.082	-0.054
Uganda	Oyam	2000	0.160	0.216	0.279
Uganda	Oyam	2017	0.059	0.077	0.096
Uganda	Oyam	2000-2017	-0.103	-0.074	-0.047
Uganda	Padyere	2000	0.142	0.188	0.248
Uganda	Padyere	2017	0.045	0.061	0.080
Uganda	Padyere	2000-2017	-0.120	-0.088	-0.052
Uganda	Pallisa	2000	0.151	0.191	0.242
Uganda	Pallisa	2017	0.063	0.081	0.102
Uganda	Pallisa	2000-2017	-0.099	-0.068	-0.038
Uganda	Pian	2000	0.137	0.186	0.244
Uganda	Pian	2017	0.055	0.072	0.093
Uganda	Pian	2000-2017	-0.088	-0.059	-0.029
Uganda	Pokot	2000	0.161	0.213	0.273
Uganda	Pokot	2017	0.053	0.069	0.088
Uganda	Pokot	2000-2017	-0.110	-0.082	-0.054
Uganda	Rubabo	2000	0.112	0.147	0.185
Uganda	Rubabo	2017	0.050	0.065	0.087
Uganda	Rubabo	2000-2017	-0.089	-0.061	-0.034
Uganda	Rubanda	2000	0.118	0.154	0.197
Uganda	Rubanda	2017	0.053	0.068	0.087
Uganda	Rubanda	2000-2017	-0.092	-0.065	-0.039
Uganda	Ruhaama	2000	0.110	0.142	0.184
Uganda	Ruhaama	2017	0.051	0.066	0.089
Uganda	Ruhaama	2000-2017	-0.089	-0.058	-0.027
Uganda	Ruhinda	2000	0.113	0.150	0.189
Uganda	Ruhinda	2017	0.048	0.062	0.081
Uganda	Ruhinda	2000-2017	-0.093	-0.064	-0.035
Uganda	Rujumbura	2000	0.116	0.153	0.193
Uganda	Rujumbura	2017	0.050	0.064	0.085
Uganda	Rujumbura	2000-2017	-0.095	-0.067	-0.038
Uganda	Rukiga	2000	0.123	0.161	0.205
Uganda	Rukiga	2017	0.053	0.069	0.089
Uganda	Rukiga	2000-2017	-0.093	-0.065	-0.037
Uganda	Rukungiri Municipality	2000	0.111	0.144	0.182
Uganda	Rukungiri Municipality	2017	0.048	0.062	0.083
Uganda	Rukungiri Municipality	2000-2017	-0.096	-0.067	-0.039
Uganda	Rushenyi	2000	0.116	0.150	0.190
Uganda	Rushenyi	2017	0.051	0.067	0.088
Uganda	Rushenyi	2000-2017	-0.089	-0.060	-0.030
Uganda	Rwampara	2000	0.114	0.151	0.195
Uganda	Rwampara	2017	0.050	0.065	0.084
Uganda	Rwampara	2000-2017	-0.093	-0.064	-0.032
Uganda	Samia-Bugwe	2000	0.152	0.193	0.242
Uganda	Samia-Bugwe	2017	0.060	0.079	0.100

Table 2: Diarrhea DALYs rate by unit *(continued)*

Country	Unit	year	mean rate	lower rate	upper rate
Uganda	Samia-Bugwe	2000-2017	-0.091	-0.065	-0.035
Uganda	Serere	2000	0.151	0.199	0.259
Uganda	Serere	2017	0.063	0.082	0.106
Uganda	Serere	2000-2017	-0.103	-0.069	-0.037
Uganda	Sheema	2000	0.109	0.144	0.184
Uganda	Sheema	2017	0.047	0.062	0.081
Uganda	Sheema	2000-2017	-0.093	-0.063	-0.034
Uganda	Sheema Municipality	2000	0.108	0.143	0.187
Uganda	Sheema Municipality	2017	0.048	0.062	0.081
Uganda	Sheema Municipality	2000-2017	-0.091	-0.061	-0.030
Uganda	Soroti	2000	0.158	0.210	0.268
Uganda	Soroti	2017	0.062	0.082	0.103
Uganda	Soroti	2000-2017	-0.104	-0.072	-0.040
Uganda	Soroti Municipality	2000	0.152	0.201	0.259
Uganda	Soroti Municipality	2017	0.062	0.081	0.101
Uganda	Soroti Municipality	2000-2017	-0.102	-0.069	-0.036
Uganda	Terego	2000	0.139	0.186	0.245
Uganda	Terego	2017	0.043	0.060	0.080
Uganda	Terego	2000-2017	-0.120	-0.089	-0.054
Uganda	Tingey	2000	0.149	0.197	0.254
Uganda	Tingey	2017	0.054	0.071	0.088
Uganda	Tingey	2000-2017	-0.114	-0.084	-0.054
Uganda	Toroma	2000	0.149	0.195	0.252
Uganda	Toroma	2017	0.061	0.080	0.100
Uganda	Toroma	2000-2017	-0.102	-0.070	-0.038
Uganda	Tororo	2000	0.164	0.206	0.254
Uganda	Tororo	2017	0.060	0.076	0.096
Uganda	Tororo	2000-2017	-0.105	-0.078	-0.045
Uganda	Tororo Municipality	2000	0.151	0.192	0.242
Uganda	Tororo Municipality	2017	0.058	0.074	0.095
Uganda	Tororo Municipality	2000-2017	-0.101	-0.073	-0.042
Uganda	Usuk	2000	0.153	0.203	0.264
Uganda	Usuk	2017	0.062	0.081	0.100
Uganda	Usuk	2000-2017	-0.103	-0.070	-0.040
Uganda	Vurra	2000	0.147	0.194	0.255
Uganda	Vurra	2017	0.043	0.059	0.080
Uganda	Vurra	2000-2017	-0.126	-0.094	-0.061
Uganda	West Budama	2000	0.153	0.195	0.242
Uganda	West Budama	2017	0.061	0.078	0.099
Uganda	West Budama	2000-2017	-0.095	-0.068	-0.036
Uganda	West Moyo	2000	0.146	0.195	0.262
Uganda	West Moyo	2017	0.051	0.069	0.091
Uganda	West Moyo	2000-2017	-0.114	-0.078	-0.050
Zambia	Chadiza	2000	0.368	0.487	0.636
Zambia	Chadiza	2017	0.096	0.130	0.168
Zambia	Chadiza	2000-2017	-0.131	-0.101	-0.070
Zambia	Chama	2000	0.307	0.399	0.531
Zambia	Chama	2017	0.079	0.114	0.144
Zambia	Chama	2000-2017	-0.114	-0.088	-0.061
Zambia	Chavuma	2000	0.247	0.355	0.489
Zambia	Chavuma	2017	0.072	0.100	0.137
Zambia	Chavuma	2000-2017	-0.116	-0.086	-0.058
Zambia	Chibombo	2000	0.360	0.482	0.640
Zambia	Chibombo	2017	0.085	0.117	0.152
Zambia	Chibombo	2000-2017	-0.126	-0.099	-0.071
Zambia	Chiengi	2000	0.346	0.486	0.671
Zambia	Chiengi	2017	0.088	0.121	0.164
Zambia	Chiengi	2000-2017	-0.128	-0.096	-0.065
Zambia	Chililabombwe	2000	0.334	0.481	0.650
Zambia	Chililabombwe	2017	0.084	0.121	0.167
Zambia	Chililabombwe	2000-2017	-0.135	-0.102	-0.072

Table 2: Diarrhea DALYs rate by unit *(continued)*

Country	Unit	year	mean rate	lower rate	upper rate
Zambia	Chilubi	2000	0.310	0.408	0.558
Zambia	Chilubi	2017	0.077	0.107	0.146
Zambia	Chilubi	2000-2017	-0.130	-0.098	-0.067
Zambia	Chingola	2000	0.310	0.445	0.604
Zambia	Chingola	2017	0.086	0.122	0.169
Zambia	Chingola	2000-2017	-0.126	-0.094	-0.064
Zambia	Chinsali	2000	0.306	0.411	0.550
Zambia	Chinsali	2017	0.083	0.118	0.157
Zambia	Chinsali	2000-2017	-0.115	-0.086	-0.058
Zambia	Chipata	2000	0.351	0.459	0.608
Zambia	Chipata	2017	0.090	0.121	0.153
Zambia	Chipata	2000-2017	-0.132	-0.102	-0.074
Zambia	Choma	2000	0.363	0.498	0.657
Zambia	Choma	2017	0.085	0.118	0.157
Zambia	Choma	2000-2017	-0.130	-0.096	-0.061
Zambia	Chongwe	2000	0.349	0.480	0.627
Zambia	Chongwe	2017	0.086	0.116	0.154
Zambia	Chongwe	2000-2017	-0.132	-0.104	-0.077
Zambia	Gwembe	2000	0.382	0.523	0.703
Zambia	Gwembe	2017	0.085	0.118	0.154
Zambia	Gwembe	2000-2017	-0.135	-0.100	-0.068
Zambia	Isoka	2000	0.297	0.391	0.522
Zambia	Isoka	2017	0.074	0.106	0.137
Zambia	Isoka	2000-2017	-0.123	-0.094	-0.065
Zambia	Itezhi-Tezhi	2000	0.356	0.485	0.667
Zambia	Itezhi-Tezhi	2017	0.090	0.124	0.167
Zambia	Itezhi-Tezhi	2000-2017	-0.122	-0.093	-0.060
Zambia	Kabompo	2000	0.258	0.359	0.505
Zambia	Kabompo	2017	0.067	0.093	0.125
Zambia	Kabompo	2000-2017	-0.125	-0.095	-0.064
Zambia	Kabwe	2000	0.334	0.474	0.636
Zambia	Kabwe	2017	0.078	0.111	0.154
Zambia	Kabwe	2000-2017	-0.136	-0.104	-0.074
Zambia	Kafue	2000	0.345	0.465	0.598
Zambia	Kafue	2017	0.079	0.111	0.143
Zambia	Kafue	2000-2017	-0.133	-0.105	-0.074
Zambia	Kalabo	2000	0.329	0.454	0.612
Zambia	Kalabo	2017	0.078	0.106	0.144
Zambia	Kalabo	2000-2017	-0.133	-0.101	-0.076
Zambia	Kalomo	2000	0.347	0.494	0.657
Zambia	Kalomo	2017	0.088	0.123	0.163
Zambia	Kalomo	2000-2017	-0.120	-0.091	-0.060
Zambia	Kalulushi	2000	0.313	0.435	0.578
Zambia	Kalulushi	2017	0.082	0.116	0.158
Zambia	Kalulushi	2000-2017	-0.131	-0.100	-0.071
Zambia	Kaoma	2000	0.298	0.404	0.555
Zambia	Kaoma	2017	0.075	0.105	0.146
Zambia	Kaoma	2000-2017	-0.125	-0.096	-0.069
Zambia	Kapiri Mposhi	2000	0.368	0.498	0.650
Zambia	Kapiri Mposhi	2017	0.090	0.125	0.161
Zambia	Kapiri Mposhi	2000-2017	-0.129	-0.101	-0.073
Zambia	Kaputa	2000	0.367	0.507	0.677
Zambia	Kaputa	2017	0.087	0.121	0.166
Zambia	Kaputa	2000-2017	-0.128	-0.096	-0.069
Zambia	Kasama	2000	0.306	0.422	0.569
Zambia	Kasama	2017	0.079	0.107	0.143
Zambia	Kasama	2000-2017	-0.125	-0.095	-0.064
Zambia	Kasempa	2000	0.291	0.418	0.562
Zambia	Kasempa	2017	0.086	0.121	0.162
Zambia	Kasempa	2000-2017	-0.117	-0.088	-0.060
Zambia	Katete	2000	0.355	0.490	0.647

Table 2: Diarrhea DALYs rate by unit *(continued)*

Country	Unit	year	mean rate	lower rate	upper rate
Zambia	Katete	2017	0.089	0.124	0.165
Zambia	Katete	2000-2017	-0.132	-0.100	-0.067
Zambia	Kawambwa	2000	0.344	0.468	0.624
Zambia	Kawambwa	2017	0.081	0.111	0.149
Zambia	Kawambwa	2000-2017	-0.135	-0.103	-0.073
Zambia	Kazungula	2000	0.366	0.497	0.653
Zambia	Kazungula	2017	0.090	0.123	0.162
Zambia	Kazungula	2000-2017	-0.118	-0.091	-0.064
Zambia	Kitwe	2000	0.319	0.446	0.584
Zambia	Kitwe	2017	0.082	0.119	0.163
Zambia	Kitwe	2000-2017	-0.130	-0.099	-0.072
Zambia	Livingstone	2000	0.307	0.416	0.564
Zambia	Livingstone	2017	0.071	0.098	0.133
Zambia	Livingstone	2000-2017	-0.128	-0.097	-0.068
Zambia	Luangwa	2000	0.315	0.442	0.593
Zambia	Luangwa	2017	0.085	0.117	0.158
Zambia	Luangwa	2000-2017	-0.125	-0.097	-0.066
Zambia	Luanshya	2000	0.321	0.453	0.593
Zambia	Luanshya	2017	0.083	0.119	0.160
Zambia	Luanshya	2000-2017	-0.132	-0.100	-0.069
Zambia	Lufwanyama	2000	0.333	0.445	0.575
Zambia	Lufwanyama	2017	0.089	0.124	0.163
Zambia	Lufwanyama	2000-2017	-0.125	-0.094	-0.067
Zambia	Lukulu	2000	0.289	0.393	0.544
Zambia	Lukulu	2017	0.075	0.101	0.142
Zambia	Lukulu	2000-2017	-0.120	-0.094	-0.068
Zambia	Lundazi	2000	0.370	0.485	0.621
Zambia	Lundazi	2017	0.094	0.127	0.162
Zambia	Lundazi	2000-2017	-0.129	-0.099	-0.072
Zambia	Lusaka	2000	0.345	0.468	0.625
Zambia	Lusaka	2017	0.081	0.114	0.148
Zambia	Lusaka	2000-2017	-0.130	-0.101	-0.071
Zambia	Luwingu	2000	0.305	0.417	0.544
Zambia	Luwingu	2017	0.079	0.106	0.142
Zambia	Luwingu	2000-2017	-0.127	-0.097	-0.069
Zambia	Mambwe	2000	0.327	0.431	0.564
Zambia	Mambwe	2017	0.084	0.115	0.150
Zambia	Mambwe	2000-2017	-0.127	-0.099	-0.070
Zambia	Mansa	2000	0.308	0.444	0.599
Zambia	Mansa	2017	0.083	0.115	0.152
Zambia	Mansa	2000-2017	-0.135	-0.106	-0.078
Zambia	Masaiti	2000	0.351	0.475	0.629
Zambia	Masaiti	2017	0.087	0.124	0.167
Zambia	Masaiti	2000-2017	-0.133	-0.102	-0.072
Zambia	Mazabuka	2000	0.358	0.497	0.648
Zambia	Mazabuka	2017	0.082	0.114	0.145
Zambia	Mazabuka	2000-2017	-0.131	-0.103	-0.068
Zambia	Mbala	2000	0.338	0.476	0.633
Zambia	Mbala	2017	0.091	0.128	0.173
Zambia	Mbala	2000-2017	-0.119	-0.088	-0.062
Zambia	Milenge	2000	0.362	0.507	0.677
Zambia	Milenge	2017	0.087	0.120	0.162
Zambia	Milenge	2000-2017	-0.140	-0.108	-0.081
Zambia	Mkushi	2000	0.335	0.475	0.660
Zambia	Mkushi	2017	0.090	0.127	0.168
Zambia	Mkushi	2000-2017	-0.131	-0.102	-0.072
Zambia	Mongu	2000	0.304	0.422	0.560
Zambia	Mongu	2017	0.077	0.106	0.145
Zambia	Mongu	2000-2017	-0.131	-0.099	-0.069
Zambia	Monze	2000	0.367	0.501	0.668
Zambia	Monze	2017	0.089	0.120	0.157

Table 2: Diarrhea DALYs rate by unit *(continued)*

Country	Unit	year	mean rate	lower rate	upper rate
Zambia	Monze	2000-2017	-0.131	-0.100	-0.063
Zambia	Mpika	2000	0.329	0.446	0.591
Zambia	Mpika	2017	0.079	0.111	0.152
Zambia	Mpika	2000-2017	-0.129	-0.099	-0.071
Zambia	MPongwe	2000	0.358	0.481	0.637
Zambia	MPongwe	2017	0.092	0.128	0.169
Zambia	MPongwe	2000-2017	-0.130	-0.101	-0.072
Zambia	Mporokoso	2000	0.308	0.421	0.573
Zambia	Mporokoso	2017	0.080	0.108	0.142
Zambia	Mporokoso	2000-2017	-0.126	-0.092	-0.061
Zambia	Mpulungu	2000	0.339	0.475	0.637
Zambia	Mpulungu	2017	0.094	0.128	0.173
Zambia	Mpulungu	2000-2017	-0.119	-0.087	-0.059
Zambia	Mufulira	2000	0.312	0.441	0.597
Zambia	Mufulira	2017	0.085	0.120	0.164
Zambia	Mufulira	2000-2017	-0.128	-0.098	-0.072
Zambia	Mufumbwe	2000	0.287	0.394	0.542
Zambia	Mufumbwe	2017	0.078	0.109	0.151
Zambia	Mufumbwe	2000-2017	-0.115	-0.090	-0.062
Zambia	Mumbwa	2000	0.359	0.485	0.645
Zambia	Mumbwa	2017	0.090	0.124	0.166
Zambia	Mumbwa	2000-2017	-0.119	-0.092	-0.062
Zambia	Mungwi	2000	0.327	0.454	0.602
Zambia	Mungwi	2017	0.085	0.117	0.160
Zambia	Mungwi	2000-2017	-0.122	-0.093	-0.065
Zambia	Mwense	2000	0.322	0.450	0.608
Zambia	Mwense	2017	0.080	0.114	0.150
Zambia	Mwense	2000-2017	-0.136	-0.109	-0.079
Zambia	Mwinilunga	2000	0.292	0.400	0.540
Zambia	Mwinilunga	2017	0.071	0.101	0.137
Zambia	Mwinilunga	2000-2017	-0.121	-0.094	-0.067
Zambia	Nakonde	2000	0.309	0.412	0.540
Zambia	Nakonde	2017	0.077	0.114	0.157
Zambia	Nakonde	2000-2017	-0.124	-0.089	-0.059
Zambia	Namwala	2000	0.329	0.470	0.633
Zambia	Namwala	2017	0.089	0.122	0.161
Zambia	Namwala	2000-2017	-0.119	-0.093	-0.066
Zambia	Nchelenge	2000	0.359	0.487	0.677
Zambia	Nchelenge	2017	0.081	0.115	0.154
Zambia	Nchelenge	2000-2017	-0.139	-0.106	-0.077
Zambia	Ndola	2000	0.322	0.451	0.598
Zambia	Ndola	2017	0.083	0.118	0.159
Zambia	Ndola	2000-2017	-0.128	-0.099	-0.066
Zambia	Nyimba	2000	0.359	0.484	0.648
Zambia	Nyimba	2017	0.085	0.121	0.160
Zambia	Nyimba	2000-2017	-0.133	-0.102	-0.071
Zambia	Petauke	2000	0.318	0.458	0.612
Zambia	Petauke	2017	0.082	0.119	0.159
Zambia	Petauke	2000-2017	-0.125	-0.098	-0.065
Zambia	Samfya	2000	0.310	0.417	0.556
Zambia	Samfya	2017	0.081	0.111	0.148
Zambia	Samfya	2000-2017	-0.132	-0.100	-0.072
Zambia	Senanga	2000	0.312	0.438	0.593
Zambia	Senanga	2017	0.080	0.112	0.158
Zambia	Senanga	2000-2017	-0.129	-0.097	-0.067
Zambia	Serenje	2000	0.329	0.447	0.605
Zambia	Serenje	2017	0.081	0.115	0.156
Zambia	Serenje	2000-2017	-0.130	-0.102	-0.072
Zambia	Sesheke	2000	0.346	0.475	0.640
Zambia	Sesheke	2017	0.083	0.118	0.163
Zambia	Sesheke	2000-2017	-0.121	-0.094	-0.065

Table 2: Diarrhea DALYs rate by unit *(continued)*

Country	Unit	year	mean rate	lower rate	upper rate
Zambia	Shangombo	2000	0.370	0.505	0.663
Zambia	Shangombo	2017	0.091	0.129	0.177
Zambia	Shangombo	2000-2017	-0.123	-0.088	-0.057
Zambia	Siavonga	2000	0.410	0.557	0.738
Zambia	Siavonga	2017	0.083	0.115	0.154
Zambia	Siavonga	2000-2017	-0.142	-0.110	-0.077
Zambia	Sinazongwe	2000	0.335	0.467	0.616
Zambia	Sinazongwe	2017	0.083	0.116	0.154
Zambia	Sinazongwe	2000-2017	-0.125	-0.090	-0.060
Zambia	Solwezi	2000	0.326	0.448	0.595
Zambia	Solwezi	2017	0.087	0.118	0.155
Zambia	Solwezi	2000-2017	-0.125	-0.095	-0.066
Zambia	Zambezi	2000	0.270	0.373	0.502
Zambia	Zambezi	2017	0.073	0.098	0.132
Zambia	Zambezi	2000-2017	-0.117	-0.091	-0.063
Zimbabwe	Beitbridge	2000	0.061	0.089	0.126
Zimbabwe	Beitbridge	2017	0.055	0.074	0.099
Zimbabwe	Beitbridge	2000-2017	-0.028	0.004	0.035
Zimbabwe	Bikita	2000	0.086	0.117	0.156
Zimbabwe	Bikita	2017	0.069	0.095	0.128
Zimbabwe	Bikita	2000-2017	-0.023	0.008	0.044
Zimbabwe	Bindura	2000	0.076	0.106	0.142
Zimbabwe	Bindura	2017	0.077	0.104	0.141
Zimbabwe	Bindura	2000-2017	-0.003	0.026	0.056
Zimbabwe	Binga	2000	0.075	0.106	0.140
Zimbabwe	Binga	2017	0.062	0.088	0.119
Zimbabwe	Binga	2000-2017	-0.017	0.017	0.049
Zimbabwe	Bubi	2000	0.062	0.088	0.120
Zimbabwe	Bubi	2017	0.058	0.076	0.101
Zimbabwe	Bubi	2000-2017	-0.026	0.011	0.044
Zimbabwe	Buhera	2000	0.084	0.115	0.154
Zimbabwe	Buhera	2017	0.071	0.095	0.124
Zimbabwe	Buhera	2000-2017	-0.022	0.007	0.035
Zimbabwe	Bulawayo	2000	0.052	0.073	0.101
Zimbabwe	Bulawayo	2017	0.047	0.063	0.087
Zimbabwe	Bulawayo	2000-2017	-0.023	0.010	0.046
Zimbabwe	Bulilima (North)	2000	0.059	0.079	0.111
Zimbabwe	Bulilima (North)	2017	0.049	0.068	0.089
Zimbabwe	Bulilima (North)	2000-2017	-0.026	0.006	0.041
Zimbabwe	Centenary	2000	0.085	0.115	0.156
Zimbabwe	Centenary	2017	0.075	0.102	0.134
Zimbabwe	Centenary	2000-2017	-0.012	0.017	0.048
Zimbabwe	Chegutu	2000	0.084	0.114	0.152
Zimbabwe	Chegutu	2017	0.079	0.105	0.142
Zimbabwe	Chegutu	2000-2017	-0.011	0.019	0.050
Zimbabwe	Chikomba	2000	0.077	0.110	0.148
Zimbabwe	Chikomba	2017	0.069	0.094	0.126
Zimbabwe	Chikomba	2000-2017	-0.018	0.010	0.040
Zimbabwe	Chimanimani	2000	0.088	0.120	0.162
Zimbabwe	Chimanimani	2017	0.070	0.094	0.124
Zimbabwe	Chimanimani	2000-2017	-0.028	0.003	0.033
Zimbabwe	Chipinge	2000	0.093	0.126	0.173
Zimbabwe	Chipinge	2017	0.074	0.099	0.130
Zimbabwe	Chipinge	2000-2017	-0.016	0.010	0.041
Zimbabwe	Chiredzi	2000	0.078	0.108	0.152
Zimbabwe	Chiredzi	2017	0.072	0.097	0.130
Zimbabwe	Chiredzi	2000-2017	-0.005	0.024	0.057
Zimbabwe	Chirumhanzu	2000	0.078	0.108	0.147
Zimbabwe	Chirumhanzu	2017	0.066	0.091	0.119
Zimbabwe	Chirumhanzu	2000-2017	-0.022	0.009	0.040
Zimbabwe	Chivi	2000	0.074	0.106	0.144

Table 2: Diarrhea DALYs rate by unit *(continued)*

Country	Unit	year	mean rate	lower rate	upper rate
Zimbabwe	Chivi	2017	0.066	0.092	0.121
Zimbabwe	Chivi	2000-2017	-0.019	0.013	0.046
Zimbabwe	Gokwe North	2000	0.094	0.129	0.168
Zimbabwe	Gokwe North	2017	0.073	0.103	0.133
Zimbabwe	Gokwe North	2000-2017	-0.029	0.007	0.038
Zimbabwe	Gokwe South	2000	0.083	0.116	0.155
Zimbabwe	Gokwe South	2017	0.069	0.095	0.123
Zimbabwe	Gokwe South	2000-2017	-0.027	0.009	0.038
Zimbabwe	Goromonzi	2000	0.076	0.105	0.146
Zimbabwe	Goromonzi	2017	0.076	0.100	0.133
Zimbabwe	Goromonzi	2000-2017	-0.001	0.027	0.058
Zimbabwe	Guruve	2000	0.088	0.120	0.157
Zimbabwe	Guruve	2017	0.076	0.102	0.132
Zimbabwe	Guruve	2000-2017	-0.016	0.011	0.041
Zimbabwe	Gutu	2000	0.083	0.113	0.153
Zimbabwe	Gutu	2017	0.070	0.095	0.127
Zimbabwe	Gutu	2000-2017	-0.018	0.010	0.043
Zimbabwe	Gwanda	2000	0.060	0.083	0.113
Zimbabwe	Gwanda	2017	0.052	0.072	0.097
Zimbabwe	Gwanda	2000-2017	-0.032	0.005	0.036
Zimbabwe	Gweru	2000	0.066	0.097	0.130
Zimbabwe	Gweru	2017	0.066	0.089	0.119
Zimbabwe	Gweru	2000-2017	-0.017	0.017	0.051
Zimbabwe	Harare	2000	0.062	0.087	0.117
Zimbabwe	Harare	2017	0.064	0.084	0.114
Zimbabwe	Harare	2000-2017	-0.006	0.024	0.054
Zimbabwe	Hurungwe	2000	0.098	0.135	0.180
Zimbabwe	Hurungwe	2017	0.080	0.108	0.140
Zimbabwe	Hurungwe	2000-2017	-0.026	0.002	0.036
Zimbabwe	Hwange	2000	0.075	0.100	0.133
Zimbabwe	Hwange	2017	0.063	0.083	0.111
Zimbabwe	Hwange	2000-2017	-0.014	0.014	0.045
Zimbabwe	Insiza	2000	0.063	0.088	0.120
Zimbabwe	Insiza	2017	0.056	0.076	0.099
Zimbabwe	Insiza	2000-2017	-0.027	0.007	0.038
Zimbabwe	Kadoma	2000	0.079	0.112	0.151
Zimbabwe	Kadoma	2017	0.076	0.101	0.135
Zimbabwe	Kadoma	2000-2017	-0.013	0.017	0.047
Zimbabwe	Kariba	2000	0.097	0.135	0.174
Zimbabwe	Kariba	2017	0.074	0.099	0.127
Zimbabwe	Kariba	2000-2017	-0.028	0.002	0.033
Zimbabwe	Kwekwe	2000	0.067	0.099	0.134
Zimbabwe	Kwekwe	2017	0.071	0.094	0.121
Zimbabwe	Kwekwe	2000-2017	-0.011	0.022	0.055
Zimbabwe	Lupane	2000	0.064	0.092	0.127
Zimbabwe	Lupane	2017	0.056	0.076	0.104
Zimbabwe	Lupane	2000-2017	-0.030	0.010	0.046
Zimbabwe	Makonde	2000	0.097	0.131	0.173
Zimbabwe	Makonde	2017	0.084	0.112	0.148
Zimbabwe	Makonde	2000-2017	-0.018	0.010	0.045
Zimbabwe	Makoni	2000	0.084	0.116	0.158
Zimbabwe	Makoni	2017	0.074	0.097	0.126
Zimbabwe	Makoni	2000-2017	-0.022	0.005	0.032
Zimbabwe	Mangwe (South)	2000	0.056	0.077	0.103
Zimbabwe	Mangwe (South)	2017	0.049	0.068	0.093
Zimbabwe	Mangwe (South)	2000-2017	-0.024	0.006	0.037
Zimbabwe	Marondera	2000	0.074	0.105	0.141
Zimbabwe	Marondera	2017	0.072	0.097	0.129
Zimbabwe	Marondera	2000-2017	-0.007	0.022	0.052
Zimbabwe	Masvingo	2000	0.075	0.107	0.146
Zimbabwe	Masvingo	2017	0.068	0.095	0.123

Table 2: Diarrhea DALYs rate by unit *(continued)*

Country	Unit	year	mean rate	lower rate	upper rate
Zimbabwe	Masvingo	2000-2017	-0.015	0.016	0.054
Zimbabwe	Matobo	2000	0.059	0.080	0.107
Zimbabwe	Matobo	2017	0.051	0.070	0.093
Zimbabwe	Matobo	2000-2017	-0.023	0.007	0.037
Zimbabwe	Mazowe	2000	0.079	0.109	0.147
Zimbabwe	Mazowe	2017	0.077	0.102	0.138
Zimbabwe	Mazowe	2000-2017	-0.007	0.022	0.052
Zimbabwe	Mberengwa	2000	0.076	0.105	0.146
Zimbabwe	Mberengwa	2017	0.062	0.087	0.117
Zimbabwe	Mberengwa	2000-2017	-0.029	0.004	0.035
Zimbabwe	Mount Darwin	2000	0.076	0.101	0.135
Zimbabwe	Mount Darwin	2017	0.072	0.099	0.131
Zimbabwe	Mount Darwin	2000-2017	-0.008	0.025	0.055
Zimbabwe	Mudzi	2000	0.068	0.093	0.122
Zimbabwe	Mudzi	2017	0.069	0.093	0.122
Zimbabwe	Mudzi	2000-2017	-0.008	0.025	0.053
Zimbabwe	Murehwa	2000	0.072	0.103	0.145
Zimbabwe	Murehwa	2017	0.073	0.096	0.129
Zimbabwe	Murehwa	2000-2017	-0.007	0.025	0.052
Zimbabwe	Mutare	2000	0.093	0.124	0.165
Zimbabwe	Mutare	2017	0.075	0.099	0.132
Zimbabwe	Mutare	2000-2017	-0.030	0.000	0.026
Zimbabwe	Mutasa	2000	0.092	0.123	0.168
Zimbabwe	Mutasa	2017	0.070	0.095	0.126
Zimbabwe	Mutasa	2000-2017	-0.036	-0.005	0.022
Zimbabwe	Mutoko	2000	0.070	0.096	0.130
Zimbabwe	Mutoko	2017	0.071	0.094	0.128
Zimbabwe	Mutoko	2000-2017	-0.006	0.026	0.057
Zimbabwe	Mwenezi	2000	0.076	0.107	0.148
Zimbabwe	Mwenezi	2017	0.066	0.090	0.121
Zimbabwe	Mwenezi	2000-2017	-0.021	0.009	0.039
Zimbabwe	Nkayi	2000	0.066	0.095	0.131
Zimbabwe	Nkayi	2017	0.061	0.082	0.108
Zimbabwe	Nkayi	2000-2017	-0.024	0.012	0.043
Zimbabwe	Nyanga	2000	0.084	0.114	0.152
Zimbabwe	Nyanga	2017	0.069	0.092	0.122
Zimbabwe	Nyanga	2000-2017	-0.027	-0.002	0.027
Zimbabwe	Rushinga	2000	0.067	0.091	0.121
Zimbabwe	Rushinga	2017	0.067	0.092	0.122
Zimbabwe	Rushinga	2000-2017	-0.006	0.026	0.058
Zimbabwe	Seke	2000	0.078	0.111	0.144
Zimbabwe	Seke	2017	0.078	0.101	0.134
Zimbabwe	Seke	2000-2017	-0.009	0.020	0.048
Zimbabwe	Shamva	2000	0.073	0.101	0.139
Zimbabwe	Shamva	2017	0.075	0.100	0.134
Zimbabwe	Shamva	2000-2017	-0.004	0.029	0.058
Zimbabwe	Shurugwi	2000	0.071	0.103	0.137
Zimbabwe	Shurugwi	2017	0.064	0.088	0.116
Zimbabwe	Shurugwi	2000-2017	-0.020	0.011	0.045
Zimbabwe	Tsholotsho	2000	0.062	0.085	0.117
Zimbabwe	Tsholotsho	2017	0.053	0.072	0.099
Zimbabwe	Tsholotsho	2000-2017	-0.025	0.007	0.045
Zimbabwe	Umguza	2000	0.061	0.084	0.117
Zimbabwe	Umguza	2017	0.054	0.072	0.097
Zimbabwe	Umguza	2000-2017	-0.025	0.007	0.040
Zimbabwe	UMP	2000	0.069	0.096	0.130
Zimbabwe	UMP	2017	0.071	0.096	0.126
Zimbabwe	UMP	2000-2017	-0.001	0.030	0.058
Zimbabwe	Umzingwane	2000	0.059	0.084	0.114
Zimbabwe	Umzingwane	2017	0.052	0.072	0.096
Zimbabwe	Umzingwane	2000-2017	-0.027	0.005	0.037

Table 2: Diarrhea DALYs rate by unit *(continued)*

Country	Unit	year	mean rate	lower rate	upper rate
Zimbabwe	Wedza	2000	0.075	0.109	0.148
Zimbabwe	Wedza	2017	0.071	0.096	0.130
Zimbabwe	Wedza	2000-2017	-0.012	0.015	0.046
Zimbabwe	Zaka	2000	0.082	0.114	0.152
Zimbabwe	Zaka	2017	0.072	0.099	0.134
Zimbabwe	Zaka	2000-2017	-0.016	0.015	0.049
Zimbabwe	Zvimba	2000	0.087	0.113	0.150
Zimbabwe	Zvimba	2017	0.077	0.102	0.139
Zimbabwe	Zvimba	2000-2017	-0.010	0.018	0.049
Zimbabwe	Zvishavane	2000	0.072	0.102	0.143
Zimbabwe	Zvishavane	2017	0.064	0.087	0.114
Zimbabwe	Zvishavane	2000-2017	-0.022	0.011	0.047

Table 3: Malaria DALYs rate by unit

Country	Unit	year	mean rate	lower rate	upper rate
**North Africa and Middle East**					
Sudan	Abu Hamad	2000	0.000	0.002	0.005
Sudan	Abu Hamad	2017	0.000	0.002	0.009
Sudan	Abu Hamad	2000-2017	-0.159	0.034	0.222
Sudan	Abu Jubaiyah	2000	0.010	0.056	0.149
Sudan	Abu Jubaiyah	2017	0.003	0.017	0.050
Sudan	Abu Jubaiyah	2000-2017	-0.183	-0.040	0.068
Sudan	Abyei	2000	0.005	0.045	0.137
Sudan	Abyei	2017	0.004	0.022	0.067
Sudan	Abyei	2000-2017	-0.143	-0.017	0.118
Sudan	Ad Damazin	2000	0.002	0.017	0.063
Sudan	Ad Damazin	2017	0.001	0.009	0.030
Sudan	Ad Damazin	2000-2017	-0.227	-0.082	0.057
Sudan	Ad Damer	2000	0.000	0.004	0.013
Sudan	Ad Damer	2017	0.000	0.003	0.013
Sudan	Ad Damer	2000-2017	-0.215	-0.015	0.148
Sudan	Ad Dinder	2000	0.007	0.031	0.090
Sudan	Ad Dinder	2017	0.002	0.017	0.055
Sudan	Ad Dinder	2000-2017	-0.185	-0.040	0.079
Sudan	Ad Douiem	2000	0.005	0.021	0.062
Sudan	Ad Douiem	2017	0.001	0.007	0.032
Sudan	Ad Douiem	2000-2017	-0.197	-0.054	0.125
Sudan	Addabah	2000	0.000	0.002	0.009
Sudan	Addabah	2017	0.000	0.001	0.004
Sudan	Addabah	2000-2017	-0.159	-0.045	0.133
Sudan	Al Deain	2000	0.005	0.045	0.138
Sudan	Al Deain	2017	0.003	0.018	0.049
Sudan	Al Deain	2000-2017	-0.146	-0.037	0.072
Sudan	Al Fasher	2000	0.003	0.018	0.065
Sudan	Al Fasher	2017	0.001	0.009	0.031
Sudan	Al Fasher	2000-2017	-0.192	-0.052	0.072
Sudan	Al Faw	2000	0.009	0.028	0.066
Sudan	Al Faw	2017	0.002	0.011	0.044
Sudan	Al Faw	2000-2017	-0.185	-0.032	0.114
Sudan	Al Fushqa	2000	0.016	0.049	0.118
Sudan	Al Fushqa	2017	0.003	0.020	0.066
Sudan	Al Fushqa	2000-2017	-0.172	-0.028	0.111
Sudan	Al Gadaref	2000	0.027	0.096	0.223
Sudan	Al Gadaref	2017	0.001	0.010	0.029
Sudan	Al Gadaref	2000-2017	-0.259	-0.087	0.042
Sudan	Al Galabat	2000	0.030	0.125	0.340
Sudan	Al Galabat	2017	0.006	0.037	0.110
Sudan	Al Galabat	2000-2017	-0.163	-0.040	0.086
Sudan	Al Gash	2000	0.005	0.023	0.055
Sudan	Al Gash	2017	0.002	0.014	0.041
Sudan	Al Gash	2000-2017	-0.140	0.005	0.135
Sudan	Al Geneina	2000	0.007	0.046	0.143
Sudan	Al Geneina	2017	0.003	0.019	0.070
Sudan	Al Geneina	2000-2017	-0.223	-0.078	0.041
Sudan	Al Gutaina	2000	0.004	0.012	0.032
Sudan	Al Gutaina	2017	0.001	0.006	0.026
Sudan	Al Gutaina	2000-2017	-0.154	-0.025	0.130
Sudan	Al Jabalian	2000	0.012	0.052	0.130
Sudan	Al Jabalian	2017	0.003	0.015	0.051
Sudan	Al Jabalian	2000-2017	-0.143	-0.038	0.090
Sudan	Al Kamlin	2000	0.012	0.032	0.078
Sudan	Al Kamlin	2017	0.001	0.014	0.045
Sudan	Al Kamlin	2000-2017	-0.127	0.000	0.154
Sudan	Al Kurumik	2000	0.006	0.035	0.109
Sudan	Al Kurumik	2017	0.002	0.013	0.057
Sudan	Al Kurumik	2000-2017	-0.215	-0.086	0.065

Table 3: Malaria DALYs rate by unit *(continued)*

Country	Unit	year	mean rate	lower rate	upper rate
Sudan	Al Mahagil	2000	0.006	0.018	0.047
Sudan	Al Mahagil	2017	0.001	0.010	0.045
Sudan	Al Mahagil	2000-2017	-0.190	-0.037	0.134
Sudan	Al Matammah	2000	0.000	0.002	0.006
Sudan	Al Matammah	2017	0.000	0.002	0.007
Sudan	Al Matammah	2000-2017	-0.154	-0.004	0.123
Sudan	Al Rahd	2000	0.011	0.042	0.088
Sudan	Al Rahd	2017	0.002	0.014	0.047
Sudan	Al Rahd	2000-2017	-0.163	-0.036	0.087
Sudan	Al Roseires	2000	0.004	0.034	0.115
Sudan	Al Roseires	2017	0.002	0.016	0.062
Sudan	Al Roseires	2000-2017	-0.234	-0.086	0.059
Sudan	As Salam	2000	0.009	0.051	0.201
Sudan	As Salam	2017	0.003	0.025	0.081
Sudan	As Salam	2000-2017	-0.183	-0.015	0.117
Sudan	Atbara	2000	0.000	0.004	0.016
Sudan	Atbara	2017	0.001	0.004	0.013
Sudan	Atbara	2000-2017	-0.135	-0.001	0.144
Sudan	Bara	2000	0.003	0.022	0.079
Sudan	Bara	2017	0.001	0.007	0.029
Sudan	Bara	2000-2017	-0.220	-0.075	0.096
Sudan	Baw	2000	0.007	0.041	0.132
Sudan	Baw	2017	0.002	0.017	0.062
Sudan	Baw	2000-2017	-0.238	-0.104	0.032
Sudan	Berber	2000	0.000	0.004	0.014
Sudan	Berber	2017	0.000	0.003	0.012
Sudan	Berber	2000-2017	-0.163	-0.005	0.136
Sudan	Buram	2000	0.018	0.074	0.227
Sudan	Buram	2017	0.012	0.051	0.123
Sudan	Buram	2000-2017	-0.133	-0.013	0.107
Sudan	Dilling	2000	0.011	0.077	0.223
Sudan	Dilling	2017	0.003	0.022	0.065
Sudan	Dilling	2000-2017	-0.202	-0.065	0.056
Sudan	Dongola	2000	0.001	0.003	0.007
Sudan	Dongola	2017	0.000	0.003	0.009
Sudan	Dongola	2000-2017	-0.220	-0.061	0.086
Sudan	East al Gazera	2000	0.007	0.018	0.036
Sudan	East al Gazera	2017	0.001	0.007	0.027
Sudan	East al Gazera	2000-2017	-0.165	-0.032	0.107
Sudan	En Nuhud	2000	0.009	0.050	0.150
Sudan	En Nuhud	2017	0.004	0.023	0.061
Sudan	En Nuhud	2000-2017	-0.202	-0.052	0.090
Sudan	Geissan	2000	0.003	0.032	0.098
Sudan	Geissan	2017	0.001	0.013	0.051
Sudan	Geissan	2000-2017	-0.269	-0.127	0.028
Sudan	Ghebeish	2000	0.008	0.042	0.116
Sudan	Ghebeish	2017	0.002	0.022	0.071
Sudan	Ghebeish	2000-2017	-0.189	-0.026	0.108
Sudan	Halayeb	2000	0.000	0.002	0.011
Sudan	Halayeb	2017	0.000	0.001	0.004
Sudan	Halayeb	2000-2017	-0.167	-0.011	0.137
Sudan	Hamashkorieb	2000	0.002	0.012	0.040
Sudan	Hamashkorieb	2017	0.001	0.010	0.039
Sudan	Hamashkorieb	2000-2017	-0.151	0.003	0.160
Sudan	Id El Ghanem	2000	0.011	0.063	0.189
Sudan	Id El Ghanem	2017	0.009	0.036	0.087
Sudan	Id El Ghanem	2000-2017	-0.116	-0.016	0.088
Sudan	Jebrat al Sheikh	2000	0.001	0.007	0.020
Sudan	Jebrat al Sheikh	2017	0.000	0.004	0.016
Sudan	Jebrat al Sheikh	2000-2017	-0.134	-0.004	0.141
Sudan	Kabkabiya	2000	0.003	0.035	0.146

Table 3: Malaria DALYs rate by unit *(continued)*

Country	Unit	year	mean rate	lower rate	upper rate
Sudan	Kabkabiya	2017	0.003	0.017	0.054
Sudan	Kabkabiya	2000-2017	-0.163	-0.038	0.088
Sudan	Kadugli	2000	0.006	0.048	0.146
Sudan	Kadugli	2017	0.002	0.021	0.075
Sudan	Kadugli	2000-2017	-0.188	-0.018	0.144
Sudan	Karary	2000	0.001	0.005	0.012
Sudan	Karary	2017	0.000	0.003	0.010
Sudan	Karary	2000-2017	-0.160	-0.009	0.103
Sudan	Kas	2000	0.002	0.025	0.098
Sudan	Kas	2017	0.002	0.013	0.044
Sudan	Kas	2000-2017	-0.224	-0.053	0.100
Sudan	Kassala	2000	0.008	0.032	0.082
Sudan	Kassala	2017	0.002	0.022	0.076
Sudan	Kassala	2000-2017	-0.196	0.009	0.158
Sudan	Khartoum	2000	0.002	0.005	0.012
Sudan	Khartoum	2017	0.000	0.003	0.013
Sudan	Khartoum	2000-2017	-0.114	0.027	0.194
Sudan	Khartoum Bahri	2000	0.001	0.005	0.012
Sudan	Khartoum Bahri	2017	0.000	0.003	0.011
Sudan	Khartoum Bahri	2000-2017	-0.151	-0.003	0.119
Sudan	Kosti	2000	0.012	0.042	0.096
Sudan	Kosti	2017	0.002	0.011	0.031
Sudan	Kosti	2000-2017	-0.166	-0.042	0.086
Sudan	Kutum	2000	0.002	0.016	0.069
Sudan	Kutum	2017	0.001	0.007	0.026
Sudan	Kutum	2000-2017	-0.145	-0.026	0.114
Sudan	Lagawa	2000	0.013	0.068	0.195
Sudan	Lagawa	2017	0.003	0.029	0.093
Sudan	Lagawa	2000-2017	-0.180	-0.032	0.097
Sudan	Mellit	2000	0.002	0.011	0.041
Sudan	Mellit	2017	0.000	0.004	0.011
Sudan	Mellit	2000-2017	-0.236	-0.074	0.057
Sudan	Merawi	2000	0.000	0.004	0.013
Sudan	Merawi	2017	0.000	0.002	0.008
Sudan	Merawi	2000-2017	-0.173	-0.026	0.128
Sudan	Mukjar	2000	0.011	0.070	0.235
Sudan	Mukjar	2017	0.005	0.026	0.070
Sudan	Mukjar	2000-2017	-0.201	-0.067	0.044
Sudan	Nahr Atbara	2000	0.010	0.030	0.073
Sudan	Nahr Atbara	2017	0.002	0.017	0.052
Sudan	Nahr Atbara	2000-2017	-0.170	-0.009	0.150
Sudan	North al Gazera	2000	0.011	0.030	0.069
Sudan	North al Gazera	2017	0.002	0.013	0.047
Sudan	North al Gazera	2000-2017	-0.156	-0.017	0.137
Sudan	Nyala	2000	0.003	0.030	0.106
Sudan	Nyala	2000	0.003	0.024	0.084
Sudan	Nyala	2017	0.002	0.012	0.041
Sudan	Nyala	2017	0.002	0.011	0.037
Sudan	Nyala	2000-2017	-0.152	-0.030	0.086
Sudan	Nyala	2000-2017	-0.152	-0.026	0.078
Sudan	Omdurman	2000	0.001	0.004	0.011
Sudan	Omdurman	2017	0.000	0.003	0.008
Sudan	Omdurman	2000-2017	-0.116	0.020	0.146
Sudan	Port Sudan	2000	0.001	0.007	0.018
Sudan	Port Sudan	2017	0.001	0.004	0.015
Sudan	Port Sudan	2000-2017	-0.131	0.020	0.178
Sudan	Rashad	2000	0.017	0.064	0.165
Sudan	Rashad	2017	0.002	0.016	0.064
Sudan	Rashad	2000-2017	-0.206	-0.078	0.067
Sudan	Sennar	2000	0.006	0.025	0.063
Sudan	Sennar	2017	0.002	0.011	0.042

Table 3: Malaria DALYs rate by unit *(continued)*

Country	Unit	year	mean rate	lower rate	upper rate
Sudan	Sennar	2000-2017	-0.171	-0.037	0.108
Sudan	Seteet	2000	0.006	0.017	0.044
Sudan	Seteet	2017	0.001	0.011	0.031
Sudan	Seteet	2000-2017	-0.166	-0.008	0.127
Sudan	Sharg En Nile	2000	0.003	0.009	0.022
Sudan	Sharg En Nile	2017	0.000	0.005	0.015
Sudan	Sharg En Nile	2000-2017	-0.111	0.013	0.150
Sudan	Sharq al Gazera	2000	0.007	0.019	0.044
Sudan	Sharq al Gazera	2017	0.001	0.008	0.035
Sudan	Sharq al Gazera	2000-2017	-0.149	-0.011	0.148
Sudan	Sheikan	2000	0.004	0.022	0.063
Sudan	Sheikan	2017	0.001	0.008	0.028
Sudan	Sheikan	2000-2017	-0.208	-0.069	0.076
Sudan	Shendi	2000	0.001	0.003	0.010
Sudan	Shendi	2017	0.000	0.003	0.010
Sudan	Shendi	2000-2017	-0.123	-0.005	0.114
Sudan	Singa	2000	0.007	0.046	0.128
Sudan	Singa	2017	0.004	0.025	0.094
Sudan	Singa	2000-2017	-0.175	-0.035	0.095
Sudan	Sinkat	2000	0.001	0.006	0.017
Sudan	Sinkat	2017	0.000	0.003	0.010
Sudan	Sinkat	2000-2017	-0.165	-0.026	0.114
Sudan	South al Gazera	2000	0.004	0.013	0.030
Sudan	South al Gazera	2017	0.001	0.007	0.024
Sudan	South al Gazera	2000-2017	-0.188	-0.037	0.110
Sudan	South Khartoum	2000	0.003	0.008	0.016
Sudan	South Khartoum	2017	0.000	0.004	0.014
Sudan	South Khartoum	2000-2017	-0.110	0.021	0.181
Sudan	Sowdari	2000	0.001	0.009	0.046
Sudan	Sowdari	2017	0.000	0.005	0.023
Sudan	Sowdari	2000-2017	-0.168	-0.041	0.075
Sudan	Talodi	2000	0.008	0.046	0.147
Sudan	Talodi	2017	0.003	0.018	0.061
Sudan	Talodi	2000-2017	-0.154	-0.020	0.112
Sudan	Tokar	2000	0.001	0.012	0.038
Sudan	Tokar	2017	0.001	0.008	0.021
Sudan	Tokar	2000-2017	-0.175	-0.026	0.129
Sudan	Tulus	2000	0.010	0.081	0.308
Sudan	Tulus	2017	0.008	0.043	0.152
Sudan	Tulus	2000-2017	-0.165	-0.007	0.131
Sudan	Um Al Gura	2000	0.017	0.045	0.089
Sudan	Um Al Gura	2017	0.001	0.015	0.051
Sudan	Um Al Gura	2000-2017	-0.210	-0.053	0.088
Sudan	Um Badda	2000	0.002	0.005	0.012
Sudan	Um Badda	2017	0.000	0.004	0.013
Sudan	Um Badda	2000-2017	-0.104	0.032	0.185
Sudan	Um Kadada	2000	0.004	0.029	0.100
Sudan	Um Kadada	2017	0.002	0.015	0.055
Sudan	Um Kadada	2000-2017	-0.154	-0.036	0.073
Sudan	Um Rawaba	2000	0.009	0.039	0.089
Sudan	Um Rawaba	2017	0.001	0.008	0.033
Sudan	Um Rawaba	2000-2017	-0.237	-0.094	0.054
Sudan	Wadi Halfa	2000	0.000	0.001	0.002
Sudan	Wadi Halfa	2017	0.000	0.001	0.003
Sudan	Wadi Halfa	2000-2017	-0.220	-0.056	0.092
Sudan	Zallingi	2000	0.005	0.047	0.197
Sudan	Zallingi	2017	0.003	0.020	0.067
Sudan	Zallingi	2000-2017	-0.193	-0.055	0.100
**Sub-Saharan Africa**					
Angola	Alto Cauale	2000	0.052	0.256	0.632
Angola	Alto Cauale	2017	0.052	0.134	0.257

Table 3: Malaria DALYs rate by unit *(continued)*

Country	Unit	year	mean rate	lower rate	upper rate
Angola	Alto Cauale	2000-2017	-0.109	-0.037	0.035
Angola	Alto Zambeze	2000	0.048	0.173	0.394
Angola	Alto Zambeze	2017	0.058	0.177	0.336
Angola	Alto Zambeze	2000-2017	-0.047	0.024	0.102
Angola	Ambaca	2000	0.046	0.237	0.663
Angola	Ambaca	2017	0.038	0.114	0.240
Angola	Ambaca	2000-2017	-0.122	-0.045	0.026
Angola	Amboim	2000	0.048	0.308	0.712
Angola	Amboim	2017	0.061	0.197	0.348
Angola	Amboim	2000-2017	-0.110	-0.034	0.041
Angola	Ambriz	2000	0.023	0.190	0.474
Angola	Ambriz	2017	0.022	0.075	0.178
Angola	Ambriz	2000-2017	-0.170	-0.063	0.030
Angola	Ambuila	2000	0.062	0.302	0.615
Angola	Ambuila	2017	0.023	0.095	0.269
Angola	Ambuila	2000-2017	-0.188	-0.080	0.008
Angola	Andulo	2000	0.028	0.177	0.411
Angola	Andulo	2017	0.029	0.080	0.182
Angola	Andulo	2000-2017	-0.115	-0.055	0.027
Angola	Baía Farta	2000	0.008	0.071	0.210
Angola	Baía Farta	2017	0.010	0.042	0.130
Angola	Baía Farta	2000-2017	-0.152	-0.046	0.066
Angola	Bailundo	2000	0.016	0.174	0.449
Angola	Bailundo	2017	0.015	0.041	0.084
Angola	Bailundo	2000-2017	-0.175	-0.098	-0.025
Angola	Balombo	2000	0.016	0.215	0.453
Angola	Balombo	2017	0.033	0.107	0.256
Angola	Balombo	2000-2017	-0.114	-0.041	0.035
Angola	Banga	2000	0.041	0.270	0.666
Angola	Banga	2017	0.092	0.222	0.404
Angola	Banga	2000-2017	-0.084	-0.019	0.062
Angola	Belize	2000	0.089	0.486	1.126
Angola	Belize	2017	0.077	0.349	0.709
Angola	Belize	2000-2017	-0.150	-0.045	0.041
Angola	Bembe	2000	0.094	0.362	0.677
Angola	Bembe	2017	0.035	0.118	0.279
Angola	Bembe	2000-2017	-0.169	-0.087	0.002
Angola	Benguela	2000	0.007	0.056	0.141
Angola	Benguela	2017	0.006	0.025	0.062
Angola	Benguela	2000-2017	-0.133	-0.047	0.049
Angola	Bibala	2000	0.016	0.095	0.418
Angola	Bibala	2017	0.010	0.030	0.063
Angola	Bibala	2000-2017	-0.171	-0.077	0.011
Angola	Bocoio	2000	0.023	0.169	0.403
Angola	Bocoio	2017	0.045	0.123	0.233
Angola	Bocoio	2000-2017	-0.082	-0.012	0.069
Angola	Bolongongo	2000	0.062	0.332	0.810
Angola	Bolongongo	2017	0.098	0.247	0.444
Angola	Bolongongo	2000-2017	-0.085	-0.025	0.053
Angola	Buco Zau	2000	0.124	0.499	1.074
Angola	Buco Zau	2017	0.147	0.422	0.732
Angola	Buco Zau	2000-2017	-0.106	-0.026	0.039
Angola	Buengas	2000	0.094	0.350	0.788
Angola	Buengas	2017	0.109	0.277	0.518
Angola	Buengas	2000-2017	-0.033	0.024	0.089
Angola	Bula Atumba	2000	0.032	0.246	0.641
Angola	Bula Atumba	2017	0.052	0.164	0.355
Angola	Bula Atumba	2000-2017	-0.106	-0.037	0.051
Angola	Bungo	2000	0.045	0.267	0.628
Angola	Bungo	2017	0.023	0.103	0.227
Angola	Bungo	2000-2017	-0.114	-0.014	0.065

Table 3: Malaria DALYs rate by unit *(continued)*

Country	Unit	year	mean rate	lower rate	upper rate
Angola	Caála	2000	0.019	0.190	0.457
Angola	Caála	2017	0.009	0.030	0.062
Angola	Caála	2000-2017	-0.202	-0.102	-0.022
Angola	Cabinda	2000	0.058	0.209	0.451
Angola	Cabinda	2017	0.025	0.084	0.164
Angola	Cabinda	2000-2017	-0.130	-0.047	0.030
Angola	Cacolo	2000	0.024	0.144	0.354
Angola	Cacolo	2017	0.018	0.078	0.180
Angola	Cacolo	2000-2017	-0.119	-0.022	0.053
Angola	Caconda	2000	0.039	0.239	0.532
Angola	Caconda	2017	0.012	0.040	0.087
Angola	Caconda	2000-2017	-0.216	-0.141	-0.050
Angola	Cacuaco	2000	0.003	0.049	0.179
Angola	Cacuaco	2017	0.011	0.035	0.065
Angola	Cacuaco	2000-2017	-0.043	0.030	0.101
Angola	Cacuzo	2000	0.039	0.263	0.572
Angola	Cacuzo	2017	0.122	0.260	0.461
Angola	Cacuzo	2000-2017	-0.059	-0.015	0.055
Angola	Cahama	2000	0.012	0.086	0.279
Angola	Cahama	2017	0.008	0.029	0.074
Angola	Cahama	2000-2017	-0.160	-0.080	0.046
Angola	Caiambambo	2000	0.043	0.206	0.438
Angola	Caiambambo	2017	0.019	0.056	0.130
Angola	Caiambambo	2000-2017	-0.173	-0.105	-0.015
Angola	Calai	2000	0.008	0.081	0.317
Angola	Calai	2017	0.006	0.042	0.111
Angola	Calai	2000-2017	-0.121	-0.017	0.069
Angola	Calandula	2000	0.032	0.221	0.541
Angola	Calandula	2017	0.060	0.149	0.299
Angola	Calandula	2000-2017	-0.106	-0.045	0.023
Angola	Caluquembe	2000	0.030	0.224	0.500
Angola	Caluquembe	2017	0.008	0.032	0.066
Angola	Caluquembe	2000-2017	-0.258	-0.156	-0.073
Angola	Camacuio	2000	0.007	0.070	0.247
Angola	Camacuio	2017	0.005	0.021	0.060
Angola	Camacuio	2000-2017	-0.179	-0.078	0.022
Angola	Camacupa	2000	0.018	0.160	0.450
Angola	Camacupa	2017	0.178	0.310	0.486
Angola	Camacupa	2000-2017	-0.006	0.055	0.149
Angola	Camanongue	2000	0.015	0.119	0.392
Angola	Camanongue	2017	0.042	0.126	0.262
Angola	Camanongue	2000-2017	-0.027	0.046	0.149
Angola	Cambambe	2000	0.053	0.279	0.598
Angola	Cambambe	2017	0.090	0.221	0.375
Angola	Cambambe	2000-2017	-0.079	-0.013	0.045
Angola	Cambulo	2000	0.034	0.250	0.552
Angola	Cambulo	2017	0.043	0.158	0.325
Angola	Cambulo	2000-2017	-0.135	-0.056	0.013
Angola	Cambundi-Catembo	2000	0.030	0.252	0.559
Angola	Cambundi-Catembo	2017	0.038	0.147	0.341
Angola	Cambundi-Catembo	2000-2017	-0.113	-0.007	0.092
Angola	Cameia	2000	0.010	0.087	0.290
Angola	Cameia	2017	0.054	0.163	0.417
Angola	Cameia	2000-2017	-0.010	0.069	0.170
Angola	Cangandala	2000	0.030	0.248	0.525
Angola	Cangandala	2017	0.044	0.142	0.298
Angola	Cangandala	2000-2017	-0.138	-0.042	0.043
Angola	Caombo	2000	0.056	0.246	0.564
Angola	Caombo	2017	0.029	0.124	0.279
Angola	Caombo	2000-2017	-0.161	-0.072	0.000
Angola	Capenda	2000	0.034	0.199	0.457

Table 3: Malaria DALYs rate by unit *(continued)*

Country	Unit	year	mean rate	lower rate	upper rate
Angola	Capenda	2017	0.038	0.107	0.242
Angola	Capenda	2000-2017	-0.105	-0.027	0.037
Angola	Cassongue	2000	0.021	0.211	0.491
Angola	Cassongue	2017	0.045	0.131	0.273
Angola	Cassongue	2000-2017	-0.073	-0.023	0.043
Angola	Catabola	2000	0.018	0.157	0.408
Angola	Catabola	2017	0.153	0.269	0.413
Angola	Catabola	2000-2017	-0.005	0.051	0.131
Angola	Catchiungo	2000	0.009	0.165	0.458
Angola	Catchiungo	2017	0.012	0.041	0.105
Angola	Catchiungo	2000-2017	-0.149	-0.056	0.041
Angola	Caungula	2000	0.029	0.216	0.536
Angola	Caungula	2017	0.025	0.082	0.242
Angola	Caungula	2000-2017	-0.174	-0.085	0.014
Angola	Cazenga	2000	0.001	0.025	0.102
Angola	Cazenga	2017	0.009	0.029	0.065
Angola	Cazenga	2000-2017	-0.027	0.059	0.145
Angola	Cazengo	2000	0.039	0.268	0.638
Angola	Cazengo	2017	0.116	0.238	0.434
Angola	Cazengo	2000-2017	-0.063	-0.014	0.053
Angola	Chibia	2000	0.011	0.110	0.333
Angola	Chibia	2017	0.008	0.030	0.075
Angola	Chibia	2000-2017	-0.212	-0.110	-0.013
Angola	Chicomba	2000	0.031	0.224	0.525
Angola	Chicomba	2017	0.016	0.048	0.110
Angola	Chicomba	2000-2017	-0.258	-0.163	-0.061
Angola	Chinguar	2000	0.011	0.166	0.445
Angola	Chinguar	2017	0.017	0.059	0.130
Angola	Chinguar	2000-2017	-0.118	-0.033	0.078
Angola	Chipindo	2000	0.027	0.196	0.479
Angola	Chipindo	2017	0.015	0.046	0.101
Angola	Chipindo	2000-2017	-0.229	-0.099	-0.007
Angola	Chitato	2000	0.040	0.241	0.557
Angola	Chitato	2017	0.030	0.104	0.224
Angola	Chitato	2000-2017	-0.173	-0.072	0.022
Angola	Chitembo	2000	0.019	0.156	0.372
Angola	Chitembo	2017	0.061	0.172	0.365
Angola	Chitembo	2000-2017	-0.005	0.063	0.140
Angola	Chongoroi	2000	0.038	0.193	0.440
Angola	Chongoroi	2017	0.011	0.035	0.083
Angola	Chongoroi	2000-2017	-0.228	-0.134	-0.049
Angola	Conda	2000	0.033	0.245	0.636
Angola	Conda	2017	0.071	0.186	0.339
Angola	Conda	2000-2017	-0.090	-0.018	0.057
Angola	Cuaba Nzogo	2000	0.037	0.223	0.520
Angola	Cuaba Nzogo	2017	0.049	0.133	0.277
Angola	Cuaba Nzogo	2000-2017	-0.125	-0.058	0.004
Angola	Cuangar	2000	0.021	0.111	0.352
Angola	Cuangar	2017	0.012	0.056	0.151
Angola	Cuangar	2000-2017	-0.140	-0.029	0.078
Angola	Cuango	2000	0.045	0.271	0.617
Angola	Cuango	2017	0.053	0.143	0.336
Angola	Cuango	2000-2017	-0.117	-0.049	0.033
Angola	Cuanhama	2000	0.031	0.136	0.415
Angola	Cuanhama	2017	0.010	0.032	0.073
Angola	Cuanhama	2000-2017	-0.208	-0.128	-0.068
Angola	Cubal	2000	0.041	0.225	0.444
Angola	Cubal	2017	0.020	0.051	0.104
Angola	Cubal	2000-2017	-0.185	-0.118	-0.042
Angola	Cuchi	2000	0.026	0.162	0.423
Angola	Cuchi	2017	0.067	0.169	0.331

Table 3: Malaria DALYs rate by unit *(continued)*

Country	Unit	year	mean rate	lower rate	upper rate
Angola	Cuchi	2000-2017	-0.051	0.040	0.122
Angola	Cuemba	2000	0.025	0.162	0.433
Angola	Cuemba	2017	0.156	0.276	0.459
Angola	Cuemba	2000-2017	-0.009	0.045	0.113
Angola	Cuilo	2000	0.034	0.205	0.491
Angola	Cuilo	2017	0.018	0.069	0.215
Angola	Cuilo	2000-2017	-0.183	-0.086	0.005
Angola	Cuimba	2000	0.093	0.308	0.574
Angola	Cuimba	2017	0.059	0.198	0.421
Angola	Cuimba	2000-2017	-0.126	-0.034	0.025
Angola	Cuito Cuanavale	2000	0.026	0.136	0.364
Angola	Cuito Cuanavale	2017	0.085	0.207	0.366
Angola	Cuito Cuanavale	2000-2017	-0.006	0.080	0.181
Angola	Cunda-dia-Baza	2000	0.046	0.256	0.601
Angola	Cunda-dia-Baza	2017	0.047	0.129	0.263
Angola	Cunda-dia-Baza	2000-2017	-0.134	-0.063	0.002
Angola	Cunhinga	2000	0.013	0.153	0.374
Angola	Cunhinga	2017	0.025	0.086	0.180
Angola	Cunhinga	2000-2017	-0.098	-0.029	0.064
Angola	Curoca	2000	0.009	0.076	0.279
Angola	Curoca	2017	0.003	0.028	0.082
Angola	Curoca	2000-2017	-0.204	-0.072	0.033
Angola	Cuvelai	2000	0.029	0.150	0.383
Angola	Cuvelai	2017	0.013	0.047	0.108
Angola	Cuvelai	2000-2017	-0.217	-0.116	-0.022
Angola	Dala	2000	0.020	0.108	0.293
Angola	Dala	2017	0.032	0.101	0.211
Angola	Dala	2000-2017	-0.044	0.029	0.117
Angola	Damba	2000	0.080	0.293	0.550
Angola	Damba	2017	0.039	0.143	0.302
Angola	Damba	2000-2017	-0.094	-0.029	0.039
Angola	Dande	2000	0.017	0.176	0.459
Angola	Dande	2017	0.026	0.080	0.156
Angola	Dande	2000-2017	-0.093	-0.034	0.030
Angola	Dembos	2000	0.026	0.246	0.556
Angola	Dembos	2017	0.043	0.142	0.331
Angola	Dembos	2000-2017	-0.128	-0.062	0.010
Angola	Dirico	2000	0.016	0.091	0.256
Angola	Dirico	2017	0.010	0.048	0.117
Angola	Dirico	2000-2017	-0.093	-0.005	0.088
Angola	Ebo	2000	0.049	0.264	0.563
Angola	Ebo	2017	0.082	0.216	0.409
Angola	Ebo	2000-2017	-0.093	-0.016	0.065
Angola	Ekunha	2000	0.012	0.157	0.402
Angola	Ekunha	2017	0.010	0.034	0.074
Angola	Ekunha	2000-2017	-0.173	-0.088	-0.007
Angola	Gambos	2000	0.017	0.115	0.362
Angola	Gambos	2017	0.008	0.036	0.118
Angola	Gambos	2000-2017	-0.194	-0.101	0.011
Angola	Ganda	2000	0.035	0.240	0.474
Angola	Ganda	2017	0.015	0.044	0.086
Angola	Ganda	2000-2017	-0.196	-0.125	-0.050
Angola	Golungo Alto	2000	0.054	0.315	0.741
Angola	Golungo Alto	2017	0.165	0.380	0.642
Angola	Golungo Alto	2000-2017	-0.067	-0.003	0.062
Angola	Huambo	2000	0.011	0.157	0.434
Angola	Huambo	2017	0.007	0.030	0.066
Angola	Huambo	2000-2017	-0.187	-0.087	0.007
Angola	Humpata	2000	0.005	0.066	0.269
Angola	Humpata	2017	0.006	0.021	0.046
Angola	Humpata	2000-2017	-0.186	-0.073	0.041

Table 3: Malaria DALYs rate by unit *(continued)*

Country	Unit	year	mean rate	lower rate	upper rate
Angola	Icolo e Bengo	2000	0.017	0.162	0.478
Angola	Icolo e Bengo	2017	0.038	0.107	0.234
Angola	Icolo e Bengo	2000-2017	-0.042	0.026	0.110
Angola	Ingombota	2000	0.001	0.028	0.118
Angola	Ingombota	2017	0.012	0.041	0.098
Angola	Ingombota	2000-2017	-0.027	0.060	0.154
Angola	Jamba	2000	0.031	0.227	0.481
Angola	Jamba	2017	0.021	0.070	0.163
Angola	Jamba	2000-2017	-0.224	-0.105	-0.013
Angola	Kilamba Kiaxi	2000	0.001	0.019	0.079
Angola	Kilamba Kiaxi	2017	0.007	0.022	0.048
Angola	Kilamba Kiaxi	2000-2017	-0.021	0.063	0.153
Angola	Kuito	2000	0.017	0.166	0.430
Angola	Kuito	2017	0.058	0.133	0.258
Angola	Kuito	2000-2017	-0.049	0.017	0.115
Angola	Kuvango	2000	0.025	0.180	0.406
Angola	Kuvango	2017	0.027	0.083	0.193
Angola	Kuvango	2000-2017	-0.139	-0.036	0.070
Angola	Landana	2000	0.071	0.249	0.534
Angola	Landana	2017	0.064	0.227	0.492
Angola	Landana	2000-2017	-0.111	-0.030	0.054
Angola	Léua	2000	0.016	0.121	0.351
Angola	Léua	2017	0.080	0.202	0.410
Angola	Léua	2000-2017	-0.008	0.077	0.207
Angola	Libolo	2000	0.081	0.330	0.610
Angola	Libolo	2017	0.085	0.217	0.387
Angola	Libolo	2000-2017	-0.102	-0.031	0.021
Angola	Lobito	2000	0.011	0.085	0.225
Angola	Lobito	2017	0.021	0.058	0.133
Angola	Lobito	2000-2017	-0.105	-0.011	0.086
Angola	Londuimbale	2000	0.013	0.179	0.435
Angola	Londuimbale	2017	0.018	0.054	0.130
Angola	Londuimbale	2000-2017	-0.169	-0.085	-0.002
Angola	Longonjo	2000	0.022	0.200	0.427
Angola	Longonjo	2017	0.009	0.030	0.066
Angola	Longonjo	2000-2017	-0.199	-0.113	-0.028
Angola	Luau	2000	0.021	0.152	0.448
Angola	Luau	2017	0.093	0.247	0.413
Angola	Luau	2000-2017	-0.038	0.039	0.135
Angola	Lubalo	2000	0.024	0.162	0.380
Angola	Lubalo	2017	0.014	0.061	0.173
Angola	Lubalo	2000-2017	-0.160	-0.067	0.010
Angola	Lubango	2000	0.009	0.094	0.295
Angola	Lubango	2017	0.007	0.022	0.045
Angola	Lubango	2000-2017	-0.181	-0.094	0.009
Angola	Lucala	2000	0.040	0.270	0.610
Angola	Lucala	2017	0.126	0.266	0.456
Angola	Lucala	2000-2017	-0.073	-0.015	0.061
Angola	Lucano	2000	0.011	0.108	0.359
Angola	Lucano	2017	0.056	0.164	0.351
Angola	Lucano	2000-2017	-0.036	0.045	0.162
Angola	Lucapa	2000	0.035	0.227	0.568
Angola	Lucapa	2017	0.031	0.090	0.199
Angola	Lucapa	2000-2017	-0.166	-0.067	0.033
Angola	Luchazes	2000	0.020	0.123	0.345
Angola	Luchazes	2017	0.032	0.105	0.263
Angola	Luchazes	2000-2017	-0.077	0.013	0.086
Angola	Lumbala-Nguimbo	2000	0.032	0.145	0.372
Angola	Lumbala-Nguimbo	2017	0.033	0.118	0.241
Angola	Lumbala-Nguimbo	2000-2017	-0.070	0.001	0.067
Angola	Luquembo	2000	0.033	0.213	0.491

Table 3: Malaria DALYs rate by unit *(continued)*

Country	Unit	year	mean rate	lower rate	upper rate
Angola	Luquembo	2017	0.076	0.192	0.338
Angola	Luquembo	2000-2017	-0.038	0.031	0.101
Angola	M’Banza Congo	2000	0.140	0.381	0.697
Angola	M’Banza Congo	2017	0.071	0.228	0.405
Angola	M’Banza Congo	2000-2017	-0.135	-0.056	-0.003
Angola	Maianga	2000	0.001	0.020	0.086
Angola	Maianga	2017	0.009	0.029	0.067
Angola	Maianga	2000-2017	-0.023	0.065	0.157
Angola	Malanje	2000	0.026	0.239	0.526
Angola	Malanje	2017	0.036	0.112	0.222
Angola	Malanje	2000-2017	-0.140	-0.060	0.023
Angola	Maquela do Zombo	2000	0.090	0.302	0.600
Angola	Maquela do Zombo	2017	0.065	0.202	0.396
Angola	Maquela do Zombo	2000-2017	-0.085	-0.015	0.035
Angola	Marimba	2000	0.031	0.194	0.475
Angola	Marimba	2017	0.032	0.116	0.280
Angola	Marimba	2000-2017	-0.146	-0.061	0.034
Angola	Massango	2000	0.056	0.260	0.619
Angola	Massango	2017	0.049	0.159	0.351
Angola	Massango	2000-2017	-0.122	-0.048	0.031
Angola	Matala	2000	0.025	0.169	0.388
Angola	Matala	2017	0.011	0.036	0.103
Angola	Matala	2000-2017	-0.264	-0.171	-0.082
Angola	Mavinga	2000	0.010	0.097	0.283
Angola	Mavinga	2017	0.012	0.068	0.169
Angola	Mavinga	2000-2017	-0.089	0.004	0.099
Angola	Menongue	2000	0.025	0.140	0.419
Angola	Menongue	2017	0.146	0.246	0.406
Angola	Menongue	2000-2017	0.034	0.098	0.170
Angola	Milunga	2000	0.064	0.372	0.837
Angola	Milunga	2017	0.134	0.346	0.662
Angola	Milunga	2000-2017	-0.041	0.008	0.080
Angola	Moxico	2000	0.024	0.135	0.399
Angola	Moxico	2017	0.043	0.123	0.240
Angola	Moxico	2000-2017	-0.088	0.011	0.073
Angola	Mucaba	2000	0.053	0.289	0.640
Angola	Mucaba	2017	0.026	0.106	0.267
Angola	Mucaba	2000-2017	-0.136	-0.041	0.039
Angola	Mucari	2000	0.032	0.259	0.636
Angola	Mucari	2017	0.050	0.149	0.323
Angola	Mucari	2000-2017	-0.123	-0.039	0.030
Angola	Muconda	2000	0.030	0.150	0.380
Angola	Muconda	2017	0.046	0.150	0.263
Angola	Muconda	2000-2017	-0.059	0.000	0.058
Angola	Mungo	2000	0.019	0.151	0.367
Angola	Mungo	2017	0.012	0.038	0.080
Angola	Mungo	2000-2017	-0.177	-0.104	-0.024
Angola	Mussende	2000	0.050	0.229	0.480
Angola	Mussende	2017	0.057	0.159	0.298
Angola	Mussende	2000-2017	-0.119	-0.031	0.025
Angola	Muxima	2000	0.037	0.223	0.514
Angola	Muxima	2017	0.064	0.166	0.309
Angola	Muxima	2000-2017	-0.060	-0.007	0.057
Angola	N’Zeto	2000	0.055	0.273	0.658
Angola	N’Zeto	2017	0.016	0.074	0.169
Angola	N’Zeto	2000-2017	-0.206	-0.112	-0.045
Angola	Namakunde	2000	0.023	0.106	0.326
Angola	Namakunde	2017	0.007	0.027	0.069
Angola	Namakunde	2000-2017	-0.208	-0.114	-0.042
Angola	Nambuangongo	2000	0.043	0.278	0.647
Angola	Nambuangongo	2017	0.035	0.108	0.252

Table 3: Malaria DALYs rate by unit *(continued)*

Country	Unit	year	mean rate	lower rate	upper rate
Angola	Nambuangongo	2000-2017	-0.175	-0.083	-0.008
Angola	Namibe	2000	0.006	0.046	0.152
Angola	Namibe	2017	0.006	0.017	0.036
Angola	Namibe	2000-2017	-0.160	-0.075	0.011
Angola	Nancova	2000	0.009	0.103	0.338
Angola	Nancova	2017	0.011	0.080	0.219
Angola	Nancova	2000-2017	-0.104	0.009	0.144
Angola	Negage	2000	0.049	0.266	0.679
Angola	Negage	2017	0.026	0.094	0.204
Angola	Negage	2000-2017	-0.136	-0.043	0.044
Angola	Ngonguembo	2000	0.028	0.205	0.515
Angola	Ngonguembo	2017	0.070	0.196	0.382
Angola	Ngonguembo	2000-2017	-0.081	-0.014	0.073
Angola	Nharea	2000	0.027	0.185	0.433
Angola	Nharea	2017	0.124	0.236	0.377
Angola	Nharea	2000-2017	-0.015	0.039	0.123
Angola	Noqui	2000	0.049	0.259	0.536
Angola	Noqui	2017	0.049	0.179	0.352
Angola	Noqui	2000-2017	-0.134	-0.064	-0.004
Angola	Ombadja	2000	0.014	0.087	0.290
Angola	Ombadja	2017	0.010	0.025	0.056
Angola	Ombadja	2000-2017	-0.176	-0.119	-0.052
Angola	Pango Aluquém	2000	0.030	0.237	0.510
Angola	Pango Aluquém	2017	0.085	0.238	0.473
Angola	Pango Aluquém	2000-2017	-0.076	-0.021	0.033
Angola	Porto Amboim	2000	0.046	0.348	0.841
Angola	Porto Amboim	2017	0.066	0.203	0.412
Angola	Porto Amboim	2000-2017	-0.118	-0.031	0.056
Angola	Puri	2000	0.043	0.233	0.609
Angola	Puri	2017	0.030	0.105	0.227
Angola	Puri	2000-2017	-0.133	-0.029	0.053
Angola	Quela	2000	0.038	0.286	0.711
Angola	Quela	2017	0.067	0.169	0.355
Angola	Quela	2000-2017	-0.108	-0.035	0.022
Angola	Quibala	2000	0.057	0.255	0.540
Angola	Quibala	2017	0.076	0.190	0.354
Angola	Quibala	2000-2017	-0.082	-0.023	0.057
Angola	Quiculungo	2000	0.043	0.251	0.662
Angola	Quiculungo	2017	0.078	0.211	0.400
Angola	Quiculungo	2000-2017	-0.081	-0.009	0.082
Angola	Quilenda	2000	0.047	0.312	0.692
Angola	Quilenda	2017	0.076	0.200	0.399
Angola	Quilenda	2000-2017	-0.104	-0.032	0.034
Angola	Quilengues	2000	0.026	0.149	0.390
Angola	Quilengues	2017	0.006	0.022	0.051
Angola	Quilengues	2000-2017	-0.249	-0.142	-0.056
Angola	Quimbele	2000	0.067	0.332	0.735
Angola	Quimbele	2017	0.130	0.314	0.604
Angola	Quimbele	2000-2017	-0.041	0.013	0.088
Angola	Quirima	2000	0.031	0.191	0.497
Angola	Quirima	2017	0.104	0.230	0.387
Angola	Quirima	2000-2017	-0.018	0.043	0.120
Angola	Quitexe	2000	0.076	0.354	0.798
Angola	Quitexe	2017	0.056	0.189	0.426
Angola	Quitexe	2000-2017	-0.126	-0.045	0.039
Angola	Rangel	2000	0.001	0.025	0.104
Angola	Rangel	2017	0.009	0.031	0.074
Angola	Rangel	2000-2017	-0.027	0.061	0.151
Angola	Rivungo	2000	0.018	0.113	0.351
Angola	Rivungo	2017	0.013	0.052	0.131
Angola	Rivungo	2000-2017	-0.115	-0.035	0.071

Table 3: Malaria DALYs rate by unit *(continued)*

Country	Unit	year	mean rate	lower rate	upper rate
Angola	Samba	2000	0.003	0.040	0.189
Angola	Samba	2017	0.011	0.035	0.076
Angola	Samba	2000-2017	-0.031	0.055	0.145
Angola	Samba Cajú	2000	0.036	0.219	0.568
Angola	Samba Cajú	2017	0.078	0.181	0.337
Angola	Samba Cajú	2000-2017	-0.082	-0.019	0.056
Angola	Sambizanga	2000	0.001	0.029	0.118
Angola	Sambizanga	2017	0.011	0.037	0.087
Angola	Sambizanga	2000-2017	-0.033	0.053	0.138
Angola	Sanza Pombo	2000	0.075	0.360	0.802
Angola	Sanza Pombo	2017	0.152	0.291	0.528
Angola	Sanza Pombo	2000-2017	-0.040	0.019	0.104
Angola	Saurimo	2000	0.029	0.171	0.409
Angola	Saurimo	2017	0.013	0.050	0.107
Angola	Saurimo	2000-2017	-0.174	-0.082	-0.008
Angola	Seles	2000	0.026	0.228	0.571
Angola	Seles	2017	0.078	0.186	0.336
Angola	Seles	2000-2017	-0.054	-0.005	0.054
Angola	Songo	2000	0.068	0.330	0.653
Angola	Songo	2017	0.029	0.115	0.313
Angola	Songo	2000-2017	-0.164	-0.065	0.023
Angola	Soyo	2000	0.046	0.275	0.605
Angola	Soyo	2017	0.013	0.046	0.114
Angola	Soyo	2000-2017	-0.216	-0.134	-0.047
Angola	Sumbe	2000	0.016	0.232	0.589
Angola	Sumbe	2017	0.057	0.168	0.299
Angola	Sumbe	2000-2017	-0.098	-0.017	0.079
Angola	Tchicala-Tcholoanga	2000	0.012	0.166	0.464
Angola	Tchicala-Tcholoanga	2017	0.013	0.037	0.087
Angola	Tchicala-Tcholoanga	2000-2017	-0.175	-0.069	0.019
Angola	Tchindjenje	2000	0.028	0.240	0.480
Angola	Tchindjenje	2017	0.014	0.043	0.096
Angola	Tchindjenje	2000-2017	-0.196	-0.115	-0.027
Angola	Tchipungo	2000	0.015	0.138	0.339
Angola	Tchipungo	2017	0.008	0.030	0.074
Angola	Tchipungo	2000-2017	-0.258	-0.155	-0.076
Angola	Tomboco	2000	0.081	0.343	0.763
Angola	Tomboco	2017	0.050	0.145	0.277
Angola	Tomboco	2000-2017	-0.151	-0.088	-0.034
Angola	Tombwa	2000	0.005	0.046	0.129
Angola	Tombwa	2017	0.003	0.017	0.052
Angola	Tombwa	2000-2017	-0.150	-0.049	0.086
Angola	Uíge	2000	0.078	0.318	0.773
Angola	Uíge	2017	0.025	0.096	0.228
Angola	Uíge	2000-2017	-0.156	-0.052	0.032
Angola	Ukuma	2000	0.017	0.211	0.416
Angola	Ukuma	2017	0.012	0.037	0.083
Angola	Ukuma	2000-2017	-0.204	-0.110	-0.028
Angola	Viana	2000	0.003	0.040	0.150
Angola	Viana	2017	0.010	0.028	0.058
Angola	Viana	2000-2017	-0.022	0.050	0.127
Angola	Virei	2000	0.014	0.096	0.283
Angola	Virei	2017	0.007	0.029	0.074
Angola	Virei	2000-2017	-0.183	-0.072	0.016
Angola	Waku Kungo	2000	0.033	0.226	0.444
Angola	Waku Kungo	2017	0.066	0.172	0.334
Angola	Waku Kungo	2000-2017	-0.078	-0.017	0.039
Angola	Xá Muteba	2000	0.029	0.270	0.603
Angola	Xá Muteba	2017	0.054	0.149	0.295
Angola	Xá Muteba	2000-2017	-0.098	-0.033	0.030
Benin	Abomey	2000	0.064	0.196	0.406

Table 3: Malaria DALYs rate by unit *(continued)*

Country	Unit	year	mean rate	lower rate	upper rate
Benin	Abomey	2017	0.053	0.224	0.481
Benin	Abomey	2000-2017	-0.162	-0.036	0.027
Benin	Abomey-Calavi	2000	0.105	0.232	0.416
Benin	Abomey-Calavi	2017	0.031	0.138	0.283
Benin	Abomey-Calavi	2000-2017	-0.101	-0.008	0.062
Benin	Adja-Ouèrè	2000	0.196	0.565	1.043
Benin	Adja-Ouèrè	2017	0.072	0.307	0.600
Benin	Adja-Ouèrè	2000-2017	-0.141	-0.047	0.028
Benin	Adjarra	2000	0.098	0.260	0.463
Benin	Adjarra	2017	0.031	0.126	0.299
Benin	Adjarra	2000-2017	-0.107	-0.017	0.062
Benin	Adjohoun	2000	0.203	0.501	0.891
Benin	Adjohoun	2017	0.060	0.261	0.529
Benin	Adjohoun	2000-2017	-0.122	-0.033	0.039
Benin	Agbangnizoun	2000	0.117	0.350	0.719
Benin	Agbangnizoun	2017	0.100	0.355	0.648
Benin	Agbangnizoun	2000-2017	-0.141	-0.029	0.023
Benin	Aguégués	2000	0.128	0.315	0.559
Benin	Aguégués	2017	0.033	0.161	0.367
Benin	Aguégués	2000-2017	-0.116	-0.017	0.055
Benin	Akpro-Missérété	2000	0.110	0.289	0.515
Benin	Akpro-Missérété	2017	0.032	0.137	0.306
Benin	Akpro-Missérété	2000-2017	-0.120	-0.025	0.050
Benin	Allada	2000	0.192	0.433	0.747
Benin	Allada	2017	0.130	0.353	0.641
Benin	Allada	2000-2017	-0.078	-0.009	0.035
Benin	Aplahoué	2000	0.188	0.448	0.843
Benin	Aplahoué	2017	0.171	0.442	0.753
Benin	Aplahoué	2000-2017	-0.091	-0.016	0.027
Benin	Athiémé	2000	0.162	0.348	0.613
Benin	Athiémé	2017	0.148	0.361	0.611
Benin	Athiémé	2000-2017	-0.054	0.003	0.040
Benin	Avrankou	2000	0.108	0.294	0.521
Benin	Avrankou	2017	0.035	0.144	0.324
Benin	Avrankou	2000-2017	-0.110	-0.021	0.057
Benin	Banikoara	2000	0.184	0.663	1.288
Benin	Banikoara	2017	0.110	0.470	0.921
Benin	Banikoara	2000-2017	-0.195	-0.053	0.017
Benin	Bantè	2000	0.227	0.490	0.923
Benin	Bantè	2017	0.187	0.433	0.775
Benin	Bantè	2000-2017	-0.072	-0.017	0.021
Benin	Bassila	2000	0.119	0.310	0.607
Benin	Bassila	2017	0.146	0.306	0.496
Benin	Bassila	2000-2017	-0.072	-0.012	0.033
Benin	Bembéréké	2000	0.051	0.244	0.458
Benin	Bembéréké	2017	0.039	0.195	0.380
Benin	Bembéréké	2000-2017	-0.174	-0.041	0.051
Benin	Bohicon	2000	0.050	0.155	0.323
Benin	Bohicon	2017	0.033	0.166	0.380
Benin	Bohicon	2000-2017	-0.160	-0.043	0.036
Benin	Bonou	2000	0.167	0.529	0.967
Benin	Bonou	2017	0.066	0.298	0.629
Benin	Bonou	2000-2017	-0.152	-0.045	0.021
Benin	Bopa	2000	0.178	0.425	0.740
Benin	Bopa	2017	0.138	0.372	0.650
Benin	Bopa	2000-2017	-0.064	0.001	0.045
Benin	Boukoumbé	2000	0.136	0.468	0.781
Benin	Boukoumbé	2017	0.104	0.369	0.744
Benin	Boukoumbé	2000-2017	-0.162	-0.053	0.001
Benin	Cobly	2000	0.213	0.670	1.100
Benin	Cobly	2017	0.128	0.472	1.062

Table 3: Malaria DALYs rate by unit *(continued)*

Country	Unit	year	mean rate	lower rate	upper rate
Benin	Cobly	2000-2017	-0.167	-0.058	-0.008
Benin	Comè	2000	0.120	0.377	0.702
Benin	Comè	2017	0.102	0.353	0.654
Benin	Comè	2000-2017	-0.057	0.027	0.092
Benin	Copargo	2000	0.085	0.316	0.580
Benin	Copargo	2017	0.074	0.280	0.494
Benin	Copargo	2000-2017	-0.151	-0.042	0.016
Benin	Cotonou	2000	0.062	0.178	0.368
Benin	Cotonou	2017	0.013	0.083	0.219
Benin	Cotonou	2000-2017	-0.121	-0.010	0.077
Benin	Covè	2000	0.085	0.292	0.601
Benin	Covè	2017	0.039	0.207	0.506
Benin	Covè	2000-2017	-0.194	-0.060	0.029
Benin	Dangbo	2000	0.304	0.632	1.115
Benin	Dangbo	2017	0.073	0.301	0.629
Benin	Dangbo	2000-2017	-0.119	-0.029	0.042
Benin	Dassa-Zoumè	2000	0.091	0.277	0.528
Benin	Dassa-Zoumè	2017	0.050	0.205	0.434
Benin	Dassa-Zoumè	2000-2017	-0.148	-0.051	0.018
Benin	Djakotomey	2000	0.105	0.270	0.535
Benin	Djakotomey	2017	0.099	0.302	0.535
Benin	Djakotomey	2000-2017	-0.085	-0.007	0.038
Benin	Djidja	2000	0.146	0.383	0.716
Benin	Djidja	2017	0.099	0.339	0.617
Benin	Djidja	2000-2017	-0.124	-0.035	0.015
Benin	Djougou	2000	0.081	0.235	0.466
Benin	Djougou	2017	0.084	0.237	0.430
Benin	Djougou	2000-2017	-0.114	-0.032	0.017
Benin	Dogbo	2000	0.156	0.359	0.661
Benin	Dogbo	2017	0.140	0.377	0.642
Benin	Dogbo	2000-2017	-0.070	-0.004	0.034
Benin	Glazoué	2000	0.102	0.295	0.570
Benin	Glazoué	2017	0.066	0.226	0.445
Benin	Glazoué	2000-2017	-0.131	-0.042	0.020
Benin	Gogounou	2000	0.083	0.393	0.833
Benin	Gogounou	2017	0.050	0.278	0.542
Benin	Gogounou	2000-2017	-0.177	-0.048	0.051
Benin	Grand-Popo	2000	0.118	0.374	0.713
Benin	Grand-Popo	2017	0.111	0.346	0.603
Benin	Grand-Popo	2000-2017	-0.040	0.026	0.083
Benin	Houéyogbé	2000	0.170	0.404	0.702
Benin	Houéyogbé	2017	0.121	0.350	0.610
Benin	Houéyogbé	2000-2017	-0.062	0.009	0.062
Benin	Ifangni	2000	0.160	0.425	0.751
Benin	Ifangni	2017	0.054	0.210	0.443
Benin	Ifangni	2000-2017	-0.113	-0.026	0.051
Benin	Kalalé	2000	0.072	0.314	0.643
Benin	Kalalé	2017	0.079	0.261	0.492
Benin	Kalalé	2000-2017	-0.134	-0.031	0.045
Benin	Kandi	2000	0.129	0.592	1.184
Benin	Kandi	2017	0.115	0.437	0.863
Benin	Kandi	2000-2017	-0.170	-0.045	0.037
Benin	Karimama	2000	0.123	0.437	0.946
Benin	Karimama	2017	0.102	0.384	0.754
Benin	Karimama	2000-2017	-0.131	-0.037	0.046
Benin	Kérou	2000	0.084	0.303	0.554
Benin	Kérou	2017	0.056	0.194	0.375
Benin	Kérou	2000-2017	-0.156	-0.060	0.016
Benin	Kétou	2000	0.135	0.473	0.904
Benin	Kétou	2017	0.059	0.251	0.568
Benin	Kétou	2000-2017	-0.169	-0.064	0.017

Table 3: Malaria DALYs rate by unit *(continued)*

Country	Unit	year	mean rate	lower rate	upper rate
Benin	Klouékanmè	2000	0.103	0.281	0.576
Benin	Klouékanmè	2017	0.096	0.291	0.522
Benin	Klouékanmè	2000-2017	-0.117	-0.021	0.031
Benin	Kouandé	2000	0.071	0.233	0.411
Benin	Kouandé	2017	0.050	0.185	0.349
Benin	Kouandé	2000-2017	-0.154	-0.055	0.005
Benin	Kpomassè	2000	0.149	0.437	0.793
Benin	Kpomassè	2017	0.088	0.346	0.637
Benin	Kpomassè	2000-2017	-0.068	0.015	0.078
Benin	Lalo	2000	0.182	0.438	0.805
Benin	Lalo	2017	0.158	0.438	0.759
Benin	Lalo	2000-2017	-0.082	-0.013	0.029
Benin	Malanville	2000	0.103	0.606	1.294
Benin	Malanville	2017	0.183	0.568	1.037
Benin	Malanville	2000-2017	-0.136	-0.028	0.047
Benin	Matéri	2000	0.147	0.521	0.938
Benin	Matéri	2017	0.103	0.356	0.771
Benin	Matéri	2000-2017	-0.179	-0.070	-0.003
Benin	N'Dali	2000	0.075	0.224	0.446
Benin	N'Dali	2017	0.051	0.200	0.388
Benin	N'Dali	2000-2017	-0.148	-0.038	0.029
Benin	Natitingou	2000	0.067	0.246	0.455
Benin	Natitingou	2017	0.061	0.204	0.380
Benin	Natitingou	2000-2017	-0.159	-0.056	0.004
Benin	Nikki	2000	0.065	0.269	0.544
Benin	Nikki	2017	0.053	0.210	0.406
Benin	Nikki	2000-2017	-0.149	-0.034	0.050
Benin	Ouaké	2000	0.106	0.335	0.679
Benin	Ouaké	2017	0.122	0.318	0.554
Benin	Ouaké	2000-2017	-0.124	-0.033	0.020
Benin	Ouèssè	2000	0.100	0.320	0.652
Benin	Ouèssè	2017	0.074	0.220	0.454
Benin	Ouèssè	2000-2017	-0.121	-0.039	0.026
Benin	Ouidah	2000	0.145	0.388	0.687
Benin	Ouidah	2017	0.070	0.267	0.500
Benin	Ouidah	2000-2017	-0.068	0.010	0.075
Benin	Ouinhi	2000	0.138	0.433	0.863
Benin	Ouinhi	2017	0.057	0.258	0.545
Benin	Ouinhi	2000-2017	-0.172	-0.055	0.019
Benin	Parakou	2000	0.027	0.136	0.294
Benin	Parakou	2017	0.027	0.145	0.316
Benin	Parakou	2000-2017	-0.126	-0.028	0.078
Benin	Péhunco	2000	0.053	0.257	0.455
Benin	Péhunco	2017	0.041	0.199	0.402
Benin	Péhunco	2000-2017	-0.181	-0.051	0.017
Benin	Pèrèrè	2000	0.065	0.245	0.484
Benin	Pèrèrè	2017	0.067	0.210	0.383
Benin	Pèrèrè	2000-2017	-0.132	-0.033	0.043
Benin	Pobè	2000	0.210	0.652	1.245
Benin	Pobè	2017	0.083	0.342	0.711
Benin	Pobè	2000-2017	-0.149	-0.055	0.028
Benin	Porto-Novo	2000	0.085	0.226	0.402
Benin	Porto-Novo	2017	0.019	0.093	0.235
Benin	Porto-Novo	2000-2017	-0.118	-0.022	0.059
Benin	Sakété	2000	0.168	0.473	0.834
Benin	Sakété	2017	0.060	0.248	0.492
Benin	Sakété	2000-2017	-0.120	-0.035	0.040
Benin	Savalou	2000	0.165	0.388	0.741
Benin	Savalou	2017	0.109	0.330	0.583
Benin	Savalou	2000-2017	-0.092	-0.032	0.011
Benin	Savè	2000	0.093	0.274	0.497

Table 3: Malaria DALYs rate by unit *(continued)*

Country	Unit	year	mean rate	lower rate	upper rate
Benin	Savè	2017	0.056	0.180	0.395
Benin	Savè	2000-2017	-0.138	-0.052	0.020
Benin	Segbana	2000	0.036	0.296	0.646
Benin	Segbana	2017	0.068	0.254	0.481
Benin	Segbana	2000-2017	-0.113	-0.024	0.054
Benin	Sèmè-Kpodji	2000	0.096	0.255	0.459
Benin	Sèmè-Kpodji	2017	0.029	0.144	0.346
Benin	Sèmè-Kpodji	2000-2017	-0.113	-0.008	0.077
Benin	Sinendé	2000	0.070	0.289	0.500
Benin	Sinendé	2017	0.043	0.212	0.434
Benin	Sinendé	2000-2017	-0.170	-0.050	0.027
Benin	Sô-Ava	2000	0.082	0.205	0.389
Benin	Sô-Ava	2017	0.026	0.131	0.300
Benin	Sô-Ava	2000-2017	-0.103	-0.005	0.073
Benin	Tanguiéta	2000	0.112	0.331	0.606
Benin	Tanguiéta	2017	0.064	0.220	0.445
Benin	Tanguiéta	2000-2017	-0.165	-0.067	-0.005
Benin	Tchaourou	2000	0.085	0.276	0.577
Benin	Tchaourou	2017	0.087	0.232	0.426
Benin	Tchaourou	2000-2017	-0.099	-0.028	0.028
Benin	Toffo	2000	0.208	0.461	0.821
Benin	Toffo	2017	0.129	0.384	0.712
Benin	Toffo	2000-2017	-0.093	-0.018	0.025
Benin	Tori-Bossito	2000	0.190	0.392	0.676
Benin	Tori-Bossito	2017	0.091	0.280	0.484
Benin	Tori-Bossito	2000-2017	-0.073	-0.006	0.042
Benin	Toucountouna	2000	0.078	0.262	0.478
Benin	Toucountouna	2017	0.052	0.195	0.368
Benin	Toucountouna	2000-2017	-0.159	-0.061	0.006
Benin	Toviklin	2000	0.128	0.319	0.621
Benin	Toviklin	2017	0.129	0.348	0.616
Benin	Toviklin	2000-2017	-0.083	-0.010	0.041
Benin	Za-Kpota	2000	0.091	0.286	0.590
Benin	Za-Kpota	2017	0.043	0.235	0.524
Benin	Za-Kpota	2000-2017	-0.168	-0.051	0.023
Benin	Zagnanado	2000	0.090	0.301	0.614
Benin	Zagnanado	2017	0.036	0.196	0.486
Benin	Zagnanado	2000-2017	-0.196	-0.061	0.028
Benin	Zè	2000	0.267	0.550	0.941
Benin	Zè	2017	0.106	0.342	0.629
Benin	Zè	2000-2017	-0.102	-0.026	0.020
Benin	Zogbodomey	2000	0.154	0.416	0.813
Benin	Zogbodomey	2017	0.080	0.312	0.587
Benin	Zogbodomey	2000-2017	-0.123	-0.034	0.021
Botswana	Barolong	2000	0.000	0.000	0.000
Botswana	Barolong	2017	0.000	0.000	0.000
Botswana	Barolong	2000-2017	NA	NA	NA
Botswana	Bobonong	2000	0.000	0.001	0.003
Botswana	Bobonong	2017	0.000	0.001	0.002
Botswana	Bobonong	2000-2017	-0.212	-0.025	0.126
Botswana	Chobe	2000	0.001	0.003	0.005
Botswana	Chobe	2017	0.000	0.001	0.004
Botswana	Chobe	2000-2017	-0.218	-0.073	0.046
Botswana	Francistown	2000	0.000	0.000	0.001
Botswana	Francistown	2017	0.000	0.000	0.001
Botswana	Francistown	2000-2017	-0.244	-0.034	0.127
Botswana	Gaborone	2000	0.000	0.000	0.000
Botswana	Gaborone	2017	0.000	0.000	0.000
Botswana	Gaborone	2000-2017	NA	NA	NA
Botswana	Gemsbok	2000	0.000	0.000	0.000
Botswana	Gemsbok	2017	0.000	0.000	0.000

Table 3: Malaria DALYs rate by unit *(continued)*

Country	Unit	year	mean rate	lower rate	upper rate
Botswana	Gemsbok	2000-2017	NA	NA	NA
Botswana	Ghanzi	2000	0.001	0.003	0.011
Botswana	Ghanzi	2017	0.000	0.002	0.005
Botswana	Ghanzi	2000-2017	-0.161	-0.045	0.053
Botswana	Hukunsti	2000	0.000	0.000	0.000
Botswana	Hukunsti	2017	0.000	0.000	0.000
Botswana	Hukunsti	2000-2017	-0.186	-0.052	0.080
Botswana	Jwaneng	2000	0.000	0.000	0.000
Botswana	Jwaneng	2017	0.000	0.000	0.000
Botswana	Jwaneng	2000-2017	NA	NA	NA
Botswana	Kgatleng	2000	0.000	0.000	0.000
Botswana	Kgatleng	2017	0.000	0.000	0.000
Botswana	Kgatleng	2000-2017	-0.231	-0.063	0.094
Botswana	Kweneng North	2000	0.000	0.000	0.000
Botswana	Kweneng North	2017	0.000	0.000	0.000
Botswana	Kweneng North	2000-2017	-0.269	-0.080	0.074
Botswana	Kweneng South	2000	0.000	0.000	0.000
Botswana	Kweneng South	2017	0.000	0.000	0.000
Botswana	Kweneng South	2000-2017	-0.217	-0.055	0.130
Botswana	Lethlakane	2000	0.000	0.001	0.002
Botswana	Lethlakane	2017	0.000	0.001	0.002
Botswana	Lethlakane	2000-2017	-0.210	-0.039	0.126
Botswana	Lobatse	2000	0.000	0.000	0.000
Botswana	Lobatse	2017	0.000	0.000	0.000
Botswana	Lobatse	2000-2017	NA	NA	NA
Botswana	Machaneng	2000	0.000	0.002	0.005
Botswana	Machaneng	2017	0.000	0.001	0.003
Botswana	Machaneng	2000-2017	-0.258	-0.055	0.099
Botswana	Mahalapye	2000	0.000	0.002	0.005
Botswana	Mahalapye	2017	0.000	0.001	0.002
Botswana	Mahalapye	2000-2017	-0.232	-0.063	0.084
Botswana	Masungu	2000	0.000	0.001	0.002
Botswana	Masungu	2017	0.000	0.001	0.002
Botswana	Masungu	2000-2017	-0.238	-0.030	0.139
Botswana	Ngamiland East	2000	0.000	0.002	0.006
Botswana	Ngamiland East	2017	0.000	0.001	0.003
Botswana	Ngamiland East	2000-2017	-0.183	-0.049	0.063
Botswana	Ngamiland West	2000	0.001	0.003	0.009
Botswana	Ngamiland West	2017	0.000	0.002	0.006
Botswana	Ngamiland West	2000-2017	-0.165	-0.027	0.100
Botswana	Ngwaketse Central	2000	0.000	0.000	0.000
Botswana	Ngwaketse Central	2017	0.000	0.000	0.000
Botswana	Ngwaketse Central	2000-2017	NA	NA	NA
Botswana	Ngwaketse North	2000	0.000	0.000	0.000
Botswana	Ngwaketse North	2017	0.000	0.000	0.000
Botswana	Ngwaketse North	2000-2017	NA	NA	NA
Botswana	Ngwaketse South	2000	0.000	0.000	0.000
Botswana	Ngwaketse South	2017	0.000	0.000	0.000
Botswana	Ngwaketse South	2000-2017	NA	NA	NA
Botswana	Palapye	2000	0.000	0.001	0.005
Botswana	Palapye	2017	0.000	0.001	0.002
Botswana	Palapye	2000-2017	-0.243	-0.045	0.103
Botswana	Selibe Phikwe	2000	0.000	0.000	0.002
Botswana	Selibe Phikwe	2017	0.000	0.000	0.001
Botswana	Selibe Phikwe	2000-2017	-0.227	-0.031	0.173
Botswana	Serowe	2000	0.000	0.001	0.004
Botswana	Serowe	2017	0.000	0.001	0.002
Botswana	Serowe	2000-2017	-0.225	-0.049	0.089
Botswana	South East	2000	0.000	0.000	0.000
Botswana	South East	2017	0.000	0.000	0.000
Botswana	South East	2000-2017	NA	NA	NA

Table 3: Malaria DALYs rate by unit *(continued)*

Country	Unit	year	mean rate	lower rate	upper rate
Botswana	Sowa	2000	0.000	0.001	0.004
Botswana	Sowa	2017	0.000	0.001	0.003
Botswana	Sowa	2000-2017	-0.278	-0.058	0.126
Botswana	Tshabong	2000	0.000	0.000	0.000
Botswana	Tshabong	2017	0.000	0.000	0.000
Botswana	Tshabong	2000-2017	NA	NA	NA
Botswana	Tuli	2000	0.001	0.003	0.008
Botswana	Tuli	2017	0.000	0.001	0.005
Botswana	Tuli	2000-2017	-0.200	-0.030	0.112
Botswana	Tutume	2000	0.001	0.002	0.005
Botswana	Tutume	2017	0.000	0.001	0.004
Botswana	Tutume	2000-2017	-0.199	-0.048	0.094
Burkina Faso	Balé	2000	0.543	1.009	1.697
Burkina Faso	Balé	2017	0.258	0.692	1.263
Burkina Faso	Balé	2000-2017	-0.123	-0.043	0.004
Burkina Faso	Bam	2000	0.310	0.556	0.941
Burkina Faso	Bam	2017	0.142	0.349	0.675
Burkina Faso	Bam	2000-2017	-0.133	-0.045	-0.002
Burkina Faso	Banwa	2000	0.695	1.302	2.075
Burkina Faso	Banwa	2017	0.421	0.971	1.699
Burkina Faso	Banwa	2000-2017	-0.087	-0.026	0.012
Burkina Faso	Bazèga	2000	0.641	1.194	2.000
Burkina Faso	Bazèga	2017	0.223	0.593	1.336
Burkina Faso	Bazèga	2000-2017	-0.145	-0.063	0.003
Burkina Faso	Bougouriba	2000	0.379	0.718	1.208
Burkina Faso	Bougouriba	2017	0.140	0.395	0.722
Burkina Faso	Bougouriba	2000-2017	-0.159	-0.061	-0.010
Burkina Faso	Boulgou	2000	0.626	1.190	1.902
Burkina Faso	Boulgou	2017	0.237	0.650	1.410
Burkina Faso	Boulgou	2000-2017	-0.138	-0.065	-0.012
Burkina Faso	Boulkiemdé	2000	0.567	1.017	1.724
Burkina Faso	Boulkiemdé	2017	0.140	0.539	1.324
Burkina Faso	Boulkiemdé	2000-2017	-0.155	-0.066	-0.004
Burkina Faso	Comoé	2000	0.300	0.546	0.903
Burkina Faso	Comoé	2017	0.145	0.339	0.771
Burkina Faso	Comoé	2000-2017	-0.136	-0.053	0.001
Burkina Faso	Ganzourgou	2000	0.475	0.890	1.502
Burkina Faso	Ganzourgou	2017	0.170	0.507	1.123
Burkina Faso	Ganzourgou	2000-2017	-0.139	-0.054	0.003
Burkina Faso	Gnagna	2000	0.282	0.554	0.938
Burkina Faso	Gnagna	2017	0.173	0.420	0.851
Burkina Faso	Gnagna	2000-2017	-0.113	-0.036	0.004
Burkina Faso	Gourma	2000	0.524	1.197	2.036
Burkina Faso	Gourma	2017	0.479	1.005	1.806
Burkina Faso	Gourma	2000-2017	-0.106	-0.025	0.016
Burkina Faso	Houet	2000	0.481	0.864	1.408
Burkina Faso	Houet	2017	0.169	0.442	0.996
Burkina Faso	Houet	2000-2017	-0.139	-0.064	-0.008
Burkina Faso	Ioba	2000	0.514	0.993	1.684
Burkina Faso	Ioba	2017	0.243	0.565	1.063
Burkina Faso	Ioba	2000-2017	-0.125	-0.058	-0.007
Burkina Faso	Kadiogo	2000	0.263	0.461	0.757
Burkina Faso	Kadiogo	2017	0.034	0.125	0.297
Burkina Faso	Kadiogo	2000-2017	-0.188	-0.104	-0.009
Burkina Faso	Kénédougou	2000	0.380	0.705	1.175
Burkina Faso	Kénédougou	2017	0.246	0.543	1.018
Burkina Faso	Kénédougou	2000-2017	-0.102	-0.030	0.011
Burkina Faso	Komandjoari	2000	0.258	0.615	1.132
Burkina Faso	Komandjoari	2017	0.181	0.496	0.962
Burkina Faso	Komandjoari	2000-2017	-0.101	-0.029	0.020
Burkina Faso	Kompienga	2000	0.413	0.945	1.563

Table 3: Malaria DALYs rate by unit *(continued)*

Country	Unit	year	mean rate	lower rate	upper rate
Burkina Faso	Kompienga	2017	0.254	0.679	1.323
Burkina Faso	Kompienga	2000-2017	-0.140	-0.040	0.000
Burkina Faso	Kossi	2000	0.584	1.063	1.789
Burkina Faso	Kossi	2017	0.413	0.837	1.408
Burkina Faso	Kossi	2000-2017	-0.070	-0.015	0.004
Burkina Faso	Koulpélogo	2000	0.557	1.194	1.966
Burkina Faso	Koulpélogo	2017	0.414	0.908	1.707
Burkina Faso	Koulpélogo	2000-2017	-0.105	-0.035	0.000
Burkina Faso	Kouritenga	2000	0.466	0.942	1.566
Burkina Faso	Kouritenga	2017	0.228	0.606	1.312
Burkina Faso	Kouritenga	2000-2017	-0.126	-0.051	0.007
Burkina Faso	Kourwéogo	2000	0.421	0.740	1.211
Burkina Faso	Kourwéogo	2017	0.088	0.293	0.637
Burkina Faso	Kourwéogo	2000-2017	-0.188	-0.087	-0.010
Burkina Faso	Léraba	2000	0.287	0.527	0.898
Burkina Faso	Léraba	2017	0.135	0.318	0.807
Burkina Faso	Léraba	2000-2017	-0.154	-0.063	0.000
Burkina Faso	Loroum	2000	0.225	0.472	0.766
Burkina Faso	Loroum	2017	0.146	0.407	0.714
Burkina Faso	Loroum	2000-2017	-0.110	-0.027	0.002
Burkina Faso	Mouhoun	2000	0.639	1.174	1.964
Burkina Faso	Mouhoun	2017	0.376	0.827	1.613
Burkina Faso	Mouhoun	2000-2017	-0.097	-0.035	0.005
Burkina Faso	Nahouri	2000	0.510	0.955	1.578
Burkina Faso	Nahouri	2017	0.114	0.375	0.846
Burkina Faso	Nahouri	2000-2017	-0.169	-0.089	-0.027
Burkina Faso	Namentenga	2000	0.314	0.540	0.905
Burkina Faso	Namentenga	2017	0.182	0.401	0.778
Burkina Faso	Namentenga	2000-2017	-0.103	-0.033	0.002
Burkina Faso	Nayala	2000	0.610	1.111	1.870
Burkina Faso	Nayala	2017	0.268	0.758	1.474
Burkina Faso	Nayala	2000-2017	-0.139	-0.040	-0.001
Burkina Faso	Noumbiel	2000	0.262	0.520	0.888
Burkina Faso	Noumbiel	2017	0.176	0.392	0.712
Burkina Faso	Noumbiel	2000-2017	-0.096	-0.033	0.003
Burkina Faso	Oubritenga	2000	0.379	0.658	1.063
Burkina Faso	Oubritenga	2017	0.064	0.237	0.549
Burkina Faso	Oubritenga	2000-2017	-0.172	-0.085	-0.001
Burkina Faso	Oudalan	2000	0.124	0.551	1.245
Burkina Faso	Oudalan	2017	0.198	0.540	1.062
Burkina Faso	Oudalan	2000-2017	-0.089	-0.015	0.045
Burkina Faso	Passoré	2000	0.566	0.998	1.707
Burkina Faso	Passoré	2017	0.160	0.544	1.172
Burkina Faso	Passoré	2000-2017	-0.165	-0.060	-0.001
Burkina Faso	Poni	2000	0.315	0.594	0.995
Burkina Faso	Poni	2017	0.178	0.416	0.742
Burkina Faso	Poni	2000-2017	-0.105	-0.036	-0.003
Burkina Faso	Sanguié	2000	0.587	1.048	1.792
Burkina Faso	Sanguié	2017	0.219	0.686	1.472
Burkina Faso	Sanguié	2000-2017	-0.150	-0.050	-0.004
Burkina Faso	Sanmatenga	2000	0.319	0.578	0.948
Burkina Faso	Sanmatenga	2017	0.122	0.317	0.609
Burkina Faso	Sanmatenga	2000-2017	-0.126	-0.053	-0.004
Burkina Faso	Séno	2000	0.203	0.651	1.288
Burkina Faso	Séno	2017	0.254	0.559	1.161
Burkina Faso	Séno	2000-2017	-0.093	-0.025	0.021
Burkina Faso	Sissili	2000	0.586	1.082	1.810
Burkina Faso	Sissili	2017	0.310	0.684	1.112
Burkina Faso	Sissili	2000-2017	-0.112	-0.047	-0.007
Burkina Faso	Soum	2000	0.243	0.537	0.906
Burkina Faso	Soum	2017	0.277	0.531	0.890

Table 3: Malaria DALYs rate by unit *(continued)*

Country	Unit	year	mean rate	lower rate	upper rate
Burkina Faso	Soum	2000-2017	-0.068	-0.010	0.012
Burkina Faso	Sourou	2000	0.464	0.815	1.318
Burkina Faso	Sourou	2017	0.199	0.576	1.080
Burkina Faso	Sourou	2000-2017	-0.125	-0.034	-0.001
Burkina Faso	Tapoa	2000	0.319	0.941	1.734
Burkina Faso	Tapoa	2017	0.285	0.742	1.527
Burkina Faso	Tapoa	2000-2017	-0.111	-0.035	0.010
Burkina Faso	Tuy	2000	0.543	1.005	1.676
Burkina Faso	Tuy	2017	0.214	0.590	1.189
Burkina Faso	Tuy	2000-2017	-0.137	-0.058	0.001
Burkina Faso	Yagha	2000	0.144	0.491	0.997
Burkina Faso	Yagha	2017	0.103	0.337	0.835
Burkina Faso	Yagha	2000-2017	-0.166	-0.044	0.019
Burkina Faso	Yatenga	2000	0.249	0.483	0.775
Burkina Faso	Yatenga	2017	0.111	0.327	0.653
Burkina Faso	Yatenga	2000-2017	-0.133	-0.047	0.000
Burkina Faso	Ziro	2000	0.537	1.000	1.664
Burkina Faso	Ziro	2017	0.255	0.656	1.145
Burkina Faso	Ziro	2000-2017	-0.104	-0.042	0.004
Burkina Faso	Zondoma	2000	0.427	0.724	1.204
Burkina Faso	Zondoma	2017	0.127	0.436	0.901
Burkina Faso	Zondoma	2000-2017	-0.165	-0.054	0.001
Burkina Faso	Zoundwéogo	2000	0.604	1.168	1.936
Burkina Faso	Zoundwéogo	2017	0.241	0.619	1.434
Burkina Faso	Zoundwéogo	2000-2017	-0.137	-0.066	-0.005
Burundi	Bisoro	2000	0.020	0.072	0.122
Burundi	Bisoro	2017	0.004	0.011	0.025
Burundi	Bisoro	2000-2017	-0.111	-0.070	-0.026
Burundi	Bubanza	2000	0.173	0.854	1.638
Burundi	Bubanza	2017	0.072	0.210	0.447
Burundi	Bubanza	2000-2017	-0.103	-0.036	0.017
Burundi	Bugabira	2000	0.320	1.124	2.150
Burundi	Bugabira	2017	0.246	0.537	1.016
Burundi	Bugabira	2000-2017	-0.036	-0.002	0.049
Burundi	Buganda	2000	0.145	0.917	1.968
Burundi	Buganda	2017	0.097	0.280	0.620
Burundi	Buganda	2000-2017	-0.087	-0.028	0.017
Burundi	Bugarama	2000	0.153	0.614	1.196
Burundi	Bugarama	2017	0.018	0.051	0.101
Burundi	Bugarama	2000-2017	-0.202	-0.140	-0.077
Burundi	Bugendana	2000	0.453	1.116	2.047
Burundi	Bugendana	2017	0.122	0.306	0.685
Burundi	Bugendana	2000-2017	-0.056	-0.028	0.002
Burundi	Bugenyuzi	2000	0.586	1.162	2.060
Burundi	Bugenyuzi	2017	0.175	0.372	0.733
Burundi	Bugenyuzi	2000-2017	-0.054	-0.035	-0.010
Burundi	Buhiga	2000	0.687	1.263	2.184
Burundi	Buhiga	2017	0.215	0.439	0.883
Burundi	Buhiga	2000-2017	-0.061	-0.037	-0.014
Burundi	Buhinyuza	2000	0.555	1.054	1.806
Burundi	Buhinyuza	2017	0.181	0.384	0.795
Burundi	Buhinyuza	2000-2017	-0.076	-0.042	-0.017
Burundi	Bukemba	2000	0.355	1.000	1.744
Burundi	Bukemba	2017	0.108	0.263	0.594
Burundi	Bukemba	2000-2017	-0.121	-0.076	-0.038
Burundi	Bukeye	2000	0.174	0.724	1.427
Burundi	Bukeye	2017	0.044	0.135	0.324
Burundi	Bukeye	2000-2017	-0.086	-0.039	0.012
Burundi	Bukinanyana	2000	0.109	0.604	1.331
Burundi	Bukinanyana	2017	0.061	0.182	0.371
Burundi	Bukinanyana	2000-2017	-0.074	-0.021	0.026

Table 3: Malaria DALYs rate by unit *(continued)*

Country	Unit	year	mean rate	lower rate	upper rate
Burundi	Bukirasazi	2000	0.113	0.331	0.573
Burundi	Bukirasazi	2017	0.021	0.058	0.120
Burundi	Bukirasazi	2000-2017	-0.126	-0.074	-0.036
Burundi	Burambi	2000	0.225	0.759	1.376
Burundi	Burambi	2017	0.030	0.086	0.168
Burundi	Burambi	2000-2017	-0.175	-0.119	-0.063
Burundi	Buraza	2000	0.133	0.372	0.650
Burundi	Buraza	2017	0.021	0.057	0.115
Burundi	Buraza	2000-2017	-0.129	-0.080	-0.043
Burundi	Bururi	2000	0.045	0.218	0.412
Burundi	Bururi	2017	0.008	0.024	0.049
Burundi	Bururi	2000-2017	-0.167	-0.114	-0.052
Burundi	Busiga	2000	0.152	0.663	1.252
Burundi	Busiga	2017	0.085	0.225	0.502
Burundi	Busiga	2000-2017	-0.051	-0.007	0.037
Burundi	Busoni	2000	0.362	1.020	1.915
Burundi	Busoni	2017	0.172	0.374	0.705
Burundi	Busoni	2000-2017	-0.056	-0.028	0.012
Burundi	Butaganzwa1	2000	0.223	0.930	1.784
Burundi	Butaganzwa1	2017	0.071	0.209	0.508
Burundi	Butaganzwa1	2000-2017	-0.073	-0.023	0.022
Burundi	Butaganzwa2	2000	0.446	0.890	1.514
Burundi	Butaganzwa2	2017	0.115	0.276	0.562
Burundi	Butaganzwa2	2000-2017	-0.087	-0.044	-0.018
Burundi	Buterere	2000	0.132	0.561	1.189
Burundi	Buterere	2017	0.026	0.073	0.155
Burundi	Buterere	2000-2017	-0.145	-0.087	-0.031
Burundi	Butezi	2000	0.372	0.671	1.131
Burundi	Butezi	2017	0.089	0.211	0.393
Burundi	Butezi	2000-2017	-0.068	-0.040	-0.021
Burundi	Butihinda	2000	0.499	1.075	1.924
Burundi	Butihinda	2017	0.201	0.411	0.820
Burundi	Butihinda	2000-2017	-0.061	-0.035	-0.005
Burundi	Buyengero	2000	0.216	0.782	1.434
Burundi	Buyengero	2017	0.031	0.090	0.178
Burundi	Buyengero	2000-2017	-0.167	-0.114	-0.057
Burundi	Buyenze	2000	0.083	0.396	0.900
Burundi	Buyenze	2017	0.017	0.045	0.098
Burundi	Buyenze	2000-2017	-0.153	-0.091	-0.032
Burundi	Bwambarangwe	2000	0.427	0.940	1.669
Burundi	Bwambarangwe	2017	0.182	0.372	0.682
Burundi	Bwambarangwe	2000-2017	-0.054	-0.029	0.007
Burundi	Bweru	2000	0.474	0.869	1.459
Burundi	Bweru	2017	0.120	0.290	0.550
Burundi	Bweru	2000-2017	-0.083	-0.052	-0.024
Burundi	Bwiza	2000	0.083	0.394	0.892
Burundi	Bwiza	2017	0.016	0.044	0.094
Burundi	Bwiza	2000-2017	-0.156	-0.094	-0.034
Burundi	Cankuzo	2000	0.466	0.864	1.443
Burundi	Cankuzo	2017	0.139	0.325	0.626
Burundi	Cankuzo	2000-2017	-0.079	-0.051	-0.025
Burundi	Cendajuru	2000	0.597	1.105	1.817
Burundi	Cendajuru	2017	0.191	0.417	0.794
Burundi	Cendajuru	2000-2017	-0.092	-0.054	-0.025
Burundi	Cibitoke	2000	0.083	0.397	0.879
Burundi	Cibitoke	2017	0.017	0.048	0.103
Burundi	Cibitoke	2000-2017	-0.148	-0.088	-0.029
Burundi	Gahombo	2000	0.243	0.974	1.830
Burundi	Gahombo	2017	0.095	0.277	0.656
Burundi	Gahombo	2000-2017	-0.065	-0.012	0.034
Burundi	Gashikanwa	2000	0.239	0.669	1.203

Table 3: Malaria DALYs rate by unit *(continued)*

Country	Unit	year	mean rate	lower rate	upper rate
Burundi	Gashikanwa	2017	0.086	0.204	0.428
Burundi	Gashikanwa	2000-2017	-0.064	-0.030	0.000
Burundi	Gashoho	2000	0.363	0.916	1.695
Burundi	Gashoho	2017	0.177	0.341	0.637
Burundi	Gashoho	2000-2017	-0.068	-0.037	-0.005
Burundi	Gasorwe	2000	0.457	1.052	1.901
Burundi	Gasorwe	2017	0.175	0.353	0.710
Burundi	Gasorwe	2000-2017	-0.079	-0.046	-0.020
Burundi	Gatara	2000	0.223	0.932	1.812
Burundi	Gatara	2017	0.091	0.263	0.612
Burundi	Gatara	2000-2017	-0.061	-0.013	0.027
Burundi	Gihanga	2000	0.228	1.014	1.996
Burundi	Gihanga	2017	0.068	0.196	0.419
Burundi	Gihanga	2000-2017	-0.129	-0.063	-0.024
Burundi	Giharo	2000	0.445	0.804	1.369
Burundi	Giharo	2017	0.124	0.276	0.527
Burundi	Giharo	2000-2017	-0.089	-0.056	-0.032
Burundi	Giheta	2000	0.423	1.159	2.127
Burundi	Giheta	2017	0.103	0.264	0.620
Burundi	Giheta	2000-2017	-0.072	-0.040	-0.006
Burundi	Gihogazi	2000	0.463	1.040	1.800
Burundi	Gihogazi	2017	0.124	0.306	0.643
Burundi	Gihogazi	2000-2017	-0.056	-0.027	-0.001
Burundi	Gihosha	2000	0.085	0.395	0.847
Burundi	Gihosha	2017	0.017	0.048	0.104
Burundi	Gihosha	2000-2017	-0.143	-0.088	-0.029
Burundi	Gisagara	2000	0.417	0.762	1.293
Burundi	Gisagara	2017	0.132	0.290	0.560
Burundi	Gisagara	2000-2017	-0.085	-0.054	-0.020
Burundi	Gishubi	2000	0.299	0.970	1.604
Burundi	Gishubi	2017	0.064	0.167	0.379
Burundi	Gishubi	2000-2017	-0.117	-0.069	-0.035
Burundi	Gisozi	2000	0.000	0.000	0.000
Burundi	Gisozi	2017	0.000	0.000	0.000
Burundi	Gisozi	2000-2017	NA	NA	NA
Burundi	Gisuru	2000	0.516	0.940	1.560
Burundi	Gisuru	2017	0.132	0.325	0.624
Burundi	Gisuru	2000-2017	-0.085	-0.054	-0.026
Burundi	Gitanga	2000	0.313	0.892	1.571
Burundi	Gitanga	2017	0.060	0.166	0.353
Burundi	Gitanga	2000-2017	-0.138	-0.089	-0.047
Burundi	Gitaramuka	2000	0.648	1.377	2.425
Burundi	Gitaramuka	2017	0.220	0.458	0.917
Burundi	Gitaramuka	2000-2017	-0.064	-0.042	-0.020
Burundi	Gitega	2000	0.399	1.120	1.907
Burundi	Gitega	2017	0.106	0.252	0.568
Burundi	Gitega	2000-2017	-0.090	-0.045	-0.015
Burundi	Giteranyi	2000	0.364	0.859	1.576
Burundi	Giteranyi	2017	0.142	0.321	0.561
Burundi	Giteranyi	2000-2017	-0.052	-0.023	0.020
Burundi	Gitobe	2000	0.454	1.105	1.971
Burundi	Gitobe	2017	0.239	0.488	0.928
Burundi	Gitobe	2000-2017	-0.046	-0.022	0.010
Burundi	Isale	2000	0.160	0.712	1.431
Burundi	Isale	2017	0.029	0.084	0.186
Burundi	Isale	2000-2017	-0.137	-0.087	-0.027
Burundi	Itaba	2000	0.303	0.896	1.543
Burundi	Itaba	2017	0.093	0.246	0.519
Burundi	Itaba	2000-2017	-0.099	-0.046	-0.007
Burundi	Kabarore	2000	0.107	0.512	1.049
Burundi	Kabarore	2017	0.069	0.187	0.422

Table 3: Malaria DALYs rate by unit *(continued)*

Country	Unit	year	mean rate	lower rate	upper rate
Burundi	Kabarore	2000-2017	-0.050	-0.005	0.037
Burundi	Kabezi	2000	0.255	1.101	2.358
Burundi	Kabezi	2017	0.041	0.117	0.252
Burundi	Kabezi	2000-2017	-0.167	-0.108	-0.055
Burundi	Kamenge	2000	0.085	0.394	0.845
Burundi	Kamenge	2017	0.017	0.048	0.104
Burundi	Kamenge	2000-2017	-0.143	-0.087	-0.029
Burundi	Kanyosha1	2000	0.195	0.816	1.702
Burundi	Kanyosha1	2017	0.030	0.086	0.194
Burundi	Kanyosha1	2000-2017	-0.156	-0.100	-0.043
Burundi	Kanyosha2	2000	0.170	0.698	1.415
Burundi	Kanyosha2	2017	0.030	0.082	0.178
Burundi	Kanyosha2	2000-2017	-0.161	-0.098	-0.043
Burundi	Kayanza	2000	0.173	0.748	1.408
Burundi	Kayanza	2017	0.093	0.253	0.567
Burundi	Kayanza	2000-2017	-0.049	-0.006	0.033
Burundi	Kayogoro	2000	0.311	0.784	1.383
Burundi	Kayogoro	2017	0.068	0.179	0.385
Burundi	Kayogoro	2000-2017	-0.141	-0.091	-0.051
Burundi	Kayokwe	2000	0.172	0.567	0.987
Burundi	Kayokwe	2017	0.036	0.094	0.216
Burundi	Kayokwe	2000-2017	-0.104	-0.062	-0.017
Burundi	Kibago	2000	0.434	1.156	2.013
Burundi	Kibago	2017	0.075	0.222	0.488
Burundi	Kibago	2000-2017	-0.165	-0.108	-0.063
Burundi	Kigamba	2000	0.418	0.784	1.309
Burundi	Kigamba	2017	0.161	0.344	0.668
Burundi	Kigamba	2000-2017	-0.069	-0.040	-0.015
Burundi	Kiganda	2000	0.124	0.523	1.007
Burundi	Kiganda	2017	0.026	0.077	0.189
Burundi	Kiganda	2000-2017	-0.104	-0.057	-0.004
Burundi	Kinama	2000	0.149	0.602	1.205
Burundi	Kinama	2017	0.029	0.082	0.180
Burundi	Kinama	2000-2017	-0.135	-0.082	-0.027
Burundi	Kinindo	2000	0.098	0.420	0.909
Burundi	Kinindo	2017	0.014	0.038	0.081
Burundi	Kinindo	2000-2017	-0.178	-0.113	-0.054
Burundi	Kinyinya	2000	0.443	0.811	1.349
Burundi	Kinyinya	2017	0.152	0.318	0.597
Burundi	Kinyinya	2000-2017	-0.079	-0.048	-0.029
Burundi	Kiremba	2000	0.325	0.982	1.778
Burundi	Kiremba	2017	0.159	0.338	0.658
Burundi	Kiremba	2000-2017	-0.065	-0.035	0.000
Burundi	Kirundo	2000	0.367	1.169	2.150
Burundi	Kirundo	2017	0.185	0.404	0.808
Burundi	Kirundo	2000-2017	-0.064	-0.034	0.002
Burundi	Lake Tanganyika	2000	0.453	1.298	2.230
Burundi	Lake Tanganyika	2000	0.401	1.243	2.205
Burundi	Lake Tanganyika	2000	0.104	0.442	0.958
Burundi	Lake Tanganyika	2000	0.234	0.934	1.909
Burundi	Lake Tanganyika	2017	0.052	0.164	0.362
Burundi	Lake Tanganyika	2017	0.037	0.101	0.210
Burundi	Lake Tanganyika	2017	0.041	0.129	0.270
Burundi	Lake Tanganyika	2017	0.016	0.045	0.096
Burundi	Lake Tanganyika	2000-2017	-0.212	-0.141	-0.091
Burundi	Lake Tanganyika	2000-2017	-0.197	-0.140	-0.078
Burundi	Lake Tanganyika	2000-2017	-0.177	-0.114	-0.064
Burundi	Lake Tanganyika	2000-2017	-0.169	-0.105	-0.047
Burundi	Mabanda	2000	0.321	0.946	1.691
Burundi	Mabanda	2017	0.047	0.145	0.318
Burundi	Mabanda	2000-2017	-0.179	-0.121	-0.074

Table 3: Malaria DALYs rate by unit *(continued)*

Country	Unit	year	mean rate	lower rate	upper rate
Burundi	Mabayi	2000	0.078	0.446	1.056
Burundi	Mabayi	2017	0.053	0.158	0.318
Burundi	Mabayi	2000-2017	-0.066	-0.018	0.032
Burundi	Makamba	2000	0.290	0.900	1.602
Burundi	Makamba	2017	0.047	0.142	0.314
Burundi	Makamba	2000-2017	-0.156	-0.111	-0.063
Burundi	Makebuko	2000	0.336	1.027	1.734
Burundi	Makebuko	2017	0.085	0.215	0.487
Burundi	Makebuko	2000-2017	-0.109	-0.059	-0.022
Burundi	Marangara	2000	0.228	0.753	1.356
Burundi	Marangara	2017	0.141	0.286	0.528
Burundi	Marangara	2000-2017	-0.061	-0.027	0.007
Burundi	Matana	2000	0.002	0.009	0.017
Burundi	Matana	2017	0.000	0.001	0.002
Burundi	Matana	2000-2017	-0.180	-0.127	-0.067
Burundi	Matongo	2000	0.149	0.646	1.206
Burundi	Matongo	2017	0.047	0.148	0.342
Burundi	Matongo	2000-2017	-0.076	-0.025	0.022
Burundi	Mbuye	2000	0.286	1.062	1.918
Burundi	Mbuye	2017	0.069	0.202	0.485
Burundi	Mbuye	2000-2017	-0.080	-0.040	0.001
Burundi	Mishiha	2000	0.431	0.785	1.312
Burundi	Mishiha	2017	0.151	0.326	0.634
Burundi	Mishiha	2000-2017	-0.089	-0.052	-0.029
Burundi	Mpanda	2000	0.268	1.054	2.075
Burundi	Mpanda	2017	0.074	0.222	0.494
Burundi	Mpanda	2000-2017	-0.111	-0.052	-0.003
Burundi	Mpinga-Kayove	2000	0.343	0.726	1.257
Burundi	Mpinga-Kayove	2017	0.079	0.206	0.411
Burundi	Mpinga-Kayove	2000-2017	-0.091	-0.057	-0.030
Burundi	Mubimbi	2000	0.186	0.833	1.625
Burundi	Mubimbi	2017	0.041	0.125	0.280
Burundi	Mubimbi	2000-2017	-0.120	-0.069	-0.011
Burundi	Mugamba	2000	0.009	0.036	0.066
Burundi	Mugamba	2017	0.001	0.003	0.007
Burundi	Mugamba	2000-2017	-0.173	-0.121	-0.060
Burundi	Mugina	2000	0.118	0.675	1.646
Burundi	Mugina	2017	0.117	0.324	0.646
Burundi	Mugina	2000-2017	-0.046	0.006	0.067
Burundi	Mugongomanga	2000	0.035	0.165	0.333
Burundi	Mugongomanga	2017	0.006	0.017	0.039
Burundi	Mugongomanga	2000-2017	-0.151	-0.094	-0.026
Burundi	Muhanga	2000	0.354	1.126	1.997
Burundi	Muhanga	2017	0.111	0.307	0.718
Burundi	Muhanga	2000-2017	-0.072	-0.022	0.021
Burundi	Muhuta	2000	0.142	0.607	1.205
Burundi	Muhuta	2017	0.019	0.053	0.108
Burundi	Muhuta	2000-2017	-0.183	-0.128	-0.073
Burundi	Mukike	2000	0.005	0.022	0.042
Burundi	Mukike	2017	0.001	0.002	0.004
Burundi	Mukike	2000-2017	-0.159	-0.107	-0.048
Burundi	Muramvya	2000	0.097	0.448	0.903
Burundi	Muramvya	2017	0.023	0.071	0.170
Burundi	Muramvya	2000-2017	-0.105	-0.053	0.006
Burundi	Muruta	2000	0.093	0.420	0.859
Burundi	Muruta	2017	0.044	0.128	0.285
Burundi	Muruta	2000-2017	-0.053	-0.008	0.039
Burundi	Murwi	2000	0.127	0.787	1.712
Burundi	Murwi	2017	0.088	0.259	0.557
Burundi	Murwi	2000-2017	-0.074	-0.020	0.023
Burundi	Musaga	2000	0.121	0.514	1.097

Table 3: Malaria DALYs rate by unit *(continued)*

Country	Unit	year	mean rate	lower rate	upper rate
Burundi	Musaga	2017	0.018	0.050	0.108
Burundi	Musaga	2000-2017	-0.171	-0.107	-0.048
Burundi	Musigati	2000	0.132	0.627	1.254
Burundi	Musigati	2017	0.056	0.165	0.357
Burundi	Musigati	2000-2017	-0.086	-0.025	0.033
Burundi	Musongati	2000	0.346	0.863	1.494
Burundi	Musongati	2017	0.085	0.225	0.464
Burundi	Musongati	2000-2017	-0.107	-0.057	-0.022
Burundi	Mutaho	2000	0.349	1.011	1.782
Burundi	Mutaho	2017	0.102	0.274	0.615
Burundi	Mutaho	2000-2017	-0.059	-0.023	0.008
Burundi	Mutambu	2000	0.196	0.859	1.817
Burundi	Mutambu	2017	0.031	0.089	0.192
Burundi	Mutambu	2000-2017	-0.154	-0.102	-0.048
Burundi	Mutimbuzi	2000	0.244	0.983	1.946
Burundi	Mutimbuzi	2017	0.052	0.151	0.329
Burundi	Mutimbuzi	2000-2017	-0.136	-0.080	-0.030
Burundi	Mutumba	2000	0.634	1.137	1.954
Burundi	Mutumba	2017	0.170	0.368	0.750
Burundi	Mutumba	2000-2017	-0.066	-0.038	-0.017
Burundi	Muyinga	2000	0.599	1.242	2.192
Burundi	Muyinga	2017	0.195	0.420	0.882
Burundi	Muyinga	2000-2017	-0.081	-0.045	-0.020
Burundi	Mwakiro	2000	0.619	1.117	1.914
Burundi	Mwakiro	2017	0.183	0.406	0.840
Burundi	Mwakiro	2000-2017	-0.059	-0.035	-0.015
Burundi	Mwumba	2000	0.178	0.605	1.112
Burundi	Mwumba	2017	0.077	0.186	0.400
Burundi	Mwumba	2000-2017	-0.061	-0.026	0.010
Burundi	Ndava	2000	0.174	0.589	1.106
Burundi	Ndava	2017	0.041	0.115	0.276
Burundi	Ndava	2000-2017	-0.089	-0.047	-0.001
Burundi	Ngagara	2000	0.083	0.399	0.899
Burundi	Ngagara	2017	0.017	0.048	0.103
Burundi	Ngagara	2000-2017	-0.148	-0.088	-0.029
Burundi	Ngozi	2000	0.242	0.749	1.344
Burundi	Ngozi	2017	0.080	0.209	0.456
Burundi	Ngozi	2000-2017	-0.069	-0.028	0.012
Burundi	Ntega	2000	0.278	0.841	1.468
Burundi	Ntega	2017	0.206	0.418	0.786
Burundi	Ntega	2000-2017	-0.032	-0.006	0.034
Burundi	Nyabihanga	2000	0.385	1.243	2.178
Burundi	Nyabihanga	2017	0.092	0.239	0.560
Burundi	Nyabihanga	2000-2017	-0.095	-0.051	-0.009
Burundi	Nyabikere	2000	0.544	1.010	1.736
Burundi	Nyabikere	2017	0.164	0.349	0.686
Burundi	Nyabikere	2000-2017	-0.057	-0.027	-0.003
Burundi	Nyabiraba	2000	0.201	0.839	1.704
Burundi	Nyabiraba	2017	0.032	0.092	0.206
Burundi	Nyabiraba	2000-2017	-0.147	-0.095	-0.041
Burundi	Nyabitsinda	2000	0.696	1.254	2.053
Burundi	Nyabitsinda	2017	0.208	0.445	0.904
Burundi	Nyabitsinda	2000-2017	-0.086	-0.048	-0.028
Burundi	Nyakabiga	2000	0.089	0.403	0.857
Burundi	Nyakabiga	2017	0.017	0.047	0.104
Burundi	Nyakabiga	2000-2017	-0.150	-0.091	-0.033
Burundi	Nyamurenza	2000	0.220	0.670	1.200
Burundi	Nyamurenza	2017	0.097	0.212	0.431
Burundi	Nyamurenza	2000-2017	-0.065	-0.035	-0.003
Burundi	Nyanrusange	2000	0.311	0.984	1.663
Burundi	Nyanrusange	2017	0.067	0.172	0.400

Table 3: Malaria DALYs rate by unit *(continued)*

Country	Unit	year	mean rate	lower rate	upper rate
Burundi	Nyanrusange	2000-2017	-0.107	-0.062	-0.026
Burundi	Nyanza-Lac	2000	0.326	0.887	1.564
Burundi	Nyanza-Lac	2017	0.051	0.147	0.310
Burundi	Nyanza-Lac	2000-2017	-0.180	-0.120	-0.072
Burundi	Rango	2000	0.284	1.027	1.916
Burundi	Rango	2017	0.085	0.239	0.571
Burundi	Rango	2000-2017	-0.069	-0.025	0.015
Burundi	Roherero	2000	0.088	0.392	0.858
Burundi	Roherero	2017	0.013	0.037	0.082
Burundi	Roherero	2000-2017	-0.173	-0.108	-0.048
Burundi	Rugazi	2000	0.192	0.790	1.485
Burundi	Rugazi	2017	0.051	0.155	0.342
Burundi	Rugazi	2000-2017	-0.105	-0.049	0.005
Burundi	Rugombo	2000	0.119	0.804	1.876
Burundi	Rugombo	2017	0.112	0.331	0.702
Burundi	Rugombo	2000-2017	-0.069	-0.010	0.048
Burundi	Ruhororo	2000	0.460	1.032	1.818
Burundi	Ruhororo	2017	0.126	0.300	0.621
Burundi	Ruhororo	2000-2017	-0.061	-0.035	-0.010
Burundi	Rumonge	2000	0.290	1.016	1.817
Burundi	Rumonge	2017	0.032	0.103	0.227
Burundi	Rumonge	2000-2017	-0.193	-0.138	-0.078
Burundi	Rusaka	2000	0.000	0.000	0.000
Burundi	Rusaka	2017	0.000	0.000	0.000
Burundi	Rusaka	2000-2017	NA	NA	NA
Burundi	Rutana	2000	0.304	0.875	1.520
Burundi	Rutana	2017	0.059	0.149	0.330
Burundi	Rutana	2000-2017	-0.139	-0.091	-0.051
Burundi	Rutegama	2000	0.346	1.208	2.297
Burundi	Rutegama	2017	0.075	0.215	0.524
Burundi	Rutegama	2000-2017	-0.090	-0.050	-0.002
Burundi	Rutovu	2000	0.076	0.237	0.427
Burundi	Rutovu	2017	0.013	0.037	0.076
Burundi	Rutovu	2000-2017	-0.139	-0.090	-0.044
Burundi	Ruyigi	2000	0.514	0.929	1.555
Burundi	Ruyigi	2017	0.112	0.280	0.548
Burundi	Ruyigi	2000-2017	-0.091	-0.056	-0.031
Burundi	Ryansoro	2000	0.000	0.000	0.001
Burundi	Ryansoro	2017	0.000	0.000	0.000
Burundi	Ryansoro	2000-2017	-0.117	-0.069	-0.033
Burundi	Shombo	2000	0.633	1.183	2.051
Burundi	Shombo	2017	0.162	0.349	0.728
Burundi	Shombo	2000-2017	-0.061	-0.036	-0.009
Burundi	Songa	2000	0.046	0.177	0.331
Burundi	Songa	2017	0.006	0.016	0.033
Burundi	Songa	2000-2017	-0.171	-0.120	-0.066
Burundi	Tangara	2000	0.494	1.225	2.240
Burundi	Tangara	2017	0.189	0.405	0.794
Burundi	Tangara	2000-2017	-0.060	-0.037	-0.010
Burundi	Vugizo	2000	0.220	0.779	1.396
Burundi	Vugizo	2017	0.041	0.131	0.285
Burundi	Vugizo	2000-2017	-0.158	-0.103	-0.048
Burundi	Vumbi	2000	0.309	0.944	1.749
Burundi	Vumbi	2017	0.172	0.352	0.662
Burundi	Vumbi	2000-2017	-0.062	-0.031	0.003
Burundi	Vyanda	2000	0.161	0.680	1.240
Burundi	Vyanda	2017	0.030	0.096	0.204
Burundi	Vyanda	2000-2017	-0.162	-0.107	-0.055
Cameroon	Bamboutos	2000	0.024	0.193	0.554
Cameroon	Bamboutos	2017	0.030	0.132	0.331
Cameroon	Bamboutos	2000-2017	-0.189	-0.070	0.030

Table 3: Malaria DALYs rate by unit *(continued)*

Country	Unit	year	mean rate	lower rate	upper rate
Cameroon	Bénoué	2000	0.151	0.446	0.882
Cameroon	Bénoué	2017	0.059	0.236	0.595
Cameroon	Bénoué	2000-2017	-0.161	-0.055	0.045
Cameroon	Boumba et Ngoko	2000	0.141	0.659	1.748
Cameroon	Boumba et Ngoko	2017	0.099	0.349	0.889
Cameroon	Boumba et Ngoko	2000-2017	-0.128	-0.046	-0.002
Cameroon	Boyo	2000	0.041	0.231	0.647
Cameroon	Boyo	2017	0.034	0.143	0.317
Cameroon	Boyo	2000-2017	-0.182	-0.072	0.025
Cameroon	Bui	2000	0.034	0.193	0.535
Cameroon	Bui	2017	0.026	0.129	0.308
Cameroon	Bui	2000-2017	-0.191	-0.066	0.042
Cameroon	Diamaré	2000	0.061	0.264	0.805
Cameroon	Diamaré	2017	0.033	0.179	0.467
Cameroon	Diamaré	2000-2017	-0.235	-0.067	0.051
Cameroon	Dja et Lobo	2000	0.461	0.905	1.608
Cameroon	Dja et Lobo	2017	0.186	0.520	0.952
Cameroon	Dja et Lobo	2000-2017	-0.119	-0.038	0.015
Cameroon	Djerem	2000	0.097	0.313	0.647
Cameroon	Djerem	2017	0.069	0.194	0.373
Cameroon	Djerem	2000-2017	-0.139	-0.041	0.029
Cameroon	Donga Mantung	2000	0.047	0.248	0.610
Cameroon	Donga Mantung	2017	0.046	0.168	0.333
Cameroon	Donga Mantung	2000-2017	-0.143	-0.049	0.032
Cameroon	Fako	2000	0.301	0.542	1.020
Cameroon	Fako	2017	0.138	0.401	0.809
Cameroon	Fako	2000-2017	-0.125	-0.037	0.018
Cameroon	Faro	2000	0.136	0.342	0.629
Cameroon	Faro	2017	0.066	0.206	0.389
Cameroon	Faro	2000-2017	-0.102	-0.036	0.023
Cameroon	Faro et Déo	2000	0.083	0.286	0.616
Cameroon	Faro et Déo	2017	0.038	0.157	0.322
Cameroon	Faro et Déo	2000-2017	-0.161	-0.040	0.044
Cameroon	Haut Nkam	2000	0.061	0.361	0.925
Cameroon	Haut Nkam	2017	0.041	0.252	0.659
Cameroon	Haut Nkam	2000-2017	-0.182	-0.048	0.049
Cameroon	Haut Nyong	2000	0.463	0.883	1.577
Cameroon	Haut Nyong	2017	0.144	0.435	0.788
Cameroon	Haut Nyong	2000-2017	-0.138	-0.046	0.020
Cameroon	Haute Sanaga	2000	0.253	0.637	1.059
Cameroon	Haute Sanaga	2017	0.080	0.366	0.731
Cameroon	Haute Sanaga	2000-2017	-0.154	-0.042	0.026
Cameroon	Hauts Plateaux	2000	0.030	0.223	0.578
Cameroon	Hauts Plateaux	2017	0.024	0.155	0.421
Cameroon	Hauts Plateaux	2000-2017	-0.200	-0.059	0.036
Cameroon	Kadey	2000	0.252	0.569	1.024
Cameroon	Kadey	2017	0.061	0.276	0.507
Cameroon	Kadey	2000-2017	-0.175	-0.053	0.008
Cameroon	Koung Khi	2000	0.029	0.201	0.514
Cameroon	Koung Khi	2017	0.024	0.139	0.388
Cameroon	Koung Khi	2000-2017	-0.206	-0.062	0.034
Cameroon	Koupé Manengouba	2000	0.236	0.655	1.286
Cameroon	Koupé Manengouba	2017	0.156	0.505	1.064
Cameroon	Koupé Manengouba	2000-2017	-0.126	-0.033	0.020
Cameroon	Lebialem	2000	0.052	0.362	0.885
Cameroon	Lebialem	2017	0.051	0.256	0.670
Cameroon	Lebialem	2000-2017	-0.159	-0.056	0.026
Cameroon	Lekié	2000	0.335	0.650	1.210
Cameroon	Lekié	2017	0.217	0.486	0.939
Cameroon	Lekié	2000-2017	-0.084	-0.025	0.020
Cameroon	Logone et Chari	2000	0.147	0.371	0.653

Table 3: Malaria DALYs rate by unit *(continued)*

Country	Unit	year	mean rate	lower rate	upper rate
Cameroon	Logone et Chari	2017	0.040	0.178	0.389
Cameroon	Logone et Chari	2000-2017	-0.165	-0.020	0.077
Cameroon	Lom et Djerem	2000	0.168	0.471	0.866
Cameroon	Lom et Djerem	2017	0.060	0.261	0.506
Cameroon	Lom et Djerem	2000-2017	-0.161	-0.045	0.017
Cameroon	Manyu	2000	0.202	0.681	1.222
Cameroon	Manyu	2017	0.118	0.456	0.919
Cameroon	Manyu	2000-2017	-0.164	-0.053	-0.007
Cameroon	Mayo Banyo	2000	0.060	0.274	0.564
Cameroon	Mayo Banyo	2017	0.080	0.201	0.389
Cameroon	Mayo Banyo	2000-2017	-0.152	-0.039	0.029
Cameroon	Mayo Danay	2000	0.042	0.218	0.702
Cameroon	Mayo Danay	2017	0.041	0.164	0.439
Cameroon	Mayo Danay	2000-2017	-0.205	-0.077	0.052
Cameroon	Mayo Kani	2000	0.066	0.233	0.681
Cameroon	Mayo Kani	2017	0.042	0.185	0.454
Cameroon	Mayo Kani	2000-2017	-0.216	-0.073	0.033
Cameroon	Mayo Louti	2000	0.069	0.407	1.016
Cameroon	Mayo Louti	2017	0.049	0.217	0.607
Cameroon	Mayo Louti	2000-2017	-0.185	-0.066	0.038
Cameroon	Mayo Rey	2000	0.115	0.303	0.555
Cameroon	Mayo Rey	2017	0.050	0.157	0.359
Cameroon	Mayo Rey	2000-2017	-0.166	-0.070	-0.003
Cameroon	Mayo Sava	2000	0.075	0.361	1.007
Cameroon	Mayo Sava	2017	0.041	0.187	0.469
Cameroon	Mayo Sava	2000-2017	-0.234	-0.075	0.040
Cameroon	Mayo Tsanaga	2000	0.088	0.362	0.861
Cameroon	Mayo Tsanaga	2017	0.051	0.182	0.447
Cameroon	Mayo Tsanaga	2000-2017	-0.196	-0.079	0.024
Cameroon	Mbam et Inoubou	2000	0.167	0.509	1.103
Cameroon	Mbam et Inoubou	2017	0.085	0.407	0.873
Cameroon	Mbam et Inoubou	2000-2017	-0.133	-0.034	0.025
Cameroon	Mbam et Kim	2000	0.179	0.462	0.918
Cameroon	Mbam et Kim	2017	0.105	0.335	0.640
Cameroon	Mbam et Kim	2000-2017	-0.142	-0.038	0.015
Cameroon	Mbéré	2000	0.062	0.310	0.593
Cameroon	Mbéré	2017	0.030	0.159	0.316
Cameroon	Mbéré	2000-2017	-0.199	-0.052	0.024
Cameroon	Mefou et Afamba	2000	0.304	0.537	0.924
Cameroon	Mefou et Afamba	2017	0.100	0.270	0.540
Cameroon	Mefou et Afamba	2000-2017	-0.111	-0.031	0.029
Cameroon	Mefou et Akono	2000	0.350	0.620	1.086
Cameroon	Mefou et Akono	2017	0.142	0.355	0.670
Cameroon	Mefou et Akono	2000-2017	-0.097	-0.024	0.024
Cameroon	Meme	2000	0.415	0.705	1.339
Cameroon	Meme	2017	0.221	0.557	1.155
Cameroon	Meme	2000-2017	-0.119	-0.030	0.017
Cameroon	Menchum	2000	0.047	0.245	0.660
Cameroon	Menchum	2017	0.042	0.147	0.309
Cameroon	Menchum	2000-2017	-0.170	-0.070	0.016
Cameroon	Menoua	2000	0.042	0.286	0.750
Cameroon	Menoua	2017	0.035	0.196	0.523
Cameroon	Menoua	2000-2017	-0.169	-0.056	0.031
Cameroon	Mezam	2000	0.024	0.175	0.556
Cameroon	Mezam	2017	0.020	0.109	0.307
Cameroon	Mezam	2000-2017	-0.208	-0.077	0.023
Cameroon	Mfoundi	2000	0.140	0.309	0.647
Cameroon	Mfoundi	2017	0.046	0.146	0.340
Cameroon	Mfoundi	2000-2017	-0.130	-0.027	0.051
Cameroon	Mifi	2000	0.017	0.148	0.388
Cameroon	Mifi	2017	0.022	0.111	0.330

Table 3: Malaria DALYs rate by unit *(continued)*

Country	Unit	year	mean rate	lower rate	upper rate
Cameroon	Mifi	2000-2017	-0.209	-0.067	0.047
Cameroon	Momo	2000	0.044	0.320	0.832
Cameroon	Momo	2017	0.041	0.209	0.582
Cameroon	Momo	2000-2017	-0.192	-0.072	0.019
Cameroon	Moungo	2000	0.321	0.617	1.148
Cameroon	Moungo	2017	0.107	0.405	0.792
Cameroon	Moungo	2000-2017	-0.133	-0.038	0.011
Cameroon	Mvila	2000	0.362	0.799	1.500
Cameroon	Mvila	2017	0.151	0.517	0.935
Cameroon	Mvila	2000-2017	-0.134	-0.032	0.024
Cameroon	Ndé	2000	0.048	0.263	0.652
Cameroon	Ndé	2017	0.026	0.197	0.512
Cameroon	Ndé	2000-2017	-0.201	-0.053	0.036
Cameroon	Ndian	2000	0.372	0.787	1.422
Cameroon	Ndian	2017	0.214	0.533	0.931
Cameroon	Ndian	2000-2017	-0.111	-0.041	0.000
Cameroon	Ngo Ketunjia	2000	0.046	0.241	0.636
Cameroon	Ngo Ketunjia	2017	0.029	0.165	0.397
Cameroon	Ngo Ketunjia	2000-2017	-0.206	-0.071	0.039
Cameroon	Nkam	2000	0.323	0.688	1.274
Cameroon	Nkam	2017	0.096	0.415	0.832
Cameroon	Nkam	2000-2017	-0.135	-0.041	0.019
Cameroon	Noun	2000	0.055	0.241	0.560
Cameroon	Noun	2017	0.049	0.178	0.391
Cameroon	Noun	2000-2017	-0.175	-0.060	0.021
Cameroon	Nyong et Kéllé	2000	0.502	0.816	1.424
Cameroon	Nyong et Kéllé	2017	0.270	0.578	0.985
Cameroon	Nyong et Kéllé	2000-2017	-0.093	-0.026	0.015
Cameroon	Nyong et Mfoumou	2000	0.513	0.939	1.573
Cameroon	Nyong et Mfoumou	2017	0.123	0.510	1.035
Cameroon	Nyong et Mfoumou	2000-2017	-0.133	-0.033	0.033
Cameroon	Nyong et So'o	2000	0.508	0.913	1.628
Cameroon	Nyong et So'o	2017	0.215	0.617	1.095
Cameroon	Nyong et So'o	2000-2017	-0.106	-0.017	0.040
Cameroon	Océan	2000	0.407	0.829	1.426
Cameroon	Océan	2017	0.215	0.537	0.977
Cameroon	Océan	2000-2017	-0.124	-0.037	0.007
Cameroon	Sanaga Maritime	2000	0.353	0.651	1.183
Cameroon	Sanaga Maritime	2017	0.100	0.380	0.744
Cameroon	Sanaga Maritime	2000-2017	-0.147	-0.045	0.016
Cameroon	Vallée du Ntem	2000	0.223	0.658	1.182
Cameroon	Vallée du Ntem	2017	0.141	0.456	0.780
Cameroon	Vallée du Ntem	2000-2017	-0.159	-0.047	0.016
Cameroon	Vina	2000	0.075	0.329	0.668
Cameroon	Vina	2017	0.037	0.167	0.382
Cameroon	Vina	2000-2017	-0.195	-0.056	0.027
Cameroon	Wouri	2000	0.119	0.347	0.698
Cameroon	Wouri	2017	0.039	0.187	0.429
Cameroon	Wouri	2000-2017	-0.173	-0.057	0.026
Central African Republic	Alindao	2000	0.080	0.371	0.681
Central African Republic	Alindao	2017	0.068	0.333	0.671
Central African Republic	Alindao	2000-2017	-0.139	-0.030	0.020
Central African Republic	Baboua	2000	0.078	0.318	0.638
Central African Republic	Baboua	2017	0.047	0.227	0.512
Central African Republic	Baboua	2000-2017	-0.150	-0.031	0.058
Central African Republic	Bakala	2000	0.057	0.254	0.579
Central African Republic	Bakala	2017	0.030	0.212	0.511
Central African Republic	Bakala	2000-2017	-0.188	-0.041	0.059
Central African Republic	Bakouma	2000	0.108	0.423	0.890
Central African Republic	Bakouma	2017	0.089	0.414	0.956
Central African Republic	Bakouma	2000-2017	-0.128	-0.019	0.041

Table 3: Malaria DALYs rate by unit *(continued)*

Country	Unit	year	mean rate	lower rate	upper rate
Central African Republic	Bambari	2000	0.052	0.250	0.570
Central African Republic	Bambari	2017	0.047	0.232	0.576
Central African Republic	Bambari	2000-2017	-0.144	-0.030	0.051
Central African Republic	Bambio	2000	0.145	0.767	1.593
Central African Republic	Bambio	2017	0.081	0.551	1.293
Central African Republic	Bambio	2000-2017	-0.208	-0.048	0.047
Central African Republic	Bamingui	2000	0.046	0.220	0.547
Central African Republic	Bamingui	2017	0.031	0.210	0.522
Central African Republic	Bamingui	2000-2017	-0.133	-0.033	0.053
Central African Republic	Bangassou	2000	0.073	0.485	0.999
Central African Republic	Bangassou	2017	0.090	0.524	1.059
Central African Republic	Bangassou	2000-2017	-0.154	-0.009	0.075
Central African Republic	Bangui	2000	0.111	0.365	0.686
Central African Republic	Bangui	2017	0.038	0.217	0.556
Central African Republic	Bangui	2000-2017	-0.205	-0.079	-0.006
Central African Republic	Baoro	2000	0.050	0.320	0.639
Central African Republic	Baoro	2017	0.026	0.230	0.566
Central African Republic	Baoro	2000-2017	-0.178	-0.042	0.060
Central African Republic	Batangafo	2000	0.041	0.237	0.599
Central African Republic	Batangafo	2017	0.047	0.246	0.601
Central African Republic	Batangafo	2000-2017	-0.204	-0.032	0.052
Central African Republic	Berbérati	2000	0.090	0.395	0.859
Central African Republic	Berbérati	2017	0.041	0.320	0.856
Central African Republic	Berbérati	2000-2017	-0.154	-0.029	0.067
Central African Republic	Bimbo	2000	0.218	0.537	0.947
Central African Republic	Bimbo	2017	0.067	0.363	0.813
Central African Republic	Bimbo	2000-2017	-0.191	-0.063	-0.006
Central African Republic	Birao	2000	0.034	0.273	0.732
Central African Republic	Birao	2017	0.044	0.233	0.685
Central African Republic	Birao	2000-2017	-0.134	-0.026	0.073
Central African Republic	Boali	2000	0.171	0.443	0.763
Central African Republic	Boali	2017	0.044	0.297	0.717
Central African Republic	Boali	2000-2017	-0.178	-0.061	0.012
Central African Republic	Bocaranga	2000	0.025	0.248	0.652
Central African Republic	Bocaranga	2017	0.019	0.195	0.552
Central African Republic	Bocaranga	2000-2017	-0.156	-0.041	0.055
Central African Republic	Boda	2000	0.104	0.401	0.775
Central African Republic	Boda	2017	0.035	0.284	0.685
Central African Republic	Boda	2000-2017	-0.210	-0.053	0.032
Central African Republic	Bossangoa	2000	0.043	0.243	0.622
Central African Republic	Bossangoa	2017	0.030	0.228	0.638
Central African Republic	Bossangoa	2000-2017	-0.166	-0.036	0.057
Central African Republic	Bouar	2000	0.036	0.253	0.529
Central African Republic	Bouar	2017	0.020	0.183	0.499
Central African Republic	Bouar	2000-2017	-0.182	-0.043	0.054
Central African Republic	Bouca	2000	0.071	0.302	0.627
Central African Republic	Bouca	2017	0.041	0.285	0.717
Central African Republic	Bouca	2000-2017	-0.178	-0.032	0.051
Central African Republic	Bozoum	2000	0.050	0.251	0.611
Central African Republic	Bozoum	2017	0.016	0.195	0.559
Central African Republic	Bozoum	2000-2017	-0.210	-0.045	0.067
Central African Republic	Bria	2000	0.057	0.226	0.518
Central African Republic	Bria	2017	0.023	0.189	0.548
Central African Republic	Bria	2000-2017	-0.151	-0.032	0.047
Central African Republic	Carnot	2000	0.105	0.368	0.733
Central African Republic	Carnot	2017	0.039	0.283	0.753
Central African Republic	Carnot	2000-2017	-0.176	-0.042	0.050
Central African Republic	Damara	2000	0.193	0.436	0.757
Central African Republic	Damara	2017	0.049	0.351	0.691
Central African Republic	Damara	2000-2017	-0.173	-0.045	0.011
Central African Republic	Dékoa	2000	0.093	0.325	0.620

Table 3: Malaria DALYs rate by unit *(continued)*

Country	Unit	year	mean rate	lower rate	upper rate
Central African Republic	Dékoa	2017	0.038	0.292	0.679
Central African Republic	Dékoa	2000-2017	-0.195	-0.034	0.057
Central African Republic	Djemah	2000	0.020	0.126	0.381
Central African Republic	Djemah	2017	0.014	0.111	0.364
Central African Republic	Djemah	2000-2017	-0.153	-0.028	0.055
Central African Republic	Gambo-Ouango	2000	0.107	0.609	1.111
Central African Republic	Gambo-Ouango	2017	0.121	0.556	1.059
Central African Republic	Gambo-Ouango	2000-2017	-0.149	-0.022	0.029
Central African Republic	Gamboula	2000	0.113	0.442	0.914
Central African Republic	Gamboula	2017	0.062	0.356	0.928
Central African Republic	Gamboula	2000-2017	-0.152	-0.023	0.086
Central African Republic	Grimari	2000	0.074	0.280	0.592
Central African Republic	Grimari	2017	0.024	0.268	0.629
Central African Republic	Grimari	2000-2017	-0.207	-0.033	0.058
Central African Republic	Ippy	2000	0.043	0.225	0.537
Central African Republic	Ippy	2017	0.025	0.196	0.518
Central African Republic	Ippy	2000-2017	-0.162	-0.034	0.052
Central African Republic	Kabo	2000	0.019	0.211	0.504
Central African Republic	Kabo	2017	0.031	0.240	0.627
Central African Republic	Kabo	2000-2017	-0.221	-0.044	0.084
Central African Republic	Kaga-Bandoro	2000	0.049	0.258	0.591
Central African Republic	Kaga-Bandoro	2017	0.038	0.230	0.559
Central African Republic	Kaga-Bandoro	2000-2017	-0.154	-0.031	0.087
Central African Republic	Kembé	2000	0.125	0.568	1.002
Central African Republic	Kembé	2017	0.125	0.510	0.983
Central African Republic	Kembé	2000-2017	-0.124	-0.025	0.035
Central African Republic	Kouango	2000	0.080	0.378	0.730
Central African Republic	Kouango	2017	0.042	0.315	0.663
Central African Republic	Kouango	2000-2017	-0.154	-0.040	0.017
Central African Republic	M'Baïki	2000	0.355	0.861	1.491
Central African Republic	M'Baïki	2017	0.104	0.524	1.179
Central African Republic	M'Baïki	2000-2017	-0.187	-0.062	0.000
Central African Republic	Markounda	2000	0.036	0.242	0.633
Central African Republic	Markounda	2017	0.034	0.260	0.583
Central African Republic	Markounda	2000-2017	-0.160	-0.034	0.066
Central African Republic	Mbrès	2000	0.034	0.239	0.563
Central African Republic	Mbrès	2017	0.023	0.196	0.480
Central African Republic	Mbrès	2000-2017	-0.200	-0.039	0.064
Central African Republic	Mingala	2000	0.064	0.335	0.649
Central African Republic	Mingala	2017	0.076	0.318	0.707
Central African Republic	Mingala	2000-2017	-0.129	-0.023	0.051
Central African Republic	Mobaye	2000	0.124	0.542	0.921
Central African Republic	Mobaye	2017	0.087	0.455	0.816
Central African Republic	Mobaye	2000-2017	-0.160	-0.032	0.034
Central African Republic	Mongoumba	2000	0.507	1.133	1.945
Central African Republic	Mongoumba	2017	0.192	0.728	1.559
Central African Republic	Mongoumba	2000-2017	-0.154	-0.056	-0.002
Central African Republic	Ndélé	2000	0.046	0.209	0.520
Central African Republic	Ndélé	2017	0.031	0.212	0.521
Central African Republic	Ndélé	2000-2017	-0.180	-0.025	0.061
Central African Republic	Nola	2000	0.108	0.645	1.462
Central African Republic	Nola	2017	0.067	0.509	1.351
Central African Republic	Nola	2000-2017	-0.179	-0.041	0.062
Central African Republic	Obo	2000	0.067	0.369	0.763
Central African Republic	Obo	2017	0.073	0.372	0.832
Central African Republic	Obo	2000-2017	-0.114	-0.009	0.060
Central African Republic	Ouadda	2000	0.040	0.197	0.526
Central African Republic	Ouadda	2017	0.027	0.191	0.582
Central African Republic	Ouadda	2000-2017	-0.096	-0.009	0.064
Central African Republic	Ouanda Djallé	2000	0.028	0.191	0.558
Central African Republic	Ouanda Djallé	2017	0.016	0.197	0.558

Table 3: Malaria DALYs rate by unit *(continued)*

Country	Unit	year	mean rate	lower rate	upper rate
Central African Republic	Ouanda Djallé	2000-2017	-0.139	-0.013	0.096
Central African Republic	Paoua	2000	0.022	0.245	0.610
Central African Republic	Paoua	2017	0.015	0.223	0.533
Central African Republic	Paoua	2000-2017	-0.186	-0.042	0.073
Central African Republic	Rafaï	2000	0.046	0.264	0.742
Central African Republic	Rafaï	2017	0.042	0.309	0.771
Central African Republic	Rafaï	2000-2017	-0.106	-0.005	0.047
Central African Republic	Sibut	2000	0.128	0.382	0.686
Central African Republic	Sibut	2017	0.040	0.357	0.768
Central African Republic	Sibut	2000-2017	-0.184	-0.033	0.034
Central African Republic	Yalinga	2000	0.029	0.159	0.485
Central African Republic	Yalinga	2017	0.024	0.149	0.476
Central African Republic	Yalinga	2000-2017	-0.115	-0.020	0.049
Central African Republic	Yaloké	2000	0.092	0.340	0.663
Central African Republic	Yaloké	2017	0.027	0.241	0.604
Central African Republic	Yaloké	2000-2017	-0.196	-0.052	0.036
Central African Republic	Zémio	2000	0.034	0.272	0.643
Central African Republic	Zémio	2017	0.044	0.277	0.707
Central African Republic	Zémio	2000-2017	-0.100	-0.007	0.067
Chad	Aboudeïa	2000	0.050	0.377	0.871
Chad	Aboudeïa	2017	0.028	0.170	0.579
Chad	Aboudeïa	2000-2017	-0.187	-0.056	0.075
Chad	Assoungha	2000	0.046	0.316	0.843
Chad	Assoungha	2017	0.008	0.100	0.331
Chad	Assoungha	2000-2017	-0.235	-0.096	0.031
Chad	Baguirmi	2000	0.067	0.416	1.048
Chad	Baguirmi	2017	0.036	0.264	0.845
Chad	Baguirmi	2000-2017	-0.173	-0.047	0.072
Chad	Barh Azoum	2000	0.037	0.346	0.805
Chad	Barh Azoum	2017	0.027	0.168	0.519
Chad	Barh Azoum	2000-2017	-0.153	-0.031	0.066
Chad	Barh El Gazel	2000	0.025	0.180	0.426
Chad	Barh El Gazel	2017	0.010	0.097	0.341
Chad	Barh El Gazel	2000-2017	-0.151	-0.002	0.121
Chad	Barh Köh	2000	0.022	0.191	0.554
Chad	Barh Köh	2017	0.026	0.171	0.447
Chad	Barh Köh	2000-2017	-0.182	-0.053	0.040
Chad	Barh Sara	2000	0.025	0.216	0.631
Chad	Barh Sara	2017	0.036	0.207	0.502
Chad	Barh Sara	2000-2017	-0.175	-0.053	0.060
Chad	Barh Signaka	2000	0.054	0.330	0.821
Chad	Barh Signaka	2017	0.020	0.174	0.557
Chad	Barh Signaka	2000-2017	-0.201	-0.056	0.080
Chad	Batha Est	2000	0.028	0.184	0.560
Chad	Batha Est	2017	0.007	0.062	0.190
Chad	Batha Est	2000-2017	-0.203	-0.023	0.108
Chad	Batha Oues	2000	0.044	0.215	0.528
Chad	Batha Oues	2017	0.012	0.089	0.276
Chad	Batha Oues	2000-2017	-0.161	-0.027	0.092
Chad	Béré	2000	0.006	0.058	0.213
Chad	Béré	2017	0.010	0.096	0.347
Chad	Béré	2000-2017	-0.254	-0.096	0.070
Chad	Biltine	2000	0.009	0.137	0.455
Chad	Biltine	2017	0.004	0.055	0.177
Chad	Biltine	2000-2017	-0.186	-0.032	0.100
Chad	Bitkine	2000	0.031	0.302	0.804
Chad	Bitkine	2017	0.026	0.177	0.579
Chad	Bitkine	2000-2017	-0.206	-0.058	0.063
Chad	Borkou	2000	0.000	0.008	0.031
Chad	Borkou	2017	0.000	0.003	0.015
Chad	Borkou	2000-2017	-0.204	-0.048	0.100

Table 3: Malaria DALYs rate by unit *(continued)*

Country	Unit	year	mean rate	lower rate	upper rate
Chad	Dababa	2000	0.029	0.217	0.491
Chad	Dababa	2017	0.015	0.132	0.402
Chad	Dababa	2000-2017	-0.161	-0.035	0.056
Chad	Dagana	2000	0.058	0.317	0.804
Chad	Dagana	2017	0.023	0.176	0.503
Chad	Dagana	2000-2017	-0.099	0.027	0.156
Chad	Dar Tama	2000	0.014	0.191	0.666
Chad	Dar Tama	2017	0.004	0.063	0.253
Chad	Dar Tama	2000-2017	-0.240	-0.063	0.060
Chad	Djourf Al Ahmar	2000	0.041	0.364	0.997
Chad	Djourf Al Ahmar	2000	0.026	0.231	0.634
Chad	Djourf Al Ahmar	2017	0.011	0.131	0.450
Chad	Djourf Al Ahmar	2017	0.007	0.080	0.350
Chad	Djourf Al Ahmar	2000-2017	-0.209	-0.053	0.069
Chad	Djourf Al Ahmar	2000-2017	-0.209	-0.066	0.080
Chad	Dodjé	2000	0.017	0.161	0.453
Chad	Dodjé	2017	0.012	0.116	0.364
Chad	Dodjé	2000-2017	-0.215	-0.088	0.033
Chad	Ennedi Est	2000	0.002	0.040	0.127
Chad	Ennedi Est	2017	0.001	0.021	0.070
Chad	Ennedi Est	2000-2017	-0.180	-0.016	0.132
Chad	Ennedi Ouest	2000	0.001	0.026	0.087
Chad	Ennedi Ouest	2017	0.000	0.014	0.060
Chad	Ennedi Ouest	2000-2017	-0.177	-0.041	0.080
Chad	Fitri	2000	0.016	0.127	0.497
Chad	Fitri	2017	0.005	0.042	0.207
Chad	Fitri	2000-2017	-0.157	-0.018	0.123
Chad	Grande Sido	2000	0.020	0.195	0.510
Chad	Grande Sido	2017	0.015	0.171	0.389
Chad	Grande Sido	2000-2017	-0.279	-0.059	0.054
Chad	Guéra	2000	0.029	0.244	0.651
Chad	Guéra	2017	0.029	0.154	0.427
Chad	Guéra	2000-2017	-0.183	-0.065	0.032
Chad	Haraze Al Biar	2000	0.082	0.370	0.774
Chad	Haraze Al Biar	2017	0.039	0.205	0.544
Chad	Haraze Al Biar	2000-2017	-0.079	0.026	0.129
Chad	Haraze Mangueigne	2000	0.037	0.245	0.650
Chad	Haraze Mangueigne	2017	0.016	0.121	0.356
Chad	Haraze Mangueigne	2000-2017	-0.166	-0.046	0.036
Chad	Kabbia	2000	0.015	0.152	0.505
Chad	Kabbia	2017	0.013	0.154	0.534
Chad	Kabbia	2000-2017	-0.236	-0.085	0.064
Chad	Kanem	2000	0.047	0.268	0.663
Chad	Kanem	2017	0.011	0.128	0.343
Chad	Kanem	2000-2017	-0.100	0.027	0.145
Chad	Kobé	2000	0.010	0.120	0.428
Chad	Kobé	2017	0.004	0.052	0.204
Chad	Kobé	2000-2017	-0.179	-0.015	0.110
Chad	Lac Iro	2000	0.037	0.223	0.626
Chad	Lac Iro	2017	0.019	0.154	0.398
Chad	Lac Iro	2000-2017	-0.176	-0.054	0.045
Chad	Lac Léré	2000	0.062	0.409	1.047
Chad	Lac Léré	2017	0.031	0.241	0.706
Chad	Lac Léré	2000-2017	-0.226	-0.083	0.044
Chad	Lac Wey	2000	0.012	0.117	0.352
Chad	Lac Wey	2017	0.013	0.115	0.377
Chad	Lac Wey	2000-2017	-0.214	-0.089	0.038
Chad	Lanya	2000	0.006	0.114	0.371
Chad	Lanya	2017	0.012	0.124	0.391
Chad	Lanya	2000-2017	-0.244	-0.076	0.053
Chad	Loug Chari	2000	0.022	0.165	0.571

Table 3: Malaria DALYs rate by unit *(continued)*

Country	Unit	year	mean rate	lower rate	upper rate
Chad	Loug Chari	2017	0.018	0.179	0.518
Chad	Loug Chari	2000-2017	-0.234	-0.080	0.031
Chad	Mamdi	2000	0.068	0.438	1.100
Chad	Mamdi	2017	0.024	0.193	0.613
Chad	Mamdi	2000-2017	-0.112	0.010	0.154
Chad	Mandoul Occidental	2000	0.012	0.142	0.439
Chad	Mandoul Occidental	2017	0.014	0.162	0.453
Chad	Mandoul Occidental	2000-2017	-0.232	-0.074	0.065
Chad	Mandoul Oriental	2000	0.015	0.143	0.419
Chad	Mandoul Oriental	2017	0.018	0.158	0.463
Chad	Mandoul Oriental	2000-2017	-0.222	-0.071	0.053
Chad	Mangalmé	2000	0.054	0.349	0.945
Chad	Mangalmé	2017	0.014	0.146	0.546
Chad	Mangalmé	2000-2017	-0.214	-0.054	0.076
Chad	Mayo-Boneye	2000	0.024	0.167	0.508
Chad	Mayo-Boneye	2017	0.017	0.138	0.426
Chad	Mayo-Boneye	2000-2017	-0.228	-0.067	0.077
Chad	Mayo-Dallah	2000	0.040	0.263	0.717
Chad	Mayo-Dallah	2017	0.025	0.177	0.563
Chad	Mayo-Dallah	2000-2017	-0.212	-0.086	0.028
Chad	Mont Illi	2000	0.022	0.234	0.882
Chad	Mont Illi	2017	0.026	0.229	0.707
Chad	Mont Illi	2000-2017	-0.231	-0.079	0.078
Chad	Monts de Lam	2000	0.019	0.217	0.545
Chad	Monts de Lam	2017	0.019	0.139	0.338
Chad	Monts de Lam	2000-2017	-0.200	-0.072	0.030
Chad	N'Djamena	2000	0.062	0.396	0.960
Chad	N'Djamena	2000	0.032	0.206	0.495
Chad	N'Djamena	2017	0.031	0.186	0.531
Chad	N'Djamena	2017	0.013	0.094	0.274
Chad	N'Djamena	2000-2017	-0.118	0.016	0.154
Chad	N'Djamena	2000-2017	-0.148	-0.006	0.117
Chad	Ngourkosso	2000	0.006	0.106	0.464
Chad	Ngourkosso	2017	0.014	0.133	0.435
Chad	Ngourkosso	2000-2017	-0.260	-0.099	0.054
Chad	Nokou	2000	0.012	0.127	0.395
Chad	Nokou	2017	0.003	0.048	0.165
Chad	Nokou	2000-2017	-0.142	-0.006	0.138
Chad	Nya Pendé	2000	0.017	0.181	0.465
Chad	Nya Pendé	2017	0.025	0.159	0.407
Chad	Nya Pendé	2000-2017	-0.184	-0.063	0.040
Chad	Ouara	2000	0.018	0.202	0.583
Chad	Ouara	2017	0.004	0.062	0.287
Chad	Ouara	2000-2017	-0.218	-0.055	0.088
Chad	Pendé	2000	0.009	0.134	0.418
Chad	Pendé	2017	0.015	0.150	0.416
Chad	Pendé	2000-2017	-0.230	-0.071	0.060
Chad	Sila	2000	0.047	0.386	1.016
Chad	Sila	2017	0.016	0.150	0.424
Chad	Sila	2000-2017	-0.190	-0.054	0.035
Chad	Tandjilé Est	2000	0.007	0.052	0.130
Chad	Tandjilé Est	2017	0.010	0.084	0.264
Chad	Tandjilé Est	2000-2017	-0.225	-0.091	0.054
Chad	Tandjilé Ouest	2000	0.015	0.102	0.396
Chad	Tandjilé Ouest	2017	0.013	0.118	0.463
Chad	Tandjilé Ouest	2000-2017	-0.239	-0.095	0.046
Chad	Tibesti	2000	0.000	0.000	0.001
Chad	Tibesti	2017	0.000	0.000	0.000
Chad	Tibesti	2000-2017	-0.236	-0.044	0.085
Chad	Wayi	2000	0.042	0.345	0.972
Chad	Wayi	2017	0.013	0.171	0.598

Table 3: Malaria DALYs rate by unit *(continued)*

Country	Unit	year	mean rate	lower rate	upper rate
Chad	Wayi	2000-2017	-0.124	0.040	0.191
Côte d'Ivoire	Abidjan	2000	0.119	0.270	0.523
Côte d'Ivoire	Abidjan	2017	0.016	0.076	0.237
Côte d'Ivoire	Abidjan	2000-2017	-0.174	-0.075	0.007
Côte d'Ivoire	Agnéby-Tiassa	2000	0.335	0.704	1.208
Côte d'Ivoire	Agnéby-Tiassa	2017	0.051	0.219	0.494
Côte d'Ivoire	Agnéby-Tiassa	2000-2017	-0.229	-0.115	-0.030
Côte d'Ivoire	Bafing	2000	0.191	0.370	0.679
Côte d'Ivoire	Bafing	2017	0.059	0.216	0.500
Côte d'Ivoire	Bafing	2000-2017	-0.146	-0.052	0.041
Côte d'Ivoire	Bagoué	2000	0.190	0.374	0.659
Côte d'Ivoire	Bagoué	2017	0.019	0.111	0.367
Côte d'Ivoire	Bagoué	2000-2017	-0.206	-0.105	0.007
Côte d'Ivoire	Bélier	2000	0.298	0.599	1.031
Côte d'Ivoire	Bélier	2017	0.020	0.121	0.295
Côte d'Ivoire	Bélier	2000-2017	-0.285	-0.158	-0.044
Côte d'Ivoire	Béré	2000	0.262	0.487	0.787
Côte d'Ivoire	Béré	2017	0.050	0.198	0.450
Côte d'Ivoire	Béré	2000-2017	-0.166	-0.054	0.041
Côte d'Ivoire	Bounkani	2000	0.116	0.306	0.595
Côte d'Ivoire	Bounkani	2017	0.047	0.188	0.391
Côte d'Ivoire	Bounkani	2000-2017	-0.167	-0.056	0.007
Côte d'Ivoire	Cavally	2000	0.226	0.539	0.964
Côte d'Ivoire	Cavally	2017	0.052	0.348	0.921
Côte d'Ivoire	Cavally	2000-2017	-0.194	-0.076	0.029
Côte d'Ivoire	Folon	2000	0.104	0.290	0.586
Côte d'Ivoire	Folon	2017	0.016	0.119	0.374
Côte d'Ivoire	Folon	2000-2017	-0.242	-0.088	0.011
Côte d'Ivoire	Gbeke	2000	0.228	0.407	0.683
Côte d'Ivoire	Gbeke	2017	0.023	0.111	0.307
Côte d'Ivoire	Gbeke	2000-2017	-0.242	-0.115	0.004
Côte d'Ivoire	Gbôkle	2000	0.309	0.746	1.426
Côte d'Ivoire	Gbôkle	2017	0.031	0.172	0.576
Côte d'Ivoire	Gbôkle	2000-2017	-0.268	-0.141	-0.017
Côte d'Ivoire	Gôh	2000	0.247	0.653	1.105
Côte d'Ivoire	Gôh	2017	0.030	0.121	0.319
Côte d'Ivoire	Gôh	2000-2017	-0.273	-0.163	-0.052
Côte d'Ivoire	Gontougo	2000	0.238	0.506	0.896
Côte d'Ivoire	Gontougo	2017	0.052	0.207	0.484
Côte d'Ivoire	Gontougo	2000-2017	-0.179	-0.087	-0.008
Côte d'Ivoire	Grands Ponts	2000	0.238	0.555	1.052
Côte d'Ivoire	Grands Ponts	2017	0.034	0.171	0.518
Côte d'Ivoire	Grands Ponts	2000-2017	-0.217	-0.107	-0.003
Côte d'Ivoire	Guémon	2000	0.280	0.675	1.317
Côte d'Ivoire	Guémon	2017	0.047	0.350	0.840
Côte d'Ivoire	Guémon	2000-2017	-0.211	-0.088	0.015
Côte d'Ivoire	Hambol	2000	0.251	0.458	0.740
Côte d'Ivoire	Hambol	2017	0.064	0.221	0.508
Côte d'Ivoire	Hambol	2000-2017	-0.165	-0.065	0.006
Côte d'Ivoire	Haut-Sassandra	2000	0.358	0.780	1.327
Côte d'Ivoire	Haut-Sassandra	2017	0.047	0.250	0.617
Côte d'Ivoire	Haut-Sassandra	2000-2017	-0.238	-0.106	-0.005
Côte d'Ivoire	Iffou	2000	0.355	0.644	1.062
Côte d'Ivoire	Iffou	2017	0.034	0.178	0.473
Côte d'Ivoire	Iffou	2000-2017	-0.254	-0.129	-0.018
Côte d'Ivoire	Indénié-Djuablin	2000	0.440	0.846	1.315
Côte d'Ivoire	Indénié-Djuablin	2017	0.061	0.261	0.600
Côte d'Ivoire	Indénié-Djuablin	2000-2017	-0.230	-0.114	-0.039
Côte d'Ivoire	Kabadougou	2000	0.166	0.385	0.701
Côte d'Ivoire	Kabadougou	2017	0.029	0.160	0.444
Côte d'Ivoire	Kabadougou	2000-2017	-0.207	-0.072	0.015

Table 3: Malaria DALYs rate by unit *(continued)*

Country	Unit	year	mean rate	lower rate	upper rate
Côte d'Ivoire	La Mé	2000	0.392	0.792	1.253
Côte d'Ivoire	La Mé	2017	0.087	0.336	0.727
Côte d'Ivoire	La Mé	2000-2017	-0.183	-0.080	-0.011
Côte d'Ivoire	Lôh-Djiboua	2000	0.246	0.625	1.188
Côte d'Ivoire	Lôh-Djiboua	2017	0.030	0.124	0.341
Côte d'Ivoire	Lôh-Djiboua	2000-2017	-0.282	-0.164	-0.068
Côte d'Ivoire	Marahoué	2000	0.366	0.716	1.183
Côte d'Ivoire	Marahoué	2017	0.037	0.207	0.575
Côte d'Ivoire	Marahoué	2000-2017	-0.220	-0.116	-0.017
Côte d'Ivoire	Moronou	2000	0.468	0.880	1.420
Côte d'Ivoire	Moronou	2017	0.030	0.179	0.490
Côte d'Ivoire	Moronou	2000-2017	-0.290	-0.151	-0.043
Côte d'Ivoire	N'zi	2000	0.295	0.548	0.919
Côte d'Ivoire	N'zi	2017	0.012	0.102	0.322
Côte d'Ivoire	N'zi	2000-2017	-0.325	-0.165	-0.036
Côte d'Ivoire	Nawa	2000	0.395	0.784	1.333
Côte d'Ivoire	Nawa	2017	0.029	0.194	0.580
Côte d'Ivoire	Nawa	2000-2017	-0.250	-0.128	-0.020
Côte d'Ivoire	Poro	2000	0.235	0.421	0.691
Côte d'Ivoire	Poro	2017	0.022	0.110	0.318
Côte d'Ivoire	Poro	2000-2017	-0.221	-0.084	0.021
Côte d'Ivoire	San-Pédro	2000	0.202	0.580	1.131
Côte d'Ivoire	San-Pédro	2017	0.031	0.200	0.522
Côte d'Ivoire	San-Pédro	2000-2017	-0.206	-0.098	-0.008
Côte d'Ivoire	Sud Comoé	2000	0.415	0.760	1.220
Côte d'Ivoire	Sud Comoé	2017	0.082	0.328	0.727
Côte d'Ivoire	Sud Comoé	2000-2017	-0.169	-0.074	-0.003
Côte d'Ivoire	Tchologo	2000	0.181	0.332	0.609
Côte d'Ivoire	Tchologo	2017	0.024	0.118	0.309
Côte d'Ivoire	Tchologo	2000-2017	-0.199	-0.084	0.014
Côte d'Ivoire	Tonkpi	2000	0.232	0.477	0.924
Côte d'Ivoire	Tonkpi	2017	0.062	0.320	0.745
Côte d'Ivoire	Tonkpi	2000-2017	-0.182	-0.069	0.025
Côte d'Ivoire	Worodougou	2000	0.259	0.498	0.862
Côte d'Ivoire	Worodougou	2017	0.057	0.231	0.621
Côte d'Ivoire	Worodougou	2000-2017	-0.169	-0.053	0.037
Côte d'Ivoire	Yamoussoukro	2000	0.212	0.465	0.783
Côte d'Ivoire	Yamoussoukro	2017	0.018	0.089	0.271
Côte d'Ivoire	Yamoussoukro	2000-2017	-0.278	-0.166	-0.047
Democratic Republic of the Congo	Aba	2000	0.189	0.542	0.929
Democratic Republic of the Congo	Aba	2017	0.108	0.263	0.521
Democratic Republic of the Congo	Aba	2000-2017	-0.134	-0.064	-0.014
Democratic Republic of the Congo	Aketi	2000	0.537	1.239	1.988
Democratic Republic of the Congo	Aketi	2017	0.343	0.809	1.356
Democratic Republic of the Congo	Aketi	2000-2017	-0.107	-0.033	-0.003
Democratic Republic of the Congo	Aketi (ville)	2000	0.576	1.281	2.134
Democratic Republic of the Congo	Aketi (ville)	2017	0.196	0.775	1.443
Democratic Republic of the Congo	Aketi (ville)	2000-2017	-0.160	-0.041	0.003
Democratic Republic of the Congo	Ango	2000	0.176	0.506	0.957
Democratic Republic of the Congo	Ango	2017	0.097	0.334	0.717
Democratic Republic of the Congo	Ango	2000-2017	-0.120	-0.027	0.027
Democratic Republic of the Congo	Ariwara	2000	0.726	1.395	2.325
Democratic Republic of the Congo	Ariwara	2017	0.137	0.483	1.019
Democratic Republic of the Congo	Ariwara	2000-2017	-0.176	-0.092	-0.031
Democratic Republic of the Congo	Aru	2000	0.617	1.143	1.889
Democratic Republic of the Congo	Aru	2017	0.168	0.504	0.922
Democratic Republic of the Congo	Aru	2000-2017	-0.146	-0.068	-0.023
Democratic Republic of the Congo	Aru (ville)	2000	0.781	1.437	2.369
Democratic Republic of the Congo	Aru (ville)	2017	0.275	0.728	1.272
Democratic Republic of the Congo	Aru (ville)	2000-2017	-0.114	-0.052	-0.016
Democratic Republic of the Congo	Bafwasende	2000	0.480	1.067	1.772

Table 3: Malaria DALYs rate by unit *(continued)*

Country	Unit	year	mean rate	lower rate	upper rate
Democratic Republic of the Congo	Bafwasende	2017	0.228	0.630	1.204
Democratic Republic of the Congo	Bafwasende	2000-2017	-0.088	-0.017	0.053
Democratic Republic of the Congo	Bagata	2000	0.236	0.630	1.062
Democratic Republic of the Congo	Bagata	2017	0.045	0.226	0.564
Democratic Republic of the Congo	Bagata	2000-2017	-0.183	-0.078	0.009
Democratic Republic of the Congo	Bambesa	2000	0.304	1.070	1.795
Democratic Republic of the Congo	Bambesa	2017	0.152	0.595	1.167
Democratic Republic of the Congo	Bambesa	2000-2017	-0.117	-0.018	0.064
Democratic Republic of the Congo	Banalia	2000	0.577	1.245	1.973
Democratic Republic of the Congo	Banalia	2017	0.446	0.894	1.478
Democratic Republic of the Congo	Banalia	2000-2017	-0.035	-0.009	0.038
Democratic Republic of the Congo	Bandundu	2000	0.116	0.685	1.420
Democratic Republic of the Congo	Bandundu	2017	0.050	0.291	0.862
Democratic Republic of the Congo	Bandundu	2000-2017	-0.176	-0.074	0.032
Democratic Republic of the Congo	Bangu	2000	0.243	0.576	1.021
Democratic Republic of the Congo	Bangu	2017	0.079	0.263	0.509
Democratic Republic of the Congo	Bangu	2000-2017	-0.155	-0.061	0.008
Democratic Republic of the Congo	Baraka	2000	0.123	0.851	1.940
Democratic Republic of the Congo	Baraka	2017	0.037	0.206	0.593
Democratic Republic of the Congo	Baraka	2000-2017	-0.198	-0.092	0.014
Democratic Republic of the Congo	Basankusu	2000	0.509	1.263	2.270
Democratic Republic of the Congo	Basankusu	2017	0.133	0.556	1.207
Democratic Republic of the Congo	Basankusu	2000-2017	-0.189	-0.074	-0.007
Democratic Republic of the Congo	Basankusu (ville)	2000	0.353	1.282	2.504
Democratic Republic of the Congo	Basankusu (ville)	2017	0.105	0.595	1.355
Democratic Republic of the Congo	Basankusu (ville)	2000-2017	-0.227	-0.077	0.018
Democratic Republic of the Congo	Basoko	2000	0.430	1.106	2.039
Democratic Republic of the Congo	Basoko	2017	0.147	0.528	1.122
Democratic Republic of the Congo	Basoko	2000-2017	-0.155	-0.047	0.008
Democratic Republic of the Congo	Basoko (ville)	2000	0.337	0.960	1.766
Democratic Republic of the Congo	Basoko (ville)	2017	0.075	0.402	0.852
Democratic Republic of the Congo	Basoko (ville)	2000-2017	-0.180	-0.059	0.021
Democratic Republic of the Congo	Befale	2000	0.556	1.256	2.035
Democratic Republic of the Congo	Befale	2017	0.129	0.490	1.198
Democratic Republic of the Congo	Befale	2000-2017	-0.160	-0.077	-0.013
Democratic Republic of the Congo	Bena-Dibele	2000	NA	NA	NA
Democratic Republic of the Congo	Bena-Dibele	2017	NA	NA	NA
Democratic Republic of the Congo	Bena-Dibele	2000-2017	NA	NA	NA
Democratic Republic of the Congo	Beni	2000	0.157	0.941	1.953
Democratic Republic of the Congo	Beni	2017	0.068	0.357	0.867
Democratic Republic of the Congo	Beni	2000-2017	-0.177	-0.070	0.029
Democratic Republic of the Congo	Bikoro	2000	0.412	0.985	1.774
Democratic Republic of the Congo	Bikoro	2017	0.139	0.475	1.168
Democratic Republic of the Congo	Bikoro	2000-2017	-0.167	-0.079	-0.020
Democratic Republic of the Congo	Boende	2000	0.716	1.403	2.266
Democratic Republic of the Congo	Boende	2017	0.176	0.617	1.540
Democratic Republic of the Congo	Boende	2000-2017	-0.161	-0.070	-0.010
Democratic Republic of the Congo	Boende (ville)	2000	0.560	1.537	2.606
Democratic Republic of the Congo	Boende (ville)	2017	0.092	0.506	1.628
Democratic Republic of the Congo	Boende (ville)	2000-2017	-0.236	-0.108	-0.004
Democratic Republic of the Congo	Bokungu	2000	0.467	1.200	2.124
Democratic Republic of the Congo	Bokungu	2017	0.221	0.715	1.454
Democratic Republic of the Congo	Bokungu	2000-2017	-0.115	-0.039	-0.008
Democratic Republic of the Congo	Bolobo	2000	0.109	0.555	1.290
Democratic Republic of the Congo	Bolobo	2017	0.057	0.236	0.599
Democratic Republic of the Congo	Bolobo	2000-2017	-0.159	-0.066	0.045
Democratic Republic of the Congo	Bolobo (ville)	2000	0.067	0.499	1.376
Democratic Republic of the Congo	Bolobo (ville)	2017	0.024	0.162	0.449
Democratic Republic of the Congo	Bolobo (ville)	2000-2017	-0.215	-0.087	0.075
Democratic Republic of the Congo	Bolomba	2000	0.371	1.023	1.780
Democratic Republic of the Congo	Bolomba	2017	0.133	0.447	1.078

Table 3: Malaria DALYs rate by unit *(continued)*

Country	Unit	year	mean rate	lower rate	upper rate
Democratic Republic of the Congo	Bolomba	2000-2017	-0.157	-0.077	-0.023
Democratic Republic of the Congo	Boma	2000	0.063	0.358	0.729
Democratic Republic of the Congo	Boma	2017	0.018	0.127	0.322
Democratic Republic of the Congo	Boma	2000-2017	-0.208	-0.092	-0.003
Democratic Republic of the Congo	Bomongo	2000	0.324	0.808	1.461
Democratic Republic of the Congo	Bomongo	2017	0.045	0.219	0.555
Democratic Republic of the Congo	Bomongo	2000-2017	-0.232	-0.124	-0.037
Democratic Republic of the Congo	Bondo	2000	0.333	0.862	1.538
Democratic Republic of the Congo	Bondo	2017	0.272	0.670	1.182
Democratic Republic of the Congo	Bondo	2000-2017	-0.070	-0.024	0.004
Democratic Republic of the Congo	Bondo (ville)	2000	0.262	1.068	1.981
Democratic Republic of the Congo	Bondo (ville)	2017	0.292	0.854	1.496
Democratic Republic of the Congo	Bondo (ville)	2000-2017	-0.101	-0.025	0.025
Democratic Republic of the Congo	Bongandanga	2000	0.450	1.063	1.819
Democratic Republic of the Congo	Bongandanga	2017	0.073	0.289	0.781
Democratic Republic of the Congo	Bongandanga	2000-2017	-0.214	-0.111	-0.026
Democratic Republic of the Congo	Bosobolo	2000	0.209	0.444	0.771
Democratic Republic of the Congo	Bosobolo	2017	0.094	0.281	0.536
Democratic Republic of the Congo	Bosobolo	2000-2017	-0.122	-0.045	-0.012
Democratic Republic of the Congo	Budjala	2000	0.414	1.160	2.076
Democratic Republic of the Congo	Budjala	2017	0.071	0.324	0.866
Democratic Republic of the Congo	Budjala	2000-2017	-0.225	-0.115	-0.028
Democratic Republic of the Congo	Bukama	2000	0.135	0.414	0.843
Democratic Republic of the Congo	Bukama	2017	0.044	0.154	0.331
Democratic Republic of the Congo	Bukama	2000-2017	-0.171	-0.071	0.010
Democratic Republic of the Congo	Bukavu	2000	0.016	0.161	0.670
Democratic Republic of the Congo	Bukavu	2017	0.023	0.082	0.182
Democratic Republic of the Congo	Bukavu	2000-2017	-0.078	0.001	0.088
Democratic Republic of the Congo	Bulungu	2000	0.268	0.766	1.356
Democratic Republic of the Congo	Bulungu	2017	0.046	0.208	0.533
Democratic Republic of the Congo	Bulungu	2000-2017	-0.207	-0.089	0.001
Democratic Republic of the Congo	Bulungu (ville)	2000	0.290	0.984	1.763
Democratic Republic of the Congo	Bulungu (ville)	2017	0.038	0.230	0.700
Democratic Republic of the Congo	Bulungu (ville)	2000-2017	-0.226	-0.097	0.014
Democratic Republic of the Congo	Bumba	2000	0.601	1.298	2.221
Democratic Republic of the Congo	Bumba	2017	0.220	0.599	1.253
Democratic Republic of the Congo	Bumba	2000-2017	-0.170	-0.063	-0.006
Democratic Republic of the Congo	Bumba (ville)	2000	0.195	1.061	2.002
Democratic Republic of the Congo	Bumba (ville)	2017	0.095	0.421	1.074
Democratic Republic of the Congo	Bumba (ville)	2000-2017	-0.211	-0.082	0.021
Democratic Republic of the Congo	Bunia	2000	0.553	1.093	1.904
Democratic Republic of the Congo	Bunia	2017	0.209	0.688	1.400
Democratic Republic of the Congo	Bunia	2000-2017	-0.138	-0.036	0.027
Democratic Republic of the Congo	Businga	2000	0.263	0.785	1.400
Democratic Republic of the Congo	Businga	2017	0.112	0.409	0.807
Democratic Republic of the Congo	Businga	2000-2017	-0.166	-0.065	-0.012
Democratic Republic of the Congo	Buta	2000	0.544	1.375	2.352
Democratic Republic of the Congo	Buta	2017	0.272	0.836	1.346
Democratic Republic of the Congo	Buta	2000-2017	-0.089	-0.020	0.035
Democratic Republic of the Congo	Buta (ville)	2000	0.394	1.343	2.602
Democratic Republic of the Congo	Buta (ville)	2017	0.171	0.703	1.440
Democratic Republic of the Congo	Buta (ville)	2000-2017	-0.141	-0.033	0.062
Democratic Republic of the Congo	Butembo	2000	0.072	0.370	0.903
Democratic Republic of the Congo	Butembo	2017	0.021	0.091	0.217
Democratic Republic of the Congo	Butembo	2000-2017	-0.177	-0.082	0.020
Democratic Republic of the Congo	Dekese	2000	0.181	0.753	1.600
Democratic Republic of the Congo	Dekese	2017	0.023	0.191	0.525
Democratic Republic of the Congo	Dekese	2000-2017	-0.232	-0.114	-0.027
Democratic Republic of the Congo	Demba	2000	0.339	1.040	1.780
Democratic Republic of the Congo	Demba	2017	0.082	0.343	0.855
Democratic Republic of the Congo	Demba	2000-2017	-0.161	-0.079	-0.023

Table 3: Malaria DALYs rate by unit *(continued)*

Country	Unit	year	mean rate	lower rate	upper rate
Democratic Republic of the Congo	Dibaya	2000	0.249	0.687	1.211
Democratic Republic of the Congo	Dibaya	2017	0.091	0.278	0.602
Democratic Republic of the Congo	Dibaya	2000-2017	-0.149	-0.074	-0.011
Democratic Republic of the Congo	Dibaya-Lubwe	2000	0.192	0.794	1.462
Democratic Republic of the Congo	Dibaya-Lubwe	2017	0.040	0.222	0.567
Democratic Republic of the Congo	Dibaya-Lubwe	2000-2017	-0.251	-0.120	-0.016
Democratic Republic of the Congo	Dilolo	2000	0.156	0.390	0.810
Democratic Republic of the Congo	Dilolo	2017	0.205	0.372	0.586
Democratic Republic of the Congo	Dilolo	2000-2017	-0.044	-0.004	0.042
Democratic Republic of the Congo	Dimbelenge	2000	0.182	0.739	1.424
Democratic Republic of the Congo	Dimbelenge	2017	0.056	0.223	0.540
Democratic Republic of the Congo	Dimbelenge	2000-2017	-0.167	-0.086	-0.018
Democratic Republic of the Congo	Dingila	2000	0.112	0.804	1.541
Democratic Republic of the Congo	Dingila	2017	0.096	0.455	0.949
Democratic Republic of the Congo	Dingila	2000-2017	-0.139	-0.026	0.068
Democratic Republic of the Congo	Djolu	2000	0.373	1.064	2.002
Democratic Republic of the Congo	Djolu	2017	0.079	0.409	0.913
Democratic Republic of the Congo	Djolu	2000-2017	-0.175	-0.071	0.001
Democratic Republic of the Congo	Djugu	2000	0.509	1.070	1.819
Democratic Republic of the Congo	Djugu	2017	0.264	0.643	1.271
Democratic Republic of the Congo	Djugu	2000-2017	-0.110	-0.037	0.003
Democratic Republic of the Congo	Dungu	2000	0.283	0.603	0.998
Democratic Republic of the Congo	Dungu	2017	0.158	0.375	0.668
Democratic Republic of the Congo	Dungu	2000-2017	-0.089	-0.042	-0.014
Democratic Republic of the Congo	Dungu (ville)	2000	0.305	0.749	1.253
Democratic Republic of the Congo	Dungu (ville)	2017	0.145	0.474	0.826
Democratic Republic of the Congo	Dungu (ville)	2000-2017	-0.123	-0.045	0.002
Democratic Republic of the Congo	Faradje	2000	0.237	0.502	0.835
Democratic Republic of the Congo	Faradje	2017	0.140	0.299	0.534
Democratic Republic of the Congo	Faradje	2000-2017	-0.107	-0.044	-0.015
Democratic Republic of the Congo	Feshi	2000	0.181	0.494	0.901
Democratic Republic of the Congo	Feshi	2017	0.018	0.100	0.252
Democratic Republic of the Congo	Feshi	2000-2017	-0.232	-0.112	-0.017
Democratic Republic of the Congo	Fizi	2000	0.141	0.564	1.081
Democratic Republic of the Congo	Fizi	2017	0.043	0.170	0.400
Democratic Republic of the Congo	Fizi	2000-2017	-0.174	-0.086	-0.009
Democratic Republic of the Congo	Gbadolite	2000	0.228	0.660	1.164
Democratic Republic of the Congo	Gbadolite	2017	0.128	0.410	0.687
Democratic Republic of the Congo	Gbadolite	2000-2017	-0.139	-0.048	0.010
Democratic Republic of the Congo	Gemena	2000	0.318	0.911	1.648
Democratic Republic of the Congo	Gemena	2017	0.103	0.385	0.829
Democratic Republic of the Congo	Gemena	2000-2017	-0.183	-0.083	-0.013
Democratic Republic of the Congo	Gemena (ville)	2000	0.171	0.830	1.592
Democratic Republic of the Congo	Gemena (ville)	2017	0.056	0.304	0.830
Democratic Republic of the Congo	Gemena (ville)	2000-2017	-0.232	-0.101	-0.006
Democratic Republic of the Congo	Goma	2000	0.036	0.283	0.883
Democratic Republic of the Congo	Goma	2017	0.032	0.127	0.290
Democratic Republic of the Congo	Goma	2000-2017	-0.088	0.012	0.095
Democratic Republic of the Congo	Gungu	2000	0.197	0.621	1.135
Democratic Republic of the Congo	Gungu	2017	0.029	0.131	0.297
Democratic Republic of the Congo	Gungu	2000-2017	-0.211	-0.112	-0.005
Democratic Republic of the Congo	Gungu (ville)	2000	0.128	0.649	1.249
Democratic Republic of the Congo	Gungu (ville)	2017	0.021	0.107	0.288
Democratic Republic of the Congo	Gungu (ville)	2000-2017	-0.233	-0.129	0.001
Democratic Republic of the Congo	Idiofa	2000	0.365	0.903	1.531
Democratic Republic of the Congo	Idiofa	2017	0.049	0.248	0.603
Democratic Republic of the Congo	Idiofa	2000-2017	-0.221	-0.105	-0.014
Democratic Republic of the Congo	Idiofa (ville)	2000	0.083	0.688	1.365
Democratic Republic of the Congo	Idiofa (ville)	2017	0.011	0.114	0.472
Democratic Republic of the Congo	Idiofa (ville)	2000-2017	-0.288	-0.136	0.003
Democratic Republic of the Congo	Idjwi	2000	0.035	0.169	0.467

Table 3: Malaria DALYs rate by unit *(continued)*

Country	Unit	year	mean rate	lower rate	upper rate
Democratic Republic of the Congo	Idjwi	2017	0.027	0.083	0.179
Democratic Republic of the Congo	Idjwi	2000-2017	-0.075	0.003	0.074
Democratic Republic of the Congo	Ikela	2000	0.290	0.963	1.894
Democratic Republic of the Congo	Ikela	2017	0.107	0.430	1.045
Democratic Republic of the Congo	Ikela	2000-2017	-0.133	-0.054	0.001
Democratic Republic of the Congo	Ilebo	2000	0.288	0.814	1.482
Democratic Republic of the Congo	Ilebo	2017	0.074	0.276	0.704
Democratic Republic of the Congo	Ilebo	2000-2017	-0.182	-0.089	-0.019
Democratic Republic of the Congo	Ilebo (ville)	2000	0.205	0.897	1.712
Democratic Republic of the Congo	Ilebo (ville)	2017	0.064	0.319	0.976
Democratic Republic of the Congo	Ilebo (ville)	2000-2017	-0.207	-0.090	0.027
Democratic Republic of the Congo	Ingbokolo	2000	0.439	0.857	1.448
Democratic Republic of the Congo	Ingbokolo	2017	0.097	0.368	0.742
Democratic Republic of the Congo	Ingbokolo	2000-2017	-0.173	-0.074	-0.020
Democratic Republic of the Congo	Ingende	2000	0.293	0.967	1.764
Democratic Republic of the Congo	Ingende	2017	0.118	0.463	1.116
Democratic Republic of the Congo	Ingende	2000-2017	-0.162	-0.072	-0.016
Democratic Republic of the Congo	Inkisi	2000	0.216	0.713	1.392
Democratic Republic of the Congo	Inkisi	2017	0.071	0.349	0.800
Democratic Republic of the Congo	Inkisi	2000-2017	-0.174	-0.061	0.017
Democratic Republic of the Congo	Inongo	2000	0.126	0.571	1.678
Democratic Republic of the Congo	Inongo	2017	0.074	0.305	0.850
Democratic Republic of the Congo	Inongo	2000-2017	-0.145	-0.061	-0.004
Democratic Republic of the Congo	Inongo (ville)	2000	0.104	0.557	1.582
Democratic Republic of the Congo	Inongo (ville)	2017	0.041	0.338	0.918
Democratic Republic of the Congo	Inongo (ville)	2000-2017	-0.199	-0.062	0.018
Democratic Republic of the Congo	Irumu	2000	0.614	1.245	2.126
Democratic Republic of the Congo	Irumu	2017	0.228	0.736	1.435
Democratic Republic of the Congo	Irumu	2000-2017	-0.126	-0.037	0.014
Democratic Republic of the Congo	Isangi	2000	0.532	1.185	2.113
Democratic Republic of the Congo	Isangi	2017	0.184	0.640	1.245
Democratic Republic of the Congo	Isangi	2000-2017	-0.109	-0.035	0.027
Democratic Republic of the Congo	Isangi (ville)	2000	0.366	1.176	2.077
Democratic Republic of the Congo	Isangi (ville)	2017	0.133	0.530	1.073
Democratic Republic of the Congo	Isangi (ville)	2000-2017	-0.161	-0.051	0.035
Democratic Republic of the Congo	Isiro	2000	0.326	1.049	2.013
Democratic Republic of the Congo	Isiro	2017	0.104	0.529	1.305
Democratic Republic of the Congo	Isiro	2000-2017	-0.150	-0.022	0.072
Democratic Republic of the Congo	Kabalo	2000	0.179	0.488	0.939
Democratic Republic of the Congo	Kabalo	2017	0.112	0.318	0.554
Democratic Republic of the Congo	Kabalo	2000-2017	-0.111	-0.041	0.013
Democratic Republic of the Congo	Kabambare	2000	0.300	0.705	1.244
Democratic Republic of the Congo	Kabambare	2017	0.170	0.456	0.746
Democratic Republic of the Congo	Kabambare	2000-2017	-0.122	-0.036	0.018
Democratic Republic of the Congo	Kabare	2000	0.044	0.272	0.705
Democratic Republic of the Congo	Kabare	2017	0.035	0.124	0.257
Democratic Republic of the Congo	Kabare	2000-2017	-0.117	-0.039	0.042
Democratic Republic of the Congo	Kabeya-Kamwanga	2000	0.174	0.749	1.531
Democratic Republic of the Congo	Kabeya-Kamwanga	2017	0.080	0.298	0.617
Democratic Republic of the Congo	Kabeya-Kamwanga	2000-2017	-0.157	-0.077	-0.003
Democratic Republic of the Congo	Kabinda	2000	0.164	0.498	1.022
Democratic Republic of the Congo	Kabinda	2017	0.074	0.262	0.561
Democratic Republic of the Congo	Kabinda	2000-2017	-0.144	-0.063	0.011
Democratic Republic of the Congo	Kabinda (ville)	2000	0.161	0.760	1.574
Democratic Republic of the Congo	Kabinda (ville)	2017	0.079	0.404	0.915
Democratic Republic of the Congo	Kabinda (ville)	2000-2017	-0.189	-0.071	0.012
Democratic Republic of the Congo	Kabongo	2000	0.079	0.426	0.897
Democratic Republic of the Congo	Kabongo	2017	0.056	0.236	0.441
Democratic Republic of the Congo	Kabongo	2000-2017	-0.163	-0.053	0.040
Democratic Republic of the Congo	Kahemba	2000	0.077	0.293	0.654
Democratic Republic of the Congo	Kahemba	2017	0.012	0.059	0.169

Table 3: Malaria DALYs rate by unit *(continued)*

Country	Unit	year	mean rate	lower rate	upper rate
Democratic Republic of the Congo	Kahemba	2000-2017	-0.239	-0.132	-0.031
Democratic Republic of the Congo	Kahemba (ville)	2000	0.025	0.256	0.627
Democratic Republic of the Congo	Kahemba (ville)	2017	0.003	0.037	0.162
Democratic Republic of the Congo	Kahemba (ville)	2000-2017	-0.330	-0.159	-0.012
Democratic Republic of the Congo	Kailo	2000	0.174	0.859	1.877
Democratic Republic of the Congo	Kailo	2017	0.164	0.567	1.278
Democratic Republic of the Congo	Kailo	2000-2017	-0.123	-0.043	0.035
Democratic Republic of the Congo	Kalehe	2000	0.084	0.337	0.841
Democratic Republic of the Congo	Kalehe	2017	0.043	0.175	0.333
Democratic Republic of the Congo	Kalehe	2000-2017	-0.121	-0.030	0.041
Democratic Republic of the Congo	Kalemie	2000	0.212	0.609	1.118
Democratic Republic of the Congo	Kalemie	2017	0.085	0.322	0.666
Democratic Republic of the Congo	Kalemie	2000-2017	-0.157	-0.067	-0.009
Democratic Republic of the Congo	Kalemie (ville)	2000	0.189	0.764	1.549
Democratic Republic of the Congo	Kalemie (ville)	2017	0.066	0.412	0.972
Democratic Republic of the Congo	Kalemie (ville)	2000-2017	-0.204	-0.072	0.011
Democratic Republic of the Congo	Kalima	2000	0.255	1.056	1.978
Democratic Republic of the Congo	Kalima	2017	0.242	0.762	1.494
Democratic Republic of the Congo	Kalima	2000-2017	-0.124	-0.028	0.049
Democratic Republic of the Congo	Kambove	2000	0.173	0.433	0.827
Democratic Republic of the Congo	Kambove	2017	0.085	0.225	0.420
Democratic Republic of the Congo	Kambove	2000-2017	-0.135	-0.042	0.026
Democratic Republic of the Congo	Kamiji	2000	0.181	0.646	1.169
Democratic Republic of the Congo	Kamiji	2017	0.099	0.348	0.663
Democratic Republic of the Congo	Kamiji	2000-2017	-0.168	-0.058	-0.009
Democratic Republic of the Congo	Kamina	2000	0.160	0.409	0.779
Democratic Republic of the Congo	Kamina	2017	0.066	0.187	0.387
Democratic Republic of the Congo	Kamina	2000-2017	-0.132	-0.067	0.020
Democratic Republic of the Congo	Kamina (ville)	2000	0.050	0.427	0.878
Democratic Republic of the Congo	Kamina (ville)	2017	0.025	0.140	0.415
Democratic Republic of the Congo	Kamina (ville)	2000-2017	-0.229	-0.099	0.055
Democratic Republic of the Congo	Kamituga	2000	0.111	0.718	1.772
Democratic Republic of the Congo	Kamituga	2017	0.047	0.292	0.753
Democratic Republic of the Congo	Kamituga	2000-2017	-0.209	-0.072	0.041
Democratic Republic of the Congo	Kamonia	2000	0.249	0.612	1.113
Democratic Republic of the Congo	Kamonia	2017	0.087	0.255	0.536
Democratic Republic of the Congo	Kamonia	2000-2017	-0.174	-0.086	-0.026
Democratic Republic of the Congo	Kananga	2000	0.261	1.038	1.800
Democratic Republic of the Congo	Kananga	2017	0.124	0.395	0.888
Democratic Republic of the Congo	Kananga	2000-2017	-0.150	-0.069	-0.002
Democratic Republic of the Congo	Kaniama	2000	0.289	0.560	0.918
Democratic Republic of the Congo	Kaniama	2017	0.153	0.347	0.601
Democratic Republic of the Congo	Kaniama	2000-2017	-0.094	-0.040	-0.007
Democratic Republic of the Congo	Kaoze	2000	0.216	0.933	2.082
Democratic Republic of the Congo	Kaoze	2017	0.255	0.699	1.363
Democratic Republic of the Congo	Kaoze	2000-2017	-0.110	-0.025	0.066
Democratic Republic of the Congo	Kapanga	2000	0.184	0.435	0.761
Democratic Republic of the Congo	Kapanga	2017	0.149	0.299	0.502
Democratic Republic of the Congo	Kapanga	2000-2017	-0.071	-0.031	-0.011
Democratic Republic of the Congo	Kasaji	2000	0.222	0.609	1.215
Democratic Republic of the Congo	Kasaji	2017	0.221	0.524	0.875
Democratic Republic of the Congo	Kasaji	2000-2017	-0.080	-0.011	0.067
Democratic Republic of the Congo	Kasangulu	2000	0.327	0.664	1.237
Democratic Republic of the Congo	Kasangulu	2017	0.065	0.353	0.649
Democratic Republic of the Congo	Kasangulu	2000-2017	-0.155	-0.055	0.008
Democratic Republic of the Congo	Kasangulu (ville)	2000	0.278	0.687	1.311
Democratic Republic of the Congo	Kasangulu (ville)	2017	0.057	0.349	0.685
Democratic Republic of the Congo	Kasangulu (ville)	2000-2017	-0.177	-0.057	0.021
Democratic Republic of the Congo	Kasenga	2000	0.227	0.493	0.869
Democratic Republic of the Congo	Kasenga	2017	0.169	0.312	0.512
Democratic Republic of the Congo	Kasenga	2000-2017	-0.076	-0.032	0.000

Table 3: Malaria DALYs rate by unit *(continued)*

Country	Unit	year	mean rate	lower rate	upper rate
Democratic Republic of the Congo	Kasongo	2000	0.262	0.792	1.415
Democratic Republic of the Congo	Kasongo	2017	0.194	0.557	1.008
Democratic Republic of the Congo	Kasongo	2000-2017	-0.130	-0.025	0.028
Democratic Republic of the Congo	Kasongo (ville)	2000	0.132	0.676	1.430
Democratic Republic of the Congo	Kasongo (ville)	2017	0.112	0.477	1.025
Democratic Republic of the Congo	Kasongo (ville)	2000-2017	-0.150	-0.014	0.075
Democratic Republic of the Congo	Kasongo-Lunda	2000	0.128	0.420	0.897
Democratic Republic of the Congo	Kasongo-Lunda	2017	0.047	0.182	0.401
Democratic Republic of the Congo	Kasongo-Lunda	2000-2017	-0.134	-0.061	-0.004
Democratic Republic of the Congo	Kasongo-Lunda (ville)	2000	0.060	0.465	1.068
Democratic Republic of the Congo	Kasongo-Lunda (ville)	2017	0.085	0.380	0.782
Democratic Republic of the Congo	Kasongo-Lunda (ville)	2000-2017	-0.111	0.003	0.105
Democratic Republic of the Congo	Katako-Kombe	2000	0.144	0.592	1.291
Democratic Republic of the Congo	Katako-Kombe	2017	0.051	0.266	0.761
Democratic Republic of the Congo	Katako-Kombe	2000-2017	-0.164	-0.054	0.030
Democratic Republic of the Congo	Katanda	2000	0.148	0.729	1.799
Democratic Republic of the Congo	Katanda	2017	0.056	0.290	0.756
Democratic Republic of the Congo	Katanda	2000-2017	-0.217	-0.095	0.014
Democratic Republic of the Congo	Kazumba	2000	0.314	0.762	1.301
Democratic Republic of the Congo	Kazumba	2017	0.087	0.253	0.579
Democratic Republic of the Congo	Kazumba	2000-2017	-0.173	-0.090	-0.022
Democratic Republic of the Congo	Kenge	2000	0.314	0.698	1.215
Democratic Republic of the Congo	Kenge	2017	0.052	0.241	0.607
Democratic Republic of the Congo	Kenge	2000-2017	-0.212	-0.076	0.006
Democratic Republic of the Congo	Kenge (ville)	2000	0.267	0.879	1.612
Democratic Republic of the Congo	Kenge (ville)	2017	0.036	0.246	0.683
Democratic Republic of the Congo	Kenge (ville)	2000-2017	-0.263	-0.102	0.007
Democratic Republic of the Congo	Kibombo	2000	0.240	0.722	1.375
Democratic Republic of the Congo	Kibombo	2017	0.136	0.424	0.842
Democratic Republic of the Congo	Kibombo	2000-2017	-0.146	-0.047	0.019
Democratic Republic of the Congo	Kikwit	2000	0.135	0.814	1.659
Democratic Republic of the Congo	Kikwit	2017	0.025	0.193	0.534
Democratic Republic of the Congo	Kikwit	2000-2017	-0.259	-0.105	0.017
Democratic Republic of the Congo	Kimvula	2000	0.106	0.437	0.889
Democratic Republic of the Congo	Kimvula	2017	0.062	0.234	0.502
Democratic Republic of the Congo	Kimvula	2000-2017	-0.150	-0.052	0.013
Democratic Republic of the Congo	Kindu	2000	0.058	0.641	1.703
Democratic Republic of the Congo	Kindu	2017	0.072	0.413	1.249
Democratic Republic of the Congo	Kindu	2000-2017	-0.173	-0.040	0.076
Democratic Republic of the Congo	Kinshasa	2000	0.095	0.295	0.618
Democratic Republic of the Congo	Kinshasa	2017	0.023	0.167	0.384
Democratic Republic of the Congo	Kinshasa	2000-2017	-0.145	-0.048	0.034
Democratic Republic of the Congo	Kipushi	2000	0.121	0.331	0.648
Democratic Republic of the Congo	Kipushi	2017	0.049	0.157	0.307
Democratic Republic of the Congo	Kipushi	2000-2017	-0.113	-0.021	0.051
Democratic Republic of the Congo	Kipushi (ville)	2000	0.045	0.192	0.487
Democratic Republic of the Congo	Kipushi (ville)	2017	0.021	0.083	0.205
Democratic Republic of the Congo	Kipushi (ville)	2000-2017	-0.118	-0.008	0.083
Democratic Republic of the Congo	Kiri	2000	0.371	1.038	2.047
Democratic Republic of the Congo	Kiri	2017	0.192	0.551	1.193
Democratic Republic of the Congo	Kiri	2000-2017	-0.137	-0.060	-0.012
Democratic Republic of the Congo	Kisangani	2000	0.388	1.118	1.972
Democratic Republic of the Congo	Kisangani	2017	0.297	0.749	1.386
Democratic Republic of the Congo	Kisangani	2000-2017	-0.089	-0.013	0.031
Democratic Republic of the Congo	Kole	2000	0.115	0.641	1.591
Democratic Republic of the Congo	Kole	2017	0.022	0.141	0.402
Democratic Republic of the Congo	Kole	2000-2017	-0.226	-0.112	-0.010
Democratic Republic of the Congo	Kolwezi	2000	0.026	0.209	0.556
Democratic Republic of the Congo	Kolwezi	2017	0.047	0.174	0.387
Democratic Republic of the Congo	Kolwezi	2000-2017	-0.127	0.005	0.121
Democratic Republic of the Congo	Kongolo	2000	0.291	0.653	1.117

Table 3: Malaria DALYs rate by unit *(continued)*

Country	Unit	year	mean rate	lower rate	upper rate
Democratic Republic of the Congo	Kongolo	2017	0.189	0.467	0.773
Democratic Republic of the Congo	Kongolo	2000-2017	-0.112	-0.030	0.016
Democratic Republic of the Congo	Kongolo (ville)	2000	0.213	0.736	1.313
Democratic Republic of the Congo	Kongolo (ville)	2017	0.133	0.503	0.883
Democratic Republic of the Congo	Kongolo (ville)	2000-2017	-0.149	-0.039	0.030
Democratic Republic of the Congo	Kungu	2000	0.486	1.044	1.768
Democratic Republic of the Congo	Kungu	2017	0.066	0.326	0.857
Democratic Republic of the Congo	Kungu	2000-2017	-0.230	-0.105	-0.018
Democratic Republic of the Congo	Kutu	2000	0.314	0.713	1.324
Democratic Republic of the Congo	Kutu	2017	0.130	0.367	0.731
Democratic Republic of the Congo	Kutu	2000-2017	-0.141	-0.062	-0.003
Democratic Republic of the Congo	Kwamouth	2000	0.208	0.635	1.233
Democratic Republic of the Congo	Kwamouth	2017	0.092	0.303	0.763
Democratic Republic of the Congo	Kwamouth	2000-2017	-0.158	-0.061	0.008
Democratic Republic of the Congo	Libenge	2000	0.332	0.781	1.342
Democratic Republic of the Congo	Libenge	2017	0.105	0.375	0.757
Democratic Republic of the Congo	Libenge	2000-2017	-0.156	-0.069	-0.009
Democratic Republic of the Congo	Likasi	2000	0.108	0.387	0.806
Democratic Republic of the Congo	Likasi	2017	0.041	0.176	0.361
Democratic Republic of the Congo	Likasi	2000-2017	-0.185	-0.054	0.043
Democratic Republic of the Congo	Lisala	2000	0.402	1.097	2.000
Democratic Republic of the Congo	Lisala	2017	0.070	0.297	0.754
Democratic Republic of the Congo	Lisala	2000-2017	-0.240	-0.119	-0.029
Democratic Republic of the Congo	Lisala (ville)	2000	0.174	0.975	2.151
Democratic Republic of the Congo	Lisala (ville)	2017	0.028	0.207	0.740
Democratic Republic of the Congo	Lisala (ville)	2000-2017	-0.310	-0.143	0.007
Democratic Republic of the Congo	Lodja	2000	0.064	0.623	2.646
Democratic Republic of the Congo	Lodja	2017	0.023	0.287	1.012
Democratic Republic of the Congo	Lodja	2000-2017	-0.183	-0.061	0.026
Democratic Republic of the Congo	Lodja (ville)	2000	NA	NA	NA
Democratic Republic of the Congo	Lodja (ville)	2017	NA	NA	NA
Democratic Republic of the Congo	Lodja (ville)	2000-2017	NA	NA	NA
Democratic Republic of the Congo	Lomela	2000	0.121	0.724	2.175
Democratic Republic of the Congo	Lomela	2017	0.035	0.298	1.015
Democratic Republic of the Congo	Lomela	2000-2017	-0.177	-0.064	0.015
Democratic Republic of the Congo	Lubao	2000	0.173	0.503	0.978
Democratic Republic of the Congo	Lubao	2017	0.112	0.301	0.580
Democratic Republic of the Congo	Lubao	2000-2017	-0.123	-0.043	0.004
Democratic Republic of the Congo	Lubao (ville)	2000	0.133	0.579	1.223
Democratic Republic of the Congo	Lubao (ville)	2017	0.106	0.347	0.689
Democratic Republic of the Congo	Lubao (ville)	2000-2017	-0.146	-0.045	0.043
Democratic Republic of the Congo	Lubefu	2000	0.110	0.423	1.012
Democratic Republic of the Congo	Lubefu	2017	0.030	0.163	0.408
Democratic Republic of the Congo	Lubefu	2000-2017	-0.210	-0.077	0.010
Democratic Republic of the Congo	Lubero	2000	0.122	0.393	0.819
Democratic Republic of the Congo	Lubero	2017	0.025	0.114	0.251
Democratic Republic of the Congo	Lubero	2000-2017	-0.180	-0.078	0.001
Democratic Republic of the Congo	Lubudi	2000	0.179	0.433	0.794
Democratic Republic of the Congo	Lubudi	2017	0.103	0.252	0.424
Democratic Republic of the Congo	Lubudi	2000-2017	-0.119	-0.045	-0.006
Democratic Republic of the Congo	Lubumbashi	2000	0.071	0.222	0.499
Democratic Republic of the Congo	Lubumbashi	2017	0.020	0.080	0.168
Democratic Republic of the Congo	Lubumbashi	2000-2017	-0.142	-0.022	0.070
Democratic Republic of the Congo	Lubutu	2000	0.427	1.173	1.953
Democratic Republic of the Congo	Lubutu	2017	0.208	0.699	1.397
Democratic Republic of the Congo	Lubutu	2000-2017	-0.130	-0.040	0.017
Democratic Republic of the Congo	Lubutu (ville)	2000	0.326	1.184	2.152
Democratic Republic of the Congo	Lubutu (ville)	2017	0.128	0.609	1.446
Democratic Republic of the Congo	Lubutu (ville)	2000-2017	-0.177	-0.058	0.032
Democratic Republic of the Congo	Luebo	2000	0.530	1.094	1.831
Democratic Republic of the Congo	Luebo	2017	0.092	0.381	0.941

Table 3: Malaria DALYs rate by unit *(continued)*

Country	Unit	year	mean rate	lower rate	upper rate
Democratic Republic of the Congo	Luebo	2000-2017	-0.187	-0.088	-0.008
Democratic Republic of the Congo	Luebo (ville)	2000	0.335	1.123	2.018
Democratic Republic of the Congo	Luebo (ville)	2017	0.041	0.307	0.929
Democratic Republic of the Congo	Luebo (ville)	2000-2017	-0.239	-0.115	-0.004
Democratic Republic of the Congo	Luilu	2000	0.355	0.708	1.196
Democratic Republic of the Congo	Luilu	2017	0.160	0.413	0.756
Democratic Republic of the Congo	Luilu	2000-2017	-0.126	-0.044	-0.010
Democratic Republic of the Congo	Luiza	2000	0.172	0.441	0.772
Democratic Republic of the Congo	Luiza	2017	0.100	0.237	0.443
Democratic Republic of the Congo	Luiza	2000-2017	-0.103	-0.052	-0.011
Democratic Republic of the Congo	Lukalaba	2000	0.239	0.935	1.961
Democratic Republic of the Congo	Lukalaba	2017	0.118	0.501	1.069
Democratic Republic of the Congo	Lukalaba	2000-2017	-0.196	-0.076	0.008
Democratic Republic of the Congo	Lukolela	2000	0.049	0.474	1.752
Democratic Republic of the Congo	Lukolela	2017	0.024	0.156	0.518
Democratic Republic of the Congo	Lukolela	2000-2017	-0.185	-0.096	-0.012
Democratic Republic of the Congo	Lukula	2000	0.286	0.920	1.761
Democratic Republic of the Congo	Lukula	2017	0.152	0.543	1.030
Democratic Republic of the Congo	Lukula	2000-2017	-0.135	-0.050	0.003
Democratic Republic of the Congo	Lukula (ville)	2000	0.178	0.632	1.236
Democratic Republic of the Congo	Lukula (ville)	2017	0.075	0.354	0.644
Democratic Republic of the Congo	Lukula (ville)	2000-2017	-0.153	-0.057	0.008
Democratic Republic of the Congo	Luozi	2000	0.162	0.523	1.051
Democratic Republic of the Congo	Luozi	2017	0.039	0.210	0.448
Democratic Republic of the Congo	Luozi	2000-2017	-0.204	-0.083	-0.007
Democratic Republic of the Congo	Lupatapata	2000	0.156	0.681	1.547
Democratic Republic of the Congo	Lupatapata	2017	0.067	0.300	0.701
Democratic Republic of the Congo	Lupatapata	2000-2017	-0.196	-0.083	0.000
Democratic Republic of the Congo	Lusambo	2000	0.154	0.696	1.528
Democratic Republic of the Congo	Lusambo	2017	0.040	0.203	0.668
Democratic Republic of the Congo	Lusambo	2000-2017	-0.211	-0.093	-0.005
Democratic Republic of the Congo	Lusambo (ville)	2000	0.074	0.629	1.440
Democratic Republic of the Congo	Lusambo (ville)	2017	0.019	0.146	0.621
Democratic Republic of the Congo	Lusambo (ville)	2000-2017	-0.289	-0.108	0.033
Democratic Republic of the Congo	Madimba	2000	0.313	0.698	1.274
Democratic Republic of the Congo	Madimba	2017	0.106	0.391	0.705
Democratic Republic of the Congo	Madimba	2000-2017	-0.148	-0.045	0.003
Democratic Republic of the Congo	Mahagi	2000	0.519	0.991	1.667
Democratic Republic of the Congo	Mahagi	2017	0.358	0.674	1.113
Democratic Republic of the Congo	Mahagi	2000-2017	-0.048	-0.014	0.017
Democratic Republic of the Congo	Mahagi (ville)	2000	0.582	1.090	1.845
Democratic Republic of the Congo	Mahagi (ville)	2017	0.403	0.743	1.261
Democratic Republic of the Congo	Mahagi (ville)	2000-2017	-0.041	-0.015	0.023
Democratic Republic of the Congo	Makanza	2000	0.411	1.033	1.861
Democratic Republic of the Congo	Makanza	2017	0.052	0.311	0.806
Democratic Republic of the Congo	Makanza	2000-2017	-0.233	-0.109	-0.009
Democratic Republic of the Congo	Malemba-Nkulu	2000	0.219	0.553	1.085
Democratic Republic of the Congo	Malemba-Nkulu	2017	0.097	0.279	0.582
Democratic Republic of the Congo	Malemba-Nkulu	2000-2017	-0.175	-0.066	0.003
Democratic Republic of the Congo	Mambasa	2000	0.559	1.166	2.041
Democratic Republic of the Congo	Mambasa	2017	0.143	0.573	1.028
Democratic Republic of the Congo	Mambasa	2000-2017	-0.133	-0.044	0.011
Democratic Republic of the Congo	Mangai	2000	0.223	0.979	1.860
Democratic Republic of the Congo	Mangai	2017	0.032	0.261	0.828
Democratic Republic of the Congo	Mangai	2000-2017	-0.268	-0.131	0.021
Democratic Republic of the Congo	Manono	2000	0.199	0.469	0.855
Democratic Republic of the Congo	Manono	2017	0.105	0.272	0.498
Democratic Republic of the Congo	Manono	2000-2017	-0.121	-0.050	-0.014
Democratic Republic of the Congo	Manono (ville)	2000	0.158	0.647	1.197
Democratic Republic of the Congo	Manono (ville)	2017	0.063	0.322	0.690
Democratic Republic of the Congo	Manono (ville)	2000-2017	-0.202	-0.073	-0.005

Table 3: Malaria DALYs rate by unit *(continued)*

Country	Unit	year	mean rate	lower rate	upper rate
Democratic Republic of the Congo	Masi-Manimba	2000	0.326	0.730	1.204
Democratic Republic of the Congo	Masi-Manimba	2017	0.053	0.232	0.616
Democratic Republic of the Congo	Masi-Manimba	2000-2017	-0.199	-0.079	0.008
Democratic Republic of the Congo	Masi-Manimba (ville)	2000	0.302	0.904	1.556
Democratic Republic of the Congo	Masi-Manimba (ville)	2017	0.033	0.239	0.694
Democratic Republic of the Congo	Masi-Manimba (ville)	2000-2017	-0.241	-0.105	0.005
Democratic Republic of the Congo	Masisi	2000	0.052	0.289	0.798
Democratic Republic of the Congo	Masisi	2017	0.031	0.125	0.299
Democratic Republic of the Congo	Masisi	2000-2017	-0.141	-0.033	0.057
Democratic Republic of the Congo	Matadi	2000	0.022	0.130	0.321
Democratic Republic of the Congo	Matadi	2017	0.016	0.081	0.213
Democratic Republic of the Congo	Matadi	2000-2017	-0.219	-0.087	0.006
Democratic Republic of the Congo	Mbandaka	2000	0.195	0.951	1.917
Democratic Republic of the Congo	Mbandaka	2017	0.047	0.310	1.010
Democratic Republic of the Congo	Mbandaka	2000-2017	-0.271	-0.125	-0.022
Democratic Republic of the Congo	Mbanza-Ngungu	2000	0.368	0.806	1.447
Democratic Republic of the Congo	Mbanza-Ngungu	2017	0.120	0.394	0.710
Democratic Republic of the Congo	Mbanza-Ngungu	2000-2017	-0.139	-0.052	0.009
Democratic Republic of the Congo	Mbanza-Ngungu (ville)	2000	0.226	0.667	1.262
Democratic Republic of the Congo	Mbanza-Ngungu (ville)	2017	0.063	0.316	0.670
Democratic Republic of the Congo	Mbanza-Ngungu (ville)	2000-2017	-0.165	-0.062	0.026
Democratic Republic of the Congo	Mbuji-Mayi	2000	0.091	0.514	1.391
Democratic Republic of the Congo	Mbuji-Mayi	2017	0.034	0.184	0.489
Democratic Republic of the Congo	Mbuji-Mayi	2000-2017	-0.233	-0.101	0.005
Democratic Republic of the Congo	Miabi	2000	0.210	0.849	1.642
Democratic Republic of the Congo	Miabi	2017	0.128	0.485	0.905
Democratic Republic of the Congo	Miabi	2000-2017	-0.167	-0.064	-0.002
Democratic Republic of the Congo	Miabi (ville)	2000	0.172	0.885	1.905
Democratic Republic of the Congo	Miabi (ville)	2017	0.105	0.455	0.939
Democratic Republic of the Congo	Miabi (ville)	2000-2017	-0.181	-0.074	0.002
Democratic Republic of the Congo	Mitwaba	2000	0.177	0.448	0.811
Democratic Republic of the Congo	Mitwaba	2017	0.102	0.268	0.469
Democratic Republic of the Congo	Mitwaba	2000-2017	-0.137	-0.045	0.009
Democratic Republic of the Congo	Moanda	2000	0.132	0.552	1.069
Democratic Republic of the Congo	Moanda	2017	0.041	0.196	0.411
Democratic Republic of the Congo	Moanda	2000-2017	-0.179	-0.082	-0.016
Democratic Republic of the Congo	Moanda (ville)	2000	0.042	0.297	0.668
Democratic Republic of the Congo	Moanda (ville)	2017	0.010	0.045	0.105
Democratic Republic of the Congo	Moanda (ville)	2000-2017	-0.213	-0.117	-0.024
Democratic Republic of the Congo	Moba	2000	0.136	0.462	0.961
Democratic Republic of the Congo	Moba	2017	0.177	0.361	0.620
Democratic Republic of the Congo	Moba	2000-2017	-0.076	-0.018	0.053
Democratic Republic of the Congo	Mobayi-Mbongo	2000	0.299	0.782	1.342
Democratic Republic of the Congo	Mobayi-Mbongo	2017	0.202	0.506	0.854
Democratic Republic of the Congo	Mobayi-Mbongo	2000-2017	-0.108	-0.041	0.001
Democratic Republic of the Congo	Mobayi-Mbongo (ville)	2000	0.148	0.623	1.069
Democratic Republic of the Congo	Mobayi-Mbongo (ville)	2017	0.105	0.405	0.719
Democratic Republic of the Congo	Mobayi-Mbongo (ville)	2000-2017	-0.148	-0.048	0.025
Democratic Republic of the Congo	Mongwalu	2000	0.452	0.913	1.582
Democratic Republic of the Congo	Mongwalu	2017	0.188	0.547	1.057
Democratic Republic of the Congo	Mongwalu	2000-2017	-0.130	-0.040	0.008
Democratic Republic of the Congo	Monkoto	2000	0.428	1.128	2.113
Democratic Republic of the Congo	Monkoto	2017	0.159	0.509	1.133
Democratic Republic of the Congo	Monkoto	2000-2017	-0.156	-0.066	-0.013
Democratic Republic of the Congo	Mushie	2000	0.156	0.586	1.290
Democratic Republic of the Congo	Mushie	2017	0.110	0.323	0.699
Democratic Republic of the Congo	Mushie	2000-2017	-0.144	-0.057	0.026
Democratic Republic of the Congo	Mutshatsha	2000	0.095	0.314	0.689
Democratic Republic of the Congo	Mutshatsha	2017	0.082	0.248	0.486
Democratic Republic of the Congo	Mutshatsha	2000-2017	-0.099	-0.010	0.069
Democratic Republic of the Congo	Mweka	2000	0.355	1.001	1.895

Table 3: Malaria DALYs rate by unit *(continued)*

Country	Unit	year	mean rate	lower rate	upper rate
Democratic Republic of the Congo	Mweka	2017	0.057	0.246	0.686
Democratic Republic of the Congo	Mweka	2000-2017	-0.228	-0.116	-0.035
Democratic Republic of the Congo	Mwene-Ditu	2000	0.224	0.851	1.635
Democratic Republic of the Congo	Mwene-Ditu	2017	0.082	0.442	0.948
Democratic Republic of the Congo	Mwene-Ditu	2000-2017	-0.186	-0.064	0.007
Democratic Republic of the Congo	Mwenga	2000	0.147	0.760	1.490
Democratic Republic of the Congo	Mwenga	2017	0.094	0.370	0.740
Democratic Republic of the Congo	Mwenga	2000-2017	-0.174	-0.059	0.025
Democratic Republic of the Congo	Namoya	2000	0.227	0.938	1.652
Democratic Republic of the Congo	Namoya	2017	0.147	0.588	1.151
Democratic Republic of the Congo	Namoya	2000-2017	-0.174	-0.046	0.035
Democratic Republic of the Congo	Ngandajika	2000	0.385	0.898	1.689
Democratic Republic of the Congo	Ngandajika	2017	0.190	0.523	1.079
Democratic Republic of the Congo	Ngandajika	2000-2017	-0.140	-0.057	-0.005
Democratic Republic of the Congo	Ngandajika (ville)	2000	0.425	0.939	1.707
Democratic Republic of the Congo	Ngandajika (ville)	2017	0.198	0.548	1.135
Democratic Republic of the Congo	Ngandajika (ville)	2000-2017	-0.148	-0.057	-0.007
Democratic Republic of the Congo	Niangara	2000	0.345	0.773	1.282
Democratic Republic of the Congo	Niangara	2017	0.177	0.516	0.829
Democratic Republic of the Congo	Niangara	2000-2017	-0.103	-0.036	0.001
Democratic Republic of the Congo	Nioki	2000	0.213	0.800	1.733
Democratic Republic of the Congo	Nioki	2017	0.150	0.471	0.977
Democratic Republic of the Congo	Nioki	2000-2017	-0.159	-0.056	0.022
Democratic Republic of the Congo	Nyiragongo	2000	0.026	0.200	0.591
Democratic Republic of the Congo	Nyiragongo	2017	0.024	0.093	0.207
Democratic Republic of the Congo	Nyiragongo	2000-2017	-0.080	0.016	0.099
Democratic Republic of the Congo	Nyunzu	2000	0.263	0.511	0.914
Democratic Republic of the Congo	Nyunzu	2017	0.085	0.299	0.547
Democratic Republic of the Congo	Nyunzu	2000-2017	-0.149	-0.050	-0.001
Democratic Republic of the Congo	Oicha	2000	0.294	0.974	1.857
Democratic Republic of the Congo	Oicha	2017	0.088	0.384	0.801
Democratic Republic of the Congo	Oicha	2000-2017	-0.158	-0.067	0.009
Democratic Republic of the Congo	Oïcha (ville)	2000	0.265	1.061	2.133
Democratic Republic of the Congo	Oïcha (ville)	2017	0.083	0.437	0.959
Democratic Republic of the Congo	Oïcha (ville)	2000-2017	-0.162	-0.063	0.032
Democratic Republic of the Congo	Opala	2000	0.392	0.975	1.881
Democratic Republic of the Congo	Opala	2017	0.181	0.582	1.198
Democratic Republic of the Congo	Opala	2000-2017	-0.097	-0.026	0.022
Democratic Republic of the Congo	Oshwe	2000	0.243	0.803	1.814
Democratic Republic of the Congo	Oshwe	2017	0.070	0.329	0.880
Democratic Republic of the Congo	Oshwe	2000-2017	-0.184	-0.080	-0.004
Democratic Republic of the Congo	Pangi	2000	0.279	0.924	1.818
Democratic Republic of the Congo	Pangi	2017	0.151	0.623	1.279
Democratic Republic of the Congo	Pangi	2000-2017	-0.157	-0.030	0.041
Democratic Republic of the Congo	Poko	2000	0.439	0.950	1.582
Democratic Republic of the Congo	Poko	2017	0.218	0.573	1.129
Democratic Republic of the Congo	Poko	2000-2017	-0.097	-0.022	0.040
Democratic Republic of the Congo	Popokabaka	2000	0.146	0.493	0.977
Democratic Republic of the Congo	Popokabaka	2017	0.043	0.200	0.529
Democratic Republic of the Congo	Popokabaka	2000-2017	-0.175	-0.065	0.013
Democratic Republic of the Congo	Punia	2000	0.363	1.123	1.933
Democratic Republic of the Congo	Punia	2017	0.222	0.722	1.413
Democratic Republic of the Congo	Punia	2000-2017	-0.146	-0.040	0.010
Democratic Republic of the Congo	Punia (ville)	2000	0.226	1.194	2.215
Democratic Republic of the Congo	Punia (ville)	2017	0.185	0.716	1.607
Democratic Republic of the Congo	Punia (ville)	2000-2017	-0.175	-0.050	0.030
Democratic Republic of the Congo	Pweto	2000	0.261	0.560	1.039
Democratic Republic of the Congo	Pweto	2017	0.233	0.421	0.679
Democratic Republic of the Congo	Pweto	2000-2017	-0.058	-0.023	0.010
Democratic Republic of the Congo	Rungu	2000	0.588	1.219	2.019
Democratic Republic of the Congo	Rungu	2017	0.256	0.710	1.302

Table 3: Malaria DALYs rate by unit *(continued)*

Country	Unit	year	mean rate	lower rate	upper rate
Democratic Republic of the Congo	Rungu	2000-2017	-0.092	-0.020	0.038
Democratic Republic of the Congo	Rutshuru	2000	0.223	0.630	1.411
Democratic Republic of the Congo	Rutshuru	2017	0.059	0.219	0.449
Democratic Republic of the Congo	Rutshuru	2000-2017	-0.125	-0.043	0.025
Democratic Republic of the Congo	Rutshuru (ville)	2000	0.181	0.845	1.871
Democratic Republic of the Congo	Rutshuru (ville)	2017	0.061	0.269	0.610
Democratic Republic of the Congo	Rutshuru (ville)	2000-2017	-0.139	-0.047	0.029
Democratic Republic of the Congo	Sakania	2000	0.066	0.244	0.541
Democratic Republic of the Congo	Sakania	2017	0.090	0.219	0.416
Democratic Republic of the Congo	Sakania	2000-2017	-0.079	-0.011	0.033
Democratic Republic of the Congo	Sandoa	2000	0.251	0.483	0.826
Democratic Republic of the Congo	Sandoa	2017	0.197	0.342	0.532
Democratic Republic of the Congo	Sandoa	2000-2017	-0.072	-0.031	-0.010
Democratic Republic of the Congo	Seke-Banza	2000	0.272	0.725	1.366
Democratic Republic of the Congo	Seke-Banza	2017	0.108	0.432	0.765
Democratic Republic of the Congo	Seke-Banza	2000-2017	-0.139	-0.058	-0.013
Democratic Republic of the Congo	Shabunda	2000	0.331	1.012	1.766
Democratic Republic of the Congo	Shabunda	2017	0.210	0.624	1.121
Democratic Republic of the Congo	Shabunda	2000-2017	-0.132	-0.041	0.016
Democratic Republic of the Congo	Shabunda (ville)	2000	0.348	1.270	2.478
Democratic Republic of the Congo	Shabunda (ville)	2017	0.126	0.721	1.490
Democratic Republic of the Congo	Shabunda (ville)	2000-2017	-0.183	-0.052	0.034
Democratic Republic of the Congo	Songololo	2000	0.278	0.575	1.017
Democratic Republic of the Congo	Songololo	2017	0.102	0.303	0.582
Democratic Republic of the Congo	Songololo	2000-2017	-0.145	-0.060	-0.001
Democratic Republic of the Congo	Tshela	2000	0.320	1.047	1.992
Democratic Republic of the Congo	Tshela	2017	0.201	0.630	1.048
Democratic Republic of the Congo	Tshela	2000-2017	-0.135	-0.044	0.006
Democratic Republic of the Congo	Tshela (ville)	2000	0.310	1.107	2.245
Democratic Republic of the Congo	Tshela (ville)	2017	0.162	0.610	1.099
Democratic Republic of the Congo	Tshela (ville)	2000-2017	-0.148	-0.053	0.008
Democratic Republic of the Congo	Tshikapa	2000	0.122	0.701	1.502
Democratic Republic of the Congo	Tshikapa	2017	0.080	0.329	0.741
Democratic Republic of the Congo	Tshikapa	2000-2017	-0.209	-0.095	-0.011
Democratic Republic of the Congo	Tshilenge	2000	0.250	0.892	1.898
Democratic Republic of the Congo	Tshilenge	2017	0.109	0.460	1.034
Democratic Republic of the Congo	Tshilenge	2000-2017	-0.186	-0.074	-0.001
Democratic Republic of the Congo	Tshilenge (ville)	2000	0.211	0.895	2.031
Democratic Republic of the Congo	Tshilenge (ville)	2017	0.070	0.379	0.979
Democratic Republic of the Congo	Tshilenge (ville)	2000-2017	-0.209	-0.093	0.008
Democratic Republic of the Congo	Tshimbulu	2000	0.176	0.935	1.862
Democratic Republic of the Congo	Tshimbulu	2017	0.104	0.387	0.975
Democratic Republic of the Congo	Tshimbulu	2000-2017	-0.199	-0.086	0.002
Democratic Republic of the Congo	Ubundu	2000	0.483	1.112	1.854
Democratic Republic of the Congo	Ubundu	2017	0.365	0.786	1.402
Democratic Republic of the Congo	Ubundu	2000-2017	-0.074	-0.017	0.038
Democratic Republic of the Congo	Uvira	2000	0.086	0.518	1.261
Democratic Republic of the Congo	Uvira	2017	0.068	0.207	0.475
Democratic Republic of the Congo	Uvira	2000-2017	-0.078	-0.016	0.034
Democratic Republic of the Congo	Uvira (ville)	2000	0.067	0.509	1.526
Democratic Republic of the Congo	Uvira (ville)	2017	0.019	0.064	0.145
Democratic Republic of the Congo	Uvira (ville)	2000-2017	-0.183	-0.109	-0.032
Democratic Republic of the Congo	Walikale	2000	0.292	0.864	1.539
Democratic Republic of the Congo	Walikale	2017	0.088	0.416	0.921
Democratic Republic of the Congo	Walikale	2000-2017	-0.160	-0.050	0.031
Democratic Republic of the Congo	Walungu	2000	0.069	0.411	1.035
Democratic Republic of the Congo	Walungu	2017	0.060	0.196	0.407
Democratic Republic of the Congo	Walungu	2000-2017	-0.111	-0.041	0.026
Democratic Republic of the Congo	Wamba	2000	0.625	1.282	2.032
Democratic Republic of the Congo	Wamba	2017	0.194	0.709	1.449
Democratic Republic of the Congo	Wamba	2000-2017	-0.126	-0.017	0.051

Table 3: Malaria DALYs rate by unit *(continued)*

Country	Unit	year	mean rate	lower rate	upper rate
Democratic Republic of the Congo	Wamba (ville)	2000	0.461	1.353	2.401
Democratic Republic of the Congo	Wamba (ville)	2017	0.190	0.684	1.608
Democratic Republic of the Congo	Wamba (ville)	2000-2017	-0.122	-0.029	0.075
Democratic Republic of the Congo	Watsa	2000	0.439	0.942	1.562
Democratic Republic of the Congo	Watsa	2017	0.185	0.558	0.979
Democratic Republic of the Congo	Watsa	2000-2017	-0.123	-0.045	-0.012
Democratic Republic of the Congo	Watsa (ville)	2000	0.426	1.095	1.861
Democratic Republic of the Congo	Watsa (ville)	2017	0.186	0.653	1.310
Democratic Republic of the Congo	Watsa (ville)	2000-2017	-0.159	-0.051	-0.014
Democratic Republic of the Congo	Yahuma	2000	0.333	0.993	1.830
Democratic Republic of the Congo	Yahuma	2017	0.122	0.448	1.035
Democratic Republic of the Congo	Yahuma	2000-2017	-0.144	-0.054	-0.003
Democratic Republic of the Congo	Yakoma	2000	0.287	0.856	1.519
Democratic Republic of the Congo	Yakoma	2017	0.142	0.492	0.929
Democratic Republic of the Congo	Yakoma	2000-2017	-0.165	-0.053	-0.007
Democratic Republic of the Congo	Yangambi	2000	0.485	1.262	2.260
Democratic Republic of the Congo	Yangambi	2017	0.155	0.627	1.287
Democratic Republic of the Congo	Yangambi	2000-2017	-0.143	-0.037	0.043
Democratic Republic of the Congo	Yumbi	2000	0.114	0.508	1.287
Democratic Republic of the Congo	Yumbi	2017	0.026	0.150	0.368
Democratic Republic of the Congo	Yumbi	2000-2017	-0.220	-0.097	0.032
Democratic Republic of the Congo	Zongo	2000	0.329	0.686	1.174
Democratic Republic of the Congo	Zongo	2017	0.174	0.449	0.752
Democratic Republic of the Congo	Zongo	2000-2017	-0.136	-0.046	-0.010
Djibouti	Alaili Dadda	2000	0.000	0.005	0.021
Djibouti	Alaili Dadda	2017	0.000	0.002	0.009
Djibouti	Alaili Dadda	2000-2017	-0.206	-0.062	0.128
Djibouti	Ali Sabieh	2000	0.000	0.001	0.002
Djibouti	Ali Sabieh	2017	0.000	0.000	0.000
Djibouti	Ali Sabieh	2000-2017	-0.206	-0.090	0.038
Djibouti	As Eyla	2000	0.000	0.000	0.001
Djibouti	As Eyla	2017	0.000	0.000	0.000
Djibouti	As Eyla	2000-2017	-0.239	-0.087	0.066
Djibouti	Balha	2000	0.000	0.002	0.009
Djibouti	Balha	2017	0.000	0.001	0.003
Djibouti	Balha	2000-2017	-0.188	-0.034	0.148
Djibouti	Dikhil	2000	0.000	0.001	0.002
Djibouti	Dikhil	2017	0.000	0.000	0.000
Djibouti	Dikhil	2000-2017	-0.221	-0.093	0.067
Djibouti	Djibouti	2000	0.000	0.000	0.000
Djibouti	Djibouti	2017	0.000	0.000	0.000
Djibouti	Djibouti	2000-2017	-0.213	-0.069	0.101
Djibouti	Dorra	2000	0.000	0.002	0.009
Djibouti	Dorra	2017	0.000	0.001	0.005
Djibouti	Dorra	2000-2017	-0.181	-0.027	0.185
Djibouti	Obock	2000	0.000	0.002	0.011
Djibouti	Obock	2017	0.000	0.001	0.007
Djibouti	Obock	2000-2017	-0.208	-0.055	0.139
Djibouti	Randa	2000	0.000	0.002	0.007
Djibouti	Randa	2017	0.000	0.001	0.005
Djibouti	Randa	2000-2017	-0.185	-0.019	0.144
Djibouti	Tadjourah	2000	0.000	0.001	0.004
Djibouti	Tadjourah	2017	0.000	0.001	0.003
Djibouti	Tadjourah	2000-2017	-0.181	-0.002	0.162
Djibouti	Yoboki	2000	0.000	0.001	0.002
Djibouti	Yoboki	2017	0.000	0.000	0.000
Djibouti	Yoboki	2000-2017	-0.232	-0.091	0.046
Equatorial Guinea	Aconibe	2000	0.196	0.542	0.936
Equatorial Guinea	Aconibe	2017	0.082	0.215	0.437
Equatorial Guinea	Aconibe	2000-2017	-0.176	-0.070	-0.027
Equatorial Guinea	Acurenam	2000	0.204	0.523	0.921

Table 3: Malaria DALYs rate by unit *(continued)*

Country	Unit	year	mean rate	lower rate	upper rate
Equatorial Guinea	Acurenam	2017	0.105	0.233	0.440
Equatorial Guinea	Acurenam	2000-2017	-0.136	-0.062	-0.025
Equatorial Guinea	Añisok	2000	0.279	0.622	1.014
Equatorial Guinea	Añisok	2017	0.091	0.246	0.476
Equatorial Guinea	Añisok	2000-2017	-0.163	-0.073	-0.028
Equatorial Guinea	Ayene	2000	0.265	0.614	0.987
Equatorial Guinea	Ayene	2017	0.086	0.232	0.451
Equatorial Guinea	Ayene	2000-2017	-0.177	-0.074	-0.028
Equatorial Guinea	Baney	2000	0.293	0.603	1.005
Equatorial Guinea	Baney	2017	0.023	0.143	0.304
Equatorial Guinea	Baney	2000-2017	-0.213	-0.066	0.018
Equatorial Guinea	Bata	2000	0.325	0.640	0.999
Equatorial Guinea	Bata	2017	0.073	0.229	0.460
Equatorial Guinea	Bata	2000-2017	-0.178	-0.069	-0.017
Equatorial Guinea	Bicurga	2000	0.194	0.554	0.960
Equatorial Guinea	Bicurga	2017	0.088	0.256	0.502
Equatorial Guinea	Bicurga	2000-2017	-0.176	-0.067	-0.021
Equatorial Guinea	Bidjabidjan	2000	0.267	0.644	1.023
Equatorial Guinea	Bidjabidjan	2017	0.071	0.208	0.443
Equatorial Guinea	Bidjabidjan	2000-2017	-0.193	-0.081	-0.026
Equatorial Guinea	Bitica	2000	0.269	0.620	0.960
Equatorial Guinea	Bitica	2017	0.105	0.248	0.468
Equatorial Guinea	Bitica	2000-2017	-0.154	-0.067	-0.028
Equatorial Guinea	Ciudad Nueva Oyala	2000	0.237	0.610	1.031
Equatorial Guinea	Ciudad Nueva Oyala	2017	0.100	0.263	0.515
Equatorial Guinea	Ciudad Nueva Oyala	2000-2017	-0.164	-0.070	-0.024
Equatorial Guinea	Cogo	2000	0.282	0.642	0.978
Equatorial Guinea	Cogo	2017	0.110	0.257	0.489
Equatorial Guinea	Cogo	2000-2017	-0.133	-0.065	-0.025
Equatorial Guinea	Corisco	2000	0.000	0.000	0.000
Equatorial Guinea	Corisco	2017	0.000	0.000	0.000
Equatorial Guinea	Corisco	2000-2017	NA	NA	NA
Equatorial Guinea	Ebebiyin	2000	0.245	0.580	0.903
Equatorial Guinea	Ebebiyin	2017	0.059	0.192	0.392
Equatorial Guinea	Ebebiyin	2000-2017	-0.201	-0.079	-0.016
Equatorial Guinea	Evinayong	2000	0.226	0.539	0.934
Equatorial Guinea	Evinayong	2017	0.099	0.243	0.456
Equatorial Guinea	Evinayong	2000-2017	-0.146	-0.065	-0.025
Equatorial Guinea	Luba	2000	0.268	0.590	0.980
Equatorial Guinea	Luba	2017	0.020	0.137	0.267
Equatorial Guinea	Luba	2000-2017	-0.228	-0.060	0.021
Equatorial Guinea	Mabana	2000	0.000	0.000	0.000
Equatorial Guinea	Mabana	2017	0.000	0.000	0.000
Equatorial Guinea	Mabana	2000-2017	NA	NA	NA
Equatorial Guinea	Machinda	2000	0.354	0.699	1.074
Equatorial Guinea	Machinda	2017	0.089	0.246	0.508
Equatorial Guinea	Machinda	2000-2017	-0.178	-0.069	-0.016
Equatorial Guinea	Malabo	2000	0.169	0.369	0.584
Equatorial Guinea	Malabo	2017	0.010	0.081	0.195
Equatorial Guinea	Malabo	2000-2017	-0.220	-0.068	0.023
Equatorial Guinea	Mbini	2000	0.328	0.673	1.036
Equatorial Guinea	Mbini	2017	0.111	0.262	0.505
Equatorial Guinea	Mbini	2000-2017	-0.152	-0.065	-0.027
Equatorial Guinea	Micomeseng	2000	0.283	0.624	0.998
Equatorial Guinea	Micomeseng	2017	0.079	0.242	0.492
Equatorial Guinea	Micomeseng	2000-2017	-0.186	-0.074	-0.027
Equatorial Guinea	Mongomeyen	2000	0.211	0.522	0.874
Equatorial Guinea	Mongomeyen	2017	0.083	0.225	0.433
Equatorial Guinea	Mongomeyen	2000-2017	-0.180	-0.067	-0.027
Equatorial Guinea	Mongomo	2000	0.264	0.584	0.913
Equatorial Guinea	Mongomo	2017	0.071	0.202	0.404

Table 3: Malaria DALYs rate by unit *(continued)*

Country	Unit	year	mean rate	lower rate	upper rate
Equatorial Guinea	Mongomo	2000-2017	-0.182	-0.077	-0.031
Equatorial Guinea	Nasng	2000	0.279	0.624	0.989
Equatorial Guinea	Nasng	2017	0.069	0.215	0.411
Equatorial Guinea	Nasng	2000-2017	-0.182	-0.078	-0.023
Equatorial Guinea	Niefang	2000	0.295	0.637	1.025
Equatorial Guinea	Niefang	2017	0.109	0.265	0.542
Equatorial Guinea	Niefang	2000-2017	-0.162	-0.070	-0.026
Equatorial Guinea	Nkimi	2000	0.320	0.690	1.081
Equatorial Guinea	Nkimi	2017	0.099	0.259	0.542
Equatorial Guinea	Nkimi	2000-2017	-0.167	-0.073	-0.030
Equatorial Guinea	Nkue	2000	0.284	0.633	0.999
Equatorial Guinea	Nkue	2017	0.068	0.224	0.431
Equatorial Guinea	Nkue	2000-2017	-0.182	-0.077	-0.030
Equatorial Guinea	Nsok-Nsomo	2000	0.293	0.636	1.003
Equatorial Guinea	Nsok-Nsomo	2017	0.077	0.221	0.410
Equatorial Guinea	Nsok-Nsomo	2000-2017	-0.179	-0.078	-0.029
Equatorial Guinea	Nsork	2000	0.177	0.538	0.993
Equatorial Guinea	Nsork	2017	0.083	0.204	0.450
Equatorial Guinea	Nsork	2000-2017	-0.166	-0.070	-0.026
Equatorial Guinea	Rebola	2000	0.332	0.701	1.117
Equatorial Guinea	Rebola	2017	0.027	0.167	0.344
Equatorial Guinea	Rebola	2000-2017	-0.214	-0.068	0.011
Equatorial Guinea	Riaba	2000	0.361	0.722	1.178
Equatorial Guinea	Riaba	2017	0.026	0.167	0.326
Equatorial Guinea	Riaba	2000-2017	-0.229	-0.064	0.011
Equatorial Guinea	Rio Campo	2000	0.223	0.581	0.923
Equatorial Guinea	Rio Campo	2017	0.109	0.233	0.421
Equatorial Guinea	Rio Campo	2000-2017	-0.131	-0.065	-0.026
Equatorial Guinea	San Antonio de Palé	2000	0.000	0.000	0.000
Equatorial Guinea	San Antonio de Palé	2017	0.000	0.000	0.000
Equatorial Guinea	San Antonio de Palé	2000-2017	NA	NA	NA
Eritrea	Adi Keyih	2000	0.000	0.000	0.001
Eritrea	Adi Keyih	2017	0.000	0.000	0.001
Eritrea	Adi Keyih	2000-2017	-0.275	-0.097	0.096
Eritrea	Adi Kwala	2000	0.001	0.002	0.003
Eritrea	Adi Kwala	2017	0.000	0.000	0.001
Eritrea	Adi Kwala	2000-2017	-0.273	-0.108	0.044
Eritrea	Adi Teklezan	2000	0.000	0.000	0.000
Eritrea	Adi Teklezan	2017	0.000	0.000	0.000
Eritrea	Adi Teklezan	2000-2017	-0.240	-0.076	0.062
Eritrea	Afabet	2000	0.000	0.000	0.000
Eritrea	Afabet	2017	0.000	0.000	0.000
Eritrea	Afabet	2000-2017	-0.227	-0.072	0.069
Eritrea	Akordat	2000	0.000	0.001	0.001
Eritrea	Akordat	2017	0.000	0.000	0.001
Eritrea	Akordat	2000-2017	-0.175	-0.032	0.114
Eritrea	Areta'	2000	0.000	0.001	0.003
Eritrea	Areta'	2017	0.000	0.000	0.001
Eritrea	Areta'	2000-2017	-0.216	-0.083	0.087
Eritrea	Areza	2000	0.000	0.001	0.001
Eritrea	Areza	2017	0.000	0.000	0.001
Eritrea	Areza	2000-2017	-0.211	-0.072	0.060
Eritrea	Asmara City	2000	0.000	0.000	0.000
Eritrea	Asmara City	2017	0.000	0.000	0.000
Eritrea	Asmara City	2000-2017	-0.234	-0.090	0.092
Eritrea	Asmat	2000	0.000	0.000	0.000
Eritrea	Asmat	2017	0.000	0.000	0.000
Eritrea	Asmat	2000-2017	-0.209	-0.055	0.118
Eritrea	Barentu	2000	0.001	0.002	0.003
Eritrea	Barentu	2017	0.000	0.001	0.002
Eritrea	Barentu	2000-2017	-0.131	-0.015	0.112

Table 3: Malaria DALYs rate by unit *(continued)*

Country	Unit	year	mean rate	lower rate	upper rate
Eritrea	Berikh	2000	0.000	0.000	0.000
Eritrea	Berikh	2017	0.000	0.000	0.000
Eritrea	Berikh	2000-2017	-0.209	-0.077	0.094
Eritrea	Central So. Red-Sea	2000	0.000	0.001	0.002
Eritrea	Central So. Red-Sea	2017	0.000	0.000	0.001
Eritrea	Central So. Red-Sea	2000-2017	-0.264	-0.082	0.063
Eritrea	Dahlak	2000	0.000	0.000	0.002
Eritrea	Dahlak	2017	0.000	0.000	0.001
Eritrea	Dahlak	2000-2017	-0.257	-0.067	0.090
Eritrea	Dekemehare	2000	0.000	0.000	0.001
Eritrea	Dekemehare	2017	0.000	0.000	0.001
Eritrea	Dekemehare	2000-2017	-0.222	-0.085	0.084
Eritrea	Dghe	2000	0.000	0.000	0.001
Eritrea	Dghe	2017	0.000	0.000	0.001
Eritrea	Dghe	2000-2017	-0.131	-0.001	0.118
Eritrea	Dibarwa	2000	0.000	0.000	0.001
Eritrea	Dibarwa	2017	0.000	0.000	0.001
Eritrea	Dibarwa	2000-2017	-0.200	-0.075	0.087
Eritrea	Elabered	2000	0.000	0.000	0.000
Eritrea	Elabered	2017	0.000	0.000	0.001
Eritrea	Elabered	2000-2017	-0.223	-0.071	0.087
Eritrea	Foro	2000	0.000	0.001	0.001
Eritrea	Foro	2017	0.000	0.000	0.001
Eritrea	Foro	2000-2017	-0.293	-0.092	0.093
Eritrea	Forto	2000	0.000	0.001	0.003
Eritrea	Forto	2017	0.000	0.001	0.004
Eritrea	Forto	2000-2017	-0.158	0.016	0.145
Eritrea	Ghala Nefhi	2000	0.000	0.000	0.001
Eritrea	Ghala Nefhi	2017	0.000	0.000	0.001
Eritrea	Ghala Nefhi	2000-2017	-0.217	-0.075	0.098
Eritrea	Ghelaelo’	2000	0.000	0.001	0.002
Eritrea	Ghelaelo’	2017	0.000	0.000	0.001
Eritrea	Ghelaelo’	2000-2017	-0.250	-0.099	0.079
Eritrea	Gheleb	2000	0.000	0.000	0.000
Eritrea	Gheleb	2017	0.000	0.000	0.000
Eritrea	Gheleb	2000-2017	-0.250	-0.078	0.100
Eritrea	Ghida‘e	2000	0.000	0.000	0.001
Eritrea	Ghida‘e	2017	0.000	0.000	0.001
Eritrea	Ghida‘e	2000-2017	-0.252	-0.088	0.071
Eritrea	Gogne	2000	0.000	0.001	0.003
Eritrea	Gogne	2017	0.000	0.001	0.002
Eritrea	Gogne	2000-2017	-0.145	-0.004	0.132
Eritrea	Habero	2000	0.000	0.000	0.000
Eritrea	Habero	2017	0.000	0.000	0.000
Eritrea	Habero	2000-2017	-0.215	-0.065	0.099
Eritrea	Hagaz	2000	0.000	0.000	0.001
Eritrea	Hagaz	2017	0.000	0.000	0.000
Eritrea	Hagaz	2000-2017	-0.188	-0.059	0.089
Eritrea	Halhal	2000	0.000	0.000	0.000
Eritrea	Halhal	2017	0.000	0.000	0.000
Eritrea	Halhal	2000-2017	-0.187	-0.066	0.084
Eritrea	Haykota	2000	0.000	0.001	0.001
Eritrea	Haykota	2017	0.000	0.001	0.002
Eritrea	Haykota	2000-2017	-0.102	0.028	0.168
Eritrea	Karora	2000	0.000	0.000	0.001
Eritrea	Karora	2017	0.000	0.000	0.001
Eritrea	Karora	2000-2017	-0.213	-0.028	0.130
Eritrea	Keren	2000	0.000	0.000	0.000
Eritrea	Keren	2017	0.000	0.000	0.000
Eritrea	Keren	2000-2017	-0.217	-0.070	0.089
Eritrea	Kerke Bet	2000	0.000	0.000	0.000

Table 3: Malaria DALYs rate by unit *(continued)*

Country	Unit	year	mean rate	lower rate	upper rate
Eritrea	Kerke Bet	2017	0.000	0.000	0.001
Eritrea	Kerke Bet	2000-2017	-0.171	-0.014	0.125
Eritrea	Kudo Bu‘er	2000	0.000	0.001	0.002
Eritrea	Kudo Bu‘er	2017	0.000	0.000	0.001
Eritrea	Kudo Bu‘er	2000-2017	-0.236	-0.087	0.044
Eritrea	La‘Elay Gash	2000	0.001	0.005	0.009
Eritrea	La‘Elay Gash	2017	0.000	0.001	0.003
Eritrea	La‘Elay Gash	2000-2017	-0.170	-0.040	0.075
Eritrea	Logo Anseba	2000	0.000	0.000	0.001
Eritrea	Logo Anseba	2017	0.000	0.000	0.001
Eritrea	Logo Anseba	2000-2017	-0.219	-0.069	0.054
Eritrea	Mansura	2000	0.000	0.000	0.001
Eritrea	Mansura	2017	0.000	0.000	0.001
Eritrea	Mansura	2000-2017	-0.183	-0.051	0.071
Eritrea	May Mine	2000	0.001	0.002	0.005
Eritrea	May Mine	2017	0.000	0.000	0.001
Eritrea	May Mine	2000-2017	-0.265	-0.113	0.014
Eritrea	Mendefera	2000	0.000	0.001	0.001
Eritrea	Mendefera	2017	0.000	0.000	0.001
Eritrea	Mendefera	2000-2017	-0.209	-0.082	0.064
Eritrea	Mitswa‘e City	2000	0.000	0.000	0.001
Eritrea	Mitswa‘e City	2017	0.000	0.000	0.000
Eritrea	Mitswa‘e City	2000-2017	-0.268	-0.085	0.058
Eritrea	Mogolo	2000	0.000	0.001	0.002
Eritrea	Mogolo	2017	0.000	0.000	0.001
Eritrea	Mogolo	2000-2017	-0.132	-0.016	0.114
Eritrea	Nakfa	2000	0.000	0.000	0.000
Eritrea	Nakfa	2017	0.000	0.000	0.000
Eritrea	Nakfa	2000-2017	-0.259	-0.071	0.092
Eritrea	Omhajer	2000	0.001	0.003	0.007
Eritrea	Omhajer	2017	0.000	0.002	0.005
Eritrea	Omhajer	2000-2017	-0.139	-0.012	0.128
Eritrea	Segeneyiti	2000	0.000	0.000	0.001
Eritrea	Segeneyiti	2017	0.000	0.000	0.001
Eritrea	Segeneyiti	2000-2017	-0.246	-0.091	0.090
Eritrea	Sel‘a	2000	0.000	0.000	0.001
Eritrea	Sel‘a	2017	0.000	0.000	0.001
Eritrea	Sel‘a	2000-2017	-0.152	-0.004	0.142
Eritrea	Senafe	2000	0.000	0.001	0.002
Eritrea	Senafe	2017	0.000	0.000	0.001
Eritrea	Senafe	2000-2017	-0.261	-0.115	0.052
Eritrea	Serejeka	2000	0.000	0.000	0.000
Eritrea	Serejeka	2017	0.000	0.000	0.000
Eritrea	Serejeka	2000-2017	-0.217	-0.081	0.081
Eritrea	Sheib	2000	0.000	0.000	0.000
Eritrea	Sheib	2017	0.000	0.000	0.000
Eritrea	Sheib	2000-2017	-0.243	-0.082	0.069
Eritrea	Shemboko	2000	0.001	0.002	0.004
Eritrea	Shemboko	2017	0.000	0.001	0.002
Eritrea	Shemboko	2000-2017	-0.177	-0.047	0.072
Eritrea	So. Southern Red-Sea	2000	0.000	0.001	0.005
Eritrea	So. Southern Red-Sea	2017	0.000	0.000	0.002
Eritrea	So. Southern Red-Sea	2000-2017	-0.239	-0.086	0.096
Eritrea	Teseneye	2000	0.000	0.001	0.002
Eritrea	Teseneye	2017	0.000	0.001	0.003
Eritrea	Teseneye	2000-2017	-0.118	0.036	0.186
Eritrea	Tsorena	2000	0.001	0.002	0.003
Eritrea	Tsorena	2017	0.000	0.000	0.001
Eritrea	Tsorena	2000-2017	-0.281	-0.123	0.027
Ethiopia	Addis Abeba	2000	0.000	0.001	0.004
Ethiopia	Addis Abeba	2017	0.000	0.000	0.001

Table 3: Malaria DALYs rate by unit *(continued)*

Country	Unit	year	mean rate	lower rate	upper rate
Ethiopia	Addis Abeba	2000-2017	-0.264	-0.136	0.042
Ethiopia	Afar Zone 1	2000	0.013	0.079	0.268
Ethiopia	Afar Zone 1	2017	0.001	0.007	0.020
Ethiopia	Afar Zone 1	2000-2017	-0.244	-0.127	-0.010
Ethiopia	Afar Zone 2	2000	0.010	0.051	0.117
Ethiopia	Afar Zone 2	2017	0.001	0.005	0.012
Ethiopia	Afar Zone 2	2000-2017	-0.262	-0.140	-0.024
Ethiopia	Afar Zone 3	2000	0.017	0.090	0.264
Ethiopia	Afar Zone 3	2017	0.003	0.016	0.051
Ethiopia	Afar Zone 3	2000-2017	-0.268	-0.112	-0.012
Ethiopia	Afar Zone 4	2000	0.016	0.093	0.321
Ethiopia	Afar Zone 4	2017	0.001	0.009	0.030
Ethiopia	Afar Zone 4	2000-2017	-0.280	-0.144	-0.042
Ethiopia	Afar Zone 5	2000	0.022	0.121	0.367
Ethiopia	Afar Zone 5	2017	0.002	0.014	0.043
Ethiopia	Afar Zone 5	2000-2017	-0.250	-0.118	-0.011
Ethiopia	Afder	2000	0.004	0.027	0.105
Ethiopia	Afder	2017	0.002	0.011	0.032
Ethiopia	Afder	2000-2017	-0.152	-0.045	0.067
Ethiopia	Agew Awi	2000	0.004	0.046	0.204
Ethiopia	Agew Awi	2017	0.001	0.006	0.018
Ethiopia	Agew Awi	2000-2017	-0.192	-0.060	0.080
Ethiopia	Agnuak	2000	0.016	0.131	0.383
Ethiopia	Agnuak	2017	0.001	0.009	0.032
Ethiopia	Agnuak	2000-2017	-0.210	-0.099	0.026
Ethiopia	Alaba	2000	0.018	0.115	0.373
Ethiopia	Alaba	2017	0.002	0.010	0.030
Ethiopia	Alaba	2000-2017	-0.280	-0.159	-0.045
Ethiopia	Alle	2000	0.009	0.073	0.255
Ethiopia	Alle	2017	0.001	0.005	0.022
Ethiopia	Alle	2000-2017	-0.324	-0.161	0.003
Ethiopia	Amaro	2000	0.011	0.080	0.297
Ethiopia	Amaro	2017	0.001	0.007	0.026
Ethiopia	Amaro	2000-2017	-0.302	-0.141	0.022
Ethiopia	Argoba	2000	0.021	0.128	0.462
Ethiopia	Argoba	2017	0.002	0.012	0.041
Ethiopia	Argoba	2000-2017	-0.255	-0.128	-0.006
Ethiopia	Arsi	2000	0.004	0.024	0.083
Ethiopia	Arsi	2017	0.000	0.002	0.006
Ethiopia	Arsi	2000-2017	-0.272	-0.154	-0.029
Ethiopia	Asosa	2000	0.013	0.097	0.363
Ethiopia	Asosa	2017	0.001	0.010	0.029
Ethiopia	Asosa	2000-2017	-0.245	-0.134	-0.007
Ethiopia	Bahir Dar Special Zone	2000	0.004	0.051	0.241
Ethiopia	Bahir Dar Special Zone	2017	0.001	0.005	0.014
Ethiopia	Bahir Dar Special Zone	2000-2017	-0.227	-0.097	0.044
Ethiopia	Bale	2000	0.004	0.040	0.127
Ethiopia	Bale	2017	0.001	0.003	0.010
Ethiopia	Bale	2000-2017	-0.234	-0.112	0.009
Ethiopia	Basketo	2000	0.009	0.106	0.384
Ethiopia	Basketo	2017	0.001	0.006	0.022
Ethiopia	Basketo	2000-2017	-0.306	-0.131	0.025
Ethiopia	Bench Maji	2000	0.021	0.198	0.662
Ethiopia	Bench Maji	2017	0.001	0.011	0.034
Ethiopia	Bench Maji	2000-2017	-0.247	-0.117	0.043
Ethiopia	Borena	2000	0.011	0.084	0.297
Ethiopia	Borena	2017	0.001	0.005	0.015
Ethiopia	Borena	2000-2017	-0.254	-0.138	-0.013
Ethiopia	Burji	2000	0.008	0.070	0.223
Ethiopia	Burji	2017	0.001	0.005	0.023
Ethiopia	Burji	2000-2017	-0.324	-0.152	0.025

Table 3: Malaria DALYs rate by unit *(continued)*

Country	Unit	year	mean rate	lower rate	upper rate
Ethiopia	Dawro	2000	0.017	0.129	0.505
Ethiopia	Dawro	2017	0.001	0.007	0.023
Ethiopia	Dawro	2000-2017	-0.293	-0.145	-0.001
Ethiopia	Debub Gondar	2000	0.006	0.061	0.249
Ethiopia	Debub Gondar	2017	0.001	0.005	0.015
Ethiopia	Debub Gondar	2000-2017	-0.284	-0.163	-0.042
Ethiopia	Debub Mirab Shewa	2000	0.014	0.063	0.160
Ethiopia	Debub Mirab Shewa	2017	0.001	0.005	0.013
Ethiopia	Debub Mirab Shewa	2000-2017	-0.300	-0.151	-0.002
Ethiopia	Debub Omo	2000	0.011	0.109	0.375
Ethiopia	Debub Omo	2017	0.001	0.007	0.026
Ethiopia	Debub Omo	2000-2017	-0.266	-0.116	0.026
Ethiopia	Debub Wollo	2000	0.006	0.036	0.137
Ethiopia	Debub Wollo	2017	0.001	0.003	0.011
Ethiopia	Debub Wollo	2000-2017	-0.286	-0.160	-0.048
Ethiopia	Debubawi	2000	0.009	0.046	0.139
Ethiopia	Debubawi	2017	0.000	0.004	0.010
Ethiopia	Debubawi	2000-2017	-0.295	-0.162	-0.046
Ethiopia	Derashe	2000	0.012	0.087	0.342
Ethiopia	Derashe	2017	0.001	0.005	0.027
Ethiopia	Derashe	2000-2017	-0.330	-0.169	-0.008
Ethiopia	Dire Dawa	2000	0.006	0.045	0.168
Ethiopia	Dire Dawa	2017	0.000	0.002	0.007
Ethiopia	Dire Dawa	2000-2017	-0.326	-0.177	-0.067
Ethiopia	Doolo	2000	0.004	0.023	0.080
Ethiopia	Doolo	2017	0.001	0.007	0.027
Ethiopia	Doolo	2000-2017	-0.224	-0.088	0.042
Ethiopia	Fafan	2000	0.009	0.056	0.192
Ethiopia	Fafan	2017	0.001	0.003	0.010
Ethiopia	Fafan	2000-2017	-0.247	-0.137	-0.009
Ethiopia	Gamo Gofa	2000	0.017	0.098	0.328
Ethiopia	Gamo Gofa	2017	0.001	0.007	0.020
Ethiopia	Gamo Gofa	2000-2017	-0.276	-0.131	0.003
Ethiopia	Gedeo	2000	0.014	0.137	0.511
Ethiopia	Gedeo	2017	0.001	0.008	0.020
Ethiopia	Gedeo	2000-2017	-0.293	-0.156	-0.029
Ethiopia	Guji	2000	0.007	0.080	0.256
Ethiopia	Guji	2017	0.001	0.004	0.014
Ethiopia	Guji	2000-2017	-0.269	-0.134	0.020
Ethiopia	Gurage	2000	0.012	0.078	0.217
Ethiopia	Gurage	2017	0.001	0.006	0.019
Ethiopia	Gurage	2000-2017	-0.285	-0.146	-0.002
Ethiopia	Hadiya	2000	0.022	0.146	0.476
Ethiopia	Hadiya	2017	0.002	0.009	0.028
Ethiopia	Hadiya	2000-2017	-0.274	-0.172	-0.072
Ethiopia	Hareri	2000	0.003	0.039	0.137
Ethiopia	Hareri	2017	0.000	0.002	0.009
Ethiopia	Hareri	2000-2017	-0.334	-0.192	-0.021
Ethiopia	Horo Guduru	2000	0.005	0.034	0.108
Ethiopia	Horo Guduru	2017	0.001	0.005	0.015
Ethiopia	Horo Guduru	2000-2017	-0.222	-0.077	0.108
Ethiopia	Ilubabor	2000	0.012	0.112	0.489
Ethiopia	Ilubabor	2017	0.001	0.006	0.018
Ethiopia	Ilubabor	2000-2017	-0.200	-0.106	0.017
Ethiopia	Jarar	2000	0.011	0.057	0.252
Ethiopia	Jarar	2017	0.001	0.006	0.020
Ethiopia	Jarar	2000-2017	-0.263	-0.139	-0.027
Ethiopia	Jimma	2000	0.010	0.097	0.361
Ethiopia	Jimma	2017	0.001	0.005	0.016
Ethiopia	Jimma	2000-2017	-0.244	-0.150	-0.024
Ethiopia	Keffa	2000	0.019	0.168	0.677

Table 3: Malaria DALYs rate by unit *(continued)*

Country	Unit	year	mean rate	lower rate	upper rate
Ethiopia	Keffa	2017	0.002	0.009	0.030
Ethiopia	Keffa	2000-2017	-0.222	-0.114	0.040
Ethiopia	Kelem Wellega	2000	0.005	0.074	0.288
Ethiopia	Kelem Wellega	2017	0.001	0.005	0.013
Ethiopia	Kelem Wellega	2000-2017	-0.216	-0.094	0.029
Ethiopia	Kemashi	2000	0.013	0.097	0.317
Ethiopia	Kemashi	2017	0.001	0.008	0.026
Ethiopia	Kemashi	2000-2017	-0.216	-0.112	-0.005
Ethiopia	Kembata Tembaro	2000	0.022	0.153	0.479
Ethiopia	Kembata Tembaro	2017	0.002	0.010	0.032
Ethiopia	Kembata Tembaro	2000-2017	-0.291	-0.171	-0.068
Ethiopia	Konso	2000	0.010	0.079	0.294
Ethiopia	Konso	2017	0.001	0.005	0.020
Ethiopia	Konso	2000-2017	-0.326	-0.173	-0.007
Ethiopia	Konta	2000	0.018	0.151	0.573
Ethiopia	Konta	2017	0.001	0.008	0.023
Ethiopia	Konta	2000-2017	-0.272	-0.131	0.033
Ethiopia	Korahe	2000	0.003	0.028	0.098
Ethiopia	Korahe	2017	0.001	0.008	0.030
Ethiopia	Korahe	2000-2017	-0.242	-0.090	0.059
Ethiopia	Liben	2000	0.005	0.043	0.177
Ethiopia	Liben	2017	0.002	0.008	0.022
Ethiopia	Liben	2000-2017	-0.154	-0.052	0.078
Ethiopia	Majang	2000	0.023	0.363	1.219
Ethiopia	Majang	2017	0.001	0.014	0.048
Ethiopia	Majang	2000-2017	-0.278	-0.115	0.067
Ethiopia	Mehakelegnaw	2000	0.013	0.036	0.088
Ethiopia	Mehakelegnaw	2017	0.001	0.003	0.010
Ethiopia	Mehakelegnaw	2000-2017	-0.294	-0.152	-0.028
Ethiopia	Metekel	2000	0.013	0.095	0.364
Ethiopia	Metekel	2017	0.003	0.013	0.041
Ethiopia	Metekel	2000-2017	-0.202	-0.070	0.077
Ethiopia	Mi'irabawi	2000	0.025	0.108	0.268
Ethiopia	Mi'irabawi	2017	0.002	0.009	0.024
Ethiopia	Mi'irabawi	2000-2017	-0.214	-0.101	0.027
Ethiopia	Mirab Arsi	2000	0.004	0.022	0.063
Ethiopia	Mirab Arsi	2017	0.001	0.002	0.007
Ethiopia	Mirab Arsi	2000-2017	-0.260	-0.151	-0.036
Ethiopia	Mirab Gojjam	2000	0.008	0.058	0.249
Ethiopia	Mirab Gojjam	2017	0.002	0.008	0.021
Ethiopia	Mirab Gojjam	2000-2017	-0.192	-0.070	0.056
Ethiopia	Mirab Hararghe	2000	0.008	0.056	0.186
Ethiopia	Mirab Hararghe	2017	0.001	0.005	0.014
Ethiopia	Mirab Hararghe	2000-2017	-0.268	-0.138	-0.023
Ethiopia	Mirab Shewa	2000	0.007	0.039	0.119
Ethiopia	Mirab Shewa	2017	0.001	0.004	0.011
Ethiopia	Mirab Shewa	2000-2017	-0.257	-0.130	0.005
Ethiopia	Mirab Welega	2000	0.008	0.082	0.293
Ethiopia	Mirab Welega	2017	0.001	0.005	0.015
Ethiopia	Mirab Welega	2000-2017	-0.214	-0.098	0.013
Ethiopia	Misraq Gojjam	2000	0.004	0.035	0.150
Ethiopia	Misraq Gojjam	2017	0.001	0.005	0.014
Ethiopia	Misraq Gojjam	2000-2017	-0.251	-0.107	0.032
Ethiopia	Misraq Harerge	2000	0.007	0.046	0.154
Ethiopia	Misraq Harerge	2017	0.000	0.003	0.009
Ethiopia	Misraq Harerge	2000-2017	-0.271	-0.159	-0.048
Ethiopia	Misraq Shewa	2000	0.013	0.063	0.171
Ethiopia	Misraq Shewa	2017	0.001	0.005	0.013
Ethiopia	Misraq Shewa	2000-2017	-0.283	-0.166	-0.043
Ethiopia	Misraq Wellega	2000	0.006	0.060	0.204
Ethiopia	Misraq Wellega	2017	0.001	0.005	0.013

Table 3: Malaria DALYs rate by unit *(continued)*

Country	Unit	year	mean rate	lower rate	upper rate
Ethiopia	Misraq Wellega	2000-2017	-0.220	-0.099	0.033
Ethiopia	Misraqawi	2000	0.005	0.018	0.051
Ethiopia	Misraqawi	2017	0.000	0.002	0.005
Ethiopia	Misraqawi	2000-2017	-0.271	-0.157	-0.024
Ethiopia	Nogob	2000	0.004	0.045	0.141
Ethiopia	Nogob	2017	0.001	0.006	0.017
Ethiopia	Nogob	2000-2017	-0.232	-0.102	0.029
Ethiopia	North Shewa	2000	0.003	0.025	0.082
Ethiopia	North Shewa	2000	0.008	0.052	0.185
Ethiopia	North Shewa	2017	0.000	0.003	0.009
Ethiopia	North Shewa	2017	0.001	0.007	0.021
Ethiopia	North Shewa	2000-2017	-0.244	-0.132	-0.028
Ethiopia	North Shewa	2000-2017	-0.262	-0.140	0.003
Ethiopia	Nuer	2000	0.015	0.118	0.384
Ethiopia	Nuer	2017	0.002	0.018	0.071
Ethiopia	Nuer	2000-2017	-0.222	-0.084	0.046
Ethiopia	Oromia	2000	0.025	0.141	0.510
Ethiopia	Oromia	2017	0.002	0.013	0.042
Ethiopia	Oromia	2000-2017	-0.255	-0.131	-0.034
Ethiopia	Semen Gondar	2000	0.017	0.083	0.241
Ethiopia	Semen Gondar	2017	0.001	0.008	0.019
Ethiopia	Semen Gondar	2000-2017	-0.217	-0.116	-0.008
Ethiopia	Semen Wello	2000	0.006	0.050	0.161
Ethiopia	Semen Wello	2017	0.001	0.004	0.013
Ethiopia	Semen Wello	2000-2017	-0.353	-0.199	-0.063
Ethiopia	Semien Mi'irabaw	2000	0.027	0.094	0.200
Ethiopia	Semien Mi'irabaw	2017	0.002	0.006	0.018
Ethiopia	Semien Mi'irabaw	2000-2017	-0.235	-0.128	-0.012
Ethiopia	Shabelle	2000	0.003	0.018	0.053
Ethiopia	Shabelle	2017	0.001	0.008	0.027
Ethiopia	Shabelle	2000-2017	-0.161	-0.058	0.079
Ethiopia	Sheka	2000	0.016	0.219	0.739
Ethiopia	Sheka	2017	0.001	0.010	0.032
Ethiopia	Sheka	2000-2017	-0.256	-0.108	0.067
Ethiopia	Sidama	2000	0.013	0.087	0.262
Ethiopia	Sidama	2017	0.001	0.008	0.026
Ethiopia	Sidama	2000-2017	-0.260	-0.154	-0.031
Ethiopia	Silti	2000	0.011	0.088	0.312
Ethiopia	Silti	2017	0.001	0.009	0.035
Ethiopia	Silti	2000-2017	-0.283	-0.134	0.006
Ethiopia	Siti	2000	0.014	0.069	0.201
Ethiopia	Siti	2017	0.001	0.006	0.015
Ethiopia	Siti	2000-2017	-0.259	-0.135	-0.037
Ethiopia	Wag Himra	2000	0.008	0.050	0.146
Ethiopia	Wag Himra	2017	0.000	0.004	0.015
Ethiopia	Wag Himra	2000-2017	-0.320	-0.174	-0.027
Ethiopia	Wolayita	2000	0.026	0.182	0.596
Ethiopia	Wolayita	2017	0.002	0.013	0.039
Ethiopia	Wolayita	2000-2017	-0.269	-0.151	-0.038
Ethiopia	Yem	2000	0.005	0.079	0.259
Ethiopia	Yem	2017	0.001	0.004	0.013
Ethiopia	Yem	2000-2017	-0.333	-0.193	-0.059
Gabon	Abanga-Bigné	2000	0.093	0.275	0.483
Gabon	Abanga-Bigné	2017	0.019	0.133	0.322
Gabon	Abanga-Bigné	2000-2017	-0.098	-0.014	0.096
Gabon	Basse Banio	2000	0.027	0.135	0.385
Gabon	Basse Banio	2017	0.009	0.110	0.349
Gabon	Basse Banio	2000-2017	-0.171	-0.021	0.104
Gabon	Bendjé	2000	0.036	0.186	0.374
Gabon	Bendjé	2017	0.016	0.098	0.262
Gabon	Bendjé	2000-2017	-0.099	0.012	0.138

Table 3: Malaria DALYs rate by unit *(continued)*

Country	Unit	year	mean rate	lower rate	upper rate
Gabon	Boumi-lowetsi	2000	0.034	0.200	0.540
Gabon	Boumi-lowetsi	2017	0.015	0.136	0.320
Gabon	Boumi-lowetsi	2000-2017	-0.152	0.012	0.121
Gabon	Dola	2000	0.019	0.128	0.294
Gabon	Dola	2017	0.010	0.088	0.251
Gabon	Dola	2000-2017	-0.126	0.016	0.133
Gabon	Douigny	2000	0.025	0.143	0.401
Gabon	Douigny	2017	0.011	0.111	0.345
Gabon	Douigny	2000-2017	-0.145	0.004	0.143
Gabon	Douya Onoye	2000	0.038	0.172	0.355
Gabon	Douya Onoye	2017	0.017	0.120	0.314
Gabon	Douya Onoye	2000-2017	-0.118	0.020	0.124
Gabon	Étimboué	2000	0.029	0.161	0.308
Gabon	Étimboué	2017	0.018	0.092	0.289
Gabon	Étimboué	2000-2017	-0.097	0.014	0.106
Gabon	Haut-Como	2000	0.064	0.227	0.504
Gabon	Haut-Como	2017	0.024	0.142	0.371
Gabon	Haut-Como	2000-2017	-0.174	-0.052	0.048
Gabon	Haut-Ntem	2000	0.033	0.190	0.617
Gabon	Haut-Ntem	2017	0.016	0.124	0.395
Gabon	Haut-Ntem	2000-2017	-0.149	-0.006	0.103
Gabon	Haute-Banio	2000	0.025	0.156	0.383
Gabon	Haute-Banio	2017	0.016	0.106	0.317
Gabon	Haute-Banio	2000-2017	-0.167	-0.041	0.086
Gabon	Ivindo	2000	0.041	0.192	0.465
Gabon	Ivindo	2017	0.021	0.140	0.352
Gabon	Ivindo	2000-2017	-0.102	0.022	0.108
Gabon	Komo	2000	0.136	0.302	0.497
Gabon	Komo	2017	0.036	0.156	0.346
Gabon	Komo	2000-2017	-0.137	-0.028	0.067
Gabon	Komo-Mondah	2000	0.056	0.189	0.384
Gabon	Komo-Mondah	2017	0.019	0.085	0.195
Gabon	Komo-Mondah	2000-2017	-0.161	-0.032	0.073
Gabon	Léboumbi-Leyou	2000	0.078	0.251	0.484
Gabon	Léboumbi-Leyou	2017	0.017	0.150	0.377
Gabon	Léboumbi-Leyou	2000-2017	-0.155	-0.039	0.062
Gabon	Léconi-Djoué	2000	0.048	0.234	0.454
Gabon	Léconi-Djoué	2017	0.018	0.159	0.399
Gabon	Léconi-Djoué	2000-2017	-0.182	-0.030	0.094
Gabon	Lékoko	2000	0.133	0.333	0.537
Gabon	Lékoko	2017	0.038	0.189	0.443
Gabon	Lékoko	2000-2017	-0.148	-0.039	0.043
Gabon	Lolo Bouenguidi	2000	0.063	0.253	0.495
Gabon	Lolo Bouenguidi	2017	0.026	0.149	0.345
Gabon	Lolo Bouenguidi	2000-2017	-0.101	0.010	0.091
Gabon	Lombo-Bouenguidi	2000	0.086	0.290	0.525
Gabon	Lombo-Bouenguidi	2017	0.027	0.156	0.352
Gabon	Lombo-Bouenguidi	2000-2017	-0.143	-0.017	0.074
Gabon	Lopé	2000	0.043	0.203	0.480
Gabon	Lopé	2017	0.018	0.134	0.362
Gabon	Lopé	2000-2017	-0.097	0.027	0.124
Gabon	Louetsi-Wano	2000	0.022	0.171	0.426
Gabon	Louetsi-Wano	2017	0.011	0.124	0.324
Gabon	Louetsi-Wano	2000-2017	-0.122	0.029	0.139
Gabon	Mougoutsi	2000	0.023	0.114	0.284
Gabon	Mougoutsi	2017	0.008	0.088	0.228
Gabon	Mougoutsi	2000-2017	-0.169	-0.010	0.110
Gabon	Mouloudnou	2000	0.075	0.256	0.514
Gabon	Mouloudnou	2017	0.023	0.164	0.404
Gabon	Mouloudnou	2000-2017	-0.095	0.006	0.092
Gabon	Mpassa	2000	0.067	0.224	0.421

Table 3: Malaria DALYs rate by unit *(continued)*

Country	Unit	year	mean rate	lower rate	upper rate
Gabon	Mpassa	2017	0.022	0.145	0.364
Gabon	Mpassa	2000-2017	-0.150	-0.022	0.091
Gabon	Mvoung	2000	0.043	0.209	0.525
Gabon	Mvoung	2017	0.030	0.158	0.436
Gabon	Mvoung	2000-2017	-0.102	0.030	0.148
Gabon	Ndolou	2000	0.064	0.211	0.415
Gabon	Ndolou	2017	0.021	0.120	0.342
Gabon	Ndolou	2000-2017	-0.107	0.007	0.100
Gabon	Ndougou	2000	0.019	0.138	0.397
Gabon	Ndougou	2017	0.008	0.098	0.351
Gabon	Ndougou	2000-2017	-0.103	0.017	0.139
Gabon	Noya	2000	0.150	0.337	0.576
Gabon	Noya	2017	0.054	0.175	0.386
Gabon	Noya	2000-2017	-0.172	-0.069	0.000
Gabon	Ntem	2000	0.061	0.247	0.513
Gabon	Ntem	2017	0.031	0.182	0.471
Gabon	Ntem	2000-2017	-0.165	-0.018	0.065
Gabon	Ogooué et des Lacs	2000	0.097	0.269	0.476
Gabon	Ogooué et des Lacs	2017	0.024	0.120	0.269
Gabon	Ogooué et des Lacs	2000-2017	-0.119	-0.018	0.096
Gabon	Ogoulou	2000	0.045	0.205	0.464
Gabon	Ogoulou	2017	0.021	0.139	0.387
Gabon	Ogoulou	2000-2017	-0.090	0.023	0.109
Gabon	Okano	2000	0.063	0.235	0.479
Gabon	Okano	2017	0.028	0.160	0.436
Gabon	Okano	2000-2017	-0.123	-0.024	0.066
Gabon	Plateaux	2000	0.017	0.127	0.270
Gabon	Plateaux	2017	0.010	0.085	0.228
Gabon	Plateaux	2000-2017	-0.165	-0.011	0.111
Gabon	Sébé-Brikolo	2000	0.054	0.221	0.558
Gabon	Sébé-Brikolo	2017	0.018	0.159	0.447
Gabon	Sébé-Brikolo	2000-2017	-0.166	-0.030	0.067
Gabon	Tsamba Mangotsi	2000	0.102	0.253	0.473
Gabon	Tsamba Mangotsi	2017	0.025	0.146	0.362
Gabon	Tsamba Mangotsi	2000-2017	-0.103	-0.004	0.097
Gabon	Woleu	2000	0.050	0.221	0.516
Gabon	Woleu	2017	0.037	0.176	0.422
Gabon	Woleu	2000-2017	-0.140	-0.017	0.076
Gabon	Zadié	2000	0.014	0.156	0.551
Gabon	Zadié	2017	0.009	0.114	0.389
Gabon	Zadié	2000-2017	-0.098	0.019	0.118
Gambia	Banjul	2000	0.018	0.063	0.137
Gambia	Banjul	2017	0.002	0.006	0.016
Gambia	Banjul	2000-2017	-0.240	-0.147	-0.063
Gambia	Central Baddibu	2000	0.173	0.393	0.691
Gambia	Central Baddibu	2017	0.004	0.012	0.028
Gambia	Central Baddibu	2000-2017	-0.251	-0.167	-0.090
Gambia	Foni Bintang Karanai	2000	0.046	0.156	0.408
Gambia	Foni Bintang Karanai	2017	0.004	0.013	0.031
Gambia	Foni Bintang Karanai	2000-2017	-0.204	-0.132	-0.061
Gambia	Foni Bondali	2000	0.061	0.157	0.352
Gambia	Foni Bondali	2017	0.003	0.009	0.021
Gambia	Foni Bondali	2000-2017	-0.212	-0.143	-0.086
Gambia	Foni Brefet	2000	0.033	0.127	0.357
Gambia	Foni Brefet	2017	0.003	0.012	0.029
Gambia	Foni Brefet	2000-2017	-0.200	-0.133	-0.062
Gambia	Foni Jarrol	2000	0.064	0.155	0.316
Gambia	Foni Jarrol	2017	0.002	0.007	0.017
Gambia	Foni Jarrol	2000-2017	-0.229	-0.157	-0.092
Gambia	Foni Kansala	2000	0.047	0.136	0.348
Gambia	Foni Kansala	2017	0.002	0.009	0.020

Table 3: Malaria DALYs rate by unit *(continued)*

Country	Unit	year	mean rate	lower rate	upper rate
Gambia	Foni Kansala	2000-2017	-0.204	-0.135	-0.061
Gambia	Fulladu East	2000	0.064	0.213	0.487
Gambia	Fulladu East	2017	0.006	0.019	0.043
Gambia	Fulladu East	2000-2017	-0.183	-0.117	-0.059
Gambia	Fulladu West	2000	0.079	0.248	0.527
Gambia	Fulladu West	2017	0.003	0.011	0.028
Gambia	Fulladu West	2000-2017	-0.240	-0.165	-0.109
Gambia	Janjanbureh	2000	0.054	0.171	0.379
Gambia	Janjanbureh	2017	0.002	0.007	0.019
Gambia	Janjanbureh	2000-2017	-0.233	-0.163	-0.109
Gambia	Jarra Central	2000	0.143	0.262	0.447
Gambia	Jarra Central	2017	0.002	0.007	0.016
Gambia	Jarra Central	2000-2017	-0.250	-0.186	-0.131
Gambia	Jarra East	2000	0.106	0.233	0.416
Gambia	Jarra East	2017	0.002	0.006	0.014
Gambia	Jarra East	2000-2017	-0.243	-0.186	-0.131
Gambia	Jarra West	2000	0.160	0.299	0.503
Gambia	Jarra West	2017	0.003	0.008	0.020
Gambia	Jarra West	2000-2017	-0.259	-0.188	-0.126
Gambia	Jokadu	2000	0.105	0.268	0.592
Gambia	Jokadu	2017	0.003	0.012	0.029
Gambia	Jokadu	2000-2017	-0.239	-0.167	-0.086
Gambia	Kanifing	2000	0.009	0.035	0.092
Gambia	Kanifing	2017	0.001	0.004	0.010
Gambia	Kanifing	2000-2017	-0.259	-0.155	-0.068
Gambia	Kantora	2000	0.059	0.242	0.608
Gambia	Kantora	2017	0.007	0.027	0.072
Gambia	Kantora	2000-2017	-0.191	-0.103	-0.013
Gambia	Kiang Central	2000	0.120	0.227	0.389
Gambia	Kiang Central	2017	0.002	0.008	0.019
Gambia	Kiang Central	2000-2017	-0.241	-0.164	-0.093
Gambia	Kiang East	2000	0.157	0.284	0.492
Gambia	Kiang East	2017	0.002	0.008	0.019
Gambia	Kiang East	2000-2017	-0.260	-0.181	-0.118
Gambia	Kiang West	2000	0.096	0.212	0.439
Gambia	Kiang West	2017	0.003	0.010	0.023
Gambia	Kiang West	2000-2017	-0.216	-0.150	-0.090
Gambia	Kombo Central	2000	0.033	0.153	0.415
Gambia	Kombo Central	2017	0.004	0.013	0.036
Gambia	Kombo Central	2000-2017	-0.229	-0.148	-0.074
Gambia	Kombo East	2000	0.037	0.144	0.394
Gambia	Kombo East	2017	0.003	0.013	0.032
Gambia	Kombo East	2000-2017	-0.215	-0.143	-0.071
Gambia	Kombo Saint Mary	2000	0.014	0.057	0.149
Gambia	Kombo Saint Mary	2017	0.001	0.006	0.017
Gambia	Kombo Saint Mary	2000-2017	-0.255	-0.151	-0.063
Gambia	Kombo South	2000	0.034	0.163	0.441
Gambia	Kombo South	2017	0.004	0.014	0.039
Gambia	Kombo South	2000-2017	-0.238	-0.149	-0.071
Gambia	Lower Baddibu	2000	0.118	0.298	0.594
Gambia	Lower Baddibu	2017	0.003	0.011	0.025
Gambia	Lower Baddibu	2000-2017	-0.240	-0.167	-0.099
Gambia	Lower Nuimi	2000	0.072	0.188	0.408
Gambia	Lower Nuimi	2017	0.003	0.012	0.028
Gambia	Lower Nuimi	2000-2017	-0.244	-0.164	-0.082
Gambia	Lower Saloum	2000	0.268	0.515	0.839
Gambia	Lower Saloum	2017	0.004	0.014	0.038
Gambia	Lower Saloum	2000-2017	-0.271	-0.193	-0.115
Gambia	Niamina Dankunku	2000	0.192	0.358	0.603
Gambia	Niamina Dankunku	2017	0.003	0.009	0.025
Gambia	Niamina Dankunku	2000-2017	-0.264	-0.191	-0.116

Table 3: Malaria DALYs rate by unit *(continued)*

Country	Unit	year	mean rate	lower rate	upper rate
Gambia	Niamina East	2000	0.131	0.331	0.640
Gambia	Niamina East	2017	0.003	0.009	0.025
Gambia	Niamina East	2000-2017	-0.251	-0.184	-0.122
Gambia	Niamina West	2000	0.124	0.274	0.477
Gambia	Niamina West	2017	0.003	0.007	0.017
Gambia	Niamina West	2000-2017	-0.252	-0.186	-0.123
Gambia	Niani	2000	0.108	0.263	0.542
Gambia	Niani	2017	0.003	0.009	0.022
Gambia	Niani	2000-2017	-0.244	-0.179	-0.121
Gambia	Nianija	2000	0.155	0.344	0.649
Gambia	Nianija	2017	0.003	0.010	0.023
Gambia	Nianija	2000-2017	-0.241	-0.186	-0.130
Gambia	Sami	2000	0.058	0.186	0.402
Gambia	Sami	2017	0.003	0.010	0.025
Gambia	Sami	2000-2017	-0.229	-0.157	-0.100
Gambia	Sandu	2000	0.063	0.212	0.506
Gambia	Sandu	2017	0.005	0.015	0.034
Gambia	Sandu	2000-2017	-0.197	-0.131	-0.076
Gambia	Upper Baddibu	2000	0.246	0.464	0.772
Gambia	Upper Baddibu	2017	0.004	0.013	0.031
Gambia	Upper Baddibu	2000-2017	-0.255	-0.183	-0.121
Gambia	Upper Nuimi	2000	0.056	0.147	0.328
Gambia	Upper Nuimi	2017	0.003	0.010	0.024
Gambia	Upper Nuimi	2000-2017	-0.221	-0.154	-0.091
Gambia	Upper Saloum	2000	0.257	0.489	0.865
Gambia	Upper Saloum	2017	0.005	0.014	0.033
Gambia	Upper Saloum	2000-2017	-0.253	-0.192	-0.123
Gambia	Wuli	2000	0.059	0.200	0.471
Gambia	Wuli	2017	0.006	0.021	0.051
Gambia	Wuli	2000-2017	-0.168	-0.110	-0.056
Ghana	Abura-Asebu-Kwamankese	2000	0.078	0.248	0.462
Ghana	Abura-Asebu-Kwamankese	2017	0.037	0.119	0.259
Ghana	Abura-Asebu-Kwamankese	2000-2017	-0.156	-0.081	-0.017
Ghana	Accra	2000	0.030	0.101	0.204
Ghana	Accra	2017	0.007	0.025	0.054
Ghana	Accra	2000-2017	-0.115	-0.066	-0.025
Ghana	Adaklu Anyigbe	2000	0.175	0.397	0.641
Ghana	Adaklu Anyigbe	2017	0.046	0.140	0.288
Ghana	Adaklu Anyigbe	2000-2017	-0.122	-0.061	-0.017
Ghana	Adansi North	2000	0.088	0.235	0.525
Ghana	Adansi North	2017	0.034	0.119	0.283
Ghana	Adansi North	2000-2017	-0.181	-0.094	-0.030
Ghana	Adansi South	2000	0.194	0.449	0.902
Ghana	Adansi South	2017	0.088	0.251	0.551
Ghana	Adansi South	2000-2017	-0.142	-0.073	-0.029
Ghana	Afigya Sekyere	2000	0.115	0.387	0.790
Ghana	Afigya Sekyere	2017	0.039	0.118	0.263
Ghana	Afigya Sekyere	2000-2017	-0.170	-0.123	-0.057
Ghana	Afram Plains	2000	0.146	0.392	0.650
Ghana	Afram Plains	2017	0.049	0.171	0.371
Ghana	Afram Plains	2000-2017	-0.169	-0.062	0.011
Ghana	Agona	2000	0.124	0.338	0.632
Ghana	Agona	2017	0.077	0.206	0.403
Ghana	Agona	2000-2017	-0.125	-0.060	0.001
Ghana	Ahafo Ano North	2000	0.185	0.515	0.848
Ghana	Ahafo Ano North	2017	0.092	0.224	0.563
Ghana	Ahafo Ano North	2000-2017	-0.161	-0.088	-0.040
Ghana	Ahafo Ano South	2000	0.150	0.470	0.859
Ghana	Ahafo Ano South	2017	0.069	0.179	0.400
Ghana	Ahafo Ano South	2000-2017	-0.155	-0.104	-0.060
Ghana	Ahanta West	2000	0.094	0.335	0.624

Table 3: Malaria DALYs rate by unit *(continued)*

Country	Unit	year	mean rate	lower rate	upper rate
Ghana	Ahanta West	2017	0.018	0.070	0.168
Ghana	Ahanta West	2000-2017	-0.233	-0.143	-0.065
Ghana	Ajumako-Enyan-Esiam	2000	0.149	0.430	0.774
Ghana	Ajumako-Enyan-Esiam	2017	0.092	0.246	0.481
Ghana	Ajumako-Enyan-Esiam	2000-2017	-0.141	-0.062	-0.003
Ghana	Akatsi	2000	0.171	0.361	0.624
Ghana	Akatsi	2017	0.064	0.168	0.317
Ghana	Akatsi	2000-2017	-0.090	-0.037	0.015
Ghana	Akwapim North	2000	0.282	0.638	1.067
Ghana	Akwapim North	2017	0.072	0.192	0.421
Ghana	Akwapim North	2000-2017	-0.116	-0.068	-0.028
Ghana	Akwapim South	2000	0.154	0.374	0.662
Ghana	Akwapim South	2017	0.029	0.102	0.227
Ghana	Akwapim South	2000-2017	-0.122	-0.073	-0.032
Ghana	Amansie Central	2000	0.129	0.334	0.688
Ghana	Amansie Central	2017	0.044	0.149	0.345
Ghana	Amansie Central	2000-2017	-0.167	-0.097	-0.051
Ghana	Amansie East	2000	0.136	0.375	0.793
Ghana	Amansie East	2017	0.063	0.191	0.436
Ghana	Amansie East	2000-2017	-0.151	-0.091	-0.044
Ghana	Amansie West	2000	0.099	0.371	0.731
Ghana	Amansie West	2017	0.056	0.158	0.348
Ghana	Amansie West	2000-2017	-0.168	-0.097	-0.043
Ghana	Aowin-Suaman	2000	0.348	0.693	1.081
Ghana	Aowin-Suaman	2017	0.102	0.348	0.616
Ghana	Aowin-Suaman	2000-2017	-0.161	-0.062	-0.015
Ghana	Asante Akim North	2000	0.137	0.408	0.791
Ghana	Asante Akim North	2017	0.058	0.184	0.359
Ghana	Asante Akim North	2000-2017	-0.161	-0.093	-0.044
Ghana	Asante Akim South	2000	0.158	0.464	0.881
Ghana	Asante Akim South	2017	0.084	0.243	0.524
Ghana	Asante Akim South	2000-2017	-0.154	-0.081	-0.034
Ghana	Asikuma Odoben Brakwa	2000	0.148	0.399	0.748
Ghana	Asikuma Odoben Brakwa	2017	0.120	0.271	0.509
Ghana	Asikuma Odoben Brakwa	2000-2017	-0.124	-0.057	-0.007
Ghana	Assin North	2000	0.163	0.389	0.728
Ghana	Assin North	2017	0.104	0.262	0.502
Ghana	Assin North	2000-2017	-0.141	-0.064	-0.017
Ghana	Assin South	2000	0.159	0.397	0.750
Ghana	Assin South	2017	0.102	0.262	0.513
Ghana	Assin South	2000-2017	-0.134	-0.064	-0.016
Ghana	Asunafo North	2000	0.336	0.638	1.044
Ghana	Asunafo North	2017	0.088	0.281	0.635
Ghana	Asunafo North	2000-2017	-0.153	-0.079	-0.026
Ghana	Asunafo South	2000	0.289	0.610	1.015
Ghana	Asunafo South	2017	0.086	0.277	0.619
Ghana	Asunafo South	2000-2017	-0.149	-0.076	-0.026
Ghana	Asuogyaman	2000	0.202	0.481	0.814
Ghana	Asuogyaman	2017	0.041	0.135	0.319
Ghana	Asuogyaman	2000-2017	-0.122	-0.067	-0.015
Ghana	Asutifi	2000	0.253	0.551	0.903
Ghana	Asutifi	2017	0.075	0.209	0.540
Ghana	Asutifi	2000-2017	-0.159	-0.093	-0.039
Ghana	Atebubu-Amantin	2000	0.206	0.419	0.722
Ghana	Atebubu-Amantin	2017	0.041	0.156	0.355
Ghana	Atebubu-Amantin	2000-2017	-0.217	-0.105	-0.027
Ghana	Atiwa	2000	0.191	0.439	0.754
Ghana	Atiwa	2017	0.066	0.209	0.463
Ghana	Atiwa	2000-2017	-0.144	-0.076	-0.013
Ghana	Atwima	2000	0.059	0.216	0.455
Ghana	Atwima	2017	0.027	0.067	0.145

Table 3: Malaria DALYs rate by unit *(continued)*

Country	Unit	year	mean rate	lower rate	upper rate
Ghana	Atwima	2000-2017	-0.168	-0.123	-0.069
Ghana	Atwima Mponua	2000	0.157	0.498	0.930
Ghana	Atwima Mponua	2017	0.078	0.232	0.524
Ghana	Atwima Mponua	2000-2017	-0.170	-0.084	-0.038
Ghana	Awutu Efutu Senya	2000	0.045	0.161	0.305
Ghana	Awutu Efutu Senya	2017	0.019	0.063	0.130
Ghana	Awutu Efutu Senya	2000-2017	-0.135	-0.067	-0.011
Ghana	Bawku Municipal	2000	0.314	0.655	1.125
Ghana	Bawku Municipal	2017	0.069	0.166	0.313
Ghana	Bawku Municipal	2000-2017	-0.202	-0.136	-0.072
Ghana	Bawku West	2000	0.278	0.550	0.904
Ghana	Bawku West	2017	0.044	0.108	0.212
Ghana	Bawku West	2000-2017	-0.227	-0.155	-0.084
Ghana	Berekum	2000	0.272	0.540	0.898
Ghana	Berekum	2017	0.039	0.137	0.325
Ghana	Berekum	2000-2017	-0.211	-0.124	-0.061
Ghana	Bia	2000	0.362	0.693	1.106
Ghana	Bia	2017	0.104	0.326	0.611
Ghana	Bia	2000-2017	-0.169	-0.076	-0.018
Ghana	Bibiani Anhwiaso Bekwai	2000	0.230	0.544	0.907
Ghana	Bibiani Anhwiaso Bekwai	2017	0.085	0.253	0.527
Ghana	Bibiani Anhwiaso Bekwai	2000-2017	-0.145	-0.073	-0.026
Ghana	Birim North	2000	0.178	0.430	0.794
Ghana	Birim North	2017	0.079	0.239	0.511
Ghana	Birim North	2000-2017	-0.132	-0.069	-0.023
Ghana	Birim South	2000	0.126	0.349	0.647
Ghana	Birim South	2017	0.090	0.233	0.448
Ghana	Birim South	2000-2017	-0.117	-0.057	-0.008
Ghana	Bole	2000	0.145	0.266	0.445
Ghana	Bole	2017	0.037	0.112	0.206
Ghana	Bole	2000-2017	-0.184	-0.089	-0.027
Ghana	Bolgatanga	2000	0.280	0.476	0.758
Ghana	Bolgatanga	2017	0.021	0.063	0.149
Ghana	Bolgatanga	2000-2017	-0.265	-0.186	-0.129
Ghana	Bongo	2000	0.342	0.596	0.949
Ghana	Bongo	2017	0.028	0.085	0.182
Ghana	Bongo	2000-2017	-0.253	-0.177	-0.109
Ghana	Bosomtwe-Kwanwoma	2000	0.059	0.208	0.416
Ghana	Bosomtwe-Kwanwoma	2017	0.023	0.066	0.149
Ghana	Bosomtwe-Kwanwoma	2000-2017	-0.170	-0.122	-0.071
Ghana	Builsa	2000	0.307	0.521	0.776
Ghana	Builsa	2017	0.027	0.097	0.200
Ghana	Builsa	2000-2017	-0.235	-0.157	-0.091
Ghana	Bunkpurugu Yunyoo	2000	0.201	0.469	0.821
Ghana	Bunkpurugu Yunyoo	2017	0.064	0.170	0.348
Ghana	Bunkpurugu Yunyoo	2000-2017	-0.172	-0.104	-0.047
Ghana	Cape Coast	2000	0.037	0.148	0.311
Ghana	Cape Coast	2017	0.017	0.062	0.143
Ghana	Cape Coast	2000-2017	-0.165	-0.086	-0.010
Ghana	Central Gonja	2000	0.164	0.297	0.477
Ghana	Central Gonja	2017	0.021	0.078	0.162
Ghana	Central Gonja	2000-2017	-0.227	-0.135	-0.058
Ghana	Dangbe East	2000	0.155	0.356	0.624
Ghana	Dangbe East	2017	0.028	0.091	0.214
Ghana	Dangbe East	2000-2017	-0.118	-0.067	-0.018
Ghana	Dangbe West	2000	0.092	0.229	0.407
Ghana	Dangbe West	2017	0.019	0.071	0.180
Ghana	Dangbe West	2000-2017	-0.138	-0.068	-0.012
Ghana	Dormaa	2000	0.370	0.644	1.033
Ghana	Dormaa	2017	0.078	0.202	0.466
Ghana	Dormaa	2000-2017	-0.197	-0.108	-0.044

Table 3: Malaria DALYs rate by unit *(continued)*

Country	Unit	year	mean rate	lower rate	upper rate
Ghana	East Akim	2000	0.233	0.504	0.850
Ghana	East Akim	2017	0.073	0.203	0.427
Ghana	East Akim	2000-2017	-0.145	-0.074	-0.020
Ghana	East Gonja	2000	0.177	0.321	0.505
Ghana	East Gonja	2017	0.059	0.158	0.296
Ghana	East Gonja	2000-2017	-0.135	-0.066	-0.022
Ghana	East Mamprusi	2000	0.285	0.564	0.915
Ghana	East Mamprusi	2017	0.045	0.136	0.294
Ghana	East Mamprusi	2000-2017	-0.217	-0.140	-0.081
Ghana	Ejisu-Juabeng	2000	0.069	0.237	0.483
Ghana	Ejisu-Juabeng	2017	0.035	0.103	0.240
Ghana	Ejisu-Juabeng	2000-2017	-0.168	-0.110	-0.062
Ghana	Ejura Sekyedumase	2000	0.163	0.361	0.636
Ghana	Ejura Sekyedumase	2017	0.035	0.110	0.238
Ghana	Ejura Sekyedumase	2000-2017	-0.199	-0.123	-0.049
Ghana	Fanteakwa	2000	0.241	0.541	0.867
Ghana	Fanteakwa	2017	0.066	0.195	0.397
Ghana	Fanteakwa	2000-2017	-0.158	-0.079	-0.013
Ghana	Ga East	2000	0.051	0.150	0.281
Ghana	Ga East	2017	0.012	0.045	0.096
Ghana	Ga East	2000-2017	-0.108	-0.062	-0.023
Ghana	Ga West	2000	0.050	0.143	0.273
Ghana	Ga West	2017	0.014	0.050	0.106
Ghana	Ga West	2000-2017	-0.114	-0.062	-0.022
Ghana	Garu Tempane	2000	0.278	0.620	1.062
Ghana	Garu Tempane	2017	0.064	0.170	0.346
Ghana	Garu Tempane	2000-2017	-0.189	-0.128	-0.066
Ghana	Gomoa	2000	0.112	0.314	0.556
Ghana	Gomoa	2017	0.053	0.165	0.326
Ghana	Gomoa	2000-2017	-0.131	-0.061	0.000
Ghana	Gushiegu	2000	0.175	0.379	0.656
Ghana	Gushiegu	2017	0.094	0.203	0.407
Ghana	Gushiegu	2000-2017	-0.140	-0.066	-0.026
Ghana	Ho	2000	0.166	0.458	0.805
Ghana	Ho	2017	0.054	0.156	0.345
Ghana	Ho	2000-2017	-0.118	-0.060	-0.010
Ghana	Hohoe	2000	0.192	0.533	0.959
Ghana	Hohoe	2017	0.057	0.180	0.388
Ghana	Hohoe	2000-2017	-0.104	-0.054	-0.007
Ghana	Jaman North	2000	0.201	0.371	0.608
Ghana	Jaman North	2017	0.029	0.119	0.272
Ghana	Jaman North	2000-2017	-0.199	-0.101	-0.018
Ghana	Jaman South	2000	0.257	0.466	0.784
Ghana	Jaman South	2017	0.039	0.141	0.290
Ghana	Jaman South	2000-2017	-0.194	-0.102	-0.040
Ghana	Jasikan	2000	0.214	0.572	0.992
Ghana	Jasikan	2017	0.051	0.190	0.434
Ghana	Jasikan	2000-2017	-0.129	-0.059	-0.006
Ghana	Jirapa Lambussie	2000	0.177	0.404	0.700
Ghana	Jirapa Lambussie	2017	0.063	0.156	0.271
Ghana	Jirapa Lambussie	2000-2017	-0.174	-0.101	-0.039
Ghana	Jomoro	2000	0.295	0.597	0.962
Ghana	Jomoro	2017	0.033	0.146	0.339
Ghana	Jomoro	2000-2017	-0.241	-0.134	-0.049
Ghana	Juabeso	2000	0.387	0.748	1.186
Ghana	Juabeso	2017	0.122	0.401	0.750
Ghana	Juabeso	2000-2017	-0.155	-0.059	-0.013
Ghana	Kadjebi	2000	0.233	0.466	0.769
Ghana	Kadjebi	2017	0.084	0.223	0.422
Ghana	Kadjebi	2000-2017	-0.120	-0.044	0.004
Ghana	Karaga	2000	0.140	0.354	0.599

Table 3: Malaria DALYs rate by unit *(continued)*

Country	Unit	year	mean rate	lower rate	upper rate
Ghana	Karaga	2017	0.046	0.122	0.294
Ghana	Karaga	2000-2017	-0.192	-0.105	-0.045
Ghana	Kassena Nankana	2000	0.312	0.533	0.844
Ghana	Kassena Nankana	2017	0.025	0.081	0.174
Ghana	Kassena Nankana	2000-2017	-0.257	-0.169	-0.105
Ghana	Keta	2000	0.094	0.273	0.530
Ghana	Keta	2017	0.040	0.120	0.237
Ghana	Keta	2000-2017	-0.104	-0.044	0.012
Ghana	Ketu	2000	0.081	0.229	0.421
Ghana	Ketu	2017	0.037	0.108	0.201
Ghana	Ketu	2000-2017	-0.086	-0.029	0.028
Ghana	Kintampo North	2000	0.174	0.328	0.533
Ghana	Kintampo North	2017	0.022	0.075	0.158
Ghana	Kintampo North	2000-2017	-0.248	-0.151	-0.068
Ghana	Kintampo South	2000	0.177	0.363	0.601
Ghana	Kintampo South	2017	0.023	0.084	0.173
Ghana	Kintampo South	2000-2017	-0.248	-0.147	-0.065
Ghana	Komenda-Edina-Eguafo-Abirem	2000	0.088	0.303	0.600
Ghana	Komenda-Edina-Eguafo-Abirem	2017	0.031	0.112	0.271
Ghana	Komenda-Edina-Eguafo-Abirem	2000-2017	-0.177	-0.097	-0.030
Ghana	Kpandu	2000	0.197	0.526	0.910
Ghana	Kpandu	2017	0.051	0.182	0.375
Ghana	Kpandu	2000-2017	-0.111	-0.059	-0.004
Ghana	Krachi	2000	0.285	0.552	0.923
Ghana	Krachi	2017	0.090	0.277	0.539
Ghana	Krachi	2000-2017	-0.152	-0.053	0.002
Ghana	Krachi East	2000	0.265	0.504	0.851
Ghana	Krachi East	2017	0.059	0.217	0.450
Ghana	Krachi East	2000-2017	-0.141	-0.054	0.011
Ghana	Kumasi	2000	0.014	0.063	0.197
Ghana	Kumasi	2017	0.007	0.021	0.049
Ghana	Kumasi	2000-2017	-0.172	-0.120	-0.061
Ghana	Kwabibirem	2000	0.152	0.372	0.670
Ghana	Kwabibirem	2017	0.070	0.211	0.453
Ghana	Kwabibirem	2000-2017	-0.122	-0.062	-0.006
Ghana	Kwabre	2000	0.039	0.157	0.325
Ghana	Kwabre	2017	0.017	0.053	0.118
Ghana	Kwabre	2000-2017	-0.163	-0.124	-0.068
Ghana	Kwahu South	2000	0.170	0.484	0.837
Ghana	Kwahu South	2017	0.065	0.216	0.464
Ghana	Kwahu South	2000-2017	-0.181	-0.076	-0.008
Ghana	Kwahu West	2000	0.129	0.362	0.619
Ghana	Kwahu West	2017	0.052	0.185	0.402
Ghana	Kwahu West	2000-2017	-0.159	-0.076	-0.014
Ghana	Lawra	2000	0.168	0.408	0.720
Ghana	Lawra	2017	0.065	0.160	0.277
Ghana	Lawra	2000-2017	-0.164	-0.097	-0.034
Ghana	Lower Denkyira	2000	0.170	0.408	0.778
Ghana	Lower Denkyira	2017	0.090	0.236	0.441
Ghana	Lower Denkyira	2000-2017	-0.152	-0.075	-0.022
Ghana	Manya Krobo	2000	0.261	0.568	0.926
Ghana	Manya Krobo	2017	0.064	0.181	0.387
Ghana	Manya Krobo	2000-2017	-0.127	-0.071	-0.022
Ghana	Mfantsiman	2000	0.091	0.332	0.625
Ghana	Mfantsiman	2017	0.049	0.155	0.322
Ghana	Mfantsiman	2000-2017	-0.147	-0.074	-0.012
Ghana	Mpohor Wassa East	2000	0.189	0.451	0.798
Ghana	Mpohor Wassa East	2017	0.044	0.169	0.365
Ghana	Mpohor Wassa East	2000-2017	-0.174	-0.098	-0.047
Ghana	Nadowli	2000	0.125	0.266	0.447
Ghana	Nadowli	2017	0.033	0.090	0.157

Table 3: Malaria DALYs rate by unit *(continued)*

Country	Unit	year	mean rate	lower rate	upper rate
Ghana	Nadowli	2000-2017	-0.200	-0.119	-0.055
Ghana	Nanumba North	2000	0.163	0.289	0.434
Ghana	Nanumba North	2017	0.103	0.207	0.354
Ghana	Nanumba North	2000-2017	-0.087	-0.034	-0.006
Ghana	Nanumba South	2000	0.176	0.317	0.503
Ghana	Nanumba South	2017	0.123	0.237	0.383
Ghana	Nanumba South	2000-2017	-0.063	-0.026	0.000
Ghana	New Juaben	2000	0.146	0.323	0.546
Ghana	New Juaben	2017	0.032	0.095	0.193
Ghana	New Juaben	2000-2017	-0.145	-0.082	-0.031
Ghana	Nkoranza	2000	0.171	0.387	0.648
Ghana	Nkoranza	2017	0.031	0.109	0.229
Ghana	Nkoranza	2000-2017	-0.210	-0.129	-0.055
Ghana	Nkwanta	2000	0.253	0.457	0.756
Ghana	Nkwanta	2017	0.150	0.287	0.464
Ghana	Nkwanta	2000-2017	-0.096	-0.031	0.002
Ghana	North Tongu	2000	0.191	0.382	0.651
Ghana	North Tongu	2017	0.033	0.110	0.268
Ghana	North Tongu	2000-2017	-0.134	-0.063	-0.012
Ghana	Nzema East	2000	0.264	0.545	0.902
Ghana	Nzema East	2017	0.034	0.133	0.275
Ghana	Nzema East	2000-2017	-0.226	-0.134	-0.055
Ghana	Obuasi Municipal	2000	0.140	0.358	0.711
Ghana	Obuasi Municipal	2017	0.066	0.202	0.407
Ghana	Obuasi Municipal	2000-2017	-0.159	-0.083	-0.026
Ghana	Offinso	2000	0.148	0.416	0.776
Ghana	Offinso	2017	0.045	0.127	0.265
Ghana	Offinso	2000-2017	-0.183	-0.122	-0.066
Ghana	Pru	2000	0.175	0.360	0.585
Ghana	Pru	2017	0.031	0.117	0.265
Ghana	Pru	2000-2017	-0.235	-0.113	-0.036
Ghana	Saboba Chereponi	2000	0.202	0.419	0.712
Ghana	Saboba Chereponi	2017	0.135	0.286	0.520
Ghana	Saboba Chereponi	2000-2017	-0.094	-0.045	-0.017
Ghana	Savelugu Nanton	2000	0.207	0.407	0.674
Ghana	Savelugu Nanton	2017	0.028	0.082	0.169
Ghana	Savelugu Nanton	2000-2017	-0.234	-0.147	-0.083
Ghana	Sawa-Tuna-Kalba	2000	0.126	0.260	0.453
Ghana	Sawa-Tuna-Kalba	2017	0.049	0.133	0.251
Ghana	Sawa-Tuna-Kalba	2000-2017	-0.163	-0.075	-0.023
Ghana	Sefwi Wiawso	2000	0.316	0.651	0.999
Ghana	Sefwi Wiawso	2017	0.098	0.310	0.602
Ghana	Sefwi Wiawso	2000-2017	-0.152	-0.067	-0.021
Ghana	Sekyere East	2000	0.157	0.380	0.674
Ghana	Sekyere East	2017	0.053	0.155	0.298
Ghana	Sekyere East	2000-2017	-0.162	-0.092	-0.045
Ghana	Sekyere West	2000	0.155	0.388	0.703
Ghana	Sekyere West	2017	0.044	0.126	0.249
Ghana	Sekyere West	2000-2017	-0.183	-0.113	-0.050
Ghana	Sene	2000	0.197	0.396	0.664
Ghana	Sene	2017	0.050	0.177	0.363
Ghana	Sene	2000-2017	-0.179	-0.069	-0.006
Ghana	Shama Ahanta East	2000	0.047	0.209	0.435
Ghana	Shama Ahanta East	2017	0.013	0.052	0.122
Ghana	Shama Ahanta East	2000-2017	-0.205	-0.118	-0.047
Ghana	Sissala East	2000	0.179	0.340	0.544
Ghana	Sissala East	2017	0.037	0.096	0.189
Ghana	Sissala East	2000-2017	-0.206	-0.129	-0.067
Ghana	Sissala West	2000	0.202	0.371	0.604
Ghana	Sissala West	2017	0.046	0.119	0.213
Ghana	Sissala West	2000-2017	-0.185	-0.115	-0.058

Table 3: Malaria DALYs rate by unit *(continued)*

Country	Unit	year	mean rate	lower rate	upper rate
Ghana	South Dayi	2000	0.244	0.592	0.995
Ghana	South Dayi	2017	0.051	0.177	0.409
Ghana	South Dayi	2000-2017	-0.141	-0.073	-0.012
Ghana	South Tongu	2000	0.144	0.328	0.589
Ghana	South Tongu	2017	0.036	0.116	0.259
Ghana	South Tongu	2000-2017	-0.113	-0.054	-0.003
Ghana	Suhum Kraboa Coaltar	2000	0.274	0.568	0.950
Ghana	Suhum Kraboa Coaltar	2017	0.078	0.240	0.530
Ghana	Suhum Kraboa Coaltar	2000-2017	-0.126	-0.061	-0.014
Ghana	Sunyani	2000	0.175	0.462	0.753
Ghana	Sunyani	2017	0.042	0.126	0.319
Ghana	Sunyani	2000-2017	-0.199	-0.125	-0.060
Ghana	Tain	2000	0.229	0.457	0.732
Ghana	Tain	2017	0.039	0.128	0.265
Ghana	Tain	2000-2017	-0.209	-0.123	-0.049
Ghana	Talensi Nabdam	2000	0.308	0.524	0.853
Ghana	Talensi Nabdam	2017	0.028	0.076	0.167
Ghana	Talensi Nabdam	2000-2017	-0.251	-0.176	-0.117
Ghana	Tamale	2000	0.097	0.229	0.393
Ghana	Tamale	2017	0.015	0.047	0.097
Ghana	Tamale	2000-2017	-0.227	-0.154	-0.087
Ghana	Tano North	2000	0.155	0.493	0.832
Ghana	Tano North	2017	0.051	0.164	0.416
Ghana	Tano North	2000-2017	-0.191	-0.118	-0.054
Ghana	Tano South	2000	0.128	0.461	0.811
Ghana	Tano South	2017	0.060	0.162	0.375
Ghana	Tano South	2000-2017	-0.189	-0.109	-0.055
Ghana	Techiman	2000	0.128	0.379	0.612
Ghana	Techiman	2017	0.027	0.103	0.222
Ghana	Techiman	2000-2017	-0.214	-0.132	-0.062
Ghana	Tema	2000	0.042	0.128	0.244
Ghana	Tema	2017	0.009	0.033	0.074
Ghana	Tema	2000-2017	-0.108	-0.066	-0.027
Ghana	Tolon-Kumbungu	2000	0.163	0.399	0.645
Ghana	Tolon-Kumbungu	2017	0.024	0.078	0.162
Ghana	Tolon-Kumbungu	2000-2017	-0.233	-0.157	-0.085
Ghana	Upper Denkyira	2000	0.138	0.388	0.676
Ghana	Upper Denkyira	2017	0.073	0.208	0.413
Ghana	Upper Denkyira	2000-2017	-0.149	-0.081	-0.029
Ghana	Wa	2000	0.098	0.222	0.376
Ghana	Wa	2017	0.019	0.066	0.148
Ghana	Wa	2000-2017	-0.235	-0.137	-0.071
Ghana	Wa East	2000	0.120	0.243	0.407
Ghana	Wa East	2017	0.015	0.067	0.162
Ghana	Wa East	2000-2017	-0.250	-0.141	-0.053
Ghana	Wa West	2000	0.134	0.259	0.448
Ghana	Wa West	2017	0.038	0.106	0.220
Ghana	Wa West	2000-2017	-0.188	-0.105	-0.045
Ghana	Wasa Amenfi East	2000	0.255	0.558	0.959
Ghana	Wasa Amenfi East	2017	0.073	0.254	0.518
Ghana	Wasa Amenfi East	2000-2017	-0.164	-0.079	-0.025
Ghana	Wasa Amenfi West	2000	0.284	0.587	0.940
Ghana	Wasa Amenfi West	2017	0.067	0.268	0.518
Ghana	Wasa Amenfi West	2000-2017	-0.169	-0.075	-0.024
Ghana	Wassa West	2000	0.189	0.407	0.686
Ghana	Wassa West	2017	0.032	0.135	0.255
Ghana	Wassa West	2000-2017	-0.188	-0.102	-0.032
Ghana	West Akim	2000	0.186	0.444	0.792
Ghana	West Akim	2017	0.082	0.241	0.510
Ghana	West Akim	2000-2017	-0.113	-0.054	0.001
Ghana	West Gonja	2000	0.129	0.259	0.430

Table 3: Malaria DALYs rate by unit *(continued)*

Country	Unit	year	mean rate	lower rate	upper rate
Ghana	West Gonja	2017	0.022	0.083	0.200
Ghana	West Gonja	2000-2017	-0.212	-0.118	-0.050
Ghana	West Mamprusi	2000	0.249	0.444	0.720
Ghana	West Mamprusi	2017	0.024	0.076	0.164
Ghana	West Mamprusi	2000-2017	-0.261	-0.163	-0.091
Ghana	Yendi	2000	0.162	0.289	0.458
Ghana	Yendi	2017	0.088	0.178	0.335
Ghana	Yendi	2000-2017	-0.114	-0.055	-0.021
Ghana	Yilo Krobo	2000	0.269	0.616	1.022
Ghana	Yilo Krobo	2017	0.069	0.191	0.410
Ghana	Yilo Krobo	2000-2017	-0.126	-0.070	-0.025
Ghana	Zabzugu Tatale	2000	0.145	0.294	0.460
Ghana	Zabzugu Tatale	2017	0.111	0.225	0.360
Ghana	Zabzugu Tatale	2000-2017	-0.086	-0.031	-0.004
Guinea	Beyla	2000	0.203	0.561	1.019
Guinea	Beyla	2017	0.105	0.351	0.691
Guinea	Beyla	2000-2017	-0.142	-0.055	-0.005
Guinea	Boffa	2000	0.135	0.563	1.057
Guinea	Boffa	2017	0.070	0.285	0.774
Guinea	Boffa	2000-2017	-0.150	-0.049	0.075
Guinea	Boké	2000	0.153	0.528	1.011
Guinea	Boké	2017	0.059	0.238	0.582
Guinea	Boké	2000-2017	-0.164	-0.053	0.058
Guinea	Conakry	2000	0.027	0.160	0.398
Guinea	Conakry	2017	0.041	0.149	0.386
Guinea	Conakry	2000-2017	-0.134	-0.014	0.125
Guinea	Coyah	2000	0.064	0.311	0.696
Guinea	Coyah	2017	0.096	0.316	0.735
Guinea	Coyah	2000-2017	-0.142	-0.020	0.102
Guinea	Dabola	2000	0.114	0.334	0.656
Guinea	Dabola	2017	0.090	0.307	0.610
Guinea	Dabola	2000-2017	-0.132	-0.030	0.042
Guinea	Dalaba	2000	0.111	0.393	0.681
Guinea	Dalaba	2017	0.056	0.242	0.524
Guinea	Dalaba	2000-2017	-0.153	-0.058	0.035
Guinea	Dinguiraye	2000	0.084	0.317	0.642
Guinea	Dinguiraye	2017	0.054	0.233	0.449
Guinea	Dinguiraye	2000-2017	-0.166	-0.043	0.036
Guinea	Dubréka	2000	0.092	0.428	0.840
Guinea	Dubréka	2017	0.103	0.324	0.763
Guinea	Dubréka	2000-2017	-0.141	-0.038	0.092
Guinea	Faranah	2000	0.165	0.428	0.820
Guinea	Faranah	2017	0.220	0.457	0.725
Guinea	Faranah	2000-2017	-0.067	-0.011	0.030
Guinea	Forécariah	2000	0.158	0.564	1.124
Guinea	Forécariah	2017	0.317	0.618	1.023
Guinea	Forécariah	2000-2017	-0.054	-0.011	0.047
Guinea	Fria	2000	0.072	0.290	0.630
Guinea	Fria	2017	0.055	0.222	0.582
Guinea	Fria	2000-2017	-0.169	-0.044	0.079
Guinea	Gaoual	2000	0.176	0.480	0.846
Guinea	Gaoual	2017	0.037	0.164	0.446
Guinea	Gaoual	2000-2017	-0.209	-0.108	-0.008
Guinea	Guéckédou	2000	0.185	0.597	1.149
Guinea	Guéckédou	2017	0.253	0.517	0.869
Guinea	Guéckédou	2000-2017	-0.081	-0.036	-0.003
Guinea	Kankan	2000	0.096	0.346	0.700
Guinea	Kankan	2017	0.090	0.311	0.563
Guinea	Kankan	2000-2017	-0.131	-0.042	0.030
Guinea	Kérouané	2000	0.148	0.463	0.946
Guinea	Kérouané	2017	0.087	0.273	0.542

Table 3: Malaria DALYs rate by unit *(continued)*

Country	Unit	year	mean rate	lower rate	upper rate
Guinea	Kérouané	2000-2017	-0.159	-0.056	0.021
Guinea	Kindia	2000	0.090	0.342	0.686
Guinea	Kindia	2017	0.172	0.412	0.728
Guinea	Kindia	2000-2017	-0.072	-0.013	0.058
Guinea	Kissidougou	2000	0.120	0.452	0.878
Guinea	Kissidougou	2017	0.235	0.453	0.711
Guinea	Kissidougou	2000-2017	-0.069	-0.022	0.015
Guinea	Koubia	2000	0.121	0.449	0.883
Guinea	Koubia	2017	0.035	0.176	0.440
Guinea	Koubia	2000-2017	-0.225	-0.102	0.010
Guinea	Koundara	2000	0.158	0.461	0.883
Guinea	Koundara	2017	0.014	0.053	0.151
Guinea	Koundara	2000-2017	-0.294	-0.188	-0.095
Guinea	Kouroussa	2000	0.135	0.371	0.713
Guinea	Kouroussa	2017	0.129	0.334	0.620
Guinea	Kouroussa	2000-2017	-0.106	-0.027	0.042
Guinea	Labé	2000	0.075	0.313	0.604
Guinea	Labé	2017	0.025	0.156	0.418
Guinea	Labé	2000-2017	-0.239	-0.102	0.040
Guinea	Lélouma	2000	0.165	0.463	0.820
Guinea	Lélouma	2017	0.034	0.179	0.484
Guinea	Lélouma	2000-2017	-0.218	-0.111	0.002
Guinea	Lola	2000	0.335	0.706	1.239
Guinea	Lola	2017	0.202	0.522	0.921
Guinea	Lola	2000-2017	-0.135	-0.047	-0.011
Guinea	Macenta	2000	0.213	0.620	1.215
Guinea	Macenta	2017	0.187	0.480	0.881
Guinea	Macenta	2000-2017	-0.129	-0.050	0.007
Guinea	Mali	2000	0.225	0.563	1.022
Guinea	Mali	2017	0.030	0.124	0.312
Guinea	Mali	2000-2017	-0.235	-0.142	-0.049
Guinea	Mamou	2000	0.149	0.371	0.713
Guinea	Mamou	2017	0.121	0.313	0.616
Guinea	Mamou	2000-2017	-0.109	-0.029	0.023
Guinea	Mandiana	2000	0.144	0.404	0.779
Guinea	Mandiana	2017	0.074	0.288	0.611
Guinea	Mandiana	2000-2017	-0.162	-0.055	0.020
Guinea	Nzérékoré	2000	0.240	0.563	1.136
Guinea	Nzérékoré	2017	0.111	0.428	0.831
Guinea	Nzérékoré	2000-2017	-0.181	-0.062	-0.005
Guinea	Pita	2000	0.117	0.335	0.591
Guinea	Pita	2017	0.043	0.225	0.526
Guinea	Pita	2000-2017	-0.168	-0.068	0.038
Guinea	Siguiri	2000	0.106	0.344	0.704
Guinea	Siguiri	2017	0.068	0.252	0.512
Guinea	Siguiri	2000-2017	-0.168	-0.056	0.023
Guinea	Télimélé	2000	0.144	0.400	0.803
Guinea	Télimélé	2017	0.080	0.274	0.697
Guinea	Télimélé	2000-2017	-0.152	-0.058	0.028
Guinea	Tougué	2000	0.069	0.337	0.590
Guinea	Tougué	2017	0.042	0.182	0.384
Guinea	Tougué	2000-2017	-0.188	-0.075	0.009
Guinea	Yamou	2000	0.372	0.957	1.834
Guinea	Yamou	2017	0.197	0.682	1.290
Guinea	Yamou	2000-2017	-0.171	-0.061	-0.012
Guinea-Bissau	Bafata	2000	0.033	0.269	0.592
Guinea-Bissau	Bafata	2017	0.004	0.025	0.096
Guinea-Bissau	Bafata	2000-2017	-0.245	-0.129	0.004
Guinea-Bissau	Bambadinca	2000	0.051	0.342	0.711
Guinea-Bissau	Bambadinca	2017	0.004	0.027	0.101
Guinea-Bissau	Bambadinca	2000-2017	-0.244	-0.120	0.039

Table 3: Malaria DALYs rate by unit *(continued)*

Country	Unit	year	mean rate	lower rate	upper rate
Guinea-Bissau	Bedanda	2000	0.086	0.406	1.007
Guinea-Bissau	Bedanda	2017	0.007	0.064	0.218
Guinea-Bissau	Bedanda	2000-2017	-0.200	-0.055	0.072
Guinea-Bissau	Bigene	2000	0.070	0.291	0.774
Guinea-Bissau	Bigene	2017	0.003	0.012	0.029
Guinea-Bissau	Bigene	2000-2017	-0.252	-0.143	-0.068
Guinea-Bissau	Bissau	2000	0.046	0.157	0.327
Guinea-Bissau	Bissau	2017	0.003	0.012	0.041
Guinea-Bissau	Bissau	2000-2017	-0.222	-0.113	0.028
Guinea-Bissau	Bissora	2000	0.073	0.276	0.636
Guinea-Bissau	Bissora	2017	0.002	0.012	0.033
Guinea-Bissau	Bissora	2000-2017	-0.267	-0.151	-0.056
Guinea-Bissau	Boe	2000	0.045	0.260	0.661
Guinea-Bissau	Boe	2017	0.004	0.035	0.125
Guinea-Bissau	Boe	2000-2017	-0.259	-0.135	-0.005
Guinea-Bissau	Bolama	2000	0.057	0.266	0.549
Guinea-Bissau	Bolama	2017	0.009	0.060	0.211
Guinea-Bissau	Bolama	2000-2017	-0.209	-0.061	0.069
Guinea-Bissau	Buba	2000	0.084	0.357	0.764
Guinea-Bissau	Buba	2017	0.005	0.044	0.156
Guinea-Bissau	Buba	2000-2017	-0.192	-0.076	0.090
Guinea-Bissau	Bubaque	2000	0.067	0.334	0.882
Guinea-Bissau	Bubaque	2017	0.000	0.000	0.000
Guinea-Bissau	Bubaque	2000-2017	NA	NA	NA
Guinea-Bissau	Bula	2000	0.070	0.275	0.697
Guinea-Bissau	Bula	2017	0.003	0.014	0.039
Guinea-Bissau	Bula	2000-2017	-0.241	-0.127	-0.033
Guinea-Bissau	Cacheu	2000	0.074	0.338	0.897
Guinea-Bissau	Cacheu	2017	0.005	0.024	0.072
Guinea-Bissau	Cacheu	2000-2017	-0.231	-0.107	-0.017
Guinea-Bissau	Cacine	2000	0.094	0.418	1.089
Guinea-Bissau	Cacine	2017	0.011	0.087	0.302
Guinea-Bissau	Cacine	2000-2017	-0.197	-0.048	0.080
Guinea-Bissau	Caio	2000	0.079	0.348	0.989
Guinea-Bissau	Caio	2017	0.005	0.038	0.132
Guinea-Bissau	Caio	2000-2017	-0.254	-0.093	0.026
Guinea-Bissau	Canghungo	2000	0.068	0.280	0.770
Guinea-Bissau	Canghungo	2017	0.004	0.021	0.071
Guinea-Bissau	Canghungo	2000-2017	-0.240	-0.107	-0.005
Guinea-Bissau	Caravela	2000	0.046	0.191	0.467
Guinea-Bissau	Caravela	2017	0.000	0.000	0.000
Guinea-Bissau	Caravela	2000-2017	NA	NA	NA
Guinea-Bissau	Catio	2000	0.079	0.415	1.015
Guinea-Bissau	Catio	2017	0.006	0.057	0.190
Guinea-Bissau	Catio	2000-2017	-0.215	-0.060	0.078
Guinea-Bissau	Contuboel	2000	0.056	0.303	0.686
Guinea-Bissau	Contuboel	2017	0.004	0.020	0.064
Guinea-Bissau	Contuboel	2000-2017	-0.243	-0.161	-0.052
Guinea-Bissau	Empada	2000	0.092	0.358	0.767
Guinea-Bissau	Empada	2017	0.006	0.048	0.160
Guinea-Bissau	Empada	2000-2017	-0.199	-0.070	0.056
Guinea-Bissau	Farim	2000	0.074	0.299	0.656
Guinea-Bissau	Farim	2017	0.003	0.014	0.037
Guinea-Bissau	Farim	2000-2017	-0.233	-0.157	-0.068
Guinea-Bissau	Fulacunda	2000	0.097	0.338	0.697
Guinea-Bissau	Fulacunda	2017	0.003	0.029	0.090
Guinea-Bissau	Fulacunda	2000-2017	-0.214	-0.104	0.026
Guinea-Bissau	Gabu	2000	0.044	0.274	0.649
Guinea-Bissau	Gabu	2017	0.003	0.029	0.094
Guinea-Bissau	Gabu	2000-2017	-0.274	-0.151	-0.041
Guinea-Bissau	Galomaro	2000	0.034	0.309	0.692

Table 3: Malaria DALYs rate by unit *(continued)*

Country	Unit	year	mean rate	lower rate	upper rate
Guinea-Bissau	Galomaro	2017	0.004	0.031	0.129
Guinea-Bissau	Galomaro	2000-2017	-0.248	-0.115	0.023
Guinea-Bissau	Gamamundo	2000	0.049	0.346	0.718
Guinea-Bissau	Gamamundo	2017	0.005	0.024	0.086
Guinea-Bissau	Gamamundo	2000-2017	-0.250	-0.137	-0.007
Guinea-Bissau	Mansaba	2000	0.066	0.326	0.698
Guinea-Bissau	Mansaba	2017	0.002	0.015	0.047
Guinea-Bissau	Mansaba	2000-2017	-0.285	-0.157	-0.034
Guinea-Bissau	Mansoa	2000	0.084	0.368	0.738
Guinea-Bissau	Mansoa	2017	0.003	0.020	0.069
Guinea-Bissau	Mansoa	2000-2017	-0.274	-0.143	-0.011
Guinea-Bissau	Nhacra	2000	0.114	0.339	0.701
Guinea-Bissau	Nhacra	2017	0.003	0.018	0.058
Guinea-Bissau	Nhacra	2000-2017	-0.243	-0.134	-0.007
Guinea-Bissau	Piche	2000	0.039	0.280	0.669
Guinea-Bissau	Piche	2017	0.003	0.020	0.060
Guinea-Bissau	Piche	2000-2017	-0.310	-0.196	-0.088
Guinea-Bissau	Pirada	2000	0.042	0.247	0.607
Guinea-Bissau	Pirada	2017	0.004	0.021	0.064
Guinea-Bissau	Pirada	2000-2017	-0.287	-0.186	-0.086
Guinea-Bissau	Prabis	2000	0.105	0.311	0.604
Guinea-Bissau	Prabis	2017	0.006	0.032	0.110
Guinea-Bissau	Prabis	2000-2017	-0.214	-0.102	0.015
Guinea-Bissau	Quebo	2000	0.060	0.312	0.669
Guinea-Bissau	Quebo	2017	0.006	0.051	0.173
Guinea-Bissau	Quebo	2000-2017	-0.202	-0.065	0.111
Guinea-Bissau	Quinhamel	2000	0.101	0.330	0.706
Guinea-Bissau	Quinhamel	2017	0.005	0.031	0.109
Guinea-Bissau	Quinhamel	2000-2017	-0.241	-0.106	0.012
Guinea-Bissau	Safim	2000	0.066	0.204	0.421
Guinea-Bissau	Safim	2017	0.004	0.016	0.054
Guinea-Bissau	Safim	2000-2017	-0.217	-0.117	0.011
Guinea-Bissau	Sao Domingos	2000	0.084	0.373	0.898
Guinea-Bissau	Sao Domingos	2017	0.009	0.032	0.091
Guinea-Bissau	Sao Domingos	2000-2017	-0.200	-0.100	-0.025
Guinea-Bissau	Sonaco	2000	0.041	0.268	0.638
Guinea-Bissau	Sonaco	2017	0.004	0.024	0.080
Guinea-Bissau	Sonaco	2000-2017	-0.252	-0.157	-0.048
Guinea-Bissau	Tite	2000	0.102	0.368	0.717
Guinea-Bissau	Tite	2017	0.004	0.033	0.106
Guinea-Bissau	Tite	2000-2017	-0.230	-0.102	0.021
Guinea-Bissau	Xitole	2000	0.063	0.311	0.690
Guinea-Bissau	Xitole	2017	0.006	0.039	0.138
Guinea-Bissau	Xitole	2000-2017	-0.224	-0.089	0.087
Kenya	Ainabkoi	2000	0.003	0.012	0.030
Kenya	Ainabkoi	2017	0.000	0.001	0.004
Kenya	Ainabkoi	2000-2017	-0.146	-0.050	0.023
Kenya	Ainamoi	2000	0.019	0.050	0.098
Kenya	Ainamoi	2017	0.001	0.004	0.010
Kenya	Ainamoi	2000-2017	-0.263	-0.175	-0.100
Kenya	Aldai	2000	0.050	0.125	0.257
Kenya	Aldai	2017	0.004	0.013	0.028
Kenya	Aldai	2000-2017	-0.229	-0.152	-0.089
Kenya	Alego Usonga	2000	0.323	0.618	1.121
Kenya	Alego Usonga	2017	0.047	0.144	0.293
Kenya	Alego Usonga	2000-2017	-0.181	-0.098	-0.037
Kenya	Awendo	2000	0.192	0.578	1.545
Kenya	Awendo	2017	0.017	0.062	0.159
Kenya	Awendo	2000-2017	-0.213	-0.141	-0.077
Kenya	Bahati	2000	0.007	0.033	0.086
Kenya	Bahati	2017	0.001	0.004	0.010

Table 3: Malaria DALYs rate by unit *(continued)*

Country	Unit	year	mean rate	lower rate	upper rate
Kenya	Bahati	2000-2017	-0.197	-0.093	0.007
Kenya	Balambala	2000	0.007	0.043	0.124
Kenya	Balambala	2017	0.000	0.002	0.006
Kenya	Balambala	2000-2017	-0.302	-0.169	-0.042
Kenya	Banissa	2000	0.003	0.032	0.112
Kenya	Banissa	2017	0.001	0.006	0.020
Kenya	Banissa	2000-2017	-0.210	-0.059	0.092
Kenya	Baringo Central	2000	0.027	0.116	0.260
Kenya	Baringo Central	2017	0.001	0.004	0.011
Kenya	Baringo Central	2000-2017	-0.204	-0.120	-0.029
Kenya	Baringo North	2000	0.028	0.114	0.216
Kenya	Baringo North	2017	0.001	0.005	0.016
Kenya	Baringo North	2000-2017	-0.194	-0.105	-0.007
Kenya	Baringo South	2000	0.026	0.102	0.249
Kenya	Baringo South	2017	0.001	0.004	0.010
Kenya	Baringo South	2000-2017	-0.205	-0.112	-0.021
Kenya	Belgut	2000	0.030	0.071	0.126
Kenya	Belgut	2017	0.002	0.007	0.017
Kenya	Belgut	2000-2017	-0.251	-0.165	-0.095
Kenya	Bobasi	2000	0.019	0.063	0.128
Kenya	Bobasi	2017	0.001	0.003	0.007
Kenya	Bobasi	2000-2017	-0.231	-0.156	-0.082
Kenya	Bomachoge Borabu	2000	0.014	0.049	0.099
Kenya	Bomachoge Borabu	2017	0.001	0.003	0.007
Kenya	Bomachoge Borabu	2000-2017	-0.247	-0.152	-0.069
Kenya	Bomachoge Chache	2000	0.026	0.080	0.165
Kenya	Bomachoge Chache	2017	0.001	0.006	0.013
Kenya	Bomachoge Chache	2000-2017	-0.220	-0.146	-0.076
Kenya	Bomet Central	2000	0.008	0.031	0.066
Kenya	Bomet Central	2017	0.000	0.001	0.003
Kenya	Bomet Central	2000-2017	-0.286	-0.200	-0.116
Kenya	Bomet East	2000	0.004	0.016	0.036
Kenya	Bomet East	2017	0.000	0.001	0.002
Kenya	Bomet East	2000-2017	-0.276	-0.181	-0.084
Kenya	Bonchari	2000	0.046	0.121	0.269
Kenya	Bonchari	2017	0.003	0.012	0.026
Kenya	Bonchari	2000-2017	-0.208	-0.132	-0.058
Kenya	Bondo	2000	0.193	0.405	0.858
Kenya	Bondo	2017	0.043	0.116	0.253
Kenya	Bondo	2000-2017	-0.142	-0.077	-0.030
Kenya	Borabu	2000	0.043	0.123	0.238
Kenya	Borabu	2017	0.001	0.003	0.007
Kenya	Borabu	2000-2017	-0.286	-0.207	-0.131
Kenya	Budalangi	2000	0.205	0.437	0.811
Kenya	Budalangi	2017	0.054	0.137	0.268
Kenya	Budalangi	2000-2017	-0.085	-0.036	-0.007
Kenya	Bumula	2000	0.179	0.345	0.608
Kenya	Bumula	2017	0.020	0.073	0.160
Kenya	Bumula	2000-2017	-0.126	-0.053	-0.009
Kenya	Bura	2000	0.011	0.055	0.142
Kenya	Bura	2017	0.001	0.008	0.021
Kenya	Bura	2000-2017	-0.198	-0.088	0.023
Kenya	Bureti	2000	0.014	0.038	0.068
Kenya	Bureti	2017	0.001	0.004	0.009
Kenya	Bureti	2000-2017	-0.232	-0.144	-0.060
Kenya	Butere	2000	0.268	0.507	0.961
Kenya	Butere	2017	0.013	0.044	0.105
Kenya	Butere	2000-2017	-0.181	-0.119	-0.064
Kenya	Butula	2000	0.337	0.616	1.010
Kenya	Butula	2017	0.036	0.116	0.259
Kenya	Butula	2000-2017	-0.151	-0.075	-0.023

Table 3: Malaria DALYs rate by unit *(continued)*

Country	Unit	year	mean rate	lower rate	upper rate
Kenya	Buuri	2000	0.004	0.015	0.037
Kenya	Buuri	2017	0.001	0.006	0.016
Kenya	Buuri	2000-2017	-0.148	-0.021	0.069
Kenya	Central Imenti	2000	0.007	0.029	0.068
Kenya	Central Imenti	2017	0.002	0.011	0.025
Kenya	Central Imenti	2000-2017	-0.137	-0.035	0.058
Kenya	Changamwe	2000	0.014	0.049	0.108
Kenya	Changamwe	2017	0.003	0.009	0.022
Kenya	Changamwe	2000-2017	-0.114	-0.046	0.024
Kenya	Chepalungu	2000	0.005	0.020	0.043
Kenya	Chepalungu	2017	0.000	0.001	0.002
Kenya	Chepalungu	2000-2017	-0.282	-0.186	-0.101
Kenya	Cherangany	2000	0.018	0.068	0.133
Kenya	Cherangany	2017	0.002	0.008	0.021
Kenya	Cherangany	2000-2017	-0.145	-0.061	0.015
Kenya	Chesumei	2000	0.015	0.053	0.115
Kenya	Chesumei	2017	0.001	0.006	0.016
Kenya	Chesumei	2000-2017	-0.196	-0.109	-0.043
Kenya	Chuka/Igambang'Ombe	2000	0.035	0.108	0.243
Kenya	Chuka/Igambang'Ombe	2017	0.003	0.016	0.036
Kenya	Chuka/Igambang'Ombe	2000-2017	-0.166	-0.079	0.009
Kenya	Daadab	2000	0.008	0.045	0.121
Kenya	Daadab	2017	0.000	0.002	0.006
Kenya	Daadab	2000-2017	-0.280	-0.144	-0.015
Kenya	Dagoretti North	2000	0.000	0.000	0.000
Kenya	Dagoretti North	2017	0.000	0.000	0.000
Kenya	Dagoretti North	2000-2017	NA	NA	NA
Kenya	Dagoretti South	2000	0.000	0.000	0.000
Kenya	Dagoretti South	2017	0.000	0.000	0.000
Kenya	Dagoretti South	2000-2017	NA	NA	NA
Kenya	Eldama Ravine	2000	0.016	0.055	0.130
Kenya	Eldama Ravine	2017	0.000	0.002	0.004
Kenya	Eldama Ravine	2000-2017	-0.243	-0.156	-0.077
Kenya	Eldas	2000	0.003	0.035	0.152
Kenya	Eldas	2017	0.000	0.003	0.008
Kenya	Eldas	2000-2017	-0.238	-0.096	0.013
Kenya	Embakasi Central	2000	0.000	0.000	0.000
Kenya	Embakasi Central	2017	0.000	0.000	0.000
Kenya	Embakasi Central	2000-2017	NA	NA	NA
Kenya	Embakasi East	2000	0.000	0.000	0.001
Kenya	Embakasi East	2017	0.000	0.000	0.000
Kenya	Embakasi East	2000-2017	-0.178	-0.070	0.018
Kenya	Embakasi North	2000	0.000	0.000	0.000
Kenya	Embakasi North	2017	0.000	0.000	0.000
Kenya	Embakasi North	2000-2017	NA	NA	NA
Kenya	Embakasi South	2000	0.000	0.000	0.000
Kenya	Embakasi South	2017	0.000	0.000	0.000
Kenya	Embakasi South	2000-2017	-0.211	-0.087	0.021
Kenya	Embakasi West	2000	0.000	0.000	0.000
Kenya	Embakasi West	2017	0.000	0.000	0.000
Kenya	Embakasi West	2000-2017	NA	NA	NA
Kenya	Emgwen	2000	0.018	0.065	0.139
Kenya	Emgwen	2017	0.001	0.006	0.014
Kenya	Emgwen	2000-2017	-0.234	-0.130	-0.060
Kenya	Emuhaya	2000	0.236	0.454	0.831
Kenya	Emuhaya	2017	0.008	0.027	0.062
Kenya	Emuhaya	2000-2017	-0.212	-0.144	-0.079
Kenya	Emurua Dikirr	2000	0.008	0.031	0.076
Kenya	Emurua Dikirr	2017	0.000	0.001	0.002
Kenya	Emurua Dikirr	2000-2017	-0.340	-0.221	-0.129
Kenya	Endebess	2000	0.016	0.073	0.136

Table 3: Malaria DALYs rate by unit *(continued)*

Country	Unit	year	mean rate	lower rate	upper rate
Kenya	Endebess	2017	0.003	0.012	0.033
Kenya	Endebess	2000-2017	-0.129	-0.053	0.021
Kenya	Fafi	2000	0.013	0.055	0.129
Kenya	Fafi	2017	0.001	0.003	0.009
Kenya	Fafi	2000-2017	-0.232	-0.133	-0.018
Kenya	Funyula	2000	0.335	0.615	1.014
Kenya	Funyula	2017	0.070	0.187	0.359
Kenya	Funyula	2000-2017	-0.112	-0.059	-0.018
Kenya	Galole	2000	0.018	0.104	0.278
Kenya	Galole	2017	0.003	0.015	0.043
Kenya	Galole	2000-2017	-0.195	-0.091	0.020
Kenya	Ganze	2000	0.222	0.375	0.638
Kenya	Ganze	2017	0.009	0.033	0.064
Kenya	Ganze	2000-2017	-0.134	-0.071	-0.001
Kenya	Garissa Township	2000	0.004	0.042	0.147
Kenya	Garissa Township	2017	0.000	0.002	0.005
Kenya	Garissa Township	2000-2017	-0.284	-0.136	0.004
Kenya	Garsen	2000	0.071	0.285	0.522
Kenya	Garsen	2017	0.004	0.020	0.050
Kenya	Garsen	2000-2017	-0.140	-0.067	0.026
Kenya	Gatanga	2000	0.005	0.025	0.072
Kenya	Gatanga	2017	0.002	0.009	0.022
Kenya	Gatanga	2000-2017	-0.142	-0.044	0.031
Kenya	Gatundu North	2000	0.005	0.027	0.078
Kenya	Gatundu North	2017	0.002	0.012	0.033
Kenya	Gatundu North	2000-2017	-0.150	-0.036	0.052
Kenya	Gatundu South	2000	0.008	0.035	0.099
Kenya	Gatundu South	2017	0.003	0.016	0.044
Kenya	Gatundu South	2000-2017	-0.139	-0.032	0.050
Kenya	Gem	2000	0.364	0.670	1.223
Kenya	Gem	2017	0.033	0.110	0.243
Kenya	Gem	2000-2017	-0.199	-0.116	-0.048
Kenya	Gichugu	2000	0.016	0.053	0.112
Kenya	Gichugu	2017	0.003	0.016	0.043
Kenya	Gichugu	2000-2017	-0.119	-0.043	0.044
Kenya	Gilgil	2000	0.004	0.020	0.054
Kenya	Gilgil	2017	0.001	0.003	0.009
Kenya	Gilgil	2000-2017	-0.195	-0.086	0.015
Kenya	Githunguri	2000	0.007	0.026	0.069
Kenya	Githunguri	2017	0.001	0.007	0.017
Kenya	Githunguri	2000-2017	-0.179	-0.073	0.006
Kenya	Hamisi	2000	0.126	0.277	0.576
Kenya	Hamisi	2017	0.003	0.012	0.026
Kenya	Hamisi	2000-2017	-0.227	-0.165	-0.101
Kenya	Homa Bay Town	2000	0.126	0.309	0.707
Kenya	Homa Bay Town	2017	0.012	0.038	0.088
Kenya	Homa Bay Town	2000-2017	-0.206	-0.131	-0.075
Kenya	Igembe Central	2000	0.011	0.055	0.134
Kenya	Igembe Central	2017	0.005	0.028	0.097
Kenya	Igembe Central	2000-2017	-0.133	-0.018	0.090
Kenya	Igembe North	2000	0.006	0.033	0.090
Kenya	Igembe North	2017	0.003	0.013	0.031
Kenya	Igembe North	2000-2017	-0.134	-0.022	0.088
Kenya	Igembe South	2000	0.036	0.166	0.443
Kenya	Igembe South	2000	0.011	0.049	0.128
Kenya	Igembe South	2017	0.002	0.017	0.047
Kenya	Igembe South	2017	0.004	0.023	0.072
Kenya	Igembe South	2000-2017	-0.208	-0.104	0.015
Kenya	Igembe South	2000-2017	-0.136	-0.025	0.086
Kenya	Ijara	2000	0.016	0.093	0.236
Kenya	Ijara	2017	0.001	0.005	0.012

Table 3: Malaria DALYs rate by unit *(continued)*

Country	Unit	year	mean rate	lower rate	upper rate
Kenya	Ijara	2000-2017	-0.243	-0.150	-0.048
Kenya	Ikolomani	2000	0.131	0.281	0.535
Kenya	Ikolomani	2017	0.006	0.022	0.053
Kenya	Ikolomani	2000-2017	-0.201	-0.131	-0.072
Kenya	Isiolo North	2000	0.024	0.118	0.309
Kenya	Isiolo North	2017	0.002	0.011	0.027
Kenya	Isiolo North	2000-2017	-0.182	-0.084	0.008
Kenya	Isiolo South	2000	0.021	0.131	0.378
Kenya	Isiolo South	2017	0.002	0.008	0.019
Kenya	Isiolo South	2000-2017	-0.221	-0.126	-0.030
Kenya	Jomvu	2000	0.019	0.061	0.130
Kenya	Jomvu	2017	0.003	0.010	0.024
Kenya	Jomvu	2000-2017	-0.138	-0.068	0.002
Kenya	Juja	2000	0.005	0.023	0.069
Kenya	Juja	2017	0.001	0.007	0.018
Kenya	Juja	2000-2017	-0.168	-0.057	0.024
Kenya	Kabete	2000	0.000	0.000	0.000
Kenya	Kabete	2017	0.000	0.000	0.000
Kenya	Kabete	2000-2017	NA	NA	NA
Kenya	Kabondo Kasipul	2000	0.236	0.515	1.065
Kenya	Kabondo Kasipul	2017	0.006	0.028	0.057
Kenya	Kabondo Kasipul	2000-2017	-0.255	-0.175	-0.104
Kenya	Kabuchai	2000	0.089	0.215	0.409
Kenya	Kabuchai	2017	0.009	0.039	0.091
Kenya	Kabuchai	2000-2017	-0.141	-0.065	-0.011
Kenya	Kacheliba	2000	0.023	0.108	0.239
Kenya	Kacheliba	2017	0.012	0.033	0.062
Kenya	Kacheliba	2000-2017	-0.045	0.011	0.089
Kenya	Kaiti	2000	0.008	0.040	0.088
Kenya	Kaiti	2017	0.000	0.001	0.002
Kenya	Kaiti	2000-2017	-0.263	-0.168	-0.064
Kenya	Kajiado Central	2000	0.013	0.071	0.155
Kenya	Kajiado Central	2017	0.001	0.006	0.015
Kenya	Kajiado Central	2000-2017	-0.252	-0.122	-0.013
Kenya	Kajiado East	2000	0.010	0.039	0.083
Kenya	Kajiado East	2017	0.001	0.003	0.007
Kenya	Kajiado East	2000-2017	-0.244	-0.138	-0.051
Kenya	Kajiado North	2000	0.003	0.012	0.030
Kenya	Kajiado North	2017	0.000	0.001	0.002
Kenya	Kajiado North	2000-2017	-0.276	-0.153	-0.051
Kenya	Kajiado South	2000	0.003	0.016	0.036
Kenya	Kajiado South	2017	0.001	0.004	0.009
Kenya	Kajiado South	2000-2017	-0.193	-0.116	-0.028
Kenya	Kajiado West	2000	0.014	0.051	0.107
Kenya	Kajiado West	2017	0.001	0.004	0.010
Kenya	Kajiado West	2000-2017	-0.259	-0.142	-0.050
Kenya	Kaloleni	2000	0.156	0.289	0.498
Kenya	Kaloleni	2017	0.008	0.028	0.058
Kenya	Kaloleni	2000-2017	-0.158	-0.091	-0.029
Kenya	Kamukunji	2000	0.000	0.000	0.000
Kenya	Kamukunji	2017	0.000	0.000	0.000
Kenya	Kamukunji	2000-2017	NA	NA	NA
Kenya	Kandara	2000	0.005	0.026	0.074
Kenya	Kandara	2017	0.002	0.010	0.025
Kenya	Kandara	2000-2017	-0.154	-0.048	0.031
Kenya	Kanduyi	2000	0.119	0.252	0.461
Kenya	Kanduyi	2017	0.011	0.042	0.096
Kenya	Kanduyi	2000-2017	-0.144	-0.070	-0.020
Kenya	Kangema	2000	0.007	0.027	0.073
Kenya	Kangema	2017	0.002	0.010	0.024
Kenya	Kangema	2000-2017	-0.155	-0.053	0.019

Table 3: Malaria DALYs rate by unit *(continued)*

Country	Unit	year	mean rate	lower rate	upper rate
Kenya	Kangundo	2000	0.011	0.037	0.083
Kenya	Kangundo	2017	0.001	0.009	0.022
Kenya	Kangundo	2000-2017	-0.140	-0.044	0.037
Kenya	Kapenguria	2000	0.016	0.081	0.170
Kenya	Kapenguria	2017	0.002	0.007	0.016
Kenya	Kapenguria	2000-2017	-0.154	-0.078	-0.012
Kenya	Kapseret	2000	0.011	0.040	0.095
Kenya	Kapseret	2017	0.001	0.004	0.012
Kenya	Kapseret	2000-2017	-0.149	-0.064	0.004
Kenya	Karachuonyo	2000	0.265	0.595	1.412
Kenya	Karachuonyo	2017	0.015	0.054	0.126
Kenya	Karachuonyo	2000-2017	-0.234	-0.156	-0.082
Kenya	Kasarani	2000	0.000	0.001	0.003
Kenya	Kasarani	2017	0.000	0.000	0.001
Kenya	Kasarani	2000-2017	-0.191	-0.082	-0.007
Kenya	Kasipul	2000	0.213	0.496	1.118
Kenya	Kasipul	2017	0.010	0.041	0.093
Kenya	Kasipul	2000-2017	-0.236	-0.148	-0.067
Kenya	Kathiani	2000	0.008	0.035	0.078
Kenya	Kathiani	2017	0.001	0.008	0.021
Kenya	Kathiani	2000-2017	-0.139	-0.047	0.048
Kenya	Keiyo North	2000	0.007	0.027	0.055
Kenya	Keiyo North	2017	0.001	0.003	0.009
Kenya	Keiyo North	2000-2017	-0.144	-0.059	0.032
Kenya	Keiyo South	2000	0.006	0.025	0.052
Kenya	Keiyo South	2017	0.001	0.002	0.006
Kenya	Keiyo South	2000-2017	-0.170	-0.085	0.014
Kenya	Kesses	2000	0.005	0.019	0.043
Kenya	Kesses	2017	0.000	0.002	0.004
Kenya	Kesses	2000-2017	-0.143	-0.075	-0.003
Kenya	Khwisero	2000	0.267	0.481	0.912
Kenya	Khwisero	2017	0.012	0.043	0.093
Kenya	Khwisero	2000-2017	-0.183	-0.120	-0.059
Kenya	Kiambaa	2000	0.001	0.002	0.006
Kenya	Kiambaa	2017	0.000	0.000	0.001
Kenya	Kiambaa	2000-2017	-0.222	-0.105	-0.021
Kenya	Kiambu	2000	0.001	0.004	0.010
Kenya	Kiambu	2017	0.000	0.001	0.002
Kenya	Kiambu	2000-2017	-0.219	-0.097	-0.011
Kenya	Kibra	2000	0.000	0.000	0.000
Kenya	Kibra	2017	0.000	0.000	0.000
Kenya	Kibra	2000-2017	NA	NA	NA
Kenya	Kibwezi East	2000	0.017	0.056	0.125
Kenya	Kibwezi East	2017	0.000	0.002	0.004
Kenya	Kibwezi East	2000-2017	-0.305	-0.173	-0.054
Kenya	Kibwezi West	2000	0.010	0.045	0.108
Kenya	Kibwezi West	2017	0.000	0.001	0.003
Kenya	Kibwezi West	2000-2017	-0.289	-0.166	-0.058
Kenya	Kieni	2000	0.001	0.003	0.007
Kenya	Kieni	2017	0.000	0.001	0.002
Kenya	Kieni	2000-2017	-0.165	-0.051	0.031
Kenya	Kigumo	2000	0.007	0.028	0.077
Kenya	Kigumo	2017	0.002	0.013	0.030
Kenya	Kigumo	2000-2017	-0.132	-0.033	0.045
Kenya	Kiharu	2000	0.008	0.028	0.075
Kenya	Kiharu	2017	0.002	0.011	0.025
Kenya	Kiharu	2000-2017	-0.125	-0.044	0.029
Kenya	Kikuyu	2000	0.000	0.002	0.005
Kenya	Kikuyu	2017	0.000	0.001	0.001
Kenya	Kikuyu	2000-2017	-0.196	-0.065	0.023
Kenya	Kilgoris	2000	0.012	0.038	0.082

Table 3: Malaria DALYs rate by unit *(continued)*

Country	Unit	year	mean rate	lower rate	upper rate
Kenya	Kilgoris	2017	0.001	0.003	0.006
Kenya	Kilgoris	2000-2017	-0.244	-0.167	-0.103
Kenya	Kilifi North	2000	0.155	0.284	0.518
Kenya	Kilifi North	2017	0.003	0.012	0.027
Kenya	Kilifi North	2000-2017	-0.187	-0.117	-0.042
Kenya	Kilifi South	2000	0.092	0.223	0.429
Kenya	Kilifi South	2017	0.006	0.022	0.050
Kenya	Kilifi South	2000-2017	-0.164	-0.093	-0.034
Kenya	Kilome	2000	0.009	0.041	0.093
Kenya	Kilome	2017	0.000	0.001	0.002
Kenya	Kilome	2000-2017	-0.265	-0.158	-0.056
Kenya	Kimilili	2000	0.055	0.148	0.290
Kenya	Kimilili	2017	0.006	0.024	0.058
Kenya	Kimilili	2000-2017	-0.141	-0.068	-0.010
Kenya	Kiminini	2000	0.024	0.081	0.154
Kenya	Kiminini	2017	0.003	0.010	0.024
Kenya	Kiminini	2000-2017	-0.156	-0.080	-0.014
Kenya	Kinango	2000	0.129	0.225	0.365
Kenya	Kinango	2017	0.006	0.023	0.055
Kenya	Kinango	2000-2017	-0.169	-0.101	-0.045
Kenya	Kinangop	2000	0.000	0.000	0.001
Kenya	Kinangop	2017	0.000	0.000	0.000
Kenya	Kinangop	2000-2017	-0.169	-0.061	0.027
Kenya	Kipipiri	2000	0.000	0.000	0.001
Kenya	Kipipiri	2017	0.000	0.000	0.000
Kenya	Kipipiri	2000-2017	-0.133	-0.025	0.064
Kenya	Kipkelion East	2000	0.004	0.015	0.032
Kenya	Kipkelion East	2017	0.000	0.001	0.004
Kenya	Kipkelion East	2000-2017	-0.248	-0.153	-0.059
Kenya	Kipkelion West	2000	0.011	0.033	0.068
Kenya	Kipkelion West	2017	0.001	0.004	0.010
Kenya	Kipkelion West	2000-2017	-0.223	-0.142	-0.060
Kenya	Kirinyaga Central	2000	0.018	0.058	0.122
Kenya	Kirinyaga Central	2017	0.003	0.012	0.032
Kenya	Kirinyaga Central	2000-2017	-0.144	-0.060	0.023
Kenya	Kisauni	2000	0.018	0.060	0.130
Kenya	Kisauni	2017	0.004	0.014	0.032
Kenya	Kisauni	2000-2017	-0.147	-0.073	0.002
Kenya	Kisumu Central	2000	0.164	0.354	0.744
Kenya	Kisumu Central	2017	0.008	0.031	0.074
Kenya	Kisumu Central	2000-2017	-0.221	-0.138	-0.062
Kenya	Kisumu East	2000	0.232	0.468	0.947
Kenya	Kisumu East	2017	0.010	0.036	0.083
Kenya	Kisumu East	2000-2017	-0.226	-0.144	-0.069
Kenya	Kisumu West	2000	0.382	0.739	1.394
Kenya	Kisumu West	2017	0.023	0.083	0.216
Kenya	Kisumu West	2000-2017	-0.200	-0.113	-0.037
Kenya	Kitui Central	2000	0.006	0.029	0.086
Kenya	Kitui Central	2017	0.000	0.003	0.009
Kenya	Kitui Central	2000-2017	-0.211	-0.097	0.021
Kenya	Kitui East	2000	0.009	0.032	0.090
Kenya	Kitui East	2017	0.001	0.003	0.009
Kenya	Kitui East	2000-2017	-0.214	-0.099	0.031
Kenya	Kitui Rural	2000	0.008	0.033	0.085
Kenya	Kitui Rural	2017	0.001	0.003	0.007
Kenya	Kitui Rural	2000-2017	-0.196	-0.096	0.008
Kenya	Kitui South	2000	0.009	0.039	0.093
Kenya	Kitui South	2017	0.001	0.003	0.009
Kenya	Kitui South	2000-2017	-0.233	-0.101	0.019
Kenya	Kitui West	2000	0.005	0.024	0.072
Kenya	Kitui West	2017	0.000	0.003	0.007

Table 3: Malaria DALYs rate by unit *(continued)*

Country	Unit	year	mean rate	lower rate	upper rate
Kenya	Kitui West	2000-2017	-0.215	-0.090	0.027
Kenya	Kitutu Chache North	2000	0.061	0.148	0.315
Kenya	Kitutu Chache North	2017	0.002	0.008	0.017
Kenya	Kitutu Chache North	2000-2017	-0.252	-0.165	-0.082
Kenya	Kitutu Chache South	2000	0.050	0.128	0.282
Kenya	Kitutu Chache South	2017	0.002	0.010	0.023
Kenya	Kitutu Chache South	2000-2017	-0.226	-0.142	-0.060
Kenya	Kitutu Masaba	2000	0.038	0.102	0.191
Kenya	Kitutu Masaba	2017	0.001	0.003	0.007
Kenya	Kitutu Masaba	2000-2017	-0.265	-0.190	-0.113
Kenya	Konoin	2000	0.012	0.038	0.075
Kenya	Konoin	2017	0.000	0.001	0.003
Kenya	Konoin	2000-2017	-0.283	-0.208	-0.135
Kenya	Kuresoi North	2000	0.000	0.000	0.000
Kenya	Kuresoi North	2017	0.000	0.000	0.000
Kenya	Kuresoi North	2000-2017	-0.256	-0.156	-0.061
Kenya	Kuresoi South	2000	0.000	0.000	0.000
Kenya	Kuresoi South	2017	0.000	0.000	0.000
Kenya	Kuresoi South	2000-2017	NA	NA	NA
Kenya	Kuria East	2000	0.078	0.276	0.678
Kenya	Kuria East	2017	0.009	0.036	0.084
Kenya	Kuria East	2000-2017	-0.212	-0.140	-0.065
Kenya	Kuria West	2000	0.128	0.448	1.117
Kenya	Kuria West	2017	0.016	0.064	0.157
Kenya	Kuria West	2000-2017	-0.213	-0.133	-0.060
Kenya	Kwanza	2000	0.018	0.070	0.142
Kenya	Kwanza	2017	0.003	0.010	0.028
Kenya	Kwanza	2000-2017	-0.133	-0.053	0.015
Kenya	Lafey	2000	0.001	0.015	0.071
Kenya	Lafey	2017	0.001	0.004	0.012
Kenya	Lafey	2000-2017	-0.160	-0.072	0.025
Kenya	Lagdera	2000	0.006	0.040	0.127
Kenya	Lagdera	2017	0.000	0.002	0.005
Kenya	Lagdera	2000-2017	-0.292	-0.161	-0.047
Kenya	Laikipia East	2000	0.001	0.004	0.011
Kenya	Laikipia East	2017	0.000	0.000	0.001
Kenya	Laikipia East	2000-2017	-0.266	-0.168	-0.082
Kenya	Laikipia North	2000	0.008	0.039	0.109
Kenya	Laikipia North	2017	0.000	0.003	0.007
Kenya	Laikipia North	2000-2017	-0.202	-0.102	-0.011
Kenya	Laikipia West	2000	0.006	0.035	0.094
Kenya	Laikipia West	2017	0.000	0.002	0.005
Kenya	Laikipia West	2000-2017	-0.226	-0.119	-0.040
Kenya	Laisamis	2000	0.004	0.039	0.113
Kenya	Laisamis	2017	0.000	0.004	0.011
Kenya	Laisamis	2000-2017	-0.200	-0.081	0.042
Kenya	Lamu East	2000	0.031	0.250	0.682
Kenya	Lamu East	2017	0.005	0.031	0.106
Kenya	Lamu East	2000-2017	-0.221	-0.111	0.031
Kenya	Lamu West	2000	0.062	0.336	0.732
Kenya	Lamu West	2017	0.004	0.025	0.075
Kenya	Lamu West	2000-2017	-0.217	-0.105	0.000
Kenya	Langata	2000	0.000	0.002	0.004
Kenya	Langata	2017	0.000	0.000	0.000
Kenya	Langata	2000-2017	-0.234	-0.113	-0.008
Kenya	Lari	2000	0.002	0.007	0.018
Kenya	Lari	2017	0.000	0.003	0.007
Kenya	Lari	2000-2017	-0.145	-0.042	0.047
Kenya	Likoni	2000	0.017	0.057	0.127
Kenya	Likoni	2017	0.003	0.012	0.026
Kenya	Likoni	2000-2017	-0.136	-0.063	0.006

Table 3: Malaria DALYs rate by unit *(continued)*

Country	Unit	year	mean rate	lower rate	upper rate
Kenya	Likuyani	2000	0.041	0.130	0.267
Kenya	Likuyani	2017	0.004	0.016	0.041
Kenya	Likuyani	2000-2017	-0.157	-0.071	0.012
Kenya	Limuru	2000	0.000	0.001	0.003
Kenya	Limuru	2017	0.000	0.000	0.001
Kenya	Limuru	2000-2017	-0.166	-0.043	0.054
Kenya	Loima	2000	0.008	0.061	0.180
Kenya	Loima	2017	0.007	0.022	0.048
Kenya	Loima	2000-2017	-0.074	-0.004	0.078
Kenya	Luanda	2000	0.254	0.503	0.923
Kenya	Luanda	2017	0.011	0.037	0.090
Kenya	Luanda	2000-2017	-0.205	-0.125	-0.058
Kenya	Lugari	2000	0.113	0.266	0.518
Kenya	Lugari	2000	0.051	0.153	0.309
Kenya	Lugari	2017	0.006	0.023	0.053
Kenya	Lugari	2017	0.006	0.022	0.053
Kenya	Lugari	2000-2017	-0.206	-0.128	-0.070
Kenya	Lugari	2000-2017	-0.162	-0.082	-0.004
Kenya	Lungalunga	2000	0.059	0.164	0.347
Kenya	Lungalunga	2017	0.007	0.023	0.057
Kenya	Lungalunga	2000-2017	-0.196	-0.122	-0.058
Kenya	Lurambi	2000	0.151	0.340	0.676
Kenya	Lurambi	2017	0.008	0.030	0.075
Kenya	Lurambi	2000-2017	-0.204	-0.134	-0.071
Kenya	Maara	2000	0.033	0.124	0.321
Kenya	Maara	2017	0.003	0.016	0.039
Kenya	Maara	2000-2017	-0.171	-0.091	-0.013
Kenya	Machakos Town	2000	0.005	0.024	0.053
Kenya	Machakos Town	2017	0.001	0.005	0.014
Kenya	Machakos Town	2000-2017	-0.151	-0.054	0.048
Kenya	Magarini	2000	0.237	0.413	0.698
Kenya	Magarini	2017	0.004	0.013	0.027
Kenya	Magarini	2000-2017	-0.197	-0.128	-0.061
Kenya	Makadara	2000	0.000	0.000	0.000
Kenya	Makadara	2017	0.000	0.000	0.000
Kenya	Makadara	2000-2017	NA	NA	NA
Kenya	Makueni	2000	0.007	0.039	0.096
Kenya	Makueni	2017	0.000	0.001	0.002
Kenya	Makueni	2000-2017	-0.271	-0.168	-0.047
Kenya	Malava	2000	0.130	0.306	0.593
Kenya	Malava	2017	0.007	0.024	0.060
Kenya	Malava	2000-2017	-0.213	-0.144	-0.084
Kenya	Malindi	2000	0.168	0.302	0.515
Kenya	Malindi	2017	0.002	0.006	0.014
Kenya	Malindi	2000-2017	-0.238	-0.169	-0.099
Kenya	Mandera East	2000	0.001	0.017	0.081
Kenya	Mandera East	2017	0.001	0.007	0.018
Kenya	Mandera East	2000-2017	-0.162	-0.055	0.033
Kenya	Mandera North	2000	0.002	0.022	0.097
Kenya	Mandera North	2017	0.001	0.005	0.015
Kenya	Mandera North	2000-2017	-0.154	-0.055	0.065
Kenya	Mandera South	2000	0.001	0.010	0.059
Kenya	Mandera South	2017	0.000	0.003	0.008
Kenya	Mandera South	2000-2017	-0.177	-0.060	0.045
Kenya	Mandera West	2000	0.002	0.027	0.115
Kenya	Mandera West	2017	0.001	0.005	0.013
Kenya	Mandera West	2000-2017	-0.166	-0.053	0.063
Kenya	Manyatta	2000	0.009	0.028	0.062
Kenya	Manyatta	2017	0.002	0.008	0.020
Kenya	Manyatta	2000-2017	-0.143	-0.061	0.010
Kenya	Maragwa	2000	0.006	0.025	0.067

Table 3: Malaria DALYs rate by unit *(continued)*

Country	Unit	year	mean rate	lower rate	upper rate
Kenya	Maragwa	2017	0.002	0.011	0.025
Kenya	Maragwa	2000-2017	-0.126	-0.032	0.049
Kenya	Marakwet East	2000	0.010	0.043	0.090
Kenya	Marakwet East	2017	0.001	0.005	0.013
Kenya	Marakwet East	2000-2017	-0.174	-0.055	0.066
Kenya	Marakwet West	2000	0.008	0.032	0.068
Kenya	Marakwet West	2017	0.001	0.003	0.008
Kenya	Marakwet West	2000-2017	-0.151	-0.056	0.048
Kenya	Masinga	2000	0.006	0.024	0.055
Kenya	Masinga	2017	0.002	0.010	0.021
Kenya	Masinga	2000-2017	-0.105	-0.005	0.076
Kenya	Matayos	2000	0.317	0.577	0.941
Kenya	Matayos	2017	0.048	0.142	0.292
Kenya	Matayos	2000-2017	-0.149	-0.072	-0.019
Kenya	Mathare	2000	0.000	0.000	0.000
Kenya	Mathare	2017	0.000	0.000	0.000
Kenya	Mathare	2000-2017	NA	NA	NA
Kenya	Mathioya	2000	0.009	0.030	0.076
Kenya	Mathioya	2017	0.002	0.011	0.025
Kenya	Mathioya	2000-2017	-0.151	-0.053	0.017
Kenya	Mathira	2000	0.010	0.033	0.078
Kenya	Mathira	2017	0.002	0.011	0.028
Kenya	Mathira	2000-2017	-0.142	-0.045	0.028
Kenya	Matuga	2000	0.125	0.249	0.475
Kenya	Matuga	2017	0.010	0.033	0.071
Kenya	Matuga	2000-2017	-0.177	-0.111	-0.060
Kenya	Matungu	2000	0.337	0.661	1.152
Kenya	Matungu	2017	0.023	0.081	0.198
Kenya	Matungu	2000-2017	-0.165	-0.098	-0.053
Kenya	Matungulu	2000	0.008	0.031	0.078
Kenya	Matungulu	2017	0.001	0.007	0.017
Kenya	Matungulu	2000-2017	-0.146	-0.055	0.023
Kenya	Mavoko	2000	0.004	0.017	0.038
Kenya	Mavoko	2017	0.001	0.003	0.009
Kenya	Mavoko	2000-2017	-0.169	-0.066	0.023
Kenya	Mbeere North	2000	0.009	0.027	0.056
Kenya	Mbeere North	2017	0.002	0.010	0.025
Kenya	Mbeere North	2000-2017	-0.131	-0.033	0.061
Kenya	Mbeere South	2000	0.008	0.028	0.061
Kenya	Mbeere South	2017	0.002	0.009	0.021
Kenya	Mbeere South	2000-2017	-0.106	-0.023	0.056
Kenya	Mbita	2000	0.164	0.455	0.988
Kenya	Mbita	2017	0.025	0.083	0.221
Kenya	Mbita	2000-2017	-0.179	-0.106	-0.051
Kenya	Mbooni	2000	0.010	0.042	0.100
Kenya	Mbooni	2017	0.000	0.001	0.003
Kenya	Mbooni	2000-2017	-0.237	-0.150	-0.065
Kenya	Mogotio	2000	0.031	0.103	0.264
Kenya	Mogotio	2017	0.001	0.004	0.011
Kenya	Mogotio	2000-2017	-0.206	-0.126	-0.051
Kenya	Moiben	2000	0.009	0.034	0.074
Kenya	Moiben	2017	0.001	0.003	0.010
Kenya	Moiben	2000-2017	-0.133	-0.047	0.035
Kenya	Molo	2000	0.001	0.003	0.007
Kenya	Molo	2017	0.000	0.000	0.001
Kenya	Molo	2000-2017	-0.208	-0.119	-0.043
Kenya	Mosop	2000	0.022	0.063	0.125
Kenya	Mosop	2017	0.002	0.009	0.025
Kenya	Mosop	2000-2017	-0.181	-0.096	-0.037
Kenya	Moyale	2000	0.003	0.043	0.179
Kenya	Moyale	2017	0.001	0.006	0.015

Table 3: Malaria DALYs rate by unit *(continued)*

Country	Unit	year	mean rate	lower rate	upper rate
Kenya	Moyale	2000-2017	-0.220	-0.074	0.054
Kenya	Msambweni	2000	0.075	0.186	0.401
Kenya	Msambweni	2017	0.006	0.024	0.052
Kenya	Msambweni	2000-2017	-0.190	-0.115	-0.065
Kenya	Mt. Elgon	2000	0.050	0.149	0.329
Kenya	Mt. Elgon	2017	0.007	0.029	0.067
Kenya	Mt. Elgon	2000-2017	-0.148	-0.070	-0.018
Kenya	Muhoroni	2000	0.207	0.460	0.939
Kenya	Muhoroni	2017	0.007	0.027	0.060
Kenya	Muhoroni	2000-2017	-0.268	-0.187	-0.123
Kenya	Mukurweini	2000	0.011	0.035	0.086
Kenya	Mukurweini	2017	0.003	0.014	0.032
Kenya	Mukurweini	2000-2017	-0.125	-0.035	0.035
Kenya	Mumias East	2000	0.251	0.511	0.957
Kenya	Mumias East	2017	0.012	0.046	0.116
Kenya	Mumias East	2000-2017	-0.194	-0.126	-0.075
Kenya	Mumias West	2000	0.295	0.546	0.988
Kenya	Mumias West	2017	0.017	0.060	0.144
Kenya	Mumias West	2000-2017	-0.176	-0.108	-0.064
Kenya	Mvita	2000	0.012	0.043	0.095
Kenya	Mvita	2017	0.003	0.010	0.023
Kenya	Mvita	2000-2017	-0.123	-0.051	0.022
Kenya	Mwala	2000	0.010	0.039	0.088
Kenya	Mwala	2017	0.001	0.008	0.022
Kenya	Mwala	2000-2017	-0.154	-0.046	0.055
Kenya	Mwatate	2000	0.034	0.119	0.249
Kenya	Mwatate	2017	0.003	0.015	0.038
Kenya	Mwatate	2000-2017	-0.173	-0.083	0.015
Kenya	Mwea	2000	0.013	0.044	0.092
Kenya	Mwea	2017	0.002	0.011	0.028
Kenya	Mwea	2000-2017	-0.127	-0.036	0.053
Kenya	Mwingi Central	2000	0.006	0.029	0.083
Kenya	Mwingi Central	2017	0.001	0.004	0.010
Kenya	Mwingi Central	2000-2017	-0.218	-0.087	0.034
Kenya	Mwingi North	2000	0.007	0.027	0.068
Kenya	Mwingi North	2017	0.001	0.003	0.008
Kenya	Mwingi North	2000-2017	-0.213	-0.102	0.007
Kenya	Mwingi West	2000	0.005	0.026	0.069
Kenya	Mwingi West	2017	0.000	0.003	0.008
Kenya	Mwingi West	2000-2017	-0.198	-0.077	0.051
Kenya	Naivasha	2000	0.002	0.015	0.049
Kenya	Naivasha	2017	0.001	0.003	0.006
Kenya	Naivasha	2000-2017	-0.208	-0.084	0.014
Kenya	Nakuru Town East	2000	0.004	0.024	0.065
Kenya	Nakuru Town East	2017	0.000	0.002	0.006
Kenya	Nakuru Town East	2000-2017	-0.211	-0.105	0.000
Kenya	Nakuru Town West	2000	0.004	0.021	0.053
Kenya	Nakuru Town West	2017	0.000	0.002	0.005
Kenya	Nakuru Town West	2000-2017	-0.215	-0.113	-0.015
Kenya	Nambale	2000	0.300	0.551	0.909
Kenya	Nambale	2017	0.030	0.102	0.233
Kenya	Nambale	2000-2017	-0.136	-0.065	-0.017
Kenya	Nandi Hills	2000	0.013	0.051	0.108
Kenya	Nandi Hills	2017	0.001	0.004	0.010
Kenya	Nandi Hills	2000-2017	-0.223	-0.126	-0.049
Kenya	Narok East	2000	0.003	0.018	0.042
Kenya	Narok East	2017	0.000	0.001	0.002
Kenya	Narok East	2000-2017	-0.263	-0.160	-0.066
Kenya	Narok North	2000	0.002	0.009	0.023
Kenya	Narok North	2017	0.000	0.000	0.001
Kenya	Narok North	2000-2017	-0.322	-0.196	-0.093

Table 3: Malaria DALYs rate by unit *(continued)*

Country	Unit	year	mean rate	lower rate	upper rate
Kenya	Narok South	2000	0.004	0.015	0.043
Kenya	Narok South	2017	0.000	0.001	0.002
Kenya	Narok South	2000-2017	-0.266	-0.168	-0.069
Kenya	Narok West	2000	0.008	0.026	0.065
Kenya	Narok West	2017	0.000	0.001	0.003
Kenya	Narok West	2000-2017	-0.282	-0.186	-0.084
Kenya	Navakholo	2000	0.184	0.433	0.834
Kenya	Navakholo	2017	0.009	0.034	0.085
Kenya	Navakholo	2000-2017	-0.202	-0.126	-0.071
Kenya	Ndaragwa	2000	0.000	0.000	0.000
Kenya	Ndaragwa	2017	0.000	0.000	0.000
Kenya	Ndaragwa	2000-2017	-0.210	-0.097	-0.008
Kenya	Ndhiwa	2000	0.153	0.369	0.834
Kenya	Ndhiwa	2017	0.019	0.067	0.154
Kenya	Ndhiwa	2000-2017	-0.172	-0.111	-0.054
Kenya	Ndia	2000	0.015	0.052	0.112
Kenya	Ndia	2017	0.003	0.012	0.032
Kenya	Ndia	2000-2017	-0.130	-0.050	0.029
Kenya	Njoro	2000	0.002	0.008	0.020
Kenya	Njoro	2017	0.000	0.001	0.003
Kenya	Njoro	2000-2017	-0.205	-0.105	-0.012
Kenya	North Horr	2000	0.002	0.028	0.089
Kenya	North Horr	2017	0.001	0.004	0.012
Kenya	North Horr	2000-2017	-0.230	-0.094	0.001
Kenya	North Imenti	2000	0.006	0.024	0.056
Kenya	North Imenti	2017	0.002	0.010	0.024
Kenya	North Imenti	2000-2017	-0.138	-0.028	0.062
Kenya	North Mugirango	2000	0.082	0.183	0.347
Kenya	North Mugirango	2017	0.001	0.004	0.008
Kenya	North Mugirango	2000-2017	-0.314	-0.230	-0.163
Kenya	Nyakach	2000	0.256	0.554	1.132
Kenya	Nyakach	2017	0.009	0.037	0.074
Kenya	Nyakach	2000-2017	-0.264	-0.180	-0.111
Kenya	Nyali	2000	0.012	0.044	0.098
Kenya	Nyali	2017	0.003	0.010	0.023
Kenya	Nyali	2000-2017	-0.142	-0.066	0.014
Kenya	Nyando	2000	0.287	0.598	1.173
Kenya	Nyando	2017	0.009	0.038	0.084
Kenya	Nyando	2000-2017	-0.275	-0.186	-0.117
Kenya	Nyaribari Chache	2000	0.027	0.082	0.170
Kenya	Nyaribari Chache	2017	0.001	0.004	0.011
Kenya	Nyaribari Chache	2000-2017	-0.237	-0.163	-0.081
Kenya	Nyaribari Masaba	2000	0.018	0.060	0.121
Kenya	Nyaribari Masaba	2017	0.000	0.002	0.005
Kenya	Nyaribari Masaba	2000-2017	-0.280	-0.173	-0.087
Kenya	Nyatike	2000	0.138	0.409	0.914
Kenya	Nyatike	2017	0.023	0.083	0.202
Kenya	Nyatike	2000-2017	-0.181	-0.119	-0.055
Kenya	Nyeri Town	2000	0.004	0.018	0.046
Kenya	Nyeri Town	2017	0.001	0.005	0.010
Kenya	Nyeri Town	2000-2017	-0.183	-0.065	0.014
Kenya	Ol Jorok	2000	0.000	0.000	0.000
Kenya	Ol Jorok	2017	0.000	0.000	0.000
Kenya	Ol Jorok	2000-2017	NA	NA	NA
Kenya	Ol Kalou	2000	0.000	0.001	0.003
Kenya	Ol Kalou	2017	0.000	0.000	0.001
Kenya	Ol Kalou	2000-2017	-0.218	-0.102	0.014
Kenya	Othaya	2000	0.007	0.026	0.065
Kenya	Othaya	2017	0.002	0.009	0.019
Kenya	Othaya	2000-2017	-0.164	-0.049	0.026
Kenya	Pokot South	2000	0.008	0.039	0.088

Table 3: Malaria DALYs rate by unit *(continued)*

Country	Unit	year	mean rate	lower rate	upper rate
Kenya	Pokot South	2017	0.001	0.003	0.007
Kenya	Pokot South	2000-2017	-0.172	-0.084	-0.020
Kenya	Rabai	2000	0.097	0.236	0.436
Kenya	Rabai	2017	0.007	0.025	0.055
Kenya	Rabai	2000-2017	-0.163	-0.099	-0.038
Kenya	Rangwe	2000	0.236	0.540	1.316
Kenya	Rangwe	2017	0.019	0.065	0.144
Kenya	Rangwe	2000-2017	-0.212	-0.132	-0.060
Kenya	Rarieda	2000	0.144	0.312	0.665
Kenya	Rarieda	2017	0.018	0.061	0.151
Kenya	Rarieda	2000-2017	-0.192	-0.114	-0.047
Kenya	Rongai	2000	0.016	0.052	0.123
Kenya	Rongai	2017	0.001	0.006	0.016
Kenya	Rongai	2000-2017	-0.188	-0.097	-0.025
Kenya	Rongo	2000	0.197	0.523	1.360
Kenya	Rongo	2017	0.014	0.050	0.113
Kenya	Rongo	2000-2017	-0.213	-0.141	-0.083
Kenya	Roysambu	2000	0.000	0.000	0.000
Kenya	Roysambu	2017	0.000	0.000	0.000
Kenya	Roysambu	2000-2017	-0.204	-0.087	-0.004
Kenya	Ruaraka	2000	0.000	0.000	0.000
Kenya	Ruaraka	2017	0.000	0.000	0.000
Kenya	Ruaraka	2000-2017	NA	NA	NA
Kenya	Ruiru	2000	0.001	0.005	0.014
Kenya	Ruiru	2017	0.000	0.001	0.003
Kenya	Ruiru	2000-2017	-0.184	-0.072	0.006
Kenya	Runyenjes	2000	0.009	0.028	0.058
Kenya	Runyenjes	2017	0.002	0.010	0.024
Kenya	Runyenjes	2000-2017	-0.133	-0.045	0.027
Kenya	Sabatia	2000	0.158	0.328	0.666
Kenya	Sabatia	2017	0.005	0.016	0.039
Kenya	Sabatia	2000-2017	-0.211	-0.149	-0.089
Kenya	Saboti	2000	0.017	0.064	0.117
Kenya	Saboti	2017	0.002	0.007	0.019
Kenya	Saboti	2000-2017	-0.145	-0.074	-0.008
Kenya	Saku	2000	0.004	0.071	0.221
Kenya	Saku	2017	0.001	0.009	0.027
Kenya	Saku	2000-2017	-0.300	-0.115	0.062
Kenya	Samburu East	2000	0.006	0.052	0.157
Kenya	Samburu East	2017	0.001	0.004	0.013
Kenya	Samburu East	2000-2017	-0.218	-0.076	0.078
Kenya	Samburu North	2000	0.007	0.048	0.126
Kenya	Samburu North	2017	0.001	0.004	0.011
Kenya	Samburu North	2000-2017	-0.186	-0.080	0.052
Kenya	Samburu West	2000	0.004	0.028	0.074
Kenya	Samburu West	2017	0.000	0.002	0.006
Kenya	Samburu West	2000-2017	-0.212	-0.087	0.040
Kenya	Seme	2000	0.448	0.854	1.588
Kenya	Seme	2017	0.042	0.148	0.374
Kenya	Seme	2000-2017	-0.211	-0.123	-0.053
Kenya	Shinyalu	2000	0.101	0.242	0.483
Kenya	Shinyalu	2017	0.005	0.020	0.048
Kenya	Shinyalu	2000-2017	-0.215	-0.149	-0.086
Kenya	Sigor	2000	0.017	0.067	0.157
Kenya	Sigor	2017	0.001	0.005	0.014
Kenya	Sigor	2000-2017	-0.174	-0.080	0.032
Kenya	Sigowet/Soin	2000	0.047	0.108	0.196
Kenya	Sigowet/Soin	2017	0.002	0.010	0.025
Kenya	Sigowet/Soin	2000-2017	-0.260	-0.171	-0.107
Kenya	Sirisia	2000	0.132	0.286	0.511
Kenya	Sirisia	2017	0.012	0.050	0.116

Table 3: Malaria DALYs rate by unit *(continued)*

Country	Unit	year	mean rate	lower rate	upper rate
Kenya	Sirisia	2000-2017	-0.144	-0.067	-0.017
Kenya	Sotik	2000	0.016	0.056	0.111
Kenya	Sotik	2017	0.000	0.001	0.004
Kenya	Sotik	2000-2017	-0.298	-0.204	-0.122
Kenya	South Imenti	2000	0.008	0.030	0.074
Kenya	South Imenti	2017	0.002	0.013	0.029
Kenya	South Imenti	2000-2017	-0.127	-0.034	0.052
Kenya	South Mugirango	2000	0.032	0.105	0.229
Kenya	South Mugirango	2017	0.002	0.010	0.024
Kenya	South Mugirango	2000-2017	-0.214	-0.137	-0.070
Kenya	Soy	2000	0.024	0.079	0.160
Kenya	Soy	2017	0.002	0.007	0.021
Kenya	Soy	2000-2017	-0.158	-0.073	0.006
Kenya	Starehe	2000	0.000	0.000	0.000
Kenya	Starehe	2017	0.000	0.000	0.000
Kenya	Starehe	2000-2017	NA	NA	NA
Kenya	Suba	2000	0.116	0.364	0.817
Kenya	Suba	2017	0.027	0.088	0.211
Kenya	Suba	2000-2017	-0.168	-0.103	-0.047
Kenya	Subukia	2000	0.007	0.031	0.086
Kenya	Subukia	2017	0.001	0.005	0.014
Kenya	Subukia	2000-2017	-0.153	-0.068	0.016
Kenya	Suna East	2000	0.074	0.277	0.670
Kenya	Suna East	2017	0.010	0.040	0.108
Kenya	Suna East	2000-2017	-0.210	-0.125	-0.042
Kenya	Suna West	2000	0.092	0.308	0.767
Kenya	Suna West	2017	0.013	0.048	0.119
Kenya	Suna West	2000-2017	-0.205	-0.131	-0.066
Kenya	Tarbaj	2000	0.003	0.026	0.111
Kenya	Tarbaj	2017	0.000	0.003	0.010
Kenya	Tarbaj	2000-2017	-0.250	-0.071	0.062
Kenya	Taveta	2000	0.007	0.029	0.074
Kenya	Taveta	2017	0.002	0.011	0.026
Kenya	Taveta	2000-2017	-0.181	-0.074	0.019
Kenya	Teso North	2000	0.357	0.644	1.084
Kenya	Teso North	2017	0.030	0.103	0.225
Kenya	Teso North	2000-2017	-0.165	-0.088	-0.039
Kenya	Teso South	2000	0.356	0.654	1.075
Kenya	Teso South	2017	0.049	0.143	0.298
Kenya	Teso South	2000-2017	-0.160	-0.082	-0.031
Kenya	Tetu	2000	0.004	0.017	0.043
Kenya	Tetu	2017	0.001	0.005	0.011
Kenya	Tetu	2000-2017	-0.165	-0.051	0.022
Kenya	Tharaka	2000	0.032	0.138	0.353
Kenya	Tharaka	2017	0.002	0.015	0.039
Kenya	Tharaka	2000-2017	-0.200	-0.100	0.016
Kenya	Thika Town	2000	0.004	0.018	0.054
Kenya	Thika Town	2017	0.001	0.005	0.012
Kenya	Thika Town	2000-2017	-0.144	-0.054	0.023
Kenya	Tiaty	2000	0.030	0.119	0.264
Kenya	Tiaty	2017	0.001	0.006	0.018
Kenya	Tiaty	2000-2017	-0.185	-0.092	0.017
Kenya	Tigania East	2000	0.009	0.040	0.104
Kenya	Tigania East	2017	0.002	0.015	0.036
Kenya	Tigania East	2000-2017	-0.143	-0.029	0.073
Kenya	Tigania West	2000	0.009	0.038	0.092
Kenya	Tigania West	2017	0.003	0.017	0.038
Kenya	Tigania West	2000-2017	-0.157	-0.026	0.073
Kenya	Tinderet	2000	0.024	0.067	0.136
Kenya	Tinderet	2017	0.001	0.005	0.014
Kenya	Tinderet	2000-2017	-0.237	-0.149	-0.083

Table 3: Malaria DALYs rate by unit *(continued)*

Country	Unit	year	mean rate	lower rate	upper rate
Kenya	Tongaren	2000	0.045	0.132	0.246
Kenya	Tongaren	2017	0.005	0.019	0.042
Kenya	Tongaren	2000-2017	-0.160	-0.077	-0.002
Kenya	Turbo	2000	0.027	0.080	0.162
Kenya	Turbo	2017	0.002	0.009	0.024
Kenya	Turbo	2000-2017	-0.170	-0.082	-0.012
Kenya	Turkana Central	2000	0.004	0.041	0.131
Kenya	Turkana Central	2017	0.001	0.006	0.017
Kenya	Turkana Central	2000-2017	-0.222	-0.078	0.057
Kenya	Turkana East	2000	0.011	0.059	0.191
Kenya	Turkana East	2017	0.001	0.004	0.010
Kenya	Turkana East	2000-2017	-0.204	-0.096	0.037
Kenya	Turkana North	2000	0.003	0.042	0.151
Kenya	Turkana North	2017	0.001	0.008	0.028
Kenya	Turkana North	2000-2017	-0.213	-0.064	0.071
Kenya	Turkana South	2000	0.009	0.067	0.225
Kenya	Turkana South	2017	0.002	0.008	0.019
Kenya	Turkana South	2000-2017	-0.177	-0.070	0.024
Kenya	Turkana West	2000	0.012	0.075	0.175
Kenya	Turkana West	2017	0.014	0.040	0.078
Kenya	Turkana West	2000-2017	-0.038	0.026	0.108
Kenya	Ugenya	2000	0.362	0.689	1.154
Kenya	Ugenya	2017	0.056	0.162	0.338
Kenya	Ugenya	2000-2017	-0.163	-0.084	-0.027
Kenya	Ugunja	2000	0.373	0.694	1.182
Kenya	Ugunja	2017	0.039	0.123	0.263
Kenya	Ugunja	2000-2017	-0.176	-0.096	-0.037
Kenya	Uriri	2000	0.089	0.306	0.720
Kenya	Uriri	2017	0.012	0.043	0.114
Kenya	Uriri	2000-2017	-0.200	-0.130	-0.069
Kenya	Vihiga	2000	0.219	0.438	0.835
Kenya	Vihiga	2017	0.007	0.023	0.055
Kenya	Vihiga	2000-2017	-0.214	-0.143	-0.077
Kenya	Voi	2000	0.053	0.144	0.272
Kenya	Voi	2017	0.003	0.015	0.036
Kenya	Voi	2000-2017	-0.164	-0.087	0.017
Kenya	Wajir East	2000	0.002	0.025	0.095
Kenya	Wajir East	2017	0.001	0.003	0.007
Kenya	Wajir East	2000-2017	-0.243	-0.079	0.032
Kenya	Wajir North	2000	0.003	0.042	0.164
Kenya	Wajir North	2017	0.001	0.003	0.009
Kenya	Wajir North	2000-2017	-0.221	-0.088	0.020
Kenya	Wajir South	2000	0.005	0.039	0.106
Kenya	Wajir South	2017	0.001	0.003	0.009
Kenya	Wajir South	2000-2017	-0.231	-0.115	-0.016
Kenya	Wajir West	2000	0.004	0.035	0.107
Kenya	Wajir West	2017	0.001	0.003	0.008
Kenya	Wajir West	2000-2017	-0.254	-0.107	-0.007
Kenya	Webute West	2000	0.076	0.190	0.376
Kenya	Webute West	2017	0.009	0.033	0.074
Kenya	Webute West	2000-2017	-0.141	-0.076	-0.021
Kenya	Webuye East	2000	0.064	0.155	0.302
Kenya	Webuye East	2017	0.008	0.030	0.069
Kenya	Webuye East	2000-2017	-0.142	-0.070	-0.015
Kenya	West Mugirango	2000	0.067	0.152	0.286
Kenya	West Mugirango	2017	0.001	0.005	0.010
Kenya	West Mugirango	2000-2017	-0.270	-0.196	-0.127
Kenya	Westlands	2000	0.000	0.000	0.000
Kenya	Westlands	2017	0.000	0.000	0.000
Kenya	Westlands	2000-2017	NA	NA	NA
Kenya	Wundanyi	2000	0.028	0.105	0.224

Table 3: Malaria DALYs rate by unit *(continued)*

Country	Unit	year	mean rate	lower rate	upper rate
Kenya	Wundanyi	2017	0.002	0.012	0.037
Kenya	Wundanyi	2000-2017	-0.168	-0.081	0.017
Kenya	Yatta	2000	0.007	0.028	0.064
Kenya	Yatta	2017	0.001	0.008	0.020
Kenya	Yatta	2000-2017	-0.133	-0.028	0.056
Lesotho	Berea	2000	0.000	0.000	0.000
Lesotho	Berea	2017	0.000	0.000	0.000
Lesotho	Berea	2000-2017	NA	NA	NA
Lesotho	Butha-Buthe	2000	0.000	0.000	0.000
Lesotho	Butha-Buthe	2017	0.000	0.000	0.000
Lesotho	Butha-Buthe	2000-2017	NA	NA	NA
Lesotho	Leribe	2000	0.000	0.000	0.000
Lesotho	Leribe	2017	0.000	0.000	0.000
Lesotho	Leribe	2000-2017	NA	NA	NA
Lesotho	Mafeteng	2000	0.000	0.000	0.000
Lesotho	Mafeteng	2017	0.000	0.000	0.000
Lesotho	Mafeteng	2000-2017	NA	NA	NA
Lesotho	Maseru	2000	0.000	0.000	0.000
Lesotho	Maseru	2017	0.000	0.000	0.000
Lesotho	Maseru	2000-2017	NA	NA	NA
Lesotho	Mohale's Hoek	2000	0.000	0.000	0.000
Lesotho	Mohale's Hoek	2017	0.000	0.000	0.000
Lesotho	Mohale's Hoek	2000-2017	NA	NA	NA
Lesotho	Mokhotlong	2000	0.000	0.000	0.000
Lesotho	Mokhotlong	2017	0.000	0.000	0.000
Lesotho	Mokhotlong	2000-2017	NA	NA	NA
Lesotho	Qacha's Nek	2000	0.000	0.000	0.000
Lesotho	Qacha's Nek	2017	0.000	0.000	0.000
Lesotho	Qacha's Nek	2000-2017	NA	NA	NA
Lesotho	Quthing	2000	0.000	0.000	0.000
Lesotho	Quthing	2017	0.000	0.000	0.000
Lesotho	Quthing	2000-2017	NA	NA	NA
Lesotho	Thaba-Tseka	2000	0.000	0.000	0.000
Lesotho	Thaba-Tseka	2017	0.000	0.000	0.000
Lesotho	Thaba-Tseka	2000-2017	NA	NA	NA
Liberia	Barrobo	2000	0.144	0.930	1.772
Liberia	Barrobo	2017	0.039	0.274	0.683
Liberia	Barrobo	2000-2017	-0.224	-0.086	0.011
Liberia	Belleh	2000	0.116	0.591	1.486
Liberia	Belleh	2017	0.035	0.198	0.522
Liberia	Belleh	2000-2017	-0.187	-0.079	0.011
Liberia	Bokomu	2000	0.110	0.572	1.195
Liberia	Bokomu	2017	0.037	0.187	0.421
Liberia	Bokomu	2000-2017	-0.198	-0.083	0.014
Liberia	Bopolu	2000	0.100	0.544	1.068
Liberia	Bopolu	2017	0.055	0.218	0.458
Liberia	Bopolu	2000-2017	-0.149	-0.058	0.015
Liberia	Buah	2000	0.118	0.829	1.641
Liberia	Buah	2017	0.041	0.266	0.644
Liberia	Buah	2000-2017	-0.201	-0.080	0.011
Liberia	Butaw	2000	0.090	0.607	1.332
Liberia	Butaw	2017	0.039	0.205	0.553
Liberia	Butaw	2000-2017	-0.212	-0.092	-0.004
Liberia	Careysburg	2000	0.071	0.369	0.959
Liberia	Careysburg	2017	0.017	0.155	0.482
Liberia	Careysburg	2000-2017	-0.214	-0.088	0.005
Liberia	Commnwealth	2000	0.046	0.364	0.911
Liberia	Commnwealth	2017	0.034	0.162	0.400
Liberia	Commnwealth	2000-2017	-0.170	-0.061	0.043
Liberia	District # 1	2000	0.098	0.451	1.093
Liberia	District # 1	2017	0.023	0.195	0.551

Table 3: Malaria DALYs rate by unit *(continued)*

Country	Unit	year	mean rate	lower rate	upper rate
Liberia	District # 1	2000-2017	-0.217	-0.089	0.009
Liberia	District # 2	2000	0.113	0.484	1.149
Liberia	District # 2	2017	0.032	0.197	0.488
Liberia	District # 2	2000-2017	-0.212	-0.092	0.007
Liberia	District # 3	2000	0.094	0.433	1.182
Liberia	District # 3	2017	0.019	0.153	0.422
Liberia	District # 3	2000-2017	-0.240	-0.106	0.010
Liberia	District # 4	2000	0.098	0.528	1.362
Liberia	District # 4	2017	0.018	0.168	0.479
Liberia	District # 4	2000-2017	-0.232	-0.110	0.009
Liberia	Dugbe River	2000	0.105	0.676	1.611
Liberia	Dugbe River	2017	0.039	0.241	0.667
Liberia	Dugbe River	2000-2017	-0.211	-0.083	0.021
Liberia	Firestone	2000	0.048	0.314	0.808
Liberia	Firestone	2017	0.012	0.127	0.368
Liberia	Firestone	2000-2017	-0.233	-0.092	-0.001
Liberia	Foya	2000	0.110	0.562	1.148
Liberia	Foya	2017	0.053	0.161	0.320
Liberia	Foya	2000-2017	-0.169	-0.098	-0.040
Liberia	Fuamah	2000	0.082	0.458	0.919
Liberia	Fuamah	2017	0.036	0.200	0.428
Liberia	Fuamah	2000-2017	-0.184	-0.064	0.025
Liberia	Garwula	2000	0.074	0.448	1.030
Liberia	Garwula	2017	0.057	0.214	0.474
Liberia	Garwula	2000-2017	-0.137	-0.051	0.024
Liberia	Gbarma	2000	0.107	0.519	1.058
Liberia	Gbarma	2017	0.059	0.215	0.442
Liberia	Gbarma	2000-2017	-0.145	-0.055	0.023
Liberia	Gbarzon	2000	0.188	0.708	1.397
Liberia	Gbarzon	2017	0.027	0.212	0.586
Liberia	Gbarzon	2000-2017	-0.249	-0.117	-0.025
Liberia	Gbeapo	2000	0.229	0.857	1.811
Liberia	Gbeapo	2017	0.036	0.251	0.656
Liberia	Gbeapo	2000-2017	-0.214	-0.082	0.007
Liberia	Gbehlageh	2000	0.251	0.758	1.756
Liberia	Gbehlageh	2017	0.026	0.211	0.538
Liberia	Gbehlageh	2000-2017	-0.256	-0.127	-0.034
Liberia	Gibi	2000	0.095	0.466	1.057
Liberia	Gibi	2017	0.028	0.201	0.479
Liberia	Gibi	2000-2017	-0.212	-0.087	0.021
Liberia	Golakonneh	2000	0.124	0.567	1.141
Liberia	Golakonneh	2017	0.071	0.254	0.515
Liberia	Golakonneh	2000-2017	-0.124	-0.045	0.018
Liberia	Greater Monrovia	2000	0.014	0.126	0.412
Liberia	Greater Monrovia	2017	0.004	0.049	0.195
Liberia	Greater Monrovia	2000-2017	-0.253	-0.093	0.051
Liberia	Greenville	2000	0.027	0.286	0.884
Liberia	Greenville	2017	0.013	0.108	0.403
Liberia	Greenville	2000-2017	-0.246	-0.099	0.032
Liberia	Jaedae Jaedepo	2000	0.087	0.635	1.610
Liberia	Jaedae Jaedepo	2017	0.041	0.215	0.504
Liberia	Jaedae Jaedepo	2000-2017	-0.184	-0.078	0.023
Liberia	Jorquelleh	2000	0.110	0.611	1.504
Liberia	Jorquelleh	2017	0.036	0.224	0.561
Liberia	Jorquelleh	2000-2017	-0.227	-0.102	-0.001
Liberia	Juarzon	2000	0.100	0.709	1.504
Liberia	Juarzon	2017	0.048	0.240	0.598
Liberia	Juarzon	2000-2017	-0.216	-0.094	0.001
Liberia	Kakata	2000	0.122	0.483	1.042
Liberia	Kakata	2017	0.034	0.225	0.566
Liberia	Kakata	2000-2017	-0.194	-0.068	0.018

Table 3: Malaria DALYs rate by unit *(continued)*

Country	Unit	year	mean rate	lower rate	upper rate
Liberia	Klay	2000	0.080	0.429	0.971
Liberia	Klay	2017	0.032	0.174	0.460
Liberia	Klay	2000-2017	-0.181	-0.074	0.003
Liberia	Kokoyah	2000	0.118	0.535	1.302
Liberia	Kokoyah	2017	0.041	0.203	0.475
Liberia	Kokoyah	2000-2017	-0.222	-0.104	-0.002
Liberia	Kolahun	2000	0.113	0.660	1.309
Liberia	Kolahun	2017	0.076	0.196	0.399
Liberia	Kolahun	2000-2017	-0.153	-0.090	-0.035
Liberia	Kongba	2000	0.179	0.787	1.554
Liberia	Kongba	2017	0.103	0.314	0.609
Liberia	Kongba	2000-2017	-0.121	-0.055	-0.008
Liberia	Konobo	2000	0.320	0.936	1.859
Liberia	Konobo	2017	0.041	0.235	0.540
Liberia	Konobo	2000-2017	-0.215	-0.103	-0.018
Liberia	Kpayan	2000	0.117	0.743	1.633
Liberia	Kpayan	2017	0.052	0.263	0.649
Liberia	Kpayan	2000-2017	-0.205	-0.085	0.028
Liberia	Lower Kru Coast	2000	0.140	0.844	1.670
Liberia	Lower Kru Coast	2000	0.095	0.668	1.323
Liberia	Lower Kru Coast	2017	0.028	0.196	0.465
Liberia	Lower Kru Coast	2017	0.129	0.310	0.645
Liberia	Lower Kru Coast	2000-2017	-0.239	-0.094	-0.001
Liberia	Lower Kru Coast	2000-2017	-0.131	-0.071	-0.024
Liberia	Mambah-Kaba	2000	0.039	0.250	0.685
Liberia	Mambah-Kaba	2017	0.008	0.095	0.323
Liberia	Mambah-Kaba	2000-2017	-0.222	-0.097	0.021
Liberia	Mecca	2000	0.091	0.481	1.034
Liberia	Mecca	2017	0.038	0.195	0.430
Liberia	Mecca	2000-2017	-0.178	-0.074	0.007
Liberia	Morweh	2000	0.108	0.606	1.477
Liberia	Morweh	2017	0.027	0.186	0.490
Liberia	Morweh	2000-2017	-0.214	-0.109	-0.015
Liberia	Owensgrove	2000	0.069	0.421	1.095
Liberia	Owensgrove	2017	0.018	0.184	0.506
Liberia	Owensgrove	2000-2017	-0.225	-0.089	0.015
Liberia	Panta-Kpa	2000	0.152	0.789	1.831
Liberia	Panta-Kpa	2017	0.043	0.274	0.650
Liberia	Panta-Kpa	2000-2017	-0.241	-0.105	0.003
Liberia	Pleebo/Sodeken	2000	0.115	0.675	1.303
Liberia	Pleebo/Sodeken	2017	0.022	0.177	0.455
Liberia	Pleebo/Sodeken	2000-2017	-0.255	-0.106	-0.017
Liberia	Porkpa	2000	0.157	0.626	1.274
Liberia	Porkpa	2017	0.083	0.291	0.608
Liberia	Porkpa	2000-2017	-0.110	-0.038	0.013
Liberia	Pyneston	2000	0.128	0.708	1.519
Liberia	Pyneston	2017	0.039	0.227	0.583
Liberia	Pyneston	2000-2017	-0.199	-0.092	0.011
Liberia	Saclepea	2000	0.144	0.723	1.560
Liberia	Saclepea	2017	0.033	0.220	0.552
Liberia	Saclepea	2000-2017	-0.257	-0.120	-0.006
Liberia	Salala	2000	0.094	0.432	0.921
Liberia	Salala	2017	0.031	0.195	0.445
Liberia	Salala	2000-2017	-0.225	-0.081	0.031
Liberia	Salayea	2000	0.206	0.777	1.681
Liberia	Salayea	2017	0.055	0.250	0.587
Liberia	Salayea	2000-2017	-0.214	-0.099	-0.008
Liberia	Sanayea	2000	0.117	0.571	1.181
Liberia	Sanayea	2017	0.040	0.203	0.457
Liberia	Sanayea	2000-2017	-0.211	-0.092	0.010
Liberia	Sanniquelleh-Mahn	2000	0.174	0.648	1.570

Table 3: Malaria DALYs rate by unit *(continued)*

Country	Unit	year	mean rate	lower rate	upper rate
Liberia	Sanniquelleh-Mahn	2017	0.024	0.173	0.465
Liberia	Sanniquelleh-Mahn	2000-2017	-0.264	-0.131	-0.027
Liberia	Sasstown	2000	0.088	0.699	1.561
Liberia	Sasstown	2017	0.048	0.249	0.630
Liberia	Sasstown	2000-2017	-0.184	-0.075	0.017
Liberia	St Paul River	2000	0.029	0.201	0.572
Liberia	St Paul River	2017	0.008	0.081	0.289
Liberia	St Paul River	2000-2017	-0.244	-0.090	0.030
Liberia	Stjohnriver	2000	0.080	0.443	1.187
Liberia	Stjohnriver	2017	0.019	0.171	0.516
Liberia	Stjohnriver	2000-2017	-0.230	-0.103	0.018
Liberia	Suakoko	2000	0.102	0.572	1.325
Liberia	Suakoko	2017	0.041	0.213	0.513
Liberia	Suakoko	2000-2017	-0.210	-0.099	0.000
Liberia	Tappita	2000	0.180	0.655	1.323
Liberia	Tappita	2017	0.024	0.190	0.492
Liberia	Tappita	2000-2017	-0.249	-0.124	-0.024
Liberia	Tchien	2000	0.226	0.846	1.671
Liberia	Tchien	2017	0.039	0.214	0.523
Liberia	Tchien	2000-2017	-0.237	-0.117	-0.024
Liberia	Tewor	2000	0.121	0.539	1.148
Liberia	Tewor	2017	0.067	0.243	0.557
Liberia	Tewor	2000-2017	-0.109	-0.041	0.037
Liberia	Timbo	2000	0.093	0.588	1.365
Liberia	Timbo	2017	0.019	0.179	0.485
Liberia	Timbo	2000-2017	-0.214	-0.105	0.002
Liberia	Todee	2000	0.101	0.441	0.982
Liberia	Todee	2017	0.029	0.197	0.506
Liberia	Todee	2000-2017	-0.185	-0.072	0.003
Liberia	Upperkrucoast	2000	0.060	0.630	1.415
Liberia	Upperkrucoast	2017	0.030	0.213	0.575
Liberia	Upperkrucoast	2000-2017	-0.201	-0.081	0.012
Liberia	Voinjama	2000	0.121	0.646	1.445
Liberia	Voinjama	2017	0.054	0.182	0.416
Liberia	Voinjama	2000-2017	-0.180	-0.094	-0.017
Liberia	Webbo	2000	0.272	0.898	1.819
Liberia	Webbo	2017	0.035	0.234	0.602
Liberia	Webbo	2000-2017	-0.249	-0.098	-0.012
Liberia	Yarwein-Mehnsohnne	2000	0.142	0.633	1.397
Liberia	Yarwein-Mehnsohnne	2017	0.029	0.214	0.500
Liberia	Yarwein-Mehnsohnne	2000-2017	-0.233	-0.114	-0.020
Liberia	Zoegeh	2000	0.193	0.694	1.462
Liberia	Zoegeh	2017	0.026	0.205	0.545
Liberia	Zoegeh	2000-2017	-0.261	-0.122	-0.021
Liberia	Zorzor	2000	0.196	0.715	1.509
Liberia	Zorzor	2017	0.059	0.200	0.473
Liberia	Zorzor	2000-2017	-0.208	-0.101	-0.004
Liberia	Zota	2000	0.137	0.786	1.887
Liberia	Zota	2017	0.047	0.256	0.610
Liberia	Zota	2000-2017	-0.229	-0.106	-0.007
Madagascar	Alaotra-Mangoro	2000	0.021	0.083	0.193
Madagascar	Alaotra-Mangoro	2017	0.011	0.029	0.062
Madagascar	Alaotra-Mangoro	2000-2017	-0.180	-0.120	-0.066
Madagascar	Amoron'i mania	2000	0.004	0.013	0.030
Madagascar	Amoron'i mania	2017	0.007	0.021	0.042
Madagascar	Amoron'i mania	2000-2017	-0.095	-0.033	0.039
Madagascar	Analamanga	2000	0.002	0.007	0.016
Madagascar	Analamanga	2017	0.008	0.023	0.050
Madagascar	Analamanga	2000-2017	-0.069	-0.020	0.046
Madagascar	Analanjirofo	2000	0.124	0.318	0.659
Madagascar	Analanjirofo	2017	0.021	0.056	0.128

Table 3: Malaria DALYs rate by unit *(continued)*

Country	Unit	year	mean rate	lower rate	upper rate
Madagascar	Analanjirofo	2000-2017	-0.204	-0.132	-0.075
Madagascar	Androy	2000	0.032	0.119	0.252
Madagascar	Androy	2017	0.006	0.017	0.037
Madagascar	Androy	2000-2017	-0.195	-0.121	-0.059
Madagascar	Anosy	2000	0.035	0.114	0.259
Madagascar	Anosy	2017	0.012	0.034	0.075
Madagascar	Anosy	2000-2017	-0.166	-0.087	-0.022
Madagascar	Atsimo-Andrefana	2000	0.026	0.100	0.233
Madagascar	Atsimo-Andrefana	2017	0.034	0.092	0.182
Madagascar	Atsimo-Andrefana	2000-2017	-0.037	0.046	0.128
Madagascar	Atsimo-Atsinana	2000	0.017	0.109	0.373
Madagascar	Atsimo-Atsinana	2017	0.051	0.134	0.244
Madagascar	Atsimo-Atsinana	2000-2017	-0.081	-0.008	0.082
Madagascar	Atsinanana	2000	0.045	0.122	0.276
Madagascar	Atsinanana	2017	0.022	0.062	0.129
Madagascar	Atsinanana	2000-2017	-0.180	-0.117	-0.062
Madagascar	Betsiboka	2000	0.012	0.050	0.134
Madagascar	Betsiboka	2017	0.008	0.023	0.045
Madagascar	Betsiboka	2000-2017	-0.153	-0.079	-0.003
Madagascar	Boeny	2000	0.019	0.093	0.207
Madagascar	Boeny	2017	0.010	0.030	0.054
Madagascar	Boeny	2000-2017	-0.175	-0.086	-0.016
Madagascar	Bongolava	2000	0.009	0.028	0.060
Madagascar	Bongolava	2017	0.007	0.020	0.043
Madagascar	Bongolava	2000-2017	-0.123	-0.071	-0.027
Madagascar	Diana	2000	0.011	0.092	0.324
Madagascar	Diana	2017	0.003	0.017	0.052
Madagascar	Diana	2000-2017	-0.199	-0.117	-0.035
Madagascar	Haute matsiatra	2000	0.004	0.011	0.026
Madagascar	Haute matsiatra	2017	0.009	0.028	0.063
Madagascar	Haute matsiatra	2000-2017	-0.063	0.009	0.080
Madagascar	Ihorombe	2000	0.015	0.053	0.123
Madagascar	Ihorombe	2017	0.011	0.034	0.074
Madagascar	Ihorombe	2000-2017	-0.091	-0.023	0.058
Madagascar	Itasy	2000	0.004	0.012	0.023
Madagascar	Itasy	2017	0.009	0.026	0.050
Madagascar	Itasy	2000-2017	-0.077	-0.026	0.034
Madagascar	Melaky	2000	0.011	0.088	0.276
Madagascar	Melaky	2017	0.008	0.038	0.115
Madagascar	Melaky	2000-2017	-0.127	-0.061	0.001
Madagascar	Menabe	2000	0.052	0.169	0.340
Madagascar	Menabe	2017	0.014	0.041	0.089
Madagascar	Menabe	2000-2017	-0.142	-0.083	-0.022
Madagascar	Sava	2000	0.038	0.221	0.561
Madagascar	Sava	2017	0.008	0.035	0.092
Madagascar	Sava	2000-2017	-0.204	-0.124	-0.044
Madagascar	Sofia	2000	0.032	0.131	0.332
Madagascar	Sofia	2017	0.008	0.030	0.067
Madagascar	Sofia	2000-2017	-0.159	-0.096	-0.029
Madagascar	Vakinankaratra	2000	0.006	0.015	0.029
Madagascar	Vakinankaratra	2017	0.006	0.016	0.033
Madagascar	Vakinankaratra	2000-2017	-0.123	-0.053	0.016
Madagascar	Vatovavy Fitovinany	2000	0.011	0.057	0.127
Madagascar	Vatovavy Fitovinany	2017	0.030	0.077	0.135
Madagascar	Vatovavy Fitovinany	2000-2017	-0.102	-0.046	0.023
Malawi	Balaka	2000	0.241	0.456	0.770
Malawi	Balaka	2017	0.037	0.107	0.239
Malawi	Balaka	2000-2017	-0.166	-0.092	-0.024
Malawi	Blantyre	2000	0.160	0.322	0.601
Malawi	Blantyre	2017	0.012	0.049	0.104
Malawi	Blantyre	2000-2017	-0.192	-0.094	-0.018

Table 3: Malaria DALYs rate by unit *(continued)*

Country	Unit	year	mean rate	lower rate	upper rate
Malawi	Chikwawa	2000	0.289	0.519	0.905
Malawi	Chikwawa	2017	0.032	0.106	0.223
Malawi	Chikwawa	2000-2017	-0.142	-0.055	0.021
Malawi	Chiradzulu	2000	0.234	0.464	0.858
Malawi	Chiradzulu	2017	0.013	0.057	0.136
Malawi	Chiradzulu	2000-2017	-0.212	-0.112	-0.031
Malawi	Chitipa	2000	0.139	0.402	0.739
Malawi	Chitipa	2017	0.035	0.105	0.209
Malawi	Chitipa	2000-2017	-0.091	-0.003	0.054
Malawi	Dedza	2000	0.150	0.428	0.810
Malawi	Dedza	2017	0.046	0.119	0.289
Malawi	Dedza	2000-2017	-0.167	-0.088	-0.008
Malawi	Dowa	2000	0.255	0.509	0.856
Malawi	Dowa	2017	0.026	0.095	0.207
Malawi	Dowa	2000-2017	-0.228	-0.126	-0.049
Malawi	Karonga	2000	0.243	0.602	1.087
Malawi	Karonga	2017	0.038	0.122	0.240
Malawi	Karonga	2000-2017	-0.113	-0.037	0.036
Malawi	Kasungu	2000	0.245	0.441	0.697
Malawi	Kasungu	2017	0.034	0.103	0.222
Malawi	Kasungu	2000-2017	-0.185	-0.105	-0.044
Malawi	Likoma	2000	0.138	0.526	1.122
Malawi	Likoma	2017	0.045	0.166	0.374
Malawi	Likoma	2000-2017	-0.193	-0.073	0.016
Malawi	Lilongwe	2000	0.254	0.504	0.840
Malawi	Lilongwe	2017	0.029	0.079	0.167
Malawi	Lilongwe	2000-2017	-0.202	-0.119	-0.034
Malawi	Machinga	2000	0.272	0.488	0.822
Malawi	Machinga	2017	0.036	0.105	0.213
Malawi	Machinga	2000-2017	-0.172	-0.084	-0.017
Malawi	Mangochi	2000	0.239	0.497	0.968
Malawi	Mangochi	2017	0.045	0.139	0.310
Malawi	Mangochi	2000-2017	-0.173	-0.078	-0.011
Malawi	Mchinji	2000	0.287	0.529	0.851
Malawi	Mchinji	2017	0.029	0.085	0.165
Malawi	Mchinji	2000-2017	-0.203	-0.115	-0.048
Malawi	Mulanje	2000	0.238	0.531	1.039
Malawi	Mulanje	2017	0.022	0.089	0.204
Malawi	Mulanje	2000-2017	-0.193	-0.103	-0.042
Malawi	Mwanza	2000	0.162	0.380	0.705
Malawi	Mwanza	2017	0.025	0.103	0.240
Malawi	Mwanza	2000-2017	-0.234	-0.110	-0.038
Malawi	Mzimba	2000	0.196	0.369	0.665
Malawi	Mzimba	2017	0.035	0.100	0.212
Malawi	Mzimba	2000-2017	-0.151	-0.067	-0.006
Malawi	Neno	2000	0.199	0.454	0.793
Malawi	Neno	2017	0.031	0.113	0.260
Malawi	Neno	2000-2017	-0.203	-0.097	-0.021
Malawi	Nkhata Bay	2000	0.311	0.626	1.153
Malawi	Nkhata Bay	2017	0.045	0.137	0.271
Malawi	Nkhata Bay	2000-2017	-0.183	-0.091	-0.013
Malawi	Nkhotakota	2000	0.290	0.630	1.054
Malawi	Nkhotakota	2017	0.036	0.126	0.258
Malawi	Nkhotakota	2000-2017	-0.207	-0.113	-0.041
Malawi	Nsanje	2000	0.284	0.555	0.957
Malawi	Nsanje	2017	0.043	0.134	0.289
Malawi	Nsanje	2000-2017	-0.118	-0.045	0.025
Malawi	Ntcheu	2000	0.178	0.417	0.797
Malawi	Ntcheu	2017	0.040	0.128	0.307
Malawi	Ntcheu	2000-2017	-0.158	-0.076	-0.007
Malawi	Ntchisi	2000	0.244	0.451	0.787

Table 3: Malaria DALYs rate by unit *(continued)*

Country	Unit	year	mean rate	lower rate	upper rate
Malawi	Ntchisi	2017	0.023	0.088	0.191
Malawi	Ntchisi	2000-2017	-0.245	-0.139	-0.063
Malawi	Phalombe	2000	0.233	0.514	0.966
Malawi	Phalombe	2017	0.020	0.077	0.194
Malawi	Phalombe	2000-2017	-0.240	-0.132	-0.050
Malawi	Rumphi	2000	0.133	0.382	0.699
Malawi	Rumphi	2017	0.034	0.103	0.210
Malawi	Rumphi	2000-2017	-0.096	-0.011	0.067
Malawi	Salima	2000	0.235	0.535	0.968
Malawi	Salima	2017	0.023	0.093	0.240
Malawi	Salima	2000-2017	-0.223	-0.132	-0.040
Malawi	Thyolo	2000	0.298	0.604	1.119
Malawi	Thyolo	2017	0.029	0.099	0.220
Malawi	Thyolo	2000-2017	-0.161	-0.068	0.001
Malawi	Zomba	2000	0.272	0.500	0.834
Malawi	Zomba	2017	0.017	0.065	0.146
Malawi	Zomba	2000-2017	-0.236	-0.136	-0.052
Mali	Abeïbara	2000	0.004	0.059	0.199
Mali	Abeïbara	2017	0.003	0.043	0.173
Mali	Abeïbara	2000-2017	-0.133	0.014	0.157
Mali	Ansongo	2000	0.028	0.260	0.876
Mali	Ansongo	2017	0.047	0.323	0.924
Mali	Ansongo	2000-2017	-0.158	-0.019	0.102
Mali	Bafoulabé	2000	0.153	0.723	1.417
Mali	Bafoulabé	2017	0.188	0.575	1.279
Mali	Bafoulabé	2000-2017	-0.087	-0.006	0.078
Mali	Bamako	2000	0.075	0.214	0.466
Mali	Bamako	2017	0.029	0.068	0.203
Mali	Bamako	2000-2017	-0.165	-0.096	-0.021
Mali	Banamba	2000	0.239	0.799	1.880
Mali	Banamba	2017	0.144	0.485	1.121
Mali	Banamba	2000-2017	-0.162	-0.078	-0.003
Mali	Bandiagara	2000	0.126	0.385	0.826
Mali	Bandiagara	2017	0.397	0.722	1.200
Mali	Bandiagara	2000-2017	0.013	0.043	0.099
Mali	Bankass	2000	0.336	0.693	1.303
Mali	Bankass	2017	0.468	0.860	1.434
Mali	Bankass	2000-2017	-0.013	0.017	0.045
Mali	Barouéli	2000	0.288	0.861	1.937
Mali	Barouéli	2017	0.131	0.452	1.172
Mali	Barouéli	2000-2017	-0.179	-0.104	-0.028
Mali	Bla	2000	0.447	1.181	2.338
Mali	Bla	2017	0.209	0.720	1.806
Mali	Bla	2000-2017	-0.164	-0.076	-0.012
Mali	Bougouni	2000	0.246	0.646	1.349
Mali	Bougouni	2017	0.214	0.608	1.266
Mali	Bougouni	2000-2017	-0.100	-0.032	0.018
Mali	Bourem	2000	0.007	0.072	0.223
Mali	Bourem	2017	0.009	0.086	0.217
Mali	Bourem	2000-2017	-0.169	0.022	0.144
Mali	Diéma	2000	0.142	0.580	1.354
Mali	Diéma	2017	0.109	0.450	1.073
Mali	Diéma	2000-2017	-0.147	-0.033	0.072
Mali	Dioïla	2000	0.243	0.752	1.752
Mali	Dioïla	2017	0.250	0.643	1.441
Mali	Dioïla	2000-2017	-0.126	-0.055	0.007
Mali	Diré	2000	0.053	0.333	0.884
Mali	Diré	2017	0.071	0.448	1.005
Mali	Diré	2000-2017	-0.128	0.025	0.157
Mali	Djenné	2000	0.176	0.583	1.187
Mali	Djenné	2017	0.191	0.558	1.013

Table 3: Malaria DALYs rate by unit *(continued)*

Country	Unit	year	mean rate	lower rate	upper rate
Mali	Djenné	2000-2017	-0.090	-0.027	0.022
Mali	Douentza	2000	0.072	0.349	0.700
Mali	Douentza	2017	0.233	0.561	1.145
Mali	Douentza	2000-2017	-0.037	0.023	0.065
Mali	Gao	2000	0.015	0.158	0.593
Mali	Gao	2017	0.026	0.217	0.629
Mali	Gao	2000-2017	-0.159	0.007	0.156
Mali	Goundam	2000	0.061	0.371	0.863
Mali	Goundam	2017	0.047	0.433	1.140
Mali	Goundam	2000-2017	-0.112	0.011	0.146
Mali	Gourma-Rharous	2000	0.046	0.257	0.685
Mali	Gourma-Rharous	2017	0.110	0.330	0.771
Mali	Gourma-Rharous	2000-2017	-0.073	0.022	0.108
Mali	Kadiolo	2000	0.308	0.704	1.392
Mali	Kadiolo	2017	0.119	0.384	1.026
Mali	Kadiolo	2000-2017	-0.157	-0.079	0.002
Mali	Kangaba	2000	0.282	0.612	1.278
Mali	Kangaba	2017	0.126	0.366	0.857
Mali	Kangaba	2000-2017	-0.123	-0.050	0.017
Mali	Kati	2000	0.241	0.532	1.147
Mali	Kati	2017	0.086	0.202	0.540
Mali	Kati	2000-2017	-0.142	-0.091	-0.037
Mali	Kayes	2000	0.069	0.485	1.062
Mali	Kayes	2017	0.098	0.324	0.671
Mali	Kayes	2000-2017	-0.085	-0.003	0.069
Mali	Kéniéba	2000	0.196	0.524	1.049
Mali	Kéniéba	2017	0.117	0.315	0.664
Mali	Kéniéba	2000-2017	-0.103	-0.037	0.029
Mali	Kidal	2000	0.014	0.165	0.440
Mali	Kidal	2017	0.014	0.125	0.484
Mali	Kidal	2000-2017	-0.119	0.043	0.168
Mali	Kita	2000	0.265	0.780	1.613
Mali	Kita	2017	0.235	0.604	1.248
Mali	Kita	2000-2017	-0.096	-0.023	0.032
Mali	Kolokani	2000	0.269	0.842	2.007
Mali	Kolokani	2017	0.150	0.495	1.139
Mali	Kolokani	2000-2017	-0.175	-0.089	-0.020
Mali	Kolondiéba	2000	0.238	0.648	1.341
Mali	Kolondiéba	2017	0.179	0.484	1.028
Mali	Kolondiéba	2000-2017	-0.134	-0.053	0.013
Mali	Koro	2000	0.276	0.598	1.169
Mali	Koro	2017	0.502	0.941	1.543
Mali	Koro	2000-2017	0.006	0.026	0.059
Mali	Koulikoro	2000	0.217	0.763	1.824
Mali	Koulikoro	2017	0.102	0.296	0.675
Mali	Koulikoro	2000-2017	-0.207	-0.115	-0.031
Mali	Koutiala	2000	0.506	1.275	2.326
Mali	Koutiala	2017	0.290	0.846	1.911
Mali	Koutiala	2000-2017	-0.131	-0.056	0.002
Mali	Macina	2000	0.230	0.589	1.169
Mali	Macina	2017	0.083	0.273	0.640
Mali	Macina	2000-2017	-0.196	-0.103	-0.030
Mali	Ménaka	2000	0.013	0.083	0.314
Mali	Ménaka	2017	0.027	0.152	0.501
Mali	Ménaka	2000-2017	-0.074	0.049	0.173
Mali	Mopti	2000	0.111	0.430	0.900
Mali	Mopti	2017	0.183	0.507	0.952
Mali	Mopti	2000-2017	-0.067	0.003	0.081
Mali	Nara	2000	0.073	0.371	0.951
Mali	Nara	2017	0.106	0.394	0.958
Mali	Nara	2000-2017	-0.089	-0.018	0.035

Table 3: Malaria DALYs rate by unit *(continued)*

Country	Unit	year	mean rate	lower rate	upper rate
Mali	Niafunké	2000	0.056	0.335	0.832
Mali	Niafunké	2017	0.065	0.401	1.054
Mali	Niafunké	2000-2017	-0.095	-0.003	0.099
Mali	Niono	2000	0.199	0.476	1.023
Mali	Niono	2017	0.084	0.328	0.688
Mali	Niono	2000-2017	-0.161	-0.067	0.018
Mali	Nioro	2000	0.055	0.323	0.821
Mali	Nioro	2017	0.071	0.278	0.609
Mali	Nioro	2000-2017	-0.132	-0.006	0.085
Mali	San	2000	0.479	1.003	1.834
Mali	San	2017	0.320	0.769	1.505
Mali	San	2000-2017	-0.115	-0.040	0.005
Mali	Ségou	2000	0.272	0.602	1.227
Mali	Ségou	2017	0.106	0.310	0.694
Mali	Ségou	2000-2017	-0.180	-0.098	-0.031
Mali	Sikasso	2000	0.276	0.700	1.411
Mali	Sikasso	2017	0.280	0.653	1.363
Mali	Sikasso	2000-2017	-0.101	-0.039	0.009
Mali	Ténenkou	2000	0.146	0.561	1.142
Mali	Ténenkou	2017	0.085	0.298	0.654
Mali	Ténenkou	2000-2017	-0.156	-0.074	0.006
Mali	Tessalit	2000	0.007	0.075	0.251
Mali	Tessalit	2017	0.005	0.069	0.228
Mali	Tessalit	2000-2017	-0.121	0.020	0.144
Mali	Tin-Essako	2000	0.009	0.100	0.282
Mali	Tin-Essako	2017	0.006	0.074	0.265
Mali	Tin-Essako	2000-2017	-0.130	0.036	0.204
Mali	Tombouctou	2000	0.015	0.125	0.405
Mali	Tombouctou	2017	0.016	0.113	0.304
Mali	Tombouctou	2000-2017	-0.176	0.018	0.186
Mali	Tominian	2000	0.542	1.047	1.799
Mali	Tominian	2017	0.529	1.011	1.812
Mali	Tominian	2000-2017	-0.063	-0.009	0.014
Mali	Yanfolila	2000	0.326	0.670	1.288
Mali	Yanfolila	2017	0.122	0.512	1.008
Mali	Yanfolila	2000-2017	-0.130	-0.032	0.018
Mali	Yélimané	2000	0.033	0.336	0.848
Mali	Yélimané	2017	0.080	0.286	0.835
Mali	Yélimané	2000-2017	-0.086	0.012	0.108
Mali	Yorosso	2000	0.850	1.794	3.021
Mali	Yorosso	2017	0.747	1.676	3.105
Mali	Yorosso	2000-2017	-0.066	-0.014	0.017
Mali	Youwarou	2000	0.081	0.423	1.042
Mali	Youwarou	2017	0.095	0.406	0.998
Mali	Youwarou	2000-2017	-0.123	-0.020	0.074
Mauritania	Aïoun	2000	0.003	0.033	0.125
Mauritania	Aïoun	2017	0.002	0.028	0.105
Mauritania	Aïoun	2000-2017	-0.169	-0.023	0.137
Mauritania	Akjoujt	2000	0.000	0.000	0.000
Mauritania	Akjoujt	2017	0.000	0.000	0.000
Mauritania	Akjoujt	2000-2017	NA	NA	NA
Mauritania	Aleg	2000	0.006	0.040	0.108
Mauritania	Aleg	2017	0.003	0.017	0.061
Mauritania	Aleg	2000-2017	-0.173	-0.057	0.063
Mauritania	Amourj	2000	0.003	0.035	0.146
Mauritania	Amourj	2017	0.003	0.025	0.106
Mauritania	Amourj	2000-2017	-0.192	-0.044	0.076
Mauritania	Aoujeft	2000	0.000	0.000	0.000
Mauritania	Aoujeft	2017	0.000	0.000	0.000
Mauritania	Aoujeft	2000-2017	-0.173	0.025	0.194
Mauritania	Atar	2000	0.000	0.000	0.000

Table 3: Malaria DALYs rate by unit *(continued)*

Country	Unit	year	mean rate	lower rate	upper rate
Mauritania	Atar	2017	0.000	0.000	0.000
Mauritania	Atar	2000-2017	NA	NA	NA
Mauritania	Bababé	2000	0.003	0.027	0.091
Mauritania	Bababé	2017	0.002	0.008	0.019
Mauritania	Bababé	2000-2017	-0.170	-0.081	-0.001
Mauritania	Barkéol	2000	0.000	0.010	0.051
Mauritania	Barkéol	2017	0.001	0.013	0.062
Mauritania	Barkéol	2000-2017	-0.163	0.011	0.166
Mauritania	Bassikounou	2000	0.003	0.047	0.191
Mauritania	Bassikounou	2017	0.003	0.031	0.130
Mauritania	Bassikounou	2000-2017	-0.230	-0.064	0.074
Mauritania	Bir Moghreïn	2000	0.000	0.000	0.000
Mauritania	Bir Moghreïn	2017	0.000	0.000	0.000
Mauritania	Bir Moghreïn	2000-2017	NA	NA	NA
Mauritania	Boghé	2000	0.021	0.096	0.265
Mauritania	Boghé	2017	0.004	0.017	0.045
Mauritania	Boghé	2000-2017	-0.187	-0.102	-0.018
Mauritania	Boumdeïd	2000	0.000	0.008	0.030
Mauritania	Boumdeïd	2017	0.001	0.010	0.044
Mauritania	Boumdeïd	2000-2017	-0.189	-0.001	0.172
Mauritania	Boutilimit	2000	0.005	0.028	0.086
Mauritania	Boutilimit	2017	0.001	0.008	0.025
Mauritania	Boutilimit	2000-2017	-0.222	-0.077	0.043
Mauritania	Chinguetti	2000	0.000	0.000	0.000
Mauritania	Chinguetti	2017	0.000	0.000	0.000
Mauritania	Chinguetti	2000-2017	NA	NA	NA
Mauritania	Djiguenni	2000	0.002	0.027	0.109
Mauritania	Djiguenni	2017	0.004	0.025	0.079
Mauritania	Djiguenni	2000-2017	-0.163	-0.016	0.115
Mauritania	F'Dérik	2000	0.000	0.000	0.000
Mauritania	F'Dérik	2017	0.000	0.000	0.000
Mauritania	F'Dérik	2000-2017	NA	NA	NA
Mauritania	Guérou	2000	0.001	0.017	0.053
Mauritania	Guérou	2017	0.002	0.025	0.106
Mauritania	Guérou	2000-2017	-0.194	0.007	0.195
Mauritania	Kaédi	2000	0.002	0.015	0.073
Mauritania	Kaédi	2017	0.002	0.008	0.018
Mauritania	Kaédi	2000-2017	-0.112	-0.034	0.051
Mauritania	Kankossa	2000	0.002	0.024	0.067
Mauritania	Kankossa	2017	0.006	0.037	0.124
Mauritania	Kankossa	2000-2017	-0.089	0.026	0.146
Mauritania	Keur-Macène	2000	0.003	0.010	0.026
Mauritania	Keur-Macène	2017	0.002	0.008	0.018
Mauritania	Keur-Macène	2000-2017	-0.131	-0.059	0.012
Mauritania	Kiffa	2000	0.001	0.013	0.041
Mauritania	Kiffa	2017	0.002	0.028	0.103
Mauritania	Kiffa	2000-2017	-0.149	0.007	0.165
Mauritania	Kobenni	2000	0.002	0.028	0.088
Mauritania	Kobenni	2017	0.004	0.023	0.060
Mauritania	Kobenni	2000-2017	-0.131	-0.009	0.121
Mauritania	M'Bagne	2000	0.002	0.024	0.077
Mauritania	M'Bagne	2017	0.002	0.008	0.019
Mauritania	M'Bagne	2000-2017	-0.157	-0.074	0.014
Mauritania	M'Bout	2000	0.001	0.014	0.069
Mauritania	M'Bout	2017	0.001	0.011	0.036
Mauritania	M'Bout	2000-2017	-0.151	0.002	0.150
Mauritania	Maghama	2000	0.002	0.022	0.084
Mauritania	Maghama	2017	0.002	0.008	0.020
Mauritania	Maghama	2000-2017	-0.145	-0.058	0.063
Mauritania	Magta-Lahjar	2000	0.001	0.015	0.049
Mauritania	Magta-Lahjar	2017	0.001	0.013	0.043

Table 3: Malaria DALYs rate by unit *(continued)*

Country	Unit	year	mean rate	lower rate	upper rate
Mauritania	Magta-Lahjar	2000-2017	-0.172	-0.008	0.128
Mauritania	Méderdra	2000	0.012	0.053	0.167
Mauritania	Méderdra	2017	0.002	0.014	0.053
Mauritania	Méderdra	2000-2017	-0.216	-0.095	0.039
Mauritania	Monguel	2000	0.001	0.017	0.092
Mauritania	Monguel	2017	0.001	0.014	0.047
Mauritania	Monguel	2000-2017	-0.148	0.006	0.154
Mauritania	Moudjéria	2000	0.001	0.014	0.060
Mauritania	Moudjéria	2017	0.001	0.013	0.043
Mauritania	Moudjéria	2000-2017	-0.186	-0.011	0.160
Mauritania	Néma	2000	0.001	0.024	0.101
Mauritania	Néma	2017	0.002	0.017	0.070
Mauritania	Néma	2000-2017	-0.204	-0.037	0.109
Mauritania	Nouadhibou	2000	0.000	0.000	0.000
Mauritania	Nouadhibou	2017	0.000	0.000	0.000
Mauritania	Nouadhibou	2000-2017	NA	NA	NA
Mauritania	Nouakchott	2000	0.000	0.004	0.018
Mauritania	Nouakchott	2017	0.000	0.002	0.008
Mauritania	Nouakchott	2000-2017	-0.273	-0.096	0.110
Mauritania	Ouad-Naga	2000	0.001	0.007	0.025
Mauritania	Ouad-Naga	2017	0.000	0.002	0.008
Mauritania	Ouad-Naga	2000-2017	-0.270	-0.106	0.070
Mauritania	Ouadane	2000	0.001	0.010	0.037
Mauritania	Ouadane	2017	0.001	0.006	0.023
Mauritania	Ouadane	2000-2017	-0.188	-0.036	0.105
Mauritania	Ould Yengé	2000	0.002	0.022	0.081
Mauritania	Ould Yengé	2017	0.003	0.022	0.075
Mauritania	Ould Yengé	2000-2017	-0.131	0.014	0.140
Mauritania	R'Kiz	2000	0.034	0.120	0.298
Mauritania	R'Kiz	2017	0.003	0.016	0.056
Mauritania	R'Kiz	2000-2017	-0.219	-0.118	-0.026
Mauritania	Rosso	2000	0.009	0.030	0.065
Mauritania	Rosso	2017	0.002	0.007	0.016
Mauritania	Rosso	2000-2017	-0.173	-0.101	-0.024
Mauritania	Sélibaby	2000	0.003	0.029	0.086
Mauritania	Sélibaby	2017	0.002	0.013	0.039
Mauritania	Sélibaby	2000-2017	-0.142	-0.038	0.062
Mauritania	Tamchakett	2000	0.001	0.017	0.056
Mauritania	Tamchakett	2017	0.002	0.016	0.063
Mauritania	Tamchakett	2000-2017	-0.156	-0.014	0.146
Mauritania	Tichitt	2000	0.000	0.003	0.015
Mauritania	Tichitt	2017	0.000	0.003	0.017
Mauritania	Tichitt	2000-2017	-0.150	0.000	0.164
Mauritania	Tidjikja	2000	0.001	0.016	0.063
Mauritania	Tidjikja	2017	0.001	0.013	0.050
Mauritania	Tidjikja	2000-2017	-0.181	-0.011	0.171
Mauritania	Timbédra	2000	0.003	0.038	0.174
Mauritania	Timbédra	2017	0.003	0.028	0.097
Mauritania	Timbédra	2000-2017	-0.166	-0.030	0.094
Mauritania	Tintane	2000	0.001	0.027	0.085
Mauritania	Tintane	2017	0.004	0.029	0.089
Mauritania	Tintane	2000-2017	-0.140	0.002	0.144
Mauritania	Zouérate	2000	0.000	0.000	0.000
Mauritania	Zouérate	2017	0.000	0.000	0.000
Mauritania	Zouérate	2000-2017	NA	NA	NA
Mozambique	Alto Molocue	2000	0.362	0.696	1.166
Mozambique	Alto Molocue	2017	0.155	0.294	0.475
Mozambique	Alto Molocue	2000-2017	-0.118	-0.051	-0.031
Mozambique	Ancuabe	2000	0.356	0.725	1.258
Mozambique	Ancuabe	2017	0.056	0.163	0.349
Mozambique	Ancuabe	2000-2017	-0.158	-0.091	-0.034

Table 3: Malaria DALYs rate by unit *(continued)*

Country	Unit	year	mean rate	lower rate	upper rate
Mozambique	Angoche	2000	0.317	0.843	1.545
Mozambique	Angoche	2017	0.210	0.390	0.602
Mozambique	Angoche	2000-2017	-0.082	-0.035	0.009
Mozambique	Angónia	2000	0.194	0.560	1.038
Mozambique	Angónia	2017	0.060	0.180	0.364
Mozambique	Angónia	2000-2017	-0.156	-0.084	-0.017
Mozambique	Balama	2000	0.201	0.567	1.102
Mozambique	Balama	2017	0.058	0.158	0.317
Mozambique	Balama	2000-2017	-0.160	-0.085	-0.012
Mozambique	Barue	2000	0.111	0.345	0.680
Mozambique	Barue	2017	0.023	0.081	0.164
Mozambique	Barue	2000-2017	-0.233	-0.138	-0.062
Mozambique	Bilene	2000	0.302	0.678	1.297
Mozambique	Bilene	2017	0.025	0.081	0.179
Mozambique	Bilene	2000-2017	-0.184	-0.120	-0.046
Mozambique	Boane	2000	0.221	0.404	0.685
Mozambique	Boane	2017	0.004	0.015	0.033
Mozambique	Boane	2000-2017	-0.248	-0.156	-0.084
Mozambique	Buzi	2000	0.274	0.705	1.227
Mozambique	Buzi	2017	0.029	0.098	0.243
Mozambique	Buzi	2000-2017	-0.222	-0.137	-0.055
Mozambique	Cahora Bassa	2000	0.080	0.305	0.706
Mozambique	Cahora Bassa	2017	0.014	0.081	0.177
Mozambique	Cahora Bassa	2000-2017	-0.183	-0.074	0.002
Mozambique	Caia	2000	0.312	0.754	1.363
Mozambique	Caia	2017	0.070	0.214	0.451
Mozambique	Caia	2000-2017	-0.130	-0.057	0.019
Mozambique	Changara	2000	0.123	0.374	0.730
Mozambique	Changara	2017	0.018	0.053	0.110
Mozambique	Changara	2000-2017	-0.206	-0.125	-0.049
Mozambique	Chemba	2000	0.361	0.687	1.208
Mozambique	Chemba	2017	0.039	0.129	0.280
Mozambique	Chemba	2000-2017	-0.149	-0.076	-0.003
Mozambique	Cheringoma	2000	0.237	0.644	1.214
Mozambique	Cheringoma	2017	0.054	0.187	0.409
Mozambique	Cheringoma	2000-2017	-0.140	-0.063	0.001
Mozambique	Chibabava	2000	0.208	0.597	1.141
Mozambique	Chibabava	2017	0.026	0.093	0.226
Mozambique	Chibabava	2000-2017	-0.217	-0.131	-0.048
Mozambique	Chibuto	2000	0.222	0.604	1.180
Mozambique	Chibuto	2017	0.025	0.080	0.206
Mozambique	Chibuto	2000-2017	-0.198	-0.119	-0.038
Mozambique	Chicualacuala	2000	0.056	0.239	0.511
Mozambique	Chicualacuala	2017	0.008	0.048	0.150
Mozambique	Chicualacuala	2000-2017	-0.258	-0.127	-0.005
Mozambique	Chifunde	2000	0.343	0.641	1.072
Mozambique	Chifunde	2017	0.032	0.092	0.204
Mozambique	Chifunde	2000-2017	-0.215	-0.147	-0.076
Mozambique	Chigubo	2000	0.063	0.317	0.679
Mozambique	Chigubo	2017	0.011	0.044	0.112
Mozambique	Chigubo	2000-2017	-0.255	-0.146	-0.059
Mozambique	Chinde	2000	0.376	0.895	1.528
Mozambique	Chinde	2017	0.097	0.294	0.560
Mozambique	Chinde	2000-2017	-0.133	-0.049	0.014
Mozambique	Chiúre	2000	0.352	0.731	1.250
Mozambique	Chiúre	2017	0.087	0.223	0.441
Mozambique	Chiúre	2000-2017	-0.136	-0.072	-0.029
Mozambique	Chiuta	2000	0.163	0.477	1.000
Mozambique	Chiuta	2017	0.041	0.112	0.250
Mozambique	Chiuta	2000-2017	-0.191	-0.102	-0.024
Mozambique	Chókwè	2000	0.359	0.675	1.265

Table 3: Malaria DALYs rate by unit *(continued)*

Country	Unit	year	mean rate	lower rate	upper rate
Mozambique	Chókwè	2017	0.018	0.058	0.135
Mozambique	Chókwè	2000-2017	-0.230	-0.155	-0.082
Mozambique	Cidade de Matola	2000	0.191	0.358	0.591
Mozambique	Cidade de Matola	2017	0.003	0.012	0.024
Mozambique	Cidade de Matola	2000-2017	-0.257	-0.169	-0.099
Mozambique	Cuamba	2000	0.276	0.575	1.018
Mozambique	Cuamba	2017	0.053	0.134	0.279
Mozambique	Cuamba	2000-2017	-0.152	-0.086	-0.021
Mozambique	Dondo	2000	0.136	0.522	0.928
Mozambique	Dondo	2017	0.013	0.053	0.122
Mozambique	Dondo	2000-2017	-0.232	-0.142	-0.045
Mozambique	Erati	2000	0.229	0.662	1.132
Mozambique	Erati	2017	0.136	0.269	0.471
Mozambique	Erati	2000-2017	-0.100	-0.050	-0.017
Mozambique	Funhalouro	2000	0.084	0.382	0.813
Mozambique	Funhalouro	2017	0.016	0.067	0.176
Mozambique	Funhalouro	2000-2017	-0.237	-0.129	-0.052
Mozambique	Gile	2000	0.395	0.733	1.237
Mozambique	Gile	2017	0.202	0.334	0.516
Mozambique	Gile	2000-2017	-0.069	-0.045	-0.032
Mozambique	Gondola	2000	0.062	0.249	0.610
Mozambique	Gondola	2017	0.027	0.082	0.194
Mozambique	Gondola	2000-2017	-0.203	-0.116	-0.039
Mozambique	Gorongosa	2000	0.155	0.533	1.088
Mozambique	Gorongosa	2017	0.027	0.091	0.194
Mozambique	Gorongosa	2000-2017	-0.248	-0.156	-0.081
Mozambique	Govuro	2000	0.390	0.813	1.434
Mozambique	Govuro	2017	0.020	0.089	0.221
Mozambique	Govuro	2000-2017	-0.260	-0.154	-0.067
Mozambique	Guijá	2000	0.259	0.629	1.181
Mozambique	Guijá	2017	0.012	0.043	0.092
Mozambique	Guijá	2000-2017	-0.270	-0.182	-0.099
Mozambique	Guro	2000	0.247	0.546	1.024
Mozambique	Guro	2017	0.022	0.070	0.150
Mozambique	Guro	2000-2017	-0.228	-0.146	-0.059
Mozambique	Gurue	2000	0.327	0.673	1.155
Mozambique	Gurue	2017	0.133	0.266	0.467
Mozambique	Gurue	2000-2017	-0.104	-0.053	-0.017
Mozambique	Homoine	2000	0.114	0.541	1.182
Mozambique	Homoine	2017	0.038	0.128	0.251
Mozambique	Homoine	2000-2017	-0.185	-0.117	-0.033
Mozambique	Ile	2000	0.396	0.718	1.173
Mozambique	Ile	2017	0.154	0.301	0.455
Mozambique	Ile	2000-2017	-0.088	-0.048	-0.026
Mozambique	Inharrime	2000	0.171	0.609	1.355
Mozambique	Inharrime	2017	0.039	0.148	0.301
Mozambique	Inharrime	2000-2017	-0.198	-0.115	-0.038
Mozambique	Inhassoro	2000	0.249	0.673	1.351
Mozambique	Inhassoro	2017	0.014	0.067	0.166
Mozambique	Inhassoro	2000-2017	-0.260	-0.155	-0.063
Mozambique	Inhassunge	2000	0.405	0.754	1.312
Mozambique	Inhassunge	2017	0.037	0.160	0.386
Mozambique	Inhassunge	2000-2017	-0.165	-0.064	0.023
Mozambique	Jangamo	2000	0.137	0.602	1.296
Mozambique	Jangamo	2017	0.037	0.116	0.256
Mozambique	Jangamo	2000-2017	-0.208	-0.132	-0.050
Mozambique	Lago	2000	0.178	0.541	0.983
Mozambique	Lago	2017	0.027	0.093	0.187
Mozambique	Lago	2000-2017	-0.220	-0.120	-0.040
Mozambique	Lalaua	2000	0.258	0.561	1.005
Mozambique	Lalaua	2017	0.101	0.246	0.430

Table 3: Malaria DALYs rate by unit *(continued)*

Country	Unit	year	mean rate	lower rate	upper rate
Mozambique	Lalaua	2000-2017	-0.116	-0.047	0.006
Mozambique	Lichinga	2000	0.184	0.509	0.953
Mozambique	Lichinga	2017	0.019	0.063	0.124
Mozambique	Lichinga	2000-2017	-0.233	-0.145	-0.080
Mozambique	Lugela	2000	0.383	0.701	1.169
Mozambique	Lugela	2017	0.140	0.264	0.447
Mozambique	Lugela	2000-2017	-0.110	-0.060	-0.028
Mozambique	Mabalane	2000	0.089	0.339	0.662
Mozambique	Mabalane	2017	0.007	0.027	0.071
Mozambique	Mabalane	2000-2017	-0.295	-0.190	-0.083
Mozambique	Mabote	2000	0.129	0.384	0.805
Mozambique	Mabote	2017	0.008	0.044	0.111
Mozambique	Mabote	2000-2017	-0.279	-0.136	-0.051
Mozambique	Macanga	2000	0.207	0.533	1.023
Mozambique	Macanga	2017	0.041	0.117	0.245
Mozambique	Macanga	2000-2017	-0.183	-0.113	-0.028
Mozambique	Machanga	2000	0.370	0.798	1.401
Mozambique	Machanga	2017	0.021	0.099	0.251
Mozambique	Machanga	2000-2017	-0.253	-0.146	-0.055
Mozambique	Machaze	2000	0.124	0.467	0.940
Mozambique	Machaze	2017	0.018	0.070	0.168
Mozambique	Machaze	2000-2017	-0.230	-0.124	-0.026
Mozambique	Macomia	2000	0.369	0.799	1.443
Mozambique	Macomia	2017	0.036	0.120	0.290
Mozambique	Macomia	2000-2017	-0.225	-0.138	-0.048
Mozambique	Macossa	2000	0.198	0.513	1.054
Mozambique	Macossa	2017	0.027	0.089	0.184
Mozambique	Macossa	2000-2017	-0.235	-0.137	-0.061
Mozambique	Maganja da Costa	2000	0.390	0.716	1.223
Mozambique	Maganja da Costa	2017	0.157	0.295	0.453
Mozambique	Maganja da Costa	2000-2017	-0.078	-0.046	-0.020
Mozambique	Magoe	2000	0.083	0.283	0.590
Mozambique	Magoe	2017	0.021	0.088	0.223
Mozambique	Magoe	2000-2017	-0.181	-0.053	0.035
Mozambique	Magude	2000	0.373	0.687	1.209
Mozambique	Magude	2017	0.022	0.081	0.176
Mozambique	Magude	2000-2017	-0.181	-0.097	-0.034
Mozambique	Majune	2000	0.158	0.506	0.893
Mozambique	Majune	2017	0.039	0.136	0.328
Mozambique	Majune	2000-2017	-0.180	-0.088	-0.005
Mozambique	Malema	2000	0.338	0.623	1.046
Mozambique	Malema	2017	0.096	0.238	0.428
Mozambique	Malema	2000-2017	-0.142	-0.067	-0.028
Mozambique	Mandimba	2000	0.307	0.542	0.968
Mozambique	Mandimba	2017	0.038	0.133	0.310
Mozambique	Mandimba	2000-2017	-0.193	-0.087	-0.023
Mozambique	Mandlakazi	2000	0.205	0.637	1.240
Mozambique	Mandlakazi	2017	0.024	0.085	0.189
Mozambique	Mandlakazi	2000-2017	-0.208	-0.129	-0.058
Mozambique	Manhiça	2000	0.332	0.689	1.310
Mozambique	Manhiça	2017	0.017	0.057	0.121
Mozambique	Manhiça	2000-2017	-0.192	-0.119	-0.055
Mozambique	Manica	2000	0.033	0.120	0.280
Mozambique	Manica	2017	0.018	0.062	0.151
Mozambique	Manica	2000-2017	-0.200	-0.114	-0.037
Mozambique	Maputo	2000	0.254	0.490	0.845
Mozambique	Maputo	2017	0.005	0.014	0.028
Mozambique	Maputo	2000-2017	-0.257	-0.172	-0.106
Mozambique	Maravia	2000	0.186	0.448	0.846
Mozambique	Maravia	2017	0.026	0.092	0.228
Mozambique	Maravia	2000-2017	-0.185	-0.106	-0.035

Table 3: Malaria DALYs rate by unit *(continued)*

Country	Unit	year	mean rate	lower rate	upper rate
Mozambique	Maringue	2000	0.192	0.568	1.086
Mozambique	Maringue	2017	0.023	0.113	0.257
Mozambique	Maringue	2000-2017	-0.215	-0.110	-0.022
Mozambique	Marracuene	2000	0.316	0.592	1.013
Mozambique	Marracuene	2017	0.007	0.024	0.045
Mozambique	Marracuene	2000-2017	-0.229	-0.154	-0.084
Mozambique	Marromeu	2000	0.350	0.838	1.414
Mozambique	Marromeu	2017	0.137	0.341	0.655
Mozambique	Marromeu	2000-2017	-0.098	-0.038	0.010
Mozambique	Marrupa	2000	0.172	0.469	0.898
Mozambique	Marrupa	2017	0.049	0.166	0.336
Mozambique	Marrupa	2000-2017	-0.129	-0.049	0.015
Mozambique	Massangena	2000	0.076	0.364	0.739
Mozambique	Massangena	2017	0.013	0.055	0.135
Mozambique	Massangena	2000-2017	-0.226	-0.115	-0.011
Mozambique	Massinga	2000	0.125	0.492	1.130
Mozambique	Massinga	2017	0.022	0.103	0.221
Mozambique	Massinga	2000-2017	-0.211	-0.123	-0.047
Mozambique	Massingir	2000	0.123	0.424	0.854
Mozambique	Massingir	2017	0.007	0.041	0.096
Mozambique	Massingir	2000-2017	-0.263	-0.161	-0.051
Mozambique	Matutuíne	2000	0.438	0.755	1.256
Mozambique	Matutuíne	2017	0.018	0.084	0.201
Mozambique	Matutuíne	2000-2017	-0.180	-0.074	0.024
Mozambique	Maúa	2000	0.167	0.465	0.817
Mozambique	Maúa	2017	0.043	0.159	0.330
Mozambique	Maúa	2000-2017	-0.158	-0.067	-0.002
Mozambique	Mavago	2000	0.096	0.432	0.846
Mozambique	Mavago	2017	0.040	0.133	0.337
Mozambique	Mavago	2000-2017	-0.168	-0.078	-0.002
Mozambique	Mecanhelas	2000	0.359	0.631	1.098
Mozambique	Mecanhelas	2017	0.042	0.118	0.245
Mozambique	Mecanhelas	2000-2017	-0.170	-0.100	-0.034
Mozambique	Meconta	2000	0.258	0.656	1.149
Mozambique	Meconta	2017	0.122	0.269	0.456
Mozambique	Meconta	2000-2017	-0.105	-0.053	-0.020
Mozambique	Mecuburi	2000	0.248	0.571	1.042
Mozambique	Mecuburi	2017	0.159	0.273	0.433
Mozambique	Mecuburi	2000-2017	-0.072	-0.038	-0.011
Mozambique	Mecufi	2000	0.450	0.986	1.720
Mozambique	Mecufi	2017	0.070	0.213	0.454
Mozambique	Mecufi	2000-2017	-0.152	-0.077	-0.006
Mozambique	Mecula	2000	0.127	0.416	0.887
Mozambique	Mecula	2017	0.045	0.143	0.315
Mozambique	Mecula	2000-2017	-0.135	-0.072	-0.007
Mozambique	Meluco	2000	0.336	0.697	1.256
Mozambique	Meluco	2017	0.037	0.129	0.270
Mozambique	Meluco	2000-2017	-0.202	-0.115	-0.036
Mozambique	Memba	2000	0.250	0.720	1.234
Mozambique	Memba	2017	0.207	0.352	0.594
Mozambique	Memba	2000-2017	-0.065	-0.026	0.012
Mozambique	Metarica	2000	0.211	0.515	0.892
Mozambique	Metarica	2017	0.049	0.166	0.330
Mozambique	Metarica	2000-2017	-0.183	-0.074	-0.023
Mozambique	Milange	2000	0.335	0.660	1.257
Mozambique	Milange	2017	0.078	0.182	0.360
Mozambique	Milange	2000-2017	-0.157	-0.086	-0.032
Mozambique	Moamba	2000	0.379	0.676	1.150
Mozambique	Moamba	2017	0.012	0.051	0.117
Mozambique	Moamba	2000-2017	-0.223	-0.127	-0.060
Mozambique	Moatize	2000	0.195	0.498	0.859

Table 3: Malaria DALYs rate by unit *(continued)*

Country	Unit	year	mean rate	lower rate	upper rate
Mozambique	Moatize	2017	0.027	0.112	0.234
Mozambique	Moatize	2000-2017	-0.212	-0.114	-0.033
Mozambique	Mocimboa da Praia	2000	0.284	0.676	1.262
Mozambique	Mocimboa da Praia	2017	0.031	0.122	0.303
Mozambique	Mocimboa da Praia	2000-2017	-0.212	-0.127	-0.048
Mozambique	Mocuba	2000	0.382	0.672	1.085
Mozambique	Mocuba	2017	0.100	0.235	0.392
Mozambique	Mocuba	2000-2017	-0.108	-0.060	-0.034
Mozambique	Mogovolas	2000	0.349	0.644	1.126
Mozambique	Mogovolas	2017	0.146	0.286	0.429
Mozambique	Mogovolas	2000-2017	-0.091	-0.045	-0.024
Mozambique	Moma	2000	0.329	0.727	1.363
Mozambique	Moma	2017	0.192	0.352	0.536
Mozambique	Moma	2000-2017	-0.080	-0.037	-0.004
Mozambique	Monapo	2000	0.188	0.601	1.013
Mozambique	Monapo	2017	0.144	0.272	0.475
Mozambique	Monapo	2000-2017	-0.108	-0.047	-0.011
Mozambique	Mongincual	2000	0.360	0.816	1.462
Mozambique	Mongincual	2017	0.194	0.365	0.599
Mozambique	Mongincual	2000-2017	-0.094	-0.045	-0.015
Mozambique	Montepuez	2000	0.157	0.582	1.170
Mozambique	Montepuez	2017	0.040	0.127	0.261
Mozambique	Montepuez	2000-2017	-0.197	-0.105	-0.029
Mozambique	Mopeia	2000	0.365	0.760	1.224
Mozambique	Mopeia	2017	0.115	0.264	0.507
Mozambique	Mopeia	2000-2017	-0.096	-0.052	-0.006
Mozambique	Morrumbala	2000	0.409	0.729	1.238
Mozambique	Morrumbala	2017	0.106	0.260	0.420
Mozambique	Morrumbala	2000-2017	-0.117	-0.059	-0.025
Mozambique	Morrumbene	2000	0.115	0.493	1.058
Mozambique	Morrumbene	2017	0.031	0.122	0.253
Mozambique	Morrumbene	2000-2017	-0.213	-0.115	-0.041
Mozambique	Mossuril	2000	0.193	0.751	1.527
Mozambique	Mossuril	2017	0.115	0.312	0.585
Mozambique	Mossuril	2000-2017	-0.139	-0.052	0.002
Mozambique	Mossurize	2000	0.088	0.309	0.692
Mozambique	Mossurize	2017	0.017	0.065	0.174
Mozambique	Mossurize	2000-2017	-0.238	-0.124	-0.031
Mozambique	Muanza	2000	0.165	0.607	1.118
Mozambique	Muanza	2017	0.036	0.148	0.291
Mozambique	Muanza	2000-2017	-0.197	-0.098	-0.022
Mozambique	Muecate	2000	0.187	0.609	1.037
Mozambique	Muecate	2017	0.128	0.277	0.466
Mozambique	Muecate	2000-2017	-0.115	-0.043	0.000
Mozambique	Mueda	2000	0.360	0.728	1.350
Mozambique	Mueda	2017	0.037	0.115	0.275
Mozambique	Mueda	2000-2017	-0.193	-0.115	-0.038
Mozambique	Muembe	2000	0.170	0.540	1.011
Mozambique	Muembe	2017	0.028	0.107	0.248
Mozambique	Muembe	2000-2017	-0.206	-0.113	-0.019
Mozambique	Muidumbe	2000	0.370	0.759	1.364
Mozambique	Muidumbe	2017	0.031	0.121	0.293
Mozambique	Muidumbe	2000-2017	-0.217	-0.127	-0.048
Mozambique	Murrupula	2000	0.328	0.643	1.117
Mozambique	Murrupula	2017	0.142	0.288	0.439
Mozambique	Murrupula	2000-2017	-0.097	-0.048	-0.027
Mozambique	Mutarara	2000	0.422	0.792	1.412
Mozambique	Mutarara	2017	0.059	0.172	0.341
Mozambique	Mutarara	2000-2017	-0.132	-0.069	-0.004
Mozambique	N'gauma	2000	0.201	0.510	0.997
Mozambique	N'gauma	2017	0.029	0.089	0.192

Table 3: Malaria DALYs rate by unit *(continued)*

Country	Unit	year	mean rate	lower rate	upper rate
Mozambique	N'gauma	2000-2017	-0.222	-0.120	-0.048
Mozambique	Nacala Velha	2000	0.113	0.544	1.041
Mozambique	Nacala Velha	2017	0.111	0.239	0.400
Mozambique	Nacala Velha	2000-2017	-0.092	-0.023	0.026
Mozambique	Nacaroa	2000	0.211	0.612	0.981
Mozambique	Nacaroa	2017	0.168	0.290	0.480
Mozambique	Nacaroa	2000-2017	-0.093	-0.041	-0.013
Mozambique	Namaacha	2000	0.226	0.438	0.820
Mozambique	Namaacha	2017	0.007	0.031	0.075
Mozambique	Namaacha	2000-2017	-0.227	-0.131	-0.042
Mozambique	Namacurra	2000	0.445	0.810	1.374
Mozambique	Namacurra	2017	0.080	0.226	0.405
Mozambique	Namacurra	2000-2017	-0.137	-0.061	-0.009
Mozambique	Namarroi	2000	0.369	0.678	1.144
Mozambique	Namarroi	2017	0.134	0.258	0.439
Mozambique	Namarroi	2000-2017	-0.110	-0.058	-0.025
Mozambique	Nampula	2000	0.149	0.438	0.813
Mozambique	Nampula	2017	0.071	0.168	0.293
Mozambique	Nampula	2000-2017	-0.109	-0.050	0.001
Mozambique	Namuno	2000	0.198	0.604	1.171
Mozambique	Namuno	2017	0.069	0.185	0.346
Mozambique	Namuno	2000-2017	-0.162	-0.081	-0.020
Mozambique	Nangade	2000	0.342	0.753	1.344
Mozambique	Nangade	2017	0.042	0.128	0.320
Mozambique	Nangade	2000-2017	-0.201	-0.118	-0.048
Mozambique	Nhamatanda	2000	0.141	0.487	0.926
Mozambique	Nhamatanda	2017	0.039	0.115	0.233
Mozambique	Nhamatanda	2000-2017	-0.206	-0.125	-0.040
Mozambique	Nicoadala	2000	0.398	0.735	1.236
Mozambique	Nicoadala	2017	0.046	0.167	0.358
Mozambique	Nicoadala	2000-2017	-0.149	-0.064	0.009
Mozambique	Nipepe	2000	0.231	0.517	0.965
Mozambique	Nipepe	2017	0.068	0.199	0.351
Mozambique	Nipepe	2000-2017	-0.153	-0.055	0.012
Mozambique	Palma	2000	0.393	0.842	1.467
Mozambique	Palma	2017	0.055	0.173	0.375
Mozambique	Palma	2000-2017	-0.203	-0.124	-0.045
Mozambique	Panda	2000	0.105	0.449	1.035
Mozambique	Panda	2017	0.035	0.110	0.208
Mozambique	Panda	2000-2017	-0.198	-0.112	-0.043
Mozambique	Pebane	2000	0.348	0.749	1.346
Mozambique	Pebane	2017	0.203	0.356	0.529
Mozambique	Pebane	2000-2017	-0.069	-0.041	-0.009
Mozambique	Pemba	2000	0.403	0.850	1.496
Mozambique	Pemba	2017	0.025	0.100	0.245
Mozambique	Pemba	2000-2017	-0.215	-0.121	-0.039
Mozambique	Quissanga	2000	0.414	0.919	1.628
Mozambique	Quissanga	2017	0.042	0.138	0.402
Mozambique	Quissanga	2000-2017	-0.229	-0.129	-0.037
Mozambique	Ribaue	2000	0.280	0.631	1.128
Mozambique	Ribaue	2017	0.146	0.296	0.499
Mozambique	Ribaue	2000-2017	-0.112	-0.045	-0.018
Mozambique	Sanga	2000	0.208	0.514	1.037
Mozambique	Sanga	2017	0.025	0.084	0.191
Mozambique	Sanga	2000-2017	-0.216	-0.125	-0.044
Mozambique	Sussundenga	2000	0.076	0.288	0.589
Mozambique	Sussundenga	2017	0.037	0.111	0.277
Mozambique	Sussundenga	2000-2017	-0.205	-0.114	-0.045
Mozambique	Tambara	2000	0.349	0.689	1.233
Mozambique	Tambara	2017	0.027	0.081	0.171
Mozambique	Tambara	2000-2017	-0.225	-0.129	-0.038

Table 3: Malaria DALYs rate by unit *(continued)*

Country	Unit	year	mean rate	lower rate	upper rate
Mozambique	Tsangano	2000	0.174	0.516	0.978
Mozambique	Tsangano	2017	0.041	0.149	0.340
Mozambique	Tsangano	2000-2017	-0.182	-0.087	-0.003
Mozambique	Vilanculos	2000	0.178	0.546	1.195
Mozambique	Vilanculos	2017	0.017	0.070	0.148
Mozambique	Vilanculos	2000-2017	-0.237	-0.143	-0.050
Mozambique	Xai-Xai	2000	0.269	0.660	1.252
Mozambique	Xai-Xai	2017	0.024	0.074	0.161
Mozambique	Xai-Xai	2000-2017	-0.221	-0.139	-0.061
Mozambique	Zavala	2000	0.191	0.653	1.405
Mozambique	Zavala	2017	0.041	0.136	0.288
Mozambique	Zavala	2000-2017	-0.197	-0.111	-0.040
Mozambique	Zumbu	2000	0.096	0.386	0.815
Mozambique	Zumbu	2017	0.028	0.114	0.265
Mozambique	Zumbu	2000-2017	-0.168	-0.071	0.012
Namibia	Aminius	2000	0.000	0.000	0.000
Namibia	Aminius	2017	0.000	0.000	0.000
Namibia	Aminius	2000-2017	NA	NA	NA
Namibia	Anamulenge	2000	0.000	0.002	0.006
Namibia	Anamulenge	2017	0.000	0.001	0.003
Namibia	Anamulenge	2000-2017	-0.198	-0.076	0.029
Namibia	Arandis	2000	0.000	0.000	0.000
Namibia	Arandis	2017	0.000	0.000	0.000
Namibia	Arandis	2000-2017	NA	NA	NA
Namibia	Berseba	2000	0.000	0.000	0.000
Namibia	Berseba	2017	0.000	0.000	0.000
Namibia	Berseba	2000-2017	NA	NA	NA
Namibia	Daures	2000	0.000	0.000	0.000
Namibia	Daures	2017	0.000	0.000	0.000
Namibia	Daures	2000-2017	-0.128	0.031	0.153
Namibia	Eenhana	2000	0.000	0.003	0.008
Namibia	Eenhana	2017	0.000	0.001	0.002
Namibia	Eenhana	2000-2017	-0.170	-0.050	0.076
Namibia	Elim	2000	0.000	0.002	0.008
Namibia	Elim	2017	0.000	0.001	0.003
Namibia	Elim	2000-2017	-0.180	-0.043	0.061
Namibia	Endola	2000	0.000	0.003	0.008
Namibia	Endola	2017	0.000	0.001	0.003
Namibia	Endola	2000-2017	-0.162	-0.059	0.054
Namibia	Engela	2000	0.000	0.003	0.009
Namibia	Engela	2017	0.000	0.001	0.003
Namibia	Engela	2000-2017	-0.149	-0.069	0.020
Namibia	Engodi	2000	0.001	0.003	0.009
Namibia	Engodi	2017	0.000	0.001	0.005
Namibia	Engodi	2000-2017	-0.122	-0.003	0.125
Namibia	Epembe	2000	0.000	0.003	0.010
Namibia	Epembe	2017	0.000	0.001	0.004
Namibia	Epembe	2000-2017	-0.186	-0.030	0.088
Namibia	Epukiro	2000	0.000	0.002	0.008
Namibia	Epukiro	2017	0.000	0.001	0.004
Namibia	Epukiro	2000-2017	-0.162	0.000	0.154
Namibia	Epupa	2000	0.001	0.006	0.020
Namibia	Epupa	2017	0.000	0.002	0.009
Namibia	Epupa	2000-2017	-0.205	-0.048	0.084
Namibia	Etayi	2000	0.001	0.003	0.009
Namibia	Etayi	2017	0.000	0.001	0.004
Namibia	Etayi	2000-2017	-0.172	-0.063	0.037
Namibia	Gibeon	2000	0.000	0.000	0.000
Namibia	Gibeon	2017	0.000	0.000	0.000
Namibia	Gibeon	2000-2017	NA	NA	NA
Namibia	Gobabis	2000	0.000	0.000	0.000

Table 3: Malaria DALYs rate by unit *(continued)*

Country	Unit	year	mean rate	lower rate	upper rate
Namibia	Gobabis	2017	0.000	0.000	0.000
Namibia	Gobabis	2000-2017	-0.195	-0.020	0.133
Namibia	Grootfontein	2000	0.000	0.003	0.008
Namibia	Grootfontein	2017	0.000	0.001	0.004
Namibia	Grootfontein	2000-2017	-0.184	-0.003	0.167
Namibia	Guinas	2000	0.001	0.004	0.010
Namibia	Guinas	2017	0.000	0.002	0.006
Namibia	Guinas	2000-2017	-0.136	0.004	0.180
Namibia	Kabe	2000	0.001	0.004	0.009
Namibia	Kabe	2017	0.000	0.002	0.009
Namibia	Kabe	2000-2017	-0.181	-0.044	0.084
Namibia	Kahenge	2000	0.001	0.005	0.015
Namibia	Kahenge	2017	0.000	0.002	0.007
Namibia	Kahenge	2000-2017	-0.125	0.000	0.128
Namibia	Kalahari	2000	0.000	0.000	0.000
Namibia	Kalahari	2017	0.000	0.000	0.000
Namibia	Kalahari	2000-2017	-0.188	-0.006	0.132
Namibia	Kamanjab	2000	0.000	0.003	0.008
Namibia	Kamanjab	2017	0.000	0.001	0.004
Namibia	Kamanjab	2000-2017	-0.107	-0.013	0.114
Namibia	Kapako	2000	0.000	0.005	0.017
Namibia	Kapako	2017	0.000	0.002	0.007
Namibia	Kapako	2000-2017	-0.107	0.005	0.119
Namibia	Karas	2000	0.000	0.000	0.000
Namibia	Karas	2017	0.000	0.000	0.000
Namibia	Karas	2000-2017	NA	NA	NA
Namibia	Karibib	2000	0.000	0.000	0.000
Namibia	Karibib	2017	0.000	0.000	0.000
Namibia	Karibib	2000-2017	NA	NA	NA
Namibia	Katima Muliro Rural	2000	0.000	0.001	0.002
Namibia	Katima Muliro Rural	2017	0.000	0.001	0.002
Namibia	Katima Muliro Rural	2000-2017	-0.182	-0.036	0.110
Namibia	Katima Muliro Urban	2000	0.000	0.001	0.003
Namibia	Katima Muliro Urban	2017	0.000	0.001	0.002
Namibia	Katima Muliro Urban	2000-2017	-0.204	-0.050	0.105
Namibia	Katutura Central	2000	0.000	0.000	0.000
Namibia	Katutura Central	2017	0.000	0.000	0.000
Namibia	Katutura Central	2000-2017	NA	NA	NA
Namibia	Katutura East	2000	0.000	0.000	0.000
Namibia	Katutura East	2017	0.000	0.000	0.000
Namibia	Katutura East	2000-2017	NA	NA	NA
Namibia	Keetmanshoop Rural	2000	0.000	0.000	0.000
Namibia	Keetmanshoop Rural	2017	0.000	0.000	0.000
Namibia	Keetmanshoop Rural	2000-2017	NA	NA	NA
Namibia	Keetmanshoop Urban	2000	0.000	0.000	0.000
Namibia	Keetmanshoop Urban	2017	0.000	0.000	0.000
Namibia	Keetmanshoop Urban	2000-2017	NA	NA	NA
Namibia	Khomasdal North	2000	0.000	0.000	0.000
Namibia	Khomasdal North	2017	0.000	0.000	0.000
Namibia	Khomasdal North	2000-2017	NA	NA	NA
Namibia	Khorixas	2000	0.000	0.000	0.000
Namibia	Khorixas	2017	0.000	0.000	0.000
Namibia	Khorixas	2000-2017	-0.156	-0.015	0.126
Namibia	Kongola	2000	0.001	0.004	0.010
Namibia	Kongola	2017	0.000	0.001	0.003
Namibia	Kongola	2000-2017	-0.192	-0.056	0.081
Namibia	Linyandi	2000	0.000	0.001	0.004
Namibia	Linyandi	2017	0.000	0.000	0.002
Namibia	Linyandi	2000-2017	-0.206	-0.033	0.115
Namibia	Luderitz	2000	0.000	0.000	0.000
Namibia	Luderitz	2017	0.000	0.000	0.000

Table 3: Malaria DALYs rate by unit *(continued)*

Country	Unit	year	mean rate	lower rate	upper rate
Namibia	Luderitz	2000-2017	NA	NA	NA
Namibia	Mariental Rural	2000	0.000	0.000	0.000
Namibia	Mariental Rural	2017	0.000	0.000	0.000
Namibia	Mariental Rural	2000-2017	NA	NA	NA
Namibia	Mariental Urban	2000	0.000	0.000	0.000
Namibia	Mariental Urban	2017	0.000	0.000	0.000
Namibia	Mariental Urban	2000-2017	NA	NA	NA
Namibia	Mashare	2000	0.001	0.006	0.021
Namibia	Mashare	2017	0.001	0.004	0.011
Namibia	Mashare	2000-2017	-0.075	0.034	0.129
Namibia	Moses Garoeb	2000	0.000	0.000	0.000
Namibia	Moses Garoeb	2017	0.000	0.000	0.000
Namibia	Moses Garoeb	2000-2017	NA	NA	NA
Namibia	Mpungu	2000	0.001	0.006	0.020
Namibia	Mpungu	2017	0.001	0.003	0.009
Namibia	Mpungu	2000-2017	-0.131	-0.015	0.125
Namibia	Mukwe	2000	0.001	0.007	0.016
Namibia	Mukwe	2017	0.001	0.003	0.008
Namibia	Mukwe	2000-2017	-0.099	0.024	0.130
Namibia	Ndiyona	2000	0.001	0.007	0.020
Namibia	Ndiyona	2017	0.001	0.004	0.012
Namibia	Ndiyona	2000-2017	-0.063	0.042	0.138
Namibia	Ogongo	2000	0.000	0.002	0.006
Namibia	Ogongo	2017	0.000	0.001	0.003
Namibia	Ogongo	2000-2017	-0.214	-0.061	0.056
Namibia	Ohangwena	2000	0.000	0.003	0.009
Namibia	Ohangwena	2017	0.000	0.001	0.002
Namibia	Ohangwena	2000-2017	-0.157	-0.064	0.041
Namibia	Okahandja	2000	0.000	0.000	0.001
Namibia	Okahandja	2017	0.000	0.000	0.000
Namibia	Okahandja	2000-2017	-0.191	0.001	0.183
Namibia	Okahao	2000	0.000	0.002	0.006
Namibia	Okahao	2017	0.000	0.001	0.004
Namibia	Okahao	2000-2017	-0.194	-0.039	0.079
Namibia	Okakarara	2000	0.000	0.003	0.007
Namibia	Okakarara	2017	0.000	0.001	0.005
Namibia	Okakarara	2000-2017	-0.161	0.001	0.155
Namibia	Okaku	2000	0.000	0.003	0.009
Namibia	Okaku	2017	0.000	0.001	0.003
Namibia	Okaku	2000-2017	-0.169	-0.053	0.067
Namibia	Okalongo	2000	0.001	0.003	0.009
Namibia	Okalongo	2017	0.000	0.001	0.005
Namibia	Okalongo	2000-2017	-0.189	-0.073	0.029
Namibia	Okankolo	2000	0.000	0.003	0.009
Namibia	Okankolo	2017	0.000	0.001	0.004
Namibia	Okankolo	2000-2017	-0.140	-0.015	0.100
Namibia	Okatana	2000	0.000	0.002	0.007
Namibia	Okatana	2017	0.000	0.001	0.003
Namibia	Okatana	2000-2017	-0.172	-0.049	0.071
Namibia	Okatyali	2000	0.000	0.002	0.005
Namibia	Okatyali	2017	0.000	0.001	0.004
Namibia	Okatyali	2000-2017	-0.159	-0.026	0.112
Namibia	Okongo	2000	0.001	0.004	0.012
Namibia	Okongo	2017	0.000	0.002	0.006
Namibia	Okongo	2000-2017	-0.160	-0.018	0.128
Namibia	Olukonda	2000	0.000	0.001	0.002
Namibia	Olukonda	2017	0.000	0.000	0.001
Namibia	Olukonda	2000-2017	-0.169	-0.028	0.101
Namibia	Omaruru	2000	0.000	0.000	0.000
Namibia	Omaruru	2017	0.000	0.000	0.000
Namibia	Omaruru	2000-2017	-0.173	0.010	0.167

Table 3: Malaria DALYs rate by unit *(continued)*

Country	Unit	year	mean rate	lower rate	upper rate
Namibia	Omatako	2000	0.000	0.002	0.006
Namibia	Omatako	2017	0.000	0.001	0.003
Namibia	Omatako	2000-2017	-0.116	0.001	0.138
Namibia	Ompundja	2000	0.000	0.002	0.007
Namibia	Ompundja	2017	0.000	0.001	0.004
Namibia	Ompundja	2000-2017	-0.178	-0.033	0.110
Namibia	Omulonga	2000	0.000	0.003	0.009
Namibia	Omulonga	2017	0.000	0.001	0.003
Namibia	Omulonga	2000-2017	-0.164	-0.059	0.056
Namibia	Omundaungilo	2000	0.001	0.007	0.023
Namibia	Omundaungilo	2017	0.001	0.003	0.010
Namibia	Omundaungilo	2000-2017	-0.194	-0.060	0.037
Namibia	Omuntele	2000	0.000	0.003	0.009
Namibia	Omuntele	2017	0.000	0.001	0.005
Namibia	Omuntele	2000-2017	-0.148	-0.012	0.110
Namibia	Omuthiyagwipundi	2000	0.000	0.003	0.009
Namibia	Omuthiyagwipundi	2017	0.000	0.001	0.004
Namibia	Omuthiyagwipundi	2000-2017	-0.133	-0.005	0.122
Namibia	Onayena	2000	0.000	0.002	0.007
Namibia	Onayena	2017	0.000	0.001	0.003
Namibia	Onayena	2000-2017	-0.167	-0.029	0.088
Namibia	Ondangwa	2000	0.000	0.002	0.005
Namibia	Ondangwa	2017	0.000	0.001	0.002
Namibia	Ondangwa	2000-2017	-0.182	-0.045	0.093
Namibia	Ondobe	2000	0.000	0.004	0.011
Namibia	Ondobe	2017	0.000	0.001	0.003
Namibia	Ondobe	2000-2017	-0.171	-0.066	0.044
Namibia	Onesi	2000	0.000	0.002	0.008
Namibia	Onesi	2017	0.000	0.001	0.005
Namibia	Onesi	2000-2017	-0.178	-0.050	0.052
Namibia	Ongenga	2000	0.001	0.004	0.012
Namibia	Ongenga	2017	0.000	0.002	0.005
Namibia	Ongenga	2000-2017	-0.170	-0.071	0.019
Namibia	Ongwediva	2000	0.000	0.002	0.005
Namibia	Ongwediva	2017	0.000	0.000	0.002
Namibia	Ongwediva	2000-2017	-0.177	-0.055	0.068
Namibia	Oniipa	2000	0.000	0.002	0.007
Namibia	Oniipa	2017	0.000	0.001	0.003
Namibia	Oniipa	2000-2017	-0.158	-0.041	0.080
Namibia	Onyaanya	2000	0.000	0.003	0.009
Namibia	Onyaanya	2017	0.000	0.001	0.004
Namibia	Onyaanya	2000-2017	-0.157	-0.015	0.102
Namibia	Opuwo	2000	0.000	0.002	0.009
Namibia	Opuwo	2017	0.000	0.001	0.004
Namibia	Opuwo	2000-2017	-0.162	-0.004	0.141
Namibia	Oranjemund	2000	0.000	0.000	0.000
Namibia	Oranjemund	2017	0.000	0.000	0.000
Namibia	Oranjemund	2000-2017	NA	NA	NA
Namibia	Oshakati East	2000	0.000	0.002	0.005
Namibia	Oshakati East	2017	0.000	0.000	0.002
Namibia	Oshakati East	2000-2017	-0.190	-0.049	0.076
Namibia	Oshakati West	2000	0.000	0.001	0.004
Namibia	Oshakati West	2017	0.000	0.000	0.001
Namibia	Oshakati West	2000-2017	-0.190	-0.054	0.073
Namibia	Oshikango	2000	0.001	0.004	0.014
Namibia	Oshikango	2017	0.000	0.001	0.004
Namibia	Oshikango	2000-2017	-0.158	-0.075	0.018
Namibia	Oshikuku	2000	0.000	0.002	0.007
Namibia	Oshikuku	2017	0.000	0.001	0.003
Namibia	Oshikuku	2000-2017	-0.185	-0.053	0.047
Namibia	Otamanzi	2000	0.000	0.002	0.006

Table 3: Malaria DALYs rate by unit *(continued)*

Country	Unit	year	mean rate	lower rate	upper rate
Namibia	Otamanzi	2017	0.000	0.001	0.004
Namibia	Otamanzi	2000-2017	-0.176	-0.033	0.092
Namibia	Otavi	2000	0.000	0.003	0.008
Namibia	Otavi	2017	0.000	0.001	0.005
Namibia	Otavi	2000-2017	-0.168	0.001	0.158
Namibia	Otjinene	2000	0.000	0.002	0.008
Namibia	Otjinene	2017	0.000	0.001	0.005
Namibia	Otjinene	2000-2017	-0.186	0.001	0.157
Namibia	Otjiwarongo	2000	0.000	0.002	0.005
Namibia	Otjiwarongo	2017	0.000	0.001	0.003
Namibia	Otjiwarongo	2000-2017	-0.171	-0.017	0.140
Namibia	Otjombinde	2000	0.000	0.002	0.010
Namibia	Otjombinde	2017	0.000	0.001	0.004
Namibia	Otjombinde	2000-2017	-0.154	-0.010	0.141
Namibia	Outapi	2000	0.000	0.002	0.006
Namibia	Outapi	2017	0.000	0.001	0.004
Namibia	Outapi	2000-2017	-0.166	-0.065	0.038
Namibia	Outjo	2000	0.000	0.003	0.008
Namibia	Outjo	2017	0.000	0.001	0.005
Namibia	Outjo	2000-2017	-0.176	-0.008	0.140
Namibia	Rehoboth East	2000	0.000	0.000	0.000
Namibia	Rehoboth East	2017	0.000	0.000	0.000
Namibia	Rehoboth East	2000-2017	NA	NA	NA
Namibia	Rehoboth Rural	2000	0.000	0.000	0.000
Namibia	Rehoboth Rural	2017	0.000	0.000	0.000
Namibia	Rehoboth Rural	2000-2017	NA	NA	NA
Namibia	Rehoboth West	2000	0.000	0.000	0.000
Namibia	Rehoboth West	2017	0.000	0.000	0.000
Namibia	Rehoboth West	2000-2017	NA	NA	NA
Namibia	Ruacana	2000	0.000	0.003	0.009
Namibia	Ruacana	2017	0.000	0.002	0.005
Namibia	Ruacana	2000-2017	-0.186	-0.056	0.062
Namibia	Rundu Rural East	2000	0.000	0.003	0.010
Namibia	Rundu Rural East	2017	0.000	0.002	0.006
Namibia	Rundu Rural East	2000-2017	-0.071	0.042	0.158
Namibia	Rundu Rural West	2000	0.000	0.002	0.006
Namibia	Rundu Rural West	2017	0.000	0.001	0.003
Namibia	Rundu Rural West	2000-2017	-0.073	0.037	0.158
Namibia	Rundu Urban	2000	0.000	0.007	0.028
Namibia	Rundu Urban	2017	0.001	0.004	0.016
Namibia	Rundu Urban	2000-2017	-0.105	0.017	0.134
Namibia	Sesfontein	2000	0.000	0.002	0.007
Namibia	Sesfontein	2017	0.000	0.001	0.004
Namibia	Sesfontein	2000-2017	-0.139	-0.014	0.143
Namibia	Sibinda	2000	0.000	0.001	0.002
Namibia	Sibinda	2017	0.000	0.000	0.002
Namibia	Sibinda	2000-2017	-0.194	-0.026	0.146
Namibia	Soweto	2000	0.000	0.000	0.000
Namibia	Soweto	2017	0.000	0.000	0.000
Namibia	Soweto	2000-2017	NA	NA	NA
Namibia	Steinhausen	2000	0.000	0.002	0.006
Namibia	Steinhausen	2017	0.000	0.001	0.003
Namibia	Steinhausen	2000-2017	-0.165	0.005	0.137
Namibia	Swakopmund	2000	0.000	0.000	0.000
Namibia	Swakopmund	2017	0.000	0.000	0.000
Namibia	Swakopmund	2000-2017	NA	NA	NA
Namibia	Tobias Hainyeko	2000	0.000	0.000	0.000
Namibia	Tobias Hainyeko	2017	0.000	0.000	0.000
Namibia	Tobias Hainyeko	2000-2017	NA	NA	NA
Namibia	Tsandi	2000	0.000	0.002	0.005
Namibia	Tsandi	2017	0.000	0.001	0.004

Table 3: Malaria DALYs rate by unit *(continued)*

Country	Unit	year	mean rate	lower rate	upper rate
Namibia	Tsandi	2000-2017	-0.198	-0.049	0.081
Namibia	Tsumeb	2000	0.000	0.003	0.008
Namibia	Tsumeb	2017	0.000	0.001	0.004
Namibia	Tsumeb	2000-2017	-0.181	-0.010	0.197
Namibia	Tsumkwe	2000	0.000	0.002	0.007
Namibia	Tsumkwe	2017	0.000	0.001	0.003
Namibia	Tsumkwe	2000-2017	-0.134	0.003	0.141
Namibia	Uukwiyu	2000	0.000	0.002	0.005
Namibia	Uukwiyu	2017	0.000	0.001	0.003
Namibia	Uukwiyu	2000-2017	-0.179	-0.038	0.107
Namibia	Uuvudhiya	2000	0.000	0.002	0.005
Namibia	Uuvudhiya	2017	0.000	0.001	0.003
Namibia	Uuvudhiya	2000-2017	-0.168	-0.027	0.108
Namibia	Walvisbay Rural	2000	0.000	0.000	0.000
Namibia	Walvisbay Rural	2017	0.000	0.000	0.000
Namibia	Walvisbay Rural	2000-2017	NA	NA	NA
Namibia	Walvisbay Urban	2000	0.000	0.000	0.000
Namibia	Walvisbay Urban	2017	0.000	0.000	0.000
Namibia	Walvisbay Urban	2000-2017	NA	NA	NA
Namibia	Wanaheda	2000	0.000	0.000	0.000
Namibia	Wanaheda	2017	0.000	0.000	0.000
Namibia	Wanaheda	2000-2017	NA	NA	NA
Namibia	Windhoek East	2000	0.000	0.000	0.000
Namibia	Windhoek East	2017	0.000	0.000	0.000
Namibia	Windhoek East	2000-2017	NA	NA	NA
Namibia	Windhoek Rural	2000	0.000	0.000	0.000
Namibia	Windhoek Rural	2017	0.000	0.000	0.000
Namibia	Windhoek Rural	2000-2017	-0.194	-0.012	0.160
Namibia	Windhoek West	2000	0.000	0.000	0.000
Namibia	Windhoek West	2017	0.000	0.000	0.000
Namibia	Windhoek West	2000-2017	NA	NA	NA
Niger	Aguié	2000	0.148	0.858	2.161
Niger	Aguié	2017	0.134	0.625	1.689
Niger	Aguié	2000-2017	-0.179	-0.059	0.044
Niger	Arlit	2000	0.010	0.093	0.270
Niger	Arlit	2017	0.006	0.051	0.198
Niger	Arlit	2000-2017	-0.162	-0.027	0.105
Niger	Bilma	2000	0.000	0.000	0.000
Niger	Bilma	2017	0.000	0.000	0.000
Niger	Bilma	2000-2017	-0.141	0.003	0.131
Niger	Bkonni	2000	0.043	0.272	1.032
Niger	Bkonni	2017	0.198	0.462	0.830
Niger	Bkonni	2000-2017	-0.073	0.023	0.103
Niger	Boboye	2000	0.082	0.498	1.125
Niger	Boboye	2017	0.153	0.421	0.799
Niger	Boboye	2000-2017	-0.114	-0.030	0.045
Niger	Bouza	2000	0.085	0.487	1.318
Niger	Bouza	2017	0.079	0.487	1.131
Niger	Bouza	2000-2017	-0.126	-0.009	0.078
Niger	Dakoro	2000	0.129	0.638	1.637
Niger	Dakoro	2017	0.140	0.555	1.352
Niger	Dakoro	2000-2017	-0.147	-0.027	0.074
Niger	Diffa	2000	0.064	0.465	1.147
Niger	Diffa	2017	0.023	0.220	0.561
Niger	Diffa	2000-2017	-0.175	-0.050	0.057
Niger	Dogon-Doutchi	2000	0.090	0.425	1.001
Niger	Dogon-Doutchi	2017	0.225	0.573	1.008
Niger	Dogon-Doutchi	2000-2017	-0.070	0.008	0.079
Niger	Dosso	2000	0.083	0.469	1.067
Niger	Dosso	2017	0.203	0.506	0.892
Niger	Dosso	2000-2017	-0.078	-0.012	0.060

Table 3: Malaria DALYs rate by unit *(continued)*

Country	Unit	year	mean rate	lower rate	upper rate
Niger	Filingué	2000	0.056	0.360	0.962
Niger	Filingué	2017	0.064	0.336	0.738
Niger	Filingué	2000-2017	-0.135	-0.022	0.079
Niger	Gaya	2000	0.096	0.541	1.087
Niger	Gaya	2017	0.216	0.624	1.116
Niger	Gaya	2000-2017	-0.078	-0.008	0.067
Niger	Gouré	2000	0.035	0.276	0.757
Niger	Gouré	2017	0.019	0.167	0.444
Niger	Gouré	2000-2017	-0.156	-0.031	0.100
Niger	Groumdji	2000	0.095	0.595	1.410
Niger	Groumdji	2017	0.113	0.594	1.452
Niger	Groumdji	2000-2017	-0.170	-0.031	0.052
Niger	Illéla	2000	0.055	0.313	1.020
Niger	Illéla	2017	0.126	0.451	0.914
Niger	Illéla	2000-2017	-0.121	0.011	0.096
Niger	Keita	2000	0.043	0.279	0.813
Niger	Keita	2017	0.064	0.324	0.775
Niger	Keita	2000-2017	-0.113	-0.003	0.077
Niger	Kollo	2000	0.099	0.481	1.130
Niger	Kollo	2017	0.084	0.366	0.796
Niger	Kollo	2000-2017	-0.169	-0.047	0.045
Niger	Loga	2000	0.090	0.498	1.245
Niger	Loga	2017	0.151	0.519	0.947
Niger	Loga	2000-2017	-0.111	-0.014	0.069
Niger	Madaoua	2000	0.096	0.507	1.275
Niger	Madaoua	2017	0.140	0.589	1.286
Niger	Madaoua	2000-2017	-0.105	0.001	0.071
Niger	Madarounfa	2000	0.076	0.510	1.201
Niger	Madarounfa	2017	0.110	0.493	1.322
Niger	Madarounfa	2000-2017	-0.162	-0.041	0.041
Niger	Magaria	2000	0.073	0.638	1.525
Niger	Magaria	2017	0.108	0.401	0.845
Niger	Magaria	2000-2017	-0.140	-0.042	0.051
Niger	Maïné-Soroa	2000	0.027	0.345	0.848
Niger	Maïné-Soroa	2017	0.031	0.197	0.624
Niger	Maïné-Soroa	2000-2017	-0.158	-0.046	0.059
Niger	Matameye	2000	0.126	0.725	1.636
Niger	Matameye	2017	0.127	0.439	1.039
Niger	Matameye	2000-2017	-0.141	-0.061	0.048
Niger	Mayahi	2000	0.127	0.802	1.898
Niger	Mayahi	2017	0.107	0.597	1.484
Niger	Mayahi	2000-2017	-0.163	-0.049	0.063
Niger	Mirriah	2000	0.071	0.589	1.446
Niger	Mirriah	2017	0.065	0.372	0.880
Niger	Mirriah	2000-2017	-0.196	-0.040	0.093
Niger	N'Guigmi	2000	0.020	0.249	0.659
Niger	N'Guigmi	2017	0.010	0.103	0.301
Niger	N'Guigmi	2000-2017	-0.184	-0.037	0.098
Niger	Niamey	2000	0.034	0.222	0.558
Niger	Niamey	2017	0.025	0.181	0.478
Niger	Niamey	2000-2017	-0.189	-0.052	0.060
Niger	Ouallam	2000	0.044	0.299	0.924
Niger	Ouallam	2017	0.047	0.232	0.570
Niger	Ouallam	2000-2017	-0.165	-0.033	0.058
Niger	Say	2000	0.096	0.444	0.926
Niger	Say	2017	0.091	0.306	0.645
Niger	Say	2000-2017	-0.139	-0.050	0.014
Niger	Tahoua	2000	0.058	0.326	1.047
Niger	Tahoua	2017	0.088	0.409	0.946
Niger	Tahoua	2000-2017	-0.104	0.002	0.109
Niger	Tanout	2000	0.039	0.394	0.942

Table 3: Malaria DALYs rate by unit *(continued)*

Country	Unit	year	mean rate	lower rate	upper rate
Niger	Tanout	2017	0.027	0.285	0.730
Niger	Tanout	2000-2017	-0.199	-0.038	0.076
Niger	Tchighozerine	2000	0.024	0.151	0.417
Niger	Tchighozerine	2017	0.013	0.092	0.346
Niger	Tchighozerine	2000-2017	-0.158	-0.039	0.090
Niger	Tchin-Tabarade	2000	0.048	0.295	0.834
Niger	Tchin-Tabarade	2017	0.054	0.307	0.795
Niger	Tchin-Tabarade	2000-2017	-0.107	-0.001	0.089
Niger	Téra	2000	0.133	0.588	1.686
Niger	Téra	2017	0.085	0.382	0.978
Niger	Téra	2000-2017	-0.175	-0.056	0.043
Niger	Tessaoua	2000	0.122	0.761	1.705
Niger	Tessaoua	2017	0.096	0.513	1.240
Niger	Tessaoua	2000-2017	-0.161	-0.057	0.062
Niger	Tillabéry	2000	0.053	0.390	1.097
Niger	Tillabéry	2017	0.053	0.281	0.744
Niger	Tillabéry	2000-2017	-0.186	-0.052	0.071
Nigeria	Aba North	2000	0.266	0.527	0.915
Nigeria	Aba North	2017	0.006	0.029	0.075
Nigeria	Aba North	2000-2017	-0.365	-0.255	-0.150
Nigeria	Aba South	2000	0.262	0.497	0.841
Nigeria	Aba South	2017	0.009	0.038	0.093
Nigeria	Aba South	2000-2017	-0.338	-0.230	-0.129
Nigeria	Abadam	2000	0.040	0.347	0.977
Nigeria	Abadam	2017	0.015	0.146	0.402
Nigeria	Abadam	2000-2017	-0.187	-0.052	0.071
Nigeria	Abaji	2000	0.193	0.467	0.831
Nigeria	Abaji	2017	0.026	0.122	0.262
Nigeria	Abaji	2000-2017	-0.190	-0.090	-0.005
Nigeria	Abak	2000	0.342	0.652	1.064
Nigeria	Abak	2017	0.014	0.078	0.196
Nigeria	Abak	2000-2017	-0.305	-0.177	-0.066
Nigeria	Abakalik	2000	0.094	0.456	0.887
Nigeria	Abakalik	2017	0.040	0.164	0.390
Nigeria	Abakalik	2000-2017	-0.197	-0.101	-0.021
Nigeria	Abeokuta South	2000	0.092	0.313	0.582
Nigeria	Abeokuta South	2017	0.016	0.067	0.147
Nigeria	Abeokuta South	2000-2017	-0.192	-0.100	0.006
Nigeria	AbeokutaNorth	2000	0.288	0.674	1.194
Nigeria	AbeokutaNorth	2017	0.064	0.229	0.480
Nigeria	AbeokutaNorth	2000-2017	-0.159	-0.074	0.016
Nigeria	Abi	2000	0.397	0.872	1.655
Nigeria	Abi	2017	0.061	0.216	0.504
Nigeria	Abi	2000-2017	-0.209	-0.126	-0.048
Nigeria	Aboh-Mba	2000	0.514	0.944	1.536
Nigeria	Aboh-Mba	2017	0.023	0.098	0.218
Nigeria	Aboh-Mba	2000-2017	-0.303	-0.192	-0.097
Nigeria	Abua/Odu	2000	0.254	0.762	1.366
Nigeria	Abua/Odu	2017	0.032	0.127	0.310
Nigeria	Abua/Odu	2000-2017	-0.253	-0.145	-0.045
Nigeria	AbujaMun	2000	0.133	0.317	0.583
Nigeria	AbujaMun	2017	0.011	0.063	0.157
Nigeria	AbujaMun	2000-2017	-0.235	-0.113	-0.030
Nigeria	Adavi	2000	0.085	0.359	0.733
Nigeria	Adavi	2017	0.012	0.058	0.138
Nigeria	Adavi	2000-2017	-0.291	-0.179	-0.072
Nigeria	Ado	2000	0.172	0.520	1.023
Nigeria	Ado	2017	0.051	0.227	0.432
Nigeria	Ado	2000-2017	-0.178	-0.083	-0.021
Nigeria	Ado-Ekiti	2000	0.182	0.758	1.383
Nigeria	Ado-Ekiti	2017	0.090	0.285	0.674

Table 3: Malaria DALYs rate by unit *(continued)*

Country	Unit	year	mean rate	lower rate	upper rate
Nigeria	Ado-Ekiti	2000-2017	-0.163	-0.086	-0.007
Nigeria	AdoOdo/Ota	2000	0.087	0.291	0.597
Nigeria	AdoOdo/Ota	2017	0.027	0.097	0.204
Nigeria	AdoOdo/Ota	2000-2017	-0.136	-0.044	0.041
Nigeria	Afijio	2000	0.414	0.744	1.244
Nigeria	Afijio	2017	0.084	0.309	0.672
Nigeria	Afijio	2000-2017	-0.161	-0.079	-0.012
Nigeria	Afikpo	2000	0.392	0.819	1.516
Nigeria	Afikpo	2017	0.051	0.174	0.410
Nigeria	Afikpo	2000-2017	-0.218	-0.137	-0.056
Nigeria	AfikpoSo	2000	0.509	0.956	1.652
Nigeria	AfikpoSo	2017	0.055	0.187	0.433
Nigeria	AfikpoSo	2000-2017	-0.226	-0.146	-0.054
Nigeria	Agaie	2000	0.188	0.524	1.026
Nigeria	Agaie	2017	0.027	0.152	0.430
Nigeria	Agaie	2000-2017	-0.190	-0.087	0.004
Nigeria	Agatu	2000	0.170	0.547	1.010
Nigeria	Agatu	2017	0.041	0.199	0.472
Nigeria	Agatu	2000-2017	-0.197	-0.092	-0.002
Nigeria	Agege	2000	0.026	0.109	0.273
Nigeria	Agege	2017	0.006	0.028	0.061
Nigeria	Agege	2000-2017	-0.159	-0.070	0.025
Nigeria	Aguata	2000	0.380	0.776	1.338
Nigeria	Aguata	2017	0.021	0.099	0.237
Nigeria	Aguata	2000-2017	-0.290	-0.186	-0.090
Nigeria	Agwara	2000	0.099	0.607	1.448
Nigeria	Agwara	2017	0.425	0.735	1.150
Nigeria	Agwara	2000-2017	-0.026	0.014	0.082
Nigeria	Ahizu-Mb	2000	0.316	0.609	0.989
Nigeria	Ahizu-Mb	2017	0.016	0.069	0.142
Nigeria	Ahizu-Mb	2000-2017	-0.294	-0.184	-0.085
Nigeria	Ahoada East	2000	0.112	0.373	0.729
Nigeria	Ahoada East	2017	0.009	0.034	0.083
Nigeria	Ahoada East	2000-2017	-0.278	-0.184	-0.082
Nigeria	Ahoada West	2000	0.135	0.453	0.899
Nigeria	Ahoada West	2017	0.013	0.052	0.135
Nigeria	Ahoada West	2000-2017	-0.266	-0.162	-0.067
Nigeria	Ajaokuta	2000	0.109	0.481	0.990
Nigeria	Ajaokuta	2017	0.015	0.071	0.169
Nigeria	Ajaokuta	2000-2017	-0.290	-0.178	-0.060
Nigeria	Ajeromi/Ifelodun	2000	0.019	0.077	0.192
Nigeria	Ajeromi/Ifelodun	2017	0.006	0.026	0.055
Nigeria	Ajeromi/Ifelodun	2000-2017	-0.149	-0.060	0.062
Nigeria	Ajingi	2000	0.153	0.970	2.564
Nigeria	Ajingi	2017	0.114	0.370	0.959
Nigeria	Ajingi	2000-2017	-0.181	-0.080	0.013
Nigeria	Akamkpa	2000	0.563	1.077	1.804
Nigeria	Akamkpa	2017	0.072	0.269	0.589
Nigeria	Akamkpa	2000-2017	-0.224	-0.116	-0.028
Nigeria	Akinyele	2000	0.345	0.636	1.076
Nigeria	Akinyele	2017	0.073	0.234	0.485
Nigeria	Akinyele	2000-2017	-0.161	-0.084	-0.017
Nigeria	Akko	2000	0.090	0.639	1.563
Nigeria	Akko	2017	0.126	0.382	1.016
Nigeria	Akko	2000-2017	-0.114	-0.047	0.024
Nigeria	Akoko North-East	2000	0.203	0.630	1.148
Nigeria	Akoko North-East	2017	0.039	0.157	0.393
Nigeria	Akoko North-East	2000-2017	-0.237	-0.137	-0.036
Nigeria	Akoko South-East	2000	0.226	0.791	1.432
Nigeria	Akoko South-East	2017	0.053	0.205	0.508
Nigeria	Akoko South-East	2000-2017	-0.240	-0.139	-0.036

Table 3: Malaria DALYs rate by unit *(continued)*

Country	Unit	year	mean rate	lower rate	upper rate
Nigeria	Akoko South-West	2000	0.232	0.774	1.406
Nigeria	Akoko South-West	2017	0.066	0.238	0.571
Nigeria	Akoko South-West	2000-2017	-0.212	-0.118	-0.024
Nigeria	Akoko-Ed	2000	0.233	0.830	1.582
Nigeria	Akoko-Ed	2017	0.044	0.173	0.413
Nigeria	Akoko-Ed	2000-2017	-0.258	-0.157	-0.052
Nigeria	AkokoNorthWest	2000	0.249	0.677	1.174
Nigeria	AkokoNorthWest	2017	0.042	0.179	0.451
Nigeria	AkokoNorthWest	2000-2017	-0.238	-0.133	-0.030
Nigeria	Akpabuyo	2000	0.402	0.766	1.268
Nigeria	Akpabuyo	2017	0.050	0.171	0.405
Nigeria	Akpabuyo	2000-2017	-0.233	-0.128	-0.032
Nigeria	Akukutor	2000	0.316	0.710	1.257
Nigeria	Akukutor	2017	0.032	0.133	0.359
Nigeria	Akukutor	2000-2017	-0.248	-0.139	-0.038
Nigeria	Akure North	2000	0.186	0.781	1.429
Nigeria	Akure North	2017	0.134	0.404	0.866
Nigeria	Akure North	2000-2017	-0.153	-0.062	0.011
Nigeria	Akure South	2000	0.138	0.596	1.176
Nigeria	Akure South	2017	0.106	0.334	0.729
Nigeria	Akure South	2000-2017	-0.144	-0.052	0.033
Nigeria	Akwanga	2000	0.230	0.461	0.793
Nigeria	Akwanga	2017	0.056	0.198	0.448
Nigeria	Akwanga	2000-2017	-0.159	-0.071	0.001
Nigeria	Albasu	2000	0.208	1.142	2.629
Nigeria	Albasu	2017	0.088	0.320	0.866
Nigeria	Albasu	2000-2017	-0.217	-0.114	-0.010
Nigeria	Aleiro	2000	0.027	0.301	0.832
Nigeria	Aleiro	2017	0.111	0.320	0.583
Nigeria	Aleiro	2000-2017	-0.112	-0.018	0.060
Nigeria	Alimosho	2000	0.037	0.149	0.353
Nigeria	Alimosho	2017	0.009	0.044	0.092
Nigeria	Alimosho	2000-2017	-0.141	-0.056	0.034
Nigeria	Alkaleri	2000	0.151	0.770	1.651
Nigeria	Alkaleri	2017	0.081	0.322	0.744
Nigeria	Alkaleri	2000-2017	-0.186	-0.090	-0.016
Nigeria	Amuwo Odofin	2000	0.024	0.092	0.223
Nigeria	Amuwo Odofin	2017	0.007	0.030	0.063
Nigeria	Amuwo Odofin	2000-2017	-0.147	-0.055	0.055
Nigeria	Anambra East	2000	0.130	0.487	1.062
Nigeria	Anambra East	2017	0.016	0.063	0.155
Nigeria	Anambra East	2000-2017	-0.293	-0.194	-0.101
Nigeria	Anambra West	2000	0.229	0.775	1.763
Nigeria	Anambra West	2017	0.029	0.113	0.293
Nigeria	Anambra West	2000-2017	-0.285	-0.183	-0.078
Nigeria	Anaocha	2000	0.271	0.642	1.116
Nigeria	Anaocha	2017	0.019	0.085	0.201
Nigeria	Anaocha	2000-2017	-0.285	-0.184	-0.087
Nigeria	Andoni/O	2000	0.555	1.077	1.778
Nigeria	Andoni/O	2017	0.030	0.138	0.307
Nigeria	Andoni/O	2000-2017	-0.294	-0.166	-0.049
Nigeria	Aninri	2000	0.366	0.864	1.619
Nigeria	Aninri	2017	0.029	0.160	0.403
Nigeria	Aninri	2000-2017	-0.271	-0.155	-0.065
Nigeria	AniochaN	2000	0.232	0.784	1.759
Nigeria	AniochaN	2017	0.028	0.126	0.368
Nigeria	AniochaN	2000-2017	-0.280	-0.179	-0.091
Nigeria	AniochaS	2000	0.225	0.729	1.646
Nigeria	AniochaS	2017	0.026	0.112	0.304
Nigeria	AniochaS	2000-2017	-0.284	-0.180	-0.088
Nigeria	Anka	2000	0.080	0.642	1.466

Table 3: Malaria DALYs rate by unit *(continued)*

Country	Unit	year	mean rate	lower rate	upper rate
Nigeria	Anka	2017	0.328	0.751	1.352
Nigeria	Anka	2000-2017	-0.046	0.015	0.102
Nigeria	Ankpa	2000	0.103	0.401	0.880
Nigeria	Ankpa	2017	0.034	0.147	0.307
Nigeria	Ankpa	2000-2017	-0.197	-0.099	-0.016
Nigeria	Apa	2000	0.116	0.450	0.874
Nigeria	Apa	2017	0.041	0.179	0.443
Nigeria	Apa	2000-2017	-0.195	-0.091	-0.002
Nigeria	Apapa	2000	0.016	0.066	0.170
Nigeria	Apapa	2017	0.005	0.021	0.045
Nigeria	Apapa	2000-2017	-0.154	-0.063	0.065
Nigeria	Ardo-Kola	2000	0.065	0.540	1.181
Nigeria	Ardo-Kola	2017	0.174	0.405	0.753
Nigeria	Ardo-Kola	2000-2017	-0.126	-0.024	0.059
Nigeria	Arewa	2000	0.063	0.456	1.180
Nigeria	Arewa	2017	0.211	0.499	0.849
Nigeria	Arewa	2000-2017	-0.080	-0.010	0.070
Nigeria	Argungu	2000	0.039	0.385	1.114
Nigeria	Argungu	2017	0.171	0.443	0.771
Nigeria	Argungu	2000-2017	-0.093	-0.006	0.070
Nigeria	Arochukw	2000	0.510	0.963	1.597
Nigeria	Arochukw	2017	0.036	0.150	0.317
Nigeria	Arochukw	2000-2017	-0.240	-0.151	-0.063
Nigeria	Asa	2000	0.129	0.344	0.598
Nigeria	Asa	2017	0.026	0.128	0.304
Nigeria	Asa	2000-2017	-0.202	-0.096	-0.015
Nigeria	Asari-To	2000	0.329	0.784	1.390
Nigeria	Asari-To	2017	0.030	0.108	0.287
Nigeria	Asari-To	2000-2017	-0.264	-0.172	-0.052
Nigeria	Askira/U	2000	0.103	0.550	1.413
Nigeria	Askira/U	2017	0.065	0.322	0.758
Nigeria	Askira/U	2000-2017	-0.167	-0.044	0.065
Nigeria	Atakumosa East	2000	0.317	0.919	1.537
Nigeria	Atakumosa East	2017	0.124	0.471	0.918
Nigeria	Atakumosa East	2000-2017	-0.150	-0.063	0.000
Nigeria	Atakumosa West	2000	0.513	1.268	2.181
Nigeria	Atakumosa West	2017	0.189	0.637	1.358
Nigeria	Atakumosa West	2000-2017	-0.152	-0.065	-0.009
Nigeria	Atiba	2000	0.289	0.554	0.937
Nigeria	Atiba	2017	0.069	0.256	0.536
Nigeria	Atiba	2000-2017	-0.140	-0.064	-0.005
Nigeria	Atisbo	2000	0.178	0.550	1.024
Nigeria	Atisbo	2017	0.122	0.340	0.599
Nigeria	Atisbo	2000-2017	-0.131	-0.035	0.019
Nigeria	Augie	2000	0.030	0.388	1.095
Nigeria	Augie	2017	0.218	0.531	0.912
Nigeria	Augie	2000-2017	-0.051	0.020	0.094
Nigeria	Auyo	2000	0.033	0.556	1.318
Nigeria	Auyo	2017	0.077	0.254	0.551
Nigeria	Auyo	2000-2017	-0.143	-0.030	0.089
Nigeria	Awe	2000	0.066	0.381	0.786
Nigeria	Awe	2017	0.043	0.166	0.409
Nigeria	Awe	2000-2017	-0.179	-0.083	0.004
Nigeria	Awgu	2000	0.249	0.696	1.469
Nigeria	Awgu	2017	0.016	0.111	0.282
Nigeria	Awgu	2000-2017	-0.297	-0.172	-0.079
Nigeria	AwkaNort	2000	0.212	0.617	1.248
Nigeria	AwkaNort	2017	0.024	0.083	0.201
Nigeria	AwkaNort	2000-2017	-0.280	-0.185	-0.097
Nigeria	AwkaSout	2000	0.149	0.434	0.883
Nigeria	AwkaSout	2017	0.013	0.057	0.135

Table 3: Malaria DALYs rate by unit *(continued)*

Country	Unit	year	mean rate	lower rate	upper rate
Nigeria	AwkaSout	2000-2017	-0.283	-0.187	-0.102
Nigeria	Ayamelum	2000	0.170	0.619	1.445
Nigeria	Ayamelum	2017	0.021	0.095	0.257
Nigeria	Ayamelum	2000-2017	-0.277	-0.178	-0.070
Nigeria	Ayedaade	2000	0.574	1.203	1.971
Nigeria	Ayedaade	2017	0.159	0.538	1.054
Nigeria	Ayedaade	2000-2017	-0.170	-0.076	-0.012
Nigeria	Ayedire	2000	0.542	1.075	1.794
Nigeria	Ayedire	2017	0.150	0.446	0.915
Nigeria	Ayedire	2000-2017	-0.178	-0.086	-0.016
Nigeria	Babura	2000	0.096	0.815	1.808
Nigeria	Babura	2017	0.110	0.388	0.931
Nigeria	Babura	2000-2017	-0.181	-0.072	0.037
Nigeria	Badagary	2000	0.117	0.357	0.693
Nigeria	Badagary	2017	0.031	0.135	0.346
Nigeria	Badagary	2000-2017	-0.129	-0.025	0.066
Nigeria	Bade	2000	0.039	0.462	1.236
Nigeria	Bade	2017	0.059	0.238	0.554
Nigeria	Bade	2000-2017	-0.152	-0.056	0.057
Nigeria	Bagudo	2000	0.106	0.644	1.541
Nigeria	Bagudo	2017	0.382	0.756	1.379
Nigeria	Bagudo	2000-2017	-0.043	0.007	0.070
Nigeria	Bagwai	2000	0.182	0.964	1.906
Nigeria	Bagwai	2017	0.085	0.364	0.957
Nigeria	Bagwai	2000-2017	-0.200	-0.096	0.022
Nigeria	Bakassi	2000	0.541	1.095	1.793
Nigeria	Bakassi	2017	0.094	0.340	0.770
Nigeria	Bakassi	2000-2017	-0.205	-0.100	-0.015
Nigeria	Bakori	2000	0.262	0.969	2.189
Nigeria	Bakori	2017	0.119	0.470	1.014
Nigeria	Bakori	2000-2017	-0.178	-0.075	0.010
Nigeria	Bakura	2000	0.055	0.460	1.110
Nigeria	Bakura	2017	0.195	0.507	0.860
Nigeria	Bakura	2000-2017	-0.057	0.016	0.104
Nigeria	Balanga	2000	0.147	0.858	1.865
Nigeria	Balanga	2017	0.220	0.701	1.268
Nigeria	Balanga	2000-2017	-0.083	-0.012	0.059
Nigeria	Bali	2000	0.068	0.373	0.742
Nigeria	Bali	2017	0.090	0.253	0.521
Nigeria	Bali	2000-2017	-0.107	-0.029	0.038
Nigeria	Bama	2000	0.124	0.459	1.176
Nigeria	Bama	2017	0.036	0.222	0.694
Nigeria	Bama	2000-2017	-0.234	-0.066	0.065
Nigeria	Barkin Ladi	2000	0.373	1.002	2.006
Nigeria	Barkin Ladi	2017	0.086	0.378	0.816
Nigeria	Barkin Ladi	2000-2017	-0.218	-0.091	0.000
Nigeria	Baruten	2000	0.136	0.438	0.815
Nigeria	Baruten	2017	0.113	0.314	0.537
Nigeria	Baruten	2000-2017	-0.097	-0.026	0.021
Nigeria	Bassa	2000	0.119	0.542	1.008
Nigeria	Bassa	2000	0.468	1.083	1.993
Nigeria	Bassa	2017	0.021	0.101	0.239
Nigeria	Bassa	2017	0.095	0.357	0.779
Nigeria	Bassa	2000-2017	-0.215	-0.095	-0.020
Nigeria	Bassa	2000-2017	-0.247	-0.147	-0.052
Nigeria	Batagarawa	2000	0.058	0.376	0.966
Nigeria	Batagarawa	2017	0.056	0.212	0.601
Nigeria	Batagarawa	2000-2017	-0.192	-0.081	0.025
Nigeria	Batsari	2000	0.071	0.482	1.150
Nigeria	Batsari	2017	0.076	0.291	0.737
Nigeria	Batsari	2000-2017	-0.174	-0.057	0.039

Table 3: Malaria DALYs rate by unit *(continued)*

Country	Unit	year	mean rate	lower rate	upper rate
Nigeria	Bauchi	2000	0.129	0.771	1.579
Nigeria	Bauchi	2017	0.046	0.204	0.545
Nigeria	Bauchi	2000-2017	-0.261	-0.127	-0.038
Nigeria	Baure	2000	0.086	0.735	1.655
Nigeria	Baure	2017	0.084	0.353	0.894
Nigeria	Baure	2000-2017	-0.209	-0.076	0.043
Nigeria	Bayo	2000	0.114	0.846	1.791
Nigeria	Bayo	2017	0.172	0.689	1.367
Nigeria	Bayo	2000-2017	-0.133	-0.019	0.059
Nigeria	Bebeji	2000	0.251	1.154	2.244
Nigeria	Bebeji	2017	0.083	0.327	0.872
Nigeria	Bebeji	2000-2017	-0.203	-0.093	0.011
Nigeria	Bekwarra	2000	0.100	0.496	1.042
Nigeria	Bekwarra	2017	0.072	0.274	0.654
Nigeria	Bekwarra	2000-2017	-0.171	-0.070	0.012
Nigeria	Bende	2000	0.550	1.034	1.712
Nigeria	Bende	2017	0.037	0.165	0.343
Nigeria	Bende	2000-2017	-0.258	-0.155	-0.061
Nigeria	Biase	2000	0.561	1.062	1.781
Nigeria	Biase	2017	0.046	0.206	0.511
Nigeria	Biase	2000-2017	-0.230	-0.141	-0.042
Nigeria	Bichi	2000	0.170	0.867	1.766
Nigeria	Bichi	2017	0.085	0.333	0.902
Nigeria	Bichi	2000-2017	-0.202	-0.102	0.005
Nigeria	Bida	2000	0.065	0.247	0.600
Nigeria	Bida	2017	0.017	0.094	0.249
Nigeria	Bida	2000-2017	-0.198	-0.078	0.037
Nigeria	Billiri	2000	0.086	0.771	1.965
Nigeria	Billiri	2017	0.154	0.461	1.060
Nigeria	Billiri	2000-2017	-0.136	-0.051	0.025
Nigeria	Bindawa	2000	0.103	0.608	1.346
Nigeria	Bindawa	2017	0.071	0.305	0.821
Nigeria	Bindawa	2000-2017	-0.204	-0.091	0.011
Nigeria	Binji	2000	0.064	0.557	1.716
Nigeria	Binji	2017	0.370	0.792	1.362
Nigeria	Binji	2000-2017	-0.056	0.030	0.103
Nigeria	Biriniwa	2000	0.044	0.802	2.006
Nigeria	Biriniwa	2017	0.092	0.371	0.808
Nigeria	Biriniwa	2000-2017	-0.126	-0.015	0.091
Nigeria	Birnin-G	2000	0.286	0.883	1.605
Nigeria	Birnin-G	2017	0.189	0.515	1.012
Nigeria	Birnin-G	2000-2017	-0.116	-0.037	0.030
Nigeria	Birnin-Magaji/Kiyaw	2000	0.096	0.635	1.415
Nigeria	Birnin-Magaji/Kiyaw	2017	0.168	0.563	1.069
Nigeria	Birnin-Magaji/Kiyaw	2000-2017	-0.093	-0.014	0.065
Nigeria	BirninKe	2000	0.035	0.310	0.931
Nigeria	BirninKe	2017	0.093	0.304	0.598
Nigeria	BirninKe	2000-2017	-0.117	-0.026	0.055
Nigeria	BirninKu	2000	0.196	0.989	2.276
Nigeria	BirninKu	2017	0.064	0.276	0.794
Nigeria	BirninKu	2000-2017	-0.243	-0.125	-0.015
Nigeria	Biu	2000	0.117	0.563	1.275
Nigeria	Biu	2017	0.099	0.460	0.899
Nigeria	Biu	2000-2017	-0.150	-0.025	0.070
Nigeria	Bodinga	2000	0.027	0.255	0.745
Nigeria	Bodinga	2017	0.143	0.382	0.651
Nigeria	Bodinga	2000-2017	-0.048	0.040	0.141
Nigeria	Bogoro	2000	0.257	0.910	1.619
Nigeria	Bogoro	2017	0.118	0.432	1.012
Nigeria	Bogoro	2000-2017	-0.195	-0.078	-0.004
Nigeria	Boki	2000	0.202	0.796	1.524

Table 3: Malaria DALYs rate by unit *(continued)*

Country	Unit	year	mean rate	lower rate	upper rate
Nigeria	Boki	2017	0.140	0.484	0.941
Nigeria	Boki	2000-2017	-0.171	-0.059	0.007
Nigeria	Bokkos	2000	0.315	1.017	1.995
Nigeria	Bokkos	2017	0.114	0.442	1.058
Nigeria	Bokkos	2000-2017	-0.183	-0.080	0.006
Nigeria	Boluwaduro	2000	0.117	0.552	1.052
Nigeria	Boluwaduro	2017	0.042	0.179	0.457
Nigeria	Boluwaduro	2000-2017	-0.226	-0.107	-0.025
Nigeria	Bomadi	2000	0.171	0.774	1.663
Nigeria	Bomadi	2017	0.033	0.168	0.415
Nigeria	Bomadi	2000-2017	-0.234	-0.106	-0.020
Nigeria	Bonny	2000	0.331	0.703	1.135
Nigeria	Bonny	2017	0.021	0.095	0.216
Nigeria	Bonny	2000-2017	-0.297	-0.160	-0.045
Nigeria	Borgu	2000	0.088	0.452	0.970
Nigeria	Borgu	2017	0.218	0.422	0.694
Nigeria	Borgu	2000-2017	-0.061	-0.009	0.052
Nigeria	Boripe	2000	0.180	0.660	1.201
Nigeria	Boripe	2017	0.060	0.238	0.547
Nigeria	Boripe	2000-2017	-0.208	-0.096	-0.018
Nigeria	Borsari	2000	0.029	0.339	0.962
Nigeria	Borsari	2017	0.046	0.181	0.368
Nigeria	Borsari	2000-2017	-0.152	-0.055	0.056
Nigeria	Bosso	2000	0.112	0.440	0.783
Nigeria	Bosso	2017	0.033	0.127	0.355
Nigeria	Bosso	2000-2017	-0.176	-0.064	0.032
Nigeria	Brass	2000	0.291	0.731	1.374
Nigeria	Brass	2017	0.044	0.208	0.538
Nigeria	Brass	2000-2017	-0.204	-0.096	-0.008
Nigeria	Buji	2000	0.175	0.967	2.090
Nigeria	Buji	2017	0.088	0.319	0.855
Nigeria	Buji	2000-2017	-0.233	-0.111	0.004
Nigeria	Bukkuyum	2000	0.115	0.728	1.780
Nigeria	Bukkuyum	2017	0.375	0.862	1.508
Nigeria	Bukkuyum	2000-2017	-0.038	0.019	0.107
Nigeria	Bungudu	2000	0.099	0.724	1.589
Nigeria	Bungudu	2017	0.295	0.747	1.387
Nigeria	Bungudu	2000-2017	-0.077	-0.002	0.078
Nigeria	Bunkure	2000	0.183	1.083	2.331
Nigeria	Bunkure	2017	0.074	0.272	0.724
Nigeria	Bunkure	2000-2017	-0.224	-0.113	-0.011
Nigeria	Bunza	2000	0.066	0.511	1.274
Nigeria	Bunza	2017	0.153	0.555	1.037
Nigeria	Bunza	2000-2017	-0.122	-0.017	0.066
Nigeria	Buruku	2000	0.046	0.366	0.745
Nigeria	Buruku	2017	0.025	0.135	0.342
Nigeria	Buruku	2000-2017	-0.237	-0.102	-0.013
Nigeria	Burutu	2000	0.112	0.667	1.389
Nigeria	Burutu	2017	0.028	0.155	0.416
Nigeria	Burutu	2000-2017	-0.211	-0.096	-0.014
Nigeria	Bwari	2000	0.163	0.344	0.606
Nigeria	Bwari	2017	0.012	0.067	0.172
Nigeria	Bwari	2000-2017	-0.223	-0.115	-0.024
Nigeria	Calabar	2000	0.237	0.469	0.840
Nigeria	Calabar	2017	0.015	0.057	0.148
Nigeria	Calabar	2000-2017	-0.292	-0.177	-0.054
Nigeria	Calabar South	2000	0.529	1.023	1.685
Nigeria	Calabar South	2017	0.053	0.183	0.440
Nigeria	Calabar South	2000-2017	-0.251	-0.146	-0.035
Nigeria	Chanchaga	2000	0.059	0.305	0.538
Nigeria	Chanchaga	2017	0.014	0.071	0.191

Table 3: Malaria DALYs rate by unit *(continued)*

Country	Unit	year	mean rate	lower rate	upper rate
Nigeria	Chanchaga	2000-2017	-0.205	-0.079	0.029
Nigeria	Charanchi	2000	0.134	0.741	1.629
Nigeria	Charanchi	2017	0.094	0.360	0.944
Nigeria	Charanchi	2000-2017	-0.191	-0.085	0.012
Nigeria	Chibok	2000	0.116	0.643	1.595
Nigeria	Chibok	2017	0.074	0.389	0.863
Nigeria	Chibok	2000-2017	-0.182	-0.043	0.081
Nigeria	Chikun	2000	0.428	0.846	1.424
Nigeria	Chikun	2017	0.132	0.351	0.769
Nigeria	Chikun	2000-2017	-0.132	-0.055	0.003
Nigeria	Dala	2000	0.066	0.365	0.846
Nigeria	Dala	2017	0.025	0.104	0.261
Nigeria	Dala	2000-2017	-0.208	-0.109	0.015
Nigeria	Damaturu	2000	0.040	0.289	0.736
Nigeria	Damaturu	2017	0.040	0.176	0.402
Nigeria	Damaturu	2000-2017	-0.198	-0.056	0.056
Nigeria	Damban	2000	0.139	0.766	1.924
Nigeria	Damban	2017	0.096	0.436	1.099
Nigeria	Damban	2000-2017	-0.189	-0.048	0.049
Nigeria	Dambatta	2000	0.104	0.725	1.667
Nigeria	Dambatta	2017	0.094	0.335	0.939
Nigeria	Dambatta	2000-2017	-0.186	-0.086	0.007
Nigeria	Damboa	2000	0.145	0.575	1.509
Nigeria	Damboa	2017	0.070	0.318	0.795
Nigeria	Damboa	2000-2017	-0.185	-0.060	0.076
Nigeria	Dandi	2000	0.108	0.646	1.457
Nigeria	Dandi	2017	0.292	0.749	1.443
Nigeria	Dandi	2000-2017	-0.083	-0.005	0.063
Nigeria	Dandume	2000	0.257	1.013	2.144
Nigeria	Dandume	2017	0.153	0.501	1.103
Nigeria	Dandume	2000-2017	-0.177	-0.066	0.029
Nigeria	Dange-Shuni	2000	0.042	0.383	1.027
Nigeria	Dange-Shuni	2017	0.187	0.509	0.822
Nigeria	Dange-Shuni	2000-2017	-0.029	0.037	0.129
Nigeria	Danja	2000	0.353	1.117	2.267
Nigeria	Danja	2017	0.113	0.456	1.021
Nigeria	Danja	2000-2017	-0.187	-0.077	0.011
Nigeria	Danko Wasagu	2000	0.124	0.818	1.864
Nigeria	Danko Wasagu	2017	0.458	0.881	1.459
Nigeria	Danko Wasagu	2000-2017	-0.034	0.014	0.109
Nigeria	Danmusa	2000	0.148	0.900	1.946
Nigeria	Danmusa	2017	0.152	0.502	1.068
Nigeria	Danmusa	2000-2017	-0.159	-0.063	0.032
Nigeria	Darazo	2000	0.137	0.713	1.862
Nigeria	Darazo	2017	0.090	0.333	0.918
Nigeria	Darazo	2000-2017	-0.173	-0.072	0.031
Nigeria	Dass	2000	0.223	0.884	1.729
Nigeria	Dass	2017	0.064	0.297	0.713
Nigeria	Dass	2000-2017	-0.247	-0.115	-0.022
Nigeria	Daura	2000	0.071	0.379	1.029
Nigeria	Daura	2017	0.032	0.142	0.375
Nigeria	Daura	2000-2017	-0.223	-0.110	-0.008
Nigeria	DawakinK	2000	0.159	0.953	2.036
Nigeria	DawakinK	2017	0.071	0.267	0.693
Nigeria	DawakinK	2000-2017	-0.221	-0.108	-0.003
Nigeria	DawakinT	2000	0.170	0.942	1.928
Nigeria	DawakinT	2017	0.090	0.350	0.935
Nigeria	DawakinT	2000-2017	-0.191	-0.099	0.024
Nigeria	Degema	2000	0.309	0.659	1.101
Nigeria	Degema	2017	0.030	0.101	0.256
Nigeria	Degema	2000-2017	-0.261	-0.158	-0.045

Table 3: Malaria DALYs rate by unit *(continued)*

Country	Unit	year	mean rate	lower rate	upper rate
Nigeria	Dekina	2000	0.132	0.501	1.005
Nigeria	Dekina	2017	0.025	0.105	0.237
Nigeria	Dekina	2000-2017	-0.238	-0.146	-0.057
Nigeria	Demsa	2000	0.102	0.540	1.071
Nigeria	Demsa	2017	0.129	0.382	0.720
Nigeria	Demsa	2000-2017	-0.088	-0.004	0.066
Nigeria	Dikwa	2000	0.118	0.437	1.155
Nigeria	Dikwa	2017	0.025	0.235	0.666
Nigeria	Dikwa	2000-2017	-0.225	-0.039	0.102
Nigeria	Doguwa	2000	0.414	1.137	2.067
Nigeria	Doguwa	2017	0.051	0.243	0.700
Nigeria	Doguwa	2000-2017	-0.240	-0.127	-0.019
Nigeria	Doma	2000	0.134	0.498	0.999
Nigeria	Doma	2017	0.048	0.225	0.556
Nigeria	Doma	2000-2017	-0.176	-0.072	0.043
Nigeria	Donga	2000	0.065	0.318	0.605
Nigeria	Donga	2017	0.041	0.186	0.432
Nigeria	Donga	2000-2017	-0.164	-0.047	0.049
Nigeria	Dukku	2000	0.173	0.720	1.747
Nigeria	Dukku	2017	0.103	0.349	0.785
Nigeria	Dukku	2000-2017	-0.155	-0.063	0.032
Nigeria	Dunukofia	2000	0.153	0.464	0.935
Nigeria	Dunukofia	2017	0.015	0.054	0.137
Nigeria	Dunukofia	2000-2017	-0.288	-0.194	-0.103
Nigeria	Dutse	2000	0.145	0.790	1.963
Nigeria	Dutse	2017	0.086	0.299	0.737
Nigeria	Dutse	2000-2017	-0.187	-0.084	0.023
Nigeria	Dutsi	2000	0.110	0.607	1.409
Nigeria	Dutsi	2017	0.068	0.271	0.724
Nigeria	Dutsi	2000-2017	-0.212	-0.099	-0.008
Nigeria	Dutsin-M	2000	0.107	0.679	1.483
Nigeria	Dutsin-M	2017	0.088	0.323	0.815
Nigeria	Dutsin-M	2000-2017	-0.201	-0.080	0.032
Nigeria	Eastern Obolo	2000	0.461	0.917	1.513
Nigeria	Eastern Obolo	2017	0.025	0.115	0.278
Nigeria	Eastern Obolo	2000-2017	-0.284	-0.166	-0.060
Nigeria	Ebonyi	2000	0.101	0.361	0.668
Nigeria	Ebonyi	2017	0.032	0.139	0.325
Nigeria	Ebonyi	2000-2017	-0.195	-0.096	-0.021
Nigeria	Edati	2000	0.130	0.420	0.928
Nigeria	Edati	2017	0.045	0.174	0.393
Nigeria	Edati	2000-2017	-0.190	-0.077	0.005
Nigeria	Ede North	2000	0.202	0.705	1.201
Nigeria	Ede North	2017	0.059	0.243	0.529
Nigeria	Ede North	2000-2017	-0.208	-0.101	-0.015
Nigeria	Ede South	2000	0.399	1.064	1.826
Nigeria	Ede South	2017	0.146	0.460	0.942
Nigeria	Ede South	2000-2017	-0.181	-0.083	-0.013
Nigeria	Edu	2000	0.119	0.448	0.923
Nigeria	Edu	2017	0.038	0.166	0.384
Nigeria	Edu	2000-2017	-0.220	-0.095	-0.010
Nigeria	Efon	2000	0.222	0.923	1.713
Nigeria	Efon	2017	0.132	0.412	0.865
Nigeria	Efon	2000-2017	-0.160	-0.073	0.000
Nigeria	EgbadoNorth	2000	0.317	0.818	1.472
Nigeria	EgbadoNorth	2017	0.082	0.358	0.748
Nigeria	EgbadoNorth	2000-2017	-0.140	-0.056	0.027
Nigeria	EgbadoSouth	2000	0.230	0.658	1.247
Nigeria	EgbadoSouth	2017	0.072	0.291	0.633
Nigeria	EgbadoSouth	2000-2017	-0.127	-0.036	0.051
Nigeria	Egbeda	2000	0.372	0.675	1.157

Table 3: Malaria DALYs rate by unit *(continued)*

Country	Unit	year	mean rate	lower rate	upper rate
Nigeria	Egbeda	2017	0.059	0.196	0.423
Nigeria	Egbeda	2000-2017	-0.195	-0.105	-0.032
Nigeria	Egbedore	2000	0.162	0.557	0.947
Nigeria	Egbedore	2017	0.044	0.185	0.404
Nigeria	Egbedore	2000-2017	-0.219	-0.106	-0.019
Nigeria	Egor	2000	0.097	0.318	0.808
Nigeria	Egor	2017	0.024	0.110	0.241
Nigeria	Egor	2000-2017	-0.243	-0.127	-0.027
Nigeria	Ehime-Mb	2000	0.317	0.609	1.011
Nigeria	Ehime-Mb	2017	0.018	0.081	0.168
Nigeria	Ehime-Mb	2000-2017	-0.290	-0.173	-0.066
Nigeria	Ejigbo	2000	0.333	0.851	1.429
Nigeria	Ejigbo	2017	0.084	0.339	0.715
Nigeria	Ejigbo	2000-2017	-0.186	-0.088	-0.011
Nigeria	Ekeremor	2000	0.129	0.598	1.139
Nigeria	Ekeremor	2017	0.036	0.179	0.442
Nigeria	Ekeremor	2000-2017	-0.185	-0.072	0.028
Nigeria	Eket	2000	0.271	0.510	0.876
Nigeria	Eket	2017	0.016	0.069	0.161
Nigeria	Eket	2000-2017	-0.255	-0.162	-0.052
Nigeria	Ekiti	2000	0.091	0.462	0.911
Nigeria	Ekiti	2017	0.034	0.146	0.403
Nigeria	Ekiti	2000-2017	-0.216	-0.107	-0.014
Nigeria	EkitiEas	2000	0.293	0.767	1.366
Nigeria	EkitiEas	2017	0.057	0.215	0.538
Nigeria	EkitiEas	2000-2017	-0.223	-0.122	-0.024
Nigeria	EkitiSouth-West	2000	0.340	1.138	2.086
Nigeria	EkitiSouth-West	2017	0.168	0.551	1.145
Nigeria	EkitiSouth-West	2000-2017	-0.142	-0.063	0.007
Nigeria	EkitiWest	2000	0.208	0.828	1.535
Nigeria	EkitiWest	2017	0.124	0.380	0.787
Nigeria	EkitiWest	2000-2017	-0.153	-0.068	0.001
Nigeria	Ekwusigo	2000	0.270	0.742	1.348
Nigeria	Ekwusigo	2017	0.020	0.076	0.184
Nigeria	Ekwusigo	2000-2017	-0.296	-0.200	-0.102
Nigeria	Eleme	2000	0.195	0.379	0.666
Nigeria	Eleme	2017	0.012	0.042	0.105
Nigeria	Eleme	2000-2017	-0.290	-0.185	-0.061
Nigeria	Emuoha	2000	0.213	0.608	1.087
Nigeria	Emuoha	2017	0.019	0.065	0.171
Nigeria	Emuoha	2000-2017	-0.270	-0.180	-0.057
Nigeria	Emure/Ise/Orun	2000	0.250	0.941	1.687
Nigeria	Emure/Ise/Orun	2017	0.103	0.373	0.853
Nigeria	Emure/Ise/Orun	2000-2017	-0.176	-0.094	-0.014
Nigeria	Enugu East	2000	0.088	0.256	0.566
Nigeria	Enugu East	2017	0.009	0.054	0.127
Nigeria	Enugu East	2000-2017	-0.247	-0.139	-0.069
Nigeria	Enugu North	2000	0.039	0.134	0.321
Nigeria	Enugu North	2017	0.004	0.028	0.072
Nigeria	Enugu North	2000-2017	-0.265	-0.151	-0.072
Nigeria	EnuguSou	2000	0.035	0.134	0.325
Nigeria	EnuguSou	2017	0.004	0.026	0.066
Nigeria	EnuguSou	2000-2017	-0.274	-0.157	-0.071
Nigeria	Epe	2000	0.192	0.666	1.293
Nigeria	Epe	2017	0.034	0.134	0.351
Nigeria	Epe	2000-2017	-0.183	-0.109	-0.025
Nigeria	EsanCent	2000	0.179	0.747	1.656
Nigeria	EsanCent	2017	0.036	0.166	0.384
Nigeria	EsanCent	2000-2017	-0.273	-0.156	-0.058
Nigeria	EsanNort	2000	0.140	0.607	1.371
Nigeria	EsanNort	2017	0.022	0.108	0.276

Table 3: Malaria DALYs rate by unit *(continued)*

Country	Unit	year	mean rate	lower rate	upper rate
Nigeria	EsanNort	2000-2017	-0.293	-0.172	-0.056
Nigeria	EsanSout	2000	0.200	0.800	1.726
Nigeria	EsanSout	2017	0.030	0.137	0.359
Nigeria	EsanSout	2000-2017	-0.284	-0.172	-0.068
Nigeria	EsanWest	2000	0.216	0.821	1.827
Nigeria	EsanWest	2017	0.049	0.203	0.443
Nigeria	EsanWest	2000-2017	-0.245	-0.140	-0.057
Nigeria	Ese-Odo	2000	0.331	0.931	1.668
Nigeria	Ese-Odo	2017	0.053	0.278	0.587
Nigeria	Ese-Odo	2000-2017	-0.253	-0.129	-0.043
Nigeria	Esit Eket	2000	0.345	0.625	0.997
Nigeria	Esit Eket	2017	0.029	0.122	0.269
Nigeria	Esit Eket	2000-2017	-0.235	-0.139	-0.033
Nigeria	Essien-U	2000	0.391	0.745	1.241
Nigeria	Essien-U	2017	0.020	0.106	0.245
Nigeria	Essien-U	2000-2017	-0.275	-0.165	-0.066
Nigeria	Etche	2000	0.298	0.559	0.890
Nigeria	Etche	2017	0.014	0.049	0.114
Nigeria	Etche	2000-2017	-0.290	-0.206	-0.089
Nigeria	Ethiope West	2000	0.077	0.429	1.039
Nigeria	Ethiope West	2017	0.034	0.155	0.341
Nigeria	Ethiope West	2000-2017	-0.213	-0.096	-0.008
Nigeria	EthiopeE	2000	0.092	0.455	1.058
Nigeria	EthiopeE	2017	0.019	0.087	0.235
Nigeria	EthiopeE	2000-2017	-0.225	-0.130	-0.038
Nigeria	Eti-Osa	2000	0.025	0.091	0.214
Nigeria	Eti-Osa	2017	0.006	0.024	0.051
Nigeria	Eti-Osa	2000-2017	-0.165	-0.078	0.039
Nigeria	EtimEkpo	2000	0.398	0.753	1.255
Nigeria	EtimEkpo	2017	0.019	0.098	0.239
Nigeria	EtimEkpo	2000-2017	-0.292	-0.176	-0.070
Nigeria	Etinan	2000	0.311	0.599	0.987
Nigeria	Etinan	2017	0.016	0.076	0.172
Nigeria	Etinan	2000-2017	-0.276	-0.167	-0.052
Nigeria	Etsako Central	2000	0.180	0.676	1.500
Nigeria	Etsako Central	2017	0.023	0.103	0.287
Nigeria	Etsako Central	2000-2017	-0.288	-0.182	-0.075
Nigeria	EtsakoEa	2000	0.155	0.579	1.336
Nigeria	EtsakoEa	2017	0.021	0.089	0.226
Nigeria	EtsakoEa	2000-2017	-0.298	-0.181	-0.063
Nigeria	EtsakoWe	2000	0.182	0.632	1.364
Nigeria	EtsakoWe	2017	0.032	0.122	0.331
Nigeria	EtsakoWe	2000-2017	-0.272	-0.165	-0.058
Nigeria	Etung	2000	0.323	0.925	1.766
Nigeria	Etung	2017	0.127	0.470	1.030
Nigeria	Etung	2000-2017	-0.196	-0.072	0.013
Nigeria	Ewekoro	2000	0.320	0.752	1.386
Nigeria	Ewekoro	2017	0.063	0.229	0.474
Nigeria	Ewekoro	2000-2017	-0.150	-0.067	0.014
Nigeria	Ezeagu	2000	0.183	0.528	1.095
Nigeria	Ezeagu	2017	0.020	0.095	0.246
Nigeria	Ezeagu	2000-2017	-0.268	-0.165	-0.079
Nigeria	Ezinihit	2000	0.513	0.949	1.553
Nigeria	Ezinihit	2017	0.024	0.107	0.220
Nigeria	Ezinihit	2000-2017	-0.294	-0.183	-0.088
Nigeria	Ezza North	2000	0.147	0.533	1.068
Nigeria	Ezza North	2017	0.039	0.154	0.355
Nigeria	Ezza North	2000-2017	-0.202	-0.115	-0.038
Nigeria	Ezza South	2000	0.259	0.767	1.431
Nigeria	Ezza South	2017	0.068	0.231	0.536
Nigeria	Ezza South	2000-2017	-0.193	-0.109	-0.037

Table 3: Malaria DALYs rate by unit *(continued)*

Country	Unit	year	mean rate	lower rate	upper rate
Nigeria	Fagge	2000	0.042	0.254	0.574
Nigeria	Fagge	2017	0.017	0.074	0.187
Nigeria	Fagge	2000-2017	-0.227	-0.114	0.012
Nigeria	Fakai	2000	0.095	0.796	2.089
Nigeria	Fakai	2017	0.341	0.888	1.478
Nigeria	Fakai	2000-2017	-0.053	0.011	0.091
Nigeria	Faskari	2000	0.195	0.969	2.337
Nigeria	Faskari	2017	0.166	0.566	1.204
Nigeria	Faskari	2000-2017	-0.156	-0.059	0.037
Nigeria	Fika	2000	0.087	0.639	1.694
Nigeria	Fika	2017	0.128	0.422	0.913
Nigeria	Fika	2000-2017	-0.173	-0.041	0.068
Nigeria	Fufore	2000	0.127	0.576	1.169
Nigeria	Fufore	2017	0.107	0.370	0.717
Nigeria	Fufore	2000-2017	-0.104	-0.014	0.060
Nigeria	Funakaye	2000	0.120	0.649	1.579
Nigeria	Funakaye	2017	0.131	0.466	1.032
Nigeria	Funakaye	2000-2017	-0.133	-0.035	0.048
Nigeria	Fune	2000	0.048	0.446	1.117
Nigeria	Fune	2017	0.070	0.301	0.659
Nigeria	Fune	2000-2017	-0.156	-0.045	0.057
Nigeria	Funtua	2000	0.207	0.770	1.668
Nigeria	Funtua	2017	0.110	0.374	0.807
Nigeria	Funtua	2000-2017	-0.177	-0.071	0.017
Nigeria	Gabasawa	2000	0.124	0.791	2.063
Nigeria	Gabasawa	2017	0.100	0.326	0.827
Nigeria	Gabasawa	2000-2017	-0.185	-0.079	0.023
Nigeria	Gada	2000	0.034	0.303	1.181
Nigeria	Gada	2017	0.148	0.405	0.721
Nigeria	Gada	2000-2017	-0.080	0.023	0.122
Nigeria	Gagarawa	2000	0.101	0.937	2.198
Nigeria	Gagarawa	2017	0.108	0.440	0.931
Nigeria	Gagarawa	2000-2017	-0.137	-0.032	0.070
Nigeria	Gamawa	2000	0.106	0.693	1.672
Nigeria	Gamawa	2017	0.114	0.381	0.780
Nigeria	Gamawa	2000-2017	-0.149	-0.047	0.040
Nigeria	Gamjuwa	2000	0.194	1.024	2.186
Nigeria	Gamjuwa	2017	0.063	0.241	0.656
Nigeria	Gamjuwa	2000-2017	-0.230	-0.139	-0.031
Nigeria	Ganye	2000	0.080	0.651	1.303
Nigeria	Ganye	2017	0.093	0.435	0.850
Nigeria	Ganye	2000-2017	-0.101	-0.014	0.071
Nigeria	Garki	2000	0.095	0.881	2.148
Nigeria	Garki	2017	0.094	0.412	0.936
Nigeria	Garki	2000-2017	-0.156	-0.059	0.044
Nigeria	Garko	2000	0.208	1.207	2.502
Nigeria	Garko	2017	0.078	0.293	0.723
Nigeria	Garko	2000-2017	-0.232	-0.122	-0.015
Nigeria	Garum Mallam	2000	0.153	0.988	2.188
Nigeria	Garum Mallam	2017	0.057	0.237	0.720
Nigeria	Garum Mallam	2000-2017	-0.227	-0.108	0.028
Nigeria	Gashaka	2000	0.088	0.375	0.718
Nigeria	Gashaka	2017	0.068	0.246	0.523
Nigeria	Gashaka	2000-2017	-0.118	-0.022	0.052
Nigeria	Gassol	2000	0.120	0.588	1.202
Nigeria	Gassol	2017	0.134	0.382	0.743
Nigeria	Gassol	2000-2017	-0.108	-0.041	0.035
Nigeria	Gaya	2000	0.194	1.151	2.719
Nigeria	Gaya	2017	0.122	0.397	1.014
Nigeria	Gaya	2000-2017	-0.185	-0.094	-0.003
Nigeria	Gbako	2000	0.140	0.451	0.997

Table 3: Malaria DALYs rate by unit *(continued)*

Country	Unit	year	mean rate	lower rate	upper rate
Nigeria	Gbako	2017	0.043	0.188	0.444
Nigeria	Gbako	2000-2017	-0.178	-0.065	0.040
Nigeria	Gboko	2000	0.045	0.353	0.711
Nigeria	Gboko	2017	0.022	0.117	0.310
Nigeria	Gboko	2000-2017	-0.250	-0.105	-0.014
Nigeria	Gboyin	2000	0.307	0.996	1.718
Nigeria	Gboyin	2017	0.105	0.365	0.842
Nigeria	Gboyin	2000-2017	-0.178	-0.099	-0.014
Nigeria	Geidam	2000	0.026	0.229	0.690
Nigeria	Geidam	2017	0.020	0.120	0.313
Nigeria	Geidam	2000-2017	-0.211	-0.065	0.050
Nigeria	Gezawa	2000	0.107	0.534	1.194
Nigeria	Gezawa	2017	0.060	0.207	0.518
Nigeria	Gezawa	2000-2017	-0.199	-0.093	0.000
Nigeria	Giade	2000	0.128	0.800	1.940
Nigeria	Giade	2017	0.094	0.337	0.981
Nigeria	Giade	2000-2017	-0.201	-0.090	0.017
Nigeria	Girie	2000	0.174	0.690	1.511
Nigeria	Girie	2017	0.135	0.540	1.102
Nigeria	Girie	2000-2017	-0.118	-0.012	0.075
Nigeria	Giwa	2000	0.511	1.209	2.038
Nigeria	Giwa	2017	0.158	0.514	1.115
Nigeria	Giwa	2000-2017	-0.151	-0.065	0.010
Nigeria	Gokana	2000	0.301	0.574	0.940
Nigeria	Gokana	2017	0.017	0.062	0.135
Nigeria	Gokana	2000-2017	-0.295	-0.182	-0.064
Nigeria	Gombe	2000	0.100	0.724	1.947
Nigeria	Gombe	2017	0.122	0.421	1.067
Nigeria	Gombe	2000-2017	-0.123	-0.044	0.036
Nigeria	Gombi	2000	0.136	0.703	1.452
Nigeria	Gombi	2017	0.126	0.448	1.003
Nigeria	Gombi	2000-2017	-0.110	-0.008	0.077
Nigeria	Goronyo	2000	0.037	0.365	1.193
Nigeria	Goronyo	2017	0.173	0.457	0.835
Nigeria	Goronyo	2000-2017	-0.059	0.018	0.094
Nigeria	Gubio	2000	0.025	0.245	0.643
Nigeria	Gubio	2017	0.010	0.098	0.308
Nigeria	Gubio	2000-2017	-0.251	-0.089	0.013
Nigeria	Gudu	2000	0.074	0.384	1.056
Nigeria	Gudu	2017	0.185	0.516	0.936
Nigeria	Gudu	2000-2017	-0.071	0.021	0.108
Nigeria	Gujba	2000	0.120	0.643	1.522
Nigeria	Gujba	2017	0.104	0.454	1.074
Nigeria	Gujba	2000-2017	-0.157	-0.045	0.061
Nigeria	Gulani	2000	0.121	0.644	1.536
Nigeria	Gulani	2017	0.130	0.491	0.978
Nigeria	Gulani	2000-2017	-0.151	-0.032	0.066
Nigeria	Guma	2000	0.079	0.381	0.791
Nigeria	Guma	2017	0.034	0.146	0.383
Nigeria	Guma	2000-2017	-0.213	-0.098	-0.001
Nigeria	Gumel	2000	0.061	0.701	1.674
Nigeria	Gumel	2017	0.067	0.318	0.668
Nigeria	Gumel	2000-2017	-0.148	-0.036	0.074
Nigeria	Gummi	2000	0.072	0.546	1.447
Nigeria	Gummi	2017	0.234	0.614	1.126
Nigeria	Gummi	2000-2017	-0.059	0.011	0.085
Nigeria	Gurara	2000	0.284	0.538	0.931
Nigeria	Gurara	2017	0.026	0.125	0.299
Nigeria	Gurara	2000-2017	-0.202	-0.101	-0.021
Nigeria	Guri	2000	0.046	0.587	1.588
Nigeria	Guri	2017	0.072	0.291	0.715

Table 3: Malaria DALYs rate by unit *(continued)*

Country	Unit	year	mean rate	lower rate	upper rate
Nigeria	Guri	2000-2017	-0.180	-0.045	0.073
Nigeria	Gusau	2000	0.096	0.625	1.356
Nigeria	Gusau	2017	0.245	0.597	1.070
Nigeria	Gusau	2000-2017	-0.078	-0.014	0.072
Nigeria	Guyuk	2000	0.149	0.877	1.971
Nigeria	Guyuk	2017	0.206	0.782	1.370
Nigeria	Guyuk	2000-2017	-0.081	0.000	0.069
Nigeria	Guzamala	2000	0.033	0.258	0.614
Nigeria	Guzamala	2017	0.010	0.107	0.321
Nigeria	Guzamala	2000-2017	-0.248	-0.076	0.062
Nigeria	Gwadabaw	2000	0.039	0.309	1.061
Nigeria	Gwadabaw	2017	0.142	0.423	0.691
Nigeria	Gwadabaw	2000-2017	-0.059	0.030	0.114
Nigeria	Gwagwala	2000	0.197	0.416	0.734
Nigeria	Gwagwala	2017	0.018	0.095	0.227
Nigeria	Gwagwala	2000-2017	-0.206	-0.102	-0.006
Nigeria	Gwale	2000	0.066	0.369	0.847
Nigeria	Gwale	2017	0.025	0.104	0.261
Nigeria	Gwale	2000-2017	-0.209	-0.110	0.013
Nigeria	Gwandu	2000	0.043	0.399	1.126
Nigeria	Gwandu	2017	0.157	0.431	0.808
Nigeria	Gwandu	2000-2017	-0.097	-0.011	0.061
Nigeria	Gwaram	2000	0.149	0.825	1.983
Nigeria	Gwaram	2017	0.056	0.234	0.762
Nigeria	Gwaram	2000-2017	-0.238	-0.131	-0.016
Nigeria	Gwarzo	2000	0.204	1.091	2.068
Nigeria	Gwarzo	2017	0.125	0.436	1.013
Nigeria	Gwarzo	2000-2017	-0.174	-0.085	0.028
Nigeria	Gwer East	2000	0.107	0.539	1.007
Nigeria	Gwer East	2017	0.073	0.260	0.547
Nigeria	Gwer East	2000-2017	-0.158	-0.071	0.000
Nigeria	GwerWest	2000	0.141	0.523	0.985
Nigeria	GwerWest	2017	0.044	0.201	0.453
Nigeria	GwerWest	2000-2017	-0.196	-0.090	0.009
Nigeria	Gwiwa	2000	0.093	0.563	1.262
Nigeria	Gwiwa	2017	0.069	0.243	0.680
Nigeria	Gwiwa	2000-2017	-0.220	-0.102	-0.005
Nigeria	Gwoza	2000	0.082	0.418	1.255
Nigeria	Gwoza	2017	0.039	0.203	0.675
Nigeria	Gwoza	2000-2017	-0.240	-0.088	0.052
Nigeria	Hadejia	2000	0.015	0.283	0.694
Nigeria	Hadejia	2017	0.039	0.120	0.255
Nigeria	Hadejia	2000-2017	-0.138	-0.020	0.096
Nigeria	Hawul	2000	0.128	0.545	1.258
Nigeria	Hawul	2017	0.088	0.453	0.896
Nigeria	Hawul	2000-2017	-0.158	-0.017	0.072
Nigeria	Hong	2000	0.101	0.544	1.352
Nigeria	Hong	2017	0.080	0.325	0.731
Nigeria	Hong	2000-2017	-0.137	-0.033	0.066
Nigeria	IbadanNorth	2000	0.226	0.471	0.872
Nigeria	IbadanNorth	2017	0.020	0.091	0.244
Nigeria	IbadanNorth	2000-2017	-0.225	-0.131	-0.045
Nigeria	IbadanNorth-East	2000	0.229	0.470	0.859
Nigeria	IbadanNorth-East	2017	0.019	0.087	0.234
Nigeria	IbadanNorth-East	2000-2017	-0.224	-0.133	-0.049
Nigeria	IbadanNorth-West	2000	0.232	0.473	0.865
Nigeria	IbadanNorth-West	2017	0.022	0.095	0.247
Nigeria	IbadanNorth-West	2000-2017	-0.224	-0.129	-0.047
Nigeria	IbadanSouth-East	2000	0.248	0.469	0.840
Nigeria	IbadanSouth-East	2017	0.020	0.089	0.229
Nigeria	IbadanSouth-East	2000-2017	-0.229	-0.132	-0.050

Table 3: Malaria DALYs rate by unit *(continued)*

Country	Unit	year	mean rate	lower rate	upper rate
Nigeria	IbadanSouth-West	2000	0.249	0.482	0.861
Nigeria	IbadanSouth-West	2017	0.021	0.092	0.234
Nigeria	IbadanSouth-West	2000-2017	-0.229	-0.132	-0.051
Nigeria	Ibaji	2000	0.178	0.653	1.514
Nigeria	Ibaji	2017	0.022	0.090	0.249
Nigeria	Ibaji	2000-2017	-0.294	-0.185	-0.073
Nigeria	Ibarapa Central	2000	0.235	0.671	1.186
Nigeria	Ibarapa Central	2017	0.070	0.269	0.618
Nigeria	Ibarapa Central	2000-2017	-0.175	-0.069	0.026
Nigeria	Ibarapa East	2000	0.276	0.609	1.038
Nigeria	Ibarapa East	2017	0.078	0.254	0.541
Nigeria	Ibarapa East	2000-2017	-0.157	-0.069	-0.003
Nigeria	Ibarapa North	2000	0.215	0.660	1.148
Nigeria	Ibarapa North	2017	0.076	0.298	0.665
Nigeria	Ibarapa North	2000-2017	-0.165	-0.061	0.018
Nigeria	Ibeju/Lekki	2000	0.118	0.534	1.086
Nigeria	Ibeju/Lekki	2017	0.021	0.090	0.262
Nigeria	Ibeju/Lekki	2000-2017	-0.189	-0.109	-0.017
Nigeria	Ibeno	2000	0.332	0.623	1.007
Nigeria	Ibeno	2017	0.031	0.132	0.302
Nigeria	Ibeno	2000-2017	-0.224	-0.127	-0.030
Nigeria	Ibesikpo Asutan	2000	0.326	0.620	1.003
Nigeria	Ibesikpo Asutan	2017	0.017	0.084	0.209
Nigeria	Ibesikpo Asutan	2000-2017	-0.279	-0.165	-0.052
Nigeria	Ibi	2000	0.098	0.470	1.017
Nigeria	Ibi	2017	0.065	0.214	0.466
Nigeria	Ibi	2000-2017	-0.149	-0.068	0.027
Nigeria	Ibiono Ibom	2000	0.426	0.793	1.297
Nigeria	Ibiono Ibom	2017	0.021	0.104	0.253
Nigeria	Ibiono Ibom	2000-2017	-0.271	-0.168	-0.062
Nigeria	Idah	2000	0.107	0.577	1.558
Nigeria	Idah	2017	0.011	0.078	0.247
Nigeria	Idah	2000-2017	-0.351	-0.198	-0.050
Nigeria	Idanre	2000	0.388	1.092	1.942
Nigeria	Idanre	2017	0.231	0.644	1.261
Nigeria	Idanre	2000-2017	-0.129	-0.049	0.009
Nigeria	Ideato South	2000	0.491	0.910	1.496
Nigeria	Ideato South	2017	0.026	0.118	0.263
Nigeria	Ideato South	2000-2017	-0.298	-0.182	-0.084
Nigeria	IdeatoNo	2000	0.562	1.008	1.645
Nigeria	IdeatoNo	2017	0.027	0.129	0.304
Nigeria	IdeatoNo	2000-2017	-0.299	-0.183	-0.084
Nigeria	Idemili North	2000	0.193	0.535	1.002
Nigeria	Idemili North	2017	0.015	0.060	0.145
Nigeria	Idemili North	2000-2017	-0.292	-0.197	-0.108
Nigeria	Idemili South	2000	0.211	0.600	1.096
Nigeria	Idemili South	2017	0.017	0.064	0.157
Nigeria	Idemili South	2000-2017	-0.295	-0.200	-0.108
Nigeria	Ido	2000	0.375	0.702	1.148
Nigeria	Ido	2017	0.078	0.252	0.510
Nigeria	Ido	2000-2017	-0.166	-0.081	-0.021
Nigeria	Ido/Osi	2000	0.138	0.693	1.274
Nigeria	Ido/Osi	2017	0.076	0.258	0.603
Nigeria	Ido/Osi	2000-2017	-0.177	-0.094	-0.011
Nigeria	Ifako/Ijaye	2000	0.040	0.159	0.379
Nigeria	Ifako/Ijaye	2017	0.009	0.040	0.090
Nigeria	Ifako/Ijaye	2000-2017	-0.157	-0.066	0.026
Nigeria	Ife East	2000	0.622	1.362	2.314
Nigeria	Ife East	2017	0.201	0.753	1.486
Nigeria	Ife East	2000-2017	-0.142	-0.057	-0.009
Nigeria	Ife North	2000	0.437	1.012	1.649

Table 3: Malaria DALYs rate by unit *(continued)*

Country	Unit	year	mean rate	lower rate	upper rate
Nigeria	Ife North	2017	0.119	0.453	0.910
Nigeria	Ife North	2000-2017	-0.165	-0.074	-0.016
Nigeria	Ife South	2000	0.517	1.220	2.033
Nigeria	Ife South	2017	0.170	0.668	1.345
Nigeria	Ife South	2000-2017	-0.148	-0.062	-0.008
Nigeria	IfeCentral	2000	0.403	1.136	1.920
Nigeria	IfeCentral	2017	0.109	0.451	0.935
Nigeria	IfeCentral	2000-2017	-0.183	-0.088	-0.021
Nigeria	Ifedayo	2000	0.127	0.594	1.098
Nigeria	Ifedayo	2017	0.063	0.240	0.570
Nigeria	Ifedayo	2000-2017	-0.198	-0.088	-0.011
Nigeria	Ifedore	2000	0.288	1.108	2.001
Nigeria	Ifedore	2017	0.210	0.618	1.264
Nigeria	Ifedore	2000-2017	-0.133	-0.055	0.019
Nigeria	Ifelodun	2000	0.125	0.531	0.948
Nigeria	Ifelodun	2000	0.146	0.452	0.834
Nigeria	Ifelodun	2017	0.035	0.159	0.381
Nigeria	Ifelodun	2017	0.041	0.160	0.378
Nigeria	Ifelodun	2000-2017	-0.195	-0.098	-0.016
Nigeria	Ifelodun	2000-2017	-0.237	-0.115	-0.028
Nigeria	Ifo	2000	0.097	0.279	0.580
Nigeria	Ifo	2017	0.020	0.075	0.161
Nigeria	Ifo	2000-2017	-0.153	-0.067	0.018
Nigeria	Igabi	2000	0.458	0.893	1.519
Nigeria	Igabi	2017	0.127	0.340	0.724
Nigeria	Igabi	2000-2017	-0.133	-0.061	0.019
Nigeria	Igalamela-Odolu	2000	0.105	0.493	1.219
Nigeria	Igalamela-Odolu	2017	0.014	0.075	0.203
Nigeria	Igalamela-Odolu	2000-2017	-0.306	-0.175	-0.055
Nigeria	Igbo-Eti	2000	0.084	0.328	0.826
Nigeria	Igbo-Eti	2017	0.013	0.069	0.180
Nigeria	Igbo-Eti	2000-2017	-0.229	-0.140	-0.039
Nigeria	Igbo-eze North	2000	0.056	0.323	0.752
Nigeria	Igbo-eze North	2017	0.016	0.083	0.203
Nigeria	Igbo-eze North	2000-2017	-0.232	-0.125	0.001
Nigeria	Igbo-eze South	2000	0.044	0.282	0.711
Nigeria	Igbo-eze South	2017	0.011	0.062	0.169
Nigeria	Igbo-eze South	2000-2017	-0.247	-0.135	-0.004
Nigeria	Igueben	2000	0.228	0.818	1.788
Nigeria	Igueben	2017	0.034	0.176	0.419
Nigeria	Igueben	2000-2017	-0.263	-0.155	-0.054
Nigeria	Ihiala	2000	0.394	0.747	1.299
Nigeria	Ihiala	2017	0.016	0.068	0.168
Nigeria	Ihiala	2000-2017	-0.299	-0.202	-0.099
Nigeria	Ihitte/U	2000	0.319	0.612	1.011
Nigeria	Ihitte/U	2017	0.016	0.076	0.158
Nigeria	Ihitte/U	2000-2017	-0.301	-0.179	-0.076
Nigeria	Ijebu North-East	2000	0.442	0.931	1.552
Nigeria	Ijebu North-East	2017	0.067	0.257	0.624
Nigeria	Ijebu North-East	2000-2017	-0.189	-0.104	-0.027
Nigeria	IjebuEast	2000	0.483	1.077	1.778
Nigeria	IjebuEast	2017	0.092	0.346	0.744
Nigeria	IjebuEast	2000-2017	-0.168	-0.094	-0.021
Nigeria	IjebuNorth	2000	0.593	1.070	1.765
Nigeria	IjebuNorth	2017	0.083	0.309	0.684
Nigeria	IjebuNorth	2000-2017	-0.172	-0.094	-0.024
Nigeria	IjebuOde	2000	0.180	0.534	0.944
Nigeria	IjebuOde	2017	0.028	0.112	0.311
Nigeria	IjebuOde	2000-2017	-0.196	-0.111	-0.027
Nigeria	Ijero	2000	0.151	0.705	1.321
Nigeria	Ijero	2017	0.087	0.286	0.668

Table 3: Malaria DALYs rate by unit *(continued)*

Country	Unit	year	mean rate	lower rate	upper rate
Nigeria	Ijero	2000-2017	-0.171	-0.083	-0.006
Nigeria	Ijumu	2000	0.204	0.515	0.950
Nigeria	Ijumu	2017	0.031	0.122	0.273
Nigeria	Ijumu	2000-2017	-0.226	-0.137	-0.036
Nigeria	Ika	2000	0.432	0.815	1.356
Nigeria	Ika	2017	0.025	0.119	0.295
Nigeria	Ika	2000-2017	-0.283	-0.171	-0.070
Nigeria	IkaNorth	2000	0.167	0.674	1.596
Nigeria	IkaNorth	2017	0.030	0.138	0.404
Nigeria	IkaNorth	2000-2017	-0.264	-0.162	-0.059
Nigeria	Ikara	2000	0.372	1.297	2.195
Nigeria	Ikara	2017	0.082	0.403	1.035
Nigeria	Ikara	2000-2017	-0.189	-0.084	0.008
Nigeria	IkaSouth	2000	0.109	0.553	1.348
Nigeria	IkaSouth	2017	0.027	0.123	0.341
Nigeria	IkaSouth	2000-2017	-0.245	-0.151	-0.045
Nigeria	Ikeduru	2000	0.387	0.734	1.197
Nigeria	Ikeduru	2017	0.020	0.086	0.188
Nigeria	Ikeduru	2000-2017	-0.289	-0.183	-0.079
Nigeria	Ikeja	2000	0.024	0.098	0.253
Nigeria	Ikeja	2017	0.005	0.025	0.054
Nigeria	Ikeja	2000-2017	-0.162	-0.072	0.026
Nigeria	Ikenne	2000	0.254	0.711	1.278
Nigeria	Ikenne	2017	0.045	0.158	0.371
Nigeria	Ikenne	2000-2017	-0.182	-0.099	-0.015
Nigeria	Ikere	2000	0.196	0.836	1.536
Nigeria	Ikere	2017	0.111	0.329	0.751
Nigeria	Ikere	2000-2017	-0.166	-0.081	-0.002
Nigeria	Ikole	2000	0.251	0.831	1.416
Nigeria	Ikole	2017	0.071	0.251	0.636
Nigeria	Ikole	2000-2017	-0.192	-0.108	-0.016
Nigeria	Ikom	2000	0.216	0.742	1.357
Nigeria	Ikom	2017	0.096	0.360	0.733
Nigeria	Ikom	2000-2017	-0.196	-0.078	0.002
Nigeria	Ikono	2000	0.377	0.719	1.188
Nigeria	Ikono	2017	0.018	0.089	0.200
Nigeria	Ikono	2000-2017	-0.272	-0.169	-0.063
Nigeria	Ikorodu	2000	0.049	0.164	0.347
Nigeria	Ikorodu	2017	0.009	0.037	0.083
Nigeria	Ikorodu	2000-2017	-0.171	-0.085	0.002
Nigeria	Ikot-Aba	2000	0.368	0.686	1.126
Nigeria	Ikot-Aba	2017	0.015	0.075	0.178
Nigeria	Ikot-Aba	2000-2017	-0.295	-0.178	-0.069
Nigeria	Ikot-Ekp	2000	0.323	0.620	0.992
Nigeria	Ikot-Ekp	2017	0.013	0.070	0.160
Nigeria	Ikot-Ekp	2000-2017	-0.283	-0.178	-0.073
Nigeria	Ikpoba-Okha	2000	0.125	0.414	1.024
Nigeria	Ikpoba-Okha	2017	0.037	0.148	0.312
Nigeria	Ikpoba-Okha	2000-2017	-0.213	-0.113	-0.012
Nigeria	Ikwerre	2000	0.239	0.490	0.831
Nigeria	Ikwerre	2017	0.013	0.041	0.103
Nigeria	Ikwerre	2000-2017	-0.295	-0.203	-0.082
Nigeria	Ikwo	2000	0.259	0.869	1.584
Nigeria	Ikwo	2017	0.093	0.303	0.656
Nigeria	Ikwo	2000-2017	-0.192	-0.105	-0.036
Nigeria	Ikwuano	2000	0.517	0.972	1.613
Nigeria	Ikwuano	2017	0.035	0.172	0.349
Nigeria	Ikwuano	2000-2017	-0.259	-0.148	-0.054
Nigeria	Ila	2000	0.120	0.556	1.063
Nigeria	Ila	2017	0.051	0.200	0.496
Nigeria	Ila	2000-2017	-0.215	-0.097	-0.016

Table 3: Malaria DALYs rate by unit *(continued)*

Country	Unit	year	mean rate	lower rate	upper rate
Nigeria	IlajeEseodo	2000	0.276	0.863	1.668
Nigeria	IlajeEseodo	2017	0.039	0.185	0.466
Nigeria	IlajeEseodo	2000-2017	-0.248	-0.142	-0.043
Nigeria	Ilejemeje	2000	0.125	0.634	1.172
Nigeria	Ilejemeje	2017	0.063	0.221	0.563
Nigeria	Ilejemeje	2000-2017	-0.196	-0.098	-0.013
Nigeria	IleOluji/Okeigbo	2000	0.491	1.360	2.350
Nigeria	IleOluji/Okeigbo	2017	0.249	0.787	1.487
Nigeria	IleOluji/Okeigbo	2000-2017	-0.120	-0.047	0.012
Nigeria	Ilesha East	2000	0.216	0.833	1.557
Nigeria	Ilesha East	2017	0.089	0.307	0.670
Nigeria	Ilesha East	2000-2017	-0.163	-0.089	-0.008
Nigeria	Ilesha West	2000	0.180	0.706	1.360
Nigeria	Ilesha West	2017	0.067	0.236	0.518
Nigeria	Ilesha West	2000-2017	-0.176	-0.096	-0.014
Nigeria	Illela	2000	0.044	0.352	1.230
Nigeria	Illela	2017	0.147	0.411	0.793
Nigeria	Illela	2000-2017	-0.081	0.012	0.102
Nigeria	Ilorin East	2000	0.076	0.256	0.475
Nigeria	Ilorin East	2017	0.018	0.083	0.187
Nigeria	Ilorin East	2000-2017	-0.215	-0.106	-0.020
Nigeria	Ilorin South	2000	0.104	0.308	0.568
Nigeria	Ilorin South	2017	0.022	0.102	0.240
Nigeria	Ilorin South	2000-2017	-0.212	-0.104	-0.018
Nigeria	IlorinWe	2000	0.057	0.229	0.467
Nigeria	IlorinWe	2017	0.012	0.064	0.154
Nigeria	IlorinWe	2000-2017	-0.240	-0.122	-0.026
Nigeria	Imeko-Afon	2000	0.201	0.639	1.170
Nigeria	Imeko-Afon	2017	0.081	0.319	0.641
Nigeria	Imeko-Afon	2000-2017	-0.148	-0.053	0.015
Nigeria	Ingawa	2000	0.125	0.621	1.349
Nigeria	Ingawa	2017	0.082	0.293	0.799
Nigeria	Ingawa	2000-2017	-0.200	-0.093	0.000
Nigeria	Ini	2000	0.478	0.908	1.513
Nigeria	Ini	2017	0.026	0.126	0.277
Nigeria	Ini	2000-2017	-0.260	-0.163	-0.072
Nigeria	Ipokia	2000	0.219	0.558	0.993
Nigeria	Ipokia	2017	0.065	0.246	0.529
Nigeria	Ipokia	2000-2017	-0.116	-0.023	0.056
Nigeria	Irele	2000	0.460	1.041	1.813
Nigeria	Irele	2017	0.105	0.408	0.798
Nigeria	Irele	2000-2017	-0.201	-0.095	-0.024
Nigeria	Irepo	2000	0.114	0.410	0.801
Nigeria	Irepo	2017	0.083	0.256	0.495
Nigeria	Irepo	2000-2017	-0.133	-0.040	0.030
Nigeria	Irepodun	2000	0.152	0.596	1.025
Nigeria	Irepodun	2000	0.123	0.497	0.910
Nigeria	Irepodun	2017	0.042	0.192	0.434
Nigeria	Irepodun	2017	0.042	0.182	0.478
Nigeria	Irepodun	2000-2017	-0.210	-0.097	-0.016
Nigeria	Irepodun	2000-2017	-0.231	-0.109	-0.016
Nigeria	Irepodun/Ifelodun	2000	0.167	0.800	1.471
Nigeria	Irepodun/Ifelodun	2017	0.091	0.297	0.680
Nigeria	Irepodun/Ifelodun	2000-2017	-0.163	-0.089	-0.012
Nigeria	Irewole	2000	0.494	0.999	1.635
Nigeria	Irewole	2017	0.107	0.361	0.721
Nigeria	Irewole	2000-2017	-0.181	-0.092	-0.024
Nigeria	Isa	2000	0.066	0.640	1.750
Nigeria	Isa	2017	0.166	0.661	1.409
Nigeria	Isa	2000-2017	-0.104	0.000	0.079
Nigeria	Ise/Orun	2000	0.292	1.074	1.889

Table 3: Malaria DALYs rate by unit *(continued)*

Country	Unit	year	mean rate	lower rate	upper rate
Nigeria	Ise/Orun	2017	0.157	0.480	1.017
Nigeria	Ise/Orun	2000-2017	-0.158	-0.079	-0.008
Nigeria	Iseyin	2000	0.248	0.567	0.993
Nigeria	Iseyin	2017	0.068	0.265	0.559
Nigeria	Iseyin	2000-2017	-0.159	-0.062	-0.007
Nigeria	Ishielu	2000	0.252	0.754	1.637
Nigeria	Ishielu	2017	0.042	0.209	0.522
Nigeria	Ishielu	2000-2017	-0.223	-0.118	-0.042
Nigeria	Isi-Uzo	2000	0.143	0.494	0.992
Nigeria	Isi-Uzo	2017	0.025	0.143	0.357
Nigeria	Isi-Uzo	2000-2017	-0.222	-0.118	-0.037
Nigeria	Isiala Ngwa North	2000	0.589	1.096	1.796
Nigeria	Isiala Ngwa North	2017	0.041	0.165	0.352
Nigeria	Isiala Ngwa North	2000-2017	-0.279	-0.164	-0.071
Nigeria	Isiala Ngwa South	2000	0.515	0.957	1.574
Nigeria	Isiala Ngwa South	2017	0.035	0.141	0.321
Nigeria	Isiala Ngwa South	2000-2017	-0.281	-0.170	-0.078
Nigeria	IsialaMb	2000	0.453	0.845	1.375
Nigeria	IsialaMb	2017	0.028	0.118	0.253
Nigeria	IsialaMb	2000-2017	-0.287	-0.173	-0.069
Nigeria	Isin	2000	0.095	0.443	0.843
Nigeria	Isin	2017	0.034	0.157	0.430
Nigeria	Isin	2000-2017	-0.216	-0.100	-0.006
Nigeria	Isokan	2000	0.708	1.327	2.164
Nigeria	Isokan	2017	0.187	0.634	1.189
Nigeria	Isokan	2000-2017	-0.153	-0.066	-0.010
Nigeria	IsokoNor	2000	0.101	0.403	0.915
Nigeria	IsokoNor	2017	0.009	0.044	0.111
Nigeria	IsokoNor	2000-2017	-0.268	-0.159	-0.055
Nigeria	IsokoSou	2000	0.110	0.484	1.021
Nigeria	IsokoSou	2017	0.012	0.056	0.130
Nigeria	IsokoSou	2000-2017	-0.250	-0.154	-0.058
Nigeria	Isu	2000	0.481	0.896	1.457
Nigeria	Isu	2017	0.029	0.109	0.231
Nigeria	Isu	2000-2017	-0.288	-0.180	-0.083
Nigeria	Isuikwua	2000	0.582	1.089	1.808
Nigeria	Isuikwua	2017	0.022	0.132	0.295
Nigeria	Isuikwua	2000-2017	-0.306	-0.180	-0.068
Nigeria	Itas/Gad	2000	0.098	0.591	1.314
Nigeria	Itas/Gad	2017	0.066	0.223	0.520
Nigeria	Itas/Gad	2000-2017	-0.190	-0.078	0.033
Nigeria	Itesiwaju	2000	0.280	0.654	1.146
Nigeria	Itesiwaju	2017	0.109	0.349	0.629
Nigeria	Itesiwaju	2000-2017	-0.145	-0.048	-0.006
Nigeria	Itu	2000	0.358	0.671	1.078
Nigeria	Itu	2017	0.016	0.080	0.194
Nigeria	Itu	2000-2017	-0.285	-0.174	-0.063
Nigeria	Ivo	2000	0.401	0.732	1.258
Nigeria	Ivo	2017	0.024	0.115	0.245
Nigeria	Ivo	2000-2017	-0.268	-0.164	-0.073
Nigeria	Iwajowa	2000	0.204	0.628	1.134
Nigeria	Iwajowa	2017	0.093	0.333	0.635
Nigeria	Iwajowa	2000-2017	-0.141	-0.047	0.011
Nigeria	Iwo	2000	0.376	0.803	1.346
Nigeria	Iwo	2017	0.075	0.274	0.612
Nigeria	Iwo	2000-2017	-0.193	-0.099	-0.023
Nigeria	Izzi	2000	0.116	0.463	0.851
Nigeria	Izzi	2017	0.054	0.207	0.482
Nigeria	Izzi	2000-2017	-0.193	-0.088	-0.011
Nigeria	Jaba	2000	0.488	0.870	1.477
Nigeria	Jaba	2017	0.069	0.287	0.742

Table 3: Malaria DALYs rate by unit *(continued)*

Country	Unit	year	mean rate	lower rate	upper rate
Nigeria	Jaba	2000-2017	-0.181	-0.084	-0.013
Nigeria	Jada	2000	0.101	0.799	1.616
Nigeria	Jada	2017	0.165	0.550	1.038
Nigeria	Jada	2000-2017	-0.108	-0.014	0.066
Nigeria	Jahun	2000	0.127	0.815	1.879
Nigeria	Jahun	2017	0.119	0.338	0.770
Nigeria	Jahun	2000-2017	-0.170	-0.059	0.054
Nigeria	Jakusko	2000	0.072	0.659	1.795
Nigeria	Jakusko	2017	0.099	0.372	0.740
Nigeria	Jakusko	2000-2017	-0.139	-0.051	0.041
Nigeria	Jalingo	2000	0.051	0.522	1.179
Nigeria	Jalingo	2017	0.139	0.376	0.750
Nigeria	Jalingo	2000-2017	-0.124	-0.025	0.064
Nigeria	Jama'are	2000	0.107	0.657	1.517
Nigeria	Jama'are	2017	0.058	0.220	0.551
Nigeria	Jama'are	2000-2017	-0.226	-0.102	0.022
Nigeria	Jega	2000	0.047	0.474	1.219
Nigeria	Jega	2017	0.225	0.537	0.943
Nigeria	Jega	2000-2017	-0.085	-0.006	0.061
Nigeria	Jema'a	2000	0.355	0.633	1.057
Nigeria	Jema'a	2017	0.064	0.240	0.504
Nigeria	Jema'a	2000-2017	-0.165	-0.077	-0.014
Nigeria	Jere	2000	0.064	0.278	1.096
Nigeria	Jere	2017	0.014	0.082	0.233
Nigeria	Jere	2000-2017	-0.225	-0.113	0.030
Nigeria	Jibia	2000	0.050	0.352	0.896
Nigeria	Jibia	2017	0.056	0.232	0.649
Nigeria	Jibia	2000-2017	-0.170	-0.055	0.037
Nigeria	Jos East	2000	0.279	0.920	1.808
Nigeria	Jos East	2017	0.074	0.327	0.717
Nigeria	Jos East	2000-2017	-0.217	-0.098	-0.012
Nigeria	Jos North	2000	0.133	0.515	0.995
Nigeria	Jos North	2017	0.039	0.173	0.372
Nigeria	Jos North	2000-2017	-0.236	-0.101	-0.012
Nigeria	Jos South	2000	0.264	0.808	1.540
Nigeria	Jos South	2017	0.058	0.266	0.567
Nigeria	Jos South	2000-2017	-0.232	-0.097	-0.008
Nigeria	Kabba/Bu	2000	0.139	0.499	0.950
Nigeria	Kabba/Bu	2017	0.034	0.111	0.283
Nigeria	Kabba/Bu	2000-2017	-0.247	-0.138	-0.046
Nigeria	Kabo	2000	0.206	1.092	2.151
Nigeria	Kabo	2017	0.113	0.379	0.906
Nigeria	Kabo	2000-2017	-0.189	-0.094	0.026
Nigeria	Kachia	2000	0.414	0.738	1.225
Nigeria	Kachia	2017	0.101	0.320	0.593
Nigeria	Kachia	2000-2017	-0.140	-0.059	-0.017
Nigeria	Kaduna North	2000	0.189	0.446	0.806
Nigeria	Kaduna North	2017	0.038	0.137	0.325
Nigeria	Kaduna North	2000-2017	-0.174	-0.070	0.023
Nigeria	Kaduna South	2000	0.187	0.456	0.829
Nigeria	Kaduna South	2017	0.034	0.131	0.314
Nigeria	Kaduna South	2000-2017	-0.183	-0.075	0.013
Nigeria	KafinHau	2000	0.070	0.664	1.549
Nigeria	KafinHau	2017	0.103	0.319	0.677
Nigeria	KafinHau	2000-2017	-0.142	-0.040	0.065
Nigeria	Kafur	2000	0.278	1.084	2.334
Nigeria	Kafur	2017	0.102	0.453	1.010
Nigeria	Kafur	2000-2017	-0.195	-0.078	0.008
Nigeria	Kaga	2000	0.095	0.509	1.343
Nigeria	Kaga	2017	0.049	0.250	0.637
Nigeria	Kaga	2000-2017	-0.182	-0.073	0.045

Table 3: Malaria DALYs rate by unit *(continued)*

Country	Unit	year	mean rate	lower rate	upper rate
Nigeria	Kagarko	2000	0.385	0.679	1.127
Nigeria	Kagarko	2017	0.046	0.198	0.431
Nigeria	Kagarko	2000-2017	-0.191	-0.088	-0.013
Nigeria	Kaiama	2000	0.129	0.402	0.733
Nigeria	Kaiama	2017	0.099	0.276	0.503
Nigeria	Kaiama	2000-2017	-0.125	-0.037	0.013
Nigeria	Kaita	2000	0.078	0.565	1.446
Nigeria	Kaita	2017	0.068	0.303	0.962
Nigeria	Kaita	2000-2017	-0.213	-0.089	0.044
Nigeria	Kajola	2000	0.228	0.692	1.257
Nigeria	Kajola	2017	0.090	0.365	0.719
Nigeria	Kajola	2000-2017	-0.170	-0.053	0.008
Nigeria	Kajuru	2000	0.619	1.127	1.880
Nigeria	Kajuru	2017	0.166	0.553	1.122
Nigeria	Kajuru	2000-2017	-0.128	-0.049	-0.002
Nigeria	Kala/Balge	2000	0.133	0.434	1.022
Nigeria	Kala/Balge	2017	0.040	0.235	0.581
Nigeria	Kala/Balge	2000-2017	-0.162	-0.012	0.094
Nigeria	Kalgo	2000	0.064	0.470	1.289
Nigeria	Kalgo	2017	0.161	0.504	0.952
Nigeria	Kalgo	2000-2017	-0.117	-0.018	0.069
Nigeria	Kaltungo	2000	0.109	0.768	1.752
Nigeria	Kaltungo	2017	0.178	0.550	1.212
Nigeria	Kaltungo	2000-2017	-0.105	-0.027	0.046
Nigeria	Kanam	2000	0.177	0.987	1.992
Nigeria	Kanam	2017	0.139	0.529	1.058
Nigeria	Kanam	2000-2017	-0.196	-0.069	-0.004
Nigeria	Kankara	2000	0.219	1.008	2.416
Nigeria	Kankara	2017	0.125	0.493	1.033
Nigeria	Kankara	2000-2017	-0.170	-0.073	0.029
Nigeria	Kanke	2000	0.237	0.960	1.988
Nigeria	Kanke	2017	0.156	0.552	1.272
Nigeria	Kanke	2000-2017	-0.166	-0.065	0.009
Nigeria	Kankiya	2000	0.190	0.881	1.817
Nigeria	Kankiya	2017	0.117	0.420	0.979
Nigeria	Kankiya	2000-2017	-0.182	-0.085	0.008
Nigeria	Kano	2000	0.070	0.389	0.854
Nigeria	Kano	2017	0.027	0.111	0.276
Nigeria	Kano	2000-2017	-0.212	-0.113	0.008
Nigeria	Karasuwa	2000	0.029	0.443	1.254
Nigeria	Karasuwa	2017	0.049	0.217	0.549
Nigeria	Karasuwa	2000-2017	-0.158	-0.060	0.062
Nigeria	Karaye	2000	0.189	0.989	2.034
Nigeria	Karaye	2017	0.112	0.349	0.815
Nigeria	Karaye	2000-2017	-0.182	-0.086	0.026
Nigeria	Karim-La	2000	0.086	0.492	1.066
Nigeria	Karim-La	2017	0.120	0.300	0.561
Nigeria	Karim-La	2000-2017	-0.131	-0.047	0.013
Nigeria	Karu	2000	0.272	0.518	0.866
Nigeria	Karu	2017	0.025	0.126	0.329
Nigeria	Karu	2000-2017	-0.221	-0.099	-0.015
Nigeria	Katagum	2000	0.117	0.566	1.393
Nigeria	Katagum	2017	0.064	0.226	0.676
Nigeria	Katagum	2000-2017	-0.200	-0.087	0.028
Nigeria	Katcha	2000	0.207	0.622	1.300
Nigeria	Katcha	2017	0.040	0.201	0.524
Nigeria	Katcha	2000-2017	-0.197	-0.080	0.013
Nigeria	Katsina (Benue)	2000	0.055	0.317	0.640
Nigeria	Katsina (Benue)	2017	0.030	0.151	0.358
Nigeria	Katsina (Benue)	2000-2017	-0.203	-0.076	0.005
Nigeria	Katsina (K)	2000	0.027	0.216	0.603

Table 3: Malaria DALYs rate by unit *(continued)*

Country	Unit	year	mean rate	lower rate	upper rate
Nigeria	Katsina (K)	2017	0.024	0.111	0.383
Nigeria	Katsina (K)	2000-2017	-0.205	-0.094	0.031
Nigeria	Kaugama	2000	0.046	0.704	1.632
Nigeria	Kaugama	2017	0.095	0.326	0.713
Nigeria	Kaugama	2000-2017	-0.131	-0.023	0.099
Nigeria	Kaura	2000	0.401	0.749	1.290
Nigeria	Kaura	2017	0.084	0.282	0.558
Nigeria	Kaura	2000-2017	-0.185	-0.079	-0.012
Nigeria	Kaura-Na	2000	0.081	0.606	1.473
Nigeria	Kaura-Na	2017	0.197	0.642	1.175
Nigeria	Kaura-Na	2000-2017	-0.088	0.001	0.076
Nigeria	Kauru	2000	0.577	1.106	1.861
Nigeria	Kauru	2017	0.130	0.388	0.814
Nigeria	Kauru	2000-2017	-0.147	-0.075	-0.014
Nigeria	Kazaure	2000	0.062	0.506	1.249
Nigeria	Kazaure	2017	0.060	0.226	0.630
Nigeria	Kazaure	2000-2017	-0.206	-0.099	0.004
Nigeria	Keana	2000	0.091	0.381	0.799
Nigeria	Keana	2017	0.043	0.196	0.454
Nigeria	Keana	2000-2017	-0.167	-0.069	0.015
Nigeria	Kebbe	2000	0.080	0.612	1.605
Nigeria	Kebbe	2017	0.301	0.666	1.163
Nigeria	Kebbe	2000-2017	-0.065	0.001	0.053
Nigeria	Keffi	2000	0.224	0.440	0.778
Nigeria	Keffi	2017	0.024	0.121	0.353
Nigeria	Keffi	2000-2017	-0.221	-0.098	0.005
Nigeria	Khana	2000	0.366	0.676	1.124
Nigeria	Khana	2017	0.017	0.070	0.161
Nigeria	Khana	2000-2017	-0.296	-0.186	-0.074
Nigeria	Kibiya	2000	0.233	1.239	2.537
Nigeria	Kibiya	2017	0.078	0.285	0.717
Nigeria	Kibiya	2000-2017	-0.232	-0.119	-0.014
Nigeria	Kirfi	2000	0.149	0.789	1.746
Nigeria	Kirfi	2017	0.076	0.284	0.863
Nigeria	Kirfi	2000-2017	-0.181	-0.095	-0.007
Nigeria	KiriKasa	2000	0.036	0.587	1.487
Nigeria	KiriKasa	2017	0.082	0.265	0.574
Nigeria	KiriKasa	2000-2017	-0.146	-0.030	0.077
Nigeria	Kiru	2000	0.244	1.144	2.315
Nigeria	Kiru	2017	0.106	0.367	0.926
Nigeria	Kiru	2000-2017	-0.190	-0.087	0.010
Nigeria	Kiyawa	2000	0.182	0.893	2.053
Nigeria	Kiyawa	2017	0.094	0.316	0.809
Nigeria	Kiyawa	2000-2017	-0.189	-0.087	0.019
Nigeria	Koko/Bes	2000	0.065	0.664	1.583
Nigeria	Koko/Bes	2017	0.393	0.815	1.458
Nigeria	Koko/Bes	2000-2017	-0.041	0.014	0.082
Nigeria	Kokona	2000	0.298	0.510	0.867
Nigeria	Kokona	2017	0.044	0.175	0.457
Nigeria	Kokona	2000-2017	-0.182	-0.080	-0.003
Nigeria	Kolokuma/Opokuma	2000	0.228	0.811	1.649
Nigeria	Kolokuma/Opokuma	2017	0.030	0.118	0.321
Nigeria	Kolokuma/Opokuma	2000-2017	-0.234	-0.131	-0.037
Nigeria	Konduga	2000	0.056	0.233	0.767
Nigeria	Konduga	2017	0.016	0.078	0.193
Nigeria	Konduga	2000-2017	-0.221	-0.102	0.036
Nigeria	Konshish	2000	0.077	0.447	0.957
Nigeria	Konshish	2017	0.057	0.239	0.530
Nigeria	Konshish	2000-2017	-0.172	-0.068	0.008
Nigeria	Kontogur	2000	0.195	0.665	1.346
Nigeria	Kontogur	2017	0.230	0.594	1.005

Table 3: Malaria DALYs rate by unit *(continued)*

Country	Unit	year	mean rate	lower rate	upper rate
Nigeria	Kontogur	2000-2017	-0.080	-0.003	0.061
Nigeria	Kosofe	2000	0.029	0.112	0.290
Nigeria	Kosofe	2017	0.005	0.026	0.054
Nigeria	Kosofe	2000-2017	-0.168	-0.076	0.025
Nigeria	Kotonkar	2000	0.172	0.655	1.188
Nigeria	Kotonkar	2017	0.030	0.142	0.316
Nigeria	Kotonkar	2000-2017	-0.233	-0.126	-0.037
Nigeria	Kubau	2000	0.530	1.305	2.299
Nigeria	Kubau	2017	0.077	0.362	0.864
Nigeria	Kubau	2000-2017	-0.192	-0.093	0.007
Nigeria	Kudan	2000	0.345	1.137	2.006
Nigeria	Kudan	2017	0.103	0.426	1.023
Nigeria	Kudan	2000-2017	-0.186	-0.074	0.015
Nigeria	Kuje	2000	0.191	0.485	0.854
Nigeria	Kuje	2017	0.031	0.128	0.297
Nigeria	Kuje	2000-2017	-0.198	-0.090	-0.008
Nigeria	Kukawa	2000	0.046	0.271	0.657
Nigeria	Kukawa	2017	0.014	0.115	0.383
Nigeria	Kukawa	2000-2017	-0.197	-0.049	0.110
Nigeria	Kumbotso	2000	0.095	0.529	1.104
Nigeria	Kumbotso	2017	0.042	0.166	0.409
Nigeria	Kumbotso	2000-2017	-0.200	-0.104	0.013
Nigeria	Kunchi	2000	0.154	0.735	1.590
Nigeria	Kunchi	2017	0.083	0.300	0.775
Nigeria	Kunchi	2000-2017	-0.194	-0.100	-0.014
Nigeria	Kura	2000	0.138	0.883	1.955
Nigeria	Kura	2017	0.060	0.237	0.650
Nigeria	Kura	2000-2017	-0.217	-0.106	0.002
Nigeria	Kurfi	2000	0.084	0.605	1.370
Nigeria	Kurfi	2017	0.084	0.319	0.837
Nigeria	Kurfi	2000-2017	-0.199	-0.074	0.031
Nigeria	Kurmi	2000	0.086	0.396	0.854
Nigeria	Kurmi	2017	0.075	0.268	0.568
Nigeria	Kurmi	2000-2017	-0.143	-0.036	0.035
Nigeria	Kusada	2000	0.220	0.871	1.833
Nigeria	Kusada	2017	0.096	0.366	0.908
Nigeria	Kusada	2000-2017	-0.200	-0.096	-0.008
Nigeria	Kwali	2000	0.199	0.516	0.916
Nigeria	Kwali	2017	0.028	0.126	0.281
Nigeria	Kwali	2000-2017	-0.199	-0.095	-0.016
Nigeria	Kwami	2000	0.132	0.739	1.805
Nigeria	Kwami	2017	0.125	0.471	1.047
Nigeria	Kwami	2000-2017	-0.117	-0.040	0.044
Nigeria	Kwande	2000	0.076	0.374	0.831
Nigeria	Kwande	2017	0.041	0.207	0.522
Nigeria	Kwande	2000-2017	-0.198	-0.071	0.007
Nigeria	Kware	2000	0.036	0.275	0.851
Nigeria	Kware	2017	0.145	0.406	0.675
Nigeria	Kware	2000-2017	-0.047	0.037	0.136
Nigeria	Kwaya Kusar	2000	0.130	0.799	1.732
Nigeria	Kwaya Kusar	2017	0.162	0.673	1.264
Nigeria	Kwaya Kusar	2000-2017	-0.140	-0.015	0.062
Nigeria	Lafia	2000	0.172	0.589	1.160
Nigeria	Lafia	2017	0.093	0.328	0.706
Nigeria	Lafia	2000-2017	-0.153	-0.055	0.025
Nigeria	Lagelu	2000	0.363	0.659	1.126
Nigeria	Lagelu	2017	0.064	0.213	0.462
Nigeria	Lagelu	2000-2017	-0.181	-0.095	-0.023
Nigeria	LagosIsland	2000	0.027	0.097	0.250
Nigeria	LagosIsland	2017	0.006	0.026	0.053
Nigeria	LagosIsland	2000-2017	-0.165	-0.076	0.035

Table 3: Malaria DALYs rate by unit *(continued)*

Country	Unit	year	mean rate	lower rate	upper rate
Nigeria	Lake Chad	2000	0.113	0.602	1.302
Nigeria	Lake Chad	2017	0.034	0.267	0.819
Nigeria	Lake Chad	2000-2017	-0.152	-0.030	0.117
Nigeria	Lamurde	2000	0.113	0.739	1.483
Nigeria	Lamurde	2017	0.230	0.619	1.054
Nigeria	Lamurde	2000-2017	-0.068	-0.005	0.050
Nigeria	Langtang North	2000	0.208	0.911	1.930
Nigeria	Langtang North	2017	0.164	0.521	1.179
Nigeria	Langtang North	2000-2017	-0.162	-0.063	0.006
Nigeria	Langtang South	2000	0.191	0.740	1.646
Nigeria	Langtang South	2017	0.101	0.361	0.795
Nigeria	Langtang South	2000-2017	-0.167	-0.069	0.022
Nigeria	Lapai	2000	0.236	0.517	0.980
Nigeria	Lapai	2017	0.032	0.138	0.333
Nigeria	Lapai	2000-2017	-0.185	-0.092	-0.018
Nigeria	Lau	2000	0.079	0.685	1.507
Nigeria	Lau	2017	0.218	0.501	0.959
Nigeria	Lau	2000-2017	-0.098	-0.022	0.051
Nigeria	Lavun	2000	0.151	0.427	0.953
Nigeria	Lavun	2017	0.048	0.179	0.384
Nigeria	Lavun	2000-2017	-0.179	-0.071	0.016
Nigeria	Lere	2000	0.552	1.068	1.839
Nigeria	Lere	2017	0.101	0.318	0.791
Nigeria	Lere	2000-2017	-0.196	-0.097	-0.019
Nigeria	Logo	2000	0.058	0.334	0.713
Nigeria	Logo	2017	0.025	0.125	0.344
Nigeria	Logo	2000-2017	-0.230	-0.100	0.001
Nigeria	Lokoja	2000	0.120	0.419	0.744
Nigeria	Lokoja	2017	0.017	0.085	0.224
Nigeria	Lokoja	2000-2017	-0.240	-0.137	-0.035
Nigeria	Machina	2000	0.033	0.530	1.527
Nigeria	Machina	2017	0.049	0.266	0.574
Nigeria	Machina	2000-2017	-0.128	-0.014	0.115
Nigeria	Madagali	2000	0.084	0.457	1.201
Nigeria	Madagali	2017	0.038	0.234	0.689
Nigeria	Madagali	2000-2017	-0.224	-0.080	0.029
Nigeria	Madobi	2000	0.170	0.970	2.062
Nigeria	Madobi	2017	0.078	0.291	0.759
Nigeria	Madobi	2000-2017	-0.202	-0.101	0.014
Nigeria	Mafa	2000	0.082	0.374	1.057
Nigeria	Mafa	2017	0.022	0.158	0.438
Nigeria	Mafa	2000-2017	-0.218	-0.077	0.067
Nigeria	Magama	2000	0.151	0.628	1.350
Nigeria	Magama	2017	0.292	0.647	1.048
Nigeria	Magama	2000-2017	-0.064	0.000	0.072
Nigeria	Magumeri	2000	0.048	0.309	0.902
Nigeria	Magumeri	2017	0.020	0.121	0.300
Nigeria	Magumeri	2000-2017	-0.233	-0.091	0.007
Nigeria	Mai'Adua	2000	0.117	0.597	1.454
Nigeria	Mai'Adua	2017	0.051	0.243	0.657
Nigeria	Mai'Adua	2000-2017	-0.204	-0.100	0.009
Nigeria	Maidugur	2000	0.029	0.154	0.695
Nigeria	Maidugur	2017	0.007	0.041	0.120
Nigeria	Maidugur	2000-2017	-0.241	-0.123	0.033
Nigeria	Maigatari	2000	0.079	0.899	2.208
Nigeria	Maigatari	2017	0.114	0.469	0.962
Nigeria	Maigatari	2000-2017	-0.121	-0.019	0.085
Nigeria	Maiha	2000	0.142	0.541	1.217
Nigeria	Maiha	2017	0.059	0.300	0.675
Nigeria	Maiha	2000-2017	-0.128	-0.039	0.044
Nigeria	Mainland	2000	0.015	0.062	0.170

Table 3: Malaria DALYs rate by unit *(continued)*

Country	Unit	year	mean rate	lower rate	upper rate
Nigeria	Mainland	2017	0.004	0.019	0.040
Nigeria	Mainland	2000-2017	-0.159	-0.069	0.052
Nigeria	Maiyama	2000	0.062	0.676	1.653
Nigeria	Maiyama	2017	0.289	0.761	1.347
Nigeria	Maiyama	2000-2017	-0.078	-0.005	0.065
Nigeria	Makarfi	2000	0.313	1.117	1.929
Nigeria	Makarfi	2017	0.087	0.393	0.947
Nigeria	Makarfi	2000-2017	-0.193	-0.077	0.018
Nigeria	Makoda	2000	0.101	0.651	1.500
Nigeria	Makoda	2017	0.072	0.269	0.741
Nigeria	Makoda	2000-2017	-0.194	-0.103	-0.013
Nigeria	Makurdi	2000	0.043	0.269	0.540
Nigeria	Makurdi	2017	0.016	0.086	0.229
Nigeria	Makurdi	2000-2017	-0.238	-0.115	0.020
Nigeria	MalamMad	2000	0.041	0.694	1.664
Nigeria	MalamMad	2017	0.094	0.344	0.707
Nigeria	MalamMad	2000-2017	-0.122	-0.009	0.108
Nigeria	Malumfashi	2000	0.247	0.971	2.020
Nigeria	Malumfashi	2017	0.095	0.445	1.059
Nigeria	Malumfashi	2000-2017	-0.181	-0.076	0.010
Nigeria	Mangu	2000	0.214	0.959	2.149
Nigeria	Mangu	2017	0.098	0.424	1.095
Nigeria	Mangu	2000-2017	-0.207	-0.090	0.004
Nigeria	Mani	2000	0.096	0.570	1.300
Nigeria	Mani	2017	0.070	0.286	0.771
Nigeria	Mani	2000-2017	-0.199	-0.090	0.019
Nigeria	Maradun	2000	0.067	0.530	1.370
Nigeria	Maradun	2017	0.181	0.566	0.979
Nigeria	Maradun	2000-2017	-0.067	0.007	0.081
Nigeria	Mariga	2000	0.190	0.739	1.548
Nigeria	Mariga	2017	0.273	0.609	1.079
Nigeria	Mariga	2000-2017	-0.059	-0.005	0.057
Nigeria	Marte	2000	0.083	0.321	0.819
Nigeria	Marte	2017	0.017	0.150	0.429
Nigeria	Marte	2000-2017	-0.206	-0.043	0.093
Nigeria	Maru	2000	0.173	0.723	1.456
Nigeria	Maru	2017	0.315	0.686	1.186
Nigeria	Maru	2000-2017	-0.065	-0.001	0.056
Nigeria	Mashegu	2000	0.167	0.515	1.028
Nigeria	Mashegu	2017	0.148	0.395	0.688
Nigeria	Mashegu	2000-2017	-0.093	-0.019	0.034
Nigeria	Mashi	2000	0.112	0.656	1.483
Nigeria	Mashi	2017	0.078	0.324	0.948
Nigeria	Mashi	2000-2017	-0.200	-0.090	0.021
Nigeria	Matazu	2000	0.201	0.913	1.852
Nigeria	Matazu	2017	0.109	0.425	1.036
Nigeria	Matazu	2000-2017	-0.181	-0.080	0.018
Nigeria	Mayo-Bel	2000	0.112	0.896	1.773
Nigeria	Mayo-Bel	2017	0.238	0.627	1.135
Nigeria	Mayo-Bel	2000-2017	-0.071	-0.007	0.052
Nigeria	Mbaitoli	2000	0.321	0.612	0.976
Nigeria	Mbaitoli	2017	0.018	0.066	0.147
Nigeria	Mbaitoli	2000-2017	-0.294	-0.193	-0.090
Nigeria	Mbo	2000	0.421	0.876	1.408
Nigeria	Mbo	2017	0.047	0.204	0.492
Nigeria	Mbo	2000-2017	-0.237	-0.124	-0.019
Nigeria	Michika	2000	0.070	0.400	1.175
Nigeria	Michika	2017	0.038	0.191	0.483
Nigeria	Michika	2000-2017	-0.194	-0.067	0.036
Nigeria	Miga	2000	0.069	0.804	1.971
Nigeria	Miga	2017	0.095	0.343	0.838

Table 3: Malaria DALYs rate by unit *(continued)*

Country	Unit	year	mean rate	lower rate	upper rate
Nigeria	Miga	2000-2017	-0.174	-0.044	0.090
Nigeria	Mikang	2000	0.275	0.981	2.002
Nigeria	Mikang	2017	0.161	0.513	1.049
Nigeria	Mikang	2000-2017	-0.163	-0.068	0.000
Nigeria	Minjibir	2000	0.151	0.844	1.946
Nigeria	Minjibir	2017	0.103	0.350	0.981
Nigeria	Minjibir	2000-2017	-0.176	-0.087	0.021
Nigeria	Misau	2000	0.116	0.713	1.820
Nigeria	Misau	2017	0.085	0.338	0.983
Nigeria	Misau	2000-2017	-0.176	-0.073	0.022
Nigeria	Mkpat Enin	2000	0.369	0.688	1.142
Nigeria	Mkpat Enin	2017	0.019	0.089	0.196
Nigeria	Mkpat Enin	2000-2017	-0.268	-0.164	-0.062
Nigeria	Moba	2000	0.105	0.608	1.160
Nigeria	Moba	2017	0.053	0.206	0.555
Nigeria	Moba	2000-2017	-0.209	-0.102	-0.018
Nigeria	Mobbar	2000	0.027	0.304	0.835
Nigeria	Mobbar	2017	0.016	0.121	0.337
Nigeria	Mobbar	2000-2017	-0.198	-0.077	0.057
Nigeria	Mokwa	2000	0.128	0.424	0.781
Nigeria	Mokwa	2017	0.056	0.204	0.427
Nigeria	Mokwa	2000-2017	-0.178	-0.072	0.006
Nigeria	Monguno	2000	0.041	0.290	0.727
Nigeria	Monguno	2017	0.011	0.124	0.368
Nigeria	Monguno	2000-2017	-0.238	-0.072	0.067
Nigeria	Mopa-Muro	2000	0.143	0.449	0.938
Nigeria	Mopa-Muro	2017	0.030	0.114	0.280
Nigeria	Mopa-Muro	2000-2017	-0.226	-0.126	-0.032
Nigeria	Moro	2000	0.154	0.407	0.752
Nigeria	Moro	2017	0.035	0.169	0.355
Nigeria	Moro	2000-2017	-0.196	-0.085	-0.010
Nigeria	Mubi North	2000	0.059	0.386	1.149
Nigeria	Mubi North	2017	0.043	0.191	0.478
Nigeria	Mubi North	2000-2017	-0.156	-0.049	0.042
Nigeria	Mubi South	2000	0.071	0.388	1.160
Nigeria	Mubi South	2017	0.039	0.187	0.466
Nigeria	Mubi South	2000-2017	-0.157	-0.050	0.033
Nigeria	Musawa	2000	0.231	1.024	2.061
Nigeria	Musawa	2017	0.103	0.465	1.129
Nigeria	Musawa	2000-2017	-0.179	-0.079	0.021
Nigeria	Mushin	2000	0.018	0.074	0.200
Nigeria	Mushin	2017	0.004	0.021	0.043
Nigeria	Mushin	2000-2017	-0.161	-0.070	0.037
Nigeria	Muya	2000	0.376	0.714	1.252
Nigeria	Muya	2017	0.064	0.212	0.479
Nigeria	Muya	2000-2017	-0.164	-0.081	-0.008
Nigeria	Nafada	2000	0.108	0.643	1.718
Nigeria	Nafada	2017	0.099	0.420	0.922
Nigeria	Nafada	2000-2017	-0.150	-0.043	0.049
Nigeria	Nangere	2000	0.066	0.599	1.480
Nigeria	Nangere	2017	0.099	0.373	0.885
Nigeria	Nangere	2000-2017	-0.198	-0.046	0.046
Nigeria	Nasarawa	2000	0.208	0.530	0.917
Nigeria	Nasarawa	2017	0.039	0.159	0.417
Nigeria	Nasarawa	2000-2017	-0.191	-0.087	-0.001
Nigeria	Nassaraw	2000	0.048	0.282	0.657
Nigeria	Nassaraw	2017	0.018	0.079	0.201
Nigeria	Nassaraw	2000-2017	-0.220	-0.114	0.014
Nigeria	Nassarawa Egon	2000	0.199	0.530	1.004
Nigeria	Nassarawa Egon	2017	0.076	0.264	0.580
Nigeria	Nassarawa Egon	2000-2017	-0.151	-0.066	0.012

Table 3: Malaria DALYs rate by unit *(continued)*

Country	Unit	year	mean rate	lower rate	upper rate
Nigeria	Ndokwa East	2000	0.190	0.524	1.101
Nigeria	Ndokwa East	2017	0.011	0.051	0.140
Nigeria	Ndokwa East	2000-2017	-0.286	-0.187	-0.093
Nigeria	Ndokwa West	2000	0.139	0.519	1.305
Nigeria	Ndokwa West	2017	0.012	0.065	0.203
Nigeria	Ndokwa West	2000-2017	-0.275	-0.166	-0.065
Nigeria	Nembe	2000	0.283	0.840	1.540
Nigeria	Nembe	2017	0.046	0.221	0.568
Nigeria	Nembe	2000-2017	-0.218	-0.101	-0.023
Nigeria	Ngala	2000	0.091	0.389	0.914
Nigeria	Ngala	2017	0.023	0.211	0.600
Nigeria	Ngala	2000-2017	-0.177	-0.014	0.116
Nigeria	Nganzai	2000	0.036	0.261	0.752
Nigeria	Nganzai	2017	0.007	0.097	0.290
Nigeria	Nganzai	2000-2017	-0.291	-0.098	0.016
Nigeria	Ngaski	2000	0.141	0.671	1.419
Nigeria	Ngaski	2017	0.387	0.715	1.122
Nigeria	Ngaski	2000-2017	-0.038	0.003	0.063
Nigeria	Ngor-Okp	2000	0.500	0.932	1.533
Nigeria	Ngor-Okp	2017	0.021	0.083	0.203
Nigeria	Ngor-Okp	2000-2017	-0.311	-0.205	-0.102
Nigeria	Nguru	2000	0.020	0.469	1.384
Nigeria	Nguru	2017	0.050	0.231	0.551
Nigeria	Nguru	2000-2017	-0.161	-0.043	0.077
Nigeria	Ningi	2000	0.268	0.986	2.005
Nigeria	Ningi	2017	0.041	0.215	0.612
Nigeria	Ningi	2000-2017	-0.259	-0.149	-0.048
Nigeria	Njaba	2000	0.406	0.761	1.232
Nigeria	Njaba	2017	0.023	0.083	0.173
Nigeria	Njaba	2000-2017	-0.291	-0.190	-0.097
Nigeria	Njikoka	2000	0.189	0.521	1.029
Nigeria	Njikoka	2017	0.017	0.064	0.155
Nigeria	Njikoka	2000-2017	-0.285	-0.191	-0.102
Nigeria	Nkanu East	2000	0.215	0.610	1.294
Nigeria	Nkanu East	2017	0.026	0.140	0.357
Nigeria	Nkanu East	2000-2017	-0.245	-0.135	-0.059
Nigeria	Nkanu West	2000	0.087	0.310	0.674
Nigeria	Nkanu West	2017	0.010	0.070	0.180
Nigeria	Nkanu West	2000-2017	-0.264	-0.146	-0.060
Nigeria	Nkwerre	2000	0.524	0.969	1.572
Nigeria	Nkwerre	2017	0.029	0.122	0.265
Nigeria	Nkwerre	2000-2017	-0.292	-0.179	-0.083
Nigeria	NnewiNort	2000	0.195	0.500	0.895
Nigeria	NnewiNort	2017	0.012	0.050	0.125
Nigeria	NnewiNort	2000-2017	-0.297	-0.202	-0.097
Nigeria	NnewiSou	2000	0.364	0.754	1.308
Nigeria	NnewiSou	2017	0.020	0.086	0.217
Nigeria	NnewiSou	2000-2017	-0.294	-0.192	-0.083
Nigeria	Nsit Atai	2000	0.444	0.829	1.364
Nigeria	Nsit Atai	2017	0.034	0.145	0.352
Nigeria	Nsit Atai	2000-2017	-0.251	-0.149	-0.037
Nigeria	Nsit Ibom	2000	0.337	0.645	1.055
Nigeria	Nsit Ibom	2017	0.019	0.091	0.219
Nigeria	Nsit Ibom	2000-2017	-0.278	-0.162	-0.050
Nigeria	Nsit Ubium	2000	0.370	0.688	1.119
Nigeria	Nsit Ubium	2017	0.026	0.109	0.250
Nigeria	Nsit Ubium	2000-2017	-0.248	-0.153	-0.040
Nigeria	Nsukka	2000	0.050	0.292	0.750
Nigeria	Nsukka	2017	0.012	0.064	0.184
Nigeria	Nsukka	2000-2017	-0.234	-0.136	-0.014
Nigeria	Numan	2000	0.114	0.755	1.497

Table 3: Malaria DALYs rate by unit *(continued)*

Country	Unit	year	mean rate	lower rate	upper rate
Nigeria	Numan	2017	0.235	0.672	1.141
Nigeria	Numan	2000-2017	-0.072	0.001	0.049
Nigeria	Nwangele	2000	0.572	1.047	1.699
Nigeria	Nwangele	2017	0.035	0.144	0.318
Nigeria	Nwangele	2000-2017	-0.287	-0.173	-0.075
Nigeria	Obafemi-Owode	2000	0.305	0.692	1.223
Nigeria	Obafemi-Owode	2017	0.050	0.187	0.398
Nigeria	Obafemi-Owode	2000-2017	-0.161	-0.083	-0.006
Nigeria	Obanliku	2000	0.110	0.518	1.050
Nigeria	Obanliku	2017	0.062	0.316	0.778
Nigeria	Obanliku	2000-2017	-0.180	-0.068	0.018
Nigeria	Obi	2000	0.104	0.607	1.215
Nigeria	Obi	2000	0.115	0.538	1.104
Nigeria	Obi	2017	0.090	0.385	0.748
Nigeria	Obi	2017	0.080	0.317	0.678
Nigeria	Obi	2000-2017	-0.163	-0.055	0.038
Nigeria	Obi	2000-2017	-0.163	-0.050	0.015
Nigeria	Obio/Akp	2000	0.146	0.382	0.736
Nigeria	Obio/Akp	2017	0.009	0.032	0.090
Nigeria	Obio/Akp	2000-2017	-0.310	-0.205	-0.069
Nigeria	Obokun	2000	0.239	0.796	1.438
Nigeria	Obokun	2017	0.103	0.331	0.732
Nigeria	Obokun	2000-2017	-0.179	-0.083	-0.010
Nigeria	Oboma Ngwa	2000	0.354	0.670	1.067
Nigeria	Oboma Ngwa	2017	0.020	0.085	0.197
Nigeria	Oboma Ngwa	2000-2017	-0.296	-0.183	-0.085
Nigeria	Obot Akara	2000	0.417	0.789	1.322
Nigeria	Obot Akara	2017	0.024	0.122	0.258
Nigeria	Obot Akara	2000-2017	-0.265	-0.157	-0.064
Nigeria	Obowo	2000	0.402	0.758	1.243
Nigeria	Obowo	2017	0.020	0.094	0.193
Nigeria	Obowo	2000-2017	-0.292	-0.177	-0.080
Nigeria	Obubra	2000	0.237	0.793	1.411
Nigeria	Obubra	2017	0.091	0.307	0.692
Nigeria	Obubra	2000-2017	-0.193	-0.094	-0.021
Nigeria	Obudu	2000	0.127	0.604	1.247
Nigeria	Obudu	2017	0.073	0.350	0.854
Nigeria	Obudu	2000-2017	-0.173	-0.068	0.015
Nigeria	Odeda	2000	0.518	0.961	1.621
Nigeria	Odeda	2017	0.130	0.372	0.721
Nigeria	Odeda	2000-2017	-0.157	-0.072	-0.010
Nigeria	Odigbo	2000	0.506	1.091	1.870
Nigeria	Odigbo	2017	0.112	0.423	0.924
Nigeria	Odigbo	2000-2017	-0.181	-0.093	-0.023
Nigeria	Odo0tin	2000	0.207	0.674	1.168
Nigeria	Odo0tin	2017	0.058	0.228	0.551
Nigeria	Odo0tin	2000-2017	-0.233	-0.106	-0.027
Nigeria	Odogbolu	2000	0.298	0.783	1.399
Nigeria	Odogbolu	2017	0.050	0.177	0.445
Nigeria	Odogbolu	2000-2017	-0.174	-0.100	-0.019
Nigeria	Odukpani	2000	0.541	1.027	1.695
Nigeria	Odukpani	2017	0.037	0.149	0.353
Nigeria	Odukpani	2000-2017	-0.261	-0.158	-0.050
Nigeria	Offa	2000	0.093	0.314	0.590
Nigeria	Offa	2017	0.025	0.101	0.262
Nigeria	Offa	2000-2017	-0.220	-0.108	-0.026
Nigeria	Ofu	2000	0.150	0.527	1.140
Nigeria	Ofu	2017	0.021	0.093	0.206
Nigeria	Ofu	2000-2017	-0.271	-0.161	-0.071
Nigeria	Ogba/Egbe	2000	0.131	0.326	0.645
Nigeria	Ogba/Egbe	2017	0.004	0.018	0.042

Table 3: Malaria DALYs rate by unit *(continued)*

Country	Unit	year	mean rate	lower rate	upper rate
Nigeria	Ogba/Egbe	2000-2017	-0.310	-0.216	-0.108
Nigeria	Ogbadibo	2000	0.114	0.533	1.074
Nigeria	Ogbadibo	2017	0.037	0.190	0.412
Nigeria	Ogbadibo	2000-2017	-0.206	-0.100	-0.011
Nigeria	Ogbaru	2000	0.362	0.834	1.536
Nigeria	Ogbaru	2017	0.019	0.083	0.199
Nigeria	Ogbaru	2000-2017	-0.288	-0.196	-0.095
Nigeria	Ogbia	2000	0.162	0.609	1.161
Nigeria	Ogbia	2017	0.034	0.143	0.344
Nigeria	Ogbia	2000-2017	-0.223	-0.110	-0.025
Nigeria	Ogbomosho North	2000	0.135	0.341	0.587
Nigeria	Ogbomosho North	2017	0.024	0.118	0.290
Nigeria	Ogbomosho North	2000-2017	-0.194	-0.100	-0.026
Nigeria	Ogbomosho South	2000	0.146	0.389	0.652
Nigeria	Ogbomosho South	2017	0.028	0.134	0.322
Nigeria	Ogbomosho South	2000-2017	-0.191	-0.098	-0.023
Nigeria	Ogo-Oluw	2000	0.331	0.777	1.271
Nigeria	Ogo-Oluw	2017	0.082	0.313	0.661
Nigeria	Ogo-Oluw	2000-2017	-0.181	-0.085	-0.014
Nigeria	Ogoja	2000	0.124	0.546	1.064
Nigeria	Ogoja	2017	0.090	0.309	0.670
Nigeria	Ogoja	2000-2017	-0.166	-0.063	0.016
Nigeria	Ogori/Magongo	2000	0.127	0.640	1.235
Nigeria	Ogori/Magongo	2017	0.029	0.125	0.282
Nigeria	Ogori/Magongo	2000-2017	-0.286	-0.166	-0.057
Nigeria	Ogu/Bolo	2000	0.359	0.701	1.133
Nigeria	Ogu/Bolo	2017	0.028	0.094	0.213
Nigeria	Ogu/Bolo	2000-2017	-0.275	-0.165	-0.049
Nigeria	OgunWaterside	2000	0.368	1.048	1.883
Nigeria	OgunWaterside	2017	0.050	0.204	0.546
Nigeria	OgunWaterside	2000-2017	-0.225	-0.137	-0.035
Nigeria	Oguta	2000	0.328	0.576	0.972
Nigeria	Oguta	2017	0.011	0.041	0.099
Nigeria	Oguta	2000-2017	-0.301	-0.215	-0.127
Nigeria	Ohafia Abia	2000	0.550	1.026	1.705
Nigeria	Ohafia Abia	2017	0.042	0.154	0.347
Nigeria	Ohafia Abia	2000-2017	-0.241	-0.158	-0.060
Nigeria	Ohaji/Eg	2000	0.339	0.613	0.998
Nigeria	Ohaji/Eg	2017	0.009	0.034	0.085
Nigeria	Ohaji/Eg	2000-2017	-0.314	-0.225	-0.127
Nigeria	Ohaozara	2000	0.266	0.605	1.160
Nigeria	Ohaozara	2017	0.034	0.133	0.319
Nigeria	Ohaozara	2000-2017	-0.219	-0.136	-0.056
Nigeria	Ohaukwu	2000	0.174	0.476	0.921
Nigeria	Ohaukwu	2017	0.036	0.171	0.388
Nigeria	Ohaukwu	2000-2017	-0.215	-0.103	-0.027
Nigeria	Ohimini	2000	0.106	0.489	1.027
Nigeria	Ohimini	2017	0.060	0.250	0.513
Nigeria	Ohimini	2000-2017	-0.160	-0.071	0.006
Nigeria	Oji-River	2000	0.246	0.658	1.325
Nigeria	Oji-River	2017	0.016	0.099	0.238
Nigeria	Oji-River	2000-2017	-0.300	-0.176	-0.091
Nigeria	Ojo	2000	0.038	0.130	0.279
Nigeria	Ojo	2017	0.012	0.046	0.100
Nigeria	Ojo	2000-2017	-0.140	-0.043	0.050
Nigeria	Oju	2000	0.091	0.476	0.977
Nigeria	Oju	2017	0.070	0.277	0.564
Nigeria	Oju	2000-2017	-0.160	-0.056	0.013
Nigeria	Oke-Ero	2000	0.087	0.509	0.997
Nigeria	Oke-Ero	2017	0.034	0.168	0.469
Nigeria	Oke-Ero	2000-2017	-0.227	-0.105	-0.012

Table 3: Malaria DALYs rate by unit *(continued)*

Country	Unit	year	mean rate	lower rate	upper rate
Nigeria	Okehi	2000	0.089	0.370	0.740
Nigeria	Okehi	2017	0.013	0.060	0.130
Nigeria	Okehi	2000-2017	-0.294	-0.177	-0.069
Nigeria	Okene	2000	0.104	0.509	0.970
Nigeria	Okene	2017	0.019	0.086	0.191
Nigeria	Okene	2000-2017	-0.299	-0.176	-0.061
Nigeria	Okigwe	2000	0.562	1.027	1.677
Nigeria	Okigwe	2017	0.022	0.130	0.308
Nigeria	Okigwe	2000-2017	-0.313	-0.183	-0.071
Nigeria	Okitipupa	2000	0.374	0.932	1.699
Nigeria	Okitipupa	2017	0.055	0.254	0.634
Nigeria	Okitipupa	2000-2017	-0.218	-0.129	-0.036
Nigeria	Okobo	2000	0.450	0.837	1.384
Nigeria	Okobo	2017	0.040	0.155	0.372
Nigeria	Okobo	2000-2017	-0.242	-0.143	-0.031
Nigeria	Okpe	2000	0.105	0.522	1.118
Nigeria	Okpe	2017	0.034	0.134	0.326
Nigeria	Okpe	2000-2017	-0.209	-0.111	-0.031
Nigeria	Okpokwu	2000	0.134	0.519	1.016
Nigeria	Okpokwu	2017	0.050	0.230	0.463
Nigeria	Okpokwu	2000-2017	-0.186	-0.083	-0.007
Nigeria	Okrika	2000	0.250	0.503	0.851
Nigeria	Okrika	2017	0.021	0.068	0.162
Nigeria	Okrika	2000-2017	-0.274	-0.166	-0.048
Nigeria	Ola-Oluwa	2000	0.425	0.944	1.615
Nigeria	Ola-Oluwa	2017	0.097	0.367	0.785
Nigeria	Ola-Oluwa	2000-2017	-0.192	-0.091	-0.019
Nigeria	Olamabor	2000	0.109	0.451	0.904
Nigeria	Olamabor	2017	0.033	0.147	0.312
Nigeria	Olamabor	2000-2017	-0.199	-0.108	-0.025
Nigeria	Olorunda	2000	0.108	0.489	0.902
Nigeria	Olorunda	2017	0.033	0.143	0.332
Nigeria	Olorunda	2000-2017	-0.231	-0.114	-0.019
Nigeria	Olorunsogo	2000	0.161	0.476	0.838
Nigeria	Olorunsogo	2017	0.062	0.251	0.508
Nigeria	Olorunsogo	2000-2017	-0.148	-0.053	0.009
Nigeria	Oluyole	2000	0.439	0.796	1.279
Nigeria	Oluyole	2017	0.073	0.250	0.518
Nigeria	Oluyole	2000-2017	-0.188	-0.094	-0.027
Nigeria	Omala	2000	0.171	0.497	0.977
Nigeria	Omala	2017	0.033	0.153	0.389
Nigeria	Omala	2000-2017	-0.217	-0.107	-0.014
Nigeria	Omumma	2000	0.372	0.700	1.137
Nigeria	Omumma	2017	0.017	0.068	0.163
Nigeria	Omumma	2000-2017	-0.303	-0.206	-0.098
Nigeria	Ona-Ara	2000	0.379	0.694	1.153
Nigeria	Ona-Ara	2017	0.063	0.218	0.429
Nigeria	Ona-Ara	2000-2017	-0.176	-0.092	-0.026
Nigeria	Ondo East	2000	0.506	1.291	2.211
Nigeria	Ondo East	2017	0.205	0.739	1.385
Nigeria	Ondo East	2000-2017	-0.130	-0.052	0.006
Nigeria	Ondo West	2000	0.414	1.040	1.740
Nigeria	Ondo West	2017	0.129	0.517	1.050
Nigeria	Ondo West	2000-2017	-0.155	-0.069	-0.005
Nigeria	Onicha	2000	0.262	0.756	1.504
Nigeria	Onicha	2017	0.053	0.205	0.518
Nigeria	Onicha	2000-2017	-0.204	-0.118	-0.038
Nigeria	Onitsha North	2000	0.066	0.264	0.571
Nigeria	Onitsha North	2017	0.006	0.022	0.056
Nigeria	Onitsha North	2000-2017	-0.316	-0.224	-0.145
Nigeria	Onitsha South	2000	0.156	0.528	1.035

Table 3: Malaria DALYs rate by unit *(continued)*

Country	Unit	year	mean rate	lower rate	upper rate
Nigeria	Onitsha South	2017	0.014	0.050	0.122
Nigeria	Onitsha South	2000-2017	-0.309	-0.208	-0.120
Nigeria	Onna	2000	0.320	0.584	0.950
Nigeria	Onna	2017	0.019	0.080	0.169
Nigeria	Onna	2000-2017	-0.258	-0.158	-0.056
Nigeria	Opobo/Nkoro	2000	0.549	1.038	1.704
Nigeria	Opobo/Nkoro	2017	0.027	0.126	0.284
Nigeria	Opobo/Nkoro	2000-2017	-0.291	-0.170	-0.053
Nigeria	Oredo Edo	2000	0.167	0.494	1.147
Nigeria	Oredo Edo	2017	0.045	0.182	0.382
Nigeria	Oredo Edo	2000-2017	-0.215	-0.109	-0.019
Nigeria	Orelope	2000	0.129	0.433	0.804
Nigeria	Orelope	2017	0.092	0.274	0.495
Nigeria	Orelope	2000-2017	-0.133	-0.037	0.028
Nigeria	Orhionmw	2000	0.193	0.714	1.696
Nigeria	Orhionmw	2017	0.047	0.194	0.473
Nigeria	Orhionmw	2000-2017	-0.223	-0.121	-0.035
Nigeria	Ori-Ire	2000	0.274	0.548	0.879
Nigeria	Ori-Ire	2017	0.063	0.243	0.531
Nigeria	Ori-Ire	2000-2017	-0.158	-0.074	-0.009
Nigeria	Oriade	2000	0.330	1.094	1.981
Nigeria	Oriade	2017	0.166	0.512	1.053
Nigeria	Oriade	2000-2017	-0.137	-0.067	-0.001
Nigeria	Orlu	2000	0.409	0.752	1.195
Nigeria	Orlu	2017	0.020	0.080	0.180
Nigeria	Orlu	2000-2017	-0.294	-0.196	-0.100
Nigeria	Orolu	2000	0.160	0.595	1.052
Nigeria	Orolu	2017	0.042	0.184	0.411
Nigeria	Orolu	2000-2017	-0.231	-0.111	-0.020
Nigeria	Oron	2000	0.385	0.796	1.302
Nigeria	Oron	2017	0.035	0.133	0.322
Nigeria	Oron	2000-2017	-0.252	-0.148	-0.036
Nigeria	Orsu	2000	0.515	0.895	1.484
Nigeria	Orsu	2017	0.023	0.099	0.236
Nigeria	Orsu	2000-2017	-0.293	-0.192	-0.093
Nigeria	Oru East	2000	0.407	0.753	1.200
Nigeria	Oru East	2017	0.020	0.071	0.158
Nigeria	Oru East	2000-2017	-0.295	-0.200	-0.106
Nigeria	Oru West	2000	0.467	0.833	1.357
Nigeria	Oru West	2017	0.019	0.069	0.163
Nigeria	Oru West	2000-2017	-0.302	-0.208	-0.112
Nigeria	Oruk-Ana	2000	0.393	0.745	1.246
Nigeria	Oruk-Ana	2017	0.018	0.084	0.203
Nigeria	Oruk-Ana	2000-2017	-0.304	-0.178	-0.071
Nigeria	OrumbaNo	2000	0.320	0.740	1.350
Nigeria	OrumbaNo	2017	0.021	0.103	0.252
Nigeria	OrumbaNo	2000-2017	-0.288	-0.182	-0.094
Nigeria	OrumbaSo	2000	0.516	1.013	1.779
Nigeria	OrumbaSo	2017	0.023	0.128	0.321
Nigeria	OrumbaSo	2000-2017	-0.311	-0.185	-0.091
Nigeria	Ose	2000	0.332	0.988	1.784
Nigeria	Ose	2017	0.111	0.382	0.842
Nigeria	Ose	2000-2017	-0.191	-0.100	-0.012
Nigeria	Oshimili North	2000	0.186	0.627	1.399
Nigeria	Oshimili North	2017	0.021	0.088	0.232
Nigeria	Oshimili North	2000-2017	-0.292	-0.188	-0.091
Nigeria	Oshimili South	2000	0.134	0.461	0.955
Nigeria	Oshimili South	2017	0.013	0.050	0.129
Nigeria	Oshimili South	2000-2017	-0.296	-0.199	-0.106
Nigeria	Oshodi/Isolo	2000	0.024	0.096	0.249
Nigeria	Oshodi/Isolo	2017	0.006	0.029	0.060

Table 3: Malaria DALYs rate by unit *(continued)*

Country	Unit	year	mean rate	lower rate	upper rate
Nigeria	Oshodi/Isolo	2000-2017	-0.146	-0.062	0.042
Nigeria	Osisioma Ngwa	2000	0.290	0.554	0.886
Nigeria	Osisioma Ngwa	2017	0.015	0.058	0.133
Nigeria	Osisioma Ngwa	2000-2017	-0.311	-0.203	-0.106
Nigeria	Osogbo	2000	0.156	0.618	1.081
Nigeria	Osogbo	2017	0.050	0.203	0.452
Nigeria	Osogbo	2000-2017	-0.212	-0.105	-0.016
Nigeria	Oturkpo	2000	0.101	0.435	0.847
Nigeria	Oturkpo	2017	0.050	0.207	0.424
Nigeria	Oturkpo	2000-2017	-0.151	-0.076	-0.004
Nigeria	OviaNort	2000	0.385	0.825	1.618
Nigeria	OviaNort	2017	0.115	0.353	0.690
Nigeria	OviaNort	2000-2017	-0.188	-0.091	-0.018
Nigeria	OviaSouth-West	2000	0.448	0.991	1.762
Nigeria	OviaSouth-West	2017	0.123	0.398	0.748
Nigeria	OviaSouth-West	2000-2017	-0.207	-0.096	-0.027
Nigeria	Owan East	2000	0.327	0.942	1.867
Nigeria	Owan East	2017	0.070	0.242	0.576
Nigeria	Owan East	2000-2017	-0.229	-0.134	-0.051
Nigeria	OwanWest	2000	0.327	0.993	1.983
Nigeria	OwanWest	2017	0.093	0.333	0.724
Nigeria	OwanWest	2000-2017	-0.203	-0.113	-0.029
Nigeria	Owerri Municipal	2000	0.218	0.431	0.747
Nigeria	Owerri Municipal	2017	0.005	0.020	0.047
Nigeria	Owerri Municipal	2000-2017	-0.361	-0.250	-0.141
Nigeria	Owerri North	2000	0.288	0.554	0.882
Nigeria	Owerri North	2017	0.009	0.038	0.088
Nigeria	Owerri North	2000-2017	-0.332	-0.223	-0.110
Nigeria	Owerri West	2000	0.288	0.550	0.877
Nigeria	Owerri West	2017	0.009	0.034	0.080
Nigeria	Owerri West	2000-2017	-0.331	-0.228	-0.121
Nigeria	Owo	2000	0.277	0.942	1.672
Nigeria	Owo	2017	0.101	0.391	0.849
Nigeria	Owo	2000-2017	-0.182	-0.090	-0.004
Nigeria	Oye	2000	0.140	0.621	1.081
Nigeria	Oye	2017	0.058	0.202	0.508
Nigeria	Oye	2000-2017	-0.179	-0.102	-0.012
Nigeria	Oyi	2000	0.114	0.375	0.772
Nigeria	Oyi	2017	0.010	0.040	0.100
Nigeria	Oyi	2000-2017	-0.294	-0.201	-0.116
Nigeria	Oyigbo	2000	0.257	0.496	0.803
Nigeria	Oyigbo	2017	0.014	0.053	0.119
Nigeria	Oyigbo	2000-2017	-0.289	-0.190	-0.077
Nigeria	Oyo East	2000	0.243	0.559	0.995
Nigeria	Oyo East	2017	0.050	0.203	0.483
Nigeria	Oyo East	2000-2017	-0.176	-0.090	-0.013
Nigeria	Oyo West	2000	0.244	0.519	0.935
Nigeria	Oyo West	2017	0.052	0.193	0.447
Nigeria	Oyo West	2000-2017	-0.160	-0.083	-0.013
Nigeria	Oyun	2000	0.169	0.465	0.789
Nigeria	Oyun	2017	0.045	0.175	0.411
Nigeria	Oyun	2000-2017	-0.206	-0.095	-0.023
Nigeria	Paikoro	2000	0.335	0.654	1.094
Nigeria	Paikoro	2017	0.051	0.176	0.396
Nigeria	Paikoro	2000-2017	-0.165	-0.081	-0.006
Nigeria	Pankshin	2000	0.249	0.874	1.906
Nigeria	Pankshin	2017	0.132	0.457	1.040
Nigeria	Pankshin	2000-2017	-0.170	-0.075	0.008
Nigeria	Patani	2000	0.213	0.883	1.739
Nigeria	Patani	2017	0.030	0.127	0.309
Nigeria	Patani	2000-2017	-0.237	-0.132	-0.047

Table 3: Malaria DALYs rate by unit *(continued)*

Country	Unit	year	mean rate	lower rate	upper rate
Nigeria	Pategi	2000	0.143	0.466	1.063
Nigeria	Pategi	2017	0.039	0.159	0.366
Nigeria	Pategi	2000-2017	-0.216	-0.096	-0.004
Nigeria	Port Harcourt	2000	0.138	0.381	0.753
Nigeria	Port Harcourt	2017	0.011	0.037	0.101
Nigeria	Port Harcourt	2000-2017	-0.303	-0.196	-0.061
Nigeria	Potiskum	2000	0.053	0.527	1.354
Nigeria	Potiskum	2017	0.070	0.330	0.781
Nigeria	Potiskum	2000-2017	-0.203	-0.046	0.056
Nigeria	Qua'anpa	2000	0.221	0.913	1.883
Nigeria	Qua'anpa	2017	0.103	0.423	1.011
Nigeria	Qua'anpa	2000-2017	-0.181	-0.080	0.004
Nigeria	Rabah	2000	0.058	0.524	1.454
Nigeria	Rabah	2017	0.264	0.635	1.099
Nigeria	Rabah	2000-2017	-0.049	0.022	0.115
Nigeria	Rafi	2000	0.210	0.704	1.404
Nigeria	Rafi	2017	0.104	0.369	0.705
Nigeria	Rafi	2000-2017	-0.109	-0.031	0.038
Nigeria	Rano	2000	0.221	1.118	2.280
Nigeria	Rano	2017	0.066	0.260	0.663
Nigeria	Rano	2000-2017	-0.224	-0.113	-0.006
Nigeria	Remo-North	2000	0.502	1.078	1.883
Nigeria	Remo-North	2017	0.084	0.297	0.662
Nigeria	Remo-North	2000-2017	-0.176	-0.091	-0.017
Nigeria	Rijau	2000	0.085	0.633	1.346
Nigeria	Rijau	2017	0.296	0.672	1.095
Nigeria	Rijau	2000-2017	-0.047	0.009	0.092
Nigeria	Rimi	2000	0.062	0.411	1.029
Nigeria	Rimi	2017	0.055	0.214	0.604
Nigeria	Rimi	2000-2017	-0.199	-0.089	0.019
Nigeria	RiminGad	2000	0.210	1.099	2.201
Nigeria	RiminGad	2017	0.114	0.398	0.949
Nigeria	RiminGad	2000-2017	-0.193	-0.095	0.020
Nigeria	Ringim	2000	0.099	0.782	2.139
Nigeria	Ringim	2017	0.091	0.327	0.816
Nigeria	Ringim	2000-2017	-0.174	-0.067	0.038
Nigeria	Riyom	2000	0.434	1.067	1.887
Nigeria	Riyom	2017	0.112	0.426	0.858
Nigeria	Riyom	2000-2017	-0.205	-0.078	0.000
Nigeria	Rogo	2000	0.270	1.127	2.341
Nigeria	Rogo	2017	0.088	0.436	1.038
Nigeria	Rogo	2000-2017	-0.207	-0.077	0.023
Nigeria	Roni	2000	0.088	0.518	1.180
Nigeria	Roni	2017	0.058	0.205	0.554
Nigeria	Roni	2000-2017	-0.220	-0.109	-0.014
Nigeria	Sabon Birni	2000	0.066	0.590	1.668
Nigeria	Sabon Birni	2017	0.153	0.595	1.273
Nigeria	Sabon Birni	2000-2017	-0.106	-0.001	0.086
Nigeria	Sabon-Ga	2000	0.199	0.650	1.184
Nigeria	Sabon-Ga	2017	0.045	0.210	0.496
Nigeria	Sabon-Ga	2000-2017	-0.177	-0.083	0.023
Nigeria	Sabuwa	2000	0.275	1.096	2.137
Nigeria	Sabuwa	2017	0.154	0.542	1.163
Nigeria	Sabuwa	2000-2017	-0.169	-0.057	0.038
Nigeria	Safana	2000	0.110	0.734	1.596
Nigeria	Safana	2017	0.128	0.444	0.986
Nigeria	Safana	2000-2017	-0.154	-0.055	0.044
Nigeria	Sagbama	2000	0.259	0.870	1.768
Nigeria	Sagbama	2017	0.035	0.147	0.357
Nigeria	Sagbama	2000-2017	-0.218	-0.120	-0.030
Nigeria	Sakaba	2000	0.081	0.749	1.636

Table 3: Malaria DALYs rate by unit *(continued)*

Country	Unit	year	mean rate	lower rate	upper rate
Nigeria	Sakaba	2017	0.365	0.772	1.301
Nigeria	Sakaba	2000-2017	-0.053	0.010	0.096
Nigeria	Saki East	2000	0.149	0.527	1.036
Nigeria	Saki East	2017	0.114	0.361	0.648
Nigeria	Saki East	2000-2017	-0.136	-0.028	0.056
Nigeria	Saki West	2000	0.124	0.500	0.927
Nigeria	Saki West	2017	0.104	0.328	0.587
Nigeria	Saki West	2000-2017	-0.116	-0.032	0.032
Nigeria	Sandamu	2000	0.081	0.433	1.112
Nigeria	Sandamu	2017	0.042	0.180	0.467
Nigeria	Sandamu	2000-2017	-0.220	-0.103	-0.001
Nigeria	Sanga	2000	0.325	0.589	0.986
Nigeria	Sanga	2017	0.079	0.275	0.548
Nigeria	Sanga	2000-2017	-0.158	-0.063	-0.005
Nigeria	Sapele	2000	0.081	0.471	1.054
Nigeria	Sapele	2017	0.036	0.167	0.380
Nigeria	Sapele	2000-2017	-0.216	-0.098	-0.012
Nigeria	Sardauna	2000	0.056	0.290	0.733
Nigeria	Sardauna	2017	0.046	0.198	0.424
Nigeria	Sardauna	2000-2017	-0.122	-0.031	0.071
Nigeria	Shagamu	2000	0.149	0.448	0.849
Nigeria	Shagamu	2017	0.029	0.098	0.233
Nigeria	Shagamu	2000-2017	-0.169	-0.088	-0.008
Nigeria	Shagari	2000	0.054	0.480	1.270
Nigeria	Shagari	2017	0.240	0.598	1.049
Nigeria	Shagari	2000-2017	-0.064	0.025	0.114
Nigeria	Shanga	2000	0.112	0.736	1.655
Nigeria	Shanga	2017	0.477	0.862	1.463
Nigeria	Shanga	2000-2017	-0.024	0.014	0.079
Nigeria	Shani	2000	0.145	0.828	1.829
Nigeria	Shani	2017	0.204	0.736	1.326
Nigeria	Shani	2000-2017	-0.098	-0.006	0.066
Nigeria	Shanono	2000	0.230	1.078	2.067
Nigeria	Shanono	2017	0.113	0.462	1.146
Nigeria	Shanono	2000-2017	-0.183	-0.085	0.021
Nigeria	Shelleng	2000	0.177	0.739	1.506
Nigeria	Shelleng	2017	0.207	0.659	1.109
Nigeria	Shelleng	2000-2017	-0.083	0.002	0.062
Nigeria	Shendam	2000	0.237	0.895	1.865
Nigeria	Shendam	2017	0.119	0.439	0.942
Nigeria	Shendam	2000-2017	-0.191	-0.075	-0.002
Nigeria	Shinkafi	2000	0.063	0.529	1.423
Nigeria	Shinkafi	2017	0.141	0.536	1.175
Nigeria	Shinkafi	2000-2017	-0.111	-0.001	0.077
Nigeria	Shira	2000	0.133	0.833	1.988
Nigeria	Shira	2017	0.080	0.304	0.982
Nigeria	Shira	2000-2017	-0.222	-0.107	0.010
Nigeria	Shiroro	2000	0.287	0.738	1.340
Nigeria	Shiroro	2017	0.069	0.270	0.614
Nigeria	Shiroro	2000-2017	-0.152	-0.060	0.014
Nigeria	Shomgom	2000	0.076	0.610	1.581
Nigeria	Shomgom	2017	0.122	0.354	0.712
Nigeria	Shomgom	2000-2017	-0.124	-0.049	0.028
Nigeria	Shomolu	2000	0.020	0.079	0.214
Nigeria	Shomolu	2017	0.004	0.021	0.043
Nigeria	Shomolu	2000-2017	-0.166	-0.072	0.035
Nigeria	Silame	2000	0.025	0.294	0.816
Nigeria	Silame	2017	0.185	0.426	0.696
Nigeria	Silame	2000-2017	-0.044	0.033	0.109
Nigeria	Soba	2000	0.450	1.240	2.092
Nigeria	Soba	2017	0.100	0.424	0.978

Table 3: Malaria DALYs rate by unit *(continued)*

Country	Unit	year	mean rate	lower rate	upper rate
Nigeria	Soba	2000-2017	-0.178	-0.074	0.013
Nigeria	Sokoto North	2000	0.009	0.090	0.323
Nigeria	Sokoto North	2017	0.054	0.164	0.281
Nigeria	Sokoto North	2000-2017	-0.059	0.046	0.156
Nigeria	Sokoto South	2000	0.009	0.091	0.297
Nigeria	Sokoto South	2017	0.049	0.145	0.250
Nigeria	Sokoto South	2000-2017	-0.050	0.041	0.145
Nigeria	Song	2000	0.149	0.600	1.241
Nigeria	Song	2017	0.116	0.461	0.861
Nigeria	Song	2000-2017	-0.085	-0.004	0.075
Nigeria	Southern Ijaw	2000	0.192	0.677	1.286
Nigeria	Southern Ijaw	2017	0.047	0.193	0.468
Nigeria	Southern Ijaw	2000-2017	-0.187	-0.079	0.003
Nigeria	Sule-Tan	2000	0.085	0.837	2.016
Nigeria	Sule-Tan	2017	0.088	0.411	0.927
Nigeria	Sule-Tan	2000-2017	-0.153	-0.048	0.054
Nigeria	Suleja	2000	0.179	0.374	0.650
Nigeria	Suleja	2017	0.013	0.071	0.166
Nigeria	Suleja	2000-2017	-0.223	-0.116	-0.031
Nigeria	Sumaila	2000	0.323	1.295	2.665
Nigeria	Sumaila	2017	0.064	0.283	0.762
Nigeria	Sumaila	2000-2017	-0.236	-0.133	-0.024
Nigeria	Suru	2000	0.072	0.613	1.620
Nigeria	Suru	2017	0.292	0.725	1.293
Nigeria	Suru	2000-2017	-0.092	-0.003	0.073
Nigeria	Surulere	2000	0.297	0.729	1.156
Nigeria	Surulere	2000	0.018	0.070	0.186
Nigeria	Surulere	2017	0.065	0.267	0.592
Nigeria	Surulere	2017	0.005	0.022	0.047
Nigeria	Surulere	2000-2017	-0.151	-0.064	0.053
Nigeria	Surulere	2000-2017	-0.205	-0.095	-0.022
Nigeria	Tafa	2000	0.209	0.415	0.713
Nigeria	Tafa	2017	0.015	0.082	0.183
Nigeria	Tafa	2000-2017	-0.213	-0.113	-0.023
Nigeria	Tafawa-B	2000	0.298	1.048	1.853
Nigeria	Tafawa-B	2017	0.110	0.431	1.085
Nigeria	Tafawa-B	2000-2017	-0.216	-0.092	-0.014
Nigeria	Tai	2000	0.274	0.524	0.856
Nigeria	Tai	2017	0.017	0.061	0.134
Nigeria	Tai	2000-2017	-0.286	-0.180	-0.062
Nigeria	Takai	2000	0.241	1.162	2.686
Nigeria	Takai	2017	0.071	0.290	0.796
Nigeria	Takai	2000-2017	-0.232	-0.127	-0.026
Nigeria	Takum	2000	0.069	0.390	0.756
Nigeria	Takum	2017	0.043	0.221	0.561
Nigeria	Takum	2000-2017	-0.175	-0.053	0.039
Nigeria	Talata-Mafara	2000	0.059	0.458	1.136
Nigeria	Talata-Mafara	2017	0.183	0.529	0.934
Nigeria	Talata-Mafara	2000-2017	-0.051	0.014	0.109
Nigeria	Tambawal	2000	0.063	0.432	1.219
Nigeria	Tambawal	2017	0.190	0.489	0.887
Nigeria	Tambawal	2000-2017	-0.079	0.001	0.059
Nigeria	Tangazar	2000	0.085	0.568	1.834
Nigeria	Tangazar	2017	0.295	0.757	1.313
Nigeria	Tangazar	2000-2017	-0.059	0.029	0.118
Nigeria	Tarauni	2000	0.051	0.302	0.679
Nigeria	Tarauni	2017	0.019	0.086	0.217
Nigeria	Tarauni	2000-2017	-0.223	-0.115	0.010
Nigeria	Tarka	2000	0.057	0.326	0.668
Nigeria	Tarka	2017	0.024	0.109	0.274
Nigeria	Tarka	2000-2017	-0.239	-0.108	-0.013

Table 3: Malaria DALYs rate by unit *(continued)*

Country	Unit	year	mean rate	lower rate	upper rate
Nigeria	Tarmuwa	2000	0.041	0.348	0.961
Nigeria	Tarmuwa	2017	0.047	0.215	0.507
Nigeria	Tarmuwa	2000-2017	-0.165	-0.052	0.044
Nigeria	Taura	2000	0.105	0.805	1.989
Nigeria	Taura	2017	0.081	0.331	0.720
Nigeria	Taura	2000-2017	-0.169	-0.053	0.058
Nigeria	Teungo	2000	0.078	0.412	0.844
Nigeria	Teungo	2017	0.073	0.259	0.476
Nigeria	Teungo	2000-2017	-0.108	-0.015	0.061
Nigeria	Tofa	2000	0.209	1.079	2.146
Nigeria	Tofa	2017	0.116	0.409	0.980
Nigeria	Tofa	2000-2017	-0.190	-0.096	0.020
Nigeria	Toro	2000	0.332	0.882	1.624
Nigeria	Toro	2017	0.059	0.233	0.546
Nigeria	Toro	2000-2017	-0.240	-0.123	-0.042
Nigeria	Toto	2000	0.171	0.537	0.983
Nigeria	Toto	2017	0.029	0.138	0.338
Nigeria	Toto	2000-2017	-0.220	-0.103	-0.004
Nigeria	Tsafe	2000	0.177	0.917	2.190
Nigeria	Tsafe	2017	0.204	0.682	1.368
Nigeria	Tsafe	2000-2017	-0.113	-0.036	0.065
Nigeria	Tsanyawa	2000	0.222	0.921	1.793
Nigeria	Tsanyawa	2017	0.091	0.380	1.017
Nigeria	Tsanyawa	2000-2017	-0.186	-0.092	0.010
Nigeria	Tundun Wada	2000	0.274	1.208	2.314
Nigeria	Tundun Wada	2017	0.071	0.279	0.755
Nigeria	Tundun Wada	2000-2017	-0.222	-0.111	0.007
Nigeria	Tureta	2000	0.073	0.670	1.683
Nigeria	Tureta	2017	0.325	0.813	1.419
Nigeria	Tureta	2000-2017	-0.060	0.024	0.124
Nigeria	Udenu	2000	0.053	0.336	0.783
Nigeria	Udenu	2017	0.016	0.092	0.245
Nigeria	Udenu	2000-2017	-0.231	-0.118	0.001
Nigeria	Udi	2000	0.109	0.321	0.729
Nigeria	Udi	2017	0.010	0.063	0.155
Nigeria	Udi	2000-2017	-0.260	-0.154	-0.077
Nigeria	Udu	2000	0.034	0.228	0.588
Nigeria	Udu	2017	0.007	0.039	0.101
Nigeria	Udu	2000-2017	-0.249	-0.125	-0.013
Nigeria	Udung Uko	2000	0.394	0.810	1.339
Nigeria	Udung Uko	2017	0.044	0.174	0.422
Nigeria	Udung Uko	2000-2017	-0.239	-0.133	-0.024
Nigeria	Ughelli North	2000	0.106	0.406	0.862
Nigeria	Ughelli North	2017	0.014	0.058	0.145
Nigeria	Ughelli North	2000-2017	-0.243	-0.137	-0.041
Nigeria	Ughelli South	2000	0.096	0.488	1.039
Nigeria	Ughelli South	2017	0.018	0.087	0.225
Nigeria	Ughelli South	2000-2017	-0.241	-0.120	-0.026
Nigeria	Ugwunagbo	2000	0.337	0.645	1.041
Nigeria	Ugwunagbo	2017	0.023	0.089	0.223
Nigeria	Ugwunagbo	2000-2017	-0.289	-0.183	-0.083
Nigeria	Uhunmwonde	2000	0.292	0.803	1.707
Nigeria	Uhunmwonde	2017	0.088	0.283	0.634
Nigeria	Uhunmwonde	2000-2017	-0.203	-0.112	-0.032
Nigeria	Ukanafun	2000	0.420	0.790	1.313
Nigeria	Ukanafun	2017	0.021	0.096	0.232
Nigeria	Ukanafun	2000-2017	-0.300	-0.179	-0.076
Nigeria	Ukum	2000	0.059	0.310	0.615
Nigeria	Ukum	2017	0.037	0.146	0.345
Nigeria	Ukum	2000-2017	-0.189	-0.076	0.024
Nigeria	Ukwa East	2000	0.436	0.815	1.342

Table 3: Malaria DALYs rate by unit *(continued)*

Country	Unit	year	mean rate	lower rate	upper rate
Nigeria	Ukwa East	2017	0.025	0.101	0.244
Nigeria	Ukwa East	2000-2017	-0.290	-0.185	-0.087
Nigeria	Ukwa West	2000	0.262	0.505	0.805
Nigeria	Ukwa West	2017	0.016	0.062	0.150
Nigeria	Ukwa West	2000-2017	-0.286	-0.187	-0.078
Nigeria	Ukwuani	2000	0.094	0.394	0.992
Nigeria	Ukwuani	2017	0.011	0.055	0.164
Nigeria	Ukwuani	2000-2017	-0.263	-0.158	-0.057
Nigeria	Umu-Nneochi	2000	0.483	0.955	1.692
Nigeria	Umu-Nneochi	2017	0.017	0.117	0.301
Nigeria	Umu-Nneochi	2000-2017	-0.332	-0.190	-0.085
Nigeria	Umuahia North	2000	0.333	0.635	1.012
Nigeria	Umuahia North	2017	0.018	0.084	0.176
Nigeria	Umuahia North	2000-2017	-0.282	-0.171	-0.075
Nigeria	Umuahia South	2000	0.548	1.006	1.636
Nigeria	Umuahia South	2017	0.033	0.148	0.302
Nigeria	Umuahia South	2000-2017	-0.278	-0.163	-0.067
Nigeria	Ungogo	2000	0.062	0.343	0.734
Nigeria	Ungogo	2017	0.028	0.113	0.274
Nigeria	Ungogo	2000-2017	-0.199	-0.107	0.014
Nigeria	Unuimo	2000	0.457	0.857	1.425
Nigeria	Unuimo	2017	0.024	0.117	0.254
Nigeria	Unuimo	2000-2017	-0.303	-0.178	-0.071
Nigeria	Uruan	2000	0.385	0.720	1.173
Nigeria	Uruan	2017	0.019	0.088	0.216
Nigeria	Uruan	2000-2017	-0.284	-0.170	-0.057
Nigeria	UrueOffo	2000	0.445	0.821	1.363
Nigeria	UrueOffo	2017	0.043	0.183	0.441
Nigeria	UrueOffo	2000-2017	-0.237	-0.131	-0.023
Nigeria	Ushongo	2000	0.048	0.376	0.795
Nigeria	Ushongo	2017	0.041	0.169	0.407
Nigeria	Ushongo	2000-2017	-0.200	-0.087	-0.001
Nigeria	Ussa	2000	0.073	0.379	0.813
Nigeria	Ussa	2017	0.049	0.219	0.547
Nigeria	Ussa	2000-2017	-0.169	-0.063	0.036
Nigeria	Uvwie	2000	0.027	0.181	0.501
Nigeria	Uvwie	2017	0.007	0.034	0.086
Nigeria	Uvwie	2000-2017	-0.241	-0.120	-0.017
Nigeria	Uyo	2000	0.215	0.421	0.707
Nigeria	Uyo	2017	0.008	0.039	0.093
Nigeria	Uyo	2000-2017	-0.323	-0.191	-0.074
Nigeria	Uzo-Uwani	2000	0.126	0.500	1.249
Nigeria	Uzo-Uwani	2017	0.024	0.084	0.216
Nigeria	Uzo-Uwani	2000-2017	-0.252	-0.163	-0.062
Nigeria	Vandeiky	2000	0.056	0.359	0.724
Nigeria	Vandeiky	2017	0.047	0.189	0.484
Nigeria	Vandeiky	2000-2017	-0.188	-0.077	0.008
Nigeria	Wamakko	2000	0.014	0.138	0.472
Nigeria	Wamakko	2017	0.085	0.241	0.422
Nigeria	Wamakko	2000-2017	-0.060	0.040	0.134
Nigeria	Wamba	2000	0.234	0.545	0.981
Nigeria	Wamba	2017	0.081	0.270	0.549
Nigeria	Wamba	2000-2017	-0.149	-0.061	0.007
Nigeria	Warawa	2000	0.145	0.809	1.789
Nigeria	Warawa	2017	0.067	0.264	0.677
Nigeria	Warawa	2000-2017	-0.209	-0.099	0.005
Nigeria	Warji	2000	0.210	0.956	2.189
Nigeria	Warji	2017	0.041	0.214	0.607
Nigeria	Warji	2000-2017	-0.265	-0.153	-0.037
Nigeria	Warri North	2000	0.085	0.524	1.109
Nigeria	Warri North	2017	0.029	0.142	0.339

Table 3: Malaria DALYs rate by unit *(continued)*

Country	Unit	year	mean rate	lower rate	upper rate
Nigeria	Warri North	2000-2017	-0.230	-0.122	-0.035
Nigeria	Warri South	2000	0.071	0.395	0.862
Nigeria	Warri South	2017	0.016	0.078	0.204
Nigeria	Warri South	2000-2017	-0.234	-0.119	-0.026
Nigeria	Warri South-West	2000	0.077	0.455	1.122
Nigeria	Warri South-West	2017	0.015	0.087	0.246
Nigeria	Warri South-West	2000-2017	-0.223	-0.122	-0.033
Nigeria	Wase	2000	0.238	0.972	1.908
Nigeria	Wase	2017	0.208	0.567	1.096
Nigeria	Wase	2000-2017	-0.159	-0.057	-0.003
Nigeria	Wudil	2000	0.149	1.008	2.205
Nigeria	Wudil	2017	0.079	0.286	0.759
Nigeria	Wudil	2000-2017	-0.217	-0.110	-0.009
Nigeria	Wukari	2000	0.074	0.349	0.714
Nigeria	Wukari	2017	0.047	0.162	0.390
Nigeria	Wukari	2000-2017	-0.162	-0.066	0.025
Nigeria	Wurno	2000	0.038	0.331	1.033
Nigeria	Wurno	2017	0.180	0.455	0.761
Nigeria	Wurno	2000-2017	-0.049	0.033	0.134
Nigeria	Wushishi	2000	0.110	0.442	0.885
Nigeria	Wushishi	2017	0.055	0.205	0.437
Nigeria	Wushishi	2000-2017	-0.119	-0.036	0.039
Nigeria	Yabo	2000	0.025	0.271	0.764
Nigeria	Yabo	2017	0.152	0.385	0.702
Nigeria	Yabo	2000-2017	-0.069	0.033	0.132
Nigeria	Yagba East	2000	0.179	0.504	1.047
Nigeria	Yagba East	2017	0.032	0.134	0.298
Nigeria	Yagba East	2000-2017	-0.236	-0.123	-0.029
Nigeria	Yagba West	2000	0.145	0.480	1.057
Nigeria	Yagba West	2017	0.022	0.137	0.383
Nigeria	Yagba West	2000-2017	-0.236	-0.115	-0.011
Nigeria	Yakurr	2000	0.403	0.808	1.474
Nigeria	Yakurr	2017	0.056	0.211	0.516
Nigeria	Yakurr	2000-2017	-0.217	-0.121	-0.039
Nigeria	Yala Cross	2000	0.100	0.454	0.876
Nigeria	Yala Cross	2017	0.078	0.252	0.541
Nigeria	Yala Cross	2000-2017	-0.166	-0.067	0.008
Nigeria	Yamaltu	2000	0.136	0.780	1.896
Nigeria	Yamaltu	2017	0.155	0.580	1.192
Nigeria	Yamaltu	2000-2017	-0.117	-0.026	0.048
Nigeria	Yankwashi	2000	0.073	0.541	1.256
Nigeria	Yankwashi	2017	0.059	0.235	0.646
Nigeria	Yankwashi	2000-2017	-0.212	-0.101	0.002
Nigeria	Yauri	2000	0.111	0.649	1.535
Nigeria	Yauri	2017	0.464	0.822	1.352
Nigeria	Yauri	2000-2017	-0.022	0.019	0.083
Nigeria	Yenegoa	2000	0.129	0.457	0.939
Nigeria	Yenegoa	2017	0.018	0.068	0.192
Nigeria	Yenegoa	2000-2017	-0.237	-0.134	-0.045
Nigeria	Yola North	2000	0.043	0.262	0.563
Nigeria	Yola North	2017	0.033	0.160	0.389
Nigeria	Yola North	2000-2017	-0.132	-0.010	0.096
Nigeria	Yola South	2000	0.093	0.449	0.959
Nigeria	Yola South	2017	0.063	0.257	0.571
Nigeria	Yola South	2000-2017	-0.134	-0.014	0.087
Nigeria	Yorro	2000	0.053	0.564	1.238
Nigeria	Yorro	2017	0.169	0.423	0.792
Nigeria	Yorro	2000-2017	-0.091	-0.010	0.066
Nigeria	Yunusari	2000	0.022	0.254	0.751
Nigeria	Yunusari	2017	0.022	0.129	0.372
Nigeria	Yunusari	2000-2017	-0.190	-0.059	0.063

Table 3: Malaria DALYs rate by unit *(continued)*

Country	Unit	year	mean rate	lower rate	upper rate
Nigeria	Yusufari	2000	0.028	0.315	0.793
Nigeria	Yusufari	2017	0.034	0.156	0.393
Nigeria	Yusufari	2000-2017	-0.153	-0.053	0.063
Nigeria	Zaki	2000	0.056	0.456	1.069
Nigeria	Zaki	2017	0.074	0.235	0.462
Nigeria	Zaki	2000-2017	-0.140	-0.041	0.053
Nigeria	Zango	2000	0.086	0.530	1.286
Nigeria	Zango	2017	0.053	0.227	0.614
Nigeria	Zango	2000-2017	-0.205	-0.091	0.015
Nigeria	ZangonKa	2000	0.460	0.800	1.347
Nigeria	ZangonKa	2017	0.100	0.341	0.648
Nigeria	ZangonKa	2000-2017	-0.168	-0.066	-0.017
Nigeria	Zaria	2000	0.265	0.712	1.218
Nigeria	Zaria	2017	0.063	0.255	0.619
Nigeria	Zaria	2000-2017	-0.170	-0.078	0.032
Nigeria	Zing	2000	0.052	0.569	1.189
Nigeria	Zing	2017	0.141	0.390	0.681
Nigeria	Zing	2000-2017	-0.083	-0.009	0.063
Nigeria	Zurmi	2000	0.075	0.506	1.290
Nigeria	Zurmi	2017	0.149	0.459	0.952
Nigeria	Zurmi	2000-2017	-0.089	-0.011	0.061
Nigeria	Zuru	2000	0.052	0.647	1.653
Nigeria	Zuru	2017	0.316	0.771	1.353
Nigeria	Zuru	2000-2017	-0.063	0.015	0.123
Republic of Congo	Abala	2000	0.034	0.270	0.590
Republic of Congo	Abala	2017	0.007	0.064	0.187
Republic of Congo	Abala	2000-2017	-0.255	-0.104	0.003
Republic of Congo	Bambama	2000	0.308	0.736	1.219
Republic of Congo	Bambama	2017	0.047	0.216	0.458
Republic of Congo	Bambama	2000-2017	-0.178	-0.077	0.000
Republic of Congo	Boko	2000	0.103	0.376	0.720
Republic of Congo	Boko	2017	0.024	0.136	0.310
Republic of Congo	Boko	2000-2017	-0.172	-0.072	0.018
Republic of Congo	Boko-Songho	2000	0.055	0.358	0.745
Republic of Congo	Boko-Songho	2017	0.011	0.085	0.203
Republic of Congo	Boko-Songho	2000-2017	-0.239	-0.101	-0.012
Republic of Congo	Boundji	2000	0.040	0.268	0.650
Republic of Congo	Boundji	2017	0.008	0.066	0.207
Republic of Congo	Boundji	2000-2017	-0.270	-0.101	0.005
Republic of Congo	Brazzaville	2000	0.041	0.143	0.330
Republic of Congo	Brazzaville	2017	0.007	0.060	0.146
Republic of Congo	Brazzaville	2000-2017	-0.167	-0.058	0.031
Republic of Congo	Divénié	2000	0.171	0.593	1.193
Republic of Congo	Divénié	2017	0.032	0.189	0.400
Republic of Congo	Divénié	2000-2017	-0.198	-0.065	0.037
Republic of Congo	Djambala	2000	0.052	0.264	0.616
Republic of Congo	Djambala	2017	0.013	0.081	0.195
Republic of Congo	Djambala	2000-2017	-0.185	-0.072	0.048
Republic of Congo	Dongou	2000	0.285	0.782	1.385
Republic of Congo	Dongou	2017	0.047	0.196	0.400
Republic of Congo	Dongou	2000-2017	-0.213	-0.100	-0.044
Republic of Congo	Epéna	2000	0.075	0.386	1.396
Republic of Congo	Epéna	2017	0.011	0.089	0.392
Republic of Congo	Epéna	2000-2017	-0.225	-0.112	-0.041
Republic of Congo	Ewo	2000	0.030	0.252	0.636
Republic of Congo	Ewo	2017	0.004	0.067	0.220
Republic of Congo	Ewo	2000-2017	-0.256	-0.094	0.007
Republic of Congo	Gamboma	2000	0.040	0.270	0.699
Republic of Congo	Gamboma	2017	0.013	0.064	0.186
Republic of Congo	Gamboma	2000-2017	-0.215	-0.101	0.035
Republic of Congo	Impfondo	2000	0.174	0.567	1.158

Table 3: Malaria DALYs rate by unit *(continued)*

Country	Unit	year	mean rate	lower rate	upper rate
Republic of Congo	Impfondo	2017	0.018	0.100	0.319
Republic of Congo	Impfondo	2000-2017	-0.219	-0.137	-0.058
Republic of Congo	Kakamoeka	2000	0.149	0.573	1.274
Republic of Congo	Kakamoeka	2017	0.038	0.203	0.485
Republic of Congo	Kakamoeka	2000-2017	-0.182	-0.074	0.025
Republic of Congo	Kéllé	2000	0.030	0.377	1.459
Republic of Congo	Kéllé	2017	0.007	0.114	0.394
Republic of Congo	Kéllé	2000-2017	-0.220	-0.075	0.022
Republic of Congo	Kibangou	2000	0.082	0.390	0.894
Republic of Congo	Kibangou	2017	0.012	0.129	0.275
Republic of Congo	Kibangou	2000-2017	-0.262	-0.078	0.030
Republic of Congo	Kimongo	2000	0.080	0.388	0.773
Republic of Congo	Kimongo	2017	0.026	0.131	0.262
Republic of Congo	Kimongo	2000-2017	-0.180	-0.078	0.015
Republic of Congo	Kindamba	2000	0.060	0.272	0.682
Republic of Congo	Kindamba	2017	0.011	0.070	0.196
Republic of Congo	Kindamba	2000-2017	-0.200	-0.073	0.020
Republic of Congo	Kinkala	2000	0.101	0.340	0.638
Republic of Congo	Kinkala	2017	0.019	0.123	0.293
Republic of Congo	Kinkala	2000-2017	-0.175	-0.065	0.016
Republic of Congo	Komono	2000	0.184	0.758	1.399
Republic of Congo	Komono	2017	0.035	0.220	0.466
Republic of Congo	Komono	2000-2017	-0.207	-0.080	0.020
Republic of Congo	Lékana	2000	0.047	0.304	0.756
Republic of Congo	Lékana	2017	0.010	0.087	0.210
Republic of Congo	Lékana	2000-2017	-0.207	-0.065	0.078
Republic of Congo	Loandjili	2000	0.075	0.260	0.445
Republic of Congo	Loandjili	2017	0.011	0.067	0.155
Republic of Congo	Loandjili	2000-2017	-0.198	-0.078	0.037
Republic of Congo	Loudima	2000	0.059	0.318	0.697
Republic of Congo	Loudima	2017	0.014	0.097	0.238
Republic of Congo	Loudima	2000-2017	-0.224	-0.084	0.026
Republic of Congo	Loukoléla	2000	0.015	0.333	2.009
Republic of Congo	Loukoléla	2017	0.005	0.085	0.482
Republic of Congo	Loukoléla	2000-2017	-0.195	-0.105	-0.004
Republic of Congo	Louvakou (Loubomo)	2000	0.080	0.359	0.781
Republic of Congo	Louvakou (Loubomo)	2017	0.017	0.123	0.269
Republic of Congo	Louvakou (Loubomo)	2000-2017	-0.215	-0.076	0.025
Republic of Congo	Madingo-Kayes	2000	0.159	0.498	1.123
Republic of Congo	Madingo-Kayes	2017	0.023	0.159	0.415
Republic of Congo	Madingo-Kayes	2000-2017	-0.199	-0.082	0.031
Republic of Congo	Madingou	2000	0.038	0.283	0.598
Republic of Congo	Madingou	2017	0.007	0.060	0.163
Republic of Congo	Madingou	2000-2017	-0.233	-0.100	0.015
Republic of Congo	Makoua	2000	0.041	0.324	1.022
Republic of Congo	Makoua	2017	0.006	0.086	0.319
Republic of Congo	Makoua	2000-2017	-0.238	-0.087	0.041
Republic of Congo	Mayama	2000	0.044	0.202	0.460
Republic of Congo	Mayama	2017	0.006	0.058	0.157
Republic of Congo	Mayama	2000-2017	-0.197	-0.075	0.018
Republic of Congo	Mayoko	2000	0.295	0.774	1.411
Republic of Congo	Mayoko	2017	0.045	0.225	0.505
Republic of Congo	Mayoko	2000-2017	-0.235	-0.081	-0.005
Republic of Congo	Mbomo	2000	0.025	0.347	1.807
Republic of Congo	Mbomo	2017	0.004	0.103	0.489
Republic of Congo	Mbomo	2000-2017	-0.221	-0.064	0.041
Republic of Congo	Mfouati	2000	0.041	0.299	0.680
Republic of Congo	Mfouati	2017	0.007	0.056	0.151
Republic of Congo	Mfouati	2000-2017	-0.229	-0.102	0.019
Republic of Congo	Mindouli	2000	0.040	0.231	0.558
Republic of Congo	Mindouli	2017	0.007	0.045	0.127

Table 3: Malaria DALYs rate by unit *(continued)*

Country	Unit	year	mean rate	lower rate	upper rate
Republic of Congo	Mindouli	2000-2017	-0.199	-0.090	0.030
Republic of Congo	Mossaka	2000	0.025	0.332	1.528
Republic of Congo	Mossaka	2017	0.005	0.072	0.374
Republic of Congo	Mossaka	2000-2017	-0.218	-0.114	-0.018
Republic of Congo	Mossendjo	2000	0.139	0.643	1.210
Republic of Congo	Mossendjo	2017	0.031	0.203	0.439
Republic of Congo	Mossendjo	2000-2017	-0.251	-0.077	0.001
Republic of Congo	Mouyondzi	2000	0.057	0.301	0.632
Republic of Congo	Mouyondzi	2017	0.008	0.065	0.159
Republic of Congo	Mouyondzi	2000-2017	-0.226	-0.094	0.020
Republic of Congo	Mvouti	2000	0.192	0.587	1.254
Republic of Congo	Mvouti	2017	0.061	0.220	0.393
Republic of Congo	Mvouti	2000-2017	-0.137	-0.062	0.001
Republic of Congo	Ngabé	2000	0.058	0.360	0.710
Republic of Congo	Ngabé	2017	0.025	0.152	0.365
Republic of Congo	Ngabé	2000-2017	-0.178	-0.054	0.044
Republic of Congo	Ngamaba	2000	0.093	0.298	0.608
Republic of Congo	Ngamaba	2017	0.017	0.109	0.230
Republic of Congo	Ngamaba	2000-2017	-0.159	-0.065	0.017
Republic of Congo	Nkayi District	2000	0.039	0.297	0.627
Republic of Congo	Nkayi District	2017	0.008	0.076	0.206
Republic of Congo	Nkayi District	2000-2017	-0.232	-0.093	0.026
Republic of Congo	Okoyo	2000	0.040	0.273	0.635
Republic of Congo	Okoyo	2017	0.007	0.072	0.198
Republic of Congo	Okoyo	2000-2017	-0.243	-0.085	0.041
Republic of Congo	Ouesso	2000	0.050	0.403	1.530
Republic of Congo	Ouesso	2017	0.016	0.117	0.446
Republic of Congo	Ouesso	2000-2017	-0.178	-0.078	-0.010
Republic of Congo	Owando	2000	0.049	0.307	0.863
Republic of Congo	Owando	2017	0.007	0.071	0.217
Republic of Congo	Owando	2000-2017	-0.237	-0.105	0.007
Republic of Congo	Pointe Noire	2000	0.058	0.237	0.476
Republic of Congo	Pointe Noire	2017	0.006	0.052	0.142
Republic of Congo	Pointe Noire	2000-2017	-0.234	-0.088	0.053
Republic of Congo	Sembé	2000	0.063	0.469	1.458
Republic of Congo	Sembé	2017	0.020	0.139	0.442
Republic of Congo	Sembé	2000-2017	-0.192	-0.068	0.021
Republic of Congo	Sibiti	2000	0.124	0.573	1.102
Republic of Congo	Sibiti	2017	0.027	0.168	0.396
Republic of Congo	Sibiti	2000-2017	-0.221	-0.083	0.001
Republic of Congo	Souanké	2000	0.045	0.436	1.651
Republic of Congo	Souanké	2017	0.018	0.145	0.634
Republic of Congo	Souanké	2000-2017	-0.159	-0.054	0.029
Republic of Congo	Zanaga	2000	0.150	0.607	1.135
Republic of Congo	Zanaga	2017	0.027	0.168	0.381
Republic of Congo	Zanaga	2000-2017	-0.206	-0.078	0.015
Rwanda	Bugesera	2000	0.108	0.647	1.597
Rwanda	Bugesera	2017	0.036	0.112	0.236
Rwanda	Bugesera	2000-2017	-0.059	-0.014	0.045
Rwanda	Burera	2000	0.001	0.004	0.012
Rwanda	Burera	2017	0.000	0.001	0.001
Rwanda	Burera	2000-2017	-0.111	-0.046	0.023
Rwanda	Gakenke	2000	0.062	0.375	1.124
Rwanda	Gakenke	2017	0.011	0.048	0.101
Rwanda	Gakenke	2000-2017	-0.111	-0.045	0.019
Rwanda	Gasabo	2000	0.053	0.365	1.114
Rwanda	Gasabo	2017	0.006	0.024	0.049
Rwanda	Gasabo	2000-2017	-0.115	-0.057	0.022
Rwanda	Gatsibo	2000	0.141	0.719	1.910
Rwanda	Gatsibo	2017	0.017	0.066	0.143
Rwanda	Gatsibo	2000-2017	-0.159	-0.069	-0.001

Table 3: Malaria DALYs rate by unit *(continued)*

Country	Unit	year	mean rate	lower rate	upper rate
Rwanda	Gicumbi	2000	0.062	0.310	0.824
Rwanda	Gicumbi	2017	0.007	0.027	0.059
Rwanda	Gicumbi	2000-2017	-0.138	-0.061	0.022
Rwanda	Gisagara	2000	0.092	0.465	1.144
Rwanda	Gisagara	2017	0.051	0.132	0.285
Rwanda	Gisagara	2000-2017	-0.045	-0.005	0.041
Rwanda	Huye	2000	0.055	0.413	1.410
Rwanda	Huye	2017	0.033	0.101	0.248
Rwanda	Huye	2000-2017	-0.055	0.005	0.074
Rwanda	Kamonyi	2000	0.071	0.486	1.392
Rwanda	Kamonyi	2017	0.013	0.051	0.108
Rwanda	Kamonyi	2000-2017	-0.087	-0.030	0.045
Rwanda	Karongi	2000	0.020	0.158	0.655
Rwanda	Karongi	2017	0.009	0.036	0.076
Rwanda	Karongi	2000-2017	-0.050	0.007	0.082
Rwanda	Kayonza	2000	0.115	0.647	1.674
Rwanda	Kayonza	2017	0.011	0.049	0.109
Rwanda	Kayonza	2000-2017	-0.157	-0.070	0.005
Rwanda	Kicukiro	2000	0.034	0.269	0.816
Rwanda	Kicukiro	2017	0.006	0.022	0.045
Rwanda	Kicukiro	2000-2017	-0.093	-0.038	0.035
Rwanda	Kirehe	2000	0.107	0.579	1.596
Rwanda	Kirehe	2017	0.020	0.066	0.132
Rwanda	Kirehe	2000-2017	-0.097	-0.042	0.019
Rwanda	Muhanga	2000	0.052	0.370	1.269
Rwanda	Muhanga	2017	0.014	0.058	0.118
Rwanda	Muhanga	2000-2017	-0.080	-0.019	0.054
Rwanda	Musanze	2000	0.016	0.107	0.333
Rwanda	Musanze	2017	0.003	0.012	0.028
Rwanda	Musanze	2000-2017	-0.136	-0.060	0.020
Rwanda	Ngoma	2000	0.102	0.551	1.429
Rwanda	Ngoma	2017	0.019	0.065	0.135
Rwanda	Ngoma	2000-2017	-0.094	-0.036	0.029
Rwanda	Ngororero	2000	0.027	0.172	0.608
Rwanda	Ngororero	2017	0.007	0.030	0.068
Rwanda	Ngororero	2000-2017	-0.095	-0.023	0.053
Rwanda	Nyabihu	2000	0.010	0.063	0.215
Rwanda	Nyabihu	2017	0.002	0.009	0.020
Rwanda	Nyabihu	2000-2017	-0.120	-0.043	0.042
Rwanda	Nyagatare	2000	0.135	0.703	1.880
Rwanda	Nyagatare	2017	0.018	0.065	0.146
Rwanda	Nyagatare	2000-2017	-0.135	-0.082	-0.026
Rwanda	Nyamagabe	2000	0.020	0.133	0.518
Rwanda	Nyamagabe	2017	0.010	0.036	0.085
Rwanda	Nyamagabe	2000-2017	-0.035	0.024	0.101
Rwanda	Nyamasheke	2000	0.025	0.180	0.857
Rwanda	Nyamasheke	2017	0.012	0.040	0.084
Rwanda	Nyamasheke	2000-2017	-0.073	0.005	0.090
Rwanda	Nyanza	2000	0.065	0.434	1.390
Rwanda	Nyanza	2017	0.037	0.106	0.253
Rwanda	Nyanza	2000-2017	-0.045	0.018	0.097
Rwanda	Nyarugenge	2000	0.026	0.217	0.694
Rwanda	Nyarugenge	2017	0.005	0.017	0.034
Rwanda	Nyarugenge	2000-2017	-0.098	-0.040	0.040
Rwanda	Nyaruguru	2000	0.034	0.241	0.772
Rwanda	Nyaruguru	2017	0.017	0.052	0.122
Rwanda	Nyaruguru	2000-2017	-0.064	-0.010	0.047
Rwanda	Rubavu	2000	0.018	0.151	0.564
Rwanda	Rubavu	2017	0.004	0.022	0.049
Rwanda	Rubavu	2000-2017	-0.120	-0.022	0.071
Rwanda	Ruhango	2000	0.047	0.360	1.295

Table 3: Malaria DALYs rate by unit *(continued)*

Country	Unit	year	mean rate	lower rate	upper rate
Rwanda	Ruhango	2017	0.021	0.073	0.167
Rwanda	Ruhango	2000-2017	-0.049	0.012	0.100
Rwanda	Rulindo	2000	0.072	0.380	0.991
Rwanda	Rulindo	2017	0.010	0.037	0.080
Rwanda	Rulindo	2000-2017	-0.111	-0.051	0.015
Rwanda	Rusizi	2000	0.021	0.195	0.994
Rwanda	Rusizi	2017	0.018	0.059	0.127
Rwanda	Rusizi	2000-2017	-0.064	0.009	0.083
Rwanda	Rutsiro	2000	0.022	0.166	0.654
Rwanda	Rutsiro	2017	0.006	0.029	0.061
Rwanda	Rutsiro	2000-2017	-0.090	-0.013	0.057
Rwanda	Rwamagana	2000	0.095	0.573	1.538
Rwanda	Rwamagana	2017	0.012	0.046	0.102
Rwanda	Rwamagana	2000-2017	-0.126	-0.056	0.019
Senegal	Bakel	2000	0.038	0.285	0.753
Senegal	Bakel	2017	0.011	0.035	0.084
Senegal	Bakel	2000-2017	-0.222	-0.143	-0.055
Senegal	Bambey	2000	0.099	0.306	0.636
Senegal	Bambey	2017	0.006	0.019	0.036
Senegal	Bambey	2000-2017	-0.212	-0.164	-0.116
Senegal	Bignona	2000	0.105	0.423	1.035
Senegal	Bignona	2017	0.013	0.038	0.089
Senegal	Bignona	2000-2017	-0.191	-0.133	-0.072
Senegal	Birkilane	2000	0.357	0.905	1.671
Senegal	Birkilane	2017	0.010	0.037	0.090
Senegal	Birkilane	2000-2017	-0.242	-0.187	-0.137
Senegal	Bounkiling	2000	0.188	0.394	0.759
Senegal	Bounkiling	2017	0.004	0.014	0.032
Senegal	Bounkiling	2000-2017	-0.248	-0.191	-0.133
Senegal	Dagana	2000	0.027	0.077	0.177
Senegal	Dagana	2017	0.005	0.017	0.041
Senegal	Dagana	2000-2017	-0.172	-0.105	-0.035
Senegal	Dakar	2000	0.009	0.044	0.152
Senegal	Dakar	2017	0.009	0.025	0.052
Senegal	Dakar	2000-2017	-0.091	-0.026	0.036
Senegal	Diourbel	2000	0.069	0.226	0.502
Senegal	Diourbel	2017	0.005	0.016	0.035
Senegal	Diourbel	2000-2017	-0.193	-0.145	-0.094
Senegal	Fatick	2000	0.192	0.419	0.830
Senegal	Fatick	2017	0.008	0.028	0.056
Senegal	Fatick	2000-2017	-0.219	-0.163	-0.113
Senegal	Foundiougne	2000	0.102	0.318	0.672
Senegal	Foundiougne	2017	0.008	0.026	0.059
Senegal	Foundiougne	2000-2017	-0.213	-0.158	-0.102
Senegal	Gossas	2000	0.125	0.416	0.913
Senegal	Gossas	2017	0.010	0.029	0.064
Senegal	Gossas	2000-2017	-0.207	-0.150	-0.103
Senegal	Goudiry	2000	0.062	0.403	1.047
Senegal	Goudiry	2017	0.015	0.039	0.093
Senegal	Goudiry	2000-2017	-0.260	-0.182	-0.095
Senegal	Goudomp	2000	0.204	0.552	1.119
Senegal	Goudomp	2017	0.010	0.025	0.054
Senegal	Goudomp	2000-2017	-0.221	-0.156	-0.098
Senegal	Guédiawaye	2000	0.009	0.047	0.163
Senegal	Guédiawaye	2017	0.007	0.021	0.045
Senegal	Guédiawaye	2000-2017	-0.100	-0.040	0.015
Senegal	Guinguinéo	2000	0.129	0.540	1.065
Senegal	Guinguinéo	2017	0.011	0.036	0.084
Senegal	Guinguinéo	2000-2017	-0.211	-0.149	-0.092
Senegal	Kaffrine	2000	0.324	0.793	1.479
Senegal	Kaffrine	2017	0.009	0.032	0.078

Table 3: Malaria DALYs rate by unit *(continued)*

Country	Unit	year	mean rate	lower rate	upper rate
Senegal	Kaffrine	2000-2017	-0.249	-0.195	-0.141
Senegal	Kanel	2000	0.012	0.124	0.323
Senegal	Kanel	2017	0.009	0.026	0.057
Senegal	Kanel	2000-2017	-0.184	-0.111	-0.028
Senegal	Kaolack	2000	0.134	0.483	0.935
Senegal	Kaolack	2017	0.010	0.030	0.065
Senegal	Kaolack	2000-2017	-0.207	-0.151	-0.097
Senegal	Kébémer	2000	0.021	0.106	0.319
Senegal	Kébémer	2017	0.005	0.016	0.035
Senegal	Kébémer	2000-2017	-0.155	-0.099	-0.048
Senegal	Kédougou	2000	0.168	0.423	0.739
Senegal	Kédougou	2017	0.008	0.021	0.049
Senegal	Kédougou	2000-2017	-0.297	-0.227	-0.171
Senegal	Kolda	2000	0.099	0.334	0.655
Senegal	Kolda	2017	0.009	0.024	0.051
Senegal	Kolda	2000-2017	-0.218	-0.162	-0.105
Senegal	Koungheul	2000	0.238	0.649	1.583
Senegal	Koungheul	2017	0.009	0.029	0.068
Senegal	Koungheul	2000-2017	-0.256	-0.192	-0.123
Senegal	Koupentoum	2000	0.220	0.600	1.447
Senegal	Koupentoum	2017	0.013	0.042	0.091
Senegal	Koupentoum	2000-2017	-0.231	-0.175	-0.111
Senegal	Linguère	2000	0.060	0.199	0.424
Senegal	Linguère	2017	0.007	0.019	0.044
Senegal	Linguère	2000-2017	-0.192	-0.132	-0.070
Senegal	Louga	2000	0.022	0.075	0.190
Senegal	Louga	2017	0.005	0.016	0.032
Senegal	Louga	2000-2017	-0.142	-0.098	-0.042
Senegal	Malème Hodar	2000	0.151	0.470	1.042
Senegal	Malème Hodar	2017	0.007	0.024	0.060
Senegal	Malème Hodar	2000-2017	-0.273	-0.191	-0.122
Senegal	Matam	2000	0.007	0.068	0.218
Senegal	Matam	2017	0.005	0.015	0.032
Senegal	Matam	2000-2017	-0.133	-0.077	-0.017
Senegal	Mbacké	2000	0.030	0.126	0.333
Senegal	Mbacké	2017	0.005	0.014	0.027
Senegal	Mbacké	2000-2017	-0.149	-0.102	-0.055
Senegal	Mbour	2000	0.068	0.203	0.469
Senegal	Mbour	2017	0.011	0.033	0.078
Senegal	Mbour	2000-2017	-0.180	-0.124	-0.076
Senegal	Médina Yoro Foula	2000	0.161	0.396	0.707
Senegal	Médina Yoro Foula	2017	0.008	0.019	0.040
Senegal	Médina Yoro Foula	2000-2017	-0.229	-0.177	-0.134
Senegal	Nioro du Rip	2000	0.425	0.991	1.721
Senegal	Nioro du Rip	2017	0.009	0.031	0.069
Senegal	Nioro du Rip	2000-2017	-0.246	-0.187	-0.134
Senegal	Oussouye	2000	0.110	0.511	1.167
Senegal	Oussouye	2017	0.023	0.071	0.165
Senegal	Oussouye	2000-2017	-0.177	-0.091	-0.002
Senegal	Pikine	2000	0.009	0.047	0.155
Senegal	Pikine	2017	0.007	0.019	0.040
Senegal	Pikine	2000-2017	-0.107	-0.048	0.004
Senegal	Podor	2000	0.096	0.258	0.533
Senegal	Podor	2017	0.005	0.017	0.037
Senegal	Podor	2000-2017	-0.227	-0.163	-0.112
Senegal	Ranérou Ferlo	2000	0.074	0.280	0.594
Senegal	Ranérou Ferlo	2017	0.008	0.031	0.070
Senegal	Ranérou Ferlo	2000-2017	-0.228	-0.154	-0.075
Senegal	Rufisque	2000	0.024	0.106	0.315
Senegal	Rufisque	2017	0.010	0.028	0.058
Senegal	Rufisque	2000-2017	-0.131	-0.078	-0.028

Table 3: Malaria DALYs rate by unit *(continued)*

Country	Unit	year	mean rate	lower rate	upper rate
Senegal	Saint-Louis	2000	0.005	0.026	0.080
Senegal	Saint-Louis	2017	0.005	0.018	0.038
Senegal	Saint-Louis	2000-2017	-0.112	-0.056	0.002
Senegal	Salémata	2000	0.113	0.369	0.665
Senegal	Salémata	2017	0.007	0.022	0.052
Senegal	Salémata	2000-2017	-0.298	-0.222	-0.144
Senegal	Saraya	2000	0.108	0.391	0.768
Senegal	Saraya	2017	0.014	0.044	0.100
Senegal	Saraya	2000-2017	-0.249	-0.167	-0.099
Senegal	Sédhiou	2000	0.159	0.437	0.904
Senegal	Sédhiou	2017	0.007	0.020	0.047
Senegal	Sédhiou	2000-2017	-0.221	-0.162	-0.104
Senegal	Tambacounda	2000	0.140	0.442	0.910
Senegal	Tambacounda	2017	0.015	0.041	0.082
Senegal	Tambacounda	2000-2017	-0.213	-0.150	-0.093
Senegal	Thiès	2000	0.046	0.188	0.457
Senegal	Thiès	2017	0.008	0.022	0.043
Senegal	Thiès	2000-2017	-0.175	-0.131	-0.095
Senegal	Tivaouane	2000	0.033	0.127	0.405
Senegal	Tivaouane	2017	0.006	0.016	0.030
Senegal	Tivaouane	2000-2017	-0.179	-0.124	-0.077
Senegal	Vélingara	2000	0.107	0.365	0.755
Senegal	Vélingara	2017	0.010	0.027	0.059
Senegal	Vélingara	2000-2017	-0.236	-0.171	-0.119
Senegal	Ziguinchor	2000	0.133	0.421	0.959
Senegal	Ziguinchor	2017	0.014	0.038	0.086
Senegal	Ziguinchor	2000-2017	-0.196	-0.112	-0.048
Sierra Leone	Bo	2000	0.337	1.207	2.160
Sierra Leone	Bo	2017	0.307	0.642	1.262
Sierra Leone	Bo	2000-2017	-0.101	-0.050	-0.015
Sierra Leone	Bombali	2000	0.146	0.677	1.584
Sierra Leone	Bombali	2017	0.263	0.451	0.844
Sierra Leone	Bombali	2000-2017	-0.074	-0.036	0.009
Sierra Leone	Bonthe	2000	0.315	0.973	1.900
Sierra Leone	Bonthe	2017	0.213	0.509	1.122
Sierra Leone	Bonthe	2000-2017	-0.096	-0.045	0.007
Sierra Leone	Kailahun	2000	0.520	1.557	2.768
Sierra Leone	Kailahun	2017	0.334	0.754	1.404
Sierra Leone	Kailahun	2000-2017	-0.098	-0.059	-0.032
Sierra Leone	Kambia	2000	0.174	0.750	1.522
Sierra Leone	Kambia	2017	0.313	0.564	0.963
Sierra Leone	Kambia	2000-2017	-0.064	-0.024	0.022
Sierra Leone	Kenema	2000	0.433	1.395	2.568
Sierra Leone	Kenema	2017	0.315	0.698	1.385
Sierra Leone	Kenema	2000-2017	-0.093	-0.057	-0.033
Sierra Leone	Koinadugu	2000	0.188	0.603	1.324
Sierra Leone	Koinadugu	2017	0.241	0.446	0.739
Sierra Leone	Koinadugu	2000-2017	-0.048	-0.025	0.024
Sierra Leone	Kono	2000	0.309	1.062	2.119
Sierra Leone	Kono	2017	0.271	0.557	1.076
Sierra Leone	Kono	2000-2017	-0.111	-0.059	-0.024
Sierra Leone	Moyamba	2000	0.365	1.147	1.981
Sierra Leone	Moyamba	2017	0.379	0.677	1.237
Sierra Leone	Moyamba	2000-2017	-0.085	-0.044	-0.006
Sierra Leone	Port Loko	2000	0.218	0.831	1.631
Sierra Leone	Port Loko	2017	0.316	0.565	1.013
Sierra Leone	Port Loko	2000-2017	-0.069	-0.034	0.014
Sierra Leone	Pujehun	2000	0.517	1.355	2.468
Sierra Leone	Pujehun	2017	0.356	0.750	1.412
Sierra Leone	Pujehun	2000-2017	-0.077	-0.036	0.005
Sierra Leone	Tonkolili	2000	0.292	0.900	1.919

Table 3: Malaria DALYs rate by unit *(continued)*

Country	Unit	year	mean rate	lower rate	upper rate
Sierra Leone	Tonkolili	2017	0.329	0.590	1.030
Sierra Leone	Tonkolili	2000-2017	-0.068	-0.036	0.004
Sierra Leone	Western Rural	2000	0.097	0.645	1.458
Sierra Leone	Western Rural	2017	0.163	0.361	0.766
Sierra Leone	Western Rural	2000-2017	-0.134	-0.061	0.022
Sierra Leone	Western Urban	2000	0.029	0.322	0.796
Sierra Leone	Western Urban	2017	0.052	0.143	0.329
Sierra Leone	Western Urban	2000-2017	-0.154	-0.077	0.011
Somalia	Aadan	2000	0.008	0.048	0.191
Somalia	Aadan	2017	0.001	0.013	0.047
Somalia	Aadan	2000-2017	-0.194	-0.054	0.091
Somalia	Afgooye	2000	0.028	0.237	0.704
Somalia	Afgooye	2017	0.002	0.013	0.038
Somalia	Afgooye	2000-2017	-0.244	-0.112	-0.008
Somalia	Afmadow	2000	0.016	0.102	0.267
Somalia	Afmadow	2017	0.002	0.012	0.036
Somalia	Afmadow	2000-2017	-0.217	-0.115	-0.003
Somalia	Baar-Dheere	2000	0.006	0.036	0.121
Somalia	Baar-Dheere	2017	0.002	0.012	0.038
Somalia	Baar-Dheere	2000-2017	-0.190	-0.066	0.052
Somalia	Badhaadhe	2000	0.025	0.236	0.640
Somalia	Badhaadhe	2017	0.005	0.028	0.077
Somalia	Badhaadhe	2000-2017	-0.241	-0.115	-0.007
Somalia	Badhan	2000	0.005	0.054	0.193
Somalia	Badhan	2017	0.002	0.011	0.031
Somalia	Badhan	2000-2017	-0.162	-0.046	0.060
Somalia	Baki	2000	0.033	0.168	0.450
Somalia	Baki	2017	0.002	0.007	0.017
Somalia	Baki	2000-2017	-0.267	-0.154	-0.048
Somalia	Balcad	2000	0.034	0.199	0.591
Somalia	Balcad	2017	0.003	0.012	0.041
Somalia	Balcad	2000-2017	-0.233	-0.112	-0.007
Somalia	Bander-Beyla	2000	0.007	0.076	0.271
Somalia	Bander-Beyla	2017	0.002	0.010	0.032
Somalia	Bander-Beyla	2000-2017	-0.211	-0.085	0.033
Somalia	Baraawe	2000	0.014	0.133	0.433
Somalia	Baraawe	2017	0.002	0.018	0.053
Somalia	Baraawe	2000-2017	-0.196	-0.053	0.083
Somalia	Baydhabo	2000	0.005	0.032	0.093
Somalia	Baydhabo	2017	0.005	0.022	0.071
Somalia	Baydhabo	2000-2017	-0.165	-0.059	0.040
Somalia	Beled Weyn	2000	0.002	0.013	0.045
Somalia	Beled Weyn	2017	0.001	0.009	0.026
Somalia	Beled Weyn	2000-2017	-0.188	-0.076	0.056
Somalia	Beled Xaawo	2000	0.003	0.046	0.185
Somalia	Beled Xaawo	2017	0.003	0.015	0.039
Somalia	Beled Xaawo	2000-2017	-0.170	-0.062	0.046
Somalia	Berbera	2000	0.008	0.062	0.223
Somalia	Berbera	2017	0.001	0.003	0.009
Somalia	Berbera	2000-2017	-0.274	-0.140	-0.040
Somalia	Boorama	2000	0.043	0.219	0.730
Somalia	Boorama	2017	0.001	0.009	0.022
Somalia	Boorama	2000-2017	-0.265	-0.139	-0.017
Somalia	Bosaaso	2000	0.006	0.054	0.182
Somalia	Bosaaso	2017	0.001	0.009	0.028
Somalia	Bosaaso	2000-2017	-0.183	-0.072	0.059
Somalia	Bu'aale	2000	0.011	0.073	0.223
Somalia	Bu'aale	2017	0.003	0.017	0.059
Somalia	Bu'aale	2000-2017	-0.241	-0.100	0.032
Somalia	Burao	2000	0.023	0.150	0.423
Somalia	Burao	2017	0.002	0.011	0.033

Table 3: Malaria DALYs rate by unit *(continued)*

Country	Unit	year	mean rate	lower rate	upper rate
Somalia	Burao	2000-2017	-0.263	-0.133	-0.020
Somalia	Burtinle	2000	0.002	0.022	0.076
Somalia	Burtinle	2017	0.001	0.009	0.024
Somalia	Burtinle	2000-2017	-0.133	-0.023	0.067
Somalia	Buuhoodle	2000	0.011	0.078	0.258
Somalia	Buuhoodle	2017	0.002	0.014	0.063
Somalia	Buuhoodle	2000-2017	-0.242	-0.088	0.056
Somalia	Buulo Burdo	2000	0.005	0.029	0.088
Somalia	Buulo Burdo	2017	0.002	0.012	0.035
Somalia	Buulo Burdo	2000-2017	-0.198	-0.087	0.048
Somalia	Buur Xakaba	2000	0.005	0.043	0.118
Somalia	Buur Xakaba	2017	0.003	0.015	0.036
Somalia	Buur Xakaba	2000-2017	-0.196	-0.074	0.002
Somalia	Caabudwaaq	2000	0.002	0.020	0.081
Somalia	Caabudwaaq	2017	0.001	0.006	0.016
Somalia	Caabudwaaq	2000-2017	-0.207	-0.109	0.003
Somalia	Cadaado	2000	0.002	0.028	0.090
Somalia	Cadaado	2017	0.001	0.008	0.027
Somalia	Cadaado	2000-2017	-0.206	-0.085	0.044
Somalia	Cadale	2000	0.015	0.100	0.291
Somalia	Cadale	2017	0.001	0.013	0.046
Somalia	Cadale	2000-2017	-0.210	-0.075	0.039
Somalia	Calawla	2000	0.004	0.044	0.197
Somalia	Calawla	2017	0.001	0.006	0.018
Somalia	Calawla	2000-2017	-0.230	-0.076	0.042
Somalia	Caynabo	2000	0.014	0.113	0.483
Somalia	Caynabo	2017	0.002	0.012	0.048
Somalia	Caynabo	2000-2017	-0.220	-0.098	0.025
Somalia	Ceel Barde	2000	0.004	0.031	0.107
Somalia	Ceel Barde	2017	0.003	0.017	0.044
Somalia	Ceel Barde	2000-2017	-0.133	-0.019	0.095
Somalia	Ceel Buur	2000	0.002	0.018	0.057
Somalia	Ceel Buur	2017	0.001	0.007	0.022
Somalia	Ceel Buur	2000-2017	-0.226	-0.113	0.003
Somalia	Ceel Dheer	2000	0.004	0.033	0.109
Somalia	Ceel Dheer	2017	0.001	0.012	0.041
Somalia	Ceel Dheer	2000-2017	-0.204	-0.073	0.065
Somalia	Ceel Waaq	2000	0.003	0.027	0.097
Somalia	Ceel Waaq	2017	0.001	0.008	0.018
Somalia	Ceel Waaq	2000-2017	-0.196	-0.072	0.046
Somalia	Ceel-Afwein	2000	0.006	0.063	0.224
Somalia	Ceel-Afwein	2017	0.001	0.006	0.022
Somalia	Ceel-Afwein	2000-2017	-0.232	-0.088	0.032
Somalia	Ceerigaabo	2000	0.003	0.046	0.205
Somalia	Ceerigaabo	2017	0.002	0.007	0.020
Somalia	Ceerigaabo	2000-2017	-0.161	-0.048	0.049
Somalia	Dhuusamareeb	2000	0.002	0.018	0.060
Somalia	Dhuusamareeb	2017	0.001	0.006	0.019
Somalia	Dhuusamareeb	2000-2017	-0.209	-0.105	0.021
Somalia	Diinsoor	2000	0.006	0.042	0.145
Somalia	Diinsoor	2017	0.003	0.016	0.051
Somalia	Diinsoor	2000-2017	-0.181	-0.078	0.027
Somalia	Dolow	2000	0.002	0.023	0.083
Somalia	Dolow	2017	0.003	0.015	0.045
Somalia	Dolow	2000-2017	-0.152	-0.033	0.080
Somalia	Eyl	2000	0.005	0.061	0.223
Somalia	Eyl	2017	0.001	0.012	0.034
Somalia	Eyl	2000-2017	-0.198	-0.073	0.050
Somalia	Gaalkacayo	2000	0.004	0.039	0.113
Somalia	Gaalkacayo	2017	0.002	0.012	0.038
Somalia	Gaalkacayo	2000-2017	-0.170	-0.057	0.058

Table 3: Malaria DALYs rate by unit *(continued)*

Country	Unit	year	mean rate	lower rate	upper rate
Somalia	Gabiley	2000	0.022	0.153	0.480
Somalia	Gabiley	2017	0.001	0.007	0.017
Somalia	Gabiley	2000-2017	-0.238	-0.118	-0.002
Somalia	Garbahaaray	2000	0.004	0.039	0.123
Somalia	Garbahaaray	2017	0.002	0.014	0.039
Somalia	Garbahaaray	2000-2017	-0.152	-0.040	0.114
Somalia	Garoowe	2000	0.003	0.030	0.142
Somalia	Garoowe	2017	0.001	0.007	0.020
Somalia	Garoowe	2000-2017	-0.184	-0.057	0.060
Somalia	Goldogob	2000	0.002	0.027	0.095
Somalia	Goldogob	2017	0.001	0.008	0.027
Somalia	Goldogob	2000-2017	-0.204	-0.087	0.063
Somalia	Hargeysa	2000	0.029	0.166	0.506
Somalia	Hargeysa	2017	0.001	0.007	0.019
Somalia	Hargeysa	2000-2017	-0.285	-0.149	-0.051
Somalia	Hobyo	2000	0.004	0.043	0.149
Somalia	Hobyo	2017	0.002	0.010	0.035
Somalia	Hobyo	2000-2017	-0.168	-0.062	0.048
Somalia	Iskushuban	2000	0.007	0.057	0.185
Somalia	Iskushuban	2017	0.001	0.007	0.024
Somalia	Iskushuban	2000-2017	-0.193	-0.089	0.035
Somalia	Jalalaqsi	2000	0.006	0.049	0.142
Somalia	Jalalaqsi	2017	0.001	0.011	0.045
Somalia	Jalalaqsi	2000-2017	-0.216	-0.100	0.055
Somalia	Jamaame	2000	0.017	0.181	0.480
Somalia	Jamaame	2017	0.002	0.013	0.043
Somalia	Jamaame	2000-2017	-0.259	-0.122	0.024
Somalia	Jariiban	2000	0.004	0.044	0.128
Somalia	Jariiban	2017	0.001	0.011	0.038
Somalia	Jariiban	2000-2017	-0.180	-0.045	0.083
Somalia	Jawhar	2000	0.015	0.123	0.423
Somalia	Jawhar	2017	0.001	0.010	0.032
Somalia	Jawhar	2000-2017	-0.245	-0.124	0.004
Somalia	Jilib	2000	0.018	0.190	0.579
Somalia	Jilib	2017	0.003	0.022	0.066
Somalia	Jilib	2000-2017	-0.233	-0.104	0.017
Somalia	Kismaayo	2000	0.014	0.139	0.326
Somalia	Kismaayo	2017	0.002	0.014	0.043
Somalia	Kismaayo	2000-2017	-0.269	-0.125	0.007
Somalia	Kuntuwaaray	2000	0.023	0.218	0.671
Somalia	Kuntuwaaray	2017	0.003	0.029	0.099
Somalia	Kuntuwaaray	2000-2017	-0.183	-0.054	0.071
Somalia	Lascaanod	2000	0.005	0.043	0.163
Somalia	Lascaanod	2017	0.002	0.010	0.032
Somalia	Lascaanod	2000-2017	-0.171	-0.065	0.067
Somalia	Lughaya	2000	0.014	0.112	0.266
Somalia	Lughaya	2017	0.001	0.005	0.016
Somalia	Lughaya	2000-2017	-0.297	-0.160	-0.047
Somalia	Luuk	2000	0.002	0.019	0.079
Somalia	Luuk	2017	0.002	0.011	0.037
Somalia	Luuk	2000-2017	-0.155	-0.031	0.092
Somalia	Marka	2000	0.041	0.348	1.028
Somalia	Marka	2017	0.003	0.018	0.064
Somalia	Marka	2000-2017	-0.202	-0.091	0.031
Somalia	Mogadisho	2000	0.024	0.194	0.671
Somalia	Mogadisho	2017	0.002	0.011	0.035
Somalia	Mogadisho	2000-2017	-0.236	-0.105	0.014
Somalia	Oodweyne	2000	0.037	0.208	0.565
Somalia	Oodweyne	2017	0.002	0.010	0.030
Somalia	Oodweyne	2000-2017	-0.313	-0.180	-0.063
Somalia	Qandala	2000	0.006	0.053	0.164

Table 3: Malaria DALYs rate by unit *(continued)*

Country	Unit	year	mean rate	lower rate	upper rate
Somalia	Qandala	2017	0.001	0.008	0.027
Somalia	Qandala	2000-2017	-0.236	-0.078	0.073
Somalia	Qansax Dheere	2000	0.004	0.041	0.175
Somalia	Qansax Dheere	2017	0.002	0.015	0.051
Somalia	Qansax Dheere	2000-2017	-0.223	-0.090	0.023
Somalia	Qardho	2000	0.007	0.103	0.294
Somalia	Qardho	2017	0.003	0.015	0.039
Somalia	Qardho	2000-2017	-0.199	-0.069	0.051
Somalia	Qoryooley	2000	0.048	0.380	1.065
Somalia	Qoryooley	2017	0.006	0.031	0.100
Somalia	Qoryooley	2000-2017	-0.172	-0.071	0.038
Somalia	Rab Dhuure	2000	0.003	0.019	0.059
Somalia	Rab Dhuure	2017	0.002	0.014	0.035
Somalia	Rab Dhuure	2000-2017	-0.166	-0.043	0.065
Somalia	Saakow	2000	0.005	0.040	0.114
Somalia	Saakow	2017	0.002	0.014	0.044
Somalia	Saakow	2000-2017	-0.223	-0.078	0.058
Somalia	Sablale	2000	0.016	0.141	0.454
Somalia	Sablale	2017	0.003	0.031	0.098
Somalia	Sablale	2000-2017	-0.193	-0.055	0.102
Somalia	Sheekh	2000	0.012	0.110	0.344
Somalia	Sheekh	2017	0.001	0.005	0.012
Somalia	Sheekh	2000-2017	-0.273	-0.141	-0.028
Somalia	Taleex	2000	0.005	0.090	0.337
Somalia	Taleex	2017	0.004	0.017	0.048
Somalia	Taleex	2000-2017	-0.172	-0.044	0.098
Somalia	Tiyeeglow	2000	0.005	0.042	0.133
Somalia	Tiyeeglow	2017	0.003	0.024	0.083
Somalia	Tiyeeglow	2000-2017	-0.210	-0.076	0.051
Somalia	Wajid	2000	0.003	0.023	0.087
Somalia	Wajid	2017	0.003	0.020	0.070
Somalia	Wajid	2000-2017	-0.149	-0.025	0.097
Somalia	Wanla Weyn	2000	0.013	0.107	0.294
Somalia	Wanla Weyn	2017	0.002	0.012	0.032
Somalia	Wanla Weyn	2000-2017	-0.235	-0.108	-0.003
Somalia	Xarardheere	2000	0.002	0.021	0.071
Somalia	Xarardheere	2017	0.001	0.007	0.025
Somalia	Xarardheere	2000-2017	-0.188	-0.068	0.058
Somalia	Xudun	2000	0.006	0.056	0.196
Somalia	Xudun	2017	0.002	0.010	0.031
Somalia	Xudun	2000-2017	-0.199	-0.057	0.069
Somalia	Xudur	2000	0.003	0.028	0.110
Somalia	Xudur	2017	0.002	0.019	0.070
Somalia	Xudur	2000-2017	-0.169	-0.043	0.077
Somalia	Zeylac	2000	0.029	0.208	0.566
Somalia	Zeylac	2017	0.002	0.010	0.027
Somalia	Zeylac	2000-2017	-0.246	-0.124	-0.019
South Sudan	Akobo	2000	0.024	0.165	0.418
South Sudan	Akobo	2017	0.014	0.092	0.263
South Sudan	Akobo	2000-2017	-0.141	-0.019	0.094
South Sudan	Al Leiri	2000	0.026	0.188	0.431
South Sudan	Al Leiri	2017	0.015	0.084	0.222
South Sudan	Al Leiri	2000-2017	-0.141	0.007	0.117
South Sudan	Al Mabien	2000	0.013	0.102	0.308
South Sudan	Al Mabien	2017	0.003	0.041	0.151
South Sudan	Al Mabien	2000-2017	-0.210	-0.069	0.067
South Sudan	Al Mayom	2000	0.008	0.143	0.469
South Sudan	Al Mayom	2017	0.007	0.045	0.124
South Sudan	Al Mayom	2000-2017	-0.148	-0.019	0.147
South Sudan	Al Renk	2000	0.022	0.161	0.464
South Sudan	Al Renk	2017	0.014	0.101	0.329

Table 3: Malaria DALYs rate by unit *(continued)*

Country	Unit	year	mean rate	lower rate	upper rate
South Sudan	Al Renk	2000-2017	-0.159	-0.025	0.103
South Sudan	Aliab	2000	0.026	0.200	0.464
South Sudan	Aliab	2017	0.013	0.096	0.282
South Sudan	Aliab	2000-2017	-0.141	-0.036	0.075
South Sudan	Amatonge	2000	0.138	0.340	0.623
South Sudan	Amatonge	2017	0.098	0.230	0.414
South Sudan	Amatonge	2000-2017	-0.045	0.004	0.053
South Sudan	Aryat	2000	0.017	0.151	0.430
South Sudan	Aryat	2017	0.013	0.053	0.191
South Sudan	Aryat	2000-2017	-0.253	-0.095	0.041
South Sudan	Aweil	2000	0.020	0.145	0.423
South Sudan	Aweil	2017	0.012	0.055	0.140
South Sudan	Aweil	2000-2017	-0.203	-0.083	0.038
South Sudan	Ayod	2000	0.024	0.132	0.331
South Sudan	Ayod	2017	0.012	0.067	0.167
South Sudan	Ayod	2000-2017	-0.141	-0.006	0.087
South Sudan	Bahr al Jabal	2000	0.075	0.295	0.578
South Sudan	Bahr al Jabal	2017	0.033	0.122	0.266
South Sudan	Bahr al Jabal	2000-2017	-0.141	-0.059	0.022
South Sudan	Baleit	2000	0.028	0.126	0.334
South Sudan	Baleit	2017	0.006	0.053	0.178
South Sudan	Baleit	2000-2017	-0.192	-0.055	0.057
South Sudan	Bor	2000	0.027	0.203	0.467
South Sudan	Bor	2017	0.012	0.117	0.355
South Sudan	Bor	2000-2017	-0.135	-0.016	0.091
South Sudan	Fam al Zaraf	2000	0.029	0.105	0.242
South Sudan	Fam al Zaraf	2017	0.009	0.065	0.171
South Sudan	Fam al Zaraf	2000-2017	-0.138	-0.018	0.098
South Sudan	Faring	2000	0.009	0.113	0.377
South Sudan	Faring	2017	0.007	0.053	0.190
South Sudan	Faring	2000-2017	-0.158	-0.019	0.128
South Sudan	Fashooda	2000	0.038	0.154	0.425
South Sudan	Fashooda	2017	0.012	0.071	0.229
South Sudan	Fashooda	2000-2017	-0.165	-0.041	0.067
South Sudan	Gogrial	2000	0.014	0.162	0.517
South Sudan	Gogrial	2017	0.011	0.044	0.111
South Sudan	Gogrial	2000-2017	-0.212	-0.073	0.054
South Sudan	Kajo Kaii	2000	0.191	0.429	0.723
South Sudan	Kajo Kaii	2017	0.036	0.140	0.314
South Sudan	Kajo Kaii	2000-2017	-0.174	-0.086	-0.016
South Sudan	Kapoeta	2000	0.057	0.251	0.563
South Sudan	Kapoeta	2017	0.050	0.197	0.409
South Sudan	Kapoeta	2000-2017	-0.080	0.007	0.088
South Sudan	Magwi	2000	0.131	0.366	0.644
South Sudan	Magwi	2017	0.055	0.189	0.365
South Sudan	Magwi	2000-2017	-0.108	-0.028	0.018
South Sudan	Malek	2000	0.012	0.156	0.473
South Sudan	Malek	2017	0.009	0.047	0.121
South Sudan	Malek	2000-2017	-0.208	-0.060	0.072
South Sudan	Malut	2000	0.019	0.151	0.511
South Sudan	Malut	2017	0.008	0.065	0.232
South Sudan	Malut	2000-2017	-0.178	-0.052	0.070
South Sudan	Mayot	2000	0.019	0.169	0.519
South Sudan	Mayot	2017	0.007	0.067	0.162
South Sudan	Mayot	2000-2017	-0.181	-0.041	0.072
South Sudan	Meridi	2000	0.104	0.281	0.539
South Sudan	Meridi	2017	0.042	0.141	0.317
South Sudan	Meridi	2000-2017	-0.165	-0.066	0.000
South Sudan	Mundri	2000	0.052	0.216	0.410
South Sudan	Mundri	2017	0.028	0.109	0.244
South Sudan	Mundri	2000-2017	-0.153	-0.063	0.015

Table 3: Malaria DALYs rate by unit *(continued)*

Country	Unit	year	mean rate	lower rate	upper rate
South Sudan	Nahr Atiem	2000	0.033	0.148	0.417
South Sudan	Nahr Atiem	2017	0.013	0.073	0.207
South Sudan	Nahr Atiem	2000-2017	-0.186	-0.038	0.054
South Sudan	Nahr Lol	2000	0.014	0.190	0.540
South Sudan	Nahr Lol	2017	0.013	0.053	0.137
South Sudan	Nahr Lol	2000-2017	-0.165	-0.058	0.067
South Sudan	Nahr Yei	2000	0.170	0.364	0.628
South Sudan	Nahr Yei	2017	0.047	0.144	0.335
South Sudan	Nahr Yei	2000-2017	-0.159	-0.078	-0.015
South Sudan	Pibor	2000	0.038	0.192	0.455
South Sudan	Pibor	2017	0.015	0.097	0.234
South Sudan	Pibor	2000-2017	-0.128	-0.026	0.074
South Sudan	Rabkona	2000	0.009	0.132	0.376
South Sudan	Rabkona	2017	0.009	0.058	0.136
South Sudan	Rabkona	2000-2017	-0.113	-0.002	0.157
South Sudan	Raja	2000	0.028	0.154	0.429
South Sudan	Raja	2017	0.019	0.083	0.228
South Sudan	Raja	2000-2017	-0.136	-0.045	0.049
South Sudan	Rumbek	2000	0.033	0.172	0.407
South Sudan	Rumbek	2017	0.016	0.092	0.242
South Sudan	Rumbek	2000-2017	-0.104	0.015	0.106
South Sudan	Shobet	2000	0.030	0.150	0.366
South Sudan	Shobet	2017	0.009	0.078	0.229
South Sudan	Shobet	2000-2017	-0.151	0.006	0.141
South Sudan	Shokodom	2000	0.119	0.368	0.709
South Sudan	Shokodom	2017	0.096	0.284	0.506
South Sudan	Shokodom	2000-2017	-0.043	0.017	0.078
South Sudan	Sobat	2000	0.019	0.176	0.545
South Sudan	Sobat	2017	0.013	0.090	0.274
South Sudan	Sobat	2000-2017	-0.176	-0.043	0.054
South Sudan	Terkaka	2000	0.039	0.239	0.546
South Sudan	Terkaka	2017	0.022	0.103	0.296
South Sudan	Terkaka	2000-2017	-0.142	-0.053	0.044
South Sudan	Tombura	2000	0.114	0.282	0.520
South Sudan	Tombura	2017	0.041	0.172	0.354
South Sudan	Tombura	2000-2017	-0.131	-0.053	0.012
South Sudan	Tonga	2000	0.037	0.132	0.305
South Sudan	Tonga	2017	0.006	0.053	0.164
South Sudan	Tonga	2000-2017	-0.188	-0.054	0.076
South Sudan	Tonj	2000	0.026	0.152	0.408
South Sudan	Tonj	2017	0.009	0.051	0.145
South Sudan	Tonj	2000-2017	-0.180	-0.059	0.087
South Sudan	Wanjuk	2000	0.027	0.211	0.571
South Sudan	Wanjuk	2017	0.014	0.061	0.189
South Sudan	Wanjuk	2000-2017	-0.194	-0.070	0.046
South Sudan	Warab	2000	0.017	0.156	0.436
South Sudan	Warab	2017	0.011	0.046	0.143
South Sudan	Warab	2000-2017	-0.199	-0.081	0.030
South Sudan	Wat	2000	0.027	0.136	0.364
South Sudan	Wat	2017	0.017	0.070	0.210
South Sudan	Wat	2000-2017	-0.142	-0.025	0.078
South Sudan	Wau	2000	0.036	0.174	0.395
South Sudan	Wau	2017	0.013	0.079	0.217
South Sudan	Wau	2000-2017	-0.140	-0.053	0.036
South Sudan	Yambio	2000	0.111	0.290	0.516
South Sudan	Yambio	2017	0.033	0.125	0.258
South Sudan	Yambio	2000-2017	-0.191	-0.083	-0.003
South Sudan	Yerol	2000	0.021	0.186	0.434
South Sudan	Yerol	2017	0.017	0.102	0.264
South Sudan	Yerol	2000-2017	-0.148	-0.002	0.111
Swaziland	Dvokodvweni	2000	0.000	0.000	0.000

Table 3: Malaria DALYs rate by unit *(continued)*

Country	Unit	year	mean rate	lower rate	upper rate
Swaziland	Dvokodvweni	2017	0.000	0.000	0.000
Swaziland	Dvokodvweni	2000-2017	NA	NA	NA
Swaziland	Ekukhanyeni	2000	0.000	0.000	0.000
Swaziland	Ekukhanyeni	2017	0.000	0.000	0.000
Swaziland	Ekukhanyeni	2000-2017	NA	NA	NA
Swaziland	Gege	2000	0.000	0.000	0.000
Swaziland	Gege	2017	0.000	0.000	0.000
Swaziland	Gege	2000-2017	NA	NA	NA
Swaziland	Hhukwini	2000	0.000	0.000	0.000
Swaziland	Hhukwini	2017	0.000	0.000	0.000
Swaziland	Hhukwini	2000-2017	NA	NA	NA
Swaziland	Hlane	2000	0.000	0.000	0.000
Swaziland	Hlane	2017	0.000	0.000	0.000
Swaziland	Hlane	2000-2017	NA	NA	NA
Swaziland	Hosea	2000	0.000	0.000	0.000
Swaziland	Hosea	2017	0.000	0.000	0.000
Swaziland	Hosea	2000-2017	NA	NA	NA
Swaziland	Kubuta	2000	0.000	0.000	0.000
Swaziland	Kubuta	2017	0.000	0.000	0.000
Swaziland	Kubuta	2000-2017	NA	NA	NA
Swaziland	Kwaluseni	2000	0.000	0.000	0.000
Swaziland	Kwaluseni	2017	0.000	0.000	0.000
Swaziland	Kwaluseni	2000-2017	NA	NA	NA
Swaziland	Lamgabhi	2000	0.000	0.000	0.000
Swaziland	Lamgabhi	2017	0.000	0.000	0.000
Swaziland	Lamgabhi	2000-2017	NA	NA	NA
Swaziland	Lobamba	2000	0.000	0.000	0.000
Swaziland	Lobamba	2017	0.000	0.000	0.000
Swaziland	Lobamba	2000-2017	NA	NA	NA
Swaziland	Lobamba Lomdzala	2000	0.000	0.000	0.000
Swaziland	Lobamba Lomdzala	2017	0.000	0.000	0.000
Swaziland	Lobamba Lomdzala	2000-2017	NA	NA	NA
Swaziland	Lomahasha	2000	0.014	0.035	0.071
Swaziland	Lomahasha	2017	0.002	0.014	0.035
Swaziland	Lomahasha	2000-2017	-0.179	-0.067	0.029
Swaziland	Lubuli	2000	0.002	0.006	0.017
Swaziland	Lubuli	2017	0.002	0.014	0.044
Swaziland	Lubuli	2000-2017	-0.142	0.015	0.146
Swaziland	Ludzeludze	2000	0.000	0.000	0.000
Swaziland	Ludzeludze	2017	0.000	0.000	0.000
Swaziland	Ludzeludze	2000-2017	NA	NA	NA
Swaziland	Lugongolweni	2000	0.009	0.024	0.055
Swaziland	Lugongolweni	2017	0.001	0.008	0.021
Swaziland	Lugongolweni	2000-2017	-0.172	-0.059	0.046
Swaziland	Madlangempisi	2000	0.000	0.000	0.000
Swaziland	Madlangempisi	2017	0.000	0.000	0.000
Swaziland	Madlangempisi	2000-2017	NA	NA	NA
Swaziland	Mafutseni	2000	0.000	0.000	0.000
Swaziland	Mafutseni	2017	0.000	0.000	0.000
Swaziland	Mafutseni	2000-2017	NA	NA	NA
Swaziland	Mahlangatja	2000	0.000	0.000	0.000
Swaziland	Mahlangatja	2017	0.000	0.000	0.000
Swaziland	Mahlangatja	2000-2017	NA	NA	NA
Swaziland	Mangcongco	2000	0.000	0.000	0.000
Swaziland	Mangcongco	2017	0.000	0.000	0.000
Swaziland	Mangcongco	2000-2017	NA	NA	NA
Swaziland	Manzini North	2000	0.000	0.000	0.000
Swaziland	Manzini North	2017	0.000	0.000	0.000
Swaziland	Manzini North	2000-2017	NA	NA	NA
Swaziland	Manzini South	2000	0.000	0.000	0.000
Swaziland	Manzini South	2017	0.000	0.000	0.000

Table 3: Malaria DALYs rate by unit *(continued)*

Country	Unit	year	mean rate	lower rate	upper rate
Swaziland	Manzini South	2000-2017	NA	NA	NA
Swaziland	Maseyisini	2000	0.000	0.000	0.000
Swaziland	Maseyisini	2017	0.000	0.000	0.000
Swaziland	Maseyisini	2000-2017	NA	NA	NA
Swaziland	Matsanjeni North	2000	0.012	0.030	0.063
Swaziland	Matsanjeni North	2017	0.002	0.011	0.034
Swaziland	Matsanjeni North	2000-2017	-0.193	-0.047	0.081
Swaziland	Matsanjeni South	2000	0.000	0.000	0.000
Swaziland	Matsanjeni South	2017	0.000	0.000	0.000
Swaziland	Matsanjeni South	2000-2017	NA	NA	NA
Swaziland	Mayiwane	2000	0.000	0.000	0.000
Swaziland	Mayiwane	2017	0.000	0.000	0.000
Swaziland	Mayiwane	2000-2017	-0.291	-0.158	-0.018
Swaziland	Mbabane East	2000	0.000	0.000	0.000
Swaziland	Mbabane East	2017	0.000	0.000	0.000
Swaziland	Mbabane East	2000-2017	NA	NA	NA
Swaziland	Mbabane West	2000	0.000	0.000	0.000
Swaziland	Mbabane West	2017	0.000	0.000	0.000
Swaziland	Mbabane West	2000-2017	NA	NA	NA
Swaziland	Mbangweni	2000	0.000	0.000	0.000
Swaziland	Mbangweni	2017	0.000	0.000	0.000
Swaziland	Mbangweni	2000-2017	NA	NA	NA
Swaziland	Mhlambanyatsi	2000	0.000	0.000	0.000
Swaziland	Mhlambanyatsi	2017	0.000	0.000	0.000
Swaziland	Mhlambanyatsi	2000-2017	NA	NA	NA
Swaziland	Mhlangatane	2000	0.000	0.001	0.003
Swaziland	Mhlangatane	2017	0.000	0.001	0.002
Swaziland	Mhlangatane	2000-2017	-0.261	-0.129	-0.015
Swaziland	Mhlume	2000	0.000	0.001	0.003
Swaziland	Mhlume	2017	0.000	0.001	0.002
Swaziland	Mhlume	2000-2017	-0.209	-0.075	0.025
Swaziland	Mkhiweni	2000	0.000	0.000	0.000
Swaziland	Mkhiweni	2017	0.000	0.000	0.000
Swaziland	Mkhiweni	2000-2017	NA	NA	NA
Swaziland	Motjane	2000	0.000	0.000	0.000
Swaziland	Motjane	2017	0.000	0.000	0.000
Swaziland	Motjane	2000-2017	NA	NA	NA
Swaziland	Mphalaleni	2000	0.000	0.000	0.000
Swaziland	Mphalaleni	2017	0.000	0.000	0.000
Swaziland	Mphalaleni	2000-2017	NA	NA	NA
Swaziland	Mpholonjeni	2000	0.000	0.000	0.000
Swaziland	Mpholonjeni	2017	0.000	0.000	0.000
Swaziland	Mpholonjeni	2000-2017	NA	NA	NA
Swaziland	Mthongwaneni	2000	0.000	0.000	0.000
Swaziland	Mthongwaneni	2017	0.000	0.000	0.000
Swaziland	Mthongwaneni	2000-2017	NA	NA	NA
Swaziland	Mtsambama	2000	0.000	0.000	0.000
Swaziland	Mtsambama	2017	0.000	0.000	0.000
Swaziland	Mtsambama	2000-2017	NA	NA	NA
Swaziland	Ndzingeni	2000	0.000	0.000	0.000
Swaziland	Ndzingeni	2017	0.000	0.000	0.000
Swaziland	Ndzingeni	2000-2017	NA	NA	NA
Swaziland	Ngudzeni	2000	0.000	0.000	0.000
Swaziland	Ngudzeni	2017	0.000	0.000	0.000
Swaziland	Ngudzeni	2000-2017	NA	NA	NA
Swaziland	Ngwenpisi	2000	0.000	0.000	0.000
Swaziland	Ngwenpisi	2017	0.000	0.000	0.000
Swaziland	Ngwenpisi	2000-2017	NA	NA	NA
Swaziland	Nhlambeni	2000	0.000	0.000	0.000
Swaziland	Nhlambeni	2017	0.000	0.000	0.000
Swaziland	Nhlambeni	2000-2017	NA	NA	NA

Table 3: Malaria DALYs rate by unit *(continued)*

Country	Unit	year	mean rate	lower rate	upper rate
Swaziland	Nkhaba	2000	0.000	0.000	0.000
Swaziland	Nkhaba	2017	0.000	0.000	0.000
Swaziland	Nkhaba	2000-2017	NA	NA	NA
Swaziland	Nkilongo	2000	0.000	0.001	0.002
Swaziland	Nkilongo	2017	0.000	0.001	0.002
Swaziland	Nkilongo	2000-2017	-0.193	-0.055	0.075
Swaziland	Nkwene	2000	0.000	0.000	0.000
Swaziland	Nkwene	2017	0.000	0.000	0.000
Swaziland	Nkwene	2000-2017	NA	NA	NA
Swaziland	Ntfonjeni	2000	0.000	0.002	0.004
Swaziland	Ntfonjeni	2017	0.000	0.001	0.005
Swaziland	Ntfonjeni	2000-2017	-0.273	-0.130	0.018
Swaziland	Ntondozi	2000	0.000	0.000	0.000
Swaziland	Ntondozi	2017	0.000	0.000	0.000
Swaziland	Ntondozi	2000-2017	NA	NA	NA
Swaziland	Pigg's Peak	2000	0.000	0.000	0.000
Swaziland	Pigg's Peak	2017	0.000	0.000	0.000
Swaziland	Pigg's Peak	2000-2017	-0.309	-0.150	-0.007
Swaziland	Sandleni	2000	0.000	0.000	0.000
Swaziland	Sandleni	2017	0.000	0.000	0.000
Swaziland	Sandleni	2000-2017	NA	NA	NA
Swaziland	Shiselweni	2000	0.000	0.000	0.000
Swaziland	Shiselweni	2017	0.000	0.000	0.000
Swaziland	Shiselweni	2000-2017	NA	NA	NA
Swaziland	Sigwe	2000	0.000	0.000	0.000
Swaziland	Sigwe	2017	0.000	0.000	0.000
Swaziland	Sigwe	2000-2017	NA	NA	NA
Swaziland	Siphofaneni	2000	0.000	0.000	0.000
Swaziland	Siphofaneni	2017	0.000	0.000	0.000
Swaziland	Siphofaneni	2000-2017	NA	NA	NA
Swaziland	Sithobela	2000	0.000	0.000	0.000
Swaziland	Sithobela	2017	0.000	0.000	0.000
Swaziland	Sithobela	2000-2017	NA	NA	NA
Swaziland	Somntongo	2000	0.004	0.016	0.047
Swaziland	Somntongo	2017	0.004	0.041	0.122
Swaziland	Somntongo	2000-2017	-0.147	0.019	0.164
Swaziland	Timpisini	2000	0.001	0.005	0.013
Swaziland	Timpisini	2017	0.000	0.004	0.017
Swaziland	Timpisini	2000-2017	-0.209	-0.073	0.073
Swaziland	Zombodze	2000	0.000	0.000	0.000
Swaziland	Zombodze	2017	0.000	0.000	0.000
Swaziland	Zombodze	2000-2017	NA	NA	NA
Tanzania	Arusha	2000	0.022	0.087	0.273
Tanzania	Arusha	2017	0.004	0.022	0.056
Tanzania	Arusha	2000-2017	-0.162	-0.062	0.023
Tanzania	Arusha Urban	2000	0.018	0.073	0.234
Tanzania	Arusha Urban	2017	0.005	0.022	0.057
Tanzania	Arusha Urban	2000-2017	-0.147	-0.049	0.035
Tanzania	Babati	2000	0.035	0.124	0.299
Tanzania	Babati	2017	0.014	0.047	0.108
Tanzania	Babati	2000-2017	-0.104	-0.038	0.037
Tanzania	Babati Urban	2000	0.036	0.127	0.354
Tanzania	Babati Urban	2017	0.016	0.053	0.131
Tanzania	Babati Urban	2000-2017	-0.100	-0.034	0.052
Tanzania	Bagamoyo	2000	0.468	0.851	1.401
Tanzania	Bagamoyo	2017	0.046	0.135	0.291
[truncated: 794,485 more chars]
